# DCLK1 promotes colorectal cancer stemness and aggressiveness via the XRCC5/COX2 axis

**DOI:** 10.7150/thno.72037

**Published:** 2022-07-04

**Authors:** Jee-Heun Kim, So-Yeon Park, So-El Jeon, Jang-Hyun Choi, Choong-Jae Lee, Tae-Young Jang, Hyeon-Ji Yun, Yuno Lee, Pilho Kim, Sang Hee Cho, Ji Shin Lee, Jeong-Seok Nam

**Affiliations:** 1 School of Life Sciences, Gwangju Institute of Science and Technology, Gwangju, 61005, Republic of Korea.; 2 Cell Logistics Research Center, Gwangju Institute of Science and Technology, Gwangju, 61005, Republic of Korea.; 3 Korea Chemical Bank, Korea Research Institute of Chemical Technology, Daejeon, 34114, Republic of Korea.; 4 Therapeutics and Biotechnology Division, Korea Research Institute of Chemical Technology, Daejeon, 34114, Republic of Korea.; 5 Department of Hemato-Oncology, Chonnam National University Medical School, Gwangju, 61469, Republic of Korea.; 6 Department of Pathology, Chonnam National University Medical School, Gwangju, 61469, Republic of Korea.

**Keywords:** Cancer stem cells, Inflammatory tumor microenvironment, Doublecortin-like kinase 1, Prostaglandin E_2_

## Abstract

**Rationale:** Doublecortin-like kinase 1 (DCLK1) is a serine/threonine kinase that selectively marks cancer stem-like cells (CSCs) and promotes malignant progression in colorectal cancer (CRC). However, the exact molecular mechanism by which DCLK1 drives the aggressive phenotype of cancer cells is incompletely determined.

**Methods:** Here, we performed comprehensive genomics and proteomics analyses to identify binding proteins of DCLK1 and discovered X-ray repair cross-complementing 5 (XRCC5). Thus, we explored the biological role and downstream events of the DCLK1/XRCC5 axis in human CRC cells and CRC mouse models.

**Results:** The results of comprehensive bioinformatics analyses suggested that DCLK1-driven CRC aggressiveness is linked to inflammation. Mechanistically, DCLK1 bound and phosphorylated XRCC5, which in turn transcriptionally activated cyclooxygenase-2 expression and enhanced prostaglandin E_2_ production; these events collectively generated the inflammatory tumor microenvironment and enhanced the aggressive behavior of CRC cells. Consistent with the discovered mechanism, inhibition of DCLK1 kinase activity strongly impaired the tumor seeding and growth capabilities in CRC mouse models.

**Conclusion:** Our study illuminates a novel mechanism that mediates the pro-inflammatory function of CSCs in driving the aggressive phenotype of CRC, broadening the biological function of DCLK1 in CRC.

## Introduction

Most patients with advanced cancer ultimately confront a fatal illness that is incurable by current therapeutic regimens. Accumulating evidence indicates that cancer stem-like cells (CSCs), which have strong tumor-initiating and self-renewal properties, are linked to malignant behavior in multiple types of cancer 1-3. However, most of the current CSC markers and their regulatory mechanisms are shared by normal stem cells, which remains a major challenge in selectively targeting CSCs. Doublecortin-like kinase 1 (DCLK1) is considered as one of the most specific CSC markers; it does not mark normal stem cells in the intestine but instead marks CSCs that continuously produce tumor cell progeny in intestinal tumors 4-6. Genetic studies have shown that ablation of DCLK1-expressing cells significantly suppresses tumor development without apparent damage to normal stem cells 5, 6. Furthermore, several studies have indicated that DCLK1 is frequently overexpressed not only in colorectal cancer (CRC) but also in many other cancers, including hepatocellular, pancreatic, and lung cancers, in which the expression level of DCLK1 is increased with worsening severity of dysplasia 7-10. This evidence strongly implies that DCLK1 promotes the aggressive phenotype of cancer; however, the underlying molecular mechanism remains unclear.

The inflammatory microenvironment is a hallmark of cancer that induces cancer initiation and all stages of cancer progression 11. Cancer cells, as well as surrounding stromal and inflammatory cells, engage in well-orchestrated reciprocal interactions to generate an inflammatory tumor microenvironment (TME) 12-14. Cancer cells within the inflammatory TME are highly plastic, continuously changing their phenotype and functional characteristics 15, 16. Moreover, cancer cells can hijack inflammatory mechanisms to favor their own growth and survival, which is considered a key step in cancer aggressiveness 17. Thus, a better understanding of tumor-associated inflammatory signaling and microenvironmental crosstalk is crucial for elucidating the mechanisms of tumorigenesis and for developing more efficient therapeutic strategies for aggressive cancers.

Here, to identify the downstream signaling cascade that mediates DCLK1-driven CRC aggressiveness, we conducted comprehensive bioinformatics analyses and found a potential link between DCLK1 and inflammatory signaling. We thus investigated DCLK1-interacting inflammatory proteins and downstream signaling pathways. This study provides mechanistic insight into the pro-inflammatory function of CSCs in driving the aggressive phenotype of CRC.

## Methods

### Animal models

Prior approval for the *in vivo* studies was obtained from the Institutional Animal Care and Use Committee (IACUC) of Gwangju Institute of Science and Technology (GIST, No. GIST2019-036). All mice were housed and cared for in an Association for Assessment and Accreditation of Laboratory Animal Care (AAALAC)-accredited animal facility under specific pathogen-free conditions. Four-week-old C57BL/6J, C57BL/6J-*Apc^Min^*/J and NSG^TM^ (NOD.Cg-Prkdc^scid^ Il2rg^tm1Wjl^/SzJ), BALB/c mice were purchased from Jackson Laboratory (Bar Harbor, ME, USA) and housed at Laboratory Animal Resource Center (LARC) GIST.

### Cell lines

Human CRC cell lines including HCT116, HT29, SW480, DLD1, LoVo, and LS174T were purchased from the Korean Cell Line Bank (Seoul, Republic of Korea). Human colon epithelial cell line FHC and human embryonic kidney cell line HEK293T were purchased from the American Type Culture Collection (Rockville, MD, USA). Murine colon adenocarcinoma cell line MC-38 was purchased from Kerafast (Boston, MA, USA). Patient-derived colorectal cancer cells (hCRC#1 and hCRC#2, Table S1) were acquired and cultured as described in previous reports 18. All cells were cultured in accordance with the supplier's instructions. All experiments were performed within 20 passages from the first thaw, and cells were routinely tested for mycoplasma contamination using the e-MycoTM mycoplasma detection kit (iNtron Biotechnology, Seongnam, Republic of Korea).

### Patient samples

All work related to human tissues obtained from CRC patients was preapproved by the Institutional Review Board (IRB) at GIST (20210115-BR-58-02-02). All work related to human tissues was conducted in accordance with the Helsinki Declaration, and informed consent forms were signed and obtained from all subjects prior to participation.

### Statistical analysis

All *in vitro* and *in vivo* data are presented as the mean ± standard error of the mean (SEM). Statistical calculations were derived from at least three independent experiments and analyzed by Student's t-test or two-way ANOVA with the Bonferroni multiple comparison test for two groups, and by one-way ANOVA with Dunnett's multiple comparison test for groups of three or more. Statistical significance of overall survival and relapse-free survival rates was determined by the log-rank test and plotted using the Kaplan-Meier method. Correlation analysis was performed by calculating Spearman's correlation coefficient. Asterisks are used to indicate statistical significance. Notably, *, ** and *** indicate *p* < 0.05, *p* < 0.01 and *p* < 0.001, respectively.

## Results

### Increased DCLK1 expression implies CRC aggressiveness

**Figure 1:**
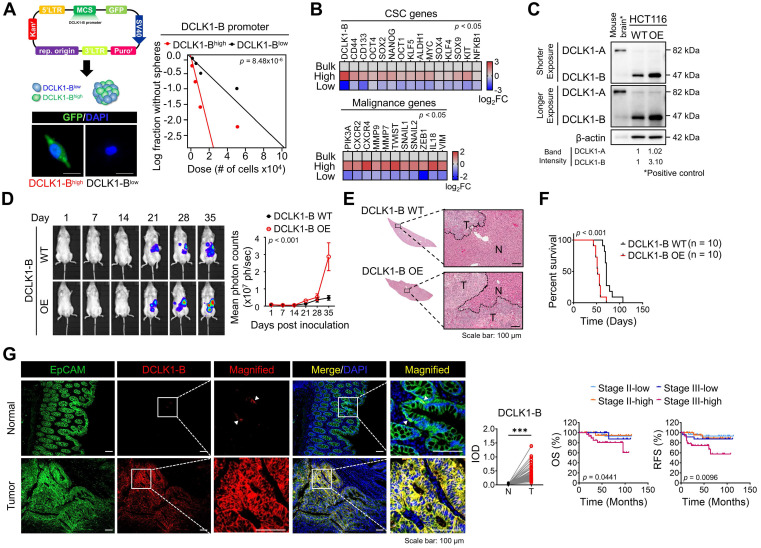
** Increased DCLK1-B expression infers CRC aggressiveness. (A)** Schematic illustration of DCLK1-B promoter-green fluorescent protein (GFP)-tagged cells. DCLK1-B^high^ and DCLK1-B^low^ cells were sorted based on GFP tagging, and the difference in stemness between the two groups was investigated via an *in vitro* LDA. **(B)** Heatmap comparing the relative expression of CSC genes and malignancy-related genes in the DCLK1-B bulk, DCLK1-B^high^, and DCLK1-B^low^ cell populations, as determined by RT-qPCR (*n* = 3 biological replicates). **(C)** Immunoblot of DCLK1-A and DCLK1-B expression in DCLK1-B WT and OE HCT116 cell lines, accompanied by a positive control (mouse brain). **(D and E)** Effect of DCLK1-B overexpression on liver metastasis. Luciferase-labeled DCLK1-B WT and DCLK1-B OE HCT116 cells were inoculated into the spleens of NSG mice. Mice were tracked for 35 days after splenic injection (n = 10 mice per group). (D) Representative *in vivo* bioluminescence images (left) of mice injected with luciferase-labeled DCLK1-B WT and DCLK1-B OE HCT116 cells, accompanied by a corresponding graph showing the quantitative analysis of the region of interest (right). (E) Representative hematoxylin and eosin-stained livers with metastasis. N: normal, T: tumor. **(F)** Survival analysis of the DCLK1-B WT- and DCLK1-B OE-inoculated groups (n = 10 mice per group). Survival curves were plotted using the Kaplan-Meier method, and statistical significance was determined by the log-rank test. **(G)** Immunofluorescence analysis of DCLK1-B expression in tumor and matched normal adjacent intestinal tissues from CRC patients (n = 123 patients). Immunofluorescence staining is shown for EpCAM (green), DCLK1-B (red), and DAPI staining (blue) with the corresponding merged and magnified images. Scale bars, 100 µm. Graphs show the integrated optical density (IOD) indicating the DCLK1-B protein level in the normal and tumor epithelium (left) and the Kaplan-Meier survival curves of the CRC patients (right). RFS, relapse-free survival; OS, overall survival. Statistical significance was determined by paired Student's t-tests for IOD quantification and by the log-rank test for Kaplan-Meier analysis. The data are presented as the means ± SEMs. *** indicates *p* < 0.001. The statistical significance of differences in tumor growth and the survival of mice with liver metastasis was determined by two-way repeated-measures ANOVA followed by the Bonferroni post hoc test.

Two types of DCLK1 protein isoforms are generated from two distinct promoter regions: the α-promoter regulates the transcription of the ~82 kDa DCLK1 isoform (DCLK1-A), and the β-promoter regulates the transcription of the ~47 kDa DCLK1 isoform (DCLK1-B). We previously reported that CRC cells predominantly express DCLK1-B rather than DCLK1-A because of increased β-promoter activity 18. We thus investigated whether the endogenous DCLK1-B level represents the degree of stemness. Therefore, we generated a DCLK1-B-GFP reporter system by inserting the β-promoter fragment 18 into the pLenti-promoterless-GFP vector (**Figure 1A** and Figure S1A) and sorted bulk CRC cells into the DCLK1-B^high^ and DCLK1-B^low^ populations. The DCLK1-B^high^ CRC population exhibited a greater sphere-forming potential than the DCLK1-B^low^ population (**Figure 1A** and Figure S1B), suggesting that the endogenous DCLK1-B level represents stronger self-renewal activity. Consistent with this finding, a comparison of transcript levels suggested a global trend of CSC enrichment in the DCLK1-B^high^ population but not in the DCLK1-B^low^ population, as evidenced by the increases in the expression of CSC surface markers and stem cell-related transcription factors (**Figure 1B**). Intriguingly, the expression of malignancy-related genes was significantly increased in the DCLK1-B^high^ population compared with the DCLK1-B^low^ population, suggesting a potential role of DCLK1-B in CRC malignancy (**Figure 1B**).

To evaluate the potential roles of DCLK1-B in CRC malignancy, we generated DCLK1-B overexpressing (OE) HCT116 cells (**Figure 1C**) and employed multiple mouse xenograft models. We subcutaneously inoculated DCLK1-B OE or wild-type (WT) cells into NOD.Cg-Prkdc^scid^ Il2rg^tm1Wjl^/SzJ (NSG) mice and observed that DCLK1-B OE cells generated a larger tumor burden than WT cells (Figure S2A-B). Notably, when we isolated DCLK1-B OE cells from the tumors and reinoculated them into NSG mice to monitor secondary tumor growth, DCLK1-B OE cells exhibited stronger tumor seeding and growth capabilities (Figure S2C-D). In parallel, we performed a splenic injection model, a common murine model, to identify the step governing metastasis and distant organ colonization. DCLK1-B OE dramatically enhanced the colonization potential of CRC cells in the liver, indicating a critical role of DCLK1-B in the metastasis of CRC cells (**Figure 1D-E** and Figure S2E). In addition, the survival times of DCLK1-B OE CRC cell-bearing mice were significantly shorter than those of WT CRC cell-bearing mice (**Figure 1F**). Collectively, these results indicate that overexpression of DCLK1-B is beneficial for CRC malignancy and stemness.

Consistent with the mouse experiments, our histological analysis of 123 patients with CRC revealed significant overexpression of DCLK1-B in the tumor epithelium compared with the normal epithelium (**Figure 1G**), as observed in a previous report 19. Interestingly, we found that increased DCLK1-B expression in the tumor epithelium was significantly correlated with T stage and recurrence (Table S1) and that DCLK1-B expression was significantly correlated with poor clinical outcomes, such as shorter overall survival and recurrence-free survival times, in patients with CRC (**Figure 1G**). These findings confirmed the connection between DCLK1-B and CRC malignancy.

### DCLK1 promotes the malignant phenotype of CRC

**Figure 2:**
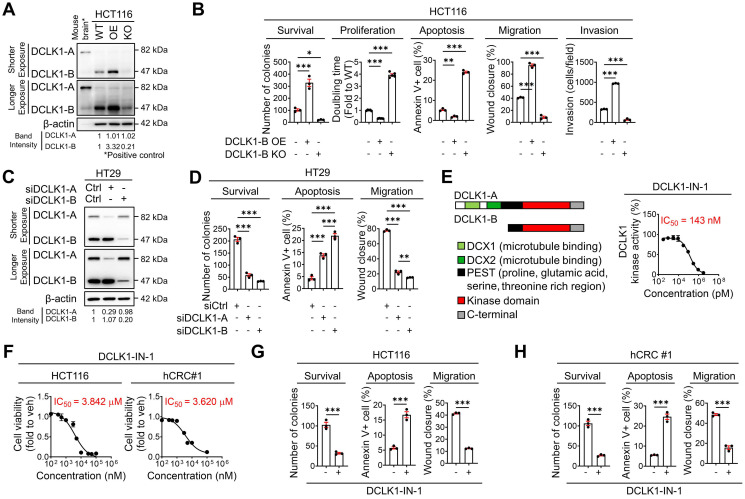
** DCLK1-B promotes cell survival, apoptosis resistance, and migration. (A)** Immunoblot of DCLK1-A and DCLK1-B expression in DCLK1-B WT, OE, and KO HCT116 cell lines, accompanied by a positive control (mouse brain). **(B)** Series of biological functional assays showing the effects of DCLK1-B expression on cancer cell survival, proliferation, apoptosis, migration, and invasion. Clonogenic assays (survival), MTT assays (proliferation), Annexin-PI FACS analysis (apoptosis), wound closure assays (migration), and invasion assays (invasion) were performed with DCLK1-B WT, OE, and KO HCT116 cells (n = 3 biological replicates). **(C)** Immunoblot of DCLK1-A and DCLK1-B expression upon independent KD of DCLK1-A and DCLK1-B by siRNA transfection. After 48 h of siRNA transfection, cells were lysed for protein analysis. **(D)** Series of biological functional assays showing the effects of DCLK1-A and DCLK1-B KD on cancer cell survival, apoptosis, and migration. **(E)** Schematic illustration showing the lengths and the shared protein kinase domain of the DCLK1 isoforms referenced in UniProt [O15075] (left). The kinase activity of DCLK1 was measured at increasing concentrations of DCLK1-IN-1 (right, n = 5 biological replicates). The IC_50_ value, 143 nM, is shown. **(F)** Cell viability rates and cytotoxic IC_50_ values were determined by an MTT assay after 48 h of treatment with DCLK1-IN-1 in both HCT116 and hCRC#1 cells (n = 5 biological replicates for both cell lines). **(G and H)** Series of biological functional assays showing the effects of DCLK1-IN-1 on cancer cell survival (3 µM), apoptosis (3 µM), and migration (1 µM) in HCT116 (G) and hCRC#1 (H) cells (n = 3 biological replicates). The data are presented as the means ± SEMs. *, ** and *** indicate *p* < 0.05, *p* < 0.01, and *p* < 0.001, respectively. Statistical significance was determined by unpaired two-tailed Student's t-tests for comparisons between two groups and one-way ANOVA with Dunnett's multiple comparison test for comparisons among three groups.

To obtain deeper insight into the biological role of DCLK1 in CRC malignancy, we performed a series of *in vitro* assays. First, we examined the phenotypic alterations in HCT116 cells upon DCLK1-B OE and DCLK1-B knockout (KO, **Figure 2A** and Figure S3A). DCLK1-B OE enhanced cancer cell survival, proliferation, migration, and invasion but reduced apoptosis, which collectively indicated increased aggressiveness (**Figure 2B** and Figure S3B-E). Conversely, DCLK1-B KO significantly attenuated the aggressive phenotypes of HCT116 cells (**Figure 2B** and Figure S3B-E). Similarly, suppression of DCLK1-B expression in patient-derived CRC cells (hCRC#1, Figure S3F-G) by small interfering RNAs (siRNAs) attenuated CRC aggressiveness (Figure S3H-J). These results indicate that DCLK1-B plays a critical role in determining the tumor formation, growth, and metastasis capacities, as observed in the mouse xenograft models.

To confirm whether those presented aggressive phenotypes are reliant to isoform specific, we used HT29 cells which is thought to be proper to investigate an independent effect of isoform in CRC aggressiveness (Figure S3K). Intriguingly, selective suppression of DCLK1-A expression also substantially attenuated aggressiveness (**Figure 2C-D** and Figure S3L-O). In parallel, we observed that selective suppression of DCLK1-B resulted in a greater decrease in CRC aggressiveness. The enhanced effectiveness of DCLK1-B knockdown (KD) in HT29 cells may be derived from the prominent expression of DCLK1-B in these cells. Notably, these results indicated that both DCLK1-A and DCLK1-B play a critical role in CRC aggressiveness, and this observation prompted us to focus on the kinase domain that is common to both DCLK1-A and DCLK1-B. To explore the role of the DCLK1 kinase domain in CRC aggressiveness, we performed a series of *in vitro* experiments using DCLK1-IN-1 20, a recently discovered selective DCLK1 inhibitor that selectively generates a considerable conformational shift in the ATP binding site inside the kinase domain without interfering with the DCX domain 21. DCLK1-IN-1 inhibited DCLK1 kinase activity by 50% at 143 nM (**Figure 2E**). In addition, DCLK1-IN-1 efficiently reduced the growth of HCT116 and hCRC#1 cells at half-maximal inhibitory concentration (IC_50_) values of 3.842 µM and 3.620 µM, respectively (**Figure 2F**). In parallel, treatment with 3 µM DCLK1-IN-1 significantly reduced the survival potential but increased the apoptosis of CRC cells (**Figure 2G-H** and Figure S3P-Q). Moreover, DCLK1-IN-1 significantly reduced the migration of CRC cells at a concentration of 1 µM, which did not alter cell growth (**Figure 2G-H** and Figure S3R). Collectively, these results indicate an indispensable role of the kinase domain of DCLK1 in CRC aggressiveness.

### DCLK1 binds X-ray repair cross-complementing 5 (XRCC5) to facilitate its phosphorylation and cyclooxygenase-2 (COX2) expression

**Figure 3:**
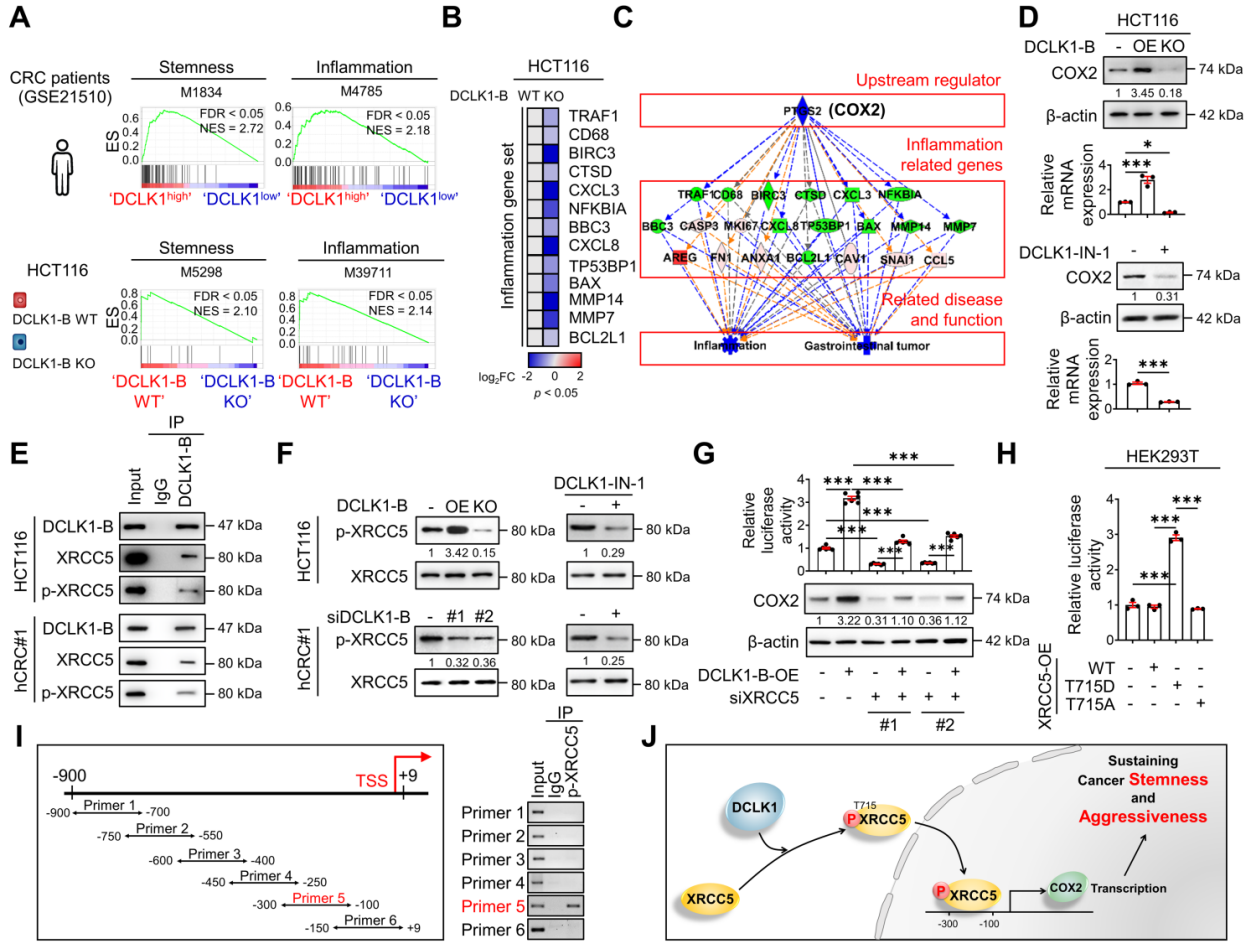
** DCLK1 enhances COX2 expression via XRCC5 phosphorylation. (A)** GSEA of pathways enriched in DCLK1^high^ patients compared to DCLK1^low^ patients from open-source CRC patient data (GSE21510) (top) and in DCLK1-B WT cells compared to DCLK1-B KO cells from RNA sequencing profiles (bottom). **(B)** Heatmap showing the relative expression of inflammation-related genes upon DCLK1-B KO, as determined by RT-qPCR (n = 3 biological replicates). **(C)** Upstream analysis suggesting a potential relation of COX2 with a related disease and function, i.e., inflammation and gastrointestinal tumors, caused by DCLK1-B expression alteration. **(D)** Alteration of COX2 expression upon DCLK1-B OE and KO (top) and DCLK1-B kinase domain inhibition (1 µM, bottom). COX2 expression was analyzed at the protein and mRNA levels. **(E)** Immunoblot of DCLK1-B, XRCC5, and phosphorylated (p-) XRCC5 in HCT116 (top) and hCRC#1 (bottom) cells subjected to immunoprecipitation with an anti-DCLK1-B antibody. **(F)** Immunoblot of p-XRCC5 and XRCC5 upon DCLK1-B expression regulation (left) and DCLK1-B kinase domain inhibition (1 µM, right) in both HCT116 (top) and hCRC#1 (bottom) cells. **(G)** Relative transcription of COX2 and its resultant translation upon DCLK1-B OE and siXRCC5 transfection. A luciferase vector containing the COX2 promoter region (-1236 to +230) was transfected into cells, and luminescence was measured and normalized to β-galactosidase (n = 3 biological replicates). **(H)** Relative transcription of COX2 upon OE of XRCC5 WT, an active mutant form of XRCC5 (T715D), and an inactive phosphomimetic form of XRCC5 (T715A). Total transcription was normalized to β-galactosidase transcription and is presented as the fold change with respect to non-transfected HEK293T cells (n = 3 biological replicates). **(I)** Potential binding site for p-XRCC5 in the COX2 promoter region identified by a chromatin immunoprecipitation assay. The COX2 promoter region was fragmented into 6 segments and immunoprecipitated with an anti-p-XRCC5 antibody. **(J)** Schematic illustration of the mechanism by which the DCLK1/p-XRCC5/COX2 axis sustains cancer stemness and aggressiveness. The data are presented as the means ± SEMs. *** indicates *p* < 0.001. Statistical significance was determined by unpaired two-tailed Student's t-tests for comparisons between two groups and one-way ANOVA with Dunnett's multiple comparison test for comparisons among three or more groups.

To obtain further mechanistic insight, we compared the gene expression profiles of tumors from 104 CRC patients (GSE21510) with higher DCLK1 expression (DCLK1^high^) and lower DCLK1 expression (DCLK1^low^). The list of differentially expressed genes in DCLK1^high^ tumors (Table S2) was subjected to gene set enrichment analysis (GSEA). As expected, the stemness gene signature was significantly enriched in DCLK1^high^ tumors (**Figure 3A**). Intriguingly, the inflammatory gene signature was also significantly enriched in DCLK1^high^ tumors (**Figure 3A**). Consistent with these clinical data, GSEA of RNA sequencing data from DCLK1-B KO HCT116 cells (Table S3) repeatedly confirmed that both the stemness and inflammatory gene signatures were downregulated by DCLK1-B KO (**Figure 3A**). In parallel, Ingenuity Pathway Analysis (IPA) of the differentially expressed genes in DCLK1-B KO HCT116 cells revealed that cancer and inflammation were among the diseases and functions most affected by DCLK1-B KO (Figure S4A). Indeed, validation with CRC cells revealed that the expression of inflammatory genes tended to decrease upon DCLK1-B deletion (**Figure 3B**).

An inflammatory TME is a strong trigger of CRC development and malignant progression, and treatment with anti-inflammatory drugs has been shown to efficiently suppress intestinal tumorigenesis 12, 16, 22, 23. We thus explored the potential molecular network involved in the relationship between DCLK1-B and inflammation by performing upstream regulator analysis using IPA. The results indicated that the suppression of inflammation and gastrointestinal tumors was presumably modulated by a set of COX2 signaling target genes (**Figure 3C**). Indeed, validation with CRC cells confirmed that both the protein and mRNA levels of COX2 were increased by DCLK1-B OE and decreased by DCLK1-B KO, DCLK1-A KD, or DCLK1 kinase inhibition (**Figure 3D** and Figure S4B-E), suggesting a strong relationship between DCLK1 and COX2. Collectively, these data suggest that COX2 is a multipotent target for the aggressive behavior of CRC cells, allowing us to focus on the potential relationship between DCLK1 and COX2.

To identify the downstream mechanisms through which DCLK1 regulates COX2 expression, potential DCLK1-B-interacting proteins were identified in protein extracts from HCT116 cells through co-immunoprecipitation with a monoclonal anti-DCLK1-B antibody followed by liquid chromatography with tandem mass spectrometry (LC-MS; Figure S5A). A total of 943 proteins were pulled down by the DCLK1-B antibody and were identified as potential DCLK1-B-interacting proteins (Table S4). Gene Ontology analysis with a web-based functional annotation platform, Database for Annotation, Visualization, and Integrated Discovery (DAVID) 24, revealed that phosphoproteins were one of the top clusters of DCLK1-interacting proteins. In particular among phosphoproteins, XRCC5 was initially found to repair DNA damage but was recently discovered to activate gene transcription 10, 18, 19. Thus, we further investigated the potential interaction between DCLK1 and XRCC5.

The DCLK1-XRCC5 interaction, especially the interaction with the phosphorylated form of XRCC5 at T715 25, was confirmed by immunoprecipitation (**Figure 3E**). Moreover, the phosphorylation status of XRCC5 was altered by modulating DCLK1 expression in multiple CRC cells, as indicated by the increase observed upon DCLK1-B OE and decrease detected upon DCLK1-B KO, DCLK1-B KD, or DCLK1-A KD (**Figure 3F** and Figure S5B). Additionally, inhibition of DCLK1 kinase activity by DCLK1-IN-1 treatment significantly reduced XRCC5 phosphorylation in CRC cells, while no alteration in the level of total XRCC5 was observed (**Figure 3F** and Figure S5C). Next, to determine the possible link between XRCC5 and COX2, we utilized a luciferase-tagged COX2 reporter and found that DCLK1-B OE transcriptionally activated COX2 expression and that XRCC5 KD diminished the DCLK1-induced transcription of COX2 (**Figure 3G**). Furthermore, to test whether the phosphorylation of XRCC5 is critical for the regulation of COX2 expression, we overexpressed three different forms of XRCC5 in HEK293T cells: WT, a phosphomimetic mutant form (active, T715D), and a non-phosphorylatable mutant form (inactive, T715A) 26. OE of the active mutant form of XRCC5 substantially induced COX2 reporter activity, while OE of the inactive mutant form of XRCC5 did not (**Figure 3H**). In parallel, immunofluorescence analysis confirmed that the active mutant form of XRCC5 preferentially translocated into the nucleus and increased COX2 expression (Figure S6), while the inactive mutant form did not. By performing chromatin immunoprecipitation assays, we confirmed that the phosphorylated form of XRCC5 directly binds at -300~-100 bp of the COX2 promoter region (**Figure 3I**). Collectively, these results indicate that DCLK1 binds and phosphorylates XRCC5, and thereby transcriptionally activates COX2 expression in CRC cells (**Figure 3J**). Intriguingly, this inhibitory effect of DCLK1-IN-1 on XRCC5 phosphorylation and COX2 expression was conserved across a broad range of cancer cell lines such as BT-474 (breast cancer), A549 (lung cancer), and PANC-1 (pancreas cancer), suggesting the versatility of the DCLK1/XRCC5/COX2 axis in many types of cancer (Figure S7).

### XRCC5 KD reduces CRC tumorigenesis and prostaglandin E_2_ (PGE_2_) production

**Figure 4:**
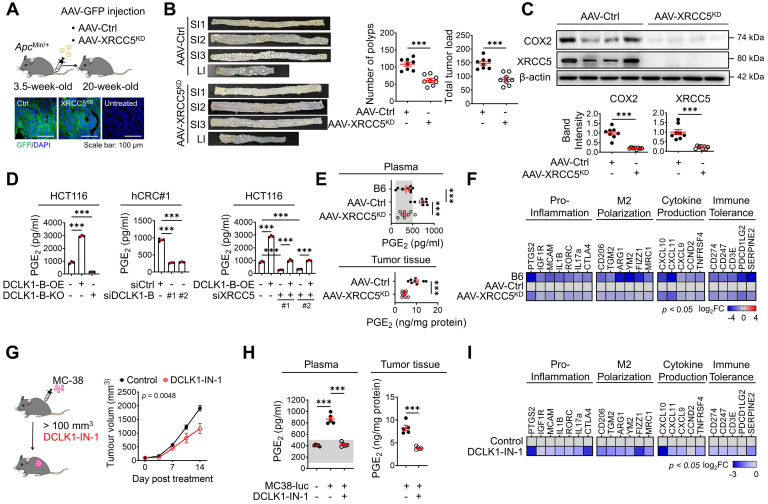
** DCLK1/XRCC5 axis shapes the pro-tumor microenvironment via COX2 signaling. (A)** Schematic illustration of the protocol for injection of shXRCC5-conjugated adeno-associated virus (AAV) into 3.5-week-old *Apc*^Min/+^ mice (top). Both the AAV-Ctrl and AAV-XRCC5^KD^ groups were monitored until the age of 20 weeks and sacrificed for analysis. Delivery of the virus was confirmed by GFP expression in the targeted organ, the intestine (bottom; n = 8 for both AAV-Ctrl and AAV-XRCC5^KD^). **(B)** The frequency of polyp formation in both the AAV-Ctrl and AAV-XRCC5^KD^ groups is presented. The number of polyps in each individual mouse was determined and used to calculate the total tumor burden. **(C)** Representative immunoblots of COX2 and XRCC5 expression in the AAV-Ctrl and AAV-XRCC5^KD^ groups. **(D)** *In vitro* ELISA showing the amount of PGE_2_ secreted into the cell culture medium upon DCLK1-B expression regulation and XRCC5 knockdown (n = 3 biological replicates). **(E)** Levels of secreted PGE_2_ in plasma and tumor tissue. Plasma was collected from B6, AAV-Ctrl, and AAV-XRCC5^KD^ mice. Tumor tissues were collected from intestinal polyps of AAV-Ctrl and AAV-XRCC5^KD^ mice (n = 8 biological replicates). The estimated normal range of the plasma PGE_2_ level was determined by the values obtained in B6 mice (shaded in gray). **(F)** Heatmap comparing the relative expression of pro-inflammation, M2 polarization-related, cytokine production-related, and immune tolerance-related genes in B6, AAV-Ctrl, and AAV-XRCC5^KD^ mice, as determined by RT-qPCR (n = 8 biological replicates). **(G)** Schematic illustration of *in vivo* experiments investigating the therapeutic effect of DCLK1-IN-1 in CRC tumorigenesis and resultant growth curves of tumors (n = 5 for both the vehicle and DCLK1-IN-1 treated group). Luciferase-labeled MC-38 cells (5x10^4^cells mixed with Matrigel/mouse) were injected subcutaneously into the inguinal folds of C57BL/6J (B6) mice prior to treatment. Once the mean volume of the xenograft tumors reached 100 mm^3^, 10 mg/kg of DCLK1-IN-1 was administered daily. Mice were tracked for a total of 21 days (14 days post treatment). **(H)** Level of PGE_2_ in plasma and tumor tissue. Plasma was collected from naïve B6 and tumor-bearing B6 mice. Tumor tissues were collected from vehicle and DCLK1-IN-1-treated tumor-bearing mice (n = 5 biological replicates). **(I)** RT-qPCR heat map of relative expression of pro-inflammation, M2 polarization, cytokine production, and immune tolerance-related genes in vehicle and DCLK1-IN-1-treated mice. The data are presented as the means ± SEMs. *** indicates *p* < 0.001. Statistical significance was determined by unpaired two-tailed Student's t-tests for comparisons between two groups and one-way ANOVA with Dunnett's multiple comparison test for comparisons among three or more groups. Two-way repeated-measures ANOVA followed by the Bonferroni post hoc test for comparison of tumor metastasis over time.

Next, we examined the biological function of XRCC5 and its contribution to COX2 expression during CRC tumorigenesis by knocking down XRCC5 using adeno-associated virus infection 16, 22 (**Figure 4A**). We utilized the adenomatous polyposis coli mutation-induced (*Apc*^Min/+^) mouse model, in which DCLK1 is markedly overexpressed in intestinal tumors (Figure S8A). Both DCLK1-A and DCLK1-B isoforms were expressed in mouse intestinal tumors, but unlike in human tumors, DCLK1-A was a predominant isoform in mouse tumors. XRCC5 KD caused significant decreases in the number of generated polyps and the tumor burden throughout the intestine (**Figure 4B** and Figure S8B), suggesting a critical role of XRCC5 in *Apc*^Min/+^-driven CRC tumorigenesis. In parallel, western blot and RT-qPCR analysis confirmed that XRCC5 KD substantially reduced COX2 expression at the protein levels in intestinal polyps (**Figure 4C**), confirming a link between XRCC5 and COX2 *in vivo*.

COX2 is known to produce PGE_2_, which plays a pivotal role in inflammation and cancer progression by shaping a TME permissive for tumor growth, including modulating inflammation and immune responses 27-29. Consistent with the experimental evidence that the COX2 expression is regulated by DCLK1 expression, we confirmed that PGE_2_ production is accompanied by an alteration in DCLK1 expression (**Figure 4D**) in CRC cells. In particular, XRCC5 KD and KO attenuated the DCLK1-induced upregulation of PGE_2_ production, suggesting that DCLK1 increases PGE_2_ production at least partially through XRCC5 (**Figure 4D**). Consistent with this finding, the elevated PGE_2_ levels in the plasma of *Apc*^Min/+^ mice compared to age-matched naïve B6 mice were dramatically reduced by XRCC5 KD (**Figure 4E**). Moreover, the PGE_2_ levels in the intestinal polyps of *Apc*^Min/+^ mice were reduced upon XRCC5 KD (**Figure 4E**). Furthermore, the transcriptional analysis showed that XRCC5 KD exhibited a global decreasing trend in the expression of PGE_2_-associated genes, such as those related to the pro-inflammatory response, M2 polarization, cytokines, and immune tolerance (**Figure 4F**), suggesting the effectiveness of XRCC5 KD in reversing the inflammatory TME.

Next, we examined whether inhibition of DCLK1 kinase activity exerted a therapeutic effect on COX2 signaling, similar to XRCC5 KD. To test the therapeutic effect of DCLK1-IN-1, we inoculated murine intestinal tumor cells (MC-38) into a syngeneic mouse model that is applicable for monitoring PGE_2_-induced microenvironmental changes, including changes in inflammation and immune responses. In this experiment, we used luciferase-tagged MC38 cells to monitor CRC growth by *in vivo* imaging. DCLK1-IN-1 treatment attenuated CRC growth in the MC38 syngeneic mouse model without inducing any significant changes in mouse body weight (**Figure 4G** and Figure S9A-B). No obvious clinical signs, including anorexia, salivation, diarrhea, vomiting, polyuria, anuria or fecal changes, were observed during DCLK1-IN-1 treatment (Figure S9C). In addition, inhibition of DCLK1 kinase activity by DCLK1-IN-1 treatment reduced the PGE_2_ level in both plasma and tumor tissues in MC38 syngeneic mice (**Figure 4H**). In parallel, DCLK1-IN-1 treatment resulted in a global decreasing trend in the expression of genes involved in the microenvironmental effects of PGE_2_ (**Figure 4I**). Intriguingly, when we inoculated murine breast cancer cells (4T1) into a syngeneic mouse model, we repeatedly confirmed the reduction in tumor growth induced by DCLK1-IN-1 treatment without any signs of side effects, along with significant reductions in PGE_2_ levels and gene expression associated with the microenvironmental effects of PGE_2_ (Figure S9D-I). Collectively, these results imply that inhibition of XRCC5 phosphorylation by limiting the kinase activity of DCLK1 efficiently reverses the PGE_2_-related inflammatory TME and suppresses CRC tumorigenesis.

### PGE_2_ produced by the DCLK1/XRCC5 axis enhances the intrinsic aggressiveness of CRC cells

**Figure 5:**
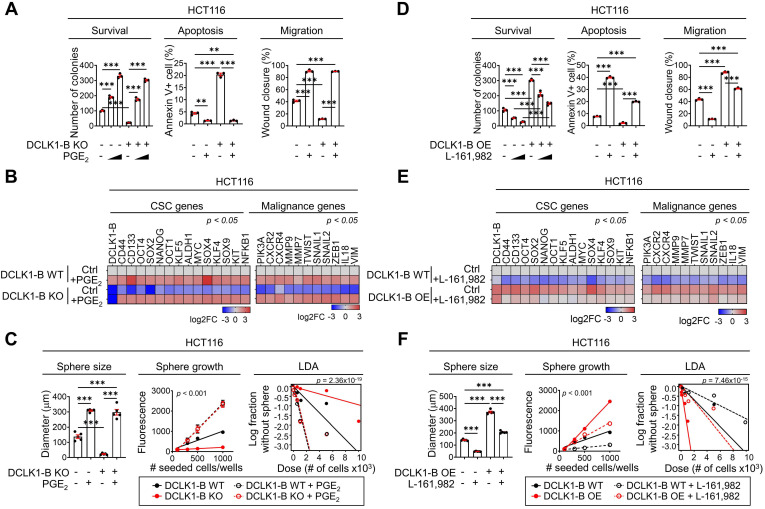
** PGE_2_ produced by the DCLK1/PGE_2_ axis mediates CRC aggressiveness. (A)** Series of biological functional assays showing the effects of the DCLK1-B/PGE_2_ axis on cancer cell survival, proliferation, apoptosis, and migration (n = 3 biological replicates). DCLK1-B WT and KO HCT116 cells were treated with PGE_2_ (2 µM and 3 µM). **(B)** Heatmap showing the relative expression of CSC genes and malignancy-related genes upon alteration of the DCLK1/PGE_2_ axis, as determined by RT-qPCR (n = 3 biological replicates). **(C)** Stemness was investigated with various methods, e.g., measurement of sphere size, sphere growth, and sphere-forming potential. Sphere size was analyzed by measuring the sphere diameter and fold change relative to the mean value of DCLK1-B WT spheres (n = 4 biological replicates). Growth was analyzed by a quantitative Cell Titer-Blue assay (n = 4 biological replicates). Sphere-forming potential was analyzed by an *in vitro* limiting dilution assay (n = 4 biological replicates). *p* values indicate statistical significance of differences in stem cell frequencies between any of the groups. **(D)** Series of biological functional assays showing the effects of the DCLK1-B/PGE_2_ axis on cancer cell survival, proliferation, apoptosis, and migration (n = 3 biological replicates). DCLK1-B WT and OE HCT116 cells were treated with the PGE_2_ receptor (EP4) inhibitor L-161,982 (1 µM and 3 µM). **(E)** Heatmap showing the relative expression of CSC genes and malignancy-related genes upon alteration of the DCLK1/PGE_2_ axis, as determined by RT-qPCR (n = 3 biological replicates). DCLK1-B WT and OE HCT116 cells were treated with L-161,982 (3 µM). **(F)** Stemness was assessed in DCLK1-B WT and OE HCT116 cells treated with L-161,982 (3 µM). *p* values indicate statistical significance of differences in stem cell frequencies between any of the groups. See panel (C) for details. The data are presented as the means ± SEMs. *, ** and *** indicate *p* < 0.05, *p* < 0.01, and *p* < 0.001, respectively. Statistical significance was determined by one-way ANOVA with Dunnett's multiple comparison test for comparisons among three or more groups and two-way repeated-measures ANOVA followed by the Bonferroni post hoc test for comparisons of sphere growth.

In addition to altering the microenvironmental effects, PGE_2_ is known to promote CSC expansion and metastasis during CRC tumorigenesis by binding to PGE receptor 4 (EP4) on the CRC cell surface 28-32. Thus, to examine the effect of PGE_2_ on the intrinsic behavior of CRC cells, we performed a series of validation assays. Treatment with PGE_2_ resulted in a significant enhancement in the malignant behaviors of CRC cells, as indicated by their increased survival and migration and decreased apoptosis (**Figure 5A** and Figure S10A-B, D-E). Notably, treatment with PGE_2_ restored the DCLK1-B KO- or KD-induced suppression of malignant behaviors (**Figure 5A** and Figure S10A-C, D-F). Consistent with this finding, PGE_2_ treatment resulted in global increases in the expression of malignancy-related and stemness genes in CRC cells, and it reversed the DCLK1-B KO- or KD-induced reductions in the expression of these genes (**Figure 5B** and Figure S10G). Moreover, the sphere-forming ability of CRC cells was significantly enhanced upon PGE_2_ treatment, and treatment with PGE_2_ reversed the reduction in sphere formation induced by DCLK1-B suppression (**Figure 5C** and Figure S10H-I). Consistently, treatment with a COX2-selective inhibitor, celecoxib, significantly reduced CRC cell migration and sphere-forming ability along with decreasing PGE_2_ levels (Figure S10J-L). More importantly, increases in CRC cell migration and the sphere-forming ability induced by DCLK1-B OE were significantly blocked by celecoxib treatment (Figure S10J-L). Collectively, these data suggest that DCLK1 promotes the aggressive phenotype of CRC cells at least partially through PGE_2_.

Next, we utilized the EP4 inhibitor L-161,982 31 to examine whether the PGE_2_-EP4 signaling cascade is involved in DCLK1-mediated CRC aggressiveness. Consistent with the results described above, we observed that the enhanced malignant behaviors of CRC cells induced by DCLK1-B OE were blocked by EP4 inhibition (Figure **5D** and Figure S11A-C). In parallel, the increases in malignancy-related and stemness gene expression induced by DCLK1-B OE were diminished upon EP4 inhibition (**Figure 5E**). Consistent with these findings, increase in the sphere-forming ability induced by DCLK1-B OE was significantly attenuated by EP4 inhibition in CRC cells (**Figure 5F** and Figure S11D). Collectively, our data suggest that PGE_2_/EP4 signaling is a key mediator of DCLK1-driven CRC cell aggressiveness. In parallel, recent studies have shown that PGE_2_ promotes CRC stemness through activating multiple signaling cascades by binding to PGE_2_ receptor 4 (EP4) on CRC cells 28. Additionally, PGE_2_/EP4 signaling is known to be responsible for CRC cell growth, anoikis resistance, and migration, facilitating cancer progression 29, 33. These previous reports support the relevance of our finding that PGE_2_ is a key mediator of DCLK1 which promotes the aggressive phenotype of CRC cells.

Considering that XRCC5 mediates DCLK1-driven PGE_2_ production in CRC cells, we tested whether XRCC5 plays an important role in the intrinsic aggressiveness of CRC cells. XRCC5 KD significantly attenuated the survival and migration of CRC cells and increased their apoptosis (Figure S11E). Notably, XRCC5 KD potently blocked the DCLK1-B OE-driven malignant behaviors of CRC cells (Figure S11E). Similarly, XRCC5 KD in CRC cells reversed the DCLK1-B OE-induced global increases in malignancy-related and stemness gene expression (Figure S11F) and the DCLK1-B OE-induced increase in sphere-forming ability (Figure S11G). Next, we tested whether XRCC5 phosphorylation is actually associated with DCLK1-B-induced aggressive behavior of CRC cells by using XRCC5 mutant forms. We overexpressed the phosphomimetic active XRCC5 (T715D) or phosphorylatable inactive XRCC5 (T715A) in DCLK1-B-KO CRC cells and examined their migration and sphere-forming abilities. Consequently, we found that OE of active XRCC5 blocked the DCLK1-B KO-induced reductions in CRC cell migration and sphere formation, while OE of inactive XRCC5 did not (Figure S11H-J). These results indicate that XRCC5 is a potential oncogenic factor that promotes the intrinsic aggressiveness of CRC cells, suggesting that XRCC5 may play a critical role in determining the tumorigenic capacity of CRC cells, as observed in the *Apc*^Min/+^ mouse model (**Figure 4A-B**).

### Inhibition of DCLK1 kinase activity efficiently attenuates stemness in CRC cells

**Figure 6:**
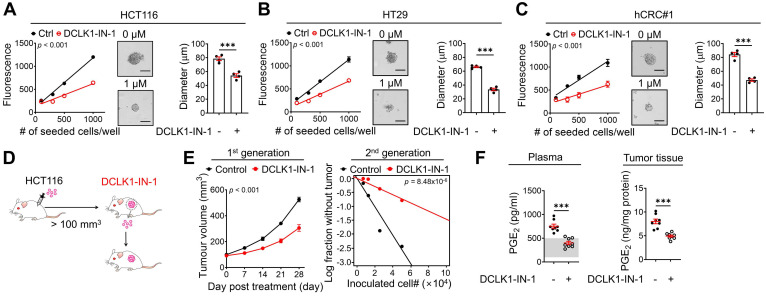
** DCLK1-IN-1 efficiently inhibits DCLK1/XRCC5/COX2 signaling and stemness in CRC. (A-C)** Alteration of stemness upon DCLK1-IN-1 treatment (1 μM) was investigated with various methods (n = 4 biological replicates), e.g., measurement of sphere growth and sphere size. Sphere growth was analyzed by a quantitative Cell Titer-Blue assay, while sphere size was analyzed by measuring the sphere diameter and fold change relative to the mean value of Ctrl spheres. (A) HCT116, (B) HT29 and (C) hCRC#1. **(D)** Schematic illustration of the protocol for *in vivo* treatment with the DCLK1 inhibitors, DCLK1-IN-1. HCT116 cells (1x10^6^ cells mixed with Matrigel/mouse) were injected subcutaneously into the inguinal folds of NSG mice prior to treatment. Once the mean volume of the xenograft tumors reached 100 mm^3^, 10 mg/kg of DCLK1-IN-1 was administered daily. Mice were tracked for 32 days after DCLK1 inhibitor injection (n = 8 mice per group). **(E)** Growth curves of the first tumor generation (left) and *in vivo* limiting dilution assay (right) comparing the tumor-repopulating potential of the second generation between the Ctrl and DCLK1-IN-1 treatment groups. **(F)** Levels of PGE_2_ in plasma and tumor tissue. Plasma and tumor tissues were collected from Ctrl and DCLK1-IN-1-treated NSG tumor-bearing mice. The estimated normal range of the plasma PGE_2_ level was determined by the values obtained in naïve mice (shaded in gray). The data are presented as the means ± SEMs. *** indicates *p* < 0.001. Statistical significance was determined by unpaired two-tailed Student's t-tests for two-group comparisons, one-way ANOVA with Dunnett's multiple comparison test for three-group comparisons, and two-way repeated-measures ANOVA followed by the Bonferroni post hoc test for tumor growth comparisons.

Next, to examine whether inhibition of DCLK1 kinase activity shows promising efficacy against the CSC behavior of CRC cells, we performed *in vitro* and *in vivo* experiments using DCLK1-IN-1. Notably, DCLK1-IN-1 treatment significantly reduced the self-renewal ability of CRC cells, as indicated by the decreases in sphere growth and size (**Figure 6A-C**). Consistent with the *in vitro* results, a series of transplantation assays performed in HCT116 xenograft mouse models (**Figure 6D**) showed that DCLK1-IN-1 treatment attenuated both primary and secondary tumor growth without any significant changes in mouse body weight (Figure S12A) or, obvious clinical signs, including anorexia, salivation, diarrhea, vomiting, polyuria, anuria and fecal alterations and serological parameters of liver and kidney toxicity (Figure S12B). Consistent with the deceleration of tumor growth, a significant reduction in tumor-repopulating potential was observed upon DCLK1-IN-1 treatment (**Figure 6E** and Figure S12C). In parallel, the DCLK1-IN-1 treatment dramatically reduced the PGE_2_ levels in plasma and primary tumor tissue (**Figure 6F**). Additionally, to build up the basic data for further drug development, we tested the *in vitro* and *in vivo* efficacies of a new compound, DDQ-1 (a dimethyldihydroisoquinoline derivative, Figure S13A), which we discovered by an *in silico* virtual 3D screen using the Korea Chemical Bank Database (Daejeon, Republic of Korea) based on the X-ray crystal structure of the DCLK1 kinase domain (Protein Data Bank ID 5JZN) 34. Similar to DCLK1-IN-1, DDQ-1 displayed potent activity against DCLK1 kinase activity (IC_50_ = 3 nM), CRC cell growth (HCT116 and hCRC#1 cells, with IC_50_ = 0.685 µM and 0.653 µM, respectively), and the XRCC5/COX2/PGE_2_ signaling cascade (Figure S13B-E). Notably, DDQ-1 treatment significantly reduced the self-renewal ability of CRC cells (Figure S13F), and attenuated both primary and secondary tumor growth (Figure S13G-H) without any obvious clinical signs of toxicity (Figure S13I-J), with the decreased tumor-repopulating potential (Figure S13K-L) and reduced PGE_2_ levels in plasma and primary tumor tissue (Figure S13M). Collectively, our data indicate that targeting DCLK1 kinase activity exert promising effects on CRC stemness, suggesting that DCLK1 may be a promising therapeutic target for aggressive CRC, and subsequent pharmacophore studies using the DCLK1 crystal structure would be valuable for new drug discovery.

## Discussion

Here, our current study proposes a novel feedback loop that may reflect the reciprocal interaction between CSCs and inflammation. CSCs play an active role throughout all stages of tumorigenesis by constantly changing their phenotypes as an adaptive response to dynamic changes in the TME 12. The plasticity of the CSC phenotype seems to be achieved by close interactions among various cellular or non-cellular components of the TME, which alter the intrinsic behavior of CSCs. However, accumulating evidence also suggests an extrinsic role of CSCs in facilitating TME remodeling by secreting ligands that activate various TME components 12, 35-41. In this context, we propose a novel molecular mechanism connecting a selective intestinal CSC marker, DCLK1, and a major inflammatory mediator, PGE_2_. DCLK1 enhances CRC-secreted PGE_2_ through XRCC5 phosphorylation, thence, generates a pro-inflammatory TME and intrinsic aggressiveness of CRC cells. Thus, our discovery of the DCLK1/XRCC5/COX2/PGE_2_ axis highlights a new extrinsic function of CSCs in shaping the inflammatory TME, which broadens the importance of CSCs in TME remodeling.

This study highlighted the indispensable role of the kinase domain of DCLK1 in CRC aggressiveness. As an effort to expand the understanding the biological function of DCLK1 in cancer specifically from the perspectives of the kinase domain has been recently a matter 15, 16, 22. To consolidate our understanding of kinase activity dependent biological functions in cancer, a selective DCLK1 inhibitor, DCLK1-IN-1 was used. Initially, DCLK1-IN-1 10 was developed from the core structure of multi-targeted kinase inhibitors LRRK2-IN-1 42, XMD8-92 43 and XMD8-85 44. Experimentally, our *in vitro* and *in vivo* findings confirmed that DCLK1 kinase activity promotes cancer cell survival, aggressiveness and stemness which were previously mainly determined through the genetic manipulation of DCLK1 expression 19, 45-51. Moreover, recent global genomics and proteomics profiling of DCLK1-IN-1 have revealed new functions of DCLK1 kinase, such as RNA processing, insulin signaling, ErbB (erythroblastic leukemia viral oncogene homolog) signaling, and proteoglycan synthesis 52. In this context, our IP-proteomics and subsequent functional annotation analysis revealed that collective RNA processings were listed as one of the top clusters of DCLK1-interactin proteins which is compatible with a previous report by Liu et al. 52. Moreover, our KEGG analysis using the RNA-seq data (Figure S14) repeatedly confirmed that DCLK1 might be positively linked to several malignant signaling pathways, some of which were compatible with previous reports 46, 52-55.

Importantly, this study is the first to identify XRCC5 as a mediator of DCLK1-driven COX2 expression. XRCC5 was originally reported to repair double-strand breaks in DNA; thus, it was considered to fuel therapeutic resistance to DNA-damaging agents in cancer 56-64. In this context, our study identified a novel function of XRCC5 as a regulator of gene expression. The active, phosphorylated form of XRCC5 was preferentially translocated into the nucleus and bound to the promoter region of COX2 to transcriptionally activate COX2 expression in CRC cells in the present study. Moreover, our study provided *in vivo* evidence that XRCC5 plays an essential role in intestinal tumorigenesis by contributing to COX2 and PGE_2_ upregulation in tumor tissues (**Figure 4**). To confirm the versatility of our findings across various cancer cells, we performed additional analyses and found that the DCLK1/XRCC5/COX2 axis is conserved in many types of solid cancer cell lines including breast, lung and pancreatic cell lines (Figure S7).

Moreover, this study provides insight into the novel function of DCLK1 in promoting the inflammatory TME. Because the COX2/PGE_2_ pathway is one of the key inflammatory mediators that sculpt the tumor-promoting microenvironment 65, 66, recent studies revealed that the inhibition of DCLK1 kinase activity significantly increases T cell-mediated anti-cancer immunity, but a definitive molecular mechanism has not been identified 67, 68. In this context, our discovery of the DCLK1/XRCC5/COX2 axis in mouse models of both colorectal cancer (**Figure 4**) and breast cancer (Figure S9) suggests a new clue for mechanistic insight into the TME-regulating role of CSCs and may broaden the preclinical rationale for expanding the clinical trials of DCLK1 inhibitors for cancer treatment.

DCLK1 has two distinct isoforms; DCLK1-A, which contains both the tandem DCX domains at the N-terminus and a kinase domain at the C-terminus, and DCLK1-B, which contains only the kinase domain at the C-terminus 69-72. Intriguingly, different tumor-specific isoform signatures have been observed in various cancers 4, 73-76 including colorectal cancer 8, 47, 77-80, although the mechanism underlying the expression of different isoforms remains to be elucidated. In terms of the controversy surrounding the functions of DCLK1 isoforms specifically in CRC, we provided fundamental evidence that both DCLK1-A and DCLK1-B, play a critical role in CRC aggressiveness (**Figure 2** and Figure S3). Additionally, inhibition of the DCLK1 kinase domain, which exists in both DCLK1-A and -B isoforms, significantly attenuated the aggressive phenotype of CRC cells (**Figure 2**). Despite the lack of mechanistic studies dissecting the different expression of these isoforms, our study has shown a prompt role of DCLK1 in tumorigenesis, hence, highlighting the DCLK1 kinase domain as an attractive target regardless of the isoform.

Finally, we provided new pharmacophore information for targeting the DCLK1 kinase domain, but additional drug development with finer tuning is still necessary for the development of more precise therapeutic interventions, despite the promising efficacy of DCLK1-IN-1. Thus, to build up the basic data for further drug development, we discovered a new compound, DDQ-1 (Figure S13). Docking simulation with CDOCKER in the Discovery Studio program (Accelrys, San Diego, CA, USA) showed that DDQ-1 adopts a type I kinase inhibitor binding mode, forming two hydrogen bonds with the hinge V468 residue and charge interactions with the side chains of D472 and D475, while the sulfonyl group on DDQ-1 interacts with the side chain of K419 by hydrogen bonding. In addition, several π-alkyl interactions, with I396, V404, A417, and L518, were identified. Because we observed the promising molecular efficacy of DDQ-1 with even more potent activity against DCLK1 kinase activity, and therapeutic efficacy against CRC stemness via the XRCC5/COX2 signaling axis, further investigations using DDQ-1 and its derivatives may be highly informative for identifying and optimizing a better pharmacophore for DCLK1 inhibition.

In summary, we discovered a pro-inflammatory function of DCLK1 in driving CRC aggressiveness via the XRCC5/COX2/PGE_2_ axis, which broadens the importance of DCLK1 in CRC pathogenesis, thus supporting the development of additional DCLK1 inhibitors for CRC treatment. Moreover, given the recalcitrance of advanced CRC in general, the therapeutic approach of DCLK1 kinase inhibition may have the potential to overcome the limitation of current therapies by suppressing the cancer stemness and inflammatory TME that fuel fatal recurrence and metastasis.

## Supplementary Material

Supplementary figures and tables.

## Abbreviations

APC: adenomatous polyposis coli
COX2: Cyclooxygenase-2
CRC: Colorectal cancer
CSC: Cancer stem cell
DAVID: Database for annotation, visualization, and integrated discovery
DCLK1: Doublecortine-like kinase 1
DEG: Differentially expressed gene
EpCAM: Epithelial cell adhesion molecule
GFP: Green fluorescent protein
GSEA: Gene set enrichment analysis
IC_50_: Half-maximal inhibitory concentration
IPA: Ingenuity pathway analysis
PGE_2_: Prostaglandin E_2_
EP4: PGE receptor 4
TME: tumor microenvironment
XRCC5: X-ray repair cross-complementing 5
