# Supplementary material for: DCLK1 promotes colorectal cancer stemness and aggressiveness via the XRCC5/COX2 axis
Source: Theranostics. 2022 Jul 4;12(12):5258–71. doi: 10.7150/thno.72037 (PMC9330537; doi:10.7150/thno.72037)
Supplement: Supplementary file 1 — Supplementary figures and tables. [file thnov12p5258s1.pdf]

Supplementary Information

DCLK1 promotes colorectal cancer stemness and aggressiveness via the XRCC5/COX2 axis

Jee-Heun Kim[1,*], So-Yeon Park[1,2,*,†], So-El Jeon[1], Jang-Hyun Choi[1], Choong-Jae Lee[1], Tae-Young Jang[1], Hyeon-Ji Yun[1], Yuno Lee[3], Pilho Kim[4], Sang Hee Cho[5], Ji Shin Lee[6], Jeong-Seok Nam[1,2#]

[*]Equal contribution

[1]School of Life Sciences, Gwangju Institute of Science and Technology, Gwangju, 61005, Republic of Korea

[2]Cell Logistics Research Center, Gwangju Institute of Science and Technology, Gwangju, 61005, Republic of Korea

[3]Korea Chemical Bank, Korea Research Institute of Chemical Technology, Daejeon, 34114, Republic of Korea

[4]Therapeutics and Biotechnology Division, Korea Research Institute of Chemical Technology, Daejeon, 34114, Republic of Korea

[5]Department of Hemato-Oncology, Chonnam National University Medical School, Gwangju, 61469, Republic of Korea

[6]Department of Pathology, Chonnam National University Medical School, Gwangju, 61469, Republic of Korea

[†]Current address: Institute for Basic Scienc, Center for Genome Engineering, 55, Expo-ro, Yuseong-gu, Daejeon, Korea, 34126

[#]**Corresponding Author:** Jeong-Seok Nam

School of Life Sciences, Gwangju Institute of Science and Technology, Gwangju, 61005, Republic of Korea

E-mail: namje@gist.ac.kr

Phone: +82 62 715 2893

Fax: +82 62 715 2484

Supplementary figures

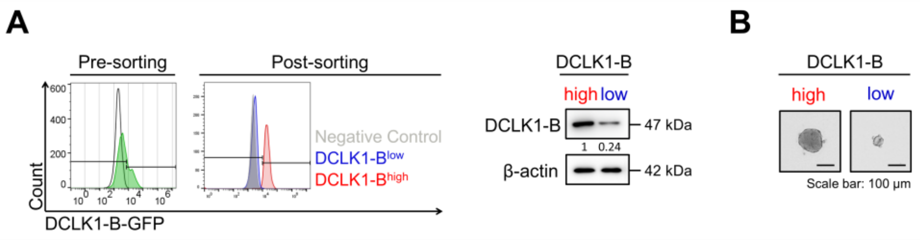

Figure S1 (related to Figure 1).

(A) FACS analysis of sorted DCLK1-B^{high} and DCLK1-B^{low} HCT116 cells (left), and immunoblot of DCLK1-B expression (right) in both cell populations. Relative band intensity of target protein in DCLK1-B^{low} cell population was normalized to β-actin in the DCLK1-B^{high} cell population. (B) Representative images of formed spheres of both DCLK1-B^{high} and DCLK1-B^{low}. The data are presented as the means ± SEMs. *** indicates $p < 0.001$. Statistical significance was determined by unpaired two-tailed Student's t tests for comparisons between two groups.

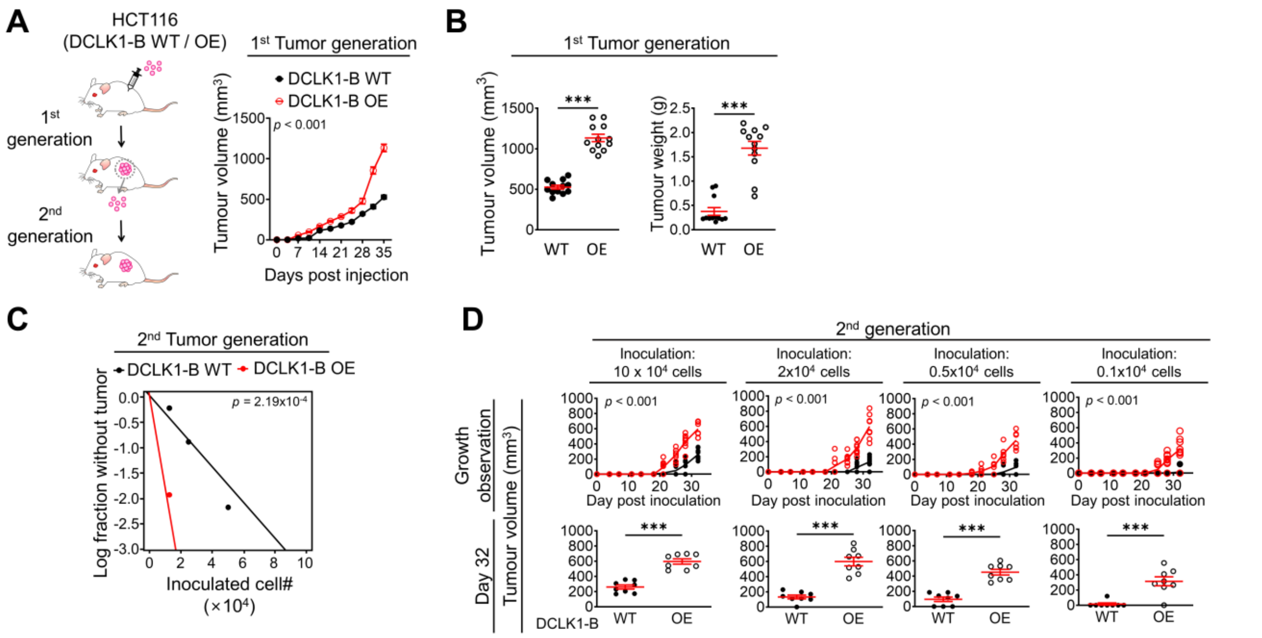

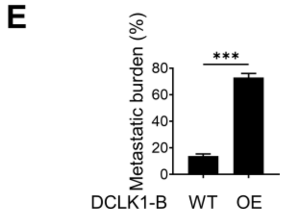

Frequency of tumour formation

Cell no. of inoculation	10x10⁴ WT	10x10⁴ OE	2x10⁴ WT	2x10⁴ OE	0.5x10⁴ WT	0.5x10⁴ OE	0.1x10⁴ WT	0.1x10⁴ OE	p-values
Day4	0% (0 of 8)	0% (0 of 8)	0% (0 of 8)	0% (0 of 8)	0% (0 of 8)	0% (0 of 8)	0% (0 of 8)	0% (0 of 8)	-
Day7	0% (0 of 8)	0% (0 of 8)	0% (0 of 8)	0% (0 of 8)	0% (0 of 8)	0% (0 of 8)	0% (0 of 8)	0% (0 of 8)	-
Day11	0% (0 of 8)	0% (0 of 8)	0% (0 of 8)	0% (0 of 8)	0% (0 of 8)	0% (0 of 8)	0% (0 of 8)	0% (0 of 8)	-
Day14	0% (0 of 8)	0% (0 of 8)	0% (0 of 8)	0% (0 of 8)	0% (0 of 8)	0% (0 of 8)	0% (0 of 8)	0% (0 of 8)	-
Day18	0% (0 of 8)	0% (0 of 8)	0% (0 of 8)	0% (0 of 8)	0% (0 of 8)	0% (0 of 8)	0% (0 of 8)	0% (0 of 8)	-
Day21	13% (1 of 8)	63% (5 of 8)	0% (0 of 8)	38% (3 of 8)	0% (0 of 8)	13% (1 of 8)	0% (0 of 8)	0% (0 of 8)	2.09E-03
Day25	38% (3 of 8)	100% (8 of 8)	0% (0 of 8)	88% (7 of 8)	0% (0 of 8)	63% (5 of 8)	0% (0 of 8)	38% (3 of 8)	3.16E-09
Day28	75% (6 of 8)	100% (8 of 8)	38% (3 of 8)	100% (8 of 8)	25% (2 of 8)	100% (8 of 8)	0% (0 of 8)	88% (7 of 8)	1.28E-10
Day32	100% (8 of 8)	100% (8 of 8)	88% (7 of 8)	100% (8 of 8)	63% (5 of 8)	100% (8 of 8)	13% (1 of 8)	88% (7 of 8)	2.19E-04

Figure S2 (related to Figure 1).

(A) Schematic illustration of *in vivo* experiments investigating the tumorigenic effect of DCLK1-B on the first-generation and second-generation xenograft tumors (left), and growth curves of first tumor generation (right). For first *in vivo* tumor generation, DCLK1-B WT and DCLK1-B OE HCT116 cells (1x10^6 cells/mouse mixed with Matrigel) were injected into the inguinal folds of NSG mice. Mice were tracked for 35 days after the subcutaneous injection (n = 12 mice per each group). (B) Final volume and weight of first generation tumors at 35 days post-injection. (C) and (D) After 35 days, first generation xenografted tumors were removed and dissociated into single cells to reinject into NSG mice (10x10^4, 2x10^4, 0.5x10^4, 0.1x10^4, n = 8/group) for *in vivo* LDA. The regrowth of tumors was monitored for 32 days. On day 32 after reinjection, mice were sacrificed and the incidence of tumor-bearing mice was determined. (C) *In vivo* LDA comparing the tumor-repopulating potential between DCLK1-B WT and DCLK1-OE inoculations. Statistical significance was determined by the extreme LDA webtool (http://bioinf.wehi.edu.ac/software/elda). (D) Second generation tumor growth was monitored for 32 days and at 32 days post-reinjection, final tumor volumes were compared. (E) Quantitative analysis of metastatic burden in microscopic level. The data are presented as the means ± SEMs. *** indicates $p < 0.001$. Statistical significance was determined by unpaired two-tailed Student's t tests for comparisons between two groups and by two-way repeated-measures ANOVA followed by the Bonferroni post hoc test for multiple comparisons of tumor growth over time.

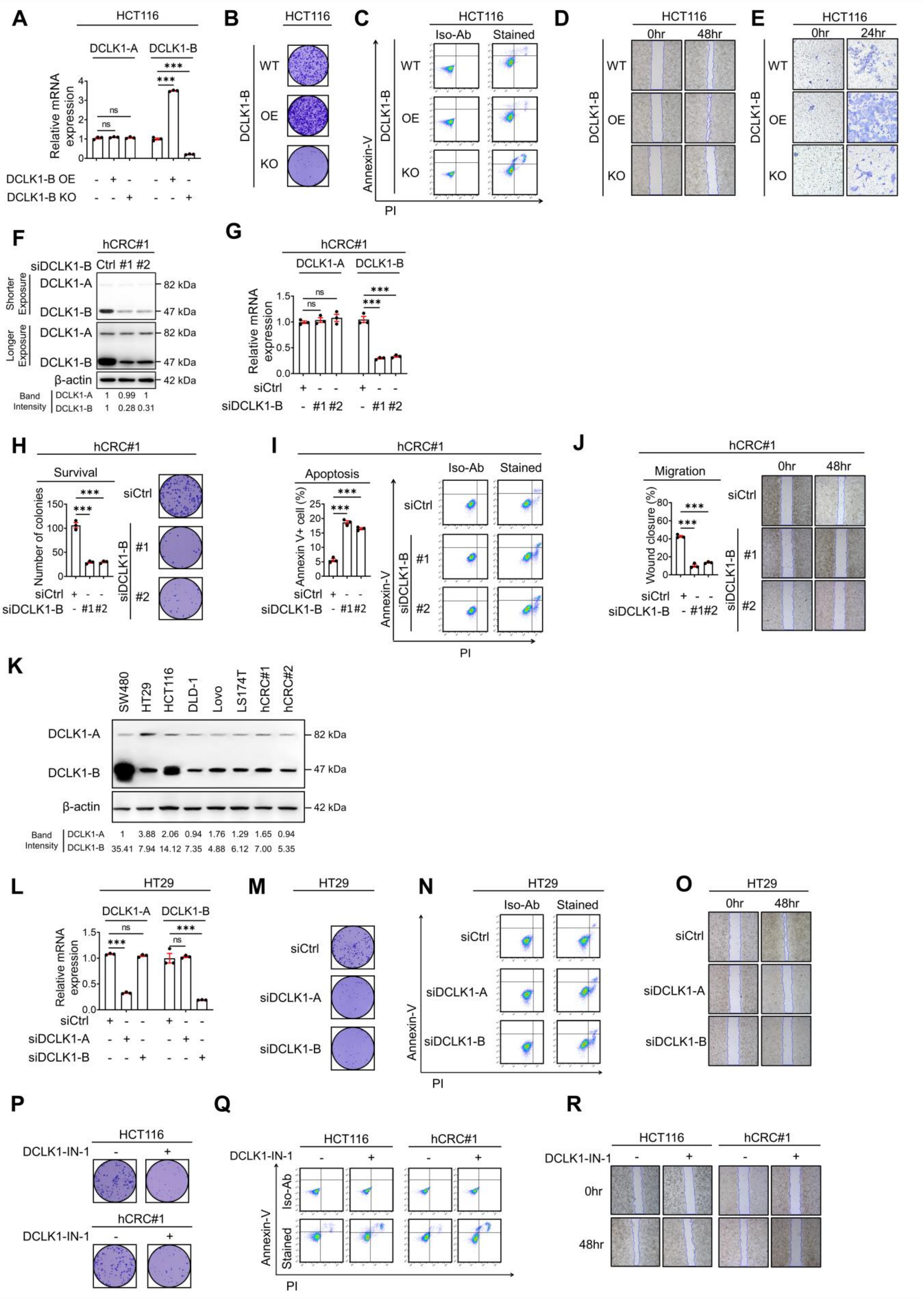
A
HCT116
DCLK1-A
DCLK1-B
Relative mRNA expression
DCLK1-B OE
DCLK1-B KO
B
HCT116
DCLK1-B
WT
OE
KO
C
HCT116
Iso-Ab
Stained
Annexin-V
PI
D
HCT116
0hr
48hr
E
24hr
F
hCRC#1
siDCLK1-B
Shorter Exposure
Longer Exposure
β-actin
82 kDa
47 kDa
42 kDa
Band Intensity
G
siCtrl
H
Survival
Number of colonies
I
Apoptosis
Annexin V+ cell (%)
J
Migration
Wound closure (%)
K
SW480
HT29
DLD-1
Lovo
LS174T
hCRC#2
L
siDCLK1-A
M
N
O
P
DCLK1-IN-1
Q
R

Figure S3 (related to Figure 2).

(A) Altered expression of DCLK1-A and DCLK1-B upon DCLK1-B OE and KO (n = 3 biological replicates). (B)-(E) Representative images of altered biological functions upon DCLK1-B OE and KO in HCT116 cell lines. (B) Representative image of surviving colonies from clonogenic assay. (C) Representative flow cytometry 2D scatter plots showing cells doubly stained with PI and Annexin-V for apoptotic analysis. The gates were established by staining with corresponding isotype antibody. (D) Representative images of wound closure assay 0 h and 48 h after IBIDI detachment. (E) Representative images of invasion assay at the time point of 24 h. (F) Immunoblot of DCLK1-A and DCLK1-B expression upon DCLK1-B KD by siRNA transfection. (G) Altered mRNA expression of DCLK1-A and DCLK1-B upon siDCLK1-B transfection in hCRC#1 cell lines (n = 3 biological replicates). (H)-(J) Representative images of altered biological functions upon DCLK1-B expression in hCRC#1 cells. (H) Representative image of surviving colonies from clonogenic assay. (I) Representative flow cytometry 2D scatter plots showing cells doubly stained with PI and Annexin-V for apoptotic analysis. The gates were established by staining with corresponding isotype antibody. (J) Representative images of wound closure assay 0 h and 48 h after IBIDI detachment. (K) Immunoblot of DCLK1-A and DCLK1-B expression in human colorectal cell lines (SW480, HT29, HCT116, DLD1, Lovo and LS174T) and colorectal patient-derived cell lines (hCRC#1 and hCRC#2). (L) Relative mRNA expression of DCLK1-A and DCLK1-B in HT29 cells upon siDCLK1-A or siDCLK1-B transfection (n = 3 biological replicates). (M)-(O) Representative images of altered biological functions upon alteration of DCLK1-A and DCLK1-B expression in HT29 cells (n = 3 biological replicates). (M) Surviving colonies from clonogenic assay. (N) Flow cytometry 2D scatter plots (right) showing cells doubly stained with PI and Annexin-V for apoptotic analysis. The gates were established by staining with corresponding isotype antibody. (O) Wound closure assay 0 h and 48 h after IBID detachment. (P) Representative images of surviving colonies from clonogenic assay upon DCLK1-IN-1 treatment (1 μM) in HCT116 (top) and hCRC#1 (bottom) cells. (Q) Representative flow cytometry 2D scatter plots showing HCT116 and hCRC#1 cells doubly stained with PI and Annexin-V for apoptotic analysis upon DCLK1-IN-1 treatment (1 μM). The gates were established by staining with corresponding isotype antibody. (R) Representative images of wound closure assay in HCT116 and hCRC#1 cells 0 h and 48 h after IBIDI detachment upon DCLK1-IN-1 treatment (1 μM). The data are presented as the means ± SEMs. *** indicates $p < 0.001$. Statistical significance was determined by unpaired two-tailed Student's t tests for comparisons between two groups and by one-way ANOVA with Dunnett's multiple comparison test for comparisons among three or more groups.

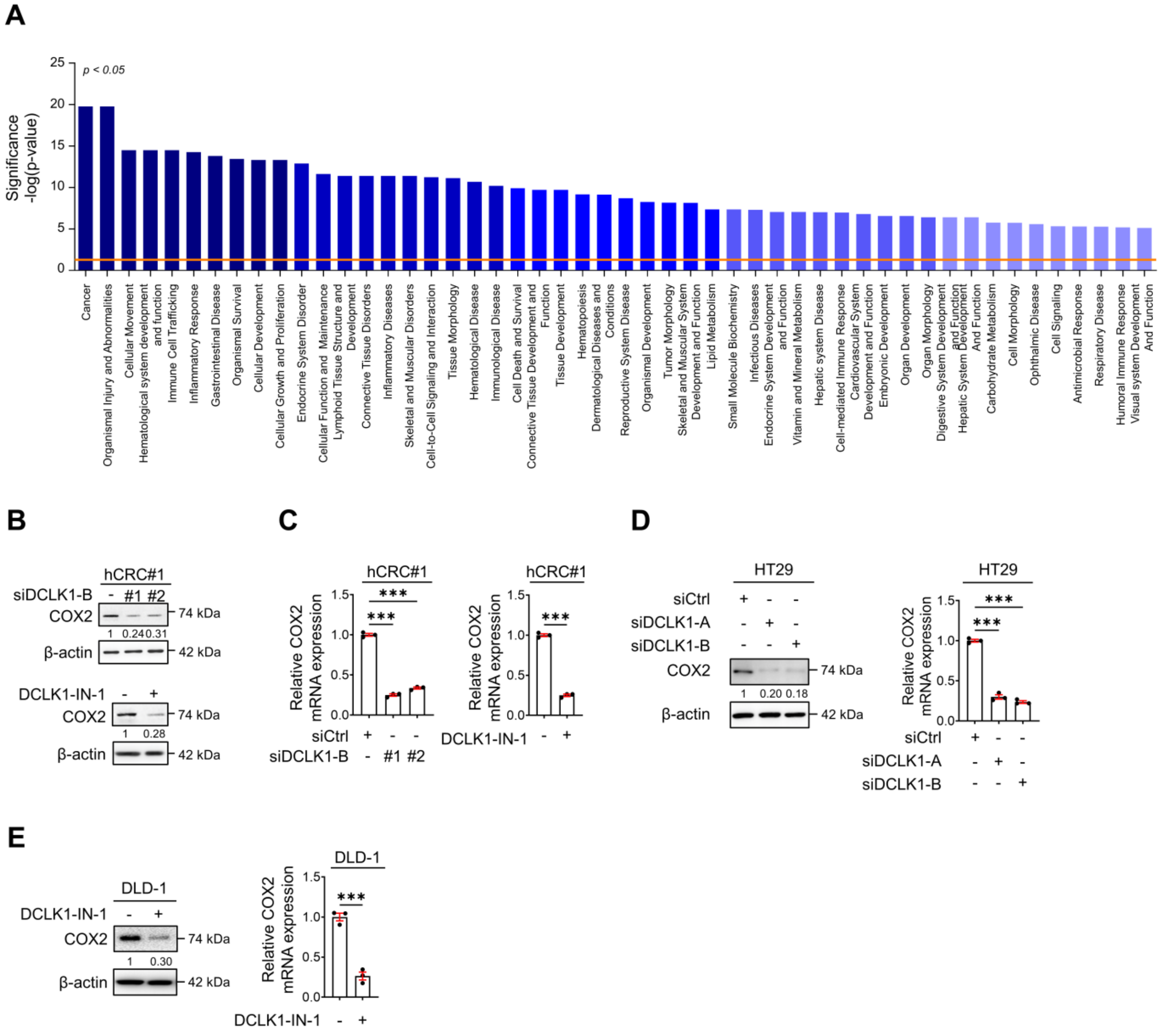

Figure S4 (related to Figure 3).

(A) Disease and function analyses show the top 50 affected categories according to IPA analysis upon DCLK1-B KO. Categories with $p < 0.05$ and |z-score| > 2 were considered statistically significant. (B) Alteration of COX2 expression upon DCLK1-B KD (top) and DCLK1-B kinase domain inhibition (bottom) in hCRC#1 cells. (C) Alteration of COX2 mRNA expression upon DCLK1-B expressional regulation (left) and DCLK1-B kinase domain inhibition (right) (n = 3 biological replicates). (D) Alteration of COX2 expression upon DCLK1-A and DCLK1-B KD in HT29 cells. (n = 3 biological replicates). (E) Alteration of COX2 protein and mRNA expression upon DCLK1-B kinase domain inhibition (1 μM) in DLD-1 cells. The data are presented as the means ± SEMs. *** indicates $p < 0.001$. Statistical significance was determined by unpaired two-tailed Student's t tests for comparisons between two groups and by one-way ANOVA with Dunnett's multiple comparison test for comparisons among three or more groups.

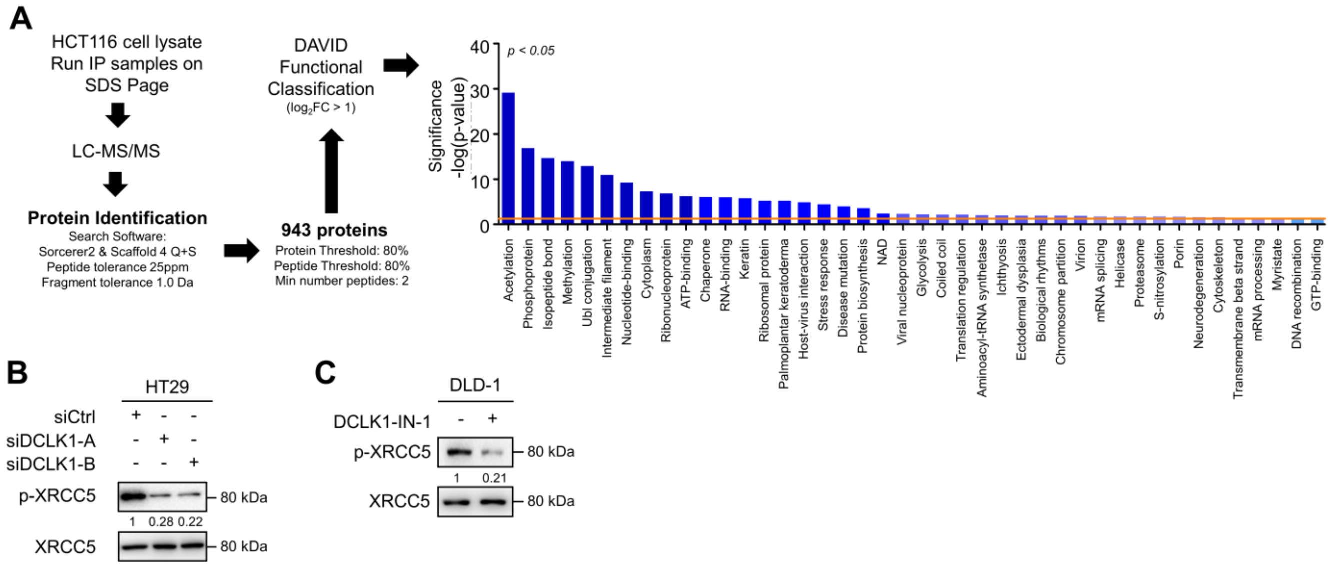

Figure S5 (related to Figure 3).

(A) Schematic flow for identification of DCLK1-B binding proteins based on Co-IP with anti-DCLK1-B monoclonal antibody and subsequent liquid chromatography with tandem mass spectrometry (LC-MS). LC-MS identified a total 943 proteins in the co-IP samples. Functional annotation was performed by DAVID functional classification analysis. Categories with $p < 0.05$ were considered statistically significant. (B) Immunoblot of phospho (p)-XRCC5 and total XRCC5 upon DCLK1-A and DCLK1-B KD independently. (C) Immunoblot of p-XRCC5 and total XRCC5 upon DCLK1-B kinase domain inhibition (1 μM).

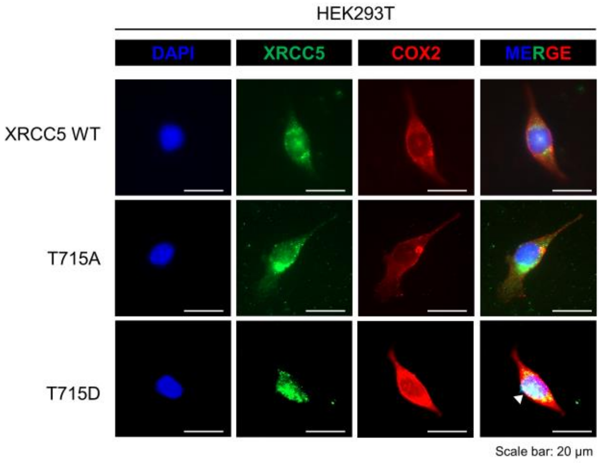

Figure S6 (related to Figure 3).

Immunofluorescence analysis of XRCC5 and COX2 localization upon OE of ectopic WT, non-phosphorylatable (inactive, T715A) and phosphomimetic (active, T715D) XRCC5. Columns show immunofluorescence staining for XRCC5 (green), COX2 (red), and DAPI (blue) with corresponding merged images. Scale bars, 20 µm.

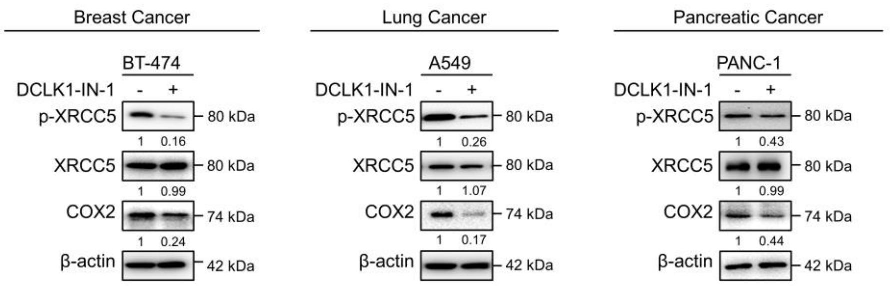

Figure S7 (related to Figure 3).

Immunoblot of COX2, p-XRCC5 and XRCC5 upon DCLK1-IN-1 treatment (1 μM) in various cancer cell lines; breast cancer cell line (BT-474), lung cancer cell line (A549) and pancreatic cancer cell line (PANC-1). Relative band intensity of target protein was normalized to β-actin as fold of the non-treated group.

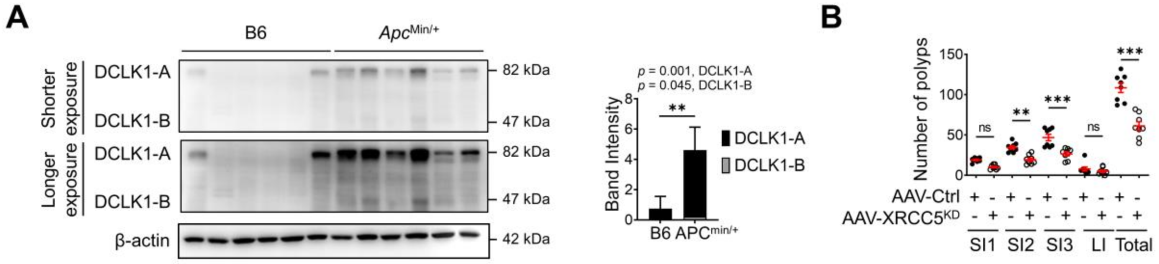

Figure S8 (related to Figure 4).

(A) Immunoblot of DCLK1-A and DCLK1-B expression in the intestine of B6 and *Apc*$^{Min/+}$ mice. Quantification of band intensity is shown in the bar graph. (B) Frequencies of polyp formation in each section of intestine (SI, small intestine; LI, large intestine) from both AAV-Ctrl and AAV-XRCC5^{KD} groups are presented along with total number of polyps (n = 9 for both AAV-Ctrl and AAV-XRCC5^{KD}). The data are presented as the means ± SEMs. *, ** and *** indicate $p < 0.05$, $p < 0.01$, and $p < 0.001$, respectively. Statistical significance was determined by unpaired two-tailed Student's t tests for comparisons between two groups and one-way ANOVA with Dunnett's multiple comparison test for comparisons among three or more groups.

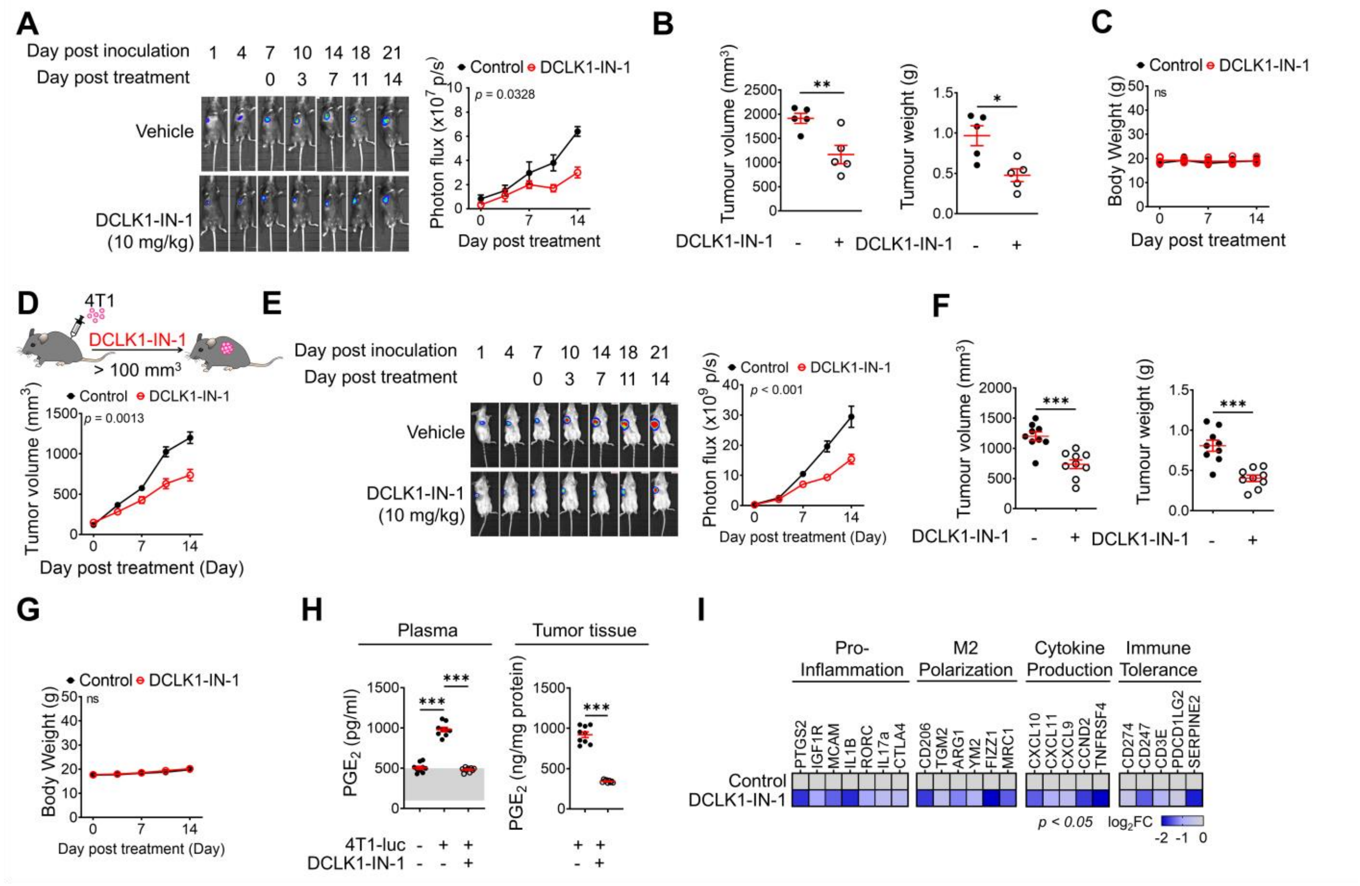

Figure S9 (related to Figure 4).

(A) *In vivo* imaging and graphing of tumor growth. (B) After 14 days of treatment, final volume and weight of tumors were measured and compared between the vehicle and DCLK1-IN-1 treated group. (C) Body weight was measured throughout DCLK1-IN-1 treatment to monitor any adverse effects. (D) Schematic illustration of *in vivo* experiments investigating the therapeutic effect of DCLK1-IN-1 in breast cancer syngeneic model. Luciferase-labeled 4T1 cells (1x10^5 cells/mouse mixed with Matrigel) were injected into BALB/c mice prior to treatment. Once the mean volume of xenograft tumors reached 100 mm^3, 10mg/kg of DCLK1-IN-1 was administered daily. Resultant growth of tumors were tracked for a total of 21 days (14 days post treatment). (n = 8 for both the vehicle and DCLK1-IN-1 treated group). (E) *In vivo* imaging and graphing of tumor growth. (F) After 14 days of treatment, final volume and weight of tumors were measured and compared between the vehicle and DCLK1-IN-1 treated groups. (G) Body weight was measured throughout DCLK1-IN-1 treatment to monitor any adverse effects. (H) Level of PGE_2 in plasma and tumor tissue. Plasma was collected from naïve BALB/c and tumor-bearing BALB/C mice. Tumor tissues were collected from vehicle and DCLK1-IN-1-treated tumor-bearing mice. (n = 8 biological replicates). (I) RT-qPCR heat map of relative expression of pro-inflammation, M2 polarization, cytokine production, and immune tolerance-related genes in vehicle and DCLK1-IN-1-treated mice. The data are presented as the means ± SEMs. *, ** and *** indicate $p < 0.05$, $p < 0.01$, and $p < 0.001$, respectively. Statistical significance was determined by unpaired two-tailed Student's t tests for comparisons between two groups, one-way ANOVA with Dunnett's multiple comparison test for comparisons among three or more groups, and two-way repeated-measures ANOVA followed by the Bonferroni post hoc test for comparison of tumor metastasis over time.

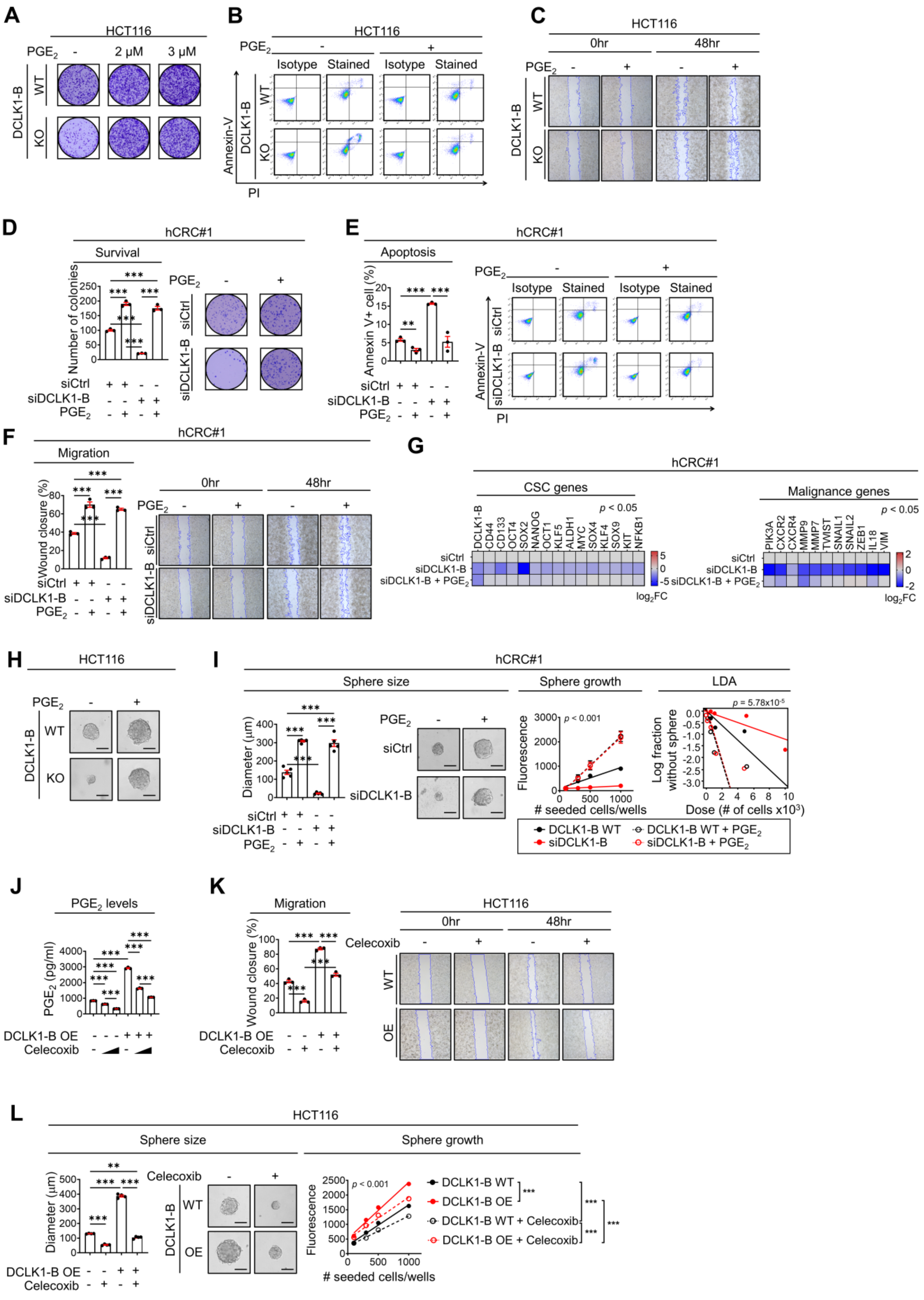
A
HCT116
PGE2 - 2 μM 3 μM
DCLK1-B WT KO
B
HCT116
PGE2 - +
Isotype Stained Isotype Stained
Annexin-V DCLK1-B WT KO
PI
C
HCT116
0hr 48hr
PGE2 - + - +
DCLK1-B WT KO
D
hCRC#1
Survival
Number of colonies
siCtrl
siDCLK1-B
PGE2
E
Apoptosis
Annexin V+ cell (%)
siCtrl
siDCLK1-B
PGE2
Isotype Stained Isotype Stained
Annexin-V
PI
F
hCRC#1
Migration
Wound closure (%)
siCtrl
siDCLK1-B
PGE2
0hr 48hr
G
hCRC#1
CSC genes
p < 0.05
DCLK1-B CD44 CD133 OCT4 SOX2 NANOG OCT1 KLF5 ALDH1 MYC SOX4 KLF4 SOX9 KIT NFKB1
siCtrl
siDCLK1-B
siDCLK1-B + PGE2
log2FC
Malignance genes
p < 0.05
PIK3A CXCR2 CXCR4 MMP9 MMP7 TWIST SNAIL1 SNAIL2 ZEB1 IL18 VIM
H
HCT116
PGE2 - +
DCLK1-B WT KO
I
hCRC#1
Sphere size
Diameter (μm)
siCtrl
siDCLK1-B
PGE2
Sphere growth
Fluorescence
p < 0.001
seeded cells/wells
LDA
p = 5.78x10-5
Log fraction without sphere
Dose (# of cells x10³)
DCLK1-B WT
siDCLK1-B
DCLK1-B WT + PGE2
siDCLK1-B + PGE2
J
PGE2 levels
PGE2 (pg/ml)
DCLK1-B OE
Celecoxib
K
Migration
Wound closure (%)
DCLK1-B OE
Celecoxib
HCT116
0hr 48hr
Celecoxib - + - +
WT
OE
L
HCT116
Sphere size
Diameter (μm)
DCLK1-B OE
Celecoxib
Celecoxib - +
DCLK1-B WT OE
Sphere growth
Fluorescence
p < 0.001
seeded cells/wells
DCLK1-B WT
DCLK1-B OE
DCLK1-B WT + Celecoxib
DCLK1-B OE + Celecoxib

Figure S10 (related to Figure 5).

(A)-(C) Representative images of altered biological functions upon PGE_2 treatment of DCLK1-B WT and KO HCT116 cells (n = 3 biological replicates). (A) Representative image of surviving colonies from clonogenic assay. (B) Representative flow cytometry 2D scatter plots showing cells doubly stained with PI and Annexin-V for apoptotic analysis. The gates were established by staining with corresponding isotype antibody. (C) Representative images of wound closure assay at 0 h and 48 h after IBID detachment. (D)-(F) Quantitative analysis (left) and representative images (right) of altered biological functions upon PGE_2 treatment and siDCLK1-B in hCRC#1 cells (n = 3 biological replicates). (D) Representative image of surviving colonies from clonogenic assay. (E) Representative flow cytometry 2D scatter plots showing cells doubly stained with PI and Annexin-V for apoptotic analysis. The gates were established by staining with corresponding isotype antibody. (F) Representative images of wound closure assay 0 h and 48 h after IBIDI detachment. (G) RT-qPCR heat map of relative expression of CSC genes and malignance genes comparing siCtrl, siDCLK1-B, and siDCLK1-B + PGE_2 groups. (H) Representative images of sphere forming assay. (I) Stemness was investigated with various methods, e.g., measuring sphere size, sphere growth, and sphere-forming potential. Sphere size was analyzed by diameter and fold of the mean value of siCtrl; growth was analyzed by cell titer blue quantification; Statistical significance was determined by one-way ANOVA with Dunnett's multiple comparison test and two-way repeated-measures ANOVA followed by Bonferroni post-test (n = 4 biological replicates). Sphere-forming potential was analyzed by *in vitro* LDA. *p* values indicate overall statistical significance of differences in stem cell frequencies between any of the groups. (J)-(L) Representative images of altered biological upon COX2 inhibitor, celecoxib treatment of DCLK1-B WT and OE HCT116 cells (n = 3 biological replicates). (J) *In vitro* ELISA showing the amount of PGE_2 secreted into the cell culture medium. (K) Representative images of wound closure assay at 0 h and 48 h after IBIDI detachment. (L) Stemness was assessed in DCLK1-B WT and OE HCT116 cells treated with celecoxib (30 μM). *p* values indicate overall statistical significance of differences in stem cell frequencies between any of the groups. The data are presented as the means ± SEMs. *** indicates $p < 0.001$. Statistical significance was determined by unpaired two-tailed Student's t tests for comparisons between two groups and one-way ANOVA with Dunnett's multiple comparison test for comparisons among three or more groups.

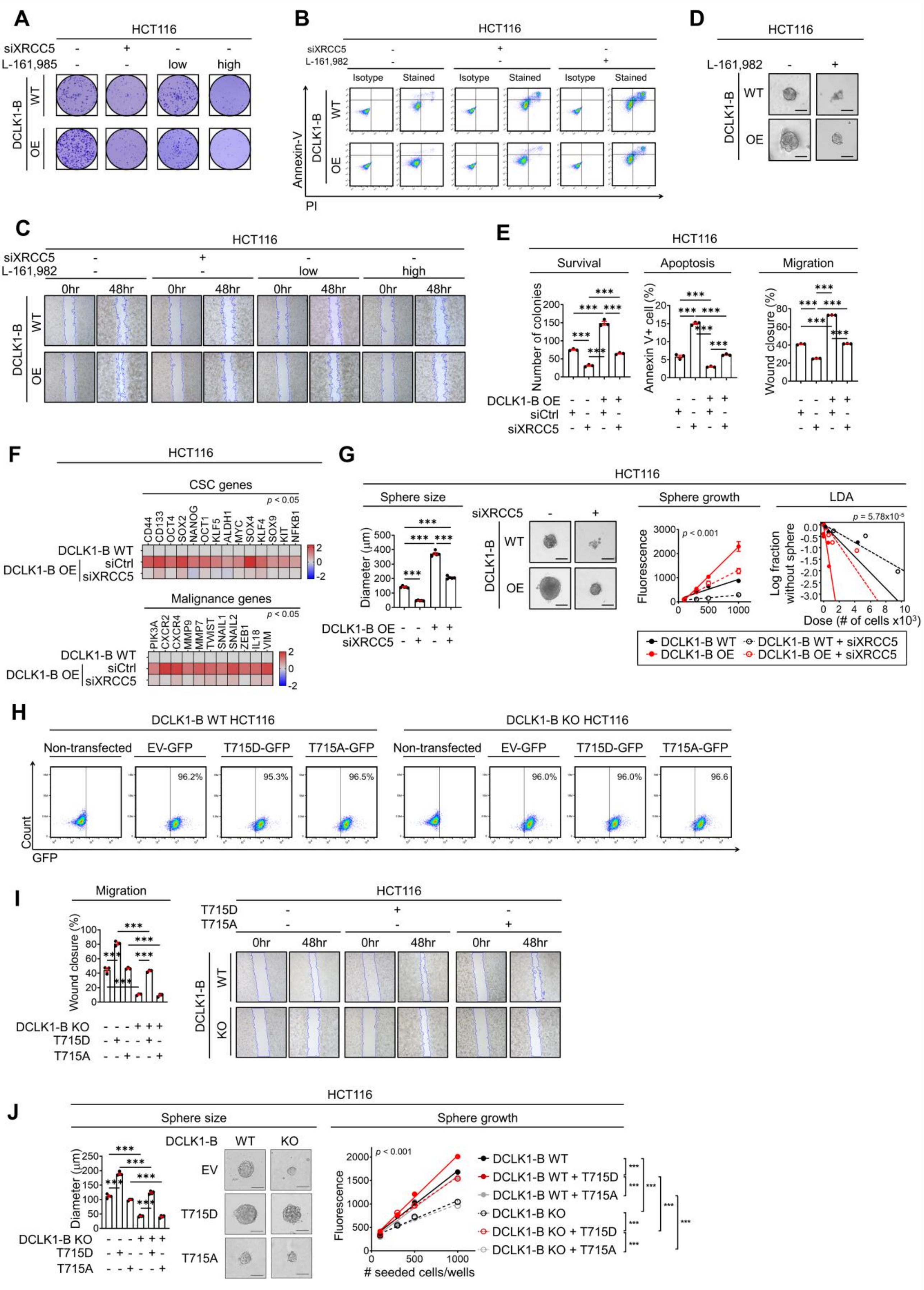
A
HCT116
siXRCC5
L-161,985
low
high
DCLK1-B
WT
OE
B
HCT116
siXRCC5
L-161,982
Isotype
Stained
Annexin-V
PI
D
HCT116
L-161,982
DCLK1-B
WT
OE
C
HCT116
siXRCC5
L-161,982
low
high
0hr
48hr
DCLK1-B
WT
OE
E
HCT116
Survival
Apoptosis
Migration
Number of colonies
Annexin V+ cell (%)
Wound closure (%)
DCLK1-B OE
siCtrl
siXRCC5
F
HCT116
CSC genes
p < 0.05
CD44
CD133
OCT4
SOX2
NANOG
OCT1
KLF5
ALDH1
MYC
SOX4
KLF4
SOX9
KIT
NFKB1
DCLK1-B WT
siCtrl
DCLK1-B OE
siXRCC5
Malignance genes
p < 0.05
PIK3A
CXCR2
CXCR4
MMP9
MMP7
TWIST
SNAIL1
SNAIL2
ZEB1
IL18
VIM
G
HCT116
Sphere size
Diameter (μm)
DCLK1-B OE
siXRCC5
Sphere growth
Fluorescence
p < 0.001
LDA
p = 5.78x10-5
Log fraction without sphere
Dose (# of cells x10³)
DCLK1-B WT
DCLK1-B WT + siXRCC5
DCLK1-B OE
DCLK1-B OE + siXRCC5
H
DCLK1-B WT HCT116
DCLK1-B KO HCT116
Non-transfected
EV-GFP
T715D-GFP
T715A-GFP
96.2%
95.3%
96.5%
96.0%
96.0%
96.6
Count
GFP
I
Migration
Wound closure (%)
DCLK1-B KO
T715D
T715A
HCT116
0hr
48hr
DCLK1-B
WT
KO
J
HCT116
Sphere size
Diameter (μm)
DCLK1-B
WT
KO
EV
T715D
T715A
Sphere growth
p < 0.001
Fluorescence
seeded cells/wells
DCLK1-B WT
DCLK1-B WT + T715D
DCLK1-B WT + T715A
DCLK1-B KO
DCLK1-B KO + T715D
DCLK1-B KO + T715A

Figure S11 (related to Figure 5).

(A)-(D) Representative images of altered biological functions upon upon KD of XRCC5 and inhibition of PGE_2 signaling. Statistical significance was determined by the one-way ANOVA with Dunnett's multiple comparison test (n = 3 biological replicates). (A) Representative images of surviving colonies from clonogenic assay. (B) Representative flow cytometry 2D scatter plots showing cells doubly stained with PI and Annexin-V for apoptotic analysis. The gates were established by staining with corresponding isotype antibody. (C) Representative images of sphere forming assay. (D) Representative images of wound closure assay 0 h and 48 h after IBIDI detachment. (E) A set of biological function assays representing the effect of altering the DCLK1/XRCC5 axis on cancer cell survival, apoptosis, and migration. (F) RT-qPCR heat map of relative expression of CSC genes and malignance genes comparing DCLK1 WT, DCLK1-B OE + siCtrl, and DCLK1-B OE + siXRCC5 groups (n = 3 biologically replicates). (G) Stemness was investigated with various methods, e.g., measuring sphere size, sphere growth, and sphere-forming potential. Sphere size were analyzed by diameter and fold of the mean value of siCtrl; growth was analyzed by cell titer blue quantification; sphere-forming potential was analyzed by *in vitro* LDA. *p* values indicate overall statistical significance of differences in stem cell frequencies between any of the groups. (H)-(J) Aggressive behaviour of CRC cells were investigated using phosphomimetic active XRCC5 (T715D) or phosphorylatable inactive XRCC5 (T715A) in DCLK1-B WT and KO HCT116 cells. (H) Establishment of phosphor-mutant XRCC5 overexpressing DCLK1-B WT and DCLK1-B KO HCT116 cell lines. Transfection efficiency was confirmed by GFP expression. (I) Quantities analysis and representative images of wound closure assay. (J) Stemness was assessed in DCLK1-B WT and OE HCT116 cells transfected with different mutant form of XRCC5, active and inactive. *p* values indicate overall statistical significance of differences in stem cell frequencies between any of the groups. The data are presented as the means ± SEMs. *** indicates $p < 0.001$. Statistical significance was determined by unpaired two-tailed Student's t tests for comparisons between two groups and one-way ANOVA with Dunnett's multiple comparison test for comparisons among three or more groups and two-way repeated-measures ANOVA followed by the Bonferroni post hoc test for comparisons of sphere growth.

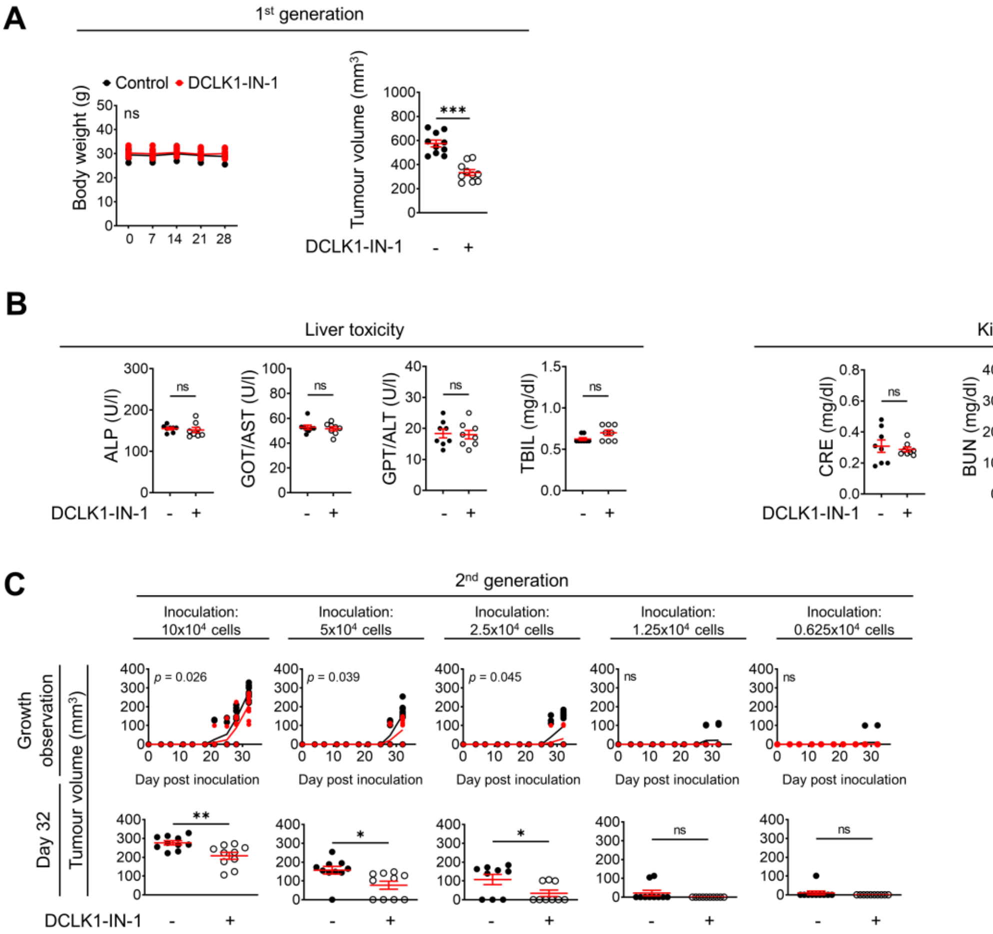

Frequency of tumour formation

	10x104		5x104		2.5x104		1.25x104		0.625x104		p-values
	Control	DCLK1-IN-1	Control	DCLK1-IN-1	Control	DCLK1-IN-1	Control	DCLK1-IN-1	Control	DCLK1-IN-1	
Day0	**0%**<br>(0 of 10)	**0%**<br>(0 of 10)	**0%**<br>(0 of 10)	**0%**<br>(0 of 10)	**0%**<br>(0 of 10)	**0%**<br>(0 of 10)	**0%**<br>(0 of 10)	**0%**<br>(0 of 10)	**0%**<br>(0 of 10)	**0%**<br>(0 of 10)	-
Day4	**0%**<br>(0 of 10)	**0%**<br>(0 of 10)	**0%**<br>(0 of 10)	**0%**<br>(0 of 10)	**0%**<br>(0 of 10)	**0%**<br>(0 of 10)	**0%**<br>(0 of 10)	**0%**<br>(0 of 10)	**0%**<br>(0 of 10)	**0%**<br>(0 of 10)	-
Day7	**0%**<br>(0 of 10)	**0%**<br>(0 of 10)	**0%**<br>(0 of 10)	**0%**<br>(0 of 10)	**0%**<br>(0 of 10)	**0%**<br>(0 of 10)	**0%**<br>(0 of 10)	**0%**<br>(0 of 10)	**0%**<br>(0 of 10)	**0%**<br>(0 of 10)	-
Day11	**0%**<br>(0 of 10)	**0%**<br>(0 of 10)	**0%**<br>(0 of 10)	**0%**<br>(0 of 10)	**0%**<br>(0 of 10)	**0%**<br>(0 of 10)	**0%**<br>(0 of 10)	**0%**<br>(0 of 10)	**0%**<br>(0 of 10)	**0%**<br>(0 of 10)	-
Day14	**0%**<br>(0 of 10)	**0%**<br>(0 of 10)	**0%**<br>(0 of 10)	**0%**<br>(0 of 10)	**0%**<br>(0 of 10)	**0%**<br>(0 of 10)	**0%**<br>(0 of 10)	**0%**<br>(0 of 10)	**0%**<br>(0 of 10)	**0%**<br>(0 of 10)	-
Day18	**0%**<br>(0 of 10)	**0%**<br>(0 of 10)	**0%**<br>(0 of 10)	**0%**<br>(0 of 10)	**0%**<br>(0 of 10)	**0%**<br>(0 of 10)	**0%**<br>(0 of 10)	**0%**<br>(0 of 10)	**0%**<br>(0 of 10)	**0%**<br>(0 of 10)	-
Day21	**20%**<br>(2 of 10)	**10%**<br>(1 of 10)	**0%**<br>(0 of 10)	**0%**<br>(0 of 10)	**0%**<br>(0 of 10)	**0%**<br>(0 of 10)	**0%**<br>(0 of 10)	**0%**<br>(0 of 10)	**0%**<br>(0 of 10)	**0%**<br>(0 of 10)	-
Day25	**40%**<br>(4 of 10)	**10%**<br>(1 of 10)	**0%**<br>(0 of 10)	**0%**<br>(0 of 10)	**0%**<br>(0 of 10)	**0%**<br>(0 of 10)	**0%**<br>(0 of 10)	**0%**<br>(0 of 10)	**0%**<br>(0 of 10)	**0%**<br>(0 of 10)	
Day28	**80%**<br>(8 of 10)	**60%**<br>(6 of 10)	**40%**<br>(4 of 10)	**20%**<br>(2 of 10)	**30%**<br>(3 of 10)	**10%**<br>(1 of 10)	**20%**<br>(2 of 10)	**0%**<br>(0 of 10)	**10%**<br>(0 of 10)	**0%**<br>(0 of 10)	0.0291
Day32	**100%**<br>(10 of 10)	**100%**<br>(10 of 10)	**90%**<br>(9 of 10)	**60%**<br>(6 of 10)	**60%**<br>(6 of 10)	**30%**<br>(3 of 10)	**20%**<br>(2 of 10)	**0%**<br>(0 of 10)	**10%**<br>(1 of 10)	**0%**<br>(0 of 10)	0.0373

Figure S12 (related to Figure 6).

(A) (Left) Body weight was measured throughout DCLK1-IN-1 treatment (n = 8 for each group). (Right) Final tumor volume upon DCLK1-IN-1 treatment. Mice were tracked for a total of 28 days (21 days post-treatment initiation). (B) Serological parameters for detecting liver and kidney toxicity. (C) Tumor growth of second-generation CRC cells was monitored for 32 days, at which time final tumor volumes were compared. Statistical significance was determined by two-way repeated-measures ANOVA followed by Bonferroni post-test for tumor growth and unpaired two-tailed Student's t-tests for tumor volume. *In vivo* LDA comparing the tumor formation frequency between second-generation Ctrl-treated and DCLK1-IN-1-treated cells.

Statistical significance was determined by the extreme LDA webtool (http://bioinf.wehi.edu.ac/software/eld). The data are presented as the means ± SEMs. *, **, and *** indicate $p < 0.05$, $p < 0.01$, and $p < 0.001$, respectively. Statistical significance was determined by unpaired two-tailed Student's t tests for comparisons between two groups and two-way repeated-measures ANOVA followed by the Bonferroni post hoc test for comparison of body weight changes and tumor repopulation.

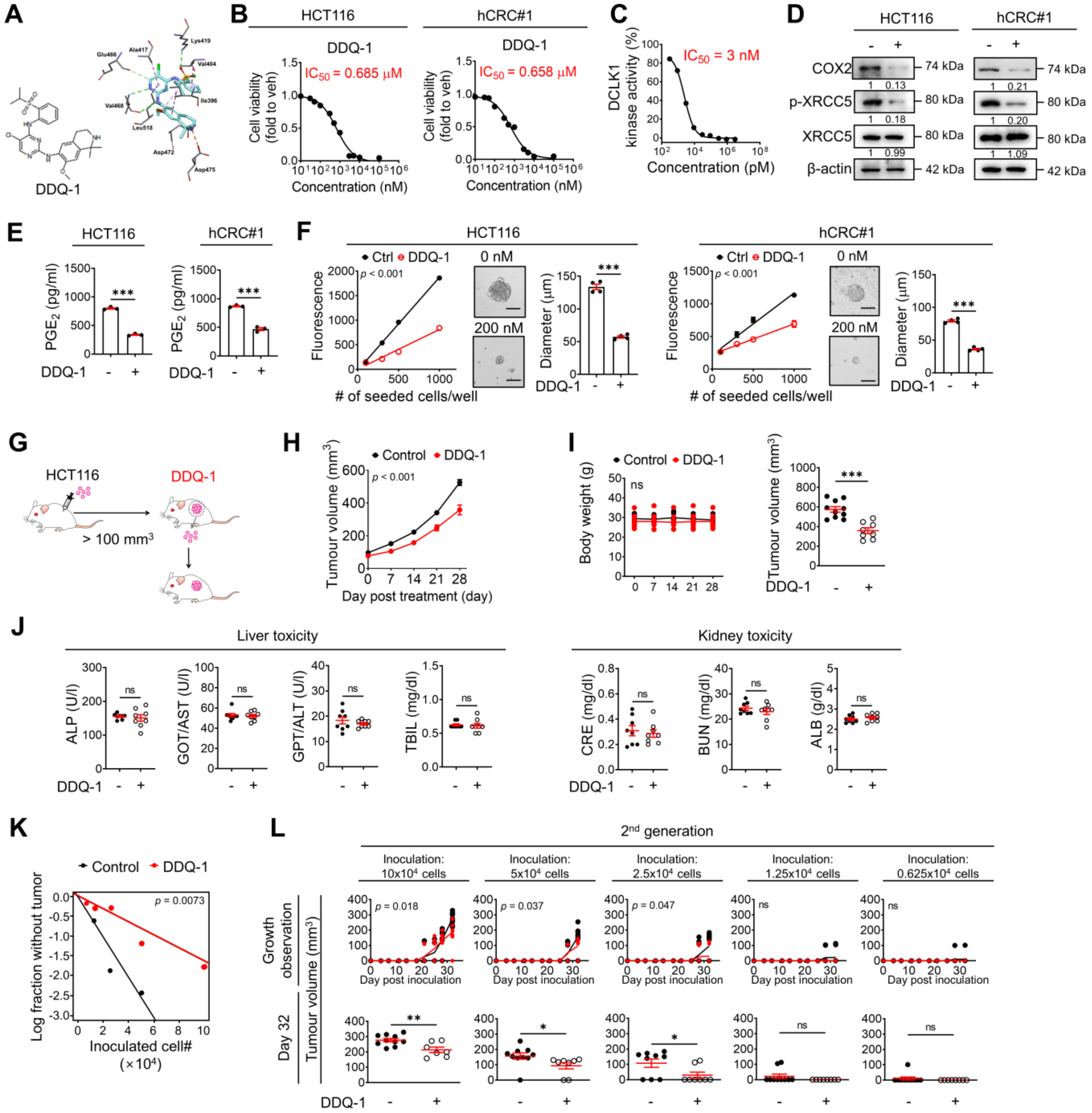

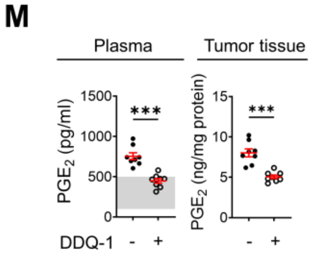

Frequency of tumour formation

	10x104		5x104		2.5x104		1.25x104		0.625x104		p-values
	Control	DDQ-1	Control	DDQ-1	Control	DDQ-1	Control	DDQ-1	Control	DDQ-1	
Day0	**0%** (0 of 10)	**0%** (0 of 8)	**0%** (0 of 10)	**0%** (0 of 8)	**0%** (0 of 10)	**0%** (0 of 8)	**0%** (0 of 10)	**0%** (0 of 8)	**0%** (0 of 10)	**0%** (0 of 8)	-
Day4	**0%** (0 of 10)	**0%** (0 of 8)	**0%** (0 of 10)	**0%** (0 of 8)	**0%** (0 of 10)	**0%** (0 of 8)	**0%** (0 of 10)	**0%** (0 of 8)	**0%** (0 of 10)	**0%** (0 of 8)	-
Day7	**0%** (0 of 10)	**0%** (0 of 8)	**0%** (0 of 10)	**0%** (0 of 8)	**0%** (0 of 10)	**0%** (0 of 8)	**0%** (0 of 10)	**0%** (0 of 8)	**0%** (0 of 10)	**0%** (0 of 8)	-
Day11	**0%** (0 of 10)	**0%** (0 of 8)	**0%** (0 of 10)	**0%** (0 of 8)	**0%** (0 of 10)	**0%** (0 of 8)	**0%** (0 of 10)	**0%** (0 of 8)	**0%** (0 of 10)	**0%** (0 of 8)	-
Day14	**0%** (0 of 10)	**0%** (0 of 8)	**0%** (0 of 10)	**0%** (0 of 8)	**0%** (0 of 10)	**0%** (0 of 8)	**0%** (0 of 10)	**0%** (0 of 8)	**0%** (0 of 10)	**0%** (0 of 8)	-
Day18	**0%** (0 of 10)	**0%** (0 of 8)	**0%** (0 of 10)	**0%** (0 of 8)	**0%** (0 of 10)	**0%** (0 of 8)	**0%** (0 of 10)	**0%** (0 of 8)	**0%** (0 of 10)	**0%** (0 of 8)	-
Day21	**20%** (2 of 10)	**25%** (2 of 8)	**0%** (0 of 10)	**0%** (0 of 8)	**0%** (0 of 10)	**0%** (0 of 8)	**0%** (0 of 10)	**0%** (0 of 8)	**0%** (0 of 10)	**0%** (0 of 8)	-
Day25	**40%** (4 of 10)	**63%** (5 of 8)	**0%** (0 of 10)	**0%** (0 of 8)	**0%** (0 of 10)	**0%** (0 of 8)	**0%** (0 of 10)	**0%** (0 of 8)	**0%** (0 of 10)	**0%** (0 of 8)	
Day28	**80%** (8 of 10)	**88%** (7 of 8)	**40%** (4 of 10)	**50%** (4 of 8)	**30%** (3 of 10)	**25%** (2 of 8)	**20%** (2 of 10)	**0%** (0 of 8)	**10%** (0 of 10)	**0%** (0 of 8)	0.0291
Day32	**100%** (10 of 10)	**88%** (7 of 8)	**90%** (9 of 10)	**75%** (6 of 8)	**60%** (6 of 10)	**25%** (2 of 8)	**20%** (2 of 10)	**0%** (0 of 8)	**10%** (1 of 10)	**0%** (0 of 8)	0.0373

Figure S13 (related to Figure 6).

(A) Docking model of DDQ-1 in the X-ray co-crystal structure of the DCLK1 kinase domain. (B) Cell viability and cytotoxic IC_{50} were determined by the MTT assay after 48 h treatment with DDQ-1 in HCT116 and hCRC#1 cells (n = 5 biological replicates). (C) A radiometric protein kinase assay was performed to determine the IC_{50} of DDQ-1 for kinase activity inhibition via scintillation counting. (D) Immunoblot of COX2, p-XRCC5 and XRCC5 upon DDQ-1 treatment. Relative band intensity of target protein was normalized to β-actin as fold of the non-treated group. (E) Level of PGE_2 altered by DDQ-1 treatment. Cell culture media was collected and the level of PGE_2 was measured by *in vitro* ELISA (n = 5 biological replicates). (F) Stemness was investigated with various methods, e.g., measuring sphere growth and sphere size. Sphere growth was analyzed by cell titer blue quantification while size was analyzed by measuring diameter and fold of the mean value of Ctrl. (G) Schematic illustration of the protocol for *in vivo* treatment with DDQ-1. HCT116 cells ($1x10^6$ cells mixed with Matrigel/mouse) were injected subcutaneously into the inguinal folds of NSG mice prior to treatment. Once the mean volume of the xenograft tumors reached 100 mm^3, 10 mg/kg of DDQ-1 was administered daily. Mice were tracked for 32 days after DCLK1 inhibitor injection (n = 8 mice per group). (H) Growth curves of the first tumor generation between the Ctrl and DDQ-1 treatment group. (I) (Left) Body weight was measured throughout DDQ-1 treatment (n = 8 for each group). (Right) Final tumor volume upon DDQ-1 treatment. Mice were tracked for a total of 28 days (21 days post-treatment initiation). (J) Serological parameters for detecting liver and kidney toxicity. (K) Tumor re-populating potential of the second generation was compared between Ctrl and DDQ-1 treated group. (L) *In vivo* LDA comparing the tumor formation frequency between second-generation Ctrl-treated and DDQ-1-treated cells. Statistical significance was determined by the extreme LDA webtool (http://bioinf.wehi.edu.ac/software/eld). (M) Levels of PGE_2 in plasma and tumor tissue. Plasma and tumor tissues were collected from ctrl- and DDQ-1 treated B6 tumor-bearing mice. The estimated normal range of the plasma PGE_2 level was determined by the values obtained in naïve mice (shaded in gray). The data are presented as the means ± SEMs. *** indicates $p < 0.001$. Statistical significance was determined by unpaired two-tailed Student's t-tests for comparisons between two groups and by two-way repeated-measures ANOVA followed by the Bonferroni post hoc test for comparison of sphere growth over time, *in vivo* body weight changes and *in vivo* tumor growth.

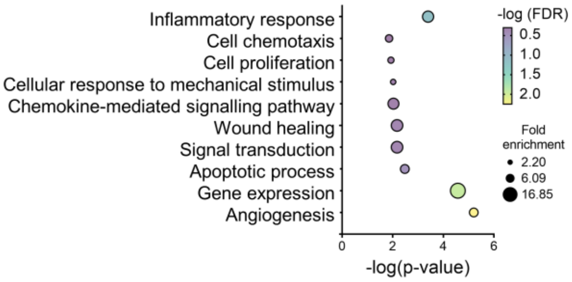

Figure S14.

KEGG analysis of pathways enriched in DCLK1-B WT cells compared to DCLK1-B KO HCT116 cells from RNA sequencing profiles.

Table S1. Clinical information of CRC patient samples used for establishing patient-derived primary CRC cells and association of clinicopathologic characteristics in patients with resected colon cancer (n = 123)

Primary tumour cells from CRC patient tissues

Cell lines	Sex	Age	Diagnosis	Surgical stage	Pathological diagnosis			
					K-ras	EGFR	P53	MS
hCRC#1	M	45	Perforated S colon cancer with liver and lung metastasis	T4aN2bM1	Wild	Mutation	Positive	MSS
hCRC#2	M	84	Proximal a-colon cancer with liver metastasis	T3N1M1	Wild	Mutation	Positive	MSS

Clinicopathological analysis of CRC patients (n = 123)

Variable	Low (< 50%, n = 62)	High (> 50%, n = 61)	*p*-value
T stage			
T2-3	59 (95.2%)	46 (75.4%)	**0.003**
T4	3 (4.8%)	15 (24.6%)	
N stage			
N0	35 (56.5%)	28 (45.9%)	0.171
N1	18 (29.0%)	19 (31.1%)	
N2	9 (14.5%)	14 (23.0%)	
TNM stage			
II	35 (56.5%)	28 (45.9%)	0.096
III	27 (43.5%)	33 (54.1%)	
Recurrence			
Yes	5 (8.1%)	12 (19.7%)	**0.021**
No	57 (85.5%)	49 (80.3%)	

Table S2. List of DEGs in DCLK1-high versus DCLK1-low tumors from patients with CRC

Attached as a separate Excel file.

Table S3. List of DEGs in DCLK1-B WT versus DCLK1-B KO HCT116 cells

Attached as a separate Excel file.

Table S4. List of DCLK1-binding proteins identified by co-IP and subsequent LC-MS analysis.

Attached as a separate Excel file.

Table S5. List of primers used for real-time RT-qPCR or ChIP assay

Primer List		
Gene	Forward	Reverse
hPPIA	TGCCATCGCCAAGGAGTAG	TGCACAGACGGTCACTCAAA
hDCLK1-A	GGAGTGGTGAAACGCCTGTAC	GGTTCCATTAACTGAGCTGG
hDCLK1-B	ACACTAAGACTGTGTCCATGTTAGAA CTC	AAGCCTTCCTCCGACACTTCT
hXRCC5	GGTTTTCTTTAAGTGGGGTGGG	GCAGAGATTGGGCCAAAAGG
hCOX2	CCCACCCATGTCAAAACCGA	TCCAAAATCCCTTGAAGTGG
hCD44	GGAGCAGCACTTCAGGAGGTT	GGAATGTGTCTTGGTCTCTGGTAGC
hCD133	CAGAGTACAACGCCAAACCA	AAATCACGATGAGGGTCAGC
hOCT4	GGGCTCTCCCATGCATTCAAAC	CACCTTCCCTCCAACCAGTTGC
hSOX2	TCGGCAGACTGATTCAAATA	CCATGCAGGTTGACACCGTT
hNANOG	TGGGATTTACAGGCGTGAGCCAC	AAGCAAAGCCTCCCAATCCCAAAC
hOCT1	CCCTGTCTCAGCCCATACAGA	GCTGCAAATTGGTGGTTGGAT
hKLF5	CCCTTGCACATACACAATGC	GGATGGAGGTGGGGTTAAAT
hALDH1A1	CAAATAGTGCACTGTCTCCAGG	ACGACACTACTTATTGTAACACCT
hMYC	CAAGTATACGTGGCAATGCGT	TCAAGAGTCCCAGGGAGAG
hSOX4	GGTCTCTAGTTCTTGCACGCTC	CGGAATCGGCACTAAGGAG
hKLF4	ACGATCGTGGCCCCGGAAAAGGACC	CAACAACCGAAAATGCACCAGCCCCA G
hSOX9	CATGAGCGAGGTGCACTCC	TCGCTTCAGGTCAGCCTTG
hKIT	GCCTATCAGCTTCAGAATGGC	CCTGCGAAGGAGGCTAAACC
hNFKB1	TGCCATTGTCTTCAAAACTCCA	GGGCATGAGCTTCTGACGTT
hPIK3CA	CATGCATTGTTTTGCACCCC	ATGGAAGACGGGAGATTCACAT
hCXCR2	ATGCTGTTACGGATCCTGCC	CCCCATGTGGGCCTTAAACA
hCXCR4	AGGAACCCTGTTTCCGTGAAG	GCAGCCTGTACTTGTCCGTC
hMMP9	CGTTCAGGGAGATGCCCATT	TCGTAGTCAGCTGTTGTGCT

hMMP7	TCGGAGGAGATGCTCACTTCGA	GGATCAGAGGAATGTCCCATACC
hTWIST	CCTGCGCAAGATCATCCCCA	GCTGCAGCTTGCCATCTTGGA
hSNAIL1	CTGGGTGCCCTCAAGATGCA	CCGGACATGGCCTTGTAGCA
hSNAIL2	TACCGCTGCTCCATTCCACG	CATGGGGGTCTGAAAGCTTGG
hZEB1	CCTACTCAGCCTCCTCCACTC	CCAGTAGCTGATGAAACAGAGGA
hIL18	TTGACCAAGGAAATCGGCCT	GGTCCGGGGTGCATTATCTC
hVIM	ACCCGCACCAACGAGAAGGT	ATTCTGCTGCTCCAGGAAGCG
hTRAF1	CGATGGCACTTTCCTGTGGAAG	TACAGCCGCAGGCACAACTTGT
hCD68	CGAGCATCATTCTTTCACCAGCT	ATGAGAGGCAGCAAGATGGACC
hBIRC3	GCTTTTGCTGTGATGGTGGACTC	CTTGACGGATGAACTCCTGTCC
hCTSD	GCAAACTGCTGGACATCGCTTG	GCCATAGTGGATGTCAAACGAGG
hCXCL3	TTCACCTCAAGAACATCCAAAGTG	TTCTTCCCATTCTTGAGTGTGGC
hNFKBIA	TCCACTCCATCCTGAAGGCTAC	CAAGGACACCAAAAGCTCCACG
hBBC3	ACGACCTCAACGCACAGTACGA	CCTAATTGGGCTCCATCTCGGG
hCXCL8	GAGAGTGATTGAGAGTGGACCAC	CACAACCCTCTGCACCCAGTTT
hTP53BP-1	AAGCCAGGCAAGAGAATGAGGC	GGCTGTTGACTCTGCCTGATTG
hBAX	TCAGGATGCGTCCACCAAGAAG	TGTGTCCACGGCGGCAATCATC
hMMP14	CCTTGGACTGTCAGGAATGAGG	TTCTCCGTGTCCATCCACTGGT
hBCL2L1	GCCACTTACCTGAATGACCACC	AACCAGCGGTTGAAGCGTTCCT
mDCLK1-A	TCAATGAGGACCAGCTCCAG	TCCGAGAGAGTTCGGGTCA
mDCLK1-B	AAGACGTCAGCCTTACGCAG	GAGAGATCCTCTGCTTCCGC
mDCLK1-A,B	AGCGGAGAACCGCATTTCAA	ATCTCTGCCGAACGACATGG
mXRCC5	TCCAATTGAACAAGCAAAGAAAGT	CTTGCCAGCAAGGGCATTATC
mGAPDH	ATGTGTCCGTCGTGGATCTGA	TTGAAGTCGCAGGAGACAACC
mPTGS2	GATGCTCTTCCGAGCTGTG	GGATTGGAACAGCAAGGATTT
mIGF1R	GCCTGACACCGTAGGTCTTT	GGCAAAAATGGCAGCGTGAA
mMCAM	CATAGCGGGCTCTACCAGTG	TCAGGGGCAGTTGGATTCAC
mIL1B	GTCGCTCAGGGTCACAAGAA	GTGCTGCCTAATGTCCCCTT

mRORC	GGGGAGAGCTTTGTGCAGAT	TCAGTAGGGTAGCCCAGGAC
mIL17a	ACTACCTCAACCGTTCCACG	ATGTGGTGGTCCAGCTTTCC
mCTLA4	GTACCTCTGCAAGGTGGAACTC	CCAAAGGAGGAAGTCAGAATCCG
mCD206	GCTGTTCAGCTATTGGACGC	CGGAATTTCTGGGATTCAGCTTC
mTGM2	CCGATCCCCTGTATGACTGC	CCAGCTTGTGGAGGCCAATA
mARG1	GCAGTGGCTTTAACCTTGGC	CTGTGATGCCCCAGATGGTT
mYM2	GTACCCTGGGTCTCGAGGAA	GCAGCCTTGGAATGTGGTTC
mFIZZ1	CTGCTACTGGGTGTGCTTGT	GCAGTGGTCCAGTCAACGAG
mMRC1	GTCAGAACAGACTGCGTGGA	CAGCAGCAGTCTCGATGGAA
mCXCL10	TAACTGGAGTGAAGCCACGC	TAGCAGCTGATGTGACCACG
mCXCL11	CCACGCTACCTTCTGTGGTT	ATGTTCGTGTGCCTCGTGAT
mCXCL9	CTGGGCAGAAGTTCCGTCTT	TTACCGAAGGGAGGTGGACA
mCCND2	CGTCAAGAGCAGCATAACGC	CGAATGGCTTCCTCACAGGT
mTNFRSF4	AAGGCGGCTCAACTGTGTTA	GGTATGATCACAGCGGCTCA
mCD274	CCAGCCACTTCTGAGCATGA	AAACATCATTCGCTGTGGCG
mCD247	GCAGAAGCCTACAGTGAGATCG	CCAAGGGTCCTTGTGAGTTCTG
mCD3E	TGCCCTCTAGACAGTGACGA	TTGAGGCTGGTGTGTAGCAG
mPDCD1LG2	TGCTGCCGATACTGAACCTG	AGTGCATTCTCTGCGGTCAA
mSERPINE2	AGAGACTCCCTCCCCACATC	TTTCTTCTGACACAGCCCCG
COX2 promoter, ChIP#1	CGTTTTGGACATTTAGCGTCCC	AGCTAAGTTGCTTTCAACAGAAGA
COX2 promoter, ChIP#2	AAGAAACAAGGAGGGGGTGAA	ACGACGCTTAATAGGCTGTATATC
COX2 promoter, ChIP#3	AAGCTATGTATGTATGTGCTGCAT	GGACACTTGGCTTCCTCTCC
COX2	CCCTCTAAAGACGTACAGACCAGA	CTCTCCTGTCTGATCCCTCCC

promoter, ChIP#4		
COX2 promoter, ChIP#5	CGGGCAAAGACTGCGAAGAA	CCCCACAAATTTTTCCCTCCTC
COX2 promoter, ChIP#6	AGCTTCCTGGGTTTCCGATTT	TGGTCGCTAACCGAGAGAAC

H, human; m, mouse.

Table S6. List of short interfering RNAs

	Sense	Antisense
siDCLK1A	GAGGCUUAAUUGACACCUU	AAGGUGUCAAUUAAGCCUC
siDCLK1B#1	CCGUUGGUAUUCAGGUCCA	UGGACCUGAAUACCAACGG
siDCLK1B#2	AUGCAGCCAAGUUACCGUC	GACGGUAACUUGGCUGCAU
siXRCC5#1	GUGGAAUGAAUACACACAU	AUGUGUGUAUUCAUUCCAC
siXRCC5#2	GACUGACCAUUGGCUCCAA	UUGGAGCCAAUGGUCAGUC

Table S7. List of antibodies

Target	Conjugation	Catalog #	Application
Human DCLK1 (total)	-	ab31704 / Abcam	IF
Human DCLK1-B	-	ABC1389 / Sigma-Aldrich	IF
Human EpCam	-	93790S / Cell Signaling Technology	IF
	Alexa488	14-9326-82 / eBioscience™	IF
Mouse DCLK1	-	ab31704 / Abcam	IF
Mouse GFP	-	A-11122 / Invitrogen	IF
Mouse COX2	-	12282S/ Cell Signaling Technology	IF
Mouse XRCC5	-	MA5-12933 / Invitrogen	IF
Mouse p-XRCC5	-	PA5-36720 / Invitrogen	IF
Goat anti-mouse	Alexa488	A32723 / Invitrogen	IF
Goat anti-mouse	Alexa555	A21422 / Invitrogen	IF
Goat anti-rabbit	Alexa405	A-31556 / Invitrogen	IF
Donkey anti-mouse	Alexa488	A-21202 / Invitrogen	IF
Donkey anti-rabbit	Alexa488	A-21206 / Invitrogen	IF
Donkey anti-rabbit	Alexa555	A-31572 / Invitrogen	IF
Donkey anti-goat	Alexa488	A-11055 / Invitrogen	IF
Human DCLK1 (total)	-	ab31704 / Abcam	Immunoblot
Human DCLK1-B	-	ABC1389 / Sigma-Aldrich	Immunoblot
Human XRCC5	-	ab80592 / Abcam	Immunoblot
Human p-XRCC5	-	PA5-38118 / Invitrogen	Immunoblot
Human COX2	-	12282S/ Cell Signaling Technology	Immunoblot
Human β-actin	-	A5316 / Sigma-Aldrich	Immunoblot
Mouse GFP	-	A-11122 / Invitrogen	Immunoblot
Mouse COX2	-	12282S/ Cell Signaling Technology	Immunoblot
Mouse XRCC5	-	ab80592 / Abcam	Immunoblot
Mouse p-XRCC5	-	PA5-36720 / Invitrogen	Immunoblot

Mouse β-actin	-	A5316 / Sigma-Aldrich	Immunoblot
Goat anti-mouse	HRP	554002 / BD Pharmigen™	Immunoblot
Goat anti-rabbit	HRP	554021 / BD Pharmigen™	Immunoblot
Goat anti-mouse, Light chain-specific	HRP	91196S / Cell Signaling Technology	Immunoblot

HRP, horseradish peroxidase; IF, immunofluorescence

Supplementary materials and methods

No statistical methods were used to predetermine sample size. The investigators were not blinded to allocation during experiments and outcome assessment.

Isolating DCLK1-B^{high} and DCLK1-B^{low} HCT116 cells

The green fluorescent protein (GFP) tagged DCLK1-B reporter was constructed by inserting the DCLK1-B promoter sequence acquired from promoter-reporter constructs used in a previous report [1] into pLenti-Promoterless-GFP (Promega, Madison, WI, USA). HCT116 cells were infected with lentiviral particles and selected with puromycin for one week. Dilution cloning was performed to obtain different monoclonal cell populations. Transfection efficiencies of multiple clones were estimated by GFP intensity detection. Then, for further experiments, DCLK1-B^{high} and DCLK1-B^{low} cells were isolated by a fluorescence-activated cell sorting (FACS) Aria instrument (BD Biosciences, Franklin Lakes, NJ, USA) according to the intrinsic level of DCLK1-B, represented by GFP intensity.

Generation of DCLK1 overexpression (OE) and knockout (KO) cell lines

Establishment of DCLK1 OE and KO HCT116 cell lines were achieved as described in our previous report [1]. The DCLK1-B KO HCT116 cell line (NM_01195415.1 in NCBI data base) was generated by Transomic Technologies (Alabama, USA). Two specific guide RNAs (gRNAs) targeting different regions of the human *DCLK1-B* gene were designed and cloned into the pCLIP-All (EFS-Puro) expression vector. gRNAs targeting *DCLK1-B* were as follows; gDCLK1-B (I): CCTGAGCGCGGAGTAGAGAGCT, gDCLK1-B (II): TTGGCGACTTGCCTGAGCGCGG. HCT116 cells were infected with lentiviral particles containing pCLIP-All and selected with puromycin for one week. Dilution cloning was performed to obtain different monoclonal cell populations. The KO efficiencies of multiple clones were estimated by real-time PCR analysis, and then we selected a single clone showing the most potent KO efficiency from each gRNA sequence. These two clones generated from two different sequences were subjected to western blot analysis for validating protein ablation. The two validated clones were used for further experiments. DCLK1-B OE vector (NM_00119541.1, cat # EX-Z8538-M68) and control empty vector (cat # EX-NEG-M68) were purchased from GeneCopoeia™ (Rockville,

MD, USA). These vectors were transfected by Lipofectamine 2000 (Invitrogen, Carlsbad, CA, USA) according to the manufacturer's recommendations. Dilution cloning was performed to obtain different monoclonal cell populations. The OE efficiency was estimated at both the mRNA level by real-time PCR and the protein level by western blotting.

Generation of XRCC5 mutant cell lines

XRCC5 (NM_021141.4) mutant vectors were generated by inducing mutations at threonine 715 of enhanced GFP-tagged XRCC5-expressing vector, pEGFP-C1-FLAG-KU80 (Addgene, Watertown, MA, USA) [2]. Mutagenesis generating non-phosphorylatable XRCC5 (T715A) and phosphomimetic XRCC5 (T715D) [3] was performed by Macrogene (Seoul, Republic of Korea). For expressional analysis of COX2 reliant to XRCC5 phosphorylation status, HEK293T cells were infected with lentiviral particles expressing wild-type (WT) XRCC5, non-phosphorylatable XRCC5 (T715A), and phosphomimetic XRCC5 (T715D) and selected with G418 for one week. For functional analysis on phosphorylation of XRCC5, DCLK1-B WT and KO HCT116 cells were infected with lentiviral particles expressing mutant forms of XRCC5. The transfection efficiency was estimated at the protein level of eGFP by flow cytometry, and transfected cells were used for further experiments.

Subcutaneous xenograft models

Luciferase-labeled MC-38 cells (C57BL/6J mouse-derived colon carcinoma cell line) or 4T1 cells (Balb/c mouse-derived breast carcinoma cell line) were inoculated subcutaneously into 5-week-old C57BL/6J mice ($5x10^4$ cells/mouse) or Balb/C mice ($1x10^5$ cells/mouse), respectively. Tumor growth was monitored by measuring luciferase activity for 21 days from cell inoculation (14 days from treatment initiation) until necropsy (n = 5 or 8 for each group, respectively). DCLK1 inhibitor (DCLK1-IN-1) was administered daily for the experimental period. After necropsy, tumor volumes were calculated as follows: tumor volume = (length x width^2)/2. Blood was collected from the abdominal vena cava, stored in ethylenediamine tetraacetic acid (EDTA) blood collection tubes, and subjected to PGE2 Elisa Assay (R&D systems, Minneapolis, MN, USA). Age-matched naïve C57BL/6J mice (n = 5) and Balb/C mice (n = 8) were used as the control group. DCLK1 OE HCT116 and wild-type HCT116 cells were inoculated subcutaneously into 8-week-old NSG mice ($1x10^6$ cells/mouse). Tumor growth was monitored

and measured for 35 days from cell inoculation until necropsy (n = 8 for each group). Tumor volumes were calculated as stated above.

In vivo limiting dilution assay

In vivo limiting dilution assays (LDAs) were performed as described in previous reports [1, 4]. Briefly, primary tumors were removed and dissociated into single cells to compare the tumor regrowth potential of each group. After depleting stromal cells with a mouse cell depletion kit (Miltenyl Biotec, Bergisch Gladhac, Germany), the isolated tumor cells were subcutaneously injected into the inguinal folds of mice with Matrigel at various dilutions. Tumors formed by this injection were monitored twice a week, and tumor volumes were measured by a caliper. The definitive ratio of tumor-bearing mice was determined by necropsy, and the frequency of stem cells was calculated by the extreme LDA webtool (http://bioinf.wehi.edu.au/software/elda).

Intrasplenic inoculation model

A splenic injection experiment was performed to estimate the step governing metastasis and distant organ colonization. In this model, DCLK1-B WT and DCLK1-B OE HCT116-luc cells ($1x10^6$ cells/mouse) were inoculated into the spleen followed by splenectomy, and the surviving cells that grew in distant organs then contributed to the formation of liver metastases. Liver metastasis was routinely monitored weekly by visualizing luciferase activity for 28 days (n = 8 for each group). After sacrifice, the livers were removed to determine liver metastasis.

AAV9-CMV-GFP construction and tail vein injection

XRCC5 knockdown (KD) and control recombinant adeno-associated virus-GFP vector 9 (AAV9-GFP) plasmids were constructed (Vigene Biosciences, Rockville, MD, USA). The administrations were performed according to previous studies [5]. Briefly, $1x10^{11}$ physical particles of AAV in 100 μL of PBS were injected into the tail veins of 4-week-old C57BL/6J and C57BL/6J-*Apc*^{Min}/J mice. Small intestine and colon were removed for GFP expression evaluation with western blotting and immunohistochemistry. Number of polyps were counted and total

tumor burden was calculated as follow: number of polyps * 1 (0 mm < polyp ≤ 3 mm), number of polyps * 2 (3 mm < polyp ≤ 5 mm), number of polyps * 3 (5 mm < polyp).

PGE_2 ELISA assay

Plasma segregated from blood of experimented mice and protein extracted from xenografted tumor tissue were analyzed for systemic and local PGE_2 levels, respectively. Level of PGE_2 was assessed by PGE_2 ELISA assay kit (R&D system, Minneapolis, MN, USA) according to the manufacturer's instructions. The quantitative analysis of PGE_2 was measured at a wavelength of 540 or 570 nm (normalized by 450 nm) using Epoch microplate reader (Biotek, Winooski, VT, USA).

RNA isolation and real time-qPCR

Total RNA was isolated from mouse tissues or cells using TRIzol reagent (Invitrogen, Waltham, MA, USA). The purity of RNA was verified by measuring the 260/280 nm and 260/230 nm absorbance ratios. The cDNA templates were synthesized from 2 μg of total RNA using the PrimeScript™ 1st strand cDNA Synthesis Kit (Takara Bio Inc., Kusatsu, Shiga, Japan) with random primers. Then, Power SYBR Green PCR Master Mix and Step-One Real-time PCR systems (Applied Biosystems, Waltham, MA, USA) were used for the PCR amplification of cDNAs. The primers used are listed in **Table S5**.

Gene expression modification via siRNA

Cells were transfected with siRNA targeting specific genes or a nonspecific negative control siRNA (Bioneer, Daejeon, Republic of Korea) in media (serum-, phenol-, antibiotic-free) with Lipofectamine™ 2000 (Invitrogen) according to the manufacturer's instructions. Knockdown efficiency was confirmed by measuring mRNA expression using reverse transcription PCR and RT-qPCR. The sequences of siRNAs are listed in **Table S6.**

Protein isolation and western blot analysis

Tissues or cells were homogenized in radioimmunoprecipitation assay (RIPA) buffer for 20 min on ice. RIPA buffer was prepared in our laboratory as previously reported [6]. The BCA Protein Assay kit was used to determine protein concentrations using the bicinchoninic acid (BCA) assay (Thermo Fisher Scientific, Waltham, MA, USA). Proteins were denatured by boiling at 95 °C for 5 min with sodium dodecyl sulfate (SDS) (Sigma-Aldrich, St. Louis, MO, USA). On 8 % acrylamide gels, equal amounts of total protein (4-15 g) were electrophoresed, and the separated proteins were transferred to a polyvinylidene difluoride membrane (MilliporeSigma, Burlington, MA, USA). The membrane was blocked with 5% bovine serum albumin (Sigma-Aldrich, St. Louis, MO, USA) before being incubated with the primary antibodies overnight at 4 °C. The membrane was then treated with secondary antibodies that were conjugated to horseradish peroxidase. Horseradish peroxidase chemiluminescence was created using ECL reagent (Atto, Tokyo, Japan) and identified using a digital imaging equipment (ProteinSimple, San Jose, CA, USA). Antibodies used for western blot analyses are listed in **Table S7**.

Apoptosis assay (Annexin V+)

The rate of cell apoptosis was quantified using apoptosis assays performed with an Annexin V-Fluorescein isothiocyanate (FITC) Apoptosis Detection Kit I. (BD Biosciences). Washing cells twice with cold PBS yielded cell suspensions ($1x10^6$ cells/mL). The suspension was then transferred to a tube containing 5 L of FITC, annexin V, and propidium iodide (PI). After gentle vortexing, the mixture was incubated at room temperature for 15 mins in the dark. After incubation, 400 L of 1 x binding buffer was added before flow cytometry analysis.

Immunofluorescence and quantification

Formalin-fixed and paraffin-embedded sections of CRC patients' intestinal tissue and primary xenografted tumors were dewaxed and hydrated in an OTTIX bath (Diapath, Martinengo, Italy). The dewaxed slides were incubated with specific primary antibodies at 4 °C overnight. After washes with Tris-buffered saline containing 0.5 % Tween-20 (TBST), slides were incubated with secondary antibodies at room temperature for 20 min. Then, slides were washed with TBST, and the nuclei were counterstained with DAPI for 10 s. Slides were then dehydrated using an OTTIX bath (Diapath) and mounted with mounting solution (Vector Laboratories, Burlingame, CA, USA). Fluorescence was visualized and analyzed using Axio Imager 2 (Carl Zeiss, Oberkochen, Germany). The

dewaxed slides were stained with hematoxylin (Sigma-Aldrich) and eosin (Diapath) to histologically observe the morphology of tissues. Images were captured with a phase-contrast microscope (Carl Zeiss). Antibodies used to visualize proteins by immunofluorescence are listed in Table S7.

Cells were seeded on poly-L-lysine and collagen I-coated cover glasses. After cells were fully attached to the cover slips, cells were fixed in 4% formalin. Cells were permeabilized, blocked, and stained with specific primary antibodies listed in Table S7. Nuclei were counterstained with DAPI (Sigma-Aldrich, St. Louis, MO, USA). Secondary antibodies conjugated with fluorescent dyes were used to visualize target proteins (Table S7). Fluorescence was visualized using Axio Imager 2 (Carl Zeiss, Oberkochen, Germany).

Luciferase reporter assay

A luciferase COX2-reporter assay was performed as described in a previous publication to analyze the transcription of COX2 [7]. CRC cells were seeded into 12-well plates. The mixture of DNA (COX2-reporter vector), β-galactosidase, and Lipofectamine was prepared in the proportions of 1 μg : 1 μg : 2 μL in 50 μL of serum-free medium/well. Twenty-four hours after the mixed solution was applied to the seeded cells, cells were lysed. Luciferase reagent was added to detect luciferase activity and detected using a luminometer (Promega, Madison, WI, USA) according to the manufacturer's recommendations. The relative COX2 transcription level was determined by measuring luciferase activity and normalizing the raw results of COX2 expression to β-galactosidase expression.

Identification of DCLK1-B-interacting proteins

Potential DCLK1-B-interacting proteins were obtained from whole-protein lysates of HCT116 cells by using monoclonal antibody-based immunoprecipitation (IP). IP samples incubated with isotype IgG control antibody were used as negative controls. Peptide samples were obtained by sodium dodecyl sulfate-polyacrylamide gel electrophoresis (SDS-PAGE) and in-gel digestion with trypsin. The peptide samples were purified and concentrated using columns containing C18 reverse-phase resin. The peptides were identified by liquid chromatography with tandem mass spectrometry (LC-MS). Raw mass spectrometric data were processed with Sorcerer 2^{TM}-SEQUEST (Sage N Research, Milpitas, CA, USA). A list of proteins enriched in the DCLK1-IP

samples versus the IgG control IP samples was obtained by estimating the protein abundance through Top3 analyses.

Clonogenic assay

A clonogenic assay was performed as described in our previous report [1]. Briefly, cells were seeded in 12-well plates (200 cells/well) and cultured for 14 days. The colonies greater than 50 μm in size were counted after staining with crystal violet (n = 3/group)

Migration and invasion assay

The Transwell system (8 μm pore size, Corning) was employed for migration and invasion assays. For migration assays, $3x10^5$ cells were seeded in the upper chambers in serum-free medium, and for invasion assays, $3x10^5$ cells were seeded in the upper chamber of a Matrigel-coated Transwell system (8 μm pore size, Corning) in serum-free medium. The bottom chamber was filled with medium supplemented with 20% FBS. After incubation for 24 h at 37 °C, the cells that had migrated to or invaded the bottom of the insert membrane were fixed, stained with crystal violet, and counted under observation with a phase-contrast microscope (Carl Zeiss, biological triplicates).

Cell viability

Cells were seeded in 6-well or 96-well plates and incubated for various time points. The numbers of viable cells were measured with an automated cell counter (Countess II, ThermoFisher Scientific, Waltham, MA, USA) or measured by staining with thiazolyl blue tetrazolium bromide (MTT, Sigma-Aldrich, St. Louis, MO, USA), and the absorbance was measured using a microplate spectrophotometer (Bio-Tek Instruments Inc., Winooski, VT, USA).

Bioinformatics analysis using an open-source database

To compare the DCLK1 mRNA expression levels between normal and tumor tissues, gene expression data were

obtained from an open-source database (R2: Genomic analysis and visualization platform). Gene set enrichment analysis (GSEA) was conducted as described in our previous study [1]. Briefly, the differentially expressed gene (DEG) list was obtained using the R2 Platform (GSE21510) by comparing two groups of patients with CRC (divided according to median DCLK1 level; n = 52/group). Total DEG lists ($p < 0.001$) were applied as a ranked gene list. The GSEA of the ranked gene list was conducted using the Java implementation of GSEA obtained from http://www.broadinstitue.org/gsea/ (1,000 permutations; minimum term size: 15; maximum term size: 500, C2: all curated genes). The normalized enrichment score accounts for the differences in gene set size. The false discovery rate q-value was used to set a significance threshold.

RNA sequencing and Ingenuity Pathway Analysis (IPA)

RNA sequencing was performed to observe the altered gene expression signature between DCLK1-B KO and control (EV-transfected) HCT116 cells. Samples were prepared in biological triplicates, and RNA sequencing analysis was performed by LAS science (Seoul, Republic of Korea). Briefly, the mRNA sequencing library was prepared using a TruSeq Stranded mRNA Sample Preparation Kit and the Illumina NextSeq platform. The whole list of DEGs altered in DCLK1-KO HCT116 cells was subjected to IPA (Qiagen, Redwood City, CA, USA) to identify the potential diseases, functions, and upstream regulators that are significantly associated with DCLK1-B KO.

ChIP assay

Reagents were used according to the manufacturer's recommendations (EZ ChIP, #17-371, Upstate). For ChIP assays, cells in culture (70 – 80 % confluent) were fixed in 1 % formaldehyde to crosslink DNA to bound proteins, and the reaction was stopped by adding 0.125 M glycine. Cells were washed with cold PBS, pelleted at 4 °C in the presence of protease inhibitor cocktail (Sigma) and resuspended in 500 μL of ChIP sonication buffer (20 % SDS and protease inhibitors), followed by sonication and centrifugation of fragments (200–700 bp long) at 10,000 RPM. The crosslinked chromatin supernatant was immunoprecipitated using target-specific antibody (2 μg purified IgG) at 4 °C overnight. Control samples contained no antibody. For obtaining input levels of the corresponding proteins, equivalent numbers of cells were also processed for western immunoblot analysis. Protein

A agarose beads, preabsorbed by salmon sperm DNA (100 μg/mL) were added to the chromatin-antibody complex and centrifuged to sediment the beads. The beads were washed with cold buffer, and DNA was eluted from the beads with elution buffer (Elution Reagent C, #20-294). DNA in the supernatant was precipitated using a high-salt method according to the manufacturer's recommendations. The extracted DNA was purified using a kit, and 2 μL of the purified DNA was used for PCR amplification with specific primers designed around the transcription factor binding site of interest. The binding site of XRCC5 on the COX2 promoter was amplified using primer sets shown in Table S5.

In vitro limiting dilution assay

To examine their self-renewal ability, cells were seeded in sphere culture conditions (poly-HEMA-coated plates; Sigma #P3932) at varying cell densities of 10, 100, 500, 1000, 5000 and 10,000. After 14 days of culture, the number of wells with spheres was counted (n = 12/group) and analyzed by the extreme LDA webtool (http://bioinf.wehi.edu.au/software/elda) as described in a previous report [6]. *p* values indicate statistical significance of differences in stem cell frequencies between any of the groups. For the sphere formation assay, cells were seeded at varying cell densities and incubated under sphere culture conditions, and then the number of spheres was counted and their size measured (n = 4/group).

In silico virtual screening

To explore a new compound targeting DCLK1 kinase, *in silico* virtual screening was performed based on the X-ray crystal structure of the DCLK1 kinase domain (Protein Data Bank ID: 5JZN). Interactions of new compound DDQ-1 and DCLK1 kinase domain was investigated by docking simulation using DCOKER algorithms in Discover Studio program (Accelrys, San Diego, CA, USA).

Supplementary references

1. Park SY, Kim JY, Choi JH, Kim JH, Lee CJ, Singh P, et al. Inhibition of LEF1-Mediated DCLK1 by Niclosamide Attenuates Colorectal Cancer Stemness. Clin Cancer Res. 2019; 25: 1415-29.
2. Britton S, Coates J, Jackson SP. A new method for high-resolution imaging of Ku foci to decipher mechanisms of DNA double-strand break repair. J Cell Biol. 2013; 202: 579-95.
3. Chen Z, Cole PA. Synthetic approaches to protein phosphorylation. Current Opinion in Chemical Biology. 2015; 28: 115-22.
4. Park S-Y, Kim J-H, Choi J-H, Lee C-J, Lee W-J, Park S, et al. Lipid raft-disrupting miltefosine preferentially induces the death of colorectal cancer stem-like cells. Clinical and Translational Medicine. 2021; 11: e552.
5. Hua F, Shang S, Yang YW, Zhang HZ, Xu TL, Yu JJ, et al. TRIB3 Interacts With beta-Catenin and TCF4 to Increase Stem Cell Features of Colorectal Cancer Stem Cells and Tumorigenesis. Gastroenterology. 2019; 156: 708-21 e15.
6. Park SY, Kim JY, Jang GB, Choi JH, Kim JH, Lee CJ, et al. Aberrant activation of the CD45-Wnt signaling axis promotes stemness and therapy resistance in colorectal cancer cells. Theranostics. 2021; 11: 8755-70.
7. Zhang Z, Zheng F, Yu Z, Hao J, Chen M, Yu W, et al. XRCC5 cooperates with p300 to promote cyclooxygenase-2 expression and tumor growth in colon cancers. PLoS One. 2017; 12: e0186900.

Table S2. List of DEGs in DCLK1-high versus DCLK1-low tumours from patients with CRC

ID	P.Value	logFC	Gene.symbol	Gene.title
235570_at	8.37E-12	7.43E-01	RBMS3	RNA binding motif single stranded interacting protein 3
238447_at	8.43E-12	8.76E-01	RBMS3	RNA binding motif single stranded interacting protein 3
229530_at	1.21E-11	1.12	GUCY1A3	guanylate cyclase 1 soluble subunit alpha
205609_at	1.26E-11	8.85E-01	ANGPT1	angiopoietin 1
213891_s_at	3.22E-11	7.88E-01	TCF4	transcription factor 4
205529_s_at	4.68E-11	7.31E-01	RUNX1T1	RUNX1 translocation partner 1
209242_at	7.23E-11	5.71E-01	PEG3	paternally expressed 3
203817_at	9.15E-11	8.68E-01	GUCY1B3	guanylate cyclase 1 soluble subunit beta
227235_at	1.09E-10	1.17	GUCY1A3	guanylate cyclase 1 soluble subunit alpha
228827_at	1.89E-10	8.51E-01	RUNX1T1	RUNX1 translocation partner 1
209708_at	3.03E-10	9.18E-01	MOXD1	monooxygenase DBH like 1
205407_at	3.11E-10	7.14E-01	RECK	reversion inducing cysteine rich protein with kazal motifs
212385_at	4.53E-10	6.73E-01	TCF4	transcription factor 4
212764_at	6.13E-10	8.14E-01	ZEB1	zinc finger E-box binding homeobox 1
235518_at	6.21E-10	8.38E-01	SLC8A1	solute carrier family 8 member A1
212386_at	8.50E-10	7.49E-01	TCF4	transcription factor 4
203753_at	1.22E-09	7.25E-01	TCF4	transcription factor 4
212387_at	1.28E-09	7.53E-01	TCF4	transcription factor 4
201843_s_at	1.39E-09	9.05E-01	EFEMP1	EGF containing fibulin like extracellular matrix protein 1
212382_at	1.73E-09	8.52E-01	TCF4	transcription factor 4
1554474_a_at	1.78E-09	4.65E-01	MOXD1	monooxygenase DBH like 1
227662_at	1.97E-09	1.21	SYNPO2	synaptopodin 2
221942_s_at	2.01E-09	9.56E-01	GUCY1A3	guanylate cyclase 1 soluble subunit alpha
228333_at	2.05E-09	6.51E-01	ZEB2	zinc finger E-box binding homeobox 2
228636_at	2.60E-09	9.43E-01	BHLHE22	basic helix-loop-helix family member e22
244050_at	3.32E-09	1.05	HACD4	3-hydroxyacyl-CoA dehydratase 4
212636_at	3.34E-09	9.27E-01	QKI	QKI, KH domain containing RNA binding
232113_at	3.44E-09	1.13		
227561_at	3.46E-09	5.33E-01	DDR2	discoidin domain receptor tyrosine kinase 2
227059_at	3.60E-09	7.75E-01	GPC6	glypican 6
217757_at	4.63E-09	7.30E-01	A2M	alpha-2-macroglobulin
202994_s_at	4.85E-09	7.38E-01	FBLN1	fibulin 1
213306_at	4.89E-09	5.22E-01	MPDZ	multiple PDZ domain crumbs cell polarity complex component
204359_at	5.69E-09	7.18E-01	LOC100506718///FLRT2	uncharacterized LOC100506718///fibronectin leucine rich transmembrane protein 2
206101_at	5.76E-09	7.39E-01	ECM2	extracellular matrix protein 2
227623_at	6.77E-09	8.49E-01	CACNA2D1	calcium voltage-gated channel auxiliary subunit alpha2delta 1
223672_at	8.00E-09	5.36E-01	SGIP1	SH3 domain GRB2 like endophilin interacting protein 1
209335_at	8.70E-09	9.40E-01	DCN	decorin
227554_at	1.22E-08	3.63E-01	MAGI2-AS3	MAGI2 antisense RNA 3
226751_at	1.28E-08	5.07E-01	CNRIP1	cannabinoid receptor interacting protein 1
223475_at	1.65E-08	8.88E-01	CRISPLD1	cysteine rich secretory protein LCCL domain containing 1
233364_s_at	1.75E-08	5.19E-01		
201842_s_at	1.87E-08	1.12	EFEMP1	EGF containing fibulin like extracellular matrix protein 1
227688_at	2.16E-08	4.73E-01	LRCH2	leucine rich repeats and calponin homology domain containing 2
1556427_s_at	3.19E-08	3.97E-01	LRRN4CL	LRRN4 C-terminal like
212758_s_at	3.20E-08	5.36E-01	ZEB1	zinc finger E-box binding homeobox 1
223343_at	3.51E-08	1.01	MS4A7	membrane spanning 4-domains A7
225381_at	3.56E-08	4.76E-01	MIR100HG	mir-100-let-7a-2 cluster host gene
205079_s_at	3.82E-08	3.12E-01	MPDZ	multiple PDZ domain crumbs cell polarity complex component
209210_s_at	3.89E-08	7.65E-01	FERMT2	fermitin family member 2
230550_at	4.11E-08	9.47E-01	MS4A6A	membrane spanning 4-domains A6A
225442_at	4.13E-08	7.36E-01	DDR2	discoidin domain receptor tyrosine kinase 2
203180_at	4.27E-08	9.59E-01	ALDH1A3	aldehyde dehydrogenase 1 family member A3
201163_s_at	4.65E-08	5.17E-01	IGFBP7	insulin like growth factor binding protein 7
212646_at	4.65E-08	6.36E-01	RFTN1	raftlin, lipid raft linker 1
211959_at	4.72E-08	9.85E-01	IGFBP5	insulin like growth factor binding protein 5
202291_s_at	5.90E-08	9.14E-01	MGP	matrix Gla protein
205399_at	5.91E-08	4.47E-01	DCLK1	doublecortin like kinase 1
209539_at	6.26E-08	6.04E-01	ARHGEF6	Rac/Cdc42 guanine nucleotide exchange factor 6
202191_s_at	6.44E-08	4.26E-01	GAS7	growth arrest specific 7
235616_at	6.97E-08	4.63E-01	TSHZ2	teashirt zinc finger homeobox 2
225710_at	7.12E-08	7.81E-01	GNB4	G protein subunit beta 4
212157_at	7.82E-08	5.22E-01	SDC2	syndecan 2
206070_s_at	8.49E-08	7.06E-01	EPHA3	EPH receptor A3
210643_at	1.04E-07	1.29	TNFSF11	tumor necrosis factor superfamily member 11
201162_at	1.05E-07	5.83E-01	IGFBP7	insulin like growth factor binding protein 7
222146_s_at	1.06E-07	5.86E-01	TCF4	transcription factor 4
229357_at	1.14E-07	7.93E-01	ADAMTS5	ADAM metallopeptidase with thrombospondin type 1 motif 5
225782_at	1.14E-07	9.62E-01	MSRB3	methionine sulfoxide reductase B3
205898_at	1.20E-07	5.33E-01	CX3CR1	C-X3-C motif chemokine receptor 1
203485_at	1.35E-07	6.72E-01	RTN1	reticulon 1
212158_at	1.46E-07	7.69E-01	SDC2	syndecan 2
244740_at	1.47E-07	3.07E-01		
230493_at	1.58E-07	1.17	SHISA2	shisa family member 2

227478_at	1.58E-07	8.88E-01	SETBP1	SET binding protein 1
230730_at	1.66E-07	4.00E-01	SGCD	sarcoglycan delta
231001_at	1.69E-07	3.20E-01	FIBIN	fin bud initiation factor homolog (zebrafish)
206481_s_at	1.76E-07	6.15E-01	LDB2	LIM domain binding 2
209200_at	1.81E-07	7.13E-01	MEF2C	myocyte enhancer factor 2C
204557_s_at	1.92E-07	2.65E-01	DZIP1	DAZ interacting zinc finger protein 1
222033_s_at	2.05E-07	3.52E-01	FLT1	fms related tyrosine kinase 1
236600_at	2.22E-07	4.97E-01	SPG20	spastic paraplegia 20 (Troyer syndrome)
219087_at	2.24E-07	1.49	ASPN	asporin
228850_s_at	2.25E-07	4.12E-01	SLIT2	slit guidance ligand 2
208308_s_at	2.36E-07	-6.74E-01	GPI	glucose-6-phosphate isomerase
208396_s_at	2.45E-07	4.35E-01	PDE1A	phosphodiesterase 1A
209348_s_at	2.53E-07	7.26E-01	MAF	MAF bZIP transcription factor
228176_at	2.56E-07	6.39E-01	S1PR3	sphingosine-1-phosphate receptor 3
226769_at	2.71E-07	7.86E-01	FIBIN	fin bud initiation factor homolog (zebrafish)
205982_x_at	2.72E-07	-1.78E-01	SFTPC	surfactant protein C
213800_at	2.78E-07	7.93E-01	CFH	complement factor H
228837_at	2.95E-07	3.27E-01	TCF4	transcription factor 4
235183_at	3.17E-07	5.52E-01	FILIP1	filamin A interacting protein 1
225163_at	3.25E-07	5.47E-01	FRMD4A	FERM domain containing 4A
212526_at	3.48E-07	6.34E-01	SPG20	spastic paraplegia 20 (Troyer syndrome)
203603_s_at	3.51E-07	7.28E-01	ZEB2	zinc finger E-box binding homeobox 2
229800_at	3.63E-07	5.28E-01	DCLK1	doublecortin like kinase 1
223395_at	3.67E-07	1.03	ABI3BP	ABI family member 3 binding protein
228438_at	3.84E-07	3.41E-01	MSC-AS1	MSC antisense RNA 1
231234_at	3.95E-07	3.67E-01	CTSC	cathepsin C
228750_at	4.15E-07	7.14E-01	COL14A1	collagen type XIV alpha 1 chain
242332_at	4.67E-07	2.58E-01	FENDRR	FOXF1 adjacent non-coding developmental regulatory RNA
218718_at	4.95E-07	8.67E-01	PDGFC	platelet derived growth factor C
205608_s_at	5.10E-07	3.31E-01	ANGPT1	angiopoietin 1
219935_at	5.24E-07	5.87E-01	ADAMTS5	ADAM metallopeptidase with thrombospondin type 1 motif 5
208747_s_at	5.41E-07	7.38E-01	C1S	complement component 1, s subcomponent
224694_at	5.42E-07	8.83E-01	ANTXR1	anthrax toxin receptor 1
227761_at	5.49E-07	6.46E-01	MYO5A	myosin VA
1552696_at	5.51E-07	-3.03E-01	NIPA1	non imprinted in Prader-Willi/Angelman syndrome 1
219666_at	5.63E-07	6.42E-01	MS4A6A	membrane spanning 4-domains A6A
212077_at	5.65E-07	6.68E-01	CALD1	caldesmon 1
65718_at	5.71E-07	2.42E-01	ADGRA2	adhesion G protein-coupled receptor A2
202555_s_at	5.90E-07	6.00E-01	MYLK	myosin light chain kinase
212353_at	6.04E-07	1.01	SULF1	sulfatase 1
244521_at	6.50E-07	4.98E-01	TSHZ2	teashirt zinc finger homeobox 2
211813_x_at	6.57E-07	7.06E-01	DCN	decorin
227070_at	7.92E-07	7.84E-01	GLT8D2	glycosyltransferase 8 domain containing 2
206317_s_at	8.01E-07	-2.25E-01	ABCB8	ATP binding cassette subfamily B member 8
219607_s_at	8.01E-07	1.01	MS4A4A	membrane spanning 4-domains A4A
212865_s_at	8.04E-07	6.52E-01	COL14A1	collagen type XIV alpha 1 chain
207173_x_at	8.32E-07	8.51E-01	CDH11	cadherin 11
219427_at	8.93E-07	7.05E-01	FAT4	FAT atypical cadherin 4
222717_at	9.05E-07	5.84E-01	SDPR	serum deprivation response
226322_at	9.36E-07	6.26E-01	TMTC1	transmembrane and tetratricopeptide repeat containing 1
213069_at	9.37E-07	5.62E-01	HEG1	heart development protein with EGF like domains 1
225895_at	9.37E-07	9.41E-01	SYNPO2	synaptopodin 2
211896_s_at	1.03E-06	8.73E-01	DCN	decorin
226621_at	1.05E-06	5.57E-01	OSMR	oncostatin M receptor
231747_at	1.10E-06	6.15E-01	CYSLTR1	cysteinyl leukotriene receptor 1
218656_s_at	1.13E-06	5.88E-01	LHFP	lipoma HMGIC fusion partner
235593_at	1.16E-06	2.75E-01	ZEB2	zinc finger E-box binding homeobox 2
223620_at	1.17E-06	8.14E-01	GPR34	G protein-coupled receptor 34
241789_at	1.19E-06	3.37E-01	RBMS3	RNA binding motif single stranded interacting protein 3
209906_at	1.24E-06	4.56E-01	C3AR1	complement component 3a receptor 1
226225_at	1.32E-06	7.06E-01	MCC	mutated in colorectal cancers
215051_x_at	1.47E-06	4.51E-01	AIF1	allograft inflammatory factor 1
225575_at	1.50E-06	7.19E-01	LIFR	leukemia inhibitory factor receptor alpha
219777_at	1.61E-06	7.55E-01	GIMAP6	GTPase, IMAP family member 6
206932_at	1.77E-06	9.81E-01	CH25H	cholesterol 25-hydroxylase
236277_at	1.85E-06	4.52E-01	PAK3	p21 (RAC1) activated kinase 3
230744_at	1.86E-06	2.61E-01	FSTL1	follistatin like 1
212230_at	1.87E-06	5.58E-01	PLPP3	phospholipid phosphatase 3
229910_at	1.88E-06	3.42E-01	SHE	Src homology 2 domain containing E
223344_s_at	1.88E-06	4.16E-01	MS4A7	membrane spanning 4-domains A7
213416_at	1.95E-06	7.35E-01	ITGA4	integrin subunit alpha 4
227705_at	2.01E-06	4.92E-01	TCEAL7	transcription elongation factor A like 7
213429_at	2.03E-06	7.15E-01	BICC1	BicC family RNA binding protein 1
240232_at	2.07E-06	4.25E-01		
1561099_at	2.08E-06	-1.31E-01	TBC1D22A-AS1	TBC1D22A antisense RNA 1
204464_s_at	2.09E-06	1.06	EDNRA	endothelin receptor type A
225720_at	2.15E-06	7.84E-01	SYNPO2	synaptopodin 2

211365_s_at	2.16E-06	-1.65E-01	PCDHA2	protocadherin alpha 2
215388_s_at	2.20E-06	7.26E-01	CFHR1///CFH	complement factor H related 1///complement factor H
215049_x_at	2.24E-06	7.02E-01	CD163	CD163 molecule
213241_at	2.24E-06	7.10E-01	PLXNC1	plexin C1
208676_s_at	2.28E-06	-2.77E-01	PA2G4	proliferation-associated 2G4
236995_x_at	2.32E-06	1.93E-01	TFEC	transcription factor EC
235318_at	2.36E-06	6.38E-01	FBN1	fibrillin 1
236151_at	2.37E-06	-1.53E-01	SRRM4	serine/arginine repetitive matrix 4
222453_at	2.41E-06	8.24E-01	CYBRD1	cytochrome b reductase 1
213423_x_at	2.42E-06	4.57E-01	TUSC3	tumor suppressor candidate 3
239710_at	2.65E-06	2.31E-01	FIGN	fidgetin, microtubule severing factor
236038_at	2.67E-06	1.92E-01	RNF150	ring finger protein 150
206408_at	2.67E-06	2.04E-01	LRRTM2	leucine rich repeat transmembrane neuronal 2
204939_s_at	2.70E-06	8.58E-01	PLN	phospholamban
228376_at	2.71E-06	4.66E-01	GGTA1P	glycoprotein, alpha-galactosyltransferase 1 pseudogene
204236_at	2.77E-06	6.19E-01	FLI1	Fli-1 proto-oncogene, ETS transcription factor
226237_at	2.79E-06	1.22	COL8A1	collagen type VIII alpha 1 chain
239262_at	2.82E-06	2.99E-01		
228554_at	2.89E-06	2.45E-01	PGR	progesterone receptor
229554_at	2.90E-06	8.37E-01	LUM	lumican
228602_at	2.91E-06	3.81E-01	SGCD	sarcoglycan delta
224356_x_at	3.02E-06	7.56E-01	MS4A6A	membrane spanning 4-domains A6A
214807_at	3.06E-06	6.41E-01	PLXDC2	plexin domain containing 2
209967_s_at	3.07E-06	4.41E-01	CREM	cAMP responsive element modulator
224823_at	3.13E-06	6.49E-01	MYLK	myosin light chain kinase
225275_at	3.14E-06	7.64E-01	EDIL3	EGF like repeats and discoidin domains 3
215951_at	3.14E-06	-1.41E-01	TBC1D2B	TBC1 domain family member 2B
219279_at	3.18E-06	5.73E-01	DOCK10	dedicator of cytokinesis 10
1561615_s_at	3.19E-06	3.91E-01	SLC8A1	solute carrier family 8 member A1
218939_at	3.32E-06	-2.76E-01	LETM1	leucine zipper and EF-hand containing transmembrane protein 1
230722_at	3.37E-06	3.73E-01	BNC2	basonuclin 2
201893_x_at	3.39E-06	7.06E-01	DCN	decorin
211485_s_at	3.61E-06	-2.58E-01	FGF18	fibroblast growth factor 18
203868_s_at	3.77E-06	7.83E-01	VCAM1	vascular cell adhesion molecule 1
226497_s_at	3.78E-06	4.83E-01	FLT1	fms related tyrosine kinase 1
202664_at	3.79E-06	7.05E-01	WIPF1	WAS/WASL interacting protein family member 1
201540_at	3.97E-06	6.41E-01	FHL1	four and a half LIM domains 1
206584_at	4.02E-06	9.27E-01	LY96	lymphocyte antigen 96
227140_at	4.03E-06	7.67E-01	INHBA	inhibin beta A subunit
235368_at	4.04E-06	5.18E-01	ADAMTS5	ADAM metallopeptidase with thrombospondin type 1 motif 5
216232_s_at	4.07E-06	-4.01E-01	GCN1	GCN1, eIF2 alpha kinase activator homolog
208782_at	4.09E-06	5.64E-01	FSTL1	follistatin like 1
232675_s_at	4.10E-06	-2.69E-01	UCKL1	uridine-cytidine kinase 1 like 1
216321_s_at	4.13E-06	8.37E-01	NR3C1	nuclear receptor subfamily 3 group C member 1
204861_s_at	4.13E-06	3.60E-01	NAIP	NLR family apoptosis inhibitory protein
218001_at	4.15E-06	-5.18E-01	MRPS2	mitochondrial ribosomal protein S2
227646_at	4.24E-06	4.75E-01	EBF1	early B-cell factor 1
225990_at	4.34E-06	3.16E-01	BOC	BOC cell adhesion associated, oncogene regulated
239580_at	4.34E-06	2.37E-01	GUCY1A3	guanylate cyclase 1 soluble subunit alpha
218918_at	4.43E-06	5.38E-01	MAN1C1	mannosidase alpha class 1C member 1
230264_s_at	4.54E-06	5.81E-01	AP1S2	adaptor related protein complex 1 sigma 2 subunit
218820_at	4.55E-06	3.60E-01	C14orf132	chromosome 14 open reading frame 132
238458_at	4.56E-06	3.60E-01	MICU3	mitochondrial calcium uptake family member 3
225481_at	4.68E-06	6.30E-01	FRMD6	FERM domain containing 6
206363_at	4.80E-06	4.68E-01	MAF	MAF bZIP transcription factor
210362_x_at	4.83E-06	-1.88E-01	PML	promyelocytic leukemia
243306_s_at	4.95E-06	-1.23E-01	GCC1	GRIP and coiled-coil domain containing 1
203505_at	4.95E-06	5.72E-01	ABCA1	ATP binding cassette subfamily A member 1
223280_x_at	5.06E-06	6.91E-01	MS4A6A	membrane spanning 4-domains A6A
210960_at	5.14E-06	-1.19E-01	ADRA1D	adrenoceptor alpha 1D
229029_at	5.24E-06	2.54E-01	CAMK4	calcium/calmodulin dependent protein kinase IV
229327_s_at	5.29E-06	6.06E-01	MAF	MAF bZIP transcription factor
212067_s_at	5.30E-06	5.26E-01	C1R	complement C1r subcomponent
226841_at	5.32E-06	6.96E-01	MPEG1	macrophage expressed 1
202159_at	5.37E-06	-3.95E-01	FARSA	phenylalanyl-tRNA synthetase alpha subunit
205347_s_at	5.45E-06	6.18E-01	TMSB15B///TMSB15A	thymosin beta 15B///thymosin beta 15a
223774_at	5.59E-06	-4.82E-01	SNORA16A///SNORA61///SNORA44///SNHG12	small nucleolar RNA, H/ACA box 16A///small nucleolar RNA, H/ACA box 61///small nucleolar RNA, H/ACA box 44///small nucleolar RNA host gene 12
228471_at	5.64E-06	6.44E-01	ANKRD44	ankyrin repeat domain 44
232090_at	5.78E-06	6.65E-01	DNM3OS	DNM3 opposite strand/antisense RNA
203923_s_at	5.85E-06	3.99E-01	CYBB	cytochrome b-245 beta chain
217926_at	5.92E-06	-3.72E-01	C19orf53	chromosome 19 open reading frame 53
225721_at	6.03E-06	5.95E-01	SYNPO2	synaptopodin 2
231167_at	6.08E-06	5.46E-01		
203185_at	6.12E-06	4.39E-01	RASSF2	Ras association domain family member 2
203645_s_at	6.14E-06	7.33E-01	CD163	CD163 molecule

202766_s_at	6.17E-06	7.35E-01	FBN1	fibrillin 1
232016_at	6.18E-06	-1.95E-01	FAN1	FANCD2/FANCI-associated nuclease 1
1554483_at	6.42E-06	-2.15E-01	TMEM37	transmembrane protein 37
222885_at	6.65E-06	5.29E-01	EMCN	endomucin
218308_at	6.65E-06	-5.08E-01	TACC3	transforming acidic coiled-coil containing protein 3
204463_s_at	6.79E-06	1.05	EDNRA	endothelin receptor type A
217764_s_at	6.88E-06	7.02E-01	RAB31	RAB31, member RAS oncogene family
217767_at	6.94E-06	9.82E-01	C3	complement component 3
209285_s_at	6.98E-06	4.69E-01	FAM208A	family with sequence similarity 208 member A
223922_x_at	7.01E-06	4.97E-01	MS4A6A	membrane spanning 4-domains A6A
209621_s_at	7.06E-06	5.89E-01	PDLIM3	PDZ and LIM domain 3
227096_at	7.07E-06	-1.86E-01	JOSD2	Josephin domain containing 2
224325_at	7.33E-06	4.36E-01	MIR4683///FZD8	microRNA 4683///frizzled class receptor 8
213910_at	7.37E-06	1.84E-01	IGFBP7	insulin like growth factor binding protein 7
221049_s_at	7.37E-06	-2.20E-01	POLL	polymerase (DNA) lambda
212609_s_at	7.39E-06	6.03E-01	AKT3	AKT serine/threonine kinase 3
204646_at	7.40E-06	9.69E-01	DPYD	dihydropyrimidine dehydrogenase
205528_s_at	7.54E-06	2.41E-01	RUNX1T1	RUNX1 translocation partner 1
201615_x_at	7.54E-06	5.09E-01	CALD1	caldesmon 1
214428_x_at	7.59E-06	5.46E-01	C4B_2///C4B///C4A	complement component 4B (Chido blood group), copy 2///complement component 4B (Chido blood group)///complement component 4A (Rodgers blood group)
209732_at	7.81E-06	7.39E-01	CLEC2B	C-type lectin domain family 2 member B
204682_at	7.94E-06	4.71E-01	LTBP2	latent transforming growth factor beta binding protein 2
201617_x_at	8.01E-06	7.59E-01	CALD1	caldesmon 1
205935_at	8.04E-06	5.59E-01	FOXF1	forkhead box F1
207539_s_at	8.25E-06	-1.21E-01	IL4	interleukin 4
222643_s_at	8.33E-06	-1.33E-01	BBS1	Bardet-Biedl syndrome 1
209550_at	8.41E-06	7.71E-01	NDN	necdin, MAGE family member
1559438_at	8.50E-06	-1.45E-01	C21orf58	chromosome 21 open reading frame 58
219243_at	8.71E-06	5.06E-01	GIMAP4	GTPase, IMAP family member 4
214227_at	8.75E-06	-1.35E-01		
212813_at	8.96E-06	3.57E-01	JAM3	junctional adhesion molecule 3
209493_at	8.96E-06	8.53E-01	PDZD2	PDZ domain containing 2
202115_s_at	9.19E-06	-3.33E-01	NOC2L	NOC2 like nucleolar associated transcriptional repressor
239952_at	9.25E-06	2.11E-01	ZEB1	zinc finger E-box binding homeobox 1
220262_s_at	9.46E-06	-1.76E-01	DLK2	delta like non-canonical Notch ligand 2
214180_at	9.67E-06	3.63E-01	MAN1C1	mannosidase alpha class 1C member 1
207426_s_at	9.81E-06	6.69E-01	TNFSF4	tumor necrosis factor superfamily member 4
225975_at	9.85E-06	6.66E-01	PCDH18	protocadherin 18
213384_x_at	9.89E-06	-2.28E-01	PLCB3	phospholipase C beta 3
222812_s_at	1.01E-05	-3.43E-01	RHOF	ras homolog family member F, filopodia associated
239294_at	1.03E-05	8.13E-01	PIK3CG	phosphatidylinositol-4,5-bisphosphate 3-kinase catalytic subunit gamma
36030_at	1.04E-05	2.80E-01	IFFO1	intermediate filament family orphan 1
213139_at	1.04E-05	6.11E-01	SNAI2	snail family transcriptional repressor 2
230414_s_at	1.08E-05	-1.28E-01	TMEM170A	transmembrane protein 170A
242828_at	1.09E-05	1.60E-01	FIGN	fidgetin, microtubule severing factor
225464_at	1.11E-05	6.42E-01	FRMD6	FERM domain containing 6
1565823_at	1.11E-05	8.05E-01	SEP7	septin 7
227095_at	1.11E-05	5.21E-01	LEPROT	leptin receptor overlapping transcript
207490_at	1.12E-05	-1.85E-01	TUBA4B	tubulin alpha 4b
1555255_a_at	1.14E-05	-3.25E-01	PPIP5K1	diphosphoinositol pentakisphosphate kinase 1
207026_s_at	1.16E-05	-1.83E-01	ATP2B3	ATPase plasma membrane Ca2+ transporting 3
204774_at	1.19E-05	8.45E-01	EVI2A	ecotropic viral integration site 2A
213095_x_at	1.20E-05	4.13E-01	AIF1	allograft inflammatory factor 1
221541_at	1.20E-05	5.94E-01	CRISPLD2	cysteine rich secretory protein LCCL domain containing 2
234721_s_at	1.20E-05	-1.64E-01	CYP26B1	cytochrome P450 family 26 subfamily B member 1
214301_s_at	1.21E-05	-1.24E-01	DPYSL4	dihydropyrimidinase like 4
212844_at	1.23E-05	-2.14E-01	RRP1B	ribosomal RNA processing 1B
201744_s_at	1.23E-05	6.24E-01	LUM	lumican
1566816_at	1.31E-05	-1.64E-01	FGF22	fibroblast growth factor 22
220146_at	1.32E-05	4.21E-01	TLR7	toll like receptor 7
217755_at	1.33E-05	-5.52E-01	HN1	hematological and neurological expressed 1
225450_at	1.34E-05	4.12E-01	AMOTL1	angiomotin like 1
218296_x_at	1.36E-05	-3.32E-01	MSTO2P///MSTO1	misato family member 2, pseudogene///misato 1, mitochondrial distribution and morphology regulator
226766_at	1.36E-05	8.70E-01	ROBO2	roundabout guidance receptor 2
214844_s_at	1.41E-05	3.32E-01	DOK5	docking protein 5
223743_s_at	1.41E-05	-5.89E-01	MRPL4	mitochondrial ribosomal protein L4
224822_at	1.42E-05	6.61E-01	DLC1	DLC1 Rho GTPase activating protein
203083_at	1.43E-05	1.14	THBS2	thrombospondin 2
228665_at	1.44E-05	4.95E-01	CYYR1	cysteine and tyrosine rich 1
209209_s_at	1.44E-05	2.76E-01	FERMT2	fermitin family member 2
224964_s_at	1.44E-05	6.48E-01	GNG2	G protein subunit gamma 2
203989_x_at	1.46E-05	4.82E-01	F2R	coagulation factor II thrombin receptor
232612_s_at	1.47E-05	-3.35E-01	ATG16L1	autophagy related 16 like 1
231213_at	1.48E-05	2.63E-01	PDE1A	phosphodiesterase 1A

200795_at	1.48E-05	7.41E-01	SPARCL1	SPARC like 1
218353_at	1.51E-05	7.08E-01	RGS5	regulator of G-protein signaling 5
202686_s_at	1.56E-05	5.29E-01	AXL	AXL receptor tyrosine kinase
215306_at	1.58E-05	3.22E-01		
222391_at	1.59E-05	4.36E-01	TMEM30A	transmembrane protein 30A
231993_at	1.60E-05	3.32E-01	ITGBL1	integrin subunit beta like 1
228692_at	1.63E-05	5.33E-01	PREX2	phosphatidylinositol-3,4,5-trisphosphate dependent Rac exchange factor 2
231983_at	1.65E-05	-3.56E-01	IBA57	IBA57 homolog, iron-sulfur cluster assembly
219574_at	1.70E-05	4.66E-01	01-3	membrane associated ring-CH-type finger 1
222671_s_at	1.72E-05	-2.88E-01	JMJD4	jumonji domain containing 4
241698_at	1.72E-05	2.15E-01	RFTN2	raftlin family member 2
229383_at	1.73E-05	4.79E-01	01-3	membrane associated ring-CH-type finger 1
1562903_at	1.74E-05	-1.63E-01	FAM86B3P	family with sequence similarity 86, member A pseudogene
207866_at	1.74E-05	-1.67E-01	BMP8A	bone morphogenetic protein 8a
214326_x_at	1.80E-05	-1.84E-01	JUND	JunD proto-oncogene, AP-1 transcription factor subunit
227360_at	1.81E-05	-1.52E-01		
238478_at	1.82E-05	3.96E-01	BNC2	basonuclin 2
206715_at	1.84E-05	7.19E-01	TFEC	transcription factor EC
238146_at	1.86E-05	4.89E-01		
223690_at	1.87E-05	3.79E-01	LTBP2	latent transforming growth factor beta binding protein 2
243366_s_at	1.90E-05	5.91E-01	ITGA4	integrin subunit alpha 4
210302_s_at	1.90E-05	4.69E-01	MAB21L2	mab-21 like 2
214770_at	1.91E-05	6.18E-01	MSR1	macrophage scavenger receptor 1
201264_at	1.94E-05	-4.71E-01	COPE	coatomer protein complex subunit epsilon
202948_at	1.95E-05	5.59E-01	IL1R1	interleukin 1 receptor type 1
1555611_s_at	1.96E-05	-1.59E-01	MBD1	methyl-CpG binding domain protein 1
243041_s_at	1.96E-05	1.86E-01		
60794_f_at	1.98E-05	4.95E-01	ZNF814	zinc finger protein 814
227874_at	1.99E-05	4.36E-01	EMCN	endomucin
204940_at	2.01E-05	4.72E-01	PLN	phospholamban
210405_x_at	2.05E-05	-3.35E-01	TNFRSF10B	TNF receptor superfamily member 10b
228149_at	2.05E-05	4.89E-01	BMT2	base methyltransferase of 25S rRNA 2 homolog
201168_x_at	2.05E-05	-2.96E-01	ARHGDIA	Rho GDP dissociation inhibitor alpha
201251_at	2.07E-05	-5.74E-01	PKM	pyruvate kinase, muscle
1554553_s_at	2.08E-05	-4.02E-01	YIF1B	Yip1 interacting factor homolog B, membrane trafficking protein
244741_s_at	2.11E-05	2.28E-01	ZNF667-AS1	ZNF667 antisense RNA 1 (head to head)
202450_s_at	2.12E-05	6.42E-01	CTSK	cathepsin K
203926_x_at	2.14E-05	-3.44E-01	ATP5D	ATP synthase, H+ transporting, mitochondrial F1 complex, delta subunit
1570307_s_at	2.16E-05	-1.62E-01	ST18	ST18, C2H2C-type zinc finger
226771_at	2.17E-05	4.12E-01	ATP8B2	ATPase phospholipid transporting 8B2
231980_at	2.20E-05	1.44E-01	DOK6	docking protein 6
1555222_a_at	2.20E-05	-1.33E-01	LOC101927122	uncharacterized LOC101927122
213726_x_at	2.20E-05	-3.95E-01	TUBB4B	tubulin beta 4B class IVb
208131_s_at	2.20E-05	4.45E-01	PTGIS	prostaglandin I2 (prostacyclin) synthase
204894_s_at	2.21E-05	4.20E-01	AOC3	amine oxidase, copper containing 3
230467_at	2.21E-05	-2.96E-01	TMEM52	transmembrane protein 52
1552316_a_at	2.27E-05	4.07E-01	GIMAP1	GTPase, IMAP family member 1
204749_at	2.27E-05	4.51E-01	NAP1L3	nucleosome assembly protein 1 like 3
203838_s_at	2.28E-05	-1.98E-01	TNK2	tyrosine kinase non receptor 2
212990_at	2.33E-05	3.99E-01	SYNJ1	synaptojanin 1
200986_at	2.35E-05	5.78E-01	SERPING1	serpin family G member 1
229859_at	2.36E-05	-1.89E-01		
232544_at	2.40E-05	2.08E-01		
229968_at	2.41E-05	4.06E-01		
226905_at	2.48E-05	4.74E-01	FAM101B	family with sequence similarity 101 member B
226047_at	2.48E-05	3.97E-01	MRVI1	murine retrovirus integration site 1 homolog
226876_at	2.49E-05	3.75E-01	FAM101B	family with sequence similarity 101 member B
204930_s_at	2.50E-05	-3.02E-01	BNIP1	BCL2 interacting protein 1
204619_s_at	2.53E-05	6.98E-01	VCAN	versican
230147_at	2.60E-05	7.06E-01	F2RL2	coagulation factor II thrombin receptor like 2
221933_at	2.60E-05	3.27E-01	NLGN4X	neuroligin 4, X-linked
236534_at	2.60E-05	-1.50E-01	BNIPL///C1orf56	BCL2 interacting protein like///chromosome 1 open reading frame 56
235385_at	2.63E-05	4.17E-01	MARCHF1	membrane associated ring-CH-type finger 1
215107_s_at	2.65E-05	-1.16E-01	TTC22	tetratricopeptide repeat domain 22
206091_at	2.66E-05	1.94E-01	MATN3	matrilin 3
209392_at	2.68E-05	9.18E-01	ENPP2	ectonucleotide pyrophosphatase/phosphodiesterase 2
203539_s_at	2.74E-05	-1.55E-01	GFAP	glial fibrillary acidic protein
205880_at	2.75E-05	5.15E-01	PRKD1	protein kinase D1
235471_at	2.76E-05	2.02E-01	VSTM4	V-set and transmembrane domain containing 4
227061_at	2.78E-05	3.38E-01	LINC01279	long intergenic non-protein coding RNA 1279
216323_x_at	2.78E-05	-2.96E-01	TUBA3D///TUBA3C	tubulin alpha 3d///tubulin alpha 3c
230145_at	2.78E-05	-1.20E-01	DUS3L	dihydrouridine synthase 3 like
228640_at	2.78E-05	1.01	PCDH7	protocadherin 7
235306_at	2.79E-05	4.62E-01	GIMAP8	GTPase, IMAP family member 8
226818_at	2.80E-05	6.58E-01	MPEG1	macrophage expressed 1
91952_at	2.83E-05	-2.90E-01	DCAF15	DDB1 and CUL4 associated factor 15
221731_x_at	2.86E-05	6.51E-01	VCAN	versican

1566324_a_at	2.86E-05	1.29E-01	MAF	MAF bZIP transcription factor
211730_s_at	2.88E-05	-5.81E-01	POLR2L	RNA polymerase II subunit L
230391_at	2.91E-05	4.88E-01	CD84	CD84 molecule
202988_s_at	2.92E-05	8.79E-01	RGS1	regulator of G-protein signaling 1
205923_at	2.94E-05	2.32E-01	RELN	reelin
1558118_at	2.95E-05	-1.39E-01	LOC100287576///DGCR5	uncharacterized LOC100287576///DiGeorge syndrome critical region gene 5 (non-protein coding)
218225_at	2.98E-05	-3.88E-01	ECSIT	ECSIT signalling integrator
236644_at	2.99E-05	1.08E-01	RNF180	ring finger protein 180
203504_s_at	2.99E-05	6.69E-01	ABCA1	ATP binding cassette subfamily A member 1
1553592_x_at	3.02E-05	-1.53E-01	BCRP3	breakpoint cluster region pseudogene 3
203131_at	3.03E-05	7.86E-01	PDGFRA	platelet derived growth factor receptor alpha
204863_s_at	3.03E-05	4.47E-01	IL6ST	interleukin 6 signal transducer
217525_at	3.05E-05	3.47E-01	OLFML1	olfactomedin like 1
209540_at	3.06E-05	2.31E-01	IGF1	insulin like growth factor 1
208977_x_at	3.08E-05	-4.49E-01	TUBB4B	tubulin beta 4B class IVb
208611_s_at	3.09E-05	-4.27E-01	SPTAN1	spectrin alpha, non-erythrocytic 1
226828_s_at	3.11E-05	3.12E-01	HEYL	hes related family bHLH transcription factor with YRPW motif-like
205409_at	3.26E-05	-2.05E-01	FOSL2	FOS like 2, AP-1 transcription factor subunit
230955_s_at	3.35E-05	-1.95E-01	NOL4L	nucleolar protein 4 like
1562924_at	3.35E-05	-1.41E-01	LOC340357	uncharacterized LOC340357
219584_at	3.37E-05	4.29E-01	PLA1A	phospholipase A1 member A
204273_at	3.39E-05	9.82E-01	EDNRB	endothelin receptor type B
236432_at	3.41E-05	4.44E-01		
203184_at	3.43E-05	6.58E-01	FBN2	fibrillin 2
242134_at	3.45E-05	7.02E-01		
214985_at	3.46E-05	3.66E-01	EXT1	exostosin glycosyltransferase 1
204787_at	3.49E-05	3.84E-01	VSIG4	V-set and immunoglobulin domain containing 4
226241_s_at	3.57E-05	-4.21E-01	MRPL52	mitochondrial ribosomal protein L52
221650_s_at	3.60E-05	-2.78E-01	LOC100996782///MED18	mediator of RNA polymerase II transcription subunit 18-like///mediator complex subunit 18
209228_x_at	3.61E-05	3.06E-01	TUSC3	tumor suppressor candidate 3
204688_at	3.62E-05	8.53E-01	SGCE	sarcoglycan epsilon
229461_x_at	3.63E-05	3.95E-01	NEGR1	neuronal growth regulator 1
209496_at	3.66E-05	5.68E-01	RARRES2	retinoic acid receptor responder 2
228776_at	3.70E-05	4.45E-01	GJC1	gap junction protein gamma 1
211671_s_at	3.75E-05	6.64E-01	NR3C1	nuclear receptor subfamily 3 group C member 1
230511_at	3.78E-05	3.59E-01	CREM	cAMP responsive element modulator
224233_s_at	3.78E-05	-3.16E-01	MSTO1	misato 1, mitochondrial distribution and morphology regulator
218688_at	3.84E-05	-3.89E-01	TKFC	triokinase and FMN cyclase
221667_s_at	3.90E-05	2.32E-01	HSPB8	heat shock protein family B (small) member 8
209370_s_at	3.92E-05	-2.14E-01	SH3BP2	SH3 domain binding protein 2
224357_s_at	3.93E-05	2.10E-01	MS4A4A	membrane spanning 4-domains A4A
232351_at	3.95E-05	3.31E-01		
216568_x_at	3.99E-05	-4.63E-01		
212587_s_at	4.03E-05	7.70E-01	PTPRC	protein tyrosine phosphatase, receptor type C
205099_s_at	4.07E-05	3.21E-01	CCR1	C-C motif chemokine receptor 1
213894_at	4.10E-05	2.37E-01	THSD7A	thrombospondin type 1 domain containing 7A
208379_x_at	4.19E-05	-1.62E-01	NPY2R	neuropeptide Y receptor Y2
214803_at	4.21E-05	4.21E-01	CDH6	cadherin 6
1554539_a_at	4.26E-05	-4.23E-01	RHOF	ras homolog family member F, filopodia associated
212607_at	4.29E-05	6.06E-01	AKT3	AKT serine/threonine kinase 3
201721_s_at	4.30E-05	5.95E-01	LAPTM5	lysosomal protein transmembrane 5
217786_at	4.32E-05	-4.50E-01	PRMT5	protein arginine methyltransferase 5
203697_at	4.35E-05	8.42E-01	FRZB	frizzled-related protein
218559_s_at	4.36E-05	5.79E-01	MAFB	MAF bZIP transcription factor B
216834_at	4.38E-05	6.83E-01	RGS1	regulator of G-protein signaling 1
1561286_a_at	4.38E-05	-3.64E-01	DIP2A	disco interacting protein 2 homolog A
228410_at	4.39E-05	3.23E-01	GAB3	GRB2 associated binding protein 3
218009_s_at	4.50E-05	-4.74E-01	PRC1	protein regulator of cytokinesis 1
226682_at	4.54E-05	8.05E-01	RORA	RAR related orphan receptor A
229367_s_at	4.57E-05	3.80E-01	GIMAP6	GTPase, IMAP family member 6
218522_s_at	4.59E-05	-3.53E-01	MAP1S	microtubule associated protein 1S
212955_s_at	4.62E-05	-4.49E-01	POLR2I	RNA polymerase II subunit I
206911_at	4.64E-05	-1.81E-01	TRIM25	tripartite motif containing 25
207922_s_at	4.65E-05	-3.59E-01	MAEA	macrophage erythroblast attacher
218901_at	4.69E-05	7.11E-01	PLSCR4	phospholipid scramblase 4
228218_at	4.74E-05	7.03E-01	LSAMP	limbic system-associated membrane protein
1554547_at	4.76E-05	6.61E-01	FAM13C	family with sequence similarity 13 member C
212218_s_at	4.78E-05	-4.06E-01	FASN	fatty acid synthase
206234_s_at	4.79E-05	-1.82E-01	MMP17	matrix metallopeptidase 17
204556_s_at	4.80E-05	2.07E-01	DZIP1	DAZ interacting zinc finger protein 1
1556075_at	4.82E-05	-1.05E-01	BMPR2	bone morphogenetic protein receptor type 2
219304_s_at	4.84E-05	7.47E-01	PDGFD	platelet derived growth factor D
219214_s_at	4.94E-05	-3.27E-01	NT5C	5', 3'-nucleotidase, cytosolic
230689_at	4.97E-05	5.78E-01		
217903_at	5.00E-05	-2.67E-01	STRN4	striatin 4

47550_at	5.02E-05	2.57E-01	LZTS1	leucine zipper tumor suppressor 1
207824_s_at	5.05E-05	-2.08E-01	MAZ	MYC associated zinc finger protein
226437_at	5.05E-05	-4.94E-01	YIF1B	Yip1 interacting factor homolog B, membrane trafficking protein
212139_at	5.07E-05	-3.27E-01	GCN1	GCN1, eIF2 alpha kinase activator homolog
227937_at	5.11E-05	-1.74E-01	MYPOP	Myb related transcription factor, partner of profilin
215841_at	5.11E-05	-1.41E-01	GUCA1B	guanylate cyclase activator 1B
31637_s_at	5.14E-05	-2.43E-01	NR1D1///THRA	nuclear receptor subfamily 1 group D member 1///thyroid hormone receptor, alpha
230422_at	5.15E-05	5.61E-01	FPR3	formyl peptide receptor 3
204793_at	5.15E-05	4.36E-01	GPRASP1	G protein-coupled receptor associated sorting protein 1
206074_s_at	5.19E-05	-4.80E-01	HMGA1	high mobility group AT-hook 1
1553178_a_at	5.21E-05	-1.14E-01	SSTR3	somatostatin receptor 3
228071_at	5.22E-05	7.16E-01	GIMAP7	GTPase, IMAP family member 7
210575_at	5.24E-05	-1.22E-01	NUDC	nuclear distribution C, dynein complex regulator
229942_at	5.24E-05	2.84E-01	BNC2	basonuclin 2
201858_s_at	5.25E-05	7.51E-01	SRGN	serglycin
222162_s_at	5.27E-05	6.47E-01	ADAMTS1	ADAM metallopeptidase with thrombospondin type 1 motif 1
238673_at	5.29E-05	7.28E-01	SAMD12	sterile alpha motif domain containing 12
64438_at	5.30E-05	-1.98E-01	OGFOD3	2-oxoglutarate and iron dependent oxygenase domain containing 3
242388_x_at	5.31E-05	3.54E-01	TAGAP	T-cell activation RhoGTPase activating protein
212588_at	5.37E-05	8.04E-01	PTPRC	protein tyrosine phosphatase, receptor type C
203710_at	5.44E-05	4.41E-01	ITPR1	inositol 1,4,5-trisphosphate receptor type 1
216490_x_at	5.48E-05	-1.78E-01		
217066_s_at	5.49E-05	-1.50E-01	DMPK	dystrophia myotonica protein kinase
219315_s_at	5.50E-05	3.66E-01	TMEM204	transmembrane protein 204
201979_s_at	5.55E-05	-2.08E-01	PPP5C	protein phosphatase 5 catalytic subunit
226397_s_at	5.57E-05	3.01E-01	PHACTR1	phosphatase and actin regulator 1
204589_at	5.58E-05	5.17E-01	NUAK1	NUAK family kinase 1
211716_x_at	5.59E-05	-3.19E-01	ARHGDIA	Rho GDP dissociation inhibitor alpha
223425_at	5.59E-05	-2.51E-01	RAVER1	ribonucleoprotein, PTB binding 1
201616_s_at	5.63E-05	6.64E-01	CALD1	caldesmon 1
241752_at	5.66E-05	1.35E-01	SLC8A1	solute carrier family 8 member A1
211905_s_at	5.78E-05	-1.79E-01	ITGB4	integrin subunit beta 4
227370_at	5.80E-05	5.81E-01	FAM171B	family with sequence similarity 171 member B
1555731_a_at	5.86E-05	-4.60E-01	AP1S3	adaptor related protein complex 1 sigma 3 subunit
1554192_s_at	5.87E-05	-1.95E-01	TRMT44	tRNA methyltransferase 44 homolog (S. cerevisiae)
209518_at	5.93E-05	-1.85E-01	SMARCD1	SWI/SNF related, matrix associated, actin dependent regulator of chromatin, subfamily d, member 1
204726_at	5.95E-05	3.58E-01	CDH13	cadherin 13
202202_s_at	6.01E-05	4.66E-01	LAMA4	laminin subunit alpha 4
229356_x_at	6.03E-05	-2.38E-01	INO80	INO80 complex subunit
238408_at	6.04E-05	6.70E-01		
219892_at	6.04E-05	2.61E-01	TM6SF1	transmembrane 6 superfamily member 1
241370_at	6.07E-05	6.77E-01	LOC286052	uncharacterized LOC286052
209901_x_at	6.09E-05	3.54E-01	AIF1	allograft inflammatory factor 1
207410_s_at	6.09E-05	-1.25E-01	TLX2	T-cell leukemia homeobox 2
201716_at	6.10E-05	-3.35E-01	SNX1	sorting nexin 1
240590_at	6.15E-05	-1.27E-01	SPATA3-AS1	SPATA3 antisense RNA 1 (head to head)
223393_s_at	6.18E-05	3.49E-01	TSHZ3	teashirt zinc finger homeobox 3
243481_at	6.18E-05	1.55E-01	RHOJ	ras homolog family member J
201175_at	6.22E-05	-3.71E-01	TMX2	thioredoxin related transmembrane protein 2
229487_at	6.27E-05	2.36E-01	EBF1	early B-cell factor 1
244593_at	6.33E-05	-1.44E-01	HID1	HID1 domain containing
227691_at	6.36E-05	-1.43E-01	AKAP8L	A-kinase anchoring protein 8 like
205280_at	6.45E-05	1.97E-01	GLRB	glycine receptor beta
1560439_at	6.48E-05	-1.29E-01	LINGO3	leucine rich repeat and Ig domain containing 3
224310_s_at	6.51E-05	-1.41E-01	BCL11B	B-cell CLL/lymphoma 11B
204620_s_at	6.57E-05	5.86E-01	VCAN	versican
211546_x_at	6.66E-05	-1.33E-01	SNCA	synuclein alpha
204959_at	6.69E-05	6.37E-01	MNDA	myeloid cell nuclear differentiation antigen
1566168_at	6.76E-05	-1.28E-01		
226201_at	6.85E-05	-3.13E-01	DOT1L	DOT1 like histone lysine methyltransferase
1555938_x_at	6.90E-05	4.83E-01	VIM	vimentin
244455_at	6.92E-05	1.91E-01	KCNT2	potassium sodium-activated channel subfamily T member 2
212432_at	6.93E-05	-4.39E-01	GRPEL1	GrpE like 1, mitochondrial
211222_s_at	6.95E-05	-1.53E-01	HAP1	huntingtin associated protein 1
1554864_a_at	6.98E-05	-1.36E-01	SDC3	syndecan 3
219947_at	7.04E-05	6.25E-01	CLEC4A	C-type lectin domain family 4 member A
1554728_at	7.18E-05	-1.00E-01	SLC9A1	solute carrier family 9 member A1
241827_at	7.25E-05	5.30E-01	ZNF615	zinc finger protein 615
226931_at	7.30E-05	3.97E-01	TMTC1	transmembrane and tetratricopeptide repeat containing 1
220630_s_at	7.32E-05	-1.65E-01	CHIA	chitinase, acidic
219213_at	7.33E-05	2.68E-01	JAM2	junctional adhesion molecule 2
213041_s_at	7.34E-05	-5.00E-01	ATP5D	ATP synthase, H+ transporting, mitochondrial F1 complex, delta subunit
214423_x_at	7.40E-05	-1.33E-01	ALDOB	aldolase, fructose-bisphosphate B
226607_at	7.44E-05	3.18E-01	C20orf194	chromosome 20 open reading frame 194
236393_at	7.45E-05	-1.45E-01		

212354_at	7.46E-05	7.53E-01	SULF1	sulfatase 1
210806_at	7.46E-05	-1.64E-01	TTLL5	tubulin tyrosine ligase like 5
64418_at	7.47E-05	3.42E-01	SYNRG	synergin, gamma
218105_s_at	7.58E-05	-3.78E-01	MRPL4	mitochondrial ribosomal protein L4
202167_s_at	7.59E-05	-3.72E-01	MMS19	MMS19 homolog, cytosolic iron-sulfur assembly component
213543_at	7.63E-05	2.86E-01	SGCD	sarcoglycan delta
1553594_a_at	7.69E-05	-1.49E-01	INSL3	insulin like 3
230866_at	7.79E-05	3.85E-01	CYSLTR1	cysteinyl leukotriene receptor 1
201865_x_at	7.81E-05	5.87E-01	NR3C1	nuclear receptor subfamily 3 group C member 1
226664_at	7.83E-05	-3.25E-01	TBC1D20	TBC1 domain family member 20
214927_at	7.92E-05	4.32E-01	ITGBL1	integrin subunit beta like 1
226641_at	7.96E-05	7.28E-01	ANKRD44	ankyrin repeat domain 44
225285_at	7.97E-05	6.53E-01	BCAT1	branched chain amino acid transaminase 1
229127_at	7.98E-05	2.11E-01	JAM2	junctional adhesion molecule 2
238472_at	8.03E-05	3.63E-01	FBXO9	F-box protein 9
218780_at	8.07E-05	-3.40E-01	HOOK2	hook microtubule tethering protein 2
235438_at	8.07E-05	4.49E-01	CYP7B1	cytochrome P450 family 7 subfamily B member 1
206702_at	8.07E-05	4.53E-01	TEK	TEK receptor tyrosine kinase
216063_at	8.09E-05	-1.39E-01	HBBP1	hemoglobin subunit beta pseudogene 1
1556209_at	8.11E-05	2.66E-01	CLEC2B	C-type lectin domain family 2 member B
210704_at	8.16E-05	-1.32E-01	FEZ2	fasciculation and elongation protein zeta 2
228423_at	8.20E-05	1.2	MAP9	microtubule associated protein 9
242845_at	8.23E-05	1.29E-01	RUNX1T1	RUNX1 translocation partner 1
220635_at	8.25E-05	-1.72E-01	PSORS1C2	psoriasis susceptibility 1 candidate 2
209031_at	8.26E-05	8.37E-01	CADM1	cell adhesion molecule 1
218824_at	8.30E-05	3.08E-01	PNMAL1	paraneoplastic Ma antigen family like 1
214794_at	8.31E-05	-4.47E-01	PA2G4	proliferation-associated 2G4
226677_at	8.35E-05	4.57E-01	ZNF521	zinc finger protein 521
219554_at	8.39E-05	-3.61E-01	RHCG	Rh family C glycoprotein
220894_x_at	8.49E-05	-1.79E-01	PRDM12	PR/SET domain 12
209199_s_at	8.61E-05	5.21E-01	MEF2C	myocyte enhancer factor 2C
203254_s_at	8.62E-05	-2.33E-01	TLN1	talin 1
206420_at	8.63E-05	5.06E-01	IGSF6	immunoglobulin superfamily member 6
209473_at	8.64E-05	3.70E-01	ENTPD1	ectonucleoside triphosphate diphosphohydrolase 1
229802_at	8.66E-05	7.06E-01	WISP1	WNT1 inducible signaling pathway protein 1
222440_s_at	8.76E-05	-1.48E-01	THRAP3	thyroid hormone receptor associated protein 3
218521_s_at	8.81E-05	4.40E-01	UBE2W	ubiquitin conjugating enzyme E2 W (putative)
224710_at	8.83E-05	3.29E-01	NARR///RAB34	nine-amino acid residue-repeats///RAB34, member RAS oncogene family
240845_at	8.85E-05	4.11E-01		
202109_at	8.86E-05	-3.24E-01	ARFIP2	ADP ribosylation factor interacting protein 2
1560620_at	8.86E-05	-1.78E-01	KCNK15	potassium two pore domain channel subfamily K member 15
222349_x_at	8.88E-05	-2.32E-01	RNF126P1	ring finger protein 126 pseudogene 1
227394_at	8.94E-05	2.77E-01	NCAM1	neural cell adhesion molecule 1
233534_at	9.06E-05	-9.30E-02	KRTAP3-2	keratin associated protein 3-2
208357_x_at	9.17E-05	-1.27E-01	CSH1	chorionic somatomammotropin hormone 1
237387_at	9.18E-05	2.33E-01		
216675_at	9.33E-05	-1.48E-01		
210328_at	9.34E-05	-1.60E-01	GNMT	glycine N-methyltransferase
231297_at	9.38E-05	-2.04E-01	DOT1L	DOT1 like histone lysine methyltransferase
1560359_at	9.39E-05	4.70E-01	ITGA1	integrin subunit alpha 1
1552395_at	9.44E-05	-1.84E-01	TSSK3	testis specific serine kinase 3
215504_x_at	9.57E-05	2.56E-01	ANKRD10-IT1	ANKRD10 intronic transcript 1
216865_at	9.64E-05	-1.19E-01	COL14A1	collagen type XIV alpha 1 chain
204052_s_at	9.67E-05	8.63E-01	SFRP4	secreted frizzled related protein 4
230371_at	9.69E-05	-1.18E-01	HPS6	HPS6, biogenesis of lysosomal organelles complex 2 subunit 3
236933_at	9.71E-05	-1.34E-01	EVPLL	envoplakin like
227082_at	9.72E-05	6.73E-01	ZBTB20	zinc finger and BTB domain containing 20
204069_at	9.73E-05	4.87E-01	MEIS1	Meis homeobox 1
229969_at	9.73E-05	4.37E-01	SEC63	SEC63 homolog, protein translocation regulator
210762_s_at	9.79E-05	5.25E-01	DLC1	DLC1 Rho GTPase activating protein
203550_s_at	9.79E-05	-2.61E-01	FAM189B	family with sequence similarity 189 member B
1554386_at	9.92E-05	-1.37E-01	CST9	cystatin 9
1562910_at	9.96E-05	-1.44E-01	SH3PXD2B	SH3 and PX domains 2B
205015_s_at	1.01E-04	-1.47E-01	TGFA	transforming growth factor alpha
223857_x_at	1.01E-04	-3.36E-01	EMC4	ER membrane protein complex subunit 4
1555715_a_at	1.01E-04	-1.49E-01	HRH3	histamine receptor H3
204424_s_at	1.01E-04	2.88E-01	LMO3	LIM domain only 3
44617_at	1.03E-04	-2.50E-01	OGFOD2	2-oxoglutarate and iron dependent oxygenase domain containing 2
204570_at	1.03E-04	2.88E-01	COX7A1	cytochrome c oxidase subunit 7A1
1553952_at	1.03E-04	-1.66E-01	ZDHHC19	zinc finger DHHC-type containing 19
210100_s_at	1.04E-04	-1.50E-01	ABCA2	ATP binding cassette subfamily A member 2
224019_at	1.04E-04	-1.16E-01	SCD5	stearoyl-CoA desaturase 5
225912_at	1.05E-04	5.82E-01	TP53INP1	tumor protein p53 inducible nuclear protein 1
227549_x_at	1.05E-04	-2.27E-01	ZDHHC24	zinc finger DHHC-type containing 24
221000_s_at	1.05E-04	-1.91E-01	KAZALD1	Kazal type serine peptidase inhibitor domain 1
225103_at	1.05E-04	-3.97E-01	MRPL38	mitochondrial ribosomal protein L38
223694_at	1.05E-04	-8.08E-01	TRIM7	tripartite motif containing 7

211012_s_at	1.07E-04	-2.71E-01	PML	promyelocytic leukemia
223809_at	1.07E-04	4.38E-01	RGS18	regulator of G-protein signaling 18
224913_s_at	1.07E-04	-5.25E-01	TIMM50	translocase of inner mitochondrial membrane 50
225350_s_at	1.07E-04	2.90E-01	ZYG11B	zyg-11 family member B, cell cycle regulator
221900_at	1.08E-04	3.34E-01	COL8A2	collagen type VIII alpha 2 chain
206953_s_at	1.09E-04	5.05E-01	ADGRL2	adhesion G protein-coupled receptor L2
209687_at	1.09E-04	6.43E-01	CXCL12	C-X-C motif chemokine ligand 12
211662_s_at	1.10E-04	-3.40E-01	VDAC2	voltage dependent anion channel 2
216243_s_at	1.10E-04	-1.76E-01	IL1RN	interleukin 1 receptor antagonist
226517_at	1.10E-04	7.07E-01	BCAT1	branched chain amino acid transaminase 1
229912_at	1.10E-04	1.87E-01	SDK1	sidekick cell adhesion molecule 1
226425_at	1.11E-04	6.49E-01	CLIP4	CAP-Gly domain containing linker protein family member 4
202945_at	1.11E-04	-3.05E-01	FPGS	folylpolyglutamate synthase
201441_at	1.11E-04	-3.31E-01	COX6B1	cytochrome c oxidase subunit 6B1
1555943_at	1.12E-04	-2.90E-01	PGAM5	PGAM family member 5, mitochondrial serine/threonine protein phosphatase
1554967_at	1.12E-04	-1.41E-01	DIP2A	disco interacting protein 2 homolog A
230413_s_at	1.13E-04	6.09E-01		
226103_at	1.13E-04	6.26E-01	NEXN	nexilin F-actin binding protein
202475_at	1.13E-04	-3.83E-01	TMEM147	transmembrane protein 147
1563302_at	1.14E-04	-1.19E-01	LOC101927534	uncharacterized LOC101927534
210775_x_at	1.14E-04	-2.16E-01	CASP9	caspase 9
211794_at	1.15E-04	2.92E-01	FYB	FYN binding protein
214462_at	1.15E-04	-3.24E-01	SOCS6	suppressor of cytokine signaling 6
202415_s_at	1.15E-04	-3.42E-01	HSPBP1	HSPA (Hsp70) binding protein 1
201859_at	1.16E-04	5.55E-01	SRGN	serglycin
228225_at	1.17E-04	3.38E-01	PEX2	peroxisomal biogenesis factor 2
227405_s_at	1.18E-04	2.89E-01	MIR4683///FZD8	microRNA 4683///frizzled class receptor 8
230071_at	1.18E-04	4.34E-01	11-9	septin 11
209126_x_at	1.19E-04	-2.66E-01	KRT6B	keratin 6B
213781_at	1.21E-04	-1.51E-01	PPP1R37	protein phosphatase 1 regulatory subunit 37
203416_at	1.21E-04	5.24E-01	CD53	CD53 molecule
230130_at	1.21E-04	8.58E-01	SLIT2	slit guidance ligand 2
217140_s_at	1.22E-04	-5.12E-01	VDAC1	voltage dependent anion channel 1
217762_s_at	1.22E-04	5.52E-01	RAB31	RAB31, member RAS oncogene family
212670_at	1.23E-04	1.42E-01	ELN	elastin
224468_s_at	1.23E-04	-4.12E-01	SNORD88C///C19orf48	small nucleolar RNA, C/D box 88C///chromosome 19 open reading frame 48
215581_s_at	1.24E-04	-1.28E-01	MCM3AP	minichromosome maintenance complex component 3 associated protein
236179_at	1.24E-04	5.86E-01	CDH11	cadherin 11
231629_x_at	1.24E-04	-1.68E-01	KLK3	kallikrein related peptidase 3
236125_at	1.25E-04	5.46E-01		
230024_at	1.25E-04	-2.24E-01	AARS2	alanyl-tRNA synthetase 2, mitochondrial
223389_s_at	1.25E-04	-3.64E-01	ZNF581	zinc finger protein 581
209030_s_at	1.26E-04	4.77E-01	CADM1	cell adhesion molecule 1
228728_at	1.26E-04	7.27E-01	CPED1	cadherin like and PC-esterase domain containing 1
201226_at	1.27E-04	-4.28E-01	NDUFB8	NADH:ubiquinone oxidoreductase subunit B8
219506_at	1.27E-04	3.79E-01	C1orf54	chromosome 1 open reading frame 54
225357_s_at	1.28E-04	-1.96E-01	INO80	INO80 complex subunit
232569_at	1.28E-04	2.61E-01		
1557961_s_at	1.28E-04	2.88E-01	C8orf88	chromosome 8 open reading frame 88
226498_at	1.28E-04	5.10E-01	FLT1	fms related tyrosine kinase 1
216381_x_at	1.29E-04	-4.58E-01	AKR7A3	aldo-keto reductase family 7 member A3
211675_s_at	1.29E-04	7.08E-01	MDFIC	MyoD family inhibitor domain containing
209541_at	1.29E-04	4.96E-01	IGF1	insulin like growth factor 1
202154_x_at	1.29E-04	-4.18E-01	TUBB3	tubulin beta 3 class III
228397_at	1.29E-04	4.50E-01	TUG1	taurine up-regulated 1 (non-protein coding)
218204_s_at	1.30E-04	3.62E-01	FYCO1	FYVE and coiled-coil domain containing 1
238031_at	1.30E-04	-1.61E-01	LOC101928707	uncharacterized LOC101928707
209071_s_at	1.31E-04	6.68E-01	RGS5	regulator of G-protein signaling 5
205422_s_at	1.31E-04	5.76E-01	ITGBL1	integrin subunit beta like 1
207720_at	1.31E-04	-1.22E-01	LOR	loricrin
234141_s_at	1.32E-04	-1.46E-01	LOC286059	tumor necrosis factor receptor superfamily member 10d, decoy with truncated death domain pseudogene
1559280_a_at	1.32E-04	2.79E-01	APELA	apelin receptor early endogenous ligand
1561179_s_at	1.32E-04	-1.31E-01	AMZ1	archaelysin family metallopeptidase 1
212154_at	1.33E-04	4.86E-01	SDC2	syndecan 2
223444_at	1.33E-04	4.12E-01	SENP7	SUMO1/sentrin specific peptidase 7
1553361_x_at	1.34E-04	-1.41E-01	FBXL18	F-box and leucine rich repeat protein 18
210966_x_at	1.34E-04	-3.00E-01	LARP1	La ribonucleoprotein domain family member 1
1554499_s_at	1.34E-04	-1.66E-01	PPA2	pyrophosphatase (inorganic) 2
1556299_s_at	1.34E-04	-1.08E-01	C12orf76	chromosome 12 open reading frame 76
227237_x_at	1.36E-04	-2.76E-01	ATAD3B	ATPase family, AAA domain containing 3B
1568598_at	1.36E-04	-1.98E-01	KAZALD1	Kazal type serine peptidase inhibitor domain 1
204115_at	1.37E-04	4.48E-01	GNG11	G protein subunit gamma 11
48659_at	1.37E-04	-2.28E-01	MIIP	migration and invasion inhibitory protein
210095_s_at	1.37E-04	6.00E-01	IGFBP3	insulin like growth factor binding protein 3
206134_at	1.37E-04	1.07	ADAMDEC1	ADAM like decysin 1
1558801_at	1.37E-04	5.60E-01	NNT-AS1	NNT antisense RNA 1

205273_s_at	1.39E-04	-3.50E-01	PITRM1	pitrilysin metallopeptidase 1
214738_s_at	1.39E-04	-1.52E-01	NEK9	NIMA related kinase 9
220638_s_at	1.41E-04	-5.20E-01	CBLC	Cbl proto-oncogene C
204331_s_at	1.42E-04	-6.04E-01	MRPS12	mitochondrial ribosomal protein S12
225719_s_at	1.42E-04	-2.89E-01	MRPL55	mitochondrial ribosomal protein L55
214969_at	1.43E-04	-1.84E-01	MAP3K9	mitogen-activated protein kinase kinase kinase 9
203500_at	1.43E-04	-3.17E-01	GCDH	glutaryl-CoA dehydrogenase
243464_at	1.43E-04	-1.27E-01		
214991_s_at	1.44E-04	-2.39E-01	PIGO	phosphatidylinositol glycan anchor biosynthesis class O
220258_s_at	1.45E-04	-2.21E-01	WRAP53	WD repeat containing antisense to TP53
205269_at	1.45E-04	5.89E-01	LCP2	lymphocyte cytosolic protein 2
1553808_a_at	1.45E-04	4.11E-01	NKX2-3	NK2 homeobox 3
236862_at	1.46E-04	5.31E-01	GOPC	golgi associated PDZ and coiled-coil motif containing
230257_s_at	1.47E-04	-2.65E-01	TSEN15	tRNA splicing endonuclease subunit 15
235494_at	1.48E-04	2.65E-01	LSAMP	limbic system-associated membrane protein
1560618_at	1.48E-04	-1.42E-01		
231889_at	1.49E-04	-1.08E-01	UBR4	ubiquitin protein ligase E3 component n-recognin 4
227510_x_at	1.51E-04	5.91E-01	MALAT1	metastasis associated lung adenocarcinoma transcript 1 (non-protein coding)
224531_at	1.54E-04	-1.24E-01	GPR61	G protein-coupled receptor 61
231807_at	1.54E-04	4.11E-01	KIAA1217	KIAA1217
227265_at	1.55E-04	8.24E-01	FGL2	fibrinogen like 2
213940_s_at	1.55E-04	3.91E-01	FNBP1	formin binding protein 1
225897_at	1.55E-04	4.65E-01	MARCKS	myristoylated alanine rich protein kinase C substrate
228766_at	1.55E-04	3.23E-01	CD36	CD36 molecule
1558463_s_at	1.55E-04	-1.52E-01	LMF1	lipase maturation factor 1
228508_at	1.56E-04	-1.12E-01	MAML3	mastermind like transcriptional coactivator 3
202712_s_at	1.56E-04	-6.67E-01	CKMT1A///CKMT1B	creatine kinase, mitochondrial 1A///creatine kinase, mitochondrial 1B
205515_at	1.57E-04	-3.32E-01	PRSS12	protease, serine 12
211752_s_at	1.57E-04	-4.88E-01	NDUFS7	NADH:ubiquinone oxidoreductase core subunit S7
1552641_s_at	1.58E-04	-2.96E-01	ATAD3B///ATAD3A	ATPase family, AAA domain containing 3B///ATPase family, AAA domain containing 3A
205686_s_at	1.58E-04	3.70E-01	CD86	CD86 molecule
221489_s_at	1.58E-04	-3.98E-01	SPRY4	sprouty RTK signaling antagonist 4
218644_at	1.59E-04	-4.67E-01	PLEK2	pleckstrin 2
243467_at	1.59E-04	-1.17E-01		
232557_at	1.60E-04	-1.11E-01	UBAP1L	ubiquitin associated protein 1 like
234273_at	1.60E-04	-1.65E-01		
1569434_at	1.61E-04	-1.23E-01	C14orf105	chromosome 14 open reading frame 105
205419_at	1.62E-04	6.66E-01	GPR183	G protein-coupled receptor 183
213002_at	1.63E-04	3.82E-01	MARCKS	myristoylated alanine rich protein kinase C substrate
203931_s_at	1.63E-04	-4.37E-01	MRPL12	mitochondrial ribosomal protein L12
209227_at	1.63E-04	2.23E-01	TUSC3	tumor suppressor candidate 3
228202_at	1.64E-04	2.70E-01		
211795_s_at	1.64E-04	5.85E-01	FYB	FYN binding protein
224607_s_at	1.65E-04	-3.12E-01	SRP68	signal recognition particle 68
230109_at	1.66E-04	2.21E-01	PDE7B	phosphodiesterase 7B
1561394_s_at	1.66E-04	1.87E-01	KIAA1755	KIAA1755
209372_x_at	1.67E-04	-2.78E-01	TUBB2B///TUBB2A	tubulin beta 2B class IIb///tubulin beta 2A class IIa
204642_at	1.67E-04	2.35E-01	S1PR1	sphingosine-1-phosphate receptor 1
1554773_at	1.68E-04	-1.22E-01	WFIKKN2	WAP, follistatin/kazal, immunoglobulin, kunitz and netrin domain containing 2
230836_at	1.68E-04	6.14E-01	ST8SIA4	ST8 alpha-N-acetyl-neuraminide alpha-2,8-sialyltransferase 4
235044_at	1.68E-04	3.64E-01	CYYR1	cysteine and tyrosine rich 1
228297_at	1.69E-04	5.40E-01	CNN3	calponin 3
238592_at	1.70E-04	4.02E-01		
201167_x_at	1.70E-04	-2.35E-01	ARHGDIA	Rho GDP dissociation inhibitor alpha
1552914_a_at	1.70E-04	-1.62E-01	CD276	CD276 molecule
221698_s_at	1.72E-04	4.32E-01	CLEC7A	C-type lectin domain family 7 member A
205648_at	1.73E-04	4.38E-01	WNT2	Wnt family member 2
1555548_at	1.73E-04	-1.18E-01	NDUFA10	NADH:ubiquinone oxidoreductase subunit A10
229130_at	1.73E-04	5.05E-01		
222611_s_at	1.73E-04	-1.85E-01	PSPC1	paraspeckle component 1
214448_x_at	1.74E-04	-2.17E-01	NFKBIB	NFKB inhibitor beta
227858_at	1.74E-04	-1.72E-01	PCNX3	pecanex homolog 3 (Drosophila)
201743_at	1.75E-04	5.35E-01	CD14	CD14 molecule
209347_s_at	1.75E-04	-1.07E-01	MAF	MAF bZIP transcription factor
240345_x_at	1.76E-04	-1.27E-01		
212288_at	1.77E-04	4.62E-01	FNBP1	formin binding protein 1
214916_x_at	1.77E-04	3.16E-01	IGHV3-23///IGHV4-31///IGHM///IGHG3///IGHG1///IGHA2///IGHA1	immunoglobulin heavy variable 3-23///immunoglobulin heavy variable 4-31///immunoglobulin heavy constant mu///immunoglobulin heavy constant gamma 3 (G3m marker)///immunoglobulin heavy constant gamma 1 (G1m marker)///immunoglobulin heavy constant alpha 2 (A2m marker)///immunoglobulin heavy constant alpha 1
202920_at	1.77E-04	3.39E-01	ANK2	ankyrin 2, neuronal
236789_at	1.77E-04	-8.78E-02	MAPT-AS1	MAPT antisense RNA 1
233550_s_at	1.77E-04	-1.82E-01	SLC4A11	solute carrier family 4 member 11
216383_at	1.78E-04	-2.88E-01		
204178_s_at	1.78E-04	-3.07E-01	RBM14	RNA binding motif protein 14

206760_s_at	1.79E-04	-1.38E-01	FCER2	Fc fragment of IgE receptor II
204267_x_at	1.79E-04	-2.20E-01	PKMYT1	protein kinase, membrane associated tyrosine/threonine 1
1555878_at	1.79E-04	-4.22E-01	RPS24	ribosomal protein S24
218664_at	1.79E-04	-2.68E-01	MECR	mitochondrial trans-2-enoyl-CoA reductase
228532_at	1.81E-04	3.67E-01	C1orf162	chromosome 1 open reading frame 162
212991_at	1.81E-04	2.41E-01	FBXO9	F-box protein 9
232568_at	1.81E-04	3.41E-01	MGC24103	uncharacterized MGC24103
210594_x_at	1.81E-04	-2.84E-01	MPZL1	myelin protein zero like 1
236437_at	1.82E-04	4.62E-01		
216602_s_at	1.83E-04	-4.97E-01	FARSA	phenylalanyl-tRNA synthetase alpha subunit
212193_s_at	1.84E-04	-4.41E-01	LARP1	La ribonucleoprotein domain family member 1
205934_at	1.84E-04	3.61E-01	PLCL1	phospholipase C like 1
1559914_at	1.84E-04	-9.85E-02		
200664_s_at	1.85E-04	-5.67E-01	DNAJB1	DnaJ heat shock protein family (Hsp40) member B1
213156_at	1.85E-04	5.87E-01	ZBTB20	zinc finger and BTB domain containing 20
226885_at	1.86E-04	1.3	RNF217	ring finger protein 217
1555360_a_at	1.87E-04	-2.38E-01	DNAJC11	DnaJ heat shock protein family (Hsp40) member C11
44563_at	1.87E-04	-2.55E-01	WRAP53	WD repeat containing antisense to TP53
218603_at	1.87E-04	3.37E-01	HECA	hdc homolog, cell cycle regulator
201231_s_at	1.88E-04	-4.60E-01	ENO1	enolase 1
1562675_at	1.88E-04	-1.10E-01	FAAP20	Fanconi anemia core complex associated protein 20
225118_at	1.88E-04	-2.26E-01	KMT5A	lysine methyltransferase 5A
221645_s_at	1.89E-04	7.13E-01	ZNF83	zinc finger protein 83
223003_at	1.89E-04	-3.74E-01	C19orf43	chromosome 19 open reading frame 43
1569520_at	1.90E-04	-1.75E-01	LOC101929747	uncharacterized LOC101929747
202237_at	1.91E-04	6.45E-01	LOC101928916///NNMT	uncharacterized LOC101928916///nicotinamide N-methyltransferase
235458_at	1.91E-04	3.72E-01	HAVCR2	hepatitis A virus cellular receptor 2
219228_at	1.91E-04	7.66E-01	ZNF331	zinc finger protein 331
203887_s_at	1.91E-04	4.68E-01	THBD	thrombomodulin
214302_x_at	1.91E-04	-1.44E-01	GJC2	gap junction protein gamma 2
1555751_a_at	1.92E-04	-2.84E-01	GEMIN7	gem nuclear organelle associated protein 7
238816_at	1.92E-04	-1.52E-01	PSEN1	presenilin 1
219358_s_at	1.92E-04	2.49E-01	ADAP2	ArfGAP with dual PH domains 2
201497_x_at	1.92E-04	3.90E-01	MYH11	myosin heavy chain 11
216641_s_at	1.93E-04	-2.83E-01	LAD1	ladinin 1
206596_s_at	1.93E-04	-1.23E-01	NRL	neural retina leucine zipper
1555734_x_at	1.93E-04	-2.24E-01	AP1S3	adaptor related protein complex 1 sigma 3 subunit
1554985_at	1.94E-04	1.69E-01	ZNF396	zinc finger protein 396
216299_s_at	1.94E-04	-2.17E-01	XRCC3	X-ray repair cross complementing 3
218702_at	1.95E-04	-3.45E-01	SARS2	seryl-tRNA synthetase 2, mitochondrial
212038_s_at	1.96E-04	-5.08E-01	VDAC1	voltage dependent anion channel 1
235432_at	1.97E-04	4.15E-01	NPHP3	nephronophthisis 3 (adolescent)
1559946_s_at	1.97E-04	-3.87E-01	RUVBL2	RuvB like AAA ATPase 2
210620_s_at	1.98E-04	-1.86E-01	GTF3C2	general transcription factor IIIC subunit 2
221700_s_at	1.98E-04	-2.35E-01	UBA52	ubiquitin A-52 residue ribosomal protein fusion product 1
1556479_at	1.98E-04	-1.37E-01		
1561136_at	1.98E-04	-7.67E-02	GYPE	glycophorin E (MNS blood group)
201377_at	1.99E-04	-3.48E-01	UBAP2L	ubiquitin associated protein 2 like
244824_at	1.99E-04	-1.43E-01	LINC01563	long intergenic non-protein coding RNA 1563
208074_s_at	1.99E-04	-4.19E-01	AP2S1	adaptor related protein complex 2 sigma 1 subunit
227212_s_at	2.00E-04	-4.14E-01	PHF19	PHD finger protein 19
1557233_at	2.01E-04	-1.04E-01	F11-AS1	F11 antisense RNA 1
215099_s_at	2.01E-04	-1.38E-01	RXRB	retinoid X receptor beta
231481_at	2.02E-04	-1.53E-01	CCNB3	cyclin B3
205249_at	2.03E-04	4.79E-01	EGR2	early growth response 2
31861_at	2.03E-04	-1.34E-01	IGHMBP2	immunoglobulin mu binding protein 2
203091_at	2.03E-04	-2.09E-01	FUBP1	far upstream element binding protein 1
241003_at	2.03E-04	-1.16E-01		
226066_at	2.03E-04	5.47E-01	MITF	melanogenesis associated transcription factor
211161_s_at	2.04E-04	4.94E-01	COL3A1	collagen type III alpha 1 chain
230214_at	2.07E-04	3.37E-01	MRVI1	murine retrovirus integration site 1 homolog
204495_s_at	2.09E-04	-2.95E-01	C15orf39	chromosome 15 open reading frame 39
203106_s_at	2.10E-04	2.94E-01	VPS41	VPS41, HOPS complex subunit
240962_at	2.10E-04	-1.55E-01		
233206_at	2.12E-04	-1.46E-01	SLC22A23	solute carrier family 22 member 23
214346_at	2.13E-04	-9.23E-02	C1QL1	complement C1q like 1
210022_at	2.13E-04	-1.86E-01	PCGF1	polycomb group ring finger 1
204989_s_at	2.14E-04	-2.35E-01	ITGB4	integrin subunit beta 4
215513_at	2.15E-04	5.07E-01	HYMAI	hydatidiform mole associated and imprinted (non-protein coding)
1559352_a_at	2.16E-04	-1.60E-01	MAFG-AS1	MAFG antisense RNA 1 (head to head)
200076_s_at	2.17E-04	-1.65E-01	KXD1	KxDL motif containing 1
210895_s_at	2.17E-04	4.67E-01	CD86	CD86 molecule
201903_at	2.18E-04	-5.01E-01	UQCRC1	ubiquinol-cytochrome c reductase core protein I
232854_at	2.18E-04	-1.21E-01	DIRAS1	DIRAS family GTPase 1
207196_s_at	2.18E-04	-3.17E-01	TNIP1	TNFAIP3 interacting protein 1
215792_s_at	2.19E-04	-2.99E-01	DNAJC11	DnaJ heat shock protein family (Hsp40) member C11

1564937_at	2.19E-04	-9.13E-02		
228974_at	2.20E-04	6.12E-01	ZNF677	zinc finger protein 677
233250_x_at	2.20E-04	-1.31E-01	FOXRED2	FAD dependent oxidoreductase domain containing 2
218305_at	2.20E-04	-3.98E-01	IPO4	importin 4
204135_at	2.21E-04	5.11E-01	FILIP1L	filamin A interacting protein 1 like
214625_s_at	2.23E-04	-1.27E-01	MINK1	misshapen like kinase 1
223921_s_at	2.24E-04	-1.90E-01	GBA2	glucosylceramidase beta 2
218880_at	2.24E-04	-5.07E-01	FOSL2	FOS like 2, AP-1 transcription factor subunit
238668_at	2.25E-04	5.84E-01	NCKAP1L	NCK associated protein 1 like
1560579_s_at	2.26E-04	-4.30E-01	SLC16A1-AS1	SLC16A1 antisense RNA 1
234436_x_at	2.27E-04	-1.42E-01	OBP2A	odorant binding protein 2A
229830_at	2.27E-04	7.10E-01		
206488_s_at	2.27E-04	4.16E-01	CD36	CD36 molecule
243529_at	2.28E-04	-3.66E-01	MARS2	methionyl-tRNA synthetase 2, mitochondrial
202283_at	2.28E-04	5.84E-01	SERPINF1	serpin family F member 1
221250_s_at	2.28E-04	-1.47E-01	MXD3	MAX dimerization protein 3
217099_s_at	2.28E-04	-3.83E-01	GEMIN4	gem nuclear organelle associated protein 4
235914_at	2.29E-04	-2.13E-01	SYNPO	synaptopodin
222060_at	2.30E-04	-3.19E-01	KRT8P12	keratin 8 pseudogene 12
214507_s_at	2.30E-04	-2.96E-01	EXOSC2	exosome component 2
217824_at	2.32E-04	3.51E-01	UBE2J1	ubiquitin conjugating enzyme E2 J1
207002_s_at	2.32E-04	8.32E-01	PLAGL1	PLAG1 like zinc finger 1
201227_s_at	2.32E-04	-2.38E-01	NDUFB8	NADH:ubiquinone oxidoreductase subunit B8
1555395_at	2.32E-04	-1.11E-01	AKAP12	A-kinase anchoring protein 12
201770_at	2.34E-04	-3.97E-01	SNRPA	small nuclear ribonucleoprotein polypeptide A
218044_x_at	2.35E-04	-1.58E-01	PTMS	parathymosin
242545_at	2.36E-04	-1.60E-01	TTLL11	tubulin tyrosine ligase like 11
1554038_at	2.36E-04	-1.85E-01	LARP1B	La ribonucleoprotein domain family member 1B
231523_at	2.36E-04	-9.16E-02	FGF14	fibroblast growth factor 14
206530_at	2.37E-04	-1.25E-01	RAB30	RAB30, member RAS oncogene family
222691_at	2.38E-04	3.28E-01	SLC35B3	solute carrier family 35 member B3
1555522_s_at	2.39E-04	-3.07E-01	DPY30///MEMO1	dpy-30, histone methyltransferase complex regulatory subunit///mediator of cell motility 1
202024_at	2.40E-04	-3.69E-01	ASNA1	arsA arsenite transporter, ATP-binding, homolog 1 (bacterial)
1554589_at	2.40E-04	-1.28E-01	LENG9	leukocyte receptor cluster member 9
233607_at	2.41E-04	2.61E-01		
223213_s_at	2.42E-04	4.27E-01	ZHX1	zinc fingers and homeoboxes 1
212192_at	2.43E-04	9.53E-01	KCTD12	potassium channel tetramerization domain containing 12
223419_at	2.43E-04	-2.57E-01	FBXW9	F-box and WD repeat domain containing 9
233140_s_at	2.44E-04	-1.82E-01	VIPAS39	VPS33B interacting protein, apical-basolateral polarity regulator, spe-39 homolog
227661_at	2.44E-04	-1.45E-01		
227971_at	2.45E-04	1.94E-01	NRK	Nik related kinase
218115_at	2.45E-04	-3.35E-01	ASF1B	anti-silencing function 1B histone chaperone
208799_at	2.45E-04	-3.75E-01	PSMB5	proteasome subunit beta 5
202836_s_at	2.46E-04	-3.53E-01	TXNL4A	thioredoxin like 4A
37226_at	2.46E-04	-2.82E-01	BNIP1	BCL2 interacting protein 1
1552807_a_at	2.47E-04	3.16E-01	SIGLEC10	sialic acid binding Ig like lectin 10
218870_at	2.47E-04	4.34E-01	LOC101928361///ARHGAP15	uncharacterized LOC101928361///Rho GTPase activating protein 15
208793_x_at	2.47E-04	-2.88E-01	SMARCA4	SWI/SNF related, matrix associated, actin dependent regulator of chromatin, subfamily a, member 4
242277_at	2.48E-04	6.54E-01		
235944_at	2.49E-04	5.76E-01	HMCN1	hemicentin 1
217077_s_at	2.50E-04	-1.37E-01	GABBR2	gamma-aminobutyric acid type B receptor subunit 2
205459_s_at	2.50E-04	-2.31E-01	NPAS2	neuronal PAS domain protein 2
203557_s_at	2.51E-04	-3.38E-01	PCBD1	pterin-4 alpha-carbinolamine dehydratase 1
1560888_x_at	2.51E-04	-1.00E-01		
212188_at	2.51E-04	8.88E-01	KCTD12	potassium channel tetramerization domain containing 12
206836_at	2.51E-04	-1.12E-01	SLC6A3	solute carrier family 6 member 3
1567443_x_at	2.51E-04	-1.06E-01	PSEN1	presenilin 1
214484_s_at	2.52E-04	-3.95E-01	SIGMAR1	sigma non-opioid intracellular receptor 1
204137_at	2.52E-04	4.99E-01	GPR137B	G protein-coupled receptor 137B
219762_s_at	2.52E-04	-4.63E-01	RPL36	ribosomal protein L36
230372_at	2.52E-04	6.70E-01	HAS2	hyaluronan synthase 2
205098_at	2.53E-04	4.46E-01	CCR1	C-C motif chemokine receptor 1
219134_at	2.54E-04	5.32E-01	ADGRL4	adhesion G protein-coupled receptor L4
1552922_at	2.56E-04	-1.01E-01	RIMS1	regulating synaptic membrane exocytosis 1
207831_x_at	2.56E-04	-3.63E-01	DHPS	deoxyhypusine synthase
229159_at	2.57E-04	1.10E-01	THSD7A	thrombospondin type 1 domain containing 7A
212626_x_at	2.59E-04	-2.60E-01	HNRNPC	heterogeneous nuclear ribonucleoprotein C (C1/C2)
202826_at	2.60E-04	-3.74E-01	SPINT1	serine peptidase inhibitor, Kunitz type 1
221777_at	2.60E-04	-1.91E-01	RITA1	RBPJ interacting and tubulin associated 1
201459_at	2.61E-04	-3.99E-01	RUVBL2	RuvB like AAA ATPase 2
234954_at	2.61E-04	-3.97E-01		
1553874_a_at	2.62E-04	-1.14E-01	ZSCAN10	zinc finger and SCAN domain containing 10
1553523_at	2.62E-04	-1.13E-01	NLRP14	NLR family pyrin domain containing 14

230135_at	2.63E-04	6.32E-01	HHIP	hedgehog interacting protein
210197_at	2.63E-04	-1.28E-01	ITPK1	inositol-tetrakisphosphate 1-kinase
206228_at	2.64E-04	-1.24E-01	PAX2	paired box 2
1553045_at	2.65E-04	-8.83E-02	WNT9A	Wnt family member 9A
215955_x_at	2.65E-04	-1.74E-01	ARHGAP26	Rho GTPase activating protein 26
1556314_a_at	2.66E-04	2.93E-01	ZNF366	zinc finger protein 366
241246_at	2.66E-04	-1.46E-01		
224864_at	2.66E-04	-3.31E-01	SRA1	steroid receptor RNA activator 1
216971_s_at	2.66E-04	-1.69E-01	PLEC	plectin
1554126_at	2.67E-04	-1.35E-01	MSRB3	methionine sulfoxide reductase B3
242693_at	2.67E-04	5.66E-01		
203194_s_at	2.68E-04	-3.84E-01	NUP98	nucleoporin 98
231411_at	2.69E-04	2.37E-01	LHFP	lipoma HMGIC fusion partner
234728_s_at	2.70E-04	-1.87E-01	DHX35	DEAH-box helicase 35
201222_s_at	2.71E-04	-2.50E-01	RAD23B	RAD23 homolog B, nucleotide excision repair protein
220330_s_at	2.71E-04	6.95E-01	SAMSN1	SAM domain, SH3 domain and nuclear localization signals 1
241887_at	2.71E-04	4.56E-01	UBE2W	ubiquitin conjugating enzyme E2 W (putative)
212950_at	2.71E-04	5.22E-01	ADGRF5	adhesion G protein-coupled receptor F5
213476_x_at	2.73E-04	-3.97E-01	TUBB3	tubulin beta 3 class III
234575_at	2.74E-04	-1.19E-01	ZNF71	zinc finger protein 71
227351_at	2.74E-04	3.59E-01	LOC101930115///C16orf52///VWA3A	von Willebrand factor A domain-containing protein 3A///chromosome 16 open reading frame 52///von Willebrand factor A domain containing 3A
223487_x_at	2.75E-04	1.17E-01	GNB4	G protein subunit beta 4
206028_s_at	2.75E-04	5.87E-01	MERTK	MER proto-oncogene, tyrosine kinase
240680_at	2.75E-04	-1.30E-01		
226317_at	2.75E-04	3.12E-01	PPP4R2	protein phosphatase 4 regulatory subunit 2
209897_s_at	2.76E-04	3.19E-01	SLIT2	slit guidance ligand 2
1552690_a_at	2.77E-04	-1.89E-01	CACNA2D4	calcium voltage-gated channel auxiliary subunit alpha2delta 4
238043_at	2.77E-04	4.06E-01	ARID1B	AT-rich interaction domain 1B
206468_s_at	2.77E-04	-2.59E-01	METTL13	methyltransferase like 13
219203_at	2.78E-04	-3.73E-01	EMC9	ER membrane protein complex subunit 9
241473_at	2.78E-04	2.66E-01		
205634_x_at	2.79E-04	-3.43E-01	ZDHHC24	zinc finger DHHC-type containing 24
230741_at	2.80E-04	4.47E-01	P2RX7	purinergic receptor P2X 7
236029_at	2.80E-04	2.98E-01	FAT3	FAT atypical cadherin 3
201525_at	2.80E-04	8.03E-01	APOD	apolipoprotein D
209777_s_at	2.80E-04	-2.14E-01	SLC19A1	solute carrier family 19 member 1
206469_x_at	2.81E-04	-4.41E-01	AKR7A3	aldo-keto reductase family 7 member A3
244252_at	2.81E-04	-1.13E-01	LOC399884	uncharacterized LOC399884
225390_s_at	2.81E-04	-3.92E-01	KLF13	Kruppel like factor 13
206750_at	2.83E-04	-1.50E-01	MAFK	MAF bZIP transcription factor K
215834_x_at	2.85E-04	-1.55E-01	SCARB1	scavenger receptor class B member 1
1556059_s_at	2.86E-04	-2.76E-01	SPEN	spen family transcriptional repressor
224559_at	2.87E-04	5.75E-01	MALAT1	metastasis associated lung adenocarcinoma transcript 1 (non-protein coding)
231302_at	2.92E-04	2.24E-01		
219895_at	2.92E-04	3.04E-01	TMEM255A	transmembrane protein 255A
218261_at	2.93E-04	-3.67E-01	AP1M2	adaptor related protein complex 1 mu 2 subunit
1553837_at	2.93E-04	-1.21E-01	PGAM5	PGAM family member 5, mitochondrial serine/threonine protein phosphatase
219111_s_at	2.94E-04	-2.71E-01	DDX54	DEAD-box helicase 54
241434_at	2.95E-04	3.35E-01	IPP	intracisternal A particle-promoted polypeptide
202120_x_at	2.97E-04	-3.49E-01	AP2S1	adaptor related protein complex 2 sigma 1 subunit
230389_at	2.99E-04	4.84E-01	FNBP1	formin binding protein 1
235759_at	2.99E-04	3.06E-01		
214255_at	3.02E-04	2.09E-01	ATP10A	ATPase phospholipid transporting 10A (putative)
202995_s_at	3.03E-04	3.72E-01	FBLN1	fibulin 1
210527_x_at	3.04E-04	-1.86E-01	TUBA3D///TUBA3C	tubulin alpha 3d///tubulin alpha 3c
203299_s_at	3.04E-04	3.78E-01	AP1S2	adaptor related protein complex 1 sigma 2 subunit
242490_at	3.04E-04	3.39E-01		
226865_at	3.04E-04	5.77E-01	PLXDC2	plexin domain containing 2
1555634_a_at	3.06E-04	-1.06E-01	LILRA5	leukocyte immunoglobulin like receptor A5
225151_at	3.06E-04	-1.15E-01	RTKN	rhotekin
205895_s_at	3.07E-04	-4.43E-01	NOLC1	nucleolar and coiled-body phosphoprotein 1
214361_s_at	3.10E-04	-1.43E-01	RGS12	regulator of G-protein signaling 12
1554857_at	3.11E-04	-1.25E-01	ELMO2	engulfment and cell motility 2
1556996_at	3.11E-04	-9.86E-02	LEPROT	leptin receptor overlapping transcript
201246_s_at	3.11E-04	-2.62E-01	LOC101927673///OTUB1	uncharacterized LOC101927673///OTU deubiquitinase, ubiquitin aldehyde binding 1
228832_at	3.12E-04	-2.27E-01	FLJ20021	uncharacterized LOC90024
204400_at	3.13E-04	1.80E-01	EFS	embryonal Fyn-associated substrate
220587_s_at	3.13E-04	-3.38E-01	MLST8	MTOR associated protein, LST8 homolog
213514_s_at	3.13E-04	-1.90E-01	DIAPH1	diaphanous related formin 1
213933_at	3.13E-04	4.16E-01	PTGER3	prostaglandin E receptor 3
210501_x_at	3.14E-04	-3.17E-01	EIF3K	eukaryotic translation initiation factor 3 subunit K
222814_s_at	3.15E-04	-1.08E-01	ZNHIT2	zinc finger HIT-type containing 2
204834_at	3.15E-04	7.16E-01	FGL2	fibrinogen like 2
220597_s_at	3.16E-04	-3.08E-01	ARL6IP4	ADP ribosylation factor like GTPase 6 interacting protein 4
1552553_a_at	3.16E-04	2.07E-01	NLRC4	NLR family CARD domain containing 4

202707_at	3.17E-04	-1.43E-01	UMPS	uridine monophosphate synthetase
236077_at	3.19E-04	-4.02E-01	GANC	glucosidase alpha, neutral C
219064_at	3.21E-04	5.16E-01	ITIH5	inter-alpha-trypsin inhibitor heavy chain family member 5
207525_s_at	3.22E-04	-2.29E-01	GIPC1	GIPC PDZ domain containing family member 1
204051_s_at	3.22E-04	7.88E-01	SFRP4	secreted frizzled related protein 4
201037_at	3.22E-04	-6.07E-01	PFKP	phosphofructokinase, platelet
219671_at	3.22E-04	-9.60E-02	HPCAL4	hippocalcin like 4
227378_x_at	3.23E-04	-3.50E-01	METTL26	methyltransferase like 26
213986_s_at	3.24E-04	-2.75E-01	TMEM259	transmembrane protein 259
231170_at	3.24E-04	2.60E-01		
232401_at	3.24E-04	-1.27E-01	KCNS2	potassium voltage-gated channel modifier subfamily S member 2
236685_at	3.25E-04	3.08E-01		
1562695_at	3.25E-04	-1.26E-01	FOXN4	forkhead box N4
217860_at	3.25E-04	-3.89E-01	NDUFA10	NADH:ubiquinone oxidoreductase subunit A10
219878_s_at	3.25E-04	-3.39E-01	KLF13	Kruppel like factor 13
211171_s_at	3.26E-04	-1.75E-01	PDE10A	phosphodiesterase 10A
1564521_x_at	3.27E-04	-2.71E-01	PRMT5	protein arginine methyltransferase 5
212376_s_at	3.27E-04	-2.06E-01	EP400	E1A binding protein p400
228080_at	3.27E-04	3.34E-01	LAYN	layilin
238512_at	3.28E-04	4.89E-01	WNT2B	Wnt family member 2B
1558530_at	3.28E-04	-1.41E-01	LRTM2	leucine rich repeats and transmembrane domains 2
225976_at	3.29E-04	3.01E-01	BTF3L4	basic transcription factor 3 like 4
204072_s_at	3.30E-04	6.70E-01	FRY	FRY microtubule binding protein
241468_at	3.31E-04	-1.48E-01		
237054_at	3.32E-04	6.62E-01	ENPP5	ectonucleotide pyrophosphatase/phosphodiesterase 5 (putative)
223745_at	3.32E-04	-1.19E-01	C16orf95	chromosome 16 open reading frame 95
201135_at	3.33E-04	-3.62E-01	ECHS1	enoyl-CoA hydratase, short chain 1
216897_s_at	3.37E-04	-1.56E-01	FAM76A	family with sequence similarity 76 member A
1570308_at	3.37E-04	-8.06E-02		
1553237_x_at	3.37E-04	-9.60E-02	PCDHAC1	protocadherin alpha subfamily C, 1
201584_s_at	3.38E-04	-3.73E-01	DDX39A	DEAD-box helicase 39A
212483_at	3.38E-04	3.40E-01	NIPBL	NIPBL, cohesin loading factor
217602_at	3.39E-04	5.09E-01	LOC101060363///PPIA	peptidyl-prolyl cis-trans isomerase A pseudogene///peptidylprolyl isomerase A
227560_at	3.39E-04	-3.36E-01	SFXN2	sideroflexin 2
239454_at	3.40E-04	-1.29E-01	SCARF2	scavenger receptor class F member 2
1557714_at	3.40E-04	-1.34E-01	CTBP1	C-terminal binding protein 1
238363_at	3.41E-04	2.23E-01		
234922_s_at	3.42E-04	-9.94E-02	NHSL1	NHS like 1
204397_at	3.45E-04	-1.25E-01	EML2	echinoderm microtubule associated protein like 2
1563101_at	3.45E-04	-7.87E-02	ARNTL2-AS1	ARNTL2 antisense RNA 1
234526_at	3.46E-04	-1.24E-01	OR51B6	olfactory receptor family 51 subfamily B member 6
231098_at	3.47E-04	2.31E-01		
214387_x_at	3.48E-04	-1.20E-01	SFTPC	surfactant protein C
203379_at	3.48E-04	-3.60E-01	RPS6KA1	ribosomal protein S6 kinase A1
223043_at	3.50E-04	-3.89E-01	EMC4	ER membrane protein complex subunit 4
205304_s_at	3.51E-04	2.89E-01	KCNJ8	potassium voltage-gated channel subfamily J member 8
208451_s_at	3.53E-04	4.62E-01	C4B_2///C4B///C4A	complement component 4B (Chido blood group), copy 2///complement component 4B (Chido blood group)///complement component 4A (Rodgers blood group)
203112_s_at	3.54E-04	-2.35E-01	MIR943///NELFA	microRNA 943///negative elongation factor complex member A
224465_s_at	3.56E-04	-2.91E-01	PYM1	PYM homolog 1, exon junction complex associated factor
202019_s_at	3.56E-04	-2.30E-01	LANCL1	LanC like 1
227980_at	3.56E-04	3.46E-01	ZNF322	zinc finger protein 322
214674_at	3.57E-04	-1.52E-01	USP19	ubiquitin specific peptidase 19
205430_at	3.57E-04	5.66E-01	BMP5	bone morphogenetic protein 5
214292_at	3.59E-04	-1.52E-01	ITGB4	integrin subunit beta 4
1564473_at	3.60E-04	-9.54E-02	ESCO2	establishment of sister chromatid cohesion N-acetyltransferase 2
216890_at	3.60E-04	-1.20E-01		
213490_s_at	3.60E-04	-2.28E-01	MAP2K2	mitogen-activated protein kinase kinase 2
200990_at	3.61E-04	-4.24E-01	TRIM28	tripartite motif containing 28
233743_x_at	3.61E-04	-1.15E-01	S1PR5	sphingosine-1-phosphate receptor 5
224267_x_at	3.61E-04	-1.23E-01	ZAN	zonadhesin (gene/pseudogene)
204271_s_at	3.63E-04	5.08E-01	EDNRB	endothelin receptor type B
232208_at	3.63E-04	-1.32E-01	ISLR2	immunoglobulin superfamily containing leucine rich repeat 2
207933_at	3.64E-04	-1.63E-01	ZP2	zona pellucida glycoprotein 2
234996_at	3.64E-04	3.44E-01	CALCRL	calcitonin receptor like receptor
201137_s_at	3.64E-04	6.15E-01	HLA-DPB1	major histocompatibility complex, class II, DP beta 1
233334_x_at	3.64E-04	-3.21E-01	SLX1B-SULT1A4///SLX1A-SULT1A3///SLX1A///SULT1A4///SLX1B///SULT1A3	SLX1B-SULT1A4 readthrough (NMD candidate)///SLX1A-SULT1A3 readthrough (NMD candidate)///SLX1 homolog A, structure-specific endonuclease subunit///sulfotransferase family 1A member 4///SLX1 homolog B, structure-specific endonuclease subunit///sulfotransferase family 1A member 3
1559800_a_at	3.65E-04	-1.11E-01		
211570_s_at	3.66E-04	-1.22E-01	RAPSN	receptor associated protein of the synapse
202878_s_at	3.66E-04	4.70E-01	CD93	CD93 molecule
234584_s_at	3.66E-04	-2.02E-01	ATE1	arginyltransferase 1

1563404_at	3.66E-04	-1.21E-01		
219122_s_at	3.67E-04	-3.71E-01	THG1L	tRNA-histidine guanylyltransferase 1 like
203698_s_at	3.67E-04	6.63E-01	FRZB	frizzled-related protein
223736_at	3.68E-04	-1.15E-01	IFT81	intraflagellar transport 81
203287_at	3.69E-04	-3.22E-01	LAD1	ladinin 1
201322_at	3.71E-04	-3.44E-01	ATP5B	ATP synthase, H+ transporting, mitochondrial F1 complex, beta polypeptide
209318_x_at	3.72E-04	8.34E-01	PLAGL1	PLAG1 like zinc finger 1
224338_s_at	3.72E-04	-1.33E-01	RNF26	ring finger protein 26
213606_s_at	3.73E-04	-3.03E-01	ARHGDIA	Rho GDP dissociation inhibitor alpha
229050_s_at	3.73E-04	-3.75E-01	SNORA17B///SNORA17A///SNHG7	small nucleolar RNA, H/ACA box 17B///small nucleolar RNA, H/ACA box 17A///small nucleolar RNA host gene 7
203424_s_at	3.74E-04	3.90E-01	IGFBP5	insulin like growth factor binding protein 5
210649_s_at	3.75E-04	-3.68E-01	ARID1A	AT-rich interaction domain 1A
232707_at	3.76E-04	8.79E-01	ISX	intestine specific homeobox
227103_s_at	3.77E-04	-3.85E-01	ECE2	endothelin converting enzyme 2
202067_s_at	3.77E-04	-3.84E-01	LDLR	low density lipoprotein receptor
242100_at	3.78E-04	4.00E-01	CHSY3	chondroitin sulfate synthase 3
234338_s_at	3.78E-04	-1.36E-01	ZBTB47	zinc finger and BTB domain containing 47
207238_s_at	3.78E-04	5.56E-01	PTPRC	protein tyrosine phosphatase, receptor type C
222724_at	3.78E-04	-1.26E-01	VWA1	von Willebrand factor A domain containing 1
218220_at	3.80E-04	-4.34E-01	C12orf10	chromosome 12 open reading frame 10
1557080_s_at	3.81E-04	3.42E-01	ITGBL1	integrin subunit beta like 1
241892_at	3.82E-04	-1.13E-01	NCL	nucleolin
203300_x_at	3.83E-04	3.72E-01	AP1S2	adaptor related protein complex 1 sigma 2 subunit
203982_s_at	3.84E-04	-1.44E-01	ABCD4	ATP binding cassette subfamily D member 4
225571_at	3.84E-04	4.68E-01	LIFR	leukemia inhibitory factor receptor alpha
206853_s_at	3.85E-04	3.91E-01	MAP3K7	mitogen-activated protein kinase kinase kinase 7
1554554_at	3.85E-04	-1.07E-01	CCDC57	coiled-coil domain containing 57
229686_at	3.85E-04	2.09E-01	P2RY8	purinergic receptor P2Y8
218807_at	3.85E-04	8.01E-01	VAV3	vav guanine nucleotide exchange factor 3
216552_x_at	3.85E-04	-1.14E-01	KIR2DS4	killer cell immunoglobulin like receptor, two Ig domains and short cytoplasmic tail 4
216164_at	3.86E-04	-1.58E-01	LRRN2	leucine rich repeat neuronal 2
214468_at	3.86E-04	-9.59E-02	MYH6	myosin heavy chain 6
224241_s_at	3.87E-04	-1.21E-01		
234829_at	3.88E-04	-1.93E-01	DUSP15	dual specificity phosphatase 15
202586_at	3.88E-04	-2.47E-01	POLR2L	RNA polymerase II subunit L
208931_s_at	3.89E-04	-4.15E-01	ILF3	interleukin enhancer binding factor 3
212347_x_at	3.89E-04	-3.13E-01	MIR4800///MXD4	microRNA 4800///MAX dimerization protein 4
201401_s_at	3.89E-04	-1.61E-01	GRK2	G protein-coupled receptor kinase 2
226137_at	3.90E-04	5.60E-01	ZFHX3	zinc finger homeobox 3
236395_at	3.91E-04	2.42E-01		
204898_at	3.92E-04	-1.35E-01	SAP30	Sin3A associated protein 30
206978_at	3.95E-04	4.89E-01	CCR2	C-C motif chemokine receptor 2
212914_at	3.95E-04	2.80E-01	CBX7	chromobox 7
209960_at	3.96E-04	5.99E-01	HGF	hepatocyte growth factor
204931_at	3.97E-04	2.81E-01	TCF21	transcription factor 21
202102_s_at	3.98E-04	-2.83E-01	BRD4	bromodomain containing 4
219815_at	3.99E-04	2.59E-01	GAL3ST4	galactose-3-O-sulfotransferase 4
238609_at	3.99E-04	4.05E-01	FAM200A	family with sequence similarity 200 member A
207748_at	4.00E-04	-1.33E-01		
207829_s_at	4.01E-04	-2.43E-01	BNIP1	BCL2 interacting protein 1
203682_s_at	4.01E-04	-4.11E-01	IVD	isovaleryl-CoA dehydrogenase
214443_at	4.05E-04	-1.63E-01	PVR	poliovirus receptor
235934_at	4.08E-04	-1.06E-01	NAPA-AS1	NAPA antisense RNA 1
221940_at	4.09E-04	-2.87E-01	RPUSD2	RNA pseudouridylate synthase domain containing 2
208909_at	4.09E-04	-2.99E-01	UQCRFS1	ubiquinol-cytochrome c reductase, Rieske iron-sulfur polypeptide 1
200001_at	4.09E-04	-4.38E-01	CAPNS1	calpain small subunit 1
223378_at	4.13E-04	3.45E-01	GLIS2	GLIS family zinc finger 2
229459_at	4.13E-04	2.07E-01	FAM19A5	family with sequence similarity 19 member A5, C-C motif chemokine like
221269_s_at	4.13E-04	-4.71E-01	SH3BGRL3	SH3 domain binding glutamate rich protein like 3
210946_at	4.14E-04	4.15E-01	PLPP1	phospholipid phosphatase 1
224860_at	4.15E-04	4.50E-01	TMEM261	transmembrane protein 261
231727_s_at	4.16E-04	-3.52E-01	MIF4GD	MIF4G domain containing
231227_at	4.16E-04	6.46E-01		
1566218_at	4.17E-04	-1.32E-01	KRTAP5-2	keratin associated protein 5-2
228147_at	4.19E-04	3.98E-01	SEPT7P2	septin 7 pseudogene 2
222364_at	4.19E-04	2.80E-01	SLC44A1	solute carrier family 44 member 1
210515_at	4.19E-04	-1.86E-01	HNF1A	HNF1 homeobox A
202839_s_at	4.21E-04	-3.07E-01	NDUFB7	NADH:ubiquinone oxidoreductase subunit B7
221898_at	4.24E-04	4.45E-01	PDPN	podoplanin
225825_at	4.26E-04	1.86E-01	C20orf194	chromosome 20 open reading frame 194
1564482_at	4.27E-04	-1.44E-01	ATP5O	ATP synthase, H+ transporting, mitochondrial F1 complex, O subunit
228472_at	4.28E-04	-2.31E-01	CCDC61	coiled-coil domain containing 61
227806_at	4.29E-04	-1.76E-01	C16orf74	chromosome 16 open reading frame 74
228863_at	4.29E-04	5.29E-01	PCDH17	protocadherin 17
213158_at	4.29E-04	5.31E-01	ZBTB20	zinc finger and BTB domain containing 20

212064_x_at	4.30E-04	-2.30E-01	MAZ	MYC associated zinc finger protein
224232_s_at	4.31E-04	-3.94E-01	PRELID1	PRELI domain containing 1
216484_x_at	4.31E-04	-2.27E-01		
237754_at	4.31E-04	-1.23E-01	LOC101928682	uncharacterized LOC101928682
200634_at	4.31E-04	-5.13E-01	PFN1	profilin 1
202264_s_at	4.32E-04	-2.49E-01	TOMM40	translocase of outer mitochondrial membrane 40
32402_s_at	4.33E-04	-1.67E-01	SYMPK	symplekin
1564931_at	4.33E-04	-1.32E-01		
201448_at	4.35E-04	3.24E-01	TIA1	TIA1 cytotoxic granule-associated RNA binding protein
241193_at	4.36E-04	5.98E-01	ETS2	ETS proto-oncogene 2, transcription factor
220270_at	4.37E-04	-9.54E-02	RNF17	ring finger protein 17
231854_at	4.39E-04	2.06E-01	PIK3CA	phosphatidylinositol-4,5-bisphosphate 3-kinase catalytic subunit alpha
202281_at	4.44E-04	-2.91E-01	GAK	cyclin G associated kinase
224733_at	4.45E-04	3.50E-01	CMTM3	CKLF like MARVEL transmembrane domain containing 3
203032_s_at	4.45E-04	-2.23E-01	FH	fumarate hydratase
213011_s_at	4.46E-04	-4.04E-01	TPI1	triosephosphate isomerase 1
225150_s_at	4.47E-04	-3.81E-01	RTKN	rhotekin
217345_at	4.47E-04	-8.36E-02	SMNP	survival of motor neuron 1, telomeric pseudogene
227071_at	4.47E-04	-1.27E-01	ZNF414	zinc finger protein 414
225814_at	4.51E-04	3.40E-01	XRN1	5'-3' exoribonuclease 1
211558_s_at	4.53E-04	-3.82E-01	DHPS	deoxyhypusine synthase
202746_at	4.53E-04	6.26E-01	ITM2A	integral membrane protein 2A
207825_s_at	4.53E-04	-1.52E-01	GHRHR	growth hormone releasing hormone receptor
220571_at	4.53E-04	-1.12E-01	PRDM11	PR/SET domain 11
200822_x_at	4.53E-04	-3.83E-01	TPI1	triosephosphate isomerase 1
229775_s_at	4.54E-04	1.50E-01	AFDN	afadin, adherens junction formation factor
240368_at	4.55E-04	-1.31E-01		
1556583_a_at	4.56E-04	1.83E-01	SLC8A1	solute carrier family 8 member A1
223188_at	4.56E-04	-2.25E-01	SMG9	SMG9, nonsense mediated mRNA decay factor
231692_at	4.56E-04	-1.22E-01	PIGG	phosphatidylinositol glycan anchor biosynthesis class G
211047_x_at	4.56E-04	-3.55E-01	AP2S1	adaptor related protein complex 2 sigma 1 subunit
220242_x_at	4.57E-04	2.81E-01	ZNF701	zinc finger protein 701
236223_s_at	4.59E-04	4.48E-01	RIT1	Ras like without CAAX 1
227266_s_at	4.60E-04	7.22E-01	FYB	FYN binding protein
209763_at	4.61E-04	3.70E-01	CHRDL1	chordin like 1
224464_s_at	4.62E-04	-2.80E-01	NUDT22	nudix hydrolase 22
235056_at	4.66E-04	3.82E-01	ETV6	ETS variant 6
228227_at	4.67E-04	-1.53E-01	ITGB1BP1	integrin subunit beta 1 binding protein 1
221807_s_at	4.68E-04	-2.88E-01	TRABD	TraB domain containing
208981_at	4.68E-04	4.01E-01	PECAM1	platelet and endothelial cell adhesion molecule 1
240689_at	4.69E-04	-1.18E-01	REC114	REC114 meiotic recombination protein
202825_at	4.70E-04	-4.01E-01	SLC25A4	solute carrier family 25 member 4
235410_at	4.70E-04	3.81E-01	NPHP3	nephronophthisis 3 (adolescent)
228790_at	4.71E-04	1.73E-01	FAM110B	family with sequence similarity 110 member B
65517_at	4.71E-04	-3.56E-01	AP1M2	adaptor related protein complex 1 mu 2 subunit
218083_at	4.72E-04	-2.50E-01	PTGES2	prostaglandin E synthase 2
202493_x_at	4.75E-04	-1.00E-01	GH1///CSHL1///CSH1	growth hormone 1///chorionic somatomammotropin hormone like 1///chorionic somatomammotropin hormone 1
236096_at	4.75E-04	-9.78E-02	SIPA1L3	signal induced proliferation associated 1 like 3
224455_s_at	4.75E-04	-2.08E-01	ADPGK	ADP dependent glucokinase
205172_x_at	4.76E-04	-4.78E-01	CLTB	clathrin light chain B
207943_x_at	4.77E-04	7.43E-01	PLAGL1	PLAG1 like zinc finger 1
243983_at	4.78E-04	-1.36E-01		
216206_x_at	4.78E-04	2.23E-01	MAP2K7	mitogen-activated protein kinase kinase 7
204691_x_at	4.81E-04	-1.68E-01	PLA2G6	phospholipase A2 group VI
210023_s_at	4.83E-04	-2.79E-01	PCGF1	polycomb group ring finger 1
201489_at	4.84E-04	-3.68E-01	PPIF	peptidylprolyl isomerase F
225957_at	4.84E-04	4.59E-01	CREBRF	CREB3 regulatory factor
200753_x_at	4.86E-04	-2.64E-01	MIR636///SRSF2	microRNA 636///serine and arginine rich splicing factor 2
235885_at	4.86E-04	1.88E-01	P2RY12	purinergic receptor P2Y12
236748_at	4.86E-04	-1.24E-01	RASGEF1C	RasGEF domain family member 1C
201596_x_at	4.88E-04	-4.22E-01	KRT18	keratin 18
201427_s_at	4.89E-04	8.34E-01	SEPP1	selenoprotein P, plasma, 1
226428_at	4.90E-04	-1.29E-01	TNPO2	transportin 2
240044_x_at	4.92E-04	3.73E-01	TNRC6B	trinucleotide repeat containing 6B
240425_x_at	4.93E-04	3.08E-01	ROBO2	roundabout guidance receptor 2
212667_at	4.94E-04	6.13E-01	SPARC	secreted protein acidic and cysteine rich
204281_at	4.94E-04	-2.73E-01	TEAD4	TEA domain transcription factor 4
235789_at	4.97E-04	-2.00E-01	LOC100507022///KDM4B	FLJ00291 protein///lysine demethylase 4B
1557203_at	4.97E-04	-1.05E-01	PABPC1L2B///PABPC1L2A	poly(A) binding protein cytoplasmic 1 like 2B///poly(A) binding protein cytoplasmic 1 like 2A
1553967_at	4.97E-04	-1.30E-01	ADAT3	adenosine deaminase, tRNA specific 3
215252_at	5.00E-04	4.44E-01	DNAJC7	DnaJ heat shock protein family (Hsp40) member C7
215159_s_at	5.00E-04	-1.68E-01	NADK	NAD kinase
202424_at	5.02E-04	-3.62E-01	MAP2K2	mitogen-activated protein kinase kinase 2
213453_x_at	5.02E-04	-3.86E-01	GAPDH	glyceraldehyde-3-phosphate dehydrogenase

244828_x_at	5.05E-04	-3.89E-01	NAF1	nuclear assembly factor 1 ribonucleoprotein
203562_at	5.06E-04	2.61E-01	FEZ1	fasciculation and elongation protein zeta 1
226516_at	5.06E-04	-1.89E-01	MFSD12	major facilitator superfamily domain containing 12
226401_at	5.07E-04	-1.22E-01	PARP10	poly(ADP-ribose) polymerase family member 10
206171_at	5.08E-04	1.99E-01	ADORA3	adenosine A3 receptor
209624_s_at	5.11E-04	-4.09E-01	MCCC2	methylcrotonoyl-CoA carboxylase 2
202556_s_at	5.14E-04	-2.75E-01	MCRS1	microspherule protein 1
212397_at	5.14E-04	8.08E-01	RDX	radixin
220947_s_at	5.14E-04	-2.79E-01	TBC1D10B	TBC1 domain family member 10B
1553585_a_at	5.14E-04	-1.02E-01	MAGEA10-MAGEA5///MAGEA5	MAGEA10-MAGEA5 readthrough///MAGE family member A5
202765_s_at	5.15E-04	3.18E-01	FBN1	fibrillin 1
206979_at	5.15E-04	-1.23E-01	C8B	complement component 8, beta polypeptide
202953_at	5.15E-04	5.81E-01	C1QB	complement C1q B chain
206722_s_at	5.17E-04	-1.94E-01	LPAR2	lysophosphatidic acid receptor 2
1565601_at	5.17E-04	-8.34E-02		
231816_s_at	5.17E-04	-1.49E-01	UBE2Q1	ubiquitin conjugating enzyme E2 Q1
201490_s_at	5.18E-04	-4.66E-01	PPIF	peptidylprolyl isomerase F
212233_at	5.19E-04	4.97E-01	MAP1B	microtubule associated protein 1B
238544_at	5.22E-04	-1.91E-01		
222838_at	5.23E-04	5.93E-01	SLAMF7	SLAM family member 7
205802_at	5.23E-04	5.12E-01	TRPC1	transient receptor potential cation channel subfamily C member 1
1569936_a_at	5.24E-04	-1.40E-01	ZNF664	zinc finger protein 664
1553634_a_at	5.25E-04	-1.60E-01	WEE2-AS1	WEE2 antisense RNA 1
209596_at	5.26E-04	5.59E-01	MXRA5	matrix remodeling associated 5
241715_x_at	5.26E-04	-1.50E-01	ACPT	acid phosphatase, testicular
218860_at	5.27E-04	-3.12E-01	NOC4L	nucleolar complex associated 4 homolog
218965_s_at	5.28E-04	-1.64E-01	TUT1	terminal uridylyl transferase 1, U6 snRNA-specific
229072_at	5.29E-04	5.92E-01	RAB30	RAB30, member RAS oncogene family
204220_at	5.29E-04	4.37E-01	GMFG	glia maturation factor gamma
209999_x_at	5.30E-04	-1.75E-01	SOCS1	suppressor of cytokine signaling 1
1552868_at	5.30E-04	-1.60E-01	CIRBP-AS1	CIRBP antisense RNA 1
1559713_at	5.31E-04	-1.10E-01		
225242_s_at	5.31E-04	4.32E-01	CCDC80	coiled-coil domain containing 80
226441_at	5.31E-04	3.91E-01	MAP3K2	mitogen-activated protein kinase kinase kinase 2
1553188_s_at	5.32E-04	-1.21E-01	PARD3B	par-3 family cell polarity regulator beta
1554317_s_at	5.32E-04	-1.37E-01	TRMT61A	tRNA methyltransferase 61A
217173_s_at	5.33E-04	-2.09E-01	LDLR	low density lipoprotein receptor
1564697_a_at	5.33E-04	-1.18E-01	LINC01144	long intergenic non-protein coding RNA 1144
229792_at	5.34E-04	-2.47E-01	KLHL17	kelch like family member 17
229723_at	5.35E-04	4.75E-01	TAGAP	T-cell activation RhoGTPase activating protein
235657_at	5.36E-04	2.00E-01		
232294_at	5.38E-04	-1.07E-01	TMEM254-AS1	TMEM254 antisense RNA 1
218366_x_at	5.38E-04	-3.34E-01	METTL17	methyltransferase like 17
242997_at	5.38E-04	-1.57E-01		
218275_at	5.39E-04	-2.87E-01	SLC25A10	solute carrier family 25 member 10
225322_s_at	5.39E-04	-1.48E-01	FAAP100	Fanconi anemia core complex associated protein 100
230068_s_at	5.40E-04	1.02E-01	PEG3-AS1	PEG3 antisense RNA 1
230856_at	5.40E-04	-2.12E-01	ARSG	arylsulfatase G
219778_at	5.40E-04	4.64E-01	ZFPM2	zinc finger protein, FOG family member 2
212201_at	5.41E-04	-2.45E-01	ANKLE2	ankyrin repeat and LEM domain containing 2
218725_at	5.41E-04	-2.37E-01	SLC25A22	solute carrier family 25 member 22
1554466_a_at	5.43E-04	-3.00E-01	METTL26	methyltransferase like 26
203190_at	5.43E-04	-3.49E-01	MIR7113///MIR4691///NDUFS8	microRNA 7113///microRNA 4691///NADH:ubiquinone oxidoreductase core subunit S8
218795_at	5.44E-04	-3.61E-01	ACP6	acid phosphatase 6, lysophosphatidic
225625_at	5.45E-04	-3.66E-01	ALKBH2	alkB homolog 2, alpha-ketoglutarate dependent dioxygenase
218897_at	5.47E-04	-3.48E-01	TMEM177	transmembrane protein 177
212152_x_at	5.47E-04	-2.64E-01	ARID1A	AT-rich interaction domain 1A
223479_s_at	5.49E-04	-4.05E-01	CHCHD5	coiled-coil-helix-coiled-coil-helix domain containing 5
202400_s_at	5.50E-04	-1.32E-01	SRF	serum response factor
204786_s_at	5.50E-04	2.46E-01	IFNAR2	interferon alpha and beta receptor subunit 2
216010_x_at	5.52E-04	-3.89E-01	FUT3	fucosyltransferase 3 (Lewis blood group)
213662_at	5.52E-04	-1.00E-01	FAM134B	family with sequence similarity 134 member B
235308_at	5.52E-04	4.71E-01	ZBTB20	zinc finger and BTB domain containing 20
239258_at	5.53E-04	3.34E-01		
221424_s_at	5.53E-04	-1.33E-01	OR51E2	olfactory receptor family 51 subfamily E member 2
243536_x_at	5.54E-04	-1.25E-01	ARHGAP27	Rho GTPase activating protein 27
214737_x_at	5.54E-04	-2.32E-01	HNRNPC	heterogeneous nuclear ribonucleoprotein C (C1/C2)
207189_s_at	5.55E-04	-1.42E-01	ZZEF1	zinc finger ZZ-type and EF-hand domain containing 1
212344_at	5.57E-04	5.18E-01	SULF1	sulfatase 1
225021_at	5.58E-04	5.14E-01	ZNF532	zinc finger protein 532
1556851_at	5.59E-04	-9.76E-02		
208502_s_at	5.62E-04	-3.25E-01	PITX1	paired like homeodomain 1
1561243_at	5.64E-04	-1.22E-01	TMEM105	transmembrane protein 105
203411_s_at	5.64E-04	-4.00E-01	LMNA	lamin A/C
225752_at	5.64E-04	-3.40E-01	NIPA1	non imprinted in Prader-Willi/Angelman syndrome 1

1560212_a_at	5.64E-04	-9.51E-02	LOC101929248	uncharacterized LOC101929248
41037_at	5.65E-04	-4.36E-01	TEAD4	TEA domain transcription factor 4
221077_at	5.66E-04	-1.13E-01	ARMC4	armadillo repeat containing 4
205885_s_at	5.70E-04	2.67E-01	ITGA4	integrin subunit alpha 4
1554309_at	5.71E-04	-2.57E-01	EIF4G3	eukaryotic translation initiation factor 4 gamma 3
221909_at	5.71E-04	-4.52E-01	RNFT2	ring finger protein, transmembrane 2
222535_at	5.72E-04	-1.14E-01	INF2	inverted formin, FH2 and WH2 domain containing
210167_s_at	5.73E-04	-1.03E-01	TEF	TEF, PAR bZIP transcription factor
212625_at	5.73E-04	-2.56E-01	STX10	syntaxin 10
227132_at	5.75E-04	3.89E-01	ZNF706	zinc finger protein 706
226338_at	5.82E-04	6.28E-01	TMEM55A	transmembrane protein 55A
1320_at	5.83E-04	-9.27E-02	PTPN21	protein tyrosine phosphatase, non-receptor type 21
1553450_s_at	5.84E-04	-8.45E-02	LINC00304	long intergenic non-protein coding RNA 304
1560744_at	5.89E-04	-1.33E-01		
218760_at	5.90E-04	-2.87E-01	COQ6	coenzyme Q6, monooxygenase
1560496_at	5.90E-04	-1.44E-01	MRGPRF-AS1	MRGPRF antisense RNA 1
216562_at	5.91E-04	-1.50E-01		
217444_at	5.92E-04	-1.07E-01		
230435_at	5.93E-04	5.08E-01	FAM228B	family with sequence similarity 228 member B
235891_at	5.95E-04	2.01E-01	DNM3OS	DNM3 opposite strand/antisense RNA
203478_at	5.95E-04	-2.71E-01	NDUFC1	NADH:ubiquinone oxidoreductase subunit C1
203858_s_at	5.96E-04	-2.40E-01	COX10	COX10, heme A:farnesyltransferase cytochrome c oxidase assembly factor
236058_at	5.97E-04	-1.96E-01	KDF1	keratinocyte differentiation factor 1
212155_at	5.99E-04	-2.61E-01	RNF187	ring finger protein 187
208917_x_at	6.03E-04	-1.61E-01	NADK	NAD kinase
211249_at	6.03E-04	-1.43E-01	GPR68	G protein-coupled receptor 68
226724_s_at	6.04E-04	-1.29E-01	PSPC1	paraspeckle component 1
242883_at	6.04E-04	-1.12E-01	OTOS	otospiralin
241655_at	6.05E-04	-7.60E-02		
217398_x_at	6.05E-04	-3.99E-01	GAPDH	glyceraldehyde-3-phosphate dehydrogenase
211250_s_at	6.05E-04	-2.38E-01	SH3BP2	SH3 domain binding protein 2
214121_x_at	6.06E-04	-1.89E-01	PDLIM7	PDZ and LIM domain 7
203875_at	6.06E-04	6.11E-01	SMARCA1	SWI/SNF related, matrix associated, actin dependent regulator of chromatin, subfamily a, member 1
206168_at	6.06E-04	-1.22E-01	ZC3H7B	zinc finger CCCH-type containing 7B
238672_at	6.07E-04	3.72E-01		
1570167_at	6.08E-04	-1.23E-01		
218191_s_at	6.08E-04	2.99E-01	LMBRD1	LMBR1 domain containing 1
216807_at	6.08E-04	-1.23E-01	CFAP74	cilia and flagella associated protein 74
221905_at	6.10E-04	4.19E-01	CYLD	CYLD lysine 63 deubiquitinase
225620_at	6.11E-04	-2.05E-01	RAB35	RAB35, member RAS oncogene family
AFFX-HUMGAPDH/M33197_M_at	6.12E-04	-4.37E-01	GAPDH	glyceraldehyde-3-phosphate dehydrogenase
228504_at	6.13E-04	1.70E-01	SCN7A	sodium voltage-gated channel alpha subunit 7
230790_x_at	6.14E-04	2.81E-01	FOXN3	forkhead box N3
1556724_at	6.19E-04	-1.20E-01	LOC100288490	uncharacterized LOC100288490
221787_at	6.19E-04	2.85E-01	C6orf120	chromosome 6 open reading frame 120
229937_x_at	6.20E-04	2.73E-01		
225798_at	6.20E-04	5.15E-01	JAZF1	JAZF zinc finger 1
215076_s_at	6.20E-04	4.73E-01	COL3A1	collagen type III alpha 1 chain
220766_at	6.20E-04	-1.33E-01	BTG4	BTG anti-proliferation factor 4
211817_s_at	6.22E-04	-1.28E-01	KCNJ5	potassium voltage-gated channel subfamily J member 5
213700_s_at	6.25E-04	-3.88E-01	PKM	pyruvate kinase, muscle
212226_s_at	6.26E-04	3.88E-01	PLPP3	phospholipid phosphatase 3
237187_at	6.26E-04	-1.25E-01	HRK	harakiri, BCL2 interacting protein
242741_x_at	6.28E-04	1.72E-01		
1555544_a_at	6.29E-04	-1.03E-01	CADM2	cell adhesion molecule 2
1552937_s_at	6.29E-04	-3.08E-01	ATRIP///TREX1	ATR interacting protein///three prime repair exonuclease 1
244776_at	6.30E-04	-1.08E-01		
1554909_at	6.32E-04	-9.73E-02	CCDC36	coiled-coil domain containing 36
223936_s_at	6.32E-04	-1.51E-01	FOXP1	forkhead box P1
238245_at	6.33E-04	-1.74E-01	ENPP7	ectonucleotide pyrophosphatase/phosphodiesterase 7
236105_at	6.35E-04	7.48E-01	ZBTB10	zinc finger and BTB domain containing 10
233131_at	6.36E-04	-1.29E-01	CFAP58	cilia and flagella associated protein 58
205035_at	6.37E-04	-1.64E-01	CTDP1	CTD phosphatase subunit 1
215218_s_at	6.37E-04	-1.42E-01	WDR62	WD repeat domain 62
211595_s_at	6.39E-04	-3.26E-01	MRPS11	mitochondrial ribosomal protein S11
225552_x_at	6.39E-04	-2.98E-01	AURKAIP1	aurora kinase A interacting protein 1
214992_s_at	6.39E-04	-1.54E-01	DNASE2	deoxyribonuclease 2, lysosomal
1553439_at	6.39E-04	-1.14E-01		
241019_at	6.40E-04	-9.05E-02		
224518_s_at	6.40E-04	5.75E-01	ZNF559	zinc finger protein 559
242723_at	6.40E-04	-3.84E-01		
201511_at	6.42E-04	-2.89E-01	AAMP	angio associated migratory cell protein
1555270_a_at	6.43E-04	-3.95E-01	WFS1	wolframin ER transmembrane glycoprotein
208576_s_at	6.43E-04	-2.72E-01	HIST1H3B	histone cluster 1, H3b

226451_at	6.47E-04	-2.38E-01	FDX1L	ferredoxin 1 like
216026_s_at	6.48E-04	-2.63E-01	POLE	DNA polymerase epsilon, catalytic subunit
1556935_at	6.49E-04	2.64E-01		
210864_x_at	6.51E-04	-1.46E-01	HFE	hemochromatosis
224811_at	6.51E-04	2.73E-01	LPP	LIM domain containing preferred translocation partner in lipoma
211181_x_at	6.54E-04	-1.28E-01	LOC101928269///LOC100506403///RUNX1	uncharacterized LOC101928269///uncharacterized LOC100506403///runt related transcription factor 1
206499_s_at	6.54E-04	-3.24E-01	RCC1	regulator of chromosome condensation 1
218112_at	6.55E-04	-2.96E-01	MRPS34	mitochondrial ribosomal protein S34
226820_at	6.55E-04	4.14E-01	ZNF362	zinc finger protein 362
228586_at	6.58E-04	-1.09E-01		
225345_s_at	6.58E-04	3.51E-01	FBXO32	F-box protein 32
203023_at	6.61E-04	-4.01E-01	NOP16	NOP16 nucleolar protein
221447_s_at	6.62E-04	2.60E-01	GLT8D2	glycosyltransferase 8 domain containing 2
229080_at	6.65E-04	-1.30E-01	COL26A1	collagen type XXVI alpha 1 chain
221494_x_at	6.66E-04	-2.95E-01	EIF3K	eukaryotic translation initiation factor 3 subunit K
1553728_at	6.66E-04	-2.02E-01	LRRC43	leucine rich repeat containing 43
1553647_at	6.69E-04	-1.23E-01	CDYL2	chromodomain Y-like 2
222509_s_at	6.71E-04	-1.33E-01	ZNF672	zinc finger protein 672
218046_s_at	6.72E-04	-3.67E-01	MRPS16	mitochondrial ribosomal protein S16
218865_at	6.73E-04	-4.43E-01	01-3	mitochondrial amidoxime reducing component 1
37892_at	6.73E-04	9.73E-01	COL11A1	collagen type XI alpha 1 chain
235556_at	6.74E-04	5.10E-01	CREBRF	CREB3 regulatory factor
241859_at	6.74E-04	1.12E-01	PLCL1	phospholipase C like 1
43934_at	6.76E-04	-1.04E-01	GPR137	G protein-coupled receptor 137
208982_at	6.76E-04	4.06E-01	PECAM1	platelet and endothelial cell adhesion molecule 1
236401_at	6.76E-04	1.20E-01	GIMAP1	GTPase, IMAP family member 1
217294_s_at	6.77E-04	-7.22E-01	ENO1	enolase 1
1561561_x_at	6.77E-04	-1.11E-01		
221937_at	6.78E-04	2.81E-01	SYNRG	synergin, gamma
202240_at	6.79E-04	-3.50E-01	PLK1	polo like kinase 1
230284_at	6.80E-04	-1.35E-01	MYOM3	myomesin 3
208634_s_at	6.81E-04	2.43E-01	MACF1	microtubule-actin crosslinking factor 1
228585_at	6.82E-04	3.27E-01	ENTPD1	ectonucleoside triphosphate diphosphohydrolase 1
219611_s_at	6.83E-04	-1.49E-01	CEP85	centrosomal protein 85
233621_s_at	6.84E-04	-1.04E-01	ARHGEF12	Rho guanine nucleotide exchange factor 12
1552309_a_at	6.84E-04	4.27E-01	NEXN	nexilin F-actin binding protein
220015_at	6.87E-04	-1.65E-01	CASZ1	castor zinc finger 1
236695_at	6.87E-04	-1.22E-01	STK4-AS1	STK4 antisense RNA 1 (head to head)
212581_x_at	6.89E-04	-3.61E-01	GAPDH	glyceraldehyde-3-phosphate dehydrogenase
211474_s_at	6.89E-04	-5.09E-01	SERPINB6	serpin family B member 6
207339_s_at	6.92E-04	2.71E-01	LTB	lymphotoxin beta
1554951_at	6.94E-04	-1.01E-01		
244830_at	6.96E-04	-9.53E-02	RASIP1	Ras interacting protein 1
239050_s_at	6.96E-04	4.56E-01	CRIPT	CXXC repeat containing interactor of PDZ3 domain
214511_x_at	6.97E-04	3.21E-01	FCGR1B	Fc fragment of IgG receptor Ib
226966_at	6.97E-04	-1.98E-01	PRPF40B	pre-mRNA processing factor 40 homolog B
227289_at	6.98E-04	4.97E-01	PCDH17	protocadherin 17
234373_x_at	6.98E-04	-1.53E-01		
210122_at	7.01E-04	-1.03E-01	PRM2	protamine 2
218441_s_at	7.02E-04	-2.47E-01	RPAP1	RNA polymerase II associated protein 1
227469_at	7.03E-04	-1.00E-01		
201417_at	7.04E-04	4.15E-01	SOX4	SRY-box 4
219350_s_at	7.06E-04	-2.41E-01	DIABLO	diablo IAP-binding mitochondrial protein
200594_x_at	7.09E-04	-1.88E-01	HNRNPU	heterogeneous nuclear ribonucleoprotein U
218110_at	7.09E-04	-1.37E-01	XAB2	XPA binding protein 2
1553222_at	7.10E-04	-1.10E-01	OXER1	oxoeicosanoid receptor 1
1562211_a_at	7.11E-04	-1.47E-01	ZNF491	zinc finger protein 491
1558589_at	7.11E-04	-8.91E-02	LINC00417	long intergenic non-protein coding RNA 417
219843_at	7.12E-04	3.15E-01	IPP	intracisternal A particle-promoted polypeptide
223614_at	7.13E-04	2.16E-01	MMP16	matrix metallopeptidase 16
234813_at	7.14E-04	-9.83E-02	TMEM108	transmembrane protein 108
244186_at	7.15E-04	-1.16E-01		
203728_at	7.16E-04	-3.38E-01	BAK1	BCL2 antagonist/killer 1
223784_at	7.17E-04	4.53E-01	TMEM27	transmembrane protein 27
206034_at	7.17E-04	-3.81E-01	SERPINB8	serpin family B member 8
230050_at	7.19E-04	-1.44E-01	NACC2	NACC family member 2
206284_x_at	7.19E-04	-4.97E-01	CLTB	clathrin light chain B
AFFX-HUMGAPDH/M33197_5_at	7.20E-04	-5.65E-01	GAPDH	glyceraldehyde-3-phosphate dehydrogenase
225254_at	7.21E-04	-1.07E-01	CCDC97	coiled-coil domain containing 97
223501_at	7.23E-04	6.04E-01	TNFSF13B	tumor necrosis factor superfamily member 13b
228885_at	7.26E-04	1.65E-01	MAMDC2	MAM domain containing 2
214247_s_at	7.26E-04	4.54E-01	DKK3	dickkopf WNT signaling pathway inhibitor 3
208260_at	7.26E-04	-1.19E-01	AVPR1B	arginine vasopressin receptor 1B
236891_at	7.27E-04	-1.04E-01		

239286_at	7.28E-04	3.15E-01	CDH11	cadherin 11
231811_at	7.31E-04	-1.23E-01	LOC100653296///LOC100288778///WASH1///LOC100134445///WASH7P///WASH5P///WASH2P///WASH3P	uncharacterized LOC100653296///WAS protein family homolog 1 pseudogene///WAS protein family homolog 1///uncharacterized LOC100134445///WAS protein family homolog 7 pseudogene///WAS protein family homolog 5 pseudogene///WAS protein family homolog 2 pseudogene///WAS protein family homolog 3 pseudogene
238250_at	7.36E-04	-1.25E-01		
214098_at	7.37E-04	4.31E-01	KIAA1107	KIAA1107
201331_s_at	7.37E-04	-2.97E-01	STAT6	signal transducer and activator of transcription 6
226930_at	7.39E-04	6.90E-01	FNDC1	fibronectin type III domain containing 1
200041_s_at	7.42E-04	-3.81E-01	ATP6V1G2-DDX39B///SNORD84///DDX39B	ATP6V1G2-DDX39B readthrough (NMD candidate)///small nucleolar RNA, C/D box 84///DEAD-box helicase 39B
202296_s_at	7.42E-04	-3.88E-01	RER1	retention in endoplasmic reticulum sorting receptor 1
204757_s_at	7.43E-04	-1.68E-01	C2CD2L	C2CD2 like
1564139_at	7.46E-04	1.76E-01	A2M-AS1	A2M antisense RNA 1 (head to head)
225853_at	7.48E-04	-3.63E-01	GNPNAT1	glucosamine-phosphate N-acetyltransferase 1
243357_at	7.48E-04	2.42E-01	NEGR1	neuronal growth regulator 1
1554261_at	7.48E-04	-1.33E-01	KLHL29	kelch like family member 29
1562022_s_at	7.48E-04	-2.32E-01	LOC100130987///RAD9A	uncharacterized LOC100130987///RAD9 checkpoint clamp component A
212554_at	7.49E-04	5.87E-01	CAP2	CAP, adenylate cyclase-associated protein, 2 (yeast)
207923_x_at	7.49E-04	-1.05E-01	PAX8	paired box 8
1554661_s_at	7.49E-04	-2.40E-01	CNST	consortin, connexin sorting protein
214576_at	7.50E-04	-1.34E-01	KRT36	keratin 36
1559515_at	7.53E-04	1.86E-01		
1564373_a_at	7.53E-04	-1.25E-01	LOC283887	uncharacterized LOC283887
229670_at	7.55E-04	3.26E-01		
221117_at	7.55E-04	-7.57E-02		
230522_s_at	7.56E-04	-1.82E-01	ARHGEF39	Rho guanine nucleotide exchange factor 39
242484_at	7.56E-04	-1.07E-01	TTC9B	tetratricopeptide repeat domain 9B
50314_i_at	7.58E-04	-2.60E-01	C20orf27	chromosome 20 open reading frame 27
211067_s_at	7.58E-04	2.85E-01	GAS7	growth arrest specific 7
213389_at	7.59E-04	-2.43E-01	ZNF592	zinc finger protein 592
244289_at	7.59E-04	3.13E-01	ZNF300P1	zinc finger protein 300 pseudogene 1
220272_at	7.61E-04	1.68E-01	BNC2	basonuclin 2
227998_at	7.62E-04	-4.90E-01	S100A16	S100 calcium binding protein A16
220252_x_at	7.65E-04	2.07E-01	CXorf21	chromosome X open reading frame 21
218443_s_at	7.66E-04	-2.40E-01	DAZAP1	DAZ associated protein 1
215235_at	7.67E-04	-3.52E-01	SPTAN1	spectrin alpha, non-erythrocytic 1
239734_at	7.68E-04	2.50E-01		
203365_s_at	7.68E-04	-3.22E-01	MMP15	matrix metallopeptidase 15
224333_s_at	7.68E-04	-2.31E-01	MRPS5	mitochondrial ribosomal protein S5
224064_s_at	7.69E-04	-2.60E-01	DHDDS	dehydrodolichyl diphosphate synthase subunit
242830_at	7.72E-04	-1.06E-01		
214955_at	7.72E-04	-1.35E-01	TMPRSS6	transmembrane protease, serine 6
232078_at	7.73E-04	-2.59E-01	NECTIN2	nectin cell adhesion molecule 2
226119_at	7.76E-04	3.53E-01	PCMTD1	protein-L-isoaspartate (D-aspartate) O-methyltransferase domain containing 1
1553812_at	7.77E-04	-1.26E-01	TLE6	transducin like enhancer of split 6
209416_s_at	7.77E-04	-1.31E-01	FZR1	fizzy/cell division cycle 20 related 1
231104_at	7.78E-04	-3.79E-01	TDRD5	tudor domain containing 5
AFFX-HUMGAPDH/M33197_3_at	7.80E-04	-2.82E-01	GAPDH	glyceraldehyde-3-phosphate dehydrogenase
232329_at	7.81E-04	-9.99E-02	RANBP10	RAN binding protein 10
239918_at	7.82E-04	-1.05E-01	PVR	poliovirus receptor
216581_at	7.83E-04	-1.38E-01		
1566690_at	7.87E-04	-1.19E-01		
218196_at	7.89E-04	3.71E-01	OSTM1	osteopetrosis associated transmembrane protein 1
239395_at	7.91E-04	-1.61E-01	ARRDC3-AS1	ARRDC3 antisense RNA 1
205303_at	7.94E-04	4.39E-01	KCNJ8	potassium voltage-gated channel subfamily J member 8
217904_s_at	7.94E-04	3.57E-01	BACE1	beta-secretase 1
232640_at	7.94E-04	-1.37E-01	COMMD5	COMM domain containing 5
223988_x_at	7.96E-04	-3.09E-01	METTL17	methyltransferase like 17
200828_s_at	7.96E-04	-2.89E-01	ZNF207	zinc finger protein 207
222379_at	7.96E-04	2.12E-01	KCNE4	potassium voltage-gated channel subfamily E regulatory subunit 4
239888_at	7.96E-04	4.40E-01	UBQLN2	ubiquilin 2
212263_at	7.97E-04	4.90E-01	QKI	QKI, KH domain containing RNA binding
205884_at	7.98E-04	2.16E-01	ITGA4	integrin subunit alpha 4
225404_at	7.99E-04	-2.63E-01	SMIM12	small integral membrane protein 12
226531_at	8.00E-04	-2.56E-01	ORAI1	ORAI calcium release-activated calcium modulator 1
212003_at	8.02E-04	-1.63E-01	SZRD1	SUZ RNA binding domain containing 1
218580_x_at	8.05E-04	-3.04E-01	AURKAIP1	aurora kinase A interacting protein 1
228993_s_at	8.05E-04	4.06E-01	BBIP1	BBSome interacting protein 1
1555301_a_at	8.05E-04	-3.35E-01	DIP2A	disco interacting protein 2 homolog A
209008_x_at	8.05E-04	-5.06E-01	KRT8	keratin 8
227978_s_at	8.06E-04	-3.86E-01	ZADH2	zinc binding alcohol dehydrogenase domain containing 2

203660_s_at	8.08E-04	-2.51E-01	PCNT	pericentrin
1557595_at	8.10E-04	-1.18E-01	GINS2	GINS complex subunit 2
209461_x_at	8.11E-04	-3.60E-01	WDR18	WD repeat domain 18
228148_at	8.12E-04	-2.00E-01	ZNF584	zinc finger protein 584
225120_at	8.13E-04	3.20E-01	MIR4657///PURB	microRNA 4657///purine rich element binding protein B
232419_at	8.14E-04	-1.43E-01	TMEM132A	transmembrane protein 132A
202849_x_at	8.14E-04	-1.31E-01	GRK6	G protein-coupled receptor kinase 6
202855_s_at	8.16E-04	-2.37E-01	MIR6787///SLC16A3	microRNA 6787///solute carrier family 16 member 3
203231_s_at	8.17E-04	6.38E-01	ATXN1	ataxin 1
220200_s_at	8.21E-04	-1.79E-01	KMT5A	lysine methyltransferase 5A
232079_s_at	8.22E-04	-3.74E-01	NECTIN2	nectin cell adhesion molecule 2
241724_x_at	8.25E-04	1.27E-01		
244064_at	8.25E-04	-1.17E-01		
232896_at	8.27E-04	-1.09E-01	ERBIN	erbb2 interacting protein
223035_s_at	8.27E-04	-2.95E-01	FARSB	phenylalanyl-tRNA synthetase beta subunit
219090_at	8.31E-04	5.04E-01	SLC24A3	solute carrier family 24 member 3
1553686_at	8.35E-04	-1.44E-01	C18orf25	chromosome 18 open reading frame 25
224130_s_at	8.38E-04	-4.08E-01	SRA1	steroid receptor RNA activator 1
235980_at	8.41E-04	3.22E-01	PIK3CA	phosphatidylinositol-4,5-bisphosphate 3-kinase catalytic subunit alpha
213756_s_at	8.42E-04	-1.33E-01	HSF1	heat shock transcription factor 1
213122_at	8.42E-04	3.56E-01	TSPYL5	TSPY like 5
202041_s_at	8.45E-04	-3.75E-01	FIBP	FGF1 intracellular binding protein
224907_s_at	8.47E-04	-2.23E-01	SH3GLB2	SH3 domain containing GRB2 like endophilin B2
212979_s_at	8.49E-04	4.15E-01	LOC100294033///TCAF1	protein FAM115A-like///TRPM8 channel associated factor 1
235752_at	8.49E-04	-1.71E-01		
1563078_at	8.53E-04	-1.04E-01	LOC101929460	uncharacterized LOC101929460
220588_at	8.53E-04	-1.44E-01	BCAS4	breast carcinoma amplified sequence 4
212298_at	8.54E-04	4.93E-01	NRP1	neuropilin 1
215024_at	8.55E-04	2.45E-01	CCZ1B	CCZ1 homolog B, vacuolar protein trafficking and biogenesis associated
240099_at	8.57E-04	-1.36E-01		
208003_s_at	8.57E-04	4.76E-01	NFAT5	nuclear factor of activated T-cells 5
243378_at	8.58E-04	2.21E-01		
209953_s_at	8.60E-04	-3.30E-01	CDC37	cell division cycle 37
211043_s_at	8.61E-04	-4.11E-01	CLTB	clathrin light chain B
221373_x_at	8.61E-04	-1.49E-01	PSPN	persephin
231773_at	8.62E-04	1.39E-01	ANGPTL1	angiopoietin like 1
221849_s_at	8.62E-04	-2.68E-01	DCAF15	DDB1 and CUL4 associated factor 15
217930_s_at	8.64E-04	-2.20E-01	TOLLIP	toll interacting protein
35201_at	8.64E-04	-2.33E-01	HNRNPL	heterogeneous nuclear ribonucleoprotein L
218070_s_at	8.64E-04	-2.44E-01	GMPPA	GDP-mannose pyrophosphorylase A
203832_at	8.66E-04	-2.88E-01	SNRPF	small nuclear ribonucleoprotein polypeptide F
1560922_s_at	8.67E-04	-1.77E-01	ZNF169	zinc finger protein 169
1557867_s_at	8.68E-04	-1.28E-01	CFAP157	cilia and flagella associated protein 157
202477_s_at	8.71E-04	-2.64E-01	TUBGCP2	tubulin gamma complex associated protein 2
206005_s_at	8.75E-04	2.67E-01	CEP162	centrosomal protein 162
238009_at	8.75E-04	4.59E-01	SOX5	SRY-box 5
1555775_a_at	8.75E-04	-9.49E-02	ZAR1	zygote arrest 1
203189_s_at	8.76E-04	-4.36E-01	MIR7113///MIR4691///NDUFS8	microRNA 7113///microRNA 4691///NADH:ubiquinone oxidoreductase core subunit S8
201950_x_at	8.76E-04	-3.42E-01	CAPZB	capping actin protein of muscle Z-line beta subunit
202072_at	8.76E-04	-3.58E-01	HNRNPL	heterogeneous nuclear ribonucleoprotein L
217349_s_at	8.77E-04	-1.35E-01	PRICKLE3	prickle planar cell polarity protein 3
201600_at	8.78E-04	-3.64E-01	PHB2	prohibitin 2
212716_s_at	8.79E-04	-3.17E-01	EIF3K	eukaryotic translation initiation factor 3 subunit K
223744_s_at	8.81E-04	-1.75E-01	SIAE	sialic acid acetylesterase
202434_s_at	8.81E-04	-1.15E-01	CYP1B1	cytochrome P450 family 1 subfamily B member 1
210161_at	8.84E-04	-1.09E-01	NFATC1	nuclear factor of activated T-cells 1
206153_at	8.84E-04	-1.83E-01	CYP4F11	cytochrome P450 family 4 subfamily F member 11
227395_at	8.85E-04	4.52E-01	TBCEL	tubulin folding cofactor E like
1557637_at	8.86E-04	-1.61E-01		
213974_at	8.86E-04	2.36E-01	ADAMTSL3	ADAMTS like 3
208667_s_at	8.88E-04	2.60E-01	ST13	suppression of tumorigenicity 13 (colon carcinoma) (Hsp70 interacting protein)
241464_s_at	8.89E-04	-2.41E-01		
214011_s_at	8.91E-04	-3.60E-01	NOP16	NOP16 nucleolar protein
224549_x_at	8.91E-04	3.07E-01		
224542_s_at	8.92E-04	-1.11E-01	NFATC2	nuclear factor of activated T-cells 2
233549_at	8.93E-04	-9.76E-02	PDE1A	phosphodiesterase 1A
1566700_at	8.94E-04	-1.24E-01	VRK3	vaccinia related kinase 3
217189_s_at	8.95E-04	-1.55E-01	SMG7	SMG7, nonsense mediated mRNA decay factor
225646_at	8.96E-04	3.39E-01	CTSC	cathepsin C
242555_at	8.97E-04	3.68E-01	C16orf87	chromosome 16 open reading frame 87
200773_x_at	8.98E-04	-1.52E-01	PTMA	prothymosin, alpha
215507_x_at	8.98E-04	1.68E-01	RAB22A	RAB22A, member RAS oncogene family
1570292_at	8.99E-04	-8.57E-02	LOC101927123	uncharacterized LOC101927123
57588_at	9.00E-04	4.60E-01	SLC24A3	solute carrier family 24 member 3
209190_s_at	9.02E-04	-2.74E-01	DIAPH1	diaphanous related formin 1

217662_x_at	9.03E-04	2.46E-01		
204473_s_at	9.04E-04	-2.51E-01	ZNF592	zinc finger protein 592
239364_at	9.05E-04	2.55E-01	ETV6	ETS variant 6
206353_at	9.05E-04	-9.35E-02	COX6A2	cytochrome c oxidase subunit 6A2
215179_x_at	9.05E-04	2.24E-01	PGF	placental growth factor
1554444_s_at	9.05E-04	-1.21E-01	SLC35F6	solute carrier family 35 member F6
217967_s_at	9.06E-04	4.84E-01	FAM129A	family with sequence similarity 129 member A
200913_at	9.06E-04	-3.38E-01	PPM1G	protein phosphatase, Mg2+/Mn2+ dependent 1G
230121_at	9.06E-04	1.69E-01	SERTAD4-AS1	SERTAD4 antisense RNA 1
1569487_at	9.06E-04	-1.39E-01		
202822_at	9.07E-04	2.12E-01	LPP	LIM domain containing preferred translocation partner in lipoma
242358_at	9.09E-04	1.06E-01	RASSF8-AS1	RASSF8 atnisense RNA 1
214062_x_at	9.14E-04	-2.08E-01	NFKBIB	NFKB inhibitor beta
217783_s_at	9.16E-04	3.41E-01	YPEL5	yippee like 5
212179_at	9.17E-04	4.51E-01	PNISR	PNN interacting serine and arginine rich protein
212137_at	9.18E-04	-2.30E-01	LARP1	La ribonucleoprotein domain family member 1
225315_at	9.19E-04	-3.67E-01	MRPL21	mitochondrial ribosomal protein L21
228167_at	9.19E-04	5.06E-01	KLHL6	kelch like family member 6
234303_s_at	9.21E-04	1.38E-01	GPR85	G protein-coupled receptor 85
201122_x_at	9.24E-04	-2.26E-01	EIF5A	eukaryotic translation initiation factor 5A
227497_at	9.25E-04	4.51E-01	SOX6	SRY-box 6
1564002_a_at	9.25E-04	2.64E-01	AK9	adenylate kinase 9
239754_at	9.27E-04	-1.32E-01	SNORD65///SNORD49B///LRRC75A-AS1///SNORD49A	small nucleolar RNA, C/D box 65///small nucleolar RNA, C/D box 49B///LRRC75A antisense RNA 1///small nucleolar RNA, C/D box 49A
1559332_at	9.27E-04	3.52E-01		
221383_at	9.27E-04	-1.25E-01	NMUR1	neuromedin U receptor 1
214096_s_at	9.28E-04	-3.48E-01	SHMT2	serine hydroxymethyltransferase 2
227171_at	9.28E-04	-1.24E-01	CCT4	chaperonin containing TCP1 subunit 4
226633_at	9.30E-04	3.44E-01	RAB8B	RAB8B, member RAS oncogene family
243538_at	9.30E-04	-1.69E-01		
217849_s_at	9.32E-04	-2.94E-01	CDC42BPB	CDC42 binding protein kinase beta
222030_at	9.32E-04	-2.40E-01	SIVA1	SIVA1 apoptosis inducing factor
206575_at	9.33E-04	-1.36E-01	CDKL5	cyclin dependent kinase like 5
227627_at	9.35E-04	4.30E-01	C8orf44-SGK3///SGK3	C8orf44-SGK3 readthrough///serum/glucocorticoid regulated kinase family member 3
210386_s_at	9.37E-04	-2.58E-01	MTX1	metaxin 1
241307_at	9.41E-04	-1.03E-01		
207788_s_at	9.44E-04	-1.27E-01	SORBS3	sorbin and SH3 domain containing 3
214531_s_at	9.46E-04	-3.25E-01	SNX1	sorting nexin 1
243140_at	9.47E-04	2.61E-01	ACTA2	actin, alpha 2, smooth muscle, aorta
227300_at	9.47E-04	1.74E-01	TMEM119	transmembrane protein 119
49306_at	9.47E-04	2.54E-01	RASSF4	Ras association domain family member 4
242958_x_at	9.51E-04	8.95E-02		
218142_s_at	9.54E-04	3.12E-01	CRBN	cereblon
1561384_a_at	9.55E-04	-8.50E-02	LOC284661	uncharacterized LOC284661
204760_s_at	9.55E-04	-2.21E-01	NR1D1///THRA	nuclear receptor subfamily 1 group D member 1///thyroid hormone receptor, alpha
217450_at	9.58E-04	-9.52E-02	AGFG2	ArfGAP with FG repeats 2
241380_at	9.59E-04	-1.08E-01	ARHGEF37	Rho guanine nucleotide exchange factor 37
212122_at	9.60E-04	3.68E-01	RHOQ	ras homolog family member Q
227148_at	9.60E-04	4.88E-01	PLEKHH2	pleckstrin homology, MyTH4 and FERM domain containing H2
206331_at	9.62E-04	3.18E-01	CALCRL	calcitonin receptor like receptor
1560353_at	9.64E-04	-1.05E-01		
212691_at	9.64E-04	-2.15E-01	NUP188	nucleoporin 188
218745_x_at	9.65E-04	-2.23E-01	TMEM161A	transmembrane protein 161A
230043_at	9.66E-04	6.43E-01	MUC20	mucin 20, cell surface associated
1560412_at	9.68E-04	-1.18E-01	LOC100507506	uncharacterized LOC100507506
213209_at	9.69E-04	-1.07E-01	TAF6L	TATA-box binding protein associated factor 6 like
203109_at	9.69E-04	-4.15E-01	UBE2M	ubiquitin conjugating enzyme E2 M
1562121_at	9.70E-04	-8.58E-02	CHL1-AS1	CHL1 antisense RNA 1
215859_at	9.71E-04	-9.61E-02	NCLN	nicalin
205145_s_at	9.72E-04	-2.59E-01	MYL5	myosin light chain 5
220323_at	9.72E-04	-1.82E-01	CNTD2	cyclin N-terminal domain containing 2
232125_at	9.72E-04	3.73E-01	ZBTB20	zinc finger and BTB domain containing 20
219207_at	9.73E-04	-2.99E-01	EDC3	enhancer of mRNA decapping 3
203477_at	9.74E-04	6.31E-01	COL15A1	collagen type XV alpha 1 chain
217347_at	9.74E-04	-1.84E-01		
242262_x_at	9.74E-04	-9.06E-02	LINC01360	long intergenic non-protein coding RNA 1360
AFFX-LysX-5_at	9.79E-04	-2.65E-01		
201173_x_at	9.79E-04	-3.06E-01	NUDC	nuclear distribution C, dynein complex regulator
209792_s_at	9.79E-04	-9.80E-01	KLK10	kallikrein related peptidase 10
1553060_at	9.80E-04	-1.28E-01	PSKH2	protein serine kinase H2
201563_at	9.81E-04	-4.50E-01	SORD	sorbitol dehydrogenase
239217_x_at	9.84E-04	-2.69E-01	ABCC3	ATP binding cassette subfamily C member 3
236297_at	9.84E-04	4.07E-01	PLXDC2	plexin domain containing 2

1554907_a_at	9.84E-04	-1.30E-01	LOC101930373///HYDIN2///HYDIN	hydrocephalus-inducing protein homolog///HYDIN2, axonemal central pair apparatus protein (pseudogene)///HYDIN, axonemal central pair apparatus protein
236458_at	9.86E-04	4.13E-01		
206139_at	9.88E-04	-1.14E-01	PI4KB	phosphatidylinositol 4-kinase beta
242803_at	9.90E-04	2.84E-01		
232964_at	9.91E-04	2.80E-01	SPDYE2B///SPDYE6///SPDYE5///SPDYE2///SPDYE1	speedy/RINGO cell cycle regulator family member E2B///speedy/RINGO cell cycle regulator family member E6///speedy/RINGO cell cycle regulator family member E5///speedy/RINGO cell cycle regulator family member E2///speedy/RINGO cell cycle regulator family member E1
203573_s_at	9.94E-04	-3.12E-01	RABGGTA	Rab geranylgeranyltransferase alpha subunit
234465_at	9.94E-04	-1.77E-01	EME1	essential meiotic structure-specific endonuclease 1
207582_at	9.95E-04	-1.18E-01	PIN1P1	peptidylprolyl cis/trans isomerase, NIMA-interacting 1 pseudogene 1
203171_s_at	9.96E-04	-2.48E-01	RRP8	ribosomal RNA processing 8, methyltransferase, homolog (yeast)
1555392_at	9.99E-04	3.57E-01		

Table S3. List of DEGs in DCLK1-B WT versus DCLK1-B KO HCT116 cells

Gene Symbol	DCLK1-B WT			DCLK1B-KO			Full descriptions of the gene	Locus on r	RefSeq mR	RefSeq mR	RefSeq ncR	RefSeq ncR	RefSeq pro	RefSeq pro	Gene type	Ensembl G	Entrez Gen	HGNC ID(s)
	WT1	WT2	WT3	KO1	KO2	KO3												
A1BG	0.057389	0	0.098051	0.121427	0	0.13007	alpha-1-B glycoprotein [Source:HGNC Symbol;Acc:HGNC:5]	chr19:5885	NM_13078	.	.	.	NP_57060	.	protein_co	ENSG0000	1	5
A1CF	0.17037	0.071402	0.029692	0.011344	0.153164	0.03151	APOBEC1 complementation factor [Source:HGNC Symbol;Acc:HG	chr10:5255	NM_00119	XM_01701	.	.	NP_00118	XP_00526	protein_co	ENSG0000	29974	24086
A2M	0.02614	0.040728	0.022744	0	0.029566	0.01349	alpha-2-macroglobulin [Source:HGNC Symbol;Acc:HGNC:7]	chr12:922	NM_00001	XM_00671	.	.	NP_00000	XP_00671	protein_co	ENSG0000	2	7
A2ML1	0	0.025338	0	0	0	0	alpha-2-macroglobulin like 1 [Source:HGNC Symbol;Acc:HGNC:23	chr12:897	NM_00128	XM_01152	.	.	NP_00126	XP_01151	protein_co	ENSG0000	144568	23336
A3GALT2	0	0.241825	0	0	0.145016	0	alpha 1,3-galactosyltransferase 2 [Source:HGNC Symbol;Acc:HGN	chr1:3377	NM_00108	.	.	.	NP_00107	.	protein_co	ENSG0000	127550	30005
A4GALT	6.66231	6.9808	8.44548	7.84005	6.9955	8.0222	alpha 1,4-galactosyltransferase [Source:HGNC Symbol;Acc:HGNC	chr22:4308	NM_00131	XM_01153	.	.	NP_00130	XP_00526	protein_co	ENSG0000	53947	18149
A4GNT	0	0	0	0	0	0	alpha-1,4-N-acetylglucosaminyltransferase [Source:HGNC Symbo	chr3:13784	NM_0161	.	.	.	NP_05724	XP_01686	protein_co	ENSG0000	51146	17968
AAAS	41.4229	41.5927	33.4998	47.0711	35.9008	30.4847	aladin WD repeat nucleoporin [Source:HGNC Symbol;Acc:HGNC:1	chr12:537	NM_00117	XM_01153	.	.	NP_00116	XP_01153	protein_co	ENSG0000	8086	13666
AACS	6.15576	7.96496	7.92365	7.74371	6.45278	6.51129	acetoacetyl-CoA synthetase [Source:HGNC Symbol;Acc:HGNC:21	chr12:125	NM_02392	XM_00525	.	.	NP_07641	.	protein_co	ENSG0000	65985	21298
AADAC	2.07691	3.25846	4.07044	1.27104	1.59527	1.78065	arylacetamide deacetylase [Source:HGNC Symbol;Acc:HGNC:17]	chr3:1515	NM_00108	XM_00524	.	.	NP_00107	XP_00524	protein_co	ENSG0000	13	17
AADACL2	0	0	0	0	0	0	arylacetamide deacetylase like 2 [Source:HGNC Symbol;Acc:HGN	chr3:1514	NM_2073	.	.	.	NP_99724	.	protein_co	ENSG0000	344752	24427
AADACL3	0	0	0	0	0	0	arylacetamide deacetylase like 3 [Source:HGNC Symbol;Acc:HGN	chr1:1277	NM_0011	.	NR_11198	.	NP_00109	.	protein_co	ENSG0000	126767	32037
AADACL4	0	0	0	0	0	0	arylacetamide deacetylase like 4 [Source:HGNC Symbol;Acc:HGN	chr1:12704	NM_00101	.	.	.	NP_00101	.	protein_co	ENSG0000	343066	32038
AADAT	9.24151	9.31816	7.88466	8.21248	8.57787	7.97648	aminoadipate aminotransferase [Source:HGNC Symbol;Acc:HGN	chr4:1709	NM_00128	XM_00671	.	.	NP_00127	XP_00671	protein_co	ENSG0000	51166	17929
AAED1	7.5649	7.00093	8.63785	6.58897	8.15617	7.29128	AhpC/TSA antioxidant enzyme domain containing 1 [Source:HGN	chr9:99401	NM_15369	XM_00525	.	.	NP_71454	XP_00525	protein_co	ENSG0000	195827	16881
AAGAB	40.4307	41.5295	42.8756	47.4052	45.4781	46.3735	alpha- and gamma-adaptin binding protein [Source:HGNC Symbo	chr15:674	NM_00127	XM_01152	.	.	NP_00125	XP_01152	protein_co	ENSG0000	79719	25662
AAK1	2.95471	2.92932	2.90223	1.45168	1.61639	1.92291	AP2 associated kinase 1 [Source:HGNC Symbol;Acc:HGNC:19679]	chr2:6968	NM_01491	.	.	.	NP_05572	.	protein_co	ENSG0000	22848	19679
AAMDC	8.23566	10.5891	6.7416	11.2327	7.33941	12.1782	adipogenesis associated Mth938 domain containing [Source:HGN	chr11:7753	NM_00131	XM_01154	.	.	NP_00130	XP_00527	protein_co	ENSG0000	28971	30205
AAMP	92.2091	94.2251	79.4299	106.624	90.9083	70.2651	angio associated migratory cell protein [Source:HGNC Symbol;Ac	chr2:21912	NM_00108	.	.	.	NP_00107	.	protein_co	ENSG0000	14	18
AANAT	0.24984	0	0.095223	0.119581	0	0.250295	aralkylamine N-acetyltransferase [Source:HGNC Symbol;Acc:HGN	chr17:744	NM_00108	XM_01152	.	.	NP_00107	XP_01152	protein_co	ENSG0000	15	19
AAR2	24.5701	24.0277	21.1824	27.2094	21.8848	18.1098	AAR2 splicing factor homolog [Source:HGNC Symbol;Acc:HGNC:1	chr20:3482	NM_00127	XM_01152	.	.	NP_00125	XP_00672	protein_co	ENSG0000	25980	15886
AARD	0	0	0	0	0	0	alanine and arginine rich domain containing protein [Source:HGN	chr8:1179	NM_00102	.	.	.	NP_00102	.	protein_co	ENSG0000	441376	33842
AARS	83.0899	88.505	54.6942	72.2496	65.7216	149.444	alanyl-tRNA synthetase [Source:HGNC Symbol;Acc:HGNC:20]	chr16:7028	NM_0016	.	.	.	NP_00159	.	protein_co	ENSG0000	16	20
AARS2	6.65889	6.96981	6.29575	5.97773	5.90678	3.65828	alanyl-tRNA synthetase 2, mitochondrial [Source:HGNC Symbol;A	chr6:4423	NM_02074	XM_01151	.	.	NP_06579	XP_00524	protein_co	ENSG0000	57505	21022
AARSD1	31.9996	30.6891	24.6546	41.0968	36.3349	23.8351	alanyl-tRNA synthetase domain containing 1 [Source:HGNC Symb	chr17:411	NM_00126	.	.	.	NP_00124	.	protein_co	ENSG0000	80755	28417
AASDH	4.88116	5.44498	6.28085	3.68406	4.02539	5.74651	aminoadipate-semialdehyde dehydrogenase [Source:HGNC Symb	chr4:57204	NM_00128	XM_01153	.	.	NP_00127	XP_00526	protein_co	ENSG0000	132949	23993
AASDHPPT	37.4594	38.3306	36.6411	34.917	45.0911	42.9293	aminoadipate-semialdehyde dehydrogenase-phosphopantethein	chr11:105	NM_01542	.	.	.	NP_05623	.	protein_co	ENSG0000	60496	14235
AASS	1.77004	1.80387	2.44173	1.3868	1.52077	2.13717	aminoadipate-semialdehyde synthase [Source:HGNC Symbol;Acc	chr7:1217	NM_00576	XM_01151	.	.	NP_00575	XP_01151	protein_co	ENSG0000	10157	17366
AATF	50.2124	51.1789	44.7588	53.5426	47.6714	39.4198	apoptosis antagonizing transcription factor [Source:HGNC Symbo	chr17:353	NM_01213	XM_01152	.	.	NP_03627	XP_01152	protein_co	ENSG0000	26574	19235
AATK	1.03003	1.2893	1.3085	0.768539	1.03937	1.00941	apoptosis associated tyrosine kinase [Source:HGNC Symbol;Acc:H	chr17:790	NM_00108	XM_01702	.	.	NP_00107	XP_00672	protein_co	ENSG0000	9625	21
AATK-AS1	0	0	0	0	0	0	AATK antisense RNA 1 [Source:HGNC Symbol;Acc:HGNC:40053]	chr17:790	.	.	NR_02725	.	.	.	protein_co	ENSG0000	388428	40053
ABAT	1.72279	2.2278	2.80507	2.39972	2.51205	2.45927	4-aminobutyrate aminotransferase [Source:HGNC Symbol;Acc:HG	chr16:876	NM_00066	XM_01152	.	.	NP_00065	XP_01152	protein_co	ENSG0000	18	23
ABC7-42404400C24.1	9.69E-09	8.49E-08	0.030503	0.077778	1.65E-08	1.06E-09	.	chr10:469	.	XM_01701	.	.	.	XP_00671	protein_co	ENSG0000	83849, 102	.
ABCA1	0.374337	0.286603	0.521352	0.007456	0.007516	0.006774	ATP binding cassette subfamily A member 1 [Source:HGNC Symb	chr9:1075	NM_00550	XM_01151	.	.	NP_00549	XP_00525	protein_co	ENSG0000	19	29
ABCA10	0.061754	0.093787	0.086264	0.028532	0.013562	0.058935	ATP binding cassette subfamily A member 10 [Source:HGNC Sym	chr17:671	NM_08028	.	.	.	NP_52502	.	protein_co	ENSG0000	10349	30
ABCA12	0.757806	0.904821	0.855142	0.630021	0.989393	1.32751	ATP binding cassette subfamily A member 12 [Source:HGNC Sym	chr2:2157	NM_01565	XM_01151	.	.	NP_05647	XP_01150	protein_co	ENSG0000	26154	14637
ABCA13	0	0.004925	0.003991	0.005244	0	0	ATP binding cassette subfamily A member 13 [Source:HGNC Sym	chr7:4821	NM_1527	XM_01151	.	.	NP_68991	XP_01151	protein_co	ENSG0000	154664	14638
ABCA2	17.1147	18.586	27.0024	15.3261	14.9731	15.9671	ATP binding cassette subfamily A member 2 [Source:HGNC Symb	chr9:1399	NM_0016	XM_01151	.	XR_00174	NP_00159	XP_00671	protein_co	ENSG0000	20	32
ABCA3	2.1218	3.20118	3.9963	1.82067	1.55872	2.29791	ATP binding cassette subfamily A member 3 [Source:HGNC Symb	chr16:232	NM_00108	.	.	.	NP_00108	.	protein_co	ENSG0000	21	33
ABCA4	0.055155	0.037197	0.206604	0.140846	0.146784	0.077734	ATP binding cassette subfamily A member 4 [Source:HGNC Symb	chr1:9445	NM_00035	.	.	.	NP_00034	.	protein_co	ENSG0000	24	34
ABCA5	1.53251	1.57418	1.96595	1.22415	1.59674	2.28664	ATP binding cassette subfamily A member 5 [Source:HGNC Symb	chr17:671	NM_01867	.	.	.	NP_06114	.	protein_co	ENSG0000	23461	35
ABCA6	0.014418	0.062244	0.025158	0	0.015821	0.10128	ATP binding cassette subfamily A member 6 [Source:HGNC Symb	chr17:670	NM_08028	XM_01702	.	.	NP_52502	XP_01152	protein_co	ENSG0000	23460	36
ABCA7	10.1438	10.7008	14.543	8.32842	7.36934	7.67579	ATP binding cassette subfamily A member 7 [Source:HGNC Symb	chr19:104	NM_01911	XM_01152	.	.	NP_06198	XP_00672	protein_co	ENSG0000	10347	37
ABCA8	0	0	0	0	0	0	ATP binding cassette subfamily A member 8 [Source:HGNC Symb	chr17:668	NM_00128	XM_01152	.	.	NP_00127	XP_00525	protein_co	ENSG0000	10351	38
ABCA9	0.011848	0.025195	0	0	0	0.023355	ATP binding cassette subfamily A member 9 [Source:HGNC Symb	chr17:669	NM_08028	XM_01702	.	.	NP_52502	XP_01152	protein_co	ENSG0000	10350	39
ABCB1	0.83012	1.18903	1.22091	1.75153	1.3666	1.71693	ATP binding cassette subfamily B member 1 [Source:HGNC Symb	chr7:8713	NM_00092	.	.	.	NP_00091	.	protein_co	ENSG0000	5243	40
ABCB10	8.2053	9.47316	8.69251	10.874	9.12956	8.64813	ATP binding cassette subfamily B member 10 [Source:HGNC Sym	chr1:2296	NM_01208	XM_01154	.	.	NP_03622	XP_01154	protein_co	ENSG0000	23456	41
ABCB11	0	0	0	0	0	0	ATP binding cassette subfamily B member 11 [Source:HGNC Sym	chr2:1697	NM_00374	XM_01151	.	.	NP_00373	XP_00671	protein_co	ENSG0000	8647	42
ABCB4	0	0.019242	0.015934	0.020571	0	0.018542	ATP binding cassette subfamily B member 4 [Source:HGNC Symb	chr7:8703	NM_00044	XM_01151	.	.	NP_00043	XP_01151	protein_co	ENSG0000	5244	45
ABCB5	0.012842	0	0.011177	0	0.028463	0	ATP binding cassette subfamily B member 5 [Source:HGNC Symb	chr7:2065	NM_00116	XM_01151	.	.	NP_00115	XP_01151	protein_co	ENSG0000	340273	46
ABCB6	7.86523	7.19947	7.85593	4.63857	4.39892	4.64576	ATP binding cassette subfamily B member 6 (Langereis blood gro	chr2:2200	NM_00568	.	.	.	NP_00568	.	protein_co	ENSG0000	10058	47
ABCB7	9.56218	8.99562	11.8415	8.11364	9.63499	10.1892	ATP binding cassette subfamily B member 7 [Source:HGNC Symb	chrX:7427	NM_00127	.	.	.	NP_00125	.	protein_co	ENSG0000	22	48
ABCB8	20.9172	20.6733	17.8252	19.4087	17.2541	14.8139	ATP binding cassette subfamily B member 8 [Source:HGNC Symb	chr7:1507	NM_00128	.	.	.	NP_00126	.	protein_co	ENSG0000	11194	49
ABCB9	4.27419	4.69374	4.76872	3.56556	3.58683	3.14655	ATP binding cassette subfamily B member 9 [Source:HGNC Symb	chr12:123	NM_00124	XM_01701	.	.	NP_00122	XP_00525	protein_co	ENSG0000	23457	50
ABCC1	18.7867	19.5997	21.0364	19.2557	18.6804	19.7543	ATP binding cassette subfamily C member 1 [Source:HGNC Symb	chr16:160	NM_00499	XM_01702	.	.	NP_00498	XP_01152	protein_co	ENSG0000	4363	51
ABCC10	7.08173	7.3925	7.83759	5.66048	6.14483	4.91167	ATP binding cassette subfamily C member 10 [Source:HGNC Sym	chr6:4339	NM_00119	XM_01701	.	.	NP_00118	XP_01151	protein_co	ENSG0000	89845	52

ABCC11	0.044898	0.030581	0.025912	0	0.05116	0.078584	ATP binding cassette subfamily C member 11 [Source:HGNC Symb	chr16:4820	NM_03258	XM_01702	.	.	NP_11597	XP_011521	protein_co	ENSG0000	85320	14639
ABCC12	0.012767	0	0.011079	0	0.014444	0.013175	ATP binding cassette subfamily C member 12 [Source:HGNC Symb	chr16:4811	NM_03322	.	.	.	NP_15022	.	protein_co	ENSG0000	94160	14640
ABCC2	14.046	12.5137	13.6564	45.8952	46.911	49.3403	ATP binding cassette subfamily C member 2 [Source:HGNC Symbo	chr10:1015	NM_00039	.	.	.	NP_00038	.	protein_co	ENSG0000	1244	53
ABCC3	4.7867	4.63481	8.04884	2.70233	2.98015	4.48812	ATP binding cassette subfamily C member 3 [Source:HGNC Symbo	chr17:4871	NM_00114	XM_01152	.	.	NP_00113	XP_005257	protein_co	ENSG0000	8714	54
ABCC4	9.73186	10.104	11.1228	10.0164	12.3684	11.6384	ATP binding cassette subfamily C member 4 [Source:HGNC Symbo	chr13:9567	NM_00110	XM_01702	.	.	NP_00109	XP_005254	protein_co	ENSG0000	10257	55
ABCC5	10.1047	9.5562	9.9481	7.56939	7.96408	6.51047	ATP binding cassette subfamily C member 5 [Source:HGNC Symbo	chr3:18363	NM_00102	XM_01700	.	.	NP_00101	XP_005247	protein_co	ENSG0000	10057	56
ABCC6	0.788705	1.37159	0.795745	0.683308	1.10751	0.132092	ATP binding cassette subfamily C member 6 [Source:HGNC Symbo	chr16:1624	NM_00107	XM_01152	.	.	NP_00107	XP_011520	protein_co	ENSG0000	105369239	57
ABCC8	0	0	0.024127	0	0.016219	0.015286	ATP binding cassette subfamily C member 8 [Source:HGNC Symbo	chr11:1741	NM_00035	XM_01701	.	.	NP_00034	XP_011518	protein_co	ENSG0000	6833	59
ABCC9	0	0.047474	0	0	0.016561	0	ATP binding cassette subfamily C member 9 [Source:HGNC Symbo	chr12:2195	NM_00569	XM_01152	.	.	NP_00568	XP_005253	protein_co	ENSG0000	10060	60
ABCD1	3.63872	3.91099	4.34637	4.15667	2.92066	3.4373	ATP binding cassette subfamily D member 1 [Source:HGNC Symbo	chrX:15299	NM_00003	.	.	.	NP_00002	.	protein_co	ENSG0000	215	61
ABCD2	0.014496	0.015872	0.012597	0.016831	0.031718	0.013661	ATP binding cassette subfamily D member 2 [Source:HGNC Symbo	chr12:3994	NM_00516	XM_01153	.	.	NP_00515	XP_011536	protein_co	ENSG0000	225	66
ABCD3	21.3344	18.2476	23.0128	19.1904	26.5183	26.399	ATP binding cassette subfamily D member 3 [Source:HGNC Symbo	chr1:94884	NM_00112	XM_01700	.	.	NP_00111	XP_006710	protein_co	ENSG0000	5825	67
ABCD4	10.2828	10.4617	9.86624	11.5835	11.206	9.17097	ATP binding cassette subfamily D member 4 [Source:HGNC Symbo	chr14:7475	NM_00505	XM_01153	.	.	NP_00504	XP_005267	protein_co	ENSG0000	5826	68
ABCE1	59.5834	55.971	58.8868	56.9617	72.9008	67.1731	ATP binding cassette subfamily E member 1 [Source:HGNC Symbo	chr4:14591	NM_00104	.	.	.	NP_00103	.	protein_co	ENSG0000	6059	69
ABCF1	28.5006	30.2555	25.5394	31.4603	27.5329	23.3034	ATP binding cassette subfamily F member 1 [Source:HGNC Symbo	chr6:30539	NM_00102	.	.	.	NP_00102	.	protein_co	ENSG0000	23	70
ABCF2	44.2659	48.0255	38.2928	47.5277	42.9703	31.7543	ATP binding cassette subfamily F member 2 [Source:HGNC Symbo	chr7:15090	NM_00569	XM_00671	.	.	NP_00568	XP_005249	protein_co	ENSG0000	10061	71
ABCF3	30.3457	31.8121	26.1559	32.0986	30.8998	23.7496	ATP binding cassette subfamily F member 3 [Source:HGNC Symbo	chr3:18390	NM_01835	XM_01151	.	.	NP_06082	XP_011511	protein_co	ENSG0000	55324	72
ABCG1	0.650622	0.654129	0.827374	0.685305	0.385344	0.663063	ATP binding cassette subfamily G member 1 [Source:HGNC Symbo	chr21:4361	NM_00491	XM_01152	.	.	NP_00490	XP_011528	protein_co	ENSG0000	9619	73
ABCG2	1.04719	0.904405	0.98829	0.837283	1.04764	0.941705	ATP binding cassette subfamily G member 2 (Junior blood group)	chr4:89011	NM_00125	XM_01153	.	.	NP_00124	XP_005263	protein_co	ENSG0000	9429	74
ABCG4	1.38912	1.21085	1.46523	1.62975	1.30947	1.5189	ATP binding cassette subfamily G member 4 [Source:HGNC Symbo	chr11:1190	NM_00114	XM_01154	.	.	NP_00113	XP_011541	protein_co	ENSG0000	64137	13884
ABCG5	0	0	0	0	0	0	ATP binding cassette subfamily G member 5 [Source:HGNC Symbo	chr2:44039	NM_02243	XM_01153	.	.	NP_07188	XP_005264	protein_co	ENSG0000	64240	13886
ABCG8	0	0	0	0	0	0	ATP binding cassette subfamily G member 8 [Source:HGNC Symbo	chr2:44066	NM_02243	XM_01153	.	.	NP_07188	XP_005264	protein_co	ENSG0000	64241	13887
ABHD1	0	0	0	0	0	0	abhydrolase domain containing 1 [Source:HGNC Symbol;Acc:HGN	chr2:27346	NM_03260	XM_01153	.	.	NP_11599	XP_011531	protein_co	ENSG0000	84696	17553
ABHD10	103.325	99.253	135.492	104.66	107.663	112.609	abhydrolase domain containing 10 [Source:HGNC Symbol;Acc:HG	chr3:11169	NM_00127	XM_01151	.	.	NP_00125	XP_011511	protein_co	ENSG0000	55347	25656
ABHD11	25.2343	24.7562	26.8277	24.5409	20.096	21.9229	abhydrolase domain containing 11 [Source:HGNC Symbol;Acc:HG	chr7:73150	NM_00114	XM_01151	NR_02691	.	NP_00113	XP_011514	protein_co	ENSG0000	83451	16407
ABHD12	16.9443	19.4969	15.0243	18.9195	15.8671	17.3723	abhydrolase domain containing 12 [Source:HGNC Symbol;Acc:HG	chr20:2522	NM_00104	XM_01152	.	.	NP_00103	XP_011527	protein_co	ENSG0000	26090	15868
ABHD12B	0	0	0	0	0	0	abhydrolase domain containing 12B [Source:HGNC Symbol;Acc:H	chr14:5132	NM_00120	XM_01153	.	.	NP_00119	XP_011534	protein_co	ENSG0000	145447	19837
ABHD13	3.33256	4.0983	3.58535	4.35285	4.39979	4.96528	abhydrolase domain containing 13 [Source:HGNC Symbol;Acc:HG	chr13:1088	NM_03285	XM_01152	.	.	NP_11624	XP_011519	protein_co	ENSG0000	84945	20293
ABHD14A	9.74317	10.9948	8.18018	11.9487	8.44448	5.81954	abhydrolase domain containing 14A [Source:HGNC Symbol;Acc:H	chr3:52002	NM_0154	.	.	.	NP_05622	.	protein_co	ENSG0000	25864	24538
ABHD14A-ACY1	1.82E-16	0.000904	0.000382	2.56E-13	6.59E-05	0.176399	ABHD14A-ACY1 readthrough [Source:HGNC Symbol;Acc:HGNC:38	chr3:52002	NM_00131	.	.	.	NP_00130	.	protein_co	ENSG0000	100526760	38856
ABHD14B	61.9407	62.9576	60.6856	59.5356	52.8489	41.2171	abhydrolase domain containing 14B [Source:HGNC Symbol;Acc:H	chr3:52002	NM_00114	XM_01153	.	.	NP_00113	XP_011532	protein_co	ENSG0000	84836	28235
ABHD15	6.76471	6.00232	7.35591	4.58573	4.44965	4.55899	abhydrolase domain containing 15 [Source:HGNC Symbol;Acc:HG	chr17:2788	NM_19814	.	.	.	NP_93779	.	protein_co	ENSG0000	116236	26971
ABHD16A	16.0538	15.6347	13.5243	15.8401	12.1485	13.013	abhydrolase domain containing 16A [Source:HGNC Symbol;Acc:H	chr6:31654	NM_00117	.	.	.	NP_00117	.	protein_co	ENSG0000	7920	13921
ABHD16B	0	0	0	0.097559	0	0	abhydrolase domain containing 16B [Source:HGNC Symbol;Acc:H	chr20:6249	NM_08062	.	.	.	NP_54218	.	protein_co	ENSG0000	140701	16128
ABHD17A	29.6388	33.7976	34.7248	39.1087	31.483	36.6876	abhydrolase domain containing 17A [Source:HGNC Symbol;Acc:H	chr19:1876	NM_00113	XM_01152	.	.	NP_00112	XP_011526	protein_co	ENSG0000	81926	28756
ABHD17B	11.0593	10.8299	11.7301	13.1788	13.2892	11.6171	abhydrolase domain containing 17B [Source:HGNC Symbol;Acc:H	chr9:74477	NM_00102	XM_01701	.	.	NP_00102	XP_006717	protein_co	ENSG0000	51104	24278
ABHD17C	21.2353	24.4815	24.1397	27.9851	32.312	28.3959	abhydrolase domain containing 17C [Source:HGNC Symbol;Acc:H	chr15:8097	NM_02121	.	.	.	NP_06703	.	protein_co	ENSG0000	58489	26925
ABHD18	4.26254	3.43553	4.15067	2.55874	3.4447	4.42664	abhydrolase domain containing 18 [Source:HGNC Symbol;Acc:HG	chr4:12883	NM_00103	XM_01153	.	.	NP_00103	XP_005263	protein_co	ENSG0000	80167	26111
ABHD2	12.5464	12.794	9.95299	10.527	12.4104	12.9818	abhydrolase domain containing 2 [Source:HGNC Symbol;Acc:HGN	chr15:8963	NM_00701	XM_01152	.	.	NP_00894	XP_005254	protein_co	ENSG0000	11057	18717
ABHD3	22.8378	23.7769	22.3126	27.4256	26.5695	25.9564	abhydrolase domain containing 3 [Source:HGNC Symbol;Acc:HGN	chr18:1923	NM_00130	XM_01152	.	.	NP_00129	XP_011524	protein_co	ENSG0000	171586	18718
ABHD4	6.34627	6.84859	5.63136	4.15607	4.09436	4.40401	abhydrolase domain containing 4 [Source:HGNC Symbol;Acc:HGN	chr14:2306	NM_02206	.	.	.	NP_07134	XP_005268	protein_co	ENSG0000	63874	20154
ABHD5	11.3124	11.6407	9.26298	12.0843	12.2961	9.72729	abhydrolase domain containing 5 [Source:HGNC Symbol;Acc:HGN	chr3:43732	NM_01600	XM_01153	.	.	NP_05709	XP_011532	protein_co	ENSG0000	51099	21396
ABHD6	6.37772	5.56475	7.19807	8.83758	6.96964	7.6615	abhydrolase domain containing 6 [Source:HGNC Symbol;Acc:HGN	chr3:58223	NM_00132	XM_00526	.	.	NP_00130	XP_005265	protein_co	ENSG0000	57406	21398
ABHD8	2.53314	3.27939	3.75529	3.28498	3.12783	5.00617	abhydrolase domain containing 8 [Source:HGNC Symbol;Acc:HGN	chr19:1740	NM_02452	.	.	.	NP_07880	.	protein_co	ENSG0000	79575	23759
ABI1	15.6435	13.5272	14.4053	12.8817	12.7244	14.1084	abl interactor 1 [Source:HGNC Symbol;Acc:HGNC:11320]	chr10:2698	NM_00101	XM_00671	.	.	NP_00101	XP_005252	protein_co	ENSG0000	10006	11320
ABI2	13.1086	13.9818	17.6979	11.567	11.5789	17.9351	abl interactor 2 [Source:HGNC Symbol;Acc:HGNC:24011]	chr2:20419	NM_00128	XM_01700	.	.	NP_00126	XP_005246	protein_co	ENSG0000	10152	24011
ABI3	0.046168	0.045094	0.063034	0	0	0.040458	ABI family member 3 [Source:HGNC Symbol;Acc:HGNC:29859]	chr17:4728	NM_00113	XM_01152	.	.	NP_00112	XP_005257	protein_co	ENSG0000	51225	29859
ABI3BP	0.031077	0.049493	0.05406	0.087661	0.085666	0.030808	ABI family member 3 binding protein [Source:HGNC Symbol;Acc:H	chr3:10046	NM_01542	XM_01151	.	.	NP_05624	XP_005247	protein_co	ENSG0000	25890	17265
ABL1	18.8129	20.5644	19.3685	19.396	18.7034	16.4743	ABL proto-oncogene 1, non-receptor tyrosine kinase [Source:HGN	chr9:13358	NM_00515	.	.	.	NP_00514	.	protein_co	ENSG0000	25	76
ABL2	5.80772	6.20951	5.84252	4.74801	5.67046	5.92615	ABL proto-oncogene 2, non-receptor tyrosine kinase [Source:HGN	chr1:17906	NM_00113	XM_00524	.	.	NP_00112	XP_005245	protein_co	ENSG0000	27	77
ABLIM1	32.0918	33.7533	46.4273	28.5652	34.628	35.8955	actin binding LIM protein 1 [Source:HGNC Symbol;Acc:HGNC:78]	chr10:1161	NM_00100	XM_01701	.	.	NP_00100	XP_005269	protein_co	ENSG0000	3983	78
ABLIM2	1.52536	1.61783	1.7361	1.60572	1.50541	1.30979	actin binding LIM protein family member 2 [Source:HGNC Symbo	chr4:79670	NM_00113	XM_01700	.	.	NP_00112	XP_005248	protein_co	ENSG0000	84448	19195
ABLIM3	5.06155	6.09768	5.98467	4.91974	4.95592	4.5213	actin binding LIM protein family member 3 [Source:HGNC Symbo	chr5:14852	NM_00130	XM_01700	.	.	NP_00128	XP_005268	protein_co	ENSG0000	22885	29132
ABO	0	0.097893	0.084509	0.21221	0.228399	0.164366	ABO blood group (transferase A, alpha 1-3-N-acetylgalactosamin	chr9:13613	NM_02046	.	.	.	NP_06520	.	protein_co	ENSG0000	28	79
ABR	18.7894	20.1244	19.0401	12.8321	12.0967	13.0258	active BCR-related [Source:HGNC Symbol;Acc:HGNC:81]	chr17:9003	NM_00109	XM_01152	.	.	NP_00108	XP_011522	protein_co	ENSG0000	29	81
ABRA	0.025626	0	0	0	0	0	actin binding Rho activating protein [Source:HGNC Symbol;Acc:H	chr8:10777	NM_13916	.	.	.	NP_63190	.	protein_co	ENSG0000	137735	30655
ABRACL	67.5801	64.912	72.2614	79.0224	72.411	87.1553	ABRA C-terminal like [Source:HGNC Symbol;Acc:HGNC:21230]	chr6:13934	NM_02124	.	.	.	NP_06706	XP_016866	protein_co	ENSG0000	58527	21230
ABT1	25.4703	26.9341	23.6093	26.5282	22.2225	16.5237	activator of basal transcription 1 [Source:HGNC Symbol;Acc:HGN	chr6:26597	NM_01337	.	.	.	NP_03750	.	protein_co	ENSG0000	29777	17369
ABTB1	6.24594	6.02615	6.8271	3.66838	2.59331	5.01877	ankyrin repeat and BTB domain containing 1 [Source:HGNC Symb	chr3:12739	NM_03254	XM_01700	.	.	NP_11593	XP_006713	protein_co	ENSG0000	80325	18275

ABTB2	4.35104	4.88672	4.41866	3.75213	3.20684	4.17114	ankyrin repeat and BTB domain containing 2 [Source:HGNC Symb	chr11:3417	NM_14580	.	.	.	NP_66580	.	protein_co	ENSG0000	25841	23842
AC000003.2	0	0	0	0	0	0	CDNA FLJ25865 fis, clone CBR01927 [Source:UniProtKB/TrEMBL;	chr17:9813	.	.	.	.	.	.	protein_co	ENSG0000	.	.
AC002310.13	1.59E-08	1.27393	0.030306	2.32E-38	4.11E-05	4.15E-41	.	chr16:3054	.	.	.	.	.	.	protein_co	ENSG0000	.	.
AC002429.5	0	0	0	0	0	0	.	chr7:94536	.	.	.	.	.	.	protein_co	ENSG0000	.	.
AC002451.1	0	0	0	0	0	0	Protein LOC100996577 [Source:UniProtKB/TrEMBL;Acc:M0R3I6]	chr7:95171	.	.	.	.	.	.	protein_co	ENSG0000	.	.
AC002472.1	0	0	0	0	0	0	Metazoan signal recognition particle RNA [Source:RFAM;Acc:RF0	chr22:2135	.	.	.	.	.	.	misc_RNA	ENSG0000	.	.
AC003002.4	2.94E-62	1.7E-105	3.95E-52	1.6E-301	1.44E-57	4.3E-161	.	chr19:5787	.	.	.	.	.	.	protein_co	ENSG0000	.	.
AC003002.6	7.68E-46	3.46E-54	8.58E-63	1.03E-80	2.6E-45	1.81E-40	.	chr19:5787	.	.	.	.	.	.	protein_co	ENSG0000	.	.
AC003006.7	3.71E-07	2.3E-06	3.89E-05	6.49E-07	2.18E-05	0.269716	.	chr19:5819	.	.	.	.	.	.	protein_co	ENSG0000	.	.
AC003043.1	0	0	0	0	0	0	.	chr17:4232	.	.	.	.	.	.	protein_co	ENSG0000	.	.
AC004017.1	0	0	0	0	0	0	Uncharacterized protein [Source:UniProtKB/TrEMBL;Acc:M0R3E	chr19:5819	.	.	.	.	.	.	protein_co	ENSG0000	.	.
AC004076.7	5.39963	4.55509	2.65644	5.98184	5.33182	4.4944	.	chr19:5787	.	.	.	.	.	.	protein_co	ENSG0000	.	.
AC004076.9	0	0	6.07E-80	0	0	0	.	chr19:5787	.	.	.	.	.	.	protein_co	ENSG0000	.	.
AC004381.6	4.24236	3.72393	3.61461	3.99824	3.77439	3.78484	Putative RNA exonuclease NEF-sp [Source:UniProtKB/Swiss-Prot;	chr16:2077	NM_00114	XM_01702	.	.	NP_00113	XP_005255	protein_co	ENSG0000	81691	.
AC004528.1	0	0	0	0	0	0	Uncharacterized protein [Source:UniProtKB/TrEMBL;Acc:M0QY0	chr19:9996	.	.	.	.	.	.	protein_co	ENSG0000	.	.
AC004754.3	0.121286	0.123168	4.93E-58	0.121379	1.69E-16	5.74E-18	.	chr16:2845	.	.	.	.	.	.	protein_co	ENSG0000	.	.
AC004817.1	0	0	0	0	0	0	HCG2028611; Uncharacterized protein [Source:UniProtKB/TrEM	chr14:7168	.	.	.	.	.	.	protein_co	ENSG0000	.	.
AC004824.2	0	0	0	0	0	0	Uncharacterized protein [Source:UniProtKB/TrEMBL;Acc:M0QXB	chr1:17634	.	.	.	.	.	.	protein_co	ENSG0000	.	.
AC004899.1	0	0	0	0	0	0	Uncharacterized protein [Source:UniProtKB/TrEMBL;Acc:M0R02	chr7:48887	.	.	.	.	.	.	protein_co	ENSG0000	.	.
AC005082.1	0	0.91289	0	0	0	0	Uncharacterized protein [Source:UniProtKB/TrEMBL;Acc:H7BYU	chr7:23145	.	.	.	.	.	.	protein_co	ENSG0000	.	.
AC005281.1	0	0	0	0.044664	0	0	.	chr7:12522	.	XM_01151	NR_13626	.	.	XP_011513	protein_co	ENSG0000	102725191	.
AC005358.1	0	0.073833	0	0	0	0	.	chr17:1256	NM_00114	.	.	.	NP_00113	.	protein_co	ENSG0000	.	.
AC005477.1	0	0	0	0	0	0	.	chr14:7239	.	.	.	.	.	.	protein_co	ENSG0000	.	.
AC005488.1	0	0	0	0	0	0	Uncharacterized protein [Source:UniProtKB/TrEMBL;Acc:M0R18	chr7:72459	.	.	.	.	.	.	protein_co	ENSG0000	.	.
AC005493.1	0	0	0	0	0	0	Uncharacterized protein [Source:UniProtKB/TrEMBL;Acc:H7BYU	chr7:34685	.	.	.	.	.	.	protein_co	ENSG0000	.	.
AC005544.1	0	0	0	0	0	0	Uncharacterized protein [Source:UniProtKB/TrEMBL;Acc:H0Y3C3	chr17:6496	.	.	.	.	.	.	protein_co	ENSG0000	10192930	.
AC005549.3	0	0	0	0	0	0	Uncharacterized protein [Source:UniProtKB/TrEMBL;Acc:M0R0Z	chr17:3258	.	.	.	.	.	.	protein_co	ENSG0000	.	.
AC005606.1	5.62E-63	8E-187	1.4E-102	0	5.3E-122	3.77E-89	LOC283868 protein; Uncharacterized protein [Source:UniProtKB/	chr16:2022	.	.	.	.	.	.	protein_co	ENSG0000	.	.
AC005609.1	0	0	0	0	0	0	Uncharacterized protein; cDNA FLJ34090 fis, clone FCBBF300639	chr5:14016	.	.	.	.	.	.	protein_co	ENSG0000	.	.
AC005841.1	0	0	0	0	0	0	Uncharacterized protein ENSP00000372125 [Source:UniProtKB/S	chr12:2958	.	XM_00525	.	.	.	XP_00527	protein_co	ENSG0000	10192946	.
AC006014.1	0	0	0	0	0	0	Uncharacterized protein [Source:UniProtKB/TrEMBL;Acc:M0R18	chr7:74999	.	.	.	.	.	.	protein_co	ENSG0000	.	.
AC006116.20	0	0	0	0	0	0	.	chr19:5673	.	.	.	.	.	.	protein_co	ENSG0000	.	.
AC006372.1	0	0	0	0	0.052354	0.04841	.	chr7:15731	.	.	.	.	.	.	protein_co	ENSG0000	.	.
AC006435.1	0.158904	0	0	0.516962	0.363119	0.169634	Uncharacterized protein [Source:UniProtKB/TrEMBL;Acc:Q4G0H	chr17:2310	.	.	.	.	.	.	protein_co	ENSG0000	.	.
AC006449.1	0.845512	0.284181	0	0.304474	0	0	NS5ATP13TP1; Uncharacterized protein [Source:UniProtKB/TrEM	chr17:3686	.	.	.	.	.	.	protein_co	ENSG0000	.	.
AC006486.1	0.206677	0.125391	0.142156	0.090759	0.141588	0.0435	.	chr19:4273	.	.	.	.	.	.	protein_co	ENSG0000	.	.
AC006486.9	9.15E-06	9.94E-06	2.21E-05	1.31E-05	0.129095	0.000181	.	chr19:4273	.	.	.	.	.	.	protein_co	ENSG0000	.	.
AC006538.4	0.384176	1.7E-30	0.721101	0.535831	0.000239	1.58E-23	.	chr19:2714	.	.	.	.	.	.	protein_co	ENSG0000	.	.
AC007040.11	0.650778	0.746953	0.720655	0.701293	0.592447	0.504297	.	chr2:71163	.	.	.	.	.	.	protein_co	ENSG0000	.	.
AC007192.4	0.00011	0.33	0.000363	0.16654	1.19488	0.000138	Phosphatidylinositol 3-kinase regulatory subunit beta [Source:Ur	chr19:1826	.	.	.	.	.	.	protein_co	ENSG0000	.	.
AC007204.1	0	0	0	0	0	0	.	chr19:2004	.	.	.	.	.	.	unprocesse	ENSG0000	.	.
AC007216.2	0	0	0	0	0	0	Uncharacterized protein [Source:UniProtKB/TrEMBL;Acc:M0QYP	chr16:1206	.	.	.	.	.	.	protein_co	ENSG0000	.	.
AC007377.1	0	0	0	0	0	0	.	chr2:40324	.	.	.	.	.	.	protein_co	ENSG0000	.	.
AC007382.1	0	0	0	0	0	0	Uncharacterized protein [Source:UniProtKB/TrEMBL;Acc:M0QZ8	chr2:37068	.	.	.	.	.	.	protein_co	ENSG0000	.	.
AC007421.1	0	0	0	0	0	0	.	chr17:7486	.	.	.	.	.	.	protein_co	ENSG0000	.	.
AC007461.1	0	0	0	0	0	0	Uncharacterized protein [Source:UniProtKB/TrEMBL;Acc:Q8IVH9	chr17:7003	.	.	.	.	.	.	protein_co	ENSG0000	.	.
AC007773.3	0	0	0	0	0	0	Uncharacterized protein [Source:UniProtKB/TrEMBL;Acc:M0QXJ	chr19:3289	.	.	.	.	.	.	protein_co	ENSG0000	.	.
AC007919.2	0	0	0	0	0	0	HCG1787166; PRO1163; Uncharacterized protein [Source:UniPro	chr3:17234	.	.	.	.	.	.	protein_co	ENSG0000	.	.
AC007952.1	0	0	0	0	0	0	Uncharacterized protein [Source:UniProtKB/TrEMBL;Acc:H7C2L0	chr17:1903	.	.	.	.	.	.	protein_co	ENSG0000	.	.
AC007956.1	1.99E-08	4.06E-08	0.847244	5.01E-08	0.409626	5.63E-08	Full-length cDNA 5-PRIME end of clone CS0CAP004YO05 of Thym	chr14:7512	.	.	.	.	.	.	protein_co	ENSG0000	.	.
AC007965.1	0	0	0	0	0	0	Uncharacterized protein [Source:UniProtKB/TrEMBL;Acc:M0QY5	chrY:28111	.	.	.	.	.	.	protein_co	ENSG0000	.	.
AC008132.13	0	0	0	0	0	0	.	chr22:1883	.	.	NR_13592	.	.	.	transcribed	ENSG0000	102725072	.
AC008267.1	0	0	0	0	0	0	HCG1983814; Uncharacterized protein [Source:UniProtKB/TrEM	chr7:65939	.	.	.	.	.	.	protein_co	ENSG0000	.	.
AC008372.1	0	0	0	0	0	0	.	chr14:7817	.	.	.	.	.	.	protein_co	ENSG0000	.	.
AC008387.1	0	0	0	0	0	0	.	chr5:72894	.	.	.	.	.	.	protein_co	ENSG0000	.	.
AC008498.1	0	0	0	0	0	0	.	chr5:60240	.	.	.	.	.	.	protein_co	ENSG0000	.	.
AC008686.1	0	0	0	0	0	0	Uncharacterized protein [Source:UniProtKB/TrEMBL;Acc:H7BYR1	chr19:1389	.	.	.	.	.	.	protein_co	ENSG0000	.	.
AC008914.1	0	0	0	0	0	0	Uncharacterized protein [Source:UniProtKB/TrEMBL;Acc:M0R27	chr5:55224	.	.	.	.	.	.	protein_co	ENSG0000	.	.
AC008948.1	0	0	0	0	0	0	.	chr5:10156	.	.	.	.	.	.	protein_co	ENSG0000	.	.

AC008964.1	0	0	0	0	0	0	.	chr5:39105	.	.	.	.	.	.	protein_co	ENSG0000	.	.
AC009014.3	0	0	0	0	0	0	.	chr5:13552	.	.	.	.	.	.	protein_co	ENSG0000	.	.
AC009041.2	0	0	0	0	0	0	.	chr16:1031	.	.	.	.	.	.	protein_co	ENSG0000	.	.
AC009060.1	0	0	0	0	0	0	Uncharacterized protein [Source:UniProtKB/TrEMBL;Acc:M0R2Z	chr16:7023	.	.	.	.	.	.	protein_co	ENSG0000	.	.
AC009065.1	0.232635	0.228864	0.597385	0.996054	0	0.252526	Uncharacterized protein [Source:UniProtKB/TrEMBL;Acc:H0Y5Z1	chr16:2303	.	.	.	.	.	.	protein_co	ENSG0000	.	.
AC009133.22	1.25E-06	0	0.887124	0	0.297799	0	.	chr16:2967	.	.	.	.	.	.	protein_co	ENSG0000	.	.
AC009802.1	0	0	0	0	0	0	Uncharacterized protein [Source:UniProtKB/TrEMBL;Acc:M0QZX	chr18:6157	.	.	.	.	.	.	protein_co	ENSG0000	.	.
AC009977.1	0	0	0	0	0	0	Uncharacterized protein [Source:UniProtKB/TrEMBL;Acc:M0R1T	chrY:21737	.	.	.	.	.	.	protein_co	ENSG0000	.	.
AC010327.2	0.053673	0.078607	0.046142	0.056746	0	0.060493	Uncharacterized protein; cDNA FLJ45856 fis, clone OCBBF202563	chr19:5573	.	.	.	.	.	.	protein_co	ENSG0000	.	.
AC010368.2	0	0	0	0	0	0	.	chr5:35048	.	.	.	.	.	.	protein_co	ENSG0000	.	.
AC010441.1	23.3765	21.7336	39.5835	24.7682	21.4679	25.3069	.	chr5:15015	.	.	.	.	.	.	protein_co	ENSG0000	.	.
AC010646.3	2.51E-20	5.47E-16	1.47E-06	0.127756	1.79E-16	2.59E-07	.	chr19:1734	.	.	.	.	.	.	protein_co	ENSG0000	.	.
AC010731.4	0	0	0	0	0	0	.	chr2:20750	NM_00110	.	.	.	NP_00109	.	protein_co	ENSG0000	200726	.
AC010760.1	0	0	0	0	0	0	.	chr15:2245	.	.	.	.	.	.	protein_co	ENSG0000	.	.
AC010877.1	0	0	0	0	0	0	Uncharacterized protein [Source:UniProtKB/TrEMBL;Acc:M0R23	chrY:15360	.	.	.	.	.	.	protein_co	ENSG0000	.	.
AC011298.1	0	0	0	0	0	0	HCG2013738; Uncharacterized protein [Source:UniProtKB/TrEM	chr2:24162	.	.	.	.	.	.	protein_co	ENSG0000	.	.
AC011308.1	0	0	0	0	0	0	.	chr2:15729	.	.	.	.	.	.	protein_co	ENSG0000	.	.
AC011475.1	0	0	0	0	0	0	Uncharacterized protein [Source:UniProtKB/TrEMBL;Acc:H0Y7T2	chr19:1071	.	.	.	.	.	.	protein_co	ENSG0000	.	.
AC011513.3	0	0	0	0	0	0	.	chr19:4221	.	.	.	.	.	.	protein_co	ENSG0000	.	.
AC011755.1	0	0	0	0	0	0	HCG2006742; Protein LOC100996685 [Source:UniProtKB/TrEMB	chr2:13342	.	.	.	.	.	.	protein_co	ENSG0000	.	.
AC011897.1	0	0	0	0	0	0	Uncharacterized protein [Source:UniProtKB/TrEMBL;Acc:J3QQN	chr2:14772	.	.	.	.	.	.	protein_co	ENSG0000	.	.
AC012067.1	0	0	0	0	0	0	.	chrY:48682	.	.	.	.	.	.	protein_co	ENSG0000	.	.
AC012215.1	0	0	0	0	0	0	Uncharacterized protein [Source:UniProtKB/TrEMBL;Acc:Q7Z2V2	chr8:35092	.	.	.	.	.	.	protein_co	ENSG0000	.	.
AC012360.1	0	0	0	0	0	0	.	chr2:10586	.	.	.	.	.	.	protein_co	ENSG0000	.	.
AC012414.1	0	0	0	0	0	0	Uncharacterized protein [Source:UniProtKB/TrEMBL;Acc:M0QZ1	chr15:2100	.	.	.	.	.	.	protein_co	ENSG0000	.	.
AC012485.1	0	0	0	0	0	0	Uncharacterized protein [Source:UniProtKB/TrEMBL;Acc:M0QY7	chr2:23915	.	.	.	.	.	.	protein_co	ENSG0000	.	.
AC013269.5	0	0	0	0	0	0	.	chr2:13129	.	.	.	.	.	.	protein_co	ENSG0000	.	.
AC013461.1	11.7493	12.076	12.0082	12.7105	13.6358	14.615	Mitogen-activated protein kinase kinase kinase MLT [Source:Uni	chr2:17394	NM_01665	XM_00524	.	.	NP_05773	XP_00524	protein_co	ENSG0000	51776	.
AC013468.1	0	0	0	0	0	0	.	chr2:19063	.	.	.	.	.	.	protein_co	ENSG0000	.	.
AC013469.1	0	0	0	0	0	0	.	chr2:24105	.	.	.	.	.	.	protein_co	ENSG0000	.	.
AC015660.1	0	0	0	0	0	0	HCG1993240; Serologically defined breast cancer antigen NY-BR-	chr15:1000	.	.	.	.	.	.	protein_co	ENSG0000	.	.
AC015987.2	0.263808	0.058001	0.138151	0.061286	0	0.099137	.	chr7:13561	.	.	.	.	.	.	protein_co	ENSG0000	.	.
AC015989.1	0	0	0	0	0	0	Uncharacterized protein [Source:UniProtKB/TrEMBL;Acc:H7BYV9	chr18:6038	.	.	.	.	.	.	protein_co	ENSG0000	.	.
AC015989.2	0	0	0	0	0	0	.	chr18:6038	.	.	.	.	.	.	protein_co	ENSG0000	.	.
AC016251.1	0	0	0	0	0	0	.	chr15:9683	.	.	.	.	.	.	protein_co	ENSG0000	.	.
AC016559.1	0	0	0	0	0	0	Uncharacterized protein [Source:UniProtKB/TrEMBL;Acc:Q9BUN	chr5:78523	.	.	.	.	.	.	protein_co	ENSG0000	.	.
AC016586.1	0	0.102303	0	0	0.078607	0.03706	.	chr19:4041	.	.	.	.	.	.	protein_co	ENSG0000	.	.
AC016745.1	0	0	0	0	0	0	HDCMB45P; Uncharacterized protein [Source:UniProtKB/TrEMBL	chr2:11419	.	.	.	.	.	.	protein_co	ENSG0000	.	.
AC016752.1	0	0	0	0	0	0	Uncharacterized protein [Source:UniProtKB/TrEMBL;Acc:M0QY5	chrY:25847	.	.	.	.	.	.	protein_co	ENSG0000	.	.
AC017028.1	0	0	0	0	0	0.75337	Uncharacterized protein [Source:UniProtKB/TrEMBL;Acc:E7ERG0	chr2:23996	.	.	.	.	.	.	protein_co	ENSG0000	.	.
AC017081.1	0	0	0	0	0	0	Uncharacterized protein [Source:UniProtKB/TrEMBL;Acc:M0R1D	chr2:20722	.	.	.	.	.	.	protein_co	ENSG0000	.	.
AC018445.1	0	0	0	0	0	0	Uncharacterized protein [Source:UniProtKB/TrEMBL;Acc:Q9NY04	chr18:7715	.	.	.	.	.	.	protein_co	ENSG0000	.	.
AC018470.1	0	0	0	0	0	0.09888	Uncharacterized protein FLJ46347 [Source:UniProtKB/Swiss-Prot	chr2:17519	.	.	.	.	.	.	protein_co	ENSG0000	.	.
AC018512.1	0	0	0	0	0	0	.	chr15:4403	.	.	.	.	.	.	protein_co	ENSG0000	.	.
AC018630.1	0	0	0	0	0	0	Uncharacterized protein [Source:UniProtKB/TrEMBL;Acc:M0R3A	chr12:1099	.	.	.	.	.	.	protein_co	ENSG0000	.	.
AC018755.1	0	0.05146	0.043337	0	0	0	HCG2008157; Uncharacterized protein; cDNA FLJ30403 fis, clone	chr19:5207	.	.	.	.	.	.	protein_co	ENSG0000	.	.
AC018755.18	0	0	0	0	0	0	.	chr19:5211	.	.	.	.	.	XP_016882	protein_co	ENSG0000	8778	.
AC018867.1	0	0	0	0	0	0	Uncharacterized protein [Source:UniProtKB/TrEMBL;Acc:H7BYP7	chr2:18735	.	.	.	.	.	.	protein_co	ENSG0000	.	.
AC018867.2	0	0	0	0	0	0	.	chr2:18735	.	.	.	.	.	.	protein_co	ENSG0000	.	.
AC019206.1	0	0	0	0	0	0	Uncharacterized protein [Source:UniProtKB/TrEMBL;Acc:M0QYB	chr2:70120	.	.	.	.	.	.	protein_co	ENSG0000	.	.
AC019294.1	0	0	0	0	0	0	.	chr15:7603	.	.	.	.	.	.	protein_co	ENSG0000	.	.
AC020629.1	0	0	0	0	0	0	Uncharacterized protein [Source:UniProtKB/TrEMBL;Acc:M0QZ0	chr12:4255	.	.	.	.	.	.	protein_co	ENSG0000	.	.
AC020922.1	1.28721	0.437093	0.867527	0.370189	0.711171	0.160105	Uncharacterized protein [Source:UniProtKB/TrEMBL;Acc:F5GYW	chr19:5585	.	.	.	.	.	.	protein_co	ENSG0000	.	.
AC020952.1	0	0	0	0	0	0	Uncharacterized protein [Source:UniProtKB/TrEMBL;Acc:M0QZE	chr19:3164	.	.	.	.	.	.	protein_co	ENSG0000	.	.
AC022210.1	0	0	0	0	0	0	.	chr2:85122	.	.	.	.	.	.	protein_co	ENSG0000	.	.
AC022400.2	0.631796	0	0	0.335257	0	0.355858	Uncharacterized protein; cDNA FLJ44715 fis, clone BRACE302143	chr10:7553	.	.	.	.	.	.	protein_co	ENSG0000	.	.
AC022498.1	0	0.040931	0	0	0	0	Uncharacterized protein [Source:UniProtKB/TrEMBL;Acc:E7EQ38	chr3:18787	.	.	.	.	.	.	protein_co	ENSG0000	.	.
AC022532.1	0.124718	0.25673	0.218049	0.136721	0.282295	0.743032	Uncharacterized protein [Source:UniProtKB/TrEMBL;Acc:F8WBX	chr10:7219	.	.	.	.	.	.	protein_co	ENSG0000	.	.
AC022819.2	0	0	0	0	0	0	Uncharacterized protein [Source:UniProtKB/TrEMBL;Acc:M0QXY	chr15:9979	.	.	.	.	.	.	protein_co	ENSG0000	.	.

AC023632.1	0	0	0	0	0	0	HCG2009141; PRO2397; Uncharacterized protein [Source:UniPro	chr8:95499	.	.	.	.	.	.	protein_co	ENSG00000	.	.
AC024257.1	0	0	0	0	0	0	CDNA FLJ26186 fis, clone ADG04757; Uncharacterized protein [S	chr12:4875	.	.	.	.	.	.	protein_co	ENSG00000	.	.
AC024580.1	0	0	0	0	0	0	Uncharacterized protein [Source:UniProtKB/TrEMBL;Acc:H7BYB4	chr19:5665	.	.	.	.	.	.	protein_co	ENSG00000	.	.
AC024592.12	1.56748	1.5391	2.62952	2.64569	2.42915	1.32441	.	chr19:5865	.	.	.	.	.	.	protein_co	ENSG00000	.	.
AC025262.1	0	0	0	0	0	0	Mesenchymal stem cell protein DSC96; Uncharacterized protein	chr12:6490	.	.	.	.	.	.	protein_co	ENSG00000	.	.
AC025278.1	0	0	0	0	0	0	Uncharacterized protein [Source:UniProtKB/TrEMBL;Acc:F8WD2	chr19:7011	.	.	.	.	.	.	protein_co	ENSG00000	.	.
AC025287.1	0	0	0	0	0	0	Uncharacterized protein [Source:UniProtKB/TrEMBL;Acc:H0YFN1	chr16:7572	.	.	.	.	.	.	protein_co	ENSG00000	.	.
AC025335.1	0.582001	0.371223	0.530997	0.362091	0.538545	0.753783	.	chr17:7816	.	.	.	.	.	.	protein_co	ENSG00000	.	.
AC026310.1	0	0	0	0	0	0	Protein 101060047 [Source:UniProtKB/TrEMBL;Acc:M0QY78]	chr12:2496	.	.	.	.	.	.	protein_co	ENSG00000	.	.
AC026369.1	0	0	0	0	0	0	Uncharacterized protein [Source:UniProtKB/TrEMBL;Acc:M0R2V	chr12:1470	.	.	.	.	.	.	protein_co	ENSG00000	.	.
AC026407.1	0	0	0	0	0	0	Uncharacterized protein [Source:UniProtKB/TrEMBL;Acc:M0R23	chr5:15715	.	.	.	.	.	.	protein_co	ENSG00000	.	.
AC026461.1	0	0	0	0	0	0	Uncharacterized protein [Source:UniProtKB/TrEMBL;Acc:M0QZH	chr16:5666	.	.	.	.	.	.	protein_co	ENSG00000	.	.
AC026740.1	0.213974	0.621664	0.185799	0	0.504957	0.358926	Uncharacterized protein [Source:UniProtKB/TrEMBL;Acc:M0R2Q	chr5:65997	.	.	.	.	.	.	protein_co	ENSG00000	.	.
AC027228.1	0.051099	0	0	0	0	0.101368	HCG2005369; Uncharacterized protein [Source:UniProtKB/TrEM	chr15:7883	.	.	.	.	.	.	protein_co	ENSG00000	.	.
AC027309.1	0	0	0	0	0	0	.	chr5:17203	.	.	.	.	.	.	protein_co	ENSG00000	.	.
AC037199.1	0	0	0	0	0	0	Uncharacterized protein [Source:UniProtKB/TrEMBL;Acc:M0R2A	chr19:5137	.	.	.	.	.	.	protein_co	ENSG00000	.	.
AC037459.4	1.07E-41	9.85E-76	5.2E-107	3.6E-99	2.25E-80	1.94E-41	.	chr8:22436	.	.	.	.	.	.	protein_co	ENSG00000	.	.
AC040160.1	0	0	0.354777	0	0.159472	0		chr16:67241042-67260930								ENSG00000237102		
AC040977.1	0.426056	0.211534	0.182665	0	0.248578	0	Uncharacterized protein [Source:UniProtKB/TrEMBL;Acc:M0QX6	chr17:6915	.	.	.	.	.	.	protein_co	ENSG00000	.	.
AC051642.1	0	0	0	0	0	0	.	chr8:23386	.	.	.	.	.	.	protein_co	ENSG00000	.	.
AC055736.1	0	0	0	0	0	0	Uncharacterized protein [Source:UniProtKB/TrEMBL;Acc:M0QZD	chr12:5292	.	.	.	.	.	.	protein_co	ENSG00000	.	.
AC061975.10	0	0	0	0	0	0	Uncharacterized protein [Source:UniProtKB/TrEMBL;Acc:M0QZS	chr17:2663	.	.	.	.	.	.	protein_co	ENSG00000	.	.
AC061992.1	0.25216	0.249972	0.219361	0.270231	0	0.274964	Uncharacterized protein [Source:UniProtKB/TrEMBL;Acc:M0R27	chr17:7637	.	.	.	.	.	.	protein_co	ENSG00000	.	.
AC068039.1	0	0	0	0	0	0	Uncharacterized protein [Source:UniProtKB/TrEMBL;Acc:M0QYR	chr2:17254	.	.	.	.	.	.	protein_co	ENSG00000	.	.
AC068533.7	2.34E-33	6.99E-13	2.43E-07	1.1E-10	6.43E-11	3.33E-24	.	chr7:65540	.	.	.	.	.	.	protein_co	ENSG00000	.	.
AC068620.1	0	0	0	0	0	0	Uncharacterized protein [Source:UniProtKB/TrEMBL;Acc:M0R18	chr4:57259	.	.	.	.	.	.	protein_co	ENSG00000	.	.
AC068987.1	0.45779	1.23496	0.587019	0.510981	0.458353	0.756556	HCG1997999; cDNA FLJ33996 fis, clone DFNES2008881 [Source:U	chr12:5198	.	.	.	.	.	.	protein_co	ENSG00000	.	.
AC069368.3	7.9E-152	7.88E-14	9.63E-36	1.81E-81	1E-258	5.21E-62	.	chr15:6513	.	.	.	.	.	.	protein_co	ENSG00000	.	.
AC069547.1	0	0	0	0	0	0	HCG1745369; PRO3073; Uncharacterized protein [Source:UniPro	chr10:5206	.	.	.	.	.	.	protein_co	ENSG00000	.	.
AC069547.2	0	0	0	0	0	0	Uncharacterized protein [Source:UniProtKB/TrEMBL;Acc:M0R0F	chr10:5206	.	.	.	.	.	.	protein_co	ENSG00000	.	.
AC073063.1	0	0	0	0	0	0	.	chr7:98992	.	.	.	.	.	.	protein_co	ENSG00000	.	.
AC073188.1	0	0	0	0	0	0	.	chr7:62858	.	.	.	.	.	.	processed_	ENSG00000	.	.
AC073333.1	0	0	0	0	0	0	Uncharacterized protein [Source:UniProtKB/TrEMBL;Acc:M0R0C	chr7:16828	.	.	.	.	.	.	protein_co	ENSG00000	.	.
AC073528.1	0	0	0	0	0	0	.	chr12:7794	.	.	.	.	.	.	protein_co	ENSG00000	.	.
AC073569.1	0	0	0	0	0	0	.	chr12:8016	.	.	.	.	.	.	protein_co	ENSG00000	.	.
AC073610.5	0.000834	5.23E-07	1.39E-07	8.66E-14	0.794734	0.589021	.	chr12:4929	.	.	.	.	.	.	protein_co	ENSG00000	.	.
AC073657.1	0	0	0	0	0	0	Uncharacterized protein [Source:UniProtKB/TrEMBL;Acc:M0QY9	chr15:4206	.	.	.	.	.	.	protein_co	ENSG00000	.	.
AC078925.1	0	0	0	0	0	0	Uncharacterized protein [Source:UniProtKB/TrEMBL;Acc:M0R37	chr12:1314	.	.	.	.	.	.	protein_co	ENSG00000	.	.
AC079210.1	0.26639	0.210342	0.463047	0.249848	0.396918	0.5307	Uncharacterized protein; cDNA FLJ45097 fis, clone BRAWH30310	chr17:6650	.	.	.	.	.	.	protein_co	ENSG00000	.	.
AC079354.2	8.1374	16.6178	0	0	0	0	Uncharacterized protein [Source:UniProtKB/TrEMBL;Acc:M0QYY	chr2:20310	.	.	.	.	.	.	protein_co	ENSG00000	.	.
AC083862.1	0	0	0	0	0	0	Uncharacterized protein [Source:UniProtKB/TrEMBL;Acc:H0Y6H1	chr7:13483	.	.	.	.	.	.	protein_co	ENSG00000	.	.
AC087239.1	0	0	0	0	0	0	Uncharacterized protein [Source:UniProtKB/TrEMBL;Acc:M0R1Q	chr12:2534	.	.	.	.	.	.	protein_co	ENSG00000	.	.
AC087477.1	0	0	0	0	0	0	Uncharacterized protein [Source:UniProtKB/TrEMBL;Acc:M0R2S	chr15:9690	.	.	.	.	.	.	protein_co	ENSG00000	.	.
AC087645.1	0.505826	0.60158	0.581183	1.10739	0.676118	0.714431	Uncharacterized protein [Source:UniProtKB/TrEMBL;Acc:M0R3F	chr17:7621	.	.	.	.	.	.	protein_co	ENSG00000	.	.
AC090186.1	0.205886	0	0	0.243032	0	0	Uncharacterized protein [Source:UniProtKB/TrEMBL;Acc:Q9HAZ	chr8:52730	.	.	.	.	.	.	protein_co	ENSG00000	.	.
AC090427.1	0	0	0	0	0	0	Uncharacterized protein [Source:UniProtKB/TrEMBL;Acc:M0R2V	chr19:1480	.	.	.	.	.	.	protein_co	ENSG00000	.	.
AC090574.1	0	0	0	0	0	0	Uncharacterized protein [Source:UniProtKB/TrEMBL;Acc:M0QYB	chr8:70850	.	.	.	.	.	.	protein_co	ENSG00000	.	.
AC090673.2	0	0	0	0	0	0	Uncharacterized protein [Source:UniProtKB/TrEMBL;Acc:M0R2U	chr12:6621	.	.	.	.	.	.	protein_co	ENSG00000	.	.
AC091150.1	0	0	0	0	0	0	HCG1818186; Uncharacterized protein [Source:UniProtKB/TrEM	chr18:4555	.	.	.	.	.	.	protein_co	ENSG00000	.	.
AC091948.1	0	0	0	0	0	0	Uncharacterized protein [Source:UniProtKB/TrEMBL;Acc:M0QYF	chr5:14769	.	.	.	.	.	.	protein_co	ENSG00000	.	.
AC092291.2	0	0	0	0	0	0	.	chr16:1452	.	.	.	.	.	.	protein_co	ENSG00000	.	.
AC092384.1	0	0	0	0	0	0	.	chr16:8888	.	.	.	.	.	.	protein_co	ENSG00000	.	.
AC092782.1	0	0	0	0	0	0	Uncharacterized protein [Source:UniProtKB/TrEMBL;Acc:M0R1Z	chr1:24328	.	.	.	.	.	.	protein_co	ENSG00000	.	.
AC092811.1	0	0.06025	0.098118	0	0	0	.	chr1:22558	.	.	.	.	.	.	protein_co	ENSG00000	.	.
AC092835.2	0	0.078935	0.033148	0	0	0	.	chr2:95873	.	.	.	.	.	.	protein_co	ENSG00000	.	.
AC092850.1	0	0	0	0	0	0	Protein LOC100996701 [Source:UniProtKB/TrEMBL;Acc:M0QZH5	chr12:1317	.	XM_00384	.	.	.	XP_00527	protein_co	ENSG00000	100996701	.
AC092964.1	0	0	0	0	0	0	Uncharacterized protein [Source:UniProtKB/TrEMBL;Acc:H7BZB2	chr3:17175	.	.	.	.	.	.	protein_co	ENSG00000	.	.
AC092964.2	0	0	0	0	0	0	Uncharacterized protein [Source:UniProtKB/TrEMBL;Acc:M0QY7	chr3:17175	.	.	.	.	.	.	protein_co	ENSG00000	.	.
AC093157.1	0.088391	0.047317	0.038244	0.200347	0.049081	0	Uncharacterized protein [Source:UniProtKB/TrEMBL;Acc:M0QYT	chr1:10145	.	.	.	.	.	.	protein_co	ENSG00000	.	.

AC093323.1	0	0	0	0	0	0	Uncharacterized protein [Source:UniProtKB/TrEMBL;Acc:M0QXP	chr4:66937	.	.	.	.	.	.	protein_co	ENSG0000(	.	.
AC096582.1	0	0	0	0	0	0	Uncharacterized protein [Source:UniProtKB/TrEMBL;Acc:M0QY8	chr7:45902	.	.	.	.	.	.	protein_co	ENSG0000(	.	.
AC096644.1	0	0	0	0	0	0	Uncharacterized protein [Source:UniProtKB/TrEMBL;Acc:H7C289	chr1:22060	.	.	.	.	.	.	protein_co	ENSG0000(	.	.
AC099344.1	0	0	0	0	0	0	.	chr2:11584	.	.	.	.	.	.	protein_co	ENSG0000(	.	.
AC103801.2	0	0	0.217776	0.270628	0	0.567683	.	chr11:2264	.	.	.	.	.	.	protein_co	ENSG0000(	.	.
AC103809.2	0.498823	0	0	0	0	0	Uncharacterized protein; cDNA FLJ33592 fis, clone BRAMY201269	chr17:7232	.	.	.	.	.	.	protein_co	ENSG0000(	.	.
AC104057.1	0	0	0	0	0	0	Protein LOC100996407 [Source:UniProtKB/TrEMBL;Acc:M0QY22	chr7:65112	.	.	.	.	.	.	protein_co	ENSG0000(	.	.
AC104304.4	0	0	0	0	0	0	.	chr3:46653	NM_00119	.	.	.	NP_00118	.	protein_co	ENSG0000(	100132146	.
AC104534.3	2.99E-05	6.85E-05	0.000106	0.0005	0.00024	3.91E-05	.	chr19:3930	.	.	.	.	.	.	protein_co	ENSG0000(	.	.
AC104841.2	0.1485	0.148561	0.042935	0.053301	0.057343	0	HCG1777198, isoform CRA_a; PRO2900; Uncharacterized protein	chr2:24216	.	.	.	.	.	.	protein_co	ENSG0000(	.	.
AC106017.1	0.202831	0.201764	0.697967	0	0.467757	0.438118	Uncharacterized protein [Source:UniProtKB/TrEMBL;Acc:F6X152	chr17:1912	.	.	.	.	.	.	protein_co	ENSG0000(	.	.
AC107021.1	0	0	0	0	0	0	HCG1786590; PRO2533; Uncharacterized protein [Source:UniPro	chr3:14578	.	.	.	.	.	.	protein_co	ENSG0000(	.	.
AC108868.1	0	0	0	0	0	0	.	chr2:10709	.	.	.	.	.	.	protein_co	ENSG0000(	.	.
AC109583.1	0	0	0	0	0	0	Uncharacterized protein [Source:UniProtKB/TrEMBL;Acc:M0R2G	chr3:46919	.	.	.	.	.	.	protein_co	ENSG0000(	.	.
AC109829.1	0	0	0	0	0	0	.	chr2:27760	.	.	.	.	.	.	protein_co	ENSG0000(	.	.
AC110084.1	0	0	0	0	0	0	.	chr2:27994	.	.	.	.	.	.	protein_co	ENSG0000(	.	.
AC110615.1	0	0	0	0	0	0	Uncharacterized protein [Source:UniProtKB/TrEMBL;Acc:M0R2X	chr4:76871	.	.	.	.	.	.	protein_co	ENSG0000(	.	.
AC110771.1	0.466239	0.444436	0.132413	0.329254	0.185598	0.176534	Uncharacterized protein [Source:UniProtKB/TrEMBL;Acc:M0R18	chr4:18711	.	.	.	.	.	.	protein_co	ENSG0000(	.	.
AC111200.1	0	0	0	0	0	0	.	chr2:87042	.	.	.	.	.	.	protein_co	ENSG0000(	.	.
AC112205.1	0	0	0	0	0	0	Uncharacterized protein [Source:UniProtKB/TrEMBL;Acc:M0R1V	chr5:16286	.	.	.	.	.	.	protein_co	ENSG0000(	.	.
AC112693.2	0.041169	0	0	0	0	0	.	chr15:9374	.	.	.	.	.	.	protein_co	ENSG0000(	.	.
AC114494.1	0	0	0	0.744199	0	0	HCG1787699; Uncharacterized protein [Source:UniProtKB/TrEM	chr1:31882	.	.	.	.	.	.	protein_co	ENSG0000(	.	.
AC114546.1	0.063236	0.350837	0.10947	0.074072	0.136204	0.058723	HCG37415; PRO1914; Uncharacterized protein [Source:UniProtK	chr15:3527	.	.	.	.	.	.	protein_co	ENSG0000(	.	.
AC114783.1	0	0	0	0	0	0	Homo sapiens uncharacterized LOC101929926 (LOC101929926),	chr2:12765	NM_00132	XM_01151	.	.	NP_00130	XP_01151	protein_co	ENSG0000(	101929926	.
AC116407.2	0	0	0	0	0	0	Uncharacterized protein [Source:UniProtKB/TrEMBL;Acc:A0PK07	chr17:3046	.	.	.	.	.	.	protein_co	ENSG0000(	.	.
AC117395.1	0.208899	0.365847	0.481553	0.789914	0.702802	0.345685	LOC646903 protein; Uncharacterized protein [Source:UniProtKB/	chr3:14968	.	.	.	.	.	.	protein_co	ENSG0000(	.	.
AC117834.1	0	0	0.046341	0.062813	0	0	.	chr8:93895	.	.	.	.	.	.	protein_co	ENSG0000(	.	.
AC119673.1	0.388198	0.157887	0.421271	0.500907	0.480312	0.375316	.	chr1:20568	.	.	.	.	.	.	protein_co	ENSG0000(	.	.
AC120194.1	0	0	0	0.134277	0.244721	0	.	chr8:71485	.	.	.	.	.	.	protein_co	ENSG0000(	.	.
AC121757.1	0	0	0	0	0	0	Uncharacterized protein [Source:UniProtKB/TrEMBL;Acc:M0R17	chr12:5270	.	.	.	.	.	.	protein_co	ENSG0000(	.	.
AC126614.1	0	0	0	0	0	0	HCG1818482; Uncharacterized protein [Source:UniProtKB/TrEM	chr12:5764	.	.	.	.	.	.	protein_co	ENSG0000(	.	.
AC127496.1	0.83291	0.476397	0.589858	0.668872	0.403069	0.456358	Uncharacterized protein [Source:UniProtKB/TrEMBL;Acc:Q9HBN	chr17:7897	.	.	.	.	.	.	protein_co	ENSG0000(	.	.
AC131097.4	0	0	0	0	0	0	.	chr2:24283	.	.	.	.	.	.	protein_co	ENSG0000(	285095	.
AC131935.1	0	0	0	0	0	0	.	chr11:1174	.	.	.	.	.	.	protein_co	ENSG0000(	.	.
AC132186.1	0.02258	0.138561	0.058697	0	0.077359	0.023667	CDNA FLJ27243 fis, clone SYN08134; Uncharacterized protein [So	chr16:6646	.	.	.	.	.	.	protein_co	ENSG0000(	.	.
AC132872.2	0.039805	0.123896	0.310596	0.088234	0.134876	0.325937	CDNA FLJ27256 fis, clone SYN09689; Uncharacterized protein [So	chr17:8018	.	.	.	.	.	.	protein_co	ENSG0000(	.	.
AC135048.1	0	0	0.102694	0	0	0.264747	Uncharacterized protein [Source:UniProtKB/TrEMBL;Acc:Q6PJ53	chr16:3099	.	.	.	.	.	.	protein_co	ENSG0000(	.	.
AC135178.1	1.38469	0.950499	0.814575	1.24009	1.36236	0.770518	HCG1985372; Uncharacterized protein; cDNA FLJ37541 fis, clone	chr17:8261	.	.	.	.	.	.	protein_co	ENSG0000(	.	.
AC135983.2	0	0	0	0	0	0	Protein LOC100996413 [Source:UniProtKB/TrEMBL;Acc:M0R198]	chr15:3277	.	XM_00384	.	.	.	XP_005276	protein_co	ENSG0000(	100996413	.
AC136297.1	0	0	0	0	0	0	Uncharacterized protein [Source:UniProtKB/TrEMBL;Acc:M0R3D	chr11:1295	.	.	.	.	.	.	protein_co	ENSG0000(	.	.
AC137056.1	0	0	0	0	0	0	Uncharacterized protein; cDNA FLJ34659 fis, clone KIDNE2018863	chr16:2049	.	.	.	.	.	.	protein_co	ENSG0000(	.	.
AC138393.1	0	0	0	0	0	0	Uncharacterized protein [Source:UniProtKB/TrEMBL;Acc:M0QYQ	chr1:22421	.	.	.	.	.	.	protein_co	ENSG0000(	.	.
AC138517.1	0	0	0	0	0	0	Uncharacterized protein [Source:UniProtKB/TrEMBL;Acc:M0R32	chr5:13885	.	.	.	.	.	.	protein_co	ENSG0000(	.	.
AC138517.6	0	0	0	0	0	0	.	chr5:13885	.	.	.	.	.	.	protein_co	ENSG0000(	.	.
AC138647.1	0	0	0	0	0	0	.	chr8:14252	.	.	.	.	.	.	protein_co	ENSG0000(	.	.
AC138655.1	0	0	0	0	0	0	CDNA: FLJ23120 fis, clone LNG07989; HCG1987724; Uncharacteri	chr2:47799	.	.	.	.	.	.	protein_co	ENSG0000(	.	.
AC138969.4	0	0	0	0	0	0	.	chr16:1642	.	.	.	.	.	XP_005255	protein_co	ENSG0000(	9284, 6427	.
AC139768.1	0	0	0	0	0	0	Uncharacterized protein [Source:UniProtKB/TrEMBL;Acc:M0R1B	chr12:5161	.	.	.	.	.	.	protein_co	ENSG0000(	.	.
AC140061.12	0	0	0	0	0	0.34057	Uncharacterized protein [Source:UniProtKB/TrEMBL;Acc:M0QZ2	chr12:5069	.	.	.	.	.	.	protein_co	ENSG0000(	.	.
AC187652.1	0	0	0	0	0	0	Protein LOC100996433 [Source:UniProtKB/TrEMBL;Acc:M0R295]	chr7:30291	.	XM_00524	.	.	.	XP_005276	protein_co	ENSG0000(	100996433	.
ACAA1	25.4757	26.5952	26.5044	22.5528	19.2304	20.7554	acetyl-CoA acyltransferase 1 [Source:HGNC Symbol;Acc:HGNC:82	chr3:38080	NM_00113	XM_00671	.	.	NP_00112	XP_006713	protein_co	ENSG0000(	30	82
ACAA2	9.35935	7.95806	10.531	10.2845	7.47748	11.9794	acetyl-CoA acyltransferase 2 [Source:HGNC Symbol;Acc:HGNC:83	chr18:4730	NM_00611	.	.	.	NP_00610	.	protein_co	ENSG0000(	10449	83
ACACA	26.9351	27.7148	33.8845	31.2634	29.0744	35.1845	acetyl-CoA carboxylase alpha [Source:HGNC Symbol;Acc:HGNC:8	chr17:3544	NM_19883	XM_01702	.	.	NP_94213	XP_005257	protein_co	ENSG0000(	31	84
ACACB	1.16272	1.2921	1.21321	0.996203	1.24488	1.31937	acetyl-CoA carboxylase beta [Source:HGNC Symbol;Acc:HGNC:85	chr12:1095	NM_00109	XM_01153	.	.	NP_00108	XP_005253	protein_co	ENSG0000(	32	85
ACAD10	6.72689	6.3848	8.08727	5.22456	4.81643	6.30049	acyl-CoA dehydrogenase family member 10 [Source:HGNC Symbo	chr12:1121	NM_00113	.	.	.	NP_00113	.	protein_co	ENSG0000(	80724	21597
ACAD11	5.44747	5.11249	8.01277	5.99732	6.64995	8.12599	acyl-CoA dehydrogenase family member 11 [Source:HGNC Symbo	chr3:13227	NM_03216	.	.	.	NP_11554	.	protein_co	ENSG0000(	84129	30211
ACAD8	12.5434	12.4053	13.0522	11.0747	9.1269	10.279	acyl-CoA dehydrogenase family member 8 [Source:HGNC Symbol	chr11:1341	NM_01438	XM_01154	.	.	NP_05519	XP_011541	protein_co	ENSG0000(	27034	87
ACAD9	21.8996	21.3637	19.064	21.8633	19.9176	15.0402	acyl-CoA dehydrogenase family member 9 [Source:HGNC Symbol	chr3:12859	NM_01404	XM_01151	.	.	NP_05476	.	protein_co	ENSG0000(	28976	21497
ACADL	0	0	0	0	0	0	acyl-CoA dehydrogenase, long chain [Source:HGNC Symbol;Acc:H	chr2:21105	NM_00160	XM_00524	.	.	NP_00159	XP_005246	protein_co	ENSG0000(	33	88

ACADM	36.0042	37.3576	43.3902	34.29	36.8752	39.4382	acyl-CoA dehydrogenase, C-4 to C-12 straight chain [Source:HGN	chr1:76190	NM_00001	.	.	.	NP_00000	.	protein_co	ENSG0000	34	89
ACADS	13.7166	15.1542	15.4918	19.8623	17.8068	15.5955	acyl-CoA dehydrogenase, C-2 to C-3 short chain [Source:HGNC Sy	chr12:1211	NM_00001	.	.	.	NP_00000	.	protein_co	ENSG0000	35	90
ACADSB	8.72132	10.2835	12.2341	10.6282	11.0592	11.0374	acyl-CoA dehydrogenase, short/branched chain [Source:HGNC Sy	chr10:1247	NM_00160	XM_01701	.	.	NP_00160	XP_01687	protein_co	ENSG0000	36	91
ACADVL	109.29	113.224	124.083	121.769	107.249	108.301	acyl-CoA dehydrogenase, very long chain [Source:HGNC Symbol;A	chr17:7093	NM_00001	XM_01152	.	.	NP_00000	XP_00672	protein_co	ENSG0000	37	92
ACAN	0	0	0	0	0	0	aggrecan [Source:HGNC Symbol;Acc:HGNC:319]	chr15:8934	NM_00113	XM_01702	.	.	NP_00112	XP_00672	protein_co	ENSG0000	176	319
ACAP1	2.37857	3.39189	2.92946	5.435	1.03384	1.63976	ArfGAP with coiled-coil, ankyrin repeat and PH domains 1 [Source	chr17:7239	NM_01471	XM_00525	.	.	NP_05553	.	protein_co	ENSG0000	9744	16467
ACAP2	9.46044	9.48657	8.54472	9.92817	12.1288	12.9367	ArfGAP with coiled-coil, ankyrin repeat and PH domains 2 [Source	chr3:19499	NM_01228	XM_01151	.	.	NP_03641	XP_00671	protein_co	ENSG0000	23527	16469
ACAP3	9.65851	9.40985	11.589	8.53331	7.39283	7.39872	ArfGAP with coiled-coil, ankyrin repeat and PH domains 3 [Source	chr1:12277	NM_03064	XM_01154	.	.	NP_08515	XP_00524	protein_co	ENSG0000	116983	16754
ACAT1	56.839	51.337	62.3701	62.3191	67.2996	72.8736	acetyl-CoA acetyltransferase 1 [Source:HGNC Symbol;Acc:HGNC:	chr11:1079	NM_00001	.	.	.	NP_00001	.	protein_co	ENSG0000	38	93
ACAT2	15.2031	13.8583	16.6637	20.2163	17.7881	19.6151	acetyl-CoA acetyltransferase 2 [Source:HGNC Symbol;Acc:HGNC:	chr6:16009	NM_00589	.	.	.	NP_00588	.	protein_co	ENSG0000	39	94
ACBD3	14.9151	14.8647	13.4546	14.1964	13.9969	15.3323	acyl-CoA binding domain containing 3 [Source:HGNC Symbol;Acc:	chr1:22633	NM_02273	.	.	.	NP_07357	.	protein_co	ENSG0000	64746	15453
ACBD4	3.14747	3.71645	6.26928	2.1319	1.41118	1.82014	acyl-CoA binding domain containing 4 [Source:HGNC Symbol;Acc:	chr17:4320	NM_00113	XM_01702	.	.	NP_00112	XP_00525	protein_co	ENSG0000	79777	23337
ACBD5	10.0924	10.9254	9.83679	9.04448	10.5701	9.50357	acyl-CoA binding domain containing 5 [Source:HGNC Symbol;Acc:	chr10:2748	NM_00104	XM_01701	.	.	NP_00103	XP_00671	protein_co	ENSG0000	91452	23338
ACBD6	20.4764	21.4029	24.3855	24.6104	22.7143	28.4586	acyl-CoA binding domain containing 6 [Source:HGNC Symbol;Acc:	chr1:18025	NM_03236	.	NR_03764	.	NP_11573	.	protein_co	ENSG0000	100527964	23339
ACBD7	1.62196	2.20262	2.78336	2.21713	2.34207	4.03797	acyl-CoA binding domain containing 7 [Source:HGNC Symbol;Acc:	chr10:1511	NM_00103	.	.	.	NP_00103	.	protein_co	ENSG0000	414149	17715
ACCS	1.46272	1.13869	2.40665	0.792276	0.955745	1.32107	1-aminocyclopropane-1-carboxylate synthase homolog (inactive)	chr11:4408	NM_00112	XM_01152	.	.	NP_00112	XP_00525	protein_co	ENSG0000	84680	23989
ACCSL	0	0.081384	0	0.130095	0	0	1-aminocyclopropane-1-carboxylate synthase homolog (inactive)	chr11:4406	NM_00103	XM_01152	.	.	NP_00102	XP_01687	protein_co	ENSG0000	390110	34391
ACD	27.79	28.0845	23.8948	28.0562	22.5113	17.8135	adrenocortical dysplasia homolog [Source:HGNC Symbol;Acc:HGN	chr16:6767	NM_00108	XM_00525	.	.	NP_00107	XP_00525	protein_co	ENSG0000	65057	25070
ACE	0.862896	0.821562	1.29666	0.713127	0.553695	0.624521	angiotensin I converting enzyme [Source:HGNC Symbol;Acc:HGN	chr17:6155	NM_00078	XM_00672	.	.	NP_00078	XP_00672	protein_co	ENSG0000	1636	2707
ACE2	0	0	0	0.021715	0	0.056353	angiotensin I converting enzyme 2 [Source:HGNC Symbol;Acc:HG	chrX:15579	NM_02180	XM_01154	.	.	NP_06857	XP_01154	protein_co	ENSG0000	59272	13557
ACER1	0	0.063605	0	0	0	0	alkaline ceramidase 1 [Source:HGNC Symbol;Acc:HGNC:18356]	chr19:6306	NM_13349	.	.	.	NP_59799	.	protein_co	ENSG0000	125981	18356
ACER2	2.04174	2.31154	3.34891	2.20322	2.98594	3.11194	alkaline ceramidase 2 [Source:HGNC Symbol;Acc:HGNC:23675]	chr9:19409	NM_00101	XM_00525	.	.	NP_00101	.	protein_co	ENSG0000	340485	23675
ACER3	6.00225	5.10479	7.06624	5.6225	7.95181	7.44497	alkaline ceramidase 3 [Source:HGNC Symbol;Acc:HGNC:16066]	chr11:7657	NM_00130	XM_01154	.	.	NP_00128	XP_01154	protein_co	ENSG0000	55331	16066
ACHE	2.15884	1.43905	2.08486	0.691425	0.566539	0.723948	acetylcholinesterase (Cartwright blood group) [Source:HGNC Sym	chr7:10048	NM_00066	XM_01151	.	.	NP_00065	XP_00671	protein_co	ENSG0000	43	108
ACIN1	56.534	57.5363	48.0239	66.8259	61.9314	46.6427	apoptotic chromatin condensation inducer 1 [Source:HGNC Symb	chr14:2352	NM_00116	XM_01153	.	.	NP_00115	XP_00526	protein_co	ENSG0000	22985	17066
ACKR1	0	0	0	0	0	0	atypical chemokine receptor 1 (Duffy blood group) [Source:HGNC	chr1:15914	NM_00112	.	.	.	NP_00111	.	protein_co	ENSG0000	2532	4035
ACKR2	0.034508	0.142446	0.266745	0	0	0.249935	atypical chemokine receptor 2 [Source:HGNC Symbol;Acc:HGNC:	chr3:42850	NM_00129	.	.	.	NP_00128	.	protein_co	ENSG0000	1238	1565
ACKR3	0.085198	0	0.036333	0	0	0	atypical chemokine receptor 3 [Source:HGNC Symbol;Acc:HGNC:	chr2:23747	NM_02031	XM_01151	.	.	NP_06470	XP_00524	protein_co	ENSG0000	57007	23692
ACKR4	0.044599	0	0.038674	0	0	0	atypical chemokine receptor 4 [Source:HGNC Symbol;Acc:HGNC:	chr3:13227	NM_01655	.	.	.	NP_05764	.	protein_co	ENSG0000	51554	1611
ACLY	70.3324	76.4087	84.0887	94.166	91.0434	80.7191	ATP citrate lyase [Source:HGNC Symbol;Acc:HGNC:115]	chr17:4002	NM_00109	XM_01702	.	.	NP_00108	XP_00525	protein_co	ENSG0000	47	115
ACMSD	0	0	0	0	0.062303	0	aminocarboxymuconate semialdehyde decarboxylase [Source:HG	chr2:13559	NM_00130	XM_01151	.	.	NP_00129	XP_00526	protein_co	ENSG0000	130013	19288
ACO1	17.2686	17.7583	23.3542	19.7641	20.4809	22.1712	aconitase 1 [Source:HGNC Symbol;Acc:HGNC:117]	chr9:32384	NM_00127	XM_01151	.	.	NP_00126	XP_00525	protein_co	ENSG0000	48	117
ACO2	42.049	43.9985	41.1415	47.8504	42.3181	44.9023	aconitase 2 [Source:HGNC Symbol;Acc:HGNC:118]	chr22:4186	NM_00109	.	.	.	NP_00108	XP_01688	protein_co	ENSG0000	50	118
ACOD1	0	0	0	0	0	0	aconitate decarboxylase 1 [Source:HGNC Symbol;Acc:HGNC:3390	chr13:7752	NM_00125	XM_01153	.	.	NP_00124	.	protein_co	ENSG0000	730249	33904
ACOT1	0	0.055723	0	0	0.064623	0	acyl-CoA thioesterase 1 [Source:HGNC Symbol;Acc:HGNC:33128]	chr14:7394	NM_00103	.	.	.	NP_00103	.	protein_co	ENSG0000	641371	33128
ACOT11	7.71151	8.17806	7.45564	10.8843	9.46773	9.42351	acyl-CoA thioesterase 11 [Source:HGNC Symbol;Acc:HGNC:18156	chr1:55013	NM_01554	.	.	.	NP_05636	.	protein_co	ENSG0000	26027	18156
ACOT12	0	0	0.032079	0	0	0	acyl-CoA thioesterase 12 [Source:HGNC Symbol;Acc:HGNC:24436	chr5:80625	NM_13076	XM_01154	.	.	NP_57012	XP_00671	protein_co	ENSG0000	134526	24436
ACOT13	10.9757	9.28159	8.15937	10.6753	9.00852	9.80686	acyl-CoA thioesterase 13 [Source:HGNC Symbol;Acc:HGNC:20999	chr6:24667	NM_00116	.	.	.	NP_00115	.	protein_co	ENSG0000	55856	20999
ACOT2	0	0	0	0	0	0	acyl-CoA thioesterase 2 [Source:HGNC Symbol;Acc:HGNC:18431]	chr14:7403	NM_00682	.	.	.	NP_00681	.	protein_co	ENSG0000	10965	18431
ACOT4	0	0	0	0	0	0	acyl-CoA thioesterase 4 [Source:HGNC Symbol;Acc:HGNC:19748]	chr14:7405	NM_15233	.	.	.	NP_68954	.	protein_co	ENSG0000	122970	19748
ACOT6	0	0	0	0	0	0	acyl-CoA thioesterase 6 [Source:HGNC Symbol;Acc:HGNC:33159]	chr14:7408	NM_00103	.	.	.	NP_00103	.	protein_co	ENSG0000	641372	33159
ACOT7	87.8841	87.8246	86.5926	123.569	98.4521	92.9443	acyl-CoA thioesterase 7 [Source:HGNC Symbol;Acc:HGNC:24157]	chr1:63243	NM_00727	.	.	.	NP_00920	.	protein_co	ENSG0000	11332	24157
ACOT8	7.23729	8.36404	9.34407	11.1256	8.77457	6.79263	acyl-CoA thioesterase 8 [Source:HGNC Symbol;Acc:HGNC:15919]	chr20:4446	NM_00546	XM_01152	.	.	NP_00546	XP_00526	protein_co	ENSG0000	10005	15919
ACOT9	31.9291	32.4506	32.8776	29.1643	31.5125	35.3452	acyl-CoA thioesterase 9 [Source:HGNC Symbol;Acc:HGNC:17152]	chrX:23720	NM_00103	XM_00527	.	.	NP_00102	XP_00527	protein_co	ENSG0000	23597	17152
ACOX1	15.1434	16.9858	16.6044	16.8612	18.5057	19.7362	acyl-CoA oxidase 1 [Source:HGNC Symbol;Acc:HGNC:119]	chr17:7393	NM_00118	XM_01152	.	.	NP_00117	XP_01152	protein_co	ENSG0000	51	119
ACOX2	2.37304	1.99156	2.02009	3.29764	2.31466	2.65058	acyl-CoA oxidase 2 [Source:HGNC Symbol;Acc:HGNC:120]	chr3:58490	NM_00350	XM_01153	.	.	NP_00349	XP_00526	protein_co	ENSG0000	8309	120
ACOX3	6.36299	5.61335	7.28319	6.69807	4.98036	6.52148	acyl-CoA oxidase 3, pristanoyl [Source:HGNC Symbol;Acc:HGNC:1	chr4:83680	NM_00110	XM_01151	.	.	NP_00109	XP_00524	protein_co	ENSG0000	8310	121
ACOXL	1.62612	1.44492	1.58908	2.14593	1.31751	1.29642	acyl-CoA oxidase-like [Source:HGNC Symbol;Acc:HGNC:25621]	chr2:11149	NM_00114	XM_01151	.	.	NP_00113	XP_01150	protein_co	ENSG0000	55289	25621
ACP1	96.2649	91.1039	85.6272	92.3882	89.0919	81.9839	acid phosphatase 1, soluble [Source:HGNC Symbol;Acc:HGNC:122	chr2:26486	NM_00104	XM_01151	.	.	NP_00103	XP_01150	protein_co	ENSG0000	52	122
ACP2	13.8836	16.5345	11.443	12.9137	11.8546	11.7057	acid phosphatase 2, lysosomal [Source:HGNC Symbol;Acc:HGNC:	chr11:4726	NM_00130	.	.	.	NP_00128	.	protein_co	ENSG0000	53	123
ACP5	0	0	0	0	0	0	acid phosphatase 5, tartrate resistant [Source:HGNC Symbol;Acc:	chr19:1168	NM_00111	XM_01152	.	.	NP_00110	XP_00525	protein_co	ENSG0000	54	124
ACP6	6.37755	4.76775	7.65552	3.5454	2.91669	2.92416	acid phosphatase 6, lysophosphatidic [Source:HGNC Symbol;Acc:	chr1:14711	NM_01636	.	.	.	NP_05744	.	protein_co	ENSG0000	51205	29609
ACP7	1.4076	1.48725	1.60515	1.3295	1.49747	1.37059	acid phosphatase 7, tartrate resistant (putative) [Source:HGNC Sy	chr19:3957	NM_00100	XM_01152	.	.	NP_00100	XP_01152	protein_co	ENSG0000	390928	33781
ACPP	0.191857	0.09136	0.479738	0.25677	0.242683	0.26594	acid phosphatase, prostate [Source:HGNC Symbol;Acc:HGNC:125	chr3:13203	NM_00109	XM_01151	.	.	NP_00109	XP_01151	protein_co	ENSG0000	55	125
ACPT	0	0	0	0	0	0	acid phosphatase, testicular [Source:HGNC Symbol;Acc:HGNC:14	chr19:5128	NM_03306	.	.	.	NP_14905	.	protein_co	ENSG0000	93650	14376
ACR	0.12312	0.252538	0.536885	0.404431	0.140481	0.127778	acrosin [Source:HGNC Symbol;Acc:HGNC:126]	chr22:5117	NM_00109	.	.	.	NP_00108	.	protein_co	ENSG0000	49	126
ACRBP	0.411756	0.292912	0.173143	0.26604	0.143198	0.290463	acrosin binding protein [Source:HGNC Symbol;Acc:HGNC:17195]	chr12:6747	NM_03248	.	.	.	NP_11587	.	protein_co	ENSG0000	84519	17195
ACRC	0.499237	0.408319	0.328642	0.301244	0.281839	0.536099	acidic repeat containing [Source:HGNC Symbol;Acc:HGNC:15805]	chrX:70798	NM_05295	XM_01702	.	.	NP_44318	XP_00672	protein_co	ENSG0000	93953	15805
ACRV1	0	0	0.031336	0.105629	0	0	acrosomal vesicle protein 1 [Source:HGNC Symbol;Acc:HGNC:127	chr11:1254	NM_00161	.	.	.	NP_00160	.	protein_co	ENSG0000	56	127

ACSBG1	0.192379	0.123178	0.320724	0.264172	0.479089	0.567965	acyl-CoA synthetase bubblegum family member 1 [Source:HGNC	chr15:7844	NM_00119	XM_01152	.	.	NP_00118	XP_011519	protein_co	ENSG0000	23205	29567
ACSBG2	0.140869	0.171683	0.163976	0.261794	0.263805	0.383231	acyl-CoA synthetase bubblegum family member 2 [Source:HGNC	chr19:6135	NM_00128	XM_01702	.	.	NP_00127	XP_005259	protein_co	ENSG0000	81616	24174
ACSF2	8.76364	7.38046	12.604	8.40531	6.27599	9.04907	acyl-CoA synthetase family member 2 [Source:HGNC Symbol;Acc:	chr17:4850	NM_00128	XM_01152	NR_11023	.	NP_00127	XP_011523	protein_co	ENSG0000	80221	26101
ACSF3	16.5097	19.2703	18.9134	19.6068	16.6617	15.2027	acyl-CoA synthetase family member 3 [Source:HGNC Symbol;Acc:	chr16:8916	NM_00112	XM_00525	NR_04566	.	NP_00112	XP_005256	protein_co	ENSG0000	197322	27288
ACSL1	5.41568	4.51141	6.74386	8.37115	8.66489	9.81266	acyl-CoA synthetase long-chain family member 1 [Source:HGNC S	chr4:18567	NM_00128	XM_01700	.	.	NP_00127	XP_005262	protein_co	ENSG0000	2180	3569
ACSL3	40.7765	40.7497	39.0326	50.851	63.7326	65.7732	acyl-CoA synthetase long-chain family member 3 [Source:HGNC S	chr2:22372	NM_00445	.	.	.	NP_00444	XP_016859	protein_co	ENSG0000	2181	3570
ACSL4	19.2339	18.9802	23.7429	24.2739	25.6428	25.6429	acyl-CoA synthetase long-chain family member 4 [Source:HGNC S	chrX:10888	NM_00131	XM_01153	.	.	NP_00130	XP_005262	protein_co	ENSG0000	2182	3571
ACSL5	81.9411	85.0447	101.132	121.563	113.246	146.838	acyl-CoA synthetase long-chain family member 5 [Source:HGNC S	chr10:1141	NM_01623	XM_01701	.	.	NP_05731	XP_016871	protein_co	ENSG0000	51703	16526
ACSL6	0.226736	0.43024	0.320387	0.422691	0.201034	0.499533	acyl-CoA synthetase long-chain family member 6 [Source:HGNC S	chr5:13128	NM_00100	.	.	.	NP_00100	.	protein_co	ENSG0000	23305	16496
ACSM1	0.028821	0.059113	0.201073	0.094767	0.037301	0.241429	acyl-CoA synthetase medium-chain family member 1 [Source:HG	chr16:2063	NM_00131	XM_00672	.	.	NP_00130	XP_005255	protein_co	ENSG0000	116285	18049
ACSM2A	0	0	0	0	0	0	acyl-CoA synthetase medium-chain family member 2A [Source:H	chr16:2046	NM_00101	XM_01702	.	.	NP_00101	XP_005255	protein_co	ENSG0000	123876	32017
ACSM2B	0	0	0	0	0	0	acyl-CoA synthetase medium-chain family member 2B [Source:H	chr16:2054	NM_00110	XM_01154	.	.	NP_00109	XP_011544	protein_co	ENSG0000	348158	30931
ACSM3	3.77937	3.38751	4.44145	4.31467	3.76626	5.67343	acyl-CoA synthetase medium-chain family member 3 [Source:HG	chr16:2077	NM_00562	XM_01154	.	.	NP_00561	XP_011544	protein_co	ENSG0000	6296	10522
ACSM4	0	0	0	0	0	0	acyl-CoA synthetase medium-chain family member 4 [Source:HG	chr12:7456	NM_00108	.	.	.	NP_00107	.	protein_co	ENSG0000	341392	32016
ACSM5	0	0	0	0	0	0	acyl-CoA synthetase medium-chain family member 5 [Source:HG	chr16:2042	NM_00132	XM_00672	.	.	NP_00131	XP_006721	protein_co	ENSG0000	54988	26060
ACSM6	0	0	0	0	0	0	acyl-CoA synthetase medium-chain family member 6 [Source:HG	chr10:9695	NM_20732	.	.	.	NP_99720	.	protein_co	ENSG0000	142827	31665
ACSS1	0.020377	0	0	0	0	0.021626	acyl-CoA synthetase short-chain family member 1 [Source:HGNC	chr20:2498	NM_00125	XM_01152	.	.	NP_00123	XP_011527	protein_co	ENSG0000	84532	16091
ACSS2	15.8068	13.5078	19.7588	16.4837	14.8989	19.6879	acyl-CoA synthetase short-chain family member 2 [Source:HGNC	chr20:3346	NM_00107	XM_01152	.	.	NP_00107	XP_005260	protein_co	ENSG0000	55902	15814
ACSS3	0	0	0.025768	0.033425	0	0.029827	acyl-CoA synthetase short-chain family member 3 [Source:HGNC	chr12:8147	NM_02456	XM_00526	.	.	NP_07883	XP_005269	protein_co	ENSG0000	79611	24723
ACTA1	0.099897	0.393063	0.128636	0.053367	0.175412	0.055767	actin, alpha 1, skeletal muscle [Source:HGNC Symbol;Acc:HGNC:1	chr1:22956	NM_00110	.	.	.	NP_00109	.	protein_co	ENSG0000	58	129
ACTA2	3.90788	3.42799	6.64306	3.14913	2.71475	4.40402	actin, alpha 2, smooth muscle, aorta [Source:HGNC Symbol;Acc:H	chr10:9063	NM_00114	XM_01154	.	.	NP_00113	XP_011538	protein_co	ENSG0000	59	130
ACTB	1135.11	1164.85	1064.06	1393.79	1270.74	1212.19	actin beta [Source:HGNC Symbol;Acc:HGNC:132]	chr7:55667	NM_00110	.	.	.	NP_00109	.	protein_co	ENSG0000	60	132
ACTBL2	0.060911	0.031955	0.052857	0.239249	0.068131	0.152723	actin, beta like 2 [Source:HGNC Symbol;Acc:HGNC:17780]	chr5:56775	NM_00101	.	.	.	NP_00101	.	protein_co	ENSG0000	345651	17780
ACTC1	0	0	0	0	0	0	actin, alpha, cardiac muscle 1 [Source:HGNC Symbol;Acc:HGNC:1	chr15:3508	NM_00515	.	.	.	NP_00515	.	protein_co	ENSG0000	70	143
ACTG1	2252.47	2327.63	2433.24	2633.19	2438.14	2612.08	actin gamma 1 [Source:HGNC Symbol;Acc:HGNC:144]	chr17:7947	NM_00119	.	.	.	NP_00118	.	protein_co	ENSG0000	71	144
ACTG2	0.053555	0	0	0	0	0	actin, gamma 2, smooth muscle, enteric [Source:HGNC Symbol;A	chr2:74120	NM_00119	.	.	.	NP_00118	.	protein_co	ENSG0000	72	145
ACTL10	0	0	0	0	0.064346	0	actin like 10 [Source:HGNC Symbol;Acc:HGNC:16127]	chr20:3224	NM_00102	.	.	.	NP_00101	.	protein_co	ENSG0000	170487	16127
ACTL6A	47.3848	44.1461	43.0363	51.8013	52.8261	57.1661	actin like 6A [Source:HGNC Symbol;Acc:HGNC:24124]	chr3:17928	NM_00430	.	.	.	NP_00429	.	protein_co	ENSG0000	86	24124
ACTL6B	0	0	0	0	0	0	actin like 6B [Source:HGNC Symbol;Acc:HGNC:160]	chr7:10024	NM_01618	.	.	.	NP_05727	.	protein_co	ENSG0000	51412	160
ACTL7A	0	0	0	0	0	0	actin like 7A [Source:HGNC Symbol;Acc:HGNC:161]	chr9:11162	NM_00668	.	.	.	NP_00667	.	protein_co	ENSG0000	10881	161
ACTL7B	0.035347	0	0	0	0	0	actin like 7B [Source:HGNC Symbol;Acc:HGNC:162]	chr9:11161	NM_00668	.	.	.	NP_00667	.	protein_co	ENSG0000	10880	162
ACTL8	1.13696	1.50535	0.406609	1.92713	1.63801	0.716936	actin like 8 [Source:HGNC Symbol;Acc:HGNC:24018]	chr1:18081	NM_03081	XM_01154	.	.	NP_11043	XP_011540	protein_co	ENSG0000	81569	24018
ACTL9	0	0	0	0	0	0	actin like 9 [Source:HGNC Symbol;Acc:HGNC:28494]	chr19:8807	NM_17852	.	.	.	NP_84862	.	protein_co	ENSG0000	284382	28494
ACTN1	35.5701	37.6663	41.7541	49.5857	45.5729	44.3389	actinin alpha 1 [Source:HGNC Symbol;Acc:HGNC:163]	chr14:6934	NM_00110	XM_01702	.	.	NP_00109	XP_011535	protein_co	ENSG0000	87	163
ACTN2	0.100213	0.058865	0.049644	0.126772	0.130454	0.074238	actinin alpha 2 [Source:HGNC Symbol;Acc:HGNC:164]	chr1:23684	NM_00110	.	.	.	NP_00109	.	protein_co	ENSG0000	88	164
ACTN3	0	0	0.037284	0	0	0	actinin alpha 3 (gene/pseudogene) [Source:HGNC Symbol;Acc:HG	chr11:6631	NM_00110	.	.	.	NP_00109	.	protein_co	ENSG0000	89	165
ACTN4	99.8226	103.556	110.286	108.976	99.6448	95.7927	actinin alpha 4 [Source:HGNC Symbol;Acc:HGNC:166]	chr19:3913	NM_00492	XM_00672	.	.	NP_00130	XP_005259	protein_co	ENSG0000	81	166
ACTR10	22.4802	19.9807	19.5751	23.6405	24.3532	21.8459	actin-related protein 10 homolog [Source:HGNC Symbol;Acc:HGN	chr14:5866	NM_01847	XM_01153	.	.	NP_06094	XP_011535	protein_co	ENSG0000	55860	17372
ACTR1A	66.055	70.1337	66.7604	75.9906	67.9167	56.7725	ARP1 actin-related protein 1 homolog A, centractin alpha [Source	chr10:1042	NM_00573	.	.	.	NP_00572	.	protein_co	ENSG0000	10121	167
ACTR1B	16.3139	17.2713	17.9813	17.1404	15.5316	18.6063	ARP1 actin-related protein 1 homolog B, centractin beta [Source:	chr2:98272	NM_00573	XM_00526	.	.	NP_00572	XP_005263	protein_co	ENSG0000	10120	168
ACTR2	80.0752	81.195	89.5208	88.7762	94.9017	112.282	ARP2 actin related protein 2 homolog [Source:HGNC Symbol;Acc:	chr2:65454	NM_00100	.	.	.	NP_00100	.	protein_co	ENSG0000	10097	169
ACTR3	95.1567	97.659	98.2405	97.7689	92.1327	114.909	ARP3 actin related protein 3 homolog [Source:HGNC Symbol;Acc:	chr2:11464	NM_00127	.	.	.	NP_00126	.	protein_co	ENSG0000	10096	170
ACTR3B	11.6753	10.5829	12.0339	10.6834	11.6199	10.0853	ARP3 actin related protein 3 homolog B [Source:HGNC Symbol;Ac	chr7:15245	NM_00104	XM_01151	.	.	NP_00103	XP_011514	protein_co	ENSG0000	57180	17256
ACTR3C	0.413416	0.311904	0.222567	0.298704	0.321867	0.480814	ARP3 actin-related protein 3 homolog C [Source:HGNC Symbol;Ac	chr7:14994	NM_00116	XM_01151	.	.	NP_00115	XP_005250	protein_co	ENSG0000	653857	37282
ACTR5	14.0772	11.6842	16.295	13.8091	11.859	11.8427	ARP5 actin-related protein 5 homolog [Source:HGNC Symbol;Acc:	chr20:3737	NM_02485	XM_00526	.	.	NP_07913	.	protein_co	ENSG0000	79913	14671
ACTR6	14.3213	13.0429	17.2929	13.1795	18.6912	23.5272	ARP6 actin-related protein 6 homolog [Source:HGNC Symbol;Acc:	chr12:1005	NM_02249	.	.	.	NP_07194	XP_016875	protein_co	ENSG0000	64431	24025
ACTR8	12.7686	13.5211	11.2856	13.6007	14.0602	10.9797	ARP8 actin-related protein 8 homolog [Source:HGNC Symbol;Acc:	chr3:53901	NM_02289	XM_01153	.	.	NP_07505	XP_005265	protein_co	ENSG0000	93973	14672
ACTRT1	0	0	0	0	0	0	actin related protein T1 [Source:HGNC Symbol;Acc:HGNC:24027]	chrX:12718	NM_13828	.	.	.	NP_61214	.	protein_co	ENSG0000	139741	24027
ACTRT2	0	0	0	0	0	0	actin related protein T2 [Source:HGNC Symbol;Acc:HGNC:24026]	chr1:29380	NM_08043	.	.	.	NP_53635	.	protein_co	ENSG0000	140625	24026
ACTRT3	1.46035	1.55729	1.0016	1.05335	1.40666	1.39842	actin related protein T3 [Source:HGNC Symbol;Acc:HGNC:24022]	chr3:16948	NM_03248	.	.	.	NP_11587	.	protein_co	ENSG0000	84517	24022
ACVR1	8.42129	8.36348	6.50194	7.05012	6.78131	7.43069	activin A receptor type 1 [Source:HGNC Symbol;Acc:HGNC:171]	chr2:15859	NM_00110	XM_01151	.	.	NP_00109	XP_005246	protein_co	ENSG0000	90	171
ACVR1B	13.1042	12.5309	10.8008	12.2746	11.8573	11.2333	activin A receptor type 1B [Source:HGNC Symbol;Acc:HGNC:172]	chr12:5234	NM_00430	.	.	.	NP_00429	XP_016875	protein_co	ENSG0000	91	172
ACVR1C	0.412555	0.675933	0.778536	0.551422	0.30603	0.418834	activin A receptor type 1C [Source:HGNC Symbol;Acc:HGNC:1812	chr2:15838	NM_00111	.	.	.	NP_00110	.	protein_co	ENSG0000	130399	18123
ACVR2A	5.01805	4.16025	4.46489	3.65097	4.15758	4.75114	activin A receptor type 2A [Source:HGNC Symbol;Acc:HGNC:173]	chr2:14860	NM_00127	.	.	.	NP_00126	.	protein_co	ENSG0000	92	173
ACVR2B	5.34108	5.6394	5.04429	3.60534	4.63959	6.52865	activin A receptor type 2B [Source:HGNC Symbol;Acc:HGNC:174]	chr3:38495	NM_00110	XM_01700	.	.	NP_00109	XP_005265	protein_co	ENSG0000	93	174
ACVRL1	0.630738	0.851947	0.539241	0.548313	0.381051	0.457213	activin A receptor like type 1 [Source:HGNC Symbol;Acc:HGNC:17	chr12:5230	NM_00002	XM_01153	.	.	NP_00001	XP_005269	protein_co	ENSG0000	94	175
ACY1	24.4263	24.9184	33.9378	27.5805	21.4029	25.1632	aminoacylase 1 [Source:HGNC Symbol;Acc:HGNC:177]	chr3:52002	NM_00066	.	.	.	NP_00065	.	protein_co	ENSG0000	95	177
ACY3	0.070747	0.096824	0.503543	0.303187	0	0.07816	aminoacylase 3 [Source:HGNC Symbol;Acc:HGNC:24104]	chr11:6741	NM_08065	XM_01154	.	.	NP_54238	XP_011543	protein_co	ENSG0000	91703	24104
ACYP1	30.0609	29.8342	26.4545	35.488	25.799	24.2153	acylphosphatase 1 [Source:HGNC Symbol;Acc:HGNC:179]	chr14:7551	NM_00110	.	.	.	NP_00109	.	protein_co	ENSG0000	97	179

ACYP2	6.52241	7.20705	7.07334	9.02656	5.27838	6.97932	acylphosphatase 2 [Source:HGNC Symbol;Acc:HGNC:180]	chr2:54091	NM_00132	.	.	.	NP_00130	.	protein_co	ENSG0000	98	180
ADA	14.9693	15.2087	15.7065	16.6892	15.0498	13.5934	adenosine deaminase [Source:HGNC Symbol;Acc:HGNC:186]	chr20:4316	NM_00002	XM_01152	.	.	NP_00001	XP_005260	protein_co	ENSG0000	100	186
ADAD1	0	0	0	0	0	0	adenosine deaminase domain containing 1 [Source:HGNC Symbo	chr4:12330	NM_00115	XM_01153	.	.	NP_00115	XP_005262	protein_co	ENSG0000	132612	30713
ADAD2	0	0	0.042885	0.106062	0.059347	0	adenosine deaminase domain containing 2 [Source:HGNC Symbo	chr16:8422	NM_00114	XM_01152	.	.	NP_00113	XP_005255	protein_co	ENSG0000	161931	30714
ADAL	1.78835	2.1109	2.08457	2.30958	2.3903	1.94238	adenosine deaminase like [Source:HGNC Symbol;Acc:HGNC:3185	chr15:4362	NM_00101	XM_01152	.	.	NP_00101	XP_005254	protein_co	ENSG0000	161823	31853
ADAM10	22.065	20.6263	25.2879	18.1142	21.5962	27.4492	ADAM metallopeptidase domain 10 [Source:HGNC Symbol;Acc:H	chr15:5888	NM_00111	XM_00525	.	.	NP_00110	XP_016877	protein_co	ENSG0000	102	188
ADAM11	2.50282	2.33385	3.98934	2.0881	1.94857	2.24054	ADAM metallopeptidase domain 11 [Source:HGNC Symbol;Acc:H	chr17:4283	NM_00131	XM_01152	.	.	NP_00130	XP_005257	protein_co	ENSG0000	4185	189
ADAM12	0	0	0	0.022029	0	0.009846	ADAM metallopeptidase domain 12 [Source:HGNC Symbol;Acc:H	chr10:1277	NM_00128	XM_01701	.	.	NP_00127	XP_016872	protein_co	ENSG0000	8038	190
ADAM15	74.6205	75.4773	69.8288	57.2771	48.4083	41.2599	ADAM metallopeptidase domain 15 [Source:HGNC Symbol;Acc:H	chr1:15502	NM_00126	.	.	.	NP_00124	.	protein_co	ENSG0000	8751	193
ADAM17	13.0639	13.1566	14.1145	8.54492	11.0238	15.1123	ADAM metallopeptidase domain 17 [Source:HGNC Symbol;Acc:H	chr2:96139	NM_00318	XM_01151	.	.	NP_00317	XP_011508	protein_co	ENSG0000	6868	195
ADAM18	0	0	0	0	0	0	ADAM metallopeptidase domain 18 [Source:HGNC Symbol;Acc:H	chr8:39442	NM_00119	.	.	.	NP_00117	.	protein_co	ENSG0000	8749	196
ADAM19	13.6217	14.1179	43.5715	20.7179	20.5137	27.6877	ADAM metallopeptidase domain 19 [Source:HGNC Symbol;Acc:H	chr5:15690	NM_03327	XM_00526	.	.	NP_15037	XP_005266	protein_co	ENSG0000	8728	197
ADAM2	0	0	0	0	0	0	ADAM metallopeptidase domain 2 [Source:HGNC Symbol;Acc:HG	chr8:39601	NM_00127	XM_01154	.	.	NP_00126	XP_005273	protein_co	ENSG0000	2515	198
ADAM20	0	0.02687	0	0	0	0.024897	ADAM metallopeptidase domain 20 [Source:HGNC Symbol;Acc:H	chr14:7098	NM_00381	XM_01153	.	.	NP_00380	XP_005268	protein_co	ENSG0000	8748	199
ADAM21	1.89263	1.23536	1.10257	1.03745	1.38497	1.04653	ADAM metallopeptidase domain 21 [Source:HGNC Symbol;Acc:H	chr14:7091	NM_00381	.	.	.	NP_00380	.	protein_co	ENSG0000	8747	200
ADAM22	2.66805	2.86785	3.38681	2.16012	2.758	2.68924	ADAM metallopeptidase domain 22 [Source:HGNC Symbol;Acc:H	chr7:87563	NM_00132	XM_01701	.	.	NP_00131	XP_005250	protein_co	ENSG0000	53616	201
ADAM23	0.025352	0	0.065945	0.015063	0	0.013132	ADAM metallopeptidase domain 23 [Source:HGNC Symbol;Acc:H	chr2:20730	NM_00381	XM_01151	.	.	NP_00380	XP_005246	protein_co	ENSG0000	8745	202
ADAM28	0.055727	0	0.117732	0	0.037402	0.01081	ADAM metallopeptidase domain 28 [Source:HGNC Symbol;Acc:H	chr8:24151	NM_00130	XM_01701	.	.	NP_00129	XP_005273	protein_co	ENSG0000	10863	206
ADAM29	0	0	0	0	0	0	ADAM metallopeptidase domain 29 [Source:HGNC Symbol;Acc:H	chr4:17583	NM_00113	XM_01153	.	.	NP_00112	XP_011529	protein_co	ENSG0000	11086	207
ADAM30	0	0	0	0	0	0	ADAM metallopeptidase domain 30 [Source:HGNC Symbol;Acc:H	chr1:12043	NM_02179	.	.	.	NP_06856	.	protein_co	ENSG0000	11085	208
ADAM32	0.391547	0.247579	0.325734	0.032975	0.042155	0.147519	ADAM metallopeptidase domain 32 [Source:HGNC Symbol;Acc:H	chr8:38965	NM_00131	XM_01701	.	.	NP_00130	XP_006716	protein_co	ENSG0000	203102	15479
ADAM33	0	0	0	0	0	0	ADAM metallopeptidase domain 33 [Source:HGNC Symbol;Acc:H	chr20:3648	NM_00128	XM_01152	.	.	NP_00126	XP_005260	protein_co	ENSG0000	80332	15478
ADAM7	0	0	0	0	0	0	ADAM metallopeptidase domain 7 [Source:HGNC Symbol;Acc:HG	chr8:24298	NM_00381	XM_00527	.	.	NP_00380	XP_005273	protein_co	ENSG0000	8756	214
ADAM8	0.383049	0.401922	0.701684	0.348662	0.545296	0.795023	ADAM metallopeptidase domain 8 [Source:HGNC Symbol;Acc:HG	chr10:1350	NM_00110	XM_01153	.	.	NP_00110	XP_011537	protein_co	ENSG0000	101	215
ADAM9	60.5181	63.972	66.8306	60.2718	63.0554	84.5314	ADAM metallopeptidase domain 9 [Source:HGNC Symbol;Acc:HG	chr8:38854	NM_00381	XM_01701	.	.	NP_00380	XP_011542	protein_co	ENSG0000	8754	216
ADAMDEC1	0	0	0	0	0	0	ADAM like decysin 1 [Source:HGNC Symbol;Acc:HGNC:16299]	chr8:24241	NM_00114	.	.	.	NP_00113	.	protein_co	ENSG0000	27299	16299
ADAMTS1	0	0	0	0	0	0	ADAM metallopeptidase with thrombospondin type 1 motif 1 [So	chr21:2820	NM_00698	.	.	.	NP_00891	.	protein_co	ENSG0000	9510	217
ADAMTS10	1.58522	1.76638	2.71995	0.962425	1.23919	1.68319	ADAM metallopeptidase with thrombospondin type 1 motif 10 [S	chr19:8645	NM_00128	XM_01702	.	.	NP_00126	XP_006722	protein_co	ENSG0000	81794	13201
ADAMTS12	0	0	0	0	0	0	ADAM metallopeptidase with thrombospondin type 1 motif 12 [S	chr5:33523	NM_03095	XM_01151	.	.	NP_00131	.	protein_co	ENSG0000	81792	14605
ADAMTS13	2.76232	1.83452	3.98449	1.56995	1.62476	1.7503	ADAM metallopeptidase with thrombospondin type 1 motif 13 [S	chr9:13628	NM_13902	XM_01151	NR_02451	.	NP_62059	XP_016869	protein_co	ENSG0000	11093	1366
ADAMTS14	4.6111	4.51669	8.66742	2.49949	2.43465	2.14537	ADAM metallopeptidase with thrombospondin type 1 motif 14 [S	chr10:7243	NM_08072	XM_01153	.	.	NP_54245	XP_011537	protein_co	ENSG0000	140766	14899
ADAMTS15	0.255454	0.521899	0.393356	0.653397	0.606244	0.715837	ADAM metallopeptidase with thrombospondin type 1 motif 15 [S	chr11:1303	NM_13905	XM_00527	.	.	NP_62068	XP_005271	protein_co	ENSG0000	170689	16305
ADAMTS16	0.567373	0.587654	0.400803	0.207888	0.149449	0.244469	ADAM metallopeptidase with thrombospondin type 1 motif 16 [S	chr5:51404	NM_13905	.	.	.	NP_62068	.	protein_co	ENSG0000	170690	17108
ADAMTS17	0.566065	0.694472	0.728841	0.600621	0.668022	0.69417	ADAM metallopeptidase with thrombospondin type 1 motif 17 [S	chr15:1005	NM_13905	XM_01152	.	.	NP_62068	XP_005254	protein_co	ENSG0000	170691	17109
ADAMTS18	0	0	0	0	0	0	ADAM metallopeptidase with thrombospondin type 1 motif 18 [S	chr16:7731	NM_00132	XM_01152	.	.	NP_00131	XP_011521	protein_co	ENSG0000	170692	17110
ADAMTS19	0	0	0	0	0	0	ADAM metallopeptidase with thrombospondin type 1 motif 19 [S	chr5:12879	NM_13363	XM_01154	.	.	NP_59837	XP_011541	protein_co	ENSG0000	171019	17111
ADAMTS2	0.935759	1.15849	1.52142	0.631874	0.838515	1.17776	ADAM metallopeptidase with thrombospondin type 1 motif 2 [So	chr5:17853	NM_01424	XM_01701	.	.	NP_05505	XP_016865	protein_co	ENSG0000	9509	218
ADAMTS20	0	0.033872	0	0	0.011884	0	ADAM metallopeptidase with thrombospondin type 1 motif 20 [S	chr12:4374	NM_02500	XM_01153	.	.	NP_07927	XP_011537	protein_co	ENSG0000	80070	17178
ADAMTS3	0	0	0	0	0	0	ADAM metallopeptidase with thrombospondin type 1 motif 3 [So	chr4:73146	NM_01424	XM_01153	.	.	NP_05505	XP_011530	protein_co	ENSG0000	9508	219
ADAMTS4	0	0.113746	0.033409	0.008179	0.089029	0.007721	ADAM metallopeptidase with thrombospondin type 1 motif 4 [So	chr1:16115	NM_00509	XM_00671	.	.	NP_00509	.	protein_co	ENSG0000	9507	220
ADAMTS5	0	0	0	0	0	0	ADAM metallopeptidase with thrombospondin type 1 motif 5 [So	chr21:2829	NM_00703	XM_01152	.	.	NP_00896	XP_011527	protein_co	ENSG0000	11096	221
ADAMTS6	0.182745	0.107848	0.167475	0.126379	0.13607	0.241331	ADAM metallopeptidase with thrombospondin type 1 motif 6 [So	chr5:64444	NM_19794	XM_01154	.	.	NP_92293	XP_011541	protein_co	ENSG0000	11174	222
ADAMTS7	3.14328	3.55137	6.21025	4.22513	3.20523	3.48945	ADAM metallopeptidase with thrombospondin type 1 motif 7 [So	chr15:7905	NM_01427	XM_00525	.	.	NP_05508	XP_005254	protein_co	ENSG0000	11173	223
ADAMTS8	0	0.020615	0	0	0	0	ADAM metallopeptidase with thrombospondin type 1 motif 8 [So	chr11:1302	NM_00703	.	.	.	NP_00896	XP_016872	protein_co	ENSG0000	11095	224
ADAMTS9	0	0	0	0	0	0	ADAM metallopeptidase with thrombospondin type 1 motif 9 [So	chr3:64501	NM_00131	XM_00526	.	.	NP_00130	XP_005265	protein_co	ENSG0000	56999	13202
ADAMTSL1	0.049937	0	0	0	0	0	ADAMTS like 1 [Source:HGNC Symbol;Acc:HGNC:14632]	chr9:18473	NM_00104	XM_01701	.	.	NP_00103	XP_011516	protein_co	ENSG0000	92949	14632
ADAMTSL2	0	0	0	0.022654	0	0.02417	ADAMTS like 2 [Source:HGNC Symbol;Acc:HGNC:14631]	chr9:13639	NM_00114	XM_01151	.	.	NP_00113	XP_005272	protein_co	ENSG0000	9719	14631
ADAMTSL3	0	0.009757	0	0	0	0	ADAMTS like 3 [Source:HGNC Symbol;Acc:HGNC:14633]	chr15:8432	NM_00130	XM_01152	.	.	NP_00128	XP_011520	protein_co	ENSG0000	57188	14633
ADAMTSL4	0.285738	0.453357	0.791878	0.362159	0.283943	0.31531	ADAMTS like 4 [Source:HGNC Symbol;Acc:HGNC:19706]	chr1:15052	NM_00128	XM_01150	.	.	NP_00127	XP_011507	protein_co	ENSG0000	54507	19706
ADAMTSL5	5.13523	5.58043	6.49657	3.62673	3.86102	4.10627	ADAMTS like 5 [Source:HGNC Symbol;Acc:HGNC:27912]	chr19:1505	NM_21360	XM_01702	.	.	NP_99876	XP_005259	protein_co	ENSG0000	339366	27912
ADAP1	13.2594	15.0243	12.8003	16.1389	12.7507	11.4242	ArfGAP with dual PH domains 1 [Source:HGNC Symbol;Acc:HGNC	chr7:93753	NM_00128	.	.	.	NP_00127	.	protein_co	ENSG0000	11033	16486
ADAP2	6.10211	6.03691	7.69248	7.28978	6.65882	5.55709	ArfGAP with dual PH domains 2 [Source:HGNC Symbol;Acc:HGNC	chr17:2924	NM_01840	XM_01152	.	.	NP_06087	XP_005258	protein_co	ENSG0000	55803	16487
ADAR	73.3445	77.9735	70.6177	71.1481	75.1308	68.9065	adenosine deaminase, RNA specific [Source:HGNC Symbol;Acc:H	chr1:15455	NM_00102	XM_01150	.	.	NP_00102	XP_006711	protein_co	ENSG0000	103	225
ADARB1	11.1118	4.66353	5.24818	3.36887	4.03781	4.12074	adenosine deaminase, RNA specific B1 [Source:HGNC Symbol;Acc	chr21:4649	NM_00111	XM_01702	NR_02767	.	NP_00110	XP_006724	protein_co	ENSG0000	104	226
ADARB2	0	0	0	0	0.011049	0	adenosine deaminase, RNA specific B2 (inactive) [Source:HGNC S	chr10:1223	NM_01870	.	.	.	NP_06117	.	protein_co	ENSG0000	105	227
ADAT1	5.23547	5.81144	6.87335	5.2202	5.15351	4.106	adenosine deaminase, tRNA specific 1 [Source:HGNC Symbol;Acc	chr16:7563	NM_01209	.	.	.	NP_00131	.	protein_co	ENSG0000	23536	228
ADAT2	9.6832	9.05244	9.88752	9.11919	10.3437	7.144	adenosine deaminase, tRNA specific 2 [Source:HGNC Symbol;Acc	chr6:14374	NM_00128	XM_01701	.	.	NP_00127	XP_011533	protein_co	ENSG0000	134637	21172
ADAT3	7.05152	6.05941	8.29206	6.97555	5.19815	7.28968	adenosine deaminase, tRNA specific 3 [Source:HGNC Symbol;Acc	chr19:1905	NM_13842	.	.	.	NP_00131	.	protein_co	ENSG0000	113179	25151
ADCK1	3.71894	4.40846	4.1971	4.25451	2.92806	4.40833	aarF domain containing kinase 1 [Source:HGNC Symbol;Acc:HGN	chr14:7826	NM_00114	XM_00526	.	.	NP_00113	XP_005267	protein_co	ENSG0000	57143	19038

ADCK2	9.58606	9.7611	9.68034	10.1792	8.91937	9.56495	aarF domain containing kinase 2 [Source:HGNC Symbol;Acc:HGN(	chr7:14037	NM_05285	XM_01151	.	.	NP_44308	XP_006716	protein_co	ENSG0000(	90956	19039
ADCK3	18.8358	19.6331	19.6223	16.1926	13.5204	14.478	coenzyme Q8A [Source:HGNC Symbol;Acc:HGNC:16812]	chr1:22708	NM_02024	XM_01154	.	.	NP_06463	XP_005273	protein_co	ENSG0000(	56997	16812
ADCK4	12.6493	12.2069	10.3218	9.44281	9.06285	9.7324	coenzyme Q8B [Source:HGNC Symbol;Acc:HGNC:19041]	chr19:4119	NM_00114	XM_01152	.	.	NP_00113	XP_005259	protein_co	ENSG0000(	79934	19041
ADCK5	9.63871	9.86471	10.352	9.55361	9.46045	7.75214	aarF domain containing kinase 5 [Source:HGNC Symbol;Acc:HGN(	chr8:14559	NM_17492	XM_01701	.	.	NP_77758	XP_006716	protein_co	ENSG0000(	203054	21738
ADCY1	0	0	0	0	0	0	adenylate cyclase 1 [Source:HGNC Symbol;Acc:HGNC:232]	chr7:45613	NM_00128	XM_00524	.	.	NP_00126	XP_005249	protein_co	ENSG0000(	107	232
ADCY10	0	0	0.118897	0.304168	0	0.013353	adenylate cyclase 10, soluble [Source:HGNC Symbol;Acc:HGNC:2	chr1:16777	NM_00116	XM_01150	.	.	NP_00116	XP_006711	protein_co	ENSG0000(	55811	21285
ADCY2	0	0	0	0	0	0	adenylate cyclase 2 [Source:HGNC Symbol;Acc:HGNC:233]	chr5:73963	NM_02054	XM_01151	.	.	NP_06543	XP_011512	protein_co	ENSG0000(	108	233
ADCY3	14.0929	13.5136	15.0092	11.7688	11.88	9.46009	adenylate cyclase 3 [Source:HGNC Symbol;Acc:HGNC:234]	chr2:25012	NM_00132	XM_01153	.	.	NP_00130	XP_005264	protein_co	ENSG0000(	109	234
ADCY4	0	0.023283	0.020144	0	0	0.02567	adenylate cyclase 4 [Source:HGNC Symbol;Acc:HGNC:235]	chr14:2478	NM_00119	.	.	.	NP_00118	.	protein_co	ENSG0000(	196883	235
ADCY5	0	0.026389	0.118962	0.14861	0.053289	0.091037	adenylate cyclase 5 [Source:HGNC Symbol;Acc:HGNC:236]	chr3:12300	NM_00119	XM_01151	.	.	NP_00118	XP_005247	protein_co	ENSG0000(	111	236
ADCY6	6.76779	7.4432	7.50469	5.74864	6.28807	6.16118	adenylate cyclase 6 [Source:HGNC Symbol;Acc:HGNC:237]	chr12:4919	NM_01527	XM_01153	.	.	NP_05608	XP_005268	protein_co	ENSG0000(	112	237
ADCY7	9.18062	9.30636	9.10827	7.19306	8.58654	6.51881	adenylate cyclase 7 [Source:HGNC Symbol;Acc:HGNC:238]	chr16:5030	NM_00111	XM_01702	.	.	NP_00110	XP_005255	protein_co	ENSG0000(	113	238
ADCY8	0	0	0	0	0.016353	0	adenylate cyclase 8 [Source:HGNC Symbol;Acc:HGNC:239]	chr8:13179	NM_00111	XM_00671	.	.	NP_00110	XP_005250	protein_co	ENSG0000(	114	239
ADCY9	2.98135	3.53072	3.88682	2.651	2.54539	3.41177	adenylate cyclase 9 [Source:HGNC Symbol;Acc:HGNC:240]	chr16:4012	NM_00111	XM_00525	.	.	NP_00110	XP_005255	protein_co	ENSG0000(	115	240
ADCYAP1	0	0	0	0	0	0	adenylate cyclase activating polypeptide 1 [Source:HGNC Symbol	chr18:9049	NM_00109	.	.	.	NP_00109	.	protein_co	ENSG0000(	116	241
ADCYAP1R1	0.023096	0.07285	0.042178	0.047407	0.05227	0.099453	ADCYAP receptor type I [Source:HGNC Symbol;Acc:HGNC:242]	chr7:31092	NM_00111	XM_01151	.	.	NP_00110	XP_005249	protein_co	ENSG0000(	117	242
ADD1	38.3146	40.2376	36.2275	34.8928	34.1291	30.9119	adducin 1 [Source:HGNC Symbol;Acc:HGNC:243]	chr4:28455	NM_00111	XM_01700	.	.	NP_00111	XP_005247	protein_co	ENSG0000(	118	243
ADD2	0.007407	0.016287	0.019644	0	0	0	adducin 2 [Source:HGNC Symbol;Acc:HGNC:244]	chr2:70883	NM_00118	XM_01153	.	.	NP_00117	XP_011530	protein_co	ENSG0000(	119	244
ADD3	14.3666	15.7473	19.2076	20.0589	23.3922	24.9042	adducin 3 [Source:HGNC Symbol;Acc:HGNC:245]	chr10:1117	NM_00112	XM_01153	.	.	NP_00111	XP_005269	protein_co	ENSG0000(	120	245
ADGB	0	0.025851	0	0	0	0	androglobin [Source:HGNC Symbol;Acc:HGNC:21212]	chr6:14692	NM_02469	XM_01701	.	.	NP_07897	XP_006715	protein_co	ENSG0000(	79747	21212
ADGRA1	0	0	0	0.023483	0	0	adhesion G protein-coupled receptor A1 [Source:HGNC Symbol;A	chr10:1348	NM_00108	XM_00525	.	.	NP_00107	XP_005252	protein_co	ENSG0000(	84435	13838
ADGRA2	3.22873	2.78245	4.22195	2.79049	2.82268	4.39732	adhesion G protein-coupled receptor A2 [Source:HGNC Symbol;A	chr8:37654	NM_03277	XM_01154	.	.	NP_11616	XP_011542	protein_co	ENSG0000(	25960	17849
ADGRA3	25.7906	24.0378	24.5367	20.8728	22.7781	19.3663	adhesion G protein-coupled receptor A3 [Source:HGNC Symbol;A	chr4:22388	NM_14529	XM_01151	.	.	NP_66033	.	protein_co	ENSG0000(	166647	13839
ADGRB1	0.524443	0.630807	1.15398	0.606068	0.661514	0.716368	adhesion G protein-coupled receptor B1 [Source:HGNC Symbol;A	chr8:14353	NM_00170	XM_01151	.	.	NP_00169	XP_011515	protein_co	ENSG0000(	575	943
ADGRB2	4.01409	4.02829	5.9673	4.32349	4.77379	4.07258	adhesion G protein-coupled receptor B2 [Source:HGNC Symbol;A	chr1:32192	NM_00129	XM_01700	.	.	NP_00128	XP_011540	protein_co	ENSG0000(	576	944
ADGRB3	0	0.054968	0	0	0	0	adhesion G protein-coupled receptor B3 [Source:HGNC Symbol;A	chr6:69345	NM_00170	XM_01153	.	.	NP_00169	XP_005248	protein_co	ENSG0000(	577	945
ADGRD1	0.408858	0.295495	0.401959	0.191141	0.565288	0.217654	adhesion G protein-coupled receptor D1 [Source:HGNC Symbol;A	chr12:1314	NM_19882	XM_01153	.	.	NP_94212	XP_005253	protein_co	ENSG0000(	283383	19893
ADGRD2	0	0	0	0	0	0	adhesion G protein-coupled receptor D2 [Source:HGNC Symbol;A	chr9:12721	.	.	.	.	.	.	protein_co	ENSG0000(	.	18651
ADGRE1	0	0.026449	0	0.030596	0	0	adhesion G protein-coupled receptor E1 [Source:HGNC Symbol;A	chr19:6887	NM_00125	XM_01152	.	.	NP_00124	XP_011526	protein_co	ENSG0000(	2015	3336
ADGRE2	3.63243	3.78452	4.19721	3.03741	3.58429	3.92281	adhesion G protein-coupled receptor E2 [Source:HGNC Symbol;A	chr19:1480	NM_00127	XM_01152	.	.	NP_00125	XP_011526	protein_co	ENSG0000(	30817	3337
ADGRE3	0	0	0	0	0	0	adhesion G protein-coupled receptor E3 [Source:HGNC Symbol;A	chr19:1472	NM_00128	XM_01152	.	.	NP_00127	XP_011526	protein_co	ENSG0000(	84658	23647
ADGRE5	27.6035	29.1734	30.3065	24.8329	24.0016	28.6856	adhesion G protein-coupled receptor E5 [Source:HGNC Symbol;A	chr19:1449	NM_00102	XM_01702	.	.	NP_00102	XP_011526	protein_co	ENSG0000(	976	1711
ADGRF1	47.4043	45.3143	38.6684	40.7797	44.1057	51.0643	adhesion G protein-coupled receptor F1 [Source:HGNC Symbol;A	chr6:46965	NM_02504	XM_01701	.	.	NP_07932	XP_005249	protein_co	ENSG0000(	266977	18990
ADGRF2	0.015339	0	0	0	0.11553	0.030443	adhesion G protein-coupled receptor F2 [Source:HGNC Symbol;A	chr6:47624	NM_15383	.	.	.	NP_72258	.	protein_co	ENSG0000(	222611	18991
ADGRF3	0	0.0417	0	0.026013	0	0	adhesion G protein-coupled receptor F3 [Source:HGNC Symbol;A	chr2:26531	NM_00114	XM_01153	.	.	NP_00113	XP_011530	protein_co	ENSG0000(	165082	18989
ADGRF4	0.023177	0	0.060397	0	0	0.023191	adhesion G protein-coupled receptor F4 [Source:HGNC Symbol;A	chr6:47624	NM_15383	.	.	.	NP_72258	.	protein_co	ENSG0000(	221393	19011
ADGRF5	0	0	0	0	0	0	adhesion G protein-coupled receptor F5 [Source:HGNC Symbol;A	chr6:46820	NM_00109	XM_01701	.	.	NP_00109	XP_005248	protein_co	ENSG0000(	221395	19030
ADGRG1	14.3275	15.2358	13.8636	12.4951	10.9362	18.1802	adhesion G protein-coupled receptor G1 [Source:HGNC Symbol;A	chr16:5765	NM_00114	XM_01702	.	.	NP_00113	XP_005256	protein_co	ENSG0000(	9289	4512
ADGRG2	0	0	0.014785	0	0	0	adhesion G protein-coupled receptor G2 [Source:HGNC Symbol;A	chrX:19007	NM_00107	XM_01154	.	.	NP_00107	XP_006724	protein_co	ENSG0000(	10149	4516
ADGRG3	0	0	0.047021	0	0.031309	0.029516	adhesion G protein-coupled receptor G3 [Source:HGNC Symbol;A	chr16:5770	NM_00130	XM_01152	.	.	NP_00129	XP_005255	protein_co	ENSG0000(	222487	13728
ADGRG4	0	0	0	0	0	0.007921	adhesion G protein-coupled receptor G4 [Source:HGNC Symbol;A	chrX:13538	NM_15383	XM_01153	.	.	NP_72257	XP_011529	protein_co	ENSG0000(	139378	18992
ADGRG5	2.36267	2.42044	2.5063	2.55165	2.3436	2.35127	adhesion G protein-coupled receptor G5 [Source:HGNC Symbol;A	chr16:5757	NM_00130	XM_01152	.	.	NP_00129	XP_006721	protein_co	ENSG0000(	221188	19010
ADGRG6	14.7917	14.5737	20.3809	9.93703	13.7361	19.8795	adhesion G protein-coupled receptor G6 [Source:HGNC Symbol;A	chr6:14262	NM_00103	XM_01153	.	.	NP_00102	XP_005267	protein_co	ENSG0000(	57211	13841
ADGRG7	0	0.048701	0.023126	0	0.100911	0	adhesion G protein-coupled receptor G7 [Source:HGNC Symbol;A	chr3:10032	NM_00130	XM_01151	.	.	NP_00129	XP_005247	protein_co	ENSG0000(	84873	19241
ADGRL1	20.1485	20.5519	20.8918	17.0985	17.2555	14.5619	adhesion G protein-coupled receptor L1 [Source:HGNC Symbol;A	chr19:1425	NM_00100	XM_01702	.	.	NP_00100	XP_005259	protein_co	ENSG0000(	22859	20973
ADGRL2	0.014303	0.015167	0	0	0.053681	0.038502	adhesion G protein-coupled receptor L2 [Source:HGNC Symbol;A	chr1:81771	NM_00129	XM_01700	.	.	NP_00128	XP_005270	protein_co	ENSG0000(	23266	18582
ADGRL3	0	0	0	0	0	0	adhesion G protein-coupled receptor L3 [Source:HGNC Symbol;A	chr4:62066	NM_00132	XM_01700	.	.	NP_00130	XP_011530	protein_co	ENSG0000(	23284	20974
ADGRL4	0	0	0	0	0	0	adhesion G protein-coupled receptor L4 [Source:HGNC Symbol;A	chr1:79355	NM_02215	.	.	.	NP_07144	.	protein_co	ENSG0000(	64123	20822
ADGRV1	0.584563	0.423054	0.723659	0.477054	0.457467	0.602194	adhesion G protein-coupled receptor V1 [Source:HGNC Symbol;A	chr5:89854	NM_03211	XM_01700	.	.	NP_11549	XP_016865	protein_co	ENSG0000(	84059	17416
ADH1A	0	0	0	0	0	0	alcohol dehydrogenase 1A (class I), alpha polypeptide [Source:HG	chr4:10019	NM_00066	.	.	.	NP_00065	.	protein_co	ENSG0000(	124	249
ADH1B	0	0	0.05274	0	0	0	alcohol dehydrogenase 1B (class I), beta polypeptide [Source:HGI	chr4:10022	NM_00066	.	.	.	NP_00065	.	protein_co	ENSG0000(	125	250
ADH1C	0	0.047175	0	0.050356	0	0.045486	alcohol dehydrogenase 1C (class I), gamma polypeptide [Source:H	chr4:10025	NM_00066	XM_01153	.	.	NP_00066	XP_011529	protein_co	ENSG0000(	126	251
ADH4	0	0	0	0	0	0	alcohol dehydrogenase 4 (class II), pi polypeptide [Source:HGNC S	chr4:10004	NM_00067	XM_01153	.	.	NP_00066	XP_011529	protein_co	ENSG0000(	127	252
ADH5	108.114	96.7662	102.555	138.491	125.839	128.841	alcohol dehydrogenase 5 (class III), chi polypeptide [Source:HGN(	chr4:99992	NM_00067	.	.	.	NP_00066	.	protein_co	ENSG0000(	128	253
ADH6	0	0	0	0	0	0	alcohol dehydrogenase 6 (class V) [Source:HGNC Symbol;Acc:HGI	chr4:10012	NM_00067	XM_00526	.	.	NP_00066	XP_005262	protein_co	ENSG0000(	130	255
ADH7	0	0	0	0	0	0	alcohol dehydrogenase 7 (class IV), mu or sigma polypeptide [Sou	chr4:10033	NM_00067	.	.	.	NP_00066	.	protein_co	ENSG0000(	131	256
ADHFE1	0.335739	0.17469	0.424416	0.186027	0.23698	0.171996	alcohol dehydrogenase, iron containing 1 [Source:HGNC Symbol;	chr8:67344	NM_14465	.	.	.	NP_65325	.	protein_co	ENSG0000(	137872	16354
ADI1	33.9852	36.1515	26.1287	34.4634	29.4357	20.3904	acireductone dioxygenase 1 [Source:HGNC Symbol;Acc:HGNC:30	chr2:35011	NM_00130	XM_00526	.	.	NP_00129	XP_005264	protein_co	ENSG0000(	55256	30576
ADIG	0	0	0	0	0	0	adipogenin [Source:HGNC Symbol;Acc:HGNC:28606]	chr20:3720	NM_00101	.	.	.	NP_00101	.	protein_co	ENSG0000(	149685	28606

ADIPOQ	0	0	0	0	0	0	adiponectin, C1Q and collagen domain containing [Source:HGNC	chr3:18656	NM_00117	XM_01151	.	.	NP_00117	XP_011511	protein_co	ENSG0000	9370	13633
ADIPOR1	71.8745	67.0404	64.8551	74.8126	67.2091	67.2195	adiponectin receptor 1 [Source:HGNC Symbol;Acc:HGNC:24040]	chr1:20290	NM_00129	.	.	.	NP_00127	.	protein_co	ENSG0000	51094	24040
ADIPOR2	39.692	41.9928	40.7477	45.0909	46.8922	52.2743	adiponectin receptor 2 [Source:HGNC Symbol;Acc:HGNC:24041]	chr12:1800	NM_02455	XM_01152	.	.	NP_07882	XP_005253	protein_co	ENSG0000	79602	24041
ADIRF	90.6691	89.3005	162.022	137.427	89.3757	158.96	adipogenesis regulatory factor [Source:HGNC Symbol;Acc:HGNC:2	chr10:8872	NM_00682	.	.	.	NP_00682	.	protein_co	ENSG0000	10974	24043
ADK	33.5573	29.4313	33.7416	35.613	36.9701	46.4803	adenosine kinase [Source:HGNC Symbol;Acc:HGNC:257]	chr10:7591	NM_00112	XM_01153	.	.	NP_00111	XP_016871	protein_co	ENSG0000	132	257
ADM	44.4955	50.0916	54.5616	52.3684	45.1484	30.8491	adrenomedullin [Source:HGNC Symbol;Acc:HGNC:259]	chr11:1032	NM_00112	.	.	.	NP_00111	.	protein_co	ENSG0000	133	259
ADM2	3.55978	3.27339	3.30941	1.77185	1.53888	5.87195	adrenomedullin 2 [Source:HGNC Symbol;Acc:HGNC:28898]	chr22:5091	NM_00125	XM_01702	.	.	NP_00124	XP_016884	protein_co	ENSG0000	79924	28898
ADM5	5.30616	5.81241	4.09223	12.9578	10.2563	8.08736	adrenomedullin 5 (putative) [Source:HGNC Symbol;Acc:HGNC:27	chr19:5019	NM_00110	.	.	.	NP_00109	.	protein_co	ENSG0000	199800	27293
ADNP	34.3569	36.4338	32.1133	35.1302	38.8586	39.886	activity dependent neuroprotector homeobox [Source:HGNC Sym	chr20:4950	NM_00128	XM_01702	.	.	NP_00126	XP_011527	protein_co	ENSG0000	23394	15766
ADNP2	22.0594	24.7498	22.4094	24.4969	26.3052	25.9708	ADNP homeobox 2 [Source:HGNC Symbol;Acc:HGNC:23803]	chr18:7786	NM_01491	XM_00526	.	.	NP_05572	XP_00526	protein_co	ENSG0000	22850	23803
ADO	16.3041	17.6725	19.6539	19.055	21.4438	20.7677	2-aminoethanethiol dioxygenase [Source:HGNC Symbol;Acc:HGN	chr10:6456	NM_03280	.	.	.	NP_11619	.	protein_co	ENSG0000	84890	23506
ADORA1	2.84944	2.6984	2.37797	3.59206	3.24982	1.51505	adenosine A1 receptor [Source:HGNC Symbol;Acc:HGNC:262]	chr1:20305	NM_00067	XM_01150	.	.	NP_00066	XP_005244	protein_co	ENSG0000	134	262
ADORA2A	2.4332	2.16642	2.49343	4.31731	3.55598	3.04434	adenosine A2a receptor [Source:HGNC Symbol;Acc:HGNC:263]	chr22:2481	NM_00067	.	.	.	NP_00066	.	protein_co	ENSG0000	135	263
ADORA2B	38.2373	32.1693	38.4207	33.6746	32.1805	26.2536	adenosine A2b receptor [Source:HGNC Symbol;Acc:HGNC:264]	chr17:1584	NM_00067	.	.	.	NP_00066	.	protein_co	ENSG0000	136	264
ADPGK	26.3372	29.2333	24.0085	33.4781	31.3288	23.3957	ADP dependent glucokinase [Source:HGNC Symbol;Acc:HGNC:25	chr15:7304	NM_03128	XM_00672	.	.	NP_11257	XP_00672	protein_co	ENSG0000	83440	25250
ADPRH	2.01929	1.79055	1.71439	1.24857	1.69303	1.30438	ADP-ribosylarginine hydrolase [Source:HGNC Symbol;Acc:HGNC:2	chr3:11929	NM_00112	XM_01151	.	.	NP_00111	XP_01151	protein_co	ENSG0000	141	269
ADPRHL1	7.0823	6.18961	7.23741	6.32207	4.81163	6.77658	ADP-ribosylhydrolase like 1 [Source:HGNC Symbol;Acc:HGNC:213	chr13:1140	NM_13843	.	.	.	NP_61243	.	protein_co	ENSG0000	113622	21303
ADPRHL2	25.8585	25.6044	22.3375	30.8868	27.7551	21.3513	ADP-ribosylhydrolase like 2 [Source:HGNC Symbol;Acc:HGNC:213	chr1:36554	NM_01782	.	.	.	NP_06029	.	protein_co	ENSG0000	54936	21304
ADPRM	5.40726	5.03252	5.67076	4.62122	4.87875	5.65749	ADP-ribose/CDP-alcohol diphosphatase, manganese dependent [	chr17:1060	NM_02023	XM_00525	.	.	NP_06461	XP_005256	protein_co	ENSG0000	56985	30925
ADRA1A	0.085001	0	0	0	0	0	adrenoceptor alpha 1A [Source:HGNC Symbol;Acc:HGNC:277]	chr8:26605	NM_00068	XM_00671	.	.	NP_00067	XP_005273	protein_co	ENSG0000	148	277
ADRA1B	0	0	0	0	0.06591	0.125117	adrenoceptor alpha 1B [Source:HGNC Symbol;Acc:HGNC:278]	chr5:15934	NM_00067	.	.	.	NP_00067	.	protein_co	ENSG0000	147	278
ADRA1D	0.045667	0	0.038796	0	0	0.051835	adrenoceptor alpha 1D [Source:HGNC Symbol;Acc:HGNC:280]	chr20:4201	NM_00067	.	.	.	NP_00066	.	protein_co	ENSG0000	146	280
ADRA2A	0.065487	0.032403	0.055417	0.035484	0	0.03539	adrenoceptor alpha 2A [Source:HGNC Symbol;Acc:HGNC:281]	chr10:1128	NM_00068	.	.	.	NP_00067	.	protein_co	ENSG0000	150	281
ADRA2B	0.430757	0.303104	0.437392	0.246624	0.204287	0.46539	adrenoceptor alpha 2B [Source:HGNC Symbol;Acc:HGNC:282]	chr2:96778	NM_00068	.	.	.	NP_00067	.	protein_co	ENSG0000	151	282
ADRA2C	19.3182	18.4045	19.5049	17.9771	14.367	14.5211	adrenoceptor alpha 2C [Source:HGNC Symbol;Acc:HGNC:283]	chr4:37680	NM_00068	.	.	.	NP_00067	.	protein_co	ENSG0000	152	283
ADRB1	0.610983	0.690159	1.21364	0.580542	1.08086	1.33689	adrenoceptor beta 1 [Source:HGNC Symbol;Acc:HGNC:285]	chr10:1158	NM_00068	.	.	.	NP_00067	.	protein_co	ENSG0000	153	285
ADRB2	0.185675	0.163531	0.296169	0.146792	0.21079	0.301243	adrenoceptor beta 2 [Source:HGNC Symbol;Acc:HGNC:286]	chr5:14820	NM_00002	.	.	.	NP_00001	.	protein_co	ENSG0000	154	286
ADRB3	0	0	0	0	0	0	adrenoceptor beta 3 [Source:HGNC Symbol;Acc:HGNC:288]	chr8:37820	NM_00002	.	.	.	NP_00001	.	protein_co	ENSG0000	155	288
ADRM1	158.8	159.856	124.385	215.452	172.188	122.91	adhesion regulating molecule 1 [Source:HGNC Symbol;Acc:HGNC	chr20:6087	NM_00128	XM_01702	.	.	NP_00126	XP_005260	protein_co	ENSG0000	11047	15759
ADSL	73.1198	68.107	63.0133	94.9825	75.5892	63.7252	adenylosuccinate lyase [Source:HGNC Symbol;Acc:HGNC:291]	chr22:4074	NM_00002	XM_01152	.	.	NP_00001	XP_011528	protein_co	ENSG0000	158	291
ADSS	25.5354	26.8247	25.7907	27.8712	35.7143	33.0313	adenylosuccinate synthase [Source:HGNC Symbol;Acc:HGNC:292]	chr1:24457	NM_00112	XM_01154	.	.	NP_00111	XP_011542	protein_co	ENSG0000	159	292
ADSSL1	2.02654	2.3062	3.78083	2.44063	1.52725	4.21636	adenylosuccinate synthase like 1 [Source:HGNC Symbol;Acc:HGN	chr14:1051	NM_00132	XM_01153	.	.	NP_00130	XP_006720	protein_co	ENSG0000	122622	20093
ADTRP	0.036747	0.282458	0.230271	0.110286	0.040584	0.217353	androgen dependent TFPI regulating protein [Source:HGNC Symb	chr6:11713	NM_00114	XM_01151	.	.	NP_00113	XP_005249	protein_co	ENSG0000	84830	21214
AEBP1	0.026409	0	0	0	0.060858	0	AE binding protein 1 [Source:HGNC Symbol;Acc:HGNC:303]	chr7:44143	NM_00112	XM_01151	NR_03979	.	NP_00112	XP_011513	protein_co	ENSG0000	10061634	303
AEBP2	14.9119	13.7245	14.9792	14.9366	16.6108	20.8307	AE binding protein 2 [Source:HGNC Symbol;Acc:HGNC:24051]	chr12:1955	NM_00111	XM_00671	.	.	NP_00110	XP_006719	protein_co	ENSG0000	121536	24051
AEN	36.9743	38.2002	36.4326	49.3731	42.3891	31.1306	apoptosis enhancing nuclease [Source:HGNC Symbol;Acc:HGNC:2	chr15:8916	NM_02276	XM_01152	.	.	NP_07360	XP_005255	protein_co	ENSG0000	64782	25722
AES	118.577	108.173	117.762	132.633	105.633	125.552	amino-terminal enhancer of split [Source:HGNC Symbol;Acc:HGN	chr19:3052	NM_00113	XM_01152	.	.	NP_00112	XP_006722	protein_co	ENSG0000	166	307
AF011889.5	4.11E-06	1.59E-06	3.94E-06	3.07E-06	4.27E-06	2.98E-06	.	chrX:14855	.	.	.	.	.	.	protein_co	ENSG0000	.	.
AF131215.5	0	0	0	0	0	0	.	chr8:10754	.	.	.	.	.	.	protein_co	ENSG0000	.	.
AF165138.7	0.109324	0	0	0	0	0	.	chr21:1596	NM_00125	.	.	.	NP_00124	.	protein_co	ENSG0000	.	.
AFAP1	6.13727	6.81294	7.07485	7.34282	7.71051	8.16001	actin filament associated protein 1 [Source:HGNC Symbol;Acc:HG	chr4:77604	NM_00113	XM_01151	.	.	NP_00112	XP_006713	protein_co	ENSG0000	60312	24017
AFAP1L1	1.58601	2.38291	2.57101	3.38888	3.22008	2.42466	actin filament associated protein 1 like 1 [Source:HGNC Symbol;A	chr5:14865	NM_00114	XM_00526	.	.	NP_00113	XP_016864	protein_co	ENSG0000	134265	26714
AFAP1L2	0.54362	0.484957	0.875659	0.261141	0.388606	0.699287	actin filament associated protein 1 like 2 [Source:HGNC Symbol;A	chr10:1160	NM_00100	XM_01701	.	.	NP_00100	XP_005270	protein_co	ENSG0000	84632	25901
AFF1	6.26177	6.38079	8.45079	5.61191	7.20696	7.69864	AF4/FMR2 family member 1 [Source:HGNC Symbol;Acc:HGNC:71	chr4:87856	NM_00116	XM_01153	.	.	NP_00116	XP_005263	protein_co	ENSG0000	4299	7135
AFF2	0	0	0	0	0	0	AF4/FMR2 family member 2 [Source:HGNC Symbol;Acc:HGNC:37	chrX:14758	NM_00116	.	.	.	NP_00116	.	protein_co	ENSG0000	2334	3776
AFF3	0.018658	0.042638	0.02899	0.016511	0	0	AF4/FMR2 family member 3 [Source:HGNC Symbol;Acc:HGNC:64	chr2:10016	NM_00102	XM_01151	.	.	NP_00102	XP_005264	protein_co	ENSG0000	3899	6473
AFF4	9.59975	9.65072	10.9231	8.55107	11.2686	12.4866	AF4/FMR2 family member 4 [Source:HGNC Symbol;Acc:HGNC:17	chr5:13221	NM_01442	XM_00671	.	.	NP_05523	XP_005272	protein_co	ENSG0000	27125	17869
AFG3L2	36.0704	39.0994	34.5183	40.9716	37.5222	31.3609	AFG3 like matrix AAA peptidase subunit 2 [Source:HGNC Symbol;	chr18:1230	NM_00679	XM_01152	.	.	NP_00678	XP_011523	protein_co	ENSG0000	10939	315
AFM	0	0	0	0	0	0	afamin [Source:HGNC Symbol;Acc:HGNC:316]	chr4:74347	NM_00113	.	.	.	NP_00112	.	protein_co	ENSG0000	173	316
AFMID	40.97	42.8214	43.8269	45.5414	46.1741	38.2349	arylformamidase [Source:HGNC Symbol;Acc:HGNC:20910]	chr17:7618	NM_00101	XM_01152	.	.	NP_00101	XP_011522	protein_co	ENSG0000	125061	20910
AFP	0	0	0	0	0	0	alpha fetoprotein [Source:HGNC Symbol;Acc:HGNC:317]	chr4:74301	NM_00113	XM_01153	.	.	NP_00112	.	protein_co	ENSG0000	174	317
AFTPH	12.7799	13.7044	11.0288	10.491	13.1644	14.8185	aftiphilin [Source:HGNC Symbol;Acc:HGNC:25951]	chr2:64751	NM_00100	XM_00526	.	.	NP_00100	XP_005264	protein_co	ENSG0000	54812	25951
AGA	3.10803	3.0995	2.6221	3.29856	3.06718	4.41722	aspartylglucosaminidase [Source:HGNC Symbol;Acc:HGNC:318]	chr4:17835	NM_00002	.	.	.	NP_00001	.	protein_co	ENSG0000	175	318
AGAP1	7.69269	8.65887	9.16092	7.87809	8.68037	9.1578	ArfGAP with GTPase domain, ankyrin repeat and PH domain 1 [Sc	chr2:23640	NM_00103	XM_01151	.	.	NP_00103	XP_005246	protein_co	ENSG0000	116987	16922
AGAP10	0.027708	0	0.024006	0	0.03162	0.01429	ArfGAP with GTPase domain, ankyrin repeat and PH domain 9 [Sc	chr10:4719	NM_00119	.	.	.	NP_00117	.	protein_co	ENSG0000	642517	23463
AGAP2	6.73189	6.97235	6.68088	7.59461	7.34773	6.02815	ArfGAP with GTPase domain, ankyrin repeat and PH domain 2 [Sc	chr12:5811	NM_00112	XM_00526	.	.	NP_00111	XP_005268	protein_co	ENSG0000	116986	16921
AGAP3	29.3037	29.1661	28.2829	31.6118	32.304	24.2873	ArfGAP with GTPase domain, ankyrin repeat and PH domain 3 [Sc	chr7:15078	NM_00104	XM_01701	.	.	NP_00103	XP_005249	protein_co	ENSG0000	116988	16923
AGAP4	0.563874	0.499927	0.400027	0.534037	0.731644	0.43781	ArfGAP with GTPase domain, ankyrin repeat and PH domain 4 [Sc	chr10:4632	NM_00127	XM_00671	.	.	NP_00126	XP_016871	protein_co	ENSG0000	119016	23459
AGAP5	1.07428	1.21944	1.18926	1.09369	1.45757	1.62287	ArfGAP with GTPase domain, ankyrin repeat and PH domain 5 [Sc	chr10:7543	NM_00114	.	.	.	NP_00113	XP_016854	protein_co	ENSG0000	729092	23467

AGAP6	5.4274	5.44655	5.19965	5.75364	5.32238	4.3215	ArfGAP with GTPase domain, ankyrin repeat and PH domain 6 [Sc	chr10:5174	NM_00107	XM_01701	.	.	NP_00107	XP_011538	protein_co	ENSG0000	414189	23466
AGAP8	0.226173	0.168809	0.152314	0.192765	0.187801	0.230329	ArfGAP with GTPase domain, ankyrin repeat and PH domain 8 [Sc	chr10:5122	NM_00127	XM_00527	.	.	NP_00126	XP_005277	protein_co	ENSG0000	119016	23464
AGAP9	0.100269	0.172152	0.058119	0.184332	0.303315	0.06968	ArfGAP with GTPase domain, ankyrin repeat and PH domain 9 [Sc	chr10:4818	NM_00119	.	.	.	NP_00117	.	protein_co	ENSG0000	642517	23463
AGBL1	0	0	0	0	0	0	Cytosolic carboxypeptidase 4 [Source:UniProtKB/Swiss-Prot;Acc:	chr15:8662	NM_15233	XM_01152	.	.	.	XP_011519	protein_co	ENSG0000	123624	.
AGBL2	0	0	0	0	0	0	ATP/GTP binding protein like 2 [Source:HGNC Symbol;Acc:HGNC:	chr11:4768	NM_02478	XM_01152	.	XR_428852	NP_07905	XP_005253	protein_co	ENSG0000	79841	26296
AGBL3	0.156977	0.120428	0.069404	0.121937	0.232233	0.036639	ATP/GTP binding protein like 3 [Source:HGNC Symbol;Acc:HGNC:	chr7:13467	NM_17856	XM_01701	.	.	NP_84865	XP_016867	protein_co	ENSG0000	340351	27981
AGBL4	0	0	0	0	0	0	ATP/GTP binding protein like 4 [Source:HGNC Symbol;Acc:HGNC:	chr1:48998	NM_03278	XM_01154	.	.	NP_00131	XP_011540	protein_co	ENSG0000	84871	25892
AGBL5	12.758	13.5264	18.8953	13.4134	12.5822	13.8608	ATP/GTP binding protein like 5 [Source:HGNC Symbol;Acc:HGNC:	chr2:27274	NM_00103	XM_01153	.	.	NP_00103	XP_005264	protein_co	ENSG0000	60509	26147
AGER	2.11434	2.9621	2.86931	2.973	2.20692	5.08091	advanced glycosylation end-product specific receptor [Source:HG	chr6:32148	NM_00113	.	.	.	NP_00112	.	protein_co	ENSG0000	177	320
AGFG1	28.1227	26.1248	24.8221	32.5476	35.9515	30.8843	ArfGAP with FG repeats 1 [Source:HGNC Symbol;Acc:HGNC:5175]	chr2:22833	NM_00113	XM_01151	.	.	NP_00112	XP_005246	protein_co	ENSG0000	3267	5175
AGFG2	2.72714	2.59456	2.81173	2.19442	2.20604	1.83606	ArfGAP with FG repeats 2 [Source:HGNC Symbol;Acc:HGNC:5177]	chr7:10013	NM_00607	XM_01701	.	.	NP_00606	XP_005250	protein_co	ENSG0000	3268	5177
AGGF1	9.20071	9.40745	9.9677	8.69365	9.75384	12.818	angiogenic factor with G-patch and FHA domains 1 [Source:HGNC	chr5:76326	NM_01804	.	.	.	NP_06051	.	protein_co	ENSG0000	55109	24684
AGK	15.9241	19.7118	16.3082	21.0019	19.4211	18.1684	acylglycerol kinase [Source:HGNC Symbol;Acc:HGNC:21869]	chr7:14125	NM_01823	XM_01151	.	.	NP_06070	XP_011514	protein_co	ENSG0000	55750	21869
AGL	7.21071	7.25598	9.17005	8.15149	9.63537	9.77625	amylo-alpha-1, 6-glucosidase, 4-alpha-glucanotransferase [Source	chr1:10031	NM_00002	XM_00527	.	.	NP_00001	XP_005270	protein_co	ENSG0000	178	321
AGMAT	0	0.071894	0	0	0	0	agmatinase [Source:HGNC Symbol;Acc:HGNC:18407]	chr1:15853	NM_02475	.	.	.	NP_07903	.	protein_co	ENSG0000	79814	18407
AGMO	0	0	0	0	0	0.036746	alkylglycerol monooxygenase [Source:HGNC Symbol;Acc:HGNC:3	chr7:15239	NM_00100	XM_01151	.	.	NP_00100	XP_006715	protein_co	ENSG0000	392636	33784
AGO1	9.46207	9.89823	10.6601	9.3612	10.0673	9.74687	argonaute 1, RISC catalytic component [Source:HGNC Symbol;Acc	chr1:36335	NM_01219	XM_01154	.	.	NP_00130	XP_011539	protein_co	ENSG0000	26523	3262
AGO2	20.9239	22.6101	23.5047	18.95	22.9421	27.5067	argonaute 2, RISC catalytic component [Source:HGNC Symbol;Acc	chr8:14153	NM_00116	XM_01701	.	.	NP_00115	XP_011515	protein_co	ENSG0000	27161	3263
AGO3	5.72924	5.38549	4.46389	4.86632	4.96734	5.81866	argonaute 3, RISC catalytic component [Source:HGNC Symbol;Acc	chr1:36396	NM_02485	XM_01700	.	.	NP_07912	XP_005270	protein_co	ENSG0000	192669	18421
AGO4	2.85792	2.96969	3.67737	2.49595	2.70376	4.47548	argonaute 4, RISC catalytic component [Source:HGNC Symbol;Acc	chr1:36273	NM_01762	XM_00527	.	.	NP_06009	XP_005270	protein_co	ENSG0000	192670	18424
AGPAT1	40.4528	41.2818	38.9958	48.4982	41.3771	37.4537	1-acylglycerol-3-phosphate O-acyltransferase 1 [Source:HGNC Sy	chr6:32132	NM_00641	XM_01151	NR_10677	.	NP_00640	XP_005248	protein_co	ENSG0000	102466190	324
AGPAT2	50.4811	52.8915	43.1363	55.1611	43.0833	38.5862	1-acylglycerol-3-phosphate O-acyltransferase 2 [Source:HGNC Sy	chr9:13956	NM_00101	.	.	.	NP_00101	.	protein_co	ENSG0000	10555	325
AGPAT3	23.1599	24.3373	20.8712	20.1901	21.7206	15.1932	1-acylglycerol-3-phosphate O-acyltransferase 3 [Source:HGNC Sy	chr21:4528	NM_00103	XM_01152	.	.	NP_00103	XP_005261	protein_co	ENSG0000	56894	326
AGPAT4	3.58436	5.5399	4.16467	3.57706	1.92726	4.9962	1-acylglycerol-3-phosphate O-acyltransferase 4 [Source:HGNC Sy	chr6:16155	NM_02013	XM_01701	.	.	NP_06451	XP_005267	protein_co	ENSG0000	56895	20885
AGPAT5	38.8055	38.1205	43.7508	41.4984	48.2397	63.0003	1-acylglycerol-3-phosphate O-acyltransferase 5 [Source:HGNC Sy	chr8:65658	NM_01836	.	.	.	NP_06083	.	protein_co	ENSG0000	55326	20886
AGPS	17.8282	18.632	14.317	16.2786	18.4159	16.2441	alkylglycerone phosphate synthase [Source:HGNC Symbol;Acc:HG	chr2:17809	NM_00365	XM_01151	.	.	NP_00365	XP_011510	protein_co	ENSG0000	8540	327
AGR2	0	0.040526	0	0	0	0	anterior gradient 2, protein disulphide isomerase family member	chr7:16831	NM_00640	XM_00524	.	.	NP_00639	XP_005249	protein_co	ENSG0000	10551	328
AGR3	0	0	0	0	0	0	anterior gradient 3, protein disulphide isomerase family member	chr7:16899	NM_17681	XM_01151	.	.	NP_78978	XP_005249	protein_co	ENSG0000	155465	24167
AGRN	22.0391	23.1597	24.7856	28.6188	26.5618	33.5187	agrin [Source:HGNC Symbol;Acc:HGNC:329]	chr1:95550	NM_00130	XM_01154	.	.	NP_00129	XP_005244	protein_co	ENSG0000	375790	329
AGRP	0	0	0	0	0	0	agouti related neuropeptide [Source:HGNC Symbol;Acc:HGNC:33	chr16:6751	NM_00113	XM_01152	.	.	NP_00112	XP_011521	protein_co	ENSG0000	181	330
AGT	0	0.069993	0.059686	0	0.039851	0	angiotensinogen [Source:HGNC Symbol;Acc:HGNC:333]	chr1:23083	NM_00002	.	.	.	NP_00002	.	protein_co	ENSG0000	183	333
AGTPBP1	6.67484	5.91713	6.58762	4.54718	6.28408	9.40676	ATP/GTP binding protein 1 [Source:HGNC Symbol;Acc:HGNC:1725	chr9:88161	NM_00128	XM_01701	.	.	NP_00127	XP_005251	protein_co	ENSG0000	23287	17258
AGTR1	0	0	0	0	0	0	angiotensin II receptor type 1 [Source:HGNC Symbol;Acc:HGNC:3	chr3:14841	NM_00068	.	.	.	NP_00067	.	protein_co	ENSG0000	185	336
AGTR2	0	0	0	0	0	0	angiotensin II receptor type 2 [Source:HGNC Symbol;Acc:HGNC:3	chrX:11530	NM_00068	XM_01153	.	.	NP_00067	XP_011535	protein_co	ENSG0000	186	338
AGTRAP	47.953	49.1809	42.6057	49.9286	37.0313	31.1805	angiotensin II receptor associated protein [Source:HGNC Symbol;	chr1:11796	NM_00104	XM_01154	.	.	NP_00103	XP_011540	protein_co	ENSG0000	57085	13539
AGXT	0	0	0	0	0	0	alanine-glyoxylate aminotransferase [Source:HGNC Symbol;Acc:H	chr2:24180	NM_00003	.	.	.	NP_00002	.	protein_co	ENSG0000	189	341
AGXT2	0	0	0	0	0	0	alanine--glyoxylate aminotransferase 2 [Source:HGNC Symbol;Acc	chr5:34998	NM_03190	XM_01151	.	.	NP_00129	XP_005248	protein_co	ENSG0000	64902	14412
AHCTF1	15.0842	14.8127	15.853	13.8967	17.7958	16.2083	AT-hook containing transcription factor 1 [Source:HGNC Symbol;A	chr1:24700	NM_00132	XM_01154	.	.	NP_00131	XP_006711	protein_co	ENSG0000	25909	24618
AHCY	186.587	180.908	219.831	223.924	189.609	181.233	adenosylhomocysteinase [Source:HGNC Symbol;Acc:HGNC:343]	chr20:3286	NM_00068	XM_01152	.	.	NP_00067	XP_005260	protein_co	ENSG0000	191	343
AHCYL1	40.8816	38.8714	45.0777	40.3252	44.9719	54.7302	adenosylhomocysteinase like 1 [Source:HGNC Symbol;Acc:HGNC:	chr1:11052	NM_00124	XM_01154	.	.	NP_00122	XP_011538	protein_co	ENSG0000	10768	344
AHCYL2	6.14418	6.02782	8.09717	6.02897	5.28896	6.78603	adenosylhomocysteinase like 2 [Source:HGNC Symbol;Acc:HGNC:	chr7:12886	NM_00113	XM_01701	.	.	NP_00112	XP_005250	protein_co	ENSG0000	23382	22204
AHDC1	7.31179	7.55688	8.61608	6.55114	7.70074	5.52311	AT-hook DNA binding motif containing 1 [Source:HGNC Symbol;A	chr1:27860	NM_00102	XM_01154	.	.	NP_00102	XP_005245	protein_co	ENSG0000	27245	25230
AHI1	4.62679	4.79868	5.79708	2.78053	3.83918	4.32743	Abelson helper integration site 1 [Source:HGNC Symbol;Acc:HGN	chr6:13560	NM_00113	XM_01701	.	.	NP_00112	XP_011534	protein_co	ENSG0000	54806	21575
AHNAK	43.0537	46.9498	48.1284	30.1259	33.1019	39.8257	AHNAK nucleoprotein [Source:HGNC Symbol;Acc:HGNC:347]	chr11:6220	NM_00162	XM_01154	.	.	NP_00161	XP_005274	protein_co	ENSG0000	79026	347
AHNAK2	15.9947	16.6181	26.4827	9.45033	9.64294	13.2079	AHNAK nucleoprotein 2 [Source:HGNC Symbol;Acc:HGNC:20125]	chr14:1053	NM_13842	XM_00526	.	.	NP_61242	XP_005267	protein_co	ENSG0000	113146	20125
AHR	9.29605	9.68633	9.15454	7.86947	8.76334	10.6405	aryl hydrocarbon receptor [Source:HGNC Symbol;Acc:HGNC:348]	chr7:17338	NM_00162	.	.	.	NP_00161	.	protein_co	ENSG0000	196	348
AHRR	9.07865	8.07093	5.82023	8.17898	8.0724	5.75021	aryl-hydrocarbon receptor repressor [Source:HGNC Symbol;Acc:H	chr5:27173	NM_00124	.	.	.	NP_00122	.	protein_co	ENSG0000	57491	346
AHSA1	174.038	170.344	120.639	220.889	187.569	103.059	activator of Hsp90 ATPase activity 1 [Source:HGNC Symbol;Acc:H	chr14:7792	NM_01211	XM_01153	.	.	NP_00130	XP_011534	protein_co	ENSG0000	10598	1189
AHSA2	10.6679	9.89112	11.8261	7.20408	6.31794	6.74198	AHA1, activator of heat shock 90kDa protein ATPase homolog 2 (	chr2:61404	NM_00132	XM_01153	.	.	NP_00130	XP_011530	protein_co	ENSG0000	130872	20437
AHSG	0.096931	0.197006	0.197186	0.211648	0.522682	0.291645	alpha 2-HS glycoprotein [Source:HGNC Symbol;Acc:HGNC:349]	chr3:18633	NM_00162	.	.	.	NP_00161	.	protein_co	ENSG0000	197	349
AHSP	0	0	0	0	0	0	alpha hemoglobin stabilizing protein [Source:HGNC Symbol;Acc:H	chr16:3153	NM_00131	XM_00525	.	.	NP_00130	XP_005255	protein_co	ENSG0000	51327	18075
AICDA	0	0	0	0	0	0	activation induced cytidine deaminase [Source:HGNC Symbol;Acc	chr12:8754	NM_02066	XM_01152	.	.	NP_06571	XP_011519	protein_co	ENSG0000	57379	13203
AIDA	12.5042	11.936	11.8592	10.0161	13.0354	16.8864	axin interactor, dorsalization associated [Source:HGNC Symbol;A	chr1:22284	NM_02283	.	.	.	NP_07374	.	protein_co	ENSG0000	64853	25761
AIF1	0.160744	0	0	0	0	0.16562	allograft inflammatory factor 1 [Source:HGNC Symbol;Acc:HGNC:	chr6:31582	NM_00131	XM_00524	.	.	NP_00130	.	protein_co	ENSG0000	199	352
AIF1L	19.6181	18.1575	24.3817	25.9017	24.0912	23.0901	allograft inflammatory factor 1 like [Source:HGNC Symbol;Acc:HG	chr9:13397	NM_00118	XM_01701	.	.	NP_00117	XP_016870	protein_co	ENSG0000	83543	28904
AIFM1	32.1715	28.9158	31.5683	30.564	28.4239	27.059	apoptosis inducing factor, mitochondria associated 1 [Source:HG	chrX:12926	NM_00113	.	.	.	NP_00112	.	protein_co	ENSG0000	9131	8768
AIFM2	18.8159	18.9221	13.0385	21.241	18.8428	14.4947	apoptosis inducing factor, mitochondria associated 2 [Source:HG	chr10:7181	NM_00119	.	.	.	NP_00118	.	protein_co	ENSG0000	84883	21411
AIFM3	1.23185	1.36686	1.38112	1.21551	0.904141	0.78688	apoptosis inducing factor, mitochondria associated 3 [Source:HG	chr22:2131	NM_00101	.	.	.	NP_00101	.	protein_co	ENSG0000	150209	26398
AIG1	28.9754	33.5681	34.2645	36.1407	26.0114	40.2156	androgen induced 1 [Source:HGNC Symbol;Acc:HGNC:21607]	chr6:14338	NM_00128	XM_01153	.	.	NP_00127	XP_005267	protein_co	ENSG0000	51390	21607

AIM1	3.98978	4.21933	5.2275	4.33959	5.25144	4.98719	absent in melanoma 1 [Source:HGNC Symbol;Acc:HGNC:356]	chr6:10680	NM_00162	XM_00526	.	.	NP_00161	XP_005266	protein_co	ENSG0000	202	356
AIM1L	1.24079	1.52262	1.65764	2.51588	1.8525	2.17574	absent in melanoma 1-like [Source:HGNC Symbol;Acc:HGNC:1729	chr1:26648	NM_00103	XM_01154	.	.	NP_00103	XP_005245	protein_co	ENSG0000	55057	17295
AIM2	0	0	0	0	0	0	absent in melanoma 2 [Source:HGNC Symbol;Acc:HGNC:357]	chr1:15903	NM_00483	XM_00524	.	.	NP_00482	XP_005245	protein_co	ENSG0000	9447	357
AIMP1	34.0164	31.7493	26.6652	35.2317	32.0717	27.5907	aminoacyl tRNA synthetase complex interacting multifunctional p	chr4:10696	NM_00114	.	.	.	NP_00113	XP_016864	protein_co	ENSG0000	9255	10648
AIMP2	56.4564	58.3771	49.3798	62.2585	47.8112	45.0583	aminoacyl tRNA synthetase complex interacting multifunctional p	chr7:60488	NM_00630	XM_00524	.	.	NP_00131	XP_005249	protein_co	ENSG0000	7965	20609
AIP	26.3356	27.4115	23.9334	28.9123	22.4295	18.5406	aryl hydrocarbon receptor interacting protein [Source:HGNC Sym	chr11:6725	NM_00130	.	.	.	NP_00128	.	protein_co	ENSG0000	9049	358
AIPL1	0	0	0.023415	0	0	0	aryl hydrocarbon receptor interacting protein like 1 [Source:HGN	chr17:6327	NM_00103	.	.	.	NP_00102	.	protein_co	ENSG0000	23746	359
AIRE	0	0	0	0	0	0	autoimmune regulator [Source:HGNC Symbol;Acc:HGNC:360]	chr21:4570	NM_00038	XM_01152	.	.	NP_00037	.	protein_co	ENSG0000	326	360
AJAP1	0	0	0	0	0	0	adherens junctions associated protein 1 [Source:HGNC Symbol;Ac	chr1:47147	NM_00104	XM_01154	.	.	NP_00103	XP_011540	protein_co	ENSG0000	55966	30801
AJUBA	15.9987	17.5016	13.4303	15.6301	15.2005	23.0033	ajuba LIM protein [Source:HGNC Symbol;Acc:HGNC:20250]	chr14:2341	NM_03287	.	.	.	NP_11626	.	protein_co	ENSG0000	84962	20250
AK1	36.2184	35.929	34.9311	41.8673	30.0723	26.3019	adenylate kinase 1 [Source:HGNC Symbol;Acc:HGNC:361]	chr9:13062	NM_00047	XM_01151	.	.	NP_00046	XP_005251	protein_co	ENSG0000	203	361
AK2	117.506	128.066	111.069	119.863	115.411	101.106	adenylate kinase 2 [Source:HGNC Symbol;Acc:HGNC:362]	chr1:33473	NM_00119	XM_01154	.	.	NP_00118	XP_011539	protein_co	ENSG0000	204	362
AK3	24.9865	27.5414	37.2343	31.9249	31.5505	38.5882	adenylate kinase 3 [Source:HGNC Symbol;Acc:HGNC:17376]	chr9:47095	NM_00119	.	.	.	NP_00118	.	protein_co	ENSG0000	50808	17376
AK4	17.9156	17.8465	23.4772	19.2184	22.2008	15.2911	adenylate kinase 4 [Source:HGNC Symbol;Acc:HGNC:363]	chr1:65613	NM_00100	XM_01700	.	.	NP_00100	XP_016856	protein_co	ENSG0000	205	363
AK5	0.021181	0	0.15471	0.386658	0.023823	0.350983	adenylate kinase 5 [Source:HGNC Symbol;Acc:HGNC:365]	chr1:77747	NM_01209	XM_01700	.	.	NP_03622	XP_005270	protein_co	ENSG0000	26289	365
AK6	18.1568	13.1983	17.877	20.2782	34.2594	30.8106	adenylate kinase 6 [Source:HGNC Symbol;Acc:HGNC:49151]	chr5:68646	NM_00101	.	.	.	NP_00101	.	protein_co	ENSG0000	102157402	49151
AK7	1.18188	0.921632	1.45247	1.47932	1.92609	2.10404	adenylate kinase 7 [Source:HGNC Symbol;Acc:HGNC:20091]	chr14:9685	NM_15232	XM_01153	.	.	NP_68954	XP_006720	protein_co	ENSG0000	122481	20091
AK8	0.82938	0.484072	0.896036	0.48738	0.518167	0.783318	adenylate kinase 8 [Source:HGNC Symbol;Acc:HGNC:26526]	chr9:13560	NM_15257	XM_01151	.	.	NP_68978	XP_005272	protein_co	ENSG0000	158067	26526
AK9	1.01309	1.01878	0.968317	0.83235	0.729981	0.963996	adenylate kinase 9 [Source:HGNC Symbol;Acc:HGNC:33814]	chr6:10981	NM_00114	XM_01701	.	.	NP_00113	XP_006715	protein_co	ENSG0000	221264	33814
AKAIN1	0	0	0	0	0	0	A kinase (PRKA) anchor inhibitor 1 [Source:HGNC Symbol;Acc:HG	chr18:5145	NM_00114	XM_01152	.	.	NP_00113	XP_011524	protein_co	ENSG0000	642597	28285
AKAP1	33.3566	33.0286	27.9208	32.9885	31.294	24.4017	A-kinase anchoring protein 1 [Source:HGNC Symbol;Acc:HGNC:36	chr17:5516	NM_00124	XM_01152	.	.	NP_00122	XP_005257	protein_co	ENSG0000	8165	367
AKAP10	10.5192	9.96415	10.0057	8.05767	9.82014	11.4576	A-kinase anchoring protein 10 [Source:HGNC Symbol;Acc:HGNC:3	chr17:1980	NM_00720	XM_00672	.	.	NP_00913	XP_006721	protein_co	ENSG0000	11216	368
AKAP11	7.56475	7.68957	9.7979	5.96678	8.40682	8.56377	A-kinase anchoring protein 11 [Source:HGNC Symbol;Acc:HGNC:3	chr13:4284	NM_01624	XM_01702	.	.	NP_05733	XP_005266	protein_co	ENSG0000	11215	369
AKAP12	349.562	366.201	552.934	310.285	339.775	308.334	A-kinase anchoring protein 12 [Source:HGNC Symbol;Acc:HGNC:3	chr6:15156	NM_00510	XM_01701	.	.	NP_00509	XP_005267	protein_co	ENSG0000	9590	370
AKAP13	5.512	6.30412	6.61715	4.7045	5.53686	7.06613	A-kinase anchoring protein 13 [Source:HGNC Symbol;Acc:HGNC:3	chr15:8592	NM_00127	.	NR_10699	.	NP_00125	.	protein_co	ENSG0000	102465803	371
AKAP14	0	0.090107	0.108565	0	0	0	A-kinase anchoring protein 14 [Source:HGNC Symbol;Acc:HGNC:2	chrX:11902	NM_00100	.	.	.	NP_00100	.	protein_co	ENSG0000	158798	24061
AKAP17A	2.02986	3.17041	2.45582	2.65141	2.26043	2.44809	A-kinase anchoring protein 17A [Source:HGNC Symbol;Acc:HGNC	chrX:17104	NM_00508	.	.	.	NP_00507	.	protein_co	ENSG0000	8227	18783
AKAP2	12.7778	14.471	15.6096	17.0283	19.0645	18.8298	A-kinase anchoring protein 2 [Source:HGNC Symbol;Acc:HGNC:37	chr9:11240	NM_00100	.	.	.	NP_00100	.	protein_co	ENSG0000	11217	372
AKAP3	1.26082	1.0134	1.18067	1.08287	1.22449	1.72289	A-kinase anchoring protein 3 [Source:HGNC Symbol;Acc:HGNC:37	chr12:4724	NM_00127	XM_01152	.	.	NP_00126	XP_005253	protein_co	ENSG0000	10566	373
AKAP4	0	0	0	0	0	0	A-kinase anchoring protein 4 [Source:HGNC Symbol;Acc:HGNC:37	chrX:49955	NM_00388	.	.	.	NP_00387	.	protein_co	ENSG0000	8852	374
AKAP5	0.457832	0.193225	0.077612	0.088126	0.196105	0.133026	A-kinase anchoring protein 5 [Source:HGNC Symbol;Acc:HGNC:37	chr14:6485	NM_00485	.	.	.	NP_00484	.	protein_co	ENSG0000	9495	375
AKAP6	0.318384	0.338605	0.617992	0.33605	0.245006	0.407199	A-kinase anchoring protein 6 [Source:HGNC Symbol;Acc:HGNC:37	chr14:3279	NM_00427	XM_00526	.	.	NP_00426	XP_016877	protein_co	ENSG0000	9472	376
AKAP7	1.58398	1.94913	1.4581	0.80924	2.46811	1.81966	A-kinase anchoring protein 7 [Source:HGNC Symbol;Acc:HGNC:37	chr6:13145	NM_00484	XM_01701	.	.	NP_00483	XP_005267	protein_co	ENSG0000	9465	377
AKAP8	13.3576	12.7044	11.288	12.6503	11.7875	8.91254	A-kinase anchoring protein 8 [Source:HGNC Symbol;Acc:HGNC:37	chr19:1546	NM_00585	XM_01702	.	.	NP_00584	XP_016881	protein_co	ENSG0000	10270	378
AKAP8L	27.8264	29.2627	30.7222	37.8456	31.8655	35.0898	A-kinase anchoring protein 8 like [Source:HGNC Symbol;Acc:HGN	chr19:1549	NM_00129	XM_00525	.	.	NP_00127	XP_005259	protein_co	ENSG0000	26993	29857
AKAP9	4.40469	4.26427	4.18663	3.21593	4.65496	4.91063	A-kinase anchoring protein 9 [Source:HGNC Symbol;Acc:HGNC:37	chr7:91570	NM_00575	XM_01151	.	.	NP_00574	XP_016867	protein_co	ENSG0000	10142	379
AKIP1	22.5588	24.3785	23.1604	24.6713	24.0421	22.2104	A-kinase interacting protein 1 [Source:HGNC Symbol;Acc:HGNC:1	chr11:8932	NM_00120	XM_01701	.	.	NP_00119	XP_006718	protein_co	ENSG0000	56672	1170
AKIRIN1	46.5249	44.6729	52.7466	47.1131	55.275	65.1268	akirin 1 [Source:HGNC Symbol;Acc:HGNC:25744]	chr1:39456	NM_00113	.	.	.	NP_00112	.	protein_co	ENSG0000	79647	25744
AKIRIN2	25.3521	25.4618	21.1027	25.0813	27.4626	31.6623	akirin 2 [Source:HGNC Symbol;Acc:HGNC:21407]	chr6:88384	NM_01806	.	.	.	NP_06053	.	protein_co	ENSG0000	55122	21407
AKNA	4.75045	4.88844	4.23421	4.70729	4.14765	9.5102	AT-hook transcription factor [Source:HGNC Symbol;Acc:HGNC:24	chr9:11709	NM_00131	XM_01701	.	.	NP_00130	XP_005252	protein_co	ENSG0000	80709	24108
AKNAD1	0.097319	0.101322	0.039989	0.194135	0.02195	0.019782	AKNA domain containing 1 [Source:HGNC Symbol;Acc:HGNC:2839	chr1:10935	NM_15276	.	.	.	NP_68997	.	protein_co	ENSG0000	254268	28398
AKR1A1	109.121	98.2229	88.2525	128.927	101.466	95.4151	aldo-keto reductase family 1 member A1 [Source:HGNC Symbol;A	chr1:46016	NM_00120	XM_01154	.	.	NP_00118	XP_011538	protein_co	ENSG0000	10327	380
AKR1B1	39.1075	40.8229	41.5634	42.0338	31.7335	32.4743	aldo-keto reductase family 1 member B [Source:HGNC Symbol;Ac	chr7:13412	NM_00162	XM_00525	.	.	NP_00161	XP_005250	protein_co	ENSG0000	231	381
AKR1B10	0.606121	0.865092	0.497248	0.798564	0.853305	0.429125	aldo-keto reductase family 1 member B10 [Source:HGNC Symbol;	chr7:13421	NM_02029	XM_01151	.	.	NP_06469	XP_011514	protein_co	ENSG0000	57016	382
AKR1B15	0	0	0	0	0	0	aldo-keto reductase family 1 member B15 [Source:HGNC Symbol;	chr7:13423	NM_00108	XM_01151	.	.	NP_00107	XP_011514	protein_co	ENSG0000	441282	37281
AKR1C1	0.488479	0.086653	0.173115	0.43192	0.219317	0.390891	aldo-keto reductase family 1 member C1 [Source:HGNC Symbol;A	chr10:5005	NM_00135	.	.	.	NP_00134	.	protein_co	ENSG0000	1645	384
AKR1C2	0	0	0	0.173355	0.082918	0.317201	aldo-keto reductase family 1 member C2 [Source:HGNC Symbol;A	chr10:5029	NM_00113	XM_01151	.	.	NP_00112	XP_011517	protein_co	ENSG0000	1646	385
AKR1C3	0.580791	0.648843	1.26702	2.77072	1.35136	3.42693	aldo-keto reductase family 1 member C3 [Source:HGNC Symbol;A	chr10:5091	NM_00125	.	.	.	NP_00124	.	protein_co	ENSG0000	8644	386
AKR1C4	0	0	0	0	0	0	aldo-keto reductase family 1 member C4 [Source:HGNC Symbol;A	chr10:5237	NM_00181	XM_01151	.	.	NP_00180	.	protein_co	ENSG0000	1109	387
AKR1D1	0	0	0	0	0.035864	0.031585	aldo-keto reductase family 1 member D1 [Source:HGNC Symbol;A	chr7:13776	NM_00119	.	.	.	NP_00117	.	protein_co	ENSG0000	6718	388
AKR1E2	1.2666	1.13925	0.791344	0.645196	0.421927	0.673152	aldo-keto reductase family 1 member E2 [Source:HGNC Symbol;A	chr10:4868	NM_00104	XM_01151	.	.	NP_00103	XP_011518	protein_co	ENSG0000	83592	23437
AKR7A2	61.0909	52.4549	56.4456	61.4334	52.8633	52.5902	aldo-keto reductase family 7 member A2 [Source:HGNC Symbol;A	chr1:19630	NM_00368	.	.	.	NP_00130	.	protein_co	ENSG0000	8574	389
AKR7A3	0.5832	1.13946	1.22653	0.623101	0.556306	1.05608	aldo-keto reductase family 7 member A3 [Source:HGNC Symbol;A	chr1:19609	NM_01206	.	.	.	NP_03619	.	protein_co	ENSG0000	22977	390
AKT1	67.8318	67.9882	69.5049	80.1073	71.2305	69.8343	AKT serine/threonine kinase 1 [Source:HGNC Symbol;Acc:HGNC:3	chr14:1052	NM_00101	XM_01153	.	.	NP_00101	XP_005267	protein_co	ENSG0000	207	391
AKT1S1	53.6258	46.6917	38.9578	45.9066	37.3922	33.0828	AKT1 substrate 1 [Source:HGNC Symbol;Acc:HGNC:28426]	chr19:5037	NM_00109	.	.	.	NP_00109	.	protein_co	ENSG0000	84335	28426
AKT2	23.7766	25.6814	26.1272	23.4787	22.5089	20.2269	AKT serine/threonine kinase 2 [Source:HGNC Symbol;Acc:HGNC:3	chr19:4073	NM_00124	XM_01152	.	.	NP_00122	XP_011524	protein_co	ENSG0000	208	392
AKT3	0.21008	0.257497	0.248288	0.164803	0.149507	0.270911	AKT serine/threonine kinase 3 [Source:HGNC Symbol;Acc:HGNC:3	chr1:24341	NM_00120	XM_01154	.	.	NP_00119	XP_005273	protein_co	ENSG0000	10000	393
AKTIP	10.1791	9.14946	8.00732	7.34888	8.79905	12.093	AKT interacting protein [Source:HGNC Symbol;Acc:HGNC:16710]	chr16:5346	NM_00101	XM_00525	.	.	NP_00101	XP_005256	protein_co	ENSG0000	64400	16710
AL021546.6	0.832537	3.13E-06	0.548605	2.27E-06	1.7E-06	2.19E-06	.	chr12:1208	.	.	.	.	.	.	protein_co	ENSG0000	.	.

AL021920.2	0	0	0	0	0	0	family with sequence similarity 231 member B [Source:HGNC Syn	chr1:17060	.	XM_00172	.	.	NP_00129	XP_001721	protein_co	ENSG0000	100133301	49506
AL022328.1	0	0	0	2.91723	0	0	Uncharacterized protein [Source:UniProtKB/TrEMBL;Acc:M0QZK	chr22:5071	.	.	.	.	.	.	protein_co	ENSG00000	.	.
AL031320.1	0	0	0	0	0	0	.	chr6:14374	.	.	.	.	.	.	protein_co	ENSG00000	.	.
AL031590.1	0	0	0	0	0	0	.	chr22:3965	.	.	.	.	.	.	protein_co	ENSG00000	.	.
AL031663.1	0	0	0	0	0	0	Uncharacterized protein [Source:UniProtKB/TrEMBL;Acc:M0R2E	chr20:4409	.	.	.	.	.	.	protein_co	ENSG00000	.	.
AL035252.1	0	0	0	0	0	0	HCG2018895; Uncharacterized protein [Source:UniProtKB/TrEMB	chr20:2517	.	.	.	.	.	.	protein_co	ENSG00000	.	.
AL035406.1	0	0	0	0	0	0	Uncharacterized protein [Source:UniProtKB/TrEMBL;Acc:M0QY6	chr1:59228	.	.	.	.	.	.	protein_co	ENSG00000	.	.
AL035588.1	0	0	0	0	0	0	.	chr6:41651	.	.	.	.	.	.	protein_co	ENSG00000	.	.
AL035681.1	0	0	0	0	0	0	.	chr22:4168	.	.	.	.	.	.	protein_co	ENSG00000	.	.
AL049829.1	3.37542	0	2.21672	1.87336	3.71853	1.74543	Uncharacterized protein [Source:UniProtKB/TrEMBL;Acc:M0R3A	chr14:2377	.	.	.	.	.	.	protein_co	ENSG00000	.	.
AL049840.1	0.008638	6.6E-219	8.6E-184	0	0.020748	0.110914	Uncharacterized protein; cDNA FLJ53535 [Source:UniProtKB/TrE	chr14:1040	.	.	.	.	.	.	protein_co	ENSG00000	.	.
AL050302.1	0	0	0	0	0	0	Uncharacterized protein [Source:UniProtKB/TrEMBL;Acc:F5GZX1	chr21:1474	.	.	.	.	.	.	protein_co	ENSG00000	.	.
AL050303.1	0	0	0	0	0	0	Uncharacterized protein; cDNA FLJ43689 fis, clone TBAES200355	chr21:1505	.	.	.	.	.	.	protein_co	ENSG00000	.	.
AL079342.1	0	0.038074	0.063289	0.040406	0.040839	0.037365	Uncharacterized protein; cDNA FLJ27030 fis, clone SLV07741 [So	chr6:89674	.	.	.	.	.	.	protein_co	ENSG00000	.	.
AL096677.1	0	0	0	0	0	0	.	chr20:2333	.	.	.	.	.	.	protein_co	ENSG00000	.	.
AL109659.1	0	0	0	0	0	0	HCG1780467; PRO0529; Uncharacterized protein [Source:UniPro	chr1:48569	.	.	.	.	.	.	protein_co	ENSG00000	.	.
AL109927.1	0	0	2.09731	0	0	0	HCG2032222; PRO2047; Uncharacterized protein [Source:UniPro	chr1:28157	.	.	.	.	.	.	protein_co	ENSG00000	.	.
AL117190.2	0	0	0	0	0	0	.	chr14:1012	.	.	.	.	.	.	protein_co	ENSG00000	.	.
AL117190.3	0	0	0	0	0	0	Esophagus cancer-related gene-2 interaction susceptibility protei	chr14:1013	.	.	.	.	.	.	protein_co	ENSG00000	.	.
AL118506.1	0	0	0	0	0	0	Uncharacterized protein [Source:UniProtKB/TrEMBL;Acc:M0R1J	chr20:6257	.	.	.	.	.	.	protein_co	ENSG00000	.	.
AL121895.1	0	0	0	0	0	0	Uncharacterized protein [Source:UniProtKB/TrEMBL;Acc:M0R34	chr20:3467	.	.	.	.	.	.	protein_co	ENSG00000	.	.
AL121901.1	0	0	0	0	0	0	Nasopharyngeal carcinoma-related protein YH1; Uncharacterized	chr20:3182	.	.	.	.	.	.	protein_co	ENSG00000	.	.
AL121963.1	0	0	0	0	0.360681	0	Uncharacterized protein [Source:UniProtKB/TrEMBL;Acc:H7C467	chr6:11642	.	.	.	.	.	.	protein_co	ENSG00000	.	.
AL132989.1	0	0	0	0	0	0	.	chr14:5875	.	.	.	.	.	.	protein_co	ENSG00000	.	.
AL133262.1	0	0	0	0	0	0	.	chr6:44355	.	.	.	.	.	.	protein_co	ENSG00000	.	.
AL133318.1	0	0	0	0	0	0	Uncharacterized protein [Source:UniProtKB/TrEMBL;Acc:M0QYP	chr13:4111	.	.	.	.	.	.	protein_co	ENSG00000	.	.
AL133373.1	0.043361	0.044686	0	0.048056	0	0	Uncharacterized protein [Source:UniProtKB/TrEMBL;Acc:M0R20	chr14:9203	.	.	.	.	.	.	protein_co	ENSG00000	.	.
AL135998.1	0	0	0	0	0	0	Uncharacterized protein [Source:UniProtKB/TrEMBL;Acc:H7BYV7	chr14:2324	.	.	.	.	.	.	protein_co	ENSG00000	.	.
AL136115.1	0	0	0	0	0	0	HCG2032337; PRO1848; Uncharacterized protein [Source:UniPro	chr1:32372	.	.	.	.	.	.	protein_co	ENSG00000	.	.
AL136218.1	0.328012	1.75644	8.23E-71	0.340909	2.92013	3.80243	Sarcoma antigen NY-SAR-79; Uncharacterized protein [Source:U	chr13:4988	.	.	.	.	.	.	protein_co	ENSG00000	.	.
AL136376.1	0	0.495233	0	0.532385	0.566725	0	Uncharacterized protein [Source:UniProtKB/TrEMBL;Acc:M0R1U	chr1:11691	.	.	.	.	.	.	protein_co	ENSG00000	.	.
AL136531.1	0	0	0	0	0	0.063591	HCG2043693; Uncharacterized protein [Source:UniProtKB/TrEMB	chr20:1316	.	.	.	.	.	.	protein_co	ENSG00000	.	.
AL137002.1	0	0	0	0	0	0	Uncharacterized protein [Source:UniProtKB/TrEMBL;Acc:M0R0V	chr13:1137	.	.	.	.	.	.	protein_co	ENSG00000	.	.
AL137026.1	0	0	0	0	0	0	Uncharacterized protein [Source:UniProtKB/TrEMBL;Acc:M0QY0	chr10:4486	.	.	.	.	.	.	protein_co	ENSG00000	.	.
AL138815.1	0	0	0	0	0	0	Uncharacterized protein [Source:UniProtKB/TrEMBL;Acc:H7BYW	chr13:2594	.	.	.	.	.	.	protein_co	ENSG00000	.	.
AL138815.2	0	0	0	0	0	0	Uncharacterized protein [Source:UniProtKB/TrEMBL;Acc:O00246	chr13:2594	.	.	.	.	.	.	protein_co	ENSG00000	.	.
AL138847.1	0	0	0	0	0	0	CDNA FLJ26506 fis, clone KDN07105; Uncharacterized protein [S	chr1:62920	.	.	.	.	.	.	protein_co	ENSG00000	.	.
AL139080.1	0	0	0	0	0	0	Uncharacterized protein [Source:UniProtKB/TrEMBL;Acc:M0R1P	chr13:2430	.	.	.	.	.	.	protein_co	ENSG00000	.	.
AL139333.1	0	0	0	0	0	0	.	chr6:83777	.	.	.	.	.	.	protein_co	ENSG00000	.	.
AL158091.1	0	0	0	0	0	0	Protein LOC100509861 [Source:UniProtKB/TrEMBL;Acc:M0R1Y7	chr20:6247	.	XM_00311	.	.	.	XP_003119	protein_co	ENSG0000	100509861	.
AL158147.2	0	0	0	0	0	0	HCG2011465; Uncharacterized protein [Source:UniProtKB/TrEMB	chr9:47928	.	.	.	.	.	.	protein_co	ENSG00000	.	.
AL160175.1	0	0	0	0	0	0	.	chr20:3025	.	.	.	.	.	.	protein_co	ENSG00000	.	.
AL160274.1	0	0	0	0	0	0	HCG17281; PRO0038; Uncharacterized protein [Source:UniProtK	chr9:35161	.	.	.	.	.	.	protein_co	ENSG00000	.	.
AL160286.1	0	0	0	0	0	0	Uncharacterized protein [Source:UniProtKB/TrEMBL;Acc:M0R0W	chr1:17933	.	.	.	.	.	.	protein_co	ENSG00000	.	.
AL161450.1	0	0	0	0	0	0	Uncharacterized protein [Source:UniProtKB/TrEMBL;Acc:M0QZJ	chr9:49852	.	.	.	.	.	.	protein_co	ENSG00000	.	.
AL161645.2	0	0	0	0	0	0	Uncharacterized protein [Source:UniProtKB/TrEMBL;Acc:M0QZJ	chr10:1353	.	.	.	.	.	.	protein_co	ENSG00000	.	.
AL161784.1	0	0	0	0	0	0	Uncharacterized protein [Source:UniProtKB/TrEMBL;Acc:M0R1V	chr9:12410	.	.	.	.	.	.	protein_co	ENSG00000	.	.
AL162389.1	0	0	0	0	0	0	Uncharacterized protein [Source:UniProtKB/TrEMBL;Acc:F8WER	chr9:11053	.	.	.	.	.	.	protein_co	ENSG00000	.	.
AL162407.1	3.00346	0.978702	1.40568	0.322394	2.26236	0.19814	CDNA FLJ20147 fis, clone COL07954; HCG1781466; Uncharacteriz	chr10:1156	.	.	.	.	.	.	protein_co	ENSG00000	.	.
AL162424.1	0	0	0	0	0	0	Uncharacterized protein [Source:UniProtKB/TrEMBL;Acc:M0QXR	chr9:12513	.	.	.	.	.	.	protein_co	ENSG00000	.	.
AL162431.1	0	0	0	0	0	0	Uncharacterized protein [Source:UniProtKB/TrEMBL;Acc:H0YFY1	chr1:18094	.	.	.	.	.	.	protein_co	ENSG00000	.	.
AL353354.1	0	0	0	0	0	0	Uncharacterized protein [Source:UniProtKB/TrEMBL;Acc:H3BLX2	chr1:28526	.	.	.	.	.	.	protein_co	ENSG00000	.	.
AL353354.2	1.16381	0	0.343168	0.845971	0	0.408668	.	chr1:28526	.	.	.	.	.	.	protein_co	ENSG00000	.	.
AL353583.1	0	0	0	0	0	0	.	chr10:2398	.	.	.	.	.	.	protein_co	ENSG00000	.	.
AL353608.1	0	0	0	0	0	0	HDCMB45P; Uncharacterized protein [Source:UniProtKB/TrEMBL	chr9:70856	.	.	.	.	.	.	protein_co	ENSG00000	.	.
AL353698.1	0	0	0	0	0	0	Uncharacterized protein [Source:UniProtKB/TrEMBL;Acc:E7EML2	chrX:56100	.	.	.	.	.	.	protein_co	ENSG00000	.	.
AL353791.1	0.028071	0	0.121236	0.065519	0.030381	0.130852	.	chr9:40028	.	.	.	.	.	.	protein_co	ENSG00000	.	.
AL354718.1	0	0	0	0	0	0	Uncharacterized protein [Source:UniProtKB/TrEMBL;Acc:Q8TBT3	chr9:45441	.	.	.	.	.	.	protein_co	ENSG00000	.	.
AL354808.2	0	0	0	0	0	0	.	chr13:2026	.	.	.	.	.	.	protein_co	ENSG00000	.	.

AL354898.1	0.034336	0	0	0	0.079718	0.075209	.	chr9:13327	.	.	.	.	.	.	protein_co	ENSG0000	.	.
AL355149.2	0	0	0	0	0	0	family with sequence similarity 231 member C [Source:EntrezGer	chr1:16865	.	.	.	.	NP_00129	XP_00672	protein_co	ENSG0000	729587, 10	.
AL355531.2	0	0	0	0	0	0	Uncharacterized protein [Source:UniProtKB/TrEMBL;Acc:M0R0Y	chr10:1312	.	.	.	.	.	.	protein_co	ENSG0000	.	.
AL356215.1	0	0	0	0	0	0	.	chr11:3515	.	.	.	.	.	.	protein_co	ENSG0000	.	.
AL356356.1	0.077755	0.039001	0.033901	0	0.044587	0	Protein LOC100996516 [Source:UniProtKB/TrEMBL;Acc:Q8WYY2	chr1:15052	.	.	.	.	.	.	protein_co	ENSG0000	.	.
AL357673.1	1.84439	0.316236	1.41544	0.059883	0.803562	1.56943	CDNA: FLJ21031 fis, clone CAE07336; HCG1780521; Uncharacteri	chr1:54635	.	.	.	.	.	.	protein_co	ENSG0000	.	.
AL358113.1	0	0	0	0.649634	0	0	Tight junction protein 2 (Zona occludens 2) isoform 1 variant; Un	chr9:71736	.	.	.	.	.	.	protein_co	ENSG0000	.	.
AL358333.1	0	0	0	0	0	0	HCG2013195; Uncharacterized protein [Source:UniProtKB/TrEM	chr14:5229	.	.	.	.	.	.	protein_co	ENSG0000	.	.
AL358813.2	0.706804	0.595276	0.49724	0.956074	0	0.732664	family with sequence similarity 231, member D [Source:HGNC Sy	chr1:14967	.	.	.	.	.	.	protein_co	ENSG0000	644634	49509
AL359091.2	0.038553	0.039679	0.033597	0	0	0.039709	CDNA: FLJ21673 fis, clone COL09042; HCG2036511; Uncharacteri	chr9:13106	.	.	.	.	.	.	protein_co	ENSG0000	.	.
AL359195.1	0.021678	0	0	0	0	0	Uncharacterized protein; cDNA FLJ46261 fis, clone TESTI4025062	chr10:8200	.	.	.	.	.	.	protein_co	ENSG0000	.	.
AL359693.1	0	0	0	0	0	0	.	chr6:82262	.	.	.	.	.	.	protein_co	ENSG0000	.	.
AL359736.1	0	0	0	0	0	0	protein PCOTH isoform 1 [Source:RefSeq peptide;Acc:NP_00101	chr13:2488	.	.	.	.	NP_00112	.	protein_co	ENSG0000	542767	.
AL360181.1	0	0	0	0	0	0	Uncharacterized protein [Source:UniProtKB/TrEMBL;Acc:M0R1D	chr10:1351	.	.	.	.	.	.	protein_co	ENSG0000	.	.
AL365202.1	0	0.661273	0	0	0	0	Metazoan signal recognition particle RNA [Source:RFAM;Acc:RF0	chr9:32182	.	.	.	.	.	.	misc_RNA	ENSG0000	.	.
AL390778.1	0	0	0	0	0	0	Uncharacterized protein [Source:UniProtKB/TrEMBL;Acc:M0QYD	chr9:13815	.	.	.	.	.	.	protein_co	ENSG0000	.	.
AL391152.1	0	0	0	0.090698	0.166358	0	.	chr14:5766	.	.	.	.	.	.	protein_co	ENSG0000	.	.
AL441883.1	0.257054	0	0.219861	0	0	0.960217	Uncharacterized protein [Source:UniProtKB/TrEMBL;Acc:M0R2E	chr6:13615	.	.	.	.	.	.	protein_co	ENSG0000	.	.
AL445665.1	0.17568	0.090486	0.226916	0	0.395018	0.092418	.	chr9:69650	.	.	.	.	.	.	transcribe	ENSG0000	.	.
AL513478.1	0	0	0	0	0	0	Uncharacterized protein [Source:UniProtKB/TrEMBL;Acc:M0R30	chr9:43135	.	.	.	.	.	.	protein_co	ENSG0000	.	.
AL583828.1	5.43E-45	1.8E-115	3.68E-25	0.425293	1.55E-26	1.23E-26	.	chr6:13358	.	.	.	.	.	.	protein_co	ENSG0000	.	.
AL589739.1	0	0	0	0	0	0	Uncharacterized protein [Source:UniProtKB/TrEMBL;Acc:M0QY5	chr1:22526	.	.	.	.	.	.	protein_co	ENSG0000	.	.
AL589765.1	7.98E-40	0	2.37E-09	0.024573	0	4.3E-08	Uncharacterized protein; cDNA FLJ36032 fis, clone TESTI2017069	chr1:15167	.	.	.	.	.	.	protein_co	ENSG0000	.	.
AL590235.1	0	0	0	0	0	1.15E-06	Uncharacterized protein [Source:UniProtKB/TrEMBL;Acc:M0QXV	chr4:35081	.	.	.	.	.	.	protein_co	ENSG0000	.	.
AL590452.1	0.049721	0	0	0	0	0.047244	Uncharacterized protein [Source:UniProtKB/TrEMBL;Acc:M0QZ7	chr1:14485	.	.	.	.	.	.	protein_co	ENSG0000	.	.
AL590483.1	0	0	0	0	0	0	Uncharacterized protein; cDNA FLJ42623 fis, clone BRACE301589	chr1:24422	.	.	.	.	.	.	protein_co	ENSG0000	.	.
AL590560.1	0	0	0	0	0	0	HCG1995379; Uncharacterized protein [Source:UniProtKB/TrEM	chr1:15987	.	.	.	.	.	.	protein_co	ENSG0000	.	.
AL590714.1	0	0	0	0	0	0	Uncharacterized protein [Source:UniProtKB/TrEMBL;Acc:M0R2L	chr1:16118	.	.	.	.	.	.	protein_co	ENSG0000	.	.
AL590822.1	0.118883	0.192744	0.170457	0.042127	0	0.088866	Uncharacterized protein [Source:UniProtKB/TrEMBL;Acc:F5H5L2	chr1:21433	.	.	.	.	.	.	protein_co	ENSG0000	.	.
AL590822.2	0.144874	0.094109	0.123452	0.051308	0	0.054014	Uncharacterized protein; cDNA FLJ36608 fis, clone TRACH201582	chr1:20049	.	.	.	.	.	.	protein_co	ENSG0000	.	.
AL591025.1	0	0	0	0	0	0	Uncharacterized protein [Source:UniProtKB/TrEMBL;Acc:H7BYS0	chr6:15895	.	.	.	.	.	.	protein_co	ENSG0000	.	.
AL591479.1	0.035234	0.034407	0.030409	0.113235	0.041171	0.078074	Uncharacterized protein [Source:UniProtKB/TrEMBL;Acc:M0R1G	chr9:70646	.	.	.	.	.	.	protein_co	ENSG0000	.	.
AL591684.1	0	0	0	0	0	0	Protein LOC100996760 [Source:UniProtKB/TrEMBL;Acc:A6NLY1]	chr10:4825	.	.	.	.	.	.	protein_co	ENSG0000	100996760	.
AL591806.1	0	0	0	0	0	0	Metazoan signal recognition particle RNA [Source:RFAM;Acc:RF0	chr1:16101	.	.	.	.	.	.	misc_RNA	ENSG0000	.	.
AL592284.1	0.768977	0.317825	0.186878	0.23254	0	0.244436	Protein LOC642441 [Source:UniProtKB/TrEMBL;Acc:F5H305]	chr1:14433	.	.	.	.	.	XP_00527	protein_co	ENSG0000	730256	.
AL596220.1	0	0	0	0	0	0	Uncharacterized protein [Source:UniProtKB/TrEMBL;Acc:M0R1Z	chr1:18634	.	.	.	.	.	.	protein_co	ENSG0000	.	.
AL603965.1	0	0	0	0	0	0	Protein LOC100996760 [Source:UniProtKB/TrEMBL;Acc:A6NF06]	chr10:4774	.	XM_00384	.	.	.	XP_00527	protein_co	ENSG0000	100996760	.
AL606500.1	0	0	0	0	0	0	.	chr1:15455	.	.	.	.	.	.	protein_co	ENSG0000	.	.
AL626787.1	0.042959	0.272048	0.11127	0.144609	0.04798	0	.	chr1:16602	.	.	.	.	.	.	protein_co	ENSG0000	.	.
AL627171.1	0	0	0	0	0	0	Uncharacterized protein [Source:UniProtKB/TrEMBL;Acc:H7BY28	chr14:5023	.	.	.	.	.	.	protein_co	ENSG0000	.	.
AL627171.2	0	0	0	0	0.076883	0	HCG1786899; PRO2610; Uncharacterized protein [Source:UniPro	chr14:5023	.	.	.	.	.	.	protein_co	ENSG0000	.	.
AL627309.1	7.83125	7.86807	7.30038	5.54629	5.57346	7.15693	Uncharacterized protein [Source:UniProtKB/TrEMBL;Acc:R4GN2	chr1:13490	.	.	.	.	.	XP_00527	protein_co	ENSG0000	728728	.
AL645608.1	0	0	0	0	0	0	Uncharacterized protein [Source:UniProtKB/TrEMBL;Acc:M0R0C	chr1:86111	.	.	.	.	.	.	protein_co	ENSG0000	.	.
AL645608.2	0	0	0	0	0	0	.	chr1:81804	.	.	.	.	.	.	protein_co	ENSG0000	.	.
AL645730.2	0	0	0	0	0	0	.	chr1:48998	.	.	.	.	.	.	protein_co	ENSG0000	.	.
AL645922.1	0	0	0	0	0	0	Uncharacterized protein [Source:UniProtKB/TrEMBL;Acc:M0R2J	chr6:31973	.	.	.	.	.	.	protein_co	ENSG0000	.	.
AL646016.1	0	0	0	0	0	0	Uncharacterized protein [Source:UniProtKB/TrEMBL;Acc:M0R2V	chr1:24025	.	.	.	.	.	.	protein_co	ENSG0000	.	.
AL669831.1	0	0	0	0	0	0	Uncharacterized protein [Source:UniProtKB/TrEMBL;Acc:M0QX2	chr1:73853	.	.	.	.	.	.	protein_co	ENSG0000	.	.
AL929472.1	0	0	0	0	0	0	.	chr1:38275	.	.	.	.	.	.	protein_co	ENSG0000	.	.
AL953854.2	0.022967	0	0.118904	0	0	0	.	chr9:47295	.	.	.	.	.	.	protein_co	ENSG0000	.	.
ALAD	5.90756	6.45389	7.19375	6.5997	5.42958	5.07957	aminolevulinate dehydratase [Source:HGNC Symbol;Acc:HGNC:3	chr9:11614	NM_00003	XM_01151	.	.	NP_00002	XP_01151	protein_co	ENSG0000	210	395
ALAS1	34.6555	38.5349	34.6867	45.5831	43.4034	41.2499	5'-aminolevulinate synthase 1 [Source:HGNC Symbol;Acc:HGNC:3	chr3:52232	NM_00068	XM_01153	.	.	NP_00067	XP_01153	protein_co	ENSG0000	211	396
ALAS2	0	0	0	0	0	0	5'-aminolevulinate synthase 2 [Source:HGNC Symbol;Acc:HGNC:3	chrX:55026	NM_00003	XM_01153	.	.	NP_00002	XP_00526	protein_co	ENSG0000	212	397
ALB	0.039481	0.113357	0.184774	0.060175	0	0.173208	albumin [Source:HGNC Symbol;Acc:HGNC:399]	chr4:74269	NM_00047	.	.	.	NP_00046	.	protein_co	ENSG0000	213	399
ALCAM	22.5474	18.7738	21.7147	20.1119	22.4946	24.8226	activated leukocyte cell adhesion molecule [Source:HGNC Symbo	chr3:10508	NM_00124	.	.	.	NP_00123	.	protein_co	ENSG0000	214	400
ALDH16A1	15.4049	16.8083	12.9357	16.8954	16.6319	10.9739	aldehyde dehydrogenase 16 family member A1 [Source:HGNC Sy	chr19:4994	NM_00114	XM_01152	.	.	NP_00113	XP_01152	protein_co	ENSG0000	126133	28114
ALDH18A1	43.5146	45.1195	42.8261	49.6193	47.1821	41.665	aldehyde dehydrogenase 18 family member A1 [Source:HGNC Sy	chr10:9736	NM_00101	XM_01154	.	.	NP_00101	XP_00671	protein_co	ENSG0000	5832	9722
ALDH1A1	0	0	0.03107	0	0	0	aldehyde dehydrogenase 1 family member A1 [Source:HGNC Sym	chr9:75515	NM_00068	.	.	.	NP_00068	.	protein_co	ENSG0000	216	402
ALDH1A2	0.022284	0.053945	0.077598	0	0.024979	0	aldehyde dehydrogenase 1 family member A2 [Source:HGNC Sym	chr15:5824	NM_00120	.	.	.	NP_00119	.	protein_co	ENSG0000	8854	15472

ALDH1A3	111.794	119.398	191.064	99.9239	103.54	62.1156	aldehyde dehydrogenase 1 family member A3 [Source:HGNC Sym	chr15:1014	NM_00069	.	.	.	NP_000684	.	protein_co	ENSG0000(	220	409
ALDH1B1	33.6734	35.4212	38.4291	40.4408	42.2881	32.2605	aldehyde dehydrogenase 1 family member B1 [Source:HGNC Sym	chr9:38392	NM_00069	XM_01151	.	.	NP_00068:	XP_011516	protein_co	ENSG0000(	219	407
ALDH1L1	0.068452	0.11791	0.13861	0.050888	0	0.050404	aldehyde dehydrogenase 1 family member L1 [Source:HGNC Sym	chr3:12582	NM_00127	XM_01151	.	.	NP_00125	XP_006713	protein_co	ENSG0000(	10840	3978
ALDH1L2	0.35743	0.487207	0.44846	0.349427	0.497052	1.22163	aldehyde dehydrogenase 1 family member L2 [Source:HGNC Sym	chr12:1053	NM_00103	XM_01701	.	.	NP_00102	XP_011536	protein_co	ENSG0000(	160428	26777
ALDH2	15.3958	17.865	18.7959	12.844	11.4829	18.9383	aldehyde dehydrogenase 2 family (mitochondrial) [Source:HGNC	chr12:1121	NM_00069	.	.	.	NP_00068	.	protein_co	ENSG0000(	217	404
ALDH3A1	0.931251	0.508176	0.440096	0.510309	0.787891	0.477363	aldehyde dehydrogenase 3 family member A1 [Source:HGNC Sym	chr17:1964	NM_00069	XM_01152	.	.	NP_00068	XP_005256	protein_co	ENSG0000(	218	405
ALDH3A2	34.8569	32.272	31.238	37.3866	37.5044	43.8949	aldehyde dehydrogenase 3 family member A2 [Source:HGNC Sym	chr17:1955	NM_00038	XM_01702	.	.	NP_00037	XP_011522	protein_co	ENSG0000(	224	403
ALDH3B1	8.66301	9.67604	8.15521	9.62961	9.52623	7.71816	aldehyde dehydrogenase 3 family member B1 [Source:HGNC Sym	chr11:6777	NM_00069	.	.	.	NP_00068	.	protein_co	ENSG0000(	221	410
ALDH3B2	0	0	0	0.032884	0.035329	0.06699	aldehyde dehydrogenase 3 family member B2 [Source:HGNC Sym	chr11:6742	NM_00069	XM_01154	.	.	NP_00068	.	protein_co	ENSG0000(	222	411
ALDH4A1	9.60981	9.97899	11.5844	13.0141	11.0449	10.1804	aldehyde dehydrogenase 4 family member A1 [Source:HGNC Sym	chr1:19197	NM_00116	XM_01154	.	.	NP_00115	XP_011540	protein_co	ENSG0000(	8659	406
ALDH5A1	5.16362	5.03823	6.57911	5.00846	4.99991	4.28923	aldehyde dehydrogenase 5 family member A1 [Source:HGNC Sym	chr6:24495	NM_00108	.	.	.	NP_00107	.	protein_co	ENSG0000(	7915	408
ALDH6A1	5.04453	5.04289	6.3582	4.1853	4.83295	5.8248	aldehyde dehydrogenase 6 family member A1 [Source:HGNC Sym	chr14:7448	NM_00127	.	.	.	NP_00126	.	protein_co	ENSG0000(	4329	7179
ALDH7A1	29.9759	29.0877	38.7099	30.0866	27.6039	36.4148	aldehyde dehydrogenase 7 family member A1 [Source:HGNC Sym	chr5:12586	NM_00118	XM_01154	.	.	NP_00117	XP_011541	protein_co	ENSG0000(	501	877
ALDH8A1	0.439712	0.39054	0.378431	0.441062	0.249679	0.644865	aldehyde dehydrogenase 8 family member A1 [Source:HGNC Sym	chr6:13523	NM_00119	XM_00671	.	.	NP_00118	.	protein_co	ENSG0000(	64577	15471
ALDH9A1	33.9349	33.9474	34.8156	36.6875	40.6224	45.0383	aldehyde dehydrogenase 9 family member A1 [Source:HGNC Sym	chr1:16563	NM_00069	XM_01150	.	.	NP_00068	XP_011507	protein_co	ENSG0000(	223	412
ALDOA	721.51	768.883	663.758	909.626	756.401	569.175	aldolase, fructose-bisphosphate A [Source:HGNC Symbol;Acc:HGI	chr16:3006	NM_00003	XM_01154	.	.	NP_00002	XP_011544	protein_co	ENSG0000(	226	414
ALDOB	0	0	0	0	0	0	aldolase, fructose-bisphosphate B [Source:HGNC Symbol;Acc:HGI	chr9:10418	NM_00003	.	.	.	NP_00002	.	protein_co	ENSG0000(	229	417
ALDOC	19.8583	20.4625	43.5128	31.7317	30.0394	40.5033	aldolase, fructose-bisphosphate C [Source:HGNC Symbol;Acc:HGI	chr17:2690	NM_00516	XM_01152	.	.	NP_00515	XP_005258	protein_co	ENSG0000(	230	418
ALG1	23.552	22.8748	19.1569	25.4343	22.1581	14.8782	ALG1, chitobiosyldiphosphodolichol beta-mannosyltransferase [S	chr16:5083	NM_01910	XM_01152	.	.	NP_06198	XP_011520	protein_co	ENSG0000(	56052	18294
ALG10	6.10259	4.48299	3.24988	3.44487	5.05226	3.80002	ALG10, alpha-1,2-glucosyltransferase [Source:HGNC Symbol;Acc:I	chr12:3417	NM_03283	.	.	.	NP_11622	.	protein_co	ENSG0000(	84920	23162
ALG10B	2.97312	2.49163	4.29587	2.45792	1.91556	3.38486	ALG10B, alpha-1,2-glucosyltransferase [Source:HGNC Symbol;Acc	chr12:3871	NM_00101	XM_01153	.	.	NP_00101	XP_005268	protein_co	ENSG0000(	144245	31088
ALG11	7.03464	8.14712	7.92435	6.54628	9.17363	9.80139	ALG11, alpha-1,2-mannosyltransferase [Source:HGNC Symbol;Acc	chr13:5258	NM_00100	.	.	.	NP_00100	.	protein_co	ENSG0000(	440138	32456
ALG12	4.23482	4.01418	3.6725	5.19359	4.26058	3.106	ALG12, alpha-1,6-mannosyltransferase [Source:HGNC Symbol;Acc	chr22:5029	NM_02410	XM_01153	.	.	NP_07701	XP_011528	protein_co	ENSG0000(	79087	19358
ALG13	27.2257	24.5044	27.1962	26.4475	28.1525	23.0445	ALG13, UDP-N-acetylglucosaminyltransferase subunit [Source:HG	chrX:11092	NM_00103	XM_01153	.	.	NP_00103	XP_005262	protein_co	ENSG0000(	79868	30881
ALG14	1.26944	0.877662	0.978674	1.22114	0.99156	1.28244	ALG14, UDP-N-acetylglucosaminyltransferase subunit [Source:HG	chr1:95439	NM_14498	.	.	.	NP_65942	.	protein_co	ENSG0000(	199857	28287
ALG1L	0.382433	0.25973	0.218923	0.277346	0.775182	0.267724	ALG1, chitobiosyldiphosphodolichol beta-mannosyltransferase lik	chr3:12564	NM_00101	.	.	.	NP_00101	XP_016861	protein_co	ENSG0000(	200810	33721
ALG1L2	0.08338	0.083869	0.144358	0.181092	0.095419	0.178411	ALG1, chitobiosyldiphosphodolichol beta-mannosyltransferase lik	chr3:12980	NM_00113	XM_01151	.	.	NP_00112	.	protein_co	ENSG0000(	644974	37258
ALG2	28.2257	28.9297	25.864	30.174	26.162	26.9018	ALG2, alpha-1,3/1,6-mannosyltransferase [Source:HGNC Symbol;	chr9:10197	NM_03308	.	.	.	NP_14907	.	protein_co	ENSG0000(	85365	23159
ALG3	41.0232	41.7109	32.784	41.8365	34.9444	22.8323	ALG3, alpha-1,3- mannosyltransferase [Source:HGNC Symbol;Acc	chr3:18396	NM_00100	XM_01151	.	.	NP_00100	XP_011510	protein_co	ENSG0000(	10195	23056
ALG5	25.6168	23.1682	19.1046	19.2402	23.4066	20.843	ALG5, dolichyl-phosphate beta-glucosyltransferase [Source:HGNC	chr13:3752	NM_00114	XM_00526	.	.	NP_00113	.	protein_co	ENSG0000(	29880	20266
ALG6	7.30815	6.70253	7.69767	5.34295	7.99984	8.80705	ALG6, alpha-1,3-glucosyltransferase [Source:HGNC Symbol;Acc:H	chr1:63833	NM_01333	.	.	.	NP_03747	.	protein_co	ENSG0000(	29929	23157
ALG8	22.008	19.2162	22.7063	26.544	25.1529	22.8777	ALG8, alpha-1,3-glucosyltransferase [Source:HGNC Symbol;Acc:H	chr11:7781	NM_00100	XM_01154	.	.	NP_00100	XP_005274	protein_co	ENSG0000(	79053	23161
ALG9	9.3088	10.8312	8.8307	9.25208	9.54482	10.4362	ALG9, alpha-1,2-mannosyltransferase [Source:HGNC Symbol;Acc:	chr11:1116	NM_00107	XM_01154	.	.	NP_00107	XP_005277	protein_co	ENSG0000(	79796	15672
ALK	0	0	0	0	0	0	anaplastic lymphoma receptor tyrosine kinase [Source:HGNC Sym	chr2:29415	NM_00430	.	.	.	NP_00429	.	protein_co	ENSG0000(	238	427
ALKBH1	8.88773	9.47158	9.03803	9.52169	9.72708	7.01463	alkB homolog 1, histone H2A dioxygenase [Source:HGNC Symbol;	chr14:7813	NM_00602	.	.	.	NP_00601	.	protein_co	ENSG0000(	8846	17911
ALKBH2	27.1396	29.9168	31.6286	29.6345	29.1007	18.9758	alkB homolog 2, alpha-ketoglutarate dependent dioxygenase [Sou	chr12:1095	NM_00100	XM_00525	.	.	NP_00100	XP_005253	protein_co	ENSG0000(	121642	32487
ALKBH3	10.9012	11.373	9.92295	12.1325	10.4527	9.39277	alkB homolog 3, alpha-ketoglutaratedependent dioxygenase [Sou	chr11:4390	NM_13917	.	.	.	NP_63191	.	protein_co	ENSG0000(	221120	30141
ALKBH4	9.83786	10.957	9.47862	12.8943	10.3684	8.60844	alkB homolog 4, lysine demethylase [Source:HGNC Symbol;Acc:H	chr7:10207	NM_01762	XM_00525	.	.	NP_06009	XP_005250	protein_co	ENSG0000(	54784	21900
ALKBH5	50.0227	51.0482	47.8033	50.8287	53.2646	46.7908	alkB homolog 5, RNA demethylase [Source:HGNC Symbol;Acc:HG	chr17:1808	NM_01775	.	.	.	NP_06022	.	protein_co	ENSG0000(	54890	25996
ALKBH6	16.6018	16.6534	15.0424	17.3637	13.4524	11.514	alkB homolog 6 [Source:HGNC Symbol;Acc:HGNC:28243]	chr19:3650	NM_00129	XM_00525	.	.	NP_00128	XP_005259	protein_co	ENSG0000(	84964	28243
ALKBH7	25.7659	29.3331	31.0653	19.9112	17.8374	18.0659	alkB homolog 7 [Source:HGNC Symbol;Acc:HGNC:21306]	chr19:6372	NM_03230	.	.	.	NP_11568	.	protein_co	ENSG0000(	84266	21306
ALKBH8	5.39732	5.64088	4.86144	5.05167	6.24701	5.50588	alkB homolog 8, tRNA methyltransferase [Source:HGNC Symbol;A	chr11:1073	NM_00130	XM_00527	.	.	NP_00128	XP_005271	protein_co	ENSG0000(	91801	25189
ALLC	0	0	0	0	0.054393	0	allantoicase [Source:HGNC Symbol;Acc:HGNC:17377]	chr2:37057	NM_01843	XM_01151	.	.	NP_06090	XP_011508	protein_co	ENSG0000(	55821	17377
ALMS1	4.00388	3.93331	4.32381	2.9924	4.0166	3.8765	ALMS1, centrosome and basal body associated protein [Source:H	chr2:73612	NM_01512	.	.	.	NP_05593	.	protein_co	ENSG0000(	7840	428
ALOX12	0	0	0.026973	0	0	0.101499	arachidonate 12-lipoxygenase, 12S type [Source:HGNC Symbol;Ac	chr17:6899	NM_00069	XM_01152	.	.	NP_00068	XP_011522	protein_co	ENSG0000(	239	429
ALOX12B	0.613753	0.639661	0.662911	0.936997	0.373197	0.564743	arachidonate 12-lipoxygenase, 12R type [Source:HGNC Symbol;A	chr17:7975	NM_00113	.	.	.	NP_00113	.	protein_co	ENSG0000(	242	430
ALOX15	0.180698	0.152078	0.440671	0.098452	0.076036	0.352952	arachidonate 15-lipoxygenase [Source:HGNC Symbol;Acc:HGNC:4	chr17:4534	NM_00114	.	.	.	NP_00113	.	protein_co	ENSG0000(	246	433
ALOX15B	0.044637	0.08936	0.164929	0	0	0.034453	arachidonate 15-lipoxygenase, type B [Source:HGNC Symbol;Acc:	chr17:7942	NM_00103	.	.	.	NP_00103	.	protein_co	ENSG0000(	247	434
ALOX5	1.30297	1.28757	1.68154	1.68424	1.61058	1.71918	arachidonate 5-lipoxygenase [Source:HGNC Symbol;Acc:HGNC:43	chr10:4586	NM_00069	XM_01153	.	.	NP_00068	.	protein_co	ENSG0000(	240	435
ALOX5AP	0	0.112058	0	0	0.120765	0	arachidonate 5-lipoxygenase activating protein [Source:HGNC Syr	chr13:3128	NM_00120	.	.	.	NP_00119	.	protein_co	ENSG0000(	241	436
ALOXE3	3.44128	3.60157	1.76814	1.91773	1.70289	1.30705	arachidonate lipoxygenase 3 [Source:HGNC Symbol;Acc:HGNC:13	chr17:7999	NM_00116	.	.	.	NP_00115	.	protein_co	ENSG0000(	59344	13743
ALPI	0	0	0.025905	0	0	0	alkaline phosphatase, intestinal [Source:HGNC Symbol;Acc:HGNC	chr2:23332	NM_00163	.	.	.	NP_00162	.	protein_co	ENSG0000(	248	437
ALPK1	3.06846	3.3076	3.11627	3.05604	3.39673	5.08809	alpha kinase 1 [Source:HGNC Symbol;Acc:HGNC:20917]	chr4:11321	NM_00110	XM_01153	.	.	NP_00109	XP_005263	protein_co	ENSG0000(	80216	20917
ALPK2	0.015579	0.048737	0.068201	0.008691	0.035113	0.135884	alpha kinase 2 [Source:HGNC Symbol;Acc:HGNC:20565]	chr18:5614	NM_05294	XM_01152	.	.	NP_44317	XP_011524	protein_co	ENSG0000(	115701	20565
ALPK3	0.347086	0.297606	0.404744	0.22554	0.188359	0.272337	alpha kinase 3 [Source:HGNC Symbol;Acc:HGNC:17574]	chr15:8535	NM_02077	XM_01702	.	.	NP_06582	XP_016877	protein_co	ENSG0000(	57538	17574
ALPL	0.188524	0.125921	0.464409	0.391799	0.505781	0.526206	alkaline phosphatase, liver/bone/kidney [Source:HGNC Symbol;A	chr1:21835	NM_00047	XM_00671	.	.	NP_00046	XP_005245	protein_co	ENSG0000(	249	438
ALPP	0	0.024671	0	0.053684	0.058131	0	alkaline phosphatase, placental [Source:HGNC Symbol;Acc:HGNC	chr2:23324	NM_00163	.	.	.	NP_00162	.	protein_co	ENSG0000(	250	439
ALPPL2	0.031391	0.030753	0	0	0.036536	0.069926	alkaline phosphatase, placental like 2 [Source:HGNC Symbol;Acc:I	chr2:23327	NM_03131	.	.	.	NP_11260	.	protein_co	ENSG0000(	251	441
ALS2	8.83833	8.85072	9.64085	8.29421	9.05733	10.5834	ALS2, alsin Rho guanine nucleotide exchange factor [Source:HGN	chr2:20256	NM_00113	XM_01700	.	.	NP_00112	XP_006712	protein_co	ENSG0000(	57679	443

ALS2CL	3.18474	2.74001	3.73038	2.02213	1.86483	1.77289	ALS2 C-terminal like [Source:HGNC Symbol;Acc:HGNC:20605]	chr3:4671	NM_00119	.	.	.	NP_00117	XP_006713	protein_co	ENSG0000	259173	20605
ALS2CR11	0	0	0	0	0	0	amyotrophic lateral sclerosis 2 chromosome region candidate 11	chr2:2023	NM_00116	XM_01151	.	.	NP_00116	XP_006712	protein_co	ENSG0000	151254	14438
ALS2CR12	0	0.06915	0.02896	0.071291	0.073574	0.033116	amyotrophic lateral sclerosis 2 chromosome region candidate 12	chr2:2021	NM_00112	XM_01151	NR_11062	.	NP_00112	XP_011508	protein_co	ENSG0000	130540	14439
ALX1	0	0	0.04717	0	0	0	ALX homeobox 1 [Source:HGNC Symbol;Acc:HGNC:1494]	chr12:8567	NM_00698	XM_00526	.	.	NP_00891	XP_005269	protein_co	ENSG0000	8092	1494
ALX3	0.093863	0.046463	0	0.050127	0.108057	0.051514	ALX homeobox 3 [Source:HGNC Symbol;Acc:HGNC:449]	chr1:1106	NM_00649	.	.	.	NP_00648	.	protein_co	ENSG0000	257	449
ALX4	0	0	0	0	0	0	ALX homeobox 4 [Source:HGNC Symbol;Acc:HGNC:450]	chr11:4428	NM_02192	.	.	.	NP_06874	.	protein_co	ENSG0000	60529	450
ALYREF	223.71	223.984	165.422	304.667	257.607	187.2	Aly/REF export factor [Source:HGNC Symbol;Acc:HGNC:19071]	chr17:7984	NM_00578	.	.	.	NP_00577	.	protein_co	ENSG0000	10189	19071
AMACR	4.75071	3.90481	3.32129	3.5926	3.47043	2.92236	alpha-methylacyl-CoA racemase [Source:HGNC Symbol;Acc:HGN	chr5:3398	NM_00116	.	.	.	NP_00116	.	protein_co	ENSG0000	23600	451
AMBN	0	0.046925	0	0	0	0	ameloblastin [Source:HGNC Symbol;Acc:HGNC:452]	chr4:71457	NM_01651	.	.	.	NP_05760	.	protein_co	ENSG0000	258	452
AMBP	0	0	0.08795	0	0	0.108777	alpha-1-microglobulin/bikunin precursor [Source:HGNC Symbol;A	chr9:11168	NM_00163	.	.	.	NP_00162	.	protein_co	ENSG0000	259	453
AMBRA1	7.935	7.87305	7.33122	7.00922	7.70355	6.47056	autophagy and beclin 1 regulator 1 [Source:HGNC Symbol;Acc:HG	chr11:4641	NM_00126	XM_00671	.	.	NP_00125	XP_005253	protein_co	ENSG0000	55626	25990
AMD1	54.1663	59.9065	63.228	71.88	82.5386	77.643	adenosylmethionine decarboxylase 1 [Source:HGNC Symbol;Acc:	chr6:11111	NM_00128	.	.	.	NP_00127	.	protein_co	ENSG0000	262	457
AMDHD1	2.37219	2.20522	2.71398	2.85235	2.50814	3.13333	amidohydrolase domain containing 1 [Source:HGNC Symbol;Acc:	chr12:9633	NM_15243	.	.	.	NP_68964	.	protein_co	ENSG0000	144193	28577
AMDHD2	16.0534	14.3255	14.9776	12.9487	9.60776	7.18922	amidohydrolase domain containing 2 [Source:HGNC Symbol;Acc:	chr16:2525	NM_00114	XM_01702	.	.	NP_00113	XP_016878	protein_co	ENSG0000	51005	24262
AMELX	0	0	0	0	0	0	amelogenin, X-linked [Source:HGNC Symbol;Acc:HGNC:461]	chrX:1115	NM_00114	XM_01154	.	.	NP_00113	XP_011543	protein_co	ENSG0000	265	461
AMELY	0	0	0	0	0	0	amelogenin, Y-linked [Source:HGNC Symbol;Acc:HGNC:462]	chrY:6733	NM_00114	XM_01153	.	.	NP_00113	XP_011529	protein_co	ENSG0000	266	462
AMER1	1.85758	2.43381	1.80572	1.83511	2.08142	1.63124	APC membrane recruitment protein 1 [Source:HGNC Symbol;Acc	chrX:6340	NM_15242	XM_01153	.	.	NP_68963	XP_011529	protein_co	ENSG0000	139285	26837
AMER2	0	0	0	0	0	0	APC membrane recruitment protein 2 [Source:HGNC Symbol;Acc	chr13:2573	NM_15270	XM_00671	.	.	NP_68991	XP_005266	protein_co	ENSG0000	219287	26360
AMER3	0	0	0	0.012868	0	0	APC membrane recruitment protein 3 [Source:HGNC Symbol;Acc	chr2:1315	NM_00110	XM_01151	.	.	NP_00109	XP_011509	protein_co	ENSG0000	205147	26771
AMFR	30.5731	30.9456	29.8442	30.6562	27.1091	28.0033	autocrine motility factor receptor [Source:HGNC Symbol;Acc:HGN	chr16:5639	NM_00114	XM_00525	.	.	NP_00113	XP_005255	protein_co	ENSG0000	267	463
AMH	5.79699	6.27117	6.47334	7.87094	6.74976	13.5429	anti-Mullerian hormone [Source:HGNC Symbol;Acc:HGNC:464]	chr19:2249	NM_00047	.	NR_03620	.	NP_00047	.	protein_co	ENSG0000	100423031	464
AMHR2	0.043356	0.151447	0.097732	0.048522	0	0	anti-Mullerian hormone receptor type 2 [Source:HGNC Symbol;A	chr12:5381	NM_00116	XM_01153	.	.	NP_00115	XP_011536	protein_co	ENSG0000	269	465
AMIGO1	0.055348	0.042778	0.058168	0.030512	0.079041	0.035813	adhesion molecule with Ig like domain 1 [Source:HGNC Symbol;A	chr1:1100	NM_02070	XM_01154	.	.	NP_06575	XP_011540	protein_co	ENSG0000	57463	20824
AMIGO2	0.769874	1.00427	0.727766	0.803106	1.24307	1.37321	adhesion molecule with Ig like domain 2 [Source:HGNC Symbol;A	chr12:4746	NM_00114	XM_00526	.	.	NP_00113	XP_005268	protein_co	ENSG0000	347902	24073
AMIGO3	0.00117	5.85E-05	3.76E-09	2.2E-07	1.83E-07	0.455646	adhesion molecule with Ig like domain 3 [Source:HGNC Symbol;A	chr3:4972	NM_19872	.	.	.	NP_94201	.	protein_co	ENSG0000	386724	24075
AMMECR1	6.29979	6.08307	5.72523	4.54169	6.60994	8.34351	Alport syndrome, mental retardation, midface hypoplasia and elli	chrX:1094	NM_00102	.	.	.	NP_00102	.	protein_co	ENSG0000	9949	467
AMMECR1L	11.4459	13.6822	10.9631	12.1851	12.6278	11.9898	AMMECR1 like [Source:HGNC Symbol;Acc:HGNC:28658]	chr2:1286	NM_00119	XM_01151	.	.	NP_00118	XP_005263	protein_co	ENSG0000	83607	28658
AMN	0.467712	0.315017	0.449796	0.34972	0.237622	0.383636	amnion associated transmembrane protein [Source:HGNC Symbo	chr14:1033	NM_03094	XM_01153	.	.	NP_11220	XP_011535	protein_co	ENSG0000	81693	14604
AMN1	13.8889	12.4004	19.8823	10.9233	13.6189	25.9594	antagonist of mitotic exit network 1 homolog [Source:HGNC Sym	chr12:3180	NM_00111	.	.	.	NP_00110	XP_016874	protein_co	ENSG0000	196394	27281
AMOT	1.82805	1.63949	2.4588	1.70582	2.10235	2.84219	angiomotin [Source:HGNC Symbol;Acc:HGNC:17810]	chrX:1120	NM_00111	XM_01153	NR_03625	.	NP_00110	XP_005262	protein_co	ENSG0000	100423009	17810
AMOTL1	8.25627	8.9231	9.44721	10.369	10.6993	11.3555	angiomotin like 1 [Source:HGNC Symbol;Acc:HGNC:17811]	chr11:945	NM_00130	XM_01154	.	.	NP_00128	XP_005273	protein_co	ENSG0000	154810	17811
AMOTL2	12.869	12.2532	8.90115	10.0165	8.86365	11.4733	angiomotin like 2 [Source:HGNC Symbol;Acc:HGNC:17812]	chr3:1340	NM_00127	XM_01700	.	.	NP_00126	XP_006713	protein_co	ENSG0000	51421	17812
AMPD1	0	0	0	0	0	0	adenosine monophosphate deaminase 1 [Source:HGNC Symbol;A	chr1:1152	NM_00003	.	.	.	NP_00002	.	protein_co	ENSG0000	270	468
AMPD2	20.2743	20.0836	17.8768	20.6108	18.6158	13.3452	adenosine monophosphate deaminase 2 [Source:HGNC Symbol;A	chr1:1101	NM_00125	XM_01154	.	.	NP_00124	XP_005270	protein_co	ENSG0000	271	469
AMPD3	7.29388	7.64555	9.00014	6.32226	6.40704	5.85347	adenosine monophosphate deaminase 3 [Source:HGNC Symbol;A	chr11:1047	NM_00048	.	.	.	NP_00047	.	protein_co	ENSG0000	272	470
AMPH	0	0	0	0	0	0	amphiphysin [Source:HGNC Symbol;Acc:HGNC:471]	chr7:3842	NM_00163	XM_01151	.	.	NP_00162	XP_006715	protein_co	ENSG0000	273	471
AMT	1.40325	1.65115	2.63656	1.54014	0.829498	1.93935	aminomethyltransferase [Source:HGNC Symbol;Acc:HGNC:473]	chr3:4945	NM_00048	.	.	.	NP_00047	.	protein_co	ENSG0000	275	473
AMTN	0	0	0	0	0	0	amelotin [Source:HGNC Symbol;Acc:HGNC:33188]	chr4:7138	NM_00128	.	.	.	NP_00127	.	protein_co	ENSG0000	401138	33188
AMY1A	0	0	0	0	0	0	amylase, alpha 1A (salivary) [Source:HGNC Symbol;Acc:HGNC:474	chr1:1041	NM_00100	.	.	.	NP_00100	.	protein_co	ENSG0000	276	474
AMY1B	0	0	0	0	0	0	amylase, alpha 1B (salivary) [Source:HGNC Symbol;Acc:HGNC:475	chr1:1042	NM_00100	XM_01700	.	.	NP_00100	XP_011539	protein_co	ENSG0000	278	475
AMY1C	0	0	0	0	0	0	amylase, alpha 1C (salivary) [Source:HGNC Symbol;Acc:HGNC:476	chr1:1042	NM_00100	XM_01154	.	.	NP_00100	XP_011539	protein_co	ENSG0000	278	476
AMY2A	0	0	0.038457	0	0	0	amylase, alpha 2A (pancreatic) [Source:HGNC Symbol;Acc:HGNC:	chr1:1041	NM_00069	.	.	.	NP_00069	.	protein_co	ENSG0000	279	477
AMY2B	0.501828	0.677003	0.725522	0.123379	0.385928	0.12187	amylase, alpha 2B (pancreatic) [Source:HGNC Symbol;Acc:HGNC:	chr1:1040	NM_02097	.	.	.	NP_06618	.	protein_co	ENSG0000	280	478
AMZ1	0	0	0	0	0	0	archaelysin family metallopeptidase 1 [Source:HGNC Symbol;Acc	chr7:2719	NM_00128	XM_01151	.	.	NP_00127	XP_011513	protein_co	ENSG0000	155185	22231
AMZ2	103.01	92.0545	101.15	97.7359	94.5129	94.6754	archaelysin family metallopeptidase 2 [Source:HGNC Symbol;Acc	chr17:6624	NM_00103	XM_01702	.	.	NP_00102	XP_005257	protein_co	ENSG0000	51321	28041
ANAPC1	13.8086	14.7178	13.856	13.8031	15.1016	13.1784	anaphase promoting complex subunit 1 [Source:HGNC Symbol;Ac	chr2:1125	NM_02266	XM_01151	.	.	NP_07315	XP_011509	protein_co	ENSG0000	64682	19988
ANAPC10	15.7127	12.8273	15.886	15.2824	18.251	16.3147	anaphase promoting complex subunit 10 [Source:HGNC Symbol;A	chr4:1459	NM_00125	XM_01153	.	.	NP_00124	XP_011529	protein_co	ENSG0000	10393	24077
ANAPC11	122.707	128.276	118.45	148.153	117.569	127.826	anaphase promoting complex subunit 11 [Source:HGNC Symbol;A	chr17:7984	NM_00100	.	.	.	NP_00100	.	protein_co	ENSG0000	51529	14452
ANAPC13	41.7669	38.7764	33.8765	40.2991	45.3414	45.3606	anaphase promoting complex subunit 13 [Source:HGNC Symbol;A	chr3:1341	NM_00124	.	.	.	NP_00122	.	protein_co	ENSG0000	25847	24540
ANAPC15	29.3575	32.414	23.1378	34.1075	25.4501	22.0366	anaphase promoting complex subunit 15 [Source:HGNC Symbol;A	chr11:717	NM_00127	.	.	.	NP_00126	XP_016872	protein_co	ENSG0000	25906	24531
ANAPC16	24.1292	22.2217	20.0572	24.963	23.51	25.4618	anaphase promoting complex subunit 16 [Source:HGNC Symbol;A	chr10:7385	NM_00124	.	.	.	NP_00122	.	protein_co	ENSG0000	119504	26976
ANAPC2	18.9213	19.9757	19.5692	22.2222	18.9597	12.7149	anaphase promoting complex subunit 2 [Source:HGNC Symbol;Ac	chr9:1400	NM_01336	.	.	.	NP_03749	.	protein_co	ENSG0000	29882	19989
ANAPC4	13.3748	12.4221	12.916	12.2144	14.1446	12.3733	anaphase promoting complex subunit 4 [Source:HGNC Symbol;Ac	chr4:2537	NM_00128	XM_01700	.	.	NP_00127	XP_011512	protein_co	ENSG0000	29945	19990
ANAPC5	57.5086	57.0831	68.1321	71.5228	64.2516	55.71	anaphase promoting complex subunit 5 [Source:HGNC Symbol;Ac	chr12:1217	NM_00113	XM_00671	.	.	NP_00113	XP_005253	protein_co	ENSG0000	51433	15713
ANAPC7	30.2006	29.7361	30.3478	37.9321	36.6541	33.8886	anaphase promoting complex subunit 7 [Source:HGNC Symbol;Ac	chr12:1108	NM_00113	XM_01153	.	.	NP_00113	XP_005253	protein_co	ENSG0000	51434	17380
ANG	6.04984	6.34655	4.44224	6.73557	5.27911	6.53562	angiogenin [Source:HGNC Symbol;Acc:HGNC:483]	chr14:2111	NM_00109	.	.	.	NP_00109	.	protein_co	ENSG0000	283	483
ANGEL1	10.0937	10.4639	13.5433	8.77481	9.19409	9.22562	angel homolog 1 [Source:HGNC Symbol;Acc:HGNC:19961]	chr14:7725	NM_01530	.	.	.	NP_05612	.	protein_co	ENSG0000	23357	19961
ANGEL2	6.72506	6.95896	5.79848	6.3262	7.51124	6.41375	angel homolog 2 [Source:HGNC Symbol;Acc:HGNC:30534]	chr1:2131	NM_00130	XM_01700	.	.	NP_00128	XP_005273	protein_co	ENSG0000	90806	30534
ANGPT1	0	0	0	0	0	0	angiopoietin 1 [Source:HGNC Symbol;Acc:HGNC:484]	chr8:1082	NM_00114	.	.	.	NP_00113	.	protein_co	ENSG0000	284	484

ANGPT2	0.42126	0.368561	0.633517	1.14502	1.69886	3.68965	angiopoietin 2 [Source:HGNC Symbol;Acc:HGNC:485]	chr8:62641	NM_00111	.	.	.	NP_00111	XP_016868	protein_co	ENSG0000	285	485
ANGPT4	0.015487	0.047393	0.026826	0	0	0.147789	angiopoietin 4 [Source:HGNC Symbol;Acc:HGNC:487]	chr20:8505	NM_01598	XM_01152	.	.	NP_05706	XP_011527	protein_co	ENSG0000	51378	487
ANGPTL1	0	0	0	0	0	0	angiopoietin like 1 [Source:HGNC Symbol;Acc:HGNC:489]	chr1:17869	NM_00467	XM_00524	.	.	NP_00466	XP_005245	protein_co	ENSG0000	9068	489
ANGPTL2	0	0	0	0.032161	0	0	angiopoietin like 2 [Source:HGNC Symbol;Acc:HGNC:490]	chr9:12967	NM_01209	.	.	.	NP_03623	.	protein_co	ENSG0000	23452	490
ANGPTL3	0	0	0	0	0	0	angiopoietin like 3 [Source:HGNC Symbol;Acc:HGNC:491]	chr1:62920	NM_01449	.	.	.	NP_05531	.	protein_co	ENSG0000	27329	491
ANGPTL4	6.35385	8.77745	13.985	5.11589	7.18094	8.03725	angiopoietin like 4 [Source:HGNC Symbol;Acc:HGNC:16039]	chr19:8429	NM_00103	XM_00527	.	.	NP_00103	XP_005272	protein_co	ENSG0000	51129	16039
ANGPTL5	0	0	0	0	0	0	angiopoietin like 5 [Source:HGNC Symbol;Acc:HGNC:19705]	chr11:1017	NM_17812	.	.	.	NP_83522	.	protein_co	ENSG0000	253935	19705
ANGPTL6	0.138981	0.135142	0.100293	0.221866	0	0.130868	angiopoietin like 6 [Source:HGNC Symbol;Acc:HGNC:23140]	chr19:1019	NM_00132	XM_01152	.	.	NP_00130	XP_005260	protein_co	ENSG0000	83854	23140
ANGPTL7	0	0	0	0	0	0	angiopoietin like 7 [Source:HGNC Symbol;Acc:HGNC:24078]	chr1:11166	NM_02114	.	.	.	NP_06696	.	protein_co	ENSG0000	10218	24078
ANGPTL8	0	0	0	0	0	0	angiopoietin like 8 [Source:HGNC Symbol;Acc:HGNC:24933]	chr19:1130	NM_01868	.	.	.	NP_06115	.	protein_co	ENSG0000	55908	24933
ANHX	0	0	0	0	0	0	anomalous homeobox [Source:HGNC Symbol;Acc:HGNC:40024]	chr12:1337	NM_00119	XM_01153	.	.	NP_00117	XP_006719	protein_co	ENSG0000	647589	40024
ANK1	0.214183	0.189806	0.241151	0.145816	0.183115	0.172646	ankyrin 1 [Source:HGNC Symbol;Acc:HGNC:492]	chr8:41510	NM_00003	XM_01154	.	.	NP_00002	XP_005273	protein_co	ENSG0000	286	492
ANK2	0.62075	0.445324	0.718969	0.301476	0.401052	1.04519	ankyrin 2, neuronal [Source:HGNC Symbol;Acc:HGNC:493]	chr4:11373	NM_00112	XM_01700	.	.	NP_00112	XP_005263	protein_co	ENSG0000	287	493
ANK3	1.633	1.04749	1.95183	1.246	1.21149	1.80639	ankyrin 3, node of Ranvier (ankyrin G) [Source:HGNC Symbol;Acc:	chr10:6178	NM_00114	XM_01701	.	.	NP_00114	XP_005269	protein_co	ENSG0000	288	494
ANKAR	0.073154	0.059701	0.031801	0.084282	0.060064	0.01741	ankyrin and armadillo repeat containing [Source:HGNC Symbol;A	chr2:19054	NM_14470	XM_01151	.	.	NP_65330	XP_011508	protein_co	ENSG0000	150709	26350
ANKDD1A	3.29069	4.22885	4.66349	3.69616	3.65395	2.76902	ankyrin repeat and death domain containing 1A [Source:HGNC Sy	chr15:6513	NM_18270	.	.	.	NP_87436	.	protein_co	ENSG0000	348094	28002
ANKDD1B	0.343724	0.298425	0.224266	0.352438	0.161498	0.174752	ankyrin repeat and death domain containing 1B [Source:HGNC Sy	chr5:74907	NM_00127	XM_01154	.	.	NP_00126	XP_011541	protein_co	ENSG0000	728780	32525
ANKEF1	9.31453	9.58708	11.7441	9.14577	10.5043	11.3075	ankyrin repeat and EF-hand domain containing 1 [Source:HGNC S	chr20:1001	NM_00130	XM_00526	.	.	NP_00129	XP_005260	protein_co	ENSG0000	63926	15803
ANKFN1	0.231711	0.184362	0.204518	0.256788	0.140903	0.26586	ankyrin repeat and fibronectin type III domain containing 1 [Sour	chr17:5423	NM_15322	XM_01152	.	.	NP_69496	XP_006721	protein_co	ENSG0000	162282	26766
ANKFY1	10.6762	11.008	10.6242	10.0033	11.2492	11.3642	ankyrin repeat and FYVE domain containing 1 [Source:HGNC Sym	chr17:4046	NM_00125	XM_01702	.	.	NP_00124	XP_005256	protein_co	ENSG0000	51479	20763
ANKH	5.23794	5.41378	8.52281	5.87741	5.13871	7.0115	ANKH inorganic pyrophosphate transport regulator [Source:HGN	chr5:14704	NM_05402	XM_01700	.	.	NP_47336	XP_016865	protein_co	ENSG0000	56172	15492
ANKHD1	28.4266	29.233	27.8729	24.4295	26.7167	27.8469	ankyrin repeat and KH domain containing 1 [Source:HGNC Symbo	chr5:13978	NM_00119	.	.	.	NP_00118	.	protein_co	ENSG0000	54882	24714
ANKHD1-EIF4EBP3	0.012319	0.001278	0.002255	0.001648	0.002962	0.038278	ANKHD1-EIF4EBP3 readthrough [Source:HGNC Symbol;Acc:HGNC	chr5:13978	NM_02069	.	.	.	NP_06574	.	protein_co	ENSG0000	404734	33530
ANKIB1	8.84925	10.0825	9.17531	12.4107	12.6985	14.0767	ankyrin repeat and IBR domain containing 1 [Source:HGNC Symb	chr7:91875	NM_01900	XM_01151	.	.	NP_06187	XP_005250	protein_co	ENSG0000	54467	22215
ANKK1	0.2244	0.277766	0.168829	0.119864	0.195851	0.308968	ankyrin repeat and kinase domain containing 1 [Source:HGNC Sy	chr11:1132	NM_17851	XM_01154	.	.	NP_84860	XP_011541	protein_co	ENSG0000	255239	21027
ANKLE1	5.97659	7.39375	5.70135	6.4965	7.35387	3.05917	ankyrin repeat and LEM domain containing 1 [Source:HGNC Sym	chr19:1739	NM_00127	.	.	.	NP_00126	.	protein_co	ENSG0000	126549	26812
ANKLE2	21.5219	21.1862	20.3148	20.4192	19.4745	17.1894	ankyrin repeat and LEM domain containing 2 [Source:HGNC Sym	chr12:1333	NM_01511	XM_00671	.	.	NP_05592	XP_005266	protein_co	ENSG0000	23141	29101
ANKMY1	4.93739	4.87102	4.35735	4.71408	4.24534	4.42626	ankyrin repeat and MYND domain containing 1 [Source:HGNC Sy	chr2:24141	NM_00128	XM_01151	.	.	NP_00126	XP_006712	protein_co	ENSG0000	51281	20987
ANKMY2	15.5926	14.2692	19.4173	14.9513	14.9156	19.9496	ankyrin repeat and MYND domain containing 2 [Source:HGNC Sy	chr7:16639	NM_02031	XM_01151	.	.	NP_06471	XP_011513	protein_co	ENSG0000	57037	25370
ANKRA2	7.02415	6.25881	9.3077	5.23213	5.02125	9.16433	ankyrin repeat family A member 2 [Source:HGNC Symbol;Acc:HG	chr5:72848	NM_02303	XM_00524	.	.	NP_07552	XP_005248	protein_co	ENSG0000	57763	13208
ANKRD1	0.168393	0.105739	0	0	0.07537	0	ankyrin repeat domain 1 [Source:HGNC Symbol;Acc:HGNC:15819	chr10:9267	NM_01439	.	.	.	NP_05520	.	protein_co	ENSG0000	27063	15819
ANKRD10	58.4705	52.4841	50.2446	34.4065	34.8678	36.4508	ankyrin repeat domain 10 [Source:HGNC Symbol;Acc:HGNC:2026	chr13:1115	NM_00128	.	.	.	NP_00127	.	protein_co	ENSG0000	55608	20265
ANKRD11	35.1192	39.6271	29.8204	29.0817	33.8076	43.4738	ankyrin repeat domain 11 [Source:HGNC Symbol;Acc:HGNC:2131	chr16:8933	NM_00125	XM_01702	.	.	NP_00124	XP_006721	protein_co	ENSG0000	29123	21316
ANKRD12	2.94403	2.82416	3.02647	2.42533	3.96472	5.21705	ankyrin repeat domain 12 [Source:HGNC Symbol;Acc:HGNC:2913	chr18:9136	NM_00108	XM_01702	.	.	NP_00107	XP_005258	protein_co	ENSG0000	23253	29135
ANKRD13A	7.47687	8.6536	9.54009	7.33399	6.98548	8.6985	ankyrin repeat domain 13A [Source:HGNC Symbol;Acc:HGNC:212	chr12:1104	NM_03312	XM_01153	.	.	NP_14911	XP_005254	protein_co	ENSG0000	88455	21268
ANKRD13B	8.83735	6.87269	6.91569	7.97273	6.38011	4.87656	ankyrin repeat domain 13B [Source:HGNC Symbol;Acc:HGNC:263	chr17:2792	NM_15234	XM_01152	.	.	NP_68955	XP_005257	protein_co	ENSG0000	124930	26363
ANKRD13C	8.3323	8.82823	8.80841	8.84033	7.96254	8.48624	ankyrin repeat domain 13C [Source:HGNC Symbol;Acc:HGNC:253	chr1:70726	NM_03081	XM_00671	.	.	NP_11044	XP_005271	protein_co	ENSG0000	81573	25374
ANKRD13D	13.9376	13.0748	14.9361	16.2779	11.516	13.9485	ankyrin repeat domain 13D [Source:HGNC Symbol;Acc:HGNC:278	chr11:6705	NM_20735	XM_01154	.	.	NP_99723	XP_011543	protein_co	ENSG0000	338692	27880
ANKRD16	4.06097	4.47528	3.06508	3.56067	3.21667	3.39834	ankyrin repeat domain 16 [Source:HGNC Symbol;Acc:HGNC:2347	chr10:5903	NM_00100	.	.	.	NP_00100	.	protein_co	ENSG0000	54522	23471
ANKRD17	19.238	20.7886	19.4878	16.6057	18.5769	17.8902	ankyrin repeat domain 17 [Source:HGNC Symbol;Acc:HGNC:2357	chr4:73939	NM_00128	XM_00526	.	.	NP_00127	XP_005265	protein_co	ENSG0000	26057	23575
ANKRD18A	2.03122	2.336	4.10483	1.84686	2.15209	2.39988	ankyrin repeat domain 18A [Source:HGNC Symbol;Acc:HGNC:236	chr9:38571	NM_14719	XM_01151	.	.	NP_67172	XP_006716	protein_co	ENSG0000	253650	23643
ANKRD18B	0.14196	0.057462	0.093115	0.040854	0.156123	0.051158	ankyrin repeat domain 18B [Source:HGNC Symbol;Acc:HGNC:236	chr9:33524	NM_00124	.	.	.	NP_00123	.	protein_co	ENSG0000	441459	23644
ANKRD2	0.062807	0	0.12262	0.06697	0	0	ankyrin repeat domain 2 [Source:HGNC Symbol;Acc:HGNC:495]	chr10:9933	NM_00112	.	.	.	NP_00112	.	protein_co	ENSG0000	26287	495
ANKRD20A1	0.057315	0.123185	0.016702	0.021854	0.08493	0.093042	ankyrin repeat domain 20 family member A1 [Source:HGNC Symb	chr9:67926	NM_03225	XM_01154	.	.	NP_11562	XP_005272	protein_co	ENSG0000	84210	23665
ANKRD20A2	0	0.423954	0.173423	0.448994	0.243346	0	ankyrin repeat domain 20 family member A2 [Source:HGNC Symb	chr9:42368	NM_00101	XM_01151	.	.	NP_00101	XP_011516	protein_co	ENSG0000	441430	31979
ANKRD20A3	0	0	0	0.020191	0.019563	0	ankyrin repeat domain 20 family member A3 [Source:HGNC Symb	chr9:43089	NM_00101	XM_01154	.	.	NP_00101	XP_005272	protein_co	ENSG0000	441425	31981
ANKRD20A4	0.094937	0.073542	0.089667	0.117549	0.133166	0.149813	ankyrin repeat domain 20 family member A4 [Source:HGNC Symb	chr9:69381	NM_00109	.	.	.	NP_00109	.	protein_co	ENSG0000	728747	31982
ANKRD22	2.21621	1.9053	1.33029	1.96333	2.05722	3.30141	ankyrin repeat domain 22 [Source:HGNC Symbol;Acc:HGNC:2832	chr10:9058	NM_14459	.	.	.	NP_65319	.	protein_co	ENSG0000	118932	28321
ANKRD23	1.97919	1.29059	1.73413	1.94742	1.502	1.42049	ankyrin repeat domain 23 [Source:HGNC Symbol;Acc:HGNC:2447	chr2:97503	NM_14499	.	.	.	NP_65943	.	protein_co	ENSG0000	200539	24470
ANKRD24	1.05625	0.92513	1.59703	0.552785	0.569194	0.813937	ankyrin repeat domain 24 [Source:HGNC Symbol;Acc:HGNC:2942	chr19:4183	NM_13347	XM_01152	.	.	NP_59773	XP_011526	protein_co	ENSG0000	170961	29424
ANKRD26	1.68989	1.25221	1.4558	0.629374	0.700805	0.688932	ankyrin repeat domain 26 [Source:HGNC Symbol;Acc:HGNC:2918	chr10:2729	NM_00125	XM_01151	.	.	NP_00124	XP_006717	protein_co	ENSG0000	22852	29186
ANKRD27	15.3851	16.0789	16.4181	13.3916	13.9806	15.9288	ankyrin repeat domain 27 [Source:HGNC Symbol;Acc:HGNC:2531	chr19:3308	NM_03213	XM_00672	.	.	NP_11551	XP_006723	protein_co	ENSG0000	84079	25310
ANKRD28	12.0434	12.0472	10.5078	8.77583	10.2175	7.86659	ankyrin repeat domain 28 [Source:HGNC Symbol;Acc:HGNC:2902	chr3:15708	NM_00119	XM_01153	.	.	NP_00118	XP_005265	protein_co	ENSG0000	23243	29024
ANKRD29	5.0775	4.77059	5.80309	6.79737	7.34599	6.82669	ankyrin repeat domain 29 [Source:HGNC Symbol;Acc:HGNC:2711	chr18:2117	NM_00130	XM_01152	.	.	NP_00129	XP_011524	protein_co	ENSG0000	147463	27110
ANKRD30A	0	0	0	0.014782	0	0	ankyrin repeat domain 30A [Source:HGNC Symbol;Acc:HGNC:172	chr10:3741	NM_05299	XM_01151	.	.	NP_44372	XP_011518	protein_co	ENSG0000	91074	17234
ANKRD30B	0	0	0	0	0	0	ankyrin repeat domain 30B [Source:HGNC Symbol;Acc:HGNC:241	chr18:1474	NM_00114	XM_01152	.	.	NP_00113	XP_006722	protein_co	ENSG0000	374860	24165
ANKRD30BL	0	0	0	0	0	0	ankyrin repeat domain 30B-like [Source:HGNC Symbol;Acc:HGNC	chr2:13290	.	.	NR_02702	.	.	.	protein_co	ENSG0000	554226	35167
ANKRD31	0.105997	0.045877	0.134868	0.02434	0.069984	0.07164	ankyrin repeat domain 31 [Source:HGNC Symbol;Acc:HGNC:2685	chr5:74364	NM_00116	XM_01700	.	.	NP_00115	XP_011541	protein_co	ENSG0000	256006	26853
ANKRD33	0	0	0.043662	0	0	0	ankyrin repeat domain 33 [Source:HGNC Symbol;Acc:HGNC:1378	chr12:5228	NM_00113	XM_01153	.	.	NP_00112	.	protein_co	ENSG0000	341405	13788

ANKRD33B	6.03456	6.57738	6.4965	3.36245	4.29342	3.94965	ankyrin repeat domain 33B [Source:HGNC Symbol;Acc:HGNC:352	chr5:10564	NM_00116	XM_00524	.	.	NP_00115	XP_005248	protein_co	ENSG0000	651746	35240
ANKRD34A	0.516795	0.491833	0.890015	0.433405	0.378633	0.608805	ankyrin repeat domain 34A [Source:HGNC Symbol;Acc:HGNC:276	chr1:14545	NM_00103	XM_01150	.	.	NP_00103	XP_005277	protein_co	ENSG0000	284615	27639
ANKRD34B	0	0	0	0	0	0	ankyrin repeat domain 34B [Source:HGNC Symbol;Acc:HGNC:337	chr5:79852	NM_00100	XM_01154	.	.	NP_00100	XP_006714	protein_co	ENSG0000	340120	33736
ANKRD34C	0	0	0	0.03537	0	0	ankyrin repeat domain 34C [Source:HGNC Symbol;Acc:HGNC:338	chr15:7958	NM_00114	XM_01152	.	.	NP_00113	XP_011519	protein_co	ENSG0000	390616	33888
ANKRD35	0.020819	0	0.018115	0.022373	0	0.022841	ankyrin repeat domain 35 [Source:HGNC Symbol;Acc:HGNC:2632	chr1:14554	NM_00128	.	.	.	NP_00126	.	protein_co	ENSG0000	148741	26323
ANKRD36	1.14073	0.913285	1.00839	0.381771	0.461058	0.65558	ankyrin repeat domain 36 [Source:HGNC Symbol;Acc:HGNC:2407	chr2:97779	NM_00116	XM_01151	.	.	NP_00115	XP_006712	protein_co	ENSG0000	375248	24079
ANKRD36B	0.497653	0.603796	0.55523	0.294186	0.393754	0.424744	ankyrin repeat domain 36B [Source:HGNC Symbol;Acc:HGNC:293	chr2:98109	NM_02519	XM_01151	.	.	NP_07946	XP_005264	protein_co	ENSG0000	57730	29333
ANKRD36C	0.906029	0.807974	1.26836	0.237437	0.688091	0.517932	ankyrin repeat domain 36C [Source:HGNC Symbol;Acc:HGNC:329	chr2:96514	.	.	.	.	.	.	protein_co	ENSG0000	.	32946
ANKRD37	4.60661	5.03654	4.89011	4.96839	4.13863	10.176	ankyrin repeat domain 37 [Source:HGNC Symbol;Acc:HGNC:2959	chr4:18631	NM_18172	.	.	.	NP_85907	XP_016863	protein_co	ENSG0000	353322	29593
ANKRD39	17.2964	15.8463	15.9671	20.436	16.8781	12.1961	ankyrin repeat domain 39 [Source:HGNC Symbol;Acc:HGNC:2864	chr2:97513	NM_01646	.	.	.	NP_05755	.	protein_co	ENSG0000	51239	28640
ANKRD40	35.0212	36.8387	34.7835	35.3546	38.1756	36.9093	ankyrin repeat domain 40 [Source:HGNC Symbol;Acc:HGNC:2823	chr17:4877	NM_05285	XM_01702	.	.	NP_44308	XP_016880	protein_co	ENSG0000	91369	28233
ANKRD42	5.19764	5.44696	5.00326	3.61093	4.66895	3.54578	ankyrin repeat domain 42 [Source:HGNC Symbol;Acc:HGNC:2675	chr11:8290	NM_00130	.	.	.	NP_00128	.	protein_co	ENSG0000	338699	26752
ANKRD44	0.56454	0.493596	0.846088	0.759005	0.509056	0.807165	ankyrin repeat domain 44 [Source:HGNC Symbol;Acc:HGNC:2525	chr2:19785	NM_15369	XM_01151	.	.	NP_71018	.	protein_co	ENSG0000	91526	25259
ANKRD45	0.389749	0.413045	0.427983	0.219917	0.435807	0.483501	ankyrin repeat domain 45 [Source:HGNC Symbol;Acc:HGNC:2478	chr1:17357	NM_19849	XM_01150	.	.	NP_94089	XP_011507	protein_co	ENSG0000	339416	24786
ANKRD46	7.03268	7.45018	8.51943	7.64054	8.49028	8.12664	ankyrin repeat domain 46 [Source:HGNC Symbol;Acc:HGNC:2722	chr8:10152	NM_00127	XM_01701	.	.	NP_00125	XP_006716	protein_co	ENSG0000	157567	27229
ANKRD49	7.29777	7.07726	8.30713	5.5296	6.84197	5.87485	ankyrin repeat domain 49 [Source:HGNC Symbol;Acc:HGNC:2597	chr11:9414	NM_01770	.	.	.	NP_06017	XP_016873	protein_co	ENSG0000	54851	25970
ANKRD50	6.2773	6.25036	7.72511	6.72071	8.98694	10.9882	ankyrin repeat domain 50 [Source:HGNC Symbol;Acc:HGNC:2922	chr4:12558	NM_00116	XM_01153	.	.	NP_00116	XP_011530	protein_co	ENSG0000	57182	29223
ANKRD52	16.7593	17.0901	16.087	16.0682	17.2652	11.5089	ankyrin repeat domain 52 [Source:HGNC Symbol;Acc:HGNC:2661	chr12:5662	NM_17359	XM_01153	.	.	NP_77586	XP_011536	protein_co	ENSG0000	283373	26614
ANKRD53	0.074767	0.084628	0	0.051542	0	0.053625	ankyrin repeat domain 53 [Source:HGNC Symbol;Acc:HGNC:2569	chr2:71163	NM_00111	XM_01153	.	.	NP_00110	XP_005264	protein_co	ENSG0000	79998	25691
ANKRD54	14.6894	14.089	14.6035	20.1789	13.1712	14.6576	ankyrin repeat domain 54 [Source:HGNC Symbol;Acc:HGNC:2518	chr22:3822	NM_13879	XM_01702	NR_03039	.	NP_62015	XP_006724	protein_co	ENSG0000	724028	25185
ANKRD55	0	0	0	0.030392	0	0	ankyrin repeat domain 55 [Source:HGNC Symbol;Acc:HGNC:2568	chr5:55395	NM_02466	XM_01700	.	.	NP_07894	XP_016865	protein_co	ENSG0000	79722	25681
ANKRD6	3.26973	3.62477	4.36334	3.62239	4.06779	3.55827	ankyrin repeat domain 6 [Source:HGNC Symbol;Acc:HGNC:17280	chr6:90142	NM_00124	XM_01701	.	.	NP_00122	XP_005248	protein_co	ENSG0000	22881	17280
ANKRD60	0	0	0	0	0	0	ankyrin repeat domain 60 [Source:HGNC Symbol;Acc:HGNC:1621	chr20:5679	NM_00130	.	.	.	NP_00129	.	protein_co	ENSG0000	140731	16217
ANKRD61	0.270516	0.110955	0.093562	0.177671	0.246826	0.171528	ankyrin repeat domain 61 [Source:HGNC Symbol;Acc:HGNC:2246	chr7:60488	NM_00127	.	.	.	NP_00125	.	protein_co	ENSG0000	10031084	22467
ANKRD62	0.095071	0.020311	0.049966	0.021599	0.06356	0.130859	ankyrin repeat domain 62 [Source:HGNC Symbol;Acc:HGNC:3524	chr18:1209	NM_00127	XM_01152	.	.	NP_00126	XP_011523	protein_co	ENSG0000	342850	35241
ANKRD63	0.448576	0	0.124667	0.468566	0.359757	0.351964	ankyrin repeat domain 63 [Source:HGNC Symbol;Acc:HGNC:4002	chr15:4057	NM_00119	.	.	.	NP_00117	.	protein_co	ENSG0000	10013124	40027
ANKRD65	0.093811	0	0.246849	0.151833	0.133498	0	ankyrin repeat domain 65 [Source:HGNC Symbol;Acc:HGNC:4295	chr1:13537	NM_00114	XM_01154	.	.	NP_00113	XP_005244	protein_co	ENSG0000	441869	42950
ANKRD66	0	0	0	0	0	0	ankyrin repeat domain 66 [Source:HGNC Symbol;Acc:HGNC:4466	chr6:46714	NM_00116	.	.	.	NP_00115	.	protein_co	ENSG0000	10028771	44669
ANKRD7	0	0	0	0	0	0	ankyrin repeat domain 7 [Source:HGNC Symbol;Acc:HGNC:18588	chr7:11785	NM_01964	.	.	.	NP_06261	.	protein_co	ENSG0000	56311	18588
ANKRD9	11.758	16.2291	16.1439	10.5901	8.44563	15.047	ankyrin repeat domain 9 [Source:HGNC Symbol;Acc:HGNC:20096	chr14:1028	NM_15232	XM_00526	.	.	NP_68953	XP_005267	protein_co	ENSG0000	122416	20096
ANKS1A	9.79337	10.3302	10.4921	11.1844	11.3028	9.64366	ankyrin repeat and sterile alpha motif domain containing 1A [Sou	chr6:34857	NM_01524	XM_01151	.	.	NP_05606	XP_005249	protein_co	ENSG0000	23294	20961
ANKS1B	0.112842	0	0	0.091316	0	0.041467	ankyrin repeat and sterile alpha motif domain containing 1B [Sou	chr12:9903	NM_00120	XM_01153	.	.	NP_00119	XP_005269	protein_co	ENSG0000	56899	24600
ANKS3	4.82329	5.2769	5.26557	5.31673	4.20865	3.93715	ankyrin repeat and sterile alpha motif domain containing 3 [Sour	chr16:4746	NM_00124	XM_01702	.	.	NP_00122	XP_005255	protein_co	ENSG0000	124401	29422
ANKS4B	0	0.016091	0	0	0.034402	0	ankyrin repeat and sterile alpha motif domain containing 4B [Sou	chr16:2124	NM_14586	.	.	.	NP_66587	.	protein_co	ENSG0000	257629	26795
ANKS6	8.8913	9.81781	10.7627	9.12054	8.36298	7.96297	ankyrin repeat and sterile alpha motif domain containing 6 [Sour	chr9:10149	NM_17355	XM_01151	.	.	NP_77582	XP_005251	protein_co	ENSG0000	203286	26724
ANKUB1	0	0	0.027917	0	0	0	ankyrin repeat and ubiquitin domain containing 1 [Source:HGNC	chr3:14947	NM_00114	XM_01151	.	.	NP_00113	XP_011511	protein_co	ENSG0000	389161	29642
ANKZF1	13.9548	14.9269	15.7736	12.9348	12.3168	16.9413	ankyrin repeat and zinc finger domain containing 1 [Source:HGN	chr2:22009	NM_00104	XM_01151	.	.	NP_00103	XP_005246	protein_co	ENSG0000	55139	25527
ANLN	40.9116	42.2137	37.3671	68.5749	73.6395	58.6996	anillin actin binding protein [Source:HGNC Symbol;Acc:HGNC:140	chr7:36363	NM_00128	XM_01701	.	.	NP_00127	XP_006715	protein_co	ENSG0000	54443	14082
ANO1	16.8643	18.0162	21.396	19.0797	15.0393	13.6804	anoctamin 1 [Source:HGNC Symbol;Acc:HGNC:21625]	chr11:6992	NM_01804	XM_01701	.	.	NP_06051	XP_006718	protein_co	ENSG0000	55107	21625
ANO10	8.31655	6.39657	8.38856	8.08033	9.69563	7.89434	anoctamin 10 [Source:HGNC Symbol;Acc:HGNC:25519]	chr3:43407	NM_00120	XM_01700	.	.	NP_00119	XP_011532	protein_co	ENSG0000	55129	25519
ANO2	0	0.066238	0	0.019667	0	0	anoctamin 2 [Source:HGNC Symbol;Acc:HGNC:1183]	chr12:5671	NM_00127	XM_01152	.	.	NP_00126	XP_006719	protein_co	ENSG0000	57101	1183
ANO3	0	0	0	0	0	0	anoctamin 3 [Source:HGNC Symbol;Acc:HGNC:14004]	chr11:2633	NM_03141	XM_01152	.	.	NP_00130	XP_016873	protein_co	ENSG0000	63982	14004
ANO4	0.656748	0.679784	0.700403	0.929378	0.803333	0.950367	anoctamin 4 [Source:HGNC Symbol;Acc:HGNC:23837]	chr12:1011	NM_00128	XM_01701	.	.	NP_00127	XP_011536	protein_co	ENSG0000	121601	23837
ANO5	0.939688	0.646206	0.740081	0.657865	1.04515	0.754412	anoctamin 5 [Source:HGNC Symbol;Acc:HGNC:27337]	chr11:2221	NM_00114	XM_01151	.	.	NP_00113	XP_005252	protein_co	ENSG0000	203859	27337
ANO6	12.9217	14.2058	14.5045	12.4717	14.8923	18.952	anoctamin 6 [Source:HGNC Symbol;Acc:HGNC:25240]	chr12:4560	NM_00102	XM_01153	.	.	NP_00102	XP_005268	protein_co	ENSG0000	196527	25240
ANO7	0.1207	0.193608	0.197319	0.133417	0.122413	0.047883	anoctamin 7 [Source:HGNC Symbol;Acc:HGNC:31677]	chr2:24212	NM_00100	XM_01151	.	.	NP_00100	XP_011509	protein_co	ENSG0000	50636	31677
ANO8	6.24699	6.39888	5.97113	5.03149	4.89794	5.33488	anoctamin 8 [Source:HGNC Symbol;Acc:HGNC:29329]	chr19:1742	NM_02095	.	.	.	NP_06601	XP_016882	protein_co	ENSG0000	57719	29329
ANO9	5.68136	5.81895	10.0243	5.61453	5.74	5.46881	anoctamin 9 [Source:HGNC Symbol;Acc:HGNC:20679]	chr11:4182	NM_00101	XM_01152	.	.	NP_00101	XP_011518	protein_co	ENSG0000	338440	20679
ANOS1	0	0	0	0	0	0	anosmin 1 [Source:HGNC Symbol;Acc:HGNC:6211]	chrX:84969	NM_00021	.	.	.	NP_00020	.	protein_co	ENSG0000	3730	6211
ANP32A	172.07	152.787	124.932	187.432	176.657	162.981	acidic nuclear phosphoprotein 32 family member A [Source:HGN	chr15:6907	NM_00630	XM_00672	.	.	NP_00629	.	protein_co	ENSG0000	8125	13233
ANP32B	101.336	102.929	81.5769	120.687	116.956	107.416	acidic nuclear phosphoprotein 32 family member B [Source:HGN	chr9:10074	NM_00640	.	.	.	NP_00639	.	protein_co	ENSG0000	10541	16677
ANP32D	0.345417	0.356735	0.309867	0.163511	0.55427	0.509378	acidic nuclear phosphoprotein 32 family member D [Source:HGN	chr12:4886	NM_01240	.	.	.	NP_03653	.	protein_co	ENSG0000	23519	16676
ANP32E	48.2323	49.5851	35.9214	59.5685	69.8742	59.5385	acidic nuclear phosphoprotein 32 family member E [Source:HGN	chr1:15019	NM_00113	XM_00524	.	.	NP_00112	XP_005245	protein_co	ENSG0000	81611	16673
ANPEP	0.018382	0.073255	0.015934	0.019771	0.021291	0.060401	alanyl aminopeptidase, membrane [Source:HGNC Symbol;Acc:HG	chr15:9032	NM_00115	XM_01152	.	.	NP_00114	XP_005254	protein_co	ENSG0000	290	500
ANTXR1	12.1788	12.9238	13.4632	7.75435	9.33395	8.52472	anthrax toxin receptor 1 [Source:HGNC Symbol;Acc:HGNC:21014]	chr2:69240	NM_01815	XM_01153	.	.	NP_06062	XP_016860	protein_co	ENSG0000	84168	21014
ANTXR2	3.24924	2.0125	2.92111	5.07434	5.60491	6.44619	anthrax toxin receptor 2 [Source:HGNC Symbol;Acc:HGNC:21732]	chr4:80822	NM_00114	XM_01153	.	.	NP_00113	XP_011529	protein_co	ENSG0000	118429	21732
ANTXRL	0	0	0	0	0	0	anthrax toxin receptor-like [Source:HGNC Symbol;Acc:HGNC:272	chr10:4765	NM_00127	XM_01153	.	.	NP_00126	XP_006717	protein_co	ENSG0000	195977	27277
ANXA1	305.891	286.977	230.732	439.413	384.96	558.714	annexin A1 [Source:HGNC Symbol;Acc:HGNC:533]	chr9:75766	NM_00070	XM_01151	.	.	NP_00069	XP_011516	protein_co	ENSG0000	301	533
ANXA10	0.429568	0.225832	0.421228	1.86088	0.961927	0.484942	annexin A10 [Source:HGNC Symbol;Acc:HGNC:534]	chr4:16901	NM_00719	XM_01153	.	.	NP_00912	XP_011529	protein_co	ENSG0000	11199	534

ANXA11	45.77	46.4827	49.0879	55.4487	46.5017	42.8787	annexin A11 [Source:HGNC Symbol;Acc:HGNC:535]	chr10:8191	NM_00115	XM_01153	.	.	NP_00114	XP_00526	protein_co	ENSG0000	311	535
ANXA13	0	0	0	0	0	0	annexin A13 [Source:HGNC Symbol;Acc:HGNC:536]	chr8:12469	NM_00100	.	.	.	NP_00100	.	protein_co	ENSG0000	312	536
ANXA2	675.479	643.464	749.36	804.356	644.656	656.593	annexin A2 [Source:HGNC Symbol;Acc:HGNC:537]	chr15:6063	NM_00100	XM_01152	.	.	NP_00100	XP_01151	protein_co	ENSG0000	302	537
ANXA2R	1.74986	1.27012	0.490874	0.640254	0.864661	0.899965	annexin A2 receptor [Source:HGNC Symbol;Acc:HGNC:33463]	chr5:43039	NM_00101	.	.	.	NP_00101	.	protein_co	ENSG0000	389289	33463
ANXA3	50.5458	48.3582	60.8116	34.0065	31.2882	52.2426	annexin A3 [Source:HGNC Symbol;Acc:HGNC:541]	chr4:79472	NM_00513	XM_01153	.	.	NP_00513	.	protein_co	ENSG0000	306	541
ANXA4	27.23	27.7288	30.1398	27.9634	25.3801	25.8213	annexin A4 [Source:HGNC Symbol;Acc:HGNC:542]	chr2:69969	NM_00115	XM_01153	.	.	NP_00114	XP_011531	protein_co	ENSG0000	307	542
ANXA5	162.118	151.418	147.82	167.003	158.435	179.817	annexin A5 [Source:HGNC Symbol;Acc:HGNC:543]	chr4:12258	NM_00115	.	.	.	NP_00114	.	protein_co	ENSG0000	308	543
ANXA6	44.8153	44.6541	37.696	58.0568	48.2933	59.9042	annexin A6 [Source:HGNC Symbol;Acc:HGNC:544]	chr5:15048	NM_00115	XM_00526	.	.	NP_00114	XP_005268	protein_co	ENSG0000	309	544
ANXA7	51.5792	50.7055	43.6571	65.9097	53.8819	52.1705	annexin A7 [Source:HGNC Symbol;Acc:HGNC:545]	chr10:7513	NM_00115	XM_01701	.	.	NP_00114	XP_016871	protein_co	ENSG0000	310	545
ANXA8	0	0	0	0	0	0	annexin A8 [Source:HGNC Symbol;Acc:HGNC:546]	chr10:4825	NM_00104	XM_01154	.	.	NP_00103	XP_005276	protein_co	ENSG0000	107984085	546
ANXA8L1	0	0	0	0	0	0	annexin A8-like 1 [Source:HGNC Symbol;Acc:HGNC:23334]	chr10:4715	NM_00109	.	NR_03395	.	NP_00109	.	protein_co	ENSG0000	728113	23334
ANXA8L2	0	0	0	0	0	0	annexin A8-like 2 [Source:HGNC Symbol;Acc:23335]	chr10:4774	NM_00163	XM_00527	.	.	NP_00162	XP_005272	protein_co	ENSG0000	244	23335
ANXA9	1.26597	0.987106	1.81216	0.864395	1.02887	1.43293	annexin A9 [Source:HGNC Symbol;Acc:HGNC:547]	chr1:15095	NM_00356	XM_01151	.	.	NP_00355	XP_011508	protein_co	ENSG0000	8416	547
AOAH	0	0.032094	0.027061	0.104328	0.175673	0.063934	acyloxyacyl hydrolase [Source:HGNC Symbol;Acc:HGNC:548]	chr7:36552	NM_00117	XM_01151	.	.	NP_00117	XP_011513	protein_co	ENSG0000	313	548
AOC1	0.086943	0.031733	0.137546	1.0749	0.702558	0.671835	amine oxidase, copper containing 1 [Source:HGNC Symbol;Acc:H	chr7:15052	NM_00109	XM_01701	.	.	NP_00108	XP_011514	protein_co	ENSG0000	26	80
AOC2	4.45279	4.27652	3.70193	6.25751	6.74779	4.2947	amine oxidase, copper containing 2 [Source:HGNC Symbol;Acc:H	chr17:4099	NM_00115	.	.	.	NP_00114	.	protein_co	ENSG0000	314	549
AOC3	2.03422	2.15546	1.62819	3.88719	4.05696	2.734	amine oxidase, copper containing 3 [Source:HGNC Symbol;Acc:H	chr17:4100	NM_00127	.	.	.	NP_00126	.	protein_co	ENSG0000	8639	550
AOX1	0.164161	0.043034	0.047436	0.09188	0.092125	0.02764	aldehyde oxidase 1 [Source:HGNC Symbol;Acc:HGNC:553]	chr2:20145	NM_00115	XM_01151	.	.	NP_00115	XP_011509	protein_co	ENSG0000	316	553
AP000275.65	4.94E-06	0.488697	9.62E-06	1.27E-05	0.234545	2.74E-06	.	chr21:3394	.	.	.	.	.	.	protein_co	ENSG0000	.	.
AP000295.9	0.177914	0.147589	7.22E-07	2.87E-09	7.45E-12	0.4668	.	chr21:3460	.	.	.	.	.	.	protein_co	ENSG0000	.	.
AP000304.1	0	0	0	0	0	0	.	chr21:3487	.	.	.	.	.	.	protein_co	ENSG0000	.	.
AP000304.12	1.7E-163	1E-117	1.96E-59	3.6E-108	1.14E-52	6.18E-74	.	chr21:3487	.	.	.	.	.	.	protein_co	ENSG0000	.	.
AP000322.53	0	0	0	0	0	0	.	chr21:3579	.	.	.	.	.	.	protein_co	ENSG0000	.	.
AP000349.1	0	0	0	0	0	0	.	chr22:2411	.	.	.	.	.	.	protein_co	ENSG0000	.	.
AP000350.10	2.68E-06	6.84E-08	6.58E-13	0.520971	3.13E-05	0.000196	.	chr22:2419	.	.	.	.	.	.	protein_co	ENSG0000	.	.
AP000688.1	0	0	0	0	0	0	Uncharacterized protein [Source:UniProtKB/TrEMBL;Acc:M0QY5	chr21:3740	.	.	.	.	.	.	protein_co	ENSG0000	.	.
AP000695.1	0	0.055172	0	0	0.060198	0	.	chr21:3783	.	.	.	.	.	.	protein_co	ENSG0000	.	.
AP000721.4	0.26283	0.228008	2.3E-114	0.688348	1.56871	0.254591	.	chr11:6374	.	.	.	.	.	.	protein_co	ENSG0000	.	.
AP000758.1	0	3.89636	0	0	4.08069	0	Uncharacterized protein [Source:UniProtKB/TrEMBL;Acc:M0QZC	chr11:1202	.	.	.	.	.	.	protein_co	ENSG0000	.	.
AP000867.1	0	0.024524	0	0	0	0	.	chr11:7129	.	.	.	.	.	.	protein_co	ENSG0000	.	.
AP000889.3	0.383518	0.099394	0.24812	0.106453	0.108262	0.0976	HCG2032453; Uncharacterized protein; cDNA FLJ25337 fis, clone	chr11:1074	.	.	NR_02832	.	.	.	protein_co	ENSG0000	643923	.
AP000974.1	0.045828	0	0	0	0	0	CDNA FLJ26432 fis, clone KDN01418; Uncharacterized protein [S	chr11:8556	.	.	.	.	.	.	protein_co	ENSG0000	.	.
AP001024.1	0	0	0	0	0	0	Metazoan signal recognition particle RNA [Source:RFAM;Acc:RF0	chr11:1076	.	.	.	.	.	.	misc_RNA	ENSG0000	.	.
AP001024.2	0	0	0	0	0	0	Uncharacterized protein [Source:UniProtKB/TrEMBL;Acc:H0Y4W	chr11:1076	.	.	.	.	.	.	protein_co	ENSG0000	.	.
AP001055.1	0	0	0.137899	0	0	0	.	chr21:4558	.	.	.	.	.	.	protein_co	ENSG0000	.	.
AP001094.1	0	0	0	0	0	0	Uncharacterized protein [Source:UniProtKB/TrEMBL;Acc:M0R2D	chr18:7566	.	.	.	.	.	.	protein_co	ENSG0000	.	.
AP001362.1	0	0	0.134405	0	0	0	Uncharacterized protein [Source:UniProtKB/TrEMBL;Acc:M0QXL	chr11:6534	.	.	.	.	.	.	protein_co	ENSG0000	.	.
AP001421.1	0	0	0	0	0	0	Uncharacterized protein [Source:UniProtKB/TrEMBL;Acc:M0QXV	chr21:3873	.	.	.	.	.	.	protein_co	ENSG0000	.	.
AP001468.1	0.362144	1.04278	0.620148	0.630183	0.665819	0.379847	Protein LOC101060037 [Source:UniProtKB/TrEMBL;Acc:M0R212]	chr21:4760	.	XM_00526	.	.	.	XP_005261	protein_co	ENSG0000	101060037	.
AP001652.1	0	0	0	0	0	0	Uncharacterized protein; cDNA FLJ60524 [Source:UniProtKB/TrE	chr11:5867	.	.	.	.	.	.	protein_co	ENSG0000	.	.
AP001888.1	0	0	0	0	0	0.265335	Uncharacterized protein [Source:UniProtKB/TrEMBL;Acc:Q14569	chr11:6958	.	.	.	.	.	.	protein_co	ENSG0000	.	.
AP001925.1	0	0	0	0	0	0	.	chr11:1080	.	.	.	.	.	.	protein_co	ENSG0000	.	.
AP002353.1	0	0	0	0	0	0	.	chr11:1075	.	.	.	.	.	.	protein_co	ENSG0000	.	.
AP002956.1	0	0	0	0	0	0	.	chr11:1190	.	.	.	.	.	.	protein_co	ENSG0000	.	.
AP003041.2	0	0	0	0	0	0	Uncharacterized protein [Source:UniProtKB/TrEMBL;Acc:M0QYB	chr11:1299	.	.	.	.	.	.	protein_co	ENSG0000	.	.
AP003062.1	0	0	0	0	0	0	CDNA FLJ27342 fis, clone TST02993; Uncharacterized protein [So	chr11:1348	.	.	.	.	.	.	protein_co	ENSG0000	.	.
AP003419.11	0.001289	1.81326	0.006028	0.000202	0.001744	0.165707	.	chr11:6711	.	.	.	.	.	.	protein_co	ENSG0000	.	.
AP003733.1	0.363768	0.245145	0.315608	0.133291	0.068915	0.063196	Uncharacterized protein; cDNA FLJ36460 fis, clone THYMU20148	chr11:6173	.	.	.	.	.	.	protein_co	ENSG0000	.	.
AP1AR	20.8999	21.1199	21.4914	20.3462	21.13	31.6668	adaptor related protein complex 1 associated regulatory protein	chr4:11315	NM_00112	XM_00526	.	.	NP_00112	.	protein_co	ENSG0000	55435	28808
AP1B1	23.599	25.0887	26.0954	30.6713	27.352	26.2338	adaptor related protein complex 1 beta 1 subunit [Source:HGNC	chr22:2972	NM_00112	.	.	.	NP_00111	.	protein_co	ENSG0000	162	554
AP1G1	24.5191	23.4043	24.5345	22.2707	27.1973	26.1905	adaptor related protein complex 1 gamma 1 subunit [Source:HGN	chr16:7176	NM_00103	.	.	.	NP_00102	.	protein_co	ENSG0000	164	555
AP1G2	22.1118	18.8994	18.9674	17.3802	14.9126	11.8099	adaptor related protein complex 1 gamma 2 subunit [Source:HGN	chr14:2402	NM_00128	XM_01153	.	.	NP_00126	XP_005268	protein_co	ENSG0000	8906	556
AP1M1	19.1944	22.6337	20.0331	23.8711	18.4033	17.8087	adaptor related protein complex 1 mu 1 subunit [Source:HGNC S	chr19:1630	NM_00113	XM_01702	.	.	NP_00112	XP_016882	protein_co	ENSG0000	8907	13667
AP1M2	47.6257	45.2088	55.0549	68.5807	55.7925	53.463	adaptor related protein complex 1 mu 2 subunit [Source:HGNC S	chr19:1068	NM_00130	XM_01152	.	.	NP_00128	.	protein_co	ENSG0000	10053	558
AP1S1	84.1078	82.4859	81.3039	102.305	80.3243	81.3429	adaptor related protein complex 1 sigma 1 subunit [Source:HGN	chr7:10079	NM_00128	.	.	.	NP_00127	.	protein_co	ENSG0000	1174	559
AP1S2	5.82373	7.45758	6.5848	7.10142	5.28659	6.80888	adaptor related protein complex 1 sigma 2 subunit [Source:HGN	chrX:15843	NM_00127	XM_01702	.	.	NP_00125	XP_011543	protein_co	ENSG0000	8905	560
AP1S3	55.8437	65.3385	69.4935	62.479	74.3885	63.1035	adaptor related protein complex 1 sigma 3 subunit [Source:HGN	chr2:22461	NM_00103	.	.	.	NP_00103	XP_011508	protein_co	ENSG0000	130340	18971
AP2A1	36.2302	39.8288	38.2308	46.4129	41.1555	39.1643	adaptor related protein complex 2 alpha 1 subunit [Source:HGN	chr19:5027	NM_01420	XM_01152	.	.	NP_05501	XP_011524	protein_co	ENSG0000	160	561
AP2A2	20.3297	20.9357	20.9813	25.5407	21.2791	20.0854	adaptor related protein complex 2 alpha 2 subunit [Source:HGN	chr11:9258	NM_00124	XM_01151	.	.	NP_00122	XP_011518	protein_co	ENSG0000	161	562

AP2B1	91.5178	94.9823	85.1241	89.7318	93.2507	90.8909	adaptor related protein complex 2 beta 1 subunit [Source:HGNC S	chr17:3391	NM_00103	XM_01152	.	.	NP_00102	XP_005257	protein_co	ENSG0000	163	563
AP2M1	203.687	204.271	185.29	225.734	193.332	177.051	adaptor related protein complex 2 mu 1 subunit [Source:HGNC Sy	chr3:18389	NM_00102	.	.	.	NP_00102	.	protein_co	ENSG0000	1173	564
AP2S1	177.149	182.042	169.172	250.217	196.66	176.897	adaptor related protein complex 2 sigma 1 subunit [Source:HGNC	chr19:4734	NM_00130	XM_01152	.	.	NP_00128	XP_011524	protein_co	ENSG0000	1175	565
AP3B1	19.1957	17.7236	22.7463	16.8605	21.4619	22.8303	adaptor related protein complex 3 beta 1 subunit [Source:HGNC S	chr5:77296	NM_00127	XM_00524	.	.	NP_00125	XP_005248	protein_co	ENSG0000	8546	566
AP3B2	0.180203	0.078053	0.171713	0.115365	0.066556	0.086455	adaptor related protein complex 3 beta 2 subunit [Source:HGNC S	chr15:8332	NM_00127	XM_01152	.	.	NP_00126	.	protein_co	ENSG0000	8120	567
AP3D1	38.1474	41.9753	36.3072	41.3984	38.4826	34.904	adaptor related protein complex 3 delta 1 subunit [Source:HGNC	chr19:2100	NM_00126	XM_00672	.	.	NP_00124	XP_006722	protein_co	ENSG0000	8943	568
AP3M1	29.7832	29.6668	27.0822	21.5369	24.9893	24.763	adaptor related protein complex 3 mu 1 subunit [Source:HGNC Sy	chr10:7575	NM_00132	XM_01153	.	.	NP_00130	XP_006717	protein_co	ENSG0000	26985	569
AP3M2	15.1687	13.6263	14.9707	11.1767	13.353	10.8865	adaptor related protein complex 3 mu 2 subunit [Source:HGNC Sy	chr8:42010	NM_00113	XM_01701	.	.	NP_00112	XP_016868	protein_co	ENSG0000	10947	570
AP3S1	109.714	108.148	131.141	125.426	138.279	181.864	adaptor related protein complex 3 sigma 1 subunit [Source:HGNC	chr5:11516	NM_00128	XM_01154	.	.	NP_00127	.	protein_co	ENSG0000	1176	2013
AP3S2	11.3767	10.7938	12.4897	7.01649	8.88141	9.22964	adaptor related protein complex 3 sigma 2 subunit [Source:HGNC	chr15:9037	NM_00582	.	.	.	NP_00582	.	protein_co	ENSG0000	10239	571
AP4B1	7.83646	7.28887	7.51071	8.08953	6.82709	5.53828	adaptor related protein complex 4 beta 1 subunit [Source:HGNC S	chr1:11443	NM_00125	XM_01154	.	.	NP_00124	XP_011538	protein_co	ENSG0000	10717	572
AP4E1	3.51877	3.14688	3.28082	2.89874	4.12795	4.3485	adaptor related protein complex 4 epsilon 1 subunit [Source:HGN	chr15:5120	NM_00125	XM_01152	.	.	NP_00123	XP_005254	protein_co	ENSG0000	23431	573
AP4M1	13.4294	11.5031	10.4376	9.47429	7.84074	9.16859	adaptor related protein complex 4 mu 1 subunit [Source:HGNC Sy	chr7:99690	NM_00472	XM_01151	.	.	NP_00471	XP_005250	protein_co	ENSG0000	9179	574
AP4S1	3.25683	2.67471	2.60557	4.67117	4.60174	3.56525	adaptor related protein complex 4 sigma 1 subunit [Source:HGNC	chr14:3134	NM_00112	XM_01153	.	.	NP_00112	XP_005267	protein_co	ENSG0000	11154	575
AP5B1	8.85149	10.2076	9.51304	10.5262	11.0259	8.38897	adaptor related protein complex 5 beta 1 subunit [Source:HGNC S	chr11:6554	NM_13836	.	.	.	NP_61237	.	protein_co	ENSG0000	91056	25104
AP5M1	11.3523	10.3257	12.5913	9.30396	13.1122	13.5524	adaptor related protein complex 5 mu 1 subunit [Source:HGNC Sy	chr14:5766	NM_01822	XM_01153	.	.	NP_06069	XP_006720	protein_co	ENSG0000	55745	20192
AP5S1	5.31748	6.60969	4.20972	6.39167	5.26837	4.26818	adaptor related protein complex 5 sigma 1 subunit [Source:HGNC	chr20:3801	NM_00120	.	.	.	NP_00119	.	protein_co	ENSG0000	55317	15875
AP5Z1	8.67349	8.3357	7.59751	7.13762	5.08063	6.56589	adaptor related protein complex 5 zeta 1 subunit [Source:HGNC S	chr7:48152	NM_01485	.	.	.	NP_05567	.	protein_co	ENSG0000	9907	22197
APAF1	5.38317	6.03454	7.61137	7.08465	8.86648	7.74218	apoptotic peptidase activating factor 1 [Source:HGNC Symbol;Acc	chr12:9903	NM_00116	XM_01153	.	.	NP_00115	XP_016874	protein_co	ENSG0000	317	576
APBA1	0.158313	0.102884	0.234873	0.210437	0.116453	0.288074	amyloid beta precursor protein binding family A member 1 [Sourc	chr9:72042	NM_00116	XM_01701	.	.	NP_00115	XP_005252	protein_co	ENSG0000	320	578
APBA2	0.01836	0.040683	0.053816	0	0.064717	0.068645	amyloid beta precursor protein binding family A member 2 [Sourc	chr15:2912	NM_00113	XM_01702	.	.	NP_00112	XP_011519	protein_co	ENSG0000	321	579
APBA3	11.9919	11.0547	9.52876	9.72283	8.15231	8.24446	amyloid beta precursor protein binding family A member 3 [Sourc	chr19:3750	NM_00488	XM_00672	.	.	NP_00487	XP_006723	protein_co	ENSG0000	9546	580
APBB1	5.06651	5.95815	7.78773	7.96166	5.48667	5.78346	amyloid beta precursor protein binding family B member 1 [Sourc	chr11:6416	NM_00116	XM_01701	NR_04751	.	NP_00115	XP_006718	protein_co	ENSG0000	322	581
APBB1IP	0	0	0	0	0	0	amyloid beta precursor protein binding family B member 1 intera	chr10:2672	NM_01904	XM_01151	.	.	NP_06191	XP_006717	protein_co	ENSG0000	54518	17379
APBB2	13.7034	13.4324	15.5796	9.09712	10.6238	15.9315	amyloid beta precursor protein binding family B member 2 [Sourc	chr4:40812	NM_00116	XM_01700	.	.	NP_00115	XP_005248	protein_co	ENSG0000	323	582
APBB3	5.80886	5.3435	6.51328	2.86403	3.64013	5.31498	amyloid beta precursor protein binding family B member 3 [Sourc	chr5:13993	NM_00605	.	NR_10688	.	NP_00604	.	protein_co	ENSG0000	102465499	20708
APC	4.72506	4.65681	4.74714	4.31638	5.23489	4.57254	APC, WNT signaling pathway regulator [Source:HGNC Symbol;Acc	chr5:11207	NM_00003	.	.	.	NP_00002	.	protein_co	ENSG0000	324	583
APC2	0.212707	0.220464	0.297633	0.155616	0.241176	0.21099	APC2, WNT signaling pathway regulator [Source:HGNC Symbol;Ac	chr19:1450	NM_00588	XM_00672	.	.	NP_00587	XP_005259	protein_co	ENSG0000	10297	24036
APCDD1	0	0	0	0	0	0	APC down-regulated 1 [Source:HGNC Symbol;Acc:HGNC:15718]	chr18:1045	NM_15300	.	.	.	NP_69454	.	protein_co	ENSG0000	147495	15718
APCDD1L	0	0	0	0	0	0	APC down-regulated 1 like [Source:HGNC Symbol;Acc:HGNC:2689	chr20:5703	NM_15336	.	.	.	NP_00129	.	protein_co	ENSG0000	164284	26892
APCS	0	0	0	0	0	0	amyloid P component, serum [Source:HGNC Symbol;Acc:HGNC:5	chr1:15955	NM_00163	.	.	.	NP_00163	.	protein_co	ENSG0000	325	584
APEH	80.4332	79.3885	86.2306	104.8	86.6407	96.5611	acylaminoacyl-peptide hydrolase [Source:HGNC Symbol;Acc:HGN	chr3:49711	NM_00164	XM_01153	.	.	NP_00163	XP_005265	protein_co	ENSG0000	327	586
APELA	0	0	0	0	0	0	apelin receptor early endogenous ligand [Source:HGNC Symbol;A	chr4:16579	NM_00129	.	.	.	NP_00128	XP_016863	protein_co	ENSG0000	100506013	48925
APEX1	230.617	222.6	191.785	240.66	212.464	168.537	apurinic/apyrimidinic endodeoxyribonuclease 1 [Source:HGNC Sy	chr14:2092	NM_00124	XM_00526	.	.	NP_00123	XP_005267	protein_co	ENSG0000	328	587
APEX2	16.6142	18.2745	19.5702	19.7353	16.1928	14.7737	apurinic/apyrimidinic endodeoxyribonuclease 2 [Source:HGNC Sy	chrX:55026	NM_01448	.	.	.	NP_05529	.	protein_co	ENSG0000	27301	17889
APH1A	97.8181	97.5373	87.9365	94.7582	76.9449	78.8172	aph-1 homolog A, gamma-secretase subunit [Source:HGNC Symb	chr1:15023	NM_00107	XM_00671	.	.	NP_00107	.	protein_co	ENSG0000	51107	29509
APH1B	2.33995	3.31865	4.0932	2.14927	2.51455	3.71838	aph-1 homolog B, gamma-secretase subunit [Source:HGNC Symb	chr15:6356	NM_00114	.	.	.	NP_00113	.	protein_co	ENSG0000	83464	24080
API5	58.5385	61.7972	59.299	60.9159	70.4603	63.5619	apoptosis inhibitor 5 [Source:HGNC Symbol;Acc:HGNC:594]	chr11:4333	NM_00114	XM_01701	.	.	NP_00113	XP_006718	protein_co	ENSG0000	8539	594
APIP	21.4284	20.8814	22.478	17.717	19.4325	22.7187	APAF1 interacting protein [Source:HGNC Symbol;Acc:HGNC:1758	chr11:3490	NM_01595	.	.	.	NP_05704	.	protein_co	ENSG0000	51074	17581
APITD1	15.148	14.6384	8.93643	15.8743	15.8007	11.0329	apoptosis-inducing, TAF9-like domain 1 [Source:HGNC Symbol;Ac	chr1:10490	NM_19929	.	.	.	NP_95498	.	protein_co	ENSG0000	378708	23163
APITD1-CORT	5.59505	7.36781	4.79058	6.2808	4.91818	4.6665	APITD1-CORT readthrough [Source:HGNC Symbol;Acc:HGNC:3884	chr1:10490	NM_00124	.	.	.	NP_00123	.	protein_co	ENSG0000	100526739	38843
APLF	0.710821	0.701702	0.674239	0.316552	0.370978	0.534785	aprataxin and PNKP like factor [Source:HGNC Symbol;Acc:HGNC:2	chr2:68870	NM_13896	.	.	.	NP_62041	.	protein_co	ENSG0000	200558	28724
APLN	0.525528	0.448315	0.359412	0.302113	0.347563	0.088035	apelin [Source:HGNC Symbol;Acc:HGNC:16665]	chrX:12877	NM_01741	.	.	.	NP_05910	.	protein_co	ENSG0000	8862	16665
APLNR	0	0	0	0	0	0	apelin receptor [Source:HGNC Symbol;Acc:HGNC:339]	chr11:5700	NM_00516	.	.	.	NP_00515	.	protein_co	ENSG0000	187	339
APLP1	12.2231	12.8154	19.801	15.021	13.5893	13.7991	amyloid beta precursor like protein 1 [Source:HGNC Symbol;Acc:H	chr19:3635	NM_00102	.	.	.	NP_00101	.	protein_co	ENSG0000	333	597
APLP2	91.7586	97.171	104.424	113.34	109.262	130.036	amyloid beta precursor like protein 2 [Source:HGNC Symbol;Acc:H	chr11:1299	NM_00114	.	.	.	NP_00113	.	protein_co	ENSG0000	334	598
APMAP	65.2679	66.0127	59.4395	81.3058	75.1921	74.9353	adipocyte plasma membrane associated protein [Source:HGNC Sy	chr20:2494	NM_02053	XM_00526	.	.	NP_06539	XP_005260	protein_co	ENSG0000	57136	13238
APOA1	0.094426	0	0.27172	0	0.555458	0.236858	apolipoprotein A1 [Source:HGNC Symbol;Acc:HGNC:600]	chr11:1167	NM_00003	XM_00527	.	.	NP_00003	XP_005271	protein_co	ENSG0000	335	600
APOA2	0.237269	0	0.216807	0	0.283417	0	apolipoprotein A2 [Source:HGNC Symbol;Acc:HGNC:601]	chr1:16118	NM_00164	.	.	.	NP_00163	.	protein_co	ENSG0000	336	601
APOA4	0	0.041567	0	0	0	0	apolipoprotein A4 [Source:HGNC Symbol;Acc:HGNC:602]	chr11:1166	NM_00048	.	.	.	NP_00047	.	protein_co	ENSG0000	337	602
APOA5	0	0	0	0.044692	0.048357	0	apolipoprotein A5 [Source:HGNC Symbol;Acc:HGNC:17288]	chr11:1166	NM_00116	.	.	.	NP_00116	XP_016872	protein_co	ENSG0000	116519	17288
APOB	0	0	0	0	0	0	apolipoprotein B [Source:HGNC Symbol;Acc:HGNC:603]	chr2:21224	NM_00038	XM_01153	.	.	NP_00037	.	protein_co	ENSG0000	338	603
APOBEC1	0	0	0	0	0.112605	0	apolipoprotein B mRNA editing enzyme catalytic subunit 1 [Sourc	chr12:7801	NM_00130	.	.	.	NP_00129	.	protein_co	ENSG0000	339	604
APOBEC2	0	0	0	0	0	0	apolipoprotein B mRNA editing enzyme catalytic subunit 2 [Sourc	chr6:41021	NM_00678	.	.	.	NP_00678	.	protein_co	ENSG0000	10930	605
APOBEC3A	4.58E-27	2.65E-33	1.29E-33	1.25E-60	3.27E-57	7.73E-55	apolipoprotein B mRNA editing enzyme catalytic subunit 3A [Sour	chr22:3934	NM_00127	.	.	.	NP_00125	.	protein_co	ENSG0000	100913187	17343
APOBEC3B	24.7719	24.3227	10.8868	15.3696	11.3338	7.8952	apolipoprotein B mRNA editing enzyme catalytic subunit 3B [Sour	chr22:3934	NM_00127	.	.	.	NP_00125	.	protein_co	ENSG0000	9582	17352
APOBEC3C	35.3541	36.6831	45.1546	34.481	34.0891	26.4355	apolipoprotein B mRNA editing enzyme catalytic subunit 3C [Sour	chr22:3941	NM_01450	.	.	.	NP_05532	.	protein_co	ENSG0000	27350	17353
APOBEC3D	1.07064	0.940821	1.18641	1.46686	1.18537	0.993714	apolipoprotein B mRNA editing enzyme catalytic subunit 3D [Sour	chr22:3941	NM_15242	XM_00526	.	.	NP_68963	XP_005261	protein_co	ENSG0000	140564	17354
APOBEC3F	5.46082	5.70098	5.09968	2.88273	2.97014	4.7414	apolipoprotein B mRNA editing enzyme catalytic subunit 3F [Sour	chr22:3943	NM_00100	XM_01152	.	.	NP_00100	XP_011528	protein_co	ENSG0000	200316	17356

APOBEC3G	0.220612	0.037683	0.339385	0	0.083306	0.038389	apolipoprotein B mRNA editing enzyme catalytic subunit 3G [Sou	chr22:3943	NM_02182	XM_00672	.	.	NP_06859	XP_006724	protein_co	ENSG0000	60489	17357
APOBEC3H	2.55689	2.41655	4.15982	3.59869	2.29393	1.28182	apolipoprotein B mRNA editing enzyme catalytic subunit 3H [Sou	chr22:3949	NM_00116	XM_01152	.	.	NP_00115	XP_011528	protein_co	ENSG0000	164668	24100
APOBEC4	0	0	0	0	0	0	apolipoprotein B mRNA editing enzyme catalytic polypeptide like	chr1:18360	NM_20345	.	.	.	NP_98227	.	protein_co	ENSG0000	403314	32152
APOBR	0.042458	0.013807	0.050043	0.045178	0	0.079844	apolipoprotein B receptor [Source:HGNC Symbol;Acc:HGNC:2408	chr16:2848	NM_01869	.	.	.	NP_06116	.	protein_co	ENSG0000	55911	24087
APOC1	0.464229	0.149587	0.434833	0.405149	0	0.16861	apolipoprotein C1 [Source:HGNC Symbol;Acc:HGNC:607]	chr19:4541	NM_00132	XM_00525	.	.	NP_00130	XP_005258	protein_co	ENSG0000	341	607
APOC2	0	0	0	0	0	0	apolipoprotein C2 [Source:HGNC Symbol;Acc:HGNC:609]	chr19:4544	.	.	.	.	.	.	protein_co	ENSG0000	.	609
APOC3	0	0	0	0	0	0	apolipoprotein C3 [Source:HGNC Symbol;Acc:HGNC:610]	chr11:1167	NM_00004	.	.	.	NP_00003	.	protein_co	ENSG0000	345	610
APOC4	0	0	0	0	0	0	apolipoprotein C4 [Source:HGNC Symbol;Acc:HGNC:611]	chr19:4544	NM_00164	.	.	.	NP_00163	.	protein_co	ENSG0000	346	611
APOC4-APOC2	0	0	0	0	0	0	APOC4-APOC2 readthrough (NMD candidate) [Source:HGNC Sym	chr19:4544	NM_00048	.	.	.	NP_00047	.	protein_co	ENSG0000	344	44426
APOD	0.732939	0.962005	1.26862	0.955044	0.452447	0.826157	apolipoprotein D [Source:HGNC Symbol;Acc:HGNC:612]	chr3:19529	NM_00164	.	.	.	NP_00163	.	protein_co	ENSG0000	347	612
APOE	0.351802	0.914993	1.42382	1.7543	0.421011	1.82479	apolipoprotein E [Source:HGNC Symbol;Acc:HGNC:613]	chr19:4540	NM_00004	.	.	.	NP_00003	.	protein_co	ENSG0000	348	613
APOF	0	0	0	0	0	0	apolipoprotein F [Source:HGNC Symbol;Acc:HGNC:615]	chr12:5567	NM_00163	.	.	.	NP_00162	.	protein_co	ENSG0000	319	615
APOH	0	0	0	0	0	0	apolipoprotein H [Source:HGNC Symbol;Acc:HGNC:616]	chr17:6420	NM_00004	.	.	.	NP_00003	.	protein_co	ENSG0000	350	616
APOL1	0.210528	0.298731	0.215754	0.148238	0.204825	0.129024	apolipoprotein L1 [Source:HGNC Symbol;Acc:HGNC:618]	chr22:3664	NM_00113	XM_00526	.	.	NP_00113	XP_005261	protein_co	ENSG0000	8542	618
APOL2	8.14707	7.18046	5.20125	5.01404	4.70186	3.33409	apolipoprotein L2 [Source:HGNC Symbol;Acc:HGNC:619]	chr22:3662	NM_03088	XM_01702	.	.	NP_11209	XP_011528	protein_co	ENSG0000	23780	619
APOL3	0.375281	0.609887	0	0.361673	0.364332	0.201289	apolipoprotein L3 [Source:HGNC Symbol;Acc:HGNC:14868]	chr22:3653	NM_01434	XM_01702	.	.	NP_05516	XP_006724	protein_co	ENSG0000	80833	14868
APOL4	0	0	0	0	0	0.020976	apolipoprotein L4 [Source:HGNC Symbol;Acc:HGNC:14867]	chr22:3658	NM_03064	.	.	.	NP_08514	.	protein_co	ENSG0000	80832	14867
APOL5	0	0	0	0	0	0	apolipoprotein L5 [Source:HGNC Symbol;Acc:HGNC:14869]	chr22:3611	NM_03064	.	.	.	.	.	protein_co	ENSG0000	80831	14869
APOL6	0.751426	0.647582	0.218101	0.229588	0.322123	0.538753	apolipoprotein L6 [Source:HGNC Symbol;Acc:HGNC:14870]	chr22:3604	NM_03064	XM_01153	.	.	NP_08514	XP_011528	protein_co	ENSG0000	80830	14870
APOLD1	1.3607	1.18578	0.969901	1.60085	1.86932	3.4323	apolipoprotein L domain containing 1 [Source:HGNC Symbol;Acc:	chr12:1287	NM_00113	.	.	.	NP_00112	.	protein_co	ENSG0000	81575	25268
APOM	1.94794	2.32798	1.08184	0.916496	1.56295	1.39367	apolipoprotein M [Source:HGNC Symbol;Acc:HGNC:13916]	chr6:31606	NM_00125	XM_00671	.	.	NP_00124	XP_006715	protein_co	ENSG0000	55937	13916
APOO	13.9772	15.4919	14.5593	17.9537	17.8411	16.9824	apolipoprotein O [Source:HGNC Symbol;Acc:HGNC:28727]	chrX:23851	NM_02412	XM_01154	.	.	NP_07702	XP_011543	protein_co	ENSG0000	79135	28727
APOOL	7.09272	6.28648	6.62848	7.11683	6.6182	7.90091	apolipoprotein O like [Source:HGNC Symbol;Acc:HGNC:24009]	chrX:84258	NM_19845	XM_01153	.	.	NP_94085	XP_011529	protein_co	ENSG0000	139322	24009
APOPT1	13.896	10.2119	10.2839	12.3188	11.5114	12.1252	apoptogenic 1, mitochondrial [Source:HGNC Symbol;Acc:HGNC:2	chr14:1040	NM_00130	.	.	.	NP_00128	.	protein_co	ENSG0000	84334	20492
APP	96.6501	104.214	116.617	81.8078	80.3874	83.1144	amyloid beta precursor protein [Source:HGNC Symbol;Acc:HGNC	chr21:2725	NM_00048	.	.	.	NP_00047	.	protein_co	ENSG0000	351	620
APPBP2	9.79603	9.37974	12.7888	9.60703	12.8273	14.8355	amyloid beta precursor protein binding protein 2 [Source:HGNC S	chr17:5852	NM_00128	XM_01152	.	.	NP_00126	XP_011522	protein_co	ENSG0000	10513	622
APPL1	8.73013	9.39103	9.24476	8.41871	10.0735	10.7524	adaptor protein, phosphotyrosine interacting with PH domain an	chr3:57261	NM_01209	XM_01153	.	.	NP_03622	XP_011531	protein_co	ENSG0000	26060	24035
APPL2	9.6397	9.37567	11.5889	8.6808	9.29929	10.6383	adaptor protein, phosphotyrosine interacting with PH domain an	chr12:1055	NM_00125	XM_01153	.	.	NP_00123	XP_006719	protein_co	ENSG0000	55198	18242
APRT	228.539	220.874	196.532	247.353	185.978	193.957	adenine phosphoribosyltransferase [Source:HGNC Symbol;Acc:HG	chr16:8887	NM_00048	.	.	.	NP_00047	.	protein_co	ENSG0000	353	626
APTX	30.4268	25.0723	24.9525	35.9192	32.3217	27.162	aprataxin [Source:HGNC Symbol;Acc:HGNC:15984]	chr9:32972	NM_00119	XM_01701	.	.	NP_00118	XP_006716	protein_co	ENSG0000	54840	15984
AQP1	0.527495	0.758556	1.04375	1.18004	0.825562	0.899006	aquaporin 1 (Colton blood group) [Source:HGNC Symbol;Acc:HGN	chr7:30811	NM_00118	.	.	.	NP_00117	.	protein_co	ENSG0000	358	633
AQP10	0	0	0	0	0	0	aquaporin 10 [Source:HGNC Symbol;Acc:HGNC:16029]	chr1:15429	NM_08042	XM_01151	.	.	NP_53635	XP_011508	protein_co	ENSG0000	89872	16029
AQP11	0.2948	0.101766	0.29537	0.109685	0.219992	0.049572	aquaporin 11 [Source:HGNC Symbol;Acc:HGNC:19940]	chr11:7730	NM_17303	.	.	.	NP_76662	.	protein_co	ENSG0000	282679	19940
AQP12A	0	0	0	0	0	0	aquaporin 12A [Source:HGNC Symbol;Acc:HGNC:19941]	chr2:24163	NM_19899	.	.	.	NP_94534	.	protein_co	ENSG0000	375318	19941
AQP12B	0	0	0	0	0	0	aquaporin 12B [Source:HGNC Symbol;Acc:HGNC:6096]	chr2:24161	NM_00110	.	.	.	NP_00109	.	protein_co	ENSG0000	653437	6096
AQP2	0	0	0	0	0.021982	0	aquaporin 2 [Source:HGNC Symbol;Acc:HGNC:634]	chr12:5034	NM_00048	.	.	.	NP_00047	.	protein_co	ENSG0000	359	634
AQP3	3.13774	2.56038	4.72546	2.80522	2.60445	2.16872	aquaporin 3 (Gill blood group) [Source:HGNC Symbol;Acc:HGNC:6	chr9:33441	NM_00492	.	.	.	NP_00491	.	protein_co	ENSG0000	360	636
AQP4	0.111232	0	0.0443	0	0	0	aquaporin 4 [Source:HGNC Symbol;Acc:HGNC:637]	chr18:2443	NM_00131	XM_01152	.	.	NP_00130	XP_011524	protein_co	ENSG0000	361	637
AQP5	0.066108	0	0	0	0.231496	0.073541	aquaporin 5 [Source:HGNC Symbol;Acc:HGNC:638]	chr12:5035	NM_00165	.	.	.	NP_00164	.	protein_co	ENSG0000	362	638
AQP6	0.035888	0	0.031062	0.210982	0.15285	0.038421	aquaporin 6 [Source:HGNC Symbol;Acc:HGNC:639]	chr12:5036	NM_00165	.	.	.	NP_00164	.	protein_co	ENSG0000	363	639
AQP7	0.153781	0	0	0	0	0.163347	aquaporin 7 [Source:HGNC Symbol;Acc:HGNC:640]	chr9:33383	NM_00117	XM_01151	.	.	NP_00116	XP_005251	protein_co	ENSG0000	364	640
AQP8	0	0.136208	0	0	0	0	aquaporin 8 [Source:HGNC Symbol;Acc:HGNC:642]	chr16:2522	NM_00116	XM_01154	.	.	NP_00116	XP_011544	protein_co	ENSG0000	343	642
AQP9	0	0	0	0	0	0	aquaporin 9 [Source:HGNC Symbol;Acc:HGNC:643]	chr15:5824	NM_02098	.	.	.	NP_00130	.	protein_co	ENSG0000	366	643
AQR	5.47086	5.42742	5.79812	8.38064	10.2035	9.21966	aquarius intron-binding spliceosomal factor [Source:HGNC Symbo	chr15:3514	NM_01469	.	.	.	NP_05550	.	protein_co	ENSG0000	9716	29513
AR	0	0.046332	0	0	0	0	androgen receptor [Source:HGNC Symbol;Acc:HGNC:644]	chrX:66763	NM_00004	.	.	.	NP_00003	.	protein_co	ENSG0000	367	644
ARAF	18.3285	19.5067	17.4567	22.8433	18.9175	15.1039	A-Raf proto-oncogene, serine/threonine kinase [Source:HGNC Sy	chrX:47420	NM_00125	XM_01154	.	.	NP_00124	XP_006724	protein_co	ENSG0000	369	646
ARAP1	16.3357	17.691	18.325	16.5782	15.9381	14.3226	ArfGAP with RhoGAP domain, ankyrin repeat and PH domain 1 [S	chr11:7239	NM_00104	.	.	.	NP_00103	.	protein_co	ENSG0000	116985	16925
ARAP2	2.57733	2.52655	3.32637	1.26708	2.09952	2.58848	ArfGAP with RhoGAP domain, ankyrin repeat and PH domain 2 [S	chr4:36067	NM_01523	XM_01700	.	.	NP_05604	XP_016863	protein_co	ENSG0000	116984	16924
ARAP3	5.48605	5.10921	6.36991	6.19489	6.16933	4.62125	ArfGAP with RhoGAP domain, ankyrin repeat and PH domain 3 [S	chr5:14103	NM_02248	XM_01153	.	.	NP_07192	XP_005268	protein_co	ENSG0000	64411	24097
ARC	8.46952	9.2716	8.88456	8.62209	7.45015	8.51301	activity regulated cytoskeleton associated protein [Source:HGNC	chr8:14369	NM_01519	.	.	.	NP_05600	.	protein_co	ENSG0000	23237	648
ARCN1	96.788	97.0501	99.4672	89.1341	102.361	100.448	archain 1 [Source:HGNC Symbol;Acc:HGNC:649]	chr11:1184	NM_00114	XM_00527	.	.	NP_00113	XP_005271	protein_co	ENSG0000	372	649
AREG	20.6918	23.3855	24.3032	47.0465	42.5242	73.0945	amphiregulin [Source:HGNC Symbol;Acc:HGNC:651]	chr4:75310	NM_00165	.	.	.	NP_00164	.	protein_co	ENSG0000	374	651
AREGB	0	0	0	0	0	0.056495	amphiregulin B [Source:HGNC Symbol;Acc:34509]	chr4:75480	.	.	.	.	.	.	protein_co	ENSG0000	374	34509
AREL1	21.3655	23.3946	20.0705	20.5759	21.4613	18.2479	apoptosis resistant E3 ubiquitin protein ligase 1 [Source:HGNC Sy	chr14:7512	NM_00103	XM_01153	.	.	NP_00103	XP_005268	protein_co	ENSG0000	9870	20363
ARF1	291.255	293.332	252.28	345.144	303.885	279.198	ADP ribosylation factor 1 [Source:HGNC Symbol;Acc:HGNC:652]	chr1:22827	NM_00102	.	NR_03741	.	NP_00101	.	protein_co	ENSG0000	10050081	652
ARF3	79.9858	78.8496	86.3832	69.7414	66.7485	73.1978	ADP ribosylation factor 3 [Source:HGNC Symbol;Acc:HGNC:654]	chr12:4929	NM_00165	XM_01153	.	.	NP_00165	XP_005268	protein_co	ENSG0000	377	654
ARF4	134.791	131.307	126.095	141.105	129.423	137.049	ADP ribosylation factor 4 [Source:HGNC Symbol;Acc:HGNC:655]	chr3:57557	NM_00166	.	.	.	NP_00165	.	protein_co	ENSG0000	378	655
ARF5	203.338	188.602	225.707	145.506	117.452	157.771	ADP ribosylation factor 5 [Source:HGNC Symbol;Acc:HGNC:658]	chr7:12722	NM_00166	.	.	.	NP_00165	.	protein_co	ENSG0000	381	658
ARF6	53.0511	58.3438	46.8954	58.2645	61.7555	69.037	ADP ribosylation factor 6 [Source:HGNC Symbol;Acc:HGNC:659]	chr14:5035	NM_00166	.	.	.	NP_00165	.	protein_co	ENSG0000	382	659

ARFGAP1	26.6913	26.0558	30.5009	30.1492	27.0669	19.875	ADP ribosylation factor GTPase activating protein 1 [Source:HGN	chr20:6190	NM_00128	XM_01152	NR_10402	.	NP_00126	XP_006723	protein_co	ENSG0000	55738	15852
ARFGAP2	29.5397	29.9852	23.3547	33.1299	28.1894	20.0268	ADP ribosylation factor GTPase activating protein 2 [Source:HGN	chr11:4695	NM_00124	XM_01701	.	.	NP_00122	XP_005253	protein_co	ENSG0000	84364	13504
ARFGAP3	7.92615	7.17524	11.8434	7.81454	8.10817	9.96436	ADP ribosylation factor GTPase activating protein 3 [Source:HGN	chr22:4319	NM_00114	XM_00526	.	.	NP_00113	XP_005261	protein_co	ENSG0000	26286	661
ARFGEF1	5.80498	5.53845	6.10778	4.42492	6.20105	6.20321	ADP ribosylation factor guanine nucleotide exchange factor 1 [So	chr8:68109	NM_00642	XM_01151	.	.	NP_00641	XP_005251	protein_co	ENSG0000	10565	15772
ARFGEF2	9.17217	9.69127	8.68742	7.69279	8.75003	8.56072	ADP ribosylation factor guanine nucleotide exchange factor 2 [So	chr20:4753	NM_00642	XM_00526	.	.	NP_00641	XP_005260	protein_co	ENSG0000	10564	15853
ARFGEF3	1.87627	1.80694	1.98705	1.00899	1.24008	1.17745	ARFGEF family member 3 [Source:HGNC Symbol;Acc:HGNC:2121	chr6:13848	NM_02034	.	.	.	NP_06507	.	protein_co	ENSG0000	57221	21213
ARFIP1	15.6063	18.1262	17.1748	17.3858	19.7482	20.6598	ADP ribosylation factor interacting protein 1 [Source:HGNC Symb	chr4:15370	NM_00102	XM_01153	.	.	NP_00102	XP_011530	protein_co	ENSG0000	27236	21496
ARFIP2	45.2057	45.5737	53.3627	43.0231	37.6655	36.0982	ADP ribosylation factor interacting protein 2 [Source:HGNC Symb	chr11:6495	NM_00124	XM_00525	.	.	NP_00122	XP_005252	protein_co	ENSG0000	23647	17160
ARFRP1	25.922	25.8033	19.5695	15.8847	15.1204	11.2862	ADP ribosylation factor related protein 1 [Source:HGNC Symbol;A	chr20:6232	NM_00113	XM_01152	.	.	NP_00112	XP_011526	protein_co	ENSG0000	10139	662
ARG1	0	0	0	0	0	0	arginase 1 [Source:HGNC Symbol;Acc:HGNC:663]	chr6:13189	NM_00004	.	.	.	NP_00003	.	protein_co	ENSG0000	383	663
ARG2	6.56024	6.90543	4.58852	9.29056	8.42031	11.392	arginase 2 [Source:HGNC Symbol;Acc:HGNC:664]	chr14:6808	NM_00117	.	.	.	NP_00116	.	protein_co	ENSG0000	384	664
ARGFX	0	0	0	0	0	0	arginine-fifty homeobox [Source:HGNC Symbol;Acc:HGNC:30146]	chr3:12128	NM_00101	XM_00524	.	.	NP_00101	XP_005247	protein_co	ENSG0000	503582	30146
ARGLU1	37.0626	39.8953	37.8881	39.646	35.0207	34.7362	arginine and glutamate rich 1 [Source:HGNC Symbol;Acc:HGNC:2	chr13:1071	NM_01801	.	.	.	NP_06048	.	protein_co	ENSG0000	55082	25482
ARHGAP1	19.5183	19.7848	19.7877	18.2844	17.1576	13.1729	Rho GTPase activating protein 1 [Source:HGNC Symbol;Acc:HGN	chr11:4669	NM_00430	XM_01152	.	.	NP_00429	XP_011518	protein_co	ENSG0000	392	673
ARHGAP10	7.3729	8.02139	8.14801	10.2707	9.20534	8.50978	Rho GTPase activating protein 10 [Source:HGNC Symbol;Acc:HGN	chr4:14865	NM_02460	XM_01153	.	.	NP_07888	XP_005263	protein_co	ENSG0000	79658	26099
ARHGAP11A	26.0097	24.1758	27.1142	30.3039	45.4821	33.3513	Rho GTPase activating protein 11A [Source:HGNC Symbol;Acc:HG	chr15:3290	NM_00128	XM_01152	.	.	NP_00127	XP_011520	protein_co	ENSG0000	9824	15783
ARHGAP11B	3.13206	2.64426	3.58657	3.57149	3.85631	4.60588	Rho GTPase activating protein 11B [Source:HGNC Symbol;Acc:HG	chr15:3091	NM_00103	.	NR_03825	.	NP_00103	.	protein_co	ENSG0000	100288637	15782
ARHGAP12	29.3185	28.997	32.7692	27.8223	35.0342	37.5093	Rho GTPase activating protein 12 [Source:HGNC Symbol;Acc:HGN	chr10:3209	NM_00127	XM_01151	.	.	NP_00125	XP_005252	protein_co	ENSG0000	94134	16348
ARHGAP15	0	0	0	0	0	0	Rho GTPase activating protein 15 [Source:HGNC Symbol;Acc:HGN	chr2:14384	NM_01846	XM_01700	.	.	NP_06093	XP_011509	protein_co	ENSG0000	55843	21030
ARHGAP17	10.9912	11.8543	10.2424	11.8542	12.3827	10.0008	Rho GTPase activating protein 17 [Source:HGNC Symbol;Acc:HGN	chr16:2493	NM_00100	XM_01154	.	.	NP_00100	XP_011544	protein_co	ENSG0000	55114	18239
ARHGAP18	27.8902	28.6066	28.1831	38.3775	42.9225	48.7863	Rho GTPase activating protein 18 [Source:HGNC Symbol;Acc:HGN	chr6:12989	NM_03351	XM_00526	.	.	NP_27705	XP_005267	protein_co	ENSG0000	93663	21035
ARHGAP19	11.7621	11.9275	13.0524	15.171	16.1567	18.5761	Rho GTPase activating protein 19 [Source:HGNC Symbol;Acc:HGN	chr10:9898	NM_00120	.	.	.	NP_00119	.	protein_co	ENSG0000	84986	23724
ARHGAP20	0	0	0.011174	0	0	0	Rho GTPase activating protein 20 [Source:HGNC Symbol;Acc:HGN	chr11:1104	NM_00125	XM_00671	.	.	NP_00124	XP_005271	protein_co	ENSG0000	57569	18357
ARHGAP21	9.63759	9.1559	9.26545	9.09624	10.04	10.1678	Rho GTPase activating protein 21 [Source:HGNC Symbol;Acc:HGN	chr10:2487	NM_02082	XM_01701	.	.	NP_06587	XP_005252	protein_co	ENSG0000	57584	23725
ARHGAP22	5.05066	4.08928	4.48774	5.47302	4.82404	3.28039	Rho GTPase activating protein 22 [Source:HGNC Symbol;Acc:HGN	chr10:4965	NM_00125	XM_01154	.	.	NP_00124	XP_005270	protein_co	ENSG0000	58504	30320
ARHGAP23	7.32229	7.51268	8.50251	7.16258	7.21644	6.04203	Rho GTPase activating protein 23 [Source:HGNC Symbol;Acc:HGN	chr17:3658	NM_00119	XM_01152	.	.	NP_00118	XP_006722	protein_co	ENSG0000	57636	29293
ARHGAP24	0.513735	0.65507	0.979781	0.21466	0.240873	0.475434	Rho GTPase activating protein 24 [Source:HGNC Symbol;Acc:HGN	chr4:86396	NM_00102	XM_01153	.	.	NP_00102	XP_005263	protein_co	ENSG0000	83478	25361
ARHGAP25	0	0	0	0	0	0.030463	Rho GTPase activating protein 25 [Source:HGNC Symbol;Acc:HGN	chr2:68961	NM_00100	XM_01153	.	.	NP_00100	XP_005264	protein_co	ENSG0000	9938	28951
ARHGAP26	5.39377	5.49741	6.38571	6.70813	7.61009	7.87735	Rho GTPase activating protein 26 [Source:HGNC Symbol;Acc:HGN	chr5:14214	NM_00113	XM_00526	.	.	NP_00112	XP_005268	protein_co	ENSG0000	23092	17073
ARHGAP27	17.7478	17.7159	17.9812	18.8833	18.9434	16.0059	Rho GTPase activating protein 27 [Source:HGNC Symbol;Acc:HGN	chr17:4347	NM_00128	XM_01152	.	.	NP_00126	XP_005257	protein_co	ENSG0000	201176	31813
ARHGAP28	0	0	0	0	0	0	Rho GTPase activating protein 28 [Source:HGNC Symbol;Acc:HGN	chr18:6729	NM_00101	XM_00525	.	.	NP_00101	XP_005258	protein_co	ENSG0000	79822	25509
ARHGAP29	51.822	50.3825	73.473	40.0733	52.4863	55.8551	Rho GTPase activating protein 29 [Source:HGNC Symbol;Acc:HGN	chr1:94637	NM_00481	XM_01154	.	.	NP_00131	XP_011540	protein_co	ENSG0000	9411	30207
ARHGAP30	0.014504	0.01988	0.102019	0.085007	0.100055	0.10959	Rho GTPase activating protein 30 [Source:HGNC Symbol;Acc:HGN	chr1:16101	NM_00102	XM_01150	.	.	NP_00102	XP_005245	protein_co	ENSG0000	257106	27414
ARHGAP31	0.69301	0.881671	0.735808	0.561097	0.552709	0.759907	Rho GTPase activating protein 31 [Source:HGNC Symbol;Acc:HGN	chr3:11901	NM_02075	XM_00671	.	.	NP_06580	XP_006713	protein_co	ENSG0000	57514	29216
ARHGAP32	4.81776	5.29258	5.19015	4.43902	5.62085	6.73654	Rho GTPase activating protein 32 [Source:HGNC Symbol;Acc:HGN	chr11:1288	NM_00114	XM_01701	.	.	NP_00113	XP_005271	protein_co	ENSG0000	9743	17399
ARHGAP33	2.60734	2.94336	3.22677	3.92025	3.41109	2.98446	Rho GTPase activating protein 33 [Source:HGNC Symbol;Acc:HGN	chr19:3626	NM_00117	XM_01152	.	.	NP_00116	XP_005258	protein_co	ENSG0000	115703	23085
ARHGAP35	14.2569	15.185	14.969	15.294	15.7065	16.9282	Rho GTPase activating protein 35 [Source:HGNC Symbol;Acc:HGN	chr19:4742	NM_00449	XM_01702	.	.	NP_00448	XP_011525	protein_co	ENSG0000	2909	4591
ARHGAP36	0	0	0	0	0	0	Rho GTPase activating protein 36 [Source:HGNC Symbol;Acc:HGN	chrX:13019	NM_00128	XM_01153	.	.	NP_00126	XP_006724	protein_co	ENSG0000	158763	26388
ARHGAP39	8.56654	9.75524	9.79176	9.46788	8.10377	7.88328	Rho GTPase activating protein 39 [Source:HGNC Symbol;Acc:HGN	chr8:14575	NM_00130	XM_01701	.	.	NP_00129	XP_005272	protein_co	ENSG0000	80728	29351
ARHGAP4	17.1584	17.0765	21.0301	20.9844	18.5079	12.822	Rho GTPase activating protein 4 [Source:HGNC Symbol;Acc:HGN	chrX:15317	NM_00116	.	.	.	NP_00115	.	protein_co	ENSG0000	393	674
ARHGAP40	1.20999	1.00195	1.27625	0.87547	0.707121	1.68797	Rho GTPase activating protein 40 [Source:HGNC Symbol;Acc:HGN	chr20:3723	NM_00116	.	.	.	NP_00115	.	protein_co	ENSG0000	343578	16226
ARHGAP42	2.48604	2.46394	3.00312	2.35348	2.88264	3.41897	Rho GTPase activating protein 42 [Source:HGNC Symbol;Acc:HGN	chr11:1005	NM_15243	XM_01154	.	.	NP_68964	XP_011540	protein_co	ENSG0000	143872	26545
ARHGAP44	3.01997	2.89142	3.04519	3.25835	2.85963	3.34722	Rho GTPase activating protein 44 [Source:HGNC Symbol;Acc:HGN	chr17:1269	NM_00132	XM_00672	.	.	NP_00130	XP_005256	protein_co	ENSG0000	9912	29096
ARHGAP45	7.30443	7.74204	8.52763	7.10347	5.85761	5.83218	Rho GTPase activating protein 45 [Source:HGNC Symbol;Acc:HGN	chr19:1065	NM_00125	XM_01152	.	.	NP_00124	XP_006722	protein_co	ENSG0000	23526	17102
ARHGAP5	11.738	13.7049	12.9026	12.8535	16.7109	19.0984	Rho GTPase activating protein 5 [Source:HGNC Symbol;Acc:HGN	chr14:3254	NM_00103	XM_00526	.	.	NP_00102	XP_005267	protein_co	ENSG0000	394	675
ARHGAP6	0	0	0	0	0	0.021441	Rho GTPase activating protein 6 [Source:HGNC Symbol;Acc:HGN	chrX:11155	NM_00128	.	.	.	NP_00127	.	protein_co	ENSG0000	395	676
ARHGAP8	18.7709	18.2125	23.4526	23.2486	19.8666	19.1444	Rho GTPase activating protein 8 [Source:HGNC Symbol;Acc:HGN	chr22:4506	NM_00101	.	.	.	NP_00101	.	protein_co	ENSG0000	23779	677
ARHGAP9	0.21296	0.07063	0.030664	0.192104	0	0.077454	Rho GTPase activating protein 9 [Source:HGNC Symbol;Acc:HGN	chr12:5785	NM_00108	XM_01153	.	.	NP_00107	XP_005269	protein_co	ENSG0000	64333	14130
ARHGDIA	213.689	228.605	188.02	305.822	253.169	185.979	Rho GDP dissociation inhibitor alpha [Source:HGNC Symbol;Acc:H	chr17:7982	NM_00118	XM_01152	.	.	NP_00117	XP_011521	protein_co	ENSG0000	396	678
ARHGDIB	0.830887	0.74408	0.663112	1.95838	1.73557	0.884903	Rho GDP dissociation inhibitor beta [Source:HGNC Symbol;Acc:H	chr12:1509	NM_00117	XM_01152	.	.	NP_00116	XP_011518	protein_co	ENSG0000	397	679
ARHGDIG	0	0	0	0	0	0	Rho GDP dissociation inhibitor gamma [Source:HGNC Symbol;Acc	chr16:3306	NM_00117	.	.	.	NP_00116	.	protein_co	ENSG0000	398	680
ARHGEF1	27.3812	26.3941	29.1971	29.8242	25.5503	24.8412	Rho guanine nucleotide exchange factor 1 [Source:HGNC Symbol;	chr19:4238	NM_00470	XM_01152	NR_13562	.	NP_00469	XP_005259	protein_co	ENSG0000	100505585	681
ARHGEF10	12.9433	13.3301	17.857	11.6286	12.8248	13.9739	Rho guanine nucleotide exchange factor 10 [Source:HGNC Symbo	chr8:17721	NM_00130	XM_01701	.	.	NP_00129	XP_005266	protein_co	ENSG0000	9639	14103
ARHGEF10L	5.04299	5.01653	4.75619	5.31102	4.4335	4.52068	Rho guanine nucleotide exchange factor 10 like [Source:HGNC Sy	chr1:17866	NM_00101	XM_01700	.	.	NP_00101	XP_005245	protein_co	ENSG0000	55160	25540
ARHGEF11	7.76231	8.30671	7.33889	6.89216	7.27042	7.01095	Rho guanine nucleotide exchange factor 11 [Source:HGNC Symbo	chr1:15690	NM_01478	XM_01700	.	.	NP_05559	XP_005245	protein_co	ENSG0000	9826	14580
ARHGEF12	25.4595	28.4389	26.4924	29.2372	33.266	38.1639	Rho guanine nucleotide exchange factor 12 [Source:HGNC Symbo	chr11:1202	NM_00119	XM_01701	.	.	NP_00118	XP_006718	protein_co	ENSG0000	23365	14193
ARHGEF15	0	0	0	0.019537	0	0	Rho guanine nucleotide exchange factor 15 [Source:HGNC Symbo	chr17:8213	NM_02501	XM_01152	.	.	NP_07929	XP_011522	protein_co	ENSG0000	22899	15590
ARHGEF16	12.6332	13.2559	14.8333	14.0203	10.4826	11.7092	Rho guanine nucleotide exchange factor 16 [Source:HGNC Symbo	chr1:33709	NM_01444	XM_01700	.	.	NP_05526	XP_016856	protein_co	ENSG0000	27237	15515
ARHGEF17	3.7221	3.96036	4.6237	3.41923	3.27828	4.09723	Rho guanine nucleotide exchange factor 17 [Source:HGNC Symbo	chr11:7301	NM_01478	XM_01154	.	.	NP_05560	.	protein_co	ENSG0000	9828	21726

ARHGEF18	9.18692	9.1573	9.11428	9.73171	9.70095	8.85674	Rho/Rac guanine nucleotide exchange factor 18 [Source:HGNC Sy	chr19:7413	NM_00113	XM_01152	.	.	NP_00112	XP_006722	protein_co	ENSG0000	23370	17090
ARHGEF19	23.3787	22.4709	22.36	26.8182	24.036	17.3847	Rho guanine nucleotide exchange factor 19 [Source:HGNC Symbo	chr1:16524	NM_15321	XM_01154	.	.	NP_69494	XP_011539	protein_co	ENSG0000	128272	26604
ARHGEF2	16.7498	16.9301	12.949	14.7486	14.7096	25.9892	Rho/Rac guanine nucleotide exchange factor 2 [Source:HGNC Syn	chr1:15591	NM_00116	XM_01700	.	.	NP_00115	XP_005245	protein_co	ENSG0000	9181	682
ARHGEF25	1.24786	1.02355	1.5932	1.19822	1.33012	1.38831	Rho guanine nucleotide exchange factor 25 [Source:HGNC Symbo	chr12:5800	NM_00111	.	.	.	NP_00110	.	protein_co	ENSG0000	115557	30275
ARHGEF26	2.60033	2.9117	4.06121	4.30746	3.99272	4.34933	Rho guanine nucleotide exchange factor 26 [Source:HGNC Symbo	chr3:15383	NM_00125	XM_01151	.	.	NP_00123	XP_011510	protein_co	ENSG0000	26084	24490
ARHGEF28	1.60519	1.89754	2.12082	1.51753	1.78966	2.39427	Rho guanine nucleotide exchange factor 28 [Source:HGNC Symbo	chr5:72921	NM_00108	XM_01154	.	.	NP_00107	XP_005248	protein_co	ENSG0000	64283	30322
ARHGEF3	8.64279	8.77048	9.94431	10.3426	10.0222	10.9702	Rho guanine nucleotide exchange factor 3 [Source:HGNC Symbol;	chr3:56761	NM_00112	XM_01153	.	.	NP_00112	XP_005265	protein_co	ENSG0000	50650	683
ARHGEF33	0	0.018337	0.030498	0.019613	0.03917	0.052944	Rho guanine nucleotide exchange factor 33 [Source:HGNC Symbo	chr2:39117	NM_00114	.	.	.	NP_00113	.	protein_co	ENSG0000	100271715	37252
ARHGEF35	2.90614	2.86004	2.58441	2.02575	2.48999	2.92783	Rho guanine nucleotide exchange factor 35 [Source:HGNC Symbo	chr7:14388	NM_00100	XM_01703	.	.	NP_00100	XP_016885	protein_co	ENSG0000	445328	33846
ARHGEF37	2.05597	2.208	2.9655	2.63867	3.26724	2.79982	Rho guanine nucleotide exchange factor 37 [Source:HGNC Symbo	chr5:14896	NM_00100	XM_01153	.	.	NP_00100	XP_005268	protein_co	ENSG0000	389337	34430
ARHGEF38	0	0	0	0.051623	0	0	Rho guanine nucleotide exchange factor 38 [Source:HGNC Symbo	chr4:10647	NM_00124	XM_01153	.	.	NP_00122	XP_011530	protein_co	ENSG0000	54848	25968
ARHGEF39	7.54094	7.32709	7.74126	10.32	8.32826	6.72328	Rho guanine nucleotide exchange factor 39 [Source:HGNC Symbo	chr9:35658	NM_03281	XM_01151	.	.	NP_11620	XP_011516	protein_co	ENSG0000	84904	25909
ARHGEF4	11.1706	10.5117	11.1495	9.25142	8.01661	9.25416	Rho guanine nucleotide exchange factor 4 [Source:HGNC Symbol;	chr2:13159	NM_01532	XM_01151	.	.	NP_05613	XP_005263	protein_co	ENSG0000	50649	684
ARHGEF40	2.89277	2.62416	2.86547	1.8646	1.8292	1.868	Rho guanine nucleotide exchange factor 40 [Source:HGNC Symbo	chr14:2148	NM_00127	XM_01153	.	.	NP_00126	XP_005267	protein_co	ENSG0000	55701	25516
ARHGEF5	13.9189	13.2707	10.5612	10.1516	10.3111	8.96967	Rho guanine nucleotide exchange factor 5 [Source:HGNC Symbol;	chr7:14405	NM_00543	.	.	.	NP_00542	XP_006726	protein_co	ENSG0000	102725117	13209
ARHGEF6	1.31936	0.946424	1.07116	0.860947	0.800328	0.993538	Rac/Cdc42 guanine nucleotide exchange factor 6 [Source:HGNC S	chrX:13574	NM_00130	XM_01153	.	.	NP_00129	XP_005262	protein_co	ENSG0000	9459	685
ARHGEF7	15.0524	15.1423	15.1179	13.6474	15.5827	13.726	Rho guanine nucleotide exchange factor 7 [Source:HGNC Symbol;	chr13:1117	NM_00111	XM_01702	.	.	NP_00110	XP_005254	protein_co	ENSG0000	8874	15607
ARHGEF9	4.04114	4.84169	4.96602	3.71357	3.9897	4.31811	Cdc42 guanine nucleotide exchange factor 9 [Source:HGNC Symb	chrX:62854	NM_00117	XM_01702	.	.	NP_00116	XP_005262	protein_co	ENSG0000	23229	14561
ARID1A	15.5158	16.3967	13.5709	15.1301	15.5458	18.6324	AT-rich interaction domain 1A [Source:HGNC Symbol;Acc:HGNC:1	chr1:27019	NM_00601	.	.	.	NP_00600	.	protein_co	ENSG0000	8289	11110
ARID1B	7.77824	8.0763	6.60773	6.38781	6.75426	6.83963	AT-rich interaction domain 1B [Source:HGNC Symbol;Acc:HGNC:1	chr6:15709	NM_01751	XM_01153	.	.	NP_05998	XP_005267	protein_co	ENSG0000	57492	18040
ARID2	9.95367	10.1612	8.50134	8.21913	9.61107	9.01103	AT-rich interaction domain 2 [Source:HGNC Symbol;Acc:HGNC:18	chr12:4612	NM_15264	XM_00671	.	.	NP_68985	XP_006719	protein_co	ENSG0000	196528	18037
ARID3A	5.04692	5.39922	5.79872	3.86321	3.75549	3.68086	AT-rich interaction domain 3A [Source:HGNC Symbol;Acc:HGNC:3	chr19:9259	NM_00522	XM_01702	.	.	NP_00521	XP_005259	protein_co	ENSG0000	1820	3031
ARID3B	2.15542	2.37109	2.53978	2.17529	2.46864	1.887	AT-rich interaction domain 3B [Source:HGNC Symbol;Acc:HGNC:1	chr15:7483	NM_00130	XM_00525	.	.	NP_00129	XP_005254	protein_co	ENSG0000	10620	14350
ARID3C	0.481558	0.29357	0.467264	0.575108	0	0.203011	AT-rich interaction domain 3C [Source:HGNC Symbol;Acc:HGNC:2	chr9:34621	NM_00101	.	.	.	NP_00101	.	protein_co	ENSG0000	138715	21209
ARID4A	2.63113	2.52942	3.0089	1.25047	1.70281	2.03686	AT-rich interaction domain 4A [Source:HGNC Symbol;Acc:HGNC:9	chr14:5876	NM_00289	XM_01702	.	.	NP_00288	XP_005268	protein_co	ENSG0000	5926	9885
ARID4B	6.57674	7.24231	6.78567	6.21868	7.77719	7.53942	AT-rich interaction domain 4B [Source:HGNC Symbol;Acc:HGNC:1	chr1:23533	NM_00120	XM_01700	.	.	NP_00119	XP_006711	protein_co	ENSG0000	51742	15550
ARID5A	6.4841	6.2599	5.74287	7.98744	7.20416	5.16011	AT-rich interaction domain 5A [Source:HGNC Symbol;Acc:HGNC:1	chr2:97202	NM_00131	XM_01151	.	.	NP_00130	XP_011508	protein_co	ENSG0000	10865	17361
ARID5B	2.89958	2.97759	3.50947	4.78187	5.55271	7.02426	AT-rich interaction domain 5B [Source:HGNC Symbol;Acc:HGNC:1	chr10:6366	NM_00124	XM_01154	.	.	NP_00123	XP_011538	protein_co	ENSG0000	84159	17362
ARIH1	7.01671	6.99489	7.74209	6.88964	8.48363	9.56779	ariadne RBR E3 ubiquitin protein ligase 1 [Source:HGNC Symbol;A	chr15:7276	NM_00574	XM_01152	NR_03035	.	NP_00573	XP_011519	protein_co	ENSG0000	693215	689
ARIH2	27.8522	27.3117	27.8656	26.307	25.942	28.5918	ariadne RBR E3 ubiquitin protein ligase 2 [Source:HGNC Symbol;A	chr3:48955	NM_00632	XM_01153	.	.	NP_00130	XP_005264	protein_co	ENSG0000	10425	690
ARIH2OS	1.75697	1.21406	0.186633	0.472705	0.513331	0.881041	ariadne homolog 2 opposite strand [Source:HGNC Symbol;Acc:HG	chr3:48955	NM_00112	.	.	.	NP_00111	.	protein_co	ENSG0000	646450	34425
ARL1	37.2594	33.8105	33.3064	41.8535	36.1474	35.8176	ADP ribosylation factor like GTPase 1 [Source:HGNC Symbol;Acc:H	chr12:1017	NM_00117	.	.	.	NP_00116	.	protein_co	ENSG0000	400	692
ARL10	0.00762	0.016012	0.032943	0.017107	0.008524	0.030615	ADP ribosylation factor like GTPase 10 [Source:HGNC Symbol;Acc	chr5:17579	NM_17366	XM_01153	.	.	NP_77593	XP_011532	protein_co	ENSG0000	285598	22042
ARL11	0	0	0	0.02138	0	0	ADP ribosylation factor like GTPase 11 [Source:HGNC Symbol;Acc	chr13:5020	NM_13845	XM_00526	.	.	NP_61245	XP_005266	protein_co	ENSG0000	115761	24046
ARL13A	0	0	0	0	0	0	ADP ribosylation factor like GTPase 13A [Source:HGNC Symbol;Ac	chrX:10022	NM_00116	XM_01153	.	.	NP_00115	XP_011529	protein_co	ENSG0000	392509	31709
ARL13B	3.90501	4.5013	3.45105	3.53661	4.68826	4.9131	ADP ribosylation factor like GTPase 13B [Source:HGNC Symbol;Ac	chr3:93698	NM_00117	XM_01151	.	.	NP_00116	XP_006713	protein_co	ENSG0000	200894	25419
ARL14	0.938309	1.47912	0.336923	0.632185	0.243996	0.53518	ADP ribosylation factor like GTPase 14 [Source:HGNC Symbol;Acc	chr3:16039	NM_02504	.	.	.	NP_07932	.	protein_co	ENSG0000	80117	22974
ARL14EP	15.0801	15.5773	13.3987	13.7715	14.4246	14.8222	ADP ribosylation factor like GTPase 14 effector protein [Source:H	chr11:3034	NM_15231	.	.	.	NP_68952	.	protein_co	ENSG0000	120534	26798
ARL14EPL	5.14991	5.74517	6.10952	4.08399	4.7232	5.37295	ADP ribosylation factor like GTPase 14 effector protein like [Sourc	chr5:11529	NM_00119	XM_01154	.	.	NP_00118	XP_011541	protein_co	ENSG0000	644100	44201
ARL15	8.56971	6.84261	11.6426	6.63784	7.81845	11.8327	ADP ribosylation factor like GTPase 15 [Source:HGNC Symbol;Acc	chr5:53179	NM_01908	XM_01154	.	.	NP_06196	XP_011541	protein_co	ENSG0000	54622	25945
ARL16	42.8447	40.288	37.9942	40.9839	38.9807	27.1565	ADP ribosylation factor like GTPase 16 [Source:HGNC Symbol;Acc	chr17:7964	NM_00104	.	.	.	NP_00103	.	protein_co	ENSG0000	339231	27902
ARL17A	0.856277	0.125269	0.679776	0.427395	0.374489	0.653405	ADP ribosylation factor like GTPase 17A [Source:HGNC Symbol;Ac	chr17:4458	NM_00111	XM_01152	.	.	NP_00110	XP_005257	protein_co	ENSG0000	100506084	24096
ARL17B	0.861422	1.10009	0.943764	0.99779	0.7991	0.575073	ADP ribosylation factor like GTPase 17B [Source:HGNC Symbol;Ac	chr17:4435	NM_00103	XM_01152	.	.	NP_00103	XP_005256	protein_co	ENSG0000	107984156	32387
ARL2	51.2547	49.6476	42.8362	71.3313	53.2862	47.0827	ADP ribosylation factor like GTPase 2 [Source:HGNC Symbol;Acc:H	chr11:6478	NM_00119	.	.	.	NP_00118	.	protein_co	ENSG0000	402	693
ARL2BP	27.7627	29.297	24.8794	24.7693	25.2053	23.8618	ADP ribosylation factor like GTPase 2 binding protein [Source:HG	chr16:5727	NM_01210	.	.	.	NP_03623	.	protein_co	ENSG0000	23568	17146
ARL3	11.8229	9.913	11.3014	14.7525	12.4987	12.2727	ADP ribosylation factor like GTPase 3 [Source:HGNC Symbol;Acc:H	chr10:1044	NM_00431	XM_01701	.	.	NP_00430	XP_016871	protein_co	ENSG0000	403	694
ARL4A	26.1534	23.0636	24.6379	34.9045	34.8443	44.8233	ADP ribosylation factor like GTPase 4A [Source:HGNC Symbol;Acc	chr7:12726	NM_00103	.	.	.	NP_00103	.	protein_co	ENSG0000	10124	695
ARL4C	3.53688	3.82388	3.47497	4.31677	4.75357	4.75581	ADP ribosylation factor like GTPase 4C [Source:HGNC Symbol;Acc	chr2:23540	NM_00128	.	.	.	NP_00126	.	protein_co	ENSG0000	10123	698
ARL4D	10.1897	9.66553	11.1062	14.5267	12.6351	11.1898	ADP ribosylation factor like GTPase 4D [Source:HGNC Symbol;Acc	chr17:4147	NM_00166	XM_01152	.	.	NP_00165	XP_011523	protein_co	ENSG0000	379	656
ARL5A	19.6444	16.7363	20.2098	18.7906	23.6195	23.0956	ADP ribosylation factor like GTPase 5A [Source:HGNC Symbol;Acc	chr2:15265	NM_00103	.	.	.	NP_00103	.	protein_co	ENSG0000	26225	696
ARL5B	9.59428	9.9088	9.41899	9.49963	12.776	15.6313	ADP ribosylation factor like GTPase 5B [Source:HGNC Symbol;Acc	chr10:1894	NM_17881	XM_00525	.	.	NP_84893	XP_005252	protein_co	ENSG0000	221079	23052
ARL5C	0	0	0	0	0	0	ADP ribosylation factor like GTPase 5C [Source:HGNC Symbol;Acc	chr17:3731	NM_00114	.	.	.	NP_00113	.	protein_co	ENSG0000	390790	31111
ARL6	1.12741	1.8378	1.00254	1.6764	1.24464	1.29897	ADP ribosylation factor like GTPase 6 [Source:HGNC Symbol;Acc:H	chr3:97483	NM_00127	XM_00671	.	.	NP_00126	XP_016862	protein_co	ENSG0000	84100	13210
ARL6IP1	162.459	156.869	149.57	231.262	278.027	239.237	ADP ribosylation factor like GTPase 6 interacting protein 1 [Sourc	chr16:1879	NM_01516	.	.	.	NP_00130	.	protein_co	ENSG0000	23204	697
ARL6IP4	74.1135	70.6835	62.8309	85.5388	63.4835	62.6485	ADP ribosylation factor like GTPase 6 interacting protein 4 [Sourc	chr12:1234	NM_00100	.	.	.	NP_00100	.	protein_co	ENSG0000	51329	18076
ARL6IP5	30.3019	29.9996	28.0565	29.4898	25.4091	29.4935	ADP ribosylation factor like GTPase 6 interacting protein 5 [Sourc	chr3:69134	NM_00640	.	.	.	NP_00639	.	protein_co	ENSG0000	10550	16937
ARL6IP6	13.7553	13.3706	15.0968	12.6005	13.8482	19.1492	ADP ribosylation factor like GTPase 6 interacting protein 6 [Sourc	chr2:15350	NM_15252	.	.	.	NP_68973	.	protein_co	ENSG0000	151188	24048
ARL8A	20.2049	19.2931	19.5026	23.5603	17.7942	18.3387	ADP ribosylation factor like GTPase 8A [Source:HGNC Symbol;Acc	chr1:20209	NM_00125	.	.	.	NP_00124	.	protein_co	ENSG0000	127829	25192
ARL8B	24.8813	23.812	23.3569	22.2089	29.0921	27.9603	ADP ribosylation factor like GTPase 8B [Source:HGNC Symbol;Acc	chr3:51639	NM_01818	XM_00526	.	.	NP_06065	XP_005265	protein_co	ENSG0000	55207	25564

ARL9	0.059078	0.062189	0	0	0.066001	0	ADP ribosylation factor like GTPase 9 [Source:HGNC Symbol;Acc:H	chr4:57371	NM_20691	XM_00671	.	.	NP_996802	XP_005265	protein_co	ENSG00000	132946	23592
ARMC1	20.1451	18.1638	18.2796	19.0298	23.9874	20.7949	armadillo repeat containing 1 [Source:HGNC Symbol;Acc:HGNC:1	chr8:66514	NM_00128	XM_01701	.	.	NP_001273	XP_005251	protein_co	ENSG00000	55156	17684
ARMC10	17.871	15.9997	19.3889	15.6396	19.4166	23.8808	armadillo repeat containing 10 [Source:HGNC Symbol;Acc:HGNC:	chr7:10271	NM_00116	XM_01151	.	.	NP_001154	XP_005250	protein_co	ENSG00000	83787	21706
ARMC12	0.259946	0.328876	0.31766	0.383407	0.341796	0.907136	armadillo repeat containing 12 [Source:HGNC Symbol;Acc:HGNC:	chr6:35704	NM_00128	XM_01151	.	.	NP_001273	XP_011512	protein_co	ENSG00000	221481	21099
ARMC2	0.676616	0.695386	0.686191	0.693222	0.574675	0.600603	armadillo repeat containing 2 [Source:HGNC Symbol;Acc:HGNC:2	chr6:10916	NM_00128	XM_01153	.	.	NP_001273	XP_005267	protein_co	ENSG00000	84071	23045
ARMC3	0.532992	0.386617	0.560699	0.691581	0.738394	1.11838	armadillo repeat containing 3 [Source:HGNC Symbol;Acc:HGNC:3	chr10:2321	NM_00128	XM_01701	.	.	NP_001269	XP_005252	protein_co	ENSG00000	219681	30964
ARMC4	4.11109	4.53343	5.40019	3.74437	4.33424	6.04695	armadillo repeat containing 4 [Source:HGNC Symbol;Acc:HGNC:2	chr10:2810	NM_00129	XM_01151	.	.	NP_001276	XP_011517	protein_co	ENSG00000	55130	25583
ARMC5	6.88256	7.23391	6.119	7.77998	6.40583	6.34737	armadillo repeat containing 5 [Source:HGNC Symbol;Acc:HGNC:2	chr16:3146	NM_00110	XM_00672	.	.	NP_001098	XP_006721	protein_co	ENSG00000	79798	25781
ARMC6	24.2469	24.0301	19.66	28.0473	23.3916	13.9658	armadillo repeat containing 6 [Source:HGNC Symbol;Acc:HGNC:2	chr19:1910	NM_00119	XM_01702	.	.	NP_001186	XP_005260	protein_co	ENSG00000	93436	25049
ARMC7	15.7543	18.815	16.6631	21.7514	17.4414	14.7732	armadillo repeat containing 7 [Source:HGNC Symbol;Acc:HGNC:2	chr17:7310	NM_00130	.	.	.	NP_001291	.	protein_co	ENSG00000	79637	26168
ARMC8	13.4522	13.4619	12.0828	13.956	16.2087	13.8794	armadillo repeat containing 8 [Source:HGNC Symbol;Acc:HGNC:2	chr3:13790	NM_00126	XM_01151	.	.	NP_001253	XP_006713	protein_co	ENSG00000	25852	24999
ARMC9	3.41256	3.78896	3.6627	3.84393	3.59987	2.95428	armadillo repeat containing 9 [Source:HGNC Symbol;Acc:HGNC:2	chr2:23206	NM_00127	XM_01700	.	.	NP_001258	XP_011510	protein_co	ENSG00000	80210	20730
ARMCX1	0	0	0	0	0	0	armadillo repeat containing, X-linked 1 [Source:HGNC Symbol;Acc	chrX:10080	NM_01660	.	.	.	NP_057692	.	protein_co	ENSG00000	51309	18073
ARMCX2	0	0	0	0	0	0	armadillo repeat containing, X-linked 2 [Source:HGNC Symbol;Acc	chrX:10091	NM_00128	XM_01702	.	.	NP_001269	XP_005278	protein_co	ENSG00000	9823	16869
ARMCX3	15.1418	16.8093	15.1941	13.0837	14.2869	12.8569	armadillo repeat containing, X-linked 3 [Source:HGNC Symbol;Acc	chrX:10087	NM_01660	XM_00526	.	.	NP_057691	XP_005262	protein_co	ENSG00000	51566	24065
ARMCX4	0	0	0.006774	0	0	0	armadillo repeat containing, X-linked 4 [Source:HGNC Symbol;Acc	chrX:10074	NM_00125	.	.	.	NP_001243	.	protein_co	ENSG00000	100131755	28615
ARMCX5	5.03076	5.82435	4.78293	6.50068	5.82233	5.57445	armadillo repeat containing, X-linked 5 [Source:HGNC Symbol;Acc	chrX:10185	NM_00116	.	.	.	NP_001161	.	protein_co	ENSG00000	64860	25772
ARMCX6	4.69373	4.92253	4.20348	5.41585	4.5788	3.37467	armadillo repeat containing, X-linked 6 [Source:HGNC Symbol;Acc	chrX:10087	NM_00100	XM_01153	.	.	NP_001009	XP_011529	protein_co	ENSG00000	54470	26094
ARMS2	0	0	0	0	0	0	age-related maculopathy susceptibility 2 [Source:HGNC Symbol;A	chr10:1242	NM_00109	.	.	.	NP_001093	.	protein_co	ENSG00000	387715	32685
ARMT1	14.5598	14.551	18.1193	16.6706	18.7813	19.7608	acidic residue methyltransferase 1 [Source:HGNC Symbol;Acc:HG	chr6:15177	NM_00128	.	.	.	NP_001273	.	protein_co	ENSG00000	79624	17872
ARNT	10.1569	10.8634	10.1188	9.36496	9.87712	11.7772	aryl hydrocarbon receptor nuclear translocator [Source:HGNC Sy	chr1:15078	NM_00119	XM_01700	.	.	NP_001184	XP_005245	protein_co	ENSG00000	405	700
ARNT2	2.57619	2.67792	2.80907	2.40067	2.19758	1.82074	aryl hydrocarbon receptor nuclear translocator 2 [Source:HGNC S	chr15:8069	NM_01486	.	NR_049837	.	NP_055677	.	protein_co	ENSG00000	100847042	16876
ARNTL	5.77997	5.8215	4.99025	6.63006	6.80534	6.25376	aryl hydrocarbon receptor nuclear translocator like [Source:HGNC	chr11:1329	NM_00103	XM_01701	.	.	NP_001025	XP_006718	protein_co	ENSG00000	406	701
ARNTL2	8.54393	8.43895	8.69075	11.949	14.2768	13.8603	aryl hydrocarbon receptor nuclear translocator like 2 [Source:HG	chr12:2748	NM_00124	XM_01701	.	.	NP_001234	XP_006719	protein_co	ENSG00000	56938	18984
ARPC1A	106.576	115.182	104.145	134.556	112.668	118.819	actin related protein 2/3 complex subunit 1A [Source:HGNC Symb	chr7:98923	NM_00119	.	.	.	NP_001177	.	protein_co	ENSG00000	10552	703
ARPC1B	123.236	133.201	155.113	150.726	121.984	127.362	actin related protein 2/3 complex subunit 1B [Source:HGNC Symb	chr7:98972	NM_00572	XM_00671	.	.	NP_005711	XP_006715	protein_co	ENSG00000	10095	704
ARPC2	136.314	130.027	132.679	142.144	141.233	144.741	actin related protein 2/3 complex subunit 2 [Source:HGNC Symbo	chr2:21908	NM_00573	.	.	.	NP_005722	.	protein_co	ENSG00000	10109	705
ARPC3	153.222	136.611	144.975	115.727	113.402	110.771	actin related protein 2/3 complex subunit 3 [Source:HGNC Symbo	chr12:1108	NM_00127	.	.	.	NP_001265	.	protein_co	ENSG00000	10094	706
ARPC4	84.6473	80.5659	74.8678	68.3127	55.9319	44.4164	actin related protein 2/3 complex subunit 4 [Source:HGNC Symbo	chr3:98215	NM_00102	.	.	.	NP_001020	.	protein_co	ENSG00000	10093	707
ARPC4-TTLL3	0.856601	0.549167	0.429534	0.615058	0.459506	1.12668	ARPC4-TTLL3 readthrough [Source:HGNC Symbol;Acc:HGNC:3883	chr3:98215	NM_00119	.	.	.	NP_001185	.	protein_co	ENSG00000	100526693	38830
ARPC5	50.9682	53.5328	49.666	50.1153	53.4863	56.1272	actin related protein 2/3 complex subunit 5 [Source:HGNC Symbo	chr1:18358	NM_00127	.	.	.	NP_001257	.	protein_co	ENSG00000	10092	708
ARPC5L	57.7392	58.2971	57.1133	76.0375	78.9222	68.3451	actin related protein 2/3 complex subunit 5 like [Source:HGNC Sy	chr9:12762	NM_03097	XM_00525	.	.	NP_112240	XP_005252	protein_co	ENSG00000	81873	23366
ARPIN	4.28745	4.35439	4.38965	3.62639	3.7305	4.39395	actin-related protein 2/3 complex inhibitor [Source:HGNC Symbo	chr15:9037	NM_00128	.	.	.	NP_001269	.	protein_co	ENSG00000	348110	28782
ARPP19	74.6088	74.4962	66.7975	77.1151	84.2805	80.7864	cAMP regulated phosphoprotein 19 [Source:HGNC Symbol;Acc:H	chr15:5283	NM_00130	XM_01702	.	.	NP_001293	XP_005254	protein_co	ENSG00000	10776	16967
ARPP21	0	0	0	0	0	0	cAMP regulated phosphoprotein 21 [Source:HGNC Symbol;Acc:H	chr3:35681	NM_00102	XM_01153	.	.	NP_001020	XP_005264	protein_co	ENSG00000	10777	16968
ARR3	0	0	0	0	0	0.057442	arrestin 3 [Source:HGNC Symbol;Acc:HGNC:710]	chrX:69488	NM_00431	.	.	.	NP_004303	XP_016885	protein_co	ENSG00000	407	710
ARRB1	6.86346	7.82109	7.98735	8.90199	7.74441	6.18701	arrestin beta 1 [Source:HGNC Symbol;Acc:HGNC:711]	chr11:7497	NM_00404	XM_01154	.	.	NP_004032	XP_011543	protein_co	ENSG00000	408	711
ARRB2	34.4183	34.2235	27.7934	36.8916	29.5826	23.9326	arrestin beta 2 [Source:HGNC Symbol;Acc:HGNC:712]	chr17:4613	NM_00125	XM_01152	.	.	NP_001244	XP_006721	protein_co	ENSG00000	409	712
ARRDC1	33.3063	34.3106	38.0272	27.9916	24.6309	34.1233	arrestin domain containing 1 [Source:HGNC Symbol;Acc:HGNC:28	chr9:14050	NM_15228	XM_00526	.	.	NP_001304	XP_005266	protein_co	ENSG00000	92714	28633
ARRDC2	16.7174	17.1117	18.1825	16.8675	13.8591	14.8937	arrestin domain containing 2 [Source:HGNC Symbol;Acc:HGNC:25	chr19:1811	NM_00102	.	.	.	NP_001020	.	protein_co	ENSG00000	27106	25225
ARRDC3	14.4042	14.3692	12.0568	10.8685	14.0953	18.2248	arrestin domain containing 3 [Source:HGNC Symbol;Acc:HGNC:29	chr5:90664	NM_02080	.	.	.	NP_065852	.	protein_co	ENSG00000	57561	29263
ARRDC4	10.7107	11.5259	8.11443	3.37462	4.21153	4.72411	arrestin domain containing 4 [Source:HGNC Symbol;Acc:HGNC:28	chr15:9850	NM_18337	.	.	.	NP_899232	.	protein_co	ENSG00000	91947	28087
ARRDC5	0	0	0	0	0	0	arrestin domain containing 5 [Source:HGNC Symbol;Acc:HGNC:31	chr19:4890	NM_00108	.	.	.	NP_001073	.	protein_co	ENSG00000	645432	31407
ARSA	5.85485	5.11223	8.25554	2.67318	2.13556	3.31682	arylsulfatase A [Source:HGNC Symbol;Acc:HGNC:713]	chr22:5106	NM_00048	XM_01153	.	.	NP_000478	XP_011528	protein_co	ENSG00000	410	713
ARSB	3.34086	3.26111	2.57748	3.41379	3.92974	3.48842	arylsulfatase B [Source:HGNC Symbol;Acc:HGNC:714]	chr5:78073	NM_00004	XM_01154	.	.	NP_000037	XP_011541	protein_co	ENSG00000	411	714
ARSD	2.28131	2.44112	2.91922	1.94835	1.92679	3.91932	arylsulfatase D [Source:HGNC Symbol;Acc:HGNC:717]	chrX:28220	NM_00166	XM_00527	.	.	NP_001660	XP_005274	protein_co	ENSG00000	414	717
ARSE	0.042486	0.193702	0.06612	0.046234	0	0	arylsulfatase E (chondrodysplasia punctata 1) [Source:HGNC Sym	chrX:28526	NM_00004	XM_01154	.	.	NP_000038	XP_005274	protein_co	ENSG00000	415	719
ARSF	0	0	0.032751	0	0	0	arylsulfatase F [Source:HGNC Symbol;Acc:HGNC:721]	chrX:29595	NM_00120	XM_01154	.	.	NP_001188	XP_011543	protein_co	ENSG00000	416	721
ARSG	3.77967	4.0334	6.25992	3.13675	2.86249	5.51422	arylsulfatase G [Source:HGNC Symbol;Acc:HGNC:24102]	chr17:6625	NM_00126	XM_01702	.	.	NP_001254	XP_005257	protein_co	ENSG00000	22901	24102
ARSH	0	0	0	0	0	0	arylsulfatase family member H [Source:HGNC Symbol;Acc:HGNC:3	chrX:29246	NM_00101	.	.	.	NP_001011	.	protein_co	ENSG00000	347527	32488
ARSI	0.080676	0.062167	0.056988	0.143968	0.074431	0.148202	arylsulfatase family member I [Source:HGNC Symbol;Acc:HGNC:3	chr5:14967	NM_00101	.	.	.	NP_001012	.	protein_co	ENSG00000	340075	32521
ARSJ	1.10278	1.2813	1.42931	0.724239	0.771365	0.640124	arylsulfatase family member J [Source:HGNC Symbol;Acc:HGNC:2	chr4:11482	NM_02459	XM_00526	.	.	NP_078866	XP_005263	protein_co	ENSG00000	79642	26286
ARSK	2.5967	3.65862	3.55676	4.08768	3.3779	3.34119	arylsulfatase family member K [Source:HGNC Symbol;Acc:HGNC:2	chr5:94890	NM_19815	XM_00527	.	.	NP_937793	XP_005271	protein_co	ENSG00000	153642	25239
ART1	0	0	0	0	0	0	ADP-ribosyltransferase 1 [Source:HGNC Symbol;Acc:HGNC:723]	chr11:3666	NM_00431	XM_01152	.	.	NP_004305	XP_011518	protein_co	ENSG00000	417	723
ART3	0	0	0	0	0	0	ADP-ribosyltransferase 3 [Source:HGNC Symbol;Acc:HGNC:725]	chr4:76932	NM_00113	XM_01700	.	.	NP_001123	XP_005263	protein_co	ENSG00000	419	725
ART4	0	0	0	0	0	0	ADP-ribosyltransferase 4 (Dombrock blood group) [Source:HGNC	chr12:1497	NM_02107	.	.	.	NP_066549	.	protein_co	ENSG00000	420	726
ART5	2.47405	3.04782	3.91846	4.39858	4.70747	3.59847	ADP-ribosyltransferase 5 [Source:HGNC Symbol;Acc:HGNC:24049	chr11:3659	NM_00107	XM_01151	.	.	NP_001073	XP_005252	protein_co	ENSG00000	116969	24049
ARTN	8.19867	13.8195	10.8397	11.6806	8.09242	8.99681	artemin [Source:HGNC Symbol;Acc:HGNC:727]	chr1:44398	NM_00113	XM_01154	.	.	NP_001129	XP_006711	protein_co	ENSG00000	9048	727
ARV1	2.77999	3.11139	2.6132	1.78952	2.6808	3.24125	ARV1 homolog, fatty acid homeostasis modulator [Source:HGNC	chr1:23111	NM_02278	.	.	.	NP_073623	XP_016857	protein_co	ENSG00000	64801	29561

ARVCF	13.3952	14.69	15.4116	13.791	12.2044	11.7403	armadillo repeat gene deleted in velocardiofacial syndrome [Sour	chr22:1986	NM_00167	XM_01153	.	.	NP_00166	XP_005261	protein_co	ENSG0000	421	728
ARX	0.211751	0.243535	0.323322	0.495612	0.250416	0.239019	aristaless related homeobox [Source:HGNC Symbol;Acc:HGNC:18	chrX:25021	NM_13905	.	.	.	NP_62068	.	protein_co	ENSG0000	170302	18060
AS3MT	0.420797	0.445745	0.403054	0.129965	0.324818	0.83955	arsenite methyltransferase [Source:HGNC Symbol;Acc:HGNC:174	chr10:1046	NM_02068	.	.	.	NP_06573	.	protein_co	ENSG0000	57412	17452
ASAH1	56.4012	51.5997	53.6299	61.1791	52.0146	46.8519	N-acylsphingosine amidohydrolase 1 [Source:HGNC Symbol;Acc:H	chr8:17913	NM_00112	XM_00527	.	.	NP_00112	XP_005273	protein_co	ENSG0000	427	735
ASAH2	0.07981	0.073717	0.049552	0.065301	0.100605	0.176744	N-acylsphingosine amidohydrolase 2 [Source:HGNC Symbol;Acc:H	chr10:5194	NM_00114	XM_01153	.	.	NP_00113	XP_011538	protein_co	ENSG0000	56624	18860
ASAH2B	2.94164	3.61745	3.26685	3.25672	3.91668	4.36306	N-acylsphingosine amidohydrolase 2B [Source:HGNC Symbol;Acc	chr10:5249	NM_00107	XM_00671	.	.	NP_00107	XP_005270	protein_co	ENSG0000	653308	23456
ASAH2C	0	0.033694	0	0	0	0.032055	N-acylsphingosine amidohydrolase (non-lysosomal ceramidase) 2	chr10:4800	.	.	.	.	.	.	protein_co	ENSG0000	.	23457
ASAP1	6.10733	7.2799	7.35227	5.37496	6.15956	7.0025	ArfGAP with SH3 domain, ankyrin repeat and PH domain 1 [Sourc	chr8:13106	NM_00124	XM_01701	.	.	NP_00123	XP_005250	protein_co	ENSG0000	50807	2720
ASAP2	5.73869	5.55908	6.73244	6.5794	6.47434	10.6634	ArfGAP with SH3 domain, ankyrin repeat and PH domain 2 [Sourc	chr2:93468	NM_00113	XM_01151	.	.	NP_00112	XP_006711	protein_co	ENSG0000	8853	2721
ASAP3	5.55241	6.66708	8.86851	8.42144	7.45742	8.57417	ArfGAP with SH3 domain, ankyrin repeat and PH domain 3 [Sourc	chr1:23755	NM_00114	XM_01700	.	.	NP_00113	XP_011540	protein_co	ENSG0000	55616	14987
ASB1	13.1128	12.6299	13.2962	10.553	12.402	14.5551	ankyrin repeat and SOCS box containing 1 [Source:HGNC Symbol;	chr2:23933	NM_00104	XM_00524	.	.	NP_00103	XP_005246	protein_co	ENSG0000	51665	16011
ASB10	0	0	0	0	0	0	ankyrin repeat and SOCS box containing 10 [Source:HGNC Symbo	chr7:15087	NM_00114	.	.	.	NP_00113	.	protein_co	ENSG0000	136371	17185
ASB11	0	0	0	0	0	0	ankyrin repeat and SOCS box containing 11 [Source:HGNC Symbo	chrX:15299	NM_00101	XM_01154	.	.	NP_00101	XP_005274	protein_co	ENSG0000	140456	17186
ASB12	0.064447	0.197511	0	0.140597	0.073748	0.272944	ankyrin repeat and SOCS box containing 12 [Source:HGNC Symbo	chrX:63444	NM_13038	.	.	.	NP_56905	.	protein_co	ENSG0000	142689	19763
ASB13	14.6496	15.3898	18.3322	13.3467	11.2015	10.2124	ankyrin repeat and SOCS box containing 13 [Source:HGNC Symbo	chr10:5680	NM_02470	.	NR_02458	.	NP_07897	.	protein_co	ENSG0000	79754	19765
ASB14	0	0	0	0	0	0	ankyrin repeat and SOCS box containing 14 [Source:HGNC Symbo	chr3:57261	NM_00114	XM_00671	.	.	NP_00113	XP_006713	protein_co	ENSG0000	142686	19766
ASB15	0	0	0	0	0	0	ankyrin repeat and SOCS box containing 15 [Source:HGNC Symbo	chr7:12320	NM_00129	XM_01151	.	.	NP_00127	XP_005250	protein_co	ENSG0000	142685	19767
ASB16	0.049172	0	0.041653	0.052302	0	0.055048	ankyrin repeat and SOCS box containing 16 [Source:HGNC Symbo	chr17:4224	NM_08086	.	.	.	NP_54313	.	protein_co	ENSG0000	92591	19768
ASB17	0	0	0	0	0	0	ankyrin repeat and SOCS box containing 17 [Source:HGNC Symbo	chr1:76384	NM_08086	XM_00671	.	.	NP_54314	XP_006710	protein_co	ENSG0000	127247	19769
ASB18	0.092299	0	0	0	0	0	ankyrin repeat and SOCS box containing 18 [Source:HGNC Symbo	chr2:23710	NM_21255	.	.	.	NP_99772	.	protein_co	ENSG0000	401036	19770
ASB2	0.32268	0.319466	0.48032	0.031647	0.067911	0	ankyrin repeat and SOCS box containing 2 [Source:HGNC Symbol;	chr14:9440	NM_00120	XM_01153	.	.	NP_00118	XP_005267	protein_co	ENSG0000	51676	16012
ASB3	9.23237	9.00242	8.86938	8.76574	11.2197	11.0328	ankyrin repeat and SOCS box containing 3 [Source:HGNC Symbol;	chr2:53897	NM_00120	.	.	.	NP_00118	.	protein_co	ENSG0000	51130	16013
ASB4	0.019673	0.020988	0.034193	0	0.056467	0.019271	ankyrin repeat and SOCS box containing 4 [Source:HGNC Symbol;	chr7:95115	NM_01611	.	.	.	NP_05720	.	protein_co	ENSG0000	51666	16009
ASB5	0	0.032721	0.069056	0	0	0	ankyrin repeat and SOCS box containing 5 [Source:HGNC Symbol;	chr4:17713	NM_08087	XM_01153	.	.	NP_54315	XP_005262	protein_co	ENSG0000	140458	17180
ASB6	18.0006	17.7472	19.9537	22.54	19.3854	11.992	ankyrin repeat and SOCS box containing 6 [Source:HGNC Symbol;	chr9:13237	NM_00120	.	.	.	NP_00118	.	protein_co	ENSG0000	140459	17181
ASB7	6.01669	7.27442	6.73279	5.52566	5.92128	6.12881	ankyrin repeat and SOCS box containing 7 [Source:HGNC Symbol;	chr15:1011	NM_02470	.	.	.	NP_07898	.	protein_co	ENSG0000	140460	17182
ASB8	7.18403	8.61312	10.1406	6.58267	6.3655	9.67842	ankyrin repeat and SOCS box containing 8 [Source:HGNC Symbol;	chr12:4854	NM_00131	XM_00526	.	.	NP_00130	XP_005268	protein_co	ENSG0000	140461	17183
ASB9	1.73245	1.72036	3.38799	1.79977	2.68792	1.9338	ankyrin repeat and SOCS box containing 9 [Source:HGNC Symbol;	chrX:15262	NM_00103	XM_01154	.	.	NP_00102	XP_005274	protein_co	ENSG0000	140462	17184
ASCC1	12.3392	11.502	13.9908	12.823	14.0564	17.2714	activating signal cointegrator 1 complex subunit 1 [Source:HGNC	chr10:7385	NM_00119	XM_01153	.	.	NP_00118	XP_006717	protein_co	ENSG0000	51008	24268
ASCC2	18.3599	20.8774	18.887	22.0328	19.3749	16.3755	activating signal cointegrator 1 complex subunit 2 [Source:HGNC	chr22:3018	NM_00124	XM_01702	.	.	NP_00122	XP_005261	protein_co	ENSG0000	84164	24103
ASCC3	13.0308	13.6257	15.6191	11.933	15.6312	14.2196	activating signal cointegrator 1 complex subunit 3 [Source:HGNC	chr6:10095	NM_00128	XM_01153	.	.	NP_00127	XP_011533	protein_co	ENSG0000	10973	18697
ASCL1	0	0	0	0	0	0	achaete-scute family bHLH transcription factor 1 [Source:HGNC S	chr12:1033	NM_00431	.	.	.	NP_00430	.	protein_co	ENSG0000	429	738
ASCL2	0	0	0	0	0	0	achaete-scute family bHLH transcription factor 2 [Source:HGNC S	chr11:2289	NM_00517	.	.	.	NP_00516	.	protein_co	ENSG0000	430	739
ASCL3	0	0	0	0	0	0	achaete-scute family bHLH transcription factor 3 [Source:HGNC S	chr11:8959	NM_02064	.	.	.	NP_06569	.	protein_co	ENSG0000	56676	740
ASCL4	0	0	0	0	0	0	achaete-scute family bHLH transcription factor 4 [Source:HGNC S	chr12:1081	NM_20343	.	.	.	NP_98226	.	protein_co	ENSG0000	121549	24311
ASCL5	0.347934	0	0.445257	0.184795	0.208148	0.29657	achaete-scute family bHLH transcription factor 5 [Source:HGNC S	chr1:20108	NM_00127	.	.	.	NP_00125	.	protein_co	ENSG0000	647219	33169
ASF1A	15.9047	15.142	13.2	19.786	22.9109	25.047	anti-silencing function 1A histone chaperone [Source:HGNC Symb	chr6:11913	NM_01403	.	.	.	NP_05475	.	protein_co	ENSG0000	25842	20995
ASF1B	68.6075	66.5351	56.3432	86.1157	73.3202	63.4283	anti-silencing function 1B histone chaperone [Source:HGNC Symb	chr19:1423	NM_01815	.	.	.	NP_06062	.	protein_co	ENSG0000	55723	20996
ASGR1	0.256298	0.632316	0.716091	0.697766	0.69839	0.571205	asialoglycoprotein receptor 1 [Source:HGNC Symbol;Acc:HGNC:74	chr17:7076	NM_00119	XM_01152	.	.	NP_00118	XP_011522	protein_co	ENSG0000	432	742
ASGR2	0	0	0	0	0	0	asialoglycoprotein receptor 2 [Source:HGNC Symbol;Acc:HGNC:74	chr17:7004	NM_00118	XM_01702	.	.	NP_00117	XP_005256	protein_co	ENSG0000	433	743
ASH1L	7.65984	8.74774	9.10631	6.38961	7.79536	7.8899	ASH1 like histone lysine methyltransferase [Source:HGNC Symbol	chr1:15530	NM_01848	XM_01700	NR_03028	.	NP_06095	XP_006711	protein_co	ENSG0000	693140	19088
ASH2L	18.0768	17.2417	17.7332	19.5314	19.4688	23.3052	ASH2 like histone lysine methyltransferase complex subunit [Sour	chr8:37962	NM_00110	XM_00671	.	.	NP_00109	XP_005273	protein_co	ENSG0000	9070	744
ASIC1	9.37519	9.77512	16.4508	7.99863	7.5077	8.6046	acid sensing ion channel subunit 1 [Source:HGNC Symbol;Acc:HG	chr12:5045	NM_00109	XM_01153	.	.	NP_00108	XP_011536	protein_co	ENSG0000	41	100
ASIC2	0	0	0	0	0	0	acid sensing ion channel subunit 2 [Source:HGNC Symbol;Acc:HG	chr17:3134	NM_00109	.	.	.	NP_00108	.	protein_co	ENSG0000	40	99
ASIC3	0.847213	0.813077	0.764561	1.32891	1.45828	1.25611	acid sensing ion channel subunit 3 [Source:HGNC Symbol;Acc:HG	chr7:15074	NM_00476	.	.	.	NP_00476	.	protein_co	ENSG0000	9311	101
ASIC4	0.335831	0.229106	0.446951	0.323843	0.17126	0.546534	acid sensing ion channel subunit family member 4 [Source:HGNC	chr2:22037	NM_18284	XM_01151	.	.	NP_87826	XP_005246	protein_co	ENSG0000	55515	21263
ASIC5	0	0	0	0	0	0	acid sensing ion channel subunit family member 5 [Source:HGNC	chr4:15675	NM_01741	.	.	.	NP_05911	.	protein_co	ENSG0000	51802	17537
ASIP	0	0	0	0	0	0	agouti signaling protein [Source:HGNC Symbol;Acc:HGNC:745]	chr20:3278	NM_00167	XM_01152	.	.	NP_00166	XP_005260	protein_co	ENSG0000	434	745
ASL	22.5504	23.9886	26.0546	24.7673	21.131	22.3374	argininosuccinate lyase [Source:HGNC Symbol;Acc:HGNC:746]	chr7:65540	NM_00004	.	.	.	NP_00003	.	protein_co	ENSG0000	435	746
ASMT	0	0	0.070369	0	0	0	acetylserotonin O-methyltransferase [Source:HGNC Symbol;Acc:H	chrX:17338	NM_00117	.	.	.	NP_00116	.	protein_co	ENSG0000	438	750
ASMTL	13.3631	14.558	18.3058	16.9798	14.6317	13.82	acetylserotonin O-methyltransferase-like [Source:HGNC Symbol;A	chrX:15220	NM_00117	XM_00527	.	.	NP_00116	XP_005274	protein_co	ENSG0000	8623	751
ASNA1	71.2939	69.8535	51.807	98.8768	75.8973	54.3516	arsA arsenite transporter, ATP-binding, homolog 1 (bacterial) [So	chr19:1284	NM_00431	.	.	.	NP_00430	.	protein_co	ENSG0000	439	752
ASNS	143.356	130.354	63.357	68.8864	64.1756	439.347	asparagine synthetase (glutamine-hydrolyzing) [Source:HGNC Syr	chr7:97481	NM_00117	.	.	.	NP_00117	.	protein_co	ENSG0000	440	753
ASNSD1	24.981	20.3794	23.7773	21.2582	28.8991	28.2078	asparagine synthetase domain containing 1 [Source:HGNC Symbo	chr2:19052	NM_01904	XM_00524	.	.	NP_06192	XP_005246	protein_co	ENSG0000	54529	24910
ASPA	0	0	0	0	0	0	aspartoacylase [Source:HGNC Symbol;Acc:HGNC:756]	chr17:3343	NM_00004	XM_00672	.	.	NP_00004	XP_006721	protein_co	ENSG0000	443	756
ASPDH	0	0	0	0	0	0	aspartate dehydrogenase domain containing [Source:HGNC Symb	chr19:5101	NM_00102	.	.	.	NP_00101	.	protein_co	ENSG0000	554235	33856
ASPG	0	0	0.047089	0	0	0	asparaginase [Source:HGNC Symbol;Acc:HGNC:20123]	chr14:1045	NM_00108	XM_01153	.	.	NP_00107	XP_005267	protein_co	ENSG0000	374569	20123
ASPH	98.5769	100.852	124.52	94.411	98.4371	141.303	aspartate beta-hydroxylase [Source:HGNC Symbol;Acc:HGNC:757	chr8:62200	NM_00116	XM_01701	.	.	NP_00115	XP_005251	protein_co	ENSG0000	444	757
ASPHD1	7.25853	7.44157	9.71158	7.26676	7.59627	5.93492	aspartate beta-hydroxylase domain containing 1 [Source:HGNC S	chr16:2991	NM_18171	.	.	.	NP_85906	.	protein_co	ENSG0000	253982	27380

ASPHD2	4.03402	3.58553	3.52977	4.05448	3.77119	3.04052	aspartate beta-hydroxylase domain containing 2 [Source:HGNC S	chr22:2682	NM_02043	.	.	.	NP_06517	.	protein_co	ENSG0000	57168	30437
ASPM	8.63105	8.1403	9.4476	10.4914	18.2143	15.7361	abnormal spindle microtubule assembly [Source:HGNC Symbol;A	chr1:19705	NM_00120	.	.	.	NP_00119	.	protein_co	ENSG0000	259266	19048
ASPN	0	0	0	0	0	0	asporin [Source:HGNC Symbol;Acc:HGNC:14872]	chr9:95059	NM_00119	.	.	.	NP_00118	.	protein_co	ENSG0000	54829	14872
ASPRV1	0.727507	0.491629	0.481126	0.453812	0.752562	0.295034	aspartic peptidase, retroviral-like 1 [Source:HGNC Symbol;Acc:HG	chr2:70187	NM_15279	.	.	.	NP_69000	.	protein_co	ENSG0000	151516	26321
ASPSCR1	14.7451	16.4426	16.3723	21.7391	16.2047	13.397	ASPSCR1, UBX domain containing tether for SLC2A4 [Source:HGN	chr17:7993	NM_00125	XM_01702	.	.	NP_00123	XP_011521	protein_co	ENSG0000	79058	13825
ASRGL1	15.5788	17.023	16.5858	21.4529	20.7871	17.3503	asparaginase like 1 [Source:HGNC Symbol;Acc:HGNC:16448]	chr11:6210	NM_00108	XM_00527	.	.	NP_00107	XP_005274	protein_co	ENSG0000	80150	16448
ASS1	30.6024	31.716	48.9395	31.0153	25.9558	49.1781	argininosuccinate synthase 1 [Source:HGNC Symbol;Acc:HGNC:75	chr9:13332	NM_00005	XM_01701	.	.	NP_00004	XP_005272	protein_co	ENSG0000	445	758
ASTE1	1.99222	1.88696	1.53011	2.13978	1.91538	1.38308	asteroid homolog 1 (Drosophila) [Source:HGNC Symbol;Acc:HGN	chr3:13056	NM_00128	XM_01700	.	.	NP_00127	XP_016861	protein_co	ENSG0000	28990	25021
ASTL	0.135475	0.199842	0.116578	0.288764	0.078784	0.150167	astacin-like metallo-endopeptidase (M12 family) [Source:HGNC S	chr2:96789	NM_00100	XM_01151	.	.	NP_00100	XP_011509	protein_co	ENSG0000	431705	31704
ASTN1	0.29507	0.195001	0.345873	0	0.021626	0	astrotactin 1 [Source:HGNC Symbol;Acc:HGNC:773]	chr1:17682	NM_00128	.	.	.	NP_00127	XP_016856	protein_co	ENSG0000	460	773
ASTN2	3.54291	3.70297	4.40551	3.95165	2.77896	3.32126	astrotactin 2 [Source:HGNC Symbol;Acc:HGNC:17021]	chr9:11918	NM_00118	.	.	.	NP_00117	.	protein_co	ENSG0000	23245	17021
ASUN	19.0636	17.8142	20.0488	22.1273	23.3251	18.8242	asunder, spermatogenesis regulator [Source:HGNC Symbol;Acc:H	chr12:2705	NM_01816	XM_01152	.	.	NP_06063	XP_005253	protein_co	ENSG0000	55726	20174
ASXL1	17.8139	18.3223	17.1813	16.1952	16.6439	14.7038	additional sex combs like 1, transcriptional regulator [Source:HGN	chr20:3094	NM_00116	XM_01152	.	.	NP_00115	XP_006723	protein_co	ENSG0000	171023	18318
ASXL2	7.03093	8.14987	8.13665	6.74485	7.90092	9.13382	additional sex combs like 2, transcriptional regulator [Source:HGN	chr2:25956	NM_01826	XM_01700	.	.	NP_06073	XP_006712	protein_co	ENSG0000	55252	23805
ASXL3	0	0	0	0	0	0	additional sex combs like 3, transcriptional regulator [Source:HGN	chr18:3115	NM_03063	XM_01702	.	.	NP_08513	XP_005258	protein_co	ENSG0000	80816	29357
ASZ1	0	0	0	0	0.047888	0	ankyrin repeat, SAM and basic leucine zipper domain containing	chr7:11700	NM_00130	XM_01151	.	.	NP_00128	.	protein_co	ENSG0000	136991	1350
ATAD1	26.8337	26.5077	26.8993	24.155	31.5754	35.3453	ATPase family, AAA domain containing 1 [Source:HGNC Symbol;A	chr10:8951	NM_00132	XM_01154	.	.	NP_00130	XP_005270	protein_co	ENSG0000	84896	25903
ATAD2	43.6921	44.1044	40.6399	54.2077	63.302	70.5353	ATPase family, AAA domain containing 2 [Source:HGNC Symbol;A	chr8:12433	NM_01410	XM_01151	.	.	NP_05482	XP_011515	protein_co	ENSG0000	29028	30123
ATAD2B	1.85753	1.68005	1.88135	1.57067	2.02366	2.18678	ATPase family, AAA domain containing 2B [Source:HGNC Symbol;	chr2:23971	NM_00124	XM_01153	.	.	NP_00122	XP_005264	protein_co	ENSG0000	54454	29230
ATAD3A	46.3332	49.9257	42.7623	73.9105	63.8693	37.1794	ATPase family, AAA domain containing 3A [Source:HGNC Symbol;	chr1:14475	NM_00117	.	.	.	NP_00116	.	protein_co	ENSG0000	55210	25567
ATAD3B	22.9452	24.2103	20.2822	34.0697	28.0363	16.6145	ATPase family, AAA domain containing 3B [Source:HGNC Symbol;	chr1:14071	NM_03192	XM_01154	.	.	NP_11412	XP_005244	protein_co	ENSG0000	83858	24007
ATAD3C	0.164969	0.308202	0.141721	0.42409	0.597401	0.647219	ATPase family, AAA domain containing 3C [Source:HGNC Symbol;	chr1:13850	NM_00103	XM_01154	.	.	NP_00103	XP_011539	protein_co	ENSG0000	219293	32151
ATAD5	2.4424	2.68737	2.68774	1.96753	3.2673	2.58225	ATPase family, AAA domain containing 5 [Source:HGNC Symbol;A	chr17:2915	NM_02485	XM_01152	.	.	NP_07913	XP_011523	protein_co	ENSG0000	79915	25752
ATAT1	8.59401	8.07079	8.19977	8.97645	6.78991	7.71806	alpha tubulin acetyltransferase 1 [Source:HGNC Symbol;Acc:HGN	chr6:30594	NM_00103	XM_00524	NR_033823	.	NP_00102	XP_005249	protein_co	ENSG0000	79969	21186
ATCAY	0	0	0	0	0	0	ATCAY, caytaxin [Source:HGNC Symbol;Acc:HGNC:779]	chr19:3880	NM_03306	.	.	.	NP_14905	.	protein_co	ENSG0000	85300	779
ATE1	10.7946	11.1196	11.5045	10.9028	12.8328	14.8505	arginyltransferase 1 [Source:HGNC Symbol;Acc:HGNC:782]	chr10:1234	NM_00100	XM_00526	.	.	NP_00100	XP_005269	protein_co	ENSG0000	11101	782
ATF1	12.8392	13.227	12.1478	12.1932	11.5095	13.2305	activating transcription factor 1 [Source:HGNC Symbol;Acc:HGNC	chr12:5115	NM_00517	XM_01701	.	.	NP_00516	XP_011536	protein_co	ENSG0000	466	783
ATF2	11.0665	9.51246	11.2101	8.24346	8.63509	10.4031	activating transcription factor 2 [Source:HGNC Symbol;Acc:HGNC	chr2:17593	NM_00125	.	.	.	NP_00124	.	protein_co	ENSG0000	1386	784
ATF3	38.5506	40.4456	15.4215	17.4756	18.5164	30.5278	activating transcription factor 3 [Source:HGNC Symbol;Acc:HGNC	chr1:21273	NM_00103	XM_01150	.	.	NP_00102	XP_005273	protein_co	ENSG0000	467	785
ATF4	457.853	440.766	297.117	383.672	299.252	657.976	activating transcription factor 4 [Source:HGNC Symbol;Acc:HGNC	chr22:3991	NM_00167	XM_01702	.	.	NP_00166	XP_016884	protein_co	ENSG0000	468	786
ATF5	18.9352	16.3779	12.163	29.0995	28.5003	39.2549	activating transcription factor 5 [Source:HGNC Symbol;Acc:HGNC	chr19:5039	NM_00119	XM_01152	NR_03990	.	NP_00118	XP_011524	protein_co	ENSG0000	100616483	790
ATF6	10.3174	9.72871	9.36512	10.785	10.9894	16.5814	activating transcription factor 6 [Source:HGNC Symbol;Acc:HGNC	chr1:16173	NM_00734	XM_01150	.	.	NP_03137	XP_006711	protein_co	ENSG0000	22926	791
ATF6B	27.6369	27.9655	21.4644	21.419	17.9525	16.3099	activating transcription factor 6 beta [Source:HGNC Symbol;Acc:H	chr6:32083	NM_00113	.	.	.	NP_00112	.	protein_co	ENSG0000	1388	2349
ATF7	10.7634	10.9574	9.54312	8.09464	8.40825	10.1194	activating transcription factor 7 [Source:HGNC Symbol;Acc:HGNC	chr12:5390	NM_00113	XM_01153	.	.	NP_00112	XP_005268	protein_co	ENSG0000	11016	792
ATF7IP	7.24062	8.66224	8.08034	7.73158	9.59291	10.7787	activating transcription factor 7 interacting protein [Source:HGN	chr12:1451	NM_00128	XM_01701	.	.	NP_00127	XP_005253	protein_co	ENSG0000	55729	20092
ATF7IP2	2.61973	2.07091	1.84404	2.47676	3.27028	2.63387	activating transcription factor 7 interacting protein 2 [Source:HG	chr16:1047	NM_00125	XM_00672	.	.	NP_00124	XP_006721	protein_co	ENSG0000	80063	20397
ATG10	7.19586	4.33418	5.69198	6.63033	4.83695	6.4004	autophagy related 10 [Source:HGNC Symbol;Acc:HGNC:20315]	chr5:81267	NM_00113	XM_00524	.	.	NP_00112	XP_005248	protein_co	ENSG0000	83734	20315
ATG101	38.8756	40.1743	31.3373	44.8788	37.1852	36.8342	autophagy related 101 [Source:HGNC Symbol;Acc:HGNC:25679]	chr12:5246	NM_00109	.	.	.	NP_00109	.	protein_co	ENSG0000	60673	25679
ATG12	37.1413	37.0212	42.1654	33.1657	32.7739	50.6768	autophagy related 12 [Source:HGNC Symbol;Acc:HGNC:588]	chr5:11516	NM_00127	.	.	.	NP_00126	.	protein_co	ENSG0000	9140	588
ATG13	20.8316	21.9565	21.4086	17.68	15.7325	19.068	autophagy related 13 [Source:HGNC Symbol;Acc:HGNC:29091]	chr11:4663	NM_00114	XM_01701	.	.	NP_00113	XP_005253	protein_co	ENSG0000	9776	29091
ATG14	5.58783	6.28525	5.37064	5.74075	5.29749	6.2479	autophagy related 14 [Source:HGNC Symbol;Acc:HGNC:19962]	chr14:5583	NM_01492	XM_01153	.	.	NP_05573	XP_011534	protein_co	ENSG0000	22863	19962
ATG16L1	14.7572	15.8221	16.1133	16.6477	16.7509	13.2286	autophagy related 16 like 1 [Source:HGNC Symbol;Acc:HGNC:214	chr2:23416	NM_00119	XM_00671	.	.	NP_00117	XP_005246	protein_co	ENSG0000	55054	21498
ATG16L2	3.08551	2.24735	4.39022	1.89429	1.57416	1.54708	autophagy related 16 like 2 [Source:HGNC Symbol;Acc:HGNC:254	chr11:7252	NM_00131	XM_01154	.	.	NP_00130	XP_005274	protein_co	ENSG0000	89849	25464
ATG2A	4.88946	5.52281	4.92679	5.17221	3.39489	3.60707	autophagy related 2A [Source:HGNC Symbol;Acc:HGNC:29028]	chr11:6466	NM_01510	XM_01154	.	.	NP_05591	XP_005273	protein_co	ENSG0000	23130	29028
ATG2B	1.69494	1.84386	2.01528	1.19062	1.40951	1.83547	autophagy related 2B [Source:HGNC Symbol;Acc:HGNC:20187]	chr14:9674	NM_01803	XM_01153	.	.	NP_06050	XP_006720	protein_co	ENSG0000	55102	20187
ATG3	26.3576	25.0607	20.3425	25.691	24.0783	21.1791	autophagy related 3 [Source:HGNC Symbol;Acc:HGNC:20962]	chr3:11225	NM_00127	XM_01151	.	.	NP_00126	XP_011511	protein_co	ENSG0000	64422	20962
ATG4A	5.95069	5.34689	6.83054	6.85375	6.71611	7.7546	autophagy related 4A cysteine peptidase [Source:HGNC Symbol;A	chrX:10733	NM_00132	XM_01153	.	.	NP_00130	XP_011529	protein_co	ENSG0000	115201	16489
ATG4B	25.7455	29.0269	29.019	33.2093	27.6906	26.9545	autophagy related 4B cysteine peptidase [Source:HGNC Symbol;A	chr2:24252	NM_01332	XM_01151	.	.	NP_03745	XP_005247	protein_co	ENSG0000	23192	20790
ATG4C	4.20281	3.91881	5.74214	4.31065	5.47209	6.36427	autophagy related 4C cysteine peptidase [Source:HGNC Symbol;A	chr1:63249	NM_03285	XM_01154	.	.	NP_11624	XP_005271	protein_co	ENSG0000	84938	16040
ATG4D	15.6528	14.1893	15.4136	17.0548	12.5505	14.6245	autophagy related 4D cysteine peptidase [Source:HGNC Symbol;A	chr19:1065	NM_00128	.	.	.	NP_00126	.	protein_co	ENSG0000	84971	20789
ATG5	15.3408	14.8002	15.7812	18.394	18.9732	20.8056	autophagy related 5 [Source:HGNC Symbol;Acc:HGNC:589]	chr6:10649	NM_00128	.	.	.	NP_00127	.	protein_co	ENSG0000	9474	589
ATG7	8.77704	9.63284	9.63221	8.6959	7.55336	8.49231	autophagy related 7 [Source:HGNC Symbol;Acc:HGNC:16935]	chr3:11314	NM_00113	XM_01153	.	.	NP_00112	XP_006712	protein_co	ENSG0000	10533	16935
ATG9A	22.8462	25.5846	24.3094	23.9598	20.9503	22.7065	autophagy related 9A [Source:HGNC Symbol;Acc:HGNC:22408]	chr2:22008	NM_00107	.	.	.	NP_00107	.	protein_co	ENSG0000	79065	22408
ATG9B	0.440663	0.102326	0.223032	0.111212	0.150264	0.343394	autophagy related 9B [Source:HGNC Symbol;Acc:HGNC:21899]	chr7:15071	NM_17368	XM_01151	.	.	NP_00130	XP_011514	protein_co	ENSG0000	285973	21899
ATHL1	8.10851	8.06227	14.2622	5.59103	5.20579	5.60816	protein-glucosylgalactosylhydroxylysine glucosidase [Source:HGN	chr11:2891	NM_02509	XM_01152	.	.	NP_07936	XP_011518	protein_co	ENSG0000	80162	26210
ATIC	64.4414	63.964	66.4721	78.6915	70.0727	67.0924	5-aminoimidazole-4-carboxamide ribonucleotide formyltransfera	chr2:21617	NM_00404	.	.	.	NP_00403	XP_016859	protein_co	ENSG0000	471	794
ATL1	1.82227	1.57434	1.1111	1.15532	1.42007	0.954415	atlastin GTPase 1 [Source:HGNC Symbol;Acc:HGNC:11231]	chr14:5099	NM_00112	.	.	.	NP_00112	.	protein_co	ENSG0000	51062	11231
ATL2	22.4148	25.0451	25.7123	25.9589	35.1378	34.2091	atlastin GTPase 2 [Source:HGNC Symbol;Acc:HGNC:24047]	chr2:38522	NM_00113	XM_01153	.	.	NP_00112	XP_006712	protein_co	ENSG0000	64225	24047
ATL3	14.199	16.5662	12.7568	13.6654	15.4616	15.0487	atlastin GTPase 3 [Source:HGNC Symbol;Acc:HGNC:24526]	chr11:6339	NM_00129	XM_00671	.	.	NP_00127	XP_006718	protein_co	ENSG0000	25923	24526

ATM	4.65485	3.83381	4.63802	3.31942	4.28741	4.01829	ATM serine/threonine kinase [Source:HGNC Symbol;Acc:HGNC:79	chr11:1080	NM_00005	XM_01701	.	.	NP_00004	XP_005271	protein_co	ENSG0000	472	795
ATMIN	33.9885	33.3832	30.5775	33.6542	39.2515	31.6913	ATM interactor [Source:HGNC Symbol;Acc:HGNC:29034]	chr16:8106	NM_00130	.	.	.	NP_00128	.	protein_co	ENSG0000	23300	29034
ATN1	32.7048	34.245	27.3918	26.9302	26.2358	22.6682	atrophin 1 [Source:HGNC Symbol;Acc:HGNC:3033]	chr12:7033	NM_00100	.	.	.	NP_00100	.	protein_co	ENSG0000	1822	3033
ATOH1	0.049816	0.198901	0.042371	0.054465	0.057497	0.159488	atonal bHLH transcription factor 1 [Source:HGNC Symbol;Acc:HGI	chr4:94749	NM_00517	.	.	.	NP_00516	.	protein_co	ENSG0000	474	797
ATOH7	0	0.182067	0.077191	0	0.206445	0	atonal bHLH transcription factor 7 [Source:HGNC Symbol;Acc:HGI	chr10:6999	NM_14517	.	.	.	NP_66016	.	protein_co	ENSG0000	220202	13907
ATOH8	0.540975	0.403602	0.355333	0.397302	0.732994	0.841961	atonal bHLH transcription factor 8 [Source:HGNC Symbol;Acc:HGI	chr2:85981	NM_03282	.	.	.	NP_11621	XP_006712	protein_co	ENSG0000	84913	24126
ATOX1	49.469	44.7655	76.7192	62.3759	42.1194	54.4406	antioxidant 1 copper chaperone [Source:HGNC Symbol;Acc:HGNC	chr5:15112	NM_00404	.	.	.	NP_00403	.	protein_co	ENSG0000	475	798
ATP10A	0	0	0	0	0	0	ATPase phospholipid transporting 10A (putative) [Source:HGNC S	chr15:2592	NM_02449	XM_01152	.	.	NP_07781	XP_005268	protein_co	ENSG0000	57194	13542
ATP10B	0	0	0	0.00956	0.009723	0	ATPase phospholipid transporting 10B (putative) [Source:HGNC S	chr5:15999	NM_02515	XM_01153	.	.	NP_07942	XP_006714	protein_co	ENSG0000	23120	13543
ATP10D	1.88874	1.85813	2.40213	2.11403	2.41799	2.61435	ATPase phospholipid transporting 10D (putative) [Source:HGNC S	chr4:47487	NM_02045	XM_01151	.	.	NP_06518	XP_005248	protein_co	ENSG0000	57205	13549
ATP11A	11.4392	11.9153	12.0394	12.1961	13.9992	15.2926	ATPase phospholipid transporting 11A [Source:HGNC Symbol;Acc	chr13:1133	NM_01520	XM_01153	.	.	NP_05602	XP_005268	protein_co	ENSG0000	23250	13552
ATP11B	9.87709	9.88669	12.076	8.72222	12.2468	17.0921	ATPase phospholipid transporting 11B (putative) [Source:HGNC S	chr3:18251	NM_01461	XM_01700	.	.	NP_05543	XP_005247	protein_co	ENSG0000	23200	13553
ATP11C	4.78275	4.85841	5.96476	3.92608	5.7904	6.49502	ATPase phospholipid transporting 11C [Source:HGNC Symbol;Acc	chrX:13880	NM_00101	XM_01702	.	.	NP_00101	XP_016884	protein_co	ENSG0000	286410	13554
ATP12A	0.199142	0.227153	0.13656	0.208994	0.198095	0.31543	ATPase H+/K+ transporting non-gastric alpha2 subunit [Source:H	chr13:2525	NM_00118	.	.	.	NP_00117	.	protein_co	ENSG0000	479	13816
ATP13A1	21.5298	22.4271	21.0664	25.7909	21.3771	17.6398	ATPase 13A1 [Source:HGNC Symbol;Acc:HGNC:24215]	chr19:1975	NM_02041	XM_01152	.	.	NP_06514	.	protein_co	ENSG0000	57130	24215
ATP13A2	21.2739	22.5139	23.2845	20.9401	18.6913	20.7444	ATPase 13A2 [Source:HGNC Symbol;Acc:HGNC:30213]	chr1:17312	NM_00114	XM_01700	.	.	NP_00113	XP_005245	protein_co	ENSG0000	23400	30213
ATP13A3	27.9092	30.2931	29.9484	25.2176	36.081	40.0082	ATPase 13A3 [Source:HGNC Symbol;Acc:HGNC:24113]	chr3:19412	NM_02452	XM_01151	.	.	NP_07880	XP_005269	protein_co	ENSG0000	79572	24113
ATP13A4	0	0	0	0	0	0	ATPase 13A4 [Source:HGNC Symbol;Acc:HGNC:25422]	chr3:19311	NM_03227	XM_01700	.	.	NP_11565	XP_016862	protein_co	ENSG0000	84239	25422
ATP13A5	0	0	0	0	0	0	ATPase 13A5 [Source:HGNC Symbol;Acc:HGNC:31789]	chr3:19299	NM_19850	XM_01151	.	.	NP_94090	XP_011511	protein_co	ENSG0000	344905	31789
ATP1A1	148.197	154.991	143.72	149.913	145.157	153.887	ATPase Na+/K+ transporting subunit alpha 1 [Source:HGNC Symb	chr1:11691	NM_00070	XM_00671	.	.	NP_00069	XP_006710	protein_co	ENSG0000	476	799
ATP1A2	0	0	0	0	0	0	ATPase Na+/K+ transporting subunit alpha 2 [Source:HGNC Symb	chr1:16008	NM_00070	.	.	.	NP_00069	.	protein_co	ENSG0000	477	800
ATP1A3	8.62292	8.63568	9.18072	8.57364	8.67089	6.91186	ATPase Na+/K+ transporting subunit alpha 3 [Source:HGNC Symb	chr19:4247	NM_00125	XM_01152	.	.	NP_00124	XP_011525	protein_co	ENSG0000	478	801
ATP1A4	0	0	0	0	0	0	ATPase Na+/K+ transporting subunit alpha 4 [Source:HGNC Symb	chr1:16012	NM_00100	XM_01150	.	.	NP_00100	XP_011507	protein_co	ENSG0000	480	14073
ATP1B1	27.5789	26.1025	22.0247	16.6711	18.226	20.1452	ATPase Na+/K+ transporting subunit beta 1 [Source:HGNC Symbo	chr1:16907	NM_00167	.	.	.	NP_00166	.	protein_co	ENSG0000	481	804
ATP1B2	0	0	0.024868	0	0	0	ATPase Na+/K+ transporting subunit beta 2 [Source:HGNC Symbo	chr17:7554	NM_00130	.	.	.	NP_00129	.	protein_co	ENSG0000	482	805
ATP1B3	109.869	107.144	109.685	128.266	120.995	146.29	ATPase Na+/K+ transporting subunit beta 3 [Source:HGNC Symbo	chr3:14159	NM_00167	.	.	.	NP_00167	.	protein_co	ENSG0000	483	806
ATP1B4	0	0	0	0	0	0	ATPase Na+/K+ transporting family member beta 4 [Source:HGNC	chrX:11949	NM_00114	.	.	.	NP_00113	.	protein_co	ENSG0000	23439	808
ATP23	4.55276	5.02937	4.77596	5.1375	4.94198	5.92107	ATP23 metallopeptidase and ATP synthase assembly factor homo	chr12:5833	NM_03327	.	.	.	NP_15059	.	protein_co	ENSG0000	91419	29452
ATP2A1	1.34045	0.581592	1.29104	0.91732	0.948128	1.51671	ATPase sarcoplasmic/endoplasmic reticulum Ca2+ transporting 1	chr16:2888	NM_00128	.	.	.	NP_00127	.	protein_co	ENSG0000	487	811
ATP2A2	99.7898	109.882	82.7727	105.024	112.91	97.2027	ATPase sarcoplasmic/endoplasmic reticulum Ca2+ transporting 2	chr12:1107	NM_00168	XM_01153	.	.	NP_00167	XP_005253	protein_co	ENSG0000	488	812
ATP2A3	6.87332	7.7994	9.62692	9.44519	8.11285	9.97567	ATPase sarcoplasmic/endoplasmic reticulum Ca2+ transporting 3	chr17:3827	NM_00517	XM_01152	.	.	NP_00516	XP_011522	protein_co	ENSG0000	489	813
ATP2B1	17.0588	17.8349	17.6528	14.0742	19.1448	20.7541	ATPase plasma membrane Ca2+ transporting 1 [Source:HGNC Syr	chr12:8998	NM_00100	XM_01153	.	.	NP_00100	XP_011536	protein_co	ENSG0000	490	814
ATP2B2	0	0.008826	0.007486	0.019039	0.019788	0.01829	ATPase plasma membrane Ca2+ transporting 2 [Source:HGNC Syr	chr3:10365	NM_00100	XM_01700	.	.	NP_00100	XP_005265	protein_co	ENSG0000	491	815
ATP2B3	0	0	0.014983	0	0	0	ATPase plasma membrane Ca2+ transporting 3 [Source:HGNC Syr	chrX:15278	NM_00100	XM_01153	.	.	NP_00100	XP_005274	protein_co	ENSG0000	492	816
ATP2B4	10.5916	10.5843	12.7839	12.7267	13.0716	15.1263	ATPase plasma membrane Ca2+ transporting 4 [Source:HGNC Syr	chr1:20359	NM_00100	.	.	.	NP_00100	.	protein_co	ENSG0000	493	817
ATP2C1	30.5997	30.1753	26.0216	30.9892	35.4081	32.4617	ATPase secretory pathway Ca2+ transporting 1 [Source:HGNC Syr	chr3:13056	NM_00100	XM_01700	.	.	NP_00100	XP_005247	protein_co	ENSG0000	27032	13211
ATP2C2	4.17257	4.03971	4.04647	5.22644	5.13413	8.73691	ATPase secretory pathway Ca2+ transporting 2 [Source:HGNC Syr	chr16:8440	NM_00128	XM_01152	.	.	NP_00127	XP_011521	protein_co	ENSG0000	9914	29103
ATP4A	0.06219	0.040884	0.017762	0.022202	0.024216	0.045748	ATPase H+/K+ transporting alpha subunit [Source:HGNC Symbol;	chr19:3604	NM_00070	.	.	.	NP_00069	.	protein_co	ENSG0000	495	819
ATP4B	0	0	0	0	0	0	ATPase H+/K+ transporting beta subunit [Source:HGNC Symbol;A	chr13:1143	NM_00070	.	.	.	NP_00069	.	protein_co	ENSG0000	496	820
ATP5A1	403.499	399.918	431.233	463.026	423.464	423.437	ATP synthase, H+ transporting, mitochondrial F1 complex, alpha s	chr18:4366	NM_00100	XM_01152	.	.	NP_00100	XP_011524	protein_co	ENSG0000	498	823
ATP5B	694.638	693.619	683.818	889.417	772.031	790.152	ATP synthase, H+ transporting, mitochondrial F1 complex, beta p	chr12:5703	NM_00168	.	.	.	NP_00167	.	protein_co	ENSG0000	506	830
ATP5C1	169.463	148.621	176.781	192.106	164.175	176.048	ATP synthase, H+ transporting, mitochondrial F1 complex, gamma	chr10:7830	NM_00100	XM_01151	.	.	NP_00100	.	protein_co	ENSG0000	509	833
ATP5D	209.911	229.79	224.881	267.768	204.168	222.056	ATP synthase, H+ transporting, mitochondrial F1 complex, delta s	chr19:1241	NM_00100	.	.	.	NP_00100	.	protein_co	ENSG0000	513	837
ATP5E	89.1927	90.1656	76.0596	132.729	96.095	94.4618	ATP synthase, H+ transporting, mitochondrial F1 complex, epsilon	chr20:5759	NM_00688	.	.	.	NP_00881	.	protein_co	ENSG0000	514	838
ATP5EP2	84.1001	76.8721	90.1638	107.026	86.6413	109.161	ATP synthase, H+ transporting, mitochondrial F1 complex, epsilon	chr13:2851	.	.	.	.	.	.	protein_co	ENSG0000	.	34026
ATP5F1	212.858	183.035	194.963	218.778	202.219	192.115	ATP synthase, H+ transporting, mitochondrial Fo complex subunit	chr1:11198	NM_00168	.	.	.	NP_00167	.	protein_co	ENSG0000	515	840
ATP5G1	420.649	402.016	397.301	452.01	335.065	261.067	ATP synthase, H+ transporting, mitochondrial Fo complex subunit	chr17:4697	NM_00100	.	.	.	NP_00100	.	protein_co	ENSG0000	516	841
ATP5G2	348.054	303.667	317.689	329.834	236.993	278.833	ATP synthase, H+ transporting, mitochondrial Fo complex subunit	chr12:5405	NM_00100	.	.	.	NP_00100	XP_016874	protein_co	ENSG0000	517	842
ATP5G3	378.133	354.688	393.396	503.311	412.384	394.454	ATP synthase, H+ transporting, mitochondrial Fo complex subunit	chr2:17604	NM_00100	.	.	.	NP_00100	.	protein_co	ENSG0000	518	843
ATP5H	366.952	324.954	335.665	426.772	330.14	350.806	ATP synthase, H+ transporting, mitochondrial Fo complex subunit	chr17:7302	NM_00100	.	.	.	NP_00100	.	protein_co	ENSG0000	10476	845
ATP5I	668.323	614.635	593.215	787.582	562.2	581.133	ATP synthase, H+ transporting, mitochondrial Fo complex subunit	chr4:66622	NM_00710	.	NR_03374	.	NP_00903	.	protein_co	ENSG0000	521	846
ATP5J	186.936	175.011	192.067	207.059	189.366	206.594	ATP synthase, H+ transporting, mitochondrial Fo complex subunit	chr21:2701	NM_00100	XM_00526	.	.	NP_00100	XP_005261	protein_co	ENSG0000	522	847
ATP5J2	567.734	553.807	506.077	656.961	480.124	454.074	ATP synthase, H+ transporting, mitochondrial Fo complex subunit	chr7:98992	NM_00100	.	.	.	NP_00100	.	protein_co	ENSG0000	9551	848
ATP5J2-PTCD1	8.87905	8.09341	7.80569	8.83099	7.44455	6.43605	ATP5J2-PTCD1 readthrough [Source:HGNC Symbol;Acc:HGNC:388	chr7:98992	NM_00119	.	.	.	NP_00118	.	protein_co	ENSG0000	100526740	38844
ATP5L	108.827	99.8001	98.6503	143.701	97.6114	106.242	ATP synthase, H+ transporting, mitochondrial Fo complex subunit	chr11:1182	NM_00647	.	.	.	NP_00646	.	protein_co	ENSG0000	10632	14247
ATP5L2	11.0141	9.94418	13.9538	14.5882	11.9062	12.0382	ATP synthase, H+ transporting, mitochondrial Fo complex subunit	chr22:4301	NM_00116	.	.	.	NP_00115	.	protein_co	ENSG0000	267020	13213
ATP5O	252.646	225.607	234.179	281.947	218.144	260.298	ATP synthase, H+ transporting, mitochondrial F1 complex, O subu	chr21:3487	NM_00169	.	.	.	NP_00168	.	protein_co	ENSG0000	539	850
ATP5S	2.62398	2.34191	3.92718	2.2252	3.02289	2.96999	ATP synthase, H+ transporting, mitochondrial Fo complex subunit	chr14:5070	NM_00100	XM_01153	.	.	NP_00100	XP_005267	protein_co	ENSG0000	27109	18799
ATP5SL	39.0755	39.5676	33.4286	50.4465	38.7275	33.7577	ATP5S like [Source:HGNC Symbol;Acc:HGNC:25496]	chr19:4193	NM_00116	XM_01152	.	.	NP_00116	XP_011525	protein_co	ENSG0000	55101	25496

ATP6AP1	53.7693	54.977	48.5297	49.8483	47.6906	42.8371	ATPase H+ transporting accessory protein 1 [Source:HGNC Symbo	chrX:15365	NM_00118	XM_01153	.	.	NP_00117	XP_011529	protein_co	ENSG0000	537	868
ATP6AP1L	5.18952	7.61508	6.25065	4.09738	3.4819	5.49365	ATPase H+ transporting accessory protein 1 like [Source:HGNC Sy	chr5:81575	NM_00101	XM_01154	.	.	NP_00101	XP_011542	protein_co	ENSG0000	92270	28091
ATP6AP2	49.6227	49.1734	55.5698	48.0668	46.4327	48.7205	ATPase H+ transporting accessory protein 2 [Source:HGNC Symbo	chrX:40440	NM_00576	.	.	.	NP_00575	.	protein_co	ENSG0000	10159	18305
ATP6V0A1	21.4215	18.9304	18.5611	18.0026	16.457	15.3888	ATPase H+ transporting V0 subunit a1 [Source:HGNC Symbol;Acc:	chr17:4061	NM_00113	XM_01152	NR_04980	.	NP_00112	XP_005257	protein_co	ENSG0000	10084704	865
ATP6V0A2	5.06424	5.50504	6.40708	5.39612	6.42914	5.31911	ATPase H+ transporting V0 subunit a2 [Source:HGNC Symbol;Acc:	chr12:1241	NM_01246	XM_00525	.	.	NP_03659	.	protein_co	ENSG0000	23545	18481
ATP6V0A4	0.114608	0.026606	0.058523	0.085556	0.173153	0.105035	ATPase H+ transporting V0 subunit a4 [Source:HGNC Symbol;Acc:	chr7:13839	NM_02063	XM_00525	.	.	NP_06568	XP_005250	protein_co	ENSG0000	50617	866
ATP6V0B	178.293	169.811	128.116	197.128	151.773	112.028	ATPase H+ transporting V0 subunit b [Source:HGNC Symbol;Acc:H	chr1:44440	NM_00103	.	.	.	NP_00103	.	protein_co	ENSG0000	533	861
ATP6V0C	158.123	159.335	130.039	191.005	149.625	129.961	ATPase H+ transporting V0 subunit c [Source:HGNC Symbol;Acc:H	chr16:2525	NM_00119	.	.	.	NP_00118	.	protein_co	ENSG0000	527	855
ATP6V0D1	31.7401	33.8503	27.8785	33.599	28.1322	21.3791	ATPase H+ transporting V0 subunit d1 [Source:HGNC Symbol;Acc:	chr16:6747	NM_00469	XM_01152	.	.	NP_00468	XP_011521	protein_co	ENSG0000	9114	13724
ATP6V0D2	0	0	0	0	0	0	ATPase H+ transporting V0 subunit d2 [Source:HGNC Symbol;Acc:	chr8:87111	NM_15256	.	.	.	NP_68977	.	protein_co	ENSG0000	245972	18266
ATP6V0E1	93.1221	83.3084	91.5998	118.749	101.979	100.673	ATPase H+ transporting V0 subunit e1 [Source:HGNC Symbol;Acc:	chr5:17241	NM_00394	.	.	.	NP_00393	.	protein_co	ENSG0000	8992	863
ATP6V0E2	27.7291	25.8009	24.7475	30.8062	23.1328	16.8123	ATPase H+ transporting V0 subunit e2 [Source:HGNC Symbol;Acc:	chr7:14957	NM_00110	.	.	.	NP_00109	.	protein_co	ENSG0000	155066	21723
ATP6V1A	20.6563	20.48	25.7628	23.3089	24.8532	23.4606	ATPase H+ transporting V1 subunit A [Source:HGNC Symbol;Acc:H	chr3:11346	NM_00169	.	.	.	NP_00168	.	protein_co	ENSG0000	523	851
ATP6V1B1	0.122056	0.121394	0.140215	0.163233	0.094279	0.488278	ATPase H+ transporting V1 subunit B1 [Source:HGNC Symbol;Acc:	chr2:71163	NM_00169	.	.	.	NP_00168	.	protein_co	ENSG0000	525	853
ATP6V1B2	40.5671	38.2182	35.4371	45.6385	42.8673	42.4333	ATPase H+ transporting V1 subunit B2 [Source:HGNC Symbol;Acc	chr8:20054	NM_00169	.	.	.	NP_00168	.	protein_co	ENSG0000	526	854
ATP6V1C1	40.1118	35.707	39.2835	33.1512	39.2495	36.904	ATPase H+ transporting V1 subunit C1 [Source:HGNC Symbol;Acc:	chr8:10403	NM_00169	.	.	.	NP_00168	.	protein_co	ENSG0000	528	856
ATP6V1C2	1.16296	1.03718	2.472	1.65392	1.00721	2.1005	ATPase H+ transporting V1 subunit C2 [Source:HGNC Symbol;Acc	chr2:10861	NM_00103	XM_01151	.	.	NP_00103	XP_011508	protein_co	ENSG0000	245973	18264
ATP6V1D	25.3873	22.1	22.7304	28.0859	24.8214	26.6654	ATPase H+ transporting V1 subunit D [Source:HGNC Symbol;Acc:H	chr14:6780	NM_01599	.	.	.	NP_05707	.	protein_co	ENSG0000	51382	13527
ATP6V1E1	78.4566	73.7827	76.6233	90.7611	71.57	77.3985	ATPase H+ transporting V1 subunit E1 [Source:HGNC Symbol;Acc:	chr22:1807	NM_00103	.	.	.	NP_00103	.	protein_co	ENSG0000	529	857
ATP6V1E2	3.94153	3.49452	2.81996	2.84114	2.53888	3.184	ATPase H+ transporting V1 subunit E2 [Source:HGNC Symbol;Acc:	chr2:46738	NM_00131	XM_01153	.	.	NP_00130	XP_005264	protein_co	ENSG0000	90423	18125
ATP6V1F	165.902	151.903	162.521	194.448	144.246	120.067	ATPase H+ transporting V1 subunit F [Source:HGNC Symbol;Acc:H	chr7:12850	NM_00119	.	.	.	NP_00118	.	protein_co	ENSG0000	9296	16832
ATP6V1G1	108.397	109.656	99.5771	132.477	113.128	106.341	ATPase H+ transporting V1 subunit G1 [Source:HGNC Symbol;Acc	chr9:11735	NM_00488	.	.	.	NP_00487	.	protein_co	ENSG0000	9550	864
ATP6V1G2	1.51118	2.37733	1.15651	1.48778	1.23204	0.792001	ATPase H+ transporting V1 subunit G2 [Source:HGNC Symbol;Acc	chr6:31512	NM_00120	.	.	.	NP_00119	.	protein_co	ENSG0000	534	862
ATP6V1G3	0	0	0	0	0	0	ATPase H+ transporting V1 subunit G3 [Source:HGNC Symbol;Acc	chr1:19849	NM_00132	XM_01150	.	.	NP_00130	XP_006711	protein_co	ENSG0000	127124	18265
ATP6V1H	16.5065	15.5501	17.8106	18.787	19.5078	17.6712	ATPase H+ transporting V1 subunit H [Source:HGNC Symbol;Acc:H	chr8:54628	NM_01594	XM_00671	.	.	NP_05702	XP_006716	protein_co	ENSG0000	51606	18303
ATP7A	1.99969	2.17455	2.66404	1.75467	2.5213	3.00558	ATPase copper transporting alpha [Source:HGNC Symbol;Acc:HGN	chrX:77166	NM_00005	.	.	.	NP_00004	.	protein_co	ENSG0000	538	869
ATP7B	1.79915	1.82907	3.3292	1.58495	2.04672	2.46076	ATPase copper transporting beta [Source:HGNC Symbol;Acc:HGN	chr13:5250	NM_00005	XM_01702	.	.	NP_00004	XP_005266	protein_co	ENSG0000	540	870
ATP8A1	0.232937	0.308927	0.385739	0.313328	0.442269	0.57964	ATPase phospholipid transporting 8A1 [Source:HGNC Symbol;Acc	chr4:42410	NM_00110	XM_01700	.	.	NP_00109	XP_005248	protein_co	ENSG0000	10396	13531
ATP8A2	0	0	0.018226	0.047349	0.023867	0.037645	ATPase phospholipid transporting 8A2 [Source:HGNC Symbol;Acc	chr13:2594	NM_01652	XM_01153	.	.	NP_05761	XP_005266	protein_co	ENSG0000	51761	13533
ATP8B1	4.22816	3.89302	3.98853	4.43431	4.71857	4.63213	ATPase phospholipid transporting 8B1 [Source:HGNC Symbol;Acc	chr18:5531	NM_00560	XM_01152	.	.	NP_00559	XP_006722	protein_co	ENSG0000	5205	3706
ATP8B2	11.6864	12.9646	11.1684	9.42571	9.61931	8.23725	ATPase phospholipid transporting 8B2 [Source:HGNC Symbol;Acc	chr1:15429	NM_00100	XM_01700	.	.	NP_00100	XP_005245	protein_co	ENSG0000	57198	13534
ATP8B3	3.91413	3.99832	4.79809	3.19541	3.22134	3.27697	ATPase phospholipid transporting 8B3 [Source:HGNC Symbol;Acc	chr19:1782	NM_00117	XM_01152	.	.	NP_00117	XP_006722	protein_co	ENSG0000	148229	13535
ATP8B4	0.095226	0.083544	0.059158	0.089073	0.105332	0.092698	ATPase phospholipid transporting 8B4 (putative) [Source:HGNC S	chr15:5015	NM_02483	XM_01702	.	.	NP_07911	XP_011520	protein_co	ENSG0000	79895	13536
ATP9A	6.76426	7.42689	9.06621	6.37716	7.49776	7.80606	ATPase phospholipid transporting 9A (putative) [Source:HGNC Sy	chr20:5021	NM_00604	XM_01152	.	.	NP_00603	XP_011526	protein_co	ENSG0000	10079	13540
ATP9B	2.91401	2.58269	2.57059	1.96361	2.48999	2.38452	ATPase phospholipid transporting 9B (putative) [Source:HGNC Sy	chr18:7682	NM_00130	XM_01152	.	.	NP_00129	XP_005266	protein_co	ENSG0000	374868	13541
ATPAF1	23.9126	21.8102	25.5811	29.0847	26.283	25.4785	ATP synthase mitochondrial F1 complex assembly factor 1 [Sourc	chr1:47098	NM_00104	.	.	.	NP_00103	.	protein_co	ENSG0000	64756	18803
ATPAF2	9.4941	9.74273	9.45477	13.3402	9.75374	9.27651	ATP synthase mitochondrial F1 complex assembly factor 2 [Sourc	chr17:1787	NM_14569	.	.	.	NP_66372	.	protein_co	ENSG0000	91647	18802
ATPIF1	236.18	228.051	189.472	237.209	188.07	211.187	ATPase inhibitory factor 1 [Source:HGNC Symbol;Acc:HGNC:871]	chr1:28562	NM_01631	.	.	.	NP_05739	.	protein_co	ENSG0000	93974	871
ATR	6.66315	6.01092	7.9946	5.37673	7.22578	6.7489	ATR serine/threonine kinase [Source:HGNC Symbol;Acc:HGNC:88	chr3:14216	NM_00118	XM_01151	.	.	NP_00117	XP_011511	protein_co	ENSG0000	545	882
ATRAID	102.648	97.6892	94.8333	115.695	80.994	99.3317	all-trans retinoic acid induced differentiation factor [Source:HGN	chr2:27422	NM_00117	.	.	.	NP_00116	.	protein_co	ENSG0000	51374	24090
ATRIP	10.2438	10.474	8.89864	11.2981	9.84151	6.29988	ATR interacting protein [Source:HGNC Symbol;Acc:HGNC:33499]	chr3:48488	NM_00127	.	.	.	NP_00125	.	protein_co	ENSG0000	84126	33499
ATRN	9.1965	9.67484	9.96448	8.99066	10.173	9.32241	attractin [Source:HGNC Symbol;Acc:HGNC:885]	chr20:3451	NM_00120	XM_01152	.	.	NP_00119	.	protein_co	ENSG0000	8455	885
ATRNL1	0.535805	0.471222	0.447817	0.148879	0.812019	0.557568	attractin like 1 [Source:HGNC Symbol;Acc:HGNC:29063]	chr10:1168	NM_00127	XM_01153	.	.	NP_00126	XP_011537	protein_co	ENSG0000	26033	29063
ATRX	6.61595	6.19906	9.38104	5.37036	7.9279	9.24043	ATRX, chromatin remodeler [Source:HGNC Symbol;Acc:HGNC:886	chrX:76760	NM_00048	XM_01702	.	.	NP_00048	XP_005262	protein_co	ENSG0000	546	886
ATXN1	3.74854	3.71752	3.96532	2.7907	3.66369	5.75635	ataxin 1 [Source:HGNC Symbol;Acc:HGNC:10548]	chr6:16299	NM_00033	.	.	.	NP_00032	.	protein_co	ENSG0000	6310	10548
ATXN10	37.4395	36.1927	40.6925	48.3911	42.4058	44.7604	ataxin 10 [Source:HGNC Symbol;Acc:HGNC:10549]	chr22:4606	NM_00116	.	.	.	NP_00116	.	protein_co	ENSG0000	25814	10549
ATXN1L	10.6421	10.631	11.0553	9.90414	10.9227	9.58607	ataxin 1 like [Source:HGNC Symbol;Acc:HGNC:33279]	chr16:7187	NM_00113	.	.	.	NP_00113	.	protein_co	ENSG0000	342371	33279
ATXN2	8.57051	9.38954	9.93736	8.65428	9.5453	9.05555	ataxin 2 [Source:HGNC Symbol;Acc:HGNC:10555]	chr12:1118	NM_00297	.	.	.	NP_00129	.	protein_co	ENSG0000	6311	10555
ATXN2L	43.3446	45.8117	35.499	38.3724	35.6564	41.203	ataxin 2 like [Source:HGNC Symbol;Acc:HGNC:31326]	chr16:2883	NM_00130	XM_01154	.	.	NP_00129	XP_005255	protein_co	ENSG0000	11273	31326
ATXN3	9.50076	8.22071	10.4728	8.13492	6.8343	12.3096	ataxin 3 [Source:HGNC Symbol;Acc:HGNC:7106]	chr14:9243	NM_00112	.	.	.	NP_00112	.	protein_co	ENSG0000	4287	7106
ATXN3L	0	0	0	0	0.037584	0	ataxin 3 like [Source:HGNC Symbol;Acc:HGNC:24173]	chrX:13336	NM_00113	.	.	.	NP_00112	.	protein_co	ENSG0000	92552	24173
ATXN7	8.221	8.64576	10.1256	6.40753	7.04236	8.66692	ataxin 7 [Source:HGNC Symbol;Acc:HGNC:10560]	chr3:63850	NM_00033	.	.	.	NP_00032	.	protein_co	ENSG0000	6314	10560
ATXN7L1	2.04824	2.61212	2.55713	1.9696	2.70144	2.28324	ataxin 7 like 1 [Source:HGNC Symbol;Acc:HGNC:22210]	chr7:10524	NM_00131	XM_01151	.	.	NP_00130	XP_005250	protein_co	ENSG0000	222255	22210
ATXN7L2	4.00245	3.75165	3.26249	4.80737	4.08842	2.06323	ataxin 7 like 2 [Source:HGNC Symbol;Acc:HGNC:28713]	chr1:11002	NM_15334	XM_00527	.	.	NP_69917	XP_005270	protein_co	ENSG0000	127002	28713
ATXN7L3	30.4372	32.5205	29.9647	27.2695	24.656	24.8563	ataxin 7 like 3 [Source:HGNC Symbol;Acc:HGNC:25416]	chr17:4226	NM_00109	XM_01702	.	.	NP_00109	XP_011523	protein_co	ENSG0000	56970	25416
ATXN7L3B	26.2146	27.5179	24.1816	24.144	25.0396	23.1285	ataxin 7 like 3B [Source:HGNC Symbol;Acc:HGNC:37931]	chr12:7493	NM_00113	.	.	.	NP_00112	.	protein_co	ENSG0000	552889	37931
AUH	11.1992	11.7959	14.535	7.83596	8.07181	11.43	AU RNA binding methylglutaconyl-CoA hydratase [Source:HGNC S	chr9:93976	NM_00130	XM_01151	.	.	NP_00129	XP_005252	protein_co	ENSG0000	549	890
AUNIP	11.8812	12.0038	10.7951	15.2828	12.9837	14.523	aurora kinase A and ninein interacting protein [Source:HGNC Sym	chr1:26146	NM_00128	.	.	.	NP_00127	XP_016857	protein_co	ENSG0000	79000	28363
AUP1	97.8151	83.3561	81.6617	93.7442	78.4833	58.8735	ancient ubiquitous protein 1 [Source:HGNC Symbol;Acc:HGNC:89	chr2:74753	NM_18157	XM_00526	.	.	NP_85355	XP_005264	protein_co	ENSG0000	550	891

AURKA	38.9772	38.4176	37.856	73.3845	72.8729	48.8193	aurora kinase A [Source:HGNC Symbol;Acc:HGNC:11393]	chr20:5494	NM_00132	XM_01702	.	.	NP_00131	XP_00526	protein_co	ENSG0000	6790	11393
AURKAIP1	154.638	150.833	136.453	198.391	156.634	119.861	aurora kinase A interacting protein 1 [Source:HGNC Symbol;Acc:H	chr1:13091	NM_00112	.	.	.	NP_00112	.	protein_co	ENSG0000	54998	24114
AURKB	50.0445	51.0639	47.7779	75.4614	61.6509	55.2782	aurora kinase B [Source:HGNC Symbol;Acc:HGNC:11390]	chr17:8108	NM_00125	XM_01702	.	.	NP_00124	XP_011522	protein_co	ENSG0000	9212	11390
AURKC	0.716837	0.229296	0.565911	0.361836	0.255811	0.474948	aurora kinase C [Source:HGNC Symbol;Acc:HGNC:11391]	chr19:5774	NM_00101	.	.	.	NP_00101	.	protein_co	ENSG0000	6795	11391
AUTS2	0	0	0	0	0	0	autism susceptibility candidate 2 [Source:HGNC Symbol;Acc:HGN	chr7:69063	NM_00112	XM_01151	.	.	NP_00112	XP_00525	protein_co	ENSG0000	26053	14262
AVEN	8.76558	8.69363	6.14079	8.56799	8.3562	6.56475	apoptosis and caspase activation inhibitor [Source:HGNC Symbol;	chr15:3415	NM_02037	.	.	.	NP_06510	.	protein_co	ENSG0000	57099	13509
AVIL	0.11931	0.244329	0.121326	0.043803	0.022558	0.10401	advillin [Source:HGNC Symbol;Acc:HGNC:14188]	chr12:5817	NM_00657	XM_01153	.	.	NP_00656	XP_016874	protein_co	ENSG0000	10677	14188
AVL9	18.742	18.6006	17.6232	16.4139	18.6156	23.7372	AVL9 cell migration associated [Source:HGNC Symbol;Acc:HGNC:2	chr7:32535	NM_01506	XM_01151	.	.	NP_05587	XP_005249	protein_co	ENSG0000	23080	28994
AVP	0	0	0	0	0	0	arginine vasopressin [Source:HGNC Symbol;Acc:HGNC:894]	chr20:3063	NM_00049	XM_01152	.	.	NP_00048	XP_011527	protein_co	ENSG0000	551	894
AVPI1	12.1704	12.931	11.5684	12.5523	10.4398	8.83381	arginine vasopressin induced 1 [Source:HGNC Symbol;Acc:HGNC:	chr10:9943	NM_02173	XM_01701	.	.	NP_06837	XP_016871	protein_co	ENSG0000	60370	30898
AVPR1A	0	0	0.014082	0	0	0.015848	arginine vasopressin receptor 1A [Source:HGNC Symbol;Acc:HGN	chr12:6353	NM_00070	XM_00526	.	.	NP_00069	.	protein_co	ENSG0000	552	895
AVPR1B	0	0	0	0	0	0	arginine vasopressin receptor 1B [Source:HGNC Symbol;Acc:HGN	chr1:20622	NM_00070	.	.	.	NP_00069	.	protein_co	ENSG0000	553	896
AVPR2	0	0	0	0	0	0	arginine vasopressin receptor 2 [Source:HGNC Symbol;Acc:HGNC	chrX:15317	NM_00005	XM_00672	.	.	NP_00004	XP_006724	protein_co	ENSG0000	554	897
AWAT1	0	0	0	0	0	0	acyl-CoA wax alcohol acyltransferase 1 [Source:HGNC Symbol;Acc	chrX:69454	NM_00101	.	.	.	NP_00101	.	protein_co	ENSG0000	158833	23252
AWAT2	0	0	0.035718	0	0.046812	0	acyl-CoA wax alcohol acyltransferase 2 [Source:HGNC Symbol;Acc	chrX:69260	NM_00100	XM_01153	.	.	NP_00100	XP_011529	protein_co	ENSG0000	158835	23251
AXDND1	0.276397	0.388899	0.325993	0.237964	0.3439	0.42234	axonemal dynein light chain domain containing 1 [Source:HGNC S	chr1:17933	NM_14469	XM_01150	.	.	NP_65329	XP_011507	protein_co	ENSG0000	126859	26564
AXIN1	8.89504	8.73186	8.8002	8.82123	7.64838	7.63966	axin 1 [Source:HGNC Symbol;Acc:HGNC:903]	chr16:3374	NM_00350	XM_01702	.	.	NP_00349	XP_01152	protein_co	ENSG0000	8312	903
AXIN2	12.5041	13.7631	16.0015	17.7636	18.1038	15.1475	axin 2 [Source:HGNC Symbol;Acc:HGNC:904]	chr17:6352	NM_00465	XM_01702	.	.	NP_00464	XP_011523	protein_co	ENSG0000	8313	904
AXL	16.0319	17.1184	13.6973	7.43896	8.0689	7.81078	AXL receptor tyrosine kinase [Source:HGNC Symbol;Acc:HGNC:90	chr19:4172	NM_00127	.	.	.	NP_00126	.	protein_co	ENSG0000	558	905
AZGP1	0.093048	0.286114	0.408292	0.563318	0.474833	0.438986	alpha-2-glycoprotein 1, zinc-binding [Source:HGNC Symbol;Acc:H	chr7:99564	NM_00118	.	.	.	NP_00117	.	protein_co	ENSG0000	563	910
AZI2	14.0743	15.6931	10.3947	15.7617	16.0039	16.6828	5-azacytidine induced 2 [Source:HGNC Symbol;Acc:HGNC:24002]	chr3:28283	NM_00113	XM_01153	.	.	NP_00112	XP_005265	protein_co	ENSG0000	64343	24002
AZIN1	80.4252	83.3801	77.1849	74.4185	91.7636	88.7556	antizyme inhibitor 1 [Source:HGNC Symbol;Acc:HGNC:16432]	chr8:10383	NM_00130	XM_01151	.	.	NP_00128	XP_005251	protein_co	ENSG0000	51582	16432
AZIN2	2.79786	2.30971	3.1704	1.8178	1.35832	1.82778	antizyme inhibitor 2 [Source:HGNC Symbol;Acc:HGNC:29957]	chr1:33546	NM_00129	XM_01154	.	.	NP_00128	XP_00527	protein_co	ENSG0000	113451	29957
AZU1	0.119722	0	0	0	0.062612	0.134052	azurocidin 1 [Source:HGNC Symbol;Acc:HGNC:913]	chr19:8250	NM_00170	.	.	.	NP_00169	.	protein_co	ENSG0000	566	913
B2M	626.366	583.982	434.479	472.957	416.396	463.582	beta-2-microglobulin [Source:HGNC Symbol;Acc:HGNC:914]	chr15:4500	NM_00404	.	.	.	NP_00403	XP_005254	protein_co	ENSG0000	567	914
B3GALNT1	7.26225	8.69457	5.56629	7.44826	6.7243	6.20566	beta-1,3-N-acetylgalactosaminyltransferase 1 (globoside blood gr	chr3:16080	NM_00103	XM_01700	.	.	NP_00103	XP_005247	protein_co	ENSG0000	8706	918
B3GALNT2	15.1321	14.6842	15.7252	14.6793	16.8411	14.8358	beta-1,3-N-acetylgalactosaminyltransferase 2 [Source:HGNC Sym	chr1:23561	NM_00127	XM_00671	.	.	NP_00126	XP_006711	protein_co	ENSG0000	148789	28596
B3GALT1	0.407672	0.493869	0.588857	0.311598	0.300432	0.477147	beta-1,3-galactosyltransferase 1 [Source:HGNC Symbol;Acc:HGN	chr2:16872	NM_02098	XM_01151	.	.	NP_06619	XP_005246	protein_co	ENSG0000	8708	916
B3GALT2	0	0	0.021083	0	0	0	beta-1,3-galactosyltransferase 2 [Source:HGNC Symbol;Acc:HGN	chr1:19309	NM_00378	.	.	.	NP_00377	.	protein_co	ENSG0000	8707	917
B3GALT4	0.700023	0.567853	0.550491	0.434103	0.136296	0.325607	beta-1,3-galactosyltransferase 4 [Source:HGNC Symbol;Acc:HGN	chr6:33244	NM_00378	.	.	.	NP_00377	.	protein_co	ENSG0000	8705	919
B3GALT5	0.635643	0.553546	0.559361	1.04683	1.62745	1.39915	beta-1,3-galactosyltransferase 5 [Source:HGNC Symbol;Acc:HGN	chr21:4092	NM_00127	.	.	.	NP_00126	XP_016883	protein_co	ENSG0000	10317	920
B3GALT6	9.48449	10.5957	10.1342	6.8167	6.15926	6.04685	beta-1,3-galactosyltransferase 6 [Source:HGNC Symbol;Acc:HGN	chr1:11676	NM_08060	.	.	.	NP_54217	.	protein_co	ENSG0000	126792	17978
B3GAT1	0	0	0	0	0	0.013728	beta-1,3-glucuronyltransferase 1 [Source:HGNC Symbol;Acc:HGN	chr11:1342	NM_01864	XM_01154	.	.	NP_06111	XP_005271	protein_co	ENSG0000	27087	921
B3GAT2	0.099287	0.035705	0.014349	0.09667	0.073049	0.063765	beta-1,3-glucuronyltransferase 2 [Source:HGNC Symbol;Acc:HGN	chr6:71377	NM_08074	.	.	.	NP_54278	.	protein_co	ENSG0000	135152	922
B3GAT3	34.2756	34.4269	32.9773	38.9347	35.9753	29.4157	beta-1,3-glucuronyltransferase 3 [Source:HGNC Symbol;Acc:HGN	chr11:6238	NM_00128	.	.	.	NP_00127	.	protein_co	ENSG0000	26229	923
B3GLCT	2.72797	2.98822	2.61084	2.61744	3.27374	3.93749	beta 3-glucosyltransferase [Source:HGNC Symbol;Acc:HGNC:2020	chr13:3177	NM_19431	XM_01702	.	.	NP_91929	XP_006719	protein_co	ENSG0000	145173	20207
B3GNT2	17.4536	17.8005	18.4037	18.4796	22.1896	23.1159	UDP-GlcNAc:betaGal beta-1,3-N-acetylglucosaminyltransferase 2	chr2:62423	NM_00131	XM_01153	.	.	NP_00130	XP_01153	protein_co	ENSG0000	10678	15629
B3GNT3	11.2597	12.1975	9.3755	11.2464	8.88374	8.89271	UDP-GlcNAc:betaGal beta-1,3-N-acetylglucosaminyltransferase 3	chr19:1790	NM_01425	XM_01152	.	.	NP_05507	XP_011525	protein_co	ENSG0000	10331	13528
B3GNT4	4.61415	2.89693	4.39903	4.12885	3.12878	2.31999	UDP-GlcNAc:betaGal beta-1,3-N-acetylglucosaminyltransferase 4	chr12:1226	NM_03076	XM_01153	.	.	NP_11039	XP_011537	protein_co	ENSG0000	79369	15683
B3GNT5	13.8964	14.3758	13.079	13.1923	17.6652	18.2509	UDP-GlcNAc:betaGal beta-1,3-N-acetylglucosaminyltransferase 5	chr3:18289	NM_03204	XM_01151	.	.	NP_11443	XP_005247	protein_co	ENSG0000	84002	15684
B3GNT6	0.041179	0.040121	0	0	0	0.04568	UDP-GlcNAc:betaGal beta-1,3-N-acetylglucosaminyltransferase 6	chr11:7674	NM_13870	.	.	.	NP_61965	.	protein_co	ENSG0000	192134	24141
B3GNT7	3.57994	3.49607	3.90245	3.4068	2.65273	2.93336	UDP-GlcNAc:betaGal beta-1,3-N-acetylglucosaminyltransferase 7	chr2:23226	NM_14523	XM_00524	.	.	NP_66027	XP_005247	protein_co	ENSG0000	93010	18811
B3GNT8	0.057134	0	0.048639	0.060559	0.066551	0.064526	UDP-GlcNAc:betaGal beta-1,3-N-acetylglucosaminyltransferase 8	chr19:4193	NM_19854	XM_01152	.	.	NP_94094	XP_011525	protein_co	ENSG0000	374907	24139
B3GNT9	1.97743	1.7047	1.8401	1.82406	0.929471	1.67913	UDP-GlcNAc:betaGal beta-1,3-N-acetylglucosaminyltransferase 9	chr16:6714	NM_03330	.	.	.	NP_17160	.	protein_co	ENSG0000	84752	28714
B3GNTL1	7.73238	7.48995	6.54228	7.76182	5.10235	6.64939	UDP-GlcNAc:betaGal beta-1,3-N-acetylglucosaminyltransferase-li	chr17:8070	NM_00100	XM_01152	.	.	NP_00100	XP_006722	protein_co	ENSG0000	146712	21727
B4GALNT1	14.8996	14.5761	12.8225	12.5726	11.4151	11.759	beta-1,4-N-acetyl-galactosaminyltransferase 1 [Source:HGNC Sym	chr12:5801	NM_00127	XM_01153	.	.	NP_00126	XP_005268	protein_co	ENSG0000	2583	4117
B4GALNT2	0	0.009464	0	0	0	0	beta-1,4-N-acetyl-galactosaminyltransferase 2 [Source:HGNC Sym	chr17:4720	NM_00115	.	.	.	NP_00115	XP_016879	protein_co	ENSG0000	124872	24136
B4GALNT3	3.96276	4.40642	5.33174	3.21461	2.92519	3.77075	beta-1,4-N-acetyl-galactosaminyltransferase 3 [Source:HGNC Sym	chr12:5695	NM_17359	.	.	.	NP_77586	.	protein_co	ENSG0000	283358	24137
B4GALNT4	9.63186	8.44496	10.5441	8.73284	8.45739	5.35769	beta-1,4-N-acetyl-galactosaminyltransferase 4 [Source:HGNC Sym	chr11:3698	NM_17853	XM_00525	.	.	NP_84863	XP_016873	protein_co	ENSG0000	338707	26315
B4GALT1	24.0412	25.5396	21.3906	18.7056	19.6507	23.3227	beta-1,4-galactosyltransferase 1 [Source:HGNC Symbol;Acc:HGN	chr9:33104	NM_00149	XM_00525	.	.	NP_00148	XP_005251	protein_co	ENSG0000	2683	924
B4GALT2	56.5482	56.9953	48.964	64.9816	54.5726	46.3034	beta-1,4-galactosyltransferase 2 [Source:HGNC Symbol;Acc:HGN	chr1:44444	NM_00100	XM_01700	.	.	NP_00100	XP_016858	protein_co	ENSG0000	8704	925
B4GALT3	32.4521	32.658	27.8267	31.961	28.0447	27.2872	beta-1,4-galactosyltransferase 3 [Source:HGNC Symbol;Acc:HGN	chr1:16113	NM_00119	XM_01151	.	.	NP_00118	XP_005245	protein_co	ENSG0000	8703	926
B4GALT4	9.26553	9.3759	7.05963	8.29584	8.83755	9.25098	beta-1,4-galactosyltransferase 4 [Source:HGNC Symbol;Acc:HGN	chr3:11893	NM_00377	XM_01151	.	.	NP_00376	XP_005247	protein_co	ENSG0000	8702	927
B4GALT5	113.759	116.339	82.1557	115.539	120.847	127.632	beta-1,4-galactosyltransferase 5 [Source:HGNC Symbol;Acc:HGN	chr20:4824	NM_00477	.	.	.	NP_00476	.	protein_co	ENSG0000	9334	928
B4GALT6	2.89074	2.97772	4.71197	2.50165	3.07932	3.81959	beta-1,4-galactosyltransferase 6 [Source:HGNC Symbol;Acc:HGN	chr18:2920	NM_00477	XM_00672	.	.	NP_00476	XP_005258	protein_co	ENSG0000	9331	929
B4GALT7	15.9493	17.0394	13.4834	12.2558	9.02472	8.4446	beta-1,4-galactosyltransferase 7 [Source:HGNC Symbol;Acc:HGN	chr5:17702	NM_00725	XM_00671	.	.	NP_00918	XP_006714	protein_co	ENSG0000	11285	930
B4GAT1	6.74037	6.62545	7.1236	8.16576	7.83582	6.10082	beta-1,4-glucuronyltransferase 1 [Source:HGNC Symbol;Acc:HGN	chr11:6611	NM_00687	.	.	.	NP_00686	.	protein_co	ENSG0000	11041	15685
B9D1	13.8565	12.3431	11.4622	10.9403	6.25755	6.40834	B9 domain containing 1 [Source:HGNC Symbol;Acc:HGNC:24123]	chr17:1924	NM_00124	XM_01152	.	.	NP_00123	XP_005256	protein_co	ENSG0000	27077	24123
B9D2	3.82989	4.6545	3.19977	4.95613	4.21069	3.45149	B9 protein domain 2 [Source:HGNC Symbol;Acc:HGNC:28636]	chr19:4183	NM_03057	XM_01152	.	.	NP_08505	XP_011525	protein_co	ENSG0000	80776	28636

BAALC	0	0	0	0	0	0	brain and acute leukemia, cytoplasmic [Source:HGNC Symbol;Acc	chr8:10415	NM_00102	.	.	.	NP_00101	.	protein_co	ENSG0000	79870	14333
BAAT	0.048819	0.130824	0.060959	0.079427	0.133261	0.034285	bile acid-CoA:amino acid N-acyltransferase [Source:HGNC Symbo	chr9:10412	NM_00112	.	.	.	NP_00112	.	protein_co	ENSG0000	570	932
BABAM1	53.1705	55.7541	41.9929	61.0344	46.9571	34.1364	BRISC and BRCA1 A complex member 1 [Source:HGNC Symbol;Ac	chr19:1737	NM_00103	.	.	.	NP_00102	.	protein_co	ENSG0000	29086	25008
BACE1	3.45068	3.87273	3.84649	5.01834	4.45587	5.33946	beta-secretase 1 [Source:HGNC Symbol;Acc:HGNC:933]	chr11:1171	NM_00120	.	.	.	NP_00119	.	protein_co	ENSG0000	23621	933
BACE2	12.6638	10.6274	10.7256	11.411	9.9274	8.94679	beta-site APP-cleaving enzyme 2 [Source:HGNC Symbol;Acc:HGN	chr21:4253	NM_01210	.	.	.	NP_03623	.	protein_co	ENSG0000	25825	934
BACH1	7.70432	7.75965	7.48019	5.24771	6.30901	7.36381	BTB domain and CNC homolog 1 [Source:HGNC Symbol;Acc:HGN	chr21:3067	NM_00118	.	NR_03336	.	NP_00117	.	protein_co	ENSG0000	100379661	935
BACH2	0.801111	0.851676	0.939613	0.317528	0.310137	0.744786	BTB domain and CNC homolog 2 [Source:HGNC Symbol;Acc:HGN	chr6:90636	NM_00117	XM_01153	.	.	NP_00116	XP_005248	protein_co	ENSG0000	60468	14078
BAD	44.5747	40.1724	38.2848	56.6744	37.0826	27.5961	BCL2 associated agonist of cell death [Source:HGNC Symbol;Acc:H	chr11:6403	NM_00432	.	.	.	NP_00431	.	protein_co	ENSG0000	572	936
BAG1	31.7273	32.1555	27.48	31.7859	26.4578	23.8485	BCL2 associated athanogene 1 [Source:HGNC Symbol;Acc:HGNC:	chr9:33252	NM_00117	.	.	.	NP_00116	.	protein_co	ENSG0000	573	937
BAG2	11.9665	12.3123	9.74442	15.0132	15.0748	10.6669	BCL2 associated athanogene 2 [Source:HGNC Symbol;Acc:HGNC:	chr6:57037	NM_00428	.	.	.	NP_00427	.	protein_co	ENSG0000	9532	938
BAG3	43.5465	49.3094	36.2152	62.8898	53.7779	32.6104	BCL2 associated athanogene 3 [Source:HGNC Symbol;Acc:HGNC:	chr10:1214	NM_00428	XM_00527	.	.	NP_00427	XP_005270	protein_co	ENSG0000	9531	939
BAG4	13.4401	13.6658	13.483	15.4639	17.3522	13.9752	BCL2 associated athanogene 4 [Source:HGNC Symbol;Acc:HGNC:	chr8:38020	NM_00120	.	.	.	NP_00119	.	protein_co	ENSG0000	9530	940
BAG5	18.1974	18.0362	16.0849	16.8693	19.3271	15.8528	BCL2 associated athanogene 5 [Source:HGNC Symbol;Acc:HGNC:	chr14:1040	NM_00101	.	.	.	NP_00101	.	protein_co	ENSG0000	9529	941
BAG6	65.2294	70.0747	64.4237	67.9808	61.8234	66.2858	BCL2 associated athanogene 6 [Source:HGNC Symbol;Acc:HGNC:	chr6:31606	NM_00109	XM_01151	.	.	NP_00109	XP_011513	protein_co	ENSG0000	7917	13919
BAHCC1	6.63576	7.11633	7.56836	5.4964	5.71048	6.14452	BAH domain and coiled-coil containing 1 [Source:HGNC Symbol;A	chr17:7936	NM_00129	XM_01702	.	.	NP_00127	XP_011523	protein_co	ENSG0000	57597	29279
BAHD1	14.978	15.9229	14.842	18.2407	15.5523	14.9584	bromo adjacent homology domain containing 1 [Source:HGNC Sy	chr15:4073	NM_00130	XM_01152	.	.	NP_00128	XP_011519	protein_co	ENSG0000	22893	29153
BAIAP2	34.5403	37.3718	28.7975	36.4243	31.0863	23.4956	BAI1 associated protein 2 [Source:HGNC Symbol;Acc:HGNC:947]	chr17:7900	NM_00114	XM_01152	.	.	NP_00113	XP_005257	protein_co	ENSG0000	10458	947
BAIAP2L1	20.7522	22.2513	19.7439	20.9062	18.5625	20.06	BAI1 associated protein 2 like 1 [Source:HGNC Symbol;Acc:HGNC	chr7:97910	NM_01884	.	.	.	NP_06133	.	protein_co	ENSG0000	55971	21649
BAIAP2L2	0.327673	0.518651	0.253099	0.348706	0.335661	0.599751	BAI1 associated protein 2 like 2 [Source:HGNC Symbol;Acc:HGNC	chr22:3848	NM_02504	XM_01153	.	.	NP_07932	XP_005261	protein_co	ENSG0000	80115	26203
BAIAP3	5.43828	5.30014	9.41573	5.55124	5.32375	7.102	BAI1 associated protein 3 [Source:HGNC Symbol;Acc:HGNC:948]	chr16:1383	NM_00119	XM_01152	.	.	NP_00118	XP_011521	protein_co	ENSG0000	8938	948
BAK1	18.448	16.9129	16.2958	20.2768	16.8714	13.6925	BCL2 antagonist/killer 1 [Source:HGNC Symbol;Acc:HGNC:949]	chr6:33540	NM_00118	XM_01151	.	.	NP_00117	XP_011513	protein_co	ENSG0000	578	949
BAMBI	18.2578	22.3931	15.4693	16.5581	15.7017	16.5287	BMP and activin membrane bound inhibitor [Source:HGNC Symb	chr10:2896	NM_01234	.	.	.	NP_03647	.	protein_co	ENSG0000	25805	30251
BANF1	233.519	227.16	182.218	278.474	221.781	198.674	barrier to autointegration factor 1 [Source:HGNC Symbol;Acc:HG	chr11:6576	NM_00114	XM_01701	.	.	NP_00113	XP_016874	protein_co	ENSG0000	8815	17397
BANF2	0	0	0	0	0	0	barrier to autointegration factor 2 [Source:HGNC Symbol;Acc:HG	chr20:1767	NM_00101	XM_00526	.	.	NP_00101	XP_005260	protein_co	ENSG0000	140836	16172
BANK1	1.03947	1.35724	1.09525	2.1286	2.00464	2.43322	B-cell scaffold protein with ankyrin repeats 1 [Source:HGNC Symb	chr4:10234	NM_00108	.	.	.	NP_00107	XP_016863	protein_co	ENSG0000	55024	18233
BANP	7.85114	8.13314	5.83258	8.83213	7.66657	5.62485	BTG3 associated nuclear protein [Source:HGNC Symbol;Acc:HGN	chr16:8798	NM_00117	XM_01702	.	.	NP_00116	XP_005256	protein_co	ENSG0000	54971	13450
BAP1	23.063	23.3533	22.6009	23.5362	23.3372	21.5146	BRCA1 associated protein 1 [Source:HGNC Symbol;Acc:HGNC:950	chr3:52435	NM_00465	XM_01700	.	.	NP_00464	XP_011532	protein_co	ENSG0000	8314	950
BARD1	6.27302	6.69615	5.16545	10.1096	9.56656	8.27867	BRCA1 associated RING domain 1 [Source:HGNC Symbol;Acc:HGN	chr2:21559	NM_00046	XM_01700	.	.	NP_00045	XP_016860	protein_co	ENSG0000	580	952
BARHL1	0	0	0	0	0	0	BarH like homeobox 1 [Source:HGNC Symbol;Acc:HGNC:953]	chr9:13545	NM_02006	.	.	.	NP_06444	.	protein_co	ENSG0000	56751	953
BARHL2	0	0	0	0	0	0	BarH like homeobox 2 [Source:HGNC Symbol;Acc:HGNC:954]	chr1:91177	NM_02006	.	.	.	NP_06444	.	protein_co	ENSG0000	343472	954
BARX1	0	0	0	0	0	0	BARX homeobox 1 [Source:HGNC Symbol;Acc:HGNC:955]	chr9:96713	NM_02157	.	.	.	NP_06754	.	protein_co	ENSG0000	56033	955
BARX2	0.222787	0	0.154736	0.097221	0.102271	0.238238	BARX homeobox 2 [Source:HGNC Symbol;Acc:HGNC:956]	chr11:1292	NM_00365	.	.	.	NP_00364	.	protein_co	ENSG0000	8538	956
BASP1	0	0	0	0	0	0	brain abundant membrane attached signal protein 1 [Source:HGN	chr5:17216	NM_00127	.	.	.	NP_00125	.	protein_co	ENSG0000	10409	957
BATF	0	0	0	0	0	0	basic leucine zipper ATF-like transcription factor [Source:HGNC Sy	chr14:7598	NM_00639	.	.	.	NP_00639	.	protein_co	ENSG0000	10538	958
BATF2	1.59802	1.29153	1.25176	0.626874	0.805648	0.80083	basic leucine zipper ATF-like transcription factor 2 [Source:HGNC	chr11:6475	NM_00130	.	.	.	NP_00128	.	protein_co	ENSG0000	116071	25163
BATF3	4.79256	4.35702	4.30363	6.28506	5.0525	4.42238	basic leucine zipper ATF-like transcription factor 3 [Source:HGNC	chr1:21285	NM_01866	.	.	.	NP_06113	.	protein_co	ENSG0000	55509	28915
BAX	75.1806	82.1249	90.1191	51.5258	40.0617	35.2062	BCL2 associated X, apoptosis regulator [Source:HGNC Symbol;Acc	chr19:4945	NM_00129	XM_01152	.	.	NP_00127	XP_016882	protein_co	ENSG0000	581	959
BAZ1A	12.6514	13.3624	13.7164	14.7244	16.6276	13.6135	bromodomain adjacent to zinc finger domain 1A [Source:HGNC S	chr14:3522	NM_01344	XM_01153	.	.	NP_03847	XP_011534	protein_co	ENSG0000	11177	960
BAZ1B	39.8289	42.2623	34.7455	41.695	44.1983	39.2095	bromodomain adjacent to zinc finger domain 1B [Source:HGNC S	chr7:72854	NM_03240	.	.	.	NP_11578	XP_016868	protein_co	ENSG0000	9031	961
BAZ2A	21.3977	23.7791	22.7111	20.6782	22.1675	21.6458	bromodomain adjacent to zinc finger domain 2A [Source:HGNC S	chr12:5691	NM_00130	XM_01153	.	.	NP_00128	XP_011536	protein_co	ENSG0000	11176	962
BAZ2B	2.87756	2.66361	3.24129	1.27256	1.68652	2.62069	bromodomain adjacent to zinc finger domain 2B [Source:HGNC S	chr2:16017	NM_00128	XM_01151	.	.	NP_00127	XP_005246	protein_co	ENSG0000	29994	963
BBC3	33.2626	33.2396	29.4144	18.6242	17.4886	26.7254	BCL2 binding component 3 [Source:HGNC Symbol;Acc:HGNC:178	chr19:4772	NM_00112	XM_00672	NR_03615	.	NP_00112	XP_006723	protein_co	ENSG0000	100422832	17868
BBIP1	17.982	19.5335	15.3937	20.3824	19.5384	21.2391	BBSome interacting protein 1 [Source:HGNC Symbol;Acc:HGNC:2	chr10:1126	NM_00119	.	.	.	NP_00118	.	protein_co	ENSG0000	92482	28093
BBOF1	1.77217	2.12376	2.22539	1.8899	1.60556	1.92226	basal body orientation factor 1 [Source:HGNC Symbol;Acc:HGNC:	chr14:7448	NM_02505	XM_01153	.	.	NP_07933	XP_005268	protein_co	ENSG0000	80127	19855
BBOX1	0	0	0	0	0	0	gamma-butyrobetaine hydroxylase 1 [Source:HGNC Symbol;Acc:H	chr11:2706	NM_00398	XM_01152	.	.	NP_00397	XP_005253	protein_co	ENSG0000	8424	964
BBS1	5.45075	6.5758	6.39789	5.62084	4.65873	5.23072	Bardet-Biedl syndrome 1 [Source:HGNC Symbol;Acc:HGNC:966]	chr11:6624	NM_02464	.	.	.	NP_07892	.	protein_co	ENSG0000	582	966
BBS10	12.641	12.2316	13.4489	11.4125	17.4978	22.3773	Bardet-Biedl syndrome 10 [Source:HGNC Symbol;Acc:HGNC:2629	chr12:7673	NM_02468	.	.	.	NP_07896	.	protein_co	ENSG0000	79738	26291
BBS12	1.82802	1.2176	1.82331	1.1507	1.28724	2.00739	Bardet-Biedl syndrome 12 [Source:HGNC Symbol;Acc:HGNC:2664	chr4:12365	NM_00117	XM_01700	.	.	NP_00117	XP_011529	protein_co	ENSG0000	166379	26648
BBS2	11.8417	10.9075	15.3803	10.0102	9.59643	11.4973	Bardet-Biedl syndrome 2 [Source:HGNC Symbol;Acc:HGNC:967]	chr16:5651	NM_03188	XM_00525	.	.	NP_11409	XP_005256	protein_co	ENSG0000	583	967
BBS4	5.72648	6.07797	6.18164	5.91126	5.64362	5.72676	Bardet-Biedl syndrome 4 [Source:HGNC Symbol;Acc:HGNC:969]	chr15:7297	NM_00125	XM_01702	.	.	NP_00123	XP_011520	protein_co	ENSG0000	585	969
BBS5	1.83149	2.82945	2.46125	2.1272	2.16766	2.21529	Bardet-Biedl syndrome 5 [Source:HGNC Symbol;Acc:HGNC:970]	chr2:17033	NM_15238	.	.	.	NP_68959	.	protein_co	ENSG0000	129880	970
BBS7	6.79101	8.09426	8.16248	7.22229	7.75377	8.45278	Bardet-Biedl syndrome 7 [Source:HGNC Symbol;Acc:HGNC:18758	chr4:12274	NM_01819	XM_01153	.	.	NP_06066	XP_005263	protein_co	ENSG0000	55212	18758
BBS9	1.51373	1.6409	2.35927	2.14303	2.21461	2.12534	Bardet-Biedl syndrome 9 [Source:HGNC Symbol;Acc:HGNC:30000	chr7:33169	NM_00103	XM_01701	.	.	NP_00102	XP_005249	protein_co	ENSG0000	27241	30000
BBX	14.5424	16.3281	15.8716	12.4541	15.9139	17.8167	BBX, HMG-box containing [Source:HGNC Symbol;Acc:HGNC:1442	chr3:10724	NM_00114	XM_01151	.	.	NP_00113	XP_005247	protein_co	ENSG0000	56987	14422
BCAM	13.6411	14.3985	18.8792	12.5154	9.6773	12.5933	basal cell adhesion molecule (Lutheran blood group) [Source:HGN	chr19:4531	NM_00101	.	.	.	NP_00101	.	protein_co	ENSG0000	4059	6722
BCAN	0	0	0	0.096122	0.024467	0.032845	brevican [Source:HGNC Symbol;Acc:HGNC:23059]	chr1:15661	NM_02194	XM_01700	.	.	NP_06876	XP_011508	protein_co	ENSG0000	63827	23059
BCAP29	9.88804	8.47122	13.0939	8.64419	14.0293	17.0286	B-cell receptor associated protein 29 [Source:HGNC Symbol;Acc:H	chr7:10722	NM_00100	XM_01151	.	.	NP_00100	XP_006716	protein_co	ENSG0000	55973	24131
BCAP31	127.244	124.993	106.707	143.274	116.613	102.311	B-cell receptor-associated protein 31 [Source:HGNC Symbol;Acc:H	chrX:15296	NM_00113	.	.	.	NP_00113	.	protein_co	ENSG0000	10134	16695
BCAR1	19.3641	21.9112	20.2001	20.4299	18.1269	15.1878	BCAR1, Cas family scaffolding protein [Source:HGNC Symbol;Acc:	chr16:7526	NM_00117	XM_01152	.	.	NP_00116	XP_005256	protein_co	ENSG0000	9564	971

BCAR3	8.83933	9.93452	8.99339	10.0233	9.64394	7.13994	breast cancer anti-estrogen resistance 3 [Source:HGNC Symbol;A	chr1:94027	NM_00126	XM_01154	.	.	NP_00124	XP_011540	protein_co	ENSG0000	8412	973
BCAS1	0	0.03492	0.085339	0	0.043975	0.017769	breast carcinoma amplified sequence 1 [Source:HGNC Symbol;Ac	chr20:5256	NM_00131	XM_01152	.	.	NP_00130	XP_005260	protein_co	ENSG0000	8537	974
BCAS2	37.5845	35.0487	31.3505	35.0465	41.5465	41.2314	breast carcinoma amplified sequence 2 [Source:HGNC Symbol;Ac	chr1:11511	NM_00587	.	.	.	NP_00586	.	protein_co	ENSG0000	10286	975
BCAS3	5.34948	5.76346	5.91308	4.74968	4.44494	5.58034	BCAS3, microtubule associated cell migration factor [Source:HGN	chr17:5875	NM_00109	XM_01152	.	.	NP_00109	XP_005257	protein_co	ENSG0000	54828	14347
BCAS4	15.1509	15.0424	16.1687	15.7057	12.6009	13.0462	breast carcinoma amplified sequence 4 [Source:HGNC Symbol;Ac	chr20:4941	NM_00101	XM_01152	.	.	NP_00101	XP_011527	protein_co	ENSG0000	55653	14367
BCAT1	0.008869	0	0	0.051382	0	0.046317	branched chain amino acid transaminase 1 [Source:HGNC Symbo	chr12:2496	NM_00117	XM_01152	.	.	NP_00117	XP_011519	protein_co	ENSG0000	586	976
BCAT2	44.9748	44.1003	35.2759	50.8193	39.2726	31.8193	branched chain amino acid transaminase 2 [Source:HGNC Symbo	chr19:4929	NM_00116	.	.	.	NP_00115	.	protein_co	ENSG0000	587	977
BCCIP	114.174	101.968	105.683	120.087	130.777	99.1797	BRCA2 and CDKN1A interacting protein [Source:HGNC Symbol;Ac	chr10:1275	NM_01656	.	.	.	NP_05765	.	protein_co	ENSG0000	56647	978
BCDIN3D	4.97047	5.77455	5.21261	5.03294	4.42635	6.19034	BCDIN3 domain containing RNA methyltransferase [Source:HGNC	chr12:5022	NM_18170	.	.	.	NP_85905	.	protein_co	ENSG0000	144233	27050
BCHE	0	0	0	0	0	0.029062	butyrylcholinesterase [Source:HGNC Symbol;Acc:HGNC:983]	chr3:16549	NM_00005	XM_01700	.	.	NP_00004	XP_016862	protein_co	ENSG0000	590	983
BCKDHA	10.3621	10.9429	10.833	4.72077	2.8189	4.5432	branched chain keto acid dehydrogenase E1, alpha polypeptide [S	chr19:4183	NM_00070	.	.	.	NP_00070	.	protein_co	ENSG0000	593	986
BCKDHB	5.61327	6.11335	8.63334	4.83782	6.64856	7.04417	branched chain keto acid dehydrogenase E1 subunit beta [Source	chr6:80816	NM_00005	XM_01153	.	.	NP_00004	XP_011534	protein_co	ENSG0000	594	987
BCKDK	28.54	32.217	34.7186	26.5572	22.9743	22.3921	branched chain ketoacid dehydrogenase kinase [Source:HGNC Sy	chr16:3111	NM_00112	.	.	.	NP_00111	.	protein_co	ENSG0000	10295	16902
BCL10	15.4374	17.0408	13.0684	11.6931	13.1274	15.9439	B-cell CLL/lymphoma 10 [Source:HGNC Symbol;Acc:HGNC:989]	chr1:85731	NM_00392	XM_00527	.	.	NP_00130	XP_011540	protein_co	ENSG0000	8915	989
BCL11A	0.243402	0.124516	0.324408	0.270537	0.356912	0.577029	B-cell CLL/lymphoma 11A [Source:HGNC Symbol;Acc:HGNC:1322	chr2:60678	NM_01801	XM_01700	.	.	NP_06048	XP_011531	protein_co	ENSG0000	53335	13221
BCL11B	0.386317	0.429351	0.284328	0.376568	0.359779	0.480402	B-cell CLL/lymphoma 11B [Source:HGNC Symbol;Acc:HGNC:1322	chr14:9963	NM_00128	.	.	.	NP_00126	.	protein_co	ENSG0000	64919	13222
BCL2	1.2978	1.17137	0.989785	0.773967	1.17746	0.995719	BCL2, apoptosis regulator [Source:HGNC Symbol;Acc:HGNC:990]	chr18:6079	NM_00063	.	.	.	NP_00062	XP_016881	protein_co	ENSG0000	596	990
BCL2A1	0	0	0	0.099422	0	0.088762	BCL2 related protein A1 [Source:HGNC Symbol;Acc:HGNC:991]	chr15:8025	NM_00111	.	.	.	NP_00110	.	protein_co	ENSG0000	597	991
BCL2L1	87.599	91.5326	116.789	69.1716	65.603	96.5784	BCL2 like 1 [Source:HGNC Symbol;Acc:HGNC:992]	chr20:3025	NM_00119	XM_01702	.	.	NP_00118	XP_005260	protein_co	ENSG0000	598	992
BCL2L10	0	0.081923	0	0	0.091397	0	BCL2 like 10 [Source:HGNC Symbol;Acc:HGNC:993]	chr15:5240	NM_00130	XM_00525	.	.	NP_00129	XP_005254	protein_co	ENSG0000	10017	993
BCL2L11	9.12033	9.35638	9.21801	8.03369	7.47689	7.03846	BCL2 like 11 [Source:HGNC Symbol;Acc:HGNC:994]	chr2:11187	NM_00120	.	.	.	NP_00119	.	protein_co	ENSG0000	10018	994
BCL2L12	75.0997	71.3419	57.6605	105.989	74.3362	64.6086	BCL2 like 12 [Source:HGNC Symbol;Acc:HGNC:13787]	chr19:5016	NM_00104	.	.	.	NP_00103	.	protein_co	ENSG0000	83596	13787
BCL2L13	20.3172	21.0968	20.5143	21.8539	19.0628	23.1007	BCL2 like 13 [Source:HGNC Symbol;Acc:HGNC:17164]	chr22:1811	NM_00127	XM_01702	.	.	NP_00125	XP_011544	protein_co	ENSG0000	23786	17164
BCL2L14	0.035645	0	0.09375	0	0.040195	0	BCL2 like 14 [Source:HGNC Symbol;Acc:HGNC:16657]	chr12:1220	NM_03076	XM_01152	.	.	NP_11039	XP_006719	protein_co	ENSG0000	79370	16657
BCL2L15	0.288517	0.760879	0.500309	0.162245	1.1615	0.946527	BCL2 like 15 [Source:HGNC Symbol;Acc:HGNC:33624]	chr1:11442	NM_00101	.	.	.	NP_00101	.	protein_co	ENSG0000	440603	33624
BCL2L2	20.2495	19.4047	16.8694	16.1742	16.9139	15.3536	BCL2 like 2 [Source:HGNC Symbol;Acc:HGNC:995]	chr14:2377	NM_00119	.	.	.	NP_00118	.	protein_co	ENSG0000	599	995
BCL2L2-PABPN1	3.03872	4.23158	0.255307	2.15617	2.98679	0.001245	BCL2L2-PABPN1 readthrough [Source:HGNC Symbol;Acc:HGNC:4	chr14:2377	NM_00119	.	.	.	NP_00118	.	protein_co	ENSG0000	100529063	42959
BCL3	10.0842	8.90509	9.72462	7.13544	4.26591	6.34971	B-cell CLL/lymphoma 3 [Source:HGNC Symbol;Acc:HGNC:998]	chr19:4525	NM_00517	XM_01152	NR_10705	.	NP_00516	XP_011525	protein_co	ENSG0000	102465879	998
BCL6	4.67622	5.23991	7.11066	4.12549	3.6496	3.5496	B-cell CLL/lymphoma 6 [Source:HGNC Symbol;Acc:HGNC:1001]	chr3:18743	NM_00113	XM_01151	.	.	NP_00112	XP_005247	protein_co	ENSG0000	604	1001
BCL6B	0.293298	0.274852	0.210334	0.322387	0.44402	0.281041	B-cell CLL/lymphoma 6B [Source:HGNC Symbol;Acc:HGNC:1002]	chr17:6926	NM_18184	.	.	.	NP_86282	.	protein_co	ENSG0000	255877	1002
BCL7A	7.66912	8.48417	6.852	9.26162	8.77104	6.71961	BCL tumor suppressor 7A [Source:HGNC Symbol;Acc:HGNC:1004]	chr12:1224	NM_00102	.	.	.	NP_00101	.	protein_co	ENSG0000	605	1004
BCL7B	25.3763	22.4891	22.0847	24.9266	21.492	19.0888	BCL tumor suppressor 7B [Source:HGNC Symbol;Acc:HGNC:1005]	chr7:72950	NM_00119	.	NR_03668	.	NP_00118	.	protein_co	ENSG0000	9275	1005
BCL7C	33.704	33.2296	30.9077	37.3267	26.1226	28.3624	BCL tumor suppressor 7C [Source:HGNC Symbol;Acc:HGNC:1006]	chr16:3084	NM_00128	XM_01154	.	.	NP_00127	XP_011544	protein_co	ENSG0000	9274	1006
BCL9	7.70265	7.89326	7.59576	8.04545	8.20303	7.73462	B-cell CLL/lymphoma 9 [Source:HGNC Symbol;Acc:HGNC:1008]	chr1:14701	NM_00432	XM_00671	.	.	NP_00431	XP_005273	protein_co	ENSG0000	607	1008
BCL9L	23.1944	24.8102	22.1661	17.8902	20.059	17.0159	B-cell CLL/lymphoma 9-like [Source:HGNC Symbol;Acc:HGNC:236	chr11:1187	NM_18255	XM_01154	.	.	NP_87236	XP_005271	protein_co	ENSG0000	283149	23688
BCLAF1	67.3086	68.1164	48.9288	62.7679	66.7681	52.785	BCL2 associated transcription factor 1 [Source:HGNC Symbol;Acc	chr6:13657	NM_00107	XM_00526	.	.	NP_00107	XP_005267	protein_co	ENSG0000	9774	16863
BCO1	0	0.050856	0.02149	0.079535	0.084145	0.07753	beta-carotene oxygenase 1 [Source:HGNC Symbol;Acc:HGNC:138	chr16:8127	NM_01742	.	.	.	NP_05912	.	protein_co	ENSG0000	53630	13815
BCO2	1.00127	0.543276	1.46508	0.605453	0.700459	0.915351	beta-carotene oxygenase 2 [Source:HGNC Symbol;Acc:HGNC:185	chr11:1120	NM_00103	.	.	.	NP_00103	.	protein_co	ENSG0000	83875	18503
BCOR	10.6331	10.4097	10.9626	7.74427	8.26732	8.19379	BCL6 corepressor [Source:HGNC Symbol;Acc:HGNC:20893]	chrX:39909	NM_00112	XM_01154	.	.	NP_00111	XP_005272	protein_co	ENSG0000	54880	20893
BCORL1	5.98772	6.73519	6.91172	5.09793	4.51177	4.66278	BCL6 corepressor-like 1 [Source:HGNC Symbol;Acc:HGNC:25657]	chrX:12911	NM_02194	XM_01153	.	.	NP_06876	XP_005262	protein_co	ENSG0000	63035	25657
BCR	15.6682	14.7918	13.7502	17.4082	14.8669	12.9691	BCR, RhoGEF and GTPase activating protein [Source:HGNC Symbo	chr22:2352	NM_00432	.	.	.	NP_00431	.	protein_co	ENSG0000	613	1014
BCS1L	28.7102	27.6096	27.2671	33.6083	28.2975	18.1248	BCS1 homolog, ubiquinol-cytochrome c reductase complex chape	chr2:21952	NM_00107	XM_01151	.	.	NP_00107	XP_005246	protein_co	ENSG0000	617	1020
BDH1	16.048	16.0519	12.6774	20.9503	17.5041	12.0245	3-hydroxybutyrate dehydrogenase, type 1 [Source:HGNC Symbol;	chr3:19723	NM_00405	XM_01151	.	.	NP_00404	XP_005269	protein_co	ENSG0000	622	1027
BDH2	9.2688	9.08567	14.0723	12.1856	11.1159	14.8909	3-hydroxybutyrate dehydrogenase, type 2 [Source:HGNC Symbol;	chr4:10400	NM_02013	XM_01153	.	.	NP_06452	XP_005263	protein_co	ENSG0000	56898	32389
BDKRB1	2.34E-70	0	1.11E-10	0.319723	0.397344	0.520891	bradykinin receptor B1 [Source:HGNC Symbol;Acc:HGNC:1029]	chr14:9667	NM_00071	.	.	.	NP_00070	.	protein_co	ENSG0000	623	1029
BDKRB2	0.230246	0.219193	0.252673	0.452627	0.22855	0.60092	bradykinin receptor B2 [Source:HGNC Symbol;Acc:HGNC:1030]	chr14:9667	NM_00062	.	.	.	NP_00061	.	protein_co	ENSG0000	624	1030
BDNF	4.7643	4.86981	6.41599	4.47842	4.8318	4.36982	brain derived neurotrophic factor [Source:HGNC Symbol;Acc:HGN	chr11:2767	NM_00114	XM_01152	.	.	NP_00113	XP_011518	protein_co	ENSG0000	627	1033
BDP1	5.49867	5.80129	5.82326	4.20792	5.83233	5.21568	B double prime 1, subunit of RNA polymerase III transcription init	chr5:70751	NM_01842	XM_01154	.	.	NP_06089	XP_011541	protein_co	ENSG0000	55814	13652
BEAN1	0.508113	0.594451	0.574772	0.088807	0.127253	0.20166	brain expressed, associated with NEDD4, 1 [Source:HGNC Symbo	chr16:6646	NM_00113	XM_01152	.	.	NP_00112	XP_011521	protein_co	ENSG0000	146227	24160
BECN1	37.5228	39.1743	38.3678	41.5292	39.2679	32.3591	beclin 1 [Source:HGNC Symbol;Acc:HGNC:1034]	chr17:4095	NM_00131	XM_00525	.	.	NP_00130	XP_005257	protein_co	ENSG0000	8678	1034
BECN2	0	0	0	0	0	0	beclin 2 [Source:HGNC Symbol;Acc:HGNC:38606]	chr1:24212	NM_00129	.	.	.	NP_00127	.	protein_co	ENSG0000	441925	38606
BEGAIN	1.97279	2.1795	2.40753	1.6831	1.17592	2.04896	brain enriched guanylate kinase associated [Source:HGNC Symbo	chr14:1010	NM_00115	XM_01153	.	.	NP_00115	XP_005267	protein_co	ENSG0000	57596	24163
BEND2	0.017125	0	0	0	0	0	BEN domain containing 2 [Source:HGNC Symbol;Acc:HGNC:28509	chrX:18181	NM_00118	XM_01154	.	.	NP_00117	XP_011543	protein_co	ENSG0000	139105	28509
BEND3	2.79727	3.00336	3.05197	2.90739	3.38329	2.45818	BEN domain containing 3 [Source:HGNC Symbol;Acc:HGNC:23040	chr6:10738	NM_00108	XM_01701	.	.	NP_00107	XP_005267	protein_co	ENSG0000	57673	23040
BEND4	0	0	0	0	0	0	BEN domain containing 4 [Source:HGNC Symbol;Acc:HGNC:23815	chr4:42112	NM_00115	XM_01700	.	.	NP_00115	XP_016863	protein_co	ENSG0000	389206	23815
BEND5	0	0	0	0	0	0	BEN domain containing 5 [Source:HGNC Symbol;Acc:HGNC:25668	chr1:48998	NM_00130	.	.	.	NP_00128	.	protein_co	ENSG0000	79656	25668
BEND6	1.16015	1.67035	1.7707	1.54263	1.33995	2.04999	BEN domain containing 6 [Source:HGNC Symbol;Acc:HGNC:20871	chr6:56819	NM_00131	XM_01151	.	.	NP_00130	XP_005248	protein_co	ENSG0000	221336	20871
BEND7	3.4049	2.79047	3.34533	3.0196	2.74537	2.91979	BEN domain containing 7 [Source:HGNC Symbol;Acc:HGNC:23514	chr10:1348	NM_00110	XM_01151	.	.	NP_00109	XP_005252	protein_co	ENSG0000	222389	23514
BEST1	1.67939	1.25502	0.676944	1.40306	0.788202	1.711	bestrophin 1 [Source:HGNC Symbol;Acc:HGNC:12703]	chr11:6171	NM_00113	XM_01154	.	.	NP_00113	XP_005274	protein_co	ENSG0000	7439	12703

BEST2	0.172736	0	0.048939	0	0.066655	0	bestrophin 2 [Source:HGNC Symbol;Acc:HGNC:17107]	chr19:1286	NM_01768	XM_00525	.	.	NP_06015	XP_00526	protein_co	ENSG0000	54831	17107
BEST3	0.200206	0.055618	0.077105	0.167713	0.127131	0.154001	bestrophin 3 [Source:HGNC Symbol;Acc:HGNC:17105]	chr12:7003	NM_00128	XM_01153	.	.	NP_00126	XP_01153	protein_co	ENSG0000	144453	17105
BEST4	0.313058	0.310173	0.383832	0.241501	0.206405	0.048734	bestrophin 4 [Source:HGNC Symbol;Acc:HGNC:17106]	chr1:45249	NM_15327	XM_01154	.	.	NP_69500	XP_00527	protein_co	ENSG0000	266675	17106
BET1	15.6845	14.7287	13.075	11.6701	10.9976	14.1799	Bet1 golgi vesicular membrane trafficking protein [Source:HGNC	chr7:93622	NM_00131	.	.	.	NP_00130	.	protein_co	ENSG0000	10282	14562
BET1L	31.8372	34.9561	32.7251	32.7457	29.2874	21.5683	Bet1 golgi vesicular membrane trafficking protein like [Source:HG	chr11:1677	NM_00109	.	.	.	NP_00109	.	protein_co	ENSG0000	51272	19348
BEX1	0	0	0	0	0	0	brain expressed X-linked 1 [Source:HGNC Symbol;Acc:HGNC:1036	chrX:10231	NM_01847	.	.	.	NP_06094	.	protein_co	ENSG0000	55859	1036
BEX2	35.7692	37.284	35.3257	23.8011	20.9286	35.5559	brain expressed X-linked 2 [Source:HGNC Symbol;Acc:HGNC:3093	chrX:10256	NM_00116	.	.	.	NP_00116	.	protein_co	ENSG0000	84707	30933
BEX3	102.538	94.7658	104.404	127.641	122.016	113.181	brain expressed X-linked 3 [Source:HGNC Symbol;Acc:HGNC:1338	chrX:10263	NM_01438	.	.	.	NP_05519	.	protein_co	ENSG0000	27018	13388
BEX4	0.59723	0.407689	0.847785	0	0	0.133869	brain expressed X-linked 4 [Source:HGNC Symbol;Acc:HGNC:2547	chrX:10247	NM_00108	.	.	.	NP_00107	.	protein_co	ENSG0000	56271	25475
BEX5	0.439251	0.09128	0.456094	0	0	0	brain expressed X-linked 5 [Source:HGNC Symbol;Acc:HGNC:2799	chrX:10140	NM_00101	.	.	.	NP_00101	.	protein_co	ENSG0000	340542	27990
BFAR	28.5963	29.7785	27.8289	31.0611	27.3153	26.0504	bifunctional apoptosis regulator [Source:HGNC Symbol;Acc:HGNC	chr16:1472	NM_01656	XM_01152	.	.	NP_05764	XP_00525	protein_co	ENSG0000	51283	17613
BFSP1	3.98729	3.44658	4.58366	4.34336	4.72679	6.27035	beaded filament structural protein 1 [Source:HGNC Symbol;Acc:H	chr20:1747	NM_00116	.	.	.	NP_00115	.	protein_co	ENSG0000	631	1040
BFSP2	0.09457	0	0.081796	0.10153	0	0.051099	beaded filament structural protein 2 [Source:HGNC Symbol;Acc:H	chr3:13311	NM_00357	.	.	.	NP_00356	XP_016862	protein_co	ENSG0000	8419	1041
BGLAP	3.13E-92	4.89E-22	0.42085	0.325034	3.1E-188	0.741036	bone gamma-carboxyglutamate protein [Source:HGNC Symbol;Ac	chr1:15618	NM_19917	.	.	.	NP_95464	.	protein_co	ENSG0000	632	1043
BGN	0	0	0	0	0	0.037708	biglycan [Source:HGNC Symbol;Acc:HGNC:1044]	chrX:15271	NM_00171	XM_01702	.	.	NP_00170	XP_01688	protein_co	ENSG0000	633	1044
BHLHA15	3.77068	4.27899	4.57755	3.55841	2.85081	4.38582	basic helix-loop-helix family member a15 [Source:HGNC Symbol;A	chr7:97840	NM_17745	.	.	.	NP_80323	.	protein_co	ENSG0000	168620	22265
BHLHA9	0	0	0	0	0	0	basic helix-loop-helix family member a9 [Source:HGNC Symbol;Ac	chr17:1173	NM_00116	.	.	.	NP_00115	.	protein_co	ENSG0000	727857	35126
BHLHB9	0.901098	0.829429	1.0762	0.680698	0.621622	1.0358	basic helix-loop-helix domain containing, class B, 9 [Source:HGNC	chrX:10197	NM_00114	.	.	.	NP_00113	.	protein_co	ENSG0000	80823	29353
BHLHE22	0	0	0	0	0	0	basic helix-loop-helix family member e22 [Source:HGNC Symbol;A	chr8:65492	NM_15241	.	.	.	NP_68962	.	protein_co	ENSG0000	27319	11963
BHLHE23	0	0	0	0	0	0	basic helix-loop-helix family member e23 [Source:HGNC Symbol;A	chr20:6163	NM_08060	.	.	.	NP_54217	.	protein_co	ENSG0000	128408	16093
BHLHE40	17.9779	20.1989	21.5369	10.8661	13.5226	15.8392	basic helix-loop-helix family member e40 [Source:HGNC Symbol;A	chr3:50208	NM_00367	.	.	.	NP_00366	.	protein_co	ENSG0000	8553	1046
BHLHE41	0.74371	0.875474	1.02904	0.778928	0.837198	1.5777	basic helix-loop-helix family member e41 [Source:HGNC Symbol;A	chr12:2627	NM_03076	.	.	.	NP_11038	.	protein_co	ENSG0000	79365	16617
BHMG1	1.15599	1.33772	1.15263	0.129646	0.167074	0.291988	basic helix-loop-helix and HMG-box containing 1 [Source:HGNC S	chr19:4623	NM_00131	XM_01152	.	.	NP_00129	XP_01152	protein_co	ENSG0000	388553	44318
BHMT	0	0	0.047347	0	0	0	betaine--homocysteine S-methyltransferase [Source:HGNC Symb	chr5:78407	NM_00171	.	.	.	NP_00170	.	protein_co	ENSG0000	635	1047
BHMT2	0	0	0	0	0	0	betaine--homocysteine S-methyltransferase 2 [Source:HGNC Sym	chr5:78365	NM_00117	.	.	.	NP_00117	.	protein_co	ENSG0000	23743	1048
BICC1	0.027787	0.014768	0.012078	0.031476	0	0	BicC family RNA binding protein 1 [Source:HGNC Symbol;Acc:HGN	chr10:6027	NM_00108	XM_01701	.	.	NP_00107	XP_00527	protein_co	ENSG0000	80114	19351
BICD1	8.15954	8.65125	6.45192	8.80719	7.85841	7.87842	BICD cargo adaptor 1 [Source:HGNC Symbol;Acc:HGNC:1049]	chr12:3225	NM_00100	XM_01152	.	.	NP_00100	XP_00525	protein_co	ENSG0000	636	1049
BICD2	9.62429	11.4471	13.034	11.1511	11.8438	10.2986	BICD cargo adaptor 2 [Source:HGNC Symbol;Acc:HGNC:17208]	chr9:95473	NM_00100	XM_01701	.	.	NP_00100	XP_01687	protein_co	ENSG0000	23299	17208
BICDL1	5.67988	5.82912	6.31438	4.63129	5.51428	4.70394	BICD family like cargo adaptor 1 [Source:HGNC Symbol;Acc:HGNC	chr12:1204	NM_20731	XM_00671	.	.	NP_99719	XP_00525	protein_co	ENSG0000	92558	28095
BICDL2	8.3046	7.48099	9.42193	6.03255	5.30028	7.6885	BICD family like cargo adaptor 2 [Source:HGNC Symbol;Acc:HGNC	chr16:3072	NM_00110	XM_01152	.	.	NP_00109	XP_00525	protein_co	ENSG0000	146439	33584
BID	34.4399	30.8693	35.3044	52.1316	39.2848	25.3891	BH3 interacting domain death agonist [Source:HGNC Symbol;Acc	chr22:1821	NM_00119	.	.	.	NP_00118	.	protein_co	ENSG0000	637	1050
BIK	46.7688	48.4815	55.5393	22.4655	20.1503	14.3312	BCL2 interacting killer [Source:HGNC Symbol;Acc:HGNC:1051]	chr22:4350	NM_00119	.	.	.	NP_00118	.	protein_co	ENSG0000	638	1051
BIN1	13.9307	15.5473	12.2722	16.6162	14.9368	12.0944	bridging integrator 1 [Source:HGNC Symbol;Acc:HGNC:1052]	chr2:12780	NM_00430	XM_01151	.	.	NP_00130	XP_00526	protein_co	ENSG0000	274	1052
BIN2	0.028669	0.029546	0.050344	0.054539	0.028059	0.109154	bridging integrator 2 [Source:HGNC Symbol;Acc:HGNC:1053]	chr12:5167	NM_00129	XM_00526	.	.	NP_00127	XP_00526	protein_co	ENSG0000	51411	1053
BIN3	15.4876	16.2449	14.9304	17.2041	13.1004	12.0596	bridging integrator 3 [Source:HGNC Symbol;Acc:HGNC:1054]	chr8:22462	NM_01868	XM_01154	.	.	NP_06115	XP_00527	protein_co	ENSG0000	55909	1054
BIRC2	29.7708	25.7097	29.7514	18.1977	24.4143	26.0033	baculoviral IAP repeat containing 2 [Source:HGNC Symbol;Acc:HG	chr11:1022	NM_00116	.	.	.	NP_00115	.	protein_co	ENSG0000	329	590
BIRC3	25.7363	24.3907	29.7522	2.62945	2.98457	3.06489	baculoviral IAP repeat containing 3 [Source:HGNC Symbol;Acc:HG	chr11:1021	NM_00116	XM_01701	.	.	NP_00115	XP_01687	protein_co	ENSG0000	330	591
BIRC5	111.972	111.131	98.3497	163.681	151.872	117.196	baculoviral IAP repeat containing 5 [Source:HGNC Symbol;Acc:HG	chr17:7621	NM_00101	.	.	.	NP_00101	.	protein_co	ENSG0000	332	593
BIRC6	8.73474	8.95261	9.82827	7.03061	8.86976	8.98894	baculoviral IAP repeat containing 6 [Source:HGNC Symbol;Acc:HG	chr2:32582	NM_01625	XM_01153	.	.	NP_05733	XP_00526	protein_co	ENSG0000	57448	13516
BIRC7	0.1484	0.216366	0.83062	0.215394	0	0.159266	baculoviral IAP repeat containing 7 [Source:HGNC Symbol;Acc:HG	chr20:6186	NM_02216	.	.	.	NP_07144	.	protein_co	ENSG0000	79444	13702
BIRC8	0	0	0	0	0	0	baculoviral IAP repeat containing 8 [Source:HGNC Symbol;Acc:HG	chr19:5379	NM_03334	.	.	.	NP_20312	.	protein_co	ENSG0000	112401	14878
BIVM	6.80782	6.4496	6.11268	4.84824	6.13437	5.5819	basic, immunoglobulin-like variable motif containing [Source:HGN	chr13:1034	NM_00115	.	.	.	NP_00115	.	protein_co	ENSG0000	54841	16034
BIVM-ERCC5	1.97934	2.08537	3.34398	1.42884	1.06221	1.64785	BIVM-ERCC5 readthrough [Source:HGNC Symbol;Acc:HGNC:4369	chr13:1034	NM_00120	.	.	.	NP_00119	.	protein_co	ENSG0000	.	43690
BLACE	0	0	0	0	0	0	B-cell acute lymphoblastic leukemia expressed [Source:HGNC Syn	chr7:15514	.	.	.	.	.	.	protein_co	ENSG0000	.	20484
BLCAP	36.6706	42.0089	37.6226	31.3466	32.5888	22.7234	bladder cancer associated protein [Source:HGNC Symbol;Acc:HGN	chr20:3614	NM_00116	.	.	.	NP_00116	XP_01688	protein_co	ENSG0000	10904	1055
BLID	0.32016	0.337688	0.185517	0	0	0	BH3-like motif containing, cell death inducer [Source:HGNC Symb	chr11:1219	NM_00100	.	.	.	NP_00100	.	protein_co	ENSG0000	414899	33495
BLK	0	0	0	0	0	0	BLK proto-oncogene, Src family tyrosine kinase [Source:HGNC Syn	chr8:11351	NM_00171	XM_01154	.	.	NP_00170	XP_011542	protein_co	ENSG0000	640	1057
BLM	8.65843	8.24141	7.50838	8.96056	11.4255	9.79496	Bloom syndrome RecQ like helicase [Source:HGNC Symbol;Acc:HG	chr15:9126	NM_00005	.	.	.	NP_00004	.	protein_co	ENSG0000	641	1058
BLMH	32.0786	32.2879	32.002	44.9981	39.3221	34.6947	bleomycin hydrolase [Source:HGNC Symbol;Acc:HGNC:1059]	chr17:2857	NM_00038	.	.	.	NP_00037	.	protein_co	ENSG0000	642	1059
BLNK	0.05509	0.095075	0.09629	0	0.04611	0.056847	B-cell linker [Source:HGNC Symbol;Acc:HGNC:14211]	chr10:9795	NM_00111	XM_01153	.	.	NP_00110	XP_01153	protein_co	ENSG0000	29760	14211
BLOC1S1	59.7926	55.5084	42.6072	59.0353	35.9542	41.3435	biogenesis of lysosomal organelles complex 1 subunit 1 [Source:H	chr12:5610	NM_00148	.	.	.	NP_00147	.	protein_co	ENSG0000	2647	4200
BLOC1S2	73.0948	63.6495	71.8253	68.6992	73.1724	72.9168	biogenesis of lysosomal organelles complex 1 subunit 2 [Source:H	chr10:1020	NM_00100	.	.	.	NP_00100	XP_016871	protein_co	ENSG0000	282991	20984
BLOC1S3	8.85463	10.574	7.40214	8.83813	7.60712	7.33618	biogenesis of lysosomal organelles complex 1 subunit 3 [Source:H	chr19:4568	NM_21255	XM_01152	.	.	NP_99771	XP_01152	protein_co	ENSG0000	388552	20914
BLOC1S4	13.4942	14.5503	16.7328	16.5019	14.1545	12.7742	biogenesis of lysosomal organelles complex 1 subunit 4 [Source:H	chr4:67177	NM_01836	.	.	.	NP_06083	.	protein_co	ENSG0000	55330	24206
BLOC1S5	5.75631	6.31558	8.16875	6.18387	8.15428	9.70988	biogenesis of lysosomal organelles complex 1 subunit 5 [Source:H	chr6:80137	NM_00119	.	.	.	NP_00118	.	protein_co	ENSG0000	63915	18561
BLOC1S6	22.845	22.6603	25.1345	21.216	24.3774	25.5784	biogenesis of lysosomal organelles complex 1 subunit 6 [Source:H	chr15:4587	NM_00131	.	.	.	NP_00129	.	protein_co	ENSG0000	26258	8549
BLVRA	30.6707	27.0038	35.274	32.2439	25.2756	30.0667	biliverdin reductase A [Source:HGNC Symbol;Acc:HGNC:1062]	chr7:43798	NM_00071	XM_01151	.	.	NP_00070	XP_01151	protein_co	ENSG0000	644	1062
BLVRB	92.1043	90.1391	60.5951	92.0791	66.9632	78.8795	biliverdin reductase B [Source:HGNC Symbol;Acc:HGNC:1063]	chr19:4095	NM_00071	.	.	.	NP_00070	.	protein_co	ENSG0000	645	1063
BLZF1	13.0533	11.2444	11.9701	13.3588	14.0316	12.6819	basic leucine zipper nuclear factor 1 [Source:HGNC Symbol;Acc:H	chr1:16933	NM_00132	XM_01151	.	.	NP_00130	XP_01150	protein_co	ENSG0000	8548	1065

BMF	0.618213	0.972961	1.02125	0.321726	0.245558	0.520324	Bcl2 modifying factor [Source:HGNC Symbol;Acc:HGNC:24132]	chr15:4038	NM_00100	XM_00672	.	.	NP_00100	XP_005254	protein_co	ENSG0000	90427	24132
BMI1	18.693	15.0878	15.932	17.1624	25.2259	26.8219	BMI1 proto-oncogene, polycomb ring finger [Source:HGNC Symb	chr10:2260	NM_00518	.	.	.	NP_00517	.	protein_co	ENSG0000	648	1066
BMP1	11.8042	11.4926	14.5718	11.5582	11.0798	10.1383	bone morphogenetic protein 1 [Source:HGNC Symbol;Acc:HGNC:	chr8:22022	NM_00119	XM_00671	.	.	NP_00119	XP_006716	protein_co	ENSG0000	649	1067
BMP10	0	0	0	0	0	0	bone morphogenetic protein 10 [Source:HGNC Symbol;Acc:HGNC	chr2:69092	NM_01448	.	.	.	NP_05529	.	protein_co	ENSG0000	27302	20869
BMP15	0	0	0	0	0	0	bone morphogenetic protein 15 [Source:HGNC Symbol;Acc:HGNC	chrX:50653	NM_00544	.	.	.	NP_00543	.	protein_co	ENSG0000	9210	1068
BMP2	0	0.026273	0.044137	0.286002	0.173088	0.235651	bone morphogenetic protein 2 [Source:HGNC Symbol;Acc:HGNC:	chr20:6748	NM_00120	.	.	.	NP_00119	.	protein_co	ENSG0000	650	1069
BMP2K	4.7648	5.29328	6.89338	4.17182	5.52038	5.80928	BMP2 inducible kinase [Source:HGNC Symbol;Acc:HGNC:18041]	chr4:79697	NM_01759	XM_01700	.	.	NP_06006	XP_005263	protein_co	ENSG0000	55589	18041
BMP3	0	0	0	0	0	0	bone morphogenetic protein 3 [Source:HGNC Symbol;Acc:HGNC:	chr4:81952	NM_00120	XM_00671	.	.	NP_00119	XP_006714	protein_co	ENSG0000	651	1070
BMP4	54.1658	51.8092	63.8892	168.315	156.63	161.552	bone morphogenetic protein 4 [Source:HGNC Symbol;Acc:HGNC:	chr14:5441	NM_00120	XM_01702	.	.	NP_00119	XP_005268	protein_co	ENSG0000	652	1071
BMP5	0	0	0.018534	0.024496	0	0	bone morphogenetic protein 5 [Source:HGNC Symbol;Acc:HGNC:	chr6:55618	NM_02107	XM_01151	.	.	NP_06655	XP_011513	protein_co	ENSG0000	653	1072
BMP6	1.33106	1.16794	1.27996	2.59547	2.25968	2.91211	bone morphogenetic protein 6 [Source:HGNC Symbol;Acc:HGNC:	chr6:77270	NM_00171	.	.	.	NP_00170	.	protein_co	ENSG0000	654	1073
BMP7	4.88365	4.67445	6.00769	5.34222	4.39938	4.4719	bone morphogenetic protein 7 [Source:HGNC Symbol;Acc:HGNC:	chr20:5574	NM_00171	.	.	.	NP_00171	.	protein_co	ENSG0000	655	1074
BMP8A	0.854834	1.38835	1.22852	1.04262	0.696176	1.49499	bone morphogenetic protein 8a [Source:HGNC Symbol;Acc:HGNC	chr1:39957	NM_18180	.	.	.	NP_86152	.	protein_co	ENSG0000	353500	21650
BMP8B	5.71424	5.65788	5.49392	6.31901	5.55819	6.40376	bone morphogenetic protein 8b [Source:HGNC Symbol;Acc:HGNC	chr1:40204	NM_00172	.	.	.	NP_00171	.	protein_co	ENSG0000	656	1075
BMPER	0	0	0.02803	0.018269	0.036453	0.016246	BMP binding endothelial regulator [Source:HGNC Symbol;Acc:HG	chr7:33944	NM_13346	XM_00524	.	.	NP_59772	XP_005249	protein_co	ENSG0000	168667	24154
BMPR1A	3.99593	3.91437	3.90181	4.22565	4.86139	5.77809	bone morphogenetic protein receptor type 1A [Source:HGNC Syn	chr10:8851	NM_00432	XM_01154	.	.	NP_00432	XP_011538	protein_co	ENSG0000	657	1076
BMPR1B	0	0.040392	0	0	0	0	bone morphogenetic protein receptor type 1B [Source:HGNC Syn	chr4:95679	NM_00120	XM_01700	.	.	NP_00119	XP_011530	protein_co	ENSG0000	658	1077
BMPR2	2.84752	3.19613	3.59601	2.73311	3.21909	3.92718	bone morphogenetic protein receptor type 2 [Source:HGNC Symb	chr2:20324	NM_00120	XM_01151	.	.	NP_00119	XP_011509	protein_co	ENSG0000	659	1078
BMS1	29.4218	30.0318	27.0359	32.3197	31.2854	24.4502	BMS1, ribosome biogenesis factor [Source:HGNC Symbol;Acc:HG	chr10:4327	NM_01475	XM_01154	.	.	NP_05556	XP_005271	protein_co	ENSG0000	9790	23505
BMT2	1.74452	1.83929	3.22356	1.89439	2.37592	3.08663	base methyltransferase of 25S rRNA 2 homolog [Source:HGNC Sy	chr7:11245	NM_15255	.	.	.	NP_68976	.	protein_co	ENSG0000	154743	26475
BMX	0	0	0.023066	0	0	0	BMX non-receptor tyrosine kinase [Source:HGNC Symbol;Acc:HG	chrX:15402	NM_00132	XM_00672	.	.	NP_00130	.	protein_co	ENSG0000	660	1079
BNC1	0	0.020307	0	0	0	0	basonuclin 1 [Source:HGNC Symbol;Acc:HGNC:1081]	chr15:8392	NM_00130	XM_01152	.	.	NP_00128	XP_011520	protein_co	ENSG0000	646	1081
BNC2	0.037626	0	0	0	0	0.019129	basonuclin 2 [Source:HGNC Symbol;Acc:HGNC:30988]	chr9:16409	NM_00131	XM_01701	.	.	NP_00130	XP_011516	protein_co	ENSG0000	54796	30988
BNIP1	15.9317	14.6113	13.0241	16.828	15.6423	12.14	BCL2 interacting protein 1 [Source:HGNC Symbol;Acc:HGNC:1082	chr5:17257	NM_00120	XM_01153	.	.	NP_00119	XP_011532	protein_co	ENSG0000	662	1082
BNIP2	21.8425	20.6828	19.8352	18.7448	20.9805	25.5603	BCL2 interacting protein 2 [Source:HGNC Symbol;Acc:HGNC:1083	chr15:5999	NM_00132	XM_00525	.	.	NP_00130	.	protein_co	ENSG0000	663	1083
BNIP3	15.68	15.116	12.9673	16.4856	15.9795	17.022	BCL2 interacting protein 3 [Source:HGNC Symbol;Acc:HGNC:1084	chr10:1337	NM_00405	.	.	.	NP_00404	.	protein_co	ENSG0000	664	1084
BNIP3L	18.2567	18.9471	23.3548	18.4488	17.7159	25.3182	BCL2 interacting protein 3 like [Source:HGNC Symbol;Acc:HGNC:1	chr8:26240	NM_00433	XM_01154	.	.	NP_00432	XP_005273	protein_co	ENSG0000	665	1085
BNIPL	0.328629	0.303116	0.169021	0.324005	0.286122	0.3357	BCL2 interacting protein like [Source:HGNC Symbol;Acc:HGNC:16	chr1:15100	NM_00115	XM_01150	.	.	NP_00115	XP_011507	protein_co	ENSG0000	149428	16976
BOC	0.912821	0.700495	1.11169	1.19208	0.577552	0.834647	BOC cell adhesion associated, oncogene regulated [Source:HGNC	chr3:11293	NM_00130	XM_01700	.	.	NP_00128	XP_005247	protein_co	ENSG0000	91653	17173
BOD1	39.8972	34.3488	35.2287	36.2968	35.0168	40.0768	biorientation of chromosomes in cell division 1 [Source:HGNC Syr	chr5:17303	NM_00115	.	.	.	NP_00115	.	protein_co	ENSG0000	91272	25114
BOD1L1	5.08465	5.30853	4.99329	3.06847	3.54052	3.8306	biorientation of chromosomes in cell division 1 like 1 [Source:HGI	chr4:13570	NM_14889	XM_01700	.	.	NP_68369	XP_005248	protein_co	ENSG0000	259282	31792
BOD1L2	0.025742	0.027146	0.022263	0	0	0.103288	biorientation of chromosomes in cell division 1 like 2 [Source:HGI	chr18:5481	NM_00125	.	.	.	NP_00124	.	protein_co	ENSG0000	284257	28505
BOK	17.1431	17.2985	18.2356	19.0783	16.6098	14.9907	BOK, BCL2 family apoptosis regulator [Source:HGNC Symbol;Acc:	chr2:24249	NM_03251	XM_01151	.	.	NP_11590	XP_005247	protein_co	ENSG0000	666	1087
BOLA1	23.8357	22.9104	19.8706	26.9678	21.752	21.3079	bolA family member 1 [Source:HGNC Symbol;Acc:HGNC:24263]	chr1:14985	NM_01607	XM_00671	.	.	NP_00130	XP_006711	protein_co	ENSG0000	51027	24263
BOLA2	0	0	0	0	0	0	bolA family member 2 [Source:HGNC Symbol;Acc:HGNC:29488]	chr16:2946	NM_00103	XM_01699	.	.	NP_00102	XP_016855	protein_co	ENSG0000	107984053	29488
BOLA2B	0	0	0	0	0	0	bolA family member 2B [Source:HGNC Symbol;Acc:HGNC:32479]	chr16:3020	NM_00103	XM_01699	NR_13530	.	NP_00103	XP_016855	protein_co	ENSG0000	107984053	32479
BOLA3	65.8905	66.5322	54.3035	89.0344	64.5455	56.5751	bolA family member 3 [Source:HGNC Symbol;Acc:HGNC:24415]	chr2:74362	NM_00103	.	.	.	NP_00103	.	protein_co	ENSG0000	388962	24415
BOLL	0	0	0	0	0	0	boule homolog, RNA binding protein [Source:HGNC Symbol;Acc:H	chr2:19859	NM_00128	XM_01151	.	.	NP_00127	XP_006712	protein_co	ENSG0000	66037	14273
BOP1	34.8774	35.0638	31.4802	48.4904	45.5237	28.3521	block of proliferation 1 [Source:HGNC Symbol;Acc:HGNC:15519]	chr8:14548	NM_01520	.	NR_10705	.	NP_05601	.	protein_co	ENSG0000	102465906	15519
BORA	16.8482	16.2975	14.3943	22.7325	24.9203	20.202	bora, aurora kinase A activator [Source:HGNC Symbol;Acc:HGNC:	chr13:7330	NM_00128	XM_00671	.	.	NP_00127	XP_006719	protein_co	ENSG0000	79866	24724
BORCS5	6.10032	4.2832	3.42448	8.04726	4.20077	5.9278	BLOC-1 related complex subunit 5 [Source:HGNC Symbol;Acc:HGI	chr12:1251	NM_00130	XM_01152	.	.	NP_00128	XP_011518	protein_co	ENSG0000	118426	17950
BORCS6	8.46092	6.17103	6.3935	6.76869	5.90281	5.44282	BLOC-1 related complex subunit 6 [Source:HGNC Symbol;Acc:HGI	chr17:8091	NM_01762	.	.	.	NP_06009	.	protein_co	ENSG0000	54785	25939
BORCS7	10.2509	12.9232	12.4916	11.0972	10.4757	9.90893	BLOC-1 related complex subunit 7 [Source:HGNC Symbol;Acc:HGI	chr10:1046	NM_00113	.	.	.	NP_00112	.	protein_co	ENSG0000	119032	23516
BORCS8	6.92448	6.71628	6.24998	5.0656	4.19244	1.96539	BLOC-1 related complex subunit 8 [Source:HGNC Symbol;Acc:HGI	chr19:1925	NM_00114	.	.	.	NP_00113	.	protein_co	ENSG0000	729991	37247
BORCS8-MEF2B	4.28339	4.12825	3.63011	6.41778	3.36873	1.99809	BORCS8-MEF2B readthrough [Source:HGNC Symbol;Acc:HGNC:39	chr19:1925	NM_00591	.	.	.	NP_00591	.	protein_co	ENSG0000	4207	39979
BPGM	11.9803	11.2901	10.859	13.9419	12.8125	12.6984	bisphosphoglycerate mutase [Source:HGNC Symbol;Acc:HGNC:10	chr7:13433	NM_00129	XM_01151	.	.	NP_00128	XP_011514	protein_co	ENSG0000	669	1093
BPHL	10.2029	12.4784	12.8967	12.7402	9.87625	10.8874	biphenyl hydrolase like [Source:HGNC Symbol;Acc:HGNC:1094]	chr6:31186	NM_00130	.	.	.	NP_00128	.	protein_co	ENSG0000	670	1094
BPI	0	0	0	0	0	0	bactericidal/permeability-increasing protein [Source:HGNC Symb	chr20:3683	NM_00172	XM_01152	.	.	NP_00171	XP_011527	protein_co	ENSG0000	671	1095
BPIFA1	0	0	0	0	0	0	BPI fold containing family A member 1 [Source:HGNC Symbol;Acc	chr20:3182	NM_00124	.	.	.	NP_00123	.	protein_co	ENSG0000	51297	15749
BPIFA2	0	0	0	0	0	0	BPI fold containing family A member 2 [Source:HGNC Symbol;Acc	chr20:3174	NM_00131	XM_00526	.	.	NP_00130	XP_005260	protein_co	ENSG0000	140683	16203
BPIFA3	0	0	0	0	0	0	BPI fold containing family A member 3 [Source:HGNC Symbol;Acc	chr20:3180	NM_00104	.	.	XR_244132	NP_00103	.	protein_co	ENSG0000	128861	16204
BPIFB1	0	0	0	0	0	0	BPI fold containing family B member 1 [Source:HGNC Symbol;Acc	chr20:3187	NM_03319	.	.	.	NP_14997	.	protein_co	ENSG0000	92747	16108
BPIFB2	0	0	0	0	0	0	BPI fold containing family B member 2 [Source:HGNC Symbol;Acc	chr20:3159	NM_02522	.	.	.	NP_07950	.	protein_co	ENSG0000	80341	16177
BPIFB3	0	0	0	0	0	0	BPI fold containing family B member 3 [Source:HGNC Symbol;Acc	chr20:3164	NM_18265	.	.	.	NP_87259	.	protein_co	ENSG0000	359710	16178
BPIFB4	0	0	0	0	0	0	BPI fold containing family B member 4 [Source:HGNC Symbol;Acc	chr20:3166	NM_18251	.	.	.	NP_87232	.	protein_co	ENSG0000	149954	16179
BPIFB6	0	0	0	0	0	0	BPI fold containing family B member 6 [Source:HGNC Symbol;Acc	chr20:3161	NM_17489	XM_01152	.	.	NP_77755	XP_011526	protein_co	ENSG0000	128859	16504
BPIFC	0	0	0	0.053808	0	0	BPI fold containing family C [Source:HGNC Symbol;Acc:HGNC:165	chr22:3280	NM_17493	XM_01702	.	.	NP_77759	XP_011528	protein_co	ENSG0000	254240	16503
BPNT1	17.0084	17.9831	15.9912	20.4097	18.9353	19.9377	3'(2'), 5'-bisphosphate nucleotidase 1 [Source:HGNC Symbol;Acc:	chr1:22023	NM_00128	XM_01700	.	.	NP_00127	XP_005273	protein_co	ENSG0000	10380	1096
BPTF	9.32198	9.72587	11.2416	8.73519	11.1143	12.0703	bromodomain PHD finger transcription factor [Source:HGNC Sym	chr17:6582	NM_00445	XM_01152	.	.	NP_00445	XP_005257	protein_co	ENSG0000	2186	3581

BPY2	0	0	0	0	0	0	basic charge, Y-linked, 2 [Source:HGNC Symbol;Acc:HGNC:13508]	chrY:25130	NM_00467	.	.	.	NP_00466	.	protein_co	ENSG0000	9083	13508
BPY2B	0	0	0	0	0	0	basic charge, Y-linked, 2B [Source:HGNC Symbol;Acc:HGNC:25449	chrY:26764	NM_00100	.	.	.	NP_00100	.	protein_co	ENSG0000	442867	25449
BPY2C	0	0	0	0	0	0	basic charge, Y-linked, 2C [Source:HGNC Symbol;Acc:HGNC:18225	chrY:27177	NM_00100	.	.	.	NP_00100	.	protein_co	ENSG0000	442868	18225
BRAF	3.62787	4.45394	3.79302	3.6469	3.6072	4.8583	B-Raf proto-oncogene, serine/threonine kinase [Source:HGNC Sy	chr7:14043	NM_00433	XM_01151	.	.	NP_00432	XP_00525	protein_co	ENSG0000	673	1097
BRAP	9.84723	8.89652	8.71884	8.56911	8.5455	7.81401	BRCA1 associated protein [Source:HGNC Symbol;Acc:HGNC:1099	chr12:1120	NM_00676	XM_01153	.	.	NP_00675	XP_005254	protein_co	ENSG0000	8315	1099
BRAT1	28.102	27.0627	28.5183	26.1261	23.7739	22.274	BRCA1 associated ATM activator 1 [Source:HGNC Symbol;Acc:HG	chr7:25775	NM_15274	XM_01701	.	.	NP_68995	XP_00524	protein_co	ENSG0000	221927	21701
BRCA1	10.8788	10.4103	9.43396	9.79806	10.5117	10.2176	BRCA1, DNA repair associated [Source:HGNC Symbol;Acc:HGNC:1	chr17:4119	NM_00729	.	.	.	NP_00922	.	protein_co	ENSG0000	672	1100
BRCA2	2.27929	2.70917	2.37544	1.60806	2.47795	2.73746	BRCA2, DNA repair associated [Source:HGNC Symbol;Acc:HGNC:1	chr13:3288	NM_00005	XM_01153	.	.	NP_00005	XP_011533	protein_co	ENSG0000	675	1101
BRCC3	11.3697	10.6052	11.2256	11.9253	15.7204	10.4671	BRCA1/BRCA2-containing complex subunit 3 [Source:HGNC Symb	chrX:15428	NM_00101	XM_01702	.	.	NP_00101	XP_005274	protein_co	ENSG0000	79184	24185
BRD1	11.9793	13.4682	10.9162	14.4054	14.8249	12.8699	bromodomain containing 1 [Source:HGNC Symbol;Acc:HGNC:110	chr22:5016	NM_00130	XM_01702	.	.	NP_00129	XP_011528	protein_co	ENSG0000	23774	1102
BRD2	90.3922	90.8678	80.4291	90.3101	87.0151	87.1234	bromodomain containing 2 [Source:HGNC Symbol;Acc:HGNC:110	chr6:32936	NM_00111	.	.	.	NP_00110	.	protein_co	ENSG0000	6046	1103
BRD3	11.3239	12.141	11.0058	9.89866	10.0944	11.7781	bromodomain containing 3 [Source:HGNC Symbol;Acc:HGNC:110	chr9:13689	NM_00737	XM_01701	.	.	NP_03139	XP_006717	protein_co	ENSG0000	8019	1104
BRD4	6.0614	6.51531	5.36989	5.67724	6.28769	6.93181	bromodomain containing 4 [Source:HGNC Symbol;Acc:HGNC:135	chr19:1534	NM_01429	XM_01152	.	.	NP_05511	XP_011526	protein_co	ENSG0000	23476	13575
BRD7	27.9362	27.4666	26.3587	29.4202	29.2404	27.9451	bromodomain containing 7 [Source:HGNC Symbol;Acc:HGNC:143	chr16:5030	NM_00117	XM_01152	.	.	NP_00116	XP_011521	protein_co	ENSG0000	29117	14310
BRD8	18.9913	19.186	17.203	16.889	17.612	14.119	bromodomain containing 8 [Source:HGNC Symbol;Acc:HGNC:198	chr5:13747	NM_00116	XM_01154	.	.	NP_00115	XP_005271	protein_co	ENSG0000	10902	19874
BRD9	15.208	14.3368	16.0527	14.2896	14.0117	11.7058	bromodomain containing 9 [Source:HGNC Symbol;Acc:HGNC:258	chr5:86423	NM_00100	XM_01151	.	.	NP_00100	.	protein_co	ENSG0000	65980	25818
BRDT	0	0	0.016579	0	0	0.02111	bromodomain testis associated [Source:HGNC Symbol;Acc:HGNC	chr1:92414	NM_00124	XM_01154	.	.	NP_00122	XP_006710	protein_co	ENSG0000	676	1105
BRE	15.8271	15.3994	14.0752	16.9861	13.6216	11.4329	brain and reproductive organ-expressed (TNFRSF1A modulator) [	chr2:27994	NM_00126	.	.	.	NP_00124	.	protein_co	ENSG0000	9577	1106
BRF1	11.631	12.0928	12.7081	10.4762	9.32797	6.56453	BRF1, RNA polymerase III transcription initiation factor 90 kDa su	chr14:1056	NM_00124	XM_01153	.	.	NP_00122	XP_005267	protein_co	ENSG0000	2972	11551
BRF2	10.2897	10.341	7.80913	12.8283	11.6271	7.69997	BRF2, RNA polymerase III transcription initiation factor 50 kDa su	chr8:37654	NM_01831	.	.	.	NP_06078	.	protein_co	ENSG0000	55290	17298
BRI3	58.1589	47.2202	48.4001	49.435	39.7608	39.9525	brain protein I3 [Source:HGNC Symbol;Acc:HGNC:1109]	chr7:97910	NM_00115	.	.	.	NP_00115	.	protein_co	ENSG0000	25798	1109
BRI3BP	28.1183	28.9657	27.9021	27.7257	30.9069	26.487	BRI3 binding protein [Source:HGNC Symbol;Acc:HGNC:14251]	chr12:1254	NM_08062	.	.	.	NP_54219	.	protein_co	ENSG0000	140707	14251
BRICD5	2.83729	1.69799	2.38459	5.38467	5.14087	4.82484	BRICHOS domain containing 5 [Source:HGNC Symbol;Acc:HGNC:2	chr16:2255	NM_18256	XM_00525	.	.	NP_87236	XP_005255	protein_co	ENSG0000	283870	28309
BRINP1	0	0	0	0	0	0	BMP/retinoic acid inducible neural specific 1 [Source:HGNC Symb	chr9:12192	NM_01461	.	.	.	NP_05543	.	protein_co	ENSG0000	1620	2687
BRINP2	0	0	0	0	0	0	BMP/retinoic acid inducible neural specific 2 [Source:HGNC Symb	chr1:17714	NM_02116	XM_01150	.	.	NP_06698	XP_005245	protein_co	ENSG0000	57795	13746
BRINP3	0	0	0	0	0.027919	0	BMP/retinoic acid inducible neural specific 3 [Source:HGNC Symb	chr1:19006	NM_19905	XM_01150	.	.	NP_95025	XP_005245	protein_co	ENSG0000	339479	22393
BRIP1	6.91544	7.01835	6.32046	7.11505	9.042	8.20226	BRCA1 interacting protein C-terminal helicase 1 [Source:HGNC Sy	chr17:5975	NM_03204	XM_01152	.	.	NP_11443	XP_011523	protein_co	ENSG0000	83990	20473
BRIX1	55.0964	46.9001	45.8338	57.6221	70.9717	57.4448	BRX1, biogenesis of ribosomes [Source:HGNC Symbol;Acc:HGNC:	chr5:34905	NM_01832	.	.	.	NP_06079	.	protein_co	ENSG0000	55299	24170
BRK1	105.575	101.188	123.682	107.608	105.725	120.153	BRICK1, SCAR/WAVE actin nucleating complex subunit [Source:H	chr3:10157	NM_01846	.	.	.	NP_06093	.	protein_co	ENSG0000	55845	23057
BRMS1	42.4546	43.2582	35.7539	56.2909	45.1554	22.3327	breast cancer metastasis suppressor 1 [Source:HGNC Symbol;Acc	chr11:6610	NM_00102	.	.	.	NP_00102	.	protein_co	ENSG0000	25855	17262
BRMS1L	3.87463	3.6886	3.46043	3.69466	5.24756	3.70648	breast cancer metastasis-suppressor 1-like [Source:HGNC Symbol	chr14:3629	NM_03235	XM_00672	.	.	NP_11572	XP_005268	protein_co	ENSG0000	84312	20512
BROX	11.0659	11.736	11.4046	8.12599	9.36996	11.8465	BRO1 domain and CAAX motif containing [Source:HGNC Symbol;A	chr1:22288	NM_00128	XM_01150	NR_10992	.	NP_00127	XP_005273	protein_co	ENSG0000	148362	26512
BRPF1	8.90118	9.35778	8.33522	9.31951	9.10991	7.34984	bromodomain and PHD finger containing 1 [Source:HGNC Symbo	chr3:97734	NM_00100	XM_01153	.	.	NP_00100	XP_005265	protein_co	ENSG0000	7862	14255
BRPF3	7.64494	8.02009	6.52498	6.57903	7.45889	6.18666	bromodomain and PHD finger containing 3 [Source:HGNC Symbo	chr6:36164	NM_01569	XM_01701	.	.	NP_05651	XP_005249	protein_co	ENSG0000	27154	14256
BRS3	0.068158	0	0	0	0.037523	0	bombesin receptor subtype 3 [Source:HGNC Symbol;Acc:HGNC:1	chrX:13557	NM_00172	XM_00526	.	.	NP_00171	XP_005262	protein_co	ENSG0000	680	1113
BRSK1	9.86343	8.09955	9.90198	4.64018	4.58185	4.68718	BR serine/threonine kinase 1 [Source:HGNC Symbol;Acc:HGNC:18	chr19:5579	NM_03243	XM_00525	.	.	NP_11580	XP_005259	protein_co	ENSG0000	84446	18994
BRSK2	7.03043	7.87648	10.5516	6.59829	5.75393	3.81742	BR serine/threonine kinase 2 [Source:HGNC Symbol;Acc:HGNC:11	chr11:1411	NM_00125	XM_01152	.	.	NP_00124	XP_005253	protein_co	ENSG0000	9024	11405
BRWD1	13.7149	13.1675	15.1085	7.87914	11.6417	14.9621	bromodomain and WD repeat domain containing 1 [Source:HGN	chr21:4055	NM_00100	XM_01702	.	.	NP_00100	XP_011527	protein_co	ENSG0000	54014	12760
BRWD3	2.14357	2.19984	2.47772	2.14197	2.65825	2.6447	bromodomain and WD repeat domain containing 3 [Source:HGN	chrX:79926	NM_15325	XM_01153	.	.	NP_69498	XP_005262	protein_co	ENSG0000	254065	17342
BSCL2	32.163	31.5391	25.8747	43.1507	38.8839	24.3551	BSCL2, seipin lipid droplet biogenesis associated [Source:HGNC Sy	chr11:6245	NM_00112	.	.	.	NP_00111	.	protein_co	ENSG0000	26580	15832
BSDC1	25.1425	24.7819	21.9016	14.9908	13.6039	14.6084	BSD domain containing 1 [Source:HGNC Symbol;Acc:HGNC:25501	chr1:32830	NM_00114	XM_01154	.	.	NP_00113	XP_005271	protein_co	ENSG0000	55108	25501
BSG	460.063	468.548	352.558	527.481	440.189	422.172	basigin (Ok blood group) [Source:HGNC Symbol;Acc:HGNC:1116]	chr19:5712	NM_00132	XM_01702	.	.	NP_00130	XP_005259	protein_co	ENSG0000	682	1116
BSN	0.477964	0.361448	0.290368	0.320382	0.33163	0.21292	bassoon presynaptic cytomatrix protein [Source:HGNC Symbol;Ac	chr3:49591	NM_00345	.	.	.	NP_00344	.	protein_co	ENSG0000	8927	1117
BSND	0	0.020924	0	0	0	0.021948	barttin CLCNK type accessory beta subunit [Source:HGNC Symbol	chr1:55464	NM_05717	.	.	.	NP_47651	.	protein_co	ENSG0000	7809	16512
BSPH1	0	0	0	0	0	0	binder of sperm protein homolog 1 [Source:HGNC Symbol;Acc:HG	chr19:4847	NM_00112	.	.	.	NP_00112	XP_016881	protein_co	ENSG0000	100131137	33906
BSPRY	13.9872	13.5928	14.826	14.412	12.9781	14.9858	B-box and SPRY domain containing [Source:HGNC Symbol;Acc:HG	chr9:11611	NM_00131	XM_00671	.	.	NP_00130	XP_006717	protein_co	ENSG0000	54836	18232
BST1	0.417503	0.254299	0.214644	0.549094	0.4169	0.29366	bone marrow stromal cell antigen 1 [Source:HGNC Symbol;Acc:H	chr4:15704	NM_00433	XM_01151	.	.	NP_00432	XP_011512	protein_co	ENSG0000	683	1118
BST2	21.3682	20.0533	6.88666	4.51052	2.22177	2.99282	bone marrow stromal cell antigen 2 [Source:HGNC Symbol;Acc:H	chr19:1751	NM_00433	.	.	.	NP_00432	.	protein_co	ENSG0000	684	1119
BSX	0	0	0	0	0	0	brain specific homeobox [Source:HGNC Symbol;Acc:HGNC:20450]	chr11:1228	NM_00109	.	.	.	NP_00109	.	protein_co	ENSG0000	390259	20450
BTAF1	16.0682	16.5735	19.5932	14.1702	17.7505	18.5106	B-TFIID TATA-box binding protein associated factor 1 [Source:HG	chr10:9368	NM_00397	XM_01701	.	.	NP_00396	XP_011538	protein_co	ENSG0000	9044	17307
BTBD1	41.516	40.6078	49.641	44.6175	46.9706	55.0542	BTB domain containing 1 [Source:HGNC Symbol;Acc:HGNC:1120]	chr15:8368	NM_00101	.	.	.	NP_00101	.	protein_co	ENSG0000	53339	1120
BTBD10	13.8863	12.9647	14.2608	12.6664	12.0859	14.2532	BTB domain containing 10 [Source:HGNC Symbol;Acc:HGNC:2144	chr11:1340	NM_00129	XM_01701	.	.	NP_00128	XP_011518	protein_co	ENSG0000	84280	21445
BTBD11	1.50326	1.52193	2.69013	1.5142	1.27238	1.96672	BTB domain containing 11 [Source:HGNC Symbol;Acc:HGNC:2384	chr12:1077	NM_00101	XM_01153	.	.	NP_00101	XP_005268	protein_co	ENSG0000	121551	23844
BTBD16	0.155569	0.080233	0.203542	0.12913	0.131555	0.323424	BTB domain containing 16 [Source:HGNC Symbol;Acc:HGNC:2634	chr10:1240	NM_00131	XM_01153	.	.	NP_00130	XP_011537	protein_co	ENSG0000	118663	26340
BTBD17	0	0	0	0	0	0.08417	BTB domain containing 17 [Source:HGNC Symbol;Acc:HGNC:3375	chr17:7235	NM_00108	.	.	.	NP_00107	XP_011523	protein_co	ENSG0000	388419	33758
BTBD18	0	0	0	0	0	0	BTB domain containing 18 [Source:HGNC Symbol;Acc:HGNC:3721	chr11:5751	NM_00114	XM_01701	.	.	NP_00113	XP_011543	protein_co	ENSG0000	643376	37214
BTBD19	0.54297	0.979524	2.93675	0.576384	0.491679	1.23319	BTB domain containing 19 [Source:HGNC Symbol;Acc:HGNC:2714	chr1:45274	NM_00113	XM_01154	.	.	NP_00113	XP_006710	protein_co	ENSG0000	149478	27145
BTBD2	35.1182	37.4538	43.9659	37.8283	30.4905	37.3276	BTB domain containing 2 [Source:HGNC Symbol;Acc:HGNC:15504	chr19:1985	NM_01779	XM_00525	.	.	NP_06026	XP_005259	protein_co	ENSG0000	55643	15504
BTBD3	9.38712	10.3548	10.0054	9.5842	9.0306	10.4978	BTB domain containing 3 [Source:HGNC Symbol;Acc:HGNC:15854	chr20:1187	NM_00128	XM_01702	.	.	NP_00126	XP_016883	protein_co	ENSG0000	22903	15854

BTBD6	31.5292	28.9241	23.798	35.5054	31.6595	26.618	BTB domain containing 6 [Source:HGNC Symbol;Acc:HGNC:19897	chr14:1056	NM_03327	XM_00526	.	.	NP_15037	XP_005268	protein_co	ENSG0000	90135	19897
BTBD7	8.0334	7.86913	8.35851	7.21602	8.94715	8.895	BTB domain containing 7 [Source:HGNC Symbol;Acc:HGNC:18269	chr14:9370	NM_00100	XM_01153	.	.	NP_00100	XP_011535	protein_co	ENSG0000	55727	18269
BTBD8	0.726129	1.91985	1.13004	0.844101	0.984903	1.60805	BTB domain containing 8 [Source:HGNC Symbol;Acc:HGNC:21019	chr1:92545	NM_18324	.	.	.	NP_89906	XP_016856	protein_co	ENSG0000	284697	21019
BTBD9	2.84356	2.95732	3.71259	3.04023	3.68808	2.75226	BTB domain containing 9 [Source:HGNC Symbol;Acc:HGNC:21228	chr6:38136	NM_00109	XM_01151	.	.	NP_00109	XP_005248	protein_co	ENSG0000	114781	21228
BTC	2.61582	3.0351	2.18107	3.46506	3.95791	4.53268	betacellulin [Source:HGNC Symbol;Acc:HGNC:1121]	chr4:75669	NM_00172	XM_01153	.	.	NP_00172	XP_011530	protein_co	ENSG0000	685	1121
BTD	2.9888	2.94589	2.60371	3.58482	3.03491	3.31051	biotinidase [Source:HGNC Symbol;Acc:HGNC:1122]	chr3:15602	NM_00006	XM_01153	.	.	NP_00005	XP_006713	protein_co	ENSG0000	686	1122
BTF3	630.603	595.897	600.584	657.364	576.554	631.009	basic transcription factor 3 [Source:HGNC Symbol;Acc:HGNC:112	chr5:72794	NM_00103	.	.	.	NP_00103	.	protein_co	ENSG0000	689	1125
BTF3L4	37.2312	37.0211	36.215	40.4835	42.3415	62.8798	basic transcription factor 3 like 4 [Source:HGNC Symbol;Acc:HGN	chr1:52485	NM_00113	.	.	.	NP_00112	.	protein_co	ENSG0000	91408	30547
BTG1	13.096	15.133	9.59497	10.7831	11.2849	16.6817	BTG anti-proliferation factor 1 [Source:HGNC Symbol;Acc:HGNC:1	chr12:9253	NM_00173	.	.	.	NP_00172	.	protein_co	ENSG0000	694	1130
BTG2	30.5205	29.5115	41.9191	27.9916	30.3206	24.1645	BTG anti-proliferation factor 2 [Source:HGNC Symbol;Acc:HGNC:1	chr1:20327	NM_00676	.	.	.	NP_00675	.	protein_co	ENSG0000	7832	1131
BTG3	61.4177	65.8757	56.2846	89.9287	82.0894	77.1658	BTG anti-proliferation factor 3 [Source:HGNC Symbol;Acc:HGNC:1	chr21:1896	NM_00113	XM_01152	.	.	NP_00112	XP_011527	protein_co	ENSG0000	10950	1132
BTG4	0	0.087006	0	0.0932	0	0	BTG anti-proliferation factor 4 [Source:HGNC Symbol;Acc:HGNC:1	chr11:1113	NM_01758	.	.	.	NP_06005	XP_011541	protein_co	ENSG0000	54766	13862
BTK	0.115399	0.083468	0.069722	0.180122	0.298869	0.376565	Bruton tyrosine kinase [Source:HGNC Symbol;Acc:HGNC:1133]	chrX:10060	NM_00006	.	.	.	NP_00005	.	protein_co	ENSG0000	695	1133
BTLA	0	0	0.093401	0	0	0	B and T lymphocyte associated [Source:HGNC Symbol;Acc:HGNC:	chr3:11218	NM_00108	XM_01151	.	.	NP_00107	XP_011510	protein_co	ENSG0000	151888	21087
BTN1A1	0.283832	0.407459	0.447948	0.191232	0.265726	0.176531	butyrophilin subfamily 1 member A1 [Source:HGNC Symbol;Acc:H	chr6:26501	NM_00173	XM_00524	.	.	NP_00172	XP_005249	protein_co	ENSG0000	696	1135
BTN2A1	7.39446	7.48216	5.69163	7.75547	5.91385	5.488	butyrophilin subfamily 2 member A1 [Source:HGNC Symbol;Acc:H	chr6:26458	NM_00119	.	.	.	NP_00118	.	protein_co	ENSG0000	11120	1136
BTN2A2	7.51997	7.48926	7.53604	6.44647	6.22761	6.65683	butyrophilin subfamily 2 member A2 [Source:HGNC Symbol;Acc:H	chr6:26383	NM_00119	XM_01151	.	.	NP_00118	XP_005248	protein_co	ENSG0000	10385	1137
BTN3A1	5.6806	5.2079	4.26543	3.43803	3.83265	2.56345	butyrophilin subfamily 3 member A1 [Source:HGNC Symbol;Acc:H	chr6:26402	NM_00114	XM_01151	.	.	NP_00113	XP_005248	protein_co	ENSG0000	11119	1138
BTN3A2	10.0005	10.0986	7.77021	9.73324	8.72302	6.84707	butyrophilin subfamily 3 member A2 [Source:HGNC Symbol;Acc:H	chr6:26365	NM_00119	XM_01701	.	.	NP_00118	XP_005248	protein_co	ENSG0000	11118	1139
BTN3A3	3.29239	3.10841	2.27824	1.09022	1.4158	0.941191	butyrophilin subfamily 3 member A3 [Source:HGNC Symbol;Acc:H	chr6:26440	NM_00124	.	.	.	NP_00122	.	protein_co	ENSG0000	10384	1140
BTNL2	0	0	0	0	0	0	butyrophilin like 2 [Source:HGNC Symbol;Acc:HGNC:1142]	chr6:32361	NM_00130	XM_01151	.	.	NP_00129	XP_016866	protein_co	ENSG0000	56244	1142
BTNL3	0	0	0	0	0	0	butyrophilin like 3 [Source:HGNC Symbol;Acc:HGNC:1143]	chr5:18041	NM_19797	.	.	.	NP_93207	.	protein_co	ENSG0000	10917	1143
BTNL8	0	0	0	0	0	0	butyrophilin like 8 [Source:HGNC Symbol;Acc:HGNC:26131]	chr5:18032	NM_00104	XM_01153	.	.	NP_00103	XP_011532	protein_co	ENSG0000	79908	26131
BTNL9	2.35849	2.47132	3.32106	1.24993	2.12876	2.27484	butyrophilin like 9 [Source:HGNC Symbol;Acc:HGNC:24176]	chr5:18046	NM_00130	XM_01700	.	.	NP_00129	XP_005265	protein_co	ENSG0000	153579	24176
BTRC	12.5663	12.8138	11.0949	11.5377	12.7035	12.2043	beta-transducin repeat containing E3 ubiquitin protein ligase [Sou	chr10:1031	NM_00125	XM_00671	.	.	NP_00124	XP_006718	protein_co	ENSG0000	8945	1144
BUB1	29.7987	30.5701	29.4742	54.5351	62.582	46.5832	BUB1 mitotic checkpoint serine/threonine kinase [Source:HGNC S	chr2:11139	NM_00127	.	.	.	NP_00126	.	protein_co	ENSG0000	699	1148
BUB1B	31.7934	31.8475	29.8959	46.3548	47.7106	33.6418	BUB1 mitotic checkpoint serine/threonine kinase B [Source:HGN	chr15:4045	NM_00121	.	.	.	NP_00120	.	protein_co	ENSG0000	701	1149
BUB1B-PAK6	8.36E-17	0.150167	4.47E-11	1.02E-13	6.91E-06	0.291375	BUB1B-PAK6 readthrough [Source:EntrezGene;Acc:106821730]	chr15:4045	NM_00112	.	.	.	NP_00112	.	protein_co	ENSG0000	10682173	.
BUB3	173.049	178.058	162.52	236.926	222.816	187.358	BUB3, mitotic checkpoint protein [Source:HGNC Symbol;Acc:HGN	chr10:1249	NM_00100	.	.	.	NP_00100	.	protein_co	ENSG0000	9184	1151
BUD13	13.2107	13.6917	12.9399	18.2021	14.6445	12.6377	BUD13 homolog [Source:HGNC Symbol;Acc:HGNC:28199]	chr11:1166	NM_00115	.	.	.	NP_00115	XP_011541	protein_co	ENSG0000	84811	28199
BUD31	73.2809	67.5189	50.6284	71.0577	53.9371	70.8432	BUD31 homolog [Source:HGNC Symbol;Acc:HGNC:29629]	chr7:98992	NM_00391	XM_00525	.	.	NP_00390	XP_005250	protein_co	ENSG0000	8896	29629
BVES	0.874851	0.950577	1.20088	1.04332	1.5082	1.4636	blood vessel epicardial substance [Source:HGNC Symbol;Acc:HGN	chr6:10554	NM_00119	XM_01153	.	.	NP_00118	XP_011533	protein_co	ENSG0000	11149	1152
BX088651.2	0	0	0	0	0	0	Uncharacterized protein [Source:UniProtKB/TrEMBL;Acc:M0QXD	chr9:44325	.	.	.	.	.	.	protein_co	ENSG0000	.	.
BX255923.1	0	0	0	0	0	0	HDCMB45P; Uncharacterized protein [Source:UniProtKB/TrEMBL	chr9:69204	.	.	.	.	.	.	protein_co	ENSG0000	.	.
BX649567.1	0	0	0	0	0	0	Uncharacterized protein [Source:UniProtKB/TrEMBL;Acc:M0QZF	chr9:67924	.	.	.	.	.	.	protein_co	ENSG0000	.	.
BX842679.1	0	0	0	0	0	0	Uncharacterized protein FLJ46360 [Source:UniProtKB/Swiss-Prot	chr1:14763	.	.	.	.	.	.	protein_co	ENSG0000	.	.
BYSL	32.2026	31.0929	26.467	39.6128	36.4522	24.9491	bystin like [Source:HGNC Symbol;Acc:HGNC:1157]	chr6:41888	NM_00405	.	.	.	NP_00404	.	protein_co	ENSG0000	705	1157
BZW1	152.966	143.539	158.376	188.001	222.578	221.917	basic leucine zipper and W2 domains 1 [Source:HGNC Symbol;Acc	chr2:20167	NM_00120	XM_01151	.	.	NP_00119	XP_011510	protein_co	ENSG0000	9689	18380
BZW2	78.4896	75.4591	93.8167	79.9483	83.9903	85.2615	basic leucine zipper and W2 domains 2 [Source:HGNC Symbol;Acc	chr7:16685	NM_00115	XM_00671	.	.	NP_00115	XP_006715	protein_co	ENSG0000	28969	18808
C10orf10	2.32159	2.59447	2.50238	0.580158	0.860593	0.576974	chromosome 10 open reading frame 10 [Source:HGNC Symbol;Ac	chr10:4545	NM_00702	.	.	.	NP_00895	.	protein_co	ENSG0000	11067	23355
C10orf105	0	0	0	0	0	0.018148	chromosome 10 open reading frame 105 [Source:HGNC Symbol;A	chr10:7315	NM_00116	XM_01153	.	.	NP_00115	XP_011538	protein_co	ENSG0000	414152	20304
C10orf107	0.101518	0.218833	0.177128	0.233393	0.223532	0.245614	chromosome 10 open reading frame 107 [Source:HGNC Symbol;A	chr10:6342	NM_17355	XM_00526	.	.	NP_77582	XP_005269	protein_co	ENSG0000	219621	28678
C10orf11	1.74916	1.58293	2.99313	1.0832	1.80196	0.933708	chromosome 10 open reading frame 11 [Source:HGNC Symbol;Ac	chr10:7719	NM_00130	XM_01154	NR_13117	.	NP_00129	XP_005270	protein_co	ENSG0000	83938	23405
C10orf113	0	0	0	0	0	0	chromosome 10 open reading frame 113 [Source:HGNC Symbol;A	chr10:2106	NM_00101	.	.	.	NP_00101	.	protein_co	ENSG0000	387638	31447
C10orf120	0	0	0	0	0	0	chromosome 10 open reading frame 120 [Source:HGNC Symbol;A	chr10:1244	NM_00101	.	.	.	NP_00101	.	protein_co	ENSG0000	399814	25707
C10orf128	0	0	0	0	0.057358	0.04427	chromosome 10 open reading frame 128 [Source:HGNC Symbol;A	chr10:5036	NM_00101	XM_01153	.	.	NP_00101	XP_011537	protein_co	ENSG0000	170371	27274
C10orf142	0	0	0	0	0	0	chromosome 10 open reading frame 142 [Source:HGNC Symbol;A	chr10:4478	NM_00125	.	.	.	NP_00124	.	protein_co	ENSG0000	10013053	51236
C10orf2	29.2609	30.0667	30.86	26.9825	24.6468	18.3911	chromosome 10 open reading frame 2 [Source:HGNC Symbol;Acc	chr10:1027	NM_00116	.	.	.	NP_00115	.	protein_co	ENSG0000	56652	1160
C10orf35	25.7153	23.058	20.1242	31.2285	22.744	18.5723	chromosome 10 open reading frame 35 [Source:HGNC Symbol;Ac	chr10:7139	NM_14530	XM_01153	.	.	NP_66034	XP_005269	protein_co	ENSG0000	219738	23519
C10orf53	0	0	0	0	0	0	chromosome 10 open reading frame 53 [Source:HGNC Symbol;Ac	chr10:5088	NM_00104	.	.	.	NP_00103	.	protein_co	ENSG0000	282966	27421
C10orf54	1.37247	1.62598	3.73256	1.35427	1.26992	1.28322	chromosome 10 open reading frame 54 [Source:HGNC Symbol;Ac	chr10:7315	NM_02215	.	.	.	NP_07143	.	protein_co	ENSG0000	64115	30085
C10orf55	0.183179	0.295476	0.253116	0.240805	0.251325	0.232335	chromosome 10 open reading frame 55 [Source:HGNC Symbol;Ac	chr10:7566	NM_00100	.	.	.	NP_00100	.	protein_co	ENSG0000	414236	31008
C10orf62	0	0	0.047294	0	0	0	chromosome 10 open reading frame 62 [Source:HGNC Symbol;Ac	chr10:9934	NM_00100	.	.	.	NP_00100	.	protein_co	ENSG0000	414157	23294
C10orf67	0	0	0	0	0	0	chromosome 10 open reading frame 67 [Source:HGNC Symbol;Ac	chr10:2349	NM_15371	.	.	.	NP_71492	XP_011517	protein_co	ENSG0000	256815	28716
C10orf71	0	0	0	0	0	0	chromosome 10 open reading frame 71 [Source:HGNC Symbol;Ac	chr10:5050	NM_00113	XM_01153	.	.	NP_00112	XP_005269	protein_co	ENSG0000	118461	26973
C10orf76	12.9223	11.6643	11.0167	11.2346	11.8974	11.2211	chromosome 10 open reading frame 76 [Source:HGNC Symbol;Ac	chr10:1036	NM_02454	XM_01154	.	.	NP_07881	XP_006718	protein_co	ENSG0000	79591	25788
C10orf82	0.195752	0	0.204835	0.318033	0.112081	0	chromosome 10 open reading frame 82 [Source:HGNC Symbol;Ac	chr10:1184	NM_14466	XM_01701	.	.	NP_65326	XP_011537	protein_co	ENSG0000	143379	28500
C10orf88	9.56735	8.63643	9.28158	8.27558	12.4969	12.1528	chromosome 10 open reading frame 88 [Source:HGNC Symbol;Ac	chr10:1246	NM_02494	.	.	.	NP_07921	.	protein_co	ENSG0000	80007	25822
C10orf90	0	0	0	0.079363	0	0	chromosome 10 open reading frame 90 [Source:HGNC Symbol;Ac	chr10:1281	NM_00100	XM_01153	.	.	NP_00100	XP_005269	protein_co	ENSG0000	118611	26563

C10orf95	1.88591	1.88556	1.05591	2.08607	1.98902	1.44089	chromosome 10 open reading frame 95 [Source:HGNC Symbol;Ac	chr10:1042	NM_02488	.	.	.	NP_07916	.	protein_co	ENSG0000	79946	25880
C10orf99	0.18664	0.094639	0	0	0	0	chromosome 10 open reading frame 99 [Source:HGNC Symbol;Ac	chr10:8593	NM_20737	XM_00526	.	.	NP_99725	XP_005269	protein_co	ENSG0000	387695	31428
C11orf1	48.9092	43.8801	44.7212	49.1081	52.1474	45.7378	chromosome 11 open reading frame 1 [Source:HGNC Symbol;Acc	chr11:1116	NM_02276	XM_01701	.	.	NP_07359	XP_011541	protein_co	ENSG0000	64776	1163
C11orf16	0	0	0	0	0	0	chromosome 11 open reading frame 16 [Source:HGNC Symbol;Ac	chr11:8932	NM_02064	.	.	.	NP_06569	XP_016873	protein_co	ENSG0000	56673	1169
C11orf21	0	0	0	0	0	0	chromosome 11 open reading frame 21 [Source:HGNC Symbol;Ac	chr11:2316	NM_00114	XM_01152	.	.	NP_00113	XP_011518	protein_co	ENSG0000	29125	13231
C11orf24	21.9154	21.8162	18.9028	24.271	20.8466	16.2892	chromosome 11 open reading frame 24 [Source:HGNC Symbol;Ac	chr11:6802	NM_00130	XM_01154	.	.	NP_00128	XP_005274	protein_co	ENSG0000	53838	1174
C11orf31	158.625	143.71	152.344	168.773	124.736	128.343	chromosome 11 open reading frame 31 [Source:HGNC Symbol;Ac	chr11:5750	NM_17074	.	.	.	NP_00130	.	protein_co	ENSG0000	280636	18251
C11orf40	0	0	0	0	0	0	chromosome 11 open reading frame 40 [Source:HGNC Symbol;Ac	chr11:4592	NM_14466	.	.	.	NP_65326	.	protein_co	ENSG0000	143501	23986
C11orf42	0	0	0	0	0	0	chromosome 11 open reading frame 42 [Source:HGNC Symbol;Ac	chr11:6226	NM_17352	.	.	.	NP_77579	.	protein_co	ENSG0000	160298	28541
C11orf45	1.81778	2.35864	2.49318	2.48798	1.29437	0.973807	chromosome 11 open reading frame 45 [Source:HGNC Symbol;Ac	chr11:1287	NM_00125	XM_01154	.	.	NP_00124	XP_011540	protein_co	ENSG0000	219833	28584
C11orf49	13.3647	13.3262	14.0093	14.0605	12.4328	13.2304	chromosome 11 open reading frame 49 [Source:HGNC Symbol;Ac	chr11:4695	NM_00100	XM_01152	.	.	NP_00100	XP_006718	protein_co	ENSG0000	79096	28720
C11orf52	3.73489	3.14658	3.46598	2.49335	1.74487	1.94137	chromosome 11 open reading frame 52 [Source:HGNC Symbol;Ac	chr11:1117	NM_08065	.	.	.	NP_54239	.	protein_co	ENSG0000	91894	30531
C11orf53	0	0	0	0	0	0	chromosome 11 open reading frame 53 [Source:HGNC Symbol;Ac	chr11:1111	NM_19849	XM_01154	.	.	NP_94090	XP_011541	protein_co	ENSG0000	341032	30527
C11orf54	14.9514	15.9012	17.6183	15.7683	14.2317	16.2186	chromosome 11 open reading frame 54 [Source:HGNC Symbol;Ac	chr11:9346	NM_00128	XM_01154	.	.	NP_00127	XP_005273	protein_co	ENSG0000	28970	30204
C11orf57	17.2619	16.4	15.5974	15.1676	16.023	14.2531	chromosome 11 open reading frame 57 [Source:HGNC Symbol;Ac	chr11:1118	NM_00108	.	.	.	NP_00107	.	protein_co	ENSG0000	55216	25569
C11orf58	80.2622	84.7547	65.1812	85.9164	82.4745	73.6517	chromosome 11 open reading frame 58 [Source:HGNC Symbol;Ac	chr11:1675	NM_01426	.	.	.	NP_05508	.	protein_co	ENSG0000	10944	16990
C11orf63	2.02674	1.93252	2.06019	1.85353	2.13005	2.70294	chromosome 11 open reading frame 63 [Source:HGNC Symbol;Ac	chr11:1227	NM_02480	XM_01701	.	.	NP_07908	XP_005271	protein_co	ENSG0000	79864	26288
C11orf65	1.22591	0.675205	0.945204	0.385314	0.537971	1.12712	chromosome 11 open reading frame 65 [Source:HGNC Symbol;Ac	chr11:1080	NM_15258	XM_01701	.	.	NP_68980	XP_005271	protein_co	ENSG0000	160140	28519
C11orf68	40.0458	39.6278	31.1591	44.4962	35.1086	28.4928	chromosome 11 open reading frame 68 [Source:HGNC Symbol;Ac	chr11:6568	NM_00113	.	.	.	NP_00112	.	protein_co	ENSG0000	83638	28801
C11orf70	0	0	0	0	0	0	chromosome 11 open reading frame 70 [Source:HGNC Symbol;Ac	chr11:1019	NM_00119	.	.	.	NP_00118	.	protein_co	ENSG0000	85016	28188
C11orf71	5.61841	3.78592	4.16324	2.84938	3.08408	3.53255	chromosome 11 open reading frame 71 [Source:HGNC Symbol;Ac	chr11:1142	NM_01902	.	.	.	NP_06189	.	protein_co	ENSG0000	54494	25937
C11orf74	8.633	6.89828	8.34319	10.7085	9.98437	12.5738	chromosome 11 open reading frame 74 [Source:HGNC Symbol;Ac	chr11:3661	NM_00127	XM_01151	.	.	NP_00126	XP_006718	protein_co	ENSG0000	119710	25142
C11orf80	5.76374	6.35163	6.50907	6.08177	4.50464	6.34509	chromosome 11 open reading frame 80 [Source:HGNC Symbol;Ac	chr11:6651	NM_00130	.	.	.	NP_00128	.	protein_co	ENSG0000	79703	26197
C11orf84	30.4968	30.7909	26.7695	44.2093	41.1898	31.2352	chromosome 11 open reading frame 84 [Source:HGNC Symbol;Ac	chr11:6358	NM_13847	XM_01154	.	.	NP_61248	XP_006718	protein_co	ENSG0000	144097	25115
C11orf86	0.072231	0	0	0	0	0.079946	chromosome 11 open reading frame 86 [Source:HGNC Symbol;Ac	chr11:6674	NM_00113	.	.	.	NP_00112	.	protein_co	ENSG0000	254439	34442
C11orf87	0	0	0	0.020947	0	0	chromosome 11 open reading frame 87 [Source:HGNC Symbol;Ac	chr11:1092	NM_20764	XM_01154	.	.	NP_99752	XP_005271	protein_co	ENSG0000	399947	33788
C11orf88	0	0	0	0	0	0	chromosome 11 open reading frame 88 [Source:HGNC Symbol;Ac	chr11:1113	NM_00110	.	.	.	NP_00109	.	protein_co	ENSG0000	399949	25061
C11orf89	0	0	0	0	0	0	chromosome 11 open reading frame 89 [Source:HGNC Symbol;Ac	chr11:1874	.	.	.	.	.	.	protein_co	ENSG0000	.	35118
C11orf91	2.22E-97	0	2.24E-80	0	0	0	chromosome 11 open reading frame 91 [Source:HGNC Symbol;Ac	chr11:3371	NM_00116	.	.	.	NP_00116	XP_016872	protein_co	ENSG0000	100131378	34444
C11orf94	0	0	0	0	0.474983	0.446418	chromosome 11 open reading frame 94 [Source:HGNC Symbol;Ac	chr11:4592	NM_00108	.	.	.	NP_00107	.	protein_co	ENSG0000	143678	37213
C11orf95	6.46539	6.55645	6.6727	7.44946	6.65543	5.91688	chromosome 11 open reading frame 95 [Source:HGNC Symbol;Ac	chr11:6352	NM_00114	.	.	.	NP_00113	.	protein_co	ENSG0000	65998	28449
C11orf96	0	0.120293	0	0	0	0	chromosome 11 open reading frame 96 [Source:HGNC Symbol;Ac	chr11:4394	NM_00114	.	.	.	NP_00113	.	protein_co	ENSG0000	387763	38675
C11orf97	0	0	0	0	0	0	chromosome 11 open reading frame 97 [Source:HGNC Symbol;Ac	chr11:9424	NM_00119	.	.	.	NP_00117	.	protein_co	ENSG0000	643037	49544
C11orf98	130.939	119.907	104.632	178.472	131.752	99.2601	chromosome 11 open reading frame 98 [Source:HGNC Symbol;Ac	chr11:6241	NM_00128	.	NR_10441	.	NP_00127	.	protein_co	ENSG0000	10228841	51238
C12orf10	47.4638	46.1094	40.5673	59.248	44.798	33.837	chromosome 12 open reading frame 10 [Source:HGNC Symbol;Ac	chr12:5369	NM_02164	.	.	.	NP_06765	.	protein_co	ENSG0000	60314	17590
C12orf29	13.817	13.768	13.7479	14.8268	14.5168	14.4709	chromosome 12 open reading frame 29 [Source:HGNC Symbol;Ac	chr12:8842	NM_00100	.	.	.	NP_00100	.	protein_co	ENSG0000	91298	25322
C12orf4	4.66211	5.2952	4.99033	3.95767	4.951	4.439	chromosome 12 open reading frame 4 [Source:HGNC Symbol;Acc	chr12:4596	NM_00130	XM_01152	.	.	NP_00129	XP_005253	protein_co	ENSG0000	57102	1184
C12orf40	0	0	0	0	0	0	chromosome 12 open reading frame 40 [Source:HGNC Symbol;Ac	chr12:4001	NM_00103	XM_00526	.	.	NP_00102	XP_005268	protein_co	ENSG0000	283461	26846
C12orf42	0	0	0	0	0	0	chromosome 12 open reading frame 42 [Source:HGNC Symbol;Ac	chr12:1036	NM_00109	XM_01153	.	.	NP_00109	XP_006719	protein_co	ENSG0000	374470	24729
C12orf43	10.8514	10.7691	9.50982	11.7033	9.37515	7.93793	chromosome 12 open reading frame 43 [Source:HGNC Symbol;Ac	chr12:1214	NM_00128	XM_01153	.	.	NP_00127	XP_016875	protein_co	ENSG0000	64897	25719
C12orf45	15.399	17.98	16.0748	23.9142	18.9752	15.0109	chromosome 12 open reading frame 45 [Source:HGNC Symbol;Ac	chr12:1053	NM_15231	.	.	.	NP_68953	.	protein_co	ENSG0000	121053	28628
C12orf49	11.6109	14.2872	14.627	15.3452	15.1174	10.611	chromosome 12 open reading frame 49 [Source:HGNC Symbol;Ac	chr12:1171	NM_02473	.	.	.	NP_07901	.	protein_co	ENSG0000	79794	26128
C12orf50	0	0.138039	0	0	0	0	chromosome 12 open reading frame 50 [Source:HGNC Symbol;Ac	chr12:8837	NM_15258	XM_01701	.	.	NP_68980	XP_016874	protein_co	ENSG0000	160419	26665
C12orf54	0	0	0	0	0	0	chromosome 12 open reading frame 54 [Source:HGNC Symbol;Ac	chr12:4887	NM_15231	XM_00526	.	.	NP_68953	XP_005268	protein_co	ENSG0000	121273	28553
C12orf56	0	0	0.023267	0	0	0	chromosome 12 open reading frame 56 [Source:HGNC Symbol;Ac	chr12:6465	NM_00109	XM_01153	.	.	NP_00109	.	protein_co	ENSG0000	115749	26967
C12orf57	282.714	268.194	209.642	324.187	227.803	237.091	chromosome 12 open reading frame 57 [Source:HGNC Symbol;Ac	chr12:7052	NM_00130	.	.	.	NP_00128	.	protein_co	ENSG0000	113246	29521
C12orf60	3.48781	2.77132	3.29489	3.9138	3.59283	3.74941	chromosome 12 open reading frame 60 [Source:HGNC Symbol;Ac	chr12:1495	NM_17587	XM_01152	.	.	NP_78707	XP_005253	protein_co	ENSG0000	144608	28726
C12orf65	16.4071	15.4789	12.9293	15.0626	15.1566	18.0802	chromosome 12 open reading frame 65 [Source:HGNC Symbol;Ac	chr12:1236	NM_00114	XM_01153	.	.	NP_00113	XP_005253	protein_co	ENSG0000	91574	26784
C12orf66	6.52467	6.86255	8.40416	5.40358	7.69258	8.62778	chromosome 12 open reading frame 66 [Source:HGNC Symbol;Ac	chr12:6458	NM_00130	.	.	.	NP_00128	.	protein_co	ENSG0000	144577	26517
C12orf71	0	0	0	0	0	0.081932	chromosome 12 open reading frame 71 [Source:HGNC Symbol;Ac	chr12:2723	NM_00108	XM_01152	.	.	NP_00107	XP_011519	protein_co	ENSG0000	728858	34452
C12orf73	16.8794	20.6892	13.3902	17.8164	15.585	15.6988	chromosome 12 open reading frame 73 [Source:HGNC Symbol;Ac	chr12:1043	NM_00113	.	.	.	NP_00112	XP_016875	protein_co	ENSG0000	728568	34450
C12orf74	0	0	0	0	0	0	chromosome 12 open reading frame 74 [Source:HGNC Symbol;Ac	chr12:9309	NM_00103	.	.	.	NP_00103	.	protein_co	ENSG0000	338809	27887
C12orf75	49.4741	50.9464	48.4895	60.0434	59.151	65.1276	chromosome 12 open reading frame 75 [Source:HGNC Symbol;Ac	chr12:1057	NM_00114	.	.	.	NP_00113	.	protein_co	ENSG0000	387882	35164
C12orf76	7.62823	8.10174	5.82342	7.09589	8.3093	6.10967	chromosome 12 open reading frame 76 [Source:HGNC Symbol;Ac	chr12:1104	NM_20743	XM_01153	.	.	NP_99731	XP_011536	protein_co	ENSG0000	400073	33790
C14orf1	18.742	18.9736	20.2307	22.5473	19.769	22.0302	chromosome 14 open reading frame 1 [Source:HGNC Symbol;Acc	chr14:7604	NM_00717	.	.	.	NP_00910	.	protein_co	ENSG0000	11161	1187
C14orf105	0	0.023152	0.018829	0	0.023702	0.021009	chromosome 14 open reading frame 105 [Source:HGNC Symbol;A	chr14:5793	NM_00128	XM_00672	.	.	NP_00126	XP_005267	protein_co	ENSG0000	55195	20189
C14orf119	60.1916	56.0807	61.0398	60.2954	54.3897	60.3367	chromosome 14 open reading frame 119 [Source:HGNC Symbol;A	chr14:2352	NM_01792	.	.	.	NP_06039	XP_016876	protein_co	ENSG0000	55017	20270
C14orf132	2.40763	2.16101	2.98838	3.83469	2.16566	2.4954	chromosome 14 open reading frame 132 [Source:HGNC Symbol;A	chr14:9650	NM_00125	.	.	.	NP_00123	XP_016876	protein_co	ENSG0000	56967	20346
C14orf142	9.97731	9.24655	9.12204	12.5758	16.2368	11.2159	GON7, KEOPS complex subunit homolog [Source:HGNC Symbol;A	chr14:9365	NM_03249	.	.	.	NP_11587	.	protein_co	ENSG0000	84520	20356

C14orf144	0	0.046114	0.039439	0	0	0	chromosome 14 open reading frame 144 [Source:HGNC Symbol;A	chr14:1047	.	.	.	.	.	.	protein_co	ENSG0000	.	20358
C14orf159	12.4113	12.2687	14.1648	11.0131	9.51281	10.0487	chromosome 14 open reading frame 159 [Source:HGNC Symbol;A	chr14:9158	NM_00110	.	.	.	NP_00109	.	protein_co	ENSG0000	80017	20498
C14orf166	102.49	88.1129	95.8863	100.502	109.726	113.27	chromosome 14 open reading frame 166 [Source:HGNC Symbol;A	chr14:5245	NM_01603	.	.	.	NP_05712	.	protein_co	ENSG0000	51637	23169
C14orf169	3.95159	3.47711	3.58729	4.40207	4.35205	3.38386	chromosome 14 open reading frame 169 [Source:HGNC Symbol;A	chr14:7394	NM_02464	.	.	.	NP_07892	.	protein_co	ENSG0000	79697	20968
C14orf177	0	0	0	0	0	0	chromosome 14 open reading frame 177 [Source:HGNC Symbol;A	chr14:9917	NM_18256	.	.	.	NP_87236	.	protein_co	ENSG0000	283598	26375
C14orf178	0	0	0.036329	0	0	0	chromosome 14 open reading frame 178 [Source:HGNC Symbol;A	chr14:7817	NM_00117	.	.	.	NP_00116	.	protein_co	ENSG0000	283579	26385
C14orf180	0	0	0	0	0	0	chromosome 14 open reading frame 180 [Source:HGNC Symbol;A	chr14:1050	NM_00100	XM_01153	.	.	NP_00100	XP_005267	protein_co	ENSG0000	400258	33795
C14orf2	191.57	145.231	157.755	228.093	180.907	177.531	chromosome 14 open reading frame 2 [Source:HGNC Symbol;Acc	chr14:1043	NM_00112	.	.	.	NP_00112	.	protein_co	ENSG0000	9556	1188
C14orf28	1.57199	1.48455	1.81792	0.93289	0.848335	2.20992	chromosome 14 open reading frame 28 [Source:HGNC Symbol;Ac	chr14:4536	NM_00101	.	.	.	NP_00101	.	protein_co	ENSG0000	122525	19834
C14orf37	0.373539	0.286257	0.516557	0.49219	0.392509	0.587078	chromosome 14 open reading frame 37 [Source:HGNC Symbol;Ac	chr14:5846	NM_00100	.	.	.	NP_00100	XP_016876	protein_co	ENSG0000	145407	19846
C14orf39	0	0	0	0	0	0	chromosome 14 open reading frame 39 [Source:HGNC Symbol;Ac	chr14:6090	NM_17497	XM_01702	.	.	NP_77763	XP_011534	protein_co	ENSG0000	317761	19849
C14orf79	8.66968	8.82078	11.084	7.937	7.28428	7.72239	chromosome 14 open reading frame 79 [Source:HGNC Symbol;Ac	chr14:1054	NM_17489	.	.	.	NP_77755	.	protein_co	ENSG0000	122616	20126
C14orf80	21.9367	23.1427	20.1109	28.0918	21.5194	16.7984	chromosome 14 open reading frame 80 [Source:HGNC Symbol;Ac	chr14:1059	NM_00113	.	.	.	NP_00112	.	protein_co	ENSG0000	283643	20127
C14orf93	6.91707	5.04303	5.62972	5.5719	5.00903	4.312	chromosome 14 open reading frame 93 [Source:HGNC Symbol;Ac	chr14:2345	NM_00113	XM_01153	.	.	NP_00112	XP_005268	protein_co	ENSG0000	60686	20162
C15ORF31	0	0	0	0	0	0	.	chr15:5942	.	.	.	.	.	.	protein_co	ENSG0000	.	.
C15ORF37	1.22645	0.821218	1.36449	1.49215	1.42483	0.924409	C15orf37 protein; Uncharacterized protein [Source:UniProtKB/Tr	chr15:8013	.	.	.	.	.	.	protein_co	ENSG0000	.	.
C15orf38-AP3S2	6.78056	4.79382	5.66089	7.52846	6.99824	4.78907	C15orf38-AP3S2 readthrough [Source:HGNC Symbol;Acc:HGNC:3	chr15:9037	NM_00119	.	.	.	NP_00118	.	protein_co	ENSG0000	100526783	38824
C15orf39	12.6585	13.3847	11.6544	13.097	11.2035	9.197	chromosome 15 open reading frame 39 [Source:HGNC Symbol;Ac	chr15:7549	NM_01549	XM_01152	.	.	NP_05630	XP_005254	protein_co	ENSG0000	56905	24497
C15orf40	14.5274	13.8021	11.7251	14.5431	10.8901	12.1973	chromosome 15 open reading frame 40 [Source:HGNC Symbol;Ac	chr15:8365	NM_00116	.	NR_02764	.	NP_00115	XP_011519	protein_co	ENSG0000	123207	28443
C15orf41	4.54105	4.31785	4.6079	4.04447	2.76389	3.82629	chromosome 15 open reading frame 41 [Source:HGNC Symbol;Ac	chr15:3687	NM_00113	XM_01152	.	.	NP_00112	XP_011520	protein_co	ENSG0000	84529	26929
C15orf48	0	0.594147	0.495569	0.577396	0.567502	0.571175	chromosome 15 open reading frame 48 [Source:HGNC Symbol;Ac	chr15:4572	NM_03241	.	NR_03059	.	NP_11578	.	protein_co	ENSG0000	100126311	29898
C15orf52	4.4331	4.17505	5.96951	1.41923	1.66752	2.17167	chromosome 15 open reading frame 52 [Source:HGNC Symbol;Ac	chr15:4062	NM_20738	.	.	.	NP_99726	.	protein_co	ENSG0000	388115	33488
C15orf53	0	0	0	0	0	0	chromosome 15 open reading frame 53 [Source:HGNC Symbol;Ac	chr15:3898	NM_20744	.	.	.	NP_99732	.	protein_co	ENSG0000	400359	33796
C15orf57	11.5185	9.0526	11.388	11.9946	11.4382	9.62309	chromosome 15 open reading frame 57 [Source:HGNC Symbol;Ac	chr15:4082	NM_00108	.	.	.	NP_00107	.	protein_co	ENSG0000	90416	28295
C15orf59	1.5084	1.05198	1.11384	0.247685	0.647781	0.560909	chromosome 15 open reading frame 59 [Source:HGNC Symbol;Ac	chr15:7402	NM_00103	XM_00525	.	.	NP_00103	XP_005254	protein_co	ENSG0000	388135	33753
C15orf61	3.81839	3.56732	2.86545	2.81767	2.92188	2.64246	chromosome 15 open reading frame 61 [Source:HGNC Symbol;Ac	chr15:6781	NM_00114	.	.	.	NP_00113	.	protein_co	ENSG0000	145853	34453
C15orf62	0.199049	0.170221	0.494463	0.245279	0.098313	0.216267	chromosome 15 open reading frame 62 [Source:HGNC Symbol;Ac	chr15:4105	NM_00113	.	.	.	NP_00112	.	protein_co	ENSG0000	643338	34489
C15orf65	1.67032	0.888979	0.965528	0.948711	0.764709	1.36751	chromosome 15 open reading frame 65 [Source:HGNC Symbol;Ac	chr15:5570	NM_00119	.	.	.	NP_00118	.	protein_co	ENSG0000	145788	44654
C16orf13	53.3176	57.4886	51.9266	61.2703	35.4073	32.4592	methyltransferase like 26 [Source:HGNC Symbol;Acc:HGNC:1414	chr16:6844	NM_00104	XM_01152	.	.	NP_00103	XP_011521	protein_co	ENSG0000	84326	14141
C16orf45	14.8847	15.34	14.2187	8.27696	7.76304	8.3334	chromosome 16 open reading frame 45 [Source:HGNC Symbol;Ac	chr16:1552	NM_00114	.	.	.	NP_00113	.	protein_co	ENSG0000	89927	19213
C16orf46	4.60944	3.24359	3.59316	3.8354	3.95296	3.89804	chromosome 16 open reading frame 46 [Source:HGNC Symbol;Ac	chr16:8108	NM_00110	XM_01152	.	.	NP_00109	XP_005255	protein_co	ENSG0000	123775	26525
C16orf52	8.72857	9.71893	15.6718	8.23697	8.47772	12.1479	chromosome 16 open reading frame 52 [Source:HGNC Symbol;Ac	chr16:2201	NM_00116	.	.	.	NP_00115	XP_006726	protein_co	ENSG0000	102725138	27087
C16orf54	0	0	0	0	0	0	chromosome 16 open reading frame 54 [Source:HGNC Symbol;Ac	chr16:2975	NM_17590	XM_01150	.	.	NP_78709	XP_011506	protein_co	ENSG0000	105369277	26649
C16orf58	29.9076	29.1274	28.2118	29.1962	25.0051	26.2853	chromosome 16 open reading frame 58 [Source:HGNC Symbol;Ac	chr16:3149	NM_02274	.	.	.	NP_07358	.	protein_co	ENSG0000	64755	25848
C16orf59	18.3322	16.3392	14.4414	17.7139	13.0373	10.2922	chromosome 16 open reading frame 59 [Source:HGNC Symbol;Ac	chr16:2510	NM_02510	XM_01152	.	.	NP_07938	XP_011520	protein_co	ENSG0000	80178	25849
C16orf62	7.84175	8.03829	7.193	7.56503	6.88301	6.95682	chromosome 16 open reading frame 62 [Source:HGNC Symbol;Ac	chr16:1956	NM_00130	XM_01154	.	.	NP_00128	XP_005255	protein_co	ENSG0000	57020	24641
C16orf70	12.7095	13.8319	13.5547	12.8289	14.6636	10.401	chromosome 16 open reading frame 70 [Source:HGNC Symbol;Ac	chr16:6714	NM_00132	XM_01152	.	.	NP_00130	XP_005256	protein_co	ENSG0000	80262	29564
C16orf71	0.182427	0.289996	0.185988	0.194865	0.121171	0.148577	chromosome 16 open reading frame 71 [Source:HGNC Symbol;Ac	chr16:4746	NM_13917	XM_00525	.	.	NP_63190	XP_005255	protein_co	ENSG0000	146562	25081
C16orf72	11.4822	11.8993	10.968	10.8064	12.7891	15.1294	chromosome 16 open reading frame 72 [Source:HGNC Symbol;Ac	chr16:9185	NM_01411	.	.	.	NP_05483	.	protein_co	ENSG0000	29035	30103
C16orf74	29.6151	28.0359	27.1049	31.4463	23.0986	17.3155	chromosome 16 open reading frame 74 [Source:HGNC Symbol;Ac	chr16:8572	NM_20696	XM_00672	.	.	NP_99685	XP_006721	protein_co	ENSG0000	404550	23362
C16orf78	0	0	0	0	0	0	chromosome 16 open reading frame 78 [Source:HGNC Symbol;Ac	chr16:4940	NM_14460	.	.	.	NP_65320	.	protein_co	ENSG0000	123970	28479
C16orf82	0	0	0	0	0	0	chromosome 16 open reading frame 82 [Source:HGNC Symbol;Ac	chr16:2707	NM_00114	.	.	.	NP_00113	.	protein_co	ENSG0000	162083	30755
C16orf86	0.13798	0	0.118491	0.072697	0	0.078342	chromosome 16 open reading frame 86 [Source:HGNC Symbol;Ac	chr16:6770	NM_00101	XM_00525	.	.	NP_00101	XP_005256	protein_co	ENSG0000	388284	33755
C16orf87	8.40433	8.96459	9.98868	10.0221	12.2619	12.0034	chromosome 16 open reading frame 87 [Source:HGNC Symbol;Ac	chr16:4683	NM_00100	.	.	.	NP_00100	.	protein_co	ENSG0000	388272	33754
C16orf89	0	0	0	0	0.058487	0	chromosome 16 open reading frame 89 [Source:HGNC Symbol;Ac	chr16:5083	NM_00109	.	.	.	NP_00109	.	protein_co	ENSG0000	146556	28687
C16orf90	0	0	0	0	0	0	chromosome 16 open reading frame 90 [Source:HGNC Symbol;Ac	chr16:3543	NM_00108	XM_00525	.	.	NP_00107	XP_005255	protein_co	ENSG0000	646174	34455
C16orf91	31.6749	31.446	26.428	33.9232	28.1777	19.5277	chromosome 16 open reading frame 91 [Source:HGNC Symbol;Ac	chr16:1469	NM_00127	.	.	.	NP_00125	.	protein_co	ENSG0000	283951	27558
C16orf92	0	0	0	0	0	0	chromosome 16 open reading frame 92 [Source:HGNC Symbol;Ac	chr16:3003	NM_00110	.	.	.	NP_00110	.	protein_co	ENSG0000	146378	26346
C16orf95	5.31648	5.39006	6.62008	6.38527	4.35905	5.9293	chromosome 16 open reading frame 95 [Source:HGNC Symbol;Ac	chr16:8733	NM_00119	.	.	.	NP_00118	.	protein_co	ENSG0000	100506581	40033
C16orf96	0	0	0	0	0	0.018342	chromosome 16 open reading frame 96 [Source:HGNC Symbol;Ac	chr16:4606	NM_00114	XM_01152	.	.	NP_00113	XP_005255	protein_co	ENSG0000	342346	40031
C17orf105	0.039262	0.041332	0.068245	0.022089	0.021957	0.078884	chromosome 17 open reading frame 105 [Source:HGNC Symbol;A	chr17:4185	NM_00113	XM_01152	.	.	NP_00112	XP_011522	protein_co	ENSG0000	284067	37241
C17orf107	0.453868	0.570494	0.787224	0.458351	0.594518	0.560043	chromosome 17 open reading frame 107 [Source:HGNC Symbol;A	chr17:4736	NM_00114	.	.	.	NP_00113	.	protein_co	ENSG0000	100130311	37238
C17orf47	0	0	0	0	0	0	chromosome 17 open reading frame 47 [Source:HGNC Symbol;Ac	chr17:5661	NM_00103	.	.	.	NP_00103	XP_011522	protein_co	ENSG0000	284083	26844
C17orf49	54.9449	50.7269	46.0487	55.5555	42.2856	50.1337	chromosome 17 open reading frame 49 [Source:HGNC Symbol;Ac	chr17:6915	NM_00114	.	.	.	NP_00113	.	protein_co	ENSG0000	124944	28737
C17orf50	0.229776	0	0.112243	0	0.157651	0	chromosome 17 open reading frame 50 [Source:HGNC Symbol;Ac	chr17:3408	NM_14527	XM_00525	.	.	NP_66031	XP_005257	protein_co	ENSG0000	146853	29581
C17orf51	3.51693	4.02539	3.51473	4.22063	3.57617	2.67436	chromosome 17 open reading frame 51 [Source:HGNC Symbol;Ac	chr17:2143	NM_00111	.	.	.	NP_00110	.	protein_co	ENSG0000	339263	27904
C17orf53	5.17947	6.82357	4.71287	4.72657	3.67193	3.09679	chromosome 17 open reading frame 53 [Source:HGNC Symbol;Ac	chr17:4221	NM_00117	XM_01702	.	.	NP_00116	XP_011523	protein_co	ENSG0000	78995	28460
C17orf58	30.3497	28.6503	30.4023	29.9125	28.6604	28.3973	chromosome 17 open reading frame 58 [Source:HGNC Symbol;Ac	chr17:6598	NM_18165	.	.	.	NP_85804	.	protein_co	ENSG0000	284018	27568
C17orf62	34.6853	34.5252	33.9557	35.0364	30.4236	30.0144	chromosome 17 open reading frame 62 [Source:HGNC Symbol;Ac	chr17:8034	NM_00103	XM_01152	NR_03651	.	NP_00102	XP_006722	protein_co	ENSG0000	79415	28672

C17orf64	0.076925	0	0	0	0.088976	0	chromosome 17 open reading frame 64 [Source:HGNC Symbol;Ac	chr17:5849	NM_18170	XM_00525	.	.	NP_85905	XP_005257	protein_co	ENSG0000	124773	26990
C17orf67	0.494942	0.743725	0.498591	0.633746	0.432253	0.6317	chromosome 17 open reading frame 67 [Source:HGNC Symbol;Ac	chr17:5486	NM_00108	.	.	.	NP_00107	.	protein_co	ENSG0000	339210	27900
C17orf74	0	0.068845	0	0.056854	0	0	chromosome 17 open reading frame 74 [Source:HGNC Symbol;Ac	chr17:7328	NM_17573	.	.	.	NP_78386	.	protein_co	ENSG0000	201243	27315
C17orf75	9.7122	8.67286	8.69701	10.2433	11.3662	11.3177	chromosome 17 open reading frame 75 [Source:HGNC Symbol;Ac	chr17:3065	NM_02234	XM_01702	.	.	NP_07173	XP_005258	protein_co	ENSG0000	64149	30173
C17orf78	0	0	0	0	0	0	chromosome 17 open reading frame 78 [Source:HGNC Symbol;Ac	chr17:3544	NM_00132	XM_01152	.	.	NP_00130	XP_011522	protein_co	ENSG0000	284099	26831
C17orf80	18.3579	17.5863	17.843	17.395	18.9321	16.3524	chromosome 17 open reading frame 80 [Source:HGNC Symbol;Ac	chr17:7118	NM_00110	XM_01702	.	.	NP_00109	XP_005257	protein_co	ENSG0000	55028	29601
C17orf89	167.084	149.882	137.365	173.685	129.758	108.459	chromosome 17 open reading frame 89 [Source:HGNC Symbol;Ac	chr17:7921	NM_00108	.	.	.	NP_00107	.	protein_co	ENSG0000	284184	33551
C17orf96	23.1041	24.5392	26.9337	34.7601	28.4438	23.4609	chromosome 17 open reading frame 96 [Source:HGNC Symbol;Ac	chr17:3682	NM_00113	.	.	.	NP_00112	.	protein_co	ENSG0000	100170841	34493
C17orf97	2.49769	2.24218	1.92996	2.12044	1.44242	1.62347	chromosome 17 open reading frame 97 [Source:HGNC Symbol;Ac	chr17:2601	NM_00101	.	.	.	NP_00101	.	protein_co	ENSG0000	400566	33800
C17orf98	0	0	0	0	0	0	chromosome 17 open reading frame 98 [Source:HGNC Symbol;Ac	chr17:3699	NM_00108	.	.	.	NP_00107	.	protein_co	ENSG0000	388381	34492
C17orf99	0	0	0	0	0.086324	0	chromosome 17 open reading frame 99 [Source:HGNC Symbol;Ac	chr17:7614	NM_00116	.	.	.	NP_00115	XP_011522	protein_co	ENSG0000	100141515	34490
C18orf21	17.9082	18.5181	16.2357	21.0417	16.6393	13.4101	chromosome 18 open reading frame 21 [Source:HGNC Symbol;Ac	chr18:3355	NM_00120	XM_00525	.	.	NP_00118	XP_005258	protein_co	ENSG0000	83608	28802
C18orf25	14.2097	14.1383	15.1892	12.836	15.8526	14.5574	chromosome 18 open reading frame 25 [Source:HGNC Symbol;Ac	chr18:4375	NM_00100	XM_01702	.	.	NP_00100	XP_011524	protein_co	ENSG0000	147339	28172
C18orf32	29.4504	28.3117	33.6095	31.0916	32.5575	43.9747	chromosome 18 open reading frame 32 [Source:HGNC Symbol;Ac	chr18:4700	NM_00103	.	.	.	NP_00103	.	protein_co	ENSG0000	497661	31690
C18orf54	3.10507	3.35177	2.8085	3.26939	4.08692	3.66236	chromosome 18 open reading frame 54 [Source:HGNC Symbol;Ac	chr18:5185	NM_00128	XM_01152	.	.	NP_00127	XP_005258	protein_co	ENSG0000	162681	13796
C18orf63	0	0	0	0	0	0	chromosome 18 open reading frame 63 [Source:HGNC Symbol;Ac	chr18:7198	NM_00117	.	.	.	NP_00116	.	protein_co	ENSG0000	644041	40037
C18orf8	13.682	15.0774	15.4924	14.3263	13.3879	14.8599	chromosome 18 open reading frame 8 [Source:HGNC Symbol;Acc	chr18:2108	NM_00127	XM_01152	.	.	NP_00126	XP_011524	protein_co	ENSG0000	29919	24326
C19orf12	9.65588	9.41664	8.90415	8.51871	9.63682	8.95581	chromosome 19 open reading frame 12 [Source:HGNC Symbol;Ac	chr19:3018	NM_00103	.	.	.	NP_00102	.	protein_co	ENSG0000	83636	25443
C19orf18	0	0	0	0	0	0	chromosome 19 open reading frame 18 [Source:HGNC Symbol;Ac	chr19:5846	NM_15247	.	.	.	NP_68968	.	protein_co	ENSG0000	147685	28642
C19orf24	49.9558	49.298	42.9635	58.154	39.0488	34.2744	chromosome 19 open reading frame 24 [Source:HGNC Symbol;Ac	chr19:1275	NM_01791	.	.	.	NP_06038	.	protein_co	ENSG0000	55009	26073
C19orf25	44.2182	43.3737	37.9563	42.0399	33.8483	26.983	chromosome 19 open reading frame 25 [Source:HGNC Symbol;Ac	chr19:1450	NM_15248	XM_00672	.	.	NP_68969	XP_005259	protein_co	ENSG0000	148223	26711
C19orf33	172.11	147.23	102.542	156.419	104.404	81.3042	chromosome 19 open reading frame 33 [Source:HGNC Symbol;Ac	chr19:3875	NM_00131	.	.	.	NP_00130	.	protein_co	ENSG0000	64073	16668
C19orf35	0	0	0	0	0	0	chromosome 19 open reading frame 35 [Source:HGNC Symbol;Ac	chr19:2274	NM_19853	.	.	.	NP_94093	.	protein_co	ENSG0000	374872	24793
C19orf38	0.167281	0.368337	0.302567	0.435435	0.095279	0.679124	chromosome 19 open reading frame 38 [Source:HGNC Symbol;Ac	chr19:1094	NM_00113	XM_00525	.	.	NP_00112	XP_005259	protein_co	ENSG0000	255809	34073
C19orf43	159.911	155.282	132.276	197.804	142.58	147.399	chromosome 19 open reading frame 43 [Source:HGNC Symbol;Ac	chr19:1284	NM_02403	.	.	.	NP_07694	.	protein_co	ENSG0000	79002	28424
C19orf44	3.7117	4.75364	3.31701	2.88383	3.38502	3.29018	chromosome 19 open reading frame 44 [Source:HGNC Symbol;Ac	chr19:1660	NM_00128	XM_01152	.	.	NP_00127	XP_006722	protein_co	ENSG0000	84167	26141
C19orf45	0.342293	0.167526	0.292368	0.183657	0.1334	0.126929	chromosome 19 open reading frame 45 [Source:HGNC Symbol;Ac	chr19:7562	NM_19853	XM_01152	.	.	NP_94093	XP_005272	protein_co	ENSG0000	374877	24745
C19orf47	8.91455	8.09051	8.73222	10.7146	9.66047	6.98497	chromosome 19 open reading frame 47 [Source:HGNC Symbol;Ac	chr19:4082	NM_00125	XM_01702	.	.	NP_00124	XP_005258	protein_co	ENSG0000	126526	26723
C19orf48	145.733	144.402	115.421	146.972	118.659	93.7502	chromosome 19 open reading frame 48 [Source:HGNC Symbol;Ac	chr19:5130	NM_00129	.	.	.	NP_00127	.	protein_co	ENSG0000	84798	29667
C19orf52	15.0967	16.54	17.1314	19.0127	17.286	17.5928	chromosome 19 open reading frame 52 [Source:HGNC Symbol;Ac	chr19:1103	NM_13835	.	.	.	NP_61236	.	protein_co	ENSG0000	90580	25152
C19orf53	108.632	98.438	101.95	131.043	91.4896	75.4999	chromosome 19 open reading frame 53 [Source:HGNC Symbol;Ac	chr19:1387	NM_01404	.	.	.	NP_05476	.	protein_co	ENSG0000	28974	24991
C19orf54	6.73194	7.15584	6.14469	7.2487	6.95589	7.53955	chromosome 19 open reading frame 54 [Source:HGNC Symbol;Ac	chr19:4122	NM_19847	XM_01702	.	.	NP_94087	XP_005258	protein_co	ENSG0000	284325	24758
C19orf57	0.63297	0.718946	0.23438	0.461664	0.539912	0.415876	chromosome 19 open reading frame 57 [Source:HGNC Symbol;Ac	chr19:1399	NM_02432	XM_00672	.	.	NP_07729	XP_006722	protein_co	ENSG0000	79173	28153
C19orf60	54.096	52.6837	45.0443	45.7829	35.0857	40.0827	chromosome 19 open reading frame 60 [Source:HGNC Symbol;Ac	chr19:1869	NM_00110	.	.	.	NP_00109	.	protein_co	ENSG0000	55049	26098
C19orf66	10.1184	10.7172	12.7784	4.98926	5.73784	5.22349	chromosome 19 open reading frame 66 [Source:HGNC Symbol;Ac	chr19:1019	NM_00130	XM_01702	.	.	NP_00129	XP_011526	protein_co	ENSG0000	55337	25649
C19orf67	0	0	0	0	0	0	chromosome 19 open reading frame 67 [Source:HGNC Symbol;Ac	chr19:1419	NM_00127	XM_01152	.	.	NP_00126	XP_011526	protein_co	ENSG0000	646457	34354
C19orf68	4.65449	4.09466	4.76607	4.92041	4.59252	3.97671	chromosome 19 open reading frame 68 [Source:HGNC Symbol;Ac	chr19:4867	NM_19934	XM_01152	.	.	NP_95537	XP_005259	protein_co	ENSG0000	374920	34495
C19orf70	57.4498	55.185	55.6132	50.1004	41.3947	36.5121	chromosome 19 open reading frame 70 [Source:HGNC Symbol;Ac	chr19:5674	NM_00130	XM_01152	.	.	NP_00129	XP_011525	protein_co	ENSG0000	125988	33702
C19orf71	0	0.577499	0	0	0.356361	0.170197	chromosome 19 open reading frame 71 [Source:HGNC Symbol;Ac	chr19:3539	NM_00113	.	.	.	NP_00112	.	protein_co	ENSG0000	100128569	34496
C19orf73	3.3836	1.46891	1.76141	2.63533	1.30983	1.87406	chromosome 19 open reading frame 73 [Source:HGNC Symbol;Ac	chr19:4961	NM_01811	.	.	.	NP_06058	.	protein_co	ENSG0000	55150	25534
C19orf81	1.90338	4.6419	2.1277	5.10668	1.89415	3.82103	chromosome 19 open reading frame 81 [Source:HGNC Symbol;Ac	chr19:5115	NM_00119	XM_01152	.	.	NP_00118	.	protein_co	ENSG0000	342918	40041
C19orf84	0.126729	0.063164	0.054811	0.119333	0	0.18099	chromosome 19 open reading frame 84 [Source:HGNC Symbol;Ac	chr19:5189	NM_00119	.	.	.	NP_00118	.	protein_co	ENSG0000	147646	27112
C1D	23.5061	24.6758	24.712	29.8037	33.5824	31.7551	C1D nuclear receptor corepressor [Source:HGNC Symbol;Acc:HGN	chr2:68268	NM_00119	.	.	.	NP_00117	.	protein_co	ENSG0000	10438	29911
C1GALT1	16.1886	12.7258	13.4045	9.99102	13.1711	15.562	core 1 synthase, glycoprotein-N-acetylgalactosamine 3-beta-gala	chr7:72222	NM_02015	XM_01701	.	.	NP_06454	XP_005249	protein_co	ENSG0000	56913	24337
C1GALT1C1	18.73	18.1428	20.1087	17.9931	20.0193	19.4656	C1GALT1 specific chaperone 1 [Source:HGNC Symbol;Acc:HGNC:2	chrX:11975	NM_00101	.	.	.	NP_00101	.	protein_co	ENSG0000	29071	24338
C1GALT1C1L	0.380101	0.203365	0.166493	0.795997	0	0.556751	C1GALT1-specific chaperone 1 like [Source:HGNC Symbol;Acc:HG	chr2:43864	NM_00110	.	.	.	NP_00109	.	protein_co	ENSG0000	728819	51617
C1orf100	0	0	0	0	0	0	chromosome 1 open reading frame 100 [Source:HGNC Symbol;Ac	chr1:24451	NM_00101	XM_01154	.	.	NP_00101	XP_011542	protein_co	ENSG0000	200159	30435
C1orf101	0.076912	0.055497	0.090145	0.058915	0.057123	0.049598	chromosome 1 open reading frame 101 [Source:HGNC Symbol;Ac	chr1:24462	NM_00113	XM_01700	.	.	NP_00112	XP_011542	protein_co	ENSG0000	257044	28491
C1orf105	0.08106	0.083998	0	0	0	0	chromosome 1 open reading frame 105 [Source:HGNC Symbol;Ac	chr1:17238	NM_00130	XM_01151	.	.	NP_00128	XP_005245	protein_co	ENSG0000	92346	29591
C1orf106	4.46977	5.67555	4.20781	4.57504	4.85214	4.48824	chromosome 1 open reading frame 106 [Source:HGNC Symbol;Ac	chr1:20086	NM_00114	XM_01150	.	.	NP_00113	XP_011508	protein_co	ENSG0000	55765	25599
C1orf109	27.5038	27.2848	24.1883	31.9521	28.3402	20.8511	chromosome 1 open reading frame 109 [Source:HGNC Symbol;Ac	chr1:38147	NM_00130	XM_01700	.	.	NP_00128	XP_005271	protein_co	ENSG0000	54955	26039
C1orf111	0	0	0	0	0	0	chromosome 1 open reading frame 111 [Source:HGNC Symbol;Ac	chr1:16234	NM_18258	XM_01150	.	.	NP_87238	XP_011507	protein_co	ENSG0000	284680	27648
C1orf112	10.3924	11.5113	10.7995	13.8596	14.4351	11.6787	chromosome 1 open reading frame 112 [Source:HGNC Symbol;Ac	chr1:16976	NM_00132	XM_01150	.	.	NP_00130	XP_005245	protein_co	ENSG0000	55732	25565
C1orf115	13.2612	13.7573	14.9292	13.4321	12.7661	14.4368	chromosome 1 open reading frame 115 [Source:HGNC Symbol;Ac	chr1:22086	NM_02470	.	.	.	NP_07898	.	protein_co	ENSG0000	79762	25873
C1orf116	1.2384	1.2427	1.20493	0.822562	0.994835	0.869715	chromosome 1 open reading frame 116 [Source:HGNC Symbol;Ac	chr1:20719	NM_00108	XM_01150	.	.	NP_00107	XP_005273	protein_co	ENSG0000	79098	28667
C1orf122	64.2461	59.2263	51.5407	69.9536	47.4523	47.4954	chromosome 1 open reading frame 122 [Source:HGNC Symbol;Ac	chr1:38268	NM_00114	.	.	.	NP_00113	.	protein_co	ENSG0000	127687	24789
C1orf123	21.3933	16.9694	19.7306	23.1827	19.9709	19.4938	chromosome 1 open reading frame 123 [Source:HGNC Symbol;Ac	chr1:53662	NM_00130	.	.	.	NP_00129	.	protein_co	ENSG0000	54987	26059
C1orf127	0.026637	0.02653	0.045889	0.028617	0.030632	0.02919	chromosome 1 open reading frame 127 [Source:HGNC Symbol;Ac	chr1:11006	NM_00117	XM_01154	.	.	NP_00116	.	protein_co	ENSG0000	148345	26730
C1orf131	12.4595	11.0345	16.1573	12.5031	11.7078	9.29988	chromosome 1 open reading frame 131 [Source:HGNC Symbol;Ac	chr1:23135	NM_00130	.	.	.	NP_00128	.	protein_co	ENSG0000	128061	25332

C1orf134	2.0201	1.05459	2.66116	4.48485	0	0	chromosome 1 open reading frame 134 [Source:HGNC Symbol;Ac	chr1:1655	.	.	.	.	.	.	protein_co	ENSG0000	.	32021
C1orf141	0	0	0	0	0	0	chromosome 1 open reading frame 141 [Source:HGNC Symbol;Ac	chr1:6755	NM_00127	XM_01154	.	.	NP_00126	XP_01153	protein_co	ENSG0000	400757	32044
C1orf145	0.115628	0.359745	0.397928	0	0.129202	0.709647	chromosome 1 open reading frame 145 [Source:HGNC Symbol;Ac	chr1:2283	.	.	.	.	.	.	protein_co	ENSG0000	.	32047
C1orf146	0	0	0	0	0	0	chromosome 1 open reading frame 146 [Source:HGNC Symbol;Ac	chr1:9268	NM_00101	XM_01154	.	.	NP_00101	XP_01153	protein_co	ENSG0000	388649	24032
C1orf158	0	0	0	0	0	0	chromosome 1 open reading frame 158 [Source:HGNC Symbol;Ac	chr1:1280	NM_15229	XM_01154	.	.	NP_68950	XP_01154	protein_co	ENSG0000	93190	28567
C1orf159	11.3104	11.065	9.48613	10.7291	10.3745	8.74029	chromosome 1 open reading frame 159 [Source:HGNC Symbol;Ac	chr1:1017	NM_01789	XM_01154	.	.	NP_06036	XP_005244	protein_co	ENSG0000	54991	26062
C1orf162	0.078523	0.526451	0.258423	0.352894	0.221064	0.299927	chromosome 1 open reading frame 162 [Source:HGNC Symbol;Ac	chr1:1120	NM_0013	XM_01154	.	.	NP_00128	XP_01153	protein_co	ENSG0000	128346	28344
C1orf167	0.050719	0.032772	0.072718	0.017868	0	0.019162	chromosome 1 open reading frame 167 [Source:HGNC Symbol;Ac	chr1:1182	NM_00101	XM_01154	.	.	NP_00101	XP_01153	protein_co	ENSG0000	.	25262
C1orf168	0	0	0	0	0	0	chromosome 1 open reading frame 168 [Source:HGNC Symbol;Ac	chr1:5718	NM_0010	XM_01700	.	.	NP_00100	XP_01153	protein_co	ENSG0000	199920	27295
C1orf174	24.3967	23.3644	20.5867	25.4993	23.0418	17.606	chromosome 1 open reading frame 174 [Source:HGNC Symbol;Ac	chr1:3805	NM_20735	.	.	.	NP_99723	.	protein_co	ENSG0000	339448	27915
C1orf185	0	0	0	0	0	0	chromosome 1 open reading frame 185 [Source:HGNC Symbol;Ac	chr1:5156	NM_00113	XM_01154	.	.	NP_00112	XP_01153	protein_co	ENSG0000	284546	28096
C1orf186	0	0	0	0	0	0	chromosome 1 open reading frame 186 [Source:HGNC Symbol;Ac	chr1:2062	NM_0010	XM_00527	.	.	NP_00100	XP_005272	protein_co	ENSG0000	440712	25341
C1orf189	0	0.290227	0	0	0	0	chromosome 1 open reading frame 189 [Source:HGNC Symbol;Ac	chr1:15417	NM_00101	.	.	.	NP_00101	.	protein_co	ENSG0000	388701	32305
C1orf194	0	0	0	0	0	0	chromosome 1 open reading frame 194 [Source:HGNC Symbol;Ac	chr1:10964	NM_00112	XM_01154	.	.	NP_00111	XP_00671	protein_co	ENSG0000	127003	32331
C1orf198	17.7891	18.1151	18.6402	20.7848	20.0445	18.8833	chromosome 1 open reading frame 198 [Source:HGNC Symbol;Ac	chr1:23097	NM_00113	.	.	.	NP_00112	XP_016858	protein_co	ENSG0000	84886	25900
C1orf204	0.739087	0.54625	1.18049	0.54027	0.403551	0.646904	chromosome 1 open reading frame 204 [Source:HGNC Symbol;Ac	chr1:1597	NM_00113	.	.	.	NP_00112	.	protein_co	ENSG0000	284677	27647
C1orf21	1.69907	1.36775	1.5984	1.53748	1.71655	1.49559	chromosome 1 open reading frame 21 [Source:HGNC Symbol;Acc	chr1:1843	NM_0308	.	.	.	NP_11043	.	protein_co	ENSG0000	81563	15494
C1orf210	6.376	7.59957	6.76362	5.51894	4.86101	6.18742	chromosome 1 open reading frame 210 [Source:HGNC Symbol;Ac	chr1:43747	NM_00116	XM_01154	.	.	NP_00115	XP_01153	protein_co	ENSG0000	149466	28755
C1orf216	12.2989	12.0244	10.7189	11.3879	11.3998	8.76674	chromosome 1 open reading frame 216 [Source:HGNC Symbol;Ac	chr1:3617	NM_15237	XM_01154	.	.	NP_68958	XP_01153	protein_co	ENSG0000	127703	26800
C1ORF220	0.034247	0.035508	0.029653	0.150919	0.038539	0.035196	chromosome 1 open reading frame 220 [Source:HGNC Symbol;Ac	chr1:17851	.	.	NR_03318	.	.	.	lincRNA	ENSG0000	400798	33805
C1orf226	3.87251	4.16752	4.47936	6.22123	6.05314	9.01488	chromosome 1 open reading frame 226 [Source:HGNC Symbol;Ac	chr1:1623	NM_00108	.	.	.	NP_00107	.	protein_co	ENSG0000	400793	34351
C1orf228	0.044775	0.318865	0.44947	0.098313	0.153214	0.277838	chromosome 1 open reading frame 228 [Source:HGNC Symbol;Ac	chr1:4514	NM_00114	XM_01154	.	.	NP_00113	XP_00671	protein_co	ENSG0000	339541	34345
C1orf234	0	0.228079	0.193268	0	0	0.212491	chromosome 1 open reading frame 234 [Source:HGNC Symbol;Ac	chr1:2333	NM_00124	.	.	.	NP_00122	.	protein_co	ENSG0000	729059	44651
C1orf27	8.26277	6.53166	7.50954	6.21999	8.25586	7.72426	chromosome 1 open reading frame 27 [Source:HGNC Symbol;Acc	chr1:1863	NM_00116	XM_01150	.	.	NP_00115	XP_011507	protein_co	ENSG0000	54953	24299
C1orf35	20.4924	20.1861	19.7683	21.5716	17.1529	15.214	chromosome 1 open reading frame 35 [Source:HGNC Symbol;Acc	chr1:2282	NM_02431	XM_01154	.	.	NP_07729	.	protein_co	ENSG0000	79169	19032
C1orf43	154.927	153.482	145.742	158.142	139.277	163.896	chromosome 1 open reading frame 43 [Source:HGNC Symbol;Acc	chr1:15417	NM_00109	XM_01150	.	.	NP_00109	.	protein_co	ENSG0000	25912	29876
C1orf50	8.60403	6.60642	6.19391	8.70128	7.14496	6.88396	chromosome 1 open reading frame 50 [Source:HGNC Symbol;Acc	chr1:4323	NM_02409	.	.	.	NP_07700	.	protein_co	ENSG0000	79078	28795
C1orf52	22.1364	22.8236	22.3915	27.9857	21.3371	19.6881	chromosome 1 open reading frame 52 [Source:HGNC Symbol;Acc	chr1:85717	NM_19807	.	NR_02411	.	NP_93234	.	protein_co	ENSG0000	148423	24871
C1orf53	3.94925	5.20306	4.9708	6.345	5.31401	3.75426	chromosome 1 open reading frame 53 [Source:HGNC Symbol;Acc	chr1:19787	NM_00102	.	.	.	NP_00101	.	protein_co	ENSG0000	388722	30003
C1orf54	1.57092	0.851373	2.81578	1.73012	1.80828	2.6796	chromosome 1 open reading frame 54 [Source:HGNC Symbol;Acc	chr1:1502	NM_0013	XM_01150	.	.	NP_00128	.	protein_co	ENSG0000	79630	26258
C1orf56	0.121336	0.165608	0.070272	0.089442	0.045749	0.042234	chromosome 1 open reading frame 56 [Source:HGNC Symbol;Acc	chr1:1510	NM_0178	.	.	.	NP_06033	.	protein_co	ENSG0000	54964	26045
C1orf61	2.57551	2.89834	2.93324	4.96601	2.37562	2.32471	chromosome 1 open reading frame 61 [Source:HGNC Symbol;Acc	chr1:15637	NM_0063	XM_01150	NR_13526	.	NP_00635	XP_006711	protein_co	ENSG0000	10485	30780
C1orf64	0	0	0	0	0	0	chromosome 1 open reading frame 64 [Source:HGNC Symbol;Acc	chr1:1633	NM_17884	.	.	.	NP_84916	.	protein_co	ENSG0000	149563	28339
C1orf68	0	0	0	0	0	0	chromosome 1 open reading frame 68 [Source:HGNC Symbol;Acc	chr1:1526	NM_00102	.	.	.	NP_00101	.	protein_co	ENSG0000	100129271	29468
C1orf74	4.67176	4.93188	4.70641	5.13482	5.4065	4.58269	chromosome 1 open reading frame 74 [Source:HGNC Symbol;Acc	chr1:2099	NM_15248	.	.	.	NP_68969	.	protein_co	ENSG0000	148304	26319
C1orf87	0.047159	0	0.053372	0.135858	0.102365	0.124253	chromosome 1 open reading frame 87 [Source:HGNC Symbol;Acc	chr1:6045	NM_15237	.	.	.	NP_68959	XP_016855	protein_co	ENSG0000	127795	28547
C1orf94	0	0	0	0	0.03723	0	chromosome 1 open reading frame 94 [Source:HGNC Symbol;Acc	chr1:3463	NM_00113	.	.	.	NP_00112	.	protein_co	ENSG0000	84970	28250
C1QA	0	0	0	0	0	0	complement C1q A chain [Source:HGNC Symbol;Acc:HGNC:1241]	chr1:2296	NM_01599	.	.	.	NP_05707	.	protein_co	ENSG0000	712	1241
C1QB	0	0	0	0	0	0	complement C1q B chain [Source:HGNC Symbol;Acc:HGNC:1242]	chr1:2297	NM_00049	XM_01154	.	.	NP_00048	XP_01154	protein_co	ENSG0000	713	1242
C1QBP	399.148	399.682	384.486	453.929	379.57	365.289	complement C1q binding protein [Source:HGNC Symbol;Acc:HGN	chr17:528	NM_00121	.	.	.	NP_00120	.	protein_co	ENSG0000	708	1243
C1QC	0	0	0	0	0	0	complement C1q C chain [Source:HGNC Symbol;Acc:HGNC:1245]	chr1:2297	NM_00111	.	.	.	NP_00110	.	protein_co	ENSG0000	714	1245
C1QL1	2.57915	2.35783	4.76063	4.81848	3.87914	4.19769	complement C1q like 1 [Source:HGNC Symbol;Acc:HGNC:24182]	chr17:430	NM_00668	.	.	.	NP_00667	.	protein_co	ENSG0000	10882	24182
C1QL2	0	0	0	0	0	0	complement C1q like 2 [Source:HGNC Symbol;Acc:HGNC:24181]	chr2:11991	NM_18252	.	.	.	NP_87233	.	protein_co	ENSG0000	165257	24181
C1QL3	0.041913	0	0	0	0	0	complement C1q like 3 [Source:HGNC Symbol;Acc:HGNC:19359]	chr10:1647	NM_00101	.	.	.	NP_00101	.	protein_co	ENSG0000	389941	19359
C1QL4	5.24256	5.05867	8.4376	6.29375	5.15225	6.01815	complement C1q like 4 [Source:HGNC Symbol;Acc:HGNC:31416]	chr12:4972	NM_0010	XM_01153	.	.	NP_00100	XP_01153	protein_co	ENSG0000	338761	31416
C1QTNF1	3.07335	3.78703	3.33293	2.51278	1.7624	2.05669	C1q and tumor necrosis factor related protein 1 [Source:HGNC Sy	chr17:7701	NM_0309	XM_00672	.	.	NP_11223	XP_006721	protein_co	ENSG0000	114897	14324
C1QTNF2	0.122567	0.186367	0.079992	0.167688	0.210234	0.194618	C1q and tumor necrosis factor related protein 2 [Source:HGNC Sy	chr5:1597	NM_0319	.	.	.	NP_11411	.	protein_co	ENSG0000	114898	14325
C1QTNF3	1.0474	0.937562	1.08771	0.90952	0.806529	0.919156	C1q and tumor necrosis factor related protein 3 [Source:HGNC Sy	chr5:3401	NM_03094	.	.	.	NP_11220	.	protein_co	ENSG0000	114899	14326
C1QTNF4	0.095096	0.356646	0.159957	0.200344	0.226975	0.333632	C1q and tumor necrosis factor related protein 4 [Source:HGNC Sy	chr11:4761	NM_0319	.	.	.	NP_11411	XP_016872	protein_co	ENSG0000	114900	14346
C1QTNF5	0	0	0	0	0	0	C1q and tumor necrosis factor related protein 5 [Source:HGNC Sy	chr11:1192	NM_00127	.	.	.	NP_00126	.	protein_co	ENSG0000	114902	14344
C1QTNF6	5.90587	6.16243	5.94446	3.65874	3.42462	3.29607	C1q and tumor necrosis factor related protein 6 [Source:HGNC Sy	chr22:3757	NM_03191	XM_01702	.	.	NP_11411	XP_011528	protein_co	ENSG0000	114904	14343
C1QTNF7	0	0	0	0	0	0	C1q and tumor necrosis factor related protein 7 [Source:HGNC Sy	chr4:15341	NM_00113	XM_01151	.	.	NP_00112	XP_011512	protein_co	ENSG0000	114905	14342
C1QTNF8	0	0	0	0	0	0	C1q and tumor necrosis factor related protein 8 [Source:HGNC Sy	chr16:114	NM_20741	XM_01152	.	.	NP_99730	XP_01152	protein_co	ENSG0000	390664	31374
C1QTNF9	0	0	0	0	0.04115	0	C1q and tumor necrosis factor related protein 9 [Source:HGNC Sy	chr13:2488	NM_0013	.	.	.	NP_00129	.	protein_co	ENSG0000	338872	28732
C1QTNF9B	0	0.052405	0	0	0	0.05593	C1q and tumor necrosis factor related protein 9B [Source:HGNC S	chr13:244	NM_0010	XM_01153	.	.	NP_00100	XP_011533	protein_co	ENSG0000	387911	34072
C1R	1.92753	1.94485	1.19759	2.48141	1.51029	1.03316	complement C1r subcomponent [Source:HGNC Symbol;Acc:HGN	chr12:718	NM_00173	XM_01701	.	.	NP_00172	XP_016875	protein_co	ENSG0000	715	1246
C1RL	7.26488	7.47387	9.07057	4.99059	6.02041	8.44544	complement C1r subcomponent like [Source:HGNC Symbol;Acc:H	chr12:724	NM_00129	XM_01701	.	.	NP_00128	XP_016874	protein_co	ENSG0000	51279	21265
C1S	0.648897	0.525531	0.546228	0.586884	0.651364	0.477039	complement component 1, s subcomponent [Source:HGNC Symb	chr12:708	NM_00173	XM_00525	.	.	NP_00172	XP_005253	protein_co	ENSG0000	716	1247
C2	0.385716	0.153722	0.370402	0.209872	0.171987	0.329346	complement component 2 [Source:HGNC Symbol;Acc:HGNC:124	chr6:3186	NM_0000	.	.	.	NP_00005	.	protein_co	ENSG0000	717	1248

C20orf141	0	0	0	0.199368	0	0	chromosome 20 open reading frame 141 [Source:HGNC Symbol;A	chr20:2795	NM_00125	.	.	.	NP_00124	.	protein_co	ENSG0000	128653	16134
C20orf144	0.236267	0	0	0	0	0	chromosome 20 open reading frame 144 [Source:HGNC Symbol;A	chr20:3224	NM_08082	.	.	.	NP_54301	.	protein_co	ENSG0000	128864	16137
C20orf173	0	0	0	0	0	0	chromosome 20 open reading frame 173 [Source:HGNC Symbol;A	chr20:3411	NM_00114	XM_00672	.	.	NP_00113	XP_006723	protein_co	ENSG0000	140873	16166
C20orf194	1.66275	2.48519	2.5272	1.82907	1.83896	2.82675	chromosome 20 open reading frame 194 [Source:HGNC Symbol;A	chr20:3229	NM_00100	XM_01152	.	.	NP_00100	XP_00526	protein_co	ENSG0000	25943	17721
C20orf196	3.89957	3.39422	2.95841	2.14982	2.16876	0.978423	chromosome 20 open reading frame 196 [Source:HGNC Symbol;A	chr20:5731	NM_00130	XM_01152	.	.	NP_00129	XP_006723	protein_co	ENSG0000	149840	26318
C20orf202	0.140755	0.097349	0.163126	0.156226	0.264427	0.193021	chromosome 20 open reading frame 202 [Source:HGNC Symbol;A	chr20:1184	NM_00100	.	.	.	NP_00100	.	protein_co	ENSG0000	400831	37254
C20orf24	79.5158	78.8343	72.8698	107.293	97.1033	81.3038	chromosome 20 open reading frame 24 [Source:HGNC Symbol;Ac	chr20:3520	NM_00119	.	.	.	NP_00118	.	protein_co	ENSG0000	55969	15870
C20orf27	54.8111	57.4456	49.4307	69.4922	55.5732	39.3716	chromosome 20 open reading frame 27 [Source:HGNC Symbol;Ac	chr20:3734	NM_00103	.	.	.	NP_00103	.	protein_co	ENSG0000	54976	15873
C20orf85	0	0	0	0	0	0	chromosome 20 open reading frame 85 [Source:HGNC Symbol;Ac	chr20:5672	NM_17845	.	.	.	NP_84855	.	protein_co	ENSG0000	128602	16216
C20orf96	9.65876	9.41362	9.34372	10.4619	9.44827	8.93369	chromosome 20 open reading frame 96 [Source:HGNC Symbol;Ac	chr20:2515	NM_08057	XM_00672	.	.	NP_54213	XP_006723	protein_co	ENSG0000	140680	16227
C21orf140	0	0	0	0	0	0	chromosome 21 open reading frame 140 [Source:HGNC Symbol;A	chr21:3574	NM_00128	.	.	.	NP_00126	XP_006724	protein_co	ENSG0000	102723451	39602
C21orf2	5.5381	5.60494	5.93881	5.52041	3.56572	4.10238	chromosome 21 open reading frame 2 [Source:HGNC Symbol;Acc	chr21:4574	NM_00127	XM_01702	.	.	NP_00125	XP_016883	protein_co	ENSG0000	755	1260
C21orf33	30.8121	46.2213	46.6929	37.0534	48.713	36.9372	chromosome 21 open reading frame 33 [Source:HGNC Symbol;Ac	chr21:4552	NM_00132	XM_01150	.	.	NP_00130	XP_005261	protein_co	ENSG0000	102724023	1273
C21orf58	3.5348	2.77162	2.30016	2.25188	2.11674	2.46895	chromosome 21 open reading frame 58 [Source:HGNC Symbol;Ac	chr21:4772	NM_00128	XM_01152	.	.	NP_00127	XP_005261	protein_co	ENSG0000	54058	1300
C21orf59	54.4031	53.4158	47.975	66.1338	51.3535	47.9256	chromosome 21 open reading frame 59 [Source:HGNC Symbol;Ac	chr21:3394	NM_02125	.	.	.	NP_06707	.	protein_co	ENSG0000	56683	1301
C21orf62	0	0	0	0	0	0	chromosome 21 open reading frame 62 [Source:HGNC Symbol;Ac	chr21:3416	NM_00116	XM_01152	.	.	NP_00115	XP_011527	protein_co	ENSG0000	56245	1305
C21orf91	4.11527	5.0972	6.09828	6.89017	8.11067	10.1391	chromosome 21 open reading frame 91 [Source:HGNC Symbol;Ac	chr21:1916	NM_00110	.	.	.	NP_00109	.	protein_co	ENSG0000	54149	16459
C22orf15	0	0	0	0	0	0	chromosome 22 open reading frame 15 [Source:HGNC Symbol;Ac	chr22:2410	NM_18252	XM_00526	.	.	NP_87232	XP_016884	protein_co	ENSG0000	150248	15558
C22orf23	0.092014	0.446144	0.431734	0.150587	0.211194	0.245052	chromosome 22 open reading frame 23 [Source:HGNC Symbol;Ac	chr22:3833	NM_00120	XM_00526	.	.	NP_00119	XP_005261	protein_co	ENSG0000	84645	18589
C22orf29	6.93589	7.64055	7.38969	7.6008	7.13901	6.3839	chromosome 22 open reading frame 29 [Source:HGNC Symbol;Ac	chr22:1974	NM_02462	.	.	.	NP_07890	.	protein_co	ENSG0000	79680	26112
C22orf31	0	0	0	0	0	0.072437	chromosome 22 open reading frame 31 [Source:HGNC Symbol;Ac	chr22:2945	NM_01537	XM_01702	.	.	NP_05618	XP_011528	protein_co	ENSG0000	25770	26931
C22orf39	19.1194	17.4559	15.3276	19.3341	14.4743	14.2818	chromosome 22 open reading frame 39 [Source:HGNC Symbol;Ac	chr22:1942	NM_00116	.	.	.	NP_00115	.	protein_co	ENSG0000	128977	27012
C22orf42	0	0	0	0	0	0	chromosome 22 open reading frame 42 [Source:HGNC Symbol;Ac	chr22:3254	NM_00101	XM_01702	.	.	NP_00101	XP_011528	protein_co	ENSG0000	150297	27160
C22orf46	4.49878	4.978	4.44379	3.12885	3.28054	3.57702	chromosome 22 open reading frame 46 [Source:HGNC Symbol;Ac	chr22:4208	NM_00114	XM_01153	.	.	NP_00113	XP_011528	protein_co	ENSG0000	79640	26294
C2CD2	7.97031	8.71551	9.30726	6.09715	6.74597	6.91928	C2 calcium dependent domain containing 2 [Source:HGNC Symbo	chr21:4330	NM_01550	XM_01702	.	.	NP_05631	XP_005261	protein_co	ENSG0000	25966	1266
C2CD2L	6.33437	6.34114	6.67697	7.31974	6.05251	6.60715	C2CD2 like [Source:HGNC Symbol;Acc:HGNC:29000]	chr11:1189	NM_00129	XM_01154	.	.	NP_00127	XP_006719	protein_co	ENSG0000	9854	29000
C2CD3	5.89032	6.58776	5.38396	6.4939	6.2576	5.75413	C2 calcium dependent domain containing 3 [Source:HGNC Symbo	chr11:7372	NM_00128	XM_01701	.	.	NP_00127	XP_011543	protein_co	ENSG0000	26005	24564
C2CD4A	0.064449	0.033148	0	0.035908	0.036477	0.032925	C2 calcium dependent domain containing 4A [Source:HGNC Symb	chr15:6235	NM_20732	.	.	.	NP_99720	.	protein_co	ENSG0000	145741	33627
C2CD4B	0	0	0	0	0	0	C2 calcium dependent domain containing 4B [Source:HGNC Symb	chr15:6245	NM_00100	.	.	.	NP_00100	XP_016877	protein_co	ENSG0000	388125	33628
C2CD4C	3.25715	4.3546	4.01147	3.39495	2.69799	3.53044	C2 calcium dependent domain containing 4C [Source:HGNC Symb	chr19:4054	NM_00113	XM_01152	.	.	NP_00112	XP_011525	protein_co	ENSG0000	126567	29417
C2CD4D	0.175538	0	0.074	0	0	0	C2 calcium dependent domain containing 4D [Source:HGNC Symb	chr1:15181	NM_00113	XM_01150	.	.	NP_00112	XP_011507	protein_co	ENSG0000	100191040	37210
C2CD5	8.91039	8.67024	9.44344	7.23728	9.87233	10.0598	C2 calcium dependent domain containing 5 [Source:HGNC Symbo	chr12:2260	NM_00128	XM_01152	.	.	NP_00127	XP_005253	protein_co	ENSG0000	9847	29062
C2orf15	1.0453	1.37507	1.31222	1.05288	1.11061	1.28123	chromosome 2 open reading frame 15 [Source:HGNC Symbol;Acc	chr2:99613	NM_14470	.	.	.	NP_00130	.	protein_co	ENSG0000	150590	28436
C2orf16	0	0	0	0	0.010526	0	chromosome 2 open reading frame 16 [Source:HGNC Symbol;Acc	chr2:27799	NM_03226	.	.	.	NP_11564	.	protein_co	ENSG0000	84226	25275
C2orf40	0	0	0	0	0	0	chromosome 2 open reading frame 40 [Source:HGNC Symbol;Acc	chr2:10667	NM_03241	XM_01151	.	.	NP_11578	XP_006712	protein_co	ENSG0000	84417	24642
C2orf42	4.52221	4.12944	3.74191	4.68974	5.47429	6.53439	chromosome 2 open reading frame 42 [Source:HGNC Symbol;Acc	chr2:70377	NM_01788	XM_01700	.	.	NP_06035	XP_005264	protein_co	ENSG0000	54980	26056
C2orf47	25.8375	22.1899	22.784	28.0507	23.6709	21.4828	chromosome 2 open reading frame 47 [Source:HGNC Symbol;Acc	chr2:20079	NM_02452	.	.	.	NP_07879	.	protein_co	ENSG0000	79568	26198
C2orf49	10.7598	11.3726	12.5319	9.97079	10.3698	9.56279	chromosome 2 open reading frame 49 [Source:HGNC Symbol;Acc	chr2:10595	NM_00128	XM_00526	.	.	NP_00127	XP_005264	protein_co	ENSG0000	79074	28772
C2orf50	0.24854	0.566295	0.176912	0.191496	0.23302	0.123841	chromosome 2 open reading frame 50 [Source:HGNC Symbol;Acc	chr2:11273	NM_18250	XM_00671	.	.	NP_87230	XP_006711	protein_co	ENSG0000	130813	26324
C2orf54	0.130891	0.130937	0.078785	0.097916	0.100283	0.051654	chromosome 2 open reading frame 54 [Source:HGNC Symbol;Acc	chr2:24182	NM_00108	XM_01151	.	.	NP_00107	XP_011510	protein_co	ENSG0000	79919	26216
C2orf57	0	0	0	0	0	0	chromosome 2 open reading frame 57 [Source:HGNC Symbol;Acc	chr2:23245	NM_15261	.	.	.	NP_68982	.	protein_co	ENSG0000	165100	28563
C2orf61	0	0.09757	0.081762	0	0	0	chromosome 2 open reading frame 61 [Source:HGNC Symbol;Acc	chr2:47314	NM_00116	.	.	.	NP_00115	.	protein_co	ENSG0000	285051	26850
C2orf66	0	0.284382	0.226087	0	0.070791	0	chromosome 2 open reading frame 66 [Source:HGNC Symbol;Acc	chr2:19766	NM_21360	.	.	.	NP_99877	.	protein_co	ENSG0000	401027	33809
C2orf68	7.56405	7.95003	7.23616	5.43342	5.86602	7.68114	chromosome 2 open reading frame 68 [Source:HGNC Symbol;Acc	chr2:85833	NM_00101	XM_00526	.	.	NP_00101	XP_005264	protein_co	ENSG0000	388969	34353
C2orf69	8.86099	9.75254	9.66488	12.8595	15.7503	13.0911	chromosome 2 open reading frame 69 [Source:HGNC Symbol;Acc	chr2:20077	NM_15368	.	.	.	NP_71015	.	protein_co	ENSG0000	205327	26799
C2orf70	3.57559	3.66095	4.87252	7.18427	2.37772	1.95475	chromosome 2 open reading frame 70 [Source:HGNC Symbol;Acc	chr2:26785	NM_00110	XM_00526	.	.	NP_00109	.	protein_co	ENSG0000	339778	27938
C2orf71	0	0	0	0	0	0	chromosome 2 open reading frame 71 [Source:HGNC Symbol;Acc	chr2:29284	NM_00102	XM_01153	.	.	NP_00102	XP_011531	protein_co	ENSG0000	388939	34383
C2orf72	3.33772	3.55346	3.91969	4.04807	3.80146	2.97703	chromosome 2 open reading frame 72 [Source:HGNC Symbol;Acc	chr2:23190	NM_00114	.	.	.	NP_00113	.	protein_co	ENSG0000	257407	27418
C2orf73	0.11823	0	0.10314	0.06716	0.19586	0.172266	chromosome 2 open reading frame 73 [Source:HGNC Symbol;Acc	chr2:54558	NM_00110	XM_01153	.	.	NP_00109	XP_011530	protein_co	ENSG0000	129852	26861
C2orf74	0	0.168419	0.119447	0.070726	0.088827	0.133861	chromosome 2 open reading frame 74 [Source:HGNC Symbol;Acc	chr2:61372	NM_00114	XM_00526	.	.	NP_00113	XP_005264	protein_co	ENSG0000	339804	34439
C2orf76	7.7762	8.0394	7.67592	9.62989	6.79421	8.04982	chromosome 2 open reading frame 76 [Source:HGNC Symbol;Acc	chr2:12005	NM_00101	XM_01151	.	.	NP_00101	XP_005263	protein_co	ENSG0000	130355	27017
C2orf78	0.245008	0.093296	0.600318	0.695708	0.598821	2.39632	chromosome 2 open reading frame 78 [Source:HGNC Symbol;Acc	chr2:74011	NM_00108	XM_00526	.	.	NP_00107	XP_005264	protein_co	ENSG0000	388960	34349
C2orf80	0	0	0	0	0	0	chromosome 2 open reading frame 80 [Source:HGNC Symbol;Acc	chr2:20903	NM_00109	XM_01700	.	.	NP_00109	XP_005246	protein_co	ENSG0000	389073	34352
C2orf81	2.30087	2.00071	2.34434	1.86223	1.40395	1.68966	chromosome 2 open reading frame 81 [Source:HGNC Symbol;Acc	chr2:74641	NM_00114	.	.	.	NP_00113	.	protein_co	ENSG0000	388963	34350
C2orf82	2.29222	1.97866	2.68803	1.54632	2.2976	2.35035	chromosome 2 open reading frame 82 [Source:HGNC Symbol;Acc	chr2:23373	NM_20689	.	.	.	NP_99677	XP_016859	protein_co	ENSG0000	389084	33763
C2orf83	0	0	0	0	0	0	chromosome 2 open reading frame 83 [Source:HGNC Symbol;Acc	chr2:22847	NM_00116	.	.	.	NP_00115	.	protein_co	ENSG0000	56918	25344
C2orf88	1.39936	1.31571	2.02641	2.18	1.68555	1.48306	chromosome 2 open reading frame 88 [Source:HGNC Symbol;Acc	chr2:19100	NM_00104	XM_01700	.	.	NP_00103	XP_005246	protein_co	ENSG0000	84281	28191
C2orf91	0	0	0	0	0	0	chromosome 2 open reading frame 91 [Source:HGNC Symbol;Acc	chr2:42162	NM_00124	.	.	.	NP_00122	.	protein_co	ENSG0000	400950	42966
C3	0.871764	0.716406	1.08247	0.196362	0.169336	0.281459	complement component 3 [Source:HGNC Symbol;Acc:HGNC:1318	chr19:6677	NM_00006	.	.	.	NP_00005	.	protein_co	ENSG0000	718	1318

C3AR1	0	0	0	0	0	0	complement component 3a receptor 1 [Source:HGNC Symbol;Acc	chr12:8210	NM_00405	.	.	.	NP_00131	.	protein_co	ENSG0000	719	1319
C3orf14	0	0	0.238527	0	0	0	chromosome 3 open reading frame 14 [Source:HGNC Symbol;Acc	chr3:62304	NM_00129	XM_01153	.	.	NP_00127	XP_011532	protein_co	ENSG0000	57415	25024
C3orf18	5.80601	4.97888	5.92655	5.09242	3.86764	3.29717	chromosome 3 open reading frame 18 [Source:HGNC Symbol;Acc	chr3:50595	NM_00117	XM_01153	.	.	NP_00116	XP_011532	protein_co	ENSG0000	51161	24837
C3orf20	0.039653	0.047452	0.020277	0.025419	0.067716	0.083877	chromosome 3 open reading frame 20 [Source:HGNC Symbol;Acc	chr3:14716	NM_00118	XM_01153	.	.	NP_00117	XP_006713	protein_co	ENSG0000	84077	25320
C3orf22	0	0	0	0	0	0	chromosome 3 open reading frame 22 [Source:HGNC Symbol;Acc	chr3:12626	NM_15253	XM_01700	.	.	NP_68974	XP_011510	protein_co	ENSG0000	152065	28534
C3orf30	0	0	0	0	0	0	chromosome 3 open reading frame 30 [Source:HGNC Symbol;Acc	chr3:11886	NM_15253	.	.	.	NP_68975	.	protein_co	ENSG0000	152405	26553
C3orf33	2.26189	3.1962	3.33508	3.36726	2.96077	3.89214	chromosome 3 open reading frame 33 [Source:HGNC Symbol;Acc	chr3:15548	NM_00130	XM_01151	.	.	NP_00129	XP_005247	protein_co	ENSG0000	285315	26434
C3orf36	0	0	0	0	0	0.042072	chromosome 3 open reading frame 36 [Source:HGNC Symbol;Acc	chr3:13364	NM_02504	.	.	.	NP_07931	.	protein_co	ENSG0000	80111	26170
C3orf38	12.465	11.6728	12.6992	12.8898	13.2297	13.5955	chromosome 3 open reading frame 38 [Source:HGNC Symbol;Acc	chr3:88101	NM_17382	XM_00526	.	.	NP_77618	XP_005264	protein_co	ENSG0000	285237	28384
C3orf49	0	0	0.04233	0	0	0	chromosome 3 open reading frame 49 [Source:HGNC Symbol;Acc	chr3:63805	.	.	.	.	.	.	protein_co	ENSG0000	.	25190
C3orf52	10.635	11.1426	11.1232	10.3765	12.5008	8.57639	chromosome 3 open reading frame 52 [Source:HGNC Symbol;Acc	chr3:11180	NM_00117	XM_01151	.	.	NP_00116	XP_011511	protein_co	ENSG0000	79669	26255
C3orf58	4.92711	5.4419	5.78327	4.48787	5.35905	4.03096	chromosome 3 open reading frame 58 [Source:HGNC Symbol;Acc	chr3:14369	NM_00113	XM_01700	.	.	NP_00112	XP_016861	protein_co	ENSG0000	205428	28490
C3orf62	3.71904	3.6527	3.75614	3.33367	3.81798	3.68849	chromosome 3 open reading frame 62 [Source:HGNC Symbol;Acc	chr3:49306	NM_19856	.	.	.	NP_94096	.	protein_co	ENSG0000	375341	24771
C3orf67	1.49715	1.69049	1.95689	1.80618	2.5956	2.70934	chromosome 3 open reading frame 67 [Source:HGNC Symbol;Acc	chr3:58727	NM_19846	XM_01700	.	.	NP_94086	XP_005264	protein_co	ENSG0000	200844	24763
C3orf70	0	0	0	0	0	0	chromosome 3 open reading frame 70 [Source:HGNC Symbol;Acc	chr3:18479	NM_00102	.	.	.	NP_00102	.	protein_co	ENSG0000	285382	33731
C3orf80	2.35995	2.32138	1.63576	2.03067	1.96576	2.87373	chromosome 3 open reading frame 80 [Source:HGNC Symbol;Acc	chr3:15994	NM_00116	.	.	.	NP_00116	.	protein_co	ENSG0000	401097	40048
C3orf84	0	0	0	0	0	0	chromosome 3 open reading frame 84 [Source:HGNC Symbol;Acc	chr3:49215	NM_00108	XM_01153	.	.	NP_00107	XP_011532	protein_co	ENSG0000	646498	44666
C4A	0.013089	0.026232	0.011309	0	0	0.014146	complement component 4A (Rodgers blood group) [Source:HGN	chr6:31949	NM_00125	.	.	.	NP_00123	.	protein_co	ENSG0000	720	1323
C4B	0.02639	0.066105	0.022817	0.02855	0.015144	0	complement component 4B (Chido blood group) [Source:HGNC S	chr6:31982	NM_00100	.	.	.	NP_00100	.	protein_co	ENSG0000	721	1324
C4BPA	0	0	0	0	0	0	complement component 4 binding protein alpha [Source:HGNC S	chr1:20727	NM_00071	XM_00527	.	.	NP_00070	XP_005273	protein_co	ENSG0000	722	1325
C4BPB	0	0	0.075712	0	0	0	complement component 4 binding protein beta [Source:HGNC Sy	chr1:20726	NM_00071	XM_00527	.	.	NP_00070	XP_005273	protein_co	ENSG0000	725	1328
C4orf17	0	0	0	0	0	0	chromosome 4 open reading frame 17 [Source:HGNC Symbol;Acc	chr4:10043	NM_03214	.	.	.	NP_11552	XP_011530	protein_co	ENSG0000	84103	25274
C4orf19	0	0	0	0	0	0	chromosome 4 open reading frame 19 [Source:HGNC Symbol;Acc	chr4:37455	NM_00110	XM_01151	.	.	NP_00109	XP_011512	protein_co	ENSG0000	55286	25618
C4orf22	0.099673	0	0	0.18637	0	0.083282	chromosome 4 open reading frame 22 [Source:HGNC Symbol;Acc	chr4:81256	NM_00120	XM_01153	.	.	NP_00119	XP_016863	protein_co	ENSG0000	255119	28554
C4orf26	0.053215	0.053976	0	0.22534	0	0.322697	chromosome 4 open reading frame 26 [Source:HGNC Symbol;Acc	chr4:76481	NM_00120	XM_01153	.	.	NP_00119	XP_011529	protein_co	ENSG0000	152816	26300
C4orf3	46.7991	40.3094	48.0916	53.3855	47.3184	46.64	chromosome 4 open reading frame 3 [Source:HGNC Symbol;Acc:	chr4:12021	NM_00100	.	.	.	NP_00100	.	protein_co	ENSG0000	401152	19225
C4orf32	2.42683	2.43266	1.95021	2.77208	3.14522	4.41136	chromosome 4 open reading frame 32 [Source:HGNC Symbol;Acc	chr4:11306	NM_15240	.	.	.	NP_68961	.	protein_co	ENSG0000	132720	26813
C4orf33	5.12836	6.42291	6.67479	5.57252	4.5317	8.57153	chromosome 4 open reading frame 33 [Source:HGNC Symbol;Acc	chr4:12980	NM_00109	XM_00526	.	.	NP_00109	XP_005262	protein_co	ENSG0000	132321	27025
C4orf36	1.2432	1.42081	1.36143	0.669518	1.31653	2.30054	chromosome 4 open reading frame 36 [Source:HGNC Symbol;Acc	chr4:87797	NM_14464	XM_01153	NR_03884	.	NP_65324	XP_011529	protein_co	ENSG0000	100506746	28386
C4orf45	0	0	0	0	0	0	chromosome 4 open reading frame 45 [Source:HGNC Symbol;Acc	chr4:15969	NM_15254	.	.	.	NP_68975	XP_016863	protein_co	ENSG0000	152940	26342
C4orf46	11.7972	11.5328	10.9703	15.0225	17.5435	17.6394	chromosome 4 open reading frame 46 [Source:HGNC Symbol;Acc	chr4:15958	NM_00100	.	.	.	NP_00100	.	protein_co	ENSG0000	201725	27320
C4orf47	0.644317	0.548038	0.79678	0.731821	0.723837	0.635523	chromosome 4 open reading frame 47 [Source:HGNC Symbol;Acc	chr4:18635	NM_00111	XM_01153	.	.	NP_00110	XP_005263	protein_co	ENSG0000	441054	34346
C4orf48	82.8048	96.3014	98.053	129.144	76.2984	103.746	chromosome 4 open reading frame 48 [Source:HGNC Symbol;Acc	chr4:20437	NM_00114	.	.	.	NP_00113	.	protein_co	ENSG0000	401115	34437
C4orf50	0	0	0.024229	0	0.014264	0.019802	chromosome 4 open reading frame 50 [Source:HGNC Symbol;Acc	chr4:58993	.	XM_01151	.	.	.	XP_011511	protein_co	ENSG0000	.	33766
C4orf51	0	0	0	0	0	0	chromosome 4 open reading frame 51 [Source:HGNC Symbol;Acc	chr4:14660	NM_00108	XM_01153	.	.	NP_00107	XP_006714	protein_co	ENSG0000	646603	37264
C5	0.108067	0.101834	0.082259	0.123539	0.059668	0.183904	complement component 5 [Source:HGNC Symbol;Acc:HGNC:133	chr9:12371	NM_00131	XM_01701	.	.	NP_00130	XP_011517	protein_co	ENSG0000	727	1331
C5AR1	1.47271	1.54983	1.65086	1.95069	1.25065	1.37334	complement component 5a receptor 1 [Source:HGNC Symbol;Acc	chr19:4781	NM_00173	XM_00525	.	.	NP_00172	XP_005259	protein_co	ENSG0000	728	1338
C5AR2	0.103187	0.037901	0.273883	0.115457	0.083349	0.375729	complement component 5a receptor 2 [Source:HGNC Symbol;Acc	chr19:4783	NM_00127	XM_01152	.	.	NP_00125	XP_011525	protein_co	ENSG0000	27202	4527
C5orf15	23.8442	26.5886	21.5585	26.8145	20.9551	22.8632	chromosome 5 open reading frame 15 [Source:HGNC Symbol;Acc	chr5:13329	NM_02019	.	.	.	NP_06458	.	protein_co	ENSG0000	56951	20656
C5orf22	12.3022	12.125	12.2092	11.5949	14.8494	12.3823	chromosome 5 open reading frame 22 [Source:HGNC Symbol;Acc	chr5:31532	NM_01835	XM_01700	.	.	NP_06082	XP_006714	protein_co	ENSG0000	55322	25639
C5orf24	16.8156	19.3652	18.2787	20.0237	21.9553	19.8409	chromosome 5 open reading frame 24 [Source:HGNC Symbol;Acc	chr5:13418	NM_00113	XM_01700	.	.	NP_00112	XP_005271	protein_co	ENSG0000	134553	26746
C5orf30	9.25798	8.46435	9.45368	9.67145	9.44619	8.03994	chromosome 5 open reading frame 30 [Source:HGNC Symbol;Acc	chr5:10259	NM_00131	XM_01154	.	.	NP_00130	XP_005272	protein_co	ENSG0000	90355	25052
C5orf34	2.87843	3.35905	2.12508	2.70319	4.08166	4.11897	chromosome 5 open reading frame 34 [Source:HGNC Symbol;Acc	chr5:43486	NM_19856	.	.	.	NP_94096	.	protein_co	ENSG0000	375444	24738
C5orf38	0	0	0	0	0	0	chromosome 5 open reading frame 38 [Source:HGNC Symbol;Acc	chr5:27522	NM_00129	XM_01151	.	.	NP_00128	XP_005248	protein_co	ENSG0000	153571	24226
C5orf42	1.00744	0.947623	1.05629	0.600205	0.850036	0.878479	chromosome 5 open reading frame 42 [Source:HGNC Symbol;Acc	chr5:37106	NM_02307	XM_01151	.	.	NP_07556	XP_005248	protein_co	ENSG0000	65250	25801
C5orf45	15.5952	12.5757	14.0289	17.0769	10.9656	11.538	chromosome 5 open reading frame 45 [Source:HGNC Symbol;Acc	chr5:17924	NM_00101	.	.	.	NP_00101	.	protein_co	ENSG0000	51149	30817
C5orf46	0	0	0.145965	0	0	0	chromosome 5 open reading frame 46 [Source:HGNC Symbol;Acc	chr5:14727	NM_20696	XM_00526	.	.	NP_99684	XP_005268	protein_co	ENSG0000	389336	33768
C5orf47	0.043814	0	0	0	0	0	chromosome 5 open reading frame 47 [Source:HGNC Symbol;Acc	chr5:17341	NM_00114	.	.	.	NP_00113	XP_016864	protein_co	ENSG0000	133491	27026
C5orf49	0	0	0	0	0	0	chromosome 5 open reading frame 49 [Source:HGNC Symbol;Acc	chr5:78304	NM_00108	.	.	.	NP_00108	.	protein_co	ENSG0000	134121	27028
C5orf51	11.7584	11.0227	10.9986	7.00706	11.6226	11.6952	chromosome 5 open reading frame 51 [Source:HGNC Symbol;Acc	chr5:41904	NM_17592	XM_01151	.	.	NP_78711	XP_011512	protein_co	ENSG0000	285636	27750
C5orf52	0	0	0	0	0	0	chromosome 5 open reading frame 52 [Source:HGNC Symbol;Acc	chr5:15709	NM_00114	XM_01153	.	.	NP_00113	XP_011532	protein_co	ENSG0000	100190949	35121
C5orf56	1.18901	2.10244	1.50868	1.87519	1.58384	1.34905	chromosome 5 open reading frame 56 [Source:HGNC Symbol;Acc	chr5:13174	.	.	.	.	.	.	protein_co	ENSG0000	.	33838
C5orf58	0	0	0	0	0	0	chromosome 5 open reading frame 58 [Source:HGNC Symbol;Acc	chr5:16965	NM_00110	XM_01700	.	.	NP_00109	XP_016864	protein_co	ENSG0000	133874	37272
C5orf63	3.10881	3.4569	2.69956	2.49505	2.87894	2.53809	chromosome 5 open reading frame 63 [Source:HGNC Symbol;Acc	chr5:12637	NM_00116	.	.	.	NP_00115	.	protein_co	ENSG0000	401207	40051
C5orf67	0	0	0	0	0	0	chromosome 5 open reading frame 67 [Source:HGNC Symbol;Acc	chr5:55807	NM_00128	.	.	.	NP_00127	XP_016864	protein_co	ENSG0000	101928448	51252
C6	0	0	0	0	0	0	complement component 6 [Source:HGNC Symbol;Acc:HGNC:133	chr5:41142	NM_00006	XM_01151	.	.	NP_00005	XP_005248	protein_co	ENSG0000	729	1339
C6orf1	14.9492	12.9144	17.5365	9.58337	7.65947	5.37446	chromosome 6 open reading frame 1 [Source:HGNC Symbol;Acc:	chr6:34214	NM_00100	.	.	.	NP_00100	.	protein_co	ENSG0000	221491	1340
C6orf10	0	0	0	0	0	0	chromosome 6 open reading frame 10 [Source:HGNC Symbol;Acc	chr6:32256	NM_00128	XM_01151	.	.	.	XP_011512	protein_co	ENSG0000	10665	13922
C6orf106	25.1332	25.4471	25.3916	29.4423	27.0753	27.1976	chromosome 6 open reading frame 106 [Source:HGNC Symbol;Ac	chr6:34555	NM_02275	.	.	.	NP_07359	XP_005249	protein_co	ENSG0000	64771	21215

C6orf118	0.038804	0	0	0	0	0	chromosome 6 open reading frame 118 [Source:HGNC Symbol;Ac	chr6:16569	NM_14498	XM_00526	.	.	NP_659417	XP_005266	protein_co	ENSG0000	168090	21233
C6orf120	12.1672	12.5142	14.5337	12.084	16.337	18.4143	chromosome 6 open reading frame 120 [Source:HGNC Symbol;Ac	chr6:17010	NM_00102	.	.	.	NP_001025	.	protein_co	ENSG0000	387263	21247
C6orf132	5.07023	4.45324	4.35049	7.18762	6.11461	5.01789	chromosome 6 open reading frame 132 [Source:HGNC Symbol;Ac	chr6:42068	NM_00116	.	.	.	NP_001157	.	protein_co	ENSG0000	647024	21288
C6orf136	15.975	15.3565	12.3406	14.7093	13.2929	8.68638	chromosome 6 open reading frame 136 [Source:HGNC Symbol;Ac	chr6:30614	NM_00110	XM_01151	.	.	NP_00110	XP_011512	protein_co	ENSG0000	221545	21301
C6orf141	9.03239	9.34909	7.18939	7.47433	6.08062	7.92348	chromosome 6 open reading frame 141 [Source:HGNC Symbol;Ac	chr6:49518	NM_00114	XM_01151	.	.	NP_00113	XP_005248	protein_co	ENSG0000	135398	21351
C6orf15	0	0	0	0	0	0	chromosome 6 open reading frame 15 [Source:HGNC Symbol;Acc	chr6:31078	NM_01407	.	.	.	NP_05478	.	protein_co	ENSG0000	29113	13927
C6orf163	0.093188	0.348579	0.5333	0.520958	0.314989	0.623844	chromosome 6 open reading frame 163 [Source:HGNC Symbol;Ac	chr6:88054	NM_00101	XM_01153	.	.	NP_00101	XP_005248	protein_co	ENSG0000	206412	21403
C6orf201	0	0	0	0	0	0	chromosome 6 open reading frame 201 [Source:HGNC Symbol;Ac	chr6:40685	NM_00108	.	.	.	NP_00107	XP_011512	protein_co	ENSG0000	404220	21620
C6orf203	13.1149	11.9096	13.3238	9.05835	12.219	10.8649	chromosome 6 open reading frame 203 [Source:HGNC Symbol;Ac	chr6:10734	NM_00114	XM_01153	.	.	NP_00113	XP_011534	protein_co	ENSG0000	51250	17971
C6orf222	0.051519	0.052311	0	0	0	0	chromosome 6 open reading frame 222 [Source:HGNC Symbol;Ac	chr6:36283	NM_00101	XM_01151	.	.	NP_00101	XP_011512	protein_co	ENSG0000	389384	33769
C6orf223	4.54532	5.15828	8.50111	5.64067	5.34563	11.2259	chromosome 6 open reading frame 223 [Source:HGNC Symbol;Ac	chr6:43968	NM_00117	XM_01151	.	.	NP_00116	XP_011512	protein_co	ENSG0000	221416	28692
C6orf226	11.4622	9.11226	7.84929	13.0208	8.07117	11.1222	chromosome 6 open reading frame 226 [Source:HGNC Symbol;Ac	chr6:42858	NM_00100	.	.	.	NP_00100	.	protein_co	ENSG0000	441150	34431
C6orf229	0	0	0	0	0	0	chromosome 6 open reading frame 229 [Source:HGNC Symbol;Ac	chr6:24797	NM_00128	.	.	.	NP_00126	.	protein_co	ENSG0000	101928603	49394
C6orf25	0.402053	0.201878	0.183873	0.047198	0	0.090517	chromosome 6 open reading frame 25 [Source:HGNC Symbol;Acc	chr6:31691	NM_02526	.	.	.	NP_07953	.	protein_co	ENSG0000	80739	13937
C6orf47	10.689	11.2359	9.05444	9.13059	9.55543	8.34174	chromosome 6 open reading frame 47 [Source:HGNC Symbol;Acc	chr6:31626	NM_02118	.	.	.	NP_06700	.	protein_co	ENSG0000	57827	19076
C6orf48	246.89	229.306	173.526	215.305	187.723	212.906	chromosome 6 open reading frame 48 [Source:HGNC Symbol;Acc	chr6:31802	NM_00104	.	.	.	NP_00103	.	protein_co	ENSG0000	50854	19078
C6ORF50	0	0	0	0	0	0	.	chr6:39829	.	.	.	.	.	.	protein_co	ENSG0000	.	.
C6orf52	1.91734	3.56132	3.24505	5.6889	2.92507	2.65794	chromosome 6 open reading frame 52 [Source:HGNC Symbol;Acc	chr6:10671	NM_00114	XM_00671	.	.	NP_00113	.	protein_co	ENSG0000	347744	20881
C6orf58	0.895719	0.401003	0.327905	0.170525	0.082539	0.072767	chromosome 6 open reading frame 58 [Source:HGNC Symbol;Acc	chr6:12789	NM_00101	.	.	.	NP_00101	.	protein_co	ENSG0000	352999	20960
C6orf62	79.4423	75.6241	63.0712	56.3111	69.4329	70.7947	chromosome 6 open reading frame 62 [Source:HGNC Symbol;Acc	chr6:24667	NM_03093	XM_01151	.	.	NP_11220	XP_011513	protein_co	ENSG0000	81688	20998
C6orf89	20.4889	23.1663	21.6698	23.2624	22.4356	20.2808	chromosome 6 open reading frame 89 [Source:HGNC Symbol;Acc	chr6:36822	NM_00128	XM_01151	.	.	NP_00127	XP_011512	protein_co	ENSG0000	221477	21114
C7	0	0	0	0	0.019754	0	complement component 7 [Source:HGNC Symbol;Acc:HGNC:134	chr5:40909	NM_00058	XM_01151	.	.	NP_00057	.	protein_co	ENSG0000	730	1346
C7orf25	8.80806	9.03618	7.67397	7.62678	8.47231	6.85526	chromosome 7 open reading frame 25 [Source:HGNC Symbol;Acc	chr7:42948	NM_00109	XM_00524	.	.	NP_00109	XP_005249	protein_co	ENSG0000	79020	21703
C7orf26	12.0828	12.5607	10.5521	14.2564	11.7502	10.9626	chromosome 7 open reading frame 26 [Source:HGNC Symbol;Acc	chr7:66296	NM_00130	.	.	.	NP_00128	.	protein_co	ENSG0000	79034	21702
C7orf31	0.6346	0.529228	0.673181	0.746462	0.832687	1.16007	chromosome 7 open reading frame 31 [Source:HGNC Symbol;Acc	chr7:25174	NM_13881	XM_00524	.	.	NP_62016	XP_005249	protein_co	ENSG0000	136895	21722
C7orf33	0	0	0	0	0	0	chromosome 7 open reading frame 33 [Source:HGNC Symbol;Acc	chr7:14828	NM_14530	.	.	.	NP_66034	.	protein_co	ENSG0000	202865	21724
C7orf34	0	0	0	0	0	0	chromosome 7 open reading frame 34 [Source:HGNC Symbol;Acc	chr7:14263	NM_17882	.	.	.	NP_84915	.	protein_co	ENSG0000	135927	21750
C7orf43	10.557	11.5959	11.4894	9.65088	9.46573	7.21739	chromosome 7 open reading frame 43 [Source:HGNC Symbol;Acc	chr7:99752	NM_00130	.	NR_03980	.	NP_00129	.	protein_co	ENSG0000	100616439	25604
C7orf49	33.8143	31.69	28.7038	34.9083	29.9491	28.7702	chromosome 7 open reading frame 49 [Source:HGNC Symbol;Acc	chr7:13483	NM_00124	.	.	.	NP_00123	XP_016868	protein_co	ENSG0000	78996	22432
C7orf50	43.3862	45.0005	35.0101	59.0232	39.9372	41.2491	chromosome 7 open reading frame 50 [Source:HGNC Symbol;Acc	chr7:10366	NM_00113	XM_00524	NR_13453	.	NP_00112	XP_005249	protein_co	ENSG0000	84310	22421
C7orf55	11.1509	12.3309	10.3943	15.0692	11.9259	9.7758	formation of mitochondrial complex V assembly factor 1 homolog	chr7:13902	NM_19796	.	.	.	NP_93206	.	protein_co	ENSG0000	154791	26946
C7orf55-LUC7L2	11.4368	12.8645	8.91549	14.6588	7.28635	4.97761	C7orf55-LUC7L2 readthrough [Source:HGNC Symbol;Acc:HGNC:4	chr7:13902	NM_00124	.	.	.	NP_00123	.	protein_co	ENSG0000	100996928	44671
C7orf57	0.048448	0.05064	0.042015	0.164007	0.054364	0.097303	chromosome 7 open reading frame 57 [Source:HGNC Symbol;Acc	chr7:48075	NM_00110	XM_01151	.	.	NP_00109	XP_006715	protein_co	ENSG0000	136288	22247
C7orf61	0.406911	0.90411	0.77554	0.532665	0.745285	1.04495	chromosome 7 open reading frame 61 [Source:HGNC Symbol;Acc	chr7:10005	NM_00100	.	.	.	NP_00100	.	protein_co	ENSG0000	402573	22135
C7orf62	0	0	0	0	0	0	chromosome 7 open reading frame 62 [Source:HGNC Symbol;Acc	chr7:88388	NM_15270	XM_01151	.	.	NP_68991	XP_011514	protein_co	ENSG0000	219557	22402
C7orf72	0	0	0	0	0	0	chromosome 7 open reading frame 72 [Source:HGNC Symbol;Acc	chr7:50135	NM_00116	XM_01151	.	.	NP_00115	XP_011513	protein_co	ENSG0000	100130988	22564
C7orf73	26.5502	28.5184	27.2935	31.0283	28.3233	28.5109	chromosome 7 open reading frame 73 [Source:HGNC Symbol;Acc	chr7:13534	NM_00113	.	.	.	NP_00112	.	protein_co	ENSG0000	647087	41909
C7orf77	0	0	0	0	0	0.169518	chromosome 7 open reading frame 77 [Source:HGNC Symbol;Acc	chr7:12441	NM_00102	.	.	.	NP_00101	.	protein_co	ENSG0000	154872	51256
C8A	0	0	0	0	0	0	complement component 8 alpha subunit [Source:HGNC Symbol;A	chr1:57320	NM_00056	.	.	.	NP_00055	.	protein_co	ENSG0000	731	1352
C8B	0	0	0	0	0	0	complement component 8, beta polypeptide [Source:HGNC Symb	chr1:57394	NM_00006	XM_01700	.	.	NP_00005	XP_016857	protein_co	ENSG0000	732	1353
C8G	0.249273	0.733306	1.38762	1.06475	0.434684	0.552552	complement component 8, gamma polypeptide [Source:HGNC Sy	chr9:13983	NM_00060	.	.	.	NP_00059	.	protein_co	ENSG0000	733	1354
C8orf22	0	0	0	0	0	0	chromosome 8 open reading frame 22 [Source:HGNC Symbol;Acc	chr8:49966	NM_00100	.	.	.	NP_00100	.	protein_co	ENSG0000	492307	31745
C8orf33	66.4492	70.7534	56.0552	58.3829	54.0291	37.813	chromosome 8 open reading frame 33 [Source:HGNC Symbol;Acc	chr8:14627	NM_02308	.	.	.	NP_07556	.	protein_co	ENSG0000	65265	26104
C8orf34	0	0	0	0	0	0	chromosome 8 open reading frame 34 [Source:HGNC Symbol;Acc	chr8:69242	NM_00119	XM_01151	.	.	NP_00118	XP_011515	protein_co	ENSG0000	116328	30905
C8orf37	3.60891	4.18264	4.39819	4.61559	5.06604	5.76323	chromosome 8 open reading frame 37 [Source:HGNC Symbol;Acc	chr8:96257	NM_17796	.	.	.	NP_80888	.	protein_co	ENSG0000	157657	27232
C8orf4	0	0	0	0	0	0	chromosome 8 open reading frame 4 [Source:HGNC Symbol;Acc:	chr8:40010	NM_02013	.	.	.	NP_06451	.	protein_co	ENSG0000	56892	1357
C8orf44	1.37541	1.18644	2.01203	1.56559	0.608732	1.007	chromosome 8 open reading frame 44 [Source:HGNC Symbol;Acc	chr8:67579	NM_01960	.	.	.	NP_06255	.	protein_co	ENSG0000	56260	25646
C8orf44-SGK3	0.438181	0.270217	0.619229	0.317771	0.595933	0.345799	C8orf44-SGK3 readthrough [Source:HGNC Symbol;Acc:HGNC:483	chr8:67579	NM_00120	.	.	.	NP_00119	.	protein_co	ENSG0000	100533105	48354
C8orf46	0.044738	0.165519	0.077669	0.050526	0.07586	0.129254	chromosome 8 open reading frame 46 [Source:HGNC Symbol;Acc	chr8:67405	NM_15276	.	.	.	NP_68997	.	protein_co	ENSG0000	254778	28498
C8orf48	0	0	0	0	0	0	chromosome 8 open reading frame 48 [Source:HGNC Symbol;Acc	chr8:13424	NM_00100	.	.	.	NP_00100	.	protein_co	ENSG0000	157773	26345
C8orf58	5.16435	5.52949	6.62593	5.26652	4.51761	4.75137	chromosome 8 open reading frame 58 [Source:HGNC Symbol;Acc	chr8:22436	NM_00101	.	.	.	NP_00101	.	protein_co	ENSG0000	541565	32233
C8orf59	84.0491	70.7845	90.836	92.5462	105.752	95.2058	chromosome 8 open reading frame 59 [Source:HGNC Symbol;Acc	chr8:86089	NM_00109	XM_01151	.	.	NP_00109	XP_011515	protein_co	ENSG0000	401466	32235
C8orf74	0	0	0	0	0	0	chromosome 8 open reading frame 74 [Source:HGNC Symbol;Acc	chr8:10530	NM_00104	XM_01154	.	.	NP_00103	XP_011542	protein_co	ENSG0000	203076	32296
C8orf76	29.191	27.4881	29.4228	25.9492	32.978	27.2022	chromosome 8 open reading frame 76 [Source:HGNC Symbol;Acc	chr8:12423	NM_03284	.	.	.	NP_11623	.	protein_co	ENSG0000	84933	25924
C8orf82	15.0552	17.0165	16.9148	14.6171	11.7181	12.6611	chromosome 8 open reading frame 82 [Source:HGNC Symbol;Acc	chr8:14574	NM_00100	.	.	.	NP_00100	.	protein_co	ENSG0000	414919	33826
C8orf86	0	0	0	0	0.035634	0	chromosome 8 open reading frame 86 [Source:HGNC Symbol;Acc	chr8:38368	NM_00130	.	.	.	NP_00129	.	protein_co	ENSG0000	389649	33774
C8orf88	0.106182	0	0.460737	0.120746	0.117889	0.208283	chromosome 8 open reading frame 88 [Source:HGNC Symbol;Acc	chr8:91634	NM_00119	.	.	.	NP_00117	XP_016868	protein_co	ENSG0000	100127983	44672
C8orf89	0	0	0	0	0.228653	0.206174	chromosome 8 open reading frame 89 [Source:HGNC Symbol;Acc	chr8:74153	NM_00124	XM_01151	.	.	NP_00123	XP_011515	protein_co	ENSG0000	100130301	51258
C9	0	0	0	0	0	0	complement component 9 [Source:HGNC Symbol;Acc:HGNC:135	chr5:39284	NM_00173	.	.	.	NP_00172	.	protein_co	ENSG0000	735	1358

C9orf114	13.7055	13.8312	12.4208	17.8285	15.4707	10.2812	SPOUT domain containing methyltransferase 1 [Source:HGNC Syr	chr9:13158	NM_01639	XM_01701	.	.	NP_05747	XP_01687	protein_co	ENSG0000	51490	26933
C9orf116	15.4022	12.7551	15.0456	15.6899	9.69658	5.33299	chromosome 9 open reading frame 116 [Source:HGNC Symbol;Ac	chr9:13838	NM_00104	XM_01151	.	.	NP_00104	XP_01151	protein_co	ENSG0000	138162	28435
C9orf129	0.161626	0	0.069995	0.08631	0	0	chromosome 9 open reading frame 129 [Source:HGNC Symbol;Ac	chr9:95947	NM_00109	XM_00671	.	.	NP_00109	XP_006717	protein_co	ENSG0000	445577	31116
C9orf131	0	0	0	0	0	0	chromosome 9 open reading frame 131 [Source:HGNC Symbol;Ac	chr9:35041	NM_00104	.	.	.	NP_00103	.	protein_co	ENSG0000	138724	31418
C9orf135	0	0	0.102053	1.14938	0	0.240677	chromosome 9 open reading frame 135 [Source:HGNC Symbol;Ac	chr9:72435	NM_00101	XM_01151	NR_13175	.	NP_00101	XP_01151	protein_co	ENSG0000	138255	31422
C9orf142	63.8727	67.024	47.732	62.9108	51.885	38.7026	chromosome 9 open reading frame 142 [Source:HGNC Symbol;Ac	chr9:13988	NM_00132	.	.	.	NP_00131	.	protein_co	ENSG0000	286257	27849
C9orf152	0.03179	0	0	0.105533	0.071062	0.032778	chromosome 9 open reading frame 152 [Source:HGNC Symbol;Ac	chr9:11296	NM_00101	.	.	.	NP_00101	.	protein_co	ENSG0000	401546	31455
C9orf153	0	0	0	0	0.042122	0.073146	chromosome 9 open reading frame 153 [Source:HGNC Symbol;Ac	chr9:88835	NM_00127	.	.	.	NP_00126	.	protein_co	ENSG0000	389766	31456
C9orf16	98.0568	100.869	130.727	117.705	78.2779	92.7802	chromosome 9 open reading frame 16 [Source:HGNC Symbol;Acc	chr9:13092	NM_02411	.	.	.	NP_07701	.	protein_co	ENSG0000	79095	17823
C9orf172	3.61841	3.76908	3.74033	3.41602	2.34231	3.60436	chromosome 9 open reading frame 172 [Source:HGNC Symbol;Ac	chr9:13973	NM_00108	.	.	.	NP_00107	.	protein_co	ENSG0000	389813	37284
C9orf24	0	0.235154	0.209953	0.533948	0.082903	0	chromosome 9 open reading frame 24 [Source:HGNC Symbol;Acc	chr9:34379	NM_00125	XM_01151	.	.	NP_00123	XP_005251	protein_co	ENSG0000	84688	19919
C9orf3	3.46916	3.19738	3.80888	9.65536	6.7729	4.44004	chromosome 9 open reading frame 3 [Source:HGNC Symbol;Acc:	chr9:97488	NM_00119	XM_01151	NR_02949	.	NP_00118	XP_005252	protein_co	ENSG0000	407012	1361
C9orf38	0	0	0	0	0	0	chromosome 9 open reading frame 38 [Source:HGNC Symbol;Acc	chr9:64133	.	.	.	.	.	.	protein_co	ENSG0000	.	23398
C9orf40	6.96421	7.46257	7.80567	9.12857	7.07008	8.92596	chromosome 9 open reading frame 40 [Source:HGNC Symbol;Acc	chr9:77561	NM_01799	.	.	.	NP_06046	.	protein_co	ENSG0000	55071	23433
C9orf43	0.836469	0.51673	0.205149	0.222945	0.263101	0.23478	chromosome 9 open reading frame 43 [Source:HGNC Symbol;Acc	chr9:11617	NM_00127	XM_01151	.	.	NP_00126	XP_01151	protein_co	ENSG0000	257169	23570
C9orf47	0	0	0	0	0	0	chromosome 9 open reading frame 47 [Source:HGNC Symbol;Acc	chr9:91605	NM_00100	.	.	.	NP_00100	.	protein_co	ENSG0000	286223	23669
C9orf50	0	0	0	0	0.152935	0	chromosome 9 open reading frame 50 [Source:HGNC Symbol;Acc	chr9:13237	NM_19935	XM_01151	.	.	NP_95538	XP_01151	protein_co	ENSG0000	375759	23677
C9orf57	0	0	0	0	0	0	chromosome 9 open reading frame 57 [Source:HGNC Symbol;Acc	chr9:74666	NM_00112	XM_01151	.	.	NP_00112	XP_01151	protein_co	ENSG0000	138240	27037
C9orf64	0	0	0	0	0	0.035145	chromosome 9 open reading frame 64 [Source:HGNC Symbol;Acc	chr9:86553	NM_00131	XM_01151	.	.	NP_00130	.	protein_co	ENSG0000	84267	28144
C9orf66	0.073471	0.112326	0.09481	0.040818	0.166377	0.113191	chromosome 9 open reading frame 66 [Source:HGNC Symbol;Acc	chr9:21282	NM_15256	.	.	.	NP_68978	.	protein_co	ENSG0000	157983	26436
C9orf69	20.5352	22.5586	18.8854	19.0587	17.6209	21.3808	chromosome 9 open reading frame 69 [Source:HGNC Symbol;Acc	chr9:13900	NM_00125	.	.	.	NP_00124	.	protein_co	ENSG0000	90120	31009
C9orf72	5.1932	5.77436	5.42932	4.92597	5.77189	7.36938	chromosome 9 open reading frame 72 [Source:HGNC Symbol;Acc	chr9:27546	NM_00125	.	.	.	NP_00124	.	protein_co	ENSG0000	203228	28337
C9orf78	40.983	38.437	35.0736	38.938	34.8795	25.652	chromosome 9 open reading frame 78 [Source:HGNC Symbol;Acc	chr9:13258	NM_01652	XM_01151	.	.	NP_05760	XP_011517	protein_co	ENSG0000	51759	24932
C9orf84	0.106878	0.335033	0.275153	0.440053	0.681492	0.429258	chromosome 9 open reading frame 84 [Source:HGNC Symbol;Acc	chr9:11444	NM_00108	XM_01151	.	.	NP_00107	XP_006717	protein_co	ENSG0000	158401	26535
C9orf85	8.3815	7.6267	8.04928	6.27915	6.74086	5.29154	chromosome 9 open reading frame 85 [Source:HGNC Symbol;Acc	chr9:74526	NM_18250	.	.	.	NP_87231	.	protein_co	ENSG0000	138241	28784
C9orf91	6.0852	6.00003	4.43107	5.06202	4.42653	6.40644	transmembrane protein 268 [Source:HGNC Symbol;Acc:HGNC:24	chr9:11737	NM_15304	XM_01151	.	.	NP_69459	XP_006717	protein_co	ENSG0000	203197	24513
C9orf92	0	0	0	0	0	0	chromosome 9 open reading frame 92 [Source:HGNC Symbol;Acc	chr9:16203	NM_00127	.	.	.	NP_00125	.	protein_co	ENSG0000	100129385	19054
CA1	0	0	0.061207	0	0	0	carbonic anhydrase 1 [Source:HGNC Symbol;Acc:HGNC:1368]	chr8:86239	NM_00112	XM_01151	.	.	NP_00112	XP_011515	protein_co	ENSG0000	759	1368
CA10	0	0	0	0	0	0	carbonic anhydrase 10 [Source:HGNC Symbol;Acc:HGNC:1369]	chr17:4970	NM_00108	.	.	.	NP_00107	XP_01688	protein_co	ENSG0000	56934	1369
CA11	10.6653	9.32937	11.4018	5.9946	5.62414	7.95183	carbonic anhydrase 11 [Source:HGNC Symbol;Acc:HGNC:1370]	chr19:4914	NM_00121	.	.	.	NP_00120	.	protein_co	ENSG0000	770	1370
CA12	2.71536	2.06704	2.43246	3.2952	3.67561	2.45927	carbonic anhydrase 12 [Source:HGNC Symbol;Acc:HGNC:1371]	chr15:6361	NM_00121	.	.	.	NP_00120	.	protein_co	ENSG0000	771	1371
CA13	2.98358	3.31223	3.16542	3.558	4.02338	6.51612	carbonic anhydrase 13 [Source:HGNC Symbol;Acc:HGNC:14914]	chr8:86157	NM_19858	.	.	.	NP_94098	.	protein_co	ENSG0000	377677	14914
CA14	0.206782	0.06072	0.179672	0.129828	0.13356	0.09275	carbonic anhydrase 14 [Source:HGNC Symbol;Acc:HGNC:1372]	chr1:15022	NM_01211	XM_01150	.	.	NP_03624	XP_005245	protein_co	ENSG0000	23632	1372
CA2	19.2608	19.6816	26.5507	26.8046	24.5592	21.8247	carbonic anhydrase 2 [Source:HGNC Symbol;Acc:HGNC:1373]	chr8:86376	NM_00006	.	.	.	NP_00005	.	protein_co	ENSG0000	760	1373
CA3	0.242459	0.35691	0.252073	0.435868	0.432946	0.582466	carbonic anhydrase 3 [Source:HGNC Symbol;Acc:HGNC:1374]	chr8:86351	NM_00518	.	.	.	NP_00517	.	protein_co	ENSG0000	761	1374
CA4	0	0	0	0	0	0	carbonic anhydrase 4 [Source:HGNC Symbol;Acc:HGNC:1375]	chr17:5822	NM_00071	XM_00525	.	.	NP_00070	XP_005257	protein_co	ENSG0000	762	1375
CA5A	0	0	0	0	0	0	carbonic anhydrase 5A [Source:HGNC Symbol;Acc:HGNC:1377]	chr16:8791	NM_00173	.	.	.	NP_00173	.	protein_co	ENSG0000	763	1377
CA5B	1.2894	1.32974	0.998733	1.15237	0.721316	1.5952	carbonic anhydrase 5B [Source:HGNC Symbol;Acc:HGNC:1378]	chrX:15756	NM_00722	XM_00527	.	.	NP_00915	XP_005274	protein_co	ENSG0000	11238	1378
CA6	0	0	0	0	0	0	carbonic anhydrase 6 [Source:HGNC Symbol;Acc:HGNC:1380]	chr1:90059	NM_00121	XM_01154	.	.	NP_00120	XP_011540	protein_co	ENSG0000	765	1380
CA7	0	0	0	0	0	0	carbonic anhydrase 7 [Source:HGNC Symbol;Acc:HGNC:1381]	chr16:6687	NM_00101	XM_01152	.	.	NP_00101	XP_005256	protein_co	ENSG0000	766	1381
CA8	0.45482	0.417882	0.546422	0.668581	0.525861	0.252219	carbonic anhydrase 8 [Source:HGNC Symbol;Acc:HGNC:1382]	chr8:61099	NM_00405	XM_01151	.	.	NP_00130	.	protein_co	ENSG0000	767	1382
CA9	0.54267	0.293148	0.596014	2.06317	1.81637	1.13317	carbonic anhydrase 9 [Source:HGNC Symbol;Acc:HGNC:1383]	chr9:35673	NM_00121	.	.	.	NP_00120	.	protein_co	ENSG0000	768	1383
CAAP1	15.1763	13.9597	13.9359	14.3304	15.057	18.3794	caspase activity and apoptosis inhibitor 1 [Source:HGNC Symbol;A	chr9:26840	NM_00116	.	.	.	NP_00116	.	protein_co	ENSG0000	79886	25834
CAB39	29.4213	31.4156	33.3663	30.9216	36.1647	34.4149	calcium binding protein 39 [Source:HGNC Symbol;Acc:HGNC:2029	chr2:23157	NM_00113	.	.	.	NP_00112	.	protein_co	ENSG0000	51719	20292
CAB39L	2.13223	1.5918	2.56443	2.88951	2.10056	2.77408	calcium binding protein 39 like [Source:HGNC Symbol;Acc:HGNC:	chr13:4988	NM_00107	XM_01702	.	.	NP_00107	XP_011533	protein_co	ENSG0000	81617	20290
CABIN1	11.8921	13.137	13.2955	11.1937	10.9574	8.97824	calcineurin binding protein 1 [Source:HGNC Symbol;Acc:HGNC:24	chr22:2440	NM_00119	XM_01702	.	.	NP_00118	XP_005261	protein_co	ENSG0000	23523	24187
CABLES1	5.43712	5.62998	6.46483	4.99279	5.00767	3.70555	Cdk5 and Abl enzyme substrate 1 [Source:HGNC Symbol;Acc:HGN	chr18:2071	NM_00110	.	.	.	NP_00109	.	protein_co	ENSG0000	91768	25097
CABLES2	5.9935	5.15846	5.08183	6.24455	5.99547	5.418	Cdk5 and Abl enzyme substrate 2 [Source:HGNC Symbol;Acc:HGN	chr20:6096	NM_03121	.	.	.	NP_11249	.	protein_co	ENSG0000	81928	16143
CABP1	0.354089	0.449028	0.833646	0.657628	0.426887	0.57229	calcium binding protein 1 [Source:HGNC Symbol;Acc:HGNC:1384]	chr12:1210	NM_00103	XM_01702	.	.	NP_00102	XP_016875	protein_co	ENSG0000	9478	1384
CABP2	0	0	0	0	0	0	calcium binding protein 2 [Source:HGNC Symbol;Acc:HGNC:1385]	chr11:6728	NM_00131	XM_00527	.	.	NP_00130	.	protein_co	ENSG0000	51475	1385
CABP4	0.026692	0	0	0	0	0	calcium binding protein 4 [Source:HGNC Symbol;Acc:HGNC:1386]	chr11:6721	NM_00130	XM_01154	.	.	NP_00128	XP_011543	protein_co	ENSG0000	57010	1386
CABP5	0	0	0.048574	0.06067	0	0	calcium binding protein 5 [Source:HGNC Symbol;Acc:HGNC:13714	chr19:4853	NM_01985	.	.	.	NP_06282	.	protein_co	ENSG0000	56344	13714
CABP7	0	0.02349	0.041241	0	0	0	calcium binding protein 7 [Source:HGNC Symbol;Acc:HGNC:20834	chr22:3011	NM_18252	XM_00672	.	.	NP_87233	XP_006724	protein_co	ENSG0000	164633	20834
CABS1	0	0	0	0	0	0	calcium binding protein, spermatid associated 1 [Source:HGNC Sy	chr4:71200	NM_03312	.	.	.	NP_14911	.	protein_co	ENSG0000	85438	30710
CABYR	0.062919	0	0.16588	0	0.045598	0	calcium binding tyrosine phosphorylation regulated [Source:HGN	chr18:2171	NM_00130	XM_01702	.	.	NP_00129	XP_005258	protein_co	ENSG0000	26256	15569
CACFD1	4.06582	4.4675	3.95355	2.97552	3.02305	3.33949	calcium channel flower domain containing 1 [Source:HGNC Symb	chr9:13632	NM_00113	XM_01151	.	.	NP_00112	XP_01151	protein_co	ENSG0000	11094	1365
CACHD1	2.87034	3.29719	3.61078	2.96307	2.78577	3.19697	cache domain containing 1 [Source:HGNC Symbol;Acc:HGNC:293	chr1:64936	NM_00129	XM_01154	.	.	NP_00128	XP_011540	protein_co	ENSG0000	57685	29314
CACNA1A	0	0	0	0	0	0	calcium voltage-gated channel subunit alpha1 A [Source:HGNC Sy	chr19:1331	NM_00006	.	.	.	NP_00005	.	protein_co	ENSG0000	773	1388
CACNA1B	0.663247	0.712253	0.727266	0.610313	0.593163	0.528911	calcium voltage-gated channel subunit alpha1 B [Source:HGNC Sy	chr9:14077	NM_00071	XM_01701	.	.	NP_00070	XP_011517	protein_co	ENSG0000	774	1389

CACNA1C	0.053417	0.011021	0.044251	0	0.036245	0.118012	calcium voltage-gated channel subunit alpha1 C [Source:HGNC Sy	chr12:2162	NM_00071	XM_01701	.	.	NP_00071	XP_006719	protein_co	ENSG0000	775	1390
CACNA1D	0.194623	0.497875	0.342639	0.275555	0.365527	0.224517	calcium voltage-gated channel subunit alpha1 D [Source:HGNC Sy	chr3:53317	NM_00072	XM_01153	.	.	NP_00071	XP_005265	protein_co	ENSG0000	776	1391
CACNA1E	0	0	0.011922	0	0	0.010284	calcium voltage-gated channel subunit alpha1 E [Source:HGNC Sy	chr1:18145	NM_00072	XM_01700	.	.	NP_000712	XP_011508	protein_co	ENSG0000	777	1392
CACNA1F	0	0.023935	0	0	0	0	calcium voltage-gated channel subunit alpha1 F [Source:HGNC Sy	chrX:49061	NM_00125	XM_01154	.	.	NP_00124	XP_011542	protein_co	ENSG0000	778	1393
CACNA1G	0.469573	0.583858	1.19425	0.669214	0.677023	0.939883	calcium voltage-gated channel subunit alpha1 G [Source:HGNC Sy	chr17:4863	NM_00125	XM_01152	.	.	NP_00124	XP_006722	protein_co	ENSG0000	8913	1394
CACNA1H	7.38747	7.89048	10.3201	6.49037	6.70529	8.29455	calcium voltage-gated channel subunit alpha1 H [Source:HGNC Sy	chr16:1203	NM_00100	XM_01152	.	.	NP_00100	XP_005255	protein_co	ENSG0000	8912	1395
CACNA1I	0.052171	0.065347	0.162074	0.088164	0.067014	0.090801	calcium voltage-gated channel subunit alpha1 I [Source:HGNC Syr	chr22:3996	NM_00100	XM_01153	.	.	NP_00100	XP_011528	protein_co	ENSG0000	8911	1396
CACNA1S	0	0.010197	0.008753	0	0.011608	0.065481	calcium voltage-gated channel subunit alpha1 S [Source:HGNC Sy	chr1:20100	NM_00006	XM_00524	.	.	NP_00006	XP_005245	protein_co	ENSG0000	779	1397
CACNA2D1	0.035018	0.019543	0	0.020852	0.0385	0.166682	calcium voltage-gated channel auxiliary subunit alpha2delta 1 [So	chr7:81575	NM_00072	XM_01151	.	.	NP_00071	XP_005250	protein_co	ENSG0000	781	1399
CACNA2D2	2.79834	2.55435	4.04848	3.27094	3.16104	3.83547	calcium voltage-gated channel auxiliary subunit alpha2delta 2 [So	chr3:50384	NM_00100	XM_01153	.	.	NP_00100	XP_005265	protein_co	ENSG0000	9254	1400
CACNA2D3	0	0	0	0	0.02115	0.069813	calcium voltage-gated channel auxiliary subunit alpha2delta 3 [So	chr3:54156	NM_01839	XM_01700	.	.	NP_060868	XP_005265	protein_co	ENSG0000	55799	15460
CACNA2D4	0.18196	0.30726	0.465782	0.122431	0.172337	0.120349	calcium voltage-gated channel auxiliary subunit alpha2delta 4 [So	chr12:1901	NM_17236	XM_01152	.	.	NP_75895	XP_011519	protein_co	ENSG0000	93589	20202
CACNB1	4.11739	3.1517	3.65587	4.12639	3.97362	3.01127	calcium voltage-gated channel auxiliary subunit beta 1 [Source:H	chr17:3732	NM_00072	XM_00525	.	.	NP_000714	XP_005257	protein_co	ENSG0000	782	1401
CACNB2	0.283335	0.327166	0.598573	0.553451	0.315041	0.532692	calcium voltage-gated channel auxiliary subunit beta 2 [Source:H	chr10:1842	NM_00072	XM_01151	.	.	NP_00071	XP_005252	protein_co	ENSG0000	783	1402
CACNB3	10.6024	10.2821	12.197	12.2746	11.6832	8.37638	calcium voltage-gated channel auxiliary subunit beta 3 [Source:H	chr12:4920	NM_00072	XM_01701	.	.	NP_00071	XP_011537	protein_co	ENSG0000	784	1403
CACNB4	0.510381	0.285852	0.662842	0.878011	1.04557	0.949313	calcium voltage-gated channel auxiliary subunit beta 4 [Source:H	chr2:15268	NM_00072	XM_01151	.	.	NP_00071	XP_006712	protein_co	ENSG0000	785	1404
CACNG1	0	0	0	0	0	0	calcium voltage-gated channel auxiliary subunit gamma 1 [Source	chr17:6504	NM_00072	.	.	.	NP_00071	.	protein_co	ENSG0000	786	1405
CACNG2	0.037574	0	0	0	0	0	calcium voltage-gated channel auxiliary subunit gamma 2 [Source	chr22:3695	NM_00607	.	.	.	NP_00606	.	protein_co	ENSG0000	10369	1406
CACNG3	0	0	0	0	0	0	calcium voltage-gated channel auxiliary subunit gamma 3 [Source	chr16:2426	NM_00653	.	.	.	NP_00653	.	protein_co	ENSG0000	10368	1407
CACNG4	0	0	0	0	0	0	calcium voltage-gated channel auxiliary subunit gamma 4 [Source	chr17:6496	NM_01440	.	.	.	NP_05522	.	protein_co	ENSG0000	27092	1408
CACNG5	0	0	0	0	0	0	calcium voltage-gated channel auxiliary subunit gamma 5 [Source	chr17:6483	NM_14581	XM_01152	.	.	NP_66581	XP_011522	protein_co	ENSG0000	27091	1409
CACNG6	0	0	0	0	0.078644	0	calcium voltage-gated channel auxiliary subunit gamma 6 [Source	chr19:5449	NM_03189	.	.	.	NP_11410	.	protein_co	ENSG0000	59285	13625
CACNG7	0	0.100826	0.035628	0.044771	0	0	calcium voltage-gated channel auxiliary subunit gamma 7 [Source	chr19:5441	NM_03189	XM_01702	.	.	NP_11410	XP_016882	protein_co	ENSG0000	59284	13626
CACNG8	0.042219	0.035574	0.049217	0.007695	0.032278	0.02977	calcium voltage-gated channel auxiliary subunit gamma 8 [Source	chr19:5446	NM_03189	.	NR_03063	.	NP_11410	.	protein_co	ENSG0000	100126325	13628
CACTIN	12.6719	15.1903	13.006	15.3486	13.8614	11.6192	cactin, spliceosome C complex subunit [Source:HGNC Symbol;Acc	chr19:3610	NM_00108	.	.	.	NP_00107	.	protein_co	ENSG0000	58509	29938
CACUL1	14.8157	15.0911	15.7872	14.0326	17.3778	18.1254	CDK2 associated cullin domain 1 [Source:HGNC Symbol;Acc:HGN	chr10:1204	NM_15381	.	.	.	NP_72251	.	protein_co	ENSG0000	143384	23727
CACYBP	129.884	111.866	89.3184	174.878	146.207	117.237	calcyclin binding protein [Source:HGNC Symbol;Acc:HGNC:30423]	chr1:17496	NM_00100	.	.	.	NP_00100	XP_016856	protein_co	ENSG0000	27101	30423
CAD	29.3396	31.8874	28.7585	38.3851	37.3594	27.9663	carbamoyl-phosphate synthetase 2, aspartate transcarbamylase,	chr2:27440	NM_00130	XM_00671	.	.	NP_00129	XP_005264	protein_co	ENSG0000	790	1424
CADM1	0	0	0	0	0	0	cell adhesion molecule 1 [Source:HGNC Symbol;Acc:HGNC:5951]	chr11:1150	NM_00109	XM_01701	.	.	NP_00109	XP_005271	protein_co	ENSG0000	23705	5951
CADM2	0	0	0	0	0	0	cell adhesion molecule 2 [Source:HGNC Symbol;Acc:HGNC:29849	chr3:85008	NM_00116	XM_01700	.	.	NP_00116	XP_006713	protein_co	ENSG0000	253559	29849
CADM3	0	0	0	0	0	0	cell adhesion molecule 3 [Source:HGNC Symbol;Acc:HGNC:17601	chr1:15914	NM_00112	.	.	.	NP_00112	.	protein_co	ENSG0000	57863	17601
CADM4	21.0751	20.6382	21.9964	24.7648	23.6949	23.6228	cell adhesion molecule 4 [Source:HGNC Symbol;Acc:HGNC:30825	chr19:4412	NM_14529	XM_01702	.	.	NP_66033	XP_005258	protein_co	ENSG0000	199731	30825
CADPS	0	0	0	0	0	0.030416	calcium dependent secretion activator [Source:HGNC Symbol;Acc	chr3:62384	NM_00371	XM_01700	.	.	NP_00370	XP_006713	protein_co	ENSG0000	8618	1426
CADPS2	3.04358	3.16122	3.7534	2.33949	2.57885	2.86376	calcium dependent secretion activator 2 [Source:HGNC Symbol;A	chr7:12195	NM_00100	XM_01701	.	.	NP_00100	XP_005250	protein_co	ENSG0000	93664	16018
CAGE1	0	0.05046	0.020494	0.026841	0.023988	0.045155	cancer antigen 1 [Source:HGNC Symbol;Acc:HGNC:21622]	chr6:73268	NM_00117	.	.	.	NP_00116	.	protein_co	ENSG0000	285782	21622
CALB1	0	0	0	0	0	0	calbindin 1 [Source:HGNC Symbol;Acc:HGNC:1434]	chr8:91070	NM_00492	.	.	.	NP_00492	.	protein_co	ENSG0000	793	1434
CALB2	2.25173	3.05374	3.85038	1.87246	1.90101	2.4991	calbindin 2 [Source:HGNC Symbol;Acc:HGNC:1435]	chr16:7139	NM_00174	XM_01152	.	.	NP_00173	.	protein_co	ENSG0000	794	1435
CALCA	0	0	0	0	0	0	calcitonin related polypeptide alpha [Source:HGNC Symbol;Acc:H	chr11:1492	NM_00103	.	.	.	NP_00102	XP_016873	protein_co	ENSG0000	796	1437
CALCB	0.276629	0.166392	0	0.089174	0	0.432617	calcitonin related polypeptide beta [Source:HGNC Symbol;Acc:HG	chr11:1492	NM_00072	.	.	.	NP_00071	.	protein_co	ENSG0000	797	1438
CALCOCO1	7.16521	6.53502	10.9808	3.66315	3.75916	5.4968	calcium binding and coiled-coil domain 1 [Source:HGNC Symbol;A	chr12:5410	NM_00114	XM_01153	.	.	NP_00113	XP_011536	protein_co	ENSG0000	57658	29306
CALCOCO2	40.1747	38.7113	33.5505	35.7954	33.8794	63.3795	calcium binding and coiled-coil domain 2 [Source:HGNC Symbol;A	chr17:4690	NM_00126	XM_01152	.	.	NP_00124	.	protein_co	ENSG0000	10241	29912
CALCR	0.024727	0.068091	0.04992	0.393763	0.055086	0.248847	calcitonin receptor [Source:HGNC Symbol;Acc:HGNC:1440]	chr7:93053	NM_00116	.	.	.	NP_00115	.	protein_co	ENSG0000	799	1440
CALCRL	0.554013	0.633711	0.539251	0.374509	0.453154	1.22379	calcitonin receptor like receptor [Source:HGNC Symbol;Acc:HGN	chr2:18820	NM_00127	XM_00524	.	.	NP_00125	XP_005246	protein_co	ENSG0000	10203	16709
CALD1	22.4282	23.725	23.5282	24.8517	27.0874	26.0521	caldesmon 1 [Source:HGNC Symbol;Acc:HGNC:1441]	chr7:13442	NM_00434	XM_01701	.	.	NP_00433	XP_016868	protein_co	ENSG0000	800	1441
CALHM1	0	0	0	0	0.027836	0	calcium homeostasis modulator 1 [Source:HGNC Symbol;Acc:HGN	chr10:1052	NM_00100	.	.	.	NP_00100	.	protein_co	ENSG0000	255022	23494
CALHM2	18.8775	16.6962	16.1604	16.2638	13.1589	17.2671	calcium homeostasis modulator 2 [Source:HGNC Symbol;Acc:HGN	chr10:1052	NM_01591	XM_01701	.	.	NP_05700	XP_006717	protein_co	ENSG0000	51063	23493
CALHM3	0.25284	0.558879	0.375918	0.202341	0.58727	0.488694	calcium homeostasis modulator 3 [Source:HGNC Symbol;Acc:HGN	chr10:1052	NM_00112	.	.	.	NP_00112	.	protein_co	ENSG0000	119395	23458
CALM1	149.087	145.723	140.1	173.574	164.151	153.846	calmodulin 1 [Source:HGNC Symbol;Acc:HGNC:1442]	chr14:9086	NM_00688	XM_00672	.	.	NP_00129	XP_006720	protein_co	ENSG0000	805	1442
CALM2	306.598	271.839	282.25	407.289	471.574	463.093	calmodulin 2 [Source:HGNC Symbol;Acc:HGNC:1445]	chr2:47387	NM_00174	.	.	.	NP_00129	.	protein_co	ENSG0000	805	1445
CALM3	201.778	203.357	195.761	285.161	248.793	230.272	calmodulin 3 [Source:HGNC Symbol;Acc:HGNC:1449]	chr19:4702	NM_00518	.	.	.	NP_00129	.	protein_co	ENSG0000	808	1449
CALML3	0	0.027106	0	0	0	0	calmodulin like 3 [Source:HGNC Symbol;Acc:HGNC:1452]	chr10:5565	NM_00518	.	.	.	NP_00517	.	protein_co	ENSG0000	810	1452
CALML4	5.08626	5.28297	5.15867	6.65013	6.55554	6.27286	calmodulin like 4 [Source:HGNC Symbol;Acc:HGNC:18445]	chr15:6834	NM_00103	.	.	.	NP_00102	.	protein_co	ENSG0000	91860	18445
CALML5	0	0	0	0	0	0	calmodulin like 5 [Source:HGNC Symbol;Acc:HGNC:18180]	chr10:5540	NM_01742	.	.	.	NP_05911	.	protein_co	ENSG0000	51806	18180
CALML6	0.172265	0.229698	0.403121	0.183396	0.200156	0.562933	calmodulin like 6 [Source:HGNC Symbol;Acc:HGNC:24193]	chr1:18462	NM_13870	XM_01154	.	.	NP_61965	XP_005244	protein_co	ENSG0000	163688	24193
CALN1	0.008524	0	0	0.009544	0	0.008643	calneuron 1 [Source:HGNC Symbol;Acc:HGNC:13248]	chr7:71244	NM_00101	XM_01701	.	.	NP_00101	XP_011514	protein_co	ENSG0000	83698	13248
CALR	816.476	800.781	394.536	867.356	753.504	524.265	calreticulin [Source:HGNC Symbol;Acc:HGNC:1455]	chr19:1304	NM_00434	.	.	.	NP_00433	.	protein_co	ENSG0000	811	1455
CALR3	0.05279	0	0	0.058686	0.05961	0.107899	calreticulin 3 [Source:HGNC Symbol;Acc:HGNC:20407]	chr19:1658	NM_14504	.	.	.	NP_65948	.	protein_co	ENSG0000	125972	20407
CALU	82.331	85.0812	70.0883	81.0049	90.5775	106.545	calumenin [Source:HGNC Symbol;Acc:HGNC:1458]	chr7:12837	NM_00113	XM_01151	.	.	NP_00112	.	protein_co	ENSG0000	813	1458
CALY	0	0.043238	0.038311	0	0	0.120057	calcyon neuron specific vesicular protein [Source:HGNC Symbol;A	chr10:1350	NM_01572	XM_01153	.	.	NP_05653	XP_011538	protein_co	ENSG0000	50632	17938

CAMK1	7.07691	7.84133	7.03016	8.22297	5.8822	5.32722	calcium/calmodulin dependent protein kinase I [Source:HGNC Sy	chr3:97916	NM_00365	XM_00526	.	.	NP_00364	XP_005265	protein_co	ENSG0000	8536	1459
CAMK1D	2.42254	2.50378	4.09262	2.45466	2.22934	3.29345	calcium/calmodulin dependent protein kinase ID [Source:HGNC S	chr10:1239	NM_02039	XM_01701	.	.	NP_06513	XP_006717	protein_co	ENSG0000	57118	19341
CAMK1G	0.115193	0.058637	0.024933	0.062744	0.131628	0.030668	calcium/calmodulin dependent protein kinase IG [Source:HGNC S	chr1:20975	NM_02043	XM_01700	.	.	NP_06517	XP_016857	protein_co	ENSG0000	57172	14585
CAMK2A	0	0	0.012272	0	0	0	calcium/calmodulin dependent protein kinase II alpha [Source:HG	chr5:14956	NM_01598	.	.	.	NP_05706	XP_016865	protein_co	ENSG0000	815	1460
CAMK2B	0.236306	0.072405	0.125241	0.039307	0.084367	0.098372	calcium/calmodulin dependent protein kinase II beta [Source:HG	chr7:44256	NM_00122	XM_01701	.	.	NP_00121	XP_005249	protein_co	ENSG0000	816	1461
CAMK2D	15.7363	15.9239	20.896	14.0251	15.7919	17.7277	calcium/calmodulin dependent protein kinase II delta [Source:HG	chr4:11437	NM_00122	XM_01700	.	.	NP_00121	XP_005263	protein_co	ENSG0000	817	1462
CAMK2G	12.886	11.8068	12.476	12.2269	11.2754	9.54082	calcium/calmodulin dependent protein kinase II gamma [Source:	chr10:7557	NM_00120	XM_01701	.	.	NP_00119	XP_005270	protein_co	ENSG0000	818	1463
CAMK2N1	9.21053	9.77838	10.5387	8.40308	8.89301	9.98652	calcium/calmodulin dependent protein kinase II inhibitor 1 [Sour	chr1:20808	NM_01858	.	.	.	NP_06105	.	protein_co	ENSG0000	55450	24190
CAMK2N2	4.19857	3.42122	4.73767	5.57763	3.81334	5.12034	calcium/calmodulin dependent protein kinase II inhibitor 2 [Sour	chr3:18396	NM_03325	.	.	.	NP_15028	.	protein_co	ENSG0000	94032	24197
CAMK4	3.02712	3.88161	3.10446	3.16757	3.58991	2.90027	calcium/calmodulin dependent protein kinase IV [Source:HGNC S	chr5:11055	NM_00132	.	.	.	NP_00131	.	protein_co	ENSG0000	814	1464
CAMKK1	4.57529	4.68776	4.18177	2.31602	2.50518	2.12293	calcium/calmodulin dependent protein kinase kinase 1 [Source:H	chr17:3763	NM_03229	XM_01702	.	.	NP_11567	XP_005256	protein_co	ENSG0000	84254	1469
CAMKK2	18.3743	18.9338	18.1863	19.1678	18.6295	15.9659	calcium/calmodulin dependent protein kinase kinase 2 [Source:H	chr12:1216	NM_00127	XM_01701	.	.	NP_00125	XP_005253	protein_co	ENSG0000	10645	1470
CAMKMT	3.93812	3.55972	4.86823	2.3937	3.90727	3.7414	calmodulin-lysine N-methyltransferase [Source:HGNC Symbol;Ac	chr2:44589	NM_02476	.	.	.	NP_07904	.	protein_co	ENSG0000	79823	26276
CAMKV	0.050556	0.045582	0.039537	0	0	0.054301	CaM kinase like vesicle associated [Source:HGNC Symbol;Acc:HG	chr3:49895	NM_02404	XM_00526	.	.	NP_00130	XP_005265	protein_co	ENSG0000	79012	28788
CAMLG	19.9226	19.9453	28.2287	18.6525	21.9011	23.1575	calcium modulating ligand [Source:HGNC Symbol;Acc:HGNC:1471	chr5:13407	NM_00174	.	.	.	NP_00173	XP_011541	protein_co	ENSG0000	819	1471
CAMP	0	0	0	0.105473	0	0	cathelicidin antimicrobial peptide [Source:HGNC Symbol;Acc:HGN	chr3:48264	NM_00434	.	.	.	NP_00433	.	protein_co	ENSG0000	820	1472
CAMSAP1	13.8436	13.5647	13.9577	12.8079	14.2264	12.2796	calmodulin regulated spectrin associated protein 1 [Source:HGNC	chr9:13870	NM_01544	XM_01151	.	.	NP_05626	XP_005263	protein_co	ENSG0000	157922	19946
CAMSAP2	10.8223	11.0492	10.81	11.1373	13.2355	13.8044	calmodulin regulated spectrin associated protein family member	chr1:20070	NM_00129	XM_00524	.	.	NP_00128	XP_005245	protein_co	ENSG0000	23271	29188
CAMSAP3	8.56018	9.33897	8.8835	10.3564	9.14404	8.31031	calmodulin regulated spectrin associated protein family member	chr19:7660	NM_00108	XM_01702	.	.	NP_00107	XP_005272	protein_co	ENSG0000	57662	29307
CAMTA1	31.5176	38.0414	36.912	40.2895	43.8486	51.7708	calmodulin binding transcription activator 1 [Source:HGNC Symb	chr1:68453	NM_00119	XM_01700	.	.	NP_00118	XP_011539	protein_co	ENSG0000	23261	18806
CAMTA2	11.9292	10.7843	11.6732	9.11528	8.25067	8.21007	calmodulin binding transcription activator 2 [Source:HGNC Symb	chr17:4871	NM_00117	XM_01152	.	.	NP_00116	XP_006721	protein_co	ENSG0000	23125	18807
CAND1	20.4069	19.5554	21.8452	19.0003	25.5123	21.4875	cullin associated and neddylation dissociated 1 [Source:HGNC Sy	chr12:6766	NM_01844	.	.	.	NP_00131	.	protein_co	ENSG0000	55832	30688
CAND2	0	0.038777	0.016877	0.020983	0	0.021793	cullin associated and neddylation dissociated 2 (putative) [Source	chr3:12837	NM_00116	XM_01153	.	.	NP_00115	XP_011531	protein_co	ENSG0000	23066	30689
CANT1	42.0649	43.2713	38.6053	44.0896	43.1327	37.2984	calcium activated nucleotidase 1 [Source:HGNC Symbol;Acc:HGN	chr17:7698	NM_00115	XM_01152	.	.	NP_00115	XP_005257	protein_co	ENSG0000	124583	19721
CANX	279.866	301.17	214.151	241.952	262.657	264.146	calnexin [Source:HGNC Symbol;Acc:HGNC:1473]	chr5:17912	NM_00102	XM_01153	.	.	NP_00101	XP_011532	protein_co	ENSG0000	821	1473
CAP1	152.716	152.437	155.9	158.512	157.758	169.288	adenylate cyclase associated protein 1 [Source:HGNC Symbol;Acc	chr1:40505	NM_00110	XM_01154	.	.	NP_00109	XP_005270	protein_co	ENSG0000	10487	20040
CAP2	3.18497	2.76262	2.41487	2.15965	2.85239	2.46786	CAP, adenylate cyclase-associated protein, 2 (yeast) [Source:HGN	chr6:17393	NM_00636	XM_01151	.	.	NP_00635	XP_011512	protein_co	ENSG0000	10486	20039
CAPG	69.3443	66.7529	77.0133	77.8284	61.9927	78.6647	capping actin protein, gelsolin like [Source:HGNC Symbol;Acc:HG	chr2:85621	NM_00125	XM_01153	.	.	NP_00124	XP_005264	protein_co	ENSG0000	822	1474
CAPN1	77.376	87.0836	94.3897	83.8508	74.7931	68.0582	calpain 1 [Source:HGNC Symbol;Acc:HGNC:1476]	chr11:6494	NM_00119	XM_01154	.	.	NP_00118	XP_006718	protein_co	ENSG0000	823	1476
CAPN10	11.9476	9.45064	11.7706	13.0508	9.97382	9.93543	calpain 10 [Source:HGNC Symbol;Acc:HGNC:1477]	chr2:24152	NM_02308	.	.	.	NP_07557	.	protein_co	ENSG0000	11132	1477
CAPN11	0	0	0.021206	0	0	0	calpain 11 [Source:HGNC Symbol;Acc:HGNC:1478]	chr6:44126	NM_00705	XM_01151	.	.	NP_00898	XP_006715	protein_co	ENSG0000	11131	1478
CAPN12	0.675763	0.697285	1.15423	0.40065	0.349314	0.287047	calpain 12 [Source:HGNC Symbol;Acc:HGNC:13249]	chr19:3913	NM_14469	XM_01152	.	.	NP_65329	XP_016881	protein_co	ENSG0000	147968	13249
CAPN13	0.127788	0.026059	0.088517	0.16793	0.174537	0.21535	calpain 13 [Source:HGNC Symbol;Acc:HGNC:16663]	chr2:30945	NM_14457	XM_01153	.	.	NP_65317	XP_011531	protein_co	ENSG0000	92291	16663
CAPN14	0	0	0	0	0	0	calpain 14 [Source:HGNC Symbol;Acc:HGNC:16664]	chr2:31397	NM_00114	XM_01153	.	.	NP_00113	XP_011531	protein_co	ENSG0000	440854	16664
CAPN15	11.4683	12.0787	9.53514	12.3066	11.4705	9.36225	calpain 15 [Source:HGNC Symbol;Acc:HGNC:11182]	chr16:5778	NM_00563	XM_01152	.	.	NP_00562	XP_011520	protein_co	ENSG0000	6650	11182
CAPN2	91.5309	94.7931	83.9867	92.0688	95.3512	80.4063	calpain 2 [Source:HGNC Symbol;Acc:HGNC:1479]	chr1:22388	NM_00114	.	.	.	NP_00113	.	protein_co	ENSG0000	824	1479
CAPN3	2.05335	1.31959	1.0643	1.0641	1.09335	0.853963	calpain 3 [Source:HGNC Symbol;Acc:HGNC:1480]	chr15:4265	NM_00007	.	.	.	NP_00006	.	protein_co	ENSG0000	825	1480
CAPN5	3.08188	3.25954	4.78766	3.67568	3.55673	4.23989	calpain 5 [Source:HGNC Symbol;Acc:HGNC:1482]	chr11:7677	NM_00405	XM_01701	.	.	NP_00404	XP_011543	protein_co	ENSG0000	726	1482
CAPN6	0.855561	0.731142	1.17079	0.231355	0.514874	0.5487	calpain 6 [Source:HGNC Symbol;Acc:HGNC:1483]	chrX:11048	NM_01428	.	.	.	NP_05510	.	protein_co	ENSG0000	827	1483
CAPN7	7.95886	6.63869	7.57303	7.20852	8.845	8.39389	calpain 7 [Source:HGNC Symbol;Acc:HGNC:1484]	chr3:15247	NM_01429	XM_01153	.	.	NP_05511	XP_011531	protein_co	ENSG0000	23473	1484
CAPN8	0.09685	0.130847	0.052134	0	0.103013	0.195627	calpain 8 [Source:HGNC Symbol;Acc:HGNC:1485]	chr1:22371	NM_00114	.	.	.	NP_00113	XP_016856	protein_co	ENSG0000	388743	1485
CAPN9	0	0	0	0	0	0	calpain 9 [Source:HGNC Symbol;Acc:HGNC:1486]	chr1:23088	NM_00131	XM_01154	.	.	NP_00130	XP_005273	protein_co	ENSG0000	10753	1486
CAPNS1	256.99	268.183	241.32	286.407	211.35	219.051	calpain small subunit 1 [Source:HGNC Symbol;Acc:HGNC:1481]	chr19:3663	NM_00100	XM_00525	.	.	NP_00100	XP_005259	protein_co	ENSG0000	826	1481
CAPNS2	0.075963	0.158288	0	0	0.085283	0.076617	calpain small subunit 2 [Source:HGNC Symbol;Acc:HGNC:16371]	chr16:5554	NM_03233	.	.	.	NP_11570	.	protein_co	ENSG0000	84290	16371
CAPRIN1	99.7745	106.042	101.688	118.651	133.555	127.503	cell cycle associated protein 1 [Source:HGNC Symbol;Acc:HGNC:6	chr11:3407	NM_00589	XM_01701	.	.	NP_00588	XP_016873	protein_co	ENSG0000	4076	6743
CAPRIN2	6.74906	6.84577	7.73802	5.74713	6.36491	8.33249	caprin family member 2 [Source:HGNC Symbol;Acc:HGNC:21259]	chr12:3086	NM_00100	XM_01152	.	.	NP_00100	XP_006719	protein_co	ENSG0000	65981	21259
CAPS	1.0466	1.31021	2.14573	0.496931	1.42548	1.09887	calcyphosine [Source:HGNC Symbol;Acc:HGNC:1487]	chr19:5911	NM_00405	XM_00525	.	.	NP_00404	XP_005259	protein_co	ENSG0000	828	1487
CAPS2	0.697688	1.04722	1.25399	1.35891	2.01303	2.29337	calcyphosine 2 [Source:HGNC Symbol;Acc:HGNC:16471]	chr12:7566	NM_00128	XM_01153	.	.	NP_00127	XP_006719	protein_co	ENSG0000	84698	16471
CAPSL	0	0	0	0	0	0	calcyphosine like [Source:HGNC Symbol;Acc:HGNC:28375]	chr5:35904	NM_00104	XM_00671	.	.	NP_00103	XP_006714	protein_co	ENSG0000	133690	28375
CAPZA1	65.6281	59.6569	66.1596	71.0004	83.8821	85.1714	capping actin protein of muscle Z-line alpha subunit 1 [Source:HG	chr1:11300	NM_00613	.	.	.	NP_00612	.	protein_co	ENSG0000	829	1488
CAPZA2	42.5938	42.4885	50.6122	54.1071	52.9648	55.1121	capping actin protein of muscle Z-line alpha subunit 2 [Source:HG	chr7:11650	NM_00613	.	.	.	NP_00612	.	protein_co	ENSG0000	830	1490
CAPZA3	0	0	0	0	0	0	capping actin protein of muscle Z-line alpha subunit 3 [Source:HG	chr12:1889	NM_03332	.	.	.	NP_20158	.	protein_co	ENSG0000	93661	24205
CAPZB	89.1718	92.4218	96.5873	114.736	96.5634	93.6569	capping actin protein of muscle Z-line beta subunit [Source:HGNC	chr1:19665	NM_00120	.	.	.	NP_00119	XP_006711	protein_co	ENSG0000	832	1491
CARD10	14.6086	14.1012	13.1378	16.6766	13.8333	11.2234	caspase recruitment domain family member 10 [Source:HGNC Sy	chr22:3788	NM_01455	.	.	.	NP_05536	.	protein_co	ENSG0000	29775	16422
CARD11	0.013881	0.02729	0.036215	0.014851	0	0.015457	caspase recruitment domain family member 11 [Source:HGNC Sy	chr7:29457	NM_00132	XM_01151	.	.	NP_00131	XP_011513	protein_co	ENSG0000	84433	16393
CARD14	0.018401	0	0.07593	0.031186	0	0.041348	caspase recruitment domain family member 14 [Source:HGNC Sy	chr17:7814	NM_00125	XM_01152	.	.	NP_00124	XP_011523	protein_co	ENSG0000	79092	16446
CARD16	0.117265	0.364247	0.102666	0.193503	0	0	caspase recruitment domain family member 16 [Source:HGNC Sy	chr11:1049	NM_00101	.	.	.	NP_00101	.	protein_co	ENSG0000	114769	33701
CARD17	0	0	0	0	0	0	caspase recruitment domain family member 17 [Source:HGNC Sy	chr11:1049	NM_00100	.	.	.	NP_00100	.	protein_co	ENSG0000	440068	33827
CARD18	0	0	0	0	0	0	caspase recruitment domain family member 18 [Source:HGNC Sy	chr11:1050	NM_02157	.	.	.	NP_06754	.	protein_co	ENSG0000	59082	28861

CARD19	39.9111	37.632	36.5481	45.7758	36.7239	35.2611	caspase recruitment domain family member 19 [Source:HGNC Sy	chr9:95858	NM_03231	XM_00525	.	.	NP_11568	.	protein_co	ENSG0000	84270	28148
CARD6	0.06335	0.016519	0.01364	0.017626	0.064738	0.031098	caspase recruitment domain family member 6 [Source:HGNC Sym	chr5:40841	NM_03258	.	.	.	NP_11597	.	protein_co	ENSG0000	84674	16394
CARD8	4.82969	4.75368	5.88453	6.13627	6.65643	5.50642	caspase recruitment domain family member 8 [Source:HGNC Sym	chr19:4867	NM_00118	XM_01702	.	.	NP_00117	XP_006723	protein_co	ENSG0000	22900	17057
CARD9	0.148425	0.212822	0.032005	0.195679	0.263802	0.213694	caspase recruitment domain family member 9 [Source:HGNC Sym	chr9:13925	NM_05281	.	.	.	NP_43470	.	protein_co	ENSG0000	64170	16391
CARF	1.0968	0.749133	1.41492	0.672656	0.973519	1.44285	calcium responsive transcription factor [Source:HGNC Symbol;Ac	chr2:20373	NM_00110	XM_01700	.	.	NP_00109	XP_005246	protein_co	ENSG0000	79800	14435
CARHSP1	42.8418	43.1644	45.6917	52.05	41.8904	38.0443	calcium regulated heat stable protein 1 [Source:HGNC Symbol;Ac	chr16:8946	NM_00104	XM_01152	.	.	NP_00103	XP_005255	protein_co	ENSG0000	23589	17150
CARM1	49.1919	53.0789	44.8977	54.7473	50.7492	47.3111	coactivator associated arginine methyltransferase 1 [Source:HGN	chr19:1098	NM_19914	XM_01152	.	.	NP_95459	XP_005259	protein_co	ENSG0000	10498	23393
CARMIL2	4.13096	3.45465	4.47666	5.67065	3.91757	2.62062	capping protein regulator and myosin 1 linker 2 [Source:HGNC Sy	chr16:6767	NM_00101	XM_01152	.	.	NP_00101	XP_005255	protein_co	ENSG0000	146206	27089
CARMIL3	0.24788	0.244282	0.289749	0.202196	0.220235	0.113625	capping protein regulator and myosin 1 linker 3 [Source:HGNC Sy	chr14:2452	NM_13836	XM_01153	.	.	NP_61236	XP_011535	protein_co	ENSG0000	90668	20272
CARNMT1	10.7053	11.1748	10.7939	10.1239	12.4715	10.4645	carnosine N-methyltransferase 1 [Source:HGNC Symbol;Acc:HGN	chr9:77595	NM_00132	XM_01701	.	.	NP_00130	XP_005251	protein_co	ENSG0000	138199	23435
CARNS1	0.393542	0.300689	0.896012	0.260101	0.249538	0.263908	carnosine synthase 1 [Source:HGNC Symbol;Acc:HGNC:29268]	chr11:6718	NM_00116	XM_01154	.	.	NP_00115	XP_011543	protein_co	ENSG0000	57571	29268
CARS	39.0277	37.326	25.7799	24.6967	20.8477	63.3069	cysteinyl-tRNA synthetase [Source:HGNC Symbol;Acc:HGNC:1493	chr11:3022	NM_00101	XM_01701	.	.	NP_00101	XP_006718	protein_co	ENSG0000	833	1493
CARS2	16.9066	15.5399	16.2197	19.6207	17.7863	15.7114	cysteinyl-tRNA synthetase 2, mitochondrial (putative) [Source:HG	chr13:1112	NM_02453	XM_01152	.	.	NP_07881	XP_006720	protein_co	ENSG0000	79587	25695
CARTPT	0	0	0	0	0	0	CART prepropeptide [Source:HGNC Symbol;Acc:HGNC:24323]	chr5:71014	NM_00429	.	.	.	NP_00428	.	protein_co	ENSG0000	9607	24323
CASC1	0.088183	0.082205	0.20613	0.101306	0.096861	0.2561	cancer susceptibility candidate 1 [Source:HGNC Symbol;Acc:HGN	chr12:2526	NM_00108	XM_01701	.	.	NP_00107	XP_005253	protein_co	ENSG0000	55259	29599
CASC10	0.773646	0.858944	1.31162	0.624233	0.853337	1.24928	cancer susceptibility candidate 10 [Source:HGNC Symbol;Acc:HGI	chr10:2178	NM_00101	.	.	.	NP_00101	.	protein_co	ENSG0000	399726	31448
CASC3	26.1293	27.3913	23.5827	23.9958	22.6513	23.5111	cancer susceptibility candidate 3 [Source:HGNC Symbol;Acc:HGN	chr17:3829	NM_00735	XM_00525	NR_10692	.	NP_03138	XP_005257	protein_co	ENSG0000	10246698	17040
CASC4	31.8555	32.9538	30.9733	28.2796	30.8009	32.0761	cancer susceptibility candidate 4 [Source:HGNC Symbol;Acc:HGN	chr15:4458	NM_13842	.	.	.	NP_61243	.	protein_co	ENSG0000	113201	24892
CASC5	6.73113	6.32483	6.83949	8.28269	12.1897	11.8329	kinetochore scaffold 1 [Source:HGNC Symbol;Acc:HGNC:24054]	chr15:4088	NM_14450	XM_01702	.	.	NP_65309	XP_016877	protein_co	ENSG0000	57082	24054
CASD1	1.05525	1.40411	1.60521	0.748568	1.15763	1.98054	CAS1 domain containing 1 [Source:HGNC Symbol;Acc:HGNC:1601	chr7:94139	NM_02290	XM_01151	.	.	NP_07505	XP_011514	protein_co	ENSG0000	64921	16014
CASK	8.50467	8.40861	8.3137	7.55784	9.11915	8.57799	calcium/calmodulin dependent serine protein kinase [Source:HGI	chrX:41374	NM_00112	XM_01154	.	.	NP_00111	XP_005272	protein_co	ENSG0000	8573	1497
CASKIN1	1.54205	1.35144	2.1526	1.28453	1.01328	1.24451	CASK interacting protein 1 [Source:HGNC Symbol;Acc:HGNC:2087	chr16:2205	NM_02076	.	.	.	NP_06581	.	protein_co	ENSG0000	57524	20879
CASKIN2	8.94869	8.95555	7.45971	9.40585	9.53673	7.81307	CASK interacting protein 2 [Source:HGNC Symbol;Acc:HGNC:1820	chr17:7349	NM_00114	.	.	.	NP_00113	.	protein_co	ENSG0000	57513	18200
CASP1	0.061911	0	0	0	0	0	caspase 1 [Source:HGNC Symbol;Acc:HGNC:1499]	chr11:1048	NM_00122	XM_01154	.	.	NP_00121	XP_016873	protein_co	ENSG0000	834	1499
CASP10	1.83452	2.13474	2.29707	1.62389	1.39189	2.08113	caspase 10 [Source:HGNC Symbol;Acc:HGNC:1500]	chr2:20204	NM_00120	XM_01151	.	.	NP_00119	XP_005246	protein_co	ENSG0000	843	1500
CASP14	0	0	0	0	0	0	caspase 14 [Source:HGNC Symbol;Acc:HGNC:1502]	chr19:1516	NM_01211	XM_01152	.	.	NP_03624	XP_011526	protein_co	ENSG0000	23581	1502
CASP2	29.0122	27.6599	24.496	27.6527	29.3856	27.4818	caspase 2 [Source:HGNC Symbol;Acc:HGNC:1503]	chr7:14298	NM_03298	.	.	.	NP_11676	.	protein_co	ENSG0000	835	1503
CASP3	12.3422	11.6634	10.5713	15.7103	17.5482	16.1025	caspase 3 [Source:HGNC Symbol;Acc:HGNC:1504]	chr4:18554	NM_00434	XM_01153	.	.	NP_00433	XP_011530	protein_co	ENSG0000	836	1504
CASP4	19.0748	17.0222	12.2125	9.24178	8.35624	8.04868	caspase 4 [Source:HGNC Symbol;Acc:HGNC:1505]	chr11:1048	NM_00122	XM_01154	.	.	NP_00121	XP_011541	protein_co	ENSG0000	837	1505
CASP5	0	0	0	0	0	0	caspase 5 [Source:HGNC Symbol;Acc:HGNC:1506]	chr11:1048	NM_00113	XM_01154	.	.	NP_00112	.	protein_co	ENSG0000	838	1506
CASP6	15.7658	14.9671	13.3988	19.2638	19.794	12.7515	caspase 6 [Source:HGNC Symbol;Acc:HGNC:1507]	chr4:11048	NM_00122	.	.	.	NP_00121	.	protein_co	ENSG0000	839	1507
CASP7	23.457	24.5807	24.4818	27.9506	26.7305	22.6583	caspase 7 [Source:HGNC Symbol;Acc:HGNC:1508]	chr10:1154	NM_00122	XM_01154	.	.	NP_00121	XP_006718	protein_co	ENSG0000	840	1508
CASP8	18.6617	19.1043	13.9211	17.8179	17.1098	14.5861	caspase 8 [Source:HGNC Symbol;Acc:HGNC:1509]	chr2:20209	NM_00108	XM_01151	.	.	NP_00107	XP_005246	protein_co	ENSG0000	841	1509
CASP8AP2	6.81252	6.26775	6.73467	6.05169	9.09095	7.58876	caspase 8 associated protein 2 [Source:HGNC Symbol;Acc:HGNC:1	chr6:90539	NM_00113	.	.	.	NP_00113	.	protein_co	ENSG0000	9994	1510
CASP9	6.36326	5.24438	6.62927	4.65976	4.89917	5.2556	caspase 9 [Source:HGNC Symbol;Acc:HGNC:1511]	chr1:15802	NM_00122	XM_01154	.	.	NP_00122	XP_005246	protein_co	ENSG0000	842	1511
CASQ1	0	0.031566	0	0	0	0	calsequestrin 1 [Source:HGNC Symbol;Acc:HGNC:1512]	chr1:16016	NM_00123	.	.	.	NP_00122	.	protein_co	ENSG0000	844	1512
CASQ2	0.024252	0	0	0	0	0	calsequestrin 2 [Source:HGNC Symbol;Acc:HGNC:1513]	chr1:11624	NM_00123	.	.	.	NP_00122	.	protein_co	ENSG0000	845	1513
CASS4	0.199668	0.228174	0.070943	0.064733	0.287023	0.115008	Cas scaffolding protein family member 4 [Source:HGNC Symbol;A	chr20:5498	NM_00116	XM_00672	.	.	NP_00115	XP_006723	protein_co	ENSG0000	57091	15878
CAST	37.0984	37.3827	32.8236	35.0927	33.0905	36.9174	calpastatin [Source:HGNC Symbol;Acc:HGNC:1515]	chr5:95997	NM_00104	XM_01700	.	.	NP_00103	XP_006714	protein_co	ENSG0000	831	1515
CASZ1	1.50865	1.68805	1.85044	1.56128	1.19081	2.0073	castor zinc finger 1 [Source:HGNC Symbol;Acc:HGNC:26002]	chr1:10696	NM_00107	XM_01154	.	.	NP_00107	XP_005263	protein_co	ENSG0000	54897	26002
CAT	24.5007	25.5194	35.278	29.5415	25.0506	35.0521	catalase [Source:HGNC Symbol;Acc:HGNC:1516]	chr11:3446	NM_00175	.	.	.	NP_00174	.	protein_co	ENSG0000	847	1516
CATIP	0.057026	0.056979	0.197468	0	0	0	ciliogenesis associated TTC17 interacting protein [Source:HGNC S	chr2:21922	NM_19855	XM_01151	.	.	NP_00130	XP_005246	protein_co	ENSG0000	375307	25062
CATSPER1	0.429822	0.40158	0.420952	0.371018	0.231463	0.625394	cation channel sperm associated 1 [Source:HGNC Symbol;Acc:HG	chr11:6578	NM_05305	.	.	.	NP_44428	.	protein_co	ENSG0000	117144	17116
CATSPER2	0.890991	0.470475	0.567611	0.405764	0.516149	0.450063	cation channel sperm associated 2 [Source:HGNC Symbol;Acc:HG	chr15:4392	NM_00128	XM_01702	.	.	NP_00126	XP_011519	protein_co	ENSG0000	117155	18810
CATSPER3	1.16601	1.65104	1.41565	0.814842	1.16838	1.09122	cation channel sperm associated 3 [Source:HGNC Symbol;Acc:HG	chr5:13424	NM_17801	.	.	.	NP_82113	.	protein_co	ENSG0000	347732	20819
CATSPER4	0.035611	0	0.030928	0	0.08189	0.038225	cation channel sperm associated 4 [Source:HGNC Symbol;Acc:HG	chr1:26517	NM_19813	XM_01154	.	.	NP_93777	XP_011539	protein_co	ENSG0000	378807	23220
CATSPERB	0.068319	0.07387	0.138651	0.078561	0.075095	0.065703	cation channel sperm associated auxiliary subunit beta [Source:H	chr14:9204	NM_02476	.	.	.	NP_07904	.	protein_co	ENSG0000	79820	20500
CATSPERD	0	0	0	0	0	0	cation channel sperm associated auxiliary subunit delta [Source:H	chr19:5720	NM_15278	XM_01152	.	.	NP_68999	XP_011526	protein_co	ENSG0000	257062	28598
CATSPERG	0.138089	0.139062	0.18719	0.021444	0.159113	0.042428	cation channel sperm associated auxiliary subunit gamma [Source	chr19:3882	NM_02118	XM_01702	.	.	NP_06700	XP_005259	protein_co	ENSG0000	57828	25243
CATX-2	0	0	0	0	0	0	HCG1989573; Uncharacterized protein [Source:UniProtKB/TrEMI	chr2:74418	.	.	.	.	.	.	protein_co	ENSG0000	.	.
CAV1	112.905	119.712	92.3458	152.472	157.794	112.117	caveolin 1 [Source:HGNC Symbol;Acc:HGNC:1527]	chr7:11616	NM_00117	.	.	.	NP_00116	.	protein_co	ENSG0000	857	1527
CAV2	53.126	53.1798	40.5488	68.3256	60.6649	49.7073	caveolin 2 [Source:HGNC Symbol;Acc:HGNC:1528]	chr7:11613	NM_00120	.	.	.	NP_00119	.	protein_co	ENSG0000	858	1528
CAV3	0	0	0	0	0	0	caveolin 3 [Source:HGNC Symbol;Acc:HGNC:1529]	chr3:87754	NM_00123	.	.	.	NP_00122	.	protein_co	ENSG0000	859	1529
CBARP	4.77845	5.10511	6.42723	4.46467	4.39329	4.5578	CACN beta subunit associated regulatory protein [Source:HGNC S	chr19:1205	NM_15276	XM_01152	.	.	NP_68998	XP_011526	protein_co	ENSG0000	255057	28617
CBFA2T2	3.3052	3.85325	4.10883	2.64708	3.59635	3.79655	CBFA2/RUNX1 translocation partner 2 [Source:HGNC Symbol;Acc	chr20:3207	NM_00103	XM_01702	.	.	NP_00102	XP_006723	protein_co	ENSG0000	9139	1536
CBFA2T3	5.35218	5.03153	5.9879	5.36486	4.57375	3.71832	CBFA2/RUNX1 translocation partner 3 [Source:HGNC Symbol;Acc	chr16:8894	NM_00518	XM_01152	.	.	NP_00517	XP_005256	protein_co	ENSG0000	863	1537
CBFB	34.2906	41.5568	32.7904	46.2573	50.1292	48.3833	core-binding factor beta subunit [Source:HGNC Symbol;Acc:HGN	chr16:6706	NM_00175	XM_00672	.	.	NP_00174	XP_005256	protein_co	ENSG0000	865	1539
CBL	9.84391	10.4669	13.0685	11.1465	12.5331	14.0834	Cbl proto-oncogene [Source:HGNC Symbol;Acc:HGNC:1541]	chr11:1190	NM_00518	XM_01701	.	.	NP_00517	XP_016874	protein_co	ENSG0000	867	1541
CBLB	2.89277	2.83684	2.22668	2.53157	3.80476	3.75682	Cbl proto-oncogene B [Source:HGNC Symbol;Acc:HGNC:1542]	chr3:10537	NM_00132	XM_01700	.	.	NP_00130	XP_005247	protein_co	ENSG0000	868	1542

CBLC	17.3734	19.1248	19.4145	19.4273	13.327	13.7527	Cbl proto-oncogene C [Source:HGNC Symbol;Acc:HGNC:15961]	chr19:4528	NM_00113	.	.	.	NP_00112	XP_005258	protein_co	ENSG0000	23624	15961
CBLL1	14.7177	19.7569	16.8075	17.338	15.2569	15.6207	Cbl proto-oncogene like 1 [Source:HGNC Symbol;Acc:HGNC:2122	chr7:10738	NM_00128	.	.	.	NP_00127	XP_016868	protein_co	ENSG0000	79872	21225
CBLN1	0	0	0	0	0	0	cerebellin 1 precursor [Source:HGNC Symbol;Acc:HGNC:1543]	chr16:4931	NM_00435	.	.	.	NP_00434	.	protein_co	ENSG0000	869	1543
CBLN2	0	0	0	0	0	0	cerebellin 2 precursor [Source:HGNC Symbol;Acc:HGNC:1544]	chr18:7020	NM_18251	XM_01152	.	.	NP_87231	XP_006722	protein_co	ENSG0000	147381	1544
CBLN3	0.745905	0.550831	1.04422	0.62737	0.464028	0.771679	cerebellin 3 precursor [Source:HGNC Symbol;Acc:HGNC:20146]	chr14:2489	NM_00103	.	.	.	NP_00103	.	protein_co	ENSG0000	643866	20146
CBLN4	0	0	0	0	0	0	cerebellin 4 precursor [Source:HGNC Symbol;Acc:HGNC:16231]	chr20:5457	NM_08061	.	.	.	NP_54218	.	protein_co	ENSG0000	140689	16231
CBR1	0	0	0	0	0	0	carbonyl reductase 1 [Source:HGNC Symbol;Acc:HGNC:1548]	chr21:3740	NM_00128	.	.	.	NP_00127	.	protein_co	ENSG0000	873	1548
CBR3	2.79509	3.20671	2.592	3.26307	2.6785	2.18174	carbonyl reductase 3 [Source:HGNC Symbol;Acc:HGNC:1549]	chr21:3750	NM_00123	.	.	.	NP_00122	.	protein_co	ENSG0000	874	1549
CBR4	12.4637	12.7808	15.0747	12.6827	14.801	17.6656	carbonyl reductase 4 [Source:HGNC Symbol;Acc:HGNC:25891]	chr4:16990	NM_03278	XM_00671	.	.	NP_11617	XP_005263	protein_co	ENSG0000	84869	25891
CBS	0.088844	0.057782	0.333152	5.55E-06	0	0	cystathionine-beta-synthase [Source:HGNC Symbol;Acc:HGNC:15	chr21:4447	NM_00007	XM_01702	.	.	NP_00006	XP_011528	protein_co	ENSG0000	102724560	1550
CBSL	0.000729	0.000737	0.000589	0.036934	0	0	cystathionine-beta-synthase like [Source:HGNC Symbol;Acc:HGN	chr21:4447	NM_00132	XM_01702	.	.	NP_00130	XP_011544	protein_co	ENSG0000	102724560	51829
CBWD1	15.1388	13.734	12.7541	14.071	13.7945	12.9112	COBW domain containing 1 [Source:HGNC Symbol;Acc:HGNC:171	chr9:12103	NM_00114	XM_01151	.	.	NP_00113	XP_005251	protein_co	ENSG0000	55871	17134
CBWD2	7.6743	7.22718	9.20118	5.05174	5.98997	6.87213	COBW domain containing 2 [Source:HGNC Symbol;Acc:HGNC:179	chr2:11419	NM_17200	XM_01151	.	.	NP_74200	XP_005263	protein_co	ENSG0000	150472	17907
CBWD3	0.341626	0.205075	0.47407	0.095136	0.140491	0.206379	COBW domain containing 3 [Source:HGNC Symbol;Acc:HGNC:185	chr9:70856	NM_00129	XM_01701	.	.	NP_00127	XP_005277	protein_co	ENSG0000	644019	18519
CBWD5	19.0182	22.6793	19.5408	18.4563	15.4472	13.8548	COBW domain containing 5 [Source:HGNC Symbol;Acc:HGNC:245	chr9:70432	NM_00102	XM_01703	.	.	NP_00102	XP_005272	protein_co	ENSG0000	644019	24584
CBWD6	2.35728	1.74399	2.60263	1.42454	1.80081	1.34043	.	chr9:69204	.	.	.	.	.	.	unprocesse	ENSG0000	.	.
CBWD7	0	0	0	0	0	0	COBW domain containing 7 [Source:HGNC Symbol;Acc:HGNC:319	chr9:42671	NM_00108	.	.	.	NP_00107	XP_016870	protein_co	ENSG0000	644019	31977
CBX1	110.839	110.173	106.301	128.306	135.885	126.834	chromobox 1 [Source:HGNC Symbol;Acc:HGNC:1551]	chr17:4614	NM_00112	.	.	.	NP_00112	.	protein_co	ENSG0000	10951	1551
CBX2	17.9103	18.8451	18.344	25.4695	24.3849	18.3051	chromobox 2 [Source:HGNC Symbol;Acc:HGNC:1552]	chr17:7775	NM_00518	XM_01152	.	.	NP_00518	XP_011523	protein_co	ENSG0000	84733	1552
CBX3	207.802	205.485	186.534	235.444	261.38	239.35	chromobox 3 [Source:HGNC Symbol;Acc:HGNC:1553]	chr7:26240	NM_00727	XM_00524	.	.	NP_00920	XP_005249	protein_co	ENSG0000	11335	1553
CBX4	21.9809	23.1501	17.0061	21.6241	20.4213	35.4465	chromobox 4 [Source:HGNC Symbol;Acc:HGNC:1554]	chr17:7780	NM_00365	.	.	.	NP_00364	.	protein_co	ENSG0000	8535	1554
CBX5	26.8632	27.0498	29.2809	32.113	39.2169	42.7413	chromobox 5 [Source:HGNC Symbol;Acc:HGNC:1555]	chr12:5462	NM_00112	.	NR_03985	.	NP_00112	.	protein_co	ENSG0000	100616400	1555
CBX6	56.0738	61.1856	57.0457	56.7689	54.9995	48.0222	chromobox 6 [Source:HGNC Symbol;Acc:HGNC:1556]	chr22:3925	NM_00130	XM_01153	.	.	NP_00129	.	protein_co	ENSG0000	23466	1556
CBX7	9.14076	9.99967	9.77432	7.94671	7.76223	7.76784	chromobox 7 [Source:HGNC Symbol;Acc:HGNC:1557]	chr22:3952	NM_17570	XM_01702	.	.	NP_78364	XP_005261	protein_co	ENSG0000	23492	1557
CBX8	10.91	10.3565	9.79965	11.2863	10.4265	8.98511	chromobox 8 [Source:HGNC Symbol;Acc:HGNC:15962]	chr17:7776	NM_02064	.	.	.	NP_06570	.	protein_co	ENSG0000	57332	15962
CBY1	21.0624	21.7347	22.8896	20.668	17.5849	20.601	chibby family member 1, beta catenin antagonist [Source:HGNC S	chr22:3905	NM_00100	.	.	.	NP_00100	.	protein_co	ENSG0000	25776	1307
CBY3	0	0	0	0	0	0	chibby family member 3 [Source:HGNC Symbol;Acc:HGNC:33278]	chr5:17910	NM_00116	.	.	.	NP_00115	XP_016865	protein_co	ENSG0000	646019	33278
CC2D1A	11.5899	11.6894	13.0747	11.2294	10.7315	12.0339	coiled-coil and C2 domain containing 1A [Source:HGNC Symbol;A	chr19:1401	NM_01772	XM_00525	.	.	NP_06019	XP_005260	protein_co	ENSG0000	54862	30237
CC2D1B	11.2028	9.92146	8.30089	7.40671	6.64326	5.24264	coiled-coil and C2 domain containing 1B [Source:HGNC Symbol;A	chr1:52816	NM_03244	XM_00671	.	.	NP_11582	XP_005270	protein_co	ENSG0000	200014	29386
CC2D2A	6.46879	7.63339	4.2128	7.48331	4.67558	4.11973	coiled-coil and C2 domain containing 2A [Source:HGNC Symbol;A	chr4:15471	NM_00108	XM_01700	.	.	NP_00107	XP_005248	protein_co	ENSG0000	57545	29253
CC2D2B	0	0.051542	0.041443	0	0.052315	0.046116	coiled-coil and C2 domain containing 2B [Source:HGNC Symbol;A	chr10:9773	NM_00100	XM_01153	.	.	NP_00100	XP_011538	protein_co	ENSG0000	100127889	31666
CCAR1	25.0603	24.5773	24.8411	28.1582	32.9698	34.0561	cell division cycle and apoptosis regulator 1 [Source:HGNC Symbo	chr10:7048	NM_00128	.	.	.	NP_00126	.	protein_co	ENSG0000	55749	24236
CCAR2	66.437	79.4181	72.5958	80.7296	77.3807	53.2432	cell cycle and apoptosis regulator 2 [Source:HGNC Symbol;Acc:H	chr8:22462	NM_02117	XM_01701	.	.	NP_06699	XP_011542	protein_co	ENSG0000	57805	23360
CCBE1	0.10071	0.138898	0.310784	0.148589	0.252602	0.407307	collagen and calcium binding EGF domains 1 [Source:HGNC Symb	chr18:5709	NM_13345	XM_00526	.	.	NP_59771	XP_005266	protein_co	ENSG0000	147372	29426
CCDC102A	2.16581	2.48043	2.0495	1.82437	1.63133	1.78247	coiled-coil domain containing 102A [Source:HGNC Symbol;Acc:H	chr16:5754	NM_03321	XM_01152	.	.	NP_14998	XP_011521	protein_co	ENSG0000	92922	28097
CCDC102B	0.107409	0.161421	0.414066	0.194493	0.080112	0.078329	coiled-coil domain containing 102B [Source:HGNC Symbol;Acc:H	chr18:6646	NM_00109	XM_01702	.	XR_93525	NP_00108	XP_011524	protein_co	ENSG0000	79839	26295
CCDC103	0.59435	0.52419	0.837876	0.3879	0.961846	0.314903	coiled-coil domain containing 103 [Source:HGNC Symbol;Acc:HG	chr17:4297	NM_00125	.	.	.	NP_00124	.	protein_co	ENSG0000	388389	32700
CCDC105	0	0	0	0	0	0	coiled-coil domain containing 105 [Source:HGNC Symbol;Acc:HG	chr19:1512	NM_17348	.	.	.	NP_77575	.	protein_co	ENSG0000	126402	26866
CCDC106	0.670061	0.680927	0.406595	0.42828	0.653034	0.331738	coiled-coil domain containing 106 [Source:HGNC Symbol;Acc:HG	chr19:5615	NM_01330	XM_00525	.	.	NP_03743	XP_005258	protein_co	ENSG0000	29903	30181
CCDC107	10.6908	8.74691	8.52686	9.0997	7.78183	6.08629	coiled-coil domain containing 107 [Source:HGNC Symbol;Acc:HG	chr9:35658	NM_00119	XM_00525	.	.	NP_00118	XP_005251	protein_co	ENSG0000	203260	28465
CCDC109B	8.29188	8.38797	9.88968	8.78666	9.16824	13.5198	mitochondrial calcium uniporter dominant negative beta subunit	chr4:11048	NM_01791	.	.	.	NP_06038	XP_006714	protein_co	ENSG0000	55013	26076
CCDC110	0.552087	0.733848	0.913555	0.646249	0.925781	1.24571	coiled-coil domain containing 110 [Source:HGNC Symbol;Acc:HG	chr4:18635	NM_00114	XM_01700	.	.	NP_00113	XP_005262	protein_co	ENSG0000	256309	28504
CCDC112	6.74601	6.35589	6.56651	7.14321	9.28088	8.29532	coiled-coil domain containing 112 [Source:HGNC Symbol;Acc:HG	chr5:11460	NM_00104	XM_00527	.	.	NP_00103	XP_005271	protein_co	ENSG0000	153733	28599
CCDC113	5.20048	5.28143	6.38459	4.82964	4.35256	5.18424	coiled-coil domain containing 113 [Source:HGNC Symbol;Acc:HG	chr16:5828	NM_00114	XM_01702	.	.	NP_00113	XP_011521	protein_co	ENSG0000	29070	25002
CCDC114	0.506238	0.36886	0.913751	0.25966	0.43422	0.704658	coiled-coil domain containing 114 [Source:HGNC Symbol;Acc:HG	chr19:4879	NM_14457	XM_01152	.	.	NP_65317	XP_005259	protein_co	ENSG0000	93233	26560
CCDC115	19.4122	21.0039	19.6662	15.9302	15.6621	12.3984	coiled-coil domain containing 115 [Source:HGNC Symbol;Acc:HG	chr2:13109	NM_03235	XM_00526	.	.	NP_00130	XP_005263	protein_co	ENSG0000	84317	28178
CCDC116	0.163169	0.128858	0.312965	0.155015	0.169594	0.181571	coiled-coil domain containing 116 [Source:HGNC Symbol;Acc:HG	chr22:2198	NM_15261	XM_00672	.	.	NP_68982	XP_006724	protein_co	ENSG0000	164592	26688
CCDC117	15.7332	15.8327	15.3586	13.2795	15.5849	16.6343	coiled-coil domain containing 117 [Source:HGNC Symbol;Acc:HG	chr22:2916	NM_00128	.	.	.	NP_00127	.	protein_co	ENSG0000	150275	26599
CCDC12	25.1222	26.2545	20.594	31.3541	23.3129	19.3056	coiled-coil domain containing 12 [Source:HGNC Symbol;Acc:HGN	chr3:46963	NM_00127	XM_01153	.	.	NP_00126	XP_011531	protein_co	ENSG0000	151903	28332
CCDC120	4.69927	5.63334	6.00988	3.78433	3.26633	3.74	coiled-coil domain containing 120 [Source:HGNC Symbol;Acc:HG	chrX:48911	NM_00116	.	.	.	NP_00115	.	protein_co	ENSG0000	90060	28910
CCDC121	1.44041	1.80544	0.90598	1.71513	1.52413	1.33004	coiled-coil domain containing 121 [Source:HGNC Symbol;Acc:HG	chr2:27848	NM_00114	XM_00526	.	.	NP_00113	XP_005264	protein_co	ENSG0000	79635	25833
CCDC122	1.14822	0.964063	1.23641	1.19998	1.22755	1.85576	coiled-coil domain containing 122 [Source:HGNC Symbol;Acc:HG	chr13:4441	NM_14497	.	.	.	NP_65941	XP_016875	protein_co	ENSG0000	160857	26478
CCDC124	115.053	121.82	106.453	138.271	108.356	90.3769	coiled-coil domain containing 124 [Source:HGNC Symbol;Acc:HG	chr19:1804	NM_00113	.	.	.	NP_00112	.	protein_co	ENSG0000	115098	25171
CCDC125	6.89817	6.61855	6.97215	6.34939	7.33355	8.57793	coiled-coil domain containing 125 [Source:HGNC Symbol;Acc:HG	chr5:68576	NM_00129	XM_01154	.	.	NP_00128	XP_005248	protein_co	ENSG0000	202243	28924
CCDC126	3.66314	2.94582	3.066	2.38162	4.07198	4.10366	coiled-coil domain containing 126 [Source:HGNC Symbol;Acc:HG	chr7:23636	NM_13877	.	.	.	NP_62012	XP_016868	protein_co	ENSG0000	90693	22398
CCDC127	2.71356	2.9581	2.52221	2.48736	2.2674	2.56506	coiled-coil domain containing 127 [Source:HGNC Symbol;Acc:HG	chr5:19698	NM_14526	.	.	.	NP_66030	.	protein_co	ENSG0000	133957	30520
CCDC129	0	0	0	0	0	0	coiled-coil domain containing 129 [Source:HGNC Symbol;Acc:HG	chr7:31553	NM_00125	XM_01151	.	.	NP_00124	XP_011513	protein_co	ENSG0000	223075	27363
CCDC13	0.216542	0.2748	0.275165	0.361602	0.319359	0.150365	coiled-coil domain containing 13 [Source:HGNC Symbol;Acc:HGN	chr3:42749	NM_14471	XM_01153	.	.	NP_65332	XP_005264	protein_co	ENSG0000	152206	26358
CCDC130	10.1074	9.02958	8.66523	10.207	8.94049	9.39167	coiled-coil domain containing 130 [Source:HGNC Symbol;Acc:HG	chr19:1384	NM_00132	XM_00526	.	.	NP_00130	XP_005260	protein_co	ENSG0000	81576	28118

CCDC134	4.59773	4.3893	4.49946	5.81031	5.85053	4.9802	coiled-coil domain containing 134 [Source:HGNC Symbol;Acc:HGN	chr22:4219	NM_00130	XM_00526	.	.	NP_00129	XP_005261	protein_co	ENSG0000	79879	26185
CCDC136	1.39402	1.68515	1.9376	1.3288	1.41617	1.4764	coiled-coil domain containing 136 [Source:HGNC Symbol;Acc:HGN	chr7:12843	NM_00120	XM_00671	.	.	NP_00118	XP_005250	protein_co	ENSG0000	64753	22225
CCDC137	41.8387	38.7406	38.6054	52.8524	44.9126	33.9545	coiled-coil domain containing 137 [Source:HGNC Symbol;Acc:HGN	chr17:7963	NM_19928	.	.	.	NP_95498	.	protein_co	ENSG0000	339230	33451
CCDC138	3.79318	2.73843	3.9603	3.09514	6.48609	5.74006	coiled-coil domain containing 138 [Source:HGNC Symbol;Acc:HGN	chr2:10940	NM_00130	XM_01151	.	.	NP_00129	XP_006712	protein_co	ENSG0000	165055	26531
CCDC14	25.9495	24.0846	27.2475	24.305	33.4499	29.3014	coiled-coil domain containing 14 [Source:HGNC Symbol;Acc:HGN	chr3:12363	NM_02275	XM_01700	.	.	NP_00129	XP_005247	protein_co	ENSG0000	64770	25766
CCDC140	0	0	0	0	0	0	coiled-coil domain containing 140 [Source:HGNC Symbol;Acc:HGN	chr2:22306	NM_15303	.	.	.	NP_69458	XP_016858	protein_co	ENSG0000	151278	26514
CCDC141	0	0	0	0	0	0	coiled-coil domain containing 141 [Source:HGNC Symbol;Acc:HGN	chr2:17973	NM_00131	.	.	.	NP_00130	.	protein_co	ENSG0000	285025	26821
CCDC142	2.53275	2.86302	3.21774	2.49449	1.93236	2.1791	coiled-coil domain containing 142 [Source:HGNC Symbol;Acc:HGN	chr2:74700	NM_03277	.	.	.	NP_11616	.	protein_co	ENSG0000	84865	25889
CCDC144A	0	0	0.020542	0	0.051576	0.009989	coiled-coil domain containing 144A [Source:HGNC Symbol;Acc:HG	chr17:1659	NM_01469	XM_00672	.	.	NP_05551	XP_016880	protein_co	ENSG0000	9720	29072
CCDC144NL	0	0	0.026276	0	0	0	coiled-coil domain containing 144 family, N-terminal like [Source:	chr17:2076	NM_00100	.	.	.	NP_00100	XP_011522	protein_co	ENSG0000	339184	33735
CCDC146	1.55888	1.20178	1.93342	1.14844	1.52697	1.99232	coiled-coil domain containing 146 [Source:HGNC Symbol;Acc:HGN	chr7:76751	NM_02087	.	.	.	NP_06593	.	protein_co	ENSG0000	57639	29296
CCDC148	0.34403	0.291177	0.162832	0.446296	0.213108	0.193749	coiled-coil domain containing 148 [Source:HGNC Symbol;Acc:HGN	chr2:15902	NM_00130	XM_01151	.	.	NP_00128	XP_005246	protein_co	ENSG0000	130940	25191
CCDC149	1.19932	1.16578	1.26544	1.25837	1.16056	1.05687	coiled-coil domain containing 149 [Source:HGNC Symbol;Acc:HGN	chr4:24807	NM_00113	XM_01700	.	.	NP_00112	XP_005248	protein_co	ENSG0000	91050	25405
CCDC15	1.2199	1.15614	0.92359	1.65364	1.60405	1.38009	coiled-coil domain containing 15 [Source:HGNC Symbol;Acc:HGN	chr11:1248	NM_02500	.	.	.	NP_07928	.	protein_co	ENSG0000	80071	25798
CCDC150	6.60018	5.6303	4.6115	4.42368	4.55098	3.03285	coiled-coil domain containing 150 [Source:HGNC Symbol;Acc:HGN	chr2:19750	NM_00108	XM_01151	.	.	NP_00107	XP_006712	protein_co	ENSG0000	284992	26834
CCDC151	0.204564	0.176848	0.507926	0.349514	0.246309	0.327306	coiled-coil domain containing 151 [Source:HGNC Symbol;Acc:HGN	chr19:1153	NM_00130	.	.	.	NP_00128	.	protein_co	ENSG0000	115948	28303
CCDC152	0	0.02642	0.021093	0	0.026542	0	coiled-coil domain containing 152 [Source:HGNC Symbol;Acc:HGN	chr5:42756	NM_00113	XM_01151	.	.	NP_00112	.	protein_co	ENSG0000	100129792	34438
CCDC153	1.73017	0.642856	1.16632	1.1097	1.12677	0.695552	coiled-coil domain containing 153 [Source:HGNC Symbol;Acc:HGN	chr11:1190	NM_00114	XM_01154	.	.	NP_00113	XP_011541	protein_co	ENSG0000	283152	27446
CCDC154	0.192259	0.061462	0.055129	0.236093	0.113452	0.07319	coiled-coil domain containing 154 [Source:HGNC Symbol;Acc:HGN	chr16:1484	NM_00114	XM_01152	.	.	NP_00113	.	protein_co	ENSG0000	645811	34454
CCDC155	0.028254	0.028018	0	0.030317	0.06525	0	coiled-coil domain containing 155 [Source:HGNC Symbol;Acc:HGN	chr19:4989	NM_14468	XM_01152	.	.	NP_65328	XP_005258	protein_co	ENSG0000	147872	26520
CCDC157	1.26225	1.24507	1.99686	1.32938	0.863314	1.02344	coiled-coil domain containing 157 [Source:HGNC Symbol;Acc:HGN	chr22:3072	NM_00101	XM_01153	.	.	NP_00101	XP_006724	protein_co	ENSG0000	550631	33854
CCDC158	0	0	0	0	0	0	coiled-coil domain containing 158 [Source:HGNC Symbol;Acc:HGN	chr4:77234	NM_00104	XM_01153	.	.	NP_00103	XP_005263	protein_co	ENSG0000	339965	26374
CCDC159	1.91452	1.78328	1.61439	1.51786	0.587507	0.693014	coiled-coil domain containing 159 [Source:HGNC Symbol;Acc:HGN	chr19:1140	NM_00108	XM_00672	.	.	NP_00107	XP_006722	protein_co	ENSG0000	126075	26996
CCDC160	0.122292	0.354705	0.178641	0.194389	0.249953	0.220237	coiled-coil domain containing 160 [Source:HGNC Symbol;Acc:HGN	chrX:13337	NM_00110	XM_00526	.	.	NP_00109	XP_005262	protein_co	ENSG0000	347475	37286
CCDC163P	1.82527	3.21066	1.80101	4.54382	3.69151	3.21745	coiled-coil domain containing 163 [Source:HGNC Symbol;Acc:HGN	chr1:45960	.	.	.	.	.	.	protein_co	ENSG0000	.	27003
CCDC166	0	0	0	0	0	0	coiled-coil domain containing 166 [Source:HGNC Symbol;Acc:HGN	chr8:14478	NM_00116	.	.	.	NP_00115	.	protein_co	ENSG0000	100130274	41910
CCDC167	24.7105	24.4959	21.0101	23.2352	19.2772	23.3894	coiled-coil domain containing 167 [Source:HGNC Symbol;Acc:HGN	chr6:37450	NM_13849	.	.	.	NP_61250	.	protein_co	ENSG0000	154467	21239
CCDC168	0.010233	0.019318	0.015788	0.014685	0.014135	0.002488	coiled-coil domain containing 168 [Source:HGNC Symbol;Acc:HGN	chr13:1033	NM_00114	XM_01152	.	.	NP_00113	XP_011519	protein_co	ENSG0000	643677	26851
CCDC169	0	0	0	0	0	0	coiled-coil domain containing 169 [Source:HGNC Symbol;Acc:HGN	chr13:3674	NM_00114	.	.	.	NP_00113	.	protein_co	ENSG0000	728591	34361
CCDC169-SOHLH2	0.007539	0	0.029482	0	0	0	CCDC169-SOHLH2 readthrough [Source:HGNC Symbol;Acc:HGNC:	chr13:3674	NM_00119	.	.	.	NP_00118	.	protein_co	ENSG0000	100526761	38866
CCDC17	0.263427	0.295279	0.03342	0.293954	0.042954	0.171057	coiled-coil domain containing 17 [Source:HGNC Symbol;Acc:HGN	chr1:46085	NM_00111	XM_01154	.	.	NP_00110	XP_011539	protein_co	ENSG0000	149483	26574
CCDC170	0.012558	0	0.032878	0.057074	0.041751	0.074458	coiled-coil domain containing 170 [Source:HGNC Symbol;Acc:HGN	chr6:15181	NM_02505	XM_01153	.	.	NP_07933	XP_011534	protein_co	ENSG0000	80129	21177
CCDC171	0.708431	0.505556	0.57115	0.466149	0.54648	0.567363	coiled-coil domain containing 171 [Source:HGNC Symbol;Acc:HGN	chr9:15552	NM_17355	XM_01151	.	.	NP_77582	XP_005251	protein_co	ENSG0000	203238	29828
CCDC172	0	0	0	0	0	0	coiled-coil domain containing 172 [Source:HGNC Symbol;Acc:HGN	chr10:1180	NM_19851	XM_00671	.	.	NP_94091	XP_006717	protein_co	ENSG0000	374355	30524
CCDC173	0.180366	0.194111	0.022856	0.20796	0.114505	0.149708	coiled-coil domain containing 173 [Source:HGNC Symbol;Acc:HGN	chr2:17050	NM_00108	XM_01151	.	.	NP_00107	XP_011508	protein_co	ENSG0000	129881	25064
CCDC174	6.62295	5.99215	6.14169	5.94654	6.15849	6.61689	coiled-coil domain containing 174 [Source:HGNC Symbol;Acc:HGN	chr3:14693	NM_01647	.	.	.	NP_05755	XP_016862	protein_co	ENSG0000	51244	28033
CCDC175	0	0	0.019963	0	0	0	coiled-coil domain containing 175 [Source:HGNC Symbol;Acc:HGN	chr14:5995	NM_00116	XM_01153	.	.	NP_00115	XP_011535	protein_co	ENSG0000	729665	19847
CCDC177	0	0.053495	0.0468	0.058939	0.032465	0.030724	coiled-coil domain containing 177 [Source:HGNC Symbol;Acc:HGN	chr14:7003	NM_00127	.	.	.	NP_00125	.	protein_co	ENSG0000	56936	23243
CCDC178	0	0	0	0	0	0	coiled-coil domain containing 178 [Source:HGNC Symbol;Acc:HGN	chr18:3051	NM_00110	XM_01702	.	.	NP_00109	XP_011524	protein_co	ENSG0000	374864	29588
CCDC179	0	0	0	0	0	0	coiled-coil domain containing 179 [Source:HGNC Symbol;Acc:HGN	chr11:2286	NM_00119	.	.	.	NP_00118	XP_011518	protein_co	ENSG0000	100500938	44653
CCDC18	2.63233	2.40956	2.79419	2.54575	4.59254	3.55991	coiled-coil domain containing 18 [Source:HGNC Symbol;Acc:HGN	chr1:93615	NM_00130	XM_01700	.	.	NP_00129	XP_011539	protein_co	ENSG0000	343099	30370
CCDC180	1.57165	1.30835	1.60288	1.59677	1.59305	1.44124	coiled-coil domain containing 180 [Source:HGNC Symbol;Acc:HGN	chr9:10006	NM_02089	.	.	.	NP_06594	.	protein_co	ENSG0000	100499483	29303
CCDC181	0	0	0	0	0	0.032337	coiled-coil domain containing 181 [Source:HGNC Symbol;Acc:HGN	chr1:16933	NM_00130	XM_01700	.	.	NP_00128	XP_005245	protein_co	ENSG0000	57821	28051
CCDC182	0	0	0	0	0.124551	0	coiled-coil domain containing 182 [Source:HGNC Symbol;Acc:HGN	chr17:5582	NM_00128	.	.	.	NP_00126	.	protein_co	ENSG0000	101927581	49392
CCDC183	0.2975	0.065599	0.60635	0.071032	0.116036	0.218876	coiled-coil domain containing 183 [Source:HGNC Symbol;Acc:HGN	chr9:13969	NM_00103	.	.	.	NP_00103	.	protein_co	ENSG0000	84960	28236
CCDC184	0.237002	0.350107	0.270884	0.17081	0.045591	0.17214	coiled-coil domain containing 184 [Source:HGNC Symbol;Acc:HGN	chr12:4857	NM_00101	.	.	.	NP_00101	.	protein_co	ENSG0000	387856	33749
CCDC185	0	0	0	0	0	0	coiled-coil domain containing 185 [Source:HGNC Symbol;Acc:HGN	chr1:22356	NM_15261	.	.	.	NP_68982	.	protein_co	ENSG0000	164127	26654
CCDC186	5.65412	5.92522	5.77146	3.63281	4.7719	6.73463	coiled-coil domain containing 186 [Source:HGNC Symbol;Acc:HGN	chr10:1158	NM_00132	XM_01153	NR_03174	.	NP_00130	XP_005269	protein_co	ENSG0000	100302224	24349
CCDC188	0	0	0	0	0	0	coiled-coil domain containing 188 [Source:HGNC Symbol;Acc:HGN	chr22:2013	.	XM_01153	.	.	.	XP_005261	protein_co	ENSG0000	388849	51899
CCDC189	3.28066	3.76981	3.43397	3.40724	2.40411	2.66766	coiled-coil domain containing 189 [Source:HGNC Symbol;Acc:HGN	chr16:3075	NM_00101	XM_01154	.	.	NP_00101	XP_011544	protein_co	ENSG0000	90835	28078
CCDC190	0	0	0	0	0	0	coiled-coil domain containing 190 [Source:HGNC Symbol;Acc:HGN	chr1:16282	NM_17855	XM_00671	.	.	NP_84864	XP_005245	protein_co	ENSG0000	339512	28736
CCDC191	1.79087	1.52782	1.91662	1.16854	1.63691	1.61492	coiled-coil domain containing 191 [Source:HGNC Symbol;Acc:HGN	chr3:11368	NM_02081	XM_01151	.	.	NP_06586	XP_006713	protein_co	ENSG0000	57577	29272
CCDC192	0.062565	0	0	0	0	0	coiled-coil domain containing 192 [Source:HGNC Symbol;Acc:HGN	chr5:12703	.	.	.	.	NP_00130	.	protein_co	ENSG0000	728586	49566
CCDC22	6.74173	7.03658	5.50827	5.37873	5.36375	4.59857	coiled-coil domain containing 22 [Source:HGNC Symbol;Acc:HGN	chrX:49091	NM_01400	XM_00527	.	.	NP_05472	XP_005272	protein_co	ENSG0000	28952	28909
CCDC24	9.9787	7.99924	11.4583	8.93964	6.80015	6.87465	coiled-coil domain containing 24 [Source:HGNC Symbol;Acc:HGN	chr1:44457	NM_15249	XM_01700	.	.	NP_68971	XP_016855	protein_co	ENSG0000	149473	28688
CCDC25	19.5996	20.7595	21.1337	20.3743	23.6902	21.0195	coiled-coil domain containing 25 [Source:HGNC Symbol;Acc:HGN	chr8:27590	NM_00130	XM_01154	.	.	NP_00129	XP_011542	protein_co	ENSG0000	55246	25591
CCDC27	0	0	0	0	0	0	coiled-coil domain containing 27 [Source:HGNC Symbol;Acc:HGN	chr1:36689	NM_15249	XM_01154	.	.	NP_68970	XP_011539	protein_co	ENSG0000	148870	26546
CCDC28A	7.11114	6.07656	6.86168	5.96327	4.22932	8.80638	coiled-coil domain containing 28A [Source:HGNC Symbol;Acc:HG	chr6:13904	NM_01543	.	.	.	NP_05625	.	protein_co	ENSG0000	25901	21098
CCDC28B	1.62174	1.47451	1.69812	2.7171	2.7647	2.00275	coiled-coil domain containing 28B [Source:HGNC Symbol;Acc:HG	chr1:32665	NM_00130	XM_01154	.	.	NP_00128	XP_011540	protein_co	ENSG0000	79140	28163

CCDC3	8.10167	6.3657	10.0956	8.83473	7.36662	9.41891	coiled-coil domain containing 3 [Source:HGNC Symbol;Acc:HGNC:	chr10:1293	NM_00128	.	.	.	NP_00126	.	protein_co	ENSG0000	83643	23813
CCDC30	0.129598	0.226979	0.168455	0.018093	0.097498	0.093246	coiled-coil domain containing 30 [Source:HGNC Symbol;Acc:HGN	chr1:42948	NM_00108	XM_01154	.	.	NP_00107	XP_011540	protein_co	ENSG0000	728621	26103
CCDC33	0	0	0.074482	0	0	0	coiled-coil domain containing 33 [Source:HGNC Symbol;Acc:HGN	chr15:7452	NM_00128	XM_01152	.	.	NP_00127	XP_005254	protein_co	ENSG0000	80125	26552
CCDC34	9.86378	9.55993	9.84104	13.3421	13.5999	9.08646	coiled-coil domain containing 34 [Source:HGNC Symbol;Acc:HGN	chr11:2736	NM_03077	.	.	.	NP_11039	.	protein_co	ENSG0000	91057	25079
CCDC36	0	0	0	0	0	0	coiled-coil domain containing 36 [Source:HGNC Symbol;Acc:HGN	chr3:49235	NM_00113	XM_01153	.	.	NP_00112	XP_006713	protein_co	ENSG0000	339834	27945
CCDC38	0.037173	0.07976	0.0643	0.201854	0.155226	0.180981	coiled-coil domain containing 38 [Source:HGNC Symbol;Acc:HGN	chr12:9625	NM_18249	XM_01153	.	.	NP_87230	XP_006719	protein_co	ENSG0000	120935	26843
CCDC39	0.072578	0.039265	0.047961	0.062866	0.059976	0	coiled-coil domain containing 39 [Source:HGNC Symbol;Acc:HGN	chr3:18031	NM_18142	.	NR_10998	.	NP_85209	.	protein_co	ENSG0000	101928882	25244
CCDC40	1.28938	1.51534	1.83283	1.92013	1.38817	1.40393	coiled-coil domain containing 40 [Source:HGNC Symbol;Acc:HGN	chr17:7801	NM_00124	XM_01152	.	.	NP_00123	XP_005257	protein_co	ENSG0000	55036	26090
CCDC42	0	0	0	0	0	0.111419	coiled-coil domain containing 42 [Source:HGNC Symbol;Acc:HGN	chr17:8633	NM_00115	.	.	.	NP_00115	.	protein_co	ENSG0000	146849	26528
CCDC43	20.5372	19.8471	21.3557	19.0326	23.8518	22.0758	coiled-coil domain containing 43 [Source:HGNC Symbol;Acc:HGN	chr17:4275	NM_00109	.	.	.	NP_00109	.	protein_co	ENSG0000	124808	26472
CCDC47	85.8019	83.9202	75.5408	74.332	83.812	71.3646	coiled-coil domain containing 47 [Source:HGNC Symbol;Acc:HGN	chr17:6182	NM_02019	XM_00525	.	.	NP_06458	XP_005257	protein_co	ENSG0000	57003	24856
CCDC50	16.7559	15.1193	14.5722	18.8146	20.3228	21.122	coiled-coil domain containing 50 [Source:HGNC Symbol;Acc:HGN	chr3:19098	NM_17490	.	.	.	NP_77756	.	protein_co	ENSG0000	152137	18111
CCDC51	30.0171	29.8909	31.6814	34.4526	30.3242	21.8551	coiled-coil domain containing 51 [Source:HGNC Symbol;Acc:HGN	chr3:48473	NM_00125	.	.	.	NP_00124	.	protein_co	ENSG0000	79714	25714
CCDC53	7.74503	6.94468	7.62145	9.29617	6.92309	7.81804	coiled-coil domain containing 53 [Source:HGNC Symbol;Acc:HGN	chr12:1024	NM_00130	.	.	.	NP_00128	XP_011536	protein_co	ENSG0000	51019	24256
CCDC54	0	0	0	0	0	0	coiled-coil domain containing 54 [Source:HGNC Symbol;Acc:HGN	chr3:10709	NM_03260	.	.	.	NP_11598	.	protein_co	ENSG0000	84692	30703
CCDC57	14.174	14.3718	17.5857	14.5692	12.9991	10.7625	coiled-coil domain containing 57 [Source:HGNC Symbol;Acc:HGN	chr17:8005	NM_19808	XM_01152	.	.	NP_00130	XP_005256	protein_co	ENSG0000	284001	27564
CCDC58	58.5976	52.0788	46.5624	61.3444	65.9861	54.2813	coiled-coil domain containing 58 [Source:HGNC Symbol;Acc:HGN	chr3:12207	NM_00101	XM_01151	.	.	NP_00101	XP_005247	protein_co	ENSG0000	131076	31136
CCDC59	21.6888	20.3101	18.9516	23.564	20.3636	24.8261	coiled-coil domain containing 59 [Source:HGNC Symbol;Acc:HGN	chr12:8274	NM_01416	.	.	.	NP_05488	.	protein_co	ENSG0000	29080	25005
CCDC6	18.7903	18.3995	17.0466	16.3883	19.1643	18.3903	coiled-coil domain containing 6 [Source:HGNC Symbol;Acc:HGNC:	chr10:6154	NM_00543	.	.	.	NP_00542	.	protein_co	ENSG0000	8030	18782
CCDC60	0	0.090218	0	0	0	0	coiled-coil domain containing 60 [Source:HGNC Symbol;Acc:HGN	chr12:1197	NM_17849	XM_01153	.	.	NP_84859	.	protein_co	ENSG0000	160777	28610
CCDC61	1.21386	2.37086	1.5575	1.51977	1.45868	0.937452	coiled-coil domain containing 61 [Source:HGNC Symbol;Acc:HGN	chr19:4649	NM_00126	XM_01152	.	.	NP_00125	XP_005259	protein_co	ENSG0000	729440	33629
CCDC62	0.483477	0.504826	0.409203	0.684748	0.683056	0.748515	coiled-coil domain containing 62 [Source:HGNC Symbol;Acc:HGN	chr12:1232	NM_20143	XM_01153	.	.	NP_95884	XP_006719	protein_co	ENSG0000	84660	30723
CCDC63	0.08216	0.131894	0.096566	0.141961	0.094221	0.165919	coiled-coil domain containing 63 [Source:HGNC Symbol;Acc:HGN	chr12:1112	NM_00128	XM_01153	.	.	NP_00127	XP_006719	protein_co	ENSG0000	160762	26669
CCDC65	0.032868	0.086296	0.072721	0.036594	0.184931	0.1684	coiled-coil domain containing 65 [Source:HGNC Symbol;Acc:HGN	chr12:4929	NM_00128	XM_01153	.	.	NP_00127	.	protein_co	ENSG0000	85478	29937
CCDC66	4.55383	4.06825	3.95535	2.93586	4.07162	3.94595	coiled-coil domain containing 66 [Source:HGNC Symbol;Acc:HGN	chr3:56591	NM_00101	XM_01700	.	.	NP_00101	XP_005265	protein_co	ENSG0000	285331	27709
CCDC67	0	0	0	0	0	0	deuterosome assembly protein 1 [Source:HGNC Symbol;Acc:HGN	chr11:9306	NM_18164	XM_01154	.	.	NP_85759	XP_005273	protein_co	ENSG0000	159989	26344
CCDC68	1.06891	1.49947	1.82153	2.37656	3.0253	4.21598	coiled-coil domain containing 68 [Source:HGNC Symbol;Acc:HGN	chr18:5256	NM_00114	XM_01152	.	.	NP_00113	XP_006722	protein_co	ENSG0000	80323	24350
CCDC69	3.04309	3.2407	4.04186	4.35476	3.5388	3.49036	coiled-coil domain containing 69 [Source:HGNC Symbol;Acc:HGN	chr5:15056	NM_01562	.	.	.	NP_05643	.	protein_co	ENSG0000	26112	24487
CCDC70	0	0	0	0	0	0	coiled-coil domain containing 70 [Source:HGNC Symbol;Acc:HGN	chr13:5243	NM_03129	.	.	.	NP_11258	.	protein_co	ENSG0000	83446	25303
CCDC71	21.484	20.1089	20.9806	24.1016	20.3886	17.5128	coiled-coil domain containing 71 [Source:HGNC Symbol;Acc:HGN	chr3:49199	NM_02290	.	.	.	NP_07505	.	protein_co	ENSG0000	64925	25760
CCDC71L	14.8569	13.8508	14.9484	12.9805	13.0928	17.5908	coiled-coil domain containing 71-like [Source:HGNC Symbol;Acc:H	chr7:10629	NM_17588	.	.	.	NP_78708	.	protein_co	ENSG0000	168455	26685
CCDC73	0	0	0.06113	0	0.03778	0.055849	coiled-coil domain containing 73 [Source:HGNC Symbol;Acc:HGN	chr11:3260	NM_00100	XM_01152	.	.	NP_00100	XP_011518	protein_co	ENSG0000	493860	23261
CCDC74A	7.26792	6.98031	6.85242	6.14787	6.73012	7.21336	coiled-coil domain containing 74A [Source:HGNC Symbol;Acc:HG	chr2:13228	NM_00125	XM_01700	.	.	NP_00124	XP_005263	protein_co	ENSG0000	90557	25197
CCDC74B	1.41291	0.728785	1.50134	1.07346	1.48471	2.10889	coiled-coil domain containing 74B [Source:HGNC Symbol;Acc:HG	chr2:13089	NM_00125	XM_00671	.	.	NP_00124	XP_005263	protein_co	ENSG0000	91409	25267
CCDC77	8.39986	7.65382	5.60432	8.1584	8.58076	7.97998	coiled-coil domain containing 77 [Source:HGNC Symbol;Acc:HGN	chr12:3892	NM_00113	.	.	.	NP_00112	.	protein_co	ENSG0000	84318	28203
CCDC78	1.53303	1.88506	2.0643	0.66788	1.37784	1.01867	coiled-coil domain containing 78 [Source:HGNC Symbol;Acc:HGN	chr16:7709	NM_00103	XM_01152	.	.	NP_00102	XP_006720	protein_co	ENSG0000	124093	14153
CCDC8	0	0	0	0.024427	0	0	coiled-coil domain containing 8 [Source:HGNC Symbol;Acc:HGNC:	chr19:4691	NM_03204	.	.	.	NP_11442	.	protein_co	ENSG0000	83987	25367
CCDC80	0.341502	0.16456	0.19815	0.194938	0.039596	0.390706	coiled-coil domain containing 80 [Source:HGNC Symbol;Acc:HGN	chr3:11231	NM_19951	.	.	.	NP_95580	.	protein_co	ENSG0000	151887	30649
CCDC81	0.364674	0.507161	0.158191	0.294518	0.244503	0.109819	coiled-coil domain containing 81 [Source:HGNC Symbol;Acc:HGN	chr11:8608	NM_00115	XM_00527	.	.	NP_00114	XP_005274	protein_co	ENSG0000	60494	26281
CCDC82	6.22981	4.5727	6.27593	4.92165	6.27184	9.33457	coiled-coil domain containing 82 [Source:HGNC Symbol;Acc:HGN	chr11:9608	NM_00131	XM_01154	.	.	NP_00130	XP_005274	protein_co	ENSG0000	79780	26282
CCDC83	0	0	0.027165	0	0	0	coiled-coil domain containing 83 [Source:HGNC Symbol;Acc:HGN	chr11:8556	NM_00128	XM_01154	.	.	NP_00127	XP_011543	protein_co	ENSG0000	220047	28535
CCDC84	8.49969	9.55362	6.4638	9.16487	6.0666	7.29189	coiled-coil domain containing 84 [Source:HGNC Symbol;Acc:HGN	chr11:1188	NM_19848	XM_01154	.	.	NP_94089	XP_011541	protein_co	ENSG0000	338657	30460
CCDC85A	0.125569	0.02128	0.126937	0	0	0	coiled-coil domain containing 85A [Source:HGNC Symbol;Acc:HG	chr2:56411	NM_00108	XM_00526	.	.	NP_00107	XP_005264	protein_co	ENSG0000	114800	29400
CCDC85B	64.4761	67.3962	66.9659	64.6548	44.3734	71.8841	coiled-coil domain containing 85B [Source:HGNC Symbol;Acc:HG	chr11:6565	NM_00684	.	.	.	NP_00683	.	protein_co	ENSG0000	11007	24926
CCDC85C	17.3652	18.9325	18.519	23.6217	23.1322	18.4798	coiled-coil domain containing 85C [Source:HGNC Symbol;Acc:HG	chr14:9994	NM_00114	XM_01153	.	.	NP_00113	XP_011535	protein_co	ENSG0000	317762	35459
CCDC86	41.3375	45.4502	36.9628	61.6571	48.113	28.4311	coiled-coil domain containing 86 [Source:HGNC Symbol;Acc:HGN	chr11:6060	NM_02409	.	.	.	NP_07700	.	protein_co	ENSG0000	79080	28359
CCDC87	0.204721	0.259851	0.044223	0.111513	0.204564	0.136445	coiled-coil domain containing 87 [Source:HGNC Symbol;Acc:HGN	chr11:6635	NM_01821	.	.	.	NP_06068	.	protein_co	ENSG0000	55231	25579
CCDC88A	2.5185	2.66565	2.91543	2.86796	3.91989	4.67275	coiled-coil domain containing 88A [Source:HGNC Symbol;Acc:HG	chr2:55514	NM_00113	XM_01153	.	.	NP_00112	XP_005264	protein_co	ENSG0000	55704	25523
CCDC88B	12.3528	9.04637	9.0017	11.4973	7.71487	6.48922	coiled-coil domain containing 88B [Source:HGNC Symbol;Acc:HG	chr11:6410	NM_03225	XM_01154	.	.	NP_11562	XP_006718	protein_co	ENSG0000	283234	26757
CCDC88C	5.43401	6.2685	5.76892	5.94391	5.97706	5.74836	coiled-coil domain containing 88C [Source:HGNC Symbol;Acc:HG	chr14:9173	NM_00108	XM_01153	.	.	NP_00107	XP_011535	protein_co	ENSG0000	440193	19967
CCDC89	0.043491	0	0.190747	0.047089	0.050333	0	coiled-coil domain containing 89 [Source:HGNC Symbol;Acc:HGN	chr11:8539	NM_15272	.	.	.	NP_68993	.	protein_co	ENSG0000	220388	26762
CCDC9	9.18934	9.43183	7.29607	9.31191	7.02574	8.4509	coiled-coil domain containing 9 [Source:HGNC Symbol;Acc:HGNC:	chr19:4775	NM_01560	XM_01702	.	.	NP_05641	XP_016882	protein_co	ENSG0000	26093	24560
CCDC90B	16.5219	18.021	21.5752	18.7076	17.8919	23.6618	coiled-coil domain containing 90B [Source:HGNC Symbol;Acc:HG	chr11:8297	NM_00128	XM_00527	.	.	NP_00127	XP_005274	protein_co	ENSG0000	60492	28108
CCDC91	5.72943	5.57687	5.82596	4.92957	6.20291	6.7361	coiled-coil domain containing 91 [Source:HGNC Symbol;Acc:HGN	chr12:2834	NM_01831	XM_01701	.	.	NP_06078	XP_005253	protein_co	ENSG0000	55297	24855
CCDC92	2.86923	2.89897	3.98844	2.98271	2.07235	3.93469	coiled-coil domain containing 92 [Source:HGNC Symbol;Acc:HGN	chr12:1242	NM_00130	XM_00525	.	.	NP_00129	XP_005253	protein_co	ENSG0000	80212	29563
CCDC93	8.91574	8.52104	8.95183	6.55747	8.2803	7.06868	coiled-coil domain containing 93 [Source:HGNC Symbol;Acc:HGN	chr2:11867	NM_01904	XM_01151	.	.	NP_06191	XP_011509	protein_co	ENSG0000	54520	25611
CCDC94	17.5419	16.6614	12.9919	20.3725	15.9565	11.2089	coiled-coil domain containing 94 [Source:HGNC Symbol;Acc:HGN	chr19:4247	NM_01807	.	.	.	NP_06054	.	protein_co	ENSG0000	55702	25518
CCDC96	0.505532	0.247452	0.387062	0.305435	0.369895	0.209591	coiled-coil domain containing 96 [Source:HGNC Symbol;Acc:HGN	chr4:70425	NM_15337	.	.	.	NP_69920	.	protein_co	ENSG0000	257236	26900
CCDC97	12.1138	12.4176	7.34377	11.0007	10.9447	9.19784	coiled-coil domain containing 97 [Source:HGNC Symbol;Acc:HGN	chr19:4181	NM_05284	.	.	.	NP_44308	.	protein_co	ENSG0000	90324	28289

CCER1	0	0	0	0	0	0	coiled-coil glutamate rich protein 1 [Source:HGNC Symbol;Acc:HG	chr12:9134	NM_15263	.	.	.	NP_68985	.	protein_co	ENSG0000	196477	28373
CCER2	0.05416	0.210099	0.144005	0.057226	0.063587	0.184126	coiled-coil glutamate rich protein 2 [Source:HGNC Symbol;Acc:HG	chr19:3939	NM_00124	XM_01152	.	.	NP_00123	XP_01152	protein_co	ENSG0000	643669	44662
CCHCR1	11.0623	12.3191	12.3891	14.8806	12.3408	10.525	coiled-coil alpha-helical rod protein 1 [Source:HGNC Symbol;Acc:	chr6:3111	NM_0011	XM_01701	.	.	NP_00109	XP_01151	protein_co	ENSG0000	54535	13930
CCIN	0.261047	0.1896	0.064479	0.405654	0.257405	0.079306	calicin [Source:HGNC Symbol;Acc:HGNC:1568]	chr9:3616	NM_0058	.	.	.	NP_00588	.	protein_co	ENSG0000	881	1568
CCK	0.548793	0.809995	0.709454	1.61103	0.640141	0.751836	cholecystokinin [Source:HGNC Symbol;Acc:HGNC:1569]	chr3:4229	NM_00072	.	.	.	NP_00072	.	protein_co	ENSG0000	885	1569
CCKAR	0	0	0	0	0	0	cholecystokinin A receptor [Source:HGNC Symbol;Acc:HGNC:157	chr4:2648	NM_00073	.	.	.	NP_00072	.	protein_co	ENSG0000	886	1570
CCKBR	0	0	0	0	0	0	cholecystokinin B receptor [Source:HGNC Symbol;Acc:HGNC:157	chr11:628	NM_17687	XM_00525	.	.	NP_00130	XP_00525	protein_co	ENSG0000	887	1571
CCL1	0	0	0	0	0	0	C-C motif chemokine ligand 1 [Source:HGNC Symbol;Acc:HGNC:1	chr17:326	NM_00298	.	.	.	NP_00297	.	protein_co	ENSG0000	6346	10609
CCL11	0	0	0	0	0	0	C-C motif chemokine ligand 11 [Source:HGNC Symbol;Acc:HGNC:	chr17:3261	NM_00298	.	.	.	NP_00297	.	protein_co	ENSG0000	6356	10610
CCL13	0	0	0	0	0	0	C-C motif chemokine ligand 13 [Source:HGNC Symbol;Acc:HGNC:	chr17:326	NM_0054	.	.	.	NP_00539	.	protein_co	ENSG0000	6357	10611
CCL14	0	0	0	0	0	0	C-C motif chemokine ligand 14 [Source:HGNC Symbol;Acc:HGNC:	chr17:3431	NM_0329	.	.	.	NP_11673	.	protein_co	ENSG0000	6358	10612
CCL15	0	0	0	0	0	0	C-C motif chemokine ligand 15 [Source:HGNC Symbol;Acc:HGNC:	chr17:343	NM_0329	.	.	.	NP_11674	.	protein_co	ENSG0000	6359	10613
CCL16	0	0	0	0	0	0	C-C motif chemokine ligand 16 [Source:HGNC Symbol;Acc:HGNC:	chr17:343	NM_00459	XM_00525	.	.	NP_00458	XP_00525	protein_co	ENSG0000	6360	10614
CCL17	0	0	0	0	0	0	C-C motif chemokine ligand 17 [Source:HGNC Symbol;Acc:HGNC:	chr16:574	NM_00298	.	.	.	NP_00297	.	protein_co	ENSG0000	6361	10615
CCL18	0	0	0	0	0	0	C-C motif chemokine ligand 18 [Source:HGNC Symbol;Acc:HGNC:	chr17:343	NM_00298	.	.	.	NP_00297	.	protein_co	ENSG0000	6362	10616
CCL19	0	0	0	0	0	0	C-C motif chemokine ligand 19 [Source:HGNC Symbol;Acc:HGNC:	chr9:3468	NM_00627	.	.	.	NP_00626	.	protein_co	ENSG0000	6363	10617
CCL2	0	0	0	0	0.145456	0	C-C motif chemokine ligand 2 [Source:HGNC Symbol;Acc:HGNC:1	chr17:325	NM_00298	.	.	.	NP_00297	.	protein_co	ENSG0000	6347	10618
CCL20	3.43811	3.12263	0.819355	0.809622	0.31023	0.135518	C-C motif chemokine ligand 20 [Source:HGNC Symbol;Acc:HGNC:	chr2:2286	NM_0011	.	.	.	NP_00112	.	protein_co	ENSG0000	6364	10619
CCL21	0	0	0	0	0	0	C-C motif chemokine ligand 21 [Source:HGNC Symbol;Acc:HGNC:	chr9:3470	NM_00298	XM_01151	.	.	NP_00298	XP_01151	protein_co	ENSG0000	6366	10620
CCL22	0.031498	0.130346	0.02707	0.2781	0.070943	0.163263	C-C motif chemokine ligand 22 [Source:HGNC Symbol;Acc:HGNC:	chr16:573	NM_00299	XM_01702	.	.	NP_00298	XP_01687	protein_co	ENSG0000	6367	10621
CCL23	0	0	0	0	0	0	C-C motif chemokine ligand 23 [Source:HGNC Symbol;Acc:HGNC:	chr17:3434	NM_0050	.	.	.	NP_00505	.	protein_co	ENSG0000	6368	10622
CCL24	0	0	0	0.176693	0	0.352582	C-C motif chemokine ligand 24 [Source:HGNC Symbol;Acc:HGNC:	chr7:7544	NM_00299	XM_01151	.	.	NP_00298	XP_011514	protein_co	ENSG0000	6369	10623
CCL25	0.102214	0	0.090298	0.226932	0.240402	0	C-C motif chemokine ligand 25 [Source:HGNC Symbol;Acc:HGNC:	chr19:811	NM_0012	XM_01702	.	.	NP_00118	XP_01152	protein_co	ENSG0000	6370	10624
CCL26	0.576012	0.593175	0.681477	0.422882	0.441076	0.603913	C-C motif chemokine ligand 26 [Source:HGNC Symbol;Acc:HGNC:	chr7:7539	NM_00607	.	.	.	NP_00606	XP_016867	protein_co	ENSG0000	10344	10625
CCL27	0.214265	0	0	0.457501	0.249208	0	C-C motif chemokine ligand 27 [Source:HGNC Symbol;Acc:HGNC:	chr9:34661	NM_0066	.	.	.	NP_00665	.	protein_co	ENSG0000	10850	10626
CCL28	0.059882	0.128075	0.05176	0	0	0	C-C motif chemokine ligand 28 [Source:HGNC Symbol;Acc:HGNC:	chr5:4337	NM_0013	.	.	.	NP_00128	.	protein_co	ENSG0000	56477	17700
CCL3	0	0	0	0	0	0	C-C motif chemokine ligand 3 [Source:HGNC Symbol;Acc:HGNC:1	chr17:3441	NM_00298	.	.	.	NP_00297	.	protein_co	ENSG0000	6348	10627
CCL3L1	0	0	0	0	0	0	C-C motif chemokine ligand 3 like 1 [Source:HGNC Symbol;Acc:HG	chr17:345	NM_0010	.	.	.	NP_00100	.	protein_co	ENSG0000	414062	10628
CCL3L3	0.138828	0.142441	0.118934	0	0	0.145526	C-C motif chemokine ligand 3 like 3 [Source:HGNC Symbol;Acc:HG	chr17:345	NM_0010	.	.	.	NP_00100	.	protein_co	ENSG0000	414062	30554
CCL4	0	0	0	0	0	0	C-C motif chemokine ligand 4 [Source:HGNC Symbol;Acc:HGNC:1	chr17:344	NM_00298	.	.	.	NP_00297	.	protein_co	ENSG0000	6351	10630
CCL4L1	0	0	0	0	0	0	chemokine (C-C motif) ligand 4-like 1 [Source:HGNC Symbol;Acc:1	chr17:345	NM_0010	.	.	.	NP_00100	.	protein_co	ENSG0000	9560	10631
CCL4L2	0	0	0	0	0.224448	0	C-C motif chemokine ligand 4 like 2 [Source:HGNC Symbol;Acc:HG	chr17:345	NM_2070	.	.	.	NP_99689	.	protein_co	ENSG0000	388372	24066
CCL5	3.20406	4.21705	0.114065	3.45534	2.5023	6.04474	C-C motif chemokine ligand 5 [Source:HGNC Symbol;Acc:HGNC:1	chr17:341	NM_00127	.	.	.	NP_00126	.	protein_co	ENSG0000	6352	10632
CCL7	0	0	0	0	0	0	C-C motif chemokine ligand 7 [Source:HGNC Symbol;Acc:HGNC:1	chr17:325	NM_00627	.	.	.	NP_00626	.	protein_co	ENSG0000	6354	10634
CCL8	0	0	0	0	0	0	C-C motif chemokine ligand 8 [Source:HGNC Symbol;Acc:HGNC:1	chr17:3264	NM_00562	.	.	.	NP_00561	.	protein_co	ENSG0000	6355	10635
CCM2	17.5149	18.2761	19.2763	21.2992	17.4421	16.6289	CCM2 scaffolding protein [Source:HGNC Symbol;Acc:HGNC:2170	chr7:4503	NM_00102	XM_01151	.	.	NP_00102	XP_006715	protein_co	ENSG0000	83605	21708
CCM2L	0.545185	0.52976	0.832953	0.540412	0.362925	1.21489	CCM2 like scaffolding protein [Source:HGNC Symbol;Acc:HGNC:1	chr20:305	NM_08062	XM_01152	.	.	NP_54219	XP_006723	protein_co	ENSG0000	140706	16153
CCNA1	0	0	0	0	0	0	cyclin A1 [Source:HGNC Symbol;Acc:HGNC:1577]	chr13:370	NM_00111	XM_01153	.	.	NP_00110	XP_011533	protein_co	ENSG0000	8900	1577
CCNA2	41.9289	43.4582	36.1159	69.28	70.8824	52.386	cyclin A2 [Source:HGNC Symbol;Acc:HGNC:1578]	chr4:1227	NM_00123	.	.	.	NP_00122	.	protein_co	ENSG0000	890	1578
CCNB1	135.083	129.432	103.831	255.925	244.926	154.285	cyclin B1 [Source:HGNC Symbol;Acc:HGNC:1579]	chr5:6846	NM_0319	.	.	.	NP_11417	.	protein_co	ENSG0000	891	1579
CCNB1IP1	56.2567	54.7752	48.329	53.1901	46.6486	65.1123	cyclin B1 interacting protein 1 [Source:HGNC Symbol;Acc:HGNC:1	chr14:207	NM_02117	.	.	.	NP_06700	.	protein_co	ENSG0000	57820	19437
CCNB2	58.9848	60.6061	50.4701	98.6	84.6169	68.467	cyclin B2 [Source:HGNC Symbol;Acc:HGNC:1580]	chr15:593	NM_0047	.	.	.	NP_00469	.	protein_co	ENSG0000	9133	1580
CCNB3	1.3406	1.21502	1.13589	1.79238	1.5945	2.12868	cyclin B3 [Source:HGNC Symbol;Acc:HGNC:18709]	chrX:4996	NM_03303	XM_01702	.	.	NP_14902	XP_01152	protein_co	ENSG0000	85417	18709
CCNC	37.5276	37.1298	39.5144	50.4498	54.6694	51.076	cyclin C [Source:HGNC Symbol;Acc:HGNC:1581]	chr6:9999	NM_00101	XM_00671	.	.	NP_00101	XP_006715	protein_co	ENSG0000	892	1581
CCND1	152.592	170.574	189.957	134.189	145.517	139.226	cyclin D1 [Source:HGNC Symbol;Acc:HGNC:1582]	chr11:694	NM_05305	.	.	.	NP_44428	.	protein_co	ENSG0000	595	1582
CCND2	0	0	0	0.014587	0	0.013163	cyclin D2 [Source:HGNC Symbol;Acc:HGNC:1583]	chr12:4382	NM_00175	.	.	.	NP_00175	.	protein_co	ENSG0000	894	1583
CCND3	25.9019	27.2086	26.7864	37.1705	31.605	24.0686	cyclin D3 [Source:HGNC Symbol;Acc:HGNC:1585]	chr6:41902	NM_00113	XM_01151	.	.	NP_00112	XP_011513	protein_co	ENSG0000	896	1585
CCNDBP1	11.5622	10.39	12.4969	9.03392	7.80987	9.37774	cyclin D1 binding protein 1 [Source:HGNC Symbol;Acc:HGNC:158	chr15:4347	NM_01214	.	.	.	NP_03627	.	protein_co	ENSG0000	23582	1587
CCNE1	29.7851	27.7922	22.68	30.4811	29.8902	22.1087	cyclin E1 [Source:HGNC Symbol;Acc:HGNC:1589]	chr19:303	NM_00123	XM_01152	.	.	NP_00122	XP_006723	protein_co	ENSG0000	898	1589
CCNE2	12.7792	11.4744	10.272	15.7052	19.3668	15.5488	cyclin E2 [Source:HGNC Symbol;Acc:HGNC:1590]	chr8:9583	NM_05774	XM_01151	.	.	NP_47709	XP_01686	protein_co	ENSG0000	9134	1590
CCNF	15.9592	17.0377	17.6741	23.7769	21.1556	16.6475	cyclin F [Source:HGNC Symbol;Acc:HGNC:1591]	chr16:247	NM_00132	XM_01702	.	.	NP_00131	XP_01687	protein_co	ENSG0000	899	1591
CCNG1	47.451	46.8877	82.5431	55.3161	81.7761	91.9338	cyclin G1 [Source:HGNC Symbol;Acc:HGNC:1592]	chr5:1628	NM_0040	XM_01153	.	.	NP_00405	XP_011532	protein_co	ENSG0000	900	1592
CCNG2	12.489	10.2965	13.6402	10.0831	13.4358	23.6633	cyclin G2 [Source:HGNC Symbol;Acc:HGNC:1593]	chr4:7807	NM_00435	XM_01153	.	.	NP_00434	XP_01153	protein_co	ENSG0000	901	1593
CCNH	33.9994	28.8908	35.8452	42.57	42.6581	45.5891	cyclin H [Source:HGNC Symbol;Acc:HGNC:1594]	chr5:8668	NM_00119	XM_00524	.	.	NP_00118	XP_005248	protein_co	ENSG0000	902	1594
CCNI	68.4613	65.8029	70.4351	56.0035	63.3514	85.4235	cyclin I [Source:HGNC Symbol;Acc:HGNC:1595]	chr4:7796	NM_00683	.	.	.	NP_00682	.	protein_co	ENSG0000	10983	1595
CCNI2	0.331606	0.424348	0.92187	0.639292	0.404815	0.078321	cyclin I family member 2 [Source:HGNC Symbol;Acc:HGNC:33869	chr5:1320	NM_00103	.	.	.	NP_00103	.	protein_co	ENSG0000	645121	33869
CCNJ	11.0021	12.1198	9.99969	11.2609	12.737	13.8263	cyclin J [Source:HGNC Symbol;Acc:HGNC:23434]	chr10:978	NM_00113	XM_01701	.	.	NP_00112	XP_011538	protein_co	ENSG0000	54619	23434
CCNJL	2.32105	2.20429	2.56228	3.25966	3.58022	2.76228	cyclin J like [Source:HGNC Symbol;Acc:HGNC:25876]	chr5:1596	NM_0013	XM_01700	NR_13176	.	NP_00129	XP_00526	protein_co	ENSG0000	79616	25876
CCNK	21.0111	20.9732	20.7861	22.4567	22.0937	23.8136	cyclin K [Source:HGNC Symbol;Acc:HGNC:1596]	chr14:9994	NM_00109	XM_01153	.	.	NP_00109	XP_005268	protein_co	ENSG0000	8812	1596

CCNL1	53.2213	46.2505	50.8677	37.7712	45.9639	48.4485	cyclin L1 [Source:HGNC Symbol;Acc:HGNC:20569]	chr3:15686	NM_00130	XM_01700	.	.	NP_00129	XP_005247	protein_co	ENSG0000	57018	20569
CCNL2	75.2538	68.3957	92.4347	63.938	65.4927	66.5773	cyclin L2 [Source:HGNC Symbol;Acc:HGNC:20570]	chr1:13218	NM_00103	XM_01154	.	.	NP_00103	XP_011540	protein_co	ENSG0000	81669	20570
CCNO	9.20095	8.79828	8.8232	5.62562	5.82852	6.27295	cyclin O [Source:HGNC Symbol;Acc:HGNC:18576]	chr5:54526	NM_02114	.	.	.	NP_06697	.	protein_co	ENSG0000	10309	18576
CCNT1	19.295	20.117	19.7583	19.2857	22.8731	23.9518	cyclin T1 [Source:HGNC Symbol;Acc:HGNC:1599]	chr12:4908	NM_00124	XM_01702	.	.	NP_00123	XP_016875	protein_co	ENSG0000	904	1599
CCNT2	10.5513	11.0423	9.63991	9.31799	10.8784	11.7554	cyclin T2 [Source:HGNC Symbol;Acc:HGNC:1600]	chr2:13567	NM_00124	.	.	.	NP_00123	.	protein_co	ENSG0000	905	1600
CCNY	24.1248	25.3787	20.7903	22.8898	22.0965	23.5942	cyclin Y [Source:HGNC Symbol;Acc:HGNC:23354]	chr10:3553	NM_00128	XM_01151	.	.	NP_00126	XP_006717	protein_co	ENSG0000	219771	23354
CCNYL1	6.64589	7.06252	8.15085	8.16485	8.59512	9.21041	cyclin Y like 1 [Source:HGNC Symbol;Acc:HGNC:26868]	chr2:20857	NM_00114	XM_00524	NR_03993	.	NP_00113	XP_005246	protein_co	ENSG0000	100616361	26868
CCP110	6.61687	7.21396	7.71357	7.11254	9.29665	11.2185	centriolar coiled-coil protein 110 [Source:HGNC Symbol;Acc:HGN	chr16:1953	NM_00119	XM_01154	.	.	NP_00118	XP_005255	protein_co	ENSG0000	9738	24342
CCPG1	4.49559	4.31614	3.11577	1.6477	2.83169	4.40796	cell cycle progression 1 [Source:HGNC Symbol;Acc:HGNC:24227]	chr15:5560	NM_00120	.	NR_03035	.	NP_00119	.	protein_co	ENSG0000	693213	24227
CCR1	0	0	0.025004	0.064481	0	0.174881	C-C motif chemokine receptor 1 [Source:HGNC Symbol;Acc:HGNC	chr3:46205	NM_00129	.	.	.	NP_00128	.	protein_co	ENSG0000	1230	1602
CCR10	1.14383	0.757889	0.725677	0.30109	0.337876	0.481847	C-C motif chemokine receptor 10 [Source:HGNC Symbol;Acc:HGN	chr17:4083	NM_01660	.	.	.	NP_05768	.	protein_co	ENSG0000	2826	4474
CCR2	0	0	0	0	0.03266	0	C-C motif chemokine receptor 2 [Source:HGNC Symbol;Acc:HGNC	chr3:46395	NM_00112	XM_01153	.	.	NP_00111	XP_011532	protein_co	ENSG0000	729230	1603
CCR3	0	0.038327	0.064122	0	0.060204	0	C-C motif chemokine receptor 3 [Source:HGNC Symbol;Acc:HGNC	chr3:46205	NM_00116	XM_01153	.	.	NP_00115	XP_006713	protein_co	ENSG0000	1232	1604
CCR4	0.028804	0.060348	0	0.161267	0.096672	0.058137	C-C motif chemokine receptor 4 [Source:HGNC Symbol;Acc:HGNC	chr3:32993	NM_00550	.	.	.	NP_00549	XP_016861	protein_co	ENSG0000	1233	1605
CCR5	0	0	0	0	0	0	C-C motif chemokine receptor 5 (gene/pseudogene) [Source:HGN	chr3:46412	NM_00057	.	.	.	NP_00057	.	protein_co	ENSG0000	1234	1606
CCR6	0.139076	0.23667	0.07205	0.031577	0.092804	0.13633	C-C motif chemokine receptor 6 [Source:HGNC Symbol;Acc:HGNC	chr6:16752	NM_00436	.	.	.	NP_00435	.	protein_co	ENSG0000	1235	1607
CCR7	0	0.071513	0.030527	0.046362	0.081188	0.038029	C-C motif chemokine receptor 7 [Source:HGNC Symbol;Acc:HGNC	chr17:3871	NM_00130	.	.	.	NP_00128	.	protein_co	ENSG0000	1236	1608
CCR8	0	0	0	0	0	0	C-C motif chemokine receptor 8 [Source:HGNC Symbol;Acc:HGNC	chr3:39371	NM_00520	.	.	.	NP_00519	.	protein_co	ENSG0000	1237	1609
CCR9	0	0	0	0.034783	0.036097	0	C-C motif chemokine receptor 9 [Source:HGNC Symbol;Acc:HGNC	chr3:45864	NM_00125	XM_01153	.	.	NP_00124	XP_011531	protein_co	ENSG0000	10803	1610
CCRL2	0	0	0	0	0.103071	0.272116	C-C motif chemokine receptor like 2 [Source:HGNC Symbol;Acc:H	chr3:46448	NM_00113	XM_01153	.	.	NP_00112	XP_011532	protein_co	ENSG0000	9034	1612
CCS	12.5771	12.661	14.6462	14.4655	13.9265	11.6572	copper chaperone for superoxide dismutase [Source:HGNC Symb	chr11:6635	NM_00512	.	.	.	NP_00511	.	protein_co	ENSG0000	9973	1613
CCSAP	5.40737	6.95189	7.43467	6.92606	8.08277	7.88593	centriole, cilia and spindle associated protein [Source:HGNC Sym	chr1:22945	NM_14525	XM_01154	.	.	NP_66030	XP_011542	protein_co	ENSG0000	126731	29578
CCSER1	0.638652	0.593174	0.805924	0.808645	0.651348	0.867021	coiled-coil serine rich protein 1 [Source:HGNC Symbol;Acc:HGNC:	chr4:91048	NM_00114	XM_01153	.	.	NP_00113	XP_011530	protein_co	ENSG0000	401145	29349
CCSER2	4.79388	4.87204	6.54221	4.40746	6.2265	6.78823	coiled-coil serine rich protein 2 [Source:HGNC Symbol;Acc:HGNC:	chr10:8608	NM_00128	XM_01701	.	.	NP_00127	XP_005269	protein_co	ENSG0000	54462	29197
CCT2	272.547	261.04	245.788	341.736	316.532	267.237	chaperonin containing TCP1 subunit 2 [Source:HGNC Symbol;Acc	chr12:6997	NM_00119	.	.	.	NP_00118	.	protein_co	ENSG0000	10576	1615
CCT3	381.763	376.053	324.003	524.908	429.702	356.926	chaperonin containing TCP1 subunit 3 [Source:HGNC Symbol;Acc	chr1:15627	NM_00100	.	.	.	NP_00100	.	protein_co	ENSG0000	7203	1616
CCT4	221.827	221.451	181.653	297.732	262.208	212.065	chaperonin containing TCP1 subunit 4 [Source:HGNC Symbol;Acc	chr2:62095	NM_00125	.	.	.	NP_00124	.	protein_co	ENSG0000	10575	1617
CCT5	276.102	261.942	215.377	338.142	310.311	257.862	chaperonin containing TCP1 subunit 5 [Source:HGNC Symbol;Acc	chr5:10250	NM_00130	XM_01151	.	.	NP_00129	XP_011512	protein_co	ENSG0000	22948	1618
CCT6A	212.535	210.198	185.536	270.693	273.239	212.656	chaperonin containing TCP1 subunit 6A [Source:HGNC Symbol;Ac	chr7:56119	NM_00100	.	.	.	NP_00100	.	protein_co	ENSG0000	908	1620
CCT6B	0.257678	0.462776	0.514875	0.153725	0.379374	1.00683	chaperonin containing TCP1 subunit 6B [Source:HGNC Symbol;Ac	chr17:3325	NM_00119	.	.	.	NP_00118	.	protein_co	ENSG0000	10693	1621
CCT7	327.044	322.735	283.964	428.718	360.321	290.01	chaperonin containing TCP1 subunit 7 [Source:HGNC Symbol;Acc	chr2:73461	NM_00100	XM_01153	.	.	NP_00100	XP_011530	protein_co	ENSG0000	10574	1622
CCT8	279.481	241.505	240.682	284.739	313.339	271.71	chaperonin containing TCP1 subunit 8 [Source:HGNC Symbol;Acc	chr21:3042	NM_00128	.	.	.	NP_00126	.	protein_co	ENSG0000	10694	1623
CCT8L2	0	0	0	0	0	0	chaperonin containing TCP1 subunit 8 like 2 [Source:HGNC Symb	chr22:1707	NM_01440	.	.	.	NP_05522	.	protein_co	ENSG0000	150160	15553
CCZ1	12.1473	13.9975	12.5648	16.5359	14.4878	13.6648	CCZ1 homolog, vacuolar protein trafficking and biogenesis associ	chr7:59383	NM_01562	.	.	.	NP_05643	.	protein_co	ENSG0000	51622	21691
CCZ1B	4.35422	3.9253	3.57404	3.74254	4.71642	5.63575	CCZ1 homolog B, vacuolar protein trafficking and biogenesis asso	chr7:67937	NM_19809	.	.	.	NP_93276	.	protein_co	ENSG0000	221960	21717
CD101	0.400382	0.554403	0.257155	0.309035	0.417327	0.252003	CD101 molecule [Source:HGNC Symbol;Acc:HGNC:5949]	chr1:11754	NM_00125	.	.	.	NP_00124	.	protein_co	ENSG0000	9398	5949
CD109	18.1464	20.2877	18.9098	24.6017	30.5818	22.5853	CD109 molecule [Source:HGNC Symbol;Acc:HGNC:21685]	chr6:74405	NM_00115	XM_01701	.	.	NP_00115	XP_005248	protein_co	ENSG0000	135228	21685
CD14	0.168453	0.236737	0.040975	0.060642	0.065044	0.103651	CD14 molecule [Source:HGNC Symbol;Acc:HGNC:1628]	chr5:14001	NM_00059	.	.	.	NP_00058	.	protein_co	ENSG0000	929	1628
CD151	182.615	168.949	166.291	165.132	137.391	142.684	CD151 molecule (Raph blood group) [Source:HGNC Symbol;Acc:H	chr11:8328	NM_00103	.	.	.	NP_00103	.	protein_co	ENSG0000	977	1630
CD160	0	0	0	0	0.053663	0	CD160 molecule [Source:HGNC Symbol;Acc:HGNC:17013]	chr1:14561	NM_00705	XM_01150	.	.	NP_00898	XP_005272	protein_co	ENSG0000	11126	17013
CD163	0.01581	0	0.01374	0	0.016209	0	CD163 molecule [Source:HGNC Symbol;Acc:HGNC:1631]	chr12:7623	NM_00424	XM_00525	.	.	NP_00423	XP_005253	protein_co	ENSG0000	9332	1631
CD163L1	2.74634	2.44034	2.378	2.49049	2.62006	2.45391	CD163 molecule like 1 [Source:HGNC Symbol;Acc:HGNC:30375]	chr12:7507	NM_00129	XM_01152	.	.	NP_00128	XP_011518	protein_co	ENSG0000	283316	30375
CD164	48.4361	58.4019	50.3472	51.8949	58.2349	72.5221	CD164 molecule [Source:HGNC Symbol;Acc:HGNC:1632]	chr6:10968	NM_00114	.	.	.	NP_00113	.	protein_co	ENSG0000	8763	1632
CD164L2	3.43865	3.88787	6.27189	4.13162	2.50991	4.55253	CD164 molecule like 2 [Source:HGNC Symbol;Acc:HGNC:32043]	chr1:27705	NM_20739	XM_01154	.	.	NP_99728	XP_005245	protein_co	ENSG0000	388611	32043
CD177	0	0.033161	0	0	0	0	CD177 molecule [Source:HGNC Symbol;Acc:HGNC:30072]	chr19:4385	NM_02040	.	.	.	NP_06513	XP_016882	protein_co	ENSG0000	57126	30072
CD180	0	0	0	0	0	0	CD180 molecule [Source:HGNC Symbol;Acc:HGNC:6726]	chr5:66475	NM_00558	.	.	.	NP_00557	XP_005248	protein_co	ENSG0000	4064	6726
CD19	0	0.035227	0.061345	0.038064	0.040713	0.116717	CD19 molecule [Source:HGNC Symbol;Acc:HGNC:1633]	chr16:2894	NM_00117	XM_00672	.	.	NP_00117	XP_006721	protein_co	ENSG0000	930	1633
CD1A	0	0	0	0	0	0	CD1a molecule [Source:HGNC Symbol;Acc:HGNC:1634]	chr1:15822	NM_00176	XM_01151	.	.	NP_00130	.	protein_co	ENSG0000	909	1634
CD1B	0	0	0	0	0	0	CD1b molecule [Source:HGNC Symbol;Acc:HGNC:1635]	chr1:15829	NM_00176	XM_01151	.	.	NP_00175	XP_011508	protein_co	ENSG0000	910	1635
CD1C	0	0	0	0	0	0	CD1c molecule [Source:HGNC Symbol;Acc:HGNC:1636]	chr1:15825	NM_00176	.	.	.	NP_00175	.	protein_co	ENSG0000	911	1636
CD1D	0.070875	0.036112	0.061245	0.078058	0	0.148244	CD1d molecule [Source:HGNC Symbol;Acc:HGNC:1637]	chr1:15814	NM_00176	XM_01151	.	.	NP_00175	XP_011508	protein_co	ENSG0000	912	1637
CD1E	0	0	0	0	0	0	CD1e molecule [Source:HGNC Symbol;Acc:HGNC:1638]	chr1:15832	NM_00104	XM_01151	.	.	NP_00103	XP_011508	protein_co	ENSG0000	913	1638
CD2	0	0	0	0	0	0	CD2 molecule [Source:HGNC Symbol;Acc:HGNC:1639]	chr1:11729	NM_00176	.	.	.	NP_00175	.	protein_co	ENSG0000	914	1639
CD200	0	0	0.041609	0.106677	0.053808	0.048828	CD200 molecule [Source:HGNC Symbol;Acc:HGNC:7203]	chr3:11205	NM_00100	XM_01700	.	.	NP_00100	XP_005247	protein_co	ENSG0000	4345	7203
CD200R1	0.085174	0	0	0.09651	0.047304	0.046219	CD200 receptor 1 [Source:HGNC Symbol;Acc:HGNC:24235]	chr3:11264	NM_13880	.	.	.	NP_62016	.	protein_co	ENSG0000	131450	24235
CD200R1L	0	0	0	0	0	0	CD200 receptor 1 like [Source:HGNC Symbol;Acc:HGNC:24665]	chr3:11253	NM_00100	XM_01151	.	.	NP_00100	XP_011511	protein_co	ENSG0000	344807	24665
CD207	0	0	0	0	0	0	CD207 molecule [Source:HGNC Symbol;Acc:HGNC:17935]	chr2:71057	NM_01571	XM_01153	.	.	NP_05653	XP_011531	protein_co	ENSG0000	50489	17935
CD209	0	0	0	0	0	0	CD209 molecule [Source:HGNC Symbol;Acc:HGNC:1641]	chr19:7804	NM_00114	XM_00527	.	.	NP_00113	XP_005272	protein_co	ENSG0000	30835	1641
CD22	1.0453	1.03	1.84668	0.874813	0.648216	1.14176	CD22 molecule [Source:HGNC Symbol;Acc:HGNC:1643]	chr19:3582	NM_00118	.	NR_04982	.	NP_00117	.	protein_co	ENSG0000	100847070	1643

CD226	0.204483	0.23262	0.130277	0.118093	0.114261	0.045158	CD226 molecule [Source:HGNC Symbol;Acc:HGNC:16961]	chr18:6752	NM_00130	XM_00526	.	.	NP_00129	XP_00526	protein_co	ENSG0000	10666	16961
CD244	0	0	0.024537	0	0	0	CD244 molecule [Source:HGNC Symbol;Acc:HGNC:18171]	chr1:1607	NM_00116	XM_01150	.	.	NP_00116	XP_01150	protein_co	ENSG0000	51744	18171
CD247	0	0	0	0.051256	0	0.101592	CD247 molecule [Source:HGNC Symbol;Acc:HGNC:1677]	chr1:1673	NM_00073	XM_01151	.	.	NP_00072	XP_01150	protein_co	ENSG0000	919	1677
CD248	0	0	0	0	0	0	CD248 molecule [Source:HGNC Symbol;Acc:HGNC:18219]	chr11:6608	NM_02040	.	.	.	NP_06513	.	protein_co	ENSG0000	57124	18219
CD27	0	0	0	0	0	0	CD27 molecule [Source:HGNC Symbol;Acc:HGNC:11922]	chr12:6554	NM_00124	.	.	.	NP_00123	.	protein_co	ENSG0000	939	11922
CD274	1.60957	1.7732	1.1213	1.29861	1.02567	1.66567	CD274 molecule [Source:HGNC Symbol;Acc:HGNC:17635]	chr9:5450	NM_00126	.	.	.	NP_00125	.	protein_co	ENSG0000	29126	17635
CD276	29.4006	30.3914	29.1975	31.9036	28.421	27.606	CD276 molecule [Source:HGNC Symbol;Acc:HGNC:19137]	chr15:7397	NM_00102	XM_01702	.	.	NP_00101	XP_00525	protein_co	ENSG0000	80381	19137
CD28	0	0	0	0	0	0	CD28 molecule [Source:HGNC Symbol;Acc:HGNC:1653]	chr2:2045	NM_00124	XM_01151	.	.	NP_00123	XP_01151	protein_co	ENSG0000	940	1653
CD2AP	14.4009	15.4701	14.7587	14.7336	17.2896	15.9177	CD2 associated protein [Source:HGNC Symbol;Acc:HGNC:14258]	chr6:4744	NM_01212	XM_01151	.	.	NP_03625	XP_00524	protein_co	ENSG0000	23607	14258
CD2BP2	33.0914	34.7652	29.1698	36.1744	31.2975	26.5499	CD2 cytoplasmic tail binding protein 2 [Source:HGNC Symbol;Acc	chr16:3036	NM_00124	.	.	.	NP_00123	.	protein_co	ENSG0000	10421	1656
CD300A	0.069906	0	0	0.137773	0	0.043739	CD300a molecule [Source:HGNC Symbol;Acc:HGNC:19319]	chr17:7246	NM_00125	XM_01152	.	.	NP_00124	XP_00525	protein_co	ENSG0000	11314	19319
CD300C	0.243539	0.292831	0.252679	0.210297	0	0.053111	CD300c molecule [Source:HGNC Symbol;Acc:HGNC:19320]	chr17:7253	NM_00667	.	.	.	NP_00666	.	protein_co	ENSG0000	10871	19320
CD300E	0	0	0	0	0	0	CD300e molecule [Source:HGNC Symbol;Acc:HGNC:28874]	chr17:7260	NM_18144	.	.	.	NP_85211	.	protein_co	ENSG0000	342510	28874
CD300LB	0	0	0	0	0	0	CD300 molecule like family member b [Source:HGNC Symbol;Acc	chr17:7251	NM_17489	.	.	.	NP_77755	.	protein_co	ENSG0000	124599	30811
CD300LD	0	0	0	0	0	0	CD300 molecule like family member d [Source:HGNC Symbol;Acc	chr17:7257	NM_00111	.	.	.	NP_00110	.	protein_co	ENSG0000	100131439	16848
CD300LF	0	0	0	0	0	0	CD300 molecule like family member f [Source:HGNC Symbol;Acc:	chr17:7266	NM_00128	XM_01702	.	.	NP_00127	XP_01687	protein_co	ENSG0000	146722	29883
CD300LG	0.04663	0	0	0	0	0	CD300 molecule like family member g [Source:HGNC Symbol;Acc	chr17:4192	NM_00116	XM_00672	.	.	NP_00116	XP_00672	protein_co	ENSG0000	146894	30455
CD302	0	0	0	0	0	0	CD302 molecule [Source:HGNC Symbol;Acc:HGNC:30843]	chr2:1605	NM_00119	.	.	.	NP_00118	.	protein_co	ENSG0000	9936	30843
CD320	74.2137	74.7208	52.2701	101.131	75.1833	50.1556	CD320 molecule [Source:HGNC Symbol;Acc:HGNC:16692]	chr19:8367	NM_00116	.	.	.	NP_00115	.	protein_co	ENSG0000	51293	16692
CD33	2.88772	3.00013	3.49401	11.6671	10.7832	8.30651	CD33 molecule [Source:HGNC Symbol;Acc:HGNC:1659]	chr19:5172	NM_00108	XM_01152	.	.	NP_00107	XP_01152	protein_co	ENSG0000	945	1659
CD34	0	0	0.028683	0	0	0.033984	CD34 molecule [Source:HGNC Symbol;Acc:HGNC:1662]	chr1:2080	NM_00102	XM_01151	.	.	NP_00102	.	protein_co	ENSG0000	947	1662
CD36	0.066162	0.09507	0.076247	0.054265	0.096462	0.045279	CD36 molecule [Source:HGNC Symbol;Acc:HGNC:1663]	chr7:7999	NM_00007	XM_01151	.	.	NP_00006	XP_00525	protein_co	ENSG0000	948	1663
CD37	0.08681	0.264487	0.150548	0.096017	0.101469	0.21289	CD37 molecule [Source:HGNC Symbol;Acc:HGNC:1666]	chr19:4983	NM_00104	XM_01702	.	.	NP_00103	XP_00525	protein_co	ENSG0000	951	1666
CD38	0	0	0.013323	0	0	0	CD38 molecule [Source:HGNC Symbol;Acc:HGNC:1667]	chr4:1577	NM_00177	.	.	.	NP_00176	.	protein_co	ENSG0000	952	1667
CD3D	0	0	0	0	0	0	CD3d molecule [Source:HGNC Symbol;Acc:HGNC:1673]	chr11:1182	NM_00073	.	.	.	NP_00072	XP_01687	protein_co	ENSG0000	915	1673
CD3E	0	0	0	0	0	0	CD3e molecule [Source:HGNC Symbol;Acc:HGNC:1674]	chr11:1181	NM_00073	.	.	.	NP_00072	.	protein_co	ENSG0000	916	1674
CD3EAP	21.9376	21.3812	20.0406	27.2361	24.5683	15.9652	CD3e molecule associated protein [Source:HGNC Symbol;Acc:HG	chr19:4588	NM_00129	XM_01702	.	.	NP_00128	XP_01688	protein_co	ENSG0000	10849	24219
CD3G	0	0	0	0	0	0	CD3g molecule [Source:HGNC Symbol;Acc:HGNC:1675]	chr11:1182	NM_00007	XM_00671	.	.	NP_00006	XP_00671	protein_co	ENSG0000	917	1675
CD4	0	0	0	0	0	0	CD4 molecule [Source:HGNC Symbol;Acc:HGNC:1678]	chr12:6898	NM_00061	.	.	.	NP_00060	.	protein_co	ENSG0000	920	1678
CD40	15.3627	16.6985	13.8673	15.4301	14.0023	12.7525	CD40 molecule [Source:HGNC Symbol;Acc:HGNC:11919]	chr20:4474	NM_00125	XM_00526	.	.	NP_00124	XP_00526	protein_co	ENSG0000	958	11919
CD40LG	0	0	0	0	0	0	CD40 ligand [Source:HGNC Symbol;Acc:HGNC:11935]	chrX:1357	NM_00007	.	.	.	NP_00006	.	protein_co	ENSG0000	959	11935
CD44	150.941	156.448	173.958	186.683	191.663	191.865	CD44 molecule (Indian blood group) [Source:HGNC Symbol;Acc:H	chr11:3516	NM_00061	XM_01152	.	.	NP_00060	XP_00525	protein_co	ENSG0000	960	1681
CD46	41.7226	41.4546	56.3252	38.2471	45.4186	53.1124	CD46 molecule [Source:HGNC Symbol;Acc:HGNC:6953]	chr1:2079	NM_00238	XM_01700	.	.	NP_00238	XP_01150	protein_co	ENSG0000	4179	6953
CD47	28.3334	23.7951	26.744	16.9713	18.7312	17.8937	CD47 molecule [Source:HGNC Symbol;Acc:HGNC:1682]	chr3:1077	NM_00177	XM_00524	.	.	NP_00176	XP_00524	protein_co	ENSG0000	961	1682
CD48	0	0	0	0	0	0	CD48 molecule [Source:HGNC Symbol;Acc:HGNC:1683]	chr1:1606	NM_00125	.	.	.	NP_00124	.	protein_co	ENSG0000	962	1683
CD5	0	0	0	0	0.054577	0	CD5 molecule [Source:HGNC Symbol;Acc:HGNC:1685]	chr11:6086	NM_01420	.	.	.	NP_05502	.	protein_co	ENSG0000	921	1685
CD52	0	0	0	0.713671	0	0.44391	CD52 molecule [Source:HGNC Symbol;Acc:HGNC:1804]	chr1:2660	NM_00180	.	.	.	NP_00179	.	protein_co	ENSG0000	1043	1804
CD53	0.053589	0	0	0.180369	0	0	CD53 molecule [Source:HGNC Symbol;Acc:HGNC:1686]	chr1:1114	NM_00056	XM_00671	.	.	NP_00055	.	protein_co	ENSG0000	963	1686
CD55	17.8984	19.9357	14.4489	14.8873	15.7278	27.7419	CD55 molecule (Cromer blood group) [Source:HGNC Symbol;Acc:	chr1:2074	NM_00057	.	.	.	NP_00056	XP_01685	protein_co	ENSG0000	1604	2665
CD58	5.65975	5.17955	7.71196	7.23846	7.27788	9.91723	CD58 molecule [Source:HGNC Symbol;Acc:HGNC:1688]	chr1:1170	NM_00114	.	.	.	NP_00113	XP_01685	protein_co	ENSG0000	965	1688
CD59	104.318	108.298	115.974	89.1759	88.6637	83.333	CD59 molecule [Source:HGNC Symbol;Acc:HGNC:1689]	chr11:3371	NM_00061	.	.	.	NP_00060	.	protein_co	ENSG0000	966	1689
CD5L	0.033197	0	0	0	0	0	CD5 molecule like [Source:HGNC Symbol;Acc:HGNC:1690]	chr1:1578	NM_00589	.	.	.	NP_00588	XP_00524	protein_co	ENSG0000	922	1690
CD6	0	0	0	0	0	0	CD6 molecule [Source:HGNC Symbol;Acc:HGNC:1691]	chr11:6073	NM_00125	XM_01154	.	.	NP_00124	XP_00671	protein_co	ENSG0000	923	1691
CD63	313.162	318.895	295.28	334.211	277.625	278.515	CD63 molecule [Source:HGNC Symbol;Acc:HGNC:1692]	chr12:5611	NM_00125	.	.	.	NP_00124	.	protein_co	ENSG0000	967	1692
CD68	8.55497	7.68352	4.96436	7.17776	6.63053	2.31903	CD68 molecule [Source:HGNC Symbol;Acc:HGNC:1693]	chr17:7482	NM_00104	.	.	.	NP_00103	.	protein_co	ENSG0000	968	1693
CD69	0	0	0	0	0	0	CD69 molecule [Source:HGNC Symbol;Acc:HGNC:1694]	chr12:9905	NM_00178	.	.	.	NP_00177	.	protein_co	ENSG0000	969	1694
CD7	0.161775	0.100926	0.138199	0.21974	0	0	CD7 molecule [Source:HGNC Symbol;Acc:HGNC:1695]	chr17:8027	NM_00613	XM_01152	.	.	NP_00612	XP_01152	protein_co	ENSG0000	924	1695
CD70	0.60205	0.591145	1.02348	0.77157	0.986061	0.932513	CD70 molecule [Source:HGNC Symbol;Acc:HGNC:11937]	chr19:6583	NM_00125	.	.	.	NP_00124	XP_01688	protein_co	ENSG0000	970	11937
CD72	0.461571	0.20532	0.808194	0.197099	0.273637	0.375683	CD72 molecule [Source:HGNC Symbol;Acc:HGNC:1696]	chr9:3560	NM_00178	XM_01151	.	.	NP_00177	XP_00671	protein_co	ENSG0000	971	1696
CD74	0.567697	0.666323	0.781987	0.600398	0.297422	0.411731	CD74 molecule [Source:HGNC Symbol;Acc:HGNC:1697]	chr5:1497	NM_00102	.	.	.	NP_00102	XP_01686	protein_co	ENSG0000	972	1697
CD79A	0	0.310115	0.053699	0.400317	0.213577	0.135242	CD79a molecule [Source:HGNC Symbol;Acc:HGNC:1698]	chr19:4238	NM_00178	.	.	.	NP_00177	.	protein_co	ENSG0000	973	1698
CD79B	0.269746	0.211266	0.83486	0.399924	0.247274	0.525481	CD79b molecule [Source:HGNC Symbol;Acc:HGNC:1699]	chr17:6200	NM_00062	XM_00525	.	.	NP_00061	.	protein_co	ENSG0000	974	1699
CD80	0.044207	0.097031	0.13003	0.119319	0.097121	0.090098	CD80 molecule [Source:HGNC Symbol;Acc:HGNC:1700]	chr3:1192	NM_00519	.	.	.	NP_00518	XP_01686	protein_co	ENSG0000	941	1700
CD81	248.939	254.323	222.456	289.811	253.065	218.021	CD81 molecule [Source:HGNC Symbol;Acc:HGNC:1701]	chr11:2398	NM_00129	XM_01152	.	.	NP_00128	XP_01151	protein_co	ENSG0000	975	1701
CD82	7.59467	8.11234	11.235	7.84749	6.82461	5.52073	CD82 molecule [Source:HGNC Symbol;Acc:HGNC:6210]	chr11:4458	NM_00102	XM_00671	.	.	NP_00102	XP_00671	protein_co	ENSG0000	3732	6210
CD83	13.0341	13.5354	12.9206	7.08335	6.69049	7.68165	CD83 molecule [Source:HGNC Symbol;Acc:HGNC:1703]	chr6:1411	NM_00104	.	.	.	NP_00103	.	protein_co	ENSG0000	9308	1703
CD84	0	0	0	0	0	0	CD84 molecule [Source:HGNC Symbol;Acc:HGNC:1704]	chr1:1605	NM_00118	XM_01151	.	.	NP_00117	XP_01150	protein_co	ENSG0000	8832	1704
CD86	0	0	0	0	0	0	CD86 molecule [Source:HGNC Symbol;Acc:HGNC:1705]	chr3:1217	NM_00120	.	.	.	NP_00119	.	protein_co	ENSG0000	942	1705
CD8A	0.612931	0.69954	0.636985	1.36703	0.815004	0.83317	CD8a molecule [Source:HGNC Symbol;Acc:HGNC:1706]	chr2:8701	NM_00114	.	.	.	NP_00113	.	protein_co	ENSG0000	925	1706

CD8B	0	0	0	0	0	0	CD8b molecule [Source:HGNC Symbol;Acc:HGNC:1707]	chr2:87042	NM_00117	XM_01153	.	.	NP_00117	XP_011531	protein_co	ENSG0000	926	1707
CD9	185.246	188.073	162.512	215.666	181.616	187.42	CD9 molecule [Source:HGNC Symbol;Acc:HGNC:1709]	chr12:6308	NM_00176	XM_01152	.	.	NP_00176	XP_005253	protein_co	ENSG0000	928	1709
CD93	0	0	0	0	0	0	CD93 molecule [Source:HGNC Symbol;Acc:HGNC:15855]	chr20:2305	NM_01207	.	.	.	NP_03620	.	protein_co	ENSG0000	22918	15855
CD96	0.22353	0.203695	0.183103	0.252482	0.45488	0.342412	CD96 molecule [Source:HGNC Symbol;Acc:HGNC:16892]	chr3:11126	NM_00131	XM_00671	.	.	NP_00130	XP_006713	protein_co	ENSG0000	10225	16892
CD99	14.7181	16.364	14.4287	16.8048	12.5032	18.3536	CD99 molecule [Source:HGNC Symbol;Acc:HGNC:7082], mapped	chrX:26092	NM_00112	XM_00527	.	.	NP_00111	XP_005274	protein_co	ENSG0000	4267	7082
CD99L2	3.52072	3.28163	3.77018	3.55264	2.28737	2.68224	CD99 molecule like 2 [Source:HGNC Symbol;Acc:HGNC:18237]	chrX:14993	NM_00118	XM_00672	.	.	NP_00117	XP_011529	protein_co	ENSG0000	83692	18237
CDA	16.8142	17.3792	14.0527	19.0017	13.7521	15.0542	cytidine deaminase [Source:HGNC Symbol;Acc:HGNC:1712]	chr1:20915	NM_00178	.	.	.	NP_00177	.	protein_co	ENSG0000	978	1712
CDADC1	2.07105	2.07986	2.37141	1.80424	1.64192	1.83397	cytidine and dCMP deaminase domain containing 1 [Source:HGN	chr13:4982	NM_00119	XM_01153	.	.	NP_00118	XP_011533	protein_co	ENSG0000	81602	20299
CDAN1	4.78012	5.66846	4.5934	5.35995	5.72326	4.9782	codanin 1 [Source:HGNC Symbol;Acc:HGNC:1713]	chr15:4301	NM_13847	XM_01152	.	.	NP_61248	XP_005254	protein_co	ENSG0000	146059	1713
CDC123	57.0961	59.7175	56.748	69.8129	66.5964	59.6326	cell division cycle 123 [Source:HGNC Symbol;Acc:HGNC:16827]	chr10:1217	NM_00602	XM_00525	.	.	NP_00601	XP_005252	protein_co	ENSG0000	8872	16827
CDC14A	2.10834	2.87202	2.45915	1.8967	2.062	2.36455	cell division cycle 14A [Source:HGNC Symbol;Acc:HGNC:1718]	chr1:10081	NM_00131	XM_01154	.	.	NP_00130	XP_005271	protein_co	ENSG0000	8556	1718
CDC14B	9.81795	9.32379	11.6073	10.27	12.0276	13.9566	cell division cycle 14B [Source:HGNC Symbol;Acc:HGNC:1719]	chr9:99262	NM_00107	XM_01151	.	.	NP_00107	XP_005252	protein_co	ENSG0000	8555	1719
CDC16	36.5736	35.7328	34.5673	31.62	33.1118	31.444	cell division cycle 16 [Source:HGNC Symbol;Acc:HGNC:1720]	chr13:1150	NM_00107	XM_01702	.	.	NP_00107	XP_005266	protein_co	ENSG0000	8881	1720
CDC20	188.042	189.751	146.362	325.93	276.727	138.829	cell division cycle 20 [Source:HGNC Symbol;Acc:HGNC:1723]	chr1:43824	NM_00125	.	.	.	NP_00124	.	protein_co	ENSG0000	991	1723
CDC20B	0	0.057311	0.047422	0.069506	0	0.190685	cell division cycle 20B [Source:HGNC Symbol;Acc:HGNC:24222]	chr5:54408	NM_00114	.	.	.	NP_00113	.	protein_co	ENSG0000	166979	24222
CDC23	29.5904	31.6678	27.4599	25.0478	33.5647	22.7784	cell division cycle 23 [Source:HGNC Symbol;Acc:HGNC:1724]	chr5:13751	NM_00466	.	.	.	NP_00465	.	protein_co	ENSG0000	8697	1724
CDC25A	15.8094	15.6332	12.5479	18.4372	18.758	13.3175	cell division cycle 25A [Source:HGNC Symbol;Acc:HGNC:1725]	chr3:48198	NM_00178	XM_01153	.	.	NP_00178	XP_006713	protein_co	ENSG0000	993	1725
CDC25B	62.1684	64.2533	67.363	67.4176	66.6618	70.9799	cell division cycle 25B [Source:HGNC Symbol;Acc:HGNC:1726]	chr20:3767	NM_00128	.	.	.	NP_00127	.	protein_co	ENSG0000	994	1726
CDC25C	9.84939	8.90555	9.62264	8.95963	7.73844	8.44604	cell division cycle 25C [Source:HGNC Symbol;Acc:HGNC:1727]	chr5:13762	NM_00128	XM_01154	.	.	NP_00127	XP_005272	protein_co	ENSG0000	995	1727
CDC26	15.8644	16.4584	13.3499	16.1096	15.9413	13.5185	cell division cycle 26 [Source:HGNC Symbol;Acc:HGNC:17839]	chr9:11602	NM_13928	XM_01701	.	.	NP_64481	XP_016870	protein_co	ENSG0000	246184	17839
CDC27	36.6496	35.3631	38.8985	42.1537	53.7688	50.7432	cell division cycle 27 [Source:HGNC Symbol;Acc:HGNC:1728]	chr17:4519	NM_00111	XM_01702	.	.	NP_00110	XP_011523	protein_co	ENSG0000	996	1728
CDC34	60.2113	59.343	54.9849	77.2226	57.8213	54.4017	cell division cycle 34 [Source:HGNC Symbol;Acc:HGNC:1734]	chr19:5317	NM_00435	XM_00672	.	.	NP_00435	XP_006723	protein_co	ENSG0000	997	1734
CDC37	181	181.366	174.504	222.547	188.979	156.758	cell division cycle 37 [Source:HGNC Symbol;Acc:HGNC:1735]	chr19:1050	NM_00706	XM_01152	NR_03159	.	NP_00899	XP_011525	protein_co	ENSG0000	10030221	1735
CDC37L1	12.1326	11.5466	11.9763	13.751	14.087	12.4951	cell division cycle 37 like 1 [Source:HGNC Symbol;Acc:HGNC:1717	chr9:46795	NM_01791	.	.	.	NP_06038	.	protein_co	ENSG0000	55664	17179
CDC40	11.812	12.1912	10.4845	13.0629	12.5484	12.5279	cell division cycle 40 [Source:HGNC Symbol;Acc:HGNC:17350]	chr6:11050	NM_01589	XM_01153	.	.	NP_05697	.	protein_co	ENSG0000	51362	17350
CDC42	174.19	166.552	176.376	195.284	202.536	208.651	cell division cycle 42 [Source:HGNC Symbol;Acc:HGNC:1736]	chr1:22379	NM_00103	.	.	.	NP_00103	.	protein_co	ENSG0000	998	1736
CDC42BPA	9.74012	9.97651	12.3757	9.06071	12.1519	15.0119	CDC42 binding protein kinase alpha [Source:HGNC Symbol;Acc:H	chr1:22717	NM_00360	XM_01154	.	.	NP_00359	XP_005273	protein_co	ENSG0000	8476	1737
CDC42BPB	18.3228	19.5249	22.3766	18.9118	19.2714	21.1752	CDC42 binding protein kinase beta [Source:HGNC Symbol;Acc:HG	chr14:1033	NM_00603	XM_00526	.	.	NP_00602	XP_005268	protein_co	ENSG0000	9578	1738
CDC42BPG	9.11556	9.84308	10.2746	8.52092	7.19336	7.18527	CDC42 binding protein kinase gamma [Source:HGNC Symbol;Acc:	chr11:6459	NM_01752	XM_01154	.	.	NP_05999	XP_011543	protein_co	ENSG0000	55561	29829
CDC42EP1	63.0905	64.0176	56.8941	53.0616	43.8956	50.6296	CDC42 effector protein 1 [Source:HGNC Symbol;Acc:HGNC:17014	chr22:3795	NM_15224	XM_01702	.	.	NP_68944	XP_006724	protein_co	ENSG0000	11135	17014
CDC42EP2	17.7341	18.2029	19.2344	15.4935	12.9095	13.8165	CDC42 effector protein 2 [Source:HGNC Symbol;Acc:HGNC:16263	chr11:6508	NM_00677	XM_01701	.	.	NP_00677	XP_016872	protein_co	ENSG0000	10435	16263
CDC42EP3	15.3644	14.8356	14.1381	18.7562	20.0428	20.7439	CDC42 effector protein 3 [Source:HGNC Symbol;Acc:HGNC:16943	chr2:37869	NM_00127	XM_01153	.	.	NP_00125	XP_011530	protein_co	ENSG0000	10602	16943
CDC42EP4	25.6889	26.0989	26.7089	25.1151	25.683	24.4592	CDC42 effector protein 4 [Source:HGNC Symbol;Acc:HGNC:17147	chr17:7127	NM_01212	XM_00525	.	.	NP_03625	XP_005257	protein_co	ENSG0000	23580	17147
CDC42EP5	1.37732	0.183579	1.32614	1.45176	0	0.682495	CDC42 effector protein 5 [Source:HGNC Symbol;Acc:HGNC:17408	chr19:5497	NM_14505	.	.	.	NP_65949	.	protein_co	ENSG0000	148170	17408
CDC42SE1	37.2986	36.5787	32.4246	34.2294	35.8138	30.6157	CDC42 small effector 1 [Source:HGNC Symbol;Acc:HGNC:17719]	chr1:15102	NM_00103	XM_01700	.	.	NP_00103	XP_016857	protein_co	ENSG0000	56882	17719
CDC42SE2	20.5292	21.893	18.4076	16.8895	20.3773	24.1349	CDC42 small effector 2 [Source:HGNC Symbol;Acc:HGNC:18547]	chr5:13059	NM_00103	.	.	.	NP_00103	XP_016865	protein_co	ENSG0000	56990	18547
CDC45	16.5396	17.4743	13.8813	17.8356	15.0169	12.7836	cell division cycle 45 [Source:HGNC Symbol;Acc:HGNC:1739]	chr22:1946	NM_00117	XM_01153	.	.	NP_00117	XP_005261	protein_co	ENSG0000	8318	1739
CDC5L	14.3504	14.0139	12.5738	15.1675	14.657	14.507	cell division cycle 5 like [Source:HGNC Symbol;Acc:HGNC:1743]	chr6:44355	NM_00125	XM_00671	.	.	NP_00124	.	protein_co	ENSG0000	988	1743
CDC6	36.3455	37.0004	30.0539	44.8415	45.4292	35.5289	cell division cycle 6 [Source:HGNC Symbol;Acc:HGNC:1744]	chr17:3844	NM_00125	XM_01152	.	.	NP_00124	XP_011523	protein_co	ENSG0000	990	1744
CDC7	15.6357	13.1492	12.7921	13.957	16.59	14.5505	cell division cycle 7 [Source:HGNC Symbol;Acc:HGNC:1745]	chr1:91966	NM_00113	XM_01700	.	.	NP_00112	XP_005271	protein_co	ENSG0000	8317	1745
CDC73	10.8661	10.3481	9.99302	14.552	13.5366	15.811	cell division cycle 73 [Source:HGNC Symbol;Acc:HGNC:16783]	chr1:19309	NM_02452	.	.	.	NP_07880	.	protein_co	ENSG0000	79577	16783
CDCA2	13.7769	13.5635	14.3004	22.5173	25.769	21.4948	cell division cycle associated 2 [Source:HGNC Symbol;Acc:HGNC:1	chr8:25316	NM_00131	XM_01154	.	.	NP_00130	XP_006716	protein_co	ENSG0000	157313	14623
CDCA3	27.2711	23.7962	20.0988	42.628	36.129	24.868	cell division cycle associated 3 [Source:HGNC Symbol;Acc:HGNC:1	chr12:6949	NM_00129	XM_01152	.	.	NP_00128	XP_011519	protein_co	ENSG0000	83461	14624
CDCA4	51.0579	50.1497	43.6135	63.0936	56.3685	58.2795	cell division cycle associated 4 [Source:HGNC Symbol;Acc:HGNC:1	chr14:1054	NM_01795	.	.	.	NP_06042	.	protein_co	ENSG0000	55038	14625
CDCA5	61.0816	61.5735	50.5115	72.97	64.6524	46.7679	cell division cycle associated 5 [Source:HGNC Symbol;Acc:HGNC:1	chr11:6484	NM_08066	.	.	.	NP_54239	.	protein_co	ENSG0000	113130	14626
CDCA7	37.0859	37.6587	34.6169	45.2594	44.8981	36.9827	cell division cycle associated 7 [Source:HGNC Symbol;Acc:HGNC:1	chr2:17421	NM_03194	XM_01151	.	.	NP_11414	XP_011510	protein_co	ENSG0000	83879	14628
CDCA7L	42.4455	40.6994	40.6475	43.2261	40.5158	40.4327	cell division cycle associated 7 like [Source:HGNC Symbol;Acc:HG	chr7:21582	NM_00112	.	.	.	NP_00112	.	protein_co	ENSG0000	55536	30777
CDCA8	30.7461	30.6677	27.1878	48.9082	45.1805	34.3359	cell division cycle associated 8 [Source:HGNC Symbol;Acc:HGNC:1	chr1:38158	NM_00125	.	.	.	NP_00124	.	protein_co	ENSG0000	55143	14629
CDCP1	49.0487	48.9853	50.2205	53.509	57.8541	82.7247	CUB domain containing protein 1 [Source:HGNC Symbol;Acc:HGN	chr3:45123	NM_02284	XM_01153	.	.	NP_07375	XP_011532	protein_co	ENSG0000	64866	24357
CDCP2	0	0	0.04069	0.102092	0.054873	0.103928	CUB domain containing protein 2 [Source:HGNC Symbol;Acc:HGN	chr1:54598	NM_20154	.	.	.	NP_96384	.	protein_co	ENSG0000	200008	27297
CDH1	17.3021	18.5722	19.5652	16.1062	16.336	24.3975	cadherin 1 [Source:HGNC Symbol;Acc:HGNC:1748]	chr16:6877	NM_00131	XM_01152	.	.	NP_00130	XP_011521	protein_co	ENSG0000	999	1748
CDH10	0	0	0	0	0	0	cadherin 10 [Source:HGNC Symbol;Acc:HGNC:1749]	chr5:24487	NM_00672	XM_01700	.	.	NP_00130	XP_011512	protein_co	ENSG0000	1008	1749
CDH11	0	0	0	0	0	0	cadherin 11 [Source:HGNC Symbol;Acc:HGNC:1750]	chr16:6497	NM_00130	XM_01152	.	.	NP_00129	XP_005255	protein_co	ENSG0000	1009	1750
CDH12	0	0	0	0	0	0	cadherin 12 [Source:HGNC Symbol;Acc:HGNC:1751]	chr5:21750	NM_00406	XM_01151	.	.	NP_00130	XP_011512	protein_co	ENSG0000	1010	1751
CDH13	0	0	0	0	0	0	cadherin 13 [Source:HGNC Symbol;Acc:HGNC:1753]	chr16:8266	NM_00122	.	.	.	NP_00120	.	protein_co	ENSG0000	1012	1753
CDH15	1.564	1.37352	1.41846	1.80526	1.67252	1.39712	cadherin 15 [Source:HGNC Symbol;Acc:HGNC:1754]	chr16:8923	NM_00493	XM_01152	.	.	NP_00492	.	protein_co	ENSG0000	1013	1754
CDH16	0	0	0	0	0	0	cadherin 16 [Source:HGNC Symbol;Acc:HGNC:1755]	chr16:6694	NM_00120	XM_01152	.	.	NP_00119	XP_005255	protein_co	ENSG0000	1014	1755
CDH17	0.037156	0	0.061419	0	0	0.030599	cadherin 17 [Source:HGNC Symbol;Acc:HGNC:1756]	chr8:95139	NM_00114	XM_01151	.	.	NP_00113	XP_011515	protein_co	ENSG0000	1015	1756
CDH18	0	0	0	0	0	0	cadherin 18 [Source:HGNC Symbol;Acc:HGNC:1757]	chr5:19473	NM_00116	XM_01700	.	.	NP_00116	XP_005248	protein_co	ENSG0000	1016	1757

CDH19	0	0	0	0	0	0	cadherin 19 [Source:HGNC Symbol;Acc:HGNC:1758]	chr18:6416	NM_00127	XM_01152	.	.	NP_00125	XP_011524	protein_co	ENSG0000	28513	1758
CDH2	0	0	0	0	0	0	cadherin 2 [Source:HGNC Symbol;Acc:HGNC:1759]	chr18:2553	NM_00130	XM_01152	.	.	NP_00129	XP_005258	protein_co	ENSG0000	1000	1759
CDH20	0	0	0.019783	0	0	0	cadherin 20 [Source:HGNC Symbol;Acc:HGNC:1760]	chr18:5900	NM_03189	XM_00526	.	.	NP_11409	XP_005266	protein_co	ENSG0000	28316	1760
CDH22	0.964877	0.598752	1.26935	0.740405	0.963693	0.872386	cadherin 22 [Source:HGNC Symbol;Acc:HGNC:13251]	chr20:4480	NM_02124	XM_01152	.	.	NP_06707	XP_011527	protein_co	ENSG0000	64405	13251
CDH23	0.017239	0	0.041369	0	0	0.016921	cadherin related 23 [Source:HGNC Symbol;Acc:HGNC:13733]	chr10:7315	NM_00117	XM_01701	.	.	NP_00116	XP_006718	protein_co	ENSG0000	64072	13733
CDH24	8.81727	8.15514	10.3496	8.93376	8.79583	8.96547	cadherin 24 [Source:HGNC Symbol;Acc:HGNC:14265]	chr14:2351	NM_02247	XM_01153	.	.	NP_07192	.	protein_co	ENSG0000	64403	14265
CDH26	0.04334	0.089593	0	0	0.024519	0.022337	cadherin 26 [Source:HGNC Symbol;Acc:HGNC:15902]	chr20:5853	NM_02181	XM_01152	.	.	NP_06858	XP_006723	protein_co	ENSG0000	60437	15902
CDH3	47.2227	50.4777	53.8769	56.702	50.997	57.3541	cadherin 3 [Source:HGNC Symbol;Acc:HGNC:1762]	chr16:6867	NM_00131	XM_01152	.	.	NP_00130	XP_011521	protein_co	ENSG0000	1001	1762
CDH4	0.022881	0.021208	0	0	0	0	cadherin 4 [Source:HGNC Symbol;Acc:HGNC:1763]	chr20:5982	NM_00125	.	.	.	NP_00123	.	protein_co	ENSG0000	1002	1763
CDH5	0	0	0	0	0	0	cadherin 5 [Source:HGNC Symbol;Acc:HGNC:1764]	chr16:6640	NM_00179	XM_01152	.	.	NP_00178	XP_011521	protein_co	ENSG0000	1003	1764
CDH6	0	0	0	0.025923	0	0	cadherin 6 [Source:HGNC Symbol;Acc:HGNC:1765]	chr5:31193	NM_00493	XM_01700	.	.	NP_00492	XP_011512	protein_co	ENSG0000	1004	1765
CDH7	0	0	0	0	0	0	cadherin 7 [Source:HGNC Symbol;Acc:HGNC:1766]	chr18:6341	NM_00131	XM_01152	.	.	NP_00130	XP_016881	protein_co	ENSG0000	1005	1766
CDH8	0	0	0	0	0	0	cadherin 8 [Source:HGNC Symbol;Acc:HGNC:1767]	chr16:6168	NM_00179	XM_00525	.	.	NP_00178	XP_005255	protein_co	ENSG0000	1006	1767
CDH9	0	0	0	0	0	0	cadherin 9 [Source:HGNC Symbol;Acc:HGNC:1768]	chr5:26880	NM_01627	XM_01151	.	.	NP_05736	XP_011512	protein_co	ENSG0000	1007	1768
CDHR1	0	0	0	0.025885	0	0	cadherin related family member 1 [Source:HGNC Symbol;Acc:HG	chr10:8595	NM_00117	XM_01154	.	.	NP_00116	XP_011538	protein_co	ENSG0000	92211	14550
CDHR2	0.014493	0.049005	0.042622	0	0.067121	0	cadherin related family member 2 [Source:HGNC Symbol;Acc:HG	chr5:17596	NM_00117	XM_01153	.	.	NP_00116	.	protein_co	ENSG0000	54825	18231
CDHR3	0.214546	0.16051	0.107059	0.092868	0.085595	0.063809	cadherin related family member 3 [Source:HGNC Symbol;Acc:HG	chr7:10560	NM_00130	XM_01151	.	.	NP_00128	XP_005250	protein_co	ENSG0000	222256	26308
CDHR4	0.031002	0	0	0.100273	0.071342	0.091494	cadherin related family member 4 [Source:HGNC Symbol;Acc:HG	chr3:49828	NM_00100	XM_01153	.	.	NP_00100	XP_011532	protein_co	ENSG0000	389118	34527
CDHR5	0	0.021007	0	0	0	0	cadherin related family member 5 [Source:HGNC Symbol;Acc:HG	chr11:6165	NM_00117	XM_01152	.	.	NP_00116	XP_006718	protein_co	ENSG0000	53841	7521
CDIP1	4.246	5.01621	6.43838	5.77122	5.01979	4.36582	cell death inducing p53 target 1 [Source:HGNC Symbol;Acc:HGNC	chr16:4560	NM_00119	XM_01152	.	.	NP_00118	XP_011520	protein_co	ENSG0000	29965	13234
CDIPT	49.2575	47.0491	48.136	45.6256	38.9978	39.4976	CDP-diacylglycerol--inositol 3-phosphatidyltransferase [Source:H	chr16:2986	NM_00128	XM_00525	.	.	NP_00127	XP_005255	protein_co	ENSG0000	10423	1769
CDK1	93.6102	93.4511	93.6222	130.9	141.241	112.797	cyclin dependent kinase 1 [Source:HGNC Symbol;Acc:HGNC:1722	chr10:6253	NM_00132	XM_00671	.	.	NP_00130	XP_005270	protein_co	ENSG0000	983	1722
CDK10	34.4931	41.4867	42.5156	40.8734	34.5912	37.0951	cyclin dependent kinase 10 [Source:HGNC Symbol;Acc:HGNC:177	chr16:8974	NM_00109	XM_01152	.	.	NP_00109	XP_006721	protein_co	ENSG0000	8558	1770
CDK11A	7.5746	7.16729	7.84792	6.75822	7.0992	6.64555	cyclin dependent kinase 11A [Source:HGNC Symbol;Acc:HGNC:17	chr1:16341	NM_02401	.	.	.	NP_00130	XP_016858	protein_co	ENSG0000	728642	1730
CDK11B	9.69212	11.714	10.5807	12.5324	9.68869	8.84711	cyclin dependent kinase 11B [Source:HGNC Symbol;Acc:HGNC:17	chr1:15706	NM_00129	XM_01154	.	.	NP_00127	XP_006711	protein_co	ENSG0000	984	1729
CDK12	12.607	13.2365	12.6717	12.8856	13.9532	12.0377	cyclin dependent kinase 12 [Source:HGNC Symbol;Acc:HGNC:242	chr17:3761	NM_01508	XM_01152	.	.	NP_05589	XP_005257	protein_co	ENSG0000	51755	24224
CDK13	7.44797	8.18305	7.06192	6.61295	7.52168	8.16595	cyclin dependent kinase 13 [Source:HGNC Symbol;Acc:HGNC:173	chr7:39989	NM_00371	.	.	.	NP_00370	.	protein_co	ENSG0000	8621	1733
CDK14	2.62706	3.23604	3.36914	2.89442	3.72154	4.29506	cyclin dependent kinase 14 [Source:HGNC Symbol;Acc:HGNC:888	chr7:90225	NM_00128	XM_01701	.	.	NP_00127	XP_005250	protein_co	ENSG0000	5218	8883
CDK15	0	0	0	0	0	0	cyclin dependent kinase 15 [Source:HGNC Symbol;Acc:HGNC:144	chr2:20265	NM_00126	XM_01151	.	.	NP_00124	XP_005246	protein_co	ENSG0000	65061	14434
CDK16	38.1295	39.5194	34.7814	46.3379	39.2081	37.9596	cyclin dependent kinase 16 [Source:HGNC Symbol;Acc:HGNC:874	chrX:47077	NM_00117	XM_01154	.	.	NP_00116	XP_011542	protein_co	ENSG0000	5127	8749
CDK17	7.49651	7.2763	7.06473	7.0685	9.12239	9.95994	cyclin dependent kinase 17 [Source:HGNC Symbol;Acc:HGNC:875	chr12:9667	NM_00117	XM_01153	.	.	NP_00116	XP_006719	protein_co	ENSG0000	5128	8750
CDK18	7.95144	7.37054	10.9564	6.64372	6.37111	7.18184	cyclin dependent kinase 18 [Source:HGNC Symbol;Acc:HGNC:875	chr1:20547	NM_00259	XM_01150	.	.	NP_00258	XP_011507	protein_co	ENSG0000	5129	8751
CDK19	4.63515	5.23125	6.20998	3.84749	5.54959	7.78874	cyclin dependent kinase 19 [Source:HGNC Symbol;Acc:HGNC:193	chr6:11093	NM_00130	XM_01701	.	.	NP_00128	XP_005266	protein_co	ENSG0000	23097	19338
CDK2	73.3137	72.075	64.7141	80.8557	78.7016	71.875	cyclin dependent kinase 2 [Source:HGNC Symbol;Acc:HGNC:1771	chr12:5636	NM_00129	XM_01153	.	.	NP_00127	XP_011536	protein_co	ENSG0000	1017	1771
CDK20	2.88983	2.70966	3.06937	3.28912	3.09115	2.83262	cyclin dependent kinase 20 [Source:HGNC Symbol;Acc:HGNC:214	chr9:90581	NM_00103	XM_01701	.	.	NP_00103	XP_016870	protein_co	ENSG0000	23552	21420
CDK2AP1	95.6857	98.1953	81.5504	100.055	112.645	96.2258	cyclin dependent kinase 2 associated protein 1 [Source:HGNC Syr	chr12:1237	NM_00127	.	.	.	NP_00125	.	protein_co	ENSG0000	8099	14002
CDK2AP2	89.8357	84.7872	40.1306	91.8089	75.9564	55.1611	cyclin dependent kinase 2 associated protein 2 [Source:HGNC Syr	chr11:6727	NM_00585	.	.	.	NP_00584	.	protein_co	ENSG0000	10263	30833
CDK3	2.24296	0.076467	1.47264	1.0385	1.10323	0.13839	cyclin dependent kinase 3 [Source:HGNC Symbol;Acc:HGNC:1772	chr17:7393	.	.	.	.	.	.	protein_co	ENSG0000	.	1772
CDK4	154.804	146.324	124.135	182.218	148.864	142.43	cyclin dependent kinase 4 [Source:HGNC Symbol;Acc:HGNC:1773	chr12:5813	NM_00007	.	.	.	NP_00006	.	protein_co	ENSG0000	1019	1773
CDK5	13.7403	14.2684	17.0466	16.4583	12.9314	9.20068	cyclin dependent kinase 5 [Source:HGNC Symbol;Acc:HGNC:1774	chr7:15075	NM_00116	.	.	.	NP_00115	.	protein_co	ENSG0000	1020	1774
CDK5R1	4.0582	4.03492	4.08815	5.05091	4.57298	3.51635	cyclin dependent kinase 5 regulatory subunit 1 [Source:HGNC Syr	chr17:3081	NM_00388	XM_01152	.	.	NP_00387	XP_011523	protein_co	ENSG0000	8851	1775
CDK5R2	0.371693	0.510259	0.541244	0.394774	0.499053	0.658225	cyclin dependent kinase 5 regulatory subunit 2 [Source:HGNC Syr	chr2:21982	NM_00393	.	.	.	NP_00392	.	protein_co	ENSG0000	8941	1776
CDK5RAP1	16.9238	17.6008	18.3088	20.8944	18.0841	16.2857	CDK5 regulatory subunit associated protein 1 [Source:HGNC Syml	chr20:3194	NM_00127	XM_01152	.	.	NP_00126	XP_011527	protein_co	ENSG0000	51654	15880
CDK5RAP2	9.0749	10.0382	8.69781	11.1523	12.4608	11.7778	CDK5 regulatory subunit associated protein 2 [Source:HGNC Syml	chr9:12315	NM_00101	XM_01151	.	.	NP_00101	XP_006717	protein_co	ENSG0000	55755	18672
CDK5RAP3	61.87	55.8031	58.9834	61.1715	56.9077	43.0057	CDK5 regulatory subunit associated protein 3 [Source:HGNC Syml	chr17:4604	NM_00127	XM_01152	.	.	NP_00126	XP_011523	protein_co	ENSG0000	80279	18673
CDK6	18.7564	21.0515	16.2569	13.9366	16.434	14.873	cyclin dependent kinase 6 [Source:HGNC Symbol;Acc:HGNC:1777	chr7:92234	NM_00114	XM_01151	.	.	NP_00113	XP_006715	protein_co	ENSG0000	1021	1777
CDK7	42.2897	40.0219	35.6001	34.7442	33.6373	33.1093	cyclin dependent kinase 7 [Source:HGNC Symbol;Acc:HGNC:1778	chr5:68530	NM_00132	XM_01154	.	.	NP_00131	XP_011541	protein_co	ENSG0000	1022	1778
CDK8	11.5672	11.8994	11.3162	11.5402	12.6477	11.4208	cyclin dependent kinase 8 [Source:HGNC Symbol;Acc:HGNC:1779	chr13:2682	NM_00126	XM_01153	.	.	NP_00125	XP_005266	protein_co	ENSG0000	1024	1779
CDK9	27.4859	28.5176	32.6069	31.0032	28.8293	29.9929	cyclin dependent kinase 9 [Source:HGNC Symbol;Acc:HGNC:1780	chr9:13054	NM_00126	.	NR_03605	.	NP_00125	.	protein_co	ENSG0000	10061625	1780
CDKAL1	6.81605	6.04182	6.24672	6.83684	6.40845	7.57218	CDK5 regulatory subunit associated protein 1 like 1 [Source:HGN	chr6:20534	NM_01777	XM_01151	.	.	NP_06024	XP_011513	protein_co	ENSG0000	54901	21050
CDKL1	1.58228	1.59604	1.83165	1.70609	2.62487	1.51599	cyclin dependent kinase like 1 [Source:HGNC Symbol;Acc:HGNC:1	chr14:5070	NM_00128	XM_01153	.	.	NP_00126	XP_005268	protein_co	ENSG0000	8814	1781
CDKL2	0.595632	0.431242	0.530379	0.421212	0.575587	0.4312	cyclin dependent kinase like 2 [Source:HGNC Symbol;Acc:HGNC:1	chr4:76503	NM_00394	XM_00671	.	.	NP_00393	XP_005263	protein_co	ENSG0000	8999	1782
CDKL3	2.04616	2.26475	3.3009	2.7124	1.35846	2.2775	cyclin dependent kinase like 3 [Source:HGNC Symbol;Acc:HGNC:1	chr5:13363	NM_00111	XM_01154	.	.	NP_00110	XP_011541	protein_co	ENSG0000	51265	15483
CDKL4	0	0	0	0	0	0	cyclin dependent kinase like 4 [Source:HGNC Symbol;Acc:HGNC:1	chr2:39405	NM_00100	XM_01153	.	.	NP_00100	XP_011531	protein_co	ENSG0000	344387	19287
CDKL5	0.741236	0.63359	0.939541	0.685717	0.793559	1.3515	cyclin dependent kinase like 5 [Source:HGNC Symbol;Acc:HGNC:1	chrX:18443	NM_00103	XM_01154	.	.	NP_00103	.	protein_co	ENSG0000	6792	11411
CDKN1A	229.278	236.274	369.923	254.365	222.208	226.159	cyclin dependent kinase inhibitor 1A [Source:HGNC Symbol;Acc:H	chr6:36644	NM_00038	.	.	.	NP_00038	.	protein_co	ENSG0000	1026	1784
CDKN1B	19.5692	19.1148	13.912	16.0938	17.1776	17.851	cyclin dependent kinase inhibitor 1B [Source:HGNC Symbol;Acc:H	chr12:1287	NM_00406	.	.	.	NP_00405	.	protein_co	ENSG0000	1027	1785
CDKN1C	9.1537	10.034	11.3394	7.19123	5.4768	13.2731	cyclin dependent kinase inhibitor 1C [Source:HGNC Symbol;Acc:H	chr11:2904	NM_00007	XM_00525	.	.	NP_00006	XP_005252	protein_co	ENSG0000	1028	1786
CDKN2A	47.1148	54.3566	51.0809	87.5134	66.6161	88.261	cyclin dependent kinase inhibitor 2A [Source:HGNC Symbol;Acc:H	chr9:21967	NM_00007	XM_01151	.	.	NP_00006	XP_005251	protein_co	ENSG0000	1029	1787

CDKN2AIP	7.38711	7.71294	6.01094	8.31657	8.26813	6.16678	CDKN2A interacting protein [Source:HGNC Symbol;Acc:HGNC:243	chr4:18436	NM_01763	XM_00526	.	.	NP_001304	.	protein_co	ENSG0000	55602	24325
CDKN2AIPNL	44.4933	42.3609	46.7469	57.3162	55.2549	48.8061	CDKN2A interacting protein N-terminal like [Source:HGNC Symbo	chr5:13373	NM_08065	.	.	.	NP_54238	.	protein_co	ENSG0000	91368	30545
CDKN2B	1.11409	1.73117	2.62634	2.67113	1.65313	3.23696	cyclin dependent kinase inhibitor 2B [Source:HGNC Symbol;Acc:H	chr9:22002	NM_00493	.	.	.	NP_004927	.	protein_co	ENSG0000	1030	1788
CDKN2C	6.91374	6.03543	4.8351	13.205	8.98061	9.46514	cyclin dependent kinase inhibitor 2C [Source:HGNC Symbol;Acc:H	chr1:51426	NM_00126	.	.	.	NP_00125	.	protein_co	ENSG0000	1031	1789
CDKN2D	12.3903	10.3361	11.0527	13.9164	12.2467	17.7766	cyclin dependent kinase inhibitor 2D [Source:HGNC Symbol;Acc:H	chr19:1067	NM_00180	.	.	.	NP_00179	.	protein_co	ENSG0000	1032	1790
CDKN3	33.3544	31.5051	25.055	65.7634	71.4545	50.4095	cyclin dependent kinase inhibitor 3 [Source:HGNC Symbol;Acc:HG	chr14:5486	NM_00113	XM_01153	.	.	NP_00112	XP_011534	protein_co	ENSG0000	1033	1791
CDNF	0.568229	0.438119	0.674196	0.175308	0.230235	0.771277	cerebral dopamine neurotrophic factor [Source:HGNC Symbol;Ac	chr10:1486	NM_00102	XM_01151	.	.	NP_00102	XP_011517	protein_co	ENSG0000	441549	24913
CDO1	0.044305	0	0.038341	0	0	0	cysteine dioxygenase type 1 [Source:HGNC Symbol;Acc:HGNC:17	chr5:11514	NM_00180	.	.	.	NP_00131	.	protein_co	ENSG0000	1036	1795
CDON	3.01445	3.12153	2.949	3.06001	3.16158	2.75595	cell adhesion associated, oncogene regulated [Source:HGNC Sym	chr11:1258	NM_00124	XM_01154	.	.	NP_00123	XP_011541	protein_co	ENSG0000	50937	17104
CDPF1	9.41145	9.38132	7.07027	9.33335	8.68578	7.93172	cysteine rich DPF motif domain containing 1 [Source:HGNC Symb	chr22:4663	NM_20732	XM_01152	.	.	NP_99721	XP_011528	protein_co	ENSG0000	150383	33710
CDR1	0	0	0	0	0	0	cerebellar degeneration related protein 1 [Source:HGNC Symbol;	chrX:13986	NM_00406	.	.	.	NP_00405	.	protein_co	ENSG0000	1038	1798
CDR2	15.7495	15.7134	14.9205	14.8809	14.4611	14.4745	cerebellar degeneration related protein 2 [Source:HGNC Symbol;	chr16:2235	NM_00180	XM_00527	.	.	NP_00179	XP_005276	protein_co	ENSG0000	101060399	1799
CDR2L	15.8517	18.0483	17.0634	23.7357	21.7783	17.4762	cerebellar degeneration related protein 2 like [Source:HGNC Sym	chr17:7298	NM_01460	XM_00672	.	.	NP_05541	XP_006721	protein_co	ENSG0000	30850	29999
CDRT1	0.094043	0.487898	0.165118	0.104116	0	0.194956	CMT1A duplicated region transcript 1 [Source:HGNC Symbol;Acc:	chr17:1547	NM_00638	.	.	.	NP_00637	.	protein_co	ENSG0000	374286	14379
CDRT15	0	0.141814	0	0	0	0	CMT1A duplicated region transcript 15 [Source:HGNC Symbol;Acc	chr17:1413	NM_00100	.	.	.	NP_00100	.	protein_co	ENSG0000	146822	14395
CDRT15L2	0	0	0	0	0	0	CMT1A duplicated region transcript 15-like 2 [Source:HGNC Symb	chr17:2048	NM_00119	.	.	.	NP_00117	.	protein_co	ENSG0000	256223	34075
CDRT4	5.62522	4.73646	3.31164	4.26401	3.55092	4.76926	CMT1A duplicated region transcript 4 [Source:HGNC Symbol;Acc:	chr17:1533	NM_00120	.	.	.	NP_00119	.	protein_co	ENSG0000	284040	14383
CDS1	8.87383	8.13979	7.7435	8.4737	9.79814	14.2036	CDP-diacylglycerol synthase 1 [Source:HGNC Symbol;Acc:HGNC:1	chr4:85504	NM_00126	XM_00526	.	.	NP_00125	XP_005262	protein_co	ENSG0000	1040	1800
CDS2	9.77544	9.56278	11.8439	8.25236	8.00993	9.92971	CDP-diacylglycerol synthase 2 [Source:HGNC Symbol;Acc:HGNC:1	chr20:5107	NM_00381	XM_01152	.	.	NP_00380	XP_006723	protein_co	ENSG0000	8760	1801
CDSN	0.175174	0.177124	0.210644	0.152245	0.319581	0.113063	corneodesmosin [Source:HGNC Symbol;Acc:HGNC:1802]	chr6:31082	NM_00126	.	.	.	NP_00125	.	protein_co	ENSG0000	1041	1802
CDT1	52.3936	50.2865	37.2017	64.4735	57.6925	49.1537	chromatin licensing and DNA replication factor 1 [Source:HGNC S	chr16:8886	NM_03092	.	.	.	NP_11219	.	protein_co	ENSG0000	81620	24576
CDV3	96.5821	95.9104	86.4298	95.8084	112.838	93.6863	CDV3 homolog [Source:HGNC Symbol;Acc:HGNC:26928]	chr3:13329	NM_00113	XM_01700	.	.	NP_00112	XP_005247	protein_co	ENSG0000	55573	26928
CDX1	0	0.053594	0	0	0	0	caudal type homeobox 1 [Source:HGNC Symbol;Acc:HGNC:1805]	chr5:14954	NM_00180	.	.	.	NP_00179	.	protein_co	ENSG0000	1044	1805
CDX2	5.69372	6.30923	6.02675	6.62336	6.72721	5.0859	caudal type homeobox 2 [Source:HGNC Symbol;Acc:HGNC:1806]	chr13:2853	NM_00126	XM_01153	.	.	NP_00125	XP_011533	protein_co	ENSG0000	1045	1806
CDX4	0	0	0	0	0	0	caudal type homeobox 4 [Source:HGNC Symbol;Acc:HGNC:1808]	chrX:72667	NM_00519	.	.	.	NP_00518	.	protein_co	ENSG0000	1046	1808
CDY1	0	0	0	0	0	0	chromodomain Y-linked 1 [Source:HGNC Symbol;Acc:HGNC:1809	chrY:27768	NM_00468	.	.	.	NP_00467	.	protein_co	ENSG0000	9085	1809
CDY1B	0	0	0	0	0	0	chromodomain Y-linked 1B [Source:HGNC Symbol;Acc:HGNC:239	chrY:26191	NM_00100	.	.	.	NP_00100	.	protein_co	ENSG0000	253175	23920
CDY2A	0	0	0	0	0	0	chromodomain Y-linked 2A [Source:HGNC Symbol;Acc:HGNC:181	chrY:20137	NM_00482	.	.	.	NP_00481	.	protein_co	ENSG0000	9426	1810
CDY2B	0	0	0	0	0	0	chromodomain Y-linked 2B [Source:HGNC Symbol;Acc:HGNC:239	chrY:19989	NM_00100	.	.	.	NP_00100	.	protein_co	ENSG0000	203611	23921
CDYL	15.9424	16.8555	15.8881	16.9812	16.5339	17.1644	chromodomain Y-like [Source:HGNC Symbol;Acc:HGNC:1811]	chr6:47063	NM_00114	XM_01151	.	.	NP_00113	XP_011513	protein_co	ENSG0000	9425	1811
CDYL2	0.019672	0	0	0	0	0.009814	chromodomain Y-like 2 [Source:HGNC Symbol;Acc:HGNC:23030]	chr16:8063	NM_15234	XM_01152	.	.	NP_68955	XP_011521	protein_co	ENSG0000	124359	23030
CEACAM1	1.60463	1.32191	0.81889	1.13504	1.25205	1.2101	carcinoembryonic antigen related cell adhesion molecule 1 [Sour	chr19:4301	NM_00102	.	.	.	NP_00102	.	protein_co	ENSG0000	634	1814
CEACAM16	0	0	0	0.052154	0	0	carcinoembryonic antigen related cell adhesion molecule 16 [Sou	chr19:4520	NM_00103	XM_01152	.	.	NP_00103	.	protein_co	ENSG0000	388551	31948
CEACAM18	0	0	0	0	0	0	carcinoembryonic antigen related cell adhesion molecule 18 [Sou	chr19:5198	NM_00127	XM_01152	.	.	NP_00126	XP_011525	protein_co	ENSG0000	.	31949
CEACAM19	1.73551	1.51489	1.5995	2.14268	1.89011	2.20994	carcinoembryonic antigen related cell adhesion molecule 19 [Sou	chr19:4517	NM_00112	XM_00672	.	.	NP_00112	.	protein_co	ENSG0000	56971	31951
CEACAM20	0	0	0.032722	0	0.052686	0	carcinoembryonic antigen related cell adhesion molecule 20 [Sou	chr19:4500	NM_00110	XM_01152	.	.	NP_00109	XP_011524	protein_co	ENSG0000	125931	24879
CEACAM21	0	0.076933	0	0	0	0.158331	carcinoembryonic antigen related cell adhesion molecule 21 [Sou	chr19:4205	NM_00109	XM_01702	.	.	NP_00109	XP_005278	protein_co	ENSG0000	90273	28834
CEACAM3	0	0	0	0	0	0	carcinoembryonic antigen related cell adhesion molecule 3 [Sour	chr19:4230	NM_00127	.	.	.	NP_00126	.	protein_co	ENSG0000	1084	1815
CEACAM4	0	0	0	0	0	0	carcinoembryonic antigen related cell adhesion molecule 4 [Sour	chr19:4212	NM_00181	XM_01152	.	.	NP_00180	XP_006723	protein_co	ENSG0000	1089	1816
CEACAM5	0	0	0	0	0	0	carcinoembryonic antigen related cell adhesion molecule 5 [Sour	chr19:4221	NM_00129	XM_01702	.	.	NP_00127	XP_011524	protein_co	ENSG0000	1048	1817
CEACAM6	0	0	0	0.029021	0	0	carcinoembryonic antigen related cell adhesion molecule 6 [Sour	chr19:4221	NM_00248	XM_01152	.	.	NP_00247	XP_011525	protein_co	ENSG0000	4680	1818
CEACAM7	0	0	0	0	0	0	carcinoembryonic antigen related cell adhesion molecule 7 [Sour	chr19:4217	NM_00129	.	.	.	NP_00127	.	protein_co	ENSG0000	1087	1819
CEACAM8	0	0	0	0	0	0	carcinoembryonic antigen related cell adhesion molecule 8 [Sour	chr19:4308	NM_00181	.	.	.	NP_00180	XP_016881	protein_co	ENSG0000	1088	1820
CEBPA	7.71712	6.67396	6.98046	5.90734	6.73842	5.31382	CCAAT/enhancer binding protein alpha [Source:HGNC Symbol;Ac	chr19:3379	NM_00128	.	.	.	NP_00127	.	protein_co	ENSG0000	1050	1833
CEBPB	43.72	45.1891	42.3243	38.9352	36.0676	86.3338	CCAAT/enhancer binding protein beta [Source:HGNC Symbol;Acc	chr20:4880	NM_00128	.	.	.	NP_00127	.	protein_co	ENSG0000	1051	1834
CEBPD	4.23375	4.50876	5.74502	3.02263	3.0555	2.82248	CCAAT/enhancer binding protein delta [Source:HGNC Symbol;Acc	chr8:48649	NM_00519	.	.	.	NP_00518	.	protein_co	ENSG0000	1052	1835
CEBPE	0	0	0	0	0	0	CCAAT/enhancer binding protein epsilon [Source:HGNC Symbol;A	chr14:2358	NM_00180	.	.	.	NP_00179	.	protein_co	ENSG0000	1053	1836
CEBPG	28.8437	29.2785	19.0322	23.7208	26.1943	47.2552	CCAAT/enhancer binding protein gamma [Source:HGNC Symbol;A	chr19:3386	NM_00125	.	.	.	NP_00123	.	protein_co	ENSG0000	1054	1837
CEBPZ	24.0379	22.8154	23.1929	22.0631	26.699	20.6885	CCAAT/enhancer binding protein zeta [Source:HGNC Symbol;Acc:	chr2:37394	NM_00576	.	.	.	NP_00575	.	protein_co	ENSG0000	10153	24218
CEBPZOS	35.1987	32.4226	33.3366	33.0602	37.0631	29.1368	CEBPZ opposite strand [Source:HGNC Symbol;Acc:HGNC:49288]	chr2:37394	NM_00132	XM_01700	.	.	NP_00130	XP_016858	protein_co	ENSG0000	10050587	49288
CECR1	0	0	0	0	0.049373	0	cat eye syndrome chromosome region, candidate 1 [Source:HGN	chr22:1765	NM_00128	XM_01154	.	.	NP_00126	XP_011544	protein_co	ENSG0000	51816	1839
CECR2	1.35215	1.46513	2.02033	1.09562	1.25479	1.49976	CECR2, histone acetyl-lysine reader [Source:HGNC Symbol;Acc:HG	chr22:1784	NM_00129	XM_01702	.	.	NP_00127	XP_011544	protein_co	ENSG0000	27443	1840
CECR5	48.0586	48.7356	42.8947	58.2237	53.4069	39.6663	cat eye syndrome chromosome region, candidate 5 [Source:HGN	chr22:1761	NM_01782	XM_01154	.	.	NP_06029	XP_005261	protein_co	ENSG0000	27440	1843
CECR6	0.852546	0.483163	0.406162	0.54635	0.454622	0.247656	cat eye syndrome chromosome region, candidate 6 [Source:HGN	chr22:1759	NM_00116	XM_01154	.	.	NP_00115	XP_011544	protein_co	ENSG0000	27439	1844
CEL	0.675031	1.06437	1.1201	1.02573	0.930032	0.355343	carboxyl ester lipase [Source:HGNC Symbol;Acc:HGNC:1848]	chr9:13593	NM_00180	.	.	.	NP_00179	.	protein_co	ENSG0000	1056	1848
CELA1	0	0	0	0	0	0	chymotrypsin like elastase family member 1 [Source:HGNC Symb	chr12:5172	NM_00197	.	.	.	NP_00196	.	protein_co	ENSG0000	1990	3308
CELA2A	0	0	0	0	0	0	chymotrypsin like elastase family member 2A [Source:HGNC Sym	chr1:15783	NM_03344	.	.	.	NP_25427	.	protein_co	ENSG0000	63036	24609
CELA2B	0	0	0	0	0	0	chymotrypsin like elastase family member 2B [Source:HGNC Sym	chr1:15802	NM_01584	.	.	.	NP_05693	.	protein_co	ENSG0000	51032	29995
CELA3A	0.148586	0	0.087112	0	0	0	chymotrypsin like elastase family member 3A [Source:HGNC Sym	chr1:22328	NM_00574	.	.	.	NP_00573	.	protein_co	ENSG0000	10136	15944

CELA3B	0	0.101108	0.175331	0	0.118215	0	chymotrypsin like elastase family member 3B [Source:HGNC Syml	chr1:22303	NM_00735	.	.	.	NP_03137	.	protein_co	ENSG0000	23436	15945
CELF1	29.8351	33.737	33.8843	30.7325	33.7786	36.127	CUGBP, Elav-like family member 1 [Source:HGNC Symbol;Acc:HGI	chr11:4748	NM_00102	XM_01701	.	.	NP_00102	XP_011518	protein_co	ENSG0000	10658	2549
CELF2	0.017706	0.075372	0	0	0	0	CUGBP, Elav-like family member 2 [Source:HGNC Symbol;Acc:HGI	chr10:1084	NM_00102	XM_01701	.	.	NP_00102	XP_005252	protein_co	ENSG0000	10659	2550
CELF3	0.038093	0	0	0.040702	0	0	CUGBP, Elav-like family member 3 [Source:HGNC Symbol;Acc:HGI	chr1:15167	NM_00117	XM_01150	.	.	NP_00116	XP_006711	protein_co	ENSG0000	11189	11967
CELF4	0	0	0	0	0	0.02571	CUGBP, Elav-like family member 4 [Source:HGNC Symbol;Acc:HGI	chr18:3482	NM_00102	XM_01152	.	.	NP_00102	XP_005258	protein_co	ENSG0000	56853	14015
CELF5	0.988753	1.33244	1.48978	0.807108	0.762162	0.767639	CUGBP, Elav-like family member 5 [Source:HGNC Symbol;Acc:HGI	chr19:3224	NM_00117	XM_01152	.	.	NP_00116	XP_006722	protein_co	ENSG0000	60680	14058
CELF6	0.952992	0.914176	0.901651	1.30971	1.28934	1.13347	CUGBP, Elav-like family member 6 [Source:HGNC Symbol;Acc:HGI	chr15:7257	NM_00117	.	.	.	NP_00116	.	protein_co	ENSG0000	60677	14059
CELSR1	5.14177	5.84501	6.2953	5.50002	5.7949	6.4354	cadherin EGF LAG seven-pass G-type receptor 1 [Source:HGNC Sy	chr22:4675	NM_01424	XM_01153	.	.	NP_05506	XP_006724	protein_co	ENSG0000	9620	1850
CELSR2	4.53619	4.58574	5.79561	5.89855	6.15129	5.28317	cadherin EGF LAG seven-pass G-type receptor 2 [Source:HGNC Sy	chr1:10979	NM_00140	XM_00527	.	.	NP_00139	XP_00527	protein_co	ENSG0000	1952	3231
CELSR3	2.10313	1.92417	2.09334	1.16074	1.27517	1.62475	cadherin EGF LAG seven-pass G-type receptor 3 [Source:HGNC Sy	chr3:48673	NM_00140	.	NR_03995	.	NP_00139	.	protein_co	ENSG0000	10061611	3230
CEMIP	2.26376	2.64281	4.03908	7.36148	8.00867	6.54959	cell migration inducing hyaluronan binding protein [Source:HGNC	chr15:8107	NM_00129	.	.	.	NP_00128	.	protein_co	ENSG0000	57214	29213
CEMP1	0.055216	0	0.047874	0.176371	0	0	cementum protein 1 [Source:HGNC Symbol;Acc:HGNC:32553]	chr16:2525	NM_00104	.	.	.	NP_00104	.	protein_co	ENSG0000	752014	32553
CEND1	0.265341	0.204967	0.227851	0.278618	0.125251	0	cell cycle exit and neuronal differentiation 1 [Source:HGNC Symb	chr11:7871	NM_01656	.	.	.	NP_05764	.	protein_co	ENSG0000	51286	24153
CENPA	17.1731	16.9969	15.5716	30.0865	27.4674	27.9508	centromere protein A [Source:HGNC Symbol;Acc:HGNC:1851]	chr2:27008	NM_00104	.	.	.	NP_00103	.	protein_co	ENSG0000	1058	1851
CENPB	32.2093	32.6205	30.6991	32.2985	27.8774	23.6458	centromere protein B [Source:HGNC Symbol;Acc:HGNC:1852]	chr20:3764	NM_00181	.	.	.	NP_00180	.	protein_co	ENSG0000	1059	1852
CENPBD1	4.925	5.2193	4.08217	4.21238	4.85115	3.21261	CENPB DNA-binding domain containing 1 [Source:HGNC Symbol;A	chr16:9003	NM_14503	.	.	.	NP_65947	.	protein_co	ENSG0000	92806	28272
CENPC	3.4824	3.45783	3.34836	3.29431	4.49586	4.55936	centromere protein C [Source:HGNC Symbol;Acc:HGNC:1854]	chr4:68334	NM_00181	XM_01153	.	.	NP_00180	XP_006714	protein_co	ENSG0000	1060	1854
CENPE	4.08476	4.03119	4.65075	4.72667	8.25704	7.7427	centromere protein E [Source:HGNC Symbol;Acc:HGNC:1856]	chr4:10402	NM_00128	XM_01153	.	.	NP_00127	XP_011529	protein_co	ENSG0000	1062	1856
CENPF	25.9628	25.8487	24.5959	38.3376	46.6722	36.7311	centromere protein F [Source:HGNC Symbol;Acc:HGNC:1857]	chr1:21477	NM_01634	XM_01700	.	.	NP_05742	XP_011507	protein_co	ENSG0000	1063	1857
CENPH	10.3212	10.6018	8.83167	10.3895	10.9005	11.832	centromere protein H [Source:HGNC Symbol;Acc:HGNC:17268]	chr5:68485	NM_02290	.	.	.	NP_07506	.	protein_co	ENSG0000	64946	17268
CENPI	4.82845	3.05381	4.23541	5.12128	7.54266	5.14559	centromere protein I [Source:HGNC Symbol;Acc:HGNC:3968]	chrX:10035	NM_00673	XM_01153	.	.	NP_00130	XP_005262	protein_co	ENSG0000	2491	3968
CENPJ	6.83558	6.91166	6.15371	6.50106	7.6713	6.8	centromere protein J [Source:HGNC Symbol;Acc:HGNC:17272]	chr13:2545	NM_01845	XM_01153	.	.	NP_06092	XP_011533	protein_co	ENSG0000	55835	17272
CENPK	9.13329	7.13406	5.66242	7.29969	8.55417	6.45698	centromere protein K [Source:HGNC Symbol;Acc:HGNC:29479]	chr5:64813	NM_00126	XM_01700	.	.	NP_00125	XP_011541	protein_co	ENSG0000	64105	29479
CENPL	7.49842	5.85043	5.59503	6.34701	7.77543	6.49957	centromere protein L [Source:HGNC Symbol;Acc:HGNC:17879]	chr1:17376	NM_00112	.	.	.	NP_00112	.	protein_co	ENSG0000	91687	17879
CENPM	29.968	30.2327	21.8748	33.7237	28.4809	16.0139	centromere protein M [Source:HGNC Symbol;Acc:HGNC:18352]	chr22:4233	NM_00100	.	.	.	NP_00100	.	protein_co	ENSG0000	79019	18352
CENPN	36.2715	34.4625	29.7611	42.4191	42.7973	40.9773	centromere protein N [Source:HGNC Symbol;Acc:HGNC:30873]	chr16:8100	NM_00110	XM_00672	.	.	NP_00109	XP_006721	protein_co	ENSG0000	55839	30873
CENPO	14.2014	14.7276	10.6139	17.3099	14.6772	9.07807	centromere protein O [Source:HGNC Symbol;Acc:HGNC:28152]	chr2:25012	NM_00119	XM_00671	.	.	NP_00118	XP_006712	protein_co	ENSG0000	79172	28152
CENPP	1.94731	2.36087	2.39043	1.44761	3.36977	3.21037	centromere protein P [Source:HGNC Symbol;Acc:HGNC:32933]	chr9:95059	NM_00101	XM_01151	.	.	NP_00101	XP_011516	protein_co	ENSG0000	401541	32933
CENPQ	7.72349	7.22559	6.21562	8.21024	9.72454	10.3156	centromere protein Q [Source:HGNC Symbol;Acc:HGNC:21347]	chr6:49431	NM_01813	XM_01151	.	.	NP_06060	XP_005249	protein_co	ENSG0000	55166	21347
CENPT	15.1455	15.123	17.5838	17.6495	15.2407	10.9507	centromere protein T [Source:HGNC Symbol;Acc:HGNC:25787]	chr16:6786	NM_02508	XM_01152	.	.	NP_07935	XP_006721	protein_co	ENSG0000	80152	25787
CENPU	13.0913	12.5658	11.708	13.392	16.6231	17.3587	centromere protein U [Source:HGNC Symbol;Acc:HGNC:21348]	chr4:18557	NM_02462	XM_00526	.	.	NP_07890	XP_005263	protein_co	ENSG0000	79682	21348
CENPV	29.1958	24.1791	30.2156	40.2004	33.0186	34.5411	centromere protein V [Source:HGNC Symbol;Acc:HGNC:29920]	chr17:1612	NM_18171	.	.	.	NP_85906	.	protein_co	ENSG0000	201161	29920
CENPVP1	0	0	0	0	0	0	centromere protein V like 1 [Source:HGNC Symbol;Acc:HGNC:318	chrX:51453	.	.	.	.	.	.	protein_co	ENSG0000	.	31851
CENPVP3	0	0	0	0	0	0	centromere protein V like 3 [Source:HGNC Symbol;Acc:HGNC:438	chrX:51360	.	.	.	.	.	.	protein_co	ENSG0000	.	43880
CENPW	38.3226	36.3784	25.7504	57.3963	47.8296	41.2036	centromere protein W [Source:HGNC Symbol;Acc:HGNC:21488]	chr6:12666	NM_00101	.	.	.	NP_00101	XP_016866	protein_co	ENSG0000	387103	21488
CEP104	6.66634	8.09751	7.48539	6.13761	6.09045	5.89685	centrosomal protein 104 [Source:HGNC Symbol;Acc:HGNC:24866	chr1:37286	NM_01470	XM_01154	.	.	NP_05551	XP_005244	protein_co	ENSG0000	9731	24866
CEP112	1.80661	2.06066	2.7751	1.80341	2.22925	2.70388	centrosomal protein 112 [Source:HGNC Symbol;Acc:HGNC:28514	chr17:6363	NM_00103	XM_01152	.	.	NP_00103	XP_005257	protein_co	ENSG0000	201134	28514
CEP120	7.3711	7.56884	7.10818	6.52675	7.39567	12.2978	centrosomal protein 120 [Source:HGNC Symbol;Acc:HGNC:26690	chr5:12268	NM_00116	XM_01154	.	.	NP_00115	XP_005271	protein_co	ENSG0000	153241	26690
CEP126	0.826702	0.710084	0.910713	0.792992	0.982287	0.823871	centrosomal protein 126 [Source:HGNC Symbol;Acc:HGNC:29264	chr11:1017	NM_02080	.	.	.	NP_06585	XP_005271	protein_co	ENSG0000	57562	29264
CEP128	1.9107	2.23336	1.50064	2.25029	2.19235	2.33132	centrosomal protein 128 [Source:HGNC Symbol;Acc:HGNC:20359	chr14:8096	NM_15244	XM_01153	.	.	NP_68965	XP_006720	protein_co	ENSG0000	145508	20359
CEP131	8.92289	8.70722	9.63071	9.61169	9.24349	7.50638	centrosomal protein 131 [Source:HGNC Symbol;Acc:HGNC:29511	chr17:7916	NM_00100	XM_01702	.	.	NP_00100	XP_011522	protein_co	ENSG0000	22994	29511
CEP135	2.97988	3.13239	2.4643	2.25102	2.9791	3.38416	centrosomal protein 135 [Source:HGNC Symbol;Acc:HGNC:29086	chr4:56815	NM_02500	XM_00671	.	.	NP_07928	XP_005265	protein_co	ENSG0000	9662	29086
CEP152	5.35605	5.4946	4.95658	4.38079	4.66089	4.81704	centrosomal protein 152 [Source:HGNC Symbol;Acc:HGNC:29298	chr15:4903	NM_00119	XM_01152	.	.	NP_00118	XP_006720	protein_co	ENSG0000	22995	29298
CEP162	1.44244	1.28568	1.59158	0.997747	1.98028	1.10618	centrosomal protein 162 [Source:HGNC Symbol;Acc:HGNC:21107	chr6:84833	NM_00128	XM_01701	.	.	NP_00127	XP_005248	protein_co	ENSG0000	22832	21107
CEP164	3.77776	3.32113	3.35895	3.05221	3.09254	2.77805	centrosomal protein 164 [Source:HGNC Symbol;Acc:HGNC:29182	chr11:1171	NM_00127	XM_01154	.	.	NP_00125	XP_005271	protein_co	ENSG0000	22897	29182
CEP170	8.72638	9.21708	11.2625	10.4942	9.82063	13.0398	centrosomal protein 170 [Source:HGNC Symbol;Acc:HGNC:28920	chr1:24328	NM_00104	XM_01700	.	.	NP_00103	XP_006711	protein_co	ENSG0000	645455	28920
CEP170B	21.8916	23.8964	21.7621	21.2685	21.218	16.3335	centrosomal protein 170B [Source:HGNC Symbol;Acc:HGNC:2036	chr14:1053	NM_00111	XM_01153	.	.	NP_00110	XP_005267	protein_co	ENSG0000	283638	20362
CEP19	2.8618	2.62775	4.09	2.69709	2.71438	3.01623	centrosomal protein 19 [Source:HGNC Symbol;Acc:HGNC:28209]	chr3:19643	NM_03289	XM_01151	.	.	NP_11628	XP_005269	protein_co	ENSG0000	84984	28209
CEP192	9.04394	8.69701	8.20957	8.87452	11.2032	9.22455	centrosomal protein 192 [Source:HGNC Symbol;Acc:HGNC:25515	chr18:1299	NM_03214	XM_01152	.	.	NP_11551	XP_005258	protein_co	ENSG0000	55125	25515
CEP250	3.68568	3.69114	3.86904	2.94651	3.11276	2.51731	centrosomal protein 250 [Source:HGNC Symbol;Acc:HGNC:1859]	chr20:3404	NM_00718	XM_01152	.	.	NP_00911	XP_005260	protein_co	ENSG0000	11190	1859
CEP290	2.93395	2.84374	3.23297	1.38093	2.04728	2.07948	centrosomal protein 290 [Source:HGNC Symbol;Acc:HGNC:29021	chr12:8842	NM_02511	XM_01701	.	.	NP_07939	XP_011537	protein_co	ENSG0000	80184	29021
CEP295	5.72964	5.07313	6.03449	4.2074	6.63271	6.77835	centrosomal protein 295 [Source:HGNC Symbol;Acc:HGNC:29366	chr11:9339	NM_03339	XM_01701	.	.	NP_20375	XP_005274	protein_co	ENSG0000	85459	29366
CEP295NL	0	0.060418	0.052834	0.032717	0.031962	0.097346	CEP295 N-terminal like [Source:HGNC Symbol;Acc:HGNC:44659]	chr17:7684	NM_00124	XM_01152	.	.	NP_00123	XP_011522	protein_co	ENSG0000	10065351	44659
CEP350	4.27965	4.56676	4.44653	3.87121	4.92767	5.44959	centrosomal protein 350 [Source:HGNC Symbol;Acc:HGNC:24238	chr1:17992	NM_01481	XM_01151	.	.	NP_05562	XP_011508	protein_co	ENSG0000	9857	24238
CEP41	3.27871	3.81165	3.01666	2.6378	3.5386	3.09451	centrosomal protein 41 [Source:HGNC Symbol;Acc:HGNC:12370]	chr7:13003	NM_00125	XM_01151	.	.	NP_00124	XP_011515	protein_co	ENSG0000	95681	12370
CEP44	3.72262	3.48066	3.61947	2.7503	3.58672	4.40335	centrosomal protein 44 [Source:HGNC Symbol;Acc:HGNC:29356]	chr4:17515	NM_00104	XM_01700	.	.	NP_00103	XP_011530	protein_co	ENSG0000	80817	29356
CEP55	34.2253	33.9766	32.22	46.823	51.0035	39.0405	centrosomal protein 55 [Source:HGNC Symbol;Acc:HGNC:1161]	chr10:9525	NM_00112	XM_01153	.	.	NP_00112	XP_011538	protein_co	ENSG0000	55165	1161
CEP57	13.7861	12.3254	12.9825	10.7914	14.4564	17.1924	centrosomal protein 57 [Source:HGNC Symbol;Acc:HGNC:30794]	chr11:9552	NM_00124	XM_00671	.	.	NP_00123	XP_006719	protein_co	ENSG0000	9702	30794
CEP57L1	4.44048	5.11665	6.00055	5.21539	7.80109	7.95131	centrosomal protein 57 like 1 [Source:HGNC Symbol;Acc:HGNC:2	chr6:10941	NM_00108	XM_01153	.	.	NP_00107	XP_005266	protein_co	ENSG0000	285753	21561

CEP63	6.4495	6.28071	5.97681	5.39552	5.6792	5.62752	centrosomal protein 63 [Source:HGNC Symbol;Acc:HGNC:25815]	chr3:13419	NM_00104	XM_01700	.	.	NP_00103	XP_005247	protein_co	ENSG0000	80254	25815
CEP68	5.85631	6.02276	7.99673	7.21572	7.37632	8.02154	centrosomal protein 68 [Source:HGNC Symbol;Acc:HGNC:29076]	chr2:65283	NM_00131	XM_00526	.	.	NP_00130	XP_005264	protein_co	ENSG0000	23177	29076
CEP70	11.2794	10.3233	12.8416	11.3276	16.891	16.6804	centrosomal protein 70 [Source:HGNC Symbol;Acc:HGNC:29972]	chr3:13821	NM_00128	XM_01151	.	.	NP_00127	XP_005247	protein_co	ENSG0000	80321	29972
CEP72	5.32924	5.49316	5.08547	7.91119	7.15786	5.69635	centrosomal protein 72 [Source:HGNC Symbol;Acc:HGNC:25547]	chr5:61238	NM_01814	XM_01151	.	.	NP_06061	XP_005248	protein_co	ENSG0000	55722	25547
CEP76	6.34744	6.35901	5.52571	6.95916	6.89882	7.15958	centrosomal protein 76 [Source:HGNC Symbol;Acc:HGNC:25727]	chr18:1265	NM_00127	XM_00525	.	.	NP_00125	XP_005258	protein_co	ENSG0000	79959	25727
CEP78	16.9015	17.2596	15.679	18.7172	19.372	14.9647	centrosomal protein 78 [Source:HGNC Symbol;Acc:HGNC:25740]	chr9:80850	NM_00109	XM_01701	.	.	NP_00109	XP_005252	protein_co	ENSG0000	84131	25740
CEP83	5.39062	4.8764	5.79977	5.07773	5.3728	5.50512	centrosomal protein 83 [Source:HGNC Symbol;Acc:HGNC:17966]	chr12:9470	NM_00104	XM_01153	.	.	NP_00103	XP_006719	protein_co	ENSG0000	51134	17966
CEP85	15.7558	16.3487	15.8397	19.7253	20.3686	19.7743	centrosomal protein 85 [Source:HGNC Symbol;Acc:HGNC:25309]	chr1:26560	NM_00128	XM_00524	.	.	NP_00126	.	protein_co	ENSG0000	64793	25309
CEP85L	1.6742	1.51071	1.38586	1.38068	1.21627	1.74399	centrosomal protein 85 like [Source:HGNC Symbol;Acc:HGNC:216	chr6:11878	NM_00104	XM_01701	.	.	NP_00103	XP_005267	protein_co	ENSG0000	387119	21638
CEP89	6.51726	6.54256	6.08306	5.4814	5.01151	4.46529	centrosomal protein 89 [Source:HGNC Symbol;Acc:HGNC:25907]	chr19:3336	NM_03281	XM_00525	.	.	NP_11620	XP_005259	protein_co	ENSG0000	84902	25907
CEP95	24.1838	26.2734	23.057	22.595	22.9495	22.7366	centrosomal protein 95 [Source:HGNC Symbol;Acc:HGNC:25141]	chr17:6250	NM_13836	XM_00672	.	.	NP_61237	XP_005257	protein_co	ENSG0000	90799	25141
CEP97	3.94601	4.44429	4.19334	4.65663	5.72877	7.21663	centrosomal protein 97 [Source:HGNC Symbol;Acc:HGNC:26244]	chr3:10144	NM_00130	XM_01151	.	.	NP_00129	XP_006713	protein_co	ENSG0000	79598	26244
CEPT1	15.1197	16.8595	17.6567	16.4343	18.0233	18.7872	choline/ethanolamine phosphotransferase 1 [Source:HGNC Symb	chr1:11165	NM_00100	XM_01154	.	.	NP_00100	XP_006710	protein_co	ENSG0000	10390	24289
CER1	0	0	0	0	0	0	cerberus 1, DAN family BMP antagonist [Source:HGNC Symbol;Ac	chr9:14719	NM_00545	.	.	.	NP_00544	.	protein_co	ENSG0000	9350	1862
CERCAM	15.632	14.3121	16.5955	13.1888	15.0541	11.3531	cerebral endothelial cell adhesion molecule [Source:HGNC Symbo	chr9:13117	NM_00128	XM_01151	.	.	NP_00127	XP_005252	protein_co	ENSG0000	51148	23723
CERK	29.495	31.768	28.543	30.0705	28.7844	23.3698	ceramide kinase [Source:HGNC Symbol;Acc:HGNC:19256]	chr22:4708	NM_02276	XM_01702	.	.	NP_07360	XP_016884	protein_co	ENSG0000	64781	19256
CERKL	0.205671	0.285997	0.185264	0.220281	0.270313	0.415534	ceramide kinase like [Source:HGNC Symbol;Acc:HGNC:21699]	chr2:18240	NM_00103	.	.	.	NP_00102	.	protein_co	ENSG0000	375298	21699
CERS1	0.210754	0.091756	0.292465	0.213886	0.049424	0.18982	ceramide synthase 1 [Source:HGNC Symbol;Acc:HGNC:14253]	chr19:1897	NM_00129	.	.	.	NP_00127	.	protein_co	ENSG0000	10715	14253
CERS2	117.449	109.389	100.639	118.005	114.392	111.481	ceramide synthase 2 [Source:HGNC Symbol;Acc:HGNC:14076]	chr1:15089	NM_02207	XM_01150	.	.	NP_07135	XP_005245	protein_co	ENSG0000	29956	14076
CERS3	0	0	0	0.023165	0.049232	0.045363	ceramide synthase 3 [Source:HGNC Symbol;Acc:HGNC:23752]	chr15:1009	NM_00129	XM_01702	.	.	NP_00127	XP_011519	protein_co	ENSG0000	204219	23752
CERS4	17.6316	16.278	16.2391	17.4216	14.6409	16.9759	ceramide synthase 4 [Source:HGNC Symbol;Acc:HGNC:23747]	chr19:8274	NM_02455	XM_01152	.	.	NP_07882	XP_006722	protein_co	ENSG0000	79603	23747
CERS5	22.2195	20.3431	22.0056	20.7792	18.4175	21.0525	ceramide synthase 5 [Source:HGNC Symbol;Acc:HGNC:23749]	chr12:5052	NM_00128	XM_01153	.	.	NP_00126	XP_005269	protein_co	ENSG0000	91012	23749
CERS6	19.0573	18.6423	19.8484	17.5107	21.8886	23.5807	ceramide synthase 6 [Source:HGNC Symbol;Acc:HGNC:23826]	chr2:16931	NM_00125	.	.	.	NP_00124	.	protein_co	ENSG0000	253782	23826
CES1	0	0	0.034838	0	0	0	carboxylesterase 1 [Source:HGNC Symbol;Acc:HGNC:1863]	chr16:5558	NM_00102	XM_00525	.	.	NP_00102	XP_005255	protein_co	ENSG0000	1066	1863
CES2	12.9128	13.7615	15.0034	12.2755	12.3788	10.6238	carboxylesterase 2 [Source:HGNC Symbol;Acc:HGNC:1864]	chr16:6696	NM_00386	XM_01152	.	.	NP_00386	XP_011521	protein_co	ENSG0000	8824	1864
CES3	1.95325	2.5055	2.85771	1.55256	1.39159	1.6987	carboxylesterase 3 [Source:HGNC Symbol;Acc:HGNC:1865]	chr16:6699	NM_00118	XM_01152	.	.	NP_00117	XP_011521	protein_co	ENSG0000	23491	1865
CES4A	0.044741	0	0.060189	0	0	0.095457	carboxylesterase 4A [Source:HGNC Symbol;Acc:HGNC:26741]	chr16:6702	NM_00119	XM_01152	.	.	NP_00117	XP_005255	protein_co	ENSG0000	283848	26741
CES5A	0	0	0	0	0	0	carboxylesterase 5A [Source:HGNC Symbol;Acc:HGNC:26459]	chr16:5558	NM_00114	.	.	.	.	.	protein_co	ENSG0000	221223	26459
CETN1	0	0	0	0	0	0	centrin 1 [Source:HGNC Symbol;Acc:HGNC:1866]	chr18:5803	NM_00406	.	.	.	NP_00405	.	protein_co	ENSG0000	1068	1866
CETN2	29.8325	23.1562	27.3628	25.1684	26.7969	25.3194	centrin 2 [Source:HGNC Symbol;Acc:HGNC:1867]	chrX:15199	NM_00434	.	.	.	NP_00433	.	protein_co	ENSG0000	1069	1867
CETN3	26.5897	23.698	25.0873	31.0193	38.4886	37.3072	centrin 3 [Source:HGNC Symbol;Acc:HGNC:1868]	chr5:89688	NM_00129	.	.	.	NP_00128	.	protein_co	ENSG0000	1070	1868
CETP	0	0	0	0	0	0	cholesteryl ester transfer protein [Source:HGNC Symbol;Acc:HGN	chr16:5699	NM_00007	.	.	.	NP_00006	.	protein_co	ENSG0000	1071	1869
CFAP100	0	0	0.024956	0	0.032839	0.031106	cilia and flagella associated protein 100 [Source:HGNC Symbol;Ac	chr3:12611	NM_18262	XM_00671	.	.	NP_87243	XP_005247	protein_co	ENSG0000	348807	26842
CFAP126	0	0	0	0.112461	0	0.106396	cilia and flagella associated protein 126 [Source:HGNC Symbol;Ac	chr1:16128	NM_00101	.	.	.	NP_00101	.	protein_co	ENSG0000	257177	32325
CFAP157	0	0	0.02651	0.033985	0	0.035193	cilia and flagella associated protein 157 [Source:HGNC Symbol;Ac	chr9:13046	NM_00101	XM_00671	.	.	NP_00101	XP_006717	protein_co	ENSG0000	286207	27843
CFAP161	0.443313	0.232774	0.076968	0.44741	0.248209	0.089511	cilia and flagella associated protein 161 [Source:HGNC Symbol;Ac	chr15:8142	NM_17352	XM_00672	.	.	NP_77579	XP_006720	protein_co	ENSG0000	161502	26782
CFAP20	52.4826	50.0256	41.5833	59.6371	48.2488	48.2365	cilia and flagella associated protein 20 [Source:HGNC Symbol;Acc	chr16:5814	NM_01324	.	.	.	NP_03737	.	protein_co	ENSG0000	29105	29523
CFAP206	0.253447	0.101411	0.137975	0.359681	0.105504	0.468007	cilia and flagella associated protein 206 [Source:HGNC Symbol;Ac	chr6:88117	NM_00103	.	.	.	NP_00102	.	protein_co	ENSG0000	154313	21405
CFAP221	0.146015	0.077127	0.084473	0.110906	0.108666	0.14559	cilia and flagella associated protein 221 [Source:HGNC Symbol;Ac	chr2:12030	NM_00127	XM_01151	.	.	NP_00125	XP_006712	protein_co	ENSG0000	200373	33720
CFAP36	24.4194	22.454	24.6888	33.313	28.9195	24.3885	cilia and flagella associated protein 36 [Source:HGNC Symbol;Acc	chr2:55746	NM_00128	.	.	.	NP_00126	.	protein_co	ENSG0000	112942	30540
CFAP43	2.20188	1.52656	1.28174	1.25961	1.14323	1.65804	cilia and flagella associated protein 43 [Source:HGNC Symbol;Acc	chr10:1058	NM_02514	XM_01701	.	.	NP_07942	XP_005270	protein_co	ENSG0000	80217	26684
CFAP44	1.0909	0.945011	1.12193	0.609485	1.0265	0.673334	cilia and flagella associated protein 44 [Source:HGNC Symbol;Acc	chr3:11308	NM_01833	XM_01151	.	.	NP_06080	XP_005247	protein_co	ENSG0000	55779	25631
CFAP45	0.413454	0.468145	0.268321	0.388436	0.372996	0.298397	cilia and flagella associated protein 45 [Source:HGNC Symbol;Acc	chr1:15984	NM_01233	.	.	.	NP_03646	.	protein_co	ENSG0000	25790	17229
CFAP46	0.625462	0.230542	0.50678	0.348202	0.470606	0.33415	cilia and flagella associated protein 46 [Source:HGNC Symbol;Acc	chr10:1346	NM_00120	XM_01153	.	.	NP_00118	.	protein_co	ENSG0000	54777	25247
CFAP47	0	0	0	0	0	0	cilia and flagella associated protein 47 [Source:HGNC Symbol;Acc	chrX:35937	NM_00130	.	.	.	NP_00129	.	protein_co	ENSG0000	286464	26708
CFAP52	0.065021	0.033483	0.158568	0.036027	0.037179	0.100737	cilia and flagella associated protein 52 [Source:HGNC Symbol;Acc	chr17:9479	NM_00108	XM_01702	.	.	NP_00107	XP_016879	protein_co	ENSG0000	146845	16053
CFAP53	0.802277	0.750474	0.966257	0.746304	0.880229	0.770341	cilia and flagella associated protein 53 [Source:HGNC Symbol;Acc	chr18:4775	NM_14502	.	.	.	NP_65945	.	protein_co	ENSG0000	220136	26530
CFAP54	0	0.008313	0.033393	0.026545	0.025281	0.022163	cilia and flagella associated protein 54 [Source:HGNC Symbol;Acc	chr12:9688	NM_00130	XM_01153	.	.	NP_00129	XP_011537	protein_co	ENSG0000	144535	26456
CFAP57	0.265805	0.165829	0.207551	0.378106	0.330835	0.282734	cilia and flagella associated protein 57 [Source:HGNC Symbol;Acc	chr1:43629	NM_00116	XM_01700	.	.	NP_00116	XP_005270	protein_co	ENSG0000	149465	26485
CFAP58	0.273701	0.238547	0.2408	0.600312	0.193674	0.402441	cilia and flagella associated protein 58 [Source:HGNC Symbol;Acc	chr10:1061	NM_00100	XM_01153	.	.	NP_00100	XP_011537	protein_co	ENSG0000	159686	26676
CFAP61	0.107416	0.05608	0.078504	0.259335	0.164198	0.018338	cilia and flagella associated protein 61 [Source:HGNC Symbol;Acc	chr20:2001	NM_00116	XM_01152	.	.	NP_00116	XP_005260	protein_co	ENSG0000	26074	15872
CFAP65	0.280185	0.065412	0.123419	0.24144	0.09387	0.114746	cilia and flagella associated protein 65 [Source:HGNC Symbol;Acc	chr2:21986	NM_00127	XM_01151	.	.	NP_00126	XP_011509	protein_co	ENSG0000	255101	25325
CFAP69	0.716998	0.472526	1.04638	0.264033	0.438897	0.625943	cilia and flagella associated protein 69 [Source:HGNC Symbol;Acc	chr7:89874	NM_00103	XM_01701	.	.	NP_00103	XP_011514	protein_co	ENSG0000	79846	26107
CFAP70	1.51022	1.40475	1.24489	0.970236	0.946398	0.804981	cilia and flagella associated protein 70 [Source:HGNC Symbol;Acc	chr10:7501	NM_14517	XM_01153	.	.	NP_66015	XP_005269	protein_co	ENSG0000	118491	30726
CFAP73	0	0.213897	0.126739	0.078604	0.177523	0.085255	cilia and flagella associated protein 73 [Source:HGNC Symbol;Acc	chr12:1135	NM_00114	.	.	.	NP_00113	.	protein_co	ENSG0000	387885	37100
CFAP74	0.310449	0.283441	0.414387	0.244022	0.207277	0.341472	cilia and flagella associated protein 74 [Source:HGNC Symbol;Acc	chr1:18533	NM_00130	XM_01154	.	.	NP_00129	XP_011540	protein_co	ENSG0000	85452	29368
CFAP77	0.652889	0.493285	0.703042	0.722266	0.79011	0.468771	cilia and flagella associated protein 77 [Source:HGNC Symbol;Acc	chr9:13528	NM_00128	XM_01151	.	.	NP_00126	XP_011516	protein_co	ENSG0000	389799	33776
CFAP97	22.0789	21.7335	26.0917	21.1196	28.4656	30.9808	cilia and flagella associated protein 97 [Source:HGNC Symbol;Acc	chr4:18608	NM_00129	XM_01700	.	.	NP_00127	XP_005263	protein_co	ENSG0000	57587	29276
CFAP99	0.074721	0.09727	0.260159	0.079712	0.075973	0.16779	cilia and flagella associated protein 99 [Source:HGNC Symbol;Acc	chr4:24207	NM_00119	XM_01151	.	.	NP_00118	XP_011511	protein_co	ENSG0000	402160	51180

CFB	5.37945	5.71255	0.5884	1.49775	1.63402	1.16054	complement factor B [Source:HGNC Symbol;Acc:HGNC:1037]	chr6:31865	NM_00171	.	.	.	NP_001701	.	protein_co	ENSG00000	629	1037
CFC1	0	0	0	0	0	0	cripto, FRL-1, cryptic family 1 [Source:HGNC Symbol;Acc:HGNC:18	chr2:13134	NM_00127	.	.	.	NP_001257	.	protein_co	ENSG00000	55997	18292
CFC1B	0	0	0	0	0	0	cripto, FRL-1, cryptic family 1B [Source:HGNC Symbol;Acc:HGNC:3	chr2:13127	NM_00107	XM_00526	.	.	NP_001072	XP_005263	protein_co	ENSG00000	653275	33983
CFD	61.361	55.8513	55.7574	69.2995	44.1628	45.0294	complement factor D [Source:HGNC Symbol;Acc:HGNC:2771]	chr19:8596	NM_00131	.	.	.	NP_001304	.	protein_co	ENSG00000	1675	2771
CFDP1	28.0476	23.9465	23.1374	25.6606	23.1535	25.331	craniofacial development protein 1 [Source:HGNC Symbol;Acc:HG	chr16:7532	NM_00632	.	.	.	NP_006315	.	protein_co	ENSG00000	10428	1873
CFH	0	0.016192	0.133075	0	0.033655	0.044223	complement factor H [Source:HGNC Symbol;Acc:HGNC:4883]	chr1:19662	NM_00018	.	.	.	NP_000177	XP_016856	protein_co	ENSG00000	3075	4883
CFHR1	0.66057	0.175028	0.240837	0.310187	0.244102	0.430188	complement factor H related 1 [Source:HGNC Symbol;Acc:HGNC:	chr1:19678	NM_00211	XM_01150	.	.	NP_002104	XP_011507	protein_co	ENSG00000	3078	4888
CFHR2	0	0	0	0	0	0	complement factor H related 2 [Source:HGNC Symbol;Acc:HGNC:	chr1:19678	NM_00566	XM_01150	.	.	NP_005657	XP_011507	protein_co	ENSG00000	3080	4890
CFHR3	0	0	0	0	0	0	complement factor H related 3 [Source:HGNC Symbol;Acc:HGNC:	chr1:19674	NM_00116	XM_01150	.	.	NP_001160	XP_011507	protein_co	ENSG00000	10878	16980
CFHR4	0	0	0	0	0	0	complement factor H related 4 [Source:HGNC Symbol;Acc:HGNC:	chr1:19678	NM_00120	XM_00671	.	.	NP_001188	XP_006711	protein_co	ENSG00000	10877	16979
CFHR5	0	0	0	0	0	0	complement factor H related 5 [Source:HGNC Symbol;Acc:HGNC:	chr1:19694	NM_03078	XM_01151	.	.	NP_110414	XP_011508	protein_co	ENSG00000	81494	24668
CFI	0.208738	0.143862	0.515302	0	0.061648	0.14269	complement factor I [Source:HGNC Symbol;Acc:HGNC:5394]	chr4:11066	NM_00020	XM_01153	.	.	NP_000195	XP_005263	protein_co	ENSG00000	3426	5394
CFL1	869.279	842.677	826.785	1088.08	930.103	877.781	cofilin 1 [Source:HGNC Symbol;Acc:HGNC:1874]	chr11:6562	NM_00550	.	.	.	NP_005498	.	protein_co	ENSG00000	1072	1874
CFL2	19.9124	19.4355	25.017	22.0576	27.4751	32.4054	cofilin 2 [Source:HGNC Symbol;Acc:HGNC:1875]	chr14:3517	NM_00124	XM_01153	.	.	NP_001230	XP_011534	protein_co	ENSG00000	1073	1875
CFLAR	10.3342	10.0815	10.362	7.82253	6.70967	7.04053	CASP8 and FADD like apoptosis regulator [Source:HGNC Symbol;A	chr2:20198	NM_00112	XM_01151	.	.	NP_001120	XP_005246	protein_co	ENSG00000	8837	1876
CFP	0.314446	0.230194	0.134982	0.222382	0.136988	0.308089	complement factor properdin [Source:HGNC Symbol;Acc:HGNC:8	chrX:47483	NM_00114	.	.	.	NP_001138	.	protein_co	ENSG00000	5199	8864
CFTR	0.011705	0.01251	0.010201	0	0	0.011466	cystic fibrosis transmembrane conductance regulator [Source:HG	chr7:11712	NM_00049	XM_01151	.	.	NP_000483	XP_011514	protein_co	ENSG00000	1080	1884
CGA	0.153521	0	0	0	0	0	glycoprotein hormones, alpha polypeptide [Source:HGNC Symbol	chr6:87795	NM_00073	.	.	.	NP_000726	.	protein_co	ENSG00000	1081	1885
CGB1	0	0	0	0	0	0	chorionic gonadotropin beta subunit 1 [Source:HGNC Symbol;Acc	chr19:4952	NM_03337	.	.	.	NP_203695	.	protein_co	ENSG00000	114335	16721
CGB2	1.03264	2.0226	1.74692	0.442557	0.966572	1.14362	chorionic gonadotropin beta subunit 2 [Source:HGNC Symbol;Acc	chr19:4952	NM_00131	XM_00525	.	.	NP_001305	.	protein_co	ENSG00000	114336	16722
CGB3	0	0	0	0	0	0	chorionic gonadotropin beta subunit 3 [Source:HGNC Symbol;Acc	chr19:4952	NM_00073	.	.	.	NP_000728	.	protein_co	ENSG00000	1082	1886
CGB5	0.127118	0.248698	0	0.272039	0.295721	0.14133	chorionic gonadotropin beta subunit 5 [Source:HGNC Symbol;Acc	chr19:4954	NM_03304	.	.	.	NP_149032	.	protein_co	ENSG00000	93659	16452
CGB7	0.640876	0.861228	1.29656	0.954554	0.503438	1.41619	chorionic gonadotropin beta subunit 7 [Source:HGNC Symbol;Acc	chr19:4955	NM_03314	XM_01152	.	.	NP_149133	XP_011525	protein_co	ENSG00000	94027	16451
CGB8	0	0.114845	0.09998	0.376991	0	0	chorionic gonadotropin beta subunit 8 [Source:HGNC Symbol;Acc	chr19:4955	NM_03318	.	.	.	NP_149439	.	protein_co	ENSG00000	94115	16453
CGGBP1	24.096	25.6161	24.0444	24.5583	21.6231	29.9523	CGG triplet repeat binding protein 1 [Source:HGNC Symbol;Acc:H	chr3:88101	NM_00100	XM_01700	.	.	NP_001008	XP_011532	protein_co	ENSG00000	8545	1888
CGN	6.05877	6.866	6.64468	3.59841	3.66424	3.38792	cingulin [Source:HGNC Symbol;Acc:HGNC:17429]	chr1:15148	NM_02077	XM_00524	.	.	NP_065821	XP_005245	protein_co	ENSG00000	57530	17429
CGNL1	1.09564	1.00428	1.14	1.43281	1.45377	1.4359	cingulin like 1 [Source:HGNC Symbol;Acc:HGNC:25931]	chr15:5766	NM_00125	XM_01702	.	.	NP_001239	XP_005254	protein_co	ENSG00000	84952	25931
CGREF1	2.87511	2.17757	3.45963	2.47773	3.10131	1.63365	cell growth regulator with EF-hand domain 1 [Source:HGNC Symb	chr2:27309	NM_00116	.	.	.	NP_001159	.	protein_co	ENSG00000	10669	16962
CGRRF1	6.48175	5.43863	4.54426	7.23012	5.89665	6.63983	cell growth regulator with ring finger domain 1 [Source:HGNC Syr	chr14:5497	NM_00656	.	.	.	NP_006559	.	protein_co	ENSG00000	10668	15528
CH17-140K24.5	0	0	0	0	0	0	.	chr5:14056	.	.	.	.	.	.	protein_co	ENSG00000	.	.
CH17-140K24.8	4.77E-05	0.138794	0	0.220475	0	0	.	chr5:14060	.	.	.	.	.	.	protein_co	ENSG00000	.	.
CH17-270A2.2	3.68E-25	8.25E-21	6.45E-14	7.01E-16	6.31E-17	3.31E-14	.	chr1:14545	.	.	.	.	.	.	protein_co	ENSG00000	.	.
CH17-296N19.1	0	0	0	0	0	0	.	chr9:43684	.	XM_01154	.	.	.	XP_011543	protein_co	ENSG00000	100289279	.
CH25H	0	0	0	0	0	0	cholesterol 25-hydroxylase [Source:HGNC Symbol;Acc:HGNC:190	chr10:9096	NM_00395	.	.	.	NP_003947	.	protein_co	ENSG00000	9023	1907
CH507-152C13.3	0	0	0	0	0	0	Homo sapiens alpha-crystallin A chain (LOC102724652), transcrip	chr21:4458	NM_00131	XM_00672	.	.	NP_001300	XP_006723	protein_co	ENSG00000	102724652	.
CH507-42P11.8	4.8457	2.57216	0.954632	6.53488	6.63995	1.97742	Homo sapiens serine/threonine-protein kinase SIK1 (LOC1027244	chr21:4483	NM_00132	XM_00672	.	.	NP_001307	XP_006723	protein_co	ENSG00000	102724428	.
CH507-9B2.1	0.000128	8.64E-10	0.231581	8.67E-07	2.35E-08	0.203537	.	chr21:4564	.	XM_00672	.	.	.	XP_006723	protein_co	ENSG00000	23308, 102	.
CH507-9B2.2	0	0	0	0	0	0	.	chr21:4566	.	.	.	.	.	.	protein_co	ENSG00000	.	.
CH507-9B2.3	28.6376	7.04247	19.7774	20.1228	0.243384	10.385	.	chr21:4552	.	XM_01702	.	.	.	XP_006723	protein_co	ENSG00000	8209, 1027	.
CH507-9B2.4	1.13E-58	0.892513	5.8E-51	0.976115	3.53E-59	9.68E-39	.	chr21:4552	.	.	.	.	.	.	protein_co	ENSG00000	.	.
CH507-9B2.5	31.8955	32.9435	31.7315	40.7237	36.869	26.5978	.	chr21:4552	.	XM_00672	.	.	.	XP_006723	protein_co	ENSG00000	102724159	.
CH507-9B2.9	0.156189	0.000158	1.64E-05	0.000536	0.000238	0.000274	.	chr21:4543	.	.	.	.	.	XP_011544	protein_co	ENSG00000	102724200	.
CHAC1	20.2576	19.0494	3.34114	7.5908	5.97333	69.6978	ChaC glutathione specific gamma-glutamylcyclotransferase 1 [Sou	chr15:4124	NM_00114	.	.	.	NP_001136	XP_016878	protein_co	ENSG00000	79094	28680
CHAC2	8.54509	6.93691	5.64573	8.77718	10.8266	8.05161	ChaC cation transport regulator homolog 2 [Source:HGNC Symbo	chr2:53897	NM_00100	.	.	.	NP_001008	.	protein_co	ENSG00000	494143	32363
CHAD	0.04696	0.046394	0.040344	0	0	0	chondroadherin [Source:HGNC Symbol;Acc:HGNC:1909]	chr17:4850	NM_00126	XM_01152	.	.	NP_001258	XP_011522	protein_co	ENSG00000	1101	1909
CHADL	0.048142	0	0.122127	0	0.057218	0	chondroadherin like [Source:HGNC Symbol;Acc:HGNC:25165]	chr22:4160	NM_13848	.	.	.	NP_612490	XP_005261	protein_co	ENSG00000	150356	25165
CHAF1A	23.7911	23.4864	21.4049	24.3841	23.4799	19.6126	chromatin assembly factor 1 subunit A [Source:HGNC Symbol;Acc	chr19:4402	NM_00548	XM_01152	.	.	NP_005474	XP_011525	protein_co	ENSG00000	10036	1910
CHAF1B	13.4923	14.2246	12.258	16.7479	12.6681	11.446	chromatin assembly factor 1 subunit B [Source:HGNC Symbol;Acc	chr21:3775	NM_00544	XM_01152	.	.	NP_005432	XP_011528	protein_co	ENSG00000	8208	1911
CHAMP1	20.8555	22.4485	21.6937	23.5848	26.2378	21.7524	chromosome alignment maintaining phosphoprotein 1 [Source:H	chr13:1150	NM_00116	.	.	.	NP_001157	.	protein_co	ENSG00000	283489	20311
CHAT	0.037411	0	0	0	0	0	choline O-acetyltransferase [Source:HGNC Symbol;Acc:HGNC:191	chr10:5081	NM_00114	.	.	.	NP_001136	.	protein_co	ENSG00000	1103	1912
CHCHD1	66.2931	62.3957	64.2566	79.8248	70.2374	57.2549	coiled-coil-helix-coiled-coil-helix domain containing 1 [Source:HG	chr10:7554	NM_20329	.	.	.	NP_976043	.	protein_co	ENSG00000	118487	23518
CHCHD10	289.948	263.33	247.519	339.995	266.312	197.362	coiled-coil-helix-coiled-coil-helix domain containing 10 [Source:H	chr22:2410	NM_00130	.	.	.	NP_001288	.	protein_co	ENSG00000	400916	15559
CHCHD2	444.099	427.674	399.181	566.334	437.137	430.073	coiled-coil-helix-coiled-coil-helix domain containing 2 [Source:HG	chr7:56169	NM_00132	.	.	.	NP_001307	.	protein_co	ENSG00000	51142	21645
CHCHD3	41.5726	43.5479	37.6826	49.6349	44.0251	39.7818	coiled-coil-helix-coiled-coil-helix domain containing 3 [Source:HG	chr7:13246	NM_00131	XM_01151	.	.	NP_001304	.	protein_co	ENSG00000	54927	21906
CHCHD4	23.2545	19.4913	17.1085	22.7098	20.7789	17.3745	coiled-coil-helix-coiled-coil-helix domain containing 4 [Source:HG	chr3:14153	NM_00109	.	.	.	NP_001091	.	protein_co	ENSG00000	131474	26467
CHCHD5	39.87	41.3611	32.4635	49.6431	34.0911	29.1381	coiled-coil-helix-coiled-coil-helix domain containing 5 [Source:HG	chr2:11334	NM_03230	XM_00526	.	.	NP_115685	XP_005263	protein_co	ENSG00000	84269	17840
CHCHD6	18.2032	21.6711	22.2335	20.4846	17.0305	16.123	coiled-coil-helix-coiled-coil-helix domain containing 6 [Source:HG	chr3:12642	NM_00132	XM_01151	.	.	NP_001307	XP_011511	protein_co	ENSG00000	84303	28184
CHCHD7	19.0337	21.0521	18.336	17.7742	17.9494	16.5285	coiled-coil-helix-coiled-coil-helix domain containing 7 [Source:HG	chr8:57124	NM_00101	.	.	.	NP_001011	.	protein_co	ENSG00000	79145	28314
CHD1	11.92	11.939	11.578	7.44553	9.95133	9.43056	chromodomain helicase DNA binding protein 1 [Source:HGNC Syr	chr5:98189	NM_00127	XM_01154	.	.	NP_001261	XP_005271	protein_co	ENSG00000	1105	1915

CHD1L	24.5467	24.8547	25.8086	24.7463	23.751	22.0165	chromodomain helicase DNA binding protein 1 like [Source:HGNC	chr1:14671	NM_00125	XM_01700	.	.	NP_00124	XP_006711	protein_co	ENSG0000	9557	1916
CHD3	11.6278	11.4086	11.5096	10.2615	10.3994	10.0726	chromodomain helicase DNA binding protein 3 [Source:HGNC Syr	chr17:7760	NM_00100	XM_01702	.	.	NP_00100	XP_005256	protein_co	ENSG0000	1107	1918
CHD4	70.8126	73.304	69.673	70.6238	68.6604	65.4799	chromodomain helicase DNA binding protein 4 [Source:HGNC Syr	chr12:6679	NM_00127	XM_01701	.	.	NP_00126	XP_005253	protein_co	ENSG0000	1108	1919
CHD5	1.91378	1.84557	2.15736	1.23092	1.33463	0.824915	chromodomain helicase DNA binding protein 5 [Source:HGNC Syr	chr1:61618	NM_01555	.	.	.	NP_05637	.	protein_co	ENSG0000	26038	16816
CHD6	4.653	4.90814	4.79604	4.06137	4.33216	5.05021	chromodomain helicase DNA binding protein 6 [Source:HGNC Syr	chr20:4003	NM_03222	XM_01702	.	.	NP_11559	XP_005260	protein_co	ENSG0000	84181	19057
CHD7	5.91057	5.94723	6.14094	6.83532	6.06298	6.75346	chromodomain helicase DNA binding protein 7 [Source:HGNC Syr	chr8:61591	NM_01778	XM_01701	.	.	NP_00130	XP_011515	protein_co	ENSG0000	55636	20626
CHD8	10.3799	11.6477	10.4669	13.9307	13.3332	12.5505	chromodomain helicase DNA binding protein 8 [Source:HGNC Syr	chr14:2185	NM_00117	.	.	.	NP_00116	.	protein_co	ENSG0000	57680	20153
CHD9	4.26408	3.71881	4.26283	3.09409	3.37516	3.71441	chromodomain helicase DNA binding protein 9 [Source:HGNC Syr	chr16:5308	NM_00130	XM_01702	.	.	NP_00129	XP_005256	protein_co	ENSG0000	80205	25701
CHDH	4.28823	3.98186	5.01575	3.59605	3.52398	2.63341	choline dehydrogenase [Source:HGNC Symbol;Acc:HGNC:24288]	chr3:53317	NM_01839	XM_01153	.	.	NP_06086	XP_006713	protein_co	ENSG0000	55349	24288
CHEK1	28.6762	27.1049	24.8572	29.9155	30.484	27.1895	checkpoint kinase 1 [Source:HGNC Symbol;Acc:HGNC:1925]	chr11:1254	NM_00111	XM_01154	.	.	NP_00110	XP_011540	protein_co	ENSG0000	1111	1925
CHEK2	23.7277	24.605	18.9161	32.4485	32.7772	22.6588	checkpoint kinase 2 [Source:HGNC Symbol;Acc:HGNC:16627]	chr22:2908	NM_00100	XM_01152	.	.	NP_00100	XP_006724	protein_co	ENSG0000	11200	16627
CHERP	24.7475	25.8325	22.2012	28.4854	26.6059	18.4164	calcium homeostasis endoplasmic reticulum protein [Source:HGN	chr19:1660	NM_00638	.	.	.	NP_00637	.	protein_co	ENSG0000	10523	16930
CHFR	0	0	0	0	0.008359	0	checkpoint with forkhead and ring finger domains, E3 ubiquitin p	chr12:1334	NM_00116	.	.	.	NP_00115	.	protein_co	ENSG0000	55743	20455
CHGA	0	0	0	0	0	0	chromogranin A [Source:HGNC Symbol;Acc:HGNC:1929]	chr14:9338	NM_00127	XM_01153	.	.	NP_00126	XP_011534	protein_co	ENSG0000	1113	1929
CHGB	0	0	0.047883	0.019705	0	0.019098	chromogranin B [Source:HGNC Symbol;Acc:HGNC:1930]	chr20:5892	NM_00181	.	.	.	NP_00181	.	protein_co	ENSG0000	1114	1930
CHI3L1	0	0	0	0.090131	0.095337	0.044602	chitinase 3 like 1 [Source:HGNC Symbol;Acc:HGNC:1932]	chr1:20314	NM_00127	XM_01150	.	.	NP_00126	.	protein_co	ENSG0000	1116	1932
CHI3L2	0.045961	0.048656	0	0	0.053981	0	chitinase 3 like 2 [Source:HGNC Symbol;Acc:HGNC:1933]	chr1:11172	NM_00102	.	.	.	NP_00102	.	protein_co	ENSG0000	1117	1933
CHIA	0	0	0	0	0	0	chitinase, acidic [Source:HGNC Symbol;Acc:HGNC:17432]	chr1:11183	NM_00104	XM_01700	.	.	NP_00103	XP_006710	protein_co	ENSG0000	27159	17432
CHIC1	0	0	0	0	0	0	cysteine rich hydrophobic domain 1 [Source:HGNC Symbol;Acc:H	chrX:72783	NM_00103	XM_01702	.	.	NP_00103	XP_016885	protein_co	ENSG0000	53344	1934
CHIC2	8.89546	8.6376	6.12211	5.29963	5.40985	9.37703	cysteine rich hydrophobic domain 2 [Source:HGNC Symbol;Acc:H	chr4:54243	NM_01211	XM_00671	.	.	NP_03624	XP_006714	protein_co	ENSG0000	26511	1935
CHID1	34.9601	37.7628	36.5523	36.3944	27.6402	31.9882	chitinase domain containing 1 [Source:HGNC Symbol;Acc:HGNC:2	chr11:8678	NM_00114	XM_01152	.	.	NP_00113	XP_005253	protein_co	ENSG0000	66005	28474
CHIT1	0	0	0	0	0	0	chitinase 1 [Source:HGNC Symbol;Acc:HGNC:1936]	chr1:20318	NM_00125	XM_01150	.	.	NP_00124	.	protein_co	ENSG0000	1118	1936
CHKA	13.6674	13.7121	16.2693	17.4265	17.6393	17.2356	choline kinase alpha [Source:HGNC Symbol;Acc:HGNC:1937]	chr11:6782	NM_00127	.	.	.	NP_00126	.	protein_co	ENSG0000	1119	1937
CHKB	11.6028	10.5445	12.9452	8.34915	7.45751	9.3357	choline kinase beta [Source:HGNC Symbol;Acc:HGNC:1938]	chr22:5101	NM_00519	.	.	.	NP_00518	.	protein_co	ENSG0000	1120	1938
CHL1	0	0	0	0	0	0	cell adhesion molecule L1 like [Source:HGNC Symbol;Acc:HGNC:1	chr3:23827	NM_00125	XM_01700	NR_04557	.	NP_00124	XP_006713	protein_co	ENSG0000	10752	1939
CHM	5.57613	6.33474	5.34861	6.28767	6.1504	7.00785	CHM, Rab escort protein 1 [Source:HGNC Symbol;Acc:HGNC:1940	chrX:85116	NM_00039	XM_01702	.	.	NP_00038	XP_016884	protein_co	ENSG0000	1121	1940
CHML	5.14886	4.89295	5.08533	5.68522	6.94386	6.49891	CHM like, Rab escort protein 2 [Source:HGNC Symbol;Acc:HGNC:1	chr1:24169	NM_00182	.	.	.	NP_00181	.	protein_co	ENSG0000	1122	1941
CHMP1A	87.9476	88.1382	86.876	110.059	98.1939	76.8919	charged multivesicular body protein 1A [Source:HGNC Symbol;Ac	chr16:8971	NM_00108	.	.	.	NP_00107	.	protein_co	ENSG0000	5119	8740
CHMP1B	26.5648	27.9862	25.952	22.8415	22.0903	25.1924	charged multivesicular body protein 1B [Source:HGNC Symbol;Ac	chr18:1168	NM_02041	.	.	.	NP_06514	.	protein_co	ENSG0000	57132	24287
CHMP2A	87.4277	80.4266	84.4728	74.0015	62.8761	78.4442	charged multivesicular body protein 2A [Source:HGNC Symbol;Ac	chr19:5906	NM_01445	XM_00525	.	.	NP_05526	XP_005258	protein_co	ENSG0000	27243	30216
CHMP2B	27.0583	24.8418	26.6412	19.0239	23.0244	22.7405	charged multivesicular body protein 2B [Source:HGNC Symbol;Ac	chr3:87276	NM_00124	.	.	.	NP_00123	.	protein_co	ENSG0000	25978	24537
CHMP3	32.7992	31.5104	34.1398	33.4963	33.7693	34.2419	charged multivesicular body protein 3 [Source:HGNC Symbol;Acc	chr2:86730	NM_00100	.	.	.	NP_00100	.	protein_co	ENSG0000	51652	29865
CHMP4A	35.4416	29.5016	25.5741	27.5924	21.3987	20.1152	charged multivesicular body protein 4A [Source:HGNC Symbol;Ac	chr14:2465	NM_01416	.	.	.	NP_05488	.	protein_co	ENSG0000	29082	20274
CHMP4B	59.2683	58.5421	55.8628	57.4302	49.1299	44.1996	charged multivesicular body protein 4B [Source:HGNC Symbol;Ac	chr20:3239	NM_17681	.	.	.	NP_78978	.	protein_co	ENSG0000	128866	16171
CHMP4C	11.2421	12.5465	9.23365	10.9491	10.655	12.6549	charged multivesicular body protein 4C [Source:HGNC Symbol;Ac	chr8:82644	NM_15228	.	.	.	NP_68949	.	protein_co	ENSG0000	92421	30599
CHMP5	40.9062	42.2413	34.5764	47.4723	41.7949	43.3623	charged multivesicular body protein 5 [Source:HGNC Symbol;Acc	chr9:33264	NM_00119	.	.	.	NP_00118	.	protein_co	ENSG0000	51510	26942
CHMP6	15.0586	16.796	14.1935	10.6833	9.74749	7.65752	charged multivesicular body protein 6 [Source:HGNC Symbol;Acc	chr17:7896	NM_02459	XM_00525	.	.	NP_07886	XP_005257	protein_co	ENSG0000	79643	25675
CHMP7	24.0707	23.909	24.1007	26.1102	24.517	22.6822	charged multivesicular body protein 7 [Source:HGNC Symbol;Acc	chr8:23101	NM_00131	XM_01154	.	.	NP_00130	XP_006716	protein_co	ENSG0000	91782	28439
CHN1	1.77532	1.75892	1.06528	2.28713	2.48923	1.92881	chimerin 1 [Source:HGNC Symbol;Acc:HGNC:1943]	chr2:17566	NM_00102	.	NR_03813	.	NP_00102	.	protein_co	ENSG0000	1123	1943
CHN2	0.815331	0.667358	0.61716	0.805861	1.1568	1.1436	chimerin 2 [Source:HGNC Symbol;Acc:HGNC:1944]	chr7:29034	NM_00103	XM_01151	NR_12052	.	NP_00103	XP_011513	protein_co	ENSG0000	101928168	1944
CHODL	0.041254	0.133132	0.088854	0	0.045791	0.049905	chondrolectin [Source:HGNC Symbol;Acc:HGNC:17807]	chr21:1927	NM_00120	XM_01702	.	.	NP_00119	XP_011527	protein_co	ENSG0000	140578	17807
CHORDC1	51.4248	61.6293	39.706	82.9771	81.9693	51.9433	cysteine and histidine rich domain containing 1 [Source:HGNC Syr	chr11:8993	NM_00114	XM_01154	.	.	NP_00113	XP_011541	protein_co	ENSG0000	26973	14525
CHP1	33.6386	34.031	41.3056	33.351	36.9496	42.4943	calcineurin like EF-hand protein 1 [Source:HGNC Symbol;Acc:HGN	chr15:4152	NM_00723	.	.	.	NP_00916	XP_016877	protein_co	ENSG0000	11261	17433
CHP2	0	0	0	0	0	0	calcineurin like EF-hand protein 2 [Source:HGNC Symbol;Acc:HGN	chr16:2376	NM_02209	.	.	.	NP_07138	.	protein_co	ENSG0000	63928	24927
CHPF	173.103	183.159	158.209	172.736	151.289	139.662	chondroitin polymerizing factor [Source:HGNC Symbol;Acc:HGNC	chr2:22040	NM_00119	.	.	.	NP_00118	.	protein_co	ENSG0000	79586	24291
CHPF2	10.4455	10.2504	10.3881	7.81112	8.54141	5.75793	chondroitin polymerizing factor 2 [Source:HGNC Symbol;Acc:HGN	chr7:15092	NM_00128	.	NR_03040	.	NP_00127	.	protein_co	ENSG0000	768213	29270
CHPT1	8.3275	8.39483	7.54885	19.5234	21.2636	19.7773	choline phosphotransferase 1 [Source:HGNC Symbol;Acc:HGNC:1	chr12:1020	NM_02024	XM_01153	.	.	NP_06462	XP_011536	protein_co	ENSG0000	56994	17852
CHRAC1	27.1082	29.0268	24.255	27.9481	28.6944	29.8988	chromatin accessibility complex 1 [Source:HGNC Symbol;Acc:HGN	chr8:14152	NM_01744	.	.	.	NP_05914	.	protein_co	ENSG0000	54108	13544
CHRD	3.47374	2.63303	4.54357	2.98345	2.62024	3.55038	chordin [Source:HGNC Symbol;Acc:HGNC:1949]	chr3:18409	NM_00130	XM_01151	.	.	NP_00129	XP_011511	protein_co	ENSG0000	8646	1949
CHRDL1	0	0	0	0	0	0	chordin like 1 [Source:HGNC Symbol;Acc:HGNC:29861]	chrX:10991	NM_00114	XM_01702	.	.	NP_00113	XP_005262	protein_co	ENSG0000	91851	29861
CHRDL2	0	0	0	0	0	0	chordin like 2 [Source:HGNC Symbol;Acc:HGNC:24168]	chr11:7440	NM_00127	.	.	.	NP_00126	.	protein_co	ENSG0000	25884	24168
CHRFAM7A	0.631771	0.428043	0.61327	0.403289	0.835457	0.592923	CHRNA7 (exons 5-10) and FAM7A (exons A-E) fusion [Source:HGN	chr15:3064	NM_13932	XM_01152	.	.	NP_64753	XP_005254	protein_co	ENSG0000	89832	15781
CHRM1	0.172686	0.056519	0.173017	0.244461	0.134291	0.224871	cholinergic receptor muscarinic 1 [Source:HGNC Symbol;Acc:HGN	chr11:6267	NM_00073	XM_01154	.	.	NP_00072	XP_011543	protein_co	ENSG0000	1128	1950
CHRM2	0	0	0	0	0	0	cholinergic receptor muscarinic 2 [Source:HGNC Symbol;Acc:HGN	chr7:13655	NM_00073	XM_01151	.	.	NP_00073	XP_011514	protein_co	ENSG0000	1129	1951
CHRM3	0	0	0	0.034852	0	0	cholinergic receptor muscarinic 3 [Source:HGNC Symbol;Acc:HGN	chr1:23979	NM_00074	XM_01700	.	.	NP_00073	XP_005273	protein_co	ENSG0000	1131	1952
CHRM4	0.672874	0.664972	1.51199	0.651869	0.789683	0.951502	cholinergic receptor muscarinic 4 [Source:HGNC Symbol;Acc:HGN	chr11:4640	NM_00074	.	.	.	NP_00073	.	protein_co	ENSG0000	1132	1953
CHRM5	0.025623	0	0	0	0	0	cholinergic receptor muscarinic 5 [Source:HGNC Symbol;Acc:HGN	chr15:3415	NM_01212	XM_01152	.	.	NP_00130	XP_005254	protein_co	ENSG0000	1133	1954
CHRNA1	0	0	0	0	0	0	cholinergic receptor nicotinic alpha 1 subunit [Source:HGNC Symb	chr2:17561	NM_00007	.	.	.	NP_00007	XP_016858	protein_co	ENSG0000	1134	1955
CHRNA10	1.68894	1.27792	1.89578	1.76257	2.0644	1.41377	cholinergic receptor nicotinic alpha 10 subunit [Source:HGNC Syr	chr11:3686	NM_00130	.	.	.	NP_00128	.	protein_co	ENSG0000	57053	13800

CHRNA2	0	0.018018	0.050421	0	0	0.046252	cholinergic receptor nicotinic alpha 2 subunit [Source:HGNC Symb	chr8:27317	NM_00074	XM_01154	.	.	NP_00073	XP_005273	protein_co	ENSG0000	1135	1956
CHRNA3	0.02639	0.054767	0.109485	0.058693	0.096901	0.1359	cholinergic receptor nicotinic alpha 3 subunit [Source:HGNC Symb	chr15:7885	NM_00074	.	.	.	NP_00073	.	protein_co	ENSG0000	1136	1957
CHRNA4	0	0	0	0	0	0	cholinergic receptor nicotinic alpha 4 subunit [Source:HGNC Symb	chr20:6197	NM_00074	XM_01152	.	.	NP_00073	XP_011526	protein_co	ENSG0000	1137	1958
CHRNA5	6.9464	7.40489	6.23308	7.67103	9.69718	8.76857	cholinergic receptor nicotinic alpha 5 subunit [Source:HGNC Symb	chr15:7885	NM_00074	XM_01152	.	.	NP_00073	XP_005254	protein_co	ENSG0000	1138	1959
CHRNA6	0	0.044211	0.08115	0	0.047049	0.083985	cholinergic receptor nicotinic alpha 6 subunit [Source:HGNC Symb	chr8:42607	NM_00119	.	.	.	NP_00118	.	protein_co	ENSG0000	8973	15963
CHRNA7	0.780426	1.50804	0.832887	0.722584	0.84294	1.04388	cholinergic receptor nicotinic alpha 7 subunit [Source:HGNC Symb	chr15:3225	NM_00074	XM_01699	.	.	NP_00073	XP_011506	protein_co	ENSG0000	10192997	1960
CHRNA9	0.066526	0	0	0	0.074522	0.033555	cholinergic receptor nicotinic alpha 9 subunit [Source:HGNC Symb	chr4:40337	NM_01758	.	.	.	NP_06005	.	protein_co	ENSG0000	55584	14079
CHRNB1	11.616	11.2988	9.76789	7.75364	6.94257	6.37108	cholinergic receptor nicotinic beta 1 subunit [Source:HGNC Symb	chr17:7348	NM_00074	.	.	.	NP_00073	.	protein_co	ENSG0000	1140	1961
CHRNB2	0	0	0.03069	0	0	0.038328	cholinergic receptor nicotinic beta 2 subunit [Source:HGNC Symb	chr1:15454	NM_00074	.	.	.	NP_00073	.	protein_co	ENSG0000	1141	1962
CHRNB3	0	0	0	0	0	0	cholinergic receptor nicotinic beta 3 subunit [Source:HGNC Symb	chr8:42552	NM_00074	.	.	.	NP_00074	.	protein_co	ENSG0000	1142	1963
CHRNB4	0.708092	0.95975	1.34912	0.640512	1.10275	0.823296	cholinergic receptor nicotinic beta 4 subunit [Source:HGNC Symb	chr15:7891	NM_00075	.	.	.	NP_00074	XP_011519	protein_co	ENSG0000	1143	1964
CHRND	0	0	0	0	0.038958	0.026701	cholinergic receptor nicotinic delta subunit [Source:HGNC Symbo	chr2:23339	NM_00075	XM_01151	.	.	NP_00074	XP_011508	protein_co	ENSG0000	1144	1965
CHRNE	0.037222	0	0	0	0.128538	0.08074	cholinergic receptor nicotinic epsilon subunit [Source:HGNC Symb	chr17:4736	NM_00008	.	.	.	NP_00007	XP_016879	protein_co	ENSG0000	1145	1966
CHRNG	0	0	0	0	0	0	cholinergic receptor nicotinic gamma subunit [Source:HGNC Symb	chr2:23340	NM_00519	.	.	.	NP_00519	.	protein_co	ENSG0000	1146	1967
CHST1	0.267363	0.553975	0.682172	0.977536	0.718008	1.41486	carbohydrate sulfotransferase 1 [Source:HGNC Symbol;Acc:HGNC	chr11:4567	NM_00365	XM_00671	.	.	NP_00364	XP_006718	protein_co	ENSG0000	8534	1969
CHST10	0	0	0	0	0	0	carbohydrate sulfotransferase 10 [Source:HGNC Symbol;Acc:HGN	chr2:10100	NM_00485	XM_01151	.	.	NP_00484	XP_011510	protein_co	ENSG0000	9486	19650
CHST11	3.31047	3.43308	4.69847	2.76439	2.56342	3.51695	carbohydrate sulfotransferase 11 [Source:HGNC Symbol;Acc:HGN	chr12:1048	NM_00117	.	.	.	NP_00116	.	protein_co	ENSG0000	50515	17422
CHST12	8.34095	7.45752	7.82904	8.02876	6.78815	5.64014	carbohydrate sulfotransferase 12 [Source:HGNC Symbol;Acc:HGN	chr7:24431	NM_00124	XM_01151	NR_10806	.	NP_00123	XP_011513	protein_co	ENSG0000	101927181	17423
CHST13	1.12351	0.990758	1.08287	0.421409	0.572366	1.66083	carbohydrate sulfotransferase 13 [Source:HGNC Symbol;Acc:HGN	chr3:12624	NM_15288	.	.	.	NP_69084	.	protein_co	ENSG0000	166012	21755
CHST14	9.65	8.1438	9.08733	10.3766	7.33299	6.9711	carbohydrate sulfotransferase 14 [Source:HGNC Symbol;Acc:HGN	chr15:4076	NM_13046	.	.	.	NP_56973	.	protein_co	ENSG0000	113189	24464
CHST15	23.5881	24.74	33.7695	18.7	20.5899	25.2447	carbohydrate sulfotransferase 15 [Source:HGNC Symbol;Acc:HGN	chr10:1257	NM_00127	XM_01701	.	.	NP_00125	XP_005269	protein_co	ENSG0000	51363	18137
CHST2	1.30582	1.7106	1.80879	1.43595	1.33757	1.35345	carbohydrate sulfotransferase 2 [Source:HGNC Symbol;Acc:HGNC	chr3:14283	NM_00426	.	.	.	NP_00425	.	protein_co	ENSG0000	9435	1970
CHST3	7.62959	7.60361	5.38713	7.22684	7.46727	5.86459	carbohydrate sulfotransferase 3 [Source:HGNC Symbol;Acc:HGNC	chr10:7372	NM_00427	XM_01154	.	.	NP_00426	XP_006718	protein_co	ENSG0000	9469	1971
CHST4	1.11323	0.885256	0.405228	0.884201	0.517585	0.209983	carbohydrate sulfotransferase 4 [Source:HGNC Symbol;Acc:HGNC	chr16:7155	NM_00116	.	.	.	NP_00115	.	protein_co	ENSG0000	10164	1972
CHST5	0.221798	0.095014	0.188681	0.17176	0.109531	0.170578	carbohydrate sulfotransferase 5 [Source:HGNC Symbol;Acc:HGNC	chr16:7556	NM_02453	.	.	.	NP_07880	.	protein_co	ENSG0000	23563	1973
CHST6	6.29758	6.35758	7.44586	8.15207	8.16925	8.72529	carbohydrate sulfotransferase 6 [Source:HGNC Symbol;Acc:HGNC	chr16:7550	NM_02161	XM_01152	.	.	NP_06762	XP_005256	protein_co	ENSG0000	4166	6938
CHST7	4.199	5.02862	5.87775	6.03313	5.61366	9.00641	carbohydrate sulfotransferase 7 [Source:HGNC Symbol;Acc:HGNC	chrX:46433	NM_01988	.	.	.	NP_06393	.	protein_co	ENSG0000	56548	13817
CHST8	0	0	0	0	0	0	carbohydrate sulfotransferase 8 [Source:HGNC Symbol;Acc:HGNC	chr19:3411	NM_00112	XM_01152	.	.	NP_00112	XP_011525	protein_co	ENSG0000	64377	15993
CHST9	0	0	0	0	0	0	carbohydrate sulfotransferase 9 [Source:HGNC Symbol;Acc:HGNC	chr18:2448	NM_00125	.	.	.	NP_00124	XP_016881	protein_co	ENSG0000	83539	19898
CHSY1	13.3392	14.9425	14.0046	14.186	15.6138	14.1001	chondroitin sulfate synthase 1 [Source:HGNC Symbol;Acc:HGNC:1	chr15:1017	NM_01491	.	.	.	NP_05573	.	protein_co	ENSG0000	22856	17198
CHSY3	1.06162	0.85903	1.25056	0.498236	0.524241	0.45284	chondroitin sulfate synthase 3 [Source:HGNC Symbol;Acc:HGNC:2	chr5:12924	NM_17585	.	.	.	NP_78705	.	protein_co	ENSG0000	337876	24293
CHTF18	19.381	20.2447	18.623	18.257	16.3668	12.1157	chromosome transmission fidelity factor 18 [Source:HGNC Symbo	chr16:8349	NM_02209	XM_01702	.	.	NP_07137	XP_005255	protein_co	ENSG0000	63922	18435
CHTF8	115.835	114.645	105	130.294	134.671	119.106	chromosome transmission fidelity factor 8 [Source:HGNC Symbol	chr16:6913	NM_00103	XM_01152	.	.	NP_00103	XP_011521	protein_co	ENSG0000	54921	24353
CHTOP	67.6298	68.0907	62.0576	65.4801	61.8666	67.2026	chromatin target of PRMT1 [Source:HGNC Symbol;Acc:HGNC:245	chr1:15360	NM_00120	XM_01700	.	.	NP_00119	XP_016856	protein_co	ENSG0000	26097	24511
CHUK	20.0846	20.1729	19.799	23.3324	27.5294	25.069	conserved helix-loop-helix ubiquitous kinase [Source:HGNC Symb	chr10:1019	NM_00127	XM_01153	.	.	NP_00126	XP_016871	protein_co	ENSG0000	1147	1974
CHURC1	63.3236	49.5944	61.5787	73.06	52.6609	66.4541	churchill domain containing 1 [Source:HGNC Symbol;Acc:HGNC:2	chr14:6538	NM_00120	.	.	.	NP_00119	.	protein_co	ENSG0000	91612	20099
CHURC1-FNTB	1.07E-61	0.673474	1.02817	4.45E-05	3.66E-28	0.670877	CHURC1-FNTB readthrough [Source:HGNC Symbol;Acc:HGNC:429	chr14:6538	NM_00120	.	.	.	NP_00118	.	protein_co	ENSG0000	100529261	42960
CIAO1	49.9517	53.5356	54.2964	63.5041	53.5878	39.8492	cytosolic iron-sulfur assembly component 1 [Source:HGNC Symbo	chr2:96931	NM_00480	.	.	.	NP_00479	.	protein_co	ENSG0000	9391	14280
CIAPIN1	44.5989	45.4856	39.5991	54.1899	46.0955	35.5039	cytokine induced apoptosis inhibitor 1 [Source:HGNC Symbol;Acc	chr16:5746	NM_00130	XM_00525	.	.	NP_00129	XP_005256	protein_co	ENSG0000	57019	28050
CIART	0.177309	0.12346	0.312959	0.24308	0.483565	0.403999	circadian associated repressor of transcription [Source:HGNC Sym	chr1:15025	NM_00130	.	.	.	NP_00128	.	protein_co	ENSG0000	148523	25200
CIB1	62.3058	61.5785	54.1431	65.8288	44.6397	37.5871	calcium and integrin binding 1 [Source:HGNC Symbol;Acc:HGNC:1	chr15:9077	NM_00127	.	.	.	NP_00126	.	protein_co	ENSG0000	10519	16920
CIB2	11.4381	10.3782	15.3632	12.9917	11.1808	11.4478	calcium and integrin binding family member 2 [Source:HGNC Sym	chr15:7837	NM_00127	XM_01152	.	.	NP_00125	XP_006720	protein_co	ENSG0000	10518	24579
CIB3	0	0	0.09513	0	0	0	calcium and integrin binding family member 3 [Source:HGNC Sym	chr19:1627	NM_00130	.	.	.	NP_00128	.	protein_co	ENSG0000	117286	24580
CIB4	0	0	0	0	0	0	calcium and integrin binding family member 4 [Source:HGNC Sym	chr2:26804	NM_00102	XM_01153	.	.	NP_00102	XP_011530	protein_co	ENSG0000	130106	33703
CIC	13.1719	14.9094	13.4591	10.7997	10.4838	9.27753	capicua transcriptional repressor [Source:HGNC Symbol;Acc:HGN	chr19:4277	NM_00130	XM_01152	.	.	NP_00129	XP_005258	protein_co	ENSG0000	23152	14214
CIDEA	0	0	0	0	0	0	cell death-inducing DFFA-like effector a [Source:HGNC Symbol;Ac	chr18:1225	NM_00127	.	.	.	NP_00127	.	protein_co	ENSG0000	1149	1976
CIDEB	0.190826	0.341058	0.288694	0	0.324317	0.285032	cell death-inducing DFFA-like effector b [Source:HGNC Symbol;Ac	chr14:2476	NM_00131	XM_01153	.	.	NP_00130	XP_005267	protein_co	ENSG0000	27141	1977
CIDEC	0.074829	0.075149	0.076928	0.482397	0.32477	0.081188	cell death inducing DFFA like effector c [Source:HGNC Symbol;Acc	chr3:99083	NM_00119	XM_00526	.	.	NP_00118	XP_005265	protein_co	ENSG0000	63924	24229
CIITA	0.073013	0.048248	0.13104	0.028324	0.030403	0.067411	class II major histocompatibility complex transactivator [Source:H	chr16:1097	NM_00024	XM_01152	.	.	NP_00023	XP_006720	protein_co	ENSG0000	4261	7067
CILP	0.023822	0.024353	0.020599	0.013041	0	0.012536	cartilage intermediate layer protein [Source:HGNC Symbol;Acc:H	chr15:6548	NM_00361	.	.	.	NP_00360	XP_016878	protein_co	ENSG0000	8483	1980
CILP2	5.60643	6.03074	8.33846	6.2714	6.26404	6.12473	cartilage intermediate layer protein 2 [Source:HGNC Symbol;Acc:	chr19:1964	NM_15322	.	.	.	NP_69495	.	protein_co	ENSG0000	148113	24213
CINP	29.774	27.7245	25.1723	37.7071	27.4093	22.8682	cyclin dependent kinase 2 interacting protein [Source:HGNC Symb	chr14:1028	NM_03263	XM_01153	.	.	NP_00130	XP_005267	protein_co	ENSG0000	51550	23789
CIPC	12.6044	11.7699	13.2909	11.333	11.9654	11.2842	CLOCK interacting pacemaker [Source:HGNC Symbol;Acc:HGNC:2	chr14:7756	NM_03342	.	.	.	NP_21949	.	protein_co	ENSG0000	85457	20365
CIR1	8.23259	7.60829	7.36796	5.7495	5.70712	7.25406	corepressor interacting with RBPJ, 1 [Source:HGNC Symbol;Acc:H	chr2:17521	NM_00488	.	.	.	NP_00487	.	protein_co	ENSG0000	9541	24217
CIRBP	195.414	186.453	184.012	183.434	174.682	152.358	cold inducible RNA binding protein [Source:HGNC Symbol;Acc:HG	chr19:1259	NM_00128	XM_00672	.	.	NP_00127	XP_006722	protein_co	ENSG0000	1153	1982
CISD1	17.0588	17.1265	15.8931	16.0139	13.2032	11.8372	CDGSH iron sulfur domain 1 [Source:HGNC Symbol;Acc:HGNC:308	chr10:6002	NM_01846	.	.	.	NP_06093	.	protein_co	ENSG0000	55847	30880
CISD2	25.5363	28.2795	18.9099	29.5764	28.9326	31.9652	CDGSH iron sulfur domain 2 [Source:HGNC Symbol;Acc:HGNC:242	chr4:10379	NM_00100	.	.	.	NP_00100	.	protein_co	ENSG0000	493856	24212
CISD3	14.4744	13.2931	14.6371	13.6369	10.523	8.86213	CDGSH iron sulfur domain 3 [Source:HGNC Symbol;Acc:HGNC:275	chr17:3688	NM_00113	.	.	.	NP_00112	.	protein_co	ENSG0000	284106	27578
CISH	2.93756	3.52499	2.00913	3.09139	3.14157	2.16467	cytokine inducible SH2 containing protein [Source:HGNC Symbol;	chr3:50643	NM_01332	.	.	.	NP_03745	XP_011531	protein_co	ENSG0000	1154	1984

CIT	5.30467	5.33491	5.49044	7.86668	8.30895	7.88902	citron rho-interacting serine/threonine kinase [Source:HGNC Sym	chr12:1201	NM_00120	XM_01701	NR_03158	.	NP_00119	XP_006719	protein_co	ENSG0000	100302274	1985
CITED1	1.61627	2.37482	2.90984	3.02229	2.91218	1.83387	Cbp/p300 interacting transactivator with Glu/Asp rich carboxy-te	chrX:71521	NM_00114	XM_01153	.	.	NP_00113	XP_011529	protein_co	ENSG0000	4435	1986
CITED2	25.1898	23.888	20.3837	24.0074	21.2482	21.1395	Cbp/p300 interacting transactivator with Glu/Asp rich carboxy-te	chr6:13969	NM_00116	.	.	.	NP_00116	.	protein_co	ENSG0000	10370	1987
CITED4	4.75358	6.04334	9.81435	4.98289	2.91333	6.69631	Cbp/p300 interacting transactivator with Glu/Asp rich carboxy-te	chr1:41326	NM_13346	.	.	.	NP_59772	.	protein_co	ENSG0000	163732	18696
CIZ1	26.5591	27.8467	22.718	28.2993	25.2911	18.9804	CDKN1A interacting zinc finger protein 1 [Source:HGNC Symbol;A	chr9:13092	NM_00113	XM_01701	.	.	NP_00112	XP_005251	protein_co	ENSG0000	25792	16744
CKAP2	29.6959	30.8332	29.7398	40.2588	51.6777	45.7699	cytoskeleton associated protein 2 [Source:HGNC Symbol;Acc:HGN	chr13:5302	NM_00109	XM_01153	.	.	NP_00109	XP_005266	protein_co	ENSG0000	26586	1990
CKAP2L	7.54177	8.66375	7.09304	10.7697	11.915	11.7573	cytoskeleton associated protein 2 like [Source:HGNC Symbol;Acc:	chr2:11347	NM_00130	.	.	.	NP_00129	.	protein_co	ENSG0000	150468	26877
CKAP4	52.3422	53.8857	38.1081	54.88	48.6637	43.0192	cytoskeleton associated protein 4 [Source:HGNC Symbol;Acc:HGN	chr12:1066	NM_00682	.	.	.	NP_00681	.	protein_co	ENSG0000	10970	16991
CKAP5	38.253	39.3176	38.8061	24.7208	26.3603	24.1684	cytoskeleton associated protein 5 [Source:HGNC Symbol;Acc:HGN	chr11:4676	NM_00100	.	.	.	NP_00100	.	protein_co	ENSG0000	9793	28959
CKB	255.522	249.308	301.272	326.6	265.284	256.928	creatine kinase B [Source:HGNC Symbol;Acc:HGNC:1991]	chr14:1039	NM_00182	.	.	.	NP_00181	XP_016876	protein_co	ENSG0000	1152	1991
CKLF	40.7094	39.9129	30.0709	56.6965	41.0766	36.0125	chemokine like factor [Source:HGNC Symbol;Acc:HGNC:13253]	chr16:6658	NM_00104	.	.	.	NP_00103	.	protein_co	ENSG0000	51192	13253
CKLF-CMTM1	2.67783	2.54332	1.4542	2.16724	3.95984	4.54E-15	CKLF-CMTM1 readthrough [Source:HGNC Symbol;Acc:HGNC:3997	chr16:6658	NM_00120	.	.	.	NP_00118	.	protein_co	ENSG0000	100529251	39977
CKM	0.038778	0	0	0	0	0	creatine kinase, M-type [Source:HGNC Symbol;Acc:HGNC:1994]	chr19:4580	NM_00182	XM_01702	.	.	NP_00181	XP_016881	protein_co	ENSG0000	1158	1994
CKMT1A	2.68692	2.48255	2.51481	3.66676	1.96611	2.86047	creatine kinase, mitochondrial 1A [Source:HGNC Symbol;Acc:HGN	chr15:4398	NM_00101	XM_01152	.	.	NP_00101	XP_005254	protein_co	ENSG0000	548596	31736
CKMT1B	2.5538	1.68359	2.54546	2.20259	1.19406	1.72251	creatine kinase, mitochondrial 1B [Source:HGNC Symbol;Acc:HGN	chr15:4388	NM_02099	XM_01152	.	.	NP_06627	XP_005254	protein_co	ENSG0000	1159	1995
CKMT2	0.043859	0	0	0	0	0	creatine kinase, mitochondrial 2 [Source:HGNC Symbol;Acc:HGNC	chr5:80529	NM_00109	.	.	.	NP_00109	.	protein_co	ENSG0000	1160	1996
CKS1B	145.216	146.607	110.291	174.27	153.659	155.118	CDC28 protein kinase regulatory subunit 1B [Source:HGNC Symbo	chr1:15494	NM_00182	.	.	.	NP_00181	.	protein_co	ENSG0000	1163	19083
CKS2	234.402	224.605	193.243	418.904	338.01	291.979	CDC28 protein kinase regulatory subunit 2 [Source:HGNC Symbol	chr9:91926	NM_00182	.	.	.	NP_00181	.	protein_co	ENSG0000	1164	2000
CLASP1	8.62022	8.20668	8.99912	8.18444	9.61279	9.31789	cytoplasmic linker associated protein 1 [Source:HGNC Symbol;Ac	chr2:12209	NM_00114	XM_01700	.	.	NP_00113	XP_006712	protein_co	ENSG0000	23332	17088
CLASP2	5.35458	4.5065	4.49813	4.02411	5.44918	5.27186	cytoplasmic linker associated protein 2 [Source:HGNC Symbol;Ac	chr3:33537	NM_00120	XM_01700	.	.	NP_00119	XP_006713	protein_co	ENSG0000	23122	17078
CLASRP	11.6423	9.68677	10.8893	10.3445	8.10897	8.08995	CLK4 associating serine/arginine rich protein [Source:HGNC Symb	chr19:4554	NM_00127	XM_01152	.	.	NP_00126	XP_011524	protein_co	ENSG0000	11129	17731
CLC	0	0	0	0	0	0	Charcot-Leyden crystal galectin [Source:HGNC Symbol;Acc:HGNC	chr19:4022	NM_00182	.	.	.	NP_00181	.	protein_co	ENSG0000	1178	2014
CLCA1	0	0	0	0	0	0	chloride channel accessory 1 [Source:HGNC Symbol;Acc:HGNC:20	chr1:86934	NM_00128	.	.	.	NP_00127	.	protein_co	ENSG0000	1179	2015
CLCA2	0	0	0.031969	0.020941	0.06086	0	chloride channel accessory 2 [Source:HGNC Symbol;Acc:HGNC:20	chr1:86889	NM_00653	.	.	.	NP_00652	.	protein_co	ENSG0000	9635	2016
CLCA4	0	0	0	0	0	0	chloride channel accessory 4 [Source:HGNC Symbol;Acc:HGNC:20	chr1:87012	NM_01212	XM_01154	.	.	NP_03626	XP_011539	protein_co	ENSG0000	22802	2018
CLCC1	8.26405	8.43856	7.37369	7.33317	6.87067	5.86339	chloride channel CLIC like 1 [Source:HGNC Symbol;Acc:HGNC:296	chr1:10935	NM_00104	.	.	.	NP_00104	.	protein_co	ENSG0000	23155	29675
CLCF1	6.29295	6.27547	5.49197	4.86072	4.36413	4.33965	cardiotrophin-like cytokine factor 1 [Source:HGNC Symbol;Acc:HG	chr11:6711	NM_00116	.	.	.	NP_00115	.	protein_co	ENSG0000	23529	17412
CLCN1	0.697665	0.55868	0.456378	0.756201	0.580966	0.360559	chloride voltage-gated channel 1 [Source:HGNC Symbol;Acc:HGN	chr7:14301	NM_00008	XM_01151	.	.	NP_00007	.	protein_co	ENSG0000	1180	2019
CLCN2	8.66479	9.51297	7.84732	9.82646	8.66522	6.67737	chloride voltage-gated channel 2 [Source:HGNC Symbol;Acc:HGN	chr3:18405	NM_00117	XM_01151	.	.	NP_00116	XP_006713	protein_co	ENSG0000	1181	2020
CLCN3	33.9042	37.5279	30.0775	33.6273	38.7601	47.9869	chloride voltage-gated channel 3 [Source:HGNC Symbol;Acc:HGN	chr4:17054	NM_00124	XM_01153	.	.	NP_00123	XP_005262	protein_co	ENSG0000	1182	2021
CLCN4	1.08876	1.14311	1.42807	0.931536	0.892243	1.18156	chloride voltage-gated channel 4 [Source:HGNC Symbol;Acc:HGN	chrX:10124	NM_00125	.	.	.	NP_00124	.	protein_co	ENSG0000	1183	2022
CLCN5	2.29995	2.6502	2.61968	2.16445	3.85429	3.27402	chloride voltage-gated channel 5 [Source:HGNC Symbol;Acc:HGN	chrX:49687	NM_00008	XM_01702	.	.	NP_00007	XP_016884	protein_co	ENSG0000	1184	2023
CLCN6	2.83822	2.88098	3.5824	2.9604	3.26698	2.39256	chloride voltage-gated channel 6 [Source:HGNC Symbol;Acc:HGN	chr1:11866	NM_00125	.	.	.	NP_00124	.	protein_co	ENSG0000	1185	2024
CLCN7	13.0029	15.7335	15.9891	12.9182	11.3778	11.8698	chloride voltage-gated channel 7 [Source:HGNC Symbol;Acc:HGN	chr16:1494	NM_00111	XM_01152	.	.	NP_00110	XP_011520	protein_co	ENSG0000	1186	2025
CLCNKA	0.033239	0.066332	0.114066	0.214766	0.161931	0.14747	chloride voltage-gated channel Ka [Source:HGNC Symbol;Acc:HG	chr1:16348	NM_00104	.	.	.	NP_00103	.	protein_co	ENSG0000	1187	2026
CLCNKB	0.288864	0.123801	0.44636	0.249591	0.313135	0.6088	chloride voltage-gated channel Kb [Source:HGNC Symbol;Acc:HG	chr1:16370	NM_00008	XM_01154	.	.	NP_00007	.	protein_co	ENSG0000	1188	2027
CLDN1	0.058766	0.031358	0.101122	0.033395	0.065296	0.403149	claudin 1 [Source:HGNC Symbol;Acc:HGNC:2032]	chr3:19002	NM_02110	.	.	.	NP_06692	.	protein_co	ENSG0000	9076	2032
CLDN10	0	0	0	0	0	0	claudin 10 [Source:HGNC Symbol;Acc:HGNC:2033]	chr13:9608	NM_00116	XM_01152	.	.	NP_00115	XP_011519	protein_co	ENSG0000	9071	2033
CLDN11	0	0	0	0	0	0.051294	claudin 11 [Source:HGNC Symbol;Acc:HGNC:8514]	chr3:17013	NM_00560	.	NR_13555	.	NP_00559	.	protein_co	ENSG0000	101928583	8514
CLDN12	17.9299	16.936	17.4064	11.5428	16.2042	17.7159	claudin 12 [Source:HGNC Symbol;Acc:HGNC:2034]	chr7:90032	NM_00118	.	.	.	NP_00117	.	protein_co	ENSG0000	9069	2034
CLDN14	0	0	0	0	0	0	claudin 14 [Source:HGNC Symbol;Acc:HGNC:2035]	chr21:3783	NM_00114	XM_01152	.	.	NP_00113	XP_011527	protein_co	ENSG0000	23562	2035
CLDN15	7.14799	7.57932	7.55885	7.09268	6.47385	6.82632	claudin 15 [Source:HGNC Symbol;Acc:HGNC:2036]	chr7:10087	NM_00118	.	.	.	NP_00117	.	protein_co	ENSG0000	24146	2036
CLDN16	0	0	0	0.036169	0	0.030692	claudin 16 [Source:HGNC Symbol;Acc:HGNC:2037]	chr3:19010	NM_00658	.	.	.	NP_00657	.	protein_co	ENSG0000	10686	2037
CLDN17	0	0	0	0	0	0	claudin 17 [Source:HGNC Symbol;Acc:HGNC:2038]	chr21:3153	NM_01213	.	.	.	NP_03626	.	protein_co	ENSG0000	26285	2038
CLDN18	0	0	0	0	0	0	claudin 18 [Source:HGNC Symbol;Acc:HGNC:2039]	chr3:13771	NM_00100	.	.	.	NP_00100	.	protein_co	ENSG0000	51208	2039
CLDN19	0	0	0	0	0	0.029261	claudin 19 [Source:HGNC Symbol;Acc:HGNC:2040]	chr1:43198	NM_00112	.	.	.	NP_00111	.	protein_co	ENSG0000	149461	2040
CLDN2	0.199968	0.512908	0.757107	0.054811	0.113068	0.183432	claudin 2 [Source:HGNC Symbol;Acc:HGNC:2041]	chrX:10614	NM_00117	.	.	.	NP_00116	.	protein_co	ENSG0000	9075	2041
CLDN20	0	0	0	0	0	0	claudin 20 [Source:HGNC Symbol;Acc:HGNC:2042]	chr6:15505	NM_00100	XM_01153	.	.	NP_00100	XP_011534	protein_co	ENSG0000	49861	2042
CLDN22	0	0	0	0	0	0	claudin 22 [Source:HGNC Symbol;Acc:HGNC:2044]	chr4:18402	NM_00111	.	.	.	NP_00110	.	protein_co	ENSG0000	53842	2044
CLDN23	2.69829	2.68502	2.52142	2.13593	1.54114	1.32933	claudin 23 [Source:HGNC Symbol;Acc:HGNC:17591]	chr8:85594	NM_19428	.	.	.	NP_91926	.	protein_co	ENSG0000	137075	17591
CLDN24	0	0	0	0	0	0	claudin 24 [Source:HGNC Symbol;Acc:HGNC:37200]	chr4:18424	NM_00118	.	.	.	NP_00117	.	protein_co	ENSG0000	100132463	37200
CLDN25	0	0	0	0	0	0	claudin 25 [Source:HGNC Symbol;Acc:HGNC:37218]	chr11:1136	NM_00110	.	.	.	NP_00109	.	protein_co	ENSG0000	644672	37218
CLDN3	7.36666	8.46693	12.6189	6.19462	6.22187	6.82854	claudin 3 [Source:HGNC Symbol;Acc:HGNC:2045]	chr7:73183	NM_00130	.	.	.	NP_00129	.	protein_co	ENSG0000	1365	2045
CLDN34	0	0	0	0	0	0	claudin 34 [Source:HGNC Symbol;Acc:HGNC:51259]	chrX:99353	NM_00119	.	.	.	NP_00118	.	protein_co	ENSG0000	100288814	51259
CLDN4	111.189	107.487	106.862	99.843	89.0662	106.516	claudin 4 [Source:HGNC Symbol;Acc:HGNC:2046]	chr7:73242	NM_00130	.	.	.	NP_00129	.	protein_co	ENSG0000	1364	2046
CLDN5	0	0	0	0	0	0	claudin 5 [Source:HGNC Symbol;Acc:HGNC:2047]	chr22:1951	NM_00113	XM_01702	.	.	NP_00112	XP_016884	protein_co	ENSG0000	7122	2047
CLDN6	0.114127	0.243463	0	0	0.242669	0.300525	claudin 6 [Source:HGNC Symbol;Acc:HGNC:2048]	chr16:3064	NM_02119	.	.	.	NP_06701	.	protein_co	ENSG0000	9074	2048
CLDN7	172.882	173.071	144.761	156.847	136.441	185.986	claudin 7 [Source:HGNC Symbol;Acc:HGNC:2049]	chr17:7146	NM_00118	.	.	.	NP_00117	.	protein_co	ENSG0000	1366	2049
CLDN8	0	0	0	0	0	0	claudin 8 [Source:HGNC Symbol;Acc:HGNC:2050]	chr21:3158	NM_19932	.	.	.	NP_95536	.	protein_co	ENSG0000	9073	2050

CLDN9	0.371931	0.407827	0.277284	0.344707	0.325762	0.261016	claudin 9 [Source:HGNC Symbol;Acc:HGNC:2051]	chr16:3062	NM_02098	.	.	.	NP_06619	.	protein_co	ENSG0000	9080	2051
CLDND1	166.524	154.338	176.522	209.908	209.014	262.011	claudin domain containing 1 [Source:HGNC Symbol;Acc:HGNC:13	chr3:9821	NM_00104	.	.	.	NP_00103	.	protein_co	ENSG0000	56650	1322
CLDND2	0.62867	1.62913	1.94997	0.22388	0.248576	0.231682	claudin domain containing 2 [Source:HGNC Symbol;Acc:HGNC:28	chr19:5187	NM_15235	XM_01152	.	.	NP_68956	XP_006723	protein_co	ENSG0000	125875	28511
CLEC10A	0	0	0	0	0	0	C-type lectin domain family 10 member A [Source:HGNC Symbol;	chr17:6977	NM_00634	XM_01152	.	.	NP_00633	XP_005256	protein_co	ENSG0000	10462	16916
CLEC11A	3.56511	4.54649	5.71339	7.80944	4.73421	6.08033	C-type lectin domain family 11 member A [Source:HGNC Symbol;	chr19:5122	NM_00297	.	.	.	NP_00296	.	protein_co	ENSG0000	6320	10576
CLEC12A	0	0	0	0	0	0	C-type lectin domain family 12 member A [Source:HGNC Symbol;	chr12:1010	NM_00120	XM_01152	.	.	NP_00119	XP_005253	protein_co	ENSG0000	160364	31713
CLEC12B	0	0	0	0	0	0	C-type lectin domain family 12 member B [Source:HGNC Symbol;	chr12:1014	NM_00112	XM_01152	.	.	NP_00112	XP_006719	protein_co	ENSG0000	387837	31966
CLEC14A	0	0	0	0	0	0	C-type lectin domain family 14 member A [Source:HGNC Symbol;	chr14:3872	NM_17506	.	.	.	NP_77823	.	protein_co	ENSG0000	161198	19832
CLEC16A	4.73458	4.45959	5.36419	4.46268	4.9323	4.91489	C-type lectin domain family 16 member A [Source:HGNC Symbol;	chr16:1103	NM_00124	XM_01152	.	.	NP_00123	XP_005255	protein_co	ENSG0000	23274	29013
CLEC17A	0	0.04551	0.081793	0.097342	0.051356	0	C-type lectin domain family 17 member A [Source:HGNC Symbol;	chr19:1469	NM_00120	XM_01152	.	.	NP_00119	.	protein_co	ENSG0000	388512	34520
CLEC18A	0	0.070372	0.071998	0	0.097264	0	C-type lectin domain family 18 member A [Source:HGNC Symbol;	chr16:6998	NM_00113	XM_01152	.	.	NP_00112	XP_005255	protein_co	ENSG0000	348174	30388
CLEC18B	0.335306	0.206141	0.07236	0.134444	0.048628	0.110494	C-type lectin domain family 18 member B [Source:HGNC Symbol;	chr16:7444	NM_00101	XM_01152	.	.	NP_00101	XP_005256	protein_co	ENSG0000	497190	33849
CLEC18C	0.080715	0	0	0	0	0	C-type lectin domain family 18 member C [Source:HGNC Symbol;	chr16:7020	NM_17361	XM_01152	.	.	NP_77589	XP_006721	protein_co	ENSG0000	283971	28538
CLEC19A	0	0	0	0	0	0	C-type lectin domain family 19 member A [Source:HGNC Symbol;	chr16:1929	NM_00125	XM_01154	.	.	NP_00124	XP_011544	protein_co	ENSG0000	728276	34522
CLEC1A	0	0	0.041208	0	0	0	C-type lectin domain family 1 member A [Source:HGNC Symbol;A	chr12:1022	NM_00129	XM_01152	.	.	NP_00128	XP_011518	protein_co	ENSG0000	51267	24355
CLEC1B	0	0	0	0	0	0	C-type lectin domain family 1 member B [Source:HGNC Symbol;A	chr12:1014	NM_00109	XM_01152	.	.	NP_00109	XP_005253	protein_co	ENSG0000	51266	24356
CLEC2A	0	0	0	0	0	0	C-type lectin domain family 2 member A [Source:HGNC Symbol;A	chr12:1005	NM_00113	XM_01152	.	.	NP_00112	XP_011518	protein_co	ENSG0000	387836	24191
CLEC2B	0.380674	0.059188	0.094589	0	0	0	C-type lectin domain family 2 member B [Source:HGNC Symbol;A	chr12:1000	NM_00512	.	.	.	NP_00511	.	protein_co	ENSG0000	9976	2053
CLEC2D	2.59849	2.44055	2.41359	2.42066	3.07948	3.02069	C-type lectin domain family 2 member D [Source:HGNC Symbol;A	chr12:9822	NM_00100	.	.	.	NP_00100	.	protein_co	ENSG0000	29121	14351
CLEC2L	0.071378	0	0	0	0	0	C-type lectin domain family 2 member L [Source:HGNC Symbol;A	chr7:13920	NM_00108	.	.	.	NP_00107	.	protein_co	ENSG0000	154790	21969
CLEC3A	0	0	0	0	0	0	C-type lectin domain family 3 member A [Source:HGNC Symbol;A	chr16:7805	NM_00575	.	.	.	NP_00574	.	protein_co	ENSG0000	10143	2052
CLEC3B	0.284939	0.575427	0.103125	0.406148	0.137441	0.131293	C-type lectin domain family 3 member B [Source:HGNC Symbol;A	chr3:45067	NM_00130	XM_00526	.	.	NP_00129	XP_005265	protein_co	ENSG0000	7123	11891
CLEC4A	0.820289	0.804239	0.574693	0.521206	0.313876	0.194106	C-type lectin domain family 4 member A [Source:HGNC Symbol;A	chr12:8276	NM_01618	.	.	.	NP_05726	.	protein_co	ENSG0000	50856	13257
CLEC4C	0	0	0	0.095508	0	0	C-type lectin domain family 4 member C [Source:HGNC Symbol;A	chr12:7882	NM_13044	.	.	.	NP_56970	.	protein_co	ENSG0000	170482	13258
CLEC4D	0	0	0	0	0	0	C-type lectin domain family 4 member D [Source:HGNC Symbol;A	chr12:8666	NM_08038	XM_01152	.	.	NP_52512	XP_011518	protein_co	ENSG0000	338339	14554
CLEC4E	0	0	0	0	0	0	C-type lectin domain family 4 member E [Source:HGNC Symbol;A	chr12:8685	NM_01435	XM_01152	.	.	NP_05517	XP_011518	protein_co	ENSG0000	26253	14555
CLEC4F	0	0.031564	0.026422	0	0	0.030606	C-type lectin domain family 4 member F [Source:HGNC Symbol;A	chr2:71035	NM_00125	XM_01153	.	.	NP_00124	XP_005264	protein_co	ENSG0000	165530	25357
CLEC4G	0	0	0	0	0	0	C-type lectin domain family 4 member G [Source:HGNC Symbol;A	chr19:7793	NM_00124	.	.	.	NP_00123	.	protein_co	ENSG0000	339390	24591
CLEC4M	0	0	0	0	0	0	C-type lectin domain family 4 member M [Source:HGNC Symbol;A	chr19:7828	NM_00114	XM_00672	.	.	NP_00113	XP_006722	protein_co	ENSG0000	10332	13523
CLEC5A	0	0	0	0	0	0	C-type lectin domain family 5 member A [Source:HGNC Symbol;A	chr7:14162	NM_00130	XM_01701	.	.	NP_00128	XP_016867	protein_co	ENSG0000	23601	2054
CLEC6A	0	0	0	0	0	0	C-type lectin domain family 6 member A [Source:HGNC Symbol;A	chr12:8608	NM_00100	.	.	.	NP_00100	.	protein_co	ENSG0000	93978	14556
CLEC7A	0	0	0	0	0	0	C-type lectin domain family 7 member A [Source:HGNC Symbol;A	chr12:1026	NM_02257	XM_00525	.	.	NP_07209	XP_005253	protein_co	ENSG0000	64581	14558
CLEC9A	0	0	0	0	0	0	C-type lectin domain family 9 member A [Source:HGNC Symbol;A	chr12:1018	NM_20734	.	.	.	NP_99722	.	protein_co	ENSG0000	283420	26705
CLECL1	0	0	0	0	0	0	C-type lectin like 1 [Source:HGNC Symbol;Acc:HGNC:24462]	chr12:9871	NM_00125	XM_01152	.	.	NP_00124	XP_011518	protein_co	ENSG0000	160365	24462
CLGN	1.46022	1.31184	1.07627	1.70404	2.07067	3.46065	calmegin [Source:HGNC Symbol;Acc:HGNC:2060]	chr4:14130	NM_00113	.	.	.	NP_00112	.	protein_co	ENSG0000	1047	2060
CLHC1	1.16935	0.824486	1.13158	0.514614	1.45655	1.60015	clathrin heavy chain linker domain containing 1 [Source:HGNC Sy	chr2:55402	NM_00113	XM_01153	.	.	NP_00112	XP_005264	protein_co	ENSG0000	130162	26453
CLIC1	388.063	378.036	287.466	408.517	304.09	321.205	chloride intracellular channel 1 [Source:HGNC Symbol;Acc:HGNC:	chr6:31694	NM_00128	.	.	.	NP_00127	.	protein_co	ENSG0000	1192	2062
CLIC2	0.369048	0.247134	0.198094	0.032811	0.093035	0.081436	chloride intracellular channel 2 [Source:HGNC Symbol;Acc:HGNC:	chrX:1545	NM_00128	.	.	.	NP_00128	.	protein_co	ENSG0000	1193	2063
CLIC3	6.34299	3.71506	5.04987	3.7514	2.46183	3.65399	chloride intracellular channel 3 [Source:HGNC Symbol;Acc:HGNC:	chr9:13988	NM_00466	.	.	.	NP_00466	XP_016870	protein_co	ENSG0000	9022	2064
CLIC4	26.5743	28.0485	27.4593	23.0554	27.8408	62.4307	chloride intracellular channel 4 [Source:HGNC Symbol;Acc:HGNC:	chr1:25071	NM_01394	.	.	.	NP_03923	.	protein_co	ENSG0000	25932	13518
CLIC5	0.046724	0.098288	0.122158	0.367016	0.519668	0.388799	chloride intracellular channel 5 [Source:HGNC Symbol;Acc:HGNC:	chr6:45866	NM_00111	XM_01701	.	.	NP_00110	XP_016866	protein_co	ENSG0000	53405	13517
CLIC6	0	0	0	0	0	0	chloride intracellular channel 6 [Source:HGNC Symbol;Acc:HGNC:	chr21:3604	NM_00131	XM_01152	.	.	NP_00130	XP_011527	protein_co	ENSG0000	54102	2065
CLINT1	32.7341	34.455	32.6864	32.9628	34.4497	26.2065	clathrin interactor 1 [Source:HGNC Symbol;Acc:HGNC:23186]	chr5:15721	NM_00119	XM_01153	.	.	NP_00118	XP_011533	protein_co	ENSG0000	9685	23186
CLIP1	13.1306	12.9365	10.712	10.4785	12.1357	12.9986	CAP-Gly domain containing linker protein 1 [Source:HGNC Symbo	chr12:1227	NM_00124	XM_01701	.	.	NP_00123	XP_005253	protein_co	ENSG0000	6249	10461
CLIP2	8.68247	9.83982	11.8391	11.0495	10.1891	11.2859	CAP-Gly domain containing linker protein 2 [Source:HGNC Symbo	chr7:73703	NM_00338	.	.	.	NP_00337	.	protein_co	ENSG0000	7461	2586
CLIP3	0.191511	0.19023	0.423015	0.230249	0.194913	0.29928	CAP-Gly domain containing linker protein 3 [Source:HGNC Symbo	chr19:3650	NM_00119	.	.	.	NP_00118	.	protein_co	ENSG0000	25999	24314
CLIP4	6.70627	6.75394	6.90345	4.13267	4.02245	8.78927	CAP-Gly domain containing linker protein family member 4 [Sourc	chr2:29320	NM_00128	XM_01700	.	.	NP_00127	XP_005264	protein_co	ENSG0000	79745	26108
CLK1	22.2866	18.3678	21.3514	15.1372	22.1741	23.4552	CDC like kinase 1 [Source:HGNC Symbol;Acc:HGNC:2068]	chr2:20171	NM_00116	.	NR_02785	.	NP_00115	.	protein_co	ENSG0000	1195	2068
CLK2	20.5872	20.0866	20.7078	17.7616	18.6097	18.4886	CDC like kinase 2 [Source:HGNC Symbol;Acc:HGNC:2069]	chr1:15523	NM_00129	XM_01150	.	.	NP_00128	XP_005244	protein_co	ENSG0000	1196	2069
CLK3	21.8852	23.2321	22.7875	21.8924	18.9204	16.6546	CDC like kinase 3 [Source:HGNC Symbol;Acc:HGNC:2071]	chr15:7490	NM_00113	XM_01152	.	.	NP_00112	XP_005254	protein_co	ENSG0000	1198	2071
CLK4	4.89755	3.93059	3.31157	3.09411	4.23411	3.6646	CDC like kinase 4 [Source:HGNC Symbol;Acc:HGNC:13659]	chr5:17802	NM_02066	XM_01153	.	.	NP_06571	XP_011532	protein_co	ENSG0000	57396	13659
CLLU1	0	0	0	0	0	0	chronic lymphocytic leukemia up-regulated 1 [Source:HGNC Symb	chr12:9281	NM_00102	.	NR_02793	.	NP_00102	.	protein_co	ENSG0000	574028	29841
CLLU1OS	0	0	0	0	0	0	chronic lymphocytic leukemia up-regulated 1 opposite strand [So	chr12:9281	NM_00102	XM_01153	.	.	NP_00102	XP_011536	protein_co	ENSG0000	574016	24070
CLMN	4.33511	3.7421	5.93635	1.68089	2.00973	3.26704	calmin [Source:HGNC Symbol;Acc:HGNC:19972]	chr14:9564	NM_02473	XM_01153	.	.	NP_07901	XP_011535	protein_co	ENSG0000	79789	19972
CLMP	0.713844	0.721113	0.60787	0.82098	0.74906	0.506464	CXADR like membrane protein [Source:HGNC Symbol;Acc:HGNC:2	chr11:1229	NM_02476	XM_01154	.	.	NP_07904	XP_011541	protein_co	ENSG0000	79827	24039
CLN3	18.9905	20.249	16.7368	19.6403	20.5162	12.0394	CLN3, battenin [Source:HGNC Symbol;Acc:HGNC:2074]	chr16:2848	NM_00008	.	.	.	NP_00007	.	protein_co	ENSG0000	1201	2074
CLN5	10.1893	10.1167	10.1967	9.52451	11.7859	10.9794	ceroid-lipofuscinosis, neuronal 5 [Source:HGNC Symbol;Acc:HGN	chr13:7756	NM_00649	XM_01153	.	.	NP_00648	XP_011533	protein_co	ENSG0000	1203	2076
CLN6	34.4305	34.8436	27.9992	36.432	34.1945	21.9158	ceroid-lipofuscinosis, neuronal 6, late infantile, variant [Source:H	chr15:6834	NM_01788	.	.	.	NP_06035	.	protein_co	ENSG0000	54982	2077
CLN8	8.43017	8.04682	11.3714	7.85208	11.2159	7.39184	ceroid-lipofuscinosis, neuronal 8 [Source:HGNC Symbol;Acc:HGN	chr8:17118	NM_01894	XM_01153	NR_03744	.	NP_06176	XP_005266	protein_co	ENSG0000	100500912	2079

CLNK	0.01709	0	0	0	0	0	cytokine dependent hematopoietic cell linker [Source:HGNC Sym	chr4:10488	NM_05296	XM_01151	.	.	NP_443196	XP_011512	protein_co	ENSG0000(	116449	17438
CLNS1A	88.3322	77.2938	95.4936	77.2079	79.7156	85.653	chloride nucleotide-sensitive channel 1A [Source:HGNC Symbol;A	chr11:7732	NM_00129	.	.	.	NP_00128	.	protein_co	ENSG0000(	1207	2080
CLOCK	6.92279	7.48725	9.49378	8.1257	11.3743	12.1253	clock circadian regulator [Source:HGNC Symbol;Acc:HGNC:2082]	chr4:56294	NM_00126	XM_01153	.	.	NP_00125	XP_005265	protein_co	ENSG0000(	9575	2082
CLP1	17.6531	18.0844	21.2358	22.0526	19.3736	15.2749	cleavage and polyadenylation factor I subunit 1 [Source:HGNC Sy	chr11:5742	NM_00114	.	.	.	NP_00113	.	protein_co	ENSG0000(	10978	16999
CLPB	10.5154	10.3113	9.83998	11.139	10.4312	9.02456	ClpB homolog, mitochondrial AAA ATPase chaperonin [Source:HG	chr11:7200	NM_00125	XM_01154	.	.	NP_00124	XP_005274	protein_co	ENSG0000(	81570	30664
CLPP	64.8656	66.1941	56.2021	78.4027	60.4331	51.5828	caseinolytic mitochondrial matrix peptidase proteolytic subunit [S	chr19:6361	NM_00601	.	.	.	NP_00600	.	protein_co	ENSG0000(	8192	2084
CLPS	0	0	0	0	0	0	colipase [Source:HGNC Symbol;Acc:HGNC:2085]	chr6:35762	NM_00125	.	.	.	NP_00123	.	protein_co	ENSG0000(	1208	2085
CLPSL1	0	0	0	0	0	0	colipase like 1 [Source:HGNC Symbol;Acc:HGNC:21251]	chr6:35748	NM_00101	.	.	.	NP_00101	.	protein_co	ENSG0000(	340204	21251
CLPSL2	0.486249	0	1.68075	0.346252	0	0.343308	colipase like 2 [Source:HGNC Symbol;Acc:HGNC:21250]	chr6:35744	NM_00128	.	NR_10446	.	NP_00127	.	protein_co	ENSG0000(	389383	21250
CLPTM1	43.9869	47.2344	37.4121	52.2563	46.2137	35.6138	CLPTM1, transmembrane protein [Source:HGNC Symbol;Acc:HGN	chr19:4545	NM_00128	.	.	.	NP_00126	.	protein_co	ENSG0000(	1209	2087
CLPTM1L	65.5935	69.311	54.044	71.3048	66.9208	54.7388	CLPTM1 like [Source:HGNC Symbol;Acc:HGNC:24308]	chr5:13178	NM_03078	XM_01151	.	.	NP_11040	XP_011512	protein_co	ENSG0000(	81037	24308
CLPX	17.459	18.819	17.2227	16.0945	17.6544	17.6084	caseinolytic mitochondrial matrix peptidase chaperone subunit [S	chr15:6544	NM_00666	XM_01152	.	.	NP_00665	XP_011519	protein_co	ENSG0000(	10845	2088
CLRN1	0	0	0	0	0	0	clarin 1 [Source:HGNC Symbol;Acc:HGNC:12605]	chr3:15064	NM_00119	.	.	.	NP_00118	.	protein_co	ENSG0000(	7401	12605
CLRN2	0	0	0	0	0	0	clarin 2 [Source:HGNC Symbol;Acc:HGNC:33939]	chr4:17516	NM_00107	.	.	.	NP_00107	.	protein_co	ENSG0000(	645104	33939
CLRN3	0	0.087035	0.143578	0	0.091671	0.24717	clarin 3 [Source:HGNC Symbol;Acc:HGNC:20795]	chr10:1296	NM_15231	.	.	.	NP_68952	.	protein_co	ENSG0000(	119467	20795
CLSPN	9.15329	10.2081	8.54635	10.6809	11.7115	9.37258	claspin [Source:HGNC Symbol;Acc:HGNC:19715]	chr1:36185	NM_00119	XM_01154	.	.	NP_00117	XP_011540	protein_co	ENSG0000(	63967	19715
CLSTN1	40.5859	42.2959	40.989	41.9892	38.9575	38.7748	calsyntenin 1 [Source:HGNC Symbol;Acc:HGNC:17447]	chr1:97117	NM_00100	.	.	.	NP_00100	.	protein_co	ENSG0000(	22883	17447
CLSTN2	0.047842	0.054958	0.079097	0.293369	0.35505	0.317509	calsyntenin 2 [Source:HGNC Symbol;Acc:HGNC:17448]	chr3:13965	NM_02213	XM_01700	.	.	NP_07141	XP_016862	protein_co	ENSG0000(	64084	17448
CLSTN3	7.76154	7.63703	9.32071	7.3583	7.87034	8.2913	calsyntenin 3 [Source:HGNC Symbol;Acc:HGNC:18371]	chr12:7282	NM_01471	XM_00671	.	.	NP_05553	XP_006719	protein_co	ENSG0000(	9746	18371
CLTA	110.422	109.434	106.301	155.309	124.34	107.045	clathrin light chain A [Source:HGNC Symbol;Acc:HGNC:2090]	chr9:36190	NM_00107	.	.	.	NP_00107	.	protein_co	ENSG0000(	1211	2090
CLTB	42.9747	39.4527	36.9983	49.3682	38.5972	31.2265	clathrin light chain B [Source:HGNC Symbol;Acc:HGNC:2091]	chr5:17581	NM_00183	.	.	.	NP_00182	XP_016864	protein_co	ENSG0000(	1212	2091
CLTC	161.291	168.287	173.652	179.042	198.186	191.816	clathrin heavy chain [Source:HGNC Symbol;Acc:HGNC:2092]	chr17:5769	NM_00128	XM_01152	.	.	NP_00127	XP_005257	protein_co	ENSG0000(	1213	2092
CLTCL1	1.38031	1.50188	1.85288	1.38585	1.57749	1.6272	clathrin heavy chain like 1 [Source:HGNC Symbol;Acc:HGNC:2093	chr22:1916	NM_00183	XM_01153	.	.	NP_00182	XP_016884	protein_co	ENSG0000(	8218	2093
CLU	66.6889	68.9478	49.0764	77.3355	65.272	45.4339	clusterin [Source:HGNC Symbol;Acc:HGNC:2095]	chr8:27454	NM_00183	XM_00671	NR_10690	.	NP_00182	XP_006716	protein_co	ENSG0000(	102465508	2095
CLUAP1	12.1353	9.8302	7.79623	10.4619	9.11318	9.50594	clusterin associated protein 1 [Source:HGNC Symbol;Acc:HGNC:1	chr16:3550	NM_01504	XM_00672	.	.	NP_05585	XP_006720	protein_co	ENSG0000(	23059	19009
CLUH	40.7795	41.924	42.4682	47.2132	42.6003	29.4065	clustered mitochondria homolog [Source:HGNC Symbol;Acc:HGN	chr17:2592	NM_01522	XM_01152	.	.	NP_05604	XP_011522	protein_co	ENSG0000(	23277	29094
CLUL1	0.432358	0.290734	0.271238	0.329247	0.23924	0.288246	clusterin like 1 [Source:HGNC Symbol;Acc:HGNC:2096]	chr18:5969	NM_00128	XM_01152	.	.	NP_00127	XP_005258	protein_co	ENSG0000(	27098	2096
CLVS1	0	0	0.023383	0.030511	0	0	clavesin 1 [Source:HGNC Symbol;Acc:HGNC:23139]	chr8:62200	NM_17351	XM_01701	.	.	NP_77579	XP_011515	protein_co	ENSG0000(	157807	23139
CLVS2	0	0	0	0	0	0	clavesin 2 [Source:HGNC Symbol;Acc:HGNC:23046]	chr6:12331	NM_00101	.	.	.	NP_00101	XP_016865	protein_co	ENSG0000(	134829	23046
CLYBL	8.71548	9.69283	14.3881	8.88903	7.48526	11.6035	citrate lyase beta like [Source:HGNC Symbol;Acc:HGNC:18355]	chr13:1002	NM_20680	XM_01152	.	.	NP_99653	XP_005254	protein_co	ENSG0000(	171425	18355
CMA1	0	0	0	0	0	0	chymase 1 [Source:HGNC Symbol;Acc:HGNC:2097]	chr14:2497	NM_00130	XM_01153	.	.	NP_00129	XP_011534	protein_co	ENSG0000(	1215	2097
CMAS	22.7887	26.0591	25.6377	21.6852	23.0453	25.9349	cytidine monophosphate N-acetylneuraminic acid synthetase [So	chr12:2219	NM_01868	.	.	.	NP_06115	.	protein_co	ENSG0000(	55907	18290
CMBL	36.2761	36.6902	40.4679	42.797	40.0208	31.3943	carboxymethylenebutenolidase homolog [Source:HGNC Symbol;A	chr5:10279	NM_13880	.	.	.	NP_62016	XP_016864	protein_co	ENSG0000(	134147	25090
CMC1	28.1194	24.3736	35.0897	34.8245	35.0923	38.0523	C-X9-C motif containing 1 [Source:HGNC Symbol;Acc:HGNC:2878	chr3:28283	NM_18252	.	.	.	NP_87232	.	protein_co	ENSG0000(	152100	28783
CMC2	77.4004	67.2641	77.3457	99.644	89.7638	94.2479	C-X9-C motif containing 2 [Source:HGNC Symbol;Acc:HGNC:2444	chr16:8100	NM_02018	.	.	.	NP_06457	XP_016878	protein_co	ENSG0000(	56942	24447
CMC4	11.0855	8.16518	10.1085	10.0572	10.7179	8.52481	C-X9-C motif containing 4 [Source:HGNC Symbol;Acc:HGNC:3542	chrX:15428	NM_00101	.	.	.	NP_00101	.	protein_co	ENSG0000(	100272147	35428
CMIP	10.7358	11.5729	12.9298	10.0602	9.23552	10.3594	c-Maf inducing protein [Source:HGNC Symbol;Acc:HGNC:24319]	chr16:8147	NM_03062	XM_01152	.	.	NP_08513	XP_005256	protein_co	ENSG0000(	80790	24319
CMKLR1	0	0	0.011216	0	0.042827	0.013326	chemerin chemokine-like receptor 1 [Source:HGNC Symbol;Acc:H	chr12:1086	NM_00114	XM_01701	.	.	NP_00113	XP_016874	protein_co	ENSG0000(	1240	2121
CMPK1	38.2008	40.5084	40.2107	41.6938	44.8535	42.7896	cytidine/uridine monophosphate kinase 1 [Source:HGNC Symbol;	chr1:47799	NM_00113	.	.	.	NP_00112	.	protein_co	ENSG0000(	51727	18170
CMPK2	1.67093	1.73423	0.69435	1.37552	1.35337	1.31868	cytidine/uridine monophosphate kinase 2 [Source:HGNC Symbol;	chr2:69807	NM_00125	.	NR_04623	.	NP_00124	.	protein_co	ENSG0000(	129607	27015
CMSS1	28.9326	28.7567	24.6321	35.5559	31.1699	28.6935	cms1 ribosomal small subunit homolog (yeast) [Source:HGNC Syn	chr3:99536	NM_00116	.	.	.	NP_00116	.	protein_co	ENSG0000(	84319	28666
CMTM1	1.49556	2.18759	1.34837	1.57707	1.0289	1.5424	CKLF like MARVEL transmembrane domain containing 1 [Source:H	chr16:6658	NM_05299	.	.	.	NP_44372	.	protein_co	ENSG0000(	113540	19172
CMTM2	0.166202	0	0.07278	0.247366	0.09645	0	CKLF like MARVEL transmembrane domain containing 2 [Source:H	chr16:6661	NM_00119	.	.	.	NP_00118	.	protein_co	ENSG0000(	146225	19173
CMTM3	17.52	21.2622	14.7736	19.8605	17.8553	14.594	CKLF like MARVEL transmembrane domain containing 3 [Source:H	chr16:6663	NM_14460	XM_01152	.	.	NP_65320	XP_006721	protein_co	ENSG0000(	123920	19174
CMTM4	37.2181	37.8809	40.5823	32.535	36.1007	42.4421	CKLF like MARVEL transmembrane domain containing 4 [Source:H	chr16:6664	NM_17881	XM_01152	.	.	NP_84893	XP_011521	protein_co	ENSG0000(	146223	19175
CMTM5	0	0	0	0	0	0	CKLF like MARVEL transmembrane domain containing 5 [Source:H	chr14:2384	NM_00103	.	.	.	NP_00103	XP_016876	protein_co	ENSG0000(	116173	19176
CMTM6	38.3334	37.8393	56.334	38.0187	47.6251	56.8633	CKLF like MARVEL transmembrane domain containing 6 [Source:H	chr3:32522	NM_01780	.	.	.	NP_06027	.	protein_co	ENSG0000(	54918	19177
CMTM7	48.4946	45.4128	70.0861	43.8886	37.8637	38.1487	CKLF like MARVEL transmembrane domain containing 7 [Source:H	chr3:32433	NM_13841	.	.	.	NP_61241	XP_011531	protein_co	ENSG0000(	112616	19178
CMTM8	19.1494	22.9577	16.8427	23.0784	20.4597	20.9271	CKLF like MARVEL transmembrane domain containing 8 [Source:H	chr3:32280	NM_00132	XM_01153	.	.	NP_00130	XP_011531	protein_co	ENSG0000(	152189	19179
CMTR1	12.0283	11.0769	11.2625	10.637	9.43978	8.26555	cap methyltransferase 1 [Source:HGNC Symbol;Acc:HGNC:21077]	chr6:37400	NM_01505	XM_00671	.	.	NP_05586	XP_005249	protein_co	ENSG0000(	23070	21077
CMTR2	10.9226	10.5641	12.3567	9.75689	12.0174	13.6829	cap methyltransferase 2 [Source:HGNC Symbol;Acc:HGNC:25635]	chr16:7131	NM_00109	XM_01152	.	.	NP_00109	XP_005256	protein_co	ENSG0000(	55783	25635
CMYA5	0.11054	0.145624	0.166882	0.055031	0.088445	0.110025	cardiomyopathy associated 5 [Source:HGNC Symbol;Acc:HGNC:1	chr5:78985	NM_15361	XM_01700	.	.	NP_70583	XP_016864	protein_co	ENSG0000(	202333	14305
CNBD1	0	0	0	0	0	0	cyclic nucleotide binding domain containing 1 [Source:HGNC Sym	chr8:87878	NM_17353	.	.	.	NP_77580	XP_016868	protein_co	ENSG0000(	168975	26663
CNBD2	0.155773	0.071277	0.060173	0.064364	0.149697	0.065153	cyclic nucleotide binding domain containing 2 [Source:HGNC Sym	chr20:3455	NM_00120	XM_01152	.	.	NP_00119	XP_005260	protein_co	ENSG0000(	140894	16145
CNBP	285.601	273.545	256.987	320.365	352.208	339.048	CCHC-type zinc finger nucleic acid binding protein [Source:HGNC	chr3:12888	NM_00112	.	.	.	NP_00112	.	protein_co	ENSG0000(	7555	13164
CNDP1	0.131114	0.033841	0.028441	0	0	0.033633	carnosine dipeptidase 1 [Source:HGNC Symbol;Acc:HGNC:20675]	chr18:7220	NM_03264	.	.	.	NP_11603	.	protein_co	ENSG0000(	84735	20675
CNDP2	37.3259	38.0723	32.3219	40.9825	36.0095	30.5698	CNDP dipeptidase 2 (metallopeptidase M20 family) [Source:HGN	chr18:7216	NM_00116	XM_01152	.	.	NP_00116	XP_005266	protein_co	ENSG0000(	55748	24437
CNEP1R1	8.92041	7.83681	7.11484	6.70706	8.52337	9.78364	CTD nuclear envelope phosphatase 1 regulatory subunit 1 [Sourc	chr16:5005	NM_00128	.	.	.	NP_00126	.	protein_co	ENSG0000(	255919	26759
CNFN	22.3167	18.0179	27.3247	20.084	13.0451	19.0439	cornifelin [Source:HGNC Symbol;Acc:HGNC:30183]	chr19:4289	NM_03248	XM_01152	.	.	NP_11587	XP_005259	protein_co	ENSG0000(	84518	30183

CNGA1	0.119578	0.195416	0.63025	0.256208	0.369482	0.395888	cyclic nucleotide gated channel alpha 1 [Source:HGNC Symbol;Ac	chr4:47937	NM_00008	XM_01700	.	.	NP_000078	XP_005248	protein_co	ENSG0000(	1259	2148
CNGA2	0	0	0	0	0	0	cyclic nucleotide gated channel alpha 2 [Source:HGNC Symbol;Ac	chrX:15090	NM_00514	.	.	.	NP_005131	.	protein_co	ENSG0000(	1260	2149
CNGA3	0	0	0	0	0	0	cyclic nucleotide gated channel alpha 3 [Source:HGNC Symbol;Ac	chr2:98962	NM_00107	XM_01151	.	.	NP_001073	XP_006712	protein_co	ENSG0000(	1261	2150
CNGA4	0.153962	0.080155	0.16564	0.041603	0.044439	0.124465	cyclic nucleotide gated channel alpha 4 [Source:HGNC Symbol;Ac	chr11:6255	NM_00103	.	.	.	NP_001032	XP_016872	protein_co	ENSG0000(	1262	2152
CNGB1	0.011461	0.029252	0.029965	0.037263	0.052844	0	cyclic nucleotide gated channel beta 1 [Source:HGNC Symbol;Acc	chr16:5791	NM_00113	XM_00672	.	.	NP_001129	XP_006721	protein_co	ENSG0000(	1258	2151
CNGB3	0	0	0	0	0	0	cyclic nucleotide gated channel beta 3 [Source:HGNC Symbol;Acc	chr8:87586	NM_01909	XM_01151	.	.	NP_061971	XP_011515	protein_co	ENSG0000(	54714	2153
CNIH1	112.667	113.012	100.771	123.465	130.24	136.427	cornichon family AMPA receptor auxiliary protein 1 [Source:HGN(	chr14:5489	NM_00577	.	.	.	NP_005767	.	protein_co	ENSG0000(	10175	19431
CNIH2	0.167087	0.327558	0.569935	0.712825	0.775363	0.741327	cornichon family AMPA receptor auxiliary protein 2 [Source:HGN(	chr11:6604	NM_18255	.	.	.	NP_872359	.	protein_co	ENSG0000(	254263	28744
CNIH3	0.141943	0.036142	0.060732	0.038771	0.120763	0.073818	cornichon family AMPA receptor auxiliary protein 3 [Source:HGN(	chr1:22480	NM_15249	.	.	.	NP_001309	.	protein_co	ENSG0000(	149111	26802
CNIH4	22.0436	19.3294	19.5437	22.4778	26.8406	27.0332	cornichon family AMPA receptor auxiliary protein 4 [Source:HGN(	chr1:22454	NM_00127	.	.	.	NP_001264	.	protein_co	ENSG0000(	29097	25013
CNKSR1	9.52518	9.68957	8.04088	9.1445	7.79888	6.79826	connector enhancer of kinase suppressor of Ras 1 [Source:HGNC	chr1:26503	NM_00129	XM_01154	.	.	NP_001284	XP_006710	protein_co	ENSG0000(	10256	19700
CNKSR2	0	0	0	0	0	0	connector enhancer of kinase suppressor of Ras 2 [Source:HGNC	chrX:21392	NM_00116	XM_01154	.	.	NP_001162	XP_011543	protein_co	ENSG0000(	22866	19701
CNKSR3	4.6873	5.40731	4.43331	5.89845	4.51316	5.19794	CNKSR family member 3 [Source:HGNC Symbol;Acc:HGNC:23034]	chr6:15470	NM_17351	XM_01153	.	.	NP_775786	XP_011533	protein_co	ENSG0000(	154043	23034
CNN1	0	0.042194	0	0.13727	0.049763	0	calponin 1 [Source:HGNC Symbol;Acc:HGNC:2155]	chr19:1164	NM_00129	XM_01152	.	.	NP_001290	XP_005259	protein_co	ENSG0000(	1264	2155
CNN2	93.5051	91.1528	92.3932	112.927	94.9496	100.22	calponin 2 [Source:HGNC Symbol;Acc:HGNC:2156]	chr19:1026	NM_00130	.	.	.	NP_001290	.	protein_co	ENSG0000(	1265	2156
CNN3	61.9833	61.9352	72.946	94.2985	88.0028	92.1659	calponin 3 [Source:HGNC Symbol;Acc:HGNC:2157]	chr1:95362	NM_00128	.	.	.	NP_001272	XP_016855	protein_co	ENSG0000(	1266	2157
CNNM1	0	0	0	0	0	0	cyclin and CBS domain divalent metal cation transport mediator 1	chr10:1010	NM_02034	XM_01153	.	.	NP_065081	XP_005269	protein_co	ENSG0000(	26507	102
CNNM2	6.20948	6.20965	7.99874	5.68163	6.12149	7.58616	cyclin and CBS domain divalent metal cation transport mediator 2	chr10:1046	NM_01764	.	.	.	NP_060119	XP_005269	protein_co	ENSG0000(	54805	103
CNNM3	12.0831	11.9872	12.4223	9.50956	10.8535	8.38084	cyclin and CBS domain divalent metal cation transport mediator 3	chr2:97481	NM_01762	.	.	.	NP_060093	XP_011509	protein_co	ENSG0000(	26505	104
CNNM4	4.1261	4.18783	3.92538	3.56535	3.97964	4.67036	cyclin and CBS domain divalent metal cation transport mediator 4	chr2:97426	NM_02018	XM_00526	.	.	NP_064569	XP_005263	protein_co	ENSG0000(	26504	105
CNOT1	48.9073	47.7748	48.982	50.3151	52.4942	63.1947	CCR4-NOT transcription complex subunit 1 [Source:HGNC Symbol	chr16:5854	NM_00126	.	.	.	NP_001252	.	protein_co	ENSG0000(	23019	7877
CNOT10	22.2107	19.516	19.4309	22.8828	25.2376	23.5891	CCR4-NOT transcription complex subunit 10 [Source:HGNC Symb	chr3:32726	NM_00125	XM_01700	.	.	NP_001243	XP_006713	protein_co	ENSG0000(	25904	23817
CNOT11	38.2822	37.1401	43.0162	39.7234	40.3079	38.1986	CCR4-NOT transcription complex subunit 11 [Source:HGNC Symb	chr2:10186	NM_01754	.	.	.	NP_060016	.	protein_co	ENSG0000(	55571	25217
CNOT2	19.5343	17.8088	19.194	15.3355	17.8437	21.1579	CCR4-NOT transcription complex subunit 2 [Source:HGNC Symbol	chr12:7063	NM_00119	XM_01153	.	.	NP_001186	XP_006719	protein_co	ENSG0000(	4848	7878
CNOT3	19.4821	19.6225	16.578	22.1482	19.6184	17.5396	CCR4-NOT transcription complex subunit 3 [Source:HGNC Symbol	chr19:5464	NM_01451	XM_01152	.	.	NP_055331	XP_005278	protein_co	ENSG0000(	4849	7879
CNOT4	8.13454	8.76626	6.4942	8.92416	8.52204	7.39835	CCR4-NOT transcription complex subunit 4 [Source:HGNC Symbol	chr7:13504	NM_00100	XM_01701	.	.	NP_001008	XP_011514	protein_co	ENSG0000(	4850	7880
CNOT6	21.1974	21.8815	20.9445	16.6309	20.2077	29.513	CCR4-NOT transcription complex subunit 6 [Source:HGNC Symbol	chr5:17992	NM_00130	XM_01700	.	.	NP_001290	XP_005266	protein_co	ENSG0000(	57472	14099
CNOT6L	3.93102	4.07959	4.41363	3.63852	4.65882	5.05235	CCR4-NOT transcription complex subunit 6 like [Source:HGNC Syr	chr4:78634	NM_00128	XM_01700	.	.	NP_001273	XP_011530	protein_co	ENSG0000(	246175	18042
CNOT7	35.5883	36.9903	36.6244	43.4202	48.412	50.7329	CCR4-NOT transcription complex subunit 7 [Source:HGNC Symbol	chr8:17082	NM_01335	XM_01154	.	.	NP_001309	XP_005273	protein_co	ENSG0000(	29883	14101
CNOT8	26.6316	25.7389	27.1017	23.2842	29.1722	32.2585	CCR4-NOT transcription complex subunit 8 [Source:HGNC Symbol	chr5:15419	NM_00130	XM_01701	.	.	NP_001288	XP_005268	protein_co	ENSG0000(	9337	9207
CNOT9	46.4253	43.2311	37.9044	46.6084	44.6907	43.1371	CCR4-NOT transcription complex subunit 9 [Source:HGNC Symbol	chr2:21943	NM_00127	XM_01151	.	.	NP_001258	XP_011510	protein_co	ENSG0000(	9125	10445
CNP	40.5789	39.0853	40.7776	47.7669	44.0063	35.7812	2',3'-cyclic nucleotide 3' phosphodiesterase [Source:HGNC Symbo	chr17:4011	NM_03313	XM_01152	.	.	NP_149124	XP_006721	protein_co	ENSG0000(	1267	2158
CNPPD1	33.6493	34.4513	32.1504	20.0537	18.8436	21.994	cyclin Pas1/PHO80 domain containing 1 [Source:HGNC Symbol;Ac	chr2:22003	NM_00132	XM_00671	.	.	NP_001308	XP_005246	protein_co	ENSG0000(	27013	25220
CNPY1	0	0	0.046253	0.060518	0	0.051589	canopy FGF signaling regulator 1 [Source:HGNC Symbol;Acc:HGN(	chr7:15526	NM_00110	.	.	.	NP_001096	.	protein_co	ENSG0000(	285888	27786
CNPY2	75.8333	74.4443	53.9758	111.101	83.6934	59.8521	canopy FGF signaling regulator 2 [Source:HGNC Symbol;Acc:HGN(	chr12:5666	NM_01425	.	.	.	NP_055070	.	protein_co	ENSG0000(	10330	13529
CNPY3	41.5817	39.0519	28.153	44.5929	35.5963	30.0928	canopy FGF signaling regulator 3 [Source:HGNC Symbol;Acc:HGN(	chr6:42896	NM_00658	XM_00524	.	.	NP_006577	.	protein_co	ENSG0000(	10695	11968
CNPY4	2.46178	2.02945	1.99491	1.6725	1.09972	0.489786	canopy FGF signaling regulator 4 [Source:HGNC Symbol;Acc:HGN(	chr7:99690	NM_15275	.	.	.	NP_689968	.	protein_co	ENSG0000(	245812	28631
CNR1	0	0	0	0	0	0	cannabinoid receptor 1 [Source:HGNC Symbol;Acc:HGNC:2159]	chr6:88849	NM_00116	XM_01701	.	.	NP_001153	XP_005248	protein_co	ENSG0000(	1268	2159
CNR2	0.014413	0.015071	0	0.080391	0.032265	0.088245	cannabinoid receptor 2 [Source:HGNC Symbol;Acc:HGNC:2160]	chr1:24197	NM_00184	XM_01154	.	.	NP_001832	XP_005245	protein_co	ENSG0000(	1269	2160
CNRIP1	0	0	0	0	0	0	cannabinoid receptor interacting protein 1 [Source:HGNC Symbol	chr2:68511	NM_00111	XM_00671	.	.	NP_001104	XP_005264	protein_co	ENSG0000(	25927	24546
CNST	5.22429	6.41794	6.27225	5.06071	5.67386	5.83232	consortin, connexin sorting protein [Source:HGNC Symbol;Acc:H(	chr1:24672	NM_00113	XM_01154	.	.	NP_001132	XP_005273	protein_co	ENSG0000(	163882	26486
CNTD1	0.062319	0	0	0	0	0.033644	cyclin N-terminal domain containing 1 [Source:HGNC Symbol;Acc	chr17:4095	NM_17347	XM_01152	.	.	NP_775749	XP_005257	protein_co	ENSG0000(	124817	26847
CNTD2	10.4323	11.2139	2.70804	11.3137	6.78388	5.55709	cyclin N-terminal domain containing 2 [Source:HGNC Symbol;Acc	chr19:4072	NM_02487	.	.	.	NP_079153	.	protein_co	ENSG0000(	79935	25805
CNTF	1.33614	1.46013	1.28015	0.831387	1.49119	0.873012	ciliary neurotrophic factor [Source:HGNC Symbol;Acc:HGNC:2169	chr11:5839	NM_00061	.	.	.	NP_000605	.	protein_co	ENSG0000(	1270	2169
CNTFR	0	0	0	0	0.055431	0.053286	ciliary neurotrophic factor receptor [Source:HGNC Symbol;Acc:H(	chr9:34551	NM_00120	XM_01701	.	.	NP_001193	XP_016869	protein_co	ENSG0000(	1271	2170
CNTLN	1.44424	1.16847	1.37147	0.953556	1.64172	1.88898	centlein [Source:HGNC Symbol;Acc:HGNC:23432]	chr9:17134	NM_00111	XM_01151	.	.	NP_001107	XP_005251	protein_co	ENSG0000(	54875	23432
CNTN1	0.683722	0.967149	1.68081	0.705922	0.847759	1.3555	contactin 1 [Source:HGNC Symbol;Acc:HGNC:2171]	chr12:4108	NM_00125	XM_01153	.	.	NP_001242	XP_005268	protein_co	ENSG0000(	1272	2171
CNTN2	0.017245	0.034865	0.007472	0.018788	0.009867	0.018426	contactin 2 [Source:HGNC Symbol;Acc:HGNC:2172]	chr1:20501	NM_00507	XM_01150	.	.	NP_005067	.	protein_co	ENSG0000(	6900	2172
CNTN3	0	0	0	0	0	0	contactin 3 [Source:HGNC Symbol;Acc:HGNC:2173]	chr3:74311	NM_02087	XM_01700	.	.	NP_065923	XP_005264	protein_co	ENSG0000(	5067	2173
CNTN4	0	0	0	0	0	0	contactin 4 [Source:HGNC Symbol;Acc:HGNC:2174]	chr3:21406	NM_00120	XM_01153	.	.	NP_001193	XP_006713	protein_co	ENSG0000(	152330	2174
CNTN5	0	0	0	0	0	0	contactin 5 [Source:HGNC Symbol;Acc:HGNC:2175]	chr11:9889	NM_00124	XM_01701	.	.	NP_001230	XP_011541	protein_co	ENSG0000(	53942	2175
CNTN6	0	0	0	0	0	0	contactin 6 [Source:HGNC Symbol;Acc:HGNC:2176]	chr3:11342	NM_00128	XM_01153	.	.	NP_001276	XP_005265	protein_co	ENSG0000(	27255	2176
CNTNAP1	6.94445	6.68868	6.27019	3.28212	2.84895	2.25772	contactin associated protein 1 [Source:HGNC Symbol;Acc:HGNC:8	chr17:4083	NM_00363	XM_00525	.	.	NP_003623	XP_005257	protein_co	ENSG0000(	8506	8011
CNTNAP2	0.190106	0.289909	0.282588	0.773248	1.0865	0.840151	contactin associated protein-like 2 [Source:HGNC Symbol;Acc:HG	chr7:14581	NM_01414	.	.	.	NP_054860	.	protein_co	ENSG0000(	26047	13830
CNTNAP3	1.45811	1.347	2.98355	0.86478	0.976335	1.3816	contactin associated protein-like 3 [Source:HGNC Symbol;Acc:HG	chr9:39072	NM_03365	XM_01151	.	.	NP_387504	.	protein_co	ENSG0000(	79937	13834
CNTNAP3B	1.35818	1.00533	2.27598	0.72818	1.03455	1.03309	contactin associated protein-like 3B [Source:HGNC Symbol;Acc:H(	chr9:43684	NM_00120	XM_01151	.	.	NP_001188	XP_011543	protein_co	ENSG0000(	100289279	32035
CNTNAP4	0	0	0	0	0	0	contactin associated protein like 4 [Source:HGNC Symbol;Acc:HG	chr16:7631	NM_03340	XM_01152	.	.	NP_001309	XP_011521	protein_co	ENSG0000(	85445	18747
CNTNAP5	0	0	0	0	0	0	contactin associated protein like 5 [Source:HGNC Symbol;Acc:HG	chr2:12478	NM_13077	XM_00671	.	.	NP_570129	XP_006712	protein_co	ENSG0000(	129684	18748
CNTRL	3.22219	2.7084	3.26144	3.63332	4.96502	5.26588	centriolin [Source:HGNC Symbol;Acc:HGNC:1858]	chr9:12383	NM_00701	XM_01701	.	.	NP_008949	XP_005251	protein_co	ENSG0000(	11064	1858

CNTROB	14.5545	14.2844	13.4956	18.1275	15.497	12.97	centrobin, centriole duplication and spindle assembly protein [So	chr17:7835	NM_00103	XM_01702	.	.	NP_00103	XP_00525	protein_co	ENSG0000	116840	29616
COA1	59.5138	61.2147	50.9492	69.6179	49.2859	50.9373	cytochrome c oxidase assembly factor 1 homolog [Source:HGNC	chr7:4367	NM_00132	XM_01701	.	.	NP_00130	XP_00524	protein_co	ENSG0000	55744	21868
COA3	49.9662	45.8081	40.0614	50.0429	38.8033	24.5321	cytochrome c oxidase assembly factor 3 [Source:HGNC Symbol;A	chr17:4094	NM_00104	.	.	.	NP_00103	.	protein_co	ENSG0000	28958	24990
COA4	77.9918	77.1195	62.1229	103.704	85.2695	66.144	cytochrome c oxidase assembly factor 4 homolog [Source:HGNC	chr11:7358	NM_01656	.	.	.	NP_05764	XP_01687	protein_co	ENSG0000	51287	24604
COA5	13.4774	14.7225	11.5	15.5972	13.8509	13.3862	cytochrome c oxidase assembly factor 5 [Source:HGNC Symbol;A	chr2:9921	NM_00100	.	.	.	NP_00100	.	protein_co	ENSG0000	493753	33848
COA6	29.3137	26.2483	23.3369	29.2899	25.4607	25.0044	cytochrome c oxidase assembly factor 6 [Source:HGNC Symbol;A	chr1:2345	NM_00101	.	.	.	NP_00101	.	protein_co	ENSG0000	388753	18025
COA7	18.7358	18.141	17.5011	17.6688	20.1434	14.5812	cytochrome c oxidase assembly factor 7 (putative) [Source:HGNC	chr1:5315	NM_02307	.	.	.	NP_07556	.	protein_co	ENSG0000	65260	25716
COASY	46.546	47.284	46.6148	34.3202	31.9306	31.7807	Coenzyme A synthase [Source:HGNC Symbol;Acc:HGNC:29932]	chr17:4071	NM_00104	XM_01152	.	.	NP_00103	XP_00672	protein_co	ENSG0000	80347	29932
COBL	6.48098	6.65963	9.4802	6.33185	6.31683	6.51351	cordon-bleu WH2 repeat protein [Source:HGNC Symbol;Acc:HGN	chr7:5108	NM_00128	XM_01701	.	.	NP_00127	XP_00527	protein_co	ENSG0000	23242	22199
COBLL1	1.41133	1.57366	1.68112	1.20158	1.19828	2.08552	cordon-bleu WH2 repeat protein like 1 [Source:HGNC Symbol;Acc	chr2:1655	NM_00127	.	.	.	NP_00126	.	protein_co	ENSG0000	22837	23571
COCH	3.7188	4.02221	3.91006	7.44532	8.31316	6.76091	cochlin [Source:HGNC Symbol;Acc:HGNC:2180]	chr14:3134	NM_00113	XM_01153	.	.	NP_00112	XP_01153	protein_co	ENSG0000	1690	2180
COG1	16.849	16.8915	14.6589	14.0894	11.8066	11.5794	component of oligomeric golgi complex 1 [Source:HGNC Symbol;	chr17:7118	NM_01871	.	.	.	NP_06118	.	protein_co	ENSG0000	9382	6545
COG2	14.3702	14.1034	15.5924	16.1935	14.7427	14.9416	component of oligomeric golgi complex 2 [Source:HGNC Symbol;	chr1:2307	NM_00114	.	.	.	NP_00113	.	protein_co	ENSG0000	22796	6546
COG3	8.69667	8.87126	8.74148	7.42655	8.69525	8.80815	component of oligomeric golgi complex 3 [Source:HGNC Symbol;	chr13:4603	NM_03143	XM_01153	.	.	NP_11361	XP_01153	protein_co	ENSG0000	83548	18619
COG4	17.3643	17.1255	17.9494	22.3217	18.3065	19.7082	component of oligomeric golgi complex 4 [Source:HGNC Symbol;	chr16:7051	NM_00119	XM_01152	.	.	NP_00118	XP_01152	protein_co	ENSG0000	25839	18620
COG5	8.01755	8.63666	10.1868	7.74827	8.60174	12.903	component of oligomeric golgi complex 5 [Source:HGNC Symbol;	chr7:1068	NM_00116	.	.	.	NP_00115	.	protein_co	ENSG0000	10466	14857
COG6	7.62704	8.35245	8.84891	6.65155	7.35223	9.36791	component of oligomeric golgi complex 6 [Source:HGNC Symbol;	chr13:4022	NM_00114	XM_01153	.	.	NP_00113	XP_01153	protein_co	ENSG0000	57511	18621
COG7	5.67378	6.3143	6.60434	6.48257	5.12006	6.64853	component of oligomeric golgi complex 7 [Source:HGNC Symbol;	chr16:2339	NM_1536	.	.	.	NP_70583	XP_01687	protein_co	ENSG0000	91949	18622
COG8	19.8617	22.3289	22.3232	23.2188	22.2689	20.867	component of oligomeric golgi complex 8 [Source:HGNC Symbol;	chr16:693	.	.	.	.	.	.	protein_co	ENSG0000	.	18623
COIL	23.6461	24.8298	21.6107	25.2471	23.1357	20.6601	coilin [Source:HGNC Symbol;Acc:HGNC:2184]	chr17:5501	NM_00464	.	.	.	NP_00463	.	protein_co	ENSG0000	8161	2184
COL10A1	0	0	0	0	0	0	collagen type X alpha 1 chain [Source:HGNC Symbol;Acc:HGNC:2	chr6:1164	NM_00049	XM_01153	.	.	NP_00048	XP_00671	protein_co	ENSG0000	1300	2185
COL11A1	0	0	0.017137	0	0	0.010264	collagen type XI alpha 1 chain [Source:HGNC Symbol;Acc:HGNC:2	chr1:1033	NM_00119	XM_01154	.	.	NP_00117	.	protein_co	ENSG0000	1301	2186
COL11A2	1.39397	1.43658	1.78763	1.0259	0.963527	1.32069	collagen type XI alpha 2 chain [Source:HGNC Symbol;Acc:HGNC:2	chr6:3313	NM_00116	XM_01701	.	.	NP_00115	XP_011512	protein_co	ENSG0000	1302	2187
COL12A1	6.78897	8.01115	9.92928	4.59601	5.0326	5.36152	collagen type XII alpha 1 chain [Source:HGNC Symbol;Acc:HGNC:2	chr6:75794	NM_00437	XM_01153	.	.	NP_00436	XP_01153	protein_co	ENSG0000	1303	2188
COL13A1	1.17373	1.02308	2.89534	0.653427	1.16327	1.58593	collagen type XIII alpha 1 chain [Source:HGNC Symbol;Acc:HGNC:	chr10:715	NM_00113	XM_01153	.	.	NP_00112	XP_01153	protein_co	ENSG0000	1305	2190
COL14A1	0.018788	0.019509	0.016447	0.011048	0.123524	0.077469	collagen type XIV alpha 1 chain [Source:HGNC Symbol;Acc:HGNC:	chr8:1211	NM_02111	XM_00671	.	.	NP_06693	XP_005251	protein_co	ENSG0000	7373	2191
COL15A1	0.01247	0.025166	0.021765	0.013596	0.014242	0.053095	collagen type XV alpha 1 chain [Source:HGNC Symbol;Acc:HGNC:	chr9:1017	NM_00185	XM_01151	.	.	NP_00184	XP_01151	protein_co	ENSG0000	1306	2192
COL16A1	0.537656	0.392648	0.694322	0.471884	0.458112	0.348245	collagen type XVI alpha 1 chain [Source:HGNC Symbol;Acc:HGNC:	chr1:3211	NM_00185	XM_01154	.	.	NP_00184	XP_00527	protein_co	ENSG0000	1307	2193
COL17A1	0.755527	0.720655	0.881647	2.20734	2.57054	1.29466	collagen type XVII alpha 1 chain [Source:HGNC Symbol;Acc:HGNC	chr10:1057	NM_00049	.	NR_03076	.	NP_00048	.	protein_co	ENSG0000	10012632	2194
COL18A1	21.104	21.7307	32.3009	19.7457	18.8052	21.3006	collagen type XVIII alpha 1 chain [Source:HGNC Symbol;Acc:HGN	chr21:4682	NM_03058	.	.	.	NP_08505	.	protein_co	ENSG0000	80781	2195
COL19A1	0	0	0	0	0	0	collagen type XIX alpha 1 chain [Source:HGNC Symbol;Acc:HGNC:	chr6:7057	NM_00185	XM_01153	.	.	NP_00184	XP_01153	protein_co	ENSG0000	1310	2196
COL1A1	3.17581	2.97176	3.6113	2.32292	1.86499	2.17747	collagen type I alpha 1 chain [Source:HGNC Symbol;Acc:HGNC:21	chr17:482	NM_00008	XM_01152	.	.	NP_00007	XP_005257	protein_co	ENSG0000	1277	2197
COL1A2	0	0	0	0	0	0	collagen type I alpha 2 chain [Source:HGNC Symbol;Acc:HGNC:21	chr7:9402	NM_00008	.	.	.	NP_00008	.	protein_co	ENSG0000	1278	2198
COL20A1	0.018801	0.019461	0.016193	0	0	0.020855	collagen type XX alpha 1 chain [Source:HGNC Symbol;Acc:HGNC:	chr20:619	NM_02088	XM_01152	.	.	NP_06593	XP_01152	protein_co	ENSG0000	57642	14670
COL21A1	0.04847	0.016794	0.049695	0	0.018186	0	collagen type XXI alpha 1 chain [Source:HGNC Symbol;Acc:HGNC:	chr6:5592	NM_00131	XM_01151	.	.	NP_00130	XP_00671	protein_co	ENSG0000	81578	17025
COL22A1	0	0	0	0	0	0	collagen type XXII alpha 1 chain [Source:HGNC Symbol;Acc:HGNC	chr8:1396	NM_15288	XM_01151	.	.	NP_69084	XP_01151	protein_co	ENSG0000	169044	22989
COL23A1	0	0	0	0	0	0	collagen type XXIII alpha 1 chain [Source:HGNC Symbol;Acc:HGN	chr5:1776	NM_17346	XM_01153	.	.	NP_77573	XP_00671	protein_co	ENSG0000	91522	22990
COL24A1	0.008458	0.008777	0.022439	0	0.038257	0.00866	collagen type XXIV alpha 1 chain [Source:HGNC Symbol;Acc:HGN	chr1:8619	NM_15289	XM_01154	.	.	NP_69085	XP_01153	protein_co	ENSG0000	255631	20821
COL25A1	0.014155	0.014648	0.098558	0.015847	0.023571	0.021773	collagen type XXV alpha 1 chain [Source:HGNC Symbol;Acc:HGN	chr4:1097	NM_00125	XM_01153	.	.	NP_00124	XP_01153	protein_co	ENSG0000	84570	18603
COL26A1	0.294985	0.183387	0.138814	0.313814	0.281188	0.480099	collagen type XXVI alpha 1 chain [Source:HGNC Symbol;Acc:HGN	chr7:1010	NM_00127	.	.	.	NP_00126	XP_01686	protein_co	ENSG0000	136227	18038
COL27A1	2.19793	2.18953	3.05214	2.09337	2.41724	1.4612	collagen type XXVII alpha 1 chain [Source:HGNC Symbol;Acc:HGN	chr9:1169	NM_03288	XM_01151	.	.	NP_11627	XP_00671	protein_co	ENSG0000	85301	22986
COL28A1	0.037558	0.025871	0	0	0.028354	0	collagen type XXVIII alpha 1 chain [Source:HGNC Symbol;Acc:HGN	chr7:7397	NM_00103	XM_01151	.	.	NP_00103	XP_011513	protein_co	ENSG0000	340267	22442
COL2A1	0.031226	0.015249	0.013516	0.016555	0.018252	0.017412	collagen type II alpha 1 chain [Source:HGNC Symbol;Acc:HGNC:2	chr12:483	NM_00184	XM_01701	.	.	NP_00183	XP_01687	protein_co	ENSG0000	1280	2200
COL3A1	0.022367	0.030047	0.009758	0.01221	0	0	collagen type III alpha 1 chain [Source:HGNC Symbol;Acc:HGNC:2	chr2:1898	NM_00009	.	NR_03740	.	NP_00008	.	protein_co	ENSG0000	10050083	2201
COL4A1	0.009778	0.009765	0.017185	0.021265	0.078851	0.021048	collagen type IV alpha 1 chain [Source:HGNC Symbol;Acc:HGNC:2	chr13:110	NM_00130	XM_01152	.	.	NP_00129	XP_01151	protein_co	ENSG0000	1282	2202
COL4A2	0.01987	0	0.008702	0.01062	0.023167	0.011054	collagen type IV alpha 2 chain [Source:HGNC Symbol;Acc:HGNC:2	chr13:110	NM_00184	.	.	.	NP_00183	.	protein_co	ENSG0000	1284	2203
COL4A3	0.687815	0.724827	0.943457	0.403714	0.365128	0.539666	collagen type IV alpha 3 chain [Source:HGNC Symbol;Acc:HGNC:2	chr2:2280	NM_00009	XM_01151	.	.	NP_00008	XP_00524	protein_co	ENSG0000	1285	2204
COL4A3BP	13.0585	13.3461	9.30591	10.2865	11.9398	12.0762	collagen type IV alpha 3 binding protein [Source:HGNC Symbol;A	chr5:7466	NM_00113	XM_01700	.	.	NP_00112	XP_00671	protein_co	ENSG0000	10087	2205
COL4A4	1.05755	1.01702	1.32654	0.898988	1.00537	1.26043	collagen type IV alpha 4 chain [Source:HGNC Symbol;Acc:HGNC:2	chr2:2278	NM_00009	XM_01151	.	.	NP_00008	XP_00524	protein_co	ENSG0000	1286	2206
COL4A5	3.29513	3.07534	4.34498	3.14127	3.05581	3.85815	collagen type IV alpha 5 chain [Source:HGNC Symbol;Acc:HGNC:2	chrX:1076	NM_00049	XM_01702	.	.	NP_00048	XP_00672	protein_co	ENSG0000	1287	2207
COL4A6	0.371954	0.354915	0.465572	0.478287	0.490113	0.62812	collagen type IV alpha 6 chain [Source:HGNC Symbol;Acc:HGNC:2	chrX:1073	NM_00128	XM_01153	.	.	NP_00127	XP_00672	protein_co	ENSG0000	1288	2208
COL5A1	0.008851	0.00864	0.030803	0.047378	0.020537	0.039328	collagen type V alpha 1 chain [Source:HGNC Symbol;Acc:HGNC:2	chr9:1375	NM_00009	.	.	.	NP_00008	XP_01686	protein_co	ENSG0000	1289	2209
COL5A2	0.365057	0.482672	0.479395	0.52	0.505648	0.476083	collagen type V alpha 2 chain [Source:HGNC Symbol;Acc:HGNC:2	chr2:1898	NM_00039	XM_01151	.	.	NP_00038	XP_011508	protein_co	ENSG0000	1290	2210
COL5A3	0.02135	0.04158	0.055921	0.022728	0.024873	0.04769	collagen type V alpha 3 chain [Source:HGNC Symbol;Acc:HGNC:1	chr19:100	NM_01571	XM_01152	.	.	NP_05653	XP_01152	protein_co	ENSG0000	50509	14864
COL6A1	21.1842	23.0336	31.0051	15.7948	16.305	17.8274	collagen type VI alpha 1 chain [Source:HGNC Symbol;Acc:HGNC:2	chr21:474	NM_00184	.	.	.	NP_00183	.	protein_co	ENSG0000	1291	2211
COL6A2	28.8319	32.1059	37.6972	26.1752	24.1995	29.2524	collagen type VI alpha 2 chain [Source:HGNC Symbol;Acc:HGNC:2	chr21:475	NM_00184	XM_01152	.	.	NP_00184	XP_01152	protein_co	ENSG0000	1292	2212
COL6A3	0.091162	0.036847	0.078328	0.006868	0.032082	0.059398	collagen type VI alpha 3 chain [Source:HGNC Symbol;Acc:HGNC:2	chr2:2382	NM_00436	XM_01700	.	.	NP_00436	XP_00524	protein_co	ENSG0000	1293	2213
COL6A5	0	0	0	0	0	0	collagen type VI alpha 5 chain [Source:HGNC Symbol;Acc:HGNC:2	chr3:1300	NM_00127	XM_01151	.	.	NP_00126	XP_01151	protein_co	ENSG0000	256076	26674
COL6A6	0	0	0	0	0	0	collagen type VI alpha 6 chain [Source:HGNC Symbol;Acc:HGNC:2	chr3:1302	NM_00110	XM_01700	.	.	NP_00109	XP_00524	protein_co	ENSG0000	131873	27023

COL7A1	0.632736	0.619292	0.929044	0.389607	0.526203	0.658617	collagen type VII alpha 1 chain [Source:HGNC Symbol;Acc:HGNC:	chr3:48601	NM_00009	XM_01153	NR_03175	.	NP_00008	XP_011531	protein_co	ENSG0000	100313843	2214
COL8A1	0	0	0	0	0	0	collagen type VIII alpha 1 chain [Source:HGNC Symbol;Acc:HGNC:	chr3:99357	NM_00185	.	.	.	NP_00184	.	protein_co	ENSG0000	1295	2215
COL8A2	0	0	0.055307	0	0	0	collagen type VIII alpha 2 chain [Source:HGNC Symbol;Acc:HGNC:	chr1:36560	NM_00129	.	.	.	NP_00128	XP_005270	protein_co	ENSG0000	1296	2216
COL9A1	0	0	0	0	0	0	collagen type IX alpha 1 chain [Source:HGNC Symbol;Acc:HGNC:2	chr6:70924	NM_00185	XM_01153	.	.	NP_00184	XP_011533	protein_co	ENSG0000	1297	2217
COL9A2	1.92795	1.73354	2.41432	1.75043	1.29157	1.55036	collagen type IX alpha 2 chain [Source:HGNC Symbol;Acc:HGNC:2	chr1:40766	NM_00185	XM_01700	.	.	NP_00184	XP_006710	protein_co	ENSG0000	1298	2218
COL9A3	9.22127	8.16433	11.4771	3.56004	3.93265	3.68517	collagen type IX alpha 3 chain [Source:HGNC Symbol;Acc:HGNC:2	chr20:6144	NM_00185	XM_01152	.	.	NP_00184	XP_011526	protein_co	ENSG0000	1299	2219
COLCA2	0	0	0	0	0	0	colorectal cancer associated 2 [Source:HGNC Symbol;Acc:HGNC:2	chr11:1111	NM_00113	XM_01154	.	.	NP_00112	XP_016872	protein_co	ENSG0000	120376	26978
COLEC10	0	0	0	0	0	0	collectin subfamily member 10 [Source:HGNC Symbol;Acc:HGNC:	chr8:12007	NM_00643	.	.	.	NP_00642	.	protein_co	ENSG0000	10584	2220
COLEC11	0.050782	0.233991	0.165357	0	0.084382	0.18562	collectin subfamily member 11 [Source:HGNC Symbol;Acc:HGNC:	chr2:36424	NM_00125	XM_00671	.	.	NP_00124	XP_005263	protein_co	ENSG0000	78989	17213
COLEC12	0.025514	0	0	0.014126	0	0.052676	collectin subfamily member 12 [Source:HGNC Symbol;Acc:HGNC:	chr18:3167	NM_13038	XM_01152	.	.	NP_56905	XP_011524	protein_co	ENSG0000	81035	16016
COLGALT1	57.7455	56.9897	49.9205	50.3851	47.6738	44.5721	collagen beta(1-O)galactosyltransferase 1 [Source:HGNC Symbol;	chr19:1766	NM_02465	XM_01152	.	.	NP_07893	XP_005260	protein_co	ENSG0000	79709	26182
COLGALT2	0.060643	0.073458	0.053855	0.019607	0.038761	0.086866	collagen beta(1-O)galactosyltransferase 2 [Source:HGNC Symbol;	chr1:18390	NM_00130	XM_01150	.	.	NP_00129	XP_011507	protein_co	ENSG0000	23127	16790
COLQ	1.37269	1.19994	1.56226	0.854646	0.715316	1.26377	collagen like tail subunit of asymmetric acetylcholinesterase [Sou	chr3:15491	NM_00567	.	.	.	NP_00566	.	protein_co	ENSG0000	8292	2226
COMMD1	14.3344	14.0323	15.159	16.4922	14.6394	14.8642	copper metabolism domain containing 1 [Source:HGNC Symbol;A	chr2:62132	NM_15251	.	.	.	NP_68972	.	protein_co	ENSG0000	150684	23024
COMMD10	9.90551	10.652	11.087	11.8737	11.8869	14.7815	COMM domain containing 10 [Source:HGNC Symbol;Acc:HGNC:3	chr5:11542	NM_01614	XM_01154	.	.	NP_00129	XP_011541	protein_co	ENSG0000	51397	30201
COMMD2	9.7094	8.86394	11.0932	10.6601	11.2981	13.0267	COMM domain containing 2 [Source:HGNC Symbol;Acc:HGNC:24	chr3:14945	NM_01609	.	.	.	NP_05717	.	protein_co	ENSG0000	51122	24993
COMMD3	34.7442	32.863	28.7561	35.705	35.0833	33.2644	COMM domain containing 3 [Source:HGNC Symbol;Acc:HGNC:23	chr10:2260	NM_01207	.	.	.	NP_03620	.	protein_co	ENSG0000	23412	23332
COMMD3-BMI1	5.96201	9.77569	7.32118	9.4162	9.76277	6.66882	COMMD3-BMI1 readthrough [Source:HGNC Symbol;Acc:HGNC:4	chr10:2260	NM_00120	.	.	.	NP_00119	.	protein_co	ENSG0000	100532731	48326
COMMD4	104.856	104.181	78.5144	134.087	114.245	72.5464	COMM domain containing 4 [Source:HGNC Symbol;Acc:HGNC:26	chr15:7562	NM_00128	XM_01152	.	.	NP_00127	XP_005254	protein_co	ENSG0000	54939	26027
COMMD5	32.5163	31.8526	29.0277	37.3273	29.7386	23.7088	COMM domain containing 5 [Source:HGNC Symbol;Acc:HGNC:17	chr8:14607	NM_00108	.	.	.	NP_00107	.	protein_co	ENSG0000	28991	17902
COMMD6	56.0325	55.4232	75.006	74.4877	54.7165	69.3765	COMM domain containing 6 [Source:HGNC Symbol;Acc:HGNC:24	chr13:7609	NM_00128	XM_01153	.	.	NP_00127	XP_011533	protein_co	ENSG0000	170622	24015
COMMD7	11.9405	13.3159	13.2808	17.4653	14.9863	12.8346	COMM domain containing 7 [Source:HGNC Symbol;Acc:HGNC:16	chr20:3129	NM_00109	.	.	.	NP_00109	.	protein_co	ENSG0000	149951	16223
COMMD8	12.5228	12.6976	13.424	13.4195	15.051	16.0366	COMM domain containing 8 [Source:HGNC Symbol;Acc:HGNC:26	chr4:47452	NM_00132	XM_00671	.	.	NP_00131	XP_016863	protein_co	ENSG0000	54951	26036
COMMD9	18.5603	17.0299	12.5601	19.1178	16.0842	9.06158	COMM domain containing 9 [Source:HGNC Symbol;Acc:HGNC:25	chr11:3629	NM_00110	XM_01152	.	.	NP_00109	XP_011518	protein_co	ENSG0000	29099	25014
COMP	0	0	0	0.031316	0	0	cartilage oligomeric matrix protein [Source:HGNC Symbol;Acc:HG	chr19:1889	NM_00009	.	.	.	NP_00008	.	protein_co	ENSG0000	1311	2227
COMT	47.95	49.9013	45.07	59.271	46.9193	40.8222	catechol-O-methyltransferase [Source:HGNC Symbol;Acc:HGNC:2	chr22:1986	NM_00075	XM_01702	NR_03991	.	NP_00074	XP_016884	protein_co	ENSG0000	100616414	2228
COMTD1	21.4593	19.6287	21.0392	20.456	14.7737	20.3546	catechol-O-methyltransferase domain containing 1 [Source:HGN	chr10:7699	NM_14458	.	.	.	NP_65319	.	protein_co	ENSG0000	118881	26309
COPA	49.0312	49.8869	44.5897	46.0231	45.2654	48.5935	coatomer protein complex subunit alpha [Source:HGNC Symbol;A	chr1:16025	NM_00109	.	.	.	NP_00109	.	protein_co	ENSG0000	1314	2230
COPB1	48.8329	43.3556	46.7275	43.0486	51.3527	58.2462	coatomer protein complex subunit beta 1 [Source:HGNC Symbol;	chr11:1447	NM_00114	.	.	.	NP_00113	.	protein_co	ENSG0000	1315	2231
COPB2	65.2876	68.2047	68.8799	56.2588	55.8731	61.4883	coatomer protein complex subunit beta 2 [Source:HGNC Symbol;	chr3:13907	NM_00476	XM_01151	.	.	NP_00475	XP_016863	protein_co	ENSG0000	9276	2232
COPE	110.199	111.272	102.152	124.675	92.8477	98.8197	coatomer protein complex subunit epsilon [Source:HGNC Symbol	chr19:1901	NM_00726	XM_01152	.	.	NP_00919	XP_011525	protein_co	ENSG0000	11316	2234
COPG1	67.4556	63.4857	59.3252	60.1934	55.5874	58.0499	coatomer protein complex subunit gamma 1 [Source:HGNC Symb	chr3:12896	NM_01612	.	.	.	NP_05721	.	protein_co	ENSG0000	22820	2236
COPG2	11.7256	11.126	11.6452	11.9753	10.2235	15.7868	coatomer protein complex subunit gamma 2 [Source:HGNC Symb	chr7:13029	NM_00129	.	.	.	NP_00127	.	protein_co	ENSG0000	26958	2237
COPRS	51.3376	46.3984	44.8299	63.0161	51.2329	45.0493	coordinator of PRMT5 and differentiation stimulator [Source:HG	chr17:3017	NM_01840	XM_00525	.	.	NP_06087	XP_005258	protein_co	ENSG0000	55352	28848
COPS2	39.853	35.5424	41.2739	39.0046	47.6132	48.2339	COP9 signalosome subunit 2 [Source:HGNC Symbol;Acc:HGNC:30	chr15:4941	NM_00114	.	.	.	NP_00113	.	protein_co	ENSG0000	9318	30747
COPS3	54.4466	55.4252	48.3226	64.5511	59.9603	54.5834	COP9 signalosome subunit 3 [Source:HGNC Symbol;Acc:HGNC:22	chr17:1715	NM_00119	XM_00525	.	.	NP_00118	XP_005256	protein_co	ENSG0000	8533	2239
COPS4	22.3598	21.4955	21.8084	27.3054	23.635	23.6052	COP9 signalosome subunit 4 [Source:HGNC Symbol;Acc:HGNC:16	chr4:83955	NM_00125	XM_01153	.	.	NP_00124	XP_011530	protein_co	ENSG0000	51138	16702
COPS5	68.6432	63.5457	51.2223	62.2455	62.3801	47.6796	COP9 signalosome subunit 5 [Source:HGNC Symbol;Acc:HGNC:22	chr8:67955	NM_00683	.	.	.	NP_00682	.	protein_co	ENSG0000	10987	2240
COPS6	94.6405	84.3176	79.9094	107.254	88.135	71.427	COP9 signalosome subunit 6 [Source:HGNC Symbol;Acc:HGNC:21	chr7:99686	NM_00683	.	.	.	NP_00682	.	protein_co	ENSG0000	10980	21749
COPS7A	57.5893	56.7968	48.6229	66.3092	56.7342	44.1285	COP9 signalosome subunit 7A [Source:HGNC Symbol;Acc:HGNC:1	chr12:6832	NM_00116	XM_00525	.	.	NP_00115	XP_005253	protein_co	ENSG0000	50813	16758
COPS7B	24.4394	26.7639	24.0571	27.9326	26.289	22.8479	COP9 signalosome subunit 7B [Source:HGNC Symbol;Acc:HGNC:1	chr2:23264	NM_00128	XM_01700	.	.	NP_00126	XP_006712	protein_co	ENSG0000	64708	16760
COPS8	38.4964	36.0631	32.2127	44.9685	46.7119	37.3241	COP9 signalosome subunit 8 [Source:HGNC Symbol;Acc:HGNC:24	chr2:23799	NM_00671	.	.	.	NP_00670	.	protein_co	ENSG0000	10920	24335
COPS9	158.812	143.526	132.75	198.693	171.468	140.802	COP9 signalosome subunit 9 [Source:HGNC Symbol;Acc:HGNC:21	chr2:24106	NM_00116	.	.	.	NP_00115	XP_016858	protein_co	ENSG0000	150678	21314
COPZ1	172.031	159.975	160.806	194.316	162.762	173.508	coatomer protein complex subunit zeta 1 [Source:HGNC Symbol;	chr12:5471	NM_00127	XM_01153	.	.	NP_00125	.	protein_co	ENSG0000	22818	2243
COPZ2	0.254135	0	0.146821	0	0	0	coatomer protein complex subunit zeta 2 [Source:HGNC Symbol;	chr17:4610	NM_01642	XM_01152	.	.	NP_05751	XP_011523	protein_co	ENSG0000	51226	19356
COQ10A	7.1273	6.95213	6.4821	5.68434	5.53972	4.86889	coenzyme Q10A [Source:HGNC Symbol;Acc:HGNC:26515]	chr12:5666	NM_00109	.	.	.	NP_00109	.	protein_co	ENSG0000	93058	26515
COQ10B	14.2744	13.2485	15.5612	14.7111	16.8932	19.7601	coenzyme Q10B [Source:HGNC Symbol;Acc:HGNC:25819]	chr2:19831	NM_02514	XM_00671	.	.	NP_00130	XP_006712	protein_co	ENSG0000	80219	25819
COQ2	14.3594	14.9667	12.1526	14.5815	12.3027	11.722	coenzyme Q2, polyprenyltransferase [Source:HGNC Symbol;Acc:	chr4:84184	NM_01569	.	.	.	NP_05651	.	protein_co	ENSG0000	27235	25223
COQ3	17.5767	15.0738	15.7975	21.2755	15.2175	19.2896	coenzyme Q3, methyltransferase [Source:HGNC Symbol;Acc:HGN	chr6:99817	NM_01742	XM_00671	.	.	NP_05911	XP_005267	protein_co	ENSG0000	51805	18175
COQ4	35.3706	38.378	33.9001	40.1062	33.3032	29.4575	coenzyme Q4 [Source:HGNC Symbol;Acc:HGNC:19693]	chr9:13106	NM_01603	.	.	.	NP_05711	.	protein_co	ENSG0000	51117	19693
COQ5	26.0996	21.5313	29.4422	25.9611	23.9216	29.5636	coenzyme Q5, methyltransferase [Source:HGNC Symbol;Acc:HGN	chr12:1209	NM_03231	XM_00671	.	.	NP_11569	XP_006719	protein_co	ENSG0000	84274	28722
COQ6	10.6287	12.6201	12.7708	12.6021	11.7664	10.4956	coenzyme Q6, monooxygenase [Source:HGNC Symbol;Acc:HGNC:	chr14:7431	NM_18247	XM_01153	.	.	NP_87228	XP_006720	protein_co	ENSG0000	51004	20233
COQ7	11.2283	12.1089	9.83581	14.6474	16.2265	15.0782	coenzyme Q7, hydroxylase [Source:HGNC Symbol;Acc:HGNC:224	chr16:1907	NM_00119	.	.	.	NP_00117	.	protein_co	ENSG0000	10229	2244
COQ9	36.0314	36.081	33.3436	46.5223	37.5619	33.0783	coenzyme Q9 [Source:HGNC Symbol;Acc:HGNC:25302]	chr16:5746	NM_02031	.	.	.	NP_06470	.	protein_co	ENSG0000	57017	25302
CORIN	0.673881	0.921491	0.714276	0.358148	0.21871	0.1072	corin, serine peptidase [Source:HGNC Symbol;Acc:HGNC:19012]	chr4:47596	NM_00127	.	.	.	NP_00126	.	protein_co	ENSG0000	10699	19012
CORO1A	3.24953	4.07721	4.83252	5.68537	5.15483	3.0938	coronin 1A [Source:HGNC Symbol;Acc:HGNC:2252]	chr16:3019	NM_00119	XM_01154	.	.	NP_00118	XP_011544	protein_co	ENSG0000	11151	2252
CORO1B	57.5444	57.1022	56.8724	51.7312	42.9664	46.9518	coronin 1B [Source:HGNC Symbol;Acc:HGNC:2253]	chr11:6720	NM_00101	.	.	.	NP_00101	.	protein_co	ENSG0000	57175	2253
CORO1C	43.9898	44.0542	47.0773	54.9123	53.1932	57.0425	coronin 1C [Source:HGNC Symbol;Acc:HGNC:2254]	chr12:1090	NM_00110	.	.	.	NP_00109	XP_016874	protein_co	ENSG0000	23603	2254
CORO2A	4.53084	4.61739	5.63204	3.76156	3.93347	4.22735	coronin 2A [Source:HGNC Symbol;Acc:HGNC:2255]	chr9:10088	NM_00338	XM_01151	.	.	NP_00338	XP_011517	protein_co	ENSG0000	7464	2255

CORO2B	1.37196	1.29352	1.6309	1.25569	0.841446	1.32145	coronin 2B [Source:HGNC Symbol;Acc:HGNC:2256]	chr15:6887	NM_00119	XM_01152	.	.	NP_00117	XP_005254	protein_co	ENSG0000	10391	2256
CORO6	2.92366	2.57507	3.99338	2.56828	3.30488	2.45698	coronin 6 [Source:HGNC Symbol;Acc:HGNC:21356]	chr17:2792	NM_03285	XM_01152	.	.	NP_11624	XP_005258	protein_co	ENSG0000	84940	21356
CORO7	3.72395	4.03062	4.81073	4.27398	3.10583	3.72	coronin 7 [Source:HGNC Symbol;Acc:HGNC:26161]	chr16:4364	NM_00120	.	.	.	NP_00118	.	protein_co	ENSG0000	79585	26161
CORO7-PAM16	5.53E-07	1.3E-15	6.44E-06	1.19E-29	1.71E-10	2.08E-07	CORO7-PAM16 readthrough [Source:HGNC Symbol;Acc:HGNC:44	chr16:4364	NM_00120	.	.	.	NP_00118	.	protein_co	ENSG0000	10052914	44424
CORT	0.099293	0.094504	0.230316	0.351948	5.12E-36	1.99E-32	cortistatin [Source:HGNC Symbol;Acc:HGNC:2257]	chr1:10490	NM_00130	.	.	.	NP_00129	.	protein_co	ENSG0000	1325	2257
COTL1	165.712	164.765	159.776	174.418	150.53	165.749	coactosin like F-actin binding protein 1 [Source:HGNC Symbol;Acc	chr16:8459	NM_02114	.	.	.	NP_06697	.	protein_co	ENSG0000	23406	18304
COX10	13.3331	12.9197	13.4264	16.0809	15.356	10.4993	COX10, heme A:farnesyltransferase cytochrome c oxidase assemb	chr17:1397	NM_00130	XM_01152	.	.	NP_00129	.	protein_co	ENSG0000	1352	2260
COX11	22.4689	23.2388	24.0357	22.7239	18.561	16.2968	COX11, cytochrome c oxidase copper chaperone [Source:HGNC S	chr17:5297	NM_00116	XM_01152	.	.	NP_00115	XP_00672	protein_co	ENSG0000	1353	2261
COX14	40.8053	34.6138	40.0955	47.5265	39.5916	39.8683	COX14, cytochrome c oxidase assembly factor [Source:HGNC Sym	chr12:5050	NM_00125	XM_01153	.	.	NP_00124	XP_011537	protein_co	ENSG0000	84987	28216
COX15	42.8314	41.9187	46.2377	55.1282	56.5283	56.0078	COX15, cytochrome c oxidase assembly homolog [Source:HGNC S	chr10:1014	NM_00437	XM_00671	.	.	NP_00130	.	protein_co	ENSG0000	1355	2263
COX16	32.1147	28.9377	26.6869	39.8693	30.7382	30.5517	COX16, cytochrome c oxidase assembly homolog [Source:HGNC S	chr14:7079	NM_01646	.	.	.	NP_05755	.	protein_co	ENSG0000	51241	20213
COX17	68.3742	67.5894	56.8122	106.187	72.2957	70.3249	COX17, cytochrome c oxidase copper chaperone [Source:HGNC S	chr3:11938	NM_00569	.	.	.	NP_00568	.	protein_co	ENSG0000	10063	2264
COX18	5.26721	4.89648	4.43884	5.00889	4.85805	5.62446	COX18, cytochrome c oxidase assembly factor [Source:HGNC Sym	chr4:73918	NM_00129	XM_01153	.	.	NP_00128	XP_005265	protein_co	ENSG0000	285521	26801
COX19	5.14208	4.80174	4.7675	5.16287	5.12848	5.52242	COX19, cytochrome c oxidase assembly factor [Source:HGNC Sym	chr7:10057	NM_00103	.	.	.	NP_00102	.	protein_co	ENSG0000	90639	28074
COX20	41.2934	33.5509	45.5039	44.6163	58.0451	50.4966	COX20, cytochrome c oxidase assembly factor [Source:HGNC Sym	chr1:24499	NM_00131	.	NR_13242	.	NP_00129	.	protein_co	ENSG0000	116228	26970
COX4I1	692.614	634.733	674.457	785.525	587.917	608.152	cytochrome c oxidase subunit 4I1 [Source:HGNC Symbol;Acc:HGN	chr16:8583	NM_00131	XM_01152	.	.	NP_00130	XP_005255	protein_co	ENSG0000	1327	2265
COX4I2	0	0	0	0	0	0	cytochrome c oxidase subunit 4I2 [Source:HGNC Symbol;Acc:HGN	chr20:3022	NM_03260	.	.	.	NP_11599	.	protein_co	ENSG0000	84701	16232
COX5A	216.976	213.284	220.848	277.023	220.964	232.907	cytochrome c oxidase subunit 5A [Source:HGNC Symbol;Acc:HGN	chr15:7521	NM_00425	.	.	.	NP_00424	.	protein_co	ENSG0000	9377	2267
COX5B	248.764	235.822	252.582	272.199	196.648	236.879	cytochrome c oxidase subunit 5B [Source:HGNC Symbol;Acc:HGN	chr2:98262	NM_00186	.	.	.	NP_00185	.	protein_co	ENSG0000	1329	2269
COX6A1	378.127	349.364	380.49	457.215	325.864	382.696	cytochrome c oxidase subunit 6A1 [Source:HGNC Symbol;Acc:HG	chr12:1208	NM_00437	.	.	.	NP_00436	.	protein_co	ENSG0000	1337	2277
COX6A2	0	0	0	0	0	0	cytochrome c oxidase subunit 6A2 [Source:HGNC Symbol;Acc:HG	chr16:3143	NM_00520	.	.	.	NP_00519	.	protein_co	ENSG0000	1339	2279
COX6B1	299.701	250.459	251.016	364.636	253.275	287.476	cytochrome c oxidase subunit 6B1 [Source:HGNC Symbol;Acc:HG	chr19:3613	NM_00186	.	.	.	NP_00185	.	protein_co	ENSG0000	1340	2280
COX6B2	0.503198	0.676335	0.965113	0.336564	0.442297	0.425312	cytochrome c oxidase subunit 6B2 [Source:HGNC Symbol;Acc:HG	chr19:5586	NM_14461	.	.	.	NP_65321	.	protein_co	ENSG0000	125965	24380
COX6C	353.398	301.235	375.478	378.31	322.023	385.138	cytochrome c oxidase subunit 6C [Source:HGNC Symbol;Acc:HGN	chr8:10089	NM_00437	XM_01701	.	.	NP_00436	XP_016868	protein_co	ENSG0000	1345	2285
COX7A1	0	0	0	0	0	0	cytochrome c oxidase subunit 7A1 [Source:HGNC Symbol;Acc:HG	chr19:3664	NM_00186	.	.	.	NP_00185	.	protein_co	ENSG0000	1346	2287
COX7A2	303.906	273.068	295.554	314.674	270.367	332.698	cytochrome c oxidase subunit 7A2 [Source:HGNC Symbol;Acc:HG	chr6:75947	NM_00186	XM_01153	.	.	NP_00185	XP_011533	protein_co	ENSG0000	1347	2288
COX7A2L	97.6678	97.8918	107.233	111.535	102.7	127.735	cytochrome c oxidase subunit 7A2 like [Source:HGNC Symbol;Acc	chr2:42576	NM_00471	XM_01153	NR_134948	.	NP_00130	XP_011531	protein_co	ENSG0000	9167	2289
COX7B	76.4502	61.0636	58.3056	88.4709	59.2197	61.5733	cytochrome c oxidase subunit 7B [Source:HGNC Symbol;Acc:HGN	chrX:77154	NM_00186	.	.	.	NP_00185	.	protein_co	ENSG0000	1349	2291
COX7B2	0	0	0	0	0	0	cytochrome c oxidase subunit 7B2 [Source:HGNC Symbol;Acc:HG	chr4:46736	NM_13090	XM_01151	.	.	NP_57097	XP_005248	protein_co	ENSG0000	170712	24381
COX7C	606.502	533.192	634.328	638.328	535.434	661.591	cytochrome c oxidase subunit 7C [Source:HGNC Symbol;Acc:HGN	chr5:85913	NM_00186	.	NR_03740	.	NP_00185	.	protein_co	ENSG0000	100500805	2292
COX8A	504.698	514.965	457.687	638.913	466.874	466.924	cytochrome c oxidase subunit 8A [Source:HGNC Symbol;Acc:HGN	chr11:6374	NM_00407	.	.	.	NP_00406	.	protein_co	ENSG0000	1351	2294
COX8C	0	0	0	0.294127	0	0	cytochrome c oxidase subunit 8C [Source:HGNC Symbol;Acc:HGN	chr14:9379	NM_18297	.	.	.	NP_89201	.	protein_co	ENSG0000	341947	24382
CP	0.0273	0.01464	0.011972	0.0156	0	0	ceruloplasmin [Source:HGNC Symbol;Acc:HGNC:2295]	chr3:14884	NM_00009	XM_01151	.	.	NP_00008	XP_006713	protein_co	ENSG0000	1356	2295
CPA1	0	0	0	0	0	0	carboxypeptidase A1 [Source:HGNC Symbol;Acc:HGNC:2296]	chr7:13002	NM_00186	.	.	.	NP_00185	.	protein_co	ENSG0000	1357	2296
CPA2	0	0	0	0.05395	0	0.099274	carboxypeptidase A2 [Source:HGNC Symbol;Acc:HGNC:2297]	chr7:12990	NM_00186	.	.	.	NP_00186	.	protein_co	ENSG0000	1358	2297
CPA3	0	0	0	0	0	0	carboxypeptidase A3 [Source:HGNC Symbol;Acc:HGNC:2298]	chr3:14858	NM_00187	.	.	.	NP_00186	.	protein_co	ENSG0000	1359	2298
CPA4	5.91985	5.742	8.7166	0.988937	1.37547	0.662848	carboxypeptidase A4 [Source:HGNC Symbol;Acc:HGNC:15740]	chr7:12993	NM_00116	.	.	.	NP_00115	.	protein_co	ENSG0000	51200	15740
CPA5	0.052297	0.159723	0.135112	0.057297	0.059412	0	carboxypeptidase A5 [Source:HGNC Symbol;Acc:HGNC:15722]	chr7:12998	NM_00112	XM_01151	.	.	NP_00112	XP_005250	protein_co	ENSG0000	93979	15722
CPA6	0	0	0	0	0	0	carboxypeptidase A6 [Source:HGNC Symbol;Acc:HGNC:17245]	chr8:68334	NM_02036	XM_01151	.	.	NP_06509	XP_011515	protein_co	ENSG0000	57094	17245
CPAMD8	0.014202	0.013976	0.012197	0	0.016497	0	C3 and PZP like, alpha-2-macroglobulin domain containing 8 [Sou	chr19:1700	NM_01569	XM_01702	.	.	NP_05650	XP_011526	protein_co	ENSG0000	27151	23228
CPB1	0	0	0	0	0	0	carboxypeptidase B1 [Source:HGNC Symbol;Acc:HGNC:2299]	chr3:14850	NM_00187	XM_01151	.	.	NP_00186	XP_005247	protein_co	ENSG0000	1360	2299
CPB2	0	0	0	0	0	0	carboxypeptidase B2 [Source:HGNC Symbol;Acc:HGNC:2300]	chr13:4662	NM_00127	XM_01702	.	.	NP_00126	XP_016875	protein_co	ENSG0000	1361	2300
CPD	17.1217	18.1635	18.7193	17.2805	22.1118	21.7388	carboxypeptidase D [Source:HGNC Symbol;Acc:HGNC:2301]	chr17:2870	NM_00119	.	.	.	NP_00118	.	protein_co	ENSG0000	1362	2301
CPE	23.3173	24.5588	34.6688	28.4647	24.6842	34.2734	carboxypeptidase E [Source:HGNC Symbol;Acc:HGNC:2303]	chr4:16630	NM_00187	.	.	.	NP_00186	.	protein_co	ENSG0000	1363	2303
CPEB1	0	0	0	0	0	0	cytoplasmic polyadenylation element binding protein 1 [Source:H	chr15:8320	NM_00107	.	.	.	NP_00107	.	protein_co	ENSG0000	64506	21744
CPEB2	1.49001	1.86361	1.85398	0.570757	0.597164	1.07658	cytoplasmic polyadenylation element binding protein 2 [Source:H	chr4:15004	NM_00117	XM_01151	.	.	NP_00117	XP_005248	protein_co	ENSG0000	132864	21745
CPEB3	0.739313	1.04634	0.872485	0.471248	0.780635	1.17855	cytoplasmic polyadenylation element binding protein 3 [Source:H	chr10:9380	NM_00117	XM_01153	.	.	NP_00117	XP_005269	protein_co	ENSG0000	22849	21746
CPEB4	4.00068	6.67268	4.33199	4.59756	6.45016	6.46555	cytoplasmic polyadenylation element binding protein 4 [Source:H	chr5:17331	NM_00130	XM_00526	.	.	NP_00129	XP_005266	protein_co	ENSG0000	80315	21747
CPED1	0.306508	0.224702	0.145395	0.259823	0.140107	0.258321	cadherin like and PC-esterase domain containing 1 [Source:HGNC	chr7:12062	NM_00110	XM_01151	.	.	NP_00109	XP_011514	protein_co	ENSG0000	79974	26159
CPLX1	6.27884	7.41873	6.1947	7.04941	7.95647	4.86651	complexin 1 [Source:HGNC Symbol;Acc:HGNC:2309]	chr4:77874	NM_00665	XM_01151	.	.	NP_00664	XP_011511	protein_co	ENSG0000	10815	2309
CPLX2	0.056766	0.164902	0.308607	0.030768	0.201162	0.076368	complexin 2 [Source:HGNC Symbol;Acc:HGNC:2310]	chr5:17522	NM_00100	XM_01153	.	.	NP_00100	XP_005265	protein_co	ENSG0000	10814	2310
CPLX3	0.062233	0.031125	0.027051	0.033589	0.035725	0	complexin 3 [Source:HGNC Symbol;Acc:HGNC:27652]	chr15:7511	NM_00103	.	.	.	NP_00102	.	protein_co	ENSG0000	594855	27652
CPLX4	0	0	0	0	0	0	complexin 4 [Source:HGNC Symbol;Acc:HGNC:24330]	chr18:5694	NM_18165	.	.	.	NP_85763	.	protein_co	ENSG0000	339302	24330
CPM	0.893831	1.04794	1.28264	1.41655	1.77563	1.94844	carboxypeptidase M [Source:HGNC Symbol;Acc:HGNC:2311]	chr12:6924	NM_00100	.	.	.	NP_00100	.	protein_co	ENSG0000	1368	2311
CPN1	0	0.04057	0.105218	0.087835	0.046182	0.215272	carboxypeptidase N subunit 1 [Source:HGNC Symbol;Acc:HGNC:2	chr10:1018	NM_00130	XM_01153	.	.	NP_00129	.	protein_co	ENSG0000	1369	2312
CPN2	0	0	0	0	0	0	carboxypeptidase N subunit 2 [Source:HGNC Symbol;Acc:HGNC:2	chr3:19406	NM_00108	XM_00526	.	.	NP_00107	XP_005269	protein_co	ENSG0000	1370	2313
CPNE1	70.3805	71.6579	71.5615	73.871	63.7031	60.5193	copine 1 [Source:HGNC Symbol;Acc:HGNC:2314]	chr20:3421	NM_00119	.	.	.	NP_00118	.	protein_co	ENSG0000	8904	2314
CPNE2	21.0802	21.545	18.346	22.8875	15.7235	15.0445	copine 2 [Source:HGNC Symbol;Acc:HGNC:2315]	chr16:5712	NM_15272	.	.	.	NP_68994	.	protein_co	ENSG0000	221184	2315
CPNE3	41.1952	41.8904	47.6718	40.4024	49.8146	42.7232	copine 3 [Source:HGNC Symbol;Acc:HGNC:2316]	chr8:87526	NM_00390	XM_01701	.	.	NP_00389	XP_005251	protein_co	ENSG0000	8895	2316

CPNE4	0	0.030669	0.02564	0	0	0	copine 4 [Source:HGNC Symbol;Acc:HGNC:2317]	chr3:13125	NM_00128	XM_01151	.	.	NP_00127	XP_01151	protein_co	ENSG0000	131034	2317
CPNE5	0.118403	0.119428	0.135834	0.160569	0.06939	0.09747	copine 5 [Source:HGNC Symbol;Acc:HGNC:2318]	chr6:36708	NM_00131	XM_01151	.	.	NP_00130	XP_005249	protein_co	ENSG0000	57699	2318
CPNE6	0.032604	0	0	0	0	0	copine 6 [Source:HGNC Symbol;Acc:HGNC:2319]	chr14:2454	NM_00128	XM_00526	.	.	NP_00126	XP_005268	protein_co	ENSG0000	9362	2319
CPNE7	36.5491	35.2149	48.474	40.9533	35.5691	31.4241	copine 7 [Source:HGNC Symbol;Acc:HGNC:2320]	chr16:8964	NM_01442	XM_01702	.	.	NP_05524	XP_011521	protein_co	ENSG0000	27132	2320
CPNE8	4.5576	4.60108	5.20766	4.32284	5.03977	5.71913	copine 8 [Source:HGNC Symbol;Acc:HGNC:23498]	chr12:3904	NM_15363	XM_01701	.	.	NP_70589	XP_016874	protein_co	ENSG0000	144402	23498
CPNE9	0.410054	0.172157	0.354501	0.297531	0.117728	0.219668	copine family member 9 [Source:HGNC Symbol;Acc:HGNC:24336]	chr3:97454	NM_00130	XM_01153	.	.	NP_00129	XP_006713	protein_co	ENSG0000	151835	24336
CPO	0.229511	0.422182	0.250339	0.063814	0	0.057849	carboxypeptidase O [Source:HGNC Symbol;Acc:HGNC:21011]	chr2:20780	NM_17307	XM_01151	.	.	NP_77510	XP_011508	protein_co	ENSG0000	130749	21011
CPOX	150.02	163.773	157.829	304.745	285.506	271.091	coproporphyrinogen oxidase [Source:HGNC Symbol;Acc:HGNC:23	chr3:98298	NM_00009	.	.	.	NP_00008	.	protein_co	ENSG0000	1371	2321
CPPED1	5.4981	6.026	6.31537	7.47853	7.50448	5.91177	calcineurin like phosphoesterase domain containing 1 [Source:HG	chr16:1275	NM_00109	.	.	.	NP_00109	.	protein_co	ENSG0000	55313	25632
CPQ	0.734672	0.57153	0.926715	0.611631	0.911983	2.10662	carboxypeptidase Q [Source:HGNC Symbol;Acc:HGNC:16910]	chr8:97657	NM_01613	XM_01151	.	.	NP_05721	XP_005250	protein_co	ENSG0000	10404	16910
CPS1	8.34278	8.87962	9.74696	9.87655	10.5481	10.926	carbamoyl-phosphate synthase 1 [Source:HGNC Symbol;Acc:HGN	chr2:21134	NM_00112	XM_01151	.	.	NP_00111	XP_011508	protein_co	ENSG0000	1373	2323
CPSF1	43.4288	46.89	47.4869	59.7914	44.6784	41.08	cleavage and polyadenylation specific factor 1 [Source:HGNC Sym	chr8:14559	NM_01329	XM_01151	NR_03063	.	NP_03742	XP_006716	protein_co	ENSG0000	102466749	2324
CPSF2	9.07768	9.46052	9.05298	10.5072	10.6964	10.1421	cleavage and polyadenylation specific factor 2 [Source:HGNC Sym	chr14:9258	NM_01743	XM_00526	.	.	NP_00130	XP_005267	protein_co	ENSG0000	53981	2325
CPSF3	32.3326	31.2551	31.6877	32.5787	31.6997	35.3645	cleavage and polyadenylation specific factor 3 [Source:HGNC Sym	chr2:95636	NM_01620	XM_01151	.	.	NP_00130	XP_005246	protein_co	ENSG0000	51692	2326
CPSF3L	51.571	46.4919	42.3548	56.856	47.633	41.583	cleavage and polyadenylation specific factor 3-like [Source:HGNC	chr1:12439	NM_00125	XM_01154	NR_10678	.	NP_00124	XP_011539	protein_co	ENSG0000	102465435	26052
CPSF4	35.739	31.952	37.8414	35.4918	34.3809	31.7703	cleavage and polyadenylation specific factor 4 [Source:HGNC Sym	chr7:98992	NM_00108	XM_01701	.	.	NP_00107	XP_011514	protein_co	ENSG0000	10898	2327
CPSF4L	0	0	0.108759	0.136027	0	0	cleavage and polyadenylation specific factor 4 like [Source:HGNC	chr17:7118	NM_00112	.	.	.	NP_00112	.	protein_co	ENSG0000	642843	33632
CPSF6	46.3039	43.5669	39.9135	53.696	48.3225	45.95	cleavage and polyadenylation specific factor 6 [Source:HGNC Sym	chr12:6963	NM_00130	XM_00526	.	.	NP_00128	XP_005268	protein_co	ENSG0000	11052	13871
CPSF7	38.1084	38.0697	38.5998	33.2378	35.2997	30.4089	cleavage and polyadenylation specific factor 7 [Source:HGNC Sym	chr11:6117	NM_00113	XM_01154	.	.	NP_00112	XP_005274	protein_co	ENSG0000	79869	30098
CPT1A	23.0322	23.1934	23.5329	18.1987	20.1368	16.2839	carnitine palmitoyltransferase 1A [Source:HGNC Symbol;Acc:HGN	chr11:6852	NM_00103	XM_00527	.	.	NP_00102	XP_005273	protein_co	ENSG0000	1374	2328
CPT1B	6.34329	5.7778	5.79887	5.82888	4.52675	5.5994	carnitine palmitoyltransferase 1B [Source:HGNC Symbol;Acc:HGN	chr22:5100	NM_00114	.	.	.	NP_00113	.	protein_co	ENSG0000	1375	2329
CPT1C	1.59997	1.42936	2.36996	1.78561	0.854028	1.54795	carnitine palmitoyltransferase 1C [Source:HGNC Symbol;Acc:HGN	chr19:5019	NM_00113	XM_01152	.	.	NP_00112	XP_005258	protein_co	ENSG0000	126129	18540
CPT2	10.9948	10.5419	14.5829	12.6277	13.7551	16.5187	carnitine palmitoyltransferase 2 [Source:HGNC Symbol;Acc:HGNC	chr1:53662	NM_00009	XM_00527	.	.	NP_00008	XP_005270	protein_co	ENSG0000	1376	2330
CPTP	11.0497	13.1203	10.4981	15.3494	12.0288	9.28594	ceramide-1-phosphate transfer protein [Source:HGNC Symbol;Ac	chr1:12601	NM_00102	XM_01154	.	.	NP_00102	XP_005244	protein_co	ENSG0000	80772	28116
CPVL	9.55635	9.43766	9.20139	8.24514	9.0712	10.3969	carboxypeptidase, vitellogenic like [Source:HGNC Symbol;Acc:HG	chr7:29034	NM_01902	XM_01151	.	.	NP_06190	XP_011513	protein_co	ENSG0000	54504	14399
CPXCR1	0	0	0	0	0	0	CPX chromosome region, candidate 1 [Source:HGNC Symbol;Acc:	chrX:88002	NM_00118	.	.	.	NP_00117	.	protein_co	ENSG0000	53336	2332
CPXM1	0.15494	0.15301	0.587273	0.099705	0.144852	0.410528	carboxypeptidase X, M14 family member 1 [Source:HGNC Symbo	chr20:2774	NM_00118	.	.	.	NP_00117	.	protein_co	ENSG0000	56265	15771
CPXM2	0	0	0	0	0.06645	0.032614	carboxypeptidase X, M14 family member 2 [Source:HGNC Symbo	chr10:1254	NM_19814	XM_01153	.	.	NP_93779	XP_005269	protein_co	ENSG0000	119587	26977
CPZ	0	0	0.033552	0.041692	0	0.043758	carboxypeptidase Z [Source:HGNC Symbol;Acc:HGNC:2333]	chr4:85943	NM_00101	.	.	.	NP_00101	.	protein_co	ENSG0000	8532	2333
CR1	0	0	0.009138	0	0	0	complement component 3b/4b receptor 1 (Knops blood group) [S	chr1:20766	NM_00057	XM_01150	.	.	NP_00056	XP_006711	protein_co	ENSG0000	1378	2334
CR1L	0	0	0	0	0	0	complement component 3b/4b receptor 1-like [Source:HGNC Syn	chr1:20781	NM_17571	.	.	.	NP_78364	.	protein_co	ENSG0000	1379	2335
CR2	2.64179	2.17162	3.85686	2.58849	2.09258	2.51848	complement component 3d receptor 2 [Source:HGNC Symbol;Ac	chr1:20762	NM_00100	XM_01150	.	.	NP_00100	XP_011507	protein_co	ENSG0000	1380	2336
CR769776.1	0	0	0	0	0	0	LOC441426 protein; Uncharacterized protein [Source:UniProtKB/	chr9:69377	.	.	.	.	.	.	protein_co	ENSG0000	.	.
CRABP1	0	0	0	0	0	0	cellular retinoic acid binding protein 1 [Source:HGNC Symbol;Acc:	chr15:7863	NM_00437	.	.	.	NP_00436	.	protein_co	ENSG0000	1381	2338
CRABP2	211.201	212.74	256.158	96.0401	76.1701	64.3888	cellular retinoic acid binding protein 2 [Source:HGNC Symbol;Acc:	chr1:15666	NM_00119	.	.	.	NP_00118	XP_016855	protein_co	ENSG0000	1382	2339
CRACR2A	4.3328	4.01193	3.82339	3.3637	3.88409	3.02547	calcium release activated channel regulator 2A [Source:HGNC Syn	chr12:3724	NM_00114	XM_01152	.	.	NP_00113	XP_006719	protein_co	ENSG0000	84766	28657
CRACR2B	2.69607	2.1196	3.5177	2.45548	1.73494	2.33993	calcium release activated channel regulator 2B [Source:HGNC Syn	chr11:8275	NM_00128	XM_01701	.	.	NP_00127	XP_011518	protein_co	ENSG0000	283229	28703
CRADD	8.74679	7.12031	8.87013	9.30221	7.1258	7.90885	CASP2 and RIPK1 domain containing adaptor with death domain	chr12:9407	NM_00132	XM_01702	.	.	NP_00130	XP_005269	protein_co	ENSG0000	8738	2340
CRAMP1	7.39005	7.48749	6.91532	6.92803	7.24496	5.87759	cramped chromatin regulator homolog 1 [Source:HGNC Symbol;A	chr16:1662	NM_02082	.	.	.	NP_06587	.	protein_co	ENSG0000	57585	14122
CRAT	8.6769	9.03591	10.2488	14.1555	11.4742	13.6946	carnitine O-acetyltransferase [Source:HGNC Symbol;Acc:HGNC:23	chr9:13185	NM_00075	XM_00525	.	.	NP_00074	XP_005251	protein_co	ENSG0000	1384	2342
CRB1	0.020698	0.021798	0.017896	0	0	0.036271	crumbs 1, cell polarity complex component [Source:HGNC Symbo	chr1:19723	NM_00119	XM_01700	.	.	NP_00118	XP_011507	protein_co	ENSG0000	23418	2343
CRB2	0.017396	0	0.059095	0	0.040657	0.03886	crumbs 2, cell polarity complex component [Source:HGNC Symbo	chr9:12611	NM_17368	XM_01151	.	.	NP_77596	XP_005251	protein_co	ENSG0000	286204	18688
CRB3	31.0444	32.3555	28.252	32.185	23.3689	29.5086	crumbs 3, cell polarity complex component [Source:HGNC Symbo	chr19:6463	NM_13916	XM_00525	.	.	NP_63190	XP_005259	protein_co	ENSG0000	92359	20237
CRBN	7.76496	7.26369	7.69647	6.76993	6.31577	6.49815	cereblon [Source:HGNC Symbol;Acc:HGNC:30185]	chr3:31916	NM_00117	XM_01153	.	.	NP_00116	XP_005265	protein_co	ENSG0000	51185	30185
CRCP	18.3819	18.5738	19.4547	18.6526	20.2482	15.0147	CGRP receptor component [Source:HGNC Symbol;Acc:HGNC:178	chr7:65540	NM_00104	.	.	.	NP_00103	.	protein_co	ENSG0000	27297	17888
CRCT1	0	0	0	0	0	0	cysteine rich C-terminal 1 [Source:HGNC Symbol;Acc:HGNC:2987	chr1:15248	NM_01906	XM_01150	.	.	NP_06193	XP_011507	protein_co	ENSG0000	54544	29875
CREB1	7.3738	8.71013	8.51244	9.59588	11.2529	10.3972	cAMP responsive element binding protein 1 [Source:HGNC Symb	chr2:20839	NM_00437	XM_01700	.	.	NP_00437	XP_011508	protein_co	ENSG0000	1385	2345
CREB3	23.5774	24.7087	19.9137	22.762	18.776	17.209	cAMP responsive element binding protein 3 [Source:HGNC Symb	chr9:35696	NM_00636	.	.	.	NP_00635	.	protein_co	ENSG0000	10488	2347
CREB3L1	0.110375	0.109111	0.238899	0.147716	0.095817	0.091698	cAMP responsive element binding protein 3 like 1 [Source:HGNC	chr11:4629	NM_05285	.	.	.	NP_44308	.	protein_co	ENSG0000	90993	18856
CREB3L2	12.5411	12.5689	12.5454	10.1567	11.1797	14.0665	cAMP responsive element binding protein 3 like 2 [Source:HGNC	chr7:13755	NM_00125	XM_01701	.	.	NP_00124	XP_005250	protein_co	ENSG0000	64764	23720
CREB3L3	0.219712	0.058737	0.085977	0.379123	0.119043	0.163623	cAMP responsive element binding protein 3 like 3 [Source:HGNC	chr19:4153	NM_00127	.	.	.	NP_00125	.	protein_co	ENSG0000	84699	18855
CREB3L4	9.60498	9.3346	6.90057	10.8347	8.40281	7.86551	cAMP responsive element binding protein 3 like 4 [Source:HGNC	chr1:15393	NM_00125	XM_01700	.	.	NP_00124	XP_006711	protein_co	ENSG0000	148327	18854
CREB5	0.821598	1.14916	0.840007	0.513836	0.717536	1.7532	cAMP responsive element binding protein 5 [Source:HGNC Symb	chr7:28338	NM_00101	XM_01701	.	.	NP_00101	XP_005249	protein_co	ENSG0000	9586	16844
CREBBP	8.33963	9.64052	7.02697	7.68884	7.52998	6.58432	CREB binding protein [Source:HGNC Symbol;Acc:HGNC:2348]	chr16:3775	NM_00107	XM_01702	.	.	NP_00107	XP_005255	protein_co	ENSG0000	1387	2348
CREBL2	7.07442	6.62923	8.44698	5.49171	6.50198	9.30153	cAMP responsive element binding protein like 2 [Source:HGNC Sy	chr12:1276	NM_00131	.	.	.	NP_00130	.	protein_co	ENSG0000	1389	2350
CREBRF	1.99233	2.22745	2.0488	0.767695	1.0412	2.10067	CREB3 regulatory factor [Source:HGNC Symbol;Acc:HGNC:24050]	chr5:17248	NM_00116	XM_01153	.	.	NP_00116	XP_005265	protein_co	ENSG0000	153222	24050
CREBZF	23.7821	24.8633	24.3649	18.6786	26.3474	16.8261	CREB/ATF bZIP transcription factor [Source:HGNC Symbol;Acc:HG	chr11:8537	NM_00103	XM_01154	.	.	NP_00103	XP_006718	protein_co	ENSG0000	58487	24905
CREG1	35.5582	34.76	45.8165	30.6504	35.8536	45.1849	cellular repressor of E1A stimulated genes 1 [Source:HGNC Symb	chr1:16751	NM_00385	.	.	.	NP_00384	.	protein_co	ENSG0000	8804	2351
CREG2	0.012338	0.039632	0.032234	0.056334	0.054687	0.072118	cellular repressor of E1A stimulated genes 2 [Source:HGNC Symb	chr2:10196	NM_15383	.	.	.	NP_72257	.	protein_co	ENSG0000	200407	14272

CRELD1	12.6245	12.9224	8.24069	11.8857	9.87703	10.6404	cysteine rich with EGF like domains 1 [Source:HGNC Symbol;Acc:	chr3:9975	NM_0010	XM_01153	.	.	NP_00102	XP_011532	protein_co	ENSG0000	78987	14630
CRELD2	76.0912	73.1532	27.7523	64.2765	51.7302	37.3623	cysteine rich with EGF like domains 2 [Source:HGNC Symbol;Acc:	chr22:5031	NM_00113	XM_00526	.	.	NP_00112	XP_005261	protein_co	ENSG0000	79174	28150
CREM	14.2344	13.8806	10.8335	12.4732	13.0363	19.6695	cAMP responsive element modulator [Source:HGNC Symbol;Acc:	chr10:3541	NM_00126	XM_01701	.	.	NP_00125	XP_006717	protein_co	ENSG0000	1390	2352
CRH	0	0	0	0	0	0	corticotropin releasing hormone [Source:HGNC Symbol;Acc:HGN	chr8:6708	NM_0007	.	.	.	NP_00074	XP_016868	protein_co	ENSG0000	1392	2355
CRHBP	0	0	0	0	0	0	corticotropin releasing hormone binding protein [Source:HGNC S	chr5:7624	NM_00188	.	.	.	NP_00187	.	protein_co	ENSG0000	1393	2356
CRHR1	0.107875	0	0	0	0	0.091192	corticotropin releasing hormone receptor 1 [Source:HGNC Symbo	chr17:438	NM_00114	.	.	.	NP_00113	.	protein_co	ENSG0000	1394	2357
CRHR2	0	0	0	0	0	0	corticotropin releasing hormone receptor 2 [Source:HGNC Symbo	chr7:30691	NM_0012	XM_01151	.	.	NP_00118	XP_01151	protein_co	ENSG0000	1395	2358
CRIM1	8.94439	9.26658	10.044	5.98361	6.40504	7.99634	cysteine rich transmembrane BMP regulator 1 [Source:HGNC Sym	chr2:3658	NM_01644	XM_01153	.	.	NP_05752	XP_005264	protein_co	ENSG0000	51232	2359
CRIP1	271.066	265.634	254.403	264.283	182.01	139.926	cysteine rich protein 1 [Source:HGNC Symbol;Acc:HGNC:2360]	chr14:105	NM_00131	.	.	.	NP_00130	.	protein_co	ENSG0000	1396	2360
CRIP2	16.0261	15.2278	21.9027	15.361	12.6089	13.9876	cysteine rich protein 2 [Source:HGNC Symbol;Acc:HGNC:2361]	chr14:105	NM_00127	.	NR_07308	.	NP_00125	.	protein_co	ENSG0000	1397	2361
CRIP3	0	0	0	0	0	0	cysteine rich protein 3 [Source:HGNC Symbol;Acc:HGNC:17751]	chr6:4326	NM_20692	XM_00524	.	.	NP_99680	XP_00524	protein_co	ENSG0000	401262	17751
CRIPAK	2.741	2.88763	3.43632	2.74509	2.22103	2.71384	cysteine rich PAK1 inhibitor [Source:HGNC Symbol;Acc:HGNC:266	chr4:1385	NM_17591	.	.	.	NP_78711	.	protein_co	ENSG0000	285464	26619
CRIPT	7.99902	8.98683	7.77637	8.2629	8.3157	10.2931	CXXC repeat containing interactor of PDZ3 domain [Source:HGNC	chr2:46844	NM_01417	.	.	.	NP_05489	.	protein_co	ENSG0000	9419	14312
CRISP1	0	0	0	0	0	0	cysteine rich secretory protein 1 [Source:HGNC Symbol;Acc:HGN	chr6:49801	NM_00113	XM_00671	.	.	NP_00112	.	protein_co	ENSG0000	167	304
CRISP2	0	0	0	0	0	0	cysteine rich secretory protein 2 [Source:HGNC Symbol;Acc:HGN	chr6:4966	NM_00114	XM_01151	.	.	NP_00113	XP_00524	protein_co	ENSG0000	7180	12024
CRISP3	0	0.078777	0.063838	0	0	0	cysteine rich secretory protein 3 [Source:HGNC Symbol;Acc:HGN	chr6:4969	NM_00119	.	.	.	NP_00117	.	protein_co	ENSG0000	10321	16904
CRISPLD1	0	0	0	0	0	0.037256	cysteine rich secretory protein LCCL domain containing 1 [Source	chr8:7589	NM_00128	.	.	.	NP_00127	.	protein_co	ENSG0000	83690	18206
CRISPLD2	1.52044	1.67835	2.22918	1.98851	2.23338	2.32246	cysteine rich secretory protein LCCL domain containing 2 [Source	chr16:848	NM_03147	XM_00525	.	.	NP_11366	XP_00525	protein_co	ENSG0000	83716	25248
CRK	38.8323	41.1661	41.4622	41.0829	46.937	46.0664	CRK proto-oncogene, adaptor protein [Source:HGNC Symbol;Acc:	chr17:132	NM_0052	.	.	.	NP_00519	.	protein_co	ENSG0000	1398	2362
CRKL	38.7401	40.1804	40.8414	40.8081	41.695	42.5524	CRK like proto-oncogene, adaptor protein [Source:HGNC Symbol;	chr22:212	NM_0052	.	.	.	NP_00519	.	protein_co	ENSG0000	1399	2363
CRLF1	20.0174	18.8925	32.4441	29.3218	23.2974	25.0886	cytokine receptor like factor 1 [Source:HGNC Symbol;Acc:HGNC:2	chr19:187	NM_00475	.	.	.	NP_00474	XP_01152	protein_co	ENSG0000	9244	2364
CRLF2	0.388743	0.392672	0.112735	0.28255	0.120726	0.13723	cytokine receptor-like factor 2 [Source:HGNC Symbol;Acc:HGNC:1	chrX:1314	NM_00101	XM_01154	.	.	NP_00101	XP_011543	protein_co	ENSG0000	64109	14281
CRLF3	14.3055	15.5753	15.9393	14.907	16.6701	16.6932	cytokine receptor like factor 3 [Source:HGNC Symbol;Acc:HGNC:1	chr17:291	NM_01598	.	.	.	NP_05707	.	protein_co	ENSG0000	51379	17177
CRLS1	16.8228	14.1584	18.3569	13.7211	20.9893	20.1177	cardiolipin synthase 1 [Source:HGNC Symbol;Acc:HGNC:16148]	chr20:598	NM_00112	XM_01152	.	.	NP_00112	XP_00526	protein_co	ENSG0000	54675	16148
CRMP1	0	0	0	0	0.028447	0	collapsin response mediator protein 1 [Source:HGNC Symbol;Acc	chr4:5822	NM_00101	.	.	.	NP_00101	.	protein_co	ENSG0000	1400	2365
CRNKL1	11.7977	12.4466	11.3351	13.5879	13.8771	12.8471	crooked neck pre-mRNA splicing factor 1 [Source:HGNC Symbol;A	chr20:2001	NM_00127	.	.	.	NP_00126	.	protein_co	ENSG0000	51340	15762
CRNN	0	0	0	0	0	0	cornulin [Source:HGNC Symbol;Acc:HGNC:1230]	chr1:1523	NM_01619	.	.	.	NP_05727	.	protein_co	ENSG0000	49860	1230
CROCC	5.21368	5.88804	4.99484	3.98962	3.3085	3.88465	ciliary rootlet coiled-coil, rootletin [Source:HGNC Symbol;Acc:HG	chr1:1724	NM_01467	XM_01700	.	.	NP_05549	XP_006711	protein_co	ENSG0000	9696	21299
CROCC2	0	0	0	0	0	0	ciliary rootlet coiled-coil, rootletin family member 2 [Source:HGN	chr2:2418	.	XM_01151	.	.	.	.	protein_co	ENSG0000	.	51677
CROT	0	0	0	0	0	0	carnitine O-octanoyltransferase [Source:HGNC Symbol;Acc:HGNC	chr7:8697	NM_00114	XM_01151	.	.	NP_00113	XP_011514	protein_co	ENSG0000	54677	2366
CRP	0	0	0	0	0	0	C-reactive protein, pentraxin-related [Source:HGNC Symbol;Acc:H	chr1:1596	NM_0005	XM_01150	.	.	NP_00055	XP_011507	protein_co	ENSG0000	1401	2367
CRTAC1	0	0	0	0	0	0	cartilage acidic protein 1 [Source:HGNC Symbol;Acc:HGNC:14882	chr10:996	NM_0012	XM_01153	.	.	NP_00119	XP_00526	protein_co	ENSG0000	55118	14882
CRTAM	0	0	0	0.038135	0	0.033817	cytotoxic and regulatory T-cell molecule [Source:HGNC Symbol;A	chr11:122	NM_0013	XM_01154	.	.	NP_00129	XP_011541	protein_co	ENSG0000	56253	24313
CRTAP	46.8461	45.303	55.4171	45.0221	42.4606	43.2856	cartilage associated protein [Source:HGNC Symbol;Acc:HGNC:237	chr3:3315	NM_00637	.	.	.	NP_00636	.	protein_co	ENSG0000	10491	2379
CRTC1	5.35959	5.69488	5.05094	5.49078	4.76855	4.74073	CREB regulated transcription coactivator 1 [Source:HGNC Symbol	chr19:187	NM_00109	XM_01152	.	.	NP_00109	XP_00525	protein_co	ENSG0000	23373	16062
CRTC2	18.675	19.3801	15.6876	16.2614	15.224	13.7576	CREB regulated transcription coactivator 2 [Source:HGNC Symbol	chr1:1539	NM_18171	XM_01150	.	.	NP_85906	XP_00524	protein_co	ENSG0000	200186	27301
CRTC3	7.32947	8.72538	8.18162	7.64699	7.81441	7.06849	CREB regulated transcription coactivator 3 [Source:HGNC Symbol	chr15:910	NM_00104	XM_01152	.	.	NP_00103	XP_00525	protein_co	ENSG0000	64784	26148
CRX	0	0	0	0	0	0.035904	cone-rod homeobox [Source:HGNC Symbol;Acc:HGNC:2383]	chr19:483	NM_00055	.	.	.	NP_00054	.	protein_co	ENSG0000	1406	2383
CRY1	11.1238	10.6839	7.50055	10.766	10.6572	12.4751	cryptochrome circadian clock 1 [Source:HGNC Symbol;Acc:HGNC	chr12:107	NM_00407	XM_01153	.	.	NP_00406	XP_016874	protein_co	ENSG0000	1407	2384
CRY2	4.55971	4.85422	4.1411	4.88987	4.33659	5.60305	cryptochrome circadian clock 2 [Source:HGNC Symbol;Acc:HGNC	chr11:458	NM_00112	.	.	.	NP_00112	.	protein_co	ENSG0000	1408	2385
CRYAA	0	0	0	0	0	0	crystallin alpha A [Source:HGNC Symbol;Acc:HGNC:2388]	chr21:445	NM_00039	XM_00526	.	.	NP_00038	XP_005261	protein_co	ENSG0000	102724652	2388
CRYAB	0	0.160088	0	0	0	0	crystallin alpha B [Source:HGNC Symbol;Acc:HGNC:2389]	chr11:111	NM_00128	XM_01154	.	.	NP_00127	XP_01154	protein_co	ENSG0000	1410	2389
CRYBA1	0	0	0	0	0	0	crystallin beta A1 [Source:HGNC Symbol;Acc:HGNC:2394]	chr17:275	NM_0052	.	.	.	NP_00519	.	protein_co	ENSG0000	1411	2394
CRYBA2	0	0	0.120276	0	0	0.128083	crystallin beta A2 [Source:HGNC Symbol;Acc:HGNC:2395]	chr2:2198	NM_05709	XM_01151	.	.	NP_47643	XP_011508	protein_co	ENSG0000	1412	2395
CRYBA4	0	0.10613	0.092542	0.232292	0	0	crystallin beta A4 [Source:HGNC Symbol;Acc:HGNC:2396]	chr22:2701	NM_00188	XM_01702	.	.	NP_00187	XP_006724	protein_co	ENSG0000	1413	2396
CRYBB1	0	0	0.06673	0.166524	0	0	crystallin beta B1 [Source:HGNC Symbol;Acc:HGNC:2397]	chr22:269	NM_00188	XM_01152	.	.	NP_00187	XP_011528	protein_co	ENSG0000	1414	2397
CRYBB2	0.24199	0.24226	0.631046	0.870209	1.2116	0.61234	crystallin beta B2 [Source:HGNC Symbol;Acc:HGNC:2398]	chr22:2561	NM_00049	XM_01152	.	.	NP_00048	XP_006724	protein_co	ENSG0000	1415	2398
CRYBB3	0.211686	0.521307	0	0.483552	0.203285	0.115959	crystallin beta B3 [Source:HGNC Symbol;Acc:HGNC:2400]	chr22:255	NM_00407	XM_01152	.	.	NP_00406	XP_011528	protein_co	ENSG0000	1417	2400
CRYBG3	3.44183	3.72964	3.66393	3.3581	4.10681	3.78773	crystallin beta-gamma domain containing 3 [Source:HGNC Symbo	chr3:9754	NM_1536	XM_00524	.	.	NP_70583	XP_005247	protein_co	ENSG0000	131544	34427
CRYGA	0	0	0	0	0	0	crystallin gamma A [Source:HGNC Symbol;Acc:HGNC:2408]	chr2:20902	NM_01461	.	.	.	NP_05543	.	protein_co	ENSG0000	1418	2408
CRYGB	0	0	0	0	0	0	crystallin gamma B [Source:HGNC Symbol;Acc:HGNC:2409]	chr2:2090	NM_00521	.	.	.	NP_00520	XP_016858	protein_co	ENSG0000	1419	2409
CRYGC	0	0	0	0	0	0.333755	crystallin gamma C [Source:HGNC Symbol;Acc:HGNC:2410]	chr2:2089	NM_02098	XM_01151	.	.	NP_06626	XP_011508	protein_co	ENSG0000	1420	2410
CRYGD	0	0	0	0	0	0	crystallin gamma D [Source:HGNC Symbol;Acc:HGNC:2411]	chr2:20898	NM_00689	.	.	.	NP_00882	.	protein_co	ENSG0000	1421	2411
CRYGN	0	0	0	0	0	0	crystallin gamma N [Source:HGNC Symbol;Acc:HGNC:20458]	chr7:1511	NM_0013	XM_00524	NR_13177	.	NP_00129	XP_00525	protein_co	ENSG0000	155051	20458
CRYGS	4.39708	2.03079	2.7	1.43041	2.97794	1.62695	crystallin gamma S [Source:HGNC Symbol;Acc:HGNC:2417]	chr3:1862	NM_01754	.	.	.	NP_06001	.	protein_co	ENSG0000	1427	2417
CRYL1	11.6305	12.6548	20.051	13.4945	12.182	18.3214	crystallin lambda 1 [Source:HGNC Symbol;Acc:HGNC:18246]	chr13:209	NM_01597	XM_00526	.	.	NP_05705	XP_00526	protein_co	ENSG0000	51084	18246
CRYM	2.72899	2.61287	4.42422	6.28217	4.01507	6.25743	crystallin mu [Source:HGNC Symbol;Acc:HGNC:2418]	chr16:212	NM_00188	XM_01154	.	.	NP_00187	XP_011544	protein_co	ENSG0000	1428	2418
CRYZ	21.9336	21.6608	17.9351	21.6668	21.115	15.8339	crystallin zeta [Source:HGNC Symbol;Acc:HGNC:2419]	chr1:75171	NM_00113	XM_01154	.	.	NP_00112	XP_00527	protein_co	ENSG0000	1429	2419
CRYZL1	8.66636	9.20415	8.85061	7.11182	7.62654	7.72816	crystallin zeta like 1 [Source:HGNC Symbol;Acc:HGNC:2420]	chr21:348	NM_14585	.	.	.	NP_66585	.	protein_co	ENSG0000	9946	2420

CS	81.1706	85.8443	83.5843	93.8371	82.2002	81.8955	citrate synthase [Source:HGNC Symbol;Acc:HGNC:2422]	chr12:5666	NM_00407	.	.	.	NP_00406	.	protein_co	ENSG0000	1431	2422
CSAD	4.53089	3.72648	4.03863	4.36012	4.20941	3.09195	cysteine sulfinic acid decarboxylase [Source:HGNC Symbol;Acc:H	chr12:5355	NM_00124	XM_01153	.	.	NP_00123	XP_011536	protein_co	ENSG0000	51380	18966
CSAG1	1.96531	0.94504	0.908795	1.68468	1.77977	1.98577	chondrosarcoma associated gene 1 [Source:HGNC Symbol;Acc:H	chrX:15190	NM_00110	.	.	.	NP_00109	.	protein_co	ENSG0000	158511	24294
CSDC2	0	0.05794	0.050891	0.062926	0	0.066422	cold shock domain containing C2 [Source:HGNC Symbol;Acc:HGN	chr22:4195	NM_01446	.	.	.	NP_05527	.	protein_co	ENSG0000	27254	30359
CSDE1	175.793	173.753	161.972	180.123	191.408	194.962	cold shock domain containing E1 [Source:HGNC Symbol;Acc:HGN	chr1:11525	NM_00100	.	.	.	NP_00100	.	protein_co	ENSG0000	7812	29905
CSE1L	86.9014	79.1692	78.1364	99.052	122.238	99.3881	chromosome segregation 1 like [Source:HGNC Symbol;Acc:HGNC	chr20:4766	NM_00125	XM_01152	.	.	NP_00124	XP_011526	protein_co	ENSG0000	1434	2431
CSF1	3.43439	3.89111	3.16948	1.70714	1.14804	0.82503	colony stimulating factor 1 [Source:HGNC Symbol;Acc:HGNC:243	chr1:11045	NM_00075	.	.	.	NP_00074	XP_016855	protein_co	ENSG0000	1435	2432
CSF1R	0.13741	0.085841	0.245883	0.110664	0.297269	0.168521	colony stimulating factor 1 receptor [Source:HGNC Symbol;Acc:H	chr5:14943	NM_00128	.	.	.	NP_00127	.	protein_co	ENSG0000	1436	2433
CSF2	0.833839	0.209436	0.181569	0.224147	0	0.11275	colony stimulating factor 2 [Source:HGNC Symbol;Acc:HGNC:243	chr5:13140	NM_00075	.	.	.	NP_00074	.	protein_co	ENSG0000	1437	2434
CSF2RA	0.748545	0.73176	1.44724	1.00537	0.828901	1.46872	colony stimulating factor 2 receptor alpha subunit [Source:HGNC	chrX:13876	NM_00116	XM_01154	.	.	NP_00115	XP_011543	protein_co	ENSG0000	1438	2435
CSF2RB	0.075225	0	0	0	0	0	colony stimulating factor 2 receptor beta common subunit [Sourc	chr22:3730	NM_00039	XM_01152	.	.	NP_00038	XP_005261	protein_co	ENSG0000	1439	2436
CSF3	0	0	0.05328	0	0	0	colony stimulating factor 3 [Source:HGNC Symbol;Acc:HGNC:243	chr17:3817	NM_00075	.	.	.	NP_00075	.	protein_co	ENSG0000	1440	2438
CSF3R	0	0	0	0	0	0	colony stimulating factor 3 receptor [Source:HGNC Symbol;Acc:H	chr1:36931	NM_00076	XM_01154	.	.	NP_00075	XP_005270	protein_co	ENSG0000	1441	2439
CSGALNACT1	0.179253	0.167908	0.312586	0.073082	0.075921	0.218969	chondroitin sulfate N-acetylgalactosaminyltransferase 1 [Source:	chr8:19261	NM_00113	XM_01701	.	.	NP_00112	XP_006716	protein_co	ENSG0000	55790	24290
CSGALNACT2	6.66327	7.02052	4.80983	4.39773	4.95852	6.97804	chondroitin sulfate N-acetylgalactosaminyltransferase 2 [Source:	chr10:4363	NM_01859	.	.	.	NP_06106	.	protein_co	ENSG0000	55454	24292
CSH1	0	0	0	0	0	0	chorionic somatomammotropin hormone 1 [Source:HGNC Symbo	chr17:6197	NM_00131	XM_01152	.	.	NP_00130	XP_011522	protein_co	ENSG0000	1444	2440
CSH2	0	0	0	0	0	0	chorionic somatomammotropin hormone 2 [Source:HGNC Symbo	chr17:6194	NM_02099	.	.	.	NP_06627	.	protein_co	ENSG0000	1443	2441
CSHL1	0	0	0	0	0	0	chorionic somatomammotropin hormone like 1 [Source:HGNC Sy	chr17:6198	NM_00131	XM_01152	.	.	NP_00130	XP_011522	protein_co	ENSG0000	1444	2442
CSK	32.8339	37.6027	32.6631	37.0394	33.1904	26.6173	c-src tyrosine kinase [Source:HGNC Symbol;Acc:HGNC:2444]	chr15:7507	NM_00112	XM_01702	.	.	NP_00112	XP_005254	protein_co	ENSG0000	1445	2444
CSMD1	0	0	0	0	0	0	CUB and Sushi multiple domains 1 [Source:HGNC Symbol;Acc:HG	chr8:27928	NM_03322	XM_01153	.	.	NP_15009	XP_011533	protein_co	ENSG0000	64478	14026
CSMD2	0	0.023644	0.080022	0.050767	0.131605	0.072863	CUB and Sushi multiple domains 2 [Source:HGNC Symbol;Acc:HG	chr1:33979	NM_00128	XM_01154	.	.	NP_00126	.	protein_co	ENSG0000	114784	19290
CSMD3	0.01183	0	0.010282	0	0.013125	0	CUB and Sushi multiple domains 3 [Source:HGNC Symbol;Acc:HG	chr8:11323	NM_05290	XM_01701	.	.	NP_44313	XP_011515	protein_co	ENSG0000	114788	19291
CSN1S1	0	0	0	0	0	0	casein alpha s1 [Source:HGNC Symbol;Acc:HGNC:2445]	chr4:70796	NM_00102	XM_00671	.	.	NP_00102	XP_006714	protein_co	ENSG0000	1446	2445
CSN2	0	0	0	0	0	0	casein beta [Source:HGNC Symbol;Acc:HGNC:2447]	chr4:70820	NM_00130	XM_01700	.	.	NP_00128	XP_011529	protein_co	ENSG0000	1447	2447
CSN3	0	0	0	0	0	0	casein kappa [Source:HGNC Symbol;Acc:HGNC:2446]	chr4:71108	NM_00521	XM_01700	.	.	NP_00520	XP_011529	protein_co	ENSG0000	1448	2446
CSNK1A1	107.185	104.461	90.7104	109.342	124.982	130.456	casein kinase 1 alpha 1 [Source:HGNC Symbol;Acc:HGNC:2451]	chr5:14887	NM_00102	.	.	.	NP_00102	.	protein_co	ENSG0000	1452	2451
CSNK1A1L	1.36607	0.978252	1.26506	1.25701	1.66757	1.61727	casein kinase 1 alpha 1 like [Source:HGNC Symbol;Acc:HGNC:202	chr13:3767	NM_14520	.	.	.	NP_66020	.	protein_co	ENSG0000	122011	20289
CSNK1D	65.9629	64.2638	67.2504	67.3058	61.8798	60.2238	casein kinase 1 delta [Source:HGNC Symbol;Acc:HGNC:2452]	chr17:8018	NM_00189	XM_00525	.	.	NP_00188	XP_005256	protein_co	ENSG0000	1453	2452
CSNK1E	50.9286	52.8412	52.601	56.4239	47.2878	52.6368	casein kinase 1 epsilon [Source:HGNC Symbol;Acc:HGNC:2453]	chr22:3868	NM_00128	.	.	.	NP_00127	.	protein_co	ENSG0000	102800317	2453
CSNK1G1	7.99818	9.01792	8.5338	6.65018	7.53626	8.24456	casein kinase 1 gamma 1 [Source:HGNC Symbol;Acc:HGNC:2454]	chr15:6445	NM_00132	.	.	.	NP_00131	.	protein_co	ENSG0000	53944	2454
CSNK1G2	52.7291	53.8708	54.0427	60.0398	51.6263	53.2642	casein kinase 1 gamma 2 [Source:HGNC Symbol;Acc:HGNC:2455]	chr19:1941	NM_00131	XM_00525	.	.	NP_00131	XP_005259	protein_co	ENSG0000	1455	2455
CSNK1G3	15.2747	15.7096	14.289	12.921	14.883	21.3316	casein kinase 1 gamma 3 [Source:HGNC Symbol;Acc:HGNC:2456]	chr5:12284	NM_00103	XM_00527	.	.	NP_00102	XP_005271	protein_co	ENSG0000	1456	2456
CSNK2A1	74.7648	76.1358	68.3935	79.6631	83.1346	90.626	casein kinase 2 alpha 1 [Source:HGNC Symbol;Acc:HGNC:2457]	chr20:4590	NM_00189	XM_01152	.	.	NP_00188	XP_011527	protein_co	ENSG0000	1457	2457
CSNK2A2	22.74	23.0958	22.3105	27.0552	27.8777	23.0829	casein kinase 2 alpha 2 [Source:HGNC Symbol;Acc:HGNC:2459]	chr16:5819	NM_00189	XM_00525	.	.	NP_00188	XP_005255	protein_co	ENSG0000	1459	2459
CSNK2A3	24.1436	21.7797	24.9754	23.1529	27.2493	30.3098	casein kinase 2 alpha 3 [Source:HGNC Symbol;Acc:HGNC:2458]	chr11:1129	NM_00125	.	.	.	NP_00124	.	protein_co	ENSG0000	283106	2458
CSNK2B	167.525	160.83	146.985	185.604	146.872	137.821	casein kinase 2 beta [Source:HGNC Symbol;Acc:HGNC:2460]	chr6:31629	NM_00128	.	.	.	NP_00126	.	protein_co	ENSG0000	1460	2460
CSPG4	6.76116	7.49153	6.61194	6.14768	6.18878	6.37084	chondroitin sulfate proteoglycan 4 [Source:HGNC Symbol;Acc:HG	chr15:7596	NM_00189	.	.	.	NP_00188	.	protein_co	ENSG0000	1464	2466
CSPG5	2.51372	2.51415	3.7125	2.18821	2.6064	3.53604	chondroitin sulfate proteoglycan 5 [Source:HGNC Symbol;Acc:HG	chr3:47603	NM_00120	.	.	.	NP_00119	.	protein_co	ENSG0000	10675	2467
CSPP1	8.79464	9.06456	9.47314	8.34717	9.91813	7.87972	centrosome and spindle pole associated protein 1 [Source:HGNC	chr8:67955	NM_00129	XM_01151	.	.	NP_00127	XP_005251	protein_co	ENSG0000	79848	26193
CSRNP1	8.55961	9.32583	6.405	6.96839	7.05026	5.84185	cysteine and serine rich nuclear protein 1 [Source:HGNC Symbol;	chr3:39183	NM_00132	XM_01153	.	.	NP_00130	XP_005265	protein_co	ENSG0000	64651	14300
CSRNP2	7.42363	7.97352	7.22103	8.76989	8.01214	10.1446	cysteine and serine rich nuclear protein 2 [Source:HGNC Symbol;	chr12:5145	NM_03080	XM_00671	.	.	NP_11043	XP_006719	protein_co	ENSG0000	81566	16006
CSRNP3	0.096112	0.080111	0.145554	0.008497	0	0.027223	cysteine and serine rich nuclear protein 3 [Source:HGNC Symbol;	chr2:16632	NM_00117	XM_00524	.	.	NP_00116	XP_005246	protein_co	ENSG0000	80034	30729
CSRP1	51.4366	49.8714	40.8442	54.7181	45.0919	39.3234	cysteine and glycine rich protein 1 [Source:HGNC Symbol;Acc:HG	chr1:20145	NM_00119	.	.	.	NP_00118	.	protein_co	ENSG0000	1465	2469
CSRP2	38.5166	30.882	33.7426	57.3995	43.681	42.3347	cysteine and glycine rich protein 2 [Source:HGNC Symbol;Acc:HG	chr12:7725	NM_00130	.	.	.	NP_00128	.	protein_co	ENSG0000	1466	2470
CSRP3	0	0	0	0	0	0	cysteine and glycine rich protein 3 [Source:HGNC Symbol;Acc:HG	chr11:1920	NM_00347	.	.	.	NP_00346	.	protein_co	ENSG0000	8048	2472
CST1	0	0	0.090584	0	0.117937	0	cystatin SN [Source:HGNC Symbol;Acc:HGNC:2473]	chr20:2372	NM_00189	.	.	.	NP_00188	.	protein_co	ENSG0000	1469	2473
CST11	0	0	0	0	0	0	cystatin 11 [Source:HGNC Symbol;Acc:HGNC:15959]	chr20:2343	NM_08083	.	.	.	NP_54302	.	protein_co	ENSG0000	140880	15959
CST2	0	0	0	0	0	0	cystatin SA [Source:HGNC Symbol;Acc:HGNC:2474]	chr20:2380	NM_00132	.	.	.	NP_00131	.	protein_co	ENSG0000	1470	2474
CST3	101.442	102.664	111.3	113.657	87.2566	97.5096	cystatin C [Source:HGNC Symbol;Acc:HGNC:2475]	chr20:2360	NM_00009	.	.	.	NP_00009	.	protein_co	ENSG0000	1471	2475
CST4	0	0	0	0	0	0	cystatin S [Source:HGNC Symbol;Acc:HGNC:2476]	chr20:2366	NM_00189	.	.	.	NP_00189	.	protein_co	ENSG0000	1472	2476
CST5	0	0	0	0	0	0	cystatin D [Source:HGNC Symbol;Acc:HGNC:2477]	chr20:2385	NM_00190	.	.	.	NP_00189	.	protein_co	ENSG0000	1473	2477
CST6	47.4552	43.8325	41.8031	57.1365	40.8986	44.944	cystatin E/M [Source:HGNC Symbol;Acc:HGNC:2478]	chr11:6557	NM_00132	.	.	.	NP_00131	.	protein_co	ENSG0000	1474	2478
CST7	0	0	0	0	0	0	cystatin F [Source:HGNC Symbol;Acc:HGNC:2479]	chr20:2492	NM_00365	.	.	.	NP_00364	.	protein_co	ENSG0000	8530	2479
CST8	0	0	0	0	0	0	cystatin 8 [Source:HGNC Symbol;Acc:HGNC:2480]	chr20:2347	NM_00128	.	.	.	NP_00126	.	protein_co	ENSG0000	10047	2480
CST9	0	0	0	0	0	0	cystatin 9 [Source:HGNC Symbol;Acc:HGNC:13261]	chr20:2358	NM_00100	.	.	.	NP_00100	.	protein_co	ENSG0000	128822	13261
CST9L	0	0	0	0	0	0	cystatin 9-like [Source:HGNC Symbol;Acc:HGNC:16233]	chr20:2354	NM_08061	.	.	.	NP_54217	.	protein_co	ENSG0000	128821	16233
CSTA	30.7393	28.4833	21.4965	5.11133	4.1241	12.2141	cystatin A [Source:HGNC Symbol;Acc:HGNC:2481]	chr3:12204	NM_00521	.	.	.	NP_00520	.	protein_co	ENSG0000	1475	2481
CSTB	63.1043	60.5832	56.1646	83.9934	65.3081	51.7238	cystatin B [Source:HGNC Symbol;Acc:HGNC:2482]	chr21:4519	NM_00010	.	.	.	NP_00009	.	protein_co	ENSG0000	1476	2482
CSTF1	15.1151	14.6387	14.8087	17.753	18.131	15.5878	cleavage stimulation factor subunit 1 [Source:HGNC Symbol;Acc:H	chr20:5496	NM_00103	XM_01152	.	.	NP_00102	XP_011526	protein_co	ENSG0000	1477	2483

CSTF2	22.0643	23.0684	17.3003	27.3009	24.8681	18.8957	cleavage stimulation factor subunit 2 [Source:HGNC Symbol;Acc:H	chrX:10007	NM_00130	XM_01153	.	.	NP_00129	XP_011529	protein_co	ENSG0000	1478	2484
CSTF2T	17.06	17.1683	13.8663	19.2505	18.4004	13.7208	cleavage stimulation factor subunit 2, tau variant [Source:HGNC S	chr10:5275	NM_01523	.	.	.	NP_05605	.	protein_co	ENSG0000	23283	17086
CSTF3	28.8142	26.5542	27.7436	24.4778	27.7504	29.9686	cleavage stimulation factor subunit 3 [Source:HGNC Symbol;Acc:H	chr11:3310	NM_00103	.	.	.	NP_00102	.	protein_co	ENSG0000	1479	2485
CSTL1	0	0	0	0	0	0	cystatin like 1 [Source:HGNC Symbol;Acc:HGNC:15958]	chr20:2342	NM_13828	.	.	.	NP_61214	.	protein_co	ENSG0000	128817	15958
CT45A1	0	0	0	0	0	0	cancer/testis antigen family 45, member A1 [Source:HGNC Symbo	chrX:13484	NM_00101	XM_01699	.	.	NP_00101	XP_005278	protein_co	ENSG0000	541466	33267
CT45A10	0.153735	0	0	0.681553	0.346172	0.312775	cancer/testis antigen family 45, member A10 [Source:HGNC Symb	chrX:13492	NM_00129	XM_01153	.	.	NP_00127	XP_011529	protein_co	ENSG0000	102723631	51263
CT45A2	0	0	0.111194	0	0	0	cancer/testis antigen family 45, member A2 [Source:HGNC Symbo	chrX:13486	NM_15258	XM_01153	.	.	NP_00130	XP_006724	protein_co	ENSG0000	102723680	28400
CT45A3	0	0	0	0	0	0	cancer/testis antigen family 45, member A3 [Source:HGNC Symbo	chrX:13488	NM_00101	XM_01699	.	.	NP_00101	XP_005278	protein_co	ENSG0000	541466	33268
CT45A4	0	0	0	0	0	0	cancer/testis antigen family 45, member A5 [Source:HGNC Symbo	chrX:13492	NM_00100	XM_01153	.	.	NP_00100	XP_006724	protein_co	ENSG0000	441521	33270
CT45A6	0	0	0	0	0	0	cancer/testis antigen family 45, member A6 [Source:HGNC Symbo	chrX:13496	NM_00101	XM_01153	.	.	NP_00101	XP_005262	protein_co	ENSG0000	541465	33271
CT45A7	0	0	0	2.55E-31	6.97E-20	0	cancer/testis antigen family 45, member A7 [Source:HGNC Symbo	chrX:13492	NM_00129	XM_01153	.	.	NP_00127	XP_003960	protein_co	ENSG0000	101060211	51260
CT45A8	0	0	0	0	0	0	cancer/testis antigen family 45, member A8 [Source:HGNC Symbo	chrX:13486	NM_00129	XM_01153	.	.	NP_00127	XP_006724	protein_co	ENSG0000	102723737	51261
CT45A9	0	0	0	0	0	0	cancer/testis antigen family 45, member A9 [Source:HGNC Symbo	chrX:13496	NM_00129	XM_01153	.	.	NP_00127	XP_006724	protein_co	ENSG0000	102723680	51262
CT47A1	0	0	0	0	0	0	cancer/testis antigen family 47, member A1 [Source:HGNC Symbo	chrX:12011	NM_00108	.	.	.	NP_00107	.	protein_co	ENSG0000	728096	33282
CT47A10	0	0	0	0	0	0	cancer/testis antigen family 47, member A10 [Source:HGNC Symb	chrX:12007	NM_00108	.	.	.	NP_00107	.	protein_co	ENSG0000	728036	33291
CT47A11	0	0	0	0	0	0	cancer/testis antigen family 47, member A11 [Source:HGNC Symb	chrX:12006	NM_17357	.	.	.	NP_77584	.	protein_co	ENSG0000	255313	27397
CT47A12	0	0	0	0	0	0	cancer/testis antigen family 47, member A12 [Source:HGNC Symb	chrX:12006	NM_00124	.	.	.	NP_00122	.	protein_co	ENSG0000	100507170	33292
CT47A2	0	0	0	0	0	0	cancer/testis antigen family 47, member A2 [Source:HGNC Symbo	chrX:12011	NM_00108	.	.	.	NP_00107	.	protein_co	ENSG0000	728090	33283
CT47A3	0	0	0	0	0	0	cancer/testis antigen family 47, member A3 [Source:HGNC Symbo	chrX:12010	NM_00108	.	.	.	NP_00107	.	protein_co	ENSG0000	728082	33284
CT47A4	0	0	0	0	0	0	cancer/testis antigen family 47, member A4 [Source:HGNC Symbo	chrX:12010	NM_00108	.	.	.	NP_00107	.	protein_co	ENSG0000	728075	33285
CT47A5	0	0	0	0	0	0	cancer/testis antigen family 47, member A5 [Source:HGNC Symbo	chrX:12009	NM_00108	.	.	.	NP_00107	.	protein_co	ENSG0000	728072	33286
CT47A6	0	0	0	0	0	0	cancer/testis antigen family 47, member A6 [Source:HGNC Symbo	chrX:12009	NM_00108	.	.	.	NP_00107	.	protein_co	ENSG0000	728062	33287
CT47A7	0	0	0	0	0	0	cancer/testis antigen family 47, member A7 [Source:HGNC Symbo	chrX:12008	NM_00108	.	.	.	NP_00107	.	protein_co	ENSG0000	653282	33288
CT47A8	0	0	0	0	0	0	cancer/testis antigen family 47, member A8 [Source:HGNC Symbo	chrX:12008	NM_00108	.	.	.	NP_00107	.	protein_co	ENSG0000	728049	33289
CT47A9	0	0	0	0	0	0	cancer/testis antigen family 47, member A9 [Source:HGNC Symbo	chrX:12007	NM_00108	.	.	.	NP_00107	.	protein_co	ENSG0000	728042	33290
CT47B1	0	0	0	0	0	0	cancer/testis antigen family 47, member B1 [Source:HGNC Symbo	chrX:12000	NM_00114	XM_01702	.	.	NP_00113	XP_016885	protein_co	ENSG0000	643311	33293
CT55	0	0	0	0	0	0	cancer/testis antigen 55 [Source:HGNC Symbol;Acc:HGNC:26047]	chrX:13429	NM_00103	XM_00526	.	.	NP_00102	XP_005262	protein_co	ENSG0000	54967	26047
CT62	0.038082	0.038964	0.033217	0	0	0	cancer/testis antigen 62 [Source:HGNC Symbol;Acc:HGNC:27286]	chr15:7138	NM_00110	XM_00672	.	.	NP_00109	XP_006720	protein_co	ENSG0000	196993	27286
CT83	0	0	0	0	0	0	cancer/testis antigen 83 [Source:HGNC Symbol;Acc:HGNC:33494]	chrX:11559	NM_00101	.	.	.	NP_00101	.	protein_co	ENSG0000	203413	33494
CTAG1A	0	0	0	0	0	0	cancer/testis antigen 1A [Source:HGNC Symbol;Acc:HGNC:24198]	chrX:15381	NM_13925	.	.	.	NP_64034	.	protein_co	ENSG0000	246100	24198
CTAG1B	0	0	0	0	0	0	cancer/testis antigen 1B [Source:HGNC Symbol;Acc:HGNC:2491]	chrX:15384	NM_00132	.	.	.	NP_00131	.	protein_co	ENSG0000	1485	2491
CTAG2	0	0	0	0	0	0	cancer/testis antigen 2 [Source:HGNC Symbol;Acc:HGNC:2492]	chrX:15388	NM_02099	.	.	.	NP_06627	.	protein_co	ENSG0000	30848	2492
CTAGE1	0.365721	0.249655	0.268569	0.221522	0.316052	0.685842	cutaneous T-cell lymphoma-associated antigen 1 [Source:HGNC S	chr18:1999	NM_17224	.	.	.	NP_75844	.	protein_co	ENSG0000	64693	24346
CTAGE15	0.543441	0.262535	0.173704	0.50504	0.191896	0.342237	CTAGE family member 15 [Source:HGNC Symbol;Acc:HGNC:3729	chr7:14326	NM_00100	.	.	.	NP_00100	.	protein_co	ENSG0000	441294	37295
CTAGE4	0.223908	0.105699	0.218447	0.140788	0.137941	0.221628	CTAGE family member 4 [Source:HGNC Symbol;Acc:HGNC:24772]	chr7:14388	NM_19849	.	.	.	NP_94089	.	protein_co	ENSG0000	100128553	24772
CTAGE5	10.1128	10.3378	9.99227	8.90323	10.6765	15.0304	CTAGE family member 5, ER export factor [Source:HGNC Symbol;	chr14:3970	NM_00124	XM_01702	.	.	NP_00123	XP_005267	protein_co	ENSG0000	4253	7057
CTAGE6	0	0	0	0	0	0	CTAGE family member 6 [Source:HGNC Symbol;Acc:HGNC:28644]	chr7:14331	NM_17856	.	.	.	NP_84865	.	protein_co	ENSG0000	340307	28644
CTAGE8	0.172874	0.367383	0.086769	0.223641	0.246598	0.293387	CTAGE family member 8 [Source:HGNC Symbol;Acc:HGNC:37294]	chr7:14396	NM_00127	.	.	.	NP_00126	.	protein_co	ENSG0000	100142659	37294
CTAGE9	0	0.028882	0.023793	0.030793	0	0.053236	CTAGE family member 9 [Source:HGNC Symbol;Acc:HGNC:37275]	chr6:13194	NM_00114	.	.	.	NP_00113	.	protein_co	ENSG0000	643854	37275
CTB-102L5.4	1.77477	0.501051	6.3E-31	2.44613	1.50238	3.57E-19	.	chr19:3875	.	.	.	.	.	.	protein_co	ENSG0000	.	.
CTB-133G6.1	0	0	0	0	0	0	.	chr19:7413	.	.	.	.	.	.	protein_co	ENSG0000	.	.
CTB-16J4.1	0	0	0	0	0	0	.	chr7:89945	.	.	.	.	.	.	protein_co	ENSG0000	.	.
CTB-25J19.1	0	0	0	0	0	0	.	chr19:7018	.	.	.	.	.	XP_011507	protein_co	ENSG0000	729458	.
CTB-50L17.10	33.7426	35.1777	33.8671	40.895	32.4266	31.2347	Hepatoma-derived growth factor-related protein 2 [Source:UniP	chr19:4472	NM_00100	XM_01152	.	.	NP_00100	XP_011526	protein_co	ENSG0000	84717	.
CTB-54O9.9	0.508773	0.675689	1.46974	1.66425	1.59643	1.48864	.	chr19:5782	.	.	.	.	.	.	protein_co	ENSG0000	.	.
CTB-60B18.10	0.293367	0.14173	0.8151	0.233967	0.171234	0.330142	.	chr19:4952	.	.	NR_07355	.	.	.	protein_co	ENSG0000	101059948	.
CTB-60B18.6	0	0.236757	0.513392	0.128279	0	0	.	chr19:4952	.	.	.	.	.	.	protein_co	ENSG0000	.	.
CTB-96E2.2	0	0	0	0	0	0	.	chr17:2669	.	.	.	.	.	.	protein_co	ENSG0000	.	.
CTBP1	55.1922	56.8444	49.4232	58.1351	52.9869	51.4722	C-terminal binding protein 1 [Source:HGNC Symbol;Acc:HGNC:24	chr4:12052	NM_00101	XM_01700	.	.	NP_00101	XP_005272	protein_co	ENSG0000	1487	2494
CTBP2	56.136	59.7176	48.6493	59.9838	61.2813	58.468	C-terminal binding protein 2 [Source:HGNC Symbol;Acc:HGNC:24	chr10:1266	NM_00108	XM_01153	.	.	NP_00107	XP_005269	protein_co	ENSG0000	1488	2495
CTBS	3.41546	3.39762	4.18286	3.49675	4.8706	6.16368	chitobiase [Source:HGNC Symbol;Acc:HGNC:2496]	chr1:85015	NM_00438	.	.	.	NP_00437	.	protein_co	ENSG0000	1486	2496
CTC1	7.37519	7.64922	8.14042	6.47024	5.19403	6.74099	CTS telomere maintenance complex component 1 [Source:HGNC	chr17:8128	NM_02509	XM_01152	.	.	NP_07937	XP_006721	protein_co	ENSG0000	80169	26169
CTC-260F20.3	2.74346	1.70624	1.98194	2.39817	3.13621	3.25269	.	chr19:1962	.	.	.	.	.	.	protein_co	ENSG0000	.	.
CTC-273B12.7	7.32E-26	0.388373	0.382017	1.17E-43	1.41E-50	5.9E-26	.	chr19:4894	.	.	.	.	.	.	protein_co	ENSG0000	.	.
CTC-281F24.5	0.62686	0.631404	0.559009	0.445756	0.621693	0.501577	.	chr17:6481	.	.	.	.	.	.	protein_co	ENSG0000	.	.
CTC-295J13.3	0	0	0	0	0	0	.	chr5:14760	.	XM_01153	.	.	.	XP_011536	protein_co	ENSG0000	105378220	.
CTC-360G5.8	9.49E-16	1.76E-19	3.79E-10	2.99E-07	1.09E-15	4.18E-31	.	chr19:3940	.	.	.	.	.	.	protein_co	ENSG0000	.	.
CTC-432M15.3	6.1E-07	0.000717	0.04136	8.64E-10	4.24E-11	5.12E-09	.	chr5:13075	.	.	.	.	.	.	protein_co	ENSG0000	.	.
CTC-435M10.3	0.000471	0.589423	1.42748	1.0184	2.34131	0.532255	.	chr19:4183	.	.	.	.	.	.	protein_co	ENSG0000	.	.

CTC-441N14.4	0.050278	0	0.043743	0.056927	0	0	.	chr5:12149	.	.	.	.	.	.	protein_co	ENSG00000	.	.
CTC-479C5.6	0.068102	0.069384	0	0	0	0	.	chr16:6804	NM_00128	.	.	.	NP_00126	.	protein_co	ENSG0000(	100131303	.
CTC-512J12.6	2.18E-86	3.7E-176	5.28E-33	1.16E-48	2.61E-16	3.5E-109	.	chr19:4483	.	.	.	.	.	.	protein_co	ENSG00000	.	.
CTC-534A2.2	1.7982	1.94006	2.22211	2.32883	2.59398	2.18499	.	chr5:64920	.	.	.	.	.	.	protein_co	ENSG00000	.	.
CTCF	20.1451	22.0147	17.6283	20.77	20.6891	20.4993	CCCTC-binding factor [Source:HGNC Symbol;Acc:HGNC:13723]	chr16:6759	NM_00119	XM_00525	.	.	NP_00117	XP_005255	protein_co	ENSG0000(	10664	13723
CTCFL	0	0.021074	0	0	0	0	CCCTC-binding factor like [Source:HGNC Symbol;Acc:HGNC:16234	chr20:5607	NM_00126	.	.	.	NP_00125	.	protein_co	ENSG0000(	140690	16234
CTD-2006C1.13	9.48E-35	3.37E-24	0.02574	8.26E-30	1.25E-50	2.16E-05	.	chr19:1203	.	.	.	.	.	.	protein_co	ENSG00000	.	.
CTD-2132N18.3	5.41E-08	2.83E-10	6E-20	1.61E-10	5.78E-34	1.77E-13	.	chr17:4026	.	.	.	.	.	.	protein_co	ENSG00000	.	.
CTD-2135J3.4	0.122323	0.230111	0.107413	9.79E-15	7.88E-06	0.206218	.	chr8:10581	.	.	.	.	.	.	protein_co	ENSG00000	.	.
CTD-2140B24.4	5.7E-128	3.2E-135	1.3E-214	1.32E-65	3.2E-109	8.7E-114	.	chr12:1337	.	.	.	.	.	.	protein_co	ENSG00000	.	.
CTD-2192J16.17	0.087471	0.037452	0.061378	0	0.019536	0.068236	.	chr19:1257	.	.	.	.	.	.	protein_co	ENSG00000	.	.
CTD-2192J16.24	6.3E-142	5.54E-41	6.8E-37	3.94E-50	2.35E-33	9.5E-45	.	chr19:1275	.	.	.	.	.	.	protein_co	ENSG00000	.	.
CTD-2207O23.12	4.91E-06	4.35E-05	7.33E-05	1.8E-06	1.96E-06	0.26537	.	chr19:7562	.	.	.	.	.	.	protein_co	ENSG00000	.	.
CTD-2207O23.3	4.73E-05	0.000524	8.48E-05	3.54E-05	5.31E-05	7.62E-05	.	chr19:7413	.	.	.	.	.	.	protein_co	ENSG00000	.	.
CTD-2287O16.3	1.87E-81	3.1E-14	8.57E-06	0.705693	3E-110	4.1E-196	.	chr5:11529	.	.	.	.	.	.	protein_co	ENSG00000	.	.
CTD-2313N18.7	0	0	0	0	0	0	.	chr11:7158	NM_00124	.	.	.	NP_00122	.	protein_co	ENSG0000(	100129216	.
CTD-2331H12.8	1.27817	1.10725	1.98111	0.08713	2.08423	1.62643	.	chr19:5337	.	.	.	.	.	.	protein_co	ENSG00000	.	.
CTD-2370N5.3	0	0	0	0	0	0	.	chr17:2942	.	.	.	.	.	.	protein_co	ENSG00000	.	.
CTD-2410N18.5	1.81E-06	3.8976	1.43E-05	2.89008	5.12E-11	1.66E-08	.	chr5:13348	.	.	.	.	.	.	protein_co	ENSG00000	.	.
CTD-2510F5.6	2.19E-40	4.68E-56	4.81E-36	6.8E-266	1.5E-27	8.54E-33	.	chr17:5723	.	.	.	.	.	.	protein_co	ENSG00000	.	.
CTD-2521M24.10	2.91E-05	0.198991	1.84E-06	2.89E-07	9.78E-09	3.96E-06	.	chr19:1754	.	.	.	.	.	.	protein_co	ENSG00000	.	.
CTD-2521M24.13	0	0	0	0	0	0	.	chr19:1750	.	.	.	.	.	XP_011507	protein_co	ENSG0000(	105372343	.
CTD-2537I9.19	0.07561	0	0	0	0	0	.	chr19:5597	.	.	.	.	.	.	protein_co	ENSG00000	.	.
CTD-2545M3.6	2.08E-80	1.22E-54	2E-103	2.1E-279	1.11E-15	0	.	chr19:5088	.	.	.	.	.	.	protein_co	ENSG00000	.	.
CTD-2568A17.1	0	0	0.603514	0	0	0	.	chr19:5128	.	.	.	.	.	.	protein_co	ENSG00000	.	.
CTD-2583A14.10	2.9E-159	0	0.135917	0	0	0.328664	.	chr19:5828	.	.	.	.	.	.	protein_co	ENSG00000	.	.
CTD-3074O7.11	7.21E-05	6.55E-05	3.58E-05	0.000146	5.17E-07	2.76E-06	.	chr11:6624	.	.	.	.	.	.	protein_co	ENSG00000	.	.
CTD-3088G3.8	0.331631	0.346796	0.605941	0.587502	0.458431	0.61078	.	chr16:1146	.	XM_01702	.	.	.	XP_016855	protein_co	ENSG0000(	400499	.
CTD-3105H18.16	0.215409	0.181488	5.01E-13	7.77E-14	4.29E-29	3.22E-32	.	chr19:1250	.	.	.	.	.	.	protein_co	ENSG00000	.	.
CTD-3214H19.16	6.07E-08	1.12E-05	4.21E-06	4.39E-07	1.06E-06	1.4E-07	.	chr19:7741	.	.	.	.	.	.	protein_co	ENSG00000	.	.
CTDNEP1	75.1081	72.6956	60.4588	79.7036	69.0925	65.5783	CTD nuclear envelope phosphatase 1 [Source:HGNC Symbol;Acc:I	chr17:7146	NM_00114	.	.	.	NP_00113	.	protein_co	ENSG0000(	23399	19085
CTDP1	11.9697	11.7094	11.0735	13.3115	12.976	9.2389	CTD phosphatase subunit 1 [Source:HGNC Symbol;Acc:HGNC:249	chr18:7743	NM_00120	XM_01702	.	.	NP_00118	XP_011524	protein_co	ENSG0000(	9150	2498
CTDSP1	38.741	38.1387	31.16	32.4103	33.257	37.4839	CTD small phosphatase 1 [Source:HGNC Symbol;Acc:HGNC:21614	chr2:21926	NM_00120	.	.	.	NP_00119	.	protein_co	ENSG0000(	58190	21614
CTDSP2	35.2006	33.4814	34.427	22.1772	21.8467	34.0694	CTD small phosphatase 2 [Source:HGNC Symbol;Acc:HGNC:17077	chr12:5821	NM_00573	XM_00526	.	.	NP_00572	XP_005268	protein_co	ENSG0000(	10106	17077
CTDSPL	13.7977	13.9742	14.5954	12.4876	12.2699	12.2516	CTD small phosphatase like [Source:HGNC Symbol;Acc:HGNC:168	chr3:37903	NM_00100	.	.	.	NP_00100	XP_016861	protein_co	ENSG0000(	10217	16890
CTDSPL2	13.0129	10.7827	14.0429	12.2381	16.1595	18.5814	CTD small phosphatase like 2 [Source:HGNC Symbol;Acc:HGNC:26	chr15:4471	NM_01639	XM_01152	.	.	NP_05748	XP_005254	protein_co	ENSG0000(	51496	26936
CTF1	2.94456	2.53464	2.17557	1.25892	0.671767	1.81678	cardiotrophin 1 [Source:HGNC Symbol;Acc:HGNC:2499]	chr16:3090	NM_00114	XM_01154	.	.	NP_00113	XP_011544	protein_co	ENSG0000(	1489	2499
CTGF	1.66332	1.47091	1.01221	0.416279	0.650421	1.26205	connective tissue growth factor [Source:HGNC Symbol;Acc:HGNC	chr6:13226	NM_00190	.	.	.	NP_00189	.	protein_co	ENSG0000(	1490	2500
CTH	8.11381	7.08033	8.25107	6.48874	9.27176	12.9552	cystathionine gamma-lyase [Source:HGNC Symbol;Acc:HGNC:250	chr1:70876	NM_00119	XM_00527	.	.	NP_00117	XP_005270	protein_co	ENSG0000(	1491	2501
CTHRC1	8.58122	8.14513	9.96878	6.25942	4.99505	6.74232	collagen triple helix repeat containing 1 [Source:HGNC Symbol;Ac	chr8:10438	NM_00125	.	.	.	NP_00124	.	protein_co	ENSG0000(	115908	18831
CTIF	4.33954	4.63262	6.46247	3.46276	3.74128	5.14431	cap binding complex dependent translation initiation factor [Sour	chr18:4606	NM_00114	XM_01702	.	.	NP_00113	XP_005258	protein_co	ENSG0000(	9811	23925
CTLA4	0.042628	0	0	0	0	0	cytotoxic T-lymphocyte associated protein 4 [Source:HGNC Symb	chr2:20473	NM_00103	.	.	.	NP_00103	.	protein_co	ENSG0000(	1493	2505
CTNNA1	92.749	98.1181	99.7762	52.1739	47.6553	59.355	catenin alpha 1 [Source:HGNC Symbol;Acc:HGNC:2509]	chr5:13808	NM_00129	XM_01154	.	.	NP_00127	XP_005271	protein_co	ENSG0000(	1495	2509
CTNNA2	0	0	0	0	0	0	catenin alpha 2 [Source:HGNC Symbol;Acc:HGNC:2510]	chr2:79412	NM_00116	XM_01700	.	.	NP_00115	XP_011530	protein_co	ENSG0000(	1496	2510
CTNNA3	0.007858	0	0.138468	0	0	0	catenin alpha 3 [Source:HGNC Symbol;Acc:HGNC:2511]	chr10:6767	NM_00112	XM_01701	.	.	NP_00112	XP_016871	protein_co	ENSG0000(	29119	2511
CTNNAL1	31.191	23.6355	31.7012	27.4898	35.7158	41.1757	catenin alpha like 1 [Source:HGNC Symbol;Acc:HGNC:2512]	chr9:11169	NM_00128	XM_01151	.	.	NP_00127	XP_005252	protein_co	ENSG0000(	8727	2512
CTNNB1	159.395	165.27	164.034	169.08	164.422	158.713	catenin beta 1 [Source:HGNC Symbol;Acc:HGNC:2514]	chr3:41236	NM_00109	XM_01700	.	.	NP_00109	XP_005264	protein_co	ENSG0000(	1499	2514
CTNNBIP1	16.7335	15.533	17.1198	20.8481	17.4189	18.4972	catenin beta interacting protein 1 [Source:HGNC Symbol;Acc:HGN	chr1:99083	NM_00101	XM_01700	.	.	NP_00101	XP_016857	protein_co	ENSG0000(	56998	16913
CTNNBL1	23.9496	20.3122	17.7122	23.837	24.6372	16.7936	catenin beta like 1 [Source:HGNC Symbol;Acc:HGNC:15879]	chr20:3632	NM_00128	XM_01152	.	.	NP_00126	XP_011527	protein_co	ENSG0000(	56259	15879
CTNND1	57.1142	60.5919	56.8722	53.9822	58.4303	61.1565	catenin delta 1 [Source:HGNC Symbol;Acc:HGNC:2515]	chr11:5752	NM_00108	.	.	.	NP_00107	.	protein_co	ENSG0000(	1500	2515
CTNND2	0	0	0	0	0	0	catenin delta 2 [Source:HGNC Symbol;Acc:HGNC:2516]	chr5:10971	NM_00128	XM_01151	.	.	NP_00127	XP_005248	protein_co	ENSG0000(	1501	2516
CTNS	7.88516	7.57477	6.81716	7.31432	5.31198	4.26729	cystinosin, lysosomal cystine transporter [Source:HGNC Symbol;A	chr17:3539	NM_00103	XM_01152	.	.	NP_00102	XP_005256	protein_co	ENSG0000(	1497	2518
CTPS1	30.7118	30.5613	28.6269	33.5604	32.5916	21.0161	CTP synthase 1 [Source:HGNC Symbol;Acc:HGNC:2519]	chr1:41445	NM_00190	XM_01154	.	.	NP_00189	XP_005270	protein_co	ENSG0000(	1503	2519
CTPS2	5.88959	6.00704	5.82646	7.36499	6.8457	6.6521	CTP synthase 2 [Source:HGNC Symbol;Acc:HGNC:2520]	chrX:16606	NM_00114	XM_01154	.	.	NP_00113	XP_005274	protein_co	ENSG0000(	56474	2520
CTR9	34.3467	37.4155	43.7622	40.4283	42.1001	34.241	CTR9 homolog, Paf1/RNA polymerase II complex component [Sou	chr11:1077	NM_01463	.	.	.	NP_05544	.	protein_co	ENSG0000(	9646	16850
CTRB1	0	0	0	0	0	0	chymotrypsinogen B1 [Source:HGNC Symbol;Acc:HGNC:2521]	chr16:7525	NM_00190	.	.	.	NP_00189	.	protein_co	ENSG0000(	1504	2521
CTRB2	0	0	0	0	0	0	chymotrypsinogen B2 [Source:HGNC Symbol;Acc:HGNC:2522]	chr16:7523	NM_00102	.	.	.	NP_00102	.	protein_co	ENSG0000(	440387	2522
CTRC	0	0.172199	0	0	0	0.096684	chymotrypsin C [Source:HGNC Symbol;Acc:HGNC:2523]	chr1:15764	NM_00727	XM_01154	.	.	NP_00920	XP_011538	protein_co	ENSG0000(	11330	2523
CTRL	2.86103	2.23042	2.46362	3.52053	3.1641	0.953952	chymotrypsin like [Source:HGNC Symbol;Acc:HGNC:2524]	chr16:6792	NM_00190	.	.	.	NP_00189	.	protein_co	ENSG0000(	1506	2524

CTSA	63.6422	59.5669	52.931	61.5616	49.9836	45.1574	cathepsin A [Source:HGNC Symbol;Acc:HGNC:9251]	chr20:4451	NM_00030	.	.	.	NP_00029	.	protein_co	ENSG0000	5476	9251
CTSB	98.2138	101.02	112.264	91.0458	88.1366	92.1842	cathepsin B [Source:HGNC Symbol;Acc:HGNC:2527]	chr8:11700	NM_00190	XM_01154	.	.	NP_00189	XP_006716	protein_co	ENSG0000	1508	2527
CTSC	33.5676	30.5267	31.2893	40.8576	38.5935	34.305	cathepsin C [Source:HGNC Symbol;Acc:HGNC:2528]	chr11:8802	NM_00111	.	.	.	NP_00110	.	protein_co	ENSG0000	1075	2528
CTSD	81.5747	85.5766	64.531	61.0127	54.3802	53.5574	cathepsin D [Source:HGNC Symbol;Acc:HGNC:2529]	chr11:1753	NM_00190	.	.	.	NP_00190	.	protein_co	ENSG0000	1509	2529
CTSE	0	0.078188	0.064837	0	0.042179	0.077186	cathepsin E [Source:HGNC Symbol;Acc:HGNC:2530]	chr1:20631	NM_00191	XM_01150	.	.	NP_00190	XP_011507	protein_co	ENSG0000	1510	2530
CTSF	0	0	0	0	0	0	cathepsin F [Source:HGNC Symbol;Acc:HGNC:2531]	chr11:6633	NM_00379	XM_01154	.	.	NP_00378	XP_011543	protein_co	ENSG0000	8722	2531
CTSG	0	0	0	0	0	0	cathepsin G [Source:HGNC Symbol;Acc:HGNC:2532]	chr14:2504	NM_00191	XM_01153	.	.	NP_00190	XP_011534	protein_co	ENSG0000	1511	2532
CTSH	12.169	13.7412	17.0419	12.4767	7.89248	10.435	cathepsin H [Source:HGNC Symbol;Acc:HGNC:2535]	chr15:7921	NM_00131	XM_00525	.	.	NP_00130	XP_005254	protein_co	ENSG0000	1512	2535
CTSK	0.967984	1.13246	1.52728	1.85451	1.265	1.65309	cathepsin K [Source:HGNC Symbol;Acc:HGNC:2536]	chr1:15076	NM_00039	.	.	.	NP_00038	.	protein_co	ENSG0000	1513	2536
CTSL	50.1228	46.9104	38.4565	39.888	36.7921	39.1498	cathepsin L [Source:HGNC Symbol;Acc:HGNC:2537]	chr9:90340	NM_00125	XM_01151	.	.	NP_00124	XP_005251	protein_co	ENSG0000	1514	2537
CTSO	0.564451	1.38581	1.22401	0.541028	0.621118	1.03009	cathepsin O [Source:HGNC Symbol;Acc:HGNC:2542]	chr4:15684	NM_00133	.	.	.	NP_00132	.	protein_co	ENSG0000	1519	2542
CTSS	0.190806	0.123406	0.100172	0.060397	0.060529	0.109138	cathepsin S [Source:HGNC Symbol;Acc:HGNC:2545]	chr1:15070	NM_00119	.	.	.	NP_00118	.	protein_co	ENSG0000	1520	2545
CTSV	15.4346	14.7596	13.2935	15.0632	16.6653	19.8099	cathepsin V [Source:HGNC Symbol;Acc:HGNC:2538]	chr9:99791	NM_00120	.	.	.	NP_00118	.	protein_co	ENSG0000	1515	2538
CTSW	0	0	0.047376	0	0	0.059337	cathepsin W [Source:HGNC Symbol;Acc:HGNC:2546]	chr11:6564	NM_00133	.	.	.	NP_00132	.	protein_co	ENSG0000	1521	2546
CTSZ	80.3633	81.0995	73.4864	69.9256	62.8406	70.5994	cathepsin Z [Source:HGNC Symbol;Acc:HGNC:2547]	chr20:5757	NM_00133	.	.	.	NP_00132	.	protein_co	ENSG0000	1522	2547
CTTN	68.1727	68.5413	70.0507	55.5498	55.2708	45.2984	cortactin [Source:HGNC Symbol;Acc:HGNC:3338]	chr11:7024	NM_00118	XM_00671	.	.	NP_00117	XP_006718	protein_co	ENSG0000	2017	3338
CTTNBP2	1.72199	1.77763	1.84918	1.8873	2.04246	1.31024	cortactin binding protein 2 [Source:HGNC Symbol;Acc:HGNC:156	chr7:11735	NM_03342	XM_01151	.	.	NP_21949	XP_005250	protein_co	ENSG0000	83992	15679
CTTNBP2NL	5.64251	5.82668	6.20395	6.04196	5.95629	7.38923	CTTNBP2 N-terminal like [Source:HGNC Symbol;Acc:HGNC:25330	chr1:11293	NM_01870	XM_01154	.	.	NP_06117	XP_011540	protein_co	ENSG0000	55917	25330
CTU1	6.06297	6.10651	5.58531	6.37961	3.22912	4.76623	cytosolic thiouridylase subunit 1 [Source:HGNC Symbol;Acc:HGN	chr19:5160	NM_14523	.	.	.	NP_66027	.	protein_co	ENSG0000	90353	29590
CTU2	38.9486	36.947	33.9271	46.6516	34.894	25.2578	cytosolic thiouridylase subunit 2 [Source:HGNC Symbol;Acc:HGN	chr16:8887	NM_00101	XM_01152	.	.	NP_00101	.	protein_co	ENSG0000	348180	28005
CTXN1	50.8594	48.6235	59.0916	52.1784	44.0728	39.3932	cortexin 1 [Source:HGNC Symbol;Acc:HGNC:31108]	chr19:7989	NM_20683	.	.	.	NP_99666	.	protein_co	ENSG0000	404217	31108
CTXN2	0	0	0	0	0	0	cortexin 2 [Source:HGNC Symbol;Acc:HGNC:31109]	chr15:4848	NM_00114	XM_01152	.	.	NP_00113	XP_005254	protein_co	ENSG0000	399697	31109
CTXN3	0	0	0	0	0	0	cortexin 3 [Source:HGNC Symbol;Acc:HGNC:31110]	chr5:12698	NM_00104	.	.	.	NP_00104	.	protein_co	ENSG0000	613212	31110
CUBN	0.074127	0.13175	0.138019	0.236788	0.274005	0.378586	cubilin [Source:HGNC Symbol;Acc:HGNC:2548]	chr10:1686	NM_00108	.	.	.	NP_00107	.	protein_co	ENSG0000	8029	2548
CUEDC1	25.8926	25.2277	27.9828	15.2867	14.3339	12.7137	CUE domain containing 1 [Source:HGNC Symbol;Acc:HGNC:3135	chr17:5593	NM_00127	XM_01152	.	.	NP_00125	XP_006721	protein_co	ENSG0000	404093	31350
CUEDC2	68.8107	66.4228	72.5069	89.6059	69.6319	62.7576	CUE domain containing 2 [Source:HGNC Symbol;Acc:HGNC:2835	chr10:1041	NM_02404	.	.	.	NP_07694	.	protein_co	ENSG0000	79004	28352
CUL1	34.858	34.8731	30.114	37.124	37.5867	31.5767	cullin 1 [Source:HGNC Symbol;Acc:HGNC:2551]	chr7:14839	NM_00359	XM_01151	.	.	NP_00358	XP_005250	protein_co	ENSG0000	8454	2551
CUL2	12.7235	11.7458	14.1867	14.0675	17.8391	19.7837	cullin 2 [Source:HGNC Symbol;Acc:HGNC:2552]	chr10:3529	NM_00119	XM_01151	.	.	NP_00118	XP_011518	protein_co	ENSG0000	8453	2552
CUL3	25.6838	28.3388	25.5258	29.5104	31.5656	31.5192	cullin 3 [Source:HGNC Symbol;Acc:HGNC:2553]	chr2:22533	NM_00125	XM_01151	.	.	NP_00124	XP_006712	protein_co	ENSG0000	8452	2553
CUL4A	38.2381	38.724	36.1001	34.5625	33.8798	31.3639	cullin 4A [Source:HGNC Symbol;Acc:HGNC:2554]	chr13:1138	NM_00100	XM_01153	.	.	NP_00100	XP_011535	protein_co	ENSG0000	8451	2554
CUL4B	13.9378	13.6192	15.6676	14.8498	19.8193	21.2281	cullin 4B [Source:HGNC Symbol;Acc:HGNC:2555]	chrX:11965	NM_00107	XM_01153	.	.	NP_00107	XP_005262	protein_co	ENSG0000	8450	2555
CUL5	8.24236	9.45728	9.40172	8.74319	11.8332	12.0267	cullin 5 [Source:HGNC Symbol;Acc:HGNC:2556]	chr11:1078	NM_00347	XM_00527	.	.	NP_00346	XP_005271	protein_co	ENSG0000	8065	2556
CUL7	8.95939	9.88133	11.7188	8.10816	7.36944	9.4896	cullin 7 [Source:HGNC Symbol;Acc:HGNC:21024]	chr6:43005	NM_00116	XM_01701	.	.	NP_00116	XP_005249	protein_co	ENSG0000	9820	21024
CUL9	4.66943	4.32054	5.83976	3.7249	3.71049	3.86637	cullin 9 [Source:HGNC Symbol;Acc:HGNC:15982]	chr6:43149	NM_01508	XM_01151	.	.	NP_05590	XP_011512	protein_co	ENSG0000	23113	15982
CUTA	151.586	144.444	138.592	173.722	135.686	132.369	cutA divalent cation tolerance homolog [Source:HGNC Symbol;A	chr6:33378	NM_00101	.	.	.	NP_00101	.	protein_co	ENSG0000	51596	21101
CUTC	34.0064	31.5183	29.017	42.0429	44.0701	37.1472	cutC copper transporter [Source:HGNC Symbol;Acc:HGNC:24271]	chr10:1014	NM_01596	.	.	.	NP_05704	.	protein_co	ENSG0000	51076	24271
CUX1	23.4958	25.5895	24.9847	25.1945	21.834	20.2931	cut like homeobox 1 [Source:HGNC Symbol;Acc:HGNC:2557]	chr7:10145	NM_00120	XM_01151	.	.	NP_00118	XP_005250	protein_co	ENSG0000	1523	2557
CUX2	0	0	0.01839	0	0	0	cut like homeobox 2 [Source:HGNC Symbol;Acc:HGNC:19347]	chr12:1114	NM_01526	XM_01701	.	.	NP_05608	XP_011536	protein_co	ENSG0000	23316	19347
CUZD1	0.544954	0.627809	0.406658	0.46544	0.646062	0.417237	CUB and zona pellucida like domains 1 [Source:HGNC Symbol;Ac	chr10:1245	NM_02203	.	.	.	NP_07131	.	protein_co	ENSG0000	50624	17937
CWC15	31.3626	26.884	24.9131	30.8845	28.37	26.4366	CWC15 spliceosome-associated protein [Source:HGNC Symbol;A	chr11:9469	NM_01640	XM_01154	.	.	NP_05748	XP_011541	protein_co	ENSG0000	51503	26939
CWC22	11.1999	11.2936	10.1744	11.1494	13.1334	13.0427	CWC22 homolog, spliceosome-associated protein [Source:HGNC	chr2:18080	NM_02094	XM_00524	.	.	NP_06599	XP_005246	protein_co	ENSG0000	57703	29322
CWC25	8.16904	7.94079	7.74076	8.61686	7.24349	6.20159	CWC25 spliceosome associated protein homolog [Source:HGNC S	chr17:3695	NM_01774	.	.	.	NP_06021	.	protein_co	ENSG0000	54883	25989
CWC27	14.7603	14.5122	13.5535	16.5166	17.0204	16.9716	CWC27 spliceosome associated protein homolog [Source:HGNC S	chr5:64064	NM_00129	XM_01154	.	.	NP_00128	XP_011541	protein_co	ENSG0000	10283	10664
CWF19L1	25.5881	24.3107	19.5023	33.5117	26.7368	22.1977	CWF19-like 1, cell cycle control (S. pombe) [Source:HGNC Symbo	chr10:1019	NM_00130	.	.	.	NP_00129	.	protein_co	ENSG0000	55280	25613
CWF19L2	2.48796	2.09727	1.9244	2.17414	2.86045	2.25774	CWF19-like 2, cell cycle control (S. pombe) [Source:HGNC Symbo	chr11:1071	NM_15243	.	.	.	NP_68964	.	protein_co	ENSG0000	143884	26508
CWH43	0	0	0	0	0	0	cell wall biogenesis 43 C-terminal homolog [Source:HGNC Symbo	chr4:48988	NM_00128	XM_01151	.	.	NP_00127	XP_011512	protein_co	ENSG0000	80157	26133
CX3CL1	0	0	0	0	0	0	C-X3-C motif chemokine ligand 1 [Source:HGNC Symbol;Acc:HGN	chr16:5740	NM_00130	.	.	.	NP_00129	.	protein_co	ENSG0000	6376	10647
CX3CR1	0.025219	0.02675	0.065968	0	0.028458	0.025733	C-X3-C motif chemokine receptor 1 [Source:HGNC Symbol;Acc:H	chr3:39304	NM_00117	.	.	.	NP_00116	.	protein_co	ENSG0000	1524	2558
CXADR	9.80416	7.70092	9.24931	13.4511	16.0206	17.501	coxsackie virus and adenovirus receptor [Source:HGNC Symbol;A	chr21:1888	NM_00120	XM_01152	.	.	NP_00119	XP_011527	protein_co	ENSG0000	1525	2559
CXCL1	21.6588	25.3172	19.5045	1.76124	1.3917	2.09806	C-X-C motif chemokine ligand 1 [Source:HGNC Symbol;Acc:HGNC	chr4:74735	NM_00151	.	.	.	NP_00150	.	protein_co	ENSG0000	2919	4602
CXCL10	3.06493	1.73149	0	1.83587	2.4403	2.00969	C-X-C motif chemokine ligand 10 [Source:HGNC Symbol;Acc:HGN	chr4:76932	NM_00156	.	.	.	NP_00155	.	protein_co	ENSG0000	3627	10637
CXCL11	0.708581	0.65336	0.047598	0.501023	0.179158	0.312508	C-X-C motif chemokine ligand 11 [Source:HGNC Symbol;Acc:HGN	chr4:76932	NM_00130	.	.	.	NP_00128	.	protein_co	ENSG0000	6373	10638
CXCL12	0	0	0	0.026159	0	0	C-X-C motif chemokine ligand 12 [Source:HGNC Symbol;Acc:HGN	chr10:4486	NM_00060	.	.	.	NP_00060	.	protein_co	ENSG0000	6387	10672
CXCL13	0.071529	0.154921	0.062683	0	0	0	C-X-C motif chemokine ligand 13 [Source:HGNC Symbol;Acc:HGN	chr4:78432	NM_00641	XM_00671	.	.	NP_00641	XP_006714	protein_co	ENSG0000	10563	10639
CXCL14	0	0	0	0	0	0	C-X-C motif chemokine ligand 14 [Source:HGNC Symbol;Acc:HGN	chr5:13490	NM_00488	.	.	.	NP_00487	.	protein_co	ENSG0000	9547	10640
CXCL16	38.8934	37.9938	37.1867	24.8018	23.8659	23.5419	C-X-C motif chemokine ligand 16 [Source:HGNC Symbol;Acc:HGN	chr17:4634	NM_00110	.	.	.	NP_00109	.	protein_co	ENSG0000	58191	16642
CXCL17	0.075351	0.235849	0.130896	0.167416	0.169569	0	C-X-C motif chemokine ligand 17 [Source:HGNC Symbol;Acc:HGN	chr19:4293	NM_19847	.	.	.	NP_94087	.	protein_co	ENSG0000	284340	19232
CXCL2	7.5232	8.16335	3.8663	1.60966	0.805729	0.72689	C-X-C motif chemokine ligand 2 [Source:HGNC Symbol;Acc:HGNC	chr4:74962	NM_00208	.	.	.	NP_00208	.	protein_co	ENSG0000	2920	4603
CXCL3	7.82945	8.32765	2.14072	0.595688	0.787323	0.964484	C-X-C motif chemokine ligand 3 [Source:HGNC Symbol;Acc:HGNC	chr4:74902	NM_00209	.	.	.	NP_00208	.	protein_co	ENSG0000	2921	4604

CXCL5	0	0	0	0	0	0	C-X-C motif chemokine ligand 5 [Source:HGNC Symbol;Acc:HGNC	chr4:74861	NM_00299	.	.	.	NP_00298	.	protein_co	ENSG0000	6374	10642
CXCL6	0	0	0	0	0	0	C-X-C motif chemokine ligand 6 [Source:HGNC Symbol;Acc:HGNC	chr4:74702	NM_00299	.	.	.	NP_00298	.	protein_co	ENSG0000	6372	10643
CXCL8	24.9064	25.8224	12.7431	3.96558	3.55558	5.1809	C-X-C motif chemokine ligand 8 [Source:HGNC Symbol;Acc:HGNC	chr4:74606	NM_00058	.	.	.	NP_00057	.	protein_co	ENSG0000	3576	6025
CXCL9	0	0	0	0	0	0	C-X-C motif chemokine ligand 9 [Source:HGNC Symbol;Acc:HGNC	chr4:76922	NM_00241	.	.	.	NP_00240	.	protein_co	ENSG0000	4283	7098
CXCR1	0.029785	0	0	0.065457	0.101324	0.093395	C-X-C motif chemokine receptor 1 [Source:HGNC Symbol;Acc:HG	chr2:21902	NM_00063	.	.	.	NP_00062	.	protein_co	ENSG0000	3577	6026
CXCR2	0.101993	0.106222	0.110556	0.028365	0.057378	0.339429	C-X-C motif chemokine receptor 2 [Source:HGNC Symbol;Acc:HG	chr2:21899	NM_00116	XM_01700	.	.	NP_00116	XP_005246	protein_co	ENSG0000	3579	6027
CXCR3	0	0	0	0	0	0	C-X-C motif chemokine receptor 3 [Source:HGNC Symbol;Acc:HG	chrX:70835	NM_00114	XM_01702	.	.	NP_00113	XP_016884	protein_co	ENSG0000	2833	4540
CXCR4	1.11405	0.441924	1.09945	0.587619	0.656641	0.486688	C-X-C motif chemokine receptor 4 [Source:HGNC Symbol;Acc:HG	chr2:13687	NM_00100	.	.	.	NP_00100	.	protein_co	ENSG0000	7852	2561
CXCR5	0.065874	0.04924	0.014246	0.035434	0.019032	0	C-X-C motif chemokine receptor 5 [Source:HGNC Symbol;Acc:HG	chr11:1187	NM_00171	.	.	.	NP_00170	.	protein_co	ENSG0000	643	1060
CXCR6	0	0	0	0	0	0	C-X-C motif chemokine receptor 6 [Source:HGNC Symbol;Acc:HG	chr3:45959	NM_00656	XM_01153	.	.	NP_00655	XP_005264	protein_co	ENSG0000	10663	16647
CXorf21	0	0	0	0	0	0	chromosome X open reading frame 21 [Source:HGNC Symbol;Acc	chrX:30576	NM_02515	.	.	.	NP_07943	.	protein_co	ENSG0000	80231	25667
CXorf23	1.66177	1.51774	2.21079	0.867717	1.89249	1.99906	chromosome X open reading frame 23 [Source:HGNC Symbol;Acc	chrX:19930	NM_19827	XM_01154	.	.	NP_93802	XP_005274	protein_co	ENSG0000	256643	27413
CXorf36	0	0	0	0	0	0	chromosome X open reading frame 36 [Source:HGNC Symbol;Acc	chrX:45007	NM_02468	.	.	.	NP_07896	.	protein_co	ENSG0000	79742	25866
CXorf38	6.55338	6.415	6.71718	4.51531	5.01275	5.81053	chromosome X open reading frame 38 [Source:HGNC Symbol;Acc	chrX:40486	NM_14497	XM_00672	.	.	NP_65940	XP_005272	protein_co	ENSG0000	159013	28589
CXorf40A	4.39113	5.34276	4.65473	5.32525	3.53271	3.72532	chromosome X open reading frame 40A [Source:HGNC Symbol;A	chrX:14862	NM_00117	XM_01153	.	.	NP_00116	XP_005262	protein_co	ENSG0000	91966	28089
CXorf40B	15.7872	17.0523	16.1939	17.2113	16.4123	13.8161	chromosome X open reading frame 40B [Source:HGNC Symbol;A	chrX:14909	NM_00101	XM_01153	.	.	NP_00101	XP_005274	protein_co	ENSG0000	541578	17402
CXorf49	0	0	0	0	0	0	chromosome X open reading frame 49 [Source:HGNC Symbol;Acc	chrX:70934	NM_00114	.	.	.	NP_00113	.	protein_co	ENSG0000	100130361	30891
CXorf49B	0	0	0	0	0	0	chromosome X open reading frame 49B [Source:HGNC Symbol;A	chrX:70983	NM_00114	.	.	.	NP_00113	.	protein_co	ENSG0000	100132994	34229
CXorf51A	0	0	0	0	0	0	chromosome X open reading frame 51A [Source:HGNC Symbol;A	chrX:14589	NM_00114	.	.	.	NP_00113	.	protein_co	ENSG0000	100129239	30533
CXorf51B	0	0	0	0	0	0	chromosome X open reading frame 51B [Source:HGNC Symbol;A	chrX:14589	NM_00124	.	.	.	NP_00123	.	protein_co	ENSG0000	100133053	42787
CXorf56	18.6471	20.0063	16.8397	20.8124	19.052	19.0133	chromosome X open reading frame 56 [Source:HGNC Symbol;Acc	chrX:11867	NM_00117	.	.	.	NP_00116	.	protein_co	ENSG0000	63932	26239
CXorf57	4.10866	3.49031	3.23599	3.82642	4.732	4.80908	chromosome X open reading frame 57 [Source:HGNC Symbol;Acc	chrX:10585	NM_00118	.	.	.	NP_00117	.	protein_co	ENSG0000	55086	25486
CXorf58	0	0.058001	0.094546	0.12526	0	0	chromosome X open reading frame 58 [Source:HGNC Symbol;Acc	chrX:23851	NM_00116	XM_01154	.	.	NP_00116	XP_011543	protein_co	ENSG0000	254158	26356
CXorf65	0	0	0.125602	0	0	0	chromosome X open reading frame 65 [Source:HGNC Symbol;Acc	chrX:70323	NM_00102	XM_00526	.	.	NP_00102	XP_005262	protein_co	ENSG0000	158830	33713
CXorf66	0	0	0	0	0	0	chromosome X open reading frame 66 [Source:HGNC Symbol;Acc	chrX:13903	NM_00101	.	.	.	NP_00101	.	protein_co	ENSG0000	347487	33743
CXorf67	0.063535	0	0	0	0	0.07071	chromosome X open reading frame 67 [Source:HGNC Symbol;Acc	chrX:51149	NM_20340	.	.	.	NP_98195	.	protein_co	ENSG0000	340602	33738
CXXC1	26.6036	25.9525	22.1503	28.4449	24.749	17.989	CXXC finger protein 1 [Source:HGNC Symbol;Acc:HGNC:24343]	chr18:4780	NM_00110	XM_01152	.	.	NP_00109	XP_011524	protein_co	ENSG0000	30827	24343
CXXC4	0.347017	0.331737	0.474163	0.374845	0.811268	0.515628	CXXC finger protein 4 [Source:HGNC Symbol;Acc:HGNC:24593]	chr4:10538	NM_02521	XM_01700	.	.	NP_07948	XP_011530	protein_co	ENSG0000	80319	24593
CXXC5	29.3091	31.0278	26.5537	28.104	26.0303	20.2694	CXXC finger protein 5 [Source:HGNC Symbol;Acc:HGNC:26943]	chr5:13902	NM_01646	XM_00671	.	.	NP_00130	XP_005268	protein_co	ENSG0000	51523	26943
CYB561	47.1735	50.3118	46.8881	48.3071	45.9541	35.4301	cytochrome b561 [Source:HGNC Symbol;Acc:HGNC:2571]	chr17:6150	NM_00101	XM_01702	.	.	NP_00101	XP_005257	protein_co	ENSG0000	1534	2571
CYB561A3	21.3037	22.5446	20.2131	26.5976	22.0892	18.6562	cytochrome b561 family member A3 [Source:HGNC Symbol;Acc:H	chr11:6106	NM_00116	XM_01154	.	.	NP_00115	XP_011543	protein_co	ENSG0000	220002	23014
CYB561D1	5.39796	5.84743	5.63594	3.38024	4.38223	3.18556	cytochrome b561 family member D1 [Source:HGNC Symbol;Acc:H	chr1:11003	NM_00113	XM_01700	.	.	NP_00112	XP_005270	protein_co	ENSG0000	284613	26804
CYB561D2	15.5017	17.6624	8.16923	16.2135	12.8709	5.51083	cytochrome b561 family member D2 [Source:HGNC Symbol;Acc:H	chr3:50384	NM_00129	.	.	.	NP_00127	.	protein_co	ENSG0000	11068	30253
CYB5A	23.1586	26.8419	32.2637	25.7115	16.942	15.2753	cytochrome b5 type A [Source:HGNC Symbol;Acc:HGNC:2570]	chr18:7191	NM_00119	XM_01152	.	.	NP_00117	XP_011524	protein_co	ENSG0000	1528	2570
CYB5B	341.709	344.646	690.351	450.811	398.89	485.594	cytochrome b5 type B [Source:HGNC Symbol;Acc:HGNC:24374]	chr16:6945	NM_03057	.	.	.	NP_08505	.	protein_co	ENSG0000	80777	24374
CYB5D1	17.6659	17.792	16.7469	12.3927	15.8264	12.4059	cytochrome b5 domain containing 1 [Source:HGNC Symbol;Acc:H	chr17:7760	NM_14460	XM_00525	.	.	NP_65320	XP_005256	protein_co	ENSG0000	124637	26516
CYB5D2	7.96456	6.69119	7.10441	4.51604	3.87953	5.94315	cytochrome b5 domain containing 2 [Source:HGNC Symbol;Acc:H	chr17:4046	NM_00125	XM_01152	.	.	NP_00124	XP_005256	protein_co	ENSG0000	124936	28471
CYB5R1	11.0803	12.2733	11.9066	8.86069	8.32608	7.96788	cytochrome b5 reductase 1 [Source:HGNC Symbol;Acc:HGNC:133	chr1:20293	NM_01624	.	.	.	NP_05732	.	protein_co	ENSG0000	51706	13397
CYB5R2	7.62607	8.30416	8.14796	10.3677	8.89608	9.17999	cytochrome b5 reductase 2 [Source:HGNC Symbol;Acc:HGNC:243	chr11:7686	NM_00130	XM_01152	.	.	NP_00128	XP_005253	protein_co	ENSG0000	51700	24376
CYB5R3	80.967	82.929	79.5957	77.5126	63.6978	64.9668	cytochrome b5 reductase 3 [Source:HGNC Symbol;Acc:HGNC:287	chr22:4301	NM_00039	.	.	.	NP_00038	.	protein_co	ENSG0000	1727	2873
CYB5R4	7.8795	6.81604	6.08324	7.68821	8.51881	7.69634	cytochrome b5 reductase 4 [Source:HGNC Symbol;Acc:HGNC:201	chr6:84569	NM_01623	.	.	.	NP_05731	.	protein_co	ENSG0000	51167	20147
CYB5RL	5.85011	8.04331	5.68616	6.4578	5.60916	4.18501	cytochrome b5 reductase like [Source:HGNC Symbol;Acc:HGNC:3	chr1:54635	NM_00103	.	.	.	NP_00102	.	protein_co	ENSG0000	606495	32220
CYBA	222.931	216.382	223.633	259.13	185.636	177.567	cytochrome b-245 alpha chain [Source:HGNC Symbol;Acc:HGNC:2	chr16:8870	NM_00010	XM_01152	.	.	NP_00009	XP_011521	protein_co	ENSG0000	1535	2577
CYBB	0.554899	0.317324	0.515177	0.338357	0.306587	0.810955	cytochrome b-245 beta chain [Source:HGNC Symbol;Acc:HGNC:2	chrX:37208	NM_00039	XM_01154	.	.	NP_00038	.	protein_co	ENSG0000	1536	2578
CYBRD1	8.41638	8.68142	11.0191	10.227	10.694	9.57213	cytochrome b reductase 1 [Source:HGNC Symbol;Acc:HGNC:2079	chr2:17237	NM_00112	.	.	.	NP_00112	.	protein_co	ENSG0000	79901	20797
CYC1	261.869	263.304	252.325	319.708	247.765	221.601	cytochrome c1 [Source:HGNC Symbol;Acc:HGNC:2579]	chr8:14514	NM_00191	.	.	.	NP_00190	.	protein_co	ENSG0000	1537	2579
CYCS	166.524	170.1	133.994	207.078	202.514	183.745	cytochrome c, somatic [Source:HGNC Symbol;Acc:HGNC:19986]	chr7:25159	NM_01894	.	.	.	NP_06182	.	protein_co	ENSG0000	54205	19986
CYFIP1	43.0159	43.1975	49.0928	43.4712	41.6106	41.5051	cytoplasmic FMR1 interacting protein 1 [Source:HGNC Symbol;Ac	chr15:2289	NM_00103	XM_01154	.	.	NP_00102	XP_011542	protein_co	ENSG0000	23191	13759
CYFIP2	17.0567	16.9379	23.3845	18.6264	18.2309	16.0291	cytoplasmic FMR1 interacting protein 2 [Source:HGNC Symbol;Ac	chr5:15669	NM_00103	XM_01700	.	.	NP_00103	XP_011532	protein_co	ENSG0000	26999	13760
CYGB	0.375593	0.233873	0.813352	0.159457	0.309831	0.218931	cytoglobin [Source:HGNC Symbol;Acc:HGNC:16505]	chr17:7452	NM_13426	.	.	.	NP_59903	.	protein_co	ENSG0000	114757	16505
CYHR1	30.3989	30.4882	30.102	24.9642	20.0133	24.137	cysteine and histidine rich 1 [Source:HGNC Symbol;Acc:HGNC:178	chr8:14567	NM_00112	XM_00671	.	.	NP_00112	XP_006716	protein_co	ENSG0000	50626	17806
CYLC1	0	0	0	0	0	0	cylicin 1 [Source:HGNC Symbol;Acc:HGNC:2582]	chrX:83116	NM_00127	XM_00526	.	.	NP_00125	XP_005262	protein_co	ENSG0000	1538	2582
CYLC2	0	0	0	0	0	0	cylicin 2 [Source:HGNC Symbol;Acc:HGNC:2583]	chr9:10575	NM_00134	.	.	.	NP_00133	.	protein_co	ENSG0000	1539	2583
CYLD	5.68041	5.73758	4.78238	3.35832	3.87037	4.25959	CYLD lysine 63 deubiquitinase [Source:HGNC Symbol;Acc:HGNC:2	chr16:5077	NM_00104	XM_01702	.	.	NP_00103	XP_005255	protein_co	ENSG0000	1540	2584
CYP11A1	0.035825	0.072005	0.186423	0.077945	0	0.077218	cytochrome P450 family 11 subfamily A member 1 [Source:HGNC	chr15:7463	NM_00078	.	.	.	NP_00077	.	protein_co	ENSG0000	1583	2590
CYP11B1	0	0.041929	0	0	0	0	cytochrome P450 family 11 subfamily B member 1 [Source:HGNC	chr8:14395	NM_00049	XM_01151	.	.	NP_00048	XP_011515	protein_co	ENSG0000	1584	2591
CYP11B2	0	0	0	0	0	0	cytochrome P450 family 11 subfamily B member 2 [Source:HGNC	chr8:14399	NM_00049	XM_01151	.	.	NP_00048	.	protein_co	ENSG0000	1585	2592
CYP17A1	0	0	0	0	0	0	cytochrome P450 family 17 subfamily A member 1 [Source:HGNC	chr10:1045	NM_00010	.	.	.	NP_00009	.	protein_co	ENSG0000	1586	2593
CYP19A1	0.105679	0.140694	0.03057	0.09401	0.09219	0.070619	cytochrome P450 family 19 subfamily A member 1 [Source:HGNC	chr15:5150	NM_00010	XM_00525	.	.	NP_00009	XP_005254	protein_co	ENSG0000	1588	2594

CYP1A1	2.7974	2.67244	1.99867	1.39666	1.34787	0.807028	cytochrome P450 family 1 subfamily A member 1 [Source:HGNC	chr15:7501	NM_00049	XM_01702	.	.	NP_00049	XP_005254	protein_co	ENSG0000	1543	2595
CYP1A2	0	0	0	0.030233	0	0	cytochrome P450 family 1 subfamily A member 2 [Source:HGNC	chr15:7504	NM_00076	.	.	.	NP_00075	.	protein_co	ENSG0000	1544	2596
CYP1B1	1.35702	1.68423	1.29025	1.23566	1.76561	1.35773	cytochrome P450 family 1 subfamily B member 1 [Source:HGNC	chr2:38294	NM_00010	.	.	.	NP_00009	.	protein_co	ENSG0000	1545	2597
CYP20A1	3.50785	3.24772	2.29559	3.08495	3.1079	5.39343	cytochrome P450 family 20 subfamily A member 1 [Source:HGNC	chr2:20410	NM_17753	XM_00671	.	.	NP_80388	XP_005246	protein_co	ENSG0000	57404	20576
CYP21A2	0.074304	0	0.075058	0.119737	0.215349	0.1226	cytochrome P450 family 21 subfamily A member 2 [Source:HGNC	chr6:32006	NM_00050	XM_01151	.	.	NP_00049	.	protein_co	ENSG0000	1589	2600
CYP24A1	4.74784	5.51921	22.0089	1.74407	1.36776	1.17465	cytochrome P450 family 24 subfamily A member 1 [Source:HGNC	chr20:5276	NM_00078	XM_01702	.	.	NP_00077	XP_005260	protein_co	ENSG0000	1591	2602
CYP26A1	0.204893	0.167151	0.447041	0.481879	0.464726	0.223139	cytochrome P450 family 26 subfamily A member 1 [Source:HGNC	chr10:9483	NM_00078	.	.	.	NP_00077	.	protein_co	ENSG0000	1592	2603
CYP26B1	6.14647	6.37907	7.13207	5.5533	5.27614	5.89134	cytochrome P450 family 26 subfamily B member 1 [Source:HGNC	chr2:72356	NM_00127	XM_01153	.	.	NP_00126	XP_005264	protein_co	ENSG0000	56603	20581
CYP26C1	0	0	0	0	0.114901	0	cytochrome P450 family 26 subfamily C member 1 [Source:HGNC	chr10:9482	NM_18337	.	.	.	NP_89923	.	protein_co	ENSG0000	340665	20577
CYP27A1	0.445168	0.219595	0.254899	0.159192	0.301342	0.203714	cytochrome P450 family 27 subfamily A member 1 [Source:HGNC	chr2:21964	NM_00078	.	.	.	NP_00077	.	protein_co	ENSG0000	1593	2605
CYP27B1	6.80056	7.60298	8.36622	5.15737	4.40221	3.8333	cytochrome P450 family 27 subfamily B member 1 [Source:HGNC	chr12:5815	NM_00078	.	.	.	NP_00077	.	protein_co	ENSG0000	1594	2606
CYP27C1	0	0	0	0	0	0	cytochrome P450 family 27 subfamily C member 1 [Source:HGNC	chr2:12794	NM_00100	.	.	.	NP_00100	.	protein_co	ENSG0000	339761	33480
CYP2A13	0	0	0	0	0	0	cytochrome P450 family 2 subfamily A member 13 [Source:HGNC	chr19:4159	NM_00076	.	.	.	NP_00075	.	protein_co	ENSG0000	1553	2608
CYP2A6	0	0	0	0	0	0	cytochrome P450 family 2 subfamily A member 6 [Source:HGNC	chr19:4134	NM_00076	.	.	.	NP_00075	.	protein_co	ENSG0000	1548	2610
CYP2A7	0	0	0	0	0	0	cytochrome P450 family 2 subfamily A member 7 [Source:HGNC	chr19:4138	NM_00076	.	.	.	NP_00075	.	protein_co	ENSG0000	1549	2611
CYP2B6	0	0	0	0.057407	0.05981	0.055381	cytochrome P450 family 2 subfamily B member 6 [Source:HGNC	chr19:4149	NM_00076	XM_01152	.	.	NP_00075	XP_005258	protein_co	ENSG0000	1555	2615
CYP2C18	0.061385	0	0.053483	0	0.034067	0.061168	cytochrome P450 family 2 subfamily C member 18 [Source:HGNC	chr10:9644	NM_00077	.	.	.	NP_00076	.	protein_co	ENSG0000	1562	2620
CYP2C19	0	0	0	0.042833	0	0.038739	cytochrome P450 family 2 subfamily C member 19 [Source:HGNC	chr10:9652	NM_00076	.	.	.	NP_00076	.	protein_co	ENSG0000	1557	2621
CYP2C8	0.041449	0	0.144438	0.174142	0.088672	0.152442	cytochrome P450 family 2 subfamily C member 8 [Source:HGNC	chr10:9679	NM_00077	.	.	.	NP_00076	.	protein_co	ENSG0000	1558	2622
CYP2C9	0.040032	0.042221	0.034777	0	0	0	cytochrome P450 family 2 subfamily C member 9 [Source:HGNC	chr10:9669	NM_00077	.	.	.	NP_00076	XP_016871	protein_co	ENSG0000	1559	2623
CYP2D6	0.36241	0.235838	0.331598	0.19322	0.336622	0.17932	cytochrome P450 family 2 subfamily D member 6 [Source:HGNC	chr22:4252	NM_00010	XM_01152	.	.	.	XP_011528	protein_co	ENSG0000	107987479	2625
CYP2E1	0.264172	0.269467	0.382911	0.04824	0.251739	0.13926	cytochrome P450 family 2 subfamily E member 1 [Source:HGNC	chr10:1353	NM_00077	.	.	.	NP_00076	.	protein_co	ENSG0000	1571	2631
CYP2F1	0	0	0	0	0	0	cytochrome P450 family 2 subfamily F member 1 [Source:HGNC	chr19:4162	NM_00077	XM_01152	.	.	NP_00076	XP_011524	protein_co	ENSG0000	1572	2632
CYP2J2	0.998544	0.657231	0.935877	1.11973	1.28633	1.4297	cytochrome P450 family 2 subfamily J member 2 [Source:HGNC S	chr1:60358	NM_00077	.	NR_13498	.	NP_00076	.	protein_co	ENSG0000	1573	2634
CYP2R1	6.66881	6.00894	7.11253	4.09482	3.89889	8.26825	cytochrome P450 family 2 subfamily R member 1 [Source:HGNC	chr11:1489	NM_02451	XM_01701	.	.	NP_07879	XP_005252	protein_co	ENSG0000	120227	20580
CYP2S1	9.30516	10.0927	7.69067	8.17601	7.13581	6.7897	cytochrome P450 family 2 subfamily S member 1 [Source:HGNC	chr19:4169	NM_03062	.	.	.	NP_08512	.	protein_co	ENSG0000	29785	15654
CYP2U1	3.37237	2.74785	3.67351	2.45267	2.74451	3.30496	cytochrome P450 family 2 subfamily U member 1 [Source:HGNC	chr4:10885	NM_18307	XM_00526	.	.	NP_89889	XP_005262	protein_co	ENSG0000	113612	20582
CYP2W1	0	0	0	0	0	0	cytochrome P450 family 2 subfamily W member 1 [Source:HGNC	chr7:10228	NM_01778	XM_01151	.	.	NP_06025	XP_011513	protein_co	ENSG0000	54905	20243
CYP39A1	0.780712	0.601036	0.905383	0.74689	0.819	1.41527	cytochrome P450 family 39 subfamily A member 1 [Source:HGNC	chr6:46517	NM_00127	XM_01151	.	.	NP_00126	XP_011512	protein_co	ENSG0000	51302	17449
CYP3A4	0	0	0	0	0	0	cytochrome P450 family 3 subfamily A member 4 [Source:HGNC	chr7:99354	NM_00120	XM_01151	.	.	NP_00118	.	protein_co	ENSG0000	1576	2637
CYP3A43	0	0	0	0	0	0	cytochrome P450 family 3 subfamily A member 43 [Source:HGNC	chr7:99425	NM_00127	XM_01151	.	.	NP_00126	.	protein_co	ENSG0000	64816	17450
CYP3A5	0.275635	0.021707	0.571345	0.098755	0.044639	0.250801	cytochrome P450 family 3 subfamily A member 5 [Source:HGNC	chr7:99245	NM_00077	XM_01151	NR_03380	.	NP_00076	XP_011514	protein_co	ENSG0000	1577	2638
CYP3A7	0	0	0	0	0	0	cytochrome P450 family 3 subfamily A member 7 [Source:HGNC	chr7:99282	NM_00076	.	.	.	NP_00075	.	protein_co	ENSG0000	1551	2640
CYP3A7-CYP3A51P	0	0	0	0	0	0	CYP3A7-CYP3A51P readthrough [Source:HGNC Symbol;Acc:HGNC	chr7:99282	NM_00125	.	.	.	NP_00124	.	protein_co	ENSG0000	100861540	51504
CYP46A1	0.114933	0.122811	0.199381	0.206203	0	0.12589	cytochrome P450 family 46 subfamily A member 1 [Source:HGNC	chr14:1001	NM_00666	XM_01153	.	.	NP_00665	XP_011534	protein_co	ENSG0000	10858	2641
CYP4A11	0	0	0	0	0	0	cytochrome P450 family 4 subfamily A member 11 [Source:HGNC	chr1:47394	NM_00077	XM_01700	.	.	NP_00076	XP_005270	protein_co	ENSG0000	1579	2642
CYP4A22	0	0	0	0	0	0	cytochrome P450 family 4 subfamily A member 22 [Source:HGNC	chr1:47603	NM_00101	XM_00527	.	.	NP_00101	XP_005270	protein_co	ENSG0000	284541	20575
CYP4B1	0	0	0	0	0	0	cytochrome P450 family 4 subfamily B member 1 [Source:HGNC	chr1:47264	NM_00077	XM_01154	.	.	NP_00077	XP_011539	protein_co	ENSG0000	1580	2644
CYP4F11	8.34836	8.5263	5.88927	7.15066	7.81104	8.67321	cytochrome P450 family 4 subfamily F member 11 [Source:HGNC	chr19:1602	NM_00112	XM_01152	.	.	NP_00112	.	protein_co	ENSG0000	57834	13265
CYP4F12	0.523125	0.24157	0.533044	0.722855	0.542006	0.566303	cytochrome P450 family 4 subfamily F member 12 [Source:HGNC	chr19:1578	NM_02394	XM_01152	NR_11708	.	NP_07643	XP_011526	protein_co	ENSG0000	66002	18857
CYP4F2	0.183847	0.249803	1.08591	0.335219	0.313791	0.949078	cytochrome P450 family 4 subfamily F member 2 [Source:HGNC	chr19:1598	NM_00108	.	.	.	NP_00107	.	protein_co	ENSG0000	8529	2645
CYP4F22	2.44349	2.18577	2.21631	2.6459	2.17788	2.5865	cytochrome P450 family 4 subfamily F member 22 [Source:HGNC	chr19:1561	NM_17348	XM_01152	.	.	NP_77575	XP_011525	protein_co	ENSG0000	126410	26820
CYP4F3	0.57694	0.848716	0.633186	0.692149	0.80952	0.963224	cytochrome P450 family 4 subfamily F member 3 [Source:HGNC	chr19:1572	NM_00089	XM_01702	.	.	NP_00088	XP_005259	protein_co	ENSG0000	4051	2646
CYP4F8	0	0.043703	0	0.094221	0	0	cytochrome P450 family 4 subfamily F member 8 [Source:HGNC	chr19:1572	NM_00725	XM_01152	.	.	NP_00918	.	protein_co	ENSG0000	11283	2648
CYP4V2	3.30813	3.50467	2.36286	2.93959	2.12901	3.78167	cytochrome P450 family 4 subfamily V member 2 [Source:HGNC	chr4:18711	NM_20735	XM_00526	.	.	NP_99723	XP_005262	protein_co	ENSG0000	285440	23198
CYP4X1	0	0	0	0	0	0	cytochrome P450 family 4 subfamily X member 1 [Source:HGNC	chr1:47489	NM_17803	XM_01154	.	.	NP_82884	XP_016856	protein_co	ENSG0000	260293	20244
CYP4Z1	0	0	0	0	0	0	cytochrome P450 family 4 subfamily Z member 1 [Source:HGNC	chr1:47533	NM_17813	.	.	.	NP_83523	.	protein_co	ENSG0000	199974	20583
CYP51A1	42.8469	43.5235	48.3912	43.8683	54.5325	59.3172	cytochrome P450 family 51 subfamily A member 1 [Source:HGNC	chr7:91741	NM_00078	.	.	.	NP_00077	.	protein_co	ENSG0000	1595	2649
CYP7A1	0	0	0	0	0	0	cytochrome P450 family 7 subfamily A member 1 [Source:HGNC	chr8:59402	NM_00078	XM_01151	.	.	NP_00077	XP_011515	protein_co	ENSG0000	1581	2651
CYP7B1	0	0	0	0	0	0	cytochrome P450 family 7 subfamily B member 1 [Source:HGNC	chr8:65508	NM_00482	.	.	.	NP_00481	.	protein_co	ENSG0000	9420	2652
CYP8B1	0	0	0	0	0	0	cytochrome P450 family 8 subfamily B member 1 [Source:HGNC	chr3:42850	NM_00439	.	.	.	NP_00438	.	protein_co	ENSG0000	1582	2653
CYR61	9.02099	11.3248	6.85576	8.27341	8.25176	7.97885	cysteine rich angiogenic inducer 61 [Source:HGNC Symbol;Acc:HG	chr1:86046	NM_00155	.	.	.	NP_00154	.	protein_co	ENSG0000	3491	2654
CYS1	2.12195	2.7506	3.41173	2.19987	2.1096	3.52945	cystin 1 [Source:HGNC Symbol;Acc:HGNC:18525]	chr2:10196	NM_00103	.	.	.	NP_00103	.	protein_co	ENSG0000	192668	18525
CYSLTR1	0	0	0	0	0	0	cysteinyl leukotriene receptor 1 [Source:HGNC Symbol;Acc:HGNC	chrX:77526	NM_00128	.	.	.	NP_00126	.	protein_co	ENSG0000	10800	17451
CYSLTR2	0	0	0	0	0	0	cysteinyl leukotriene receptor 2 [Source:HGNC Symbol;Acc:HGNC	chr13:4922	NM_02037	.	.	.	NP_00129	.	protein_co	ENSG0000	57105	18274
CYSRT1	2.16417	3.17647	4.40398	2.13794	2.01772	1.21957	cysteine rich tail 1 [Source:HGNC Symbol;Acc:HGNC:30529]	chr9:14011	NM_19900	.	.	.	NP_94535	.	protein_co	ENSG0000	375791	30529
CYSTM1	44.6182	40.4672	39.9749	51.9559	45.0185	47.8977	cysteine rich transmembrane module containing 1 [Source:HGNC	chr5:13955	NM_03241	.	.	.	NP_11578	.	protein_co	ENSG0000	84418	30239
CYTH1	17.281	17.2708	16.5609	16.6472	18.2187	18.0939	cytohesin 1 [Source:HGNC Symbol;Acc:HGNC:9501]	chr17:7667	NM_00129	XM_01702	.	.	NP_00127	XP_011523	protein_co	ENSG0000	9267	9501
CYTH2	15.547	13.7853	14.7722	13.2189	12.9153	16.1832	cytohesin 2 [Source:HGNC Symbol;Acc:HGNC:9502]	chr19:4894	NM_00422	XM_00672	.	.	NP_00421	XP_006723	protein_co	ENSG0000	9266	9502

CYTH3	8.48187	9.82693	10.6167	9.165	9.3579	10.3048	cytohesin 3 [Source:HGNC Symbol;Acc:HGNC:9504]	chr7:62014	NM_00422	.	.	.	NP_00421	.	protein_co	ENSG0000	9265	9504
CYTH4	1.65124	1.1846	1.43865	1.53007	1.61384	0.986134	cytohesin 4 [Source:HGNC Symbol;Acc:HGNC:9505]	chr22:3767	NM_00131	XM_01153	.	.	NP_00130	XP_011528	protein_co	ENSG0000	27128	9505
CYTIP	0	0	0	0	0	0.072339	cytohesin 1 interacting protein [Source:HGNC Symbol;Acc:HGNC:	chr2:15827	NM_00428	.	.	.	NP_00427	.	protein_co	ENSG0000	9595	9506
CYTL1	0	0	0	0	0	0	cytokine like 1 [Source:HGNC Symbol;Acc:HGNC:24435]	chr4:50163	NM_01865	.	.	.	NP_06112	.	protein_co	ENSG0000	54360	24435
CYYR1	0	0	0	0	0	0	cysteine and tyrosine rich 1 [Source:HGNC Symbol;Acc:HGNC:162	chr21:2783	NM_00132	XM_01152	.	.	NP_00130	.	protein_co	ENSG0000	116159	16274
D2HGDH	6.25437	7.48315	12.1993	7.61724	5.77422	7.8724	D-2-hydroxyglutarate dehydrogenase [Source:HGNC Symbol;Acc:	chr2:24267	NM_00128	XM_01700	.	.	NP_00127	XP_01151	protein_co	ENSG0000	728294	28358
DAAM1	2.9791	2.65926	2.93222	2.08278	2.41068	4.18867	dishevelled associated activator of morphogenesis 1 [Source:HGN	chr14:5965	NM_00127	XM_00526	.	.	NP_00125	XP_005267	protein_co	ENSG0000	23002	18142
DAAM2	0	0	0	0	0	0	dishevelled associated activator of morphogenesis 2 [Source:HGN	chr6:39760	NM_00120	XM_01151	.	.	NP_00118	XP_006715	protein_co	ENSG0000	23500	18143
DAB1	0	0	0	0	0.016648	0.040501	DAB1, reelin adaptor protein [Source:HGNC Symbol;Acc:HGNC:26	chr1:57460	NM_02108	.	.	.	NP_06656	.	protein_co	ENSG0000	1600	2661
DAB2	3.20318	2.94034	3.20553	2.62905	2.98017	3.50487	DAB2, clathrin adaptor protein [Source:HGNC Symbol;Acc:HGNC:	chr5:39371	NM_00124	.	.	.	NP_00123	.	protein_co	ENSG0000	1601	2662
DAB2IP	9.33459	8.716	8.9406	13.2086	13.084	11.5479	DAB2 interacting protein [Source:HGNC Symbol;Acc:HGNC:17294	chr9:12432	NM_03255	XM_01701	.	.	NP_11594	XP_005251	protein_co	ENSG0000	153090	17294
DACH1	0	0	0.022116	0	0	0	dachshund family transcription factor 1 [Source:HGNC Symbol;Ac	chr13:7201	NM_00439	XM_01153	.	.	NP_00438	XP_011533	protein_co	ENSG0000	1602	2663
DACH2	0	0	0	0	0	0	dachshund family transcription factor 2 [Source:HGNC Symbol;Ac	chrX:85403	NM_00113	XM_01153	.	.	NP_00113	XP_011529	protein_co	ENSG0000	117154	16814
DACT1	0.405249	0.484559	0.793296	2.79485	3.09765	2.05838	dishevelled binding antagonist of beta catenin 1 [Source:HGNC Sy	chr14:5910	NM_00107	XM_00672	.	.	NP_00107	XP_00672	protein_co	ENSG0000	51339	17748
DACT2	0	0	0	0	0	0	dishevelled binding antagonist of beta catenin 2 [Source:HGNC Sy	chr6:16869	NM_00128	.	.	.	NP_00127	.	protein_co	ENSG0000	168002	21231
DACT3	1.83226	2.10566	3.19769	2.1689	2.55024	2.47404	dishevelled binding antagonist of beta catenin 3 [Source:HGNC Sy	chr19:4715	NM_00130	XM_01152	.	.	NP_00128	XP_011524	protein_co	ENSG0000	147906	30745
DAD1	181.452	159.66	169.951	183.309	173.079	203.333	defender against cell death 1 [Source:HGNC Symbol;Acc:HGNC:26	chr14:2303	NM_00134	.	.	.	NP_00133	.	protein_co	ENSG0000	1603	2664
DAG1	48.948	50.3507	44.2903	43.1289	45.8255	40.0446	dystroglycan 1 [Source:HGNC Symbol;Acc:HGNC:2666]	chr3:49506	NM_00116	.	.	.	NP_00115	.	protein_co	ENSG0000	1605	2666
DAGLA	1.98053	1.66336	1.96183	1.3768	1.68774	1.25125	diacylglycerol lipase alpha [Source:HGNC Symbol;Acc:HGNC:1165	chr11:6144	NM_00613	XM_01154	.	.	NP_00612	XP_005274	protein_co	ENSG0000	747	1165
DAGLB	12.442	12.1918	9.81656	9.76012	7.30767	7.31829	diacylglycerol lipase beta [Source:HGNC Symbol;Acc:HGNC:28923	chr7:64487	NM_00114	.	.	.	NP_00113	.	protein_co	ENSG0000	221955	28923
DALRD3	21.1933	17.3988	21.2527	21.405	18.6147	16.7848	DALR anticodon binding domain containing 3 [Source:HGNC Symb	chr3:49027	NM_00100	XM_01153	.	.	NP_00100	XP_005265	protein_co	ENSG0000	55152	25536
DAND5	0.049438	0.173455	0	0.093308	0.170356	0.106512	DAN domain BMP antagonist family member 5 [Source:HGNC Syr	chr19:1307	NM_15265	.	.	.	NP_68986	.	protein_co	ENSG0000	199699	26780
DAO	0	0.053505	0	0	0	0	D-amino acid oxidase [Source:HGNC Symbol;Acc:HGNC:2671]	chr12:1092	NM_00191	XM_01153	.	.	NP_00190	XP_005268	protein_co	ENSG0000	1610	2671
DAOA	0	0	0	0	0	0	D-amino acid oxidase activator [Source:HGNC Symbol;Acc:HGNC:	chr13:1061	NM_00116	XM_00525	.	.	NP_00115	XP_005254	protein_co	ENSG0000	267012	21191
DAP	49.6377	50.7322	51.7765	52.2546	46.6146	45.123	death associated protein [Source:HGNC Symbol;Acc:HGNC:2672]	chr5:10679	NM_00129	.	.	.	NP_00127	.	protein_co	ENSG0000	1611	2672
DAP3	87.4329	89.5957	81.4587	104.944	89.3818	89.0183	death associated protein 3 [Source:HGNC Symbol;Acc:HGNC:267	chr1:15562	NM_00119	XM_01700	.	.	NP_00118	XP_005245	protein_co	ENSG0000	7818	2673
DAPK1	1.93655	1.76842	2.33153	1.74377	2.03245	2.01862	death associated protein kinase 1 [Source:HGNC Symbol;Acc:HGN	chr9:90112	NM_00128	.	.	.	NP_00127	.	protein_co	ENSG0000	1612	2674
DAPK2	1.29426	1.23754	1.69467	1.29759	1.2876	1.70488	death associated protein kinase 2 [Source:HGNC Symbol;Acc:HGN	chr15:6419	NM_01432	XM_01152	.	.	NP_05514	XP_011519	protein_co	ENSG0000	23604	2675
DAPK3	24.5575	23.2624	22.8805	27.8422	24.244	20.1873	death associated protein kinase 3 [Source:HGNC Symbol;Acc:HGN	chr19:3958	NM_00134	XM_00525	NR_03036	.	NP_00133	XP_005259	protein_co	ENSG0000	693222	2676
DAPL1	0	0	0.144515	0	0.190491	0.1723	death associated protein like 1 [Source:HGNC Symbol;Acc:HGNC:	chr2:15965	NM_00101	.	.	.	NP_00101	.	protein_co	ENSG0000	92196	21490
DAPP1	1.01714	0.832055	0.27936	0.122475	0.16932	0.186713	dual adaptor of phosphotyrosine and 3-phosphoinositides 1 [Sou	chr4:10073	NM_00130	XM_01153	.	.	NP_00129	XP_01153	protein_co	ENSG0000	27071	16500
DARS	51.3114	48.2146	47.8582	46.5415	54.7758	56.1328	aspartyl-tRNA synthetase [Source:HGNC Symbol;Acc:HGNC:2678]	chr2:13666	NM_00129	.	.	.	NP_00128	XP_016858	protein_co	ENSG0000	1615	2678
DARS2	17.9991	19.7007	12.0537	20.0926	19.9323	14.1042	aspartyl-tRNA synthetase 2, mitochondrial [Source:HGNC Symbol	chr1:17379	NM_01812	XM_00671	.	.	NP_06059	XP_006711	protein_co	ENSG0000	55157	25538
DAW1	0.178001	0.046762	0.038525	0	0.050007	0	dynein assembly factor with WD repeats 1 [Source:HGNC Symbol	chr2:22873	NM_17882	XM_01151	.	.	NP_84914	XP_011509	protein_co	ENSG0000	164781	26383
DAXX	31.3109	34.7985	26.8927	37.3362	33.1375	25.3208	death domain associated protein [Source:HGNC Symbol;Acc:HGN	chr6:33286	NM_00114	.	.	.	NP_00113	.	protein_co	ENSG0000	1616	2681
DAZ1	0	0	0	0	0	0	deleted in azoospermia 1 [Source:HGNC Symbol;Acc:HGNC:2682]	chrY:25275	NM_00408	XM_01153	.	.	NP_00407	XP_011529	protein_co	ENSG0000	1617	2682
DAZ2	0	0	0	0	0	0	deleted in azoospermia 2 [Source:HGNC Symbol;Acc:HGNC:15964	chrY:25365	NM_00100	XM_01153	.	.	NP_00100	XP_011529	protein_co	ENSG0000	57135	15964
DAZ3	0	0	0	0	0	0	deleted in azoospermia 3 [Source:HGNC Symbol;Acc:HGNC:15965	chrY:26909	NM_02036	XM_01153	.	.	NP_06509	.	protein_co	ENSG0000	57054	15965
DAZ4	0	0	0	0	0	0	deleted in azoospermia 4 [Source:HGNC Symbol;Acc:HGNC:15966	chrY:26979	NM_00100	XM_01153	.	.	NP_00100	XP_005262	protein_co	ENSG0000	57135	15966
DAZAP1	78.2762	83.9995	63.2287	103.295	79.1391	86.407	DAZ associated protein 1 [Source:HGNC Symbol;Acc:HGNC:2683]	chr19:1407	NM_01895	XM_01702	.	.	NP_06183	XP_005259	protein_co	ENSG0000	26528	2683
DAZAP2	166.839	171.87	163.189	169.084	158.296	162.586	DAZ associated protein 2 [Source:HGNC Symbol;Acc:HGNC:2684]	chr12:5163	NM_00113	.	.	.	NP_00112	.	protein_co	ENSG0000	9802	2684
DAZL	0	0	0	0	0	0	deleted in azoospermia like [Source:HGNC Symbol;Acc:HGNC:268	chr3:16628	NM_00119	.	.	.	NP_00117	.	protein_co	ENSG0000	1618	2685
DBF4	10.0184	10.8589	11.0121	13.7286	18.8627	17.6014	DBF4 zinc finger [Source:HGNC Symbol;Acc:HGNC:17364]	chr7:87462	NM_00671	XM_01151	.	.	NP_00670	.	protein_co	ENSG0000	10926	17364
DBF4B	3.36289	3.39268	3.74722	4.68007	4.10392	3.11498	DBF4 zinc finger B [Source:HGNC Symbol;Acc:HGNC:17883]	chr17:4278	NM_02510	XM_01152	.	.	NP_07938	XP_006722	protein_co	ENSG0000	80174	17883
DBH	0	0	0	0	0	0	dopamine beta-hydroxylase [Source:HGNC Symbol;Acc:HGNC:26	chr9:13650	NM_00078	.	.	.	NP_00077	.	protein_co	ENSG0000	1621	2689
DBI	194.758	173.388	239.927	276.708	225.828	280.151	diazepam binding inhibitor, acyl-CoA binding protein [Source:HG	chr2:12012	NM_00107	.	.	.	NP_00107	.	protein_co	ENSG0000	1622	2690
DBN1	85.6548	88.2584	86.6758	109.071	100.377	91.1932	drebrin 1 [Source:HGNC Symbol;Acc:HGNC:2695]	chr5:17688	NM_00439	XM_01153	.	.	NP_00438	XP_005265	protein_co	ENSG0000	1627	2695
DBNDD1	35.2652	34.1119	42.1972	39.8586	33.9563	32.7973	dysbindin domain containing 1 [Source:HGNC Symbol;Acc:HGNC:	chr16:9007	NM_00104	.	.	.	NP_00103	.	protein_co	ENSG0000	79007	28455
DBNDD2	36.3396	31.5944	31.7288	36.809	31.0816	24.1315	dysbindin domain containing 2 [Source:HGNC Symbol;Acc:HGNC:	chr20:4403	NM_00104	.	.	.	NP_00104	.	protein_co	ENSG0000	55861	15881
DBNL	33.4009	33.0764	28.9213	30.7927	28.9006	21.9522	drebrin like [Source:HGNC Symbol;Acc:HGNC:2696]	chr7:44084	NM_00101	XM_01151	NR_10689	.	NP_00101	XP_011513	protein_co	ENSG0000	102466985	2696
DBP	10.3798	8.8491	12.2586	9.97062	6.99856	11.1479	D-box binding PAR bZIP transcription factor [Source:HGNC Symbo	chr19:4911	NM_00135	.	.	.	NP_00134	.	protein_co	ENSG0000	1628	2697
DBR1	13.4853	13.5786	12.4613	15.1285	14.6447	12.2641	debranching RNA lariats 1 [Source:HGNC Symbol;Acc:HGNC:1559	chr3:13787	NM_01621	.	.	.	NP_05730	.	protein_co	ENSG0000	51163	15594
DBT	4.59701	5.54362	5.78704	4.51609	6.90046	6.94951	dihydrolipoamide branched chain transacylase E2 [Source:HGNC	chr1:10065	NM_00191	XM_00527	.	.	NP_00190	XP_00527	protein_co	ENSG0000	1629	2698
DBX1	0	0	0	0	0	0	developing brain homeobox 1 [Source:HGNC Symbol;Acc:HGNC:3	chr11:2017	NM_00102	.	.	.	NP_00102	.	protein_co	ENSG0000	120237	33185
DBX2	0	0	0	0	0	0	developing brain homeobox 2 [Source:HGNC Symbol;Acc:HGNC:3	chr12:4540	NM_00100	.	.	.	NP_00100	.	protein_co	ENSG0000	440097	33186
DCAF1	16.1863	16.5229	15.8684	14.5253	15.0917	14.1512	DDB1 and CUL4 associated factor 1 [Source:HGNC Symbol;Acc:HG	chr3:51428	NM_00117	XM_01700	.	.	NP_00116	XP_005276	protein_co	ENSG0000	9730	30911
DCAF10	6.18465	6.08635	7.48623	4.60513	5.69947	7.77248	DDB1 and CUL4 associated factor 10 [Source:HGNC Symbol;Acc:H	chr9:37800	NM_00128	XM_01701	.	.	NP_00127	XP_005251	protein_co	ENSG0000	79269	23686
DCAF11	21.4683	21.1134	21.9166	20.3075	17.3476	21.9517	DDB1 and CUL4 associated factor 11 [Source:HGNC Symbol;Acc:H	chr14:2454	NM_00116	.	.	.	NP_00115	.	protein_co	ENSG0000	80344	20258
DCAF12	25.3963	24.7587	25.2662	28.9704	27.8839	29.3766	DDB1 and CUL4 associated factor 12 [Source:HGNC Symbol;Acc:H	chr9:34086	NM_01539	.	.	.	NP_05621	.	protein_co	ENSG0000	25853	19911

DCAF12L1	0	0	0	0	0	0	DDB1 and CUL4 associated factor 12 like 1 [Source:HGNC Symbol	chrX:12568	NM_17847	.	.	.	NP_848565	.	protein_co	ENSG0000	139170	29395
DCAF12L2	0	0	0	0	0	0	DDB1 and CUL4 associated factor 12 like 2 [Source:HGNC Symbol	chrX:12529	NM_00101	.	.	.	NP_001013	.	protein_co	ENSG0000	340578	32950
DCAF13	89.9412	84.3135	81.9974	96.4006	89.0824	73.9111	DDB1 and CUL4 associated factor 13 [Source:HGNC Symbol;Acc:H	chr8:10441	NM_01542	.	.	.	NP_056235	.	protein_co	ENSG0000	25879	24535
DCAF15	20.2001	19.4324	17.564	27.3046	23.0835	20.3561	DDB1 and CUL4 associated factor 15 [Source:HGNC Symbol;Acc:H	chr19:1404	NM_13835	.	.	.	NP_612362	.	protein_co	ENSG0000	90379	25095
DCAF16	22.8939	23.4176	22.503	23.1161	27.7747	24.4387	DDB1 and CUL4 associated factor 16 [Source:HGNC Symbol;Acc:H	chr4:17802	NM_01774	XM_01151	.	.	NP_060211	XP_005248	protein_co	ENSG0000	54876	25987
DCAF17	6.79377	7.13386	6.02014	5.51824	5.91548	6.68772	DDB1 and CUL4 associated factor 17 [Source:HGNC Symbol;Acc:H	chr2:17217	NM_00116	XM_01151	.	.	NP_001158	XP_006712	protein_co	ENSG0000	80067	25784
DCAF4	8.0654	7.87233	8.81522	7.42091	7.13353	8.07512	DDB1 and CUL4 associated factor 4 [Source:HGNC Symbol;Acc:HG	chr14:7339	NM_00116	XM_01702	.	.	NP_001156	XP_016876	protein_co	ENSG0000	26094	20229
DCAF4L1	0.575622	0.40308	0.372064	0.318918	0.31004	0.128672	DDB1 and CUL4 associated factor 4 like 1 [Source:HGNC Symbol;A	chr4:41983	NM_00102	.	.	.	NP_001025	.	protein_co	ENSG0000	285429	27723
DCAF4L2	0	0	0	0	0	0	DDB1 and CUL4 associated factor 4 like 2 [Source:HGNC Symbol;A	chr8:88882	NM_15241	.	.	.	NP_689631	.	protein_co	ENSG0000	138009	26657
DCAF5	10.6202	10.1792	10.8256	10.7964	9.7402	9.6556	DDB1 and CUL4 associated factor 5 [Source:HGNC Symbol;Acc:HG	chr14:6951	NM_00128	XM_01702	.	.	NP_001271	XP_006720	protein_co	ENSG0000	8816	20224
DCAF6	13.0112	12.7475	18.4675	13.7581	12.8796	15.516	DDB1 and CUL4 associated factor 6 [Source:HGNC Symbol;Acc:HG	chr1:16788	NM_00101	XM_01700	.	.	NP_001017	XP_005245	protein_co	ENSG0000	55827	30002
DCAF7	40.6241	42.0063	48.6615	49.5627	48.2823	52.0523	DDB1 and CUL4 associated factor 7 [Source:HGNC Symbol;Acc:HG	chr17:6162	NM_00582	.	.	.	NP_005819	.	protein_co	ENSG0000	10238	30915
DCAF8	22.097	22.0594	22.4255	25.0555	23.098	21.3632	DDB1 and CUL4 associated factor 8 [Source:HGNC Symbol;Acc:HG	chr1:16018	NM_01572	.	.	.	NP_056541	.	protein_co	ENSG0000	50717	24891
DCAF8L1	0	0	0	0	0	0	DDB1 and CUL4 associated factor 8 like 1 [Source:HGNC Symbol;A	chrX:27996	NM_00101	.	.	.	NP_001017	.	protein_co	ENSG0000	139425	31810
DCAF8L2	0	0	0.055426	0	0	0	DDB1 and CUL4 associated factor 8 like 2 [Source:HGNC Symbol;A	chrX:27608	NM_00113	.	.	.	NP_001130	.	protein_co	ENSG0000	347442	31811
DCAKD	17.2006	16.1806	15.9991	14.4187	12.1389	13.7032	dephospho-CoA kinase domain containing [Source:HGNC Symbol	chr17:4310	NM_00112	XM_01152	.	.	NP_001122	XP_006722	protein_co	ENSG0000	79877	26238
DCANP1	0	0	0	0	0	0	dendritic cell associated nuclear protein [Source:HGNC Symbol;A	chr5:13477	NM_13084	.	.	.	NP_570900	.	protein_co	ENSG0000	140947	24459
DCBLD1	8.46554	8.77622	9.9684	9.049	9.84373	15.3925	discoidin, CUB and LCCL domain containing 1 [Source:HGNC Symb	chr6:11760	NM_17367	XM_01701	.	.	NP_775945	XP_005266	protein_co	ENSG0000	285761	21479
DCBLD2	216.33	220.724	190.098	303.879	346.489	414.597	discoidin, CUB and LCCL domain containing 2 [Source:HGNC Symb	chr3:98514	NM_08092	XM_01151	.	.	NP_563615	XP_011510	protein_co	ENSG0000	131566	24627
DCC	0	0	0	0	0	0	DCC netrin 1 receptor [Source:HGNC Symbol;Acc:HGNC:2701]	chr18:4986	NM_00521	XM_01702	.	.	NP_005206	XP_011524	protein_co	ENSG0000	1630	2701
DCD	0	0.108154	0	0	0	0	dermcidin [Source:HGNC Symbol;Acc:HGNC:14669]	chr12:5503	NM_00130	.	.	.	NP_001287	.	protein_co	ENSG0000	117159	14669
DCDC1	0.252794	0.161362	0.142923	0.219978	0.048148	0.224217	doublecortin domain containing 1 [Source:HGNC Symbol;Acc:HGN	chr11:3088	NM_02086	.	.	.	NP_065920	.	protein_co	ENSG0000	100506627	20625
DCDC2	0.016657	0	0.09839	0.181145	0.018457	0.048702	doublecortin domain containing 2 [Source:HGNC Symbol;Acc:HGN	chr6:24171	NM_00119	.	.	.	NP_001182	.	protein_co	ENSG0000	51473	18141
DCDC2B	0	0	0	0	0	0	doublecortin domain containing 2B [Source:HGNC Symbol;Acc:HG	chr1:32674	NM_00109	.	.	.	NP_001092	.	protein_co	ENSG0000	149069	32576
DCDC2C	0	0	0	0	0	0	doublecortin domain containing 2C [Source:HGNC Symbol;Acc:HG	chr2:37511	NM_00128	XM_01151	.	.	NP_001274	XP_006711	protein_co	ENSG0000	728597	32696
DCHS1	0.169854	0.11884	0.127288	0.14387	0.123432	0.132869	dachsous cadherin-related 1 [Source:HGNC Symbol;Acc:HGNC:13	chr11:6642	NM_00373	.	.	.	NP_003728	.	protein_co	ENSG0000	8642	13681
DCHS2	0	0	0	0	0	0	dachsous cadherin-related 2 [Source:HGNC Symbol;Acc:HGNC:23	chr4:15515	NM_00114	.	.	.	NP_001136	XP_016863	protein_co	ENSG0000	54798	23111
DCK	22.1533	23.2163	19.1176	24.7629	29.8761	24.7668	deoxycytidine kinase [Source:HGNC Symbol;Acc:HGNC:2704]	chr4:71859	NM_00078	.	.	.	NP_000779	.	protein_co	ENSG0000	1633	2704
DCLK1	0.184031	0.176854	0.278835	0.037792	0.037355	0.116447	doublecortin like kinase 1 [Source:HGNC Symbol;Acc:HGNC:2700]	chr13:3634	NM_00119	XM_01702	.	.	NP_001182	XP_005266	protein_co	ENSG0000	9201	2700
DCLK2	0.771062	1.35021	1.45729	0.780275	0.92439	0.960217	doublecortin like kinase 2 [Source:HGNC Symbol;Acc:HGNC:1900	chr4:15099	NM_00104	XM_00526	.	.	NP_001035	XP_005262	protein_co	ENSG0000	166614	19002
DCLK3	0	0	0	0	0	0	doublecortin like kinase 3 [Source:HGNC Symbol;Acc:HGNC:1900	chr3:36753	NM_03340	XM_01153	.	.	NP_208382	XP_011532	protein_co	ENSG0000	85443	19005
DCLRE1A	13.5462	13.9785	10.7761	13.1363	15.2287	14.0504	DNA cross-link repair 1A [Source:HGNC Symbol;Acc:HGNC:17660]	chr10:1155	NM_00127	XM_01154	.	.	NP_001258	XP_006718	protein_co	ENSG0000	9937	17660
DCLRE1B	8.28768	8.97745	8.07236	8.44897	7.66823	6.72384	DNA cross-link repair 1B [Source:HGNC Symbol;Acc:HGNC:17641]	chr1:11444	NM_02283	.	.	.	NP_073747	.	protein_co	ENSG0000	64858	17641
DCLRE1C	4.87317	5.79388	4.41315	4.04679	5.17009	4.39342	DNA cross-link repair 1C [Source:HGNC Symbol;Acc:HGNC:17642]	chr10:1492	NM_00103	XM_01151	.	.	NP_001029	XP_006717	protein_co	ENSG0000	64421	17642
DCN	0	0	0	0	0	0	decorin [Source:HGNC Symbol;Acc:HGNC:2705]	chr12:9153	NM_00192	XM_00671	.	.	NP_001911	XP_005268	protein_co	ENSG0000	1634	2705
DCP1A	11.6457	12.3302	12.0494	9.84938	11.6452	13.4304	decapping mRNA 1A [Source:HGNC Symbol;Acc:HGNC:18714]	chr3:53317	NM_00129	.	.	.	NP_001277	.	protein_co	ENSG0000	55802	18714
DCP1B	9.88281	8.22926	9.31186	8.33682	7.88206	8.55723	decapping mRNA 1B [Source:HGNC Symbol;Acc:HGNC:24451]	chr12:2055	NM_00131	XM_01152	.	.	NP_001306	XP_011519	protein_co	ENSG0000	196513	24451
DCP2	10.7934	12.4452	12.6366	10.1281	13.7139	19.37	decapping mRNA 2 [Source:HGNC Symbol;Acc:HGNC:24452]	chr5:11231	NM_00124	XM_01700	.	.	NP_001229	XP_016864	protein_co	ENSG0000	167227	24452
DCPS	14.5339	13.3344	13.0644	14.2347	11.048	11.3419	decapping enzyme, scavenger [Source:HGNC Symbol;Acc:HGNC:2	chr11:1261	NM_01402	XM_01154	.	.	NP_054745	XP_011541	protein_co	ENSG0000	28960	29812
DCST1	0.145803	0	0.093756	0.039305	0.084019	0.118849	DC-STAMP domain containing 1 [Source:HGNC Symbol;Acc:HGNC	chr1:15500	NM_00114	.	.	.	NP_001137	.	protein_co	ENSG0000	149095	26539
DCST2	0.554898	0.406327	0.538936	0.47725	0.213877	0.563582	DC-STAMP domain containing 2 [Source:HGNC Symbol;Acc:HGNC	chr1:15499	NM_14462	.	.	.	NP_653223	.	protein_co	ENSG0000	127579	26562
DCSTAMP	0	0	0	0	0	0	dendrocyte expressed seven transmembrane protein [Source:HG	chr8:10535	NM_00125	XM_01151	.	.	NP_001244	XP_005251	protein_co	ENSG0000	81501	18549
DCT	0.048669	0.127593	0.056303	0.147791	0.068387	0.09057	dopachrome tautomerase [Source:HGNC Symbol;Acc:HGNC:2709	chr13:9508	NM_00112	.	.	.	NP_001123	XP_016875	protein_co	ENSG0000	1638	2709
DCTD	44.4895	44.0168	47.1423	45.1575	42.76	38.6388	dCMP deaminase [Source:HGNC Symbol;Acc:HGNC:2710]	chr4:18381	NM_00101	XM_01700	.	.	NP_001012	XP_005262	protein_co	ENSG0000	1635	2710
DCTN1	36.1588	39.3352	33.3724	42.3907	40.2523	32.5266	dynactin subunit 1 [Source:HGNC Symbol;Acc:HGNC:2711]	chr2:74588	NM_00113	.	.	.	NP_001128	.	protein_co	ENSG0000	1639	2711
DCTN2	47.6719	47.2237	47.5966	57.3689	44.3728	50.988	dynactin subunit 2 [Source:HGNC Symbol;Acc:HGNC:2712]	chr12:5791	NM_00126	XM_01153	.	.	NP_001248	XP_011536	protein_co	ENSG0000	10540	2712
DCTN3	35.7208	33.5346	32.116	49.2938	43.231	36.741	dynactin subunit 3 [Source:HGNC Symbol;Acc:HGNC:2713]	chr9:34613	NM_00128	.	.	.	NP_001268	.	protein_co	ENSG0000	11258	2713
DCTN4	27.893	31.2356	27.4948	28.4425	32.831	32.4775	dynactin subunit 4 [Source:HGNC Symbol;Acc:HGNC:15518]	chr5:15008	NM_00113	XM_01153	.	.	NP_001129	XP_005268	protein_co	ENSG0000	51164	15518
DCTN5	14.2319	13.4133	12.9675	15.998	14.5694	12.909	dynactin subunit 5 [Source:HGNC Symbol;Acc:HGNC:24594]	chr16:2365	NM_00119	.	.	.	NP_001185	.	protein_co	ENSG0000	84516	24594
DCTN6	31.3819	29.0731	30.9458	33.4272	28.9913	30.4519	dynactin subunit 6 [Source:HGNC Symbol;Acc:HGNC:16964]	chr8:30013	NM_00657	.	.	.	NP_006562	.	protein_co	ENSG0000	10671	16964
DCTPP1	156.261	150.751	137.519	193.822	146.834	103.673	dCTP pyrophosphatase 1 [Source:HGNC Symbol;Acc:HGNC:28777	chr16:3041	NM_02409	.	.	.	NP_077001	.	protein_co	ENSG0000	79077	28777
DCUN1D1	10.6548	10.5237	12.4922	9.5403	12.7197	16.4093	defective in cullin neddylation 1 domain containing 1 [Source:HG	chr3:18265	NM_00130	XM_01151	.	.	NP_001295	XP_005247	protein_co	ENSG0000	54165	18184
DCUN1D2	10.5192	9.83442	9.24064	8.70844	8.84878	9.16461	defective in cullin neddylation 1 domain containing 2 [Source:HG	chr13:1141	NM_00101	.	.	.	NP_001014	XP_016876	protein_co	ENSG0000	55208	20328
DCUN1D3	8.90309	9.9909	11.102	7.10497	7.90326	7.14908	defective in cullin neddylation 1 domain containing 3 [Source:HG	chr16:2077	NM_17347	.	.	.	NP_775746	.	protein_co	ENSG0000	123879	28734
DCUN1D4	11.1105	9.93541	11.3851	8.20265	10.7039	11.2386	defective in cullin neddylation 1 domain containing 4 [Source:HG	chr4:52709	NM_00104	XM_01153	.	.	NP_001035	XP_005265	protein_co	ENSG0000	23142	28998
DCUN1D5	42.0931	40.3465	44.4759	47.1245	52.9644	53.942	defective in cullin neddylation 1 domain containing 5 [Source:HG	chr11:1029	NM_03229	XM_00527	.	.	NP_115675	.	protein_co	ENSG0000	84259	28409
DCX	0.009103	0	0	0	0	0	doublecortin [Source:HGNC Symbol;Acc:HGNC:2714]	chrX:11053	NM_00055	XM_01702	.	.	NP_000546	XP_011529	protein_co	ENSG0000	1641	2714
DCXR	126.281	124.397	114.182	167.537	131.591	94.3308	dicarbonyl and L-xylulose reductase [Source:HGNC Symbol;Acc:H	chr17:7999	NM_00119	.	.	.	NP_001182	.	protein_co	ENSG0000	51181	18985
DDA1	20.2045	19.3321	18.5863	21.9542	18.846	17.3527	DET1 and DDB1 associated 1 [Source:HGNC Symbol;Acc:HGNC:28	chr19:1742	NM_02405	.	.	.	NP_076955	.	protein_co	ENSG0000	79016	28360

DDAH1	7.24835	7.5559	12.1663	6.62444	7.5235	10.9329	dimethylarginine dimethylaminohydrolase 1 [Source:HGNC Symb	chr1:85784	NM_00113	XM_01154	.	.	NP_00112	XP_00527	protein_co	ENSG0000	23576	2715
DDAH2	25.6687	26.6323	31.1125	31.1796	27.0094	34.6406	dimethylarginine dimethylaminohydrolase 2 [Source:HGNC Symb	chr6:31694	NM_00130	XM_01151	.	.	NP_00128	XP_011512	protein_co	ENSG0000	23564	2716
DDB1	99.4999	105.789	98.1318	115.738	100.521	85.6557	damage specific DNA binding protein 1 [Source:HGNC Symbol;Ac	chr11:6106	NM_00192	.	.	.	NP_00191	.	protein_co	ENSG0000	1642	2717
DDB2	28.1352	26.8162	35.2125	32.1074	28.0059	23.0769	damage specific DNA binding protein 2 [Source:HGNC Symbol;Ac	chr11:4723	NM_00010	.	.	.	NP_00009	.	protein_co	ENSG0000	1643	2718
DDC	0.095506	0.139402	0	0.158274	0.054288	0.148001	dopa decarboxylase [Source:HGNC Symbol;Acc:HGNC:2719]	chr7:50511	NM_00079	XM_01151	.	.	NP_00078	XP_005271	protein_co	ENSG0000	1644	2719
DDHD1	7.67231	8.36348	8.01952	10.0901	12.9758	10.9226	DDHD domain containing 1 [Source:HGNC Symbol;Acc:HGNC:197	chr14:5350	NM_00116	XM_01153	.	.	NP_00115	XP_005268	protein_co	ENSG0000	80821	19714
DDHD2	12.2292	11.6303	13.1717	10.7804	12.5557	15.0169	DDHD domain containing 2 [Source:HGNC Symbol;Acc:HGNC:291	chr8:38088	NM_00116	XM_01701	.	.	NP_00115	XP_005273	protein_co	ENSG0000	23259	29106
DDI1	0	0	0	0	0	0	DNA damage inducible 1 homolog 1 [Source:HGNC Symbol;Acc:H	chr11:1037	NM_00100	.	.	.	NP_00100	.	protein_co	ENSG0000	414301	18961
DDI2	9.21953	9.64949	10.5016	7.51708	9.52428	8.32338	DNA damage inducible 1 homolog 2 [Source:HGNC Symbol;Acc:H	chr1:15943	NM_03234	.	.	.	NP_11571	.	protein_co	ENSG0000	84301	24578
DDIAS	9.83358	9.67613	8.85346	9.57677	14.7765	10.4164	DNA damage induced apoptosis suppressor [Source:HGNC Symbo	chr11:8261	NM_14501	XM_01701	.	.	NP_65945	XP_011543	protein_co	ENSG0000	220042	26351
DDIT3	70.3019	63.4647	14.685	31.4359	23.463	94.0415	DNA damage inducible transcript 3 [Source:HGNC Symbol;Acc:HG	chr12:5785	NM_00119	.	.	.	NP_00118	.	protein_co	ENSG0000	1649	2726
DDIT4	114.992	128.426	75.1956	96.1209	101.549	342.206	DNA damage inducible transcript 4 [Source:HGNC Symbol;Acc:HG	chr10:7403	NM_01905	.	.	.	NP_06193	.	protein_co	ENSG0000	54541	24944
DDIT4L	3.69776	3.15455	6.60772	5.7642	7.51965	11.1823	DNA damage inducible transcript 4 like [Source:HGNC Symbol;Ac	chr4:10110	NM_14524	XM_00526	.	.	NP_66028	XP_005262	protein_co	ENSG0000	115265	30555
DDN	5.20501	5.44627	5.4529	7.34323	5.86692	4.27073	dendrin [Source:HGNC Symbol;Acc:HGNC:24458]	chr12:4938	NM_01508	.	.	.	NP_05590	XP_011536	protein_co	ENSG0000	23109	24458
DDO	0	0	0.102364	0	0	0	D-aspartate oxidase [Source:HGNC Symbol;Acc:HGNC:2727]	chr6:11071	NM_00364	.	.	.	NP_00364	.	protein_co	ENSG0000	8528	2727
DDOST	349.735	357.327	290.516	321.452	281.206	292.132	dolichyl-diphosphooligosaccharide--protein glycosyltransferase n	chr1:20978	NM_00521	.	.	.	NP_00520	.	protein_co	ENSG0000	1650	2728
DDR1	79.2501	82.7195	85.2682	60.4885	57.2257	55.5131	discoidin domain receptor tyrosine kinase 1 [Source:HGNC Symbo	chr6:30848	NM_00120	XM_01701	NR_03978	.	NP_00118	XP_006715	protein_co	ENSG0000	100616237	2730
DDR2	0.162075	0.325181	0.270256	0.104059	0.148369	0.172945	discoidin domain receptor tyrosine kinase 2 [Source:HGNC Symbo	chr1:16260	NM_00101	XM_01150	.	.	NP_00101	XP_006711	protein_co	ENSG0000	4921	2731
DDRGK1	22.1181	22.1278	16.2819	23.467	20.5694	15.8678	DDRGK domain containing 1 [Source:HGNC Symbol;Acc:HGNC:16	chr20:3170	NM_02393	.	.	.	NP_07642	.	protein_co	ENSG0000	65992	16110
DDT	100.804	93.4517	110.162	78.3541	62.1111	54.9278	D-dopachrome tautomerase [Source:HGNC Symbol;Acc:HGNC:27	chr22:2430	NM_00108	.	.	.	NP_00107	XP_011528	protein_co	ENSG0000	100037417	2732
DDTL	1.00112	0.872564	0.739664	0.660357	0.569892	0.419158	D-dopachrome tautomerase-like [Source:HGNC Symbol;Acc:HGN	chr22:2430	NM_00108	.	.	.	NP_00107	.	protein_co	ENSG0000	100037417	33446
DDX1	80.4551	77.9503	70.8148	92.7513	91.3727	77.0425	DEAD/H-box helicase 1 [Source:HGNC Symbol;Acc:HGNC:2734]	chr2:15731	NM_00493	.	.	.	NP_00493	.	protein_co	ENSG0000	1653	2734
DDX10	23.2958	23.1625	21.2575	26.4327	26.9158	20.0824	DEAD-box helicase 10 [Source:HGNC Symbol;Acc:HGNC:2735]	chr11:1085	NM_00439	.	.	.	NP_00438	.	protein_co	ENSG0000	1662	2735
DDX11	19.3861	20.95	18.6483	19.1881	18.438	13.9922	DEAD/H-box helicase 11 [Source:HGNC Symbol;Acc:HGNC:2736]	chr12:3122	NM_00125	XM_01701	.	.	NP_00124	XP_005253	protein_co	ENSG0000	1663	2736
DDX17	75.5823	74.5336	80.0946	58.2488	70.6781	70.1455	DEAD-box helicase 17 [Source:HGNC Symbol;Acc:HGNC:2740]	chr22:3886	NM_00109	.	.	.	NP_00109	.	protein_co	ENSG0000	10521	2740
DDX18	58.6078	58.9577	49.9927	66.4875	76.5323	55.6657	DEAD-box helicase 18 [Source:HGNC Symbol;Acc:HGNC:2741]	chr2:11857	NM_00677	.	.	.	NP_00676	.	protein_co	ENSG0000	8886	2741
DDX19A	24.4683	24.3249	23.4944	26.5887	24.9682	19.368	DEAD-box helicase 19A [Source:HGNC Symbol;Acc:HGNC:25628]	chr16:7032	NM_00132	XM_00525	.	.	NP_00130	.	protein_co	ENSG0000	55308	25628
DDX19B	9.8806	10.7307	9.79646	12.407	11.5227	10.7098	DEAD-box helicase 19B [Source:HGNC Symbol;Acc:HGNC:2742]	chr16:7032	NM_00101	XM_01152	.	.	NP_00101	XP_006721	protein_co	ENSG0000	11269	2742
DDX20	14.3714	13.1331	12.2739	13.0106	14.5402	12.2578	DEAD-box helicase 20 [Source:HGNC Symbol;Acc:HGNC:2743]	chr1:11226	NM_00720	.	.	.	NP_00913	.	protein_co	ENSG0000	11218	2743
DDX21	83.955	84.0454	84.201	93.2514	115.175	102.717	DEAD-box helicase 21 [Source:HGNC Symbol;Acc:HGNC:2744]	chr10:7071	NM_00125	XM_01154	.	.	NP_00124	XP_011538	protein_co	ENSG0000	9188	2744
DDX23	33.2825	34.1325	30.7416	41.084	35.777	22.8793	DEAD-box helicase 23 [Source:HGNC Symbol;Acc:HGNC:17347]	chr12:4922	NM_00481	.	.	.	NP_00480	.	protein_co	ENSG0000	9416	17347
DDX24	80.8531	77.9428	75.2164	97.3043	90.4265	69.0197	DEAD-box helicase 24 [Source:HGNC Symbol;Acc:HGNC:13266]	chr14:9449	NM_02041	.	.	.	NP_06514	.	protein_co	ENSG0000	57062	13266
DDX25	0.302078	0.272501	0.149766	0.338672	0.144913	0.259765	DEAD-box helicase 25 [Source:HGNC Symbol;Acc:HGNC:18698]	chr11:1257	NM_01326	XM_01701	.	.	NP_03739	XP_011541	protein_co	ENSG0000	29118	18698
DDX27	18.9538	18.8963	16.9891	19.8926	18.7197	15.1148	DEAD-box helicase 27 [Source:HGNC Symbol;Acc:HGNC:15837]	chr20:4783	NM_01789	XM_01152	.	.	NP_06036	XP_011527	protein_co	ENSG0000	55661	15837
DDX28	15.0144	14.5121	15.338	14.0792	12.0277	10.2802	DEAD-box helicase 28 [Source:HGNC Symbol;Acc:HGNC:17330]	chr16:6805	NM_01838	.	.	.	NP_06085	.	protein_co	ENSG0000	55794	17330
DDX31	12.1724	13.0528	11.462	14.1432	11.0824	9.56909	DEAD-box helicase 31 [Source:HGNC Symbol;Acc:HGNC:16715]	chr9:13546	NM_00132	XM_01151	.	.	NP_00130	XP_005272	protein_co	ENSG0000	64794	16715
DDX39A	91.0606	88.0302	63.9767	120.855	97.0576	62.0387	DEAD-box helicase 39A [Source:HGNC Symbol;Acc:HGNC:17821]	chr19:1451	NM_00580	XM_01152	.	.	NP_00579	XP_011525	protein_co	ENSG0000	10212	17821
DDX39B	153.772	146.179	140.507	165.327	143.617	153.333	DEAD-box helicase 39B [Source:HGNC Symbol;Acc:HGNC:13917]	chr6:31496	NM_00464	.	.	.	NP_00463	.	protein_co	ENSG0000	7919	13917
DDX3X	90.634	93.1721	90.3348	96.8112	116.328	118.302	DEAD-box helicase 3, X-linked [Source:HGNC Symbol;Acc:HGNC:2	chrX:41192	NM_00119	XM_01154	NR_12609	.	NP_00118	XP_011542	protein_co	ENSG0000	1654	2745
DDX3Y	2.64294	2.80953	2.77172	0	0.026602	0	DEAD-box helicase 3, Y-linked [Source:HGNC Symbol;Acc:HGNC:2	chrY:15016	NM_00112	XM_00672	.	.	NP_00111	.	protein_co	ENSG0000	8653	2699
DDX4	0	0	0.032384	0.084457	0	0.036543	DEAD-box helicase 4 [Source:HGNC Symbol;Acc:HGNC:18700]	chr5:55033	NM_00114	XM_01154	.	.	NP_00113	XP_011541	protein_co	ENSG0000	54514	18700
DDX41	36.8951	40.2982	36.0852	42.9382	37.0815	31.0653	DEAD-box helicase 41 [Source:HGNC Symbol;Acc:HGNC:18674]	chr5:17693	NM_01622	XM_00671	.	.	NP_05730	.	protein_co	ENSG0000	51428	18674
DDX42	68.0627	70.8499	67.8101	67.5053	69.2969	63.2204	DEAD-box helicase 42 [Source:HGNC Symbol;Acc:HGNC:18676]	chr17:6185	NM_00737	XM_01702	.	.	NP_03139	XP_006721	protein_co	ENSG0000	11325	18676
DDX43	0	0	0	0	0	0.027101	DEAD-box helicase 43 [Source:HGNC Symbol;Acc:HGNC:18677]	chr6:74078	NM_01866	XM_01153	.	.	NP_06113	XP_011534	protein_co	ENSG0000	55510	18677
DDX46	40.6619	40.1413	36.4787	47.5333	50.3996	39.3328	DEAD-box helicase 46 [Source:HGNC Symbol;Acc:HGNC:18681]	chr5:13409	NM_00130	.	.	.	NP_00128	.	protein_co	ENSG0000	9879	18681
DDX47	66.5295	66.4748	67.0783	77.1435	73.0991	65.9548	DEAD-box helicase 47 [Source:HGNC Symbol;Acc:HGNC:18682]	chr12:1296	NM_01635	.	.	.	NP_05743	.	protein_co	ENSG0000	51202	18682
DDX49	33.4935	32.762	27.9404	34.4209	28.6451	20.229	DEAD-box helicase 49 [Source:HGNC Symbol;Acc:HGNC:18684]	chr19:1903	NM_01907	XM_01152	.	.	NP_06194	XP_011526	protein_co	ENSG0000	54555	18684
DDX5	393.321	384.802	383.922	400.126	433.317	340.873	DEAD-box helicase 5 [Source:HGNC Symbol;Acc:HGNC:2746]	chr17:6249	NM_00439	XM_01152	NR_03989	.	NP_00130	XP_005257	protein_co	ENSG0000	100616408	2746
DDX50	15.9937	15.1611	14.4538	14.41	16.8749	16.3271	DEAD-box helicase 50 [Source:HGNC Symbol;Acc:HGNC:17906]	chr10:7066	NM_02404	XM_01154	.	.	NP_07695	XP_005270	protein_co	ENSG0000	79009	17906
DDX51	8.92932	9.95608	10.3966	9.86502	9.3035	7.00285	DEAD-box helicase 51 [Source:HGNC Symbol;Acc:HGNC:20082]	chr12:1326	NM_17506	.	.	.	NP_77823	.	protein_co	ENSG0000	317781	20082
DDX52	10.5553	10.4671	10.6575	11.235	12.9756	11.5993	DEAD-box helicase 52 [Source:HGNC Symbol;Acc:HGNC:20038]	chr17:3596	NM_00129	XM_01152	.	.	NP_00127	XP_011522	protein_co	ENSG0000	11056	20038
DDX53	0	0	0	0	0	0	DEAD-box helicase 53 [Source:HGNC Symbol;Acc:HGNC:20083]	chrX:23018	NM_18269	.	.	.	NP_87435	.	protein_co	ENSG0000	168400	20083
DDX54	30.2869	31.8964	28.6664	36.0084	30.1328	24.5055	DEAD-box helicase 54 [Source:HGNC Symbol;Acc:HGNC:20084]	chr12:1135	NM_00111	.	.	.	NP_00110	.	protein_co	ENSG0000	79039	20084
DDX55	17.9033	16.9406	17.9186	20.1301	20.3027	15.5316	DEAD-box helicase 55 [Source:HGNC Symbol;Acc:HGNC:20085]	chr12:1240	NM_02093	XM_01701	.	.	NP_06598	XP_016875	protein_co	ENSG0000	57696	20085
DDX56	60.0979	61.3782	51.837	67.2924	60.807	38.0854	DEAD-box helicase 56 [Source:HGNC Symbol;Acc:HGNC:18193]	chr7:44605	NM_00125	.	.	.	NP_00124	.	protein_co	ENSG0000	54606	18193
DDX58	14.4364	14.1488	5.17129	8.95466	12.4438	11.2733	DEXD/H-box helicase 58 [Source:HGNC Symbol;Acc:HGNC:19102]	chr9:32455	NM_01431	.	.	.	NP_05512	.	protein_co	ENSG0000	23586	19102
DDX59	7.40851	7.48342	6.88483	6.01298	6.29914	7.66947	DEAD-box helicase 59 [Source:HGNC Symbol;Acc:HGNC:25360]	chr1:20059	NM_00103	XM_01151	.	.	NP_00102	XP_005245	protein_co	ENSG0000	83479	25360
DDX6	58.608	62.5932	52.4796	61.782	72.7477	71.0152	DEAD-box helicase 6 [Source:HGNC Symbol;Acc:HGNC:2747]	chr11:1186	NM_00125	XM_01154	.	.	NP_00124	XP_005271	protein_co	ENSG0000	1656	2747
DDX60	4.62605	4.42784	2.02024	2.13095	3.22116	3.06331	DEXD/H-box helicase 60 [Source:HGNC Symbol;Acc:HGNC:25942]	chr4:16913	NM_01763	XM_01153	.	.	NP_06010	XP_011530	protein_co	ENSG0000	55601	25942

DDX60L	4.62628	4.50469	5.14699	3.59174	5.75886	6.4446	DEAD-box helicase 60-like [Source:HGNC Symbol;Acc:HGNC:2642	chr4:16927	NM_00101	XM_01153	.	.	NP_001012	XP_005263	protein_co	ENSG0000	91351	26429
DEAF1	8.83452	8.39982	9.28245	8.95793	6.9976	6.45685	DEAF1, transcription factor [Source:HGNC Symbol;Acc:HGNC:146	chr11:6442	NM_02100	XM_01151	.	.	NP_066288	XP_011518	protein_co	ENSG0000	10522	14677
DEC1	0	0	0	0	0	0	deleted in esophageal cancer 1 [Source:HGNC Symbol;Acc:HGNC:	chr9:11790	NM_01741	XM_01151	.	.	NP_059114	XP_011517	protein_co	ENSG0000	50514	23658
DECR1	43.4763	40.9701	57.1925	51.4697	49.562	68.0932	2,4-dienoyl-CoA reductase 1, mitochondrial [Source:HGNC Symbo	chr8:91013	NM_00135	XM_01701	.	.	NP_001350	XP_005250	protein_co	ENSG0000	1666	2753
DECR2	10.2791	10.6369	7.97683	7.01622	5.98069	6.94185	2,4-dienoyl-CoA reductase 2, peroxisomal [Source:HGNC Symbol;	chr16:4518	NM_02066	.	.	.	NP_065715	.	protein_co	ENSG0000	26063	2754
DEDD	18.6735	19.4448	17.0055	19.2654	17.8497	19.3091	death effector domain containing [Source:HGNC Symbol;Acc:HGN	chr1:16107	NM_00103	XM_00524	.	.	NP_001034	XP_005245	protein_co	ENSG0000	9191	2755
DEDD2	31.6498	32.7439	26.6763	33.5066	30.3261	23.7532	death effector domain containing 2 [Source:HGNC Symbol;Acc:HG	chr19:4270	NM_00127	XM_01152	NR_07304	.	NP_00125	XP_011524	protein_co	ENSG0000	162989	24450
DEF6	19.8316	18.8124	17.8388	22.8013	20.1758	20.2645	DEF6, guanine nucleotide exchange factor [Source:HGNC Symbol;	chr6:35265	NM_02204	.	.	.	NP_071330	.	protein_co	ENSG0000	50619	2760
DEF8	61.6363	56.253	59.7038	70.8802	54.8653	50.1985	differentially expressed in FDCP 8 homolog [Source:HGNC Symbo	chr16:9001	NM_00124	XM_01702	.	.	NP_001229	XP_005256	protein_co	ENSG0000	54849	25969
DEFA1	0	0	0	0	0	0	defensin alpha 1 [Source:HGNC Symbol;Acc:HGNC:2761]	chr8:68351	NM_00408	.	.	.	NP_001289	XP_011533	protein_co	ENSG0000	728358	2761
DEFA1B	0	0	0	0	0	0	defensin alpha 1B [Source:HGNC Symbol;Acc:HGNC:33596]	chr8:68542	NM_00104	.	.	.	NP_001035	XP_011533	protein_co	ENSG0000	728358	33596
DEFA3	0	0	0	0	0	0	defensin alpha 3 [Source:HGNC Symbol;Acc:HGNC:2762]	chr8:68733	NM_00521	.	.	.	NP_005208	XP_011533	protein_co	ENSG0000	1668	2762
DEFA4	0	0	0	0	0	0	defensin alpha 4 [Source:HGNC Symbol;Acc:HGNC:2763]	chr8:67933	NM_00192	.	.	.	NP_001916	.	protein_co	ENSG0000	1669	2763
DEFA5	0	0	0	0	0	0	defensin alpha 5 [Source:HGNC Symbol;Acc:HGNC:2764]	chr8:69128	NM_02101	.	.	.	NP_066290	.	protein_co	ENSG0000	1670	2764
DEFA6	0	0	0	0	0	0	defensin alpha 6 [Source:HGNC Symbol;Acc:HGNC:2765]	chr8:67822	NM_00192	.	.	.	NP_001917	.	protein_co	ENSG0000	1671	2765
DEFB1	0	0	0	0.296625	0	0	defensin beta 1 [Source:HGNC Symbol;Acc:HGNC:2766]	chr8:67280	NM_00521	.	.	.	NP_005209	.	protein_co	ENSG0000	1672	2766
DEFB103A	0	0	0	0	0	0	defensin beta 103A [Source:HGNC Symbol;Acc:HGNC:15967]	chr8:77387	NM_00108	XM_01703	.	.	NP_001075	XP_016885	protein_co	ENSG0000	414325	15967
DEFB103B	0	0	0	0	0	0	defensin beta 103B [Source:HGNC Symbol;Acc:HGNC:31702]	chr8:72864	NM_01866	XM_01703	.	.	NP_061131	XP_016885	protein_co	ENSG0000	414325	31702
DEFB104A	0	0	0	0	0	0	defensin beta 104A [Source:HGNC Symbol;Acc:HGNC:18115]	chr8:76939	NM_08038	.	.	.	NP_525128	XP_016885	protein_co	ENSG0000	107984148	18115
DEFB104B	0	0	0	0	0	0	defensin beta 104B [Source:HGNC Symbol;Acc:HGNC:26165]	chr8:73278	NM_00104	.	.	.	NP_001035	XP_016885	protein_co	ENSG0000	107984148	26165
DEFB105A	0	0	0	0	0	0	defensin beta 105A [Source:HGNC Symbol;Acc:HGNC:18087]	chr8:76794	NM_15225	.	.	.	NP_689463	.	protein_co	ENSG0000	245908	18087
DEFB105B	0	0	0	0	0	0	defensin beta 105B [Source:HGNC Symbol;Acc:HGNC:29930]	chr8:73451	NM_00104	.	.	.	NP_001035	.	protein_co	ENSG0000	504180	29930
DEFB106A	0	0	0	0	0	0	defensin beta 106A [Source:HGNC Symbol;Acc:HGNC:18088]	chr8:76826	NM_15225	.	.	.	NP_689464	.	protein_co	ENSG0000	245909	18088
DEFB106B	0	0	0	0	0	0	defensin beta 106B [Source:HGNC Symbol;Acc:HGNC:28879]	chr8:73400	NM_00104	.	.	.	NP_001035	.	protein_co	ENSG0000	503841	28879
DEFB107A	0	0	0	0	0	0	defensin beta 107A [Source:HGNC Symbol;Acc:HGNC:18086]	chr8:76692	NM_00103	.	.	.	NP_001032	.	protein_co	ENSG0000	245910	18086
DEFB107B	0	0	0	0	0	0	defensin beta 107B [Source:HGNC Symbol;Acc:HGNC:31918]	chr8:73533	NM_00104	.	.	.	NP_001035	.	protein_co	ENSG0000	503614	31918
DEFB108B	0	0	0	0	0	0	defensin beta 108B [Source:HGNC Symbol;Acc:HGNC:29966]	chr11:7154	NM_00100	.	.	.	NP_001002	XP_016885	protein_co	ENSG0000	107987553	29966
DEFB110	0	0	0	0	0	0	defensin beta 110 [Source:HGNC Symbol;Acc:HGNC:18091]	chr6:49976	NM_00103	.	.	.	NP_001032	.	protein_co	ENSG0000	245913	18091
DEFB112	0	0	0	0	0	0	defensin beta 112 [Source:HGNC Symbol;Acc:HGNC:18093]	chr6:50011	NM_00103	.	.	.	NP_001032	.	protein_co	ENSG0000	245915	18093
DEFB113	0	0	0	0	0	0	defensin beta 113 [Source:HGNC Symbol;Acc:HGNC:18094]	chr6:49936	NM_00103	.	.	.	NP_001032	.	protein_co	ENSG0000	245927	18094
DEFB114	0	0	0	0	0	0	defensin beta 114 [Source:HGNC Symbol;Acc:HGNC:18095]	chr6:49927	NM_00103	.	.	.	NP_001032	.	protein_co	ENSG0000	245928	18095
DEFB115	0	0	0	0	0	0	defensin beta 115 [Source:HGNC Symbol;Acc:HGNC:18096]	chr20:2984	NM_00103	.	.	.	NP_001032	.	protein_co	ENSG0000	245929	18096
DEFB116	0	0	0	0	0	0	defensin beta 116 [Source:HGNC Symbol;Acc:HGNC:18097]	chr20:2989	NM_00103	.	.	.	NP_001032	.	protein_co	ENSG0000	245930	18097
DEFB118	0	0	0	0	0	0	defensin beta 118 [Source:HGNC Symbol;Acc:HGNC:16196]	chr20:2995	NM_05411	.	.	.	NP_473453	.	protein_co	ENSG0000	117285	16196
DEFB119	0	0	0	0	0	0	defensin beta 119 [Source:HGNC Symbol;Acc:HGNC:18099]	chr20:2996	NM_00127	.	.	.	NP_001258	.	protein_co	ENSG0000	245932	18099
DEFB121	0	0	0	0	0	0	defensin beta 121 [Source:HGNC Symbol;Acc:HGNC:18101]	chr20:2999	NM_00101	.	.	.	NP_001011	.	protein_co	ENSG0000	245934	18101
DEFB123	0	0	0	0	0	0	defensin beta 123 [Source:HGNC Symbol;Acc:HGNC:18103]	chr20:3002	NM_15332	.	.	.	NP_697019	.	protein_co	ENSG0000	245936	18103
DEFB124	0	0	0	0	0	0	defensin beta 124 [Source:HGNC Symbol;Acc:HGNC:18104]	chr20:3005	NM_00103	.	.	.	NP_001032	.	protein_co	ENSG0000	245937	18104
DEFB125	0	0	0	0	0	0	defensin beta 125 [Source:HGNC Symbol;Acc:HGNC:18105]	chr20:6835	NM_15332	.	.	.	NP_697020	.	protein_co	ENSG0000	245938	18105
DEFB126	0	0	0	0	0	0	defensin beta 126 [Source:HGNC Symbol;Acc:HGNC:15900]	chr20:1230	NM_03093	.	.	.	NP_112193	.	protein_co	ENSG0000	81623	15900
DEFB127	0	0	0	0	0	0	defensin beta 127 [Source:HGNC Symbol;Acc:HGNC:16206]	chr20:1381	NM_13907	.	.	.	NP_620713	.	protein_co	ENSG0000	140850	16206
DEFB128	0	0	0	0	0	0	defensin beta 128 [Source:HGNC Symbol;Acc:HGNC:18106]	chr20:1684	NM_00103	.	.	.	NP_001032	.	protein_co	ENSG0000	245939	18106
DEFB129	0	0	0	0	0	0	defensin beta 129 [Source:HGNC Symbol;Acc:HGNC:16218]	chr20:2078	NM_08083	.	.	.	NP_543021	.	protein_co	ENSG0000	140881	16218
DEFB130	0	0	0	0	0	0	defensin beta 130 [Source:HGNC Symbol;Acc:HGNC:18107]	chr8:12168	NM_00103	.	.	.	NP_001032	.	protein_co	ENSG0000	245940	18107
DEFB131	0	0	0	0	0	0	defensin beta 131 [Source:HGNC Symbol;Acc:HGNC:18108]	chr4:94462	NM_00104	.	.	.	NP_001035	XP_011547	protein_co	ENSG0000	105369240	18108
DEFB132	0	0	0	0	0	0	defensin beta 132 [Source:HGNC Symbol;Acc:HGNC:33806]	chr20:2383	NM_20746	.	.	.	NP_997352	.	protein_co	ENSG0000	400830	33806
DEFB133	0	0	0	0	0	0	defensin beta 133 [Source:HGNC Symbol;Acc:HGNC:31331]	chr6:49913	NM_00116	XM_01701	.	.	NP_001159	XP_016866	protein_co	ENSG0000	403339	31331
DEFB134	0	0	0.07353	0	0.093594	0	defensin beta 134 [Source:HGNC Symbol;Acc:HGNC:32399]	chr8:11850	NM_00130	XM_01703	.	.	NP_001289	XP_016869	protein_co	ENSG0000	105379765	32399
DEFB135	0	0	0	0	0	0	defensin beta 135 [Source:HGNC Symbol;Acc:HGNC:32400]	chr8:11839	NM_00103	.	.	.	NP_001028	XP_016885	protein_co	ENSG0000	107987561	32400
DEFB136	0	0	0	0	0	0	defensin beta 136 [Source:HGNC Symbol;Acc:HGNC:34433]	chr8:11831	NM_00103	.	.	.	NP_001028	XP_016885	protein_co	ENSG0000	107987560	34433
DEFB4A	0	0	0	0	0	0	defensin beta 4A [Source:HGNC Symbol;Acc:HGNC:2767]	chr8:77521	NM_00494	.	.	.	NP_004933	.	protein_co	ENSG0000	1673	2767
DEFB4B	0	0	0	0	0	0	defensin beta 4B [Source:HGNC Symbol;Acc:HGNC:30193]	chr8:72723	NM_00120	.	.	.	NP_001192	.	protein_co	ENSG0000	100289462	30193
DEGS1	31.1239	28.0397	30.336	28.2484	32.5866	26.4942	delta 4-desaturase, sphingolipid 1 [Source:HGNC Symbol;Acc:HGN	chr1:22437	NM_00367	.	.	.	NP_003667	.	protein_co	ENSG0000	8560	13709
DEGS2	0.720318	0.270675	1.1058	0.294279	0.175072	0.62551	delta 4-desaturase, sphingolipid 2 [Source:HGNC Symbol;Acc:HGN	chr14:1004	NM_20691	.	.	.	NP_996801	.	protein_co	ENSG0000	123099	20113
DEK	39.7257	37.3334	39.5915	37.688	49.2983	54.7159	DEK proto-oncogene [Source:HGNC Symbol;Acc:HGNC:2768]	chr6:18224	NM_00113	.	.	.	NP_001128	XP_016866	protein_co	ENSG0000	7913	2768
DENND1A	5.74484	6.3003	6.48527	5.94877	6.54847	7.50617	DENN domain containing 1A [Source:HGNC Symbol;Acc:HGNC:29	chr9:12614	NM_02094	XM_01151	.	.	NP_065997	XP_005252	protein_co	ENSG0000	57706	29324
DENND1B	1.50856	1.18457	1.63961	1.19463	1.42946	2.10324	DENN domain containing 1B [Source:HGNC Symbol;Acc:HGNC:28	chr1:19747	NM_00119	XM_01700	.	.	NP_001182	XP_005244	protein_co	ENSG0000	163486	28404
DENND1C	0.140006	0.098598	0.285573	0.150793	0.353956	0.358997	DENN domain containing 1C [Source:HGNC Symbol;Acc:HGNC:26	chr19:6463	NM_00129	XM_01152	.	.	NP_001277	XP_006722	protein_co	ENSG0000	79958	26225
DENND2A	0.179304	0.233673	0.23482	0.156352	0.061417	0.133012	DENN domain containing 2A [Source:HGNC Symbol;Acc:HGNC:22	chr7:14021	NM_00131	XM_01701	.	.	NP_001304	XP_005250	protein_co	ENSG0000	27147	22212

DENND2C	1.51429	1.36002	1.73291	1.18963	1.86308	2.1263	DENN domain containing 2C [Source:HGNC Symbol;Acc:HGNC:24	chr1:11512	NM_00125	.	.	.	NP_00124	.	protein_co	ENSG0000	163259	24748
DENND2D	12.4804	11.8136	14.0909	11.2024	9.84745	9.0296	DENN domain containing 2D [Source:HGNC Symbol;Acc:HGNC:26	chr1:11172	NM_00127	XM_01154	.	.	NP_00125	XP_00671	protein_co	ENSG0000	79961	26192
DENND3	3.70446	3.76902	4.60834	3.74138	4.06143	4.1436	DENN domain containing 3 [Source:HGNC Symbol;Acc:HGNC:291	chr8:14213	NM_01495	XM_01151	.	.	NP_05577	XP_00525	protein_co	ENSG0000	22898	29134
DENND4A	2.53877	2.49781	3.28147	1.58121	2.72613	3.92266	DENN domain containing 4A [Source:HGNC Symbol;Acc:HGNC:24	chr15:6590	NM_00114	XM_01702	.	.	NP_00113	XP_005254	protein_co	ENSG0000	10260	24321
DENND4B	10.3404	10.447	10.8802	10.4687	10.3493	9.91887	DENN domain containing 4B [Source:HGNC Symbol;Acc:HGNC:29	chr1:1539	NM_01485	XM_01151	.	.	NP_05567	XP_005245	protein_co	ENSG0000	9909	29044
DENND4C	5.97178	5.39161	6.97591	5.13208	6.87608	7.11436	DENN domain containing 4C [Source:HGNC Symbol;Acc:HGNC:26	chr9:1923	NM_01792	XM_00671	.	.	NP_06039	XP_00671	protein_co	ENSG0000	55667	26079
DENND5A	12.5711	13.457	15.008	11.6086	11.552	14.9537	DENN domain containing 5A [Source:HGNC Symbol;Acc:HGNC:19	chr11:916	NM_00124	XM_01151	.	.	NP_00123	XP_005252	protein_co	ENSG0000	23258	19344
DENND5B	3.05036	3.70586	6.43704	3.21889	3.32406	3.67015	DENN domain containing 5B [Source:HGNC Symbol;Acc:HGNC:28	chr12:3153	NM_00130	XM_01152	.	.	NP_00129	XP_00525	protein_co	ENSG0000	160518	28338
DENND6A	6.20343	5.96378	6.94171	4.7297	5.87319	8.95446	DENN domain containing 6A [Source:HGNC Symbol;Acc:HGNC:26	chr3:57611	NM_15267	XM_01153	.	.	NP_68989	XP_00671	protein_co	ENSG0000	201627	26635
DENND6B	1.39916	1.43036	1.70339	1.06188	0.736481	1.13333	DENN domain containing 6B [Source:HGNC Symbol;Acc:HGNC:32	chr22:5074	NM_00100	XM_01153	.	.	NP_00100	XP_005261	protein_co	ENSG0000	414918	32690
DENR	34.3122	37.7686	39.3902	36.6756	41.6849	46.5528	density regulated re-initiation and release factor [Source:HGNC S	chr12:1232	NM_00367	.	.	.	NP_00366	.	protein_co	ENSG0000	8562	2769
DEPDC1	15.7092	14.9911	18.477	20.0002	30.5984	33.8494	DEP domain containing 1 [Source:HGNC Symbol;Acc:HGNC:22949	chr1:6893	NM_00111	.	.	.	NP_00110	.	protein_co	ENSG0000	55635	22949
DEPDC1B	20.0169	21.3256	15.5152	32.5745	31.9816	26.2198	DEP domain containing 1B [Source:HGNC Symbol;Acc:HGNC:2490	chr5:59892	NM_00114	XM_01154	.	.	NP_00113	XP_011541	protein_co	ENSG0000	55789	24902
DEPDC4	0.986941	0.639987	0.865009	0.52964	1.08049	0.954481	DEP domain containing 4 [Source:HGNC Symbol;Acc:HGNC:22952	chr12:1006	NM_00131	.	.	.	NP_00130	XP_016874	protein_co	ENSG0000	120863	22952
DEPDC5	4.12319	4.06817	4.07153	4.3669	4.48865	4.85674	DEP domain containing 5 [Source:HGNC Symbol;Acc:HGNC:18423	chr22:3214	NM_00100	XM_01702	.	.	NP_00100	XP_005261	protein_co	ENSG0000	9681	18423
DEPDC7	5.86078	5.92301	7.03872	5.66803	9.69816	7.58701	DEP domain containing 7 [Source:HGNC Symbol;Acc:HGNC:29899	chr11:3303	NM_00107	.	.	.	NP_00107	.	protein_co	ENSG0000	91614	29899
DEPTOR	1.86695	1.39822	3.82122	1.46536	1.59533	2.72407	DEP domain containing MTOR-interacting protein [Source:HGNC	chr8:12088	NM_00128	.	.	.	NP_00126	.	protein_co	ENSG0000	64798	22953
DERA	23.9338	25.5781	24.2222	26.4837	22.9232	28.9268	deoxyribose-phosphate aldolase [Source:HGNC Symbol;Acc:HGN	chr12:1606	NM_00130	.	.	.	NP_00128	.	protein_co	ENSG0000	51071	24269
DERL1	48.0529	44.8981	47.4655	45.2942	49.926	54.0082	derlin 1 [Source:HGNC Symbol;Acc:HGNC:28454]	chr8:12402	NM_00113	XM_01151	.	.	NP_00112	XP_00671	protein_co	ENSG0000	79139	28454
DERL2	27.6159	23.0197	14.7756	20.7687	23.4483	17.5641	derlin 2 [Source:HGNC Symbol;Acc:HGNC:17943]	chr17:5374	NM_00130	XM_01152	.	.	NP_00129	XP_011522	protein_co	ENSG0000	51009	17943
DERL3	0	0	0	0	0	0	derlin 3 [Source:HGNC Symbol;Acc:HGNC:14236]	chr22:2412	NM_00100	XM_01153	.	.	NP_00100	XP_011528	protein_co	ENSG0000	91319	14236
DES	0.888555	0.683937	1.23588	1.89974	1.62917	2.90212	desmin [Source:HGNC Symbol;Acc:HGNC:2770]	chr2:22028	NM_00192	.	.	.	NP_00191	.	protein_co	ENSG0000	1674	2770
DESI1	21.169	21.4348	19.8095	22.6764	20.3125	15.7613	desumoylating isopeptidase 1 [Source:HGNC Symbol;Acc:HGNC:2	chr22:4199	NM_01570	.	.	.	NP_05651	.	protein_co	ENSG0000	27351	24577
DESI2	21.8001	22.4938	22.6823	23.3556	28.315	35.8939	desumoylating isopeptidase 2 [Source:HGNC Symbol;Acc:HGNC:2	chr1:24481	NM_00129	XM_01154	.	.	NP_00128	XP_011542	protein_co	ENSG0000	51029	24264
DET1	2.34711	3.36868	2.29932	2.0298	2.17838	2.13665	de-etiolated homolog 1 (Arabidopsis) [Source:HGNC Symbol;Acc:	chr15:8905	NM_00114	XM_01152	.	.	NP_00113	XP_01152	protein_co	ENSG0000	55070	25477
DEXI	28.2718	26.906	26.1011	30.7733	26.0941	27.0725	Dexi homolog [Source:HGNC Symbol;Acc:HGNC:13267]	chr16:1097	NM_01401	.	.	.	NP_05473	XP_016878	protein_co	ENSG0000	28955	13267
DFFA	32.9394	33.7303	35.9971	39.3947	34.9881	25.57	DNA fragmentation factor subunit alpha [Source:HGNC Symbol;A	chr1:1051	NM_00440	.	.	.	NP_00439	.	protein_co	ENSG0000	1676	2772
DFFB	9.04811	7.92434	8.44463	8.33149	7.4889	4.25114	DNA fragmentation factor subunit beta [Source:HGNC Symbol;Ac	chr1:37738	NM_00128	XM_01154	.	.	NP_00126	.	protein_co	ENSG0000	1677	2773
DFNA5	7.47768	6.84513	7.7943	5.19617	4.44924	5.92269	DFNA5, deafness associated tumor suppressor [Source:HGNC Syn	chr7:24737	NM_00112	XM_01701	.	.	NP_00112	XP_016867	protein_co	ENSG0000	1687	2810
DFNB59	0.775073	0.614908	1.01137	0.418849	0.60695	1.25291	deafness, autosomal recessive 59 [Source:HGNC Symbol;Acc:HGN	chr2:17931	NM_00104	XM_01151	.	.	NP_00103	XP_00524	protein_co	ENSG0000	494513	29502
DGAT1	35.1307	32.8095	37.1595	36.5796	28.5179	30.3034	diacylglycerol O-acyltransferase 1 [Source:HGNC Symbol;Acc:HGN	chr8:14553	NM_01207	XM_01151	NR_10690	.	NP_03621	XP_011515	protein_co	ENSG0000	102465511	2843
DGAT2	4.29598	4.616	3.28267	3.9615	2.92268	3.3647	diacylglycerol O-acyltransferase 2 [Source:HGNC Symbol;Acc:HGN	chr11:7547	NM_00125	.	.	.	NP_00124	XP_011543	protein_co	ENSG0000	84649	16940
DGAT2L6	0	0	0	0	0	0	diacylglycerol O-acyltransferase 2 like 6 [Source:HGNC Symbol;Ac	chrX:69397	NM_19851	.	.	.	NP_94091	.	protein_co	ENSG0000	347516	23250
DGCR14	5.09367	4.66511	3.61418	5.72705	4.8164	3.58215	DiGeorge syndrome critical region gene 14 [Source:HGNC Symbol	chr22:1911	NM_02271	XM_00672	.	.	NP_07321	XP_005261	protein_co	ENSG0000	8220	16817
DGCR2	24.1455	25.3554	24.0481	24.9864	23.0714	21.3438	DiGeorge syndrome critical region gene 2 [Source:HGNC Symbol;	chr22:1902	NM_00117	.	.	.	NP_00116	.	protein_co	ENSG0000	9993	2845
DGCR6	10.0487	9.80848	10.1547	6.67226	6.84419	6.21194	DiGeorge syndrome critical region gene 6 [Source:HGNC Symbol;	chr22:1889	NM_00567	XM_01153	.	.	NP_00566	XP_011528	protein_co	ENSG0000	8214	2846
DGCR6L	40.2373	36.4374	32.2024	45.3873	31.8381	24.9663	DiGeorge syndrome critical region gene 6-like [Source:HGNC Sym	chr22:2030	NM_03325	XM_01153	.	.	NP_15028	.	protein_co	ENSG0000	85359	18551
DGCR8	14.7199	14.5261	12.9047	13.9057	13.9521	12.2031	DGCR8 microprocessor complex subunit [Source:HGNC Symbol;A	chr22:2006	NM_00119	.	NR_03170	.	NP_00117	.	protein_co	ENSG0000	100500860	2847
DGKA	12.879	12.7725	15.2138	13.313	12.4329	10.8107	diacylglycerol kinase alpha [Source:HGNC Symbol;Acc:HGNC:284	chr12:5629	NM_00134	XM_01701	.	.	NP_00133	XP_005268	protein_co	ENSG0000	1606	2849
DGKB	0	0.037951	0.036297	0	0.015411	0.054132	diacylglycerol kinase beta [Source:HGNC Symbol;Acc:HGNC:2850]	chr7:14184	NM_00408	XM_01151	.	.	NP_00407	XP_005249	protein_co	ENSG0000	1607	2850
DGKD	10.8365	12.9158	11.8906	10.646	10.6908	10.5866	diacylglycerol kinase delta [Source:HGNC Symbol;Acc:HGNC:2851	chr2:2342	NM_00364	XM_01700	.	.	NP_00363	XP_00524	protein_co	ENSG0000	8527	2851
DGKE	1.15488	1.40494	1.10112	1.11683	1.34583	1.54679	diacylglycerol kinase epsilon [Source:HGNC Symbol;Acc:HGNC:28	chr17:5491	NM_00364	XM_01152	.	.	NP_00363	XP_011523	protein_co	ENSG0000	8526	2852
DGKG	2.42754	2.55277	1.55021	2.22466	2.28655	1.51605	diacylglycerol kinase gamma [Source:HGNC Symbol;Acc:HGNC:28	chr3:1858	NM_00108	.	.	.	NP_00107	.	protein_co	ENSG0000	1608	2853
DGKH	1.26765	1.30282	1.97476	0.780165	1.24555	1.66638	diacylglycerol kinase eta [Source:HGNC Symbol;Acc:HGNC:2854]	chr13:4261	NM_00120	.	NR_12371	.	NP_00119	.	protein_co	ENSG0000	160851	2854
DGKI	0	0	0	0	0	0.005869	diacylglycerol kinase iota [Source:HGNC Symbol;Acc:HGNC:2855]	chr7:1370	NM_00471	XM_01151	.	.	NP_00130	XP_011514	protein_co	ENSG0000	9162	2855
DGKK	0	0	0	0	0	0	diacylglycerol kinase kappa [Source:HGNC Symbol;Acc:HGNC:323	chrX:5010	NM_00101	.	.	.	NP_00101	.	protein_co	ENSG0000	139189	32395
DGKQ	13.0055	13.0409	13.9174	5.99974	6.12805	7.49208	diacylglycerol kinase theta [Source:HGNC Symbol;Acc:HGNC:285	chr4:95267	NM_00134	XM_01151	.	.	NP_00133	XP_011511	protein_co	ENSG0000	1609	2856
DGKZ	23.4211	24.4839	24.5309	28.9388	24.5143	20.8252	diacylglycerol kinase zeta [Source:HGNC Symbol;Acc:HGNC:2857]	chr11:4635	NM_00110	XM_01701	.	.	NP_00109	XP_005253	protein_co	ENSG0000	8525	2857
DGUOK	75.4724	78.1333	67.9193	92.3012	71.2968	72.0501	deoxyguanosine kinase [Source:HGNC Symbol;Acc:HGNC:2858]	chr2:74153	NM_00131	XM_01153	.	.	NP_00130	XP_01153	protein_co	ENSG0000	1716	2858
DHCR24	89.8564	90.0662	99.2621	114.771	109.401	101.263	24-dehydrocholesterol reductase [Source:HGNC Symbol;Acc:HGN	chr1:55315	NM_01476	.	.	.	NP_05557	.	protein_co	ENSG0000	1718	2859
DHCR7	48.3367	47.7641	60.706	59.4206	53.0384	48.1392	7-dehydrocholesterol reductase [Source:HGNC Symbol;Acc:HGN	chr11:7114	NM_00116	XM_01154	.	.	NP_00115	XP_011543	protein_co	ENSG0000	1717	2860
DHDDS	21.8337	22.1312	20.6945	23.3624	17.3862	14.0011	dehydrodolichyl diphosphate synthase subunit [Source:HGNC Syn	chr1:26758	NM_00124	XM_01700	.	.	NP_00123	XP_00671	protein_co	ENSG0000	79947	20603
DHDH	5.09609	5.06076	5.37049	4.56046	3.47253	3.6569	dihydrodiol dehydrogenase [Source:HGNC Symbol;Acc:HGNC:178	chr19:4943	NM_01447	.	.	.	NP_05529	.	protein_co	ENSG0000	27294	17887
DHFR	25.996	24.3713	22.0175	26.2936	31.1017	24.272	dihydrofolate reductase [Source:HGNC Symbol;Acc:HGNC:2861]	chr5:79922	NM_00079	.	.	.	NP_00078	.	protein_co	ENSG0000	1719	2861
DHFRL1	1.95801	2.20968	1.63815	2.19113	2.02392	2.11411	dihydrofolate reductase 2 [Source:HGNC Symbol;Acc:HGNC:2730	chr3:9377	NM_00119	XM_01151	.	.	NP_00118	XP_01151	protein_co	ENSG0000	200895	27309
DHH	0.116487	0	0.098728	0.124093	0.137568	0.195971	desert hedgehog [Source:HGNC Symbol;Acc:HGNC:2865]	chr12:4948	NM_02104	.	.	.	NP_06638	.	protein_co	ENSG0000	50846	2865
DHODH	11.8875	10.9055	10.8458	12.209	9.54288	8.40013	dihydroorotate dehydrogenase (quinone) [Source:HGNC Symbol;	chr16:7204	NM_00136	XM_01702	.	.	NP_00135	XP_005255	protein_co	ENSG0000	1723	2867
DHPS	38.7247	42.1841	39.3417	40.6261	35.3445	30.6286	deoxyhypusine synthase [Source:HGNC Symbol;Acc:HGNC:2869]	chr19:1275	NM_00120	XM_01152	.	.	NP_00119	XP_011526	protein_co	ENSG0000	1725	2869
DHRS1	12.8899	12.7996	18.8351	12.0945	11.0501	11.9723	dehydrogenase/reductase 1 [Source:HGNC Symbol;Acc:HGNC:16	chr14:2475	NM_00113	.	.	.	NP_00112	.	protein_co	ENSG0000	115817	16445

DHRS11	10.1788	9.03749	8.78045	10.4091	9.69158	7.6911	dehydrogenase/reductase 11 [Source:HGNC Symbol;Acc:HGNC:2	chr17:3494	NM_02430	XM_01152	.	.	NP_07728	XP_005257	protein_co	ENSG0000	79154	28639
DHRS12	1.84245	1.55162	2.87074	1.46165	0.954996	2.0839	dehydrogenase/reductase 12 [Source:HGNC Symbol;Acc:HGNC:2	chr13:5234	NM_00103	XM_01153	.	.	NP_00102	XP_00526	protein_co	ENSG0000	79758	25832
DHRS13	11.7648	12.7846	8.64399	12.5608	10.0975	6.32609	dehydrogenase/reductase 13 [Source:HGNC Symbol;Acc:HGNC:2	chr17:2722	NM_14468	.	.	.	NP_65328	.	protein_co	ENSG0000	147015	28326
DHRS2	19.3948	19.8241	16.5528	25.2965	24.9188	11.2593	dehydrogenase/reductase 2 [Source:HGNC Symbol;Acc:HGNC:18	chr14:2410	NM_00579	XM_01153	.	XR_00175	NP_00578	XP_005267	protein_co	ENSG0000	10202	18349
DHRS3	6.56533	5.98164	22.1312	4.88903	4.12188	4.19677	dehydrogenase/reductase 3 [Source:HGNC Symbol;Acc:HGNC:17	chr1:12627	NM_00475	.	.	.	NP_00474	.	protein_co	ENSG0000	9249	17693
DHRS4	14.858	14.8968	12.9345	16.1203	14.9493	13.3065	dehydrogenase/reductase 4 [Source:HGNC Symbol;Acc:HGNC:16	chr14:2442	NM_00128	XM_00672	.	.	NP_00126	XP_00672	protein_co	ENSG0000	728635	16985
DHRS4-AS1	0.070095	0.075673	0	0	0.077222	0	DHRS4 antisense RNA 1 [Source:HGNC Symbol;Acc:HGNC:23175]	chr14:2440	.	.	NR_02392	.	.	.	antisense	ENSG0000	55449	23175
DHRS4L2	11.7331	11.2719	13.1465	17.4473	14.0385	12.3652	dehydrogenase/reductase 4 like 2 [Source:HGNC Symbol;Acc:HG	chr14:2443	NM_00119	XM_01153	.	.	NP_00118	XP_011534	protein_co	ENSG0000	317749	19731
DHRS7	44.8357	43.0996	39.2916	45.9578	47.4036	46.8731	dehydrogenase/reductase 7 [Source:HGNC Symbol;Acc:HGNC:21	chr14:6055	NM_01602	.	.	.	NP_00130	XP_01687	protein_co	ENSG0000	51635	21524
DHRS7B	13.0519	12.098	12.5035	15.3507	12.1278	12.7335	dehydrogenase/reductase 7B [Source:HGNC Symbol;Acc:HGNC:2	chr17:2102	NM_01551	XM_00525	.	.	NP_05632	XP_00525	protein_co	ENSG0000	25979	24547
DHRS7C	0	0	0	0	0	0	dehydrogenase/reductase 7C [Source:HGNC Symbol;Acc:HGNC:3	chr17:9674	NM_00110	XM_00525	.	.	NP_00109	XP_00525	protein_co	ENSG0000	201140	32423
DHRS9	0.202887	0.217331	0.06259	0.152272	0.399701	0.153246	dehydrogenase/reductase 9 [Source:HGNC Symbol;Acc:HGNC:16	chr2:16992	NM_00114	XM_01151	.	.	NP_00113	XP_011508	protein_co	ENSG0000	10170	16888
DHRSX	1.70917	1.00991	1.21176	1.92143	1.26726	1.58789	dehydrogenase/reductase X-linked [Source:HGNC Symbol;Acc:HG	chrX:21375	NM_14517	.	.	.	NP_66016	.	protein_co	ENSG0000	207063	18399
DHTKD1	6.5284	6.13448	8.47203	6.98066	6.71709	8.2863	dehydrogenase E1 and transketolase domain containing 1 [Sourc	chr10:1211	NM_01870	.	.	.	NP_06117	.	protein_co	ENSG0000	55526	23537
DHX15	86.6322	85.463	88.717	97.938	108.376	101.775	DEAH-box helicase 15 [Source:HGNC Symbol;Acc:HGNC:2738]	chr4:24529	NM_00135	.	.	.	NP_00134	.	protein_co	ENSG0000	1665	2738
DHX16	25.0045	25.6084	24.2094	29.4311	24.9349	21.3332	DEAH-box helicase 16 [Source:HGNC Symbol;Acc:HGNC:2739]	chr6:30614	NM_00116	XM_01151	.	.	NP_00115	XP_011513	protein_co	ENSG0000	8449	2739
DHX29	11.9738	12.4633	10.7039	10.9726	14.0578	10.6764	DEAH-box helicase 29 [Source:HGNC Symbol;Acc:HGNC:15815]	chr5:54552	NM_01903	XM_01154	.	.	NP_06190	XP_006714	protein_co	ENSG0000	54505	15815
DHX30	47.4178	50.2115	44.3794	61.477	54.8741	42.6137	DEAH-box helicase 30 [Source:HGNC Symbol;Acc:HGNC:16716]	chr3:47844	NM_01496	XM_01700	NR_07507	.	NP_05578	XP_006713	protein_co	ENSG0000	22907	16716
DHX32	22.6716	22.2773	23.392	19.3484	21.6817	26.1481	DEAH-box helicase 32 (putative) [Source:HGNC Symbol;Acc:HGN	chr10:1275	NM_01818	XM_01701	.	.	NP_06065	XP_016871	protein_co	ENSG0000	55760	16717
DHX33	18.0747	18.5376	18.7796	18.9096	20.1563	16.7704	DEAH-box helicase 33 [Source:HGNC Symbol;Acc:HGNC:16718]	chr17:5344	NM_00119	.	.	.	NP_00118	.	protein_co	ENSG0000	56919	16718
DHX34	11.2535	12.3686	12.0179	12.1526	10.3857	7.63062	DEAH-box helicase 34 [Source:HGNC Symbol;Acc:HGNC:16719]	chr19:4785	NM_01468	XM_01152	.	.	NP_05549	XP_005259	protein_co	ENSG0000	9704	16719
DHX35	6.43529	7.40268	6.3255	7.03424	6.84075	5.29864	DEAH-box helicase 35 [Source:HGNC Symbol;Acc:HGNC:15861]	chr20:3759	NM_00119	XM_01152	.	.	NP_00117	XP_011527	protein_co	ENSG0000	60625	15861
DHX36	12.2451	12.2878	12.0691	10.1835	15.1529	14.4461	DEAH-box helicase 36 [Source:HGNC Symbol;Acc:HGNC:14410]	chr3:15399	NM_00111	.	.	.	NP_00110	.	protein_co	ENSG0000	170506	14410
DHX37	14.5532	15.305	15.6965	19.0775	17.7918	10.3057	DEAH-box helicase 37 [Source:HGNC Symbol;Acc:HGNC:17210]	chr12:1254	NM_03265	XM_01153	.	XR_001748	NP_11604	XP_005253	protein_co	ENSG0000	57647	17210
DHX38	17.5938	18.6398	15.8241	19.2079	19.3736	13.6444	DEAH-box helicase 38 [Source:HGNC Symbol;Acc:HGNC:17211]	chr16:7211	NM_01400	XM_01152	.	.	NP_05472	XP_00525	protein_co	ENSG0000	9785	17211
DHX40	28.343	26.3993	36.5034	22.9144	26.18	30.8949	DEAH-box helicase 40 [Source:HGNC Symbol;Acc:HGNC:18018]	chr17:5764	NM_00116	XM_01152	.	.	NP_00115	XP_006722	protein_co	ENSG0000	79665	18018
DHX57	8.36529	8.45142	8.00673	8.47093	9.34165	8.24043	DEAH-box helicase 57 [Source:HGNC Symbol;Acc:HGNC:20086]	chr2:39024	NM_19896	XM_01153	.	.	NP_94531	XP_011531	protein_co	ENSG0000	90957	20086
DHX58	1.21366	1.41935	0.213299	1.12211	0.898594	1.08867	DEXH-box helicase 58 [Source:HGNC Symbol;Acc:HGNC:29517]	chr17:4025	NM_02411	XM_01702	.	.	NP_07702	XP_01688	protein_co	ENSG0000	79132	29517
DHX8	14.1441	14.9621	13.8102	17.446	15.403	14.2036	DEAH-box helicase 8 [Source:HGNC Symbol;Acc:HGNC:2749]	chr17:4156	NM_00130	XM_00525	.	.	NP_00128	.	protein_co	ENSG0000	1659	2749
DHX9	112.818	114.057	90.1522	126.7	137.114	103.994	DEAH-box helicase 9 [Source:HGNC Symbol;Acc:HGNC:2750]	chr1:18280	NM_00135	.	.	.	NP_00134	.	protein_co	ENSG0000	1660	2750
DIABLO	44.6535	41.9142	38.166	45.4321	40.329	38.0982	diablo IAP-binding mitochondrial protein [Source:HGNC Symbol;A	chr12:1226	NM_00127	.	.	.	NP_00126	.	protein_co	ENSG0000	56616	21528
DIAPH1	46.3468	46.9846	41.6963	55.6691	52.9427	45.9379	diaphanous related formin 1 [Source:HGNC Symbol;Acc:HGNC:28	chr5:14089	NM_00107	XM_01153	.	.	NP_00107	XP_011535	protein_co	ENSG0000	1729	2876
DIAPH2	2.41396	1.85889	3.33852	2.51792	2.79319	3.59499	diaphanous related formin 2 [Source:HGNC Symbol;Acc:HGNC:28	chrX:95939	NM_00672	.	.	.	NP_00672	.	protein_co	ENSG0000	1730	2877
DIAPH3	7.45242	7.16335	6.41171	6.54978	8.58451	8.52445	diaphanous related formin 3 [Source:HGNC Symbol;Acc:HGNC:15	chr13:6023	NM_00104	XM_01153	.	.	NP_00103	XP_006719	protein_co	ENSG0000	81624	15480
DICER1	11.4537	11.9012	11.4292	9.64013	12.0336	11.3085	dicer 1, ribonuclease III [Source:HGNC Symbol;Acc:HGNC:17098]	chr14:9555	NM_00119	XM_01702	.	.	NP_00118	XP_011534	protein_co	ENSG0000	23405	17098
DIDO1	19.088	21.1667	17.1677	19.9498	19.5972	15.6789	death inducer-obliterator 1 [Source:HGNC Symbol;Acc:HGNC:268	chr20:6150	NM_00119	XM_01152	.	.	NP_00118	XP_011526	protein_co	ENSG0000	11083	2680
DIEXF	7.59965	7.74276	7.66785	6.87767	6.35097	5.75021	digestive organ expansion factor homolog (zebrafish) [Source:HG	chr1:21000	NM_01438	XM_01150	.	.	NP_05520	XP_011507	protein_co	ENSG0000	27042	28440
DIMT1	26.9629	26.6774	26.2315	22.4147	26.0958	18.9011	DIM1 dimethyladenosine transferase 1 homolog [Source:HGNC S	chr5:61683	NM_01447	XM_01154	.	.	NP_05528	XP_011541	protein_co	ENSG0000	27292	30217
DIO1	0.15809	0.048104	0	0	0.05074	0	deiodinase, iodothyronine type I [Source:HGNC Symbol;Acc:HGN	chr1:54359	NM_00079	.	.	.	NP_00078	.	protein_co	ENSG0000	1733	2883
DIO2	0.012953	0.201965	0	0	0.014347	0	deiodinase, iodothyronine, type II [Source:HGNC Symbol;Acc:HG	chr14:8066	NM_00079	.	.	.	NP_00078	.	protein_co	ENSG0000	1734	2884
DIO3	0.512823	0.551874	0.599076	0.453548	0.864622	0.356471	deiodinase, iodothyronine, type III [Source:HGNC Symbol;Acc:HG	chr14:1020	NM_00136	.	.	.	NP_00135	.	protein_co	ENSG0000	1735	2885
DIP2A	7.09498	7.76535	7.84343	6.02007	5.98974	6.37034	disco interacting protein 2 homolog A [Source:HGNC Symbol;Acc:	chr21:4787	NM_00114	XM_01152	.	.	NP_00113	XP_011527	protein_co	ENSG0000	23181	17217
DIP2B	10.8155	11.3671	12.9246	10.5612	11.9574	11.9756	disco interacting protein 2 homolog B [Source:HGNC Symbol;Acc:	chr12:5089	NM_17360	XM_01701	.	.	NP_77587	XP_005269	protein_co	ENSG0000	57609	29284
DIP2C	5.02598	5.14565	4.65946	4.84059	5.04356	4.51596	disco interacting protein 2 homolog C [Source:HGNC Symbol;Acc:	chr10:3201	NM_01497	XM_01151	.	.	NP_05578	XP_005252	protein_co	ENSG0000	22982	29150
DIRAS1	16.6498	16.9192	17.2504	21.3708	20.1367	17.9697	DIRAS family GTPase 1 [Source:HGNC Symbol;Acc:HGNC:19127]	chr19:2714	NM_14517	XM_01152	.	.	NP_66015	XP_011526	protein_co	ENSG0000	148252	19127
DIRAS2	0.019326	0	0	0.086085	0.065352	0.039357	DIRAS family GTPase 2 [Source:HGNC Symbol;Acc:HGNC:19323]	chr9:93372	NM_01759	.	.	.	NP_06006	.	protein_co	ENSG0000	54769	19323
DIRAS3	0.676497	0.993616	1.53288	1.55313	1.37757	2.14084	DIRAS family GTPase 3 [Source:HGNC Symbol;Acc:HGNC:687]	chr1:68511	NM_00467	XM_00671	.	.	NP_00466	XP_016858	protein_co	ENSG0000	9077	687
DIRC1	0	0	0	0	0	0	disrupted in renal carcinoma 1 [Source:HGNC Symbol;Acc:HGNC:	chr2:18959	NM_05295	XM_01151	.	.	NP_44318	XP_011508	protein_co	ENSG0000	116093	15760
DIRC2	5.89227	5.06166	5.28571	4.93241	5.95905	7.60874	disrupted in renal carcinoma 2 [Source:HGNC Symbol;Acc:HGNC:	chr3:12251	NM_03283	.	.	.	NP_11622	.	protein_co	ENSG0000	84925	16628
DIRC3	0	0.369408	0	0	0	0	disrupted in renal carcinoma 3 [Source:HGNC Symbol;Acc:HGNC:	chr2:21856	.	.	.	.	.	.	protein_co	ENSG0000	.	17805
DIS3	22.8334	20.5914	25.0539	20.5381	26.1365	26.4742	DIS3 homolog, exosome endoribonuclease and 3'-5' exoribonucle	chr13:7330	NM_00112	XM_00671	.	.	NP_00112	XP_00526	protein_co	ENSG0000	22894	20604
DIS3L	10.2777	10.4523	9.08251	10.466	10.8233	9.63795	DIS3 like exosome 3'-5' exoribonuclease [Source:HGNC Symbol;A	chr15:6658	NM_00114	XM_00525	.	.	NP_00113	XP_005254	protein_co	ENSG0000	115752	28698
DIS3L2	11.7286	10.2649	9.60118	11.0065	9.35055	8.84461	DIS3 like 3'-5' exoribonuclease 2 [Source:HGNC Symbol;Acc:HGN	chr2:23282	NM_00125	.	.	.	NP_00124	.	protein_co	ENSG0000	129563	28648
DISC1	0.405809	0.330476	0.745615	0.608174	0.636832	0.535964	disrupted in schizophrenia 1 [Source:HGNC Symbol;Acc:HGNC:28	chr1:23176	NM_00101	.	.	.	NP_00101	.	protein_co	ENSG0000	27185	2888
DISP1	2.44488	2.52609	2.44624	1.81688	1.67717	2.32762	dispatched RND transporter family member 1 [Source:HGNC Sym	chr1:22310	NM_03289	XM_01151	.	.	NP_11627	XP_005273	protein_co	ENSG0000	84976	19711
DISP2	0.760932	1.03296	1.03182	0.592544	0.58143	0.554855	dispatched RND transporter family member 2 [Source:HGNC Sym	chr15:4065	NM_03351	XM_01152	.	.	NP_27704	XP_01152	protein_co	ENSG0000	85455	19712
DISP3	0.697198	0.416857	0.569795	0.719148	1.17724	0.887548	dispatched RND transporter family member 3 [Source:HGNC Sym	chr1:11539	NM_02078	XM_01154	.	.	NP_06583	XP_01154	protein_co	ENSG0000	57540	29251
DIXDC1	2.6118	2.79514	3.02937	2.07527	2.58719	2.01852	DIX domain containing 1 [Source:HGNC Symbol;Acc:HGNC:23695	chr11:1117	NM_00103	XM_01701	.	.	NP_00103	XP_016873	protein_co	ENSG0000	85458	23695
DKC1	78.0609	81.1346	64.7272	90.085	88.128	73.3431	dyskerin pseudouridine synthase 1 [Source:HGNC Symbol;Acc:HG	chrX:15399	NM_00114	.	NR_04984	.	NP_00113	.	protein_co	ENSG0000	100847052	2890

DKK1	130.424	126.363	112.724	184.815	171.494	63.1772	dickkopf WNT signaling pathway inhibitor 1 [Source:HGNC Symbo	chr10:5407	NM_01224	.	.	.	NP_036374	.	protein_co	ENSG0000	22943	2891
DKK2	0	0	0	0	0	0	dickkopf WNT signaling pathway inhibitor 2 [Source:HGNC Symbo	chr4:10784	NM_01442	.	.	.	NP_05523	.	protein_co	ENSG0000	27123	2892
DKK3	0.116241	0.11798	0.200704	0.196828	0.133268	0.061319	dickkopf WNT signaling pathway inhibitor 3 [Source:HGNC Symbo	chr11:1198	NM_00101	XM_00671	.	.	NP_001018	XP_006718	protein_co	ENSG0000	27122	2893
DKK4	0	0	0	0.090504	0	0.087748	dickkopf WNT signaling pathway inhibitor 4 [Source:HGNC Symbo	chr8:42231	NM_01442	XM_01154	.	.	NP_05523	XP_011542	protein_co	ENSG0000	27121	2894
DKKL1	0.379262	1.03866	0.684282	0.192299	0.628071	0.73295	dickkopf like acrosomal protein 1 [Source:HGNC Symbol;Acc:HGN	chr19:4983	NM_00119	XM_01152	.	.	NP_00118	XP_006723	protein_co	ENSG0000	27120	16528
DLAT	21.7669	23.4714	23.8204	28.3751	28.0754	24.5464	dihydrolipoamide S-acetyltransferase [Source:HGNC Symbol;Acc:	chr11:1118	NM_00193	XM_01154	.	.	NP_00192	XP_011540	protein_co	ENSG0000	1737	2896
DLC1	0.009292	0.016446	0.032526	0	0	0	DLC1 Rho GTPase activating protein [Source:HGNC Symbol;Acc:H	chr8:12940	NM_00116	XM_01701	.	.	NP_00115	XP_005273	protein_co	ENSG0000	10395	2897
DLD	59.5597	54.938	64.3457	58.2064	61.3075	66.5785	dihydrolipoamide dehydrogenase [Source:HGNC Symbol;Acc:HGN	chr7:10753	NM_00010	.	.	.	NP_00009	.	protein_co	ENSG0000	1738	2898
DLEC1	0	0.024955	0.027613	0.069598	0.024441	0.013209	deleted in lung and esophageal cancer 1 [Source:HGNC Symbol;A	chr3:38080	NM_00733	XM_01153	.	.	NP_00130	XP_006713	protein_co	ENSG0000	9940	2899
DLEU1	18.6173	16.4501	13.4246	20.738	19.1279	15.03	deleted in lymphocytic leukemia 1 [Source:HGNC Symbol;Acc:HG	chr13:5065	.	.	.	.	.	.	protein_co	ENSG0000	.	13747
DLEU7	0	0	0.081091	0	0	0	deleted in lymphocytic leukemia, 7 [Source:HGNC Symbol;Acc:HG	chr13:5128	NM_00130	XM_00526	.	.	NP_00129	XP_005266	protein_co	ENSG0000	220107	17567
DLG1	11.7368	11.2741	14.9227	10.9454	13.6304	16.5885	discs large MAGUK scaffold protein 1 [Source:HGNC Symbol;Acc:	chr3:19676	NM_00109	XM_01700	.	.	NP_00109	XP_005269	protein_co	ENSG0000	1739	2900
DLG2	0	0.012973	0.02151	0	0	0.024581	discs large MAGUK scaffold protein 2 [Source:HGNC Symbol;Acc:	chr11:8316	NM_00114	XM_01701	.	.	NP_00113	XP_005273	protein_co	ENSG0000	1740	2901
DLG3	17.5609	17.4048	16.7398	19.5898	18.6499	22.2856	discs large MAGUK scaffold protein 3 [Source:HGNC Symbol;Acc:	chrX:69664	NM_00116	XM_01153	.	.	NP_00115	XP_005262	protein_co	ENSG0000	1741	2902
DLG4	2.52157	2.70894	3.15633	1.6965	1.57064	1.39175	discs large MAGUK scaffold protein 4 [Source:HGNC Symbol;Acc:	chr17:7093	NM_00112	XM_01702	.	.	NP_00112	XP_005256	protein_co	ENSG0000	1742	2903
DLG5	15.9782	17.9088	17.9684	16.2031	17.1598	13.9566	discs large MAGUK scaffold protein 5 [Source:HGNC Symbol;Acc:	chr10:7955	NM_00474	XM_01701	.	.	NP_00473	XP_005270	protein_co	ENSG0000	9231	2904
DLGAP1	0.030449	0.016068	0.183097	0.138095	0.190381	0.336714	DLG associated protein 1 [Source:HGNC Symbol;Acc:HGNC:2905]	chr18:3496	NM_00100	XM_01702	.	.	NP_00100	XP_005258	protein_co	ENSG0000	9229	2905
DLGAP2	0.016664	0.017101	0.007186	0.018481	0.028288	0.01724	DLG associated protein 2 [Source:HGNC Symbol;Acc:HGNC:2906]	chr8:68759	NM_00127	.	NR_07339	.	NP_00126	.	protein_co	ENSG0000	619343	2906
DLGAP3	0.02633	0.025216	0	0.027679	0	0.030139	DLG associated protein 3 [Source:HGNC Symbol;Acc:HGNC:30368	chr1:35331	NM_00108	XM_01154	.	.	NP_00107	XP_005271	protein_co	ENSG0000	58512	30368
DLGAP4	17.3538	16.3016	17.1981	17.027	16.8172	18.3455	DLG associated protein 4 [Source:HGNC Symbol;Acc:HGNC:24476	chr20:3489	NM_00104	XM_01152	.	.	NP_00103	XP_005260	protein_co	ENSG0000	22839	24476
DLGAP5	21.9659	19.6257	21.7702	35.7027	45.1294	38.7882	DLG associated protein 5 [Source:HGNC Symbol;Acc:HGNC:16864	chr14:5561	NM_00114	XM_01702	.	.	NP_00113	XP_011535	protein_co	ENSG0000	9787	16864
DLK1	0	0	0	0	0	0.086045	delta like non-canonical Notch ligand 1 [Source:HGNC Symbol;Acc	chr14:1011	NM_00383	.	.	.	NP_00130	.	protein_co	ENSG0000	8788	2907
DLK2	0.927131	1.49602	0.952175	0.775186	0.776342	0.516136	delta like non-canonical Notch ligand 2 [Source:HGNC Symbol;Acc	chr6:43395	NM_00128	XM_01151	.	.	NP_00127	XP_005249	protein_co	ENSG0000	65989	21113
DLL1	2.23526	2.73312	2.17611	2.64312	1.93849	1.64885	delta like canonical Notch ligand 1 [Source:HGNC Symbol;Acc:HG	chr6:17059	NM_00561	XM_01153	.	.	NP_00560	XP_005266	protein_co	ENSG0000	28514	2908
DLL3	0.206912	0	0	0.073334	0	0	delta like canonical Notch ligand 3 [Source:HGNC Symbol;Acc:HG	chr19:3998	NM_01694	.	.	.	NP_05863	.	protein_co	ENSG0000	10683	2909
DLL4	0.867297	0.682082	0.983687	0.630839	0.742686	1.56302	delta like canonical Notch ligand 4 [Source:HGNC Symbol;Acc:HG	chr15:4122	NM_01907	.	.	.	NP_06194	.	protein_co	ENSG0000	54567	2910
DLST	56.3627	53.794	55.2936	63.1666	57.7732	53.7534	dihydrolipoamide S-succinyltransferase [Source:HGNC Symbol;Ac	chr14:7534	NM_00193	.	.	.	NP_00192	.	protein_co	ENSG0000	1743	2911
DLX1	1.78091	1.95632	1.6495	1.56886	1.08511	1.44883	distal-less homeobox 1 [Source:HGNC Symbol;Acc:HGNC:2914]	chr2:17294	NM_00103	.	.	.	NP_00103	.	protein_co	ENSG0000	1745	2914
DLX2	1.3925	1.56255	1.97783	1.71871	1.61136	2.25034	distal-less homeobox 2 [Source:HGNC Symbol;Acc:HGNC:2915]	chr2:17296	NM_00440	.	.	.	NP_00439	.	protein_co	ENSG0000	1746	2915
DLX3	0.096857	0.129722	0.027886	0.280386	0.222496	0.173661	distal-less homeobox 3 [Source:HGNC Symbol;Acc:HGNC:2916]	chr17:4806	NM_00522	.	.	.	NP_00521	.	protein_co	ENSG0000	1747	2916
DLX4	6.28846	6.40855	9.66457	6.41398	6.56633	7.29058	distal-less homeobox 4 [Source:HGNC Symbol;Acc:HGNC:2917]	chr17:4804	NM_00193	XM_01152	.	.	NP_00192	XP_011522	protein_co	ENSG0000	1748	2917
DLX5	0	0	0	0	0	0	distal-less homeobox 5 [Source:HGNC Symbol;Acc:HGNC:2918]	chr7:96649	NM_00522	.	.	.	NP_00521	.	protein_co	ENSG0000	1749	2918
DLX6	0	0	0	0	0.04465	0	distal-less homeobox 6 [Source:HGNC Symbol;Acc:HGNC:2919]	chr7:96634	NM_00522	.	.	.	NP_00521	.	protein_co	ENSG0000	1750	2919
DMAP1	11.1922	11.3459	11.4571	15.5211	11.263	10.1432	DNA methyltransferase 1 associated protein 1 [Source:HGNC Sym	chr1:44679	NM_00103	XM_01154	.	.	NP_00102	XP_005271	protein_co	ENSG0000	55929	18291
DMBT1	0.035588	0.03619	0.068303	0.037156	0.064721	0.167041	deleted in malignant brain tumors 1 [Source:HGNC Symbol;Acc:H	chr10:1243	NM_00132	XM_01153	.	.	NP_00130	XP_006717	protein_co	ENSG0000	1755	2926
DMBX1	2.02684	2.68649	2.85507	2.45213	3.70272	2.96651	diencephalon/mesencephalon homeobox 1 [Source:HGNC Symbo	chr1:46972	NM_14719	XM_01700	.	.	NP_67172	XP_011538	protein_co	ENSG0000	127343	19026
DMC1	0.058606	0.197454	0.118294	0.384143	0.163315	0.264193	DNA meiotic recombinase 1 [Source:HGNC Symbol;Acc:HGNC:292	chr22:3891	NM_00127	XM_01152	NR_10347	.	NP_00126	XP_006724	protein_co	ENSG0000	11144	2927
DMD	0	0	0	0.004952	0	0	dystrophin [Source:HGNC Symbol;Acc:HGNC:2928]	chrX:31137	NM_00010	XM_01154	.	.	NP_00010	XP_006724	protein_co	ENSG0000	1756	2928
DMGDH	0.410682	0.570952	0.301278	0.390058	0.265295	0.623416	dimethylglycine dehydrogenase [Source:HGNC Symbol;Acc:HGNC	chr5:78293	NM_01339	XM_01154	.	.	NP_03752	XP_006714	protein_co	ENSG0000	29958	24475
DMKN	163.351	140.476	128.91	170.5	130.935	103.237	dermokine [Source:HGNC Symbol;Acc:HGNC:25063]	chr19:3598	NM_00103	XM_01702	.	.	NP_00103	XP_006723	protein_co	ENSG0000	93099	25063
DMP1	0.018604	0.038462	0.016494	0	0	0	dentin matrix acidic phosphoprotein 1 [Source:HGNC Symbol;Acc	chr4:88571	NM_00107	.	.	.	NP_00107	XP_011530	protein_co	ENSG0000	1758	2932
DMPK	11.8045	12.6083	14.1844	10.2822	9.5451	11.9897	dystrophia myotonica protein kinase [Source:HGNC Symbol;Acc:H	chr19:4627	NM_00108	.	.	.	NP_00107	.	protein_co	ENSG0000	1760	2933
DMRT1	2.37715	2.39837	1.56213	4.21666	4.60323	2.53633	doublesex and mab-3 related transcription factor 1 [Source:HGN	chr9:84168	NM_02195	XM_01151	.	.	NP_06877	XP_006716	protein_co	ENSG0000	1761	2934
DMRT2	0	0	0	0	0.055277	0.046803	doublesex and mab-3 related transcription factor 2 [Source:HGN	chr9:10498	NM_00113	XM_01151	.	.	NP_00112	XP_006716	protein_co	ENSG0000	10655	2935
DMRT3	0.083272	0	0	0.091931	0	0	doublesex and mab-3 related transcription factor 3 [Source:HGN	chr9:97696	NM_02124	.	.	.	NP_06706	.	protein_co	ENSG0000	58524	13909
DMRTA1	0.017409	0	0	0.020045	0.019165	0.016727	DMRT like family A1 [Source:HGNC Symbol;Acc:HGNC:13826]	chr9:22446	NM_02216	.	.	.	NP_07144	.	protein_co	ENSG0000	63951	13826
DMRTA2	0	0	0.044217	0	0	0.057097	DMRT like family A2 [Source:HGNC Symbol;Acc:HGNC:13908]	chr1:50883	NM_03211	XM_01154	.	.	NP_11548	XP_011540	protein_co	ENSG0000	63950	13908
DMRTB1	0	0	0	0	0	0	DMRT like family B with proline rich C-terminal 1 [Source:HGNC S	chr1:53925	NM_03306	.	.	.	NP_14905	.	protein_co	ENSG0000	63948	13913
DMRTC1	0	0	0	0	0	0	DMRT like family C1 [Source:HGNC Symbol;Acc:HGNC:13910]	chrX:72091	NM_03305	XM_00526	.	.	NP_14904	XP_005262	protein_co	ENSG0000	63947	13910
DMRTC1B	0	0	0	0	0	0	DMRT like family C1B [Source:HGNC Symbol;Acc:HGNC:31686]	chrX:71996	NM_00108	XM_01702	.	.	NP_00107	XP_006724	protein_co	ENSG0000	728656	31686
DMRTC2	0	0	0	0	0	0	DMRT like family C2 [Source:HGNC Symbol;Acc:HGNC:13911]	chr19:4234	NM_00104	XM_01702	.	.	NP_00103	XP_005259	protein_co	ENSG0000	63946	13911
DMTF1	12.9389	11.0121	14.44	9.12589	12.8684	18.9366	cyclin D binding myb like transcription factor 1 [Source:HGNC Syn	chr7:86781	NM_00114	XM_01701	.	.	NP_00113	XP_005250	protein_co	ENSG0000	9988	14603
DMTN	18.8024	21.0187	21.1219	20.3025	17.6444	17.7789	dematin actin binding protein [Source:HGNC Symbol;Acc:HGNC:3	chr8:21911	NM_00111	XM_01701	.	.	NP_00110	XP_005273	protein_co	ENSG0000	2039	3382
DMWD	8.41186	8.81743	9.80242	8.41138	7.65613	9.59885	dystrophia myotonica, WD repeat containing [Source:HGNC Symb	chr19:4628	NM_00494	.	.	.	NP_00493	.	protein_co	ENSG0000	1762	2936
DMXL1	3.30779	3.04078	4.01498	2.40679	3.2783	4.49342	Dmx like 1 [Source:HGNC Symbol;Acc:HGNC:2937]	chr5:11840	NM_00129	XM_01700	.	.	NP_00127	XP_005271	protein_co	ENSG0000	1657	2937
DMXL2	3.84887	4.22744	4.87775	2.83424	3.82543	4.446	Dmx like 2 [Source:HGNC Symbol;Acc:HGNC:2938]	chr15:5173	NM_00117	XM_00525	.	.	NP_00116	XP_005254	protein_co	ENSG0000	23312	2938
DNA2	5.16965	4.53302	4.3378	5.32714	6.48956	5.92286	DNA replication helicase/nuclease 2 [Source:HGNC Symbol;Acc:H	chr10:7017	NM_00108	XM_00671	.	.	NP_00107	XP_006717	protein_co	ENSG0000	1763	2939
DNAAF1	0.091956	0.046409	0.020103	0.025073	0	0.04902	dynein axonemal assembly factor 1 [Source:HGNC Symbol;Acc:HG	chr16:8417	NM_17845	XM_01152	.	.	NP_84854	XP_006721	protein_co	ENSG0000	123872	30539
DNAAF2	11.908	12.0472	12.8705	14.1926	16.4902	16.2462	dynein axonemal assembly factor 2 [Source:HGNC Symbol;Acc:HG	chr14:5009	NM_00108	.	.	.	NP_00107	.	protein_co	ENSG0000	55172	20188

DNAAF3	28.9855	27.0314	21.4041	33.3503	27.1888	14.7594	dynein axonemal assembly factor 3 [Source:HGNC Symbol;Acc:H	chr19:5567	NM_00125	.	.	.	NP_00124	.	protein_co	ENSG0000	352909	30492
DNAAF5	31.2229	31.1212	32.1064	39.4192	36.4377	32.3276	dynein axonemal assembly factor 5 [Source:HGNC Symbol;Acc:H	chr7:58883	NM_01780	.	.	.	NP_06027	.	protein_co	ENSG0000	54919	26013
DNAH1	0.553317	0.536059	0.68465	0.399512	0.454238	0.677228	dynein axonemal heavy chain 1 [Source:HGNC Symbol;Acc:HGNC	chr3:52350	NM_01551	XM_01153	.	.	NP_05632	XP_016861	protein_co	ENSG0000	25981	2940
DNAH10	0.308161	0.322104	0.313423	0.316814	0.242399	0.37262	dynein axonemal heavy chain 10 [Source:HGNC Symbol;Acc:HGN	chr12:1242	NM_20743	XM_01153	.	.	NP_99732	XP_005253	protein_co	ENSG0000	196385	2941
DNAH10OS	0.162738	0.220996	0.305817	0.296502	0.154558	0.445687	dynein axonemal heavy chain 10 opposite strand [Source:HGNC S	chr12:1242	.	.	.	.	.	.	protein_co	ENSG0000	.	37121
DNAH11	0.017979	0.009757	0.019733	0.005076	0.005014	0	dynein axonemal heavy chain 11 [Source:HGNC Symbol;Acc:HGN	chr7:21582	NM_00127	.	.	.	NP_00126	.	protein_co	ENSG0000	8701	2942
DNAH12	0.01496	0.016404	0.119358	0	0.008392	0.014452	dynein axonemal heavy chain 12 [Source:HGNC Symbol;Acc:HGN	chr3:57327	NM_00129	XM_01700	.	.	NP_00127	XP_011531	protein_co	ENSG0000	201625	2943
DNAH14	3.28198	3.6127	3.93841	2.59957	4.29784	5.5297	dynein axonemal heavy chain 14 [Source:HGNC Symbol;Acc:HGN	chr1:22508	NM_00114	XM_01154	.	.	NP_00113	XP_006711	protein_co	ENSG0000	127602	2945
DNAH17	0.146385	0.180446	0.116113	0.054491	0.077075	0.130889	dynein axonemal heavy chain 17 [Source:HGNC Symbol;Acc:HGN	chr17:7637	NM_17362	XM_01152	.	.	NP_77589	XP_011523	protein_co	ENSG0000	8632	2946
DNAH2	0.19279	0.136443	0.221501	0.51353	0.396117	0.516127	dynein axonemal heavy chain 2 [Source:HGNC Symbol;Acc:HGNC	chr17:7620	NM_00130	XM_01152	.	.	NP_00129	XP_011521	protein_co	ENSG0000	146754	2948
DNAH3	0.071581	0.054358	0.037388	0.116516	0.124206	0.024641	dynein axonemal heavy chain 3 [Source:HGNC Symbol;Acc:HGNC	chr16:2094	NM_01753	XM_01154	.	.	NP_06000	XP_006721	protein_co	ENSG0000	55567	2949
DNAH5	0.118277	0.098116	0.095587	0.03339	0.023604	0.046328	dynein axonemal heavy chain 5 [Source:HGNC Symbol;Acc:HGNC	chr5:13690	NM_00136	XM_01700	.	.	NP_00136	XP_005248	protein_co	ENSG0000	1767	2950
DNAH6	0.272006	0.234074	0.474793	0.190748	0.165637	0.329289	dynein axonemal heavy chain 6 [Source:HGNC Symbol;Acc:HGNC	chr2:84743	NM_00137	XM_01700	.	.	NP_00136	XP_006712	protein_co	ENSG0000	1768	2951
DNAH7	0	0	0	0	0	0	dynein axonemal heavy chain 7 [Source:HGNC Symbol;Acc:HGNC	chr2:19660	NM_01889	XM_01151	.	.	NP_06172	XP_011509	protein_co	ENSG0000	56171	18661
DNAH8	0	0.005568	0	0	0	0.005032	dynein axonemal heavy chain 8 [Source:HGNC Symbol;Acc:HGNC	chr6:38683	NM_00120	XM_01701	.	.	NP_00119	XP_011512	protein_co	ENSG0000	1769	2952
DNAH9	0	0.040237	0	0	0.022359	0.082668	dynein axonemal heavy chain 9 [Source:HGNC Symbol;Acc:HGNC	chr17:1150	NM_00137	XM_01152	.	.	NP_00136	XP_011522	protein_co	ENSG0000	1770	2953
DNAI1	0.155709	0.250527	0.195172	0.072176	0.126995	0.140964	dynein axonemal intermediate chain 1 [Source:HGNC Symbol;Acc	chr9:34458	NM_00128	XM_01151	.	.	NP_00126	XP_011516	protein_co	ENSG0000	27019	2954
DNAI2	0	0	0	0	0	0	dynein axonemal intermediate chain 2 [Source:HGNC Symbol;Acc	chr17:7227	NM_00117	XM_01152	.	.	NP_00116	XP_011523	protein_co	ENSG0000	64446	18744
DNAJA1	157.907	153.252	113.39	214.097	195.448	115.198	DnaJ heat shock protein family (Hsp40) member A1 [Source:HGN	chr9:33025	NM_00153	.	.	.	NP_00153	.	protein_co	ENSG0000	3301	5229
DNAJA2	36.2623	37.3012	35.6675	43.0885	44.0011	36.351	DnaJ heat shock protein family (Hsp40) member A2 [Source:HGN	chr16:4698	NM_00588	.	.	.	NP_00587	.	protein_co	ENSG0000	10294	14884
DNAJA3	49.645	49.9709	45.4279	53.6033	46.935	47.0789	DnaJ heat shock protein family (Hsp40) member A3 [Source:HGN	chr16:4475	NM_00113	.	.	.	NP_00112	.	protein_co	ENSG0000	9093	11808
DNAJA4	40.0481	38.1209	38.7601	42.0203	52.9829	28.9006	DnaJ heat shock protein family (Hsp40) member A4 [Source:HGN	chr15:7855	NM_00113	XM_00672	.	.	NP_00112	XP_005254	protein_co	ENSG0000	55466	14885
DNAJB1	116.743	112.642	103.865	164.241	151.782	89.0141	DnaJ heat shock protein family (Hsp40) member B1 [Source:HGN	chr19:1462	NM_00130	XM_01152	.	.	NP_00128	XP_006722	protein_co	ENSG0000	3337	5270
DNAJB11	110.689	106.647	51.4858	119.391	103.13	79.0118	DnaJ heat shock protein family (Hsp40) member B11 [Source:HG	chr3:18625	NM_01630	.	.	.	NP_05739	.	protein_co	ENSG0000	51726	14889
DNAJB12	9.14322	10.073	7.58275	9.14377	8.88258	8.49465	DnaJ heat shock protein family (Hsp40) member B12 [Source:HG	chr10:7409	NM_00100	XM_00526	.	.	NP_00100	XP_005269	protein_co	ENSG0000	54788	14891
DNAJB13	0.076277	0.111247	0.034191	0	0	0.037734	DnaJ heat shock protein family (Hsp40) member B13 [Source:HG	chr11:7366	NM_15361	XM_00527	.	.	NP_70584	XP_005274	protein_co	ENSG0000	374407	30718
DNAJB14	10.0951	9.61569	10.0662	11.1968	12.2684	12.4573	DnaJ heat shock protein family (Hsp40) member B14 [Source:HG	chr4:10081	NM_00103	XM_01153	.	.	NP_00102	.	protein_co	ENSG0000	79982	25881
DNAJB2	19.9858	20.9731	18.1065	14.2391	14.2982	11.1852	DnaJ heat shock protein family (Hsp40) member B2 [Source:HGN	chr2:22014	NM_00103	.	.	.	NP_00103	.	protein_co	ENSG0000	3300	5228
DNAJB4	12.9787	12.4077	8.89157	10.1915	12.7715	10.3829	DnaJ heat shock protein family (Hsp40) member B4 [Source:HGN	chr1:78470	NM_00131	.	.	.	NP_00130	.	protein_co	ENSG0000	11080	14886
DNAJB5	3.97985	4.448	4.02326	4.91028	4.13995	3.1177	DnaJ heat shock protein family (Hsp40) member B5 [Source:HGN	chr9:34989	NM_00113	XM_01151	.	.	NP_00112	XP_005251	protein_co	ENSG0000	25822	14887
DNAJB6	49.4104	47.6078	46.0242	60.8176	58.8073	54.6221	DnaJ heat shock protein family (Hsp40) member B6 [Source:HGN	chr7:15712	NM_00549	XM_01151	.	.	NP_00548	XP_005249	protein_co	ENSG0000	10049	14888
DNAJB7	0	0	0	0	0	0	DnaJ heat shock protein family (Hsp40) member B7 [Source:HGN	chr22:4125	NM_14517	.	.	.	NP_66015	.	protein_co	ENSG0000	150353	24986
DNAJB8	0	0	0	0	0	0.034541	DnaJ heat shock protein family (Hsp40) member B8 [Source:HGN	chr3:12818	NM_15333	XM_00671	.	.	NP_69916	XP_006713	protein_co	ENSG0000	165721	23699
DNAJB9	10.8278	9.87342	5.67476	6.38037	7.84544	8.56838	DnaJ heat shock protein family (Hsp40) member B9 [Source:HGN	chr7:10820	NM_01232	.	.	.	NP_03646	.	protein_co	ENSG0000	4189	6968
DNAJC1	8.43306	8.82333	8.14319	9.6216	9.8211	11.5891	DnaJ heat shock protein family (Hsp40) member C1 [Source:HGN	chr10:2204	NM_02236	.	.	.	NP_07176	.	protein_co	ENSG0000	64215	20090
DNAJC10	42.7166	36.9272	47.5716	28.0166	40.3391	33.832	DnaJ heat shock protein family (Hsp40) member C10 [Source:HG	chr2:18358	NM_00127	.	.	.	NP_00125	.	protein_co	ENSG0000	54431	24637
DNAJC11	29.5833	31.4073	28.9722	36.4139	33.3827	24.4665	DnaJ heat shock protein family (Hsp40) member C11 [Source:HG	chr1:66849	NM_01819	.	.	.	NP_06066	.	protein_co	ENSG0000	55735	25570
DNAJC12	4.4418	4.03075	3.89301	2.2567	3.35298	2.57467	DnaJ heat shock protein family (Hsp40) member C12 [Source:HG	chr10:6955	NM_02180	XM_01153	.	.	NP_06857	.	protein_co	ENSG0000	56521	28908
DNAJC13	8.94487	8.44833	9.0332	7.07438	9.96471	10.1299	DnaJ heat shock protein family (Hsp40) member C13 [Source:HG	chr3:13213	NM_00132	XM_00524	.	.	NP_00131	.	protein_co	ENSG0000	23317	30343
DNAJC14	27.5294	27.9605	28.3595	25.5969	25.2809	24.0244	DnaJ heat shock protein family (Hsp40) member C14 [Source:HG	chr12:5613	NM_03236	.	.	.	NP_11574	.	protein_co	ENSG0000	85406	24581
DNAJC15	3.30428	2.97347	2.77872	4.02472	3.6515	3.74141	DnaJ heat shock protein family (Hsp40) member C15 [Source:HG	chr13:4359	NM_01323	.	.	.	NP_03737	.	protein_co	ENSG0000	29103	20325
DNAJC16	9.62262	9.65642	10.131	8.2128	9.60954	8.92115	DnaJ heat shock protein family (Hsp40) member C16 [Source:HG	chr1:15853	NM_00128	.	.	.	NP_00127	.	protein_co	ENSG0000	23341	29157
DNAJC17	13.5003	11.0449	9.53026	13.4059	12.1822	6.16497	DnaJ heat shock protein family (Hsp40) member C17 [Source:HG	chr15:4105	NM_01816	XM_00525	.	.	NP_06063	XP_016877	protein_co	ENSG0000	55192	25556
DNAJC18	1.5502	1.88634	2.20152	1.32953	1.5998	1.78324	DnaJ heat shock protein family (Hsp40) member C18 [Source:HG	chr5:13874	NM_15268	.	.	.	NP_68989	.	protein_co	ENSG0000	202052	28429
DNAJC19	23.8103	21.9302	24.4175	19.973	20.431	20.6839	DnaJ heat shock protein family (Hsp40) member C19 [Source:HG	chr3:18070	NM_00119	.	.	.	NP_00117	.	protein_co	ENSG0000	131118	30528
DNAJC2	24.1055	23.8259	20.4301	24.9297	27.6283	18.9552	DnaJ heat shock protein family (Hsp40) member C2 [Source:HGN	chr7:10293	NM_00112	XM_01151	.	.	NP_00112	XP_005250	protein_co	ENSG0000	27000	13192
DNAJC21	15.6091	13.7131	13.3908	15.7126	14.0437	15.2029	DnaJ heat shock protein family (Hsp40) member C21 [Source:HG	chr5:34929	NM_00101	XM_01151	.	.	NP_00101	XP_005248	protein_co	ENSG0000	134218	27030
DNAJC22	12.6616	12.0786	17.3368	15.8485	15.8574	17.5462	DnaJ heat shock protein family (Hsp40) member C22 [Source:HG	chr12:4974	NM_00130	.	.	.	NP_00129	.	protein_co	ENSG0000	79962	25802
DNAJC24	8.12871	8.3143	8.9789	9.70926	10.1938	8.09589	DnaJ heat shock protein family (Hsp40) member C24 [Source:HG	chr11:3139	NM_18170	.	.	.	NP_85905	.	protein_co	ENSG0000	120526	26979
DNAJC25	6.54157	6.29447	5.72888	6.89465	7.55332	5.80057	DnaJ heat shock protein family (Hsp40) member C25 [Source:HG	chr9:11439	NM_00101	.	.	.	NP_00101	.	protein_co	ENSG0000	548645	34187
DNAJC25-GNG10	4.11982	3.37433	4.32933	4.40922	6.69654	6.51165	DNAJC25-GNG10 readthrough [Source:HGNC Symbol;Acc:HGNC:3	chr9:11439	NM_00412	.	.	.	NP_00411	.	protein_co	ENSG0000	552891	37501
DNAJC27	1.48834	1.51048	1.51654	1.46738	1.21425	1.53784	DnaJ heat shock protein family (Hsp40) member C27 [Source:HG	chr2:25166	NM_00119	XM_01700	.	.	NP_00118	XP_016859	protein_co	ENSG0000	51277	30290
DNAJC28	1.16287	0.711826	1.36647	0.372241	0.938677	1.25131	DnaJ heat shock protein family (Hsp40) member C28 [Source:HG	chr21:3485	NM_00104	XM_00672	.	.	NP_00103	XP_006724	protein_co	ENSG0000	54943	1297
DNAJC3	28.8975	28.1637	13.4201	25.0151	28.408	22.7055	DnaJ heat shock protein family (Hsp40) member C3 [Source:HGN	chr13:9632	NM_00626	XM_01702	.	.	NP_00625	XP_011519	protein_co	ENSG0000	5611	9439
DNAJC30	6.1162	5.15214	5.56506	3.99498	3.51392	3.24035	DnaJ heat shock protein family (Hsp40) member C30 [Source:HG	chr7:73095	NM_03231	.	.	.	NP_11569	.	protein_co	ENSG0000	84277	16410
DNAJC4	16.7926	15.8064	18.2545	19.5151	12.9975	17.0206	DnaJ heat shock protein family (Hsp40) member C4 [Source:HGN	chr11:6399	NM_00130	XM_01154	.	.	NP_00129	XP_011543	protein_co	ENSG0000	3338	5271
DNAJC5	25.0662	25.2567	26.0761	28.134	26.3981	23.1279	DnaJ heat shock protein family (Hsp40) member C5 [Source:HGN	chr20:6252	NM_02521	XM_01152	.	.	NP_07949	XP_011527	protein_co	ENSG0000	80331	16235
DNAJC5B	0.276572	0.347334	0.432647	0.309055	0.061982	0.111264	DnaJ heat shock protein family (Hsp40) member C5 beta [Source:	chr8:66933	NM_03310	XM_01151	.	.	NP_14909	XP_011515	protein_co	ENSG0000	85479	24138
DNAJC5G	0.591701	0.720887	0.807585	0.700288	0.692174	0.698222	DnaJ heat shock protein family (Hsp40) member C5 gamma [Sour	chr2:27498	NM_00130	XM_01700	.	.	NP_00129	XP_006712	protein_co	ENSG0000	285126	24844

DNAJC6	4.83152	4.64935	3.7815	3.99756	4.44593	5.24671	DnaJ heat shock protein family (Hsp40) member C6 [Source:HGN	chr1:6573	NM_00125	XM_01154	.	.	NP_00124	XP_01154	protein_co	ENSG0000	9829	15469
DNAJC7	63.2553	59.9465	50.1157	61.3147	60.4843	50.7395	DnaJ heat shock protein family (Hsp40) member C7 [Source:HGN	chr17:4011	NM_00114	XM_01152	.	.	NP_00113	XP_011523	protein_co	ENSG0000	7266	12392
DNAJC8	83.9245	79.0088	75.4046	102.551	89.7011	82.5897	DnaJ heat shock protein family (Hsp40) member C8 [Source:HGN	chr1:28526	NM_01428	.	.	.	NP_05509	.	protein_co	ENSG0000	22826	15470
DNAJC9	37.8693	33.5352	26.6786	33.6524	32.7319	30.2885	DnaJ heat shock protein family (Hsp40) member C9 [Source:HGN	chr10:7492	NM_01519	.	.	.	NP_05600	.	protein_co	ENSG0000	23234	19123
DNAL1	5.57482	8.31421	6.13674	6.28966	3.97886	6.40091	dynein axonemal light chain 1 [Source:HGNC Symbol;Acc:HGNC:2	chr14:7411	NM_00120	XM_01153	.	.	NP_00118	XP_011535	protein_co	ENSG0000	83544	23247
DNAL4	14.2652	14.5965	12.9654	12.2362	12.0804	13.7337	dynein axonemal light chain 4 [Source:HGNC Symbol;Acc:HGNC:2	chr22:3913	NM_00574	.	.	.	NP_00573	.	protein_co	ENSG0000	10126	2955
DNALI1	0.139283	0.115119	0.556321	0.154168	0.189438	0.577587	dynein axonemal light intermediate chain 1 [Source:HGNC Symbo	chr1:38022	NM_00346	.	.	.	NP_00345	.	protein_co	ENSG0000	7802	14353
DNASE1	1.66979	1.85135	1.93712	1.1657	1.35358	2.14223	deoxyribonuclease 1 [Source:HGNC Symbol;Acc:HGNC:2956]	chr16:3692	NM_00522	XM_01152	.	.	NP_00521	XP_005255	protein_co	ENSG0000	1773	2956
DNASE1L1	4.36781	4.73397	6.18815	4.39738	4.04652	5.83074	deoxyribonuclease 1 like 1 [Source:HGNC Symbol;Acc:HGNC:2957	chrX:15362	NM_00100	XM_01153	.	.	NP_00100	XP_005277	protein_co	ENSG0000	1774	2957
DNASE1L2	0.418611	0.695645	0.529088	0.909752	0.623287	0.231024	deoxyribonuclease 1 like 2 [Source:HGNC Symbol;Acc:HGNC:2958	chr16:2285	NM_00130	XM_01152	.	.	NP_00128	XP_011520	protein_co	ENSG0000	1775	2958
DNASE1L3	0	0	0.043024	0	0	0	deoxyribonuclease 1 like 3 [Source:HGNC Symbol;Acc:HGNC:2959	chr3:58177	NM_00125	.	.	.	NP_00124	.	protein_co	ENSG0000	1776	2959
DNASE2	20.9676	20.8034	16.162	16.8787	14.0949	21.1249	deoxyribonuclease 2, lysosomal [Source:HGNC Symbol;Acc:HGNC	chr19:1298	NM_00137	.	.	.	NP_00136	.	protein_co	ENSG0000	1777	2960
DNASE2B	0.496328	0.224573	0.18535	0	0.369489	0.165805	deoxyribonuclease 2 beta [Source:HGNC Symbol;Acc:HGNC:2887	chr1:84864	NM_02123	XM_01154	.	.	NP_06705	XP_011540	protein_co	ENSG0000	58511	28875
DND1	1.9684	0.865857	0.924801	1.15409	1.51077	1.05623	DND microRNA-mediated repression inhibitor 1 [Source:HGNC Sy	chr5:14004	NM_19424	.	.	.	NP_91922	.	protein_co	ENSG0000	373863	23799
DNER	0.733129	0.448178	0.83813	1.56644	1.10698	1.03222	delta/notch like EGF repeat containing [Source:HGNC Symbol;Acc	chr2:23022	NM_13907	XM_00524	.	.	NP_62071	XP_005247	protein_co	ENSG0000	92737	24456
DNHD1	1.02142	1.14578	1.06782	0.844057	1.00045	1.1908	dynein heavy chain domain 1 [Source:HGNC Symbol;Acc:HGNC:2	chr11:6518	NM_14466	.	.	.	NP_65326	.	protein_co	ENSG0000	144132	26532
DNLZ	20.9827	14.1267	20.4078	19.9253	16.4208	11.5046	DNL-type zinc finger [Source:HGNC Symbol;Acc:HGNC:33879]	chr9:13922	NM_00108	.	.	.	NP_00107	.	protein_co	ENSG0000	728489	33879
DNM1	1.61661	1.54545	2.24524	1.64702	1.30693	1.22227	dynamin 1 [Source:HGNC Symbol;Acc:HGNC:2972]	chr9:13092	NM_00100	XM_01701	.	.	NP_00100	XP_005251	protein_co	ENSG0000	1759	2972
DNM1L	25.1073	23.7306	26.7388	19.9193	26.0159	29.3495	dynamin 1 like [Source:HGNC Symbol;Acc:HGNC:2973]	chr12:3283	NM_00127	XM_01152	.	.	NP_00126	XP_005253	protein_co	ENSG0000	10059	2973
DNM2	31.1087	33.4747	32.1672	38.5783	36.5979	36.6503	dynamin 2 [Source:HGNC Symbol;Acc:HGNC:2974]	chr19:1082	NM_00100	.	.	.	NP_00100	.	protein_co	ENSG0000	1785	2974
DNM3	0.371151	0.56497	0.93517	0.379152	0.610057	0.738479	dynamin 3 [Source:HGNC Symbol;Acc:HGNC:29125]	chr1:17181	NM_00113	XM_01150	.	.	NP_00112	XP_005245	protein_co	ENSG0000	26052	29125
DNMBP	29.7777	30.0976	33.384	30.4409	31.3182	32.4524	dynamin binding protein [Source:HGNC Symbol;Acc:HGNC:30373	chr10:1016	NM_00131	XM_01153	.	.	NP_00130	XP_006717	protein_co	ENSG0000	23268	30373
DNMT1	38.5595	41.1883	35.1267	48.8785	47.9376	39.4532	DNA methyltransferase 1 [Source:HGNC Symbol;Acc:HGNC:2976]	chr19:1024	NM_00113	XM_01152	.	.	NP_00112	.	protein_co	ENSG0000	1786	2976
DNMT3A	3.43726	3.56754	4.00535	1.96676	2.49583	3.00284	DNA methyltransferase 3 alpha [Source:HGNC Symbol;Acc:HGNC	chr2:25450	NM_00132	XM_01700	.	.	NP_00130	XP_005264	protein_co	ENSG0000	1788	2978
DNMT3B	9.39092	8.68582	8.63593	9.87646	11.1841	12.7111	DNA methyltransferase 3 beta [Source:HGNC Symbol;Acc:HGNC:2	chr20:3135	NM_00120	XM_01152	.	.	NP_00119	XP_011526	protein_co	ENSG0000	1789	2979
DNMT3L	0	0	0	0	0	0	DNA methyltransferase 3 like [Source:HGNC Symbol;Acc:HGNC:2	chr21:4566	NM_01336	XM_01152	.	.	NP_03750	XP_011527	protein_co	ENSG0000	29947	2980
DNPEP	36.7929	37.4129	30.7906	38.8257	31.8655	25.3826	aspartyl aminopeptidase [Source:HGNC Symbol;Acc:HGNC:2981]	chr2:22023	NM_00131	XM_01151	.	.	NP_00130	XP_011509	protein_co	ENSG0000	23549	2981
DNPH1	97.2203	92.5976	114.942	73.4342	56.5776	80.3853	2'-deoxynucleoside 5'-phosphate N-hydrolase 1 [Source:HGNC Sy	chr6:43193	NM_00644	.	.	.	NP_00643	.	protein_co	ENSG0000	10591	21218
DNTT	0	0	0	0	0	0	DNA nucleotidylexotransferase [Source:HGNC Symbol;Acc:HGNC:	chr10:9806	NM_00101	.	.	.	NP_00101	.	protein_co	ENSG0000	1791	2983
DNTTIP1	44.022	42.8201	34.534	41.3353	34.8599	32.0716	deoxynucleotidyltransferase terminal interacting protein 1 [Sourc	chr20:4442	NM_05295	.	.	.	NP_44318	.	protein_co	ENSG0000	116092	16160
DNTTIP2	22.2437	19.3168	18.1516	23.4936	26.7953	18.3178	deoxynucleotidyltransferase terminal interacting protein 2 [Sourc	chr1:94331	NM_01459	.	.	.	NP_05541	.	protein_co	ENSG0000	30836	24013
DOC2A	6.00289	6.25841	7.20366	7.59558	5.17782	6.37805	double C2 domain alpha [Source:HGNC Symbol;Acc:HGNC:2985]	chr16:3000	NM_00128	XM_01154	.	.	NP_00126	XP_005255	protein_co	ENSG0000	8448	2985
DOC2B	5.75502	5.24829	6.58327	5.94637	5.49953	6.0366	double C2 domain beta [Source:HGNC Symbol;Acc:HGNC:2986]	chr17:6010	NM_00358	XM_01153	.	.	NP_00357	XP_011533	protein_co	ENSG0000	8447	2986
DOCK1	9.14193	8.90629	12.4022	8.08526	8.3967	10.8275	dedicator of cytokinesis 1 [Source:HGNC Symbol;Acc:HGNC:2987	chr10:1285	NM_00129	XM_01701	.	.	NP_00127	XP_011537	protein_co	ENSG0000	1793	2987
DOCK10	0	0.009359	0.007722	0.040009	0.059309	0.017729	dedicator of cytokinesis 10 [Source:HGNC Symbol;Acc:HGNC:234	chr2:22562	NM_00129	XM_00671	.	.	NP_00127	XP_005246	protein_co	ENSG0000	55619	23479
DOCK11	0.042156	0.136035	0.137548	0.120682	0.174462	0.225811	dedicator of cytokinesis 11 [Source:HGNC Symbol;Acc:HGNC:234	chrX:11762	NM_14465	XM_01153	.	.	NP_65325	XP_005262	protein_co	ENSG0000	139818	23483
DOCK2	0.038907	0	0	0	0	0.013054	dedicator of cytokinesis 2 [Source:HGNC Symbol;Acc:HGNC:2988	chr5:16906	NM_00494	.	.	.	NP_00493	XP_005265	protein_co	ENSG0000	1794	2988
DOCK3	1.59686	1.48938	1.94764	1.19781	1.09199	1.39827	dedicator of cytokinesis 3 [Source:HGNC Symbol;Acc:HGNC:2989	chr3:50712	NM_00494	XM_01700	.	.	NP_00493	XP_005264	protein_co	ENSG0000	1795	2989
DOCK4	0.65454	0.774751	0.87276	0.713565	0.793192	0.909999	dedicator of cytokinesis 4 [Source:HGNC Symbol;Acc:HGNC:1919	chr7:11136	NM_01470	XM_01701	.	.	NP_05552	XP_006716	protein_co	ENSG0000	9732	19192
DOCK5	9.90597	10.5948	12.771	9.53812	11.2253	12.6316	dedicator of cytokinesis 5 [Source:HGNC Symbol;Acc:HGNC:2347	chr8:25042	NM_00132	XM_01701	.	.	NP_00130	XP_011542	protein_co	ENSG0000	80005	23476
DOCK6	7.79123	7.79555	9.50466	8.2508	7.61267	8.4941	dedicator of cytokinesis 6 [Source:HGNC Symbol;Acc:HGNC:1918	chr19:1130	NM_02081	XM_01152	.	.	NP_06586	XP_005260	protein_co	ENSG0000	57572	19189
DOCK7	9.32084	9.5191	10.2958	8.71118	11.0616	11.6678	dedicator of cytokinesis 7 [Source:HGNC Symbol;Acc:HGNC:1919	chr1:62920	NM_00127	XM_01700	.	.	NP_00125	XP_005271	protein_co	ENSG0000	85440	19190
DOCK8	0.046386	0.023108	0.025894	0.02207	0.041069	0.046023	dedicator of cytokinesis 8 [Source:HGNC Symbol;Acc:HGNC:1919	chr9:21282	NM_00119	XM_01701	.	.	NP_00117	XP_011516	protein_co	ENSG0000	81704	19191
DOCK9	6.2523	6.55388	8.4216	5.80015	6.08608	6.54367	dedicator of cytokinesis 9 [Source:HGNC Symbol;Acc:HGNC:1413	chr13:9944	NM_00113	XM_01702	.	.	NP_00112	XP_005254	protein_co	ENSG0000	23348	14132
DOHH	18.2396	19.429	15.5987	24.3769	20.492	14.0531	deoxyhypusine hydroxylase/monooxygenase [Source:HGNC Symb	chr19:3490	NM_00114	XM_01152	.	.	NP_00113	XP_011526	protein_co	ENSG0000	83475	28662
DOK1	9.88698	11.2471	9.47558	14.3163	10.833	9.76816	docking protein 1 [Source:HGNC Symbol;Acc:HGNC:2990]	chr2:74753	NM_00119	XM_01153	.	.	NP_00118	.	protein_co	ENSG0000	1796	2990
DOK2	0.09007	0	0	0.048127	0	0.099631	docking protein 2 [Source:HGNC Symbol;Acc:HGNC:2991]	chr8:21766	NM_00397	.	.	.	NP_00396	.	protein_co	ENSG0000	9046	2991
DOK3	2.9058	1.71823	1.48036	3.16197	3.39957	1.8947	docking protein 3 [Source:HGNC Symbol;Acc:HGNC:24583]	chr5:17692	NM_00114	XM_01153	.	.	NP_00113	XP_005266	protein_co	ENSG0000	79930	24583
DOK4	9.05976	9.45194	10.2099	9.74715	8.30356	7.8743	docking protein 4 [Source:HGNC Symbol;Acc:HGNC:19868]	chr16:5749	NM_01811	XM_01152	.	.	NP_06058	XP_005256	protein_co	ENSG0000	55715	19868
DOK5	0	0	0	0	0	0	docking protein 5 [Source:HGNC Symbol;Acc:HGNC:16173]	chr20:5309	NM_01843	XM_01152	.	.	NP_06090	XP_011527	protein_co	ENSG0000	55816	16173
DOK6	0	0	0	0	0	0	docking protein 6 [Source:HGNC Symbol;Acc:HGNC:28301]	chr18:6706	NM_15272	XM_01152	.	.	NP_68993	XP_011524	protein_co	ENSG0000	220164	28301
DOK7	1.13474	0.909623	2.30722	1.527	1.06371	0.988409	docking protein 7 [Source:HGNC Symbol;Acc:HGNC:26594]	chr4:34650	NM_00116	.	.	.	NP_00115	.	protein_co	ENSG0000	285489	26594
DOLK	14.6684	13.7799	12.8442	15.3429	13.2289	10.5537	dolichol kinase [Source:HGNC Symbol;Acc:HGNC:23406]	chr9:13168	NM_01490	.	.	.	NP_05572	.	protein_co	ENSG0000	22845	23406
DOLPP1	13.5738	17.0422	11.0987	17.3748	14.757	12.4127	dolichyldiphosphatase 1 [Source:HGNC Symbol;Acc:HGNC:29565	chr9:13184	NM_00113	.	.	.	NP_00112	.	protein_co	ENSG0000	57171	29565
DONSON	19.4089	17.1401	17.8782	22.241	23.2059	18.7175	downstream neighbor of SON [Source:HGNC Symbol;Acc:HGNC:2	chr21:3487	NM_01761	.	.	.	NP_06008	.	protein_co	ENSG0000	29980	2993
DOPEY1	1.90902	1.75212	2.0972	1.02932	1.82636	1.60948	dopey family member 1 [Source:HGNC Symbol;Acc:HGNC:21194]	chr6:83777	NM_00119	XM_01153	.	.	NP_00118	XP_011533	protein_co	ENSG0000	23033	21194
DOPEY2	2.75401	3.06964	3.47515	2.58368	2.60832	3.5587	dopey family member 2 [Source:HGNC Symbol;Acc:HGNC:1291]	chr21:3753	NM_00132	XM_01702	.	.	NP_00130	XP_006724	protein_co	ENSG0000	9980	1291
DOT1L	9.08776	10.1873	9.3622	9.76245	9.22682	8.13708	DOT1 like histone lysine methyltransferase [Source:HGNC Symbo	chr19:2164	NM_03248	XM_01152	.	.	NP_11587	XP_005259	protein_co	ENSG0000	84444	24948
DPAGT1	25.7853	24.5324	19.8096	21.5088	21.5747	14.8418	dolichyl-phosphate N-acetylglucosaminephosphotransferase 1 [S	chr11:1189	NM_00138	XM_01154	.	.	NP_00137	XP_005271	protein_co	ENSG0000	1798	2995

DPCD	38.7923	30.2	37.8985	46.8271	32.1531	29.4496	deleted in primary ciliary dyskinesia homolog (mouse) [Source:H(	chr10:1033	NM_01544	.	.	.	NP_05626:	.	protein_co	ENSG0000(	25911	24542
DPCR1	0.04751	0.074285	0.041236	0.052627	0.052855	0.070842	diffuse panbronchiolitis critical region 1 [Source:HGNC Symbol;Ac	chr6:30899	NM_08087	.	.	.	NP_54314(	.	protein_co	ENSG0000(	135656	21666
DPEP1	0.236766	0.231548	0.32578	3.22898	2.99225	3.05051	dipeptidase 1 (renal) [Source:HGNC Symbol;Acc:HGNC:3002]	chr16:8967	NM_00112	XM_01152	.	.	NP_00112:	XP_005256	protein_co	ENSG0000(	1800	3002
DPEP2	0	0	0	0	0.0527	0	dipeptidase 2 [Source:HGNC Symbol;Acc:HGNC:23028]	chr16:6802	NM_02235	XM_01152	.	.	NP_07175(	XP_005256	protein_co	ENSG0000(	64174	23028
DPEP3	0	0	0	0	0	0	dipeptidase 3 [Source:HGNC Symbol;Acc:HGNC:23029]	chr16:6800	NM_00112	.	.	.	NP_00112:	.	protein_co	ENSG0000(	64180	23029
DPF1	4.08488	4.07572	4.28823	3.79011	3.70648	3.42533	double PHD fingers 1 [Source:HGNC Symbol;Acc:HGNC:20225]	chr19:3870	NM_00113	XM_01152	.	.	NP_00112{	XP_005259	protein_co	ENSG0000(	8193	20225
DPF2	37.1301	40.642	32.9839	43.332	36.27	31.7559	double PHD fingers 2 [Source:HGNC Symbol;Acc:HGNC:9964]	chr11:6510	NM_00626	XM_01701	.	.	NP_00625!	XP_005274	protein_co	ENSG0000(	5977	9964
DPF3	0.205237	0.153603	0.487991	0.187345	0.202117	0.130466	double PHD fingers 3 [Source:HGNC Symbol;Acc:HGNC:17427]	chr14:7308	NM_00128	XM_01153	.	.	NP_00126	XP_016877	protein_co	ENSG0000(	8110	17427
DPH1	16.1846	16.9081	19.1233	13.699	12.6486	12.9834	diphthamide biosynthesis 1 [Source:HGNC Symbol;Acc:HGNC:300	chr17:1933	NM_00138	.	.	.	NP_00137	.	protein_co	ENSG0000(	1801	3003
DPH2	36.8345	37.4624	34.3405	41.6505	33.2359	22.7398	DPH2 homolog [Source:HGNC Symbol;Acc:HGNC:3004]	chr1:44435	NM_00103	XM_01154	.	.	NP_00103	XP_016855	protein_co	ENSG0000(	1802	3004
DPH3	9.06851	9.73502	8.6584	10.4604	12.2744	9.82067	diphthamide biosynthesis 3 [Source:HGNC Symbol;Acc:HGNC:277	chr3:16299	NM_00104	.	.	.	NP_00104(	.	protein_co	ENSG0000(	285381	27717
DPH5	35.5883	29.9879	36.5625	39.2452	38.1975	39.0335	diphthamide biosynthesis 5 [Source:HGNC Symbol;Acc:HGNC:242	chr1:10145	NM_00107	XM_01154	.	.	NP_00107(	XP_005270	protein_co	ENSG0000(	51611	24270
DPH6	6.12615	6.30951	5.29369	5.27659	4.91392	6.11291	diphthamine biosynthesis 6 [Source:HGNC Symbol;Acc:HGNC:305	chr15:3566	NM_00114	.	.	.	NP_00113!	XP_011520	protein_co	ENSG0000(	89978	30543
DPH7	21.6633	19.9119	20.5325	22.8907	20.197	14.1875	diphthamide biosynthesis 7 [Source:HGNC Symbol;Acc:HGNC:251	chr9:14044	NM_13877	XM_01151	.	.	NP_62013:	XP_005266	protein_co	ENSG0000(	92715	25199
DPM1	49.6635	47.2392	49.6046	47.5215	54.4214	46.8069	dolichyl-phosphate mannosyltransferase subunit 1, catalytic [Sou	chr20:4955	NM_00131	XM_00526	NR_13364(	.	NP_00130:	XP_005260	protein_co	ENSG0000(	8813	3005
DPM2	37.3528	37.2426	28.3558	31.7942	24.7203	21.2923	dolichyl-phosphate mannosyltransferase subunit 2, regulatory [S(	chr9:13069	NM_00386	.	.	.	NP_00385	.	protein_co	ENSG0000(	8818	3006
DPM3	66.0983	54.6283	66.8575	92.1131	61.8949	54.9863	dolichyl-phosphate mannosyltransferase subunit 3 [Source:HGN(	chr1:15511	NM_01897	XM_01700	.	.	NP_06184(	XP_016856	protein_co	ENSG0000(	54344	3007
DPP10	0	0	0	0	0	0	dipeptidyl peptidase like 10 [Source:HGNC Symbol;Acc:HGNC:208	chr2:11519	NM_00100	XM_01151	.	.	NP_00100	XP_011509	protein_co	ENSG0000(	57628	20823
DPP3	24.1159	25.5195	23.5656	32.1879	25.6191	19.3617	dipeptidyl peptidase 3 [Source:HGNC Symbol;Acc:HGNC:3008]	chr11:6624	NM_00125	.	.	.	NP_00124:	.	protein_co	ENSG0000(	10072	3008
DPP4	1.40115	1.33846	2.2934	6.49781	6.46712	6.86393	dipeptidyl peptidase 4 [Source:HGNC Symbol;Acc:HGNC:3009]	chr2:16284	NM_00193	XM_00524	.	.	NP_00192(	XP_005246	protein_co	ENSG0000(	1803	3009
DPP6	0	0	0	0	0	0	dipeptidyl peptidase like 6 [Source:HGNC Symbol;Acc:HGNC:3010	chr7:15358	NM_00103	XM_01151	.	.	NP_00103	XP_016867	protein_co	ENSG0000(	1804	3010
DPP7	95.8457	94.3743	95.4508	105.146	88.8395	72.0105	dipeptidyl peptidase 7 [Source:HGNC Symbol;Acc:HGNC:14892]	chr9:14000	NM_01337	XM_01151	.	.	NP_03751	XP_005266	protein_co	ENSG0000(	29952	14892
DPP8	7.47861	7.8932	8.56207	7.24709	8.50408	9.28009	dipeptidyl peptidase 8 [Source:HGNC Symbol;Acc:HGNC:16490]	chr15:6573	NM_01774	XM_01702	.	.	NP_00130	XP_005254	protein_co	ENSG0000(	54878	16490
DPP9	20.7684	21.8756	19.0444	22.8408	20.7308	14.0969	dipeptidyl peptidase 9 [Source:HGNC Symbol;Acc:HGNC:18648]	chr19:4675	NM_13915	XM_01152	.	.	NP_63189{	XP_005259	protein_co	ENSG0000(	91039	18648
DPPA2	0.048222	0.050592	0.084487	0	0	0	developmental pluripotency associated 2 [Source:HGNC Symbol;/	chr3:10901	NM_13881	XM_01151	.	.	NP_62017(	XP_011510	protein_co	ENSG0000(	151871	19197
DPPA3	0	0	0	0	0	0	developmental pluripotency associated 3 [Source:HGNC Symbol;/	chr12:7864	NM_19928	.	.	.	NP_95498(	.	protein_co	ENSG0000(	359787	19199
DPPA4	0	0	0	0	0	0	developmental pluripotency associated 4 [Source:HGNC Symbol;/	chr3:10904	NM_01818	.	.	.	NP_06065!	.	protein_co	ENSG0000(	55211	19200
DPPA5	0	0	0	0	0.165275	0	developmental pluripotency associated 5 [Source:HGNC Symbol;/	chr6:74062	NM_00102	.	.	.	NP_00102(	.	protein_co	ENSG0000(	340168	19201
DPRX	0	0	0	0	0	0	divergent-paired related homeobox [Source:HGNC Symbol;Acc:H(	chr19:5413	NM_00101	XM_01152	.	.	NP_00101	XP_011525	protein_co	ENSG0000(	503834	32166
DPT	0	0	0	0	0	0	dermatopontin [Source:HGNC Symbol;Acc:HGNC:3011]	chr1:16866	NM_00193	.	.	.	NP_00192{	.	protein_co	ENSG0000(	1805	3011
DPY19L1	23.7671	25.8796	30.8527	20.4244	26.4731	39.5915	dpy-19 like 1 [Source:HGNC Symbol;Acc:HGNC:22205]	chr7:34968	NM_01528	XM_01151	.	.	NP_05609{	XP_011513	protein_co	ENSG0000(	23333	22205
DPY19L2	0	0	0.020526	0	0	0.022533	dpy-19 like 2 [Source:HGNC Symbol;Acc:HGNC:19414]	chr12:6395	NM_17381	XM_01153	.	.	NP_77617:	XP_006719	protein_co	ENSG0000(	283417	19414
DPY19L3	4.16934	4.19492	5.06223	2.92028	3.23268	3.66678	dpy-19 like 3 (C. elegans) [Source:HGNC Symbol;Acc:HGNC:27120	chr19:3289	NM_00117	XM_01152	.	.	NP_00116(	XP_006723	protein_co	ENSG0000(	147991	27120
DPY19L4	6.63224	6.50571	8.06814	6.03843	9.12639	9.52085	dpy-19 like 4 (C. elegans) [Source:HGNC Symbol;Acc:HGNC:27829	chr8:95732	NM_18178	XM_01151	.	.	NP_86145	XP_005250	protein_co	ENSG0000(	286148	27829
DPY30	49.6844	42.6954	45.7903	50.951	49.6205	49.877	dpy-30, histone methyltransferase complex regulatory subunit [S(	chr2:32248	NM_00132	XM_00671	.	.	NP_00130{	XP_006712	protein_co	ENSG0000(	84661	24590
DPYD	0.268641	0.32423	0.588315	0.345037	0.419355	0.511019	dihydropyrimidine dehydrogenase [Source:HGNC Symbol;Acc:HG	chr1:97543	NM_00011	XM_01700	.	.	NP_00010	XP_005270	protein_co	ENSG0000(	1806	3012
DPYS	0	0	0.029305	0	0	0	dihydropyrimidinase [Source:HGNC Symbol;Acc:HGNC:3013]	chr8:10539	NM_00138	XM_00525	.	.	NP_00137(	XP_005250	protein_co	ENSG0000(	1807	3013
DPYSL2	12.0789	11.7597	12.8572	13.8856	16.2548	15.1135	dihydropyrimidinase like 2 [Source:HGNC Symbol;Acc:HGNC:3014	chr8:26362	NM_00119	.	.	.	NP_00118	.	protein_co	ENSG0000(	1808	3014
DPYSL3	13.1192	13.5347	12.6514	15.8162	14.5631	14.8801	dihydropyrimidinase like 3 [Source:HGNC Symbol;Acc:HGNC:3015	chr5:14677	NM_00119	XM_01153	.	.	NP_00118	XP_011535	protein_co	ENSG0000(	1809	3015
DPYSL4	2.57386	2.34491	2.6075	3.29386	2.60352	2.36165	dihydropyrimidinase like 4 [Source:HGNC Symbol;Acc:HGNC:3016	chr10:1340	NM_00642	XM_01153	.	.	NP_00641	XP_005252	protein_co	ENSG0000(	10570	3016
DPYSL5	3.28832	4.03344	4.61978	4.5122	3.95979	4.35778	dihydropyrimidinase like 5 [Source:HGNC Symbol;Acc:HGNC:2063	chr2:27070	NM_00125	.	.	.	NP_00124(	.	protein_co	ENSG0000(	56896	20637
DQX1	0.133018	0.027153	0.262978	0.261537	0.11473	0.084533	DEAQ-box RNA dependent ATPase 1 [Source:HGNC Symbol;Acc:H	chr2:74745	NM_13363	XM_01153	.	.	NP_59837(	XP_011530	protein_co	ENSG0000(	165545	20410
DR1	42.8065	44.4051	36.4042	45.1683	48.2918	38.5418	down-regulator of transcription 1 [Source:HGNC Symbol;Acc:HGN	chr1:93811	NM_00193	.	.	.	NP_00192!	.	protein_co	ENSG0000(	1810	3017
DRAM1	13.7357	11.3102	14.5555	10.0288	11.0224	11.9836	DNA damage regulated autophagy modulator 1 [Source:HGNC Sy	chr12:1022	NM_01837	XM_00526	.	.	NP_06084(	XP_005269	protein_co	ENSG0000(	55332	25645
DRAM2	9.69861	9.5887	11.2929	9.87392	12.4825	15.0474	DNA damage regulated autophagy modulator 2 [Source:HGNC Sy	chr1:11165	NM_17845	XM_01154	.	.	NP_84854!	XP_005270	protein_co	ENSG0000(	128338	28769
DRAP1	103.415	98.5426	90.5828	119.938	95.7332	103.618	DR1 associated protein 1 [Source:HGNC Symbol;Acc:HGNC:3019]	chr11:6568	NM_00644	.	.	.	NP_00643:	.	protein_co	ENSG0000(	10589	3019
DRAXIN	0.755959	0.772498	1.21443	1.1772	1.14847	1.19258	dorsal inhibitory axon guidance protein [Source:HGNC Symbol;Ac	chr1:11751	NM_19854	.	.	.	NP_94094	XP_016856	protein_co	ENSG0000(	374946	25054
DRC1	0	0.021879	0.037546	0.023527	0	0	dynein regulatory complex subunit 1 [Source:HGNC Symbol;Acc:H	chr2:26624	NM_14503	XM_00526	.	.	NP_65947!	XP_005264	protein_co	ENSG0000(	92749	24245
DRC3	0.415243	0.458355	0.247018	0.277613	0.168377	0.243531	dynein regulatory complex subunit 3 [Source:HGNC Symbol;Acc:H	chr17:1787	NM_00113	XM_01152	.	.	NP_00112:	XP_006721	protein_co	ENSG0000(	83450	25384
DRC7	0.261635	0.112435	0.365157	0.217594	0.219261	0.219422	dynein regulatory complex subunit 7 [Source:HGNC Symbol;Acc:H	chr16:5772	NM_00128	XM_01152	.	.	NP_00127(	XP_011521	protein_co	ENSG0000(	84229	25289
DRD1	0.988086	1.05212	0.890785	1.07961	1.36372	1.65414	dopamine receptor D1 [Source:HGNC Symbol;Acc:HGNC:3020]	chr5:17486	NM_00079	.	.	.	NP_00078!	.	protein_co	ENSG0000(	1812	3020
DRD2	0	0.102715	0	0	0	0	dopamine receptor D2 [Source:HGNC Symbol;Acc:HGNC:3023]	chr11:1132	NM_00079	.	.	.	NP_00078(	XP_016872	protein_co	ENSG0000(	1813	3023
DRD3	0	0	0	0	0	0	dopamine receptor D3 [Source:HGNC Symbol;Acc:HGNC:3024]	chr3:11384	NM_00079	XM_01151	.	.	NP_00078	XP_011510	protein_co	ENSG0000(	1814	3024
DRD4	1.307	1.33412	0.752884	1.36034	0.944168	0.918697	dopamine receptor D4 [Source:HGNC Symbol;Acc:HGNC:3025]	chr11:6372	NM_00079	.	.	.	NP_00078{	.	protein_co	ENSG0000(	1815	3025
DRD5	0	0	0	0	0	0	dopamine receptor D5 [Source:HGNC Symbol;Acc:HGNC:3026]	chr4:97833	NM_00079	.	.	.	NP_00078!	.	protein_co	ENSG0000(	1816	3026
DRG1	48.1266	49.8468	43.3991	62.5434	53.097	44.1681	developmentally regulated GTP binding protein 1 [Source:HGNC !	chr22:3179	NM_00414	.	.	.	NP_00413{	.	protein_co	ENSG0000(	4733	3029
DRG2	19.5656	20.2766	18.0771	21.3889	16.5744	13.92	developmentally regulated GTP binding protein 2 [Source:HGNC !	chr17:1799	NM_00138	XM_01702	.	.	NP_00137!	XP_005256	protein_co	ENSG0000(	1819	3030
DRGX	0	0	0.026168	0	0	0	dorsal root ganglia homeobox [Source:HGNC Symbol;Acc:HGNC:2	chr10:5057	NM_00127	XM_01154	.	.	NP_00126:	XP_011538	protein_co	ENSG0000(	644168	21536
DRICH1	0.325063	0.424367	0.409434	0.456963	0.375836	0.463104	aspartate rich 1 [Source:HGNC Symbol;Acc:HGNC:28031]	chr22:2395	NM_01644	XM_01153	.	.	NP_05753:	XP_011528	protein_co	ENSG0000(	51233	28031

DROSHA	12.9198	13.1236	14.4519	10.7859	11.4395	9.66613	drosha ribonuclease III [Source:HGNC Symbol;Acc:HGNC:17904]	chr5:31400	NM_00110	XM_01151	.	.	NP_00109	XP_005248	protein_co	ENSG0000	29102	17904
DRP2	0.381946	0.255917	0.348593	0.35317	0.327177	0.310884	dystrophin related protein 2 [Source:HGNC Symbol;Acc:HGNC:30	chrX:10047	NM_00117	.	.	.	NP_00116	.	protein_co	ENSG0000	1821	3032
DSC1	0	0	0	0.018712	0.018154	0.064386	desmocollin 1 [Source:HGNC Symbol;Acc:HGNC:3035]	chr18:2870	NM_00494	.	.	.	NP_00493	.	protein_co	ENSG0000	1823	3035
DSC2	8.81811	9.13298	8.92723	11.5528	13.5362	15.3867	desmocollin 2 [Source:HGNC Symbol;Acc:HGNC:3036]	chr18:2863	NM_00494	XM_00525	.	.	NP_00494	XP_005258	protein_co	ENSG0000	1824	3036
DSC3	3.23742	3.2364	2.28722	5.82486	6.45063	5.9442	desmocollin 3 [Source:HGNC Symbol;Acc:HGNC:3037]	chr18:2856	NM_00194	.	.	.	NP_00193	.	protein_co	ENSG0000	1825	3037
DSCAM	0	0	0	0	0	0	DS cell adhesion molecule [Source:HGNC Symbol;Acc:HGNC:3039	chr21:4138	NM_00127	.	.	.	NP_00125	XP_016883	protein_co	ENSG0000	1826	3039
DSCAML1	0	0	0.008673	0.011999	0.013056	0	DS cell adhesion molecule like 1 [Source:HGNC Symbol;Acc:HGNC	chr11:1172	NM_02069	XM_01154	.	.	NP_06574	XP_011541	protein_co	ENSG0000	57453	14656
DSCC1	23.0835	21.9292	23.2499	27.5383	33.8798	28.206	DNA replication and sister chromatid cohesion 1 [Source:HGNC Sy	chr8:12084	NM_02409	XM_00525	.	.	NP_07699	XP_005251	protein_co	ENSG0000	79075	24453
DSCR3	16.2546	17.1537	18.0713	16.9729	16.8754	20.0575	DSCR3 arrestin fold containing [Source:HGNC Symbol;Acc:HGNC:3	chr21:3859	NM_00605	XM_01152	.	.	NP_00604	XP_005260	protein_co	ENSG0000	10311	3044
DSCR4	0	0	0	0	0	0	Down syndrome critical region 4 [Source:HGNC Symbol;Acc:HGNC	chr21:3932	NM_00586	.	.	.	NP_00585	.	protein_co	ENSG0000	10281	3045
DSCR8	0	0	0	0	0	0	Down syndrome critical region 8 [Source:HGNC Symbol;Acc:HGNC	chr21:3949	.	.	NR_02683	.	.	.	protein_co	ENSG0000	84677	16707
DSE	13.5433	14.4643	14.3623	12.1791	11.9672	12.8185	dermatan sulfate epimerase [Source:HGNC Symbol;Acc:HGNC:21	chr6:11660	NM_00108	.	.	.	NP_00107	XP_016866	protein_co	ENSG0000	29940	21144
DSEL	0	0	0	0	0	0	dermatan sulfate epimerase-like [Source:HGNC Symbol;Acc:HGNC	chr18:6517	NM_03216	.	.	.	NP_11553	.	protein_co	ENSG0000	92126	18144
DSG1	0.036708	0	0.032916	0.082341	0.01583	0.036873	desmoglein 1 [Source:HGNC Symbol;Acc:HGNC:3048]	chr18:2889	NM_00194	.	.	.	NP_00193	.	protein_co	ENSG0000	1828	3048
DSG2	50.554	52.9225	55.8822	52.9647	70.6523	68.8052	desmoglein 2 [Source:HGNC Symbol;Acc:HGNC:3049]	chr18:2907	NM_00194	.	.	.	NP_00193	.	protein_co	ENSG0000	1829	3049
DSG3	0.013784	0	0.035908	0.01561	0.015363	0.040986	desmoglein 3 [Source:HGNC Symbol;Acc:HGNC:3050]	chr18:2902	NM_00194	XM_01152	.	.	NP_00193	XP_011524	protein_co	ENSG0000	1830	3050
DSG4	0.055127	0.038755	0.015991	0.07388	0.082309	0.069428	desmoglein 4 [Source:HGNC Symbol;Acc:HGNC:21307]	chr18:2895	NM_00113	.	.	.	NP_00112	.	protein_co	ENSG0000	147409	21307
DSN1	18.7367	17.2448	15.7452	15.5918	17.548	15.769	DSN1 homolog, MIS12 kinetochore complex component [Source:	chr20:3538	NM_00114	XM_00672	.	.	NP_00113	XP_006723	protein_co	ENSG0000	79980	16165
DSP	38.5262	41.5691	41.4635	39.4875	41.3119	40.4407	desmoplakin [Source:HGNC Symbol;Acc:HGNC:3052]	chr6:75418	NM_00100	XM_01151	.	.	NP_00100	.	protein_co	ENSG0000	1832	3052
DSPP	0	0	0.010378	0	0	0	dentin sialophosphoprotein [Source:HGNC Symbol;Acc:HGNC:305	chr4:88529	NM_01420	.	.	.	NP_05502	.	protein_co	ENSG0000	1834	3054
DST	14.404	15.4088	17.7792	9.62217	13.0089	13.5747	dystonin [Source:HGNC Symbol;Acc:HGNC:1090]	chr6:56322	NM_00114	XM_01701	.	.	NP_00113	XP_005249	protein_co	ENSG0000	667	1090
DSTN	115.439	101.581	126.204	109.968	142.464	140.4	destrin, actin depolymerizing factor [Source:HGNC Symbol;Acc:HG	chr20:1755	NM_00101	XM_01152	.	.	NP_00101	XP_011527	protein_co	ENSG0000	11034	15750
DSTYK	3.41454	3.44433	3.89162	3.15085	3.75655	3.65821	dual serine/threonine and tyrosine protein kinase [Source:HGNC	chr1:20511	NM_01537	XM_01150	.	.	NP_05619	XP_011507	protein_co	ENSG0000	25778	29043
DTD1	41.9612	43.3031	40.2069	47.6225	38.8738	45.8535	D-tyrosyl-tRNA deacylase 1 [Source:HGNC Symbol;Acc:HGNC:162	chr20:1856	NM_08082	.	.	.	NP_54301	.	protein_co	ENSG0000	92675	16219
DTD2	8.34641	7.53165	9.05664	8.58169	8.07157	9.4326	D-tyrosyl-tRNA deacylase 2 (putative) [Source:HGNC Symbol;Acc:	chr14:3176	NM_08066	.	.	.	NP_54239	.	protein_co	ENSG0000	112487	20277
DTHD1	0	0	0	0	0	0	death domain containing 1 [Source:HGNC Symbol;Acc:HGNC:372	chr4:36283	NM_00113	XM_01151	.	.	NP_00113	XP_006714	protein_co	ENSG0000	401124	37261
DTL	18.5976	17.7018	14.1771	18.9983	19.9414	17.9024	denticleless E3 ubiquitin protein ligase homolog [Source:HGNC Sy	chr1:21220	NM_00128	XM_01150	.	.	NP_00127	XP_011507	protein_co	ENSG0000	51514	30288
DTNA	2.04478	2.33384	2.26401	2.4867	1.54098	2.5596	dystrobrevin alpha [Source:HGNC Symbol;Acc:HGNC:3057]	chr18:3207	NM_00112	XM_01702	.	.	NP_00112	XP_005258	protein_co	ENSG0000	1837	3057
DTNB	4.34112	4.04231	4.99952	5.36865	5.61843	6.0332	dystrobrevin beta [Source:HGNC Symbol;Acc:HGNC:3058]	chr2:25600	NM_00125	XM_01700	.	.	NP_00124	XP_005264	protein_co	ENSG0000	1838	3058
DTNBP1	10.2726	10.0336	8.73006	11.2933	9.52621	9.07236	dystrobrevin binding protein 1 [Source:HGNC Symbol;Acc:HGNC:1	chr6:15523	NM_00127	XM_01151	.	.	NP_00125	XP_005249	protein_co	ENSG0000	84062	17328
DTWD1	6.95938	6.5794	8.99238	6.53655	12.5099	10.9269	DTW domain containing 1 [Source:HGNC Symbol;Acc:HGNC:3092	chr15:4991	NM_00114	.	.	.	NP_00113	XP_016877	protein_co	ENSG0000	56986	30926
DTWD2	2.77235	2.78158	3.06721	2.839	2.76799	3.25048	DTW domain containing 2 [Source:HGNC Symbol;Acc:HGNC:1933	chr5:11817	NM_00130	XM_01154	.	.	NP_00129	XP_011541	protein_co	ENSG0000	285605	19334
DTX1	0.02474	0.096538	0.063533	0	0.029013	0.082898	deltex E3 ubiquitin ligase 1 [Source:HGNC Symbol;Acc:HGNC:306	chr12:1134	NM_00441	XM_01153	.	.	NP_00440	XP_011536	protein_co	ENSG0000	1840	3060
DTX2	7.5259	8.66606	9.22112	9.11118	8.54333	8.1836	deltex E3 ubiquitin ligase 2 [Source:HGNC Symbol;Acc:HGNC:159	chr7:76091	NM_00110	XM_01701	.	.	NP_00109	XP_005250	protein_co	ENSG0000	113878	15973
DTX3	0	0	0	0	0	0	deltex E3 ubiquitin ligase 3 [Source:HGNC Symbol;Acc:HGNC:244	chr12:5799	NM_00128	XM_01153	.	.	NP_00127	XP_005268	protein_co	ENSG0000	196403	24457
DTX3L	7.76613	8.24243	5.95593	4.80158	5.28768	4.60315	deltex E3 ubiquitin ligase 3L [Source:HGNC Symbol;Acc:HGNC:303	chr3:12224	NM_13828	.	.	.	NP_61214	.	protein_co	ENSG0000	151636	30323
DTX4	3.17469	3.53921	4.16131	2.63886	2.54871	2.47723	deltex E3 ubiquitin ligase 4 [Source:HGNC Symbol;Acc:HGNC:291	chr11:5893	NM_00130	XM_00671	.	.	NP_00128	XP_006718	protein_co	ENSG0000	23220	29151
DTYMK	56.6819	57.9415	46.4649	98.2084	72.8091	49.8464	deoxythymidylate kinase [Source:HGNC Symbol;Acc:HGNC:3061]	chr2:24261	NM_00116	.	.	.	NP_00115	.	protein_co	ENSG0000	1841	3061
DUOX1	3.27254	2.75762	3.96969	2.37953	2.22513	1.80406	dual oxidase 1 [Source:HGNC Symbol;Acc:HGNC:3062]	chr15:4542	NM_01743	XM_01152	.	.	NP_05913	XP_011519	protein_co	ENSG0000	53905	3062
DUOX2	0.188711	0.166029	0.366267	0.153668	0.162288	0.281632	dual oxidase 2 [Source:HGNC Symbol;Acc:HGNC:13273]	chr15:4538	NM_01408	XM_00525	.	.	NP_05479	XP_005254	protein_co	ENSG0000	50506	13273
DUOXA1	0.584865	0.327952	0.839529	0.317857	0.415654	0.505408	dual oxidase maturation factor 1 [Source:HGNC Symbol;Acc:HGNC	chr15:4538	NM_00127	XM_01152	.	.	NP_00126	XP_005254	protein_co	ENSG0000	90527	26507
DUOXA2	0	0	0	0	0	0	dual oxidase maturation factor 2 [Source:HGNC Symbol;Acc:HGNC	chr15:4538	NM_20758	.	.	.	NP_99746	XP_016877	protein_co	ENSG0000	405753	32698
DUPD1	0	0	0	0	0	0	dual specificity phosphatase and pro isomerase domain containin	chr10:7679	NM_00100	XM_01153	.	.	NP_00100	XP_005269	protein_co	ENSG0000	338599	23481
DUS1L	59.9854	59.1145	59.4388	67.5276	54.6177	45.7566	dihydrouridine synthase 1 like [Source:HGNC Symbol;Acc:HGNC:3	chr17:8001	NM_02215	XM_00672	.	.	NP_07143	XP_005256	protein_co	ENSG0000	64118	30086
DUS2	11.629	9.97467	9.17707	12.0901	10.8669	8.8405	dihydrouridine synthase 2 [Source:HGNC Symbol;Acc:HGNC:2601	chr16:6805	NM_00127	.	.	.	NP_00125	.	protein_co	ENSG0000	54920	26014
DUS3L	19.2568	21.8943	15.706	22.0005	19.8264	15.685	dihydrouridine synthase 3 like [Source:HGNC Symbol;Acc:HGNC:2	chr19:5782	NM_00116	.	.	.	NP_00115	XP_016882	protein_co	ENSG0000	56931	26920
DUS4L	4.43867	4.80672	3.42579	3.77411	4.19807	4.26814	dihydrouridine synthase 4 like [Source:HGNC Symbol;Acc:HGNC:2	chr7:10680	NM_00127	XM_01701	.	.	NP_00125	XP_005250	protein_co	ENSG0000	11062	21517
DUSP1	9.68197	11.1934	6.2583	8.64073	7.10793	6.03116	dual specificity phosphatase 1 [Source:HGNC Symbol;Acc:HGNC:3	chr5:17219	NM_00441	.	.	.	NP_00440	.	protein_co	ENSG0000	1843	3064
DUSP10	1.6816	1.31534	1.09744	0.842932	0.916402	1.08996	dual specificity phosphatase 10 [Source:HGNC Symbol;Acc:HGNC	chr1:22187	NM_00720	.	.	.	NP_00913	.	protein_co	ENSG0000	11221	3065
DUSP11	19.7329	19.3542	17.9785	23.3603	17.2518	19.2748	dual specificity phosphatase 11 [Source:HGNC Symbol;Acc:HGNC	chr2:73989	NM_00358	.	.	.	NP_00357	.	protein_co	ENSG0000	8446	3066
DUSP12	20.7485	21.3238	18.5757	27.8656	26.8769	28.7456	dual specificity phosphatase 12 [Source:HGNC Symbol;Acc:HGNC	chr1:16171	NM_00724	XM_00524	.	.	NP_00917	XP_005244	protein_co	ENSG0000	11266	3067
DUSP13	0.331206	0.48906	0.24872	0.33369	0.342702	0.877867	dual specificity phosphatase 13 [Source:HGNC Symbol;Acc:HGNC	chr10:7685	NM_00100	XM_01701	.	.	NP_00100	XP_005269	protein_co	ENSG0000	51207	19681
DUSP14	37.7036	36.2894	35.1315	35.967	33.6153	28.8035	dual specificity phosphatase 14 [Source:HGNC Symbol;Acc:HGNC	chr17:3584	NM_00702	XM_01152	.	.	NP_00895	XP_005257	protein_co	ENSG0000	11072	17007
DUSP15	0.117012	0.114528	0	0	0	0	dual specificity phosphatase 15 [Source:HGNC Symbol;Acc:HGNC	chr20:3043	NM_00101	.	.	.	NP_00101	XP_016883	protein_co	ENSG0000	128853	16236
DUSP16	8.35055	7.72742	7.50498	5.65392	6.34386	9.03775	dual specificity phosphatase 16 [Source:HGNC Symbol;Acc:HGNC	chr12:1262	NM_03064	XM_01152	.	.	NP_08514	XP_005253	protein_co	ENSG0000	80824	17909
DUSP18	3.96616	5.38613	4.3407	3.84033	4.35106	3.90319	dual specificity phosphatase 18 [Source:HGNC Symbol;Acc:HGNC	chr22:3103	NM_00130	XM_01152	.	.	NP_00129	XP_005261	protein_co	ENSG0000	150290	18484
DUSP19	0.598996	0.675418	0.917083	0.940085	0.58562	1.24541	dual specificity phosphatase 19 [Source:HGNC Symbol;Acc:HGNC	chr2:18394	NM_00114	.	.	.	NP_00113	.	protein_co	ENSG0000	142679	18894
DUSP2	12.8263	12.4268	14.491	18.8313	13.9625	8.75109	dual specificity phosphatase 2 [Source:HGNC Symbol;Acc:HGNC:3	chr2:96808	NM_00441	.	.	.	NP_00440	.	protein_co	ENSG0000	1844	3068
DUSP21	0	0	0	0	0	0	dual specificity phosphatase 21 [Source:HGNC Symbol;Acc:HGNC	chrX:44703	NM_02207	.	.	.	NP_07135	.	protein_co	ENSG0000	63904	20476

DUSP22	9.51964	8.40434	10.4453	7.18812	6.42859	8.29564	dual specificity phosphatase 22 [Source:HGNC Symbol;Acc:HGNC	chr6:29209	NM_00128	.	.	.	NP_00127	.	protein_co	ENSG0000	56940	16077
DUSP23	38.3416	37.8369	33.1061	41.163	30.7795	24.298	dual specificity phosphatase 23 [Source:HGNC Symbol;Acc:HGNC	chr1:15975	NM_00131	XM_00524	.	.	NP_00130	XP_005245	protein_co	ENSG0000	54935	21480
DUSP26	0.066127	0.130708	0.19285	0	0	0.097965	dual specificity phosphatase 26 (putative) [Source:HGNC Symbol;	chr8:33448	NM_00130	XM_00527	.	.	NP_00129	XP_005273	protein_co	ENSG0000	78986	28161
DUSP27	0	0	0	0.016542	0	0	dual specificity phosphatase 27 (putative) [Source:HGNC Symbol;	chr1:16706	NM_00108	XM_01151	.	.	NP_00107	XP_005245	protein_co	ENSG0000	92235	25034
DUSP28	2.22655	3.53808	1.55003	1.63462	2.53335	2.53525	dual specificity phosphatase 28 [Source:HGNC Symbol;Acc:HGNC	chr2:24141	NM_00103	.	.	.	NP_00102	.	protein_co	ENSG0000	285193	33237
DUSP3	16.677	17.8816	17.3972	15.0515	15.101	13.0921	dual specificity phosphatase 3 [Source:HGNC Symbol;Acc:HGNC:3	chr17:4184	NM_00409	.	.	.	NP_00408	.	protein_co	ENSG0000	1845	3069
DUSP4	30.1333	32.4892	32.5843	32.448	32.792	33.27	dual specificity phosphatase 4 [Source:HGNC Symbol;Acc:HGNC:3	chr8:29190	NM_00139	XM_01154	.	.	NP_00138	XP_011542	protein_co	ENSG0000	1846	3070
DUSP5	83.5524	79.4477	81.8885	63.2905	62.8867	72.4767	dual specificity phosphatase 5 [Source:HGNC Symbol;Acc:HGNC:3	chr10:1122	NM_00441	.	.	.	NP_00441	.	protein_co	ENSG0000	1847	3071
DUSP6	117.451	107.782	203.807	145.94	137.945	325.776	dual specificity phosphatase 6 [Source:HGNC Symbol;Acc:HGNC:3	chr12:8974	NM_00194	.	.	.	NP_00193	.	protein_co	ENSG0000	1848	3072
DUSP7	15.3469	16.1789	14.6982	20.8904	21.1772	12.5422	dual specificity phosphatase 7 [Source:HGNC Symbol;Acc:HGNC:3	chr3:52082	NM_00194	.	.	.	NP_00193	.	protein_co	ENSG0000	1849	3073
DUSP8	4.86417	5.20467	4.44716	3.77042	3.11653	5.70028	dual specificity phosphatase 8 [Source:HGNC Symbol;Acc:HGNC:3	chr11:1575	NM_00442	XM_01151	.	.	NP_00441	XP_011518	protein_co	ENSG0000	1850	3074
DUSP9	11.7111	11.7441	11.9082	12.9526	13.854	11.5034	dual specificity phosphatase 9 [Source:HGNC Symbol;Acc:HGNC:3	chrX:15290	NM_00131	XM_01153	.	.	NP_00130	XP_005274	protein_co	ENSG0000	1852	3076
DUT	62.246	64.9984	57.3921	84.0052	82.489	71.002	deoxyuridine triphosphatase [Source:HGNC Symbol;Acc:HGNC:30	chr15:4862	NM_00102	XM_01152	.	.	NP_00102	XP_011519	protein_co	ENSG0000	1854	3078
DUXA	0	0	0	0	0	0	double homeobox A [Source:HGNC Symbol;Acc:HGNC:32179]	chr19:5766	NM_00101	.	.	.	NP_00101	.	protein_co	ENSG0000	503835	32179
DVL1	59.3991	58.5947	47.7444	52.7794	48.4148	39.3914	dishevelled segment polarity protein 1 [Source:HGNC Symbol;Acc	chr1:12666	NM_00442	XM_00524	NR_10686	.	NP_00441	XP_005244	protein_co	ENSG0000	102466740	3084
DVL2	23.7517	25.766	25.5651	23.8182	27.0202	23.7707	dishevelled segment polarity protein 2 [Source:HGNC Symbol;Acc	chr17:7128	NM_00442	XM_00672	.	.	NP_00441	XP_005256	protein_co	ENSG0000	1856	3086
DVL3	19.1308	19.1705	18.5176	20.2001	18.8362	15.0927	dishevelled segment polarity protein 3 [Source:HGNC Symbol;Acc	chr3:18387	NM_00442	XM_00524	.	.	NP_00441	XP_005247	protein_co	ENSG0000	1857	3087
DXO	8.75075	9.18858	6.58626	7.55335	6.35679	6.50289	decapping exoribonuclease [Source:HGNC Symbol;Acc:HGNC:299	chr6:31937	NM_00551	XM_00671	.	.	NP_00550	XP_006715	protein_co	ENSG0000	1797	2992
DYDC1	0	0	0	0	0	0	DPY30 domain containing 1 [Source:HGNC Symbol;Acc:HGNC:234	chr10:8209	NM_00126	XM_01701	.	.	NP_00125	XP_005269	protein_co	ENSG0000	143241	23460
DYDC2	0.061417	0	0.053856	0.042988	0.040342	0.063631	DPY30 domain containing 2 [Source:HGNC Symbol;Acc:HGNC:234	chr10:8209	NM_00127	XM_01154	.	.	NP_00125	XP_011538	protein_co	ENSG0000	84332	23468
DYM	10.5053	10.7103	11.8357	11.7775	12.7039	14.3867	dymeclin [Source:HGNC Symbol;Acc:HGNC:21317]	chr18:4656	NM_01765	XM_01152	.	.	NP_06012	XP_006722	protein_co	ENSG0000	54808	21317
DYNAP	0.134964	0.087541	0.034993	0	0.075515	0	dynactin associated protein [Source:HGNC Symbol;Acc:HGNC:268	chr18:5225	NM_00130	XM_01152	.	.	NP_00129	XP_011524	protein_co	ENSG0000	284254	26808
DYNC1H1	43.0134	46.5995	43.3772	43.4211	44.8591	45.8089	dynein cytoplasmic 1 heavy chain 1 [Source:HGNC Symbol;Acc:HG	chr14:1024	NM_00137	.	.	.	NP_00136	.	protein_co	ENSG0000	1778	2961
DYNC1I1	0.056646	0.159903	0.067565	0.102344	0.13032	0.070908	dynein cytoplasmic 1 intermediate chain 1 [Source:HGNC Symbol	chr7:95401	NM_00113	XM_01701	.	.	NP_00112	XP_011514	protein_co	ENSG0000	1780	2963
DYNC1I2	25.6648	25.0598	24.1258	26.7046	25.3796	25.9064	dynein cytoplasmic 1 intermediate chain 2 [Source:HGNC Symbol	chr2:17254	NM_00127	XM_01700	.	.	NP_00125	XP_005246	protein_co	ENSG0000	1781	2964
DYNC1LI1	23.2287	24.9942	21.5309	27.7984	26.1724	24.4926	dynein cytoplasmic 1 light intermediate chain 1 [Source:HGNC Sy	chr3:32567	NM_01614	.	.	.	NP_00131	.	protein_co	ENSG0000	51143	18745
DYNC1LI2	33.5753	33.4088	37.2048	32.2942	36.3251	39.9712	dynein cytoplasmic 1 light intermediate chain 2 [Source:HGNC Sy	chr16:6675	NM_00128	.	.	.	NP_00127	XP_016878	protein_co	ENSG0000	1783	2966
DYNC2H1	0.410349	0.291088	0.521817	0.176211	0.332532	0.312244	dynein cytoplasmic 2 heavy chain 1 [Source:HGNC Symbol;Acc:HG	chr11:1029	NM_00108	XM_01701	.	.	NP_00107	XP_006718	protein_co	ENSG0000	79659	2962
DYNC2LI1	10.5531	10.9247	10.3301	7.18198	9.03086	7.08151	dynein cytoplasmic 2 light intermediate chain 1 [Source:HGNC Sy	chr2:44001	NM_00119	XM_00526	.	.	NP_00118	XP_005264	protein_co	ENSG0000	51626	24595
DYNLL1	286.809	270.935	215.448	399.255	341.449	227.372	dynein light chain LC8-type 1 [Source:HGNC Symbol;Acc:HGNC:15	chr12:1209	NM_00103	.	.	.	NP_00103	.	protein_co	ENSG0000	8655	15476
DYNLL2	58.693	60.1446	47.7301	63.2568	70.2845	53.3903	dynein light chain LC8-type 2 [Source:HGNC Symbol;Acc:HGNC:24	chr17:5616	NM_08067	.	.	.	NP_54240	.	protein_co	ENSG0000	140735	24596
DYNLRB1	85.7673	87.1928	89.6512	112.457	79.2617	89.3213	dynein light chain roadblock-type 1 [Source:HGNC Symbol;Acc:HG	chr20:3310	NM_00128	.	.	.	NP_00126	.	protein_co	ENSG0000	83658	15468
DYNLRB2	0	0	0	0	0	0	dynein light chain roadblock-type 2 [Source:HGNC Symbol;Acc:HG	chr16:8057	NM_00130	.	.	.	NP_00129	XP_006721	protein_co	ENSG0000	83657	15467
DYNLT1	133.07	116.193	159.923	161.47	135.672	150.996	dynein light chain Tctex-type 1 [Source:HGNC Symbol;Acc:HGNC:	chr6:15905	NM_00129	.	.	.	NP_00127	.	protein_co	ENSG0000	6993	11697
DYNLT3	13.5951	14.1315	14.8838	12.9787	15.4409	17.3294	dynein light chain Tctex-type 3 [Source:HGNC Symbol;Acc:HGNC:	chrX:37208	NM_00652	.	.	.	NP_00651	.	protein_co	ENSG0000	6990	11694
DYRK1A	10.6521	11.2766	10.4947	11.0791	11.2056	11.8568	dual specificity tyrosine phosphorylation regulated kinase 1A [So	chr21:3873	NM_00139	XM_01152	.	.	NP_00138	XP_006724	protein_co	ENSG0000	1859	3091
DYRK1B	2.74066	2.8023	1.95816	2.76728	3.29969	2.71583	dual specificity tyrosine phosphorylation regulated kinase 1B [So	chr19:4031	NM_00471	XM_01152	.	.	NP_00470	XP_005259	protein_co	ENSG0000	9149	3092
DYRK2	10.4362	12.5663	12.3462	11.7257	12.7522	10.6	dual specificity tyrosine phosphorylation regulated kinase 2 [Sour	chr12:6804	NM_00358	XM_01702	.	.	NP_00357	XP_016875	protein_co	ENSG0000	8445	3093
DYRK3	4.57487	4.11093	5.05122	5.55704	5.27079	6.00734	dual specificity tyrosine phosphorylation regulated kinase 3 [Sour	chr1:20680	NM_00100	XM_00671	.	.	NP_00100	XP_005273	protein_co	ENSG0000	8444	3094
DYRK4	8.75335	7.71922	7.08114	9.43426	8.15219	7.48681	dual specificity tyrosine phosphorylation regulated kinase 4 [Sour	chr12:4671	NM_00384	.	NR_10411	.	NP_00383	.	protein_co	ENSG0000	8798	3095
DYSF	0.220756	0.266185	0.354019	0.199026	0.372133	0.257077	dysferlin [Source:HGNC Symbol;Acc:HGNC:3097]	chr2:71680	NM_00113	XM_00526	.	.	NP_00112	XP_005264	protein_co	ENSG0000	8291	3097
DYTN	0	0	0	0	0	0	dystrotelin [Source:HGNC Symbol;Acc:HGNC:23279]	chr2:20751	NM_00109	.	.	.	NP_00108	.	protein_co	ENSG0000	391475	23279
DYX1C1	2.89663	2.49021	2.62928	2.54484	2.58927	2.27595	dyslexia susceptibility 1 candidate 1 [Source:HGNC Symbol;Acc:H	chr15:5570	NM_00103	.	.	.	NP_00102	.	protein_co	ENSG0000	161582	21493
DZANK1	0.849359	0.908016	0.885983	0.763271	0.875225	0.852875	double zinc ribbon and ankyrin repeat domains 1 [Source:HGNC S	chr20:1836	NM_00109	XM_01702	.	.	NP_00109	XP_005260	protein_co	ENSG0000	55184	15858
DZIP1	0	0	0	0	0.019682	0	DAZ interacting zinc finger protein 1 [Source:HGNC Symbol;Acc:H	chr13:9608	NM_01493	XM_00671	.	.	NP_05574	XP_005254	protein_co	ENSG0000	22873	20908
DZIP1L	1.71217	1.32817	1.12798	1.46246	1.18106	1.17318	DAZ interacting zinc finger protein 1 like [Source:HGNC Symbol;A	chr3:13778	NM_00117	XM_01151	.	.	NP_00116	XP_005247	protein_co	ENSG0000	199221	26551
DZIP3	5.8258	5.56247	7.47132	5.62013	7.28197	7.67816	DAZ interacting zinc finger protein 3 [Source:HGNC Symbol;Acc:H	chr3:10830	NM_01464	XM_01151	.	.	NP_05546	XP_005247	protein_co	ENSG0000	9666	30938
E2F1	20.0005	20.6804	16.9687	18.1706	17.7157	21.4441	E2F transcription factor 1 [Source:HGNC Symbol;Acc:HGNC:3113]	chr20:3226	NM_00522	.	.	.	NP_00521	.	protein_co	ENSG0000	1869	3113
E2F2	9.77082	10.0937	7.88859	12.1169	11.8053	10.6131	E2F transcription factor 2 [Source:HGNC Symbol;Acc:HGNC:3114]	chr1:23832	NM_00409	XM_01154	.	.	NP_00408	XP_011539	protein_co	ENSG0000	1870	3114
E2F3	22.0532	22.7122	21.4357	23.7234	26.1486	19.9487	E2F transcription factor 3 [Source:HGNC Symbol;Acc:HGNC:3115]	chr6:20402	NM_00124	XM_01151	.	.	NP_00123	XP_005248	protein_co	ENSG0000	1871	3115
E2F4	65.0841	64.3578	62.3935	70.0696	62.7542	47.0184	E2F transcription factor 4 [Source:HGNC Symbol;Acc:HGNC:3118]	chr16:6722	NM_00195	.	.	.	NP_00194	.	protein_co	ENSG0000	1874	3118
E2F5	4.68607	4.52307	5.76006	6.60509	7.55879	7.99825	E2F transcription factor 5 [Source:HGNC Symbol;Acc:HGNC:3119]	chr8:86089	NM_00108	XM_00671	.	.	NP_00107	XP_006716	protein_co	ENSG0000	1875	3119
E2F6	9.45066	9.62517	8.87104	9.37975	10.991	9.47502	E2F transcription factor 6 [Source:HGNC Symbol;Acc:HGNC:3120]	chr2:11584	NM_00127	XM_01151	.	.	NP_00126	.	protein_co	ENSG0000	1876	3120
E2F7	7.97037	7.25827	6.75637	5.79592	6.397	7.05475	E2F transcription factor 7 [Source:HGNC Symbol;Acc:HGNC:23820	chr12:7741	NM_20339	XM_01153	.	.	NP_97632	XP_011536	protein_co	ENSG0000	144455	23820
E2F8	7.61737	7.73105	6.19808	9.08137	10.3435	8.92996	E2F transcription factor 8 [Source:HGNC Symbol;Acc:HGNC:24727	chr11:1924	NM_00125	XM_01152	.	.	NP_00124	XP_011518	protein_co	ENSG0000	79733	24727
E4F1	19.2245	18.8883	17.814	20.1303	17.5946	12.6694	E4F transcription factor 1 [Source:HGNC Symbol;Acc:HGNC:3121]	chr16:2273	NM_00128	XM_01152	.	.	NP_00127	XP_005255	protein_co	ENSG0000	1877	3121
EAF1	16.7367	18.1942	17.9621	18.3863	19.2981	18.4924	ELL associated factor 1 [Source:HGNC Symbol;Acc:HGNC:20907]	chr3:15422	NM_03308	.	.	.	NP_14907	.	protein_co	ENSG0000	85403	20907
EAF2	3.15054	3.3319	2.49486	2.97402	4.00612	3.86528	ELL associated factor 2 [Source:HGNC Symbol;Acc:HGNC:23115]	chr3:12155	NM_00132	.	.	.	NP_00130	XP_016862	protein_co	ENSG0000	55840	23115
EAPP	18.5178	18.0237	15.5553	13.9157	14.8133	16.3794	E2F associated phosphoprotein [Source:HGNC Symbol;Acc:HGNC	chr14:3498	NM_01845	XM_01153	.	.	NP_06092	XP_011535	protein_co	ENSG0000	55837	19312

EARS2	18.4354	18.6325	18.2368	23.4892	22.3515	18.3342	glutamyl-tRNA synthetase 2, mitochondrial [Source:HGNC Symbo	chr16:2353	NM_00108	XM_01154	.	.	NP_00107	XP_006721	protein_co	ENSG0000	124454	29419
EBAG9	22.813	21.7767	18.7964	15.3883	16.756	16.1225	estrogen receptor binding site associated, antigen, 9 [Source:HGN	chr8:11055	NM_00127	.	.	.	NP_00126	XP_016869	protein_co	ENSG0000	9166	3123
EBF1	0	0	0	0	0	0	early B-cell factor 1 [Source:HGNC Symbol;Acc:HGNC:3126]	chr5:15812	NM_00129	XM_01153	.	.	NP_00127	XP_016864	protein_co	ENSG0000	1879	3126
EBF2	1.0333	0.717863	0.97744	1.23236	1.20578	1.52594	early B-cell factor 2 [Source:HGNC Symbol;Acc:HGNC:19090]	chr8:25699	NM_02265	.	.	.	NP_07315	.	protein_co	ENSG0000	64641	19090
EBF3	0.226906	0.199801	0.173941	0.113006	0.136149	0.305605	early B-cell factor 3 [Source:HGNC Symbol;Acc:HGNC:19087]	chr10:1316	NM_00100	XM_00671	.	.	NP_00100	XP_005252	protein_co	ENSG0000	253738	19087
EBF4	2.32597	2.14269	3.65951	1.91604	1.67002	2.69393	early B-cell factor 4 [Source:HGNC Symbol;Acc:HGNC:29278]	chr20:2673	NM_00111	XM_00672	.	.	NP_00110	XP_006723	protein_co	ENSG0000	57593	29278
EBI3	1.84699	2.24824	3.58586	0.995287	0.386675	0.459411	Epstein-Barr virus induced 3 [Source:HGNC Symbol;Acc:HGNC:31	chr19:4229	NM_00575	XM_01152	.	.	NP_00574	XP_011525	protein_co	ENSG0000	10148	3129
EBLN1	0	0	0	0	0	0	endogenous Bornavirus-like nucleoprotein 1 [Source:HGNC Symb	chr10:2249	NM_00119	.	.	.	NP_00118	.	protein_co	ENSG0000	340900	39430
EBLN2	1.15709	1.4319	1.40965	0.788452	0.720379	1.46532	endogenous Bornavirus-like nucleoprotein 2 [Source:HGNC Symb	chr3:73045	NM_01802	.	.	.	NP_06049	.	protein_co	ENSG0000	55096	25493
EBNA1BP2	93.1687	94.0106	73.4741	120.056	96.1068	62.963	EBNA1 binding protein 2 [Source:HGNC Symbol;Acc:HGNC:15531	chr1:43629	NM_00115	.	NR_10679	.	NP_00115	.	protein_co	ENSG0000	102465439	15531
EBP	42.5794	41.5579	33.1095	57.2087	42.8612	36.4392	emopamil binding protein (sterol isomerase) [Source:HGNC Symb	chrX:48379	NM_00657	.	.	.	NP_00657	.	protein_co	ENSG0000	10682	3133
EBPL	42.5161	41.8901	48.4228	46.9492	39.7342	43.4712	emopamil binding protein like [Source:HGNC Symbol;Acc:HGNC:1	chr13:5023	NM_00127	.	NR_10380	.	NP_00126	.	protein_co	ENSG0000	84650	18061
ECD	15.5426	16.5989	14.4439	18.523	16.1458	16.2601	ecdysoneless cell cycle regulator [Source:HGNC Symbol;Acc:HGN	chr10:7489	NM_00113	.	.	.	NP_00112	.	protein_co	ENSG0000	11319	17029
ECE1	27.6578	29.4622	25.7425	20.5587	19.7372	22.4069	endothelin converting enzyme 1 [Source:HGNC Symbol;Acc:HGN	chr1:21543	NM_00111	XM_01700	.	.	NP_00110	XP_006710	protein_co	ENSG0000	1889	3146
ECE2	10.6154	10.6449	9.32732	13.3246	9.82717	8.80328	endothelin converting enzyme 2 [Source:HGNC Symbol;Acc:HGN	chr3:18396	NM_00103	.	.	.	NP_00103	.	protein_co	ENSG0000	9718	13275
ECEL1	0	0	0	0	0	0	endothelin converting enzyme like 1 [Source:HGNC Symbol;Acc:H	chr2:23334	NM_00129	.	.	.	NP_00127	.	protein_co	ENSG0000	9427	3147
ECH1	36.2744	35.4429	39.5781	48.3573	36.59	42.8106	enoyl-CoA hydratase 1 [Source:HGNC Symbol;Acc:HGNC:3149]	chr19:3930	NM_00139	.	.	.	NP_00138	.	protein_co	ENSG0000	1891	3149
ECHDC1	45.8563	47.4592	39.1048	44.2416	49.1911	44.5919	ethylmalonyl-CoA decarboxylase 1 [Source:HGNC Symbol;Acc:HG	chr6:12760	NM_00100	XM_00526	.	.	NP_00100	XP_005267	protein_co	ENSG0000	55862	21489
ECHDC2	7.98129	6.17777	8.95365	6.089	5.3596	6.66796	enoyl-CoA hydratase domain containing 2 [Source:HGNC Symbol;	chr1:53361	NM_00119	XM_01154	.	.	NP_00118	XP_011540	protein_co	ENSG0000	55268	23408
ECHDC3	7.75633	8.32394	12.0652	7.88281	6.21211	7.50775	enoyl-CoA hydratase domain containing 3 [Source:HGNC Symbol;	chr10:1178	NM_02469	.	.	.	NP_07896	.	protein_co	ENSG0000	79746	23489
ECHS1	45.554	45.1989	49.971	53.5984	42.241	36.6782	enoyl-CoA hydratase, short chain 1 [Source:HGNC Symbol;Acc:HG	chr10:1351	NM_00409	.	.	.	NP_00408	.	protein_co	ENSG0000	1892	3151
ECI1	64.7943	59.9108	60.4293	73.3541	59.154	71.6545	enoyl-CoA delta isomerase 1 [Source:HGNC Symbol;Acc:HGNC:27	chr16:2289	NM_00117	.	.	.	NP_00117	.	protein_co	ENSG0000	1632	2703
ECI2	38.1578	41.5049	41.248	50.3996	43.7764	44.8013	enoyl-CoA delta isomerase 2 [Source:HGNC Symbol;Acc:HGNC:14	chr6:40685	NM_00116	XM_00671	.	.	NP_00115	XP_006715	protein_co	ENSG0000	10455	14601
ECM1	2.48721	2.31201	1.64603	4.89057	3.99615	2.71404	extracellular matrix protein 1 [Source:HGNC Symbol;Acc:HGNC:3	chr1:15048	NM_00120	.	.	.	NP_00118	.	protein_co	ENSG0000	1893	3153
ECM2	0.022043	0	0	0.027049	0	0.04333	extracellular matrix protein 2 [Source:HGNC Symbol;Acc:HGNC:3	chr9:95059	NM_00119	.	.	.	NP_00118	.	protein_co	ENSG0000	1842	3154
ECSCR	0	0	0	0	0	0	endothelial cell surface expressed chemotaxis and apoptosis regu	chr5:13883	NM_00107	.	.	.	NP_00107	.	protein_co	ENSG0000	641700	35454
ECSIT	15.1852	15.7556	13.1435	26.0618	18.8428	18.7228	ECSIT signalling integrator [Source:HGNC Symbol;Acc:HGNC:2954	chr19:1159	NM_00114	.	.	.	NP_00113	.	protein_co	ENSG0000	51295	29548
ECT2	27.4965	27.8629	28.2451	41.4243	48.0063	50.2148	epithelial cell transforming 2 [Source:HGNC Symbol;Acc:HGNC:31	chr3:17246	NM_00125	XM_01700	.	.	NP_00124	XP_005247	protein_co	ENSG0000	1894	3155
ECT2L	0	0.018307	0.04477	0	0	0.016849	epithelial cell transforming 2 like [Source:HGNC Symbol;Acc:HGN	chr6:13911	NM_00107	XM_01153	.	.	NP_00107	XP_006715	protein_co	ENSG0000	345930	21118
EDA	1.48583	1.38981	1.68697	2.11481	2.0987	2.35613	ectodysplasin A [Source:HGNC Symbol;Acc:HGNC:3157]	chrX:68835	NM_00100	XM_00672	.	.	NP_00100	XP_006724	protein_co	ENSG0000	1896	3157
EDA2R	0.06973	0.124759	0.263656	0.106417	0.079608	0	ectodysplasin A2 receptor [Source:HGNC Symbol;Acc:HGNC:1775	chrX:65815	NM_00119	XM_01153	.	.	NP_00118	XP_011529	protein_co	ENSG0000	60401	17756
EDAR	0.065536	0.067237	0.35522	0	0.01851	0	ectodysplasin A receptor [Source:HGNC Symbol;Acc:HGNC:2895]	chr2:10951	NM_02233	XM_01151	.	.	NP_07173	XP_006712	protein_co	ENSG0000	10913	2895
EDARADD	25.839	25.6925	24.6838	30.9928	29.4233	27.4892	EDAR associated death domain [Source:HGNC Symbol;Acc:HGNC:	chr1:23651	NM_08073	.	.	.	NP_54277	.	protein_co	ENSG0000	128178	14341
EDC3	16.0108	16.2931	16.3164	17.3596	16.9903	14.1066	enhancer of mRNA decapping 3 [Source:HGNC Symbol;Acc:HGNC	chr15:7492	NM_00114	XM_01152	.	.	NP_00113	XP_011520	protein_co	ENSG0000	80153	26114
EDC4	25.3344	26.391	23.9452	29.0388	26.9997	19.2863	enhancer of mRNA decapping 4 [Source:HGNC Symbol;Acc:HGNC	chr16:6790	NM_01432	.	.	.	NP_05514	.	protein_co	ENSG0000	23644	17157
EDDM3A	0	0	0	0	0	0	epididymal protein 3A [Source:HGNC Symbol;Acc:HGNC:16978]	chr14:2119	NM_00668	.	.	.	NP_00667	XP_016876	protein_co	ENSG0000	10876	16978
EDDM3B	0	0	0	0	0	0	epididymal protein 3B [Source:HGNC Symbol;Acc:HGNC:19223]	chr14:2123	NM_02236	.	.	.	NP_07175	.	protein_co	ENSG0000	64184	19223
EDEM1	8.71389	8.97494	8.00852	5.9965	7.47645	8.75215	ER degradation enhancing alpha-mannosidase like protein 1 [Sou	chr3:52293	NM_01467	XM_01153	.	.	NP_05548	XP_011532	protein_co	ENSG0000	9695	18967
EDEM2	11.5248	12.8187	11.2542	12.3241	8.97336	10.1346	ER degradation enhancing alpha-mannosidase like protein 2 [Sou	chr20:3370	NM_00114	.	.	.	NP_00113	.	protein_co	ENSG0000	55741	15877
EDEM3	6.13591	6.81206	6.26029	4.41018	5.87677	7.04376	ER degradation enhancing alpha-mannosidase like protein 3 [Sou	chr1:18465	NM_00131	XM_01151	.	.	NP_00130	XP_005245	protein_co	ENSG0000	80267	16787
EDF1	269.535	260.999	213.227	292.769	203.23	220.968	endothelial differentiation related factor 1 [Source:HGNC Symbol	chr9:13975	NM_00128	.	.	.	NP_00126	.	protein_co	ENSG0000	8721	3164
EDIL3	0	0	0	0	0	0	EGF like repeats and discoidin domains 3 [Source:HGNC Symbol;A	chr5:83236	NM_00127	.	.	.	NP_00126	.	protein_co	ENSG0000	10085	3173
EDN1	0.390637	0.286048	0.272597	0.219855	0.08745	0	endothelin 1 [Source:HGNC Symbol;Acc:HGNC:3176]	chr6:12290	NM_00116	XM_01151	.	.	NP_00116	XP_011512	protein_co	ENSG0000	1906	3176
EDN2	0.137655	0.067963	0.176988	0.587885	0.159259	0.15183	endothelin 2 [Source:HGNC Symbol;Acc:HGNC:3177]	chr1:41944	NM_00195	.	.	.	NP_00194	.	protein_co	ENSG0000	1907	3177
EDN3	0	0	0	0	0	0	endothelin 3 [Source:HGNC Symbol;Acc:HGNC:3178]	chr20:5787	NM_00130	XM_01152	.	.	NP_00128	XP_005260	protein_co	ENSG0000	1908	3178
EDNRA	1.45123	1.7162	1.87995	1.32789	1.61655	1.9158	endothelin receptor type A [Source:HGNC Symbol;Acc:HGNC:317	chr4:14840	NM_00116	.	.	.	NP_00115	.	protein_co	ENSG0000	1909	3179
EDNRB	0	0	0	0	0	0	endothelin receptor type B [Source:HGNC Symbol;Acc:HGNC:318	chr13:7846	NM_00011	XM_01153	.	.	NP_00010	XP_011533	protein_co	ENSG0000	1910	3180
EDRF1	12.8991	12.8708	13.1829	11.5919	15.3426	11.147	erythroid differentiation regulatory factor 1 [Source:HGNC Symb	chr10:1274	NM_00120	XM_01153	.	.	NP_00118	.	protein_co	ENSG0000	26098	24640
EEA1	2.62319	2.58256	3.13271	2.18598	3.34538	3.10861	early endosome antigen 1 [Source:HGNC Symbol;Acc:HGNC:3185	chr12:9311	NM_00356	XM_01153	.	.	NP_00355	XP_011537	protein_co	ENSG0000	8411	3185
EED	15.4559	14.0856	13.0966	15.1485	19.3249	16.4352	embryonic ectoderm development [Source:HGNC Symbol;Acc:HG	chr11:8595	NM_00130	XM_01154	NR_10681	.	NP_00129	XP_005274	protein_co	ENSG0000	102465452	3188
EEF1A1	4707.55	4493.71	4737.07	4131.52	3639.22	4603.21	eukaryotic translation elongation factor 1 alpha 1 [Source:HGNC	chr6:74225	NM_00140	XM_01153	.	.	NP_00139	XP_011533	protein_co	ENSG0000	1915	3189
EEF1A2	216.054	224.457	226.729	224.352	194.311	189.496	eukaryotic translation elongation factor 1 alpha 2 [Source:HGNC	chr20:6211	NM_00195	.	.	.	NP_00194	.	protein_co	ENSG0000	1917	3192
EEF1AKMT1	9.98453	7.8057	6.35536	11.3242	10.1612	7.25104	eukaryotic translation elongation factor 1 alpha lysine methyltran	chr13:2130	NM_17492	XM_00526	.	.	NP_00130	XP_005266	protein_co	ENSG0000	221143	27351
EEF1B2	934.972	867.067	958.145	962.642	764.347	836.902	eukaryotic translation elongation factor 1 beta 2 [Source:HGNC S	chr2:20697	NM_00103	.	.	.	NP_00103	.	protein_co	ENSG0000	1933	3208
EEF1D	386.486	365.706	329.29	447.083	353.456	361.11	eukaryotic translation elongation factor 1 delta [Source:HGNC Sy	chr8:14466	NM_00113	XM_01151	.	.	NP_00112	XP_005250	protein_co	ENSG0000	1936	3211
EEF1E1	77.3417	76.1475	59.9929	99.7837	77.6881	62.6504	eukaryotic translation elongation factor 1 epsilon 1 [Source:HGN	chr6:80735	NM_00113	.	.	.	NP_00112	.	protein_co	ENSG0000	9521	3212
EEF1G	1126.92	1079.74	1200.39	1213.8	967.928	1042.78	eukaryotic translation elongation factor 1 gamma [Source:HGNC	chr11:6232	NM_00140	.	.	.	NP_00139	.	protein_co	ENSG0000	1937	3213
EEF2	965.843	1021.28	1083.88	992.513	901.509	1054.99	eukaryotic translation elongation factor 2 [Source:HGNC Symbol;	chr19:3976	NM_00196	.	.	.	NP_00195	.	protein_co	ENSG0000	1938	3214
EEF2K	7.65629	7.63793	8.82101	6.80722	7.95128	7.8785	eukaryotic elongation factor 2 kinase [Source:HGNC Symbol;Acc:H	chr16:2221	NM_01330	XM_01702	.	.	NP_03743	XP_005276	protein_co	ENSG0000	101930123	24615

EEF2KMT	15.2031	18.0578	15.7605	19.2778	18.9912	11.6462	eukaryotic elongation factor 2 lysine methyltransferase [Source:H	chr16:5083	NM_00128	XM_00525	.	.	NP_00127	XP_005255	protein_co	ENSG0000	196483	32221
EEFSEC	8.76021	8.60561	9.77949	11.9435	9.56438	9.38019	eukaryotic elongation factor, selenocysteine-tRNA specific [Sourc	chr3:12777	NM_02193	XM_01151	.	.	NP_06875	XP_005247	protein_co	ENSG0000	60678	24614
EEPD1	2.50479	2.38254	2.56457	2.93654	2.46679	2.20069	endonuclease/exonuclease/phosphatase family domain containi	chr7:36192	NM_03063	.	.	.	NP_08513	.	protein_co	ENSG0000	80820	22223
EFCAB1	0	0	0	0	0	0	EF-hand calcium binding domain 1 [Source:HGNC Symbol;Acc:HG	chr8:49627	NM_00114	XM_01151	.	.	NP_00113	XP_005251	protein_co	ENSG0000	79645	25678
EFCAB10	0.836313	1.62577	1.61526	1.40593	0.750301	1.22997	EF-hand calcium binding domain 10 [Source:HGNC Symbol;Acc:H	chr7:10517	.	.	.	.	.	.	protein_co	ENSG0000	.	34531
EFCAB11	13.1499	13.3544	8.33608	12.6989	12.7097	12.1588	EF-hand calcium binding domain 11 [Source:HGNC Symbol;Acc:H	chr14:9026	NM_00128	.	.	.	NP_00127	.	protein_co	ENSG0000	90141	20357
EFCAB12	0.272836	0.213363	0.105673	0.164356	0.315483	0.164935	EF-hand calcium binding domain 12 [Source:HGNC Symbol;Acc:H	chr3:12912	NM_20730	.	.	.	NP_99719	XP_011511	protein_co	ENSG0000	90288	28061
EFCAB13	0.020373	0.022141	0.017881	0	0	0	EF-hand calcium binding domain 13 [Source:HGNC Symbol;Acc:H	chr17:4540	NM_00119	.	.	.	NP_00118	.	protein_co	ENSG0000	124989	26864
EFCAB14	20.5505	20.6183	20.1064	17.5649	19.5146	20.4005	EF-hand calcium binding domain 14 [Source:HGNC Symbol;Acc:H	chr1:47140	NM_01477	.	.	.	NP_05558	.	protein_co	ENSG0000	9813	29051
EFCAB2	3.75669	3.51131	1.70342	1.62414	2.38345	0.710668	EF-hand calcium binding domain 2 [Source:HGNC Symbol;Acc:HG	chr1:24513	NM_00114	XM_01154	.	.	NP_00113	XP_011542	protein_co	ENSG0000	84288	28166
EFCAB3	0.049524	0.212465	0.21578	0.169114	0.109228	0.096748	EF-hand calcium binding domain 3 [Source:HGNC Symbol;Acc:HG	chr17:6044	NM_00114	XM_01152	.	.	NP_00113	XP_011522	protein_co	ENSG0000	146779	26379
EFCAB5	0.069936	0.037405	0	0.039856	0.038812	0.017276	EF-hand calcium binding domain 5 [Source:HGNC Symbol;Acc:HG	chr17:2795	NM_00114	XM_01152	.	.	NP_00113	XP_011523	protein_co	ENSG0000	374786	24801
EFCAB6	0.224934	0.090677	0.183602	0.146059	0.15408	0.143818	EF-hand calcium binding domain 6 [Source:HGNC Symbol;Acc:HG	chr22:4392	NM_02278	XM_01702	.	.	NP_07362	XP_005261	protein_co	ENSG0000	64800	24204
EFCAB7	3.25504	3.88812	2.82592	4.71623	4.86558	3.40714	EF-hand calcium binding domain 7 [Source:HGNC Symbol;Acc:HG	chr1:63989	NM_03243	XM_01154	.	.	NP_11581	XP_005271	protein_co	ENSG0000	84455	29379
EFCAB8	0.047082	0.031995	0.095451	0.034358	0.035877	0.124684	EF-hand calcium binding domain 8 [Source:HGNC Symbol;Acc:HG	chr20:3144	.	XM_01152	.	.	.	XP_011507	protein_co	ENSG0000	388795	34532
EFCAB9	0	0	0	0	0	0.115182	EF-hand calcium binding domain 9 [Source:HGNC Symbol;Acc:HG	chr5:17162	NM_00117	.	.	.	NP_00116	.	protein_co	ENSG0000	285588	34530
EFCC1	1.10793	1.32824	0.982823	1.55089	0.884661	1.59303	EF-hand and coiled-coil domain containing 1 [Source:HGNC Symb	chr3:12872	NM_02476	XM_00524	.	.	NP_07904	XP_005247	protein_co	ENSG0000	79825	25692
EFEMP1	0	0	0	0	0	0	EGF containing fibulin like extracellular matrix protein 1 [Source:H	chr2:56093	NM_00103	.	.	.	NP_00103	.	protein_co	ENSG0000	2202	3218
EFEMP2	0.299727	0.305398	1.08072	0.764708	0.399917	0.521677	EGF containing fibulin like extracellular matrix protein 2 [Source:H	chr11:6563	NM_01693	.	.	.	NP_05863	.	protein_co	ENSG0000	30008	3219
EFHB	0.238589	0.056225	0.092651	0.078061	0.190711	0.084866	EF-hand domain family member B [Source:HGNC Symbol;Acc:HGI	chr3:19920	NM_14471	XM_01700	.	.	NP_65331	XP_005264	protein_co	ENSG0000	151651	26330
EFHC1	4.06526	3.58721	5.07312	2.40805	2.31491	2.28264	EF-hand domain containing 1 [Source:HGNC Symbol;Acc:HGNC:1	chr6:52226	NM_00117	.	.	.	NP_00116	.	protein_co	ENSG0000	114327	16406
EFHC2	0	0	0	0	0	0	EF-hand domain containing 2 [Source:HGNC Symbol;Acc:HGNC:2	chrX:44007	NM_02518	XM_00527	.	.	NP_07946	.	protein_co	ENSG0000	80258	26233
EFHD1	0.41688	0.366547	0.6135	0.433416	0.739517	1.37378	EF-hand domain family member D1 [Source:HGNC Symbol;Acc:H	chr2:23347	NM_00124	XM_01151	.	.	NP_00123	XP_011510	protein_co	ENSG0000	80303	29556
EFHD2	45.2609	48.8628	49.0954	44.5981	43.1384	30.3367	EF-hand domain family member D2 [Source:HGNC Symbol;Acc:H	chr1:15736	NM_02432	.	.	.	NP_07730	.	protein_co	ENSG0000	79180	28670
EFL1	8.48275	7.54205	7.75299	6.45438	6.38282	6.24345	elongation factor like GTPase 1 [Source:HGNC Symbol;Acc:HGNC:	chr15:8242	NM_00104	XM_01152	.	.	NP_00103	XP_011520	protein_co	ENSG0000	79631	25789
EFNA1	14.3952	16.3061	12.1143	12.6494	9.96581	8.32091	ephrin A1 [Source:HGNC Symbol;Acc:HGNC:3221]	chr1:15509	NM_00442	.	.	.	NP_00441	.	protein_co	ENSG0000	1942	3221
EFNA2	8.9016	6.53192	6.40395	6.95783	6.72527	7.18057	ephrin A2 [Source:HGNC Symbol;Acc:HGNC:3222]	chr19:1286	NM_00140	.	.	.	NP_00139	.	protein_co	ENSG0000	1943	3222
EFNA3	16.2281	16.6373	17.9769	21.1128	18.095	12.7744	ephrin A3 [Source:HGNC Symbol;Acc:HGNC:3223]	chr1:15503	NM_00495	.	.	.	NP_00494	.	protein_co	ENSG0000	1944	3223
EFNA4	10.8929	11.2507	9.37526	14.9177	13.8756	8.35308	ephrin A4 [Source:HGNC Symbol;Acc:HGNC:3224]	chr1:15503	NM_00522	.	.	.	NP_00521	.	protein_co	ENSG0000	1945	3224
EFNA5	0.264599	0.156056	0.425292	0.30004	0.328944	0.28351	ephrin A5 [Source:HGNC Symbol;Acc:HGNC:3225]	chr5:10671	NM_00196	XM_01154	.	.	NP_00195	XP_006714	protein_co	ENSG0000	1946	3225
EFNB1	11.5456	13.0139	13.2644	14.4488	12.8092	11.1105	ephrin B1 [Source:HGNC Symbol;Acc:HGNC:3226]	chrX:68048	NM_00442	.	.	.	NP_00442	.	protein_co	ENSG0000	1947	3226
EFNB2	12.5069	12.7299	14.4753	23.9841	27.9297	26.8905	ephrin B2 [Source:HGNC Symbol;Acc:HGNC:3227]	chr13:1071	NM_00409	.	.	.	NP_00408	.	protein_co	ENSG0000	1948	3227
EFNB3	0.085306	0	0.024431	0.123158	0.162673	0.152699	ephrin B3 [Source:HGNC Symbol;Acc:HGNC:3228]	chr17:7608	NM_00140	.	.	.	NP_00139	.	protein_co	ENSG0000	1949	3228
EFR3A	31.2165	31.4558	33.6779	28.4397	33.0471	41.9473	EFR3 homolog A [Source:HGNC Symbol;Acc:HGNC:28970]	chr8:13291	NM_00132	XM_01151	.	.	NP_00131	XP_005250	protein_co	ENSG0000	23167	28970
EFR3B	0.320952	0.383614	0.467532	0.296955	0.388222	0.434187	EFR3 homolog B [Source:HGNC Symbol;Acc:HGNC:29155]	chr2:25264	NM_00131	XM_01153	.	.	NP_00130	XP_011531	protein_co	ENSG0000	22979	29155
EFS	0	0	0	0	0	0	embryonal Fyn-associated substrate [Source:HGNC Symbol;Acc:H	chr14:2382	NM_00127	XM_00526	.	.	NP_00126	XP_005267	protein_co	ENSG0000	10278	16898
EFTUD2	39.7389	42.1706	41.1246	42.3944	36.8535	33.7573	elongation factor Tu GTP binding domain containing 2 [Source:H	chr17:4292	NM_00114	.	.	.	NP_00113	.	protein_co	ENSG0000	9343	30858
EGF	0.275625	0.251124	0.102848	0.285237	0.268958	0.266147	epidermal growth factor [Source:HGNC Symbol;Acc:HGNC:3229]	chr4:11083	NM_00117	XM_01153	.	.	NP_00117	XP_005262	protein_co	ENSG0000	1950	3229
EGFL6	0	0	0	0	0	0	EGF like domain multiple 6 [Source:HGNC Symbol;Acc:HGNC:323	chrX:13587	NM_00116	.	.	.	NP_00116	.	protein_co	ENSG0000	25975	3235
EGFL7	51.6752	55.9685	53.5566	56.9592	42.5337	51.915	EGF like domain multiple 7 [Source:HGNC Symbol;Acc:HGNC:205	chr9:13955	NM_01621	XM_01151	.	.	NP_05729	XP_006717	protein_co	ENSG0000	51162	20594
EGFL8	6.87424	5.28166	7.46781	3.85942	4.76155	3.25864	EGF like domain multiple 8 [Source:HGNC Symbol;Acc:HGNC:139	chr6:32132	NM_03065	.	.	.	NP_08515	.	protein_co	ENSG0000	80864	13944
EGFLAM	0	0	0.045505	0	0	0.12368	EGF like, fibronectin type III and laminin G domains [Source:HGN	chr5:38258	NM_00120	.	.	.	NP_00119	.	protein_co	ENSG0000	133584	26810
EGFR	12.507	13.7311	14.7647	11.289	13.1579	15.2438	epidermal growth factor receptor [Source:HGNC Symbol;Acc:HGN	chr7:55086	NM_00522	.	.	.	NP_00521	.	protein_co	ENSG0000	1956	3236
EGLN1	9.18359	9.14731	9.01998	8.86689	10.007	10.2585	egl-9 family hypoxia inducible factor 1 [Source:HGNC Symbol;Acc	chr1:23149	NM_02205	.	.	.	NP_07133	.	protein_co	ENSG0000	54583	1232
EGLN2	32.2588	32.9673	29.9107	37.3739	33.7154	24.668	egl-9 family hypoxia inducible factor 2 [Source:HGNC Symbol;Acc	chr19:4130	NM_05304	.	.	.	NP_44427	.	protein_co	ENSG0000	112398	14660
EGLN3	3.44084	3.40285	5.23952	3.98464	4.92727	3.98743	egl-9 family hypoxia inducible factor 3 [Source:HGNC Symbol;Acc	chr14:3439	NM_00130	XM_00672	.	.	NP_00129	XP_006720	protein_co	ENSG0000	112399	14661
EGR1	32.7012	34.4462	64.5326	25.6929	28.0631	52.9899	early growth response 1 [Source:HGNC Symbol;Acc:HGNC:3238]	chr5:13780	NM_00196	.	.	.	NP_00195	.	protein_co	ENSG0000	1958	3238
EGR2	1.27644	1.50501	0.979739	0.459167	0.643203	1.0961	early growth response 2 [Source:HGNC Symbol;Acc:HGNC:3239]	chr10:6457	NM_00039	XM_01153	.	.	NP_00039	XP_011537	protein_co	ENSG0000	1959	3239
EGR3	2.18225	1.9969	1.35936	0.86098	1.00372	1.27524	early growth response 3 [Source:HGNC Symbol;Acc:HGNC:3240]	chr8:22545	NM_00119	XM_00527	.	.	NP_00118	XP_005273	protein_co	ENSG0000	1960	3240
EGR4	0.50282	0.539716	0.105917	0.40073	0.666181	0.071544	early growth response 4 [Source:HGNC Symbol;Acc:HGNC:3241]	chr2:73518	NM_00196	.	.	.	NP_00195	.	protein_co	ENSG0000	1961	3241
EHBP1	9.84488	10.7295	18.5165	12.4889	16.4637	20.4399	EH domain binding protein 1 [Source:HGNC Symbol;Acc:HGNC:29	chr2:62900	NM_00114	XM_01700	.	.	NP_00113	XP_005264	protein_co	ENSG0000	23301	29144
EHBP1L1	19.3429	20.5503	25.8353	15.7851	17.4571	18.6417	EH domain binding protein 1 like 1 [Source:HGNC Symbol;Acc:HG	chr11:6534	NM_00109	XM_01154	.	.	NP_00109	XP_005273	protein_co	ENSG0000	254102	30682
EHD1	19.4479	21.0777	19.4065	19.2677	17.8443	16.2347	EH domain containing 1 [Source:HGNC Symbol;Acc:HGNC:3242]	chr11:6461	NM_00128	XM_01154	.	.	NP_00126	XP_011543	protein_co	ENSG0000	10938	3242
EHD2	23.5411	24.8969	21.4203	19.4715	18.2481	18.6755	EH domain containing 2 [Source:HGNC Symbol;Acc:HGNC:3243]	chr19:4821	NM_01460	.	.	.	NP_05541	.	protein_co	ENSG0000	30846	3243
EHD3	0	0	0	0	0	0	EH domain containing 3 [Source:HGNC Symbol;Acc:HGNC:3244]	chr2:31457	NM_01460	.	.	.	NP_05541	.	protein_co	ENSG0000	30845	3244
EHD4	14.5844	15.7218	17.0353	16.8563	14.7716	17.0166	EH domain containing 4 [Source:HGNC Symbol;Acc:HGNC:3245]	chr15:4218	NM_13926	.	.	.	NP_64467	.	protein_co	ENSG0000	30844	3245
EHF	19.7421	20.3324	33.8422	0.126732	0.239956	0.367339	ETS homologous factor [Source:HGNC Symbol;Acc:HGNC:3246]	chr11:3464	NM_00120	XM_01151	.	.	NP_00119	XP_005252	protein_co	ENSG0000	26298	3246
EHHADH	5.05097	4.99559	5.69026	5.77186	5.98121	6.1001	enoyl-CoA, hydratase/3-hydroxyacyl CoA dehydrogenase [Source	chr3:18490	NM_00116	XM_01151	.	.	NP_00115	XP_006713	protein_co	ENSG0000	1962	3247
EHMT1	13.0945	14.3798	13.3738	14.9966	14.6962	12.786	euchromatic histone lysine methyltransferase 1 [Source:HGNC Sy	chr9:14051	NM_00114	XM_01701	.	.	NP_00113	XP_005266	protein_co	ENSG0000	79813	24650

EHMT2	23.7481	25.8468	22.734	27.4138	24.2427	17.8721	euchromatic histone lysine methyltransferase 2 [Source:HGNC Sy	chr6:31847	NM_00128	XM_01701	.	.	.	XP_005248	protein_co	ENSG0000	10919	14129
EI24	145.248	142.208	143.224	141.339	137.736	113.652	EI24, autophagy associated transmembrane protein [Source:HGN	chr11:1254	NM_00129	XM_01154	.	.	NP_00127	XP_01154	protein_co	ENSG0000	9538	13276
EID1	62.2588	61.4844	73.911	77.013	73.3867	58.431	EP300 interacting inhibitor of differentiation 1 [Source:HGNC Sym	chr15:4911	NM_01433	.	.	.	NP_05515	.	protein_co	ENSG0000	23741	1191
EID2	11.685	11.3394	11.2738	14.9405	15.1543	12.6301	EP300 interacting inhibitor of differentiation 2 [Source:HGNC Sym	chr19:4002	NM_15323	.	.	.	NP_69496	.	protein_co	ENSG0000	163126	28292
EID2B	1.58299	1.47453	1.3715	1.54147	1.28736	1.2141	EP300 interacting inhibitor of differentiation 2B [Source:HGNC Sy	chr19:4002	NM_15236	.	.	.	NP_68957	.	protein_co	ENSG0000	126272	26796
EID3	0.331742	0.401365	0.144293	0.558559	0.371679	0.390401	EP300 interacting inhibitor of differentiation 3 [Source:HGNC Sym	chr12:1046	NM_00100	.	.	.	NP_00100	.	protein_co	ENSG0000	493861	32961
EIF1	456.714	445.018	316.868	411.819	353.485	488.644	eukaryotic translation initiation factor 1 [Source:HGNC Symbol;Ac	chr17:3984	NM_00580	.	.	.	NP_00579	.	protein_co	ENSG0000	10209	3249
EIF1AD	25.511	25.6687	20.4182	30.4862	33.8363	18.9544	eukaryotic translation initiation factor 1A domain containing [Sou	chr11:6576	NM_00124	.	.	.	NP_00122	XP_016873	protein_co	ENSG0000	84285	28147
EIF1AX	30.2739	30.5269	27.4893	35.2475	33.4169	34.6694	eukaryotic translation initiation factor 1A, X-linked [Source:HGNC	chrX:20142	NM_00141	.	.	.	NP_00140	XP_016855	protein_co	ENSG0000	107984923	3250
EIF1AY	2.2297	1.66333	1.78352	0	0	0	eukaryotic translation initiation factor 1A, Y-linked [Source:HGNC	chrY:22737	NM_00127	.	.	.	NP_00126	.	protein_co	ENSG0000	9086	3252
EIF1B	39.5317	41.4631	39.465	41.4888	46.0943	38.7299	eukaryotic translation initiation factor 1B [Source:HGNC Symbol;	chr3:40351	NM_00587	.	.	.	NP_00586	.	protein_co	ENSG0000	10289	30792
EIF2A	57.9355	55.2375	63.308	60.2305	63.1516	71.9864	eukaryotic translation initiation factor 2A [Source:HGNC Symbol;	chr3:15025	NM_03202	XM_01151	.	.	NP_00130	XP_01151	protein_co	ENSG0000	83939	3254
EIF2AK1	47.3668	48.1131	46.041	45.9369	45.8164	47.4772	eukaryotic translation initiation factor 2 alpha kinase 1 [Source:H	chr7:60488	NM_00113	.	.	.	NP_00112	.	protein_co	ENSG0000	27102	24921
EIF2AK2	11.8898	12.5411	12.7271	9.71666	13.5943	12.4826	eukaryotic translation initiation factor 2 alpha kinase 2 [Source:H	chr2:37311	NM_00113	XM_01153	.	.	NP_00112	XP_011531	protein_co	ENSG0000	5610	9437
EIF2AK3	6.3771	6.83447	4.47655	4.66944	5.63244	6.50798	eukaryotic translation initiation factor 2 alpha kinase 3 [Source:H	chr2:88856	NM_00131	XM_00526	.	.	NP_00130	XP_016860	protein_co	ENSG0000	9451	3255
EIF2AK4	15.9224	16.2376	16.4931	16.1549	16.4351	14.0403	eukaryotic translation initiation factor 2 alpha kinase 4 [Source:H	chr15:4022	NM_00101	XM_01152	.	.	NP_00101	XP_005254	protein_co	ENSG0000	440275	19687
EIF2B1	25.6536	27.3496	27.7114	33.9609	31.8731	22.5088	eukaryotic translation initiation factor 2B subunit alpha [Source:H	chr12:1240	NM_00141	.	.	.	NP_00140	.	protein_co	ENSG0000	1967	3257
EIF2B2	11.8126	11.8357	10.7572	14.417	11.1908	9.81161	eukaryotic translation initiation factor 2B subunit beta [Source:H	chr14:7546	NM_01423	.	.	.	NP_05505	.	protein_co	ENSG0000	8892	3258
EIF2B3	11.3622	9.00922	8.82149	12.2779	10.3291	8.88818	eukaryotic translation initiation factor 2B subunit gamma [Source	chr1:45316	NM_00116	XM_01154	.	.	NP_00116	.	protein_co	ENSG0000	8891	3259
EIF2B4	33.219	31.1636	28.9217	37.5916	32.0251	22.9699	eukaryotic translation initiation factor 2B subunit delta [Source:H	chr2:27587	NM_00103	XM_01153	.	.	NP_00102	XP_006712	protein_co	ENSG0000	8890	3260
EIF2B5	27.3923	25.9832	27.3854	35.5668	32.971	27.8148	eukaryotic translation initiation factor 2B subunit epsilon [Source	chr3:18385	NM_00390	.	.	.	NP_00389	.	protein_co	ENSG0000	8893	3261
EIF2D	27.7152	27.9194	29.5918	25.98	20.4693	21.605	eukaryotic translation initiation factor 2D [Source:HGNC Symbol;	chr1:20676	NM_00120	XM_01150	.	.	NP_00118	XP_005277	protein_co	ENSG0000	1939	6583
EIF2S1	62.3821	64.0048	56.644	71.2029	68.4147	54.3162	eukaryotic translation initiation factor 2 subunit alpha [Source:HG	chr14:6780	NM_00409	.	.	.	NP_00408	.	protein_co	ENSG0000	1965	3265
EIF2S2	74.4051	66.4546	56.3707	68.9748	67.8926	95.4741	eukaryotic translation initiation factor 2 subunit beta [Source:HG	chr20:3267	NM_00131	XM_00526	.	.	NP_00130	XP_016883	protein_co	ENSG0000	8894	3266
EIF2S3	112.14	113.925	121.189	97.0669	108.53	123.161	eukaryotic translation initiation factor 2 subunit gamma [Source:	chrX:24072	NM_00141	.	.	.	NP_00140	.	protein_co	ENSG0000	1968	3267
EIF2S3L	26.9334	26.305	29.2883	21.3902	24.9861	31.5898	Putative eukaryotic translation initiation factor 2 subunit 3-like pr	chr12:1065	.	.	.	.	.	.	protein_co	ENSG0000	.	.
EIF3A	120.28	127.522	127.002	126.68	141.04	138.166	eukaryotic translation initiation factor 3 subunit A [Source:HGNC	chr10:1207	NM_00375	.	.	.	NP_00374	.	protein_co	ENSG0000	8661	3271
EIF3B	139.664	149.964	131.031	186.416	161.782	126.379	eukaryotic translation initiation factor 3 subunit B [Source:HGNC	chr7:23945	NM_00103	XM_01151	.	.	NP_00103	XP_011513	protein_co	ENSG0000	8662	3280
EIF3C	3.45786	3.24238	2.59932	2.85931	2.82235	2.37955	eukaryotic translation initiation factor 3 subunit C [Source:HGNC	chr16:2869	NM_00103	.	.	.	NP_00103	XP_016879	protein_co	ENSG0000	728689	3279
EIF3CL	0.266112	0.358638	0.217763	0.192894	0.102058	0.152232	eukaryotic translation initiation factor 3 subunit C-like [Source:H	chr16:2839	NM_00109	XM_00525	.	.	NP_00109	XP_005255	protein_co	ENSG0000	728689	26347
EIF3D	177.233	171.277	172.079	202.038	165.87	166.278	eukaryotic translation initiation factor 3 subunit D [Source:HGNC	chr22:3690	NM_00375	XM_01153	.	.	NP_00374	XP_011528	protein_co	ENSG0000	8664	3278
EIF3E	307.435	245.828	366.288	266.939	331.646	429.174	eukaryotic translation initiation factor 3 subunit E [Source:HGNC	chr8:10921	NM_00156	.	.	.	NP_00155	XP_016868	protein_co	ENSG0000	3646	3277
EIF3F	130.409	128.235	142.886	156.55	123.087	127.42	eukaryotic translation initiation factor 3 subunit F [Source:HGNC	chr11:7991	NM_00375	.	.	.	NP_00374	.	protein_co	ENSG0000	8665	3275
EIF3G	119.019	123.035	108.92	150.64	112.35	94.2668	eukaryotic translation initiation factor 3 subunit G [Source:HGNC	chr19:1021	NM_00375	.	.	.	NP_00374	.	protein_co	ENSG0000	8666	3274
EIF3H	239.273	215.7	275.762	233.199	243.831	283.003	eukaryotic translation initiation factor 3 subunit H [Source:HGNC	chr8:11765	NM_00375	.	.	.	NP_00374	.	protein_co	ENSG0000	8667	3273
EIF3I	206.386	196.495	197.626	238.535	197.131	161.567	eukaryotic translation initiation factor 3 subunit I [Source:HGNC S	chr1:32687	NM_00375	XM_01154	.	.	NP_00374	XP_011540	protein_co	ENSG0000	8668	3272
EIF3J	36.9649	34.1471	32.4716	35.4971	52.1685	43.9759	eukaryotic translation initiation factor 3 subunit J [Source:HGNC S	chr15:4482	NM_00128	.	.	.	NP_00127	.	protein_co	ENSG0000	8669	3270
EIF3K	199.58	180.927	177.643	214.012	162.861	134.643	eukaryotic translation initiation factor 3 subunit K [Source:HGNC	chr19:3910	NM_00130	XM_01152	.	.	NP_00128	XP_011525	protein_co	ENSG0000	27335	24656
EIF3L	193.505	196.773	224.176	193.935	173.142	200.718	eukaryotic translation initiation factor 3 subunit L [Source:HGNC S	chr22:3822	NM_00124	XM_00672	.	.	NP_00122	XP_005261	protein_co	ENSG0000	51386	18138
EIF3M	120.896	94.6218	115.575	118.157	124.389	125.58	eukaryotic translation initiation factor 3 subunit M [Source:HGNC	chr11:3260	NM_00130	XM_01151	.	.	NP_00129	XP_011518	protein_co	ENSG0000	10480	24460
EIF4A1	844.736	807.2	678.929	939.73	796.897	694.502	eukaryotic translation initiation factor 4A1 [Source:HGNC Symbol	chr17:7476	NM_00120	.	.	.	NP_00119	.	protein_co	ENSG0000	1973	3282
EIF4A2	203.366	182.166	220.702	221.05	266.209	277.111	eukaryotic translation initiation factor 4A2 [Source:HGNC Symbol	chr3:18650	NM_00196	.	NR_03165	.	NP_00195	.	protein_co	ENSG0000	100302143	3284
EIF4A3	129.002	129.186	103.854	172.61	151.208	114.636	eukaryotic translation initiation factor 4A3 [Source:HGNC Symbol	chr17:7810	NM_01474	XM_01152	.	.	NP_05555	XP_011523	protein_co	ENSG0000	9775	18683
EIF4B	215.143	222.445	267.433	232.817	236.344	281.973	eukaryotic translation initiation factor 4B [Source:HGNC Symbol;	chr12:5339	NM_00130	XM_00671	.	.	NP_00128	.	protein_co	ENSG0000	1975	3285
EIF4E	70.7067	65.8435	57.3569	77.7765	83.6255	82.6141	eukaryotic translation initiation factor 4E [Source:HGNC Symbol;	chr4:99792	NM_00113	XM_00671	.	.	NP_00112	XP_006714	protein_co	ENSG0000	1977	3287
EIF4E1B	0.077131	0.115315	0.167928	0.208142	0.17886	0.251421	eukaryotic translation initiation factor 4E family member 1B [Sou	chr5:17605	NM_00109	XM_01153	.	.	NP_00109	XP_011532	protein_co	ENSG0000	253314	33179
EIF4E2	45.2692	48.6825	39.6772	49.2063	40.9687	37.8918	eukaryotic translation initiation factor 4E family member 2 [Sourc	chr2:23341	NM_00127	XM_01700	.	.	NP_00126	XP_005247	protein_co	ENSG0000	9470	3293
EIF4E3	3.98604	4.28527	5.22385	4.8672	5.47427	5.6913	eukaryotic translation initiation factor 4E family member 3 [Sourc	chr3:71724	NM_00113	.	.	.	NP_00112	XP_016861	protein_co	ENSG0000	317649	31837
EIF4EBP1	87.6171	85.0859	63.3626	85.955	60.595	110.51	eukaryotic translation initiation factor 4E binding protein 1 [Sourc	chr8:37887	NM_00409	.	.	.	NP_00408	.	protein_co	ENSG0000	1978	3288
EIF4EBP2	22.5298	22.6515	28.3298	23.3096	24.46	31.7815	eukaryotic translation initiation factor 4E binding protein 2 [Sourc	chr10:7216	NM_00409	.	.	.	NP_00408	.	protein_co	ENSG0000	1979	3289
EIF4EBP3	2.72E-90	0.493933	1.3E-276	1.31511	0.628077	2.38609	eukaryotic translation initiation factor 4E binding protein 3 [Sourc	chr5:13978	NM_00373	.	.	.	NP_00372	.	protein_co	ENSG0000	8637	3290
EIF4ENIF1	7.92138	8.83451	7.51511	7.79084	8.09748	6.37542	eukaryotic translation initiation factor 4E nuclear import factor 1	chr22:3183	NM_00116	XM_01702	.	.	NP_00115	XP_005261	protein_co	ENSG0000	56478	16687
EIF4G1	138.032	146.104	122.596	163.279	154.655	113.676	eukaryotic translation initiation factor 4 gamma 1 [Source:HGNC	chr3:18403	NM_00119	.	.	.	NP_00118	.	protein_co	ENSG0000	1981	3296
EIF4G2	318.532	318.755	269.565	394.163	435.345	356.388	eukaryotic translation initiation factor 4 gamma 2 [Source:HGNC	chr11:1081	NM_00104	.	.	.	NP_00103	.	protein_co	ENSG0000	1982	3297
EIF4G3	9.27236	8.70682	12.1414	8.29616	9.81395	12.8334	eukaryotic translation initiation factor 4 gamma 3 [Source:HGNC	chr1:21132	NM_00119	XM_01700	.	.	NP_00118	XP_011540	protein_co	ENSG0000	8672	3298
EIF4H	199.276	197.959	203.213	228.793	234.159	235.112	eukaryotic translation initiation factor 4H [Source:HGNC Symbol;	chr7:73588	NM_02217	XM_01151	.	.	NP_07149	XP_011514	protein_co	ENSG0000	7458	12741
EIF5	79.6594	77.3902	69.5841	76.959	97.2857	75.3483	eukaryotic translation initiation factor 5 [Source:HGNC Symbol;Ac	chr14:1038	NM_00196	.	.	.	NP_00196	.	protein_co	ENSG0000	1983	3299
EIF5A	905.652	891.779	744.557	1081.44	897.009	812.574	eukaryotic translation initiation factor 5A [Source:HGNC Symbol;	chr17:7210	NM_00114	XM_00525	.	.	NP_00113	XP_005256	protein_co	ENSG0000	1984	3300
EIF5A2	5.04657	6.71716	7.18191	6.51112	8.88033	8.35326	eukaryotic translation initiation factor 5A2 [Source:HGNC Symbol	chr3:17060	NM_02039	.	.	.	NP_06512	.	protein_co	ENSG0000	56648	3301

EIF5AL1	9.46914	9.19236	8.37392	9.59475	10.2014	9.5202	eukaryotic translation initiation factor 5A-like 1 [Source:HGNC Sy	chr10:8127	NM_00109	.	.	.	NP_00109	.	protein_co	ENSG0000	143244	17419
EIF5B	52.0911	49.37	49.1668	48.0634	59.0151	45.7291	eukaryotic translation initiation factor 5B [Source:HGNC Symbol;	chr2:99953	NM_01590	.	.	.	NP_05698	.	protein_co	ENSG0000	9669	30793
EIF6	203.205	193.305	165.863	224.584	169.884	120.724	eukaryotic translation initiation factor 6 [Source:HGNC Symbol;Ac	chr20:3386	NM_00126	.	.	.	NP_00125	.	protein_co	ENSG0000	3692	6159
ELAC1	7.25079	8.46163	6.72606	7.64688	6.91954	6.32089	elaC ribonuclease Z 1 [Source:HGNC Symbol;Acc:HGNC:14197]	chr18:4849	NM_01869	.	.	.	NP_06116	.	protein_co	ENSG0000	55520	14197
ELAC2	48.5978	49.1387	45.4453	53.9943	50.1502	34.1527	elaC ribonuclease Z 2 [Source:HGNC Symbol;Acc:HGNC:14198]	chr17:1289	NM_00116	.	.	.	NP_00115	.	protein_co	ENSG0000	60528	14198
ELANE	0	0	0	0	0	0	elastase, neutrophil expressed [Source:HGNC Symbol;Acc:HGNC:	chr19:8510	NM_00197	XM_01152	.	.	NP_00196	XP_011526	protein_co	ENSG0000	1991	3309
ELAVL1	52.776	57.3458	49.0157	71.1628	63.3834	46.3978	ELAV like RNA binding protein 1 [Source:HGNC Symbol;Acc:HGNC	chr19:8023	NM_00141	XM_01152	.	.	NP_00141	.	protein_co	ENSG0000	1994	3312
ELAVL2	0	0	0	0	0	0	ELAV like neuron-specific RNA binding protein 2 [Source:HGNC Sy	chr9:23690	NM_00117	XM_01701	.	.	NP_00116	XP_005251	protein_co	ENSG0000	1993	3313
ELAVL3	0.017008	0	0.014682	0.132272	0.03937	0.068581	ELAV like neuron-specific RNA binding protein 3 [Source:HGNC Sy	chr19:1156	NM_00142	XM_01152	.	.	NP_00141	XP_011526	protein_co	ENSG0000	1995	3314
ELAVL4	0	0	0	0	0	0	ELAV like neuron-specific RNA binding protein 4 [Source:HGNC Sy	chr1:50513	NM_00114	XM_01700	.	.	NP_00113	XP_006710	protein_co	ENSG0000	1996	3315
ELF1	12.2707	13.7957	12.6115	13.6787	13.8932	10.3839	E74 like ETS transcription factor 1 [Source:HGNC Symbol;Acc:HGN	chr13:4150	NM_00114	XM_01702	.	.	NP_00113	XP_005266	protein_co	ENSG0000	1997	3316
ELF2	9.59931	10.8402	11.0028	10.6768	11.0141	10.5474	E74 like ETS transcription factor 2 [Source:HGNC Symbol;Acc:HGN	chr4:13997	NM_00127	XM_00671	.	.	NP_00126	XP_005262	protein_co	ENSG0000	1998	3317
ELF3	34.6112	36.3777	48.4113	19.5152	16.3652	19.0408	E74 like ETS transcription factor 3 [Source:HGNC Symbol;Acc:HGN	chr1:20197	NM_00111	.	.	.	NP_00110	.	protein_co	ENSG0000	1999	3318
ELF4	12.0554	12.3828	10.5338	11.6582	10.325	8.06154	E74 like ETS transcription factor 4 [Source:HGNC Symbol;Acc:HGN	chrX:12919	NM_00112	XM_01153	.	.	NP_00112	XP_005262	protein_co	ENSG0000	2000	3319
ELF5	0.1416	0.037658	0.288465	0.200158	0.131464	0.230486	E74 like ETS transcription factor 5 [Source:HGNC Symbol;Acc:HGN	chr11:3450	NM_00124	.	.	.	NP_00123	XP_016872	protein_co	ENSG0000	2001	3320
ELFN1	1.70418	2.05352	1.9785	1.71417	1.65381	2.18941	extracellular leucine rich repeat and fibronectin type III domain c	chr7:17277	NM_00112	XM_01151	.	.	NP_00112	XP_006715	protein_co	ENSG0000	392617	33154
ELFN2	11.4958	12.3008	13.4949	8.32097	8.59446	8.54965	extracellular leucine rich repeat and fibronectin type III domain c	chr22:3776	NM_05290	.	.	.	NP_44313	.	protein_co	ENSG0000	114794	29396
ELK1	26.4097	27.4543	24.2415	27.7819	29.068	25.0721	ELK1, ETS transcription factor [Source:HGNC Symbol;Acc:HGNC:3	chrX:47494	NM_00111	XM_01702	.	.	NP_00110	XP_016884	protein_co	ENSG0000	2002	3321
ELK3	24.868	27.0992	28.0311	21.5902	23.3382	25.0105	ELK3, ETS transcription factor [Source:HGNC Symbol;Acc:HGNC:3	chr12:9658	NM_00523	XM_00671	.	.	NP_00522	XP_006719	protein_co	ENSG0000	2004	3325
ELK4	9.93317	10.5775	11.0371	7.11778	9.38437	12.637	ELK4, ETS transcription factor [Source:HGNC Symbol;Acc:HGNC:3	chr1:20557	NM_00197	XM_00524	.	.	NP_00196	XP_005245	protein_co	ENSG0000	2005	3326
ELL	8.83386	9.58199	8.70681	9.09796	8.52304	8.02216	elongation factor for RNA polymerase II [Source:HGNC Symbol;Ac	chr19:1855	NM_00653	XM_01702	.	.	NP_00652	XP_016882	protein_co	ENSG0000	8178	23114
ELL2	7.09152	7.2296	6.26509	6.28562	6.98322	9.10884	elongation factor for RNA polymerase II 2 [Source:HGNC Symbol;	chr5:95220	NM_01208	XM_01700	.	.	NP_03621	XP_006714	protein_co	ENSG0000	22936	17064
ELL3	6.83604	5.36511	7.51057	6.23514	4.77375	4.24628	elongation factor for RNA polymerase II 3 [Source:HGNC Symbol;	chr15:4403	NM_02516	.	.	.	NP_07944	.	protein_co	ENSG0000	80237	23113
ELMO1	0.025406	0	0.0333	0.084489	0.043977	0.1233	engulfment and cell motility 1 [Source:HGNC Symbol;Acc:HGNC:1	chr7:36893	NM_00103	XM_01151	.	.	NP_00103	XP_005249	protein_co	ENSG0000	9844	16286
ELMO2	8.20912	7.99416	7.52528	7.80171	7.96284	7.28332	engulfment and cell motility 2 [Source:HGNC Symbol;Acc:HGNC:1	chr20:4499	NM_13317	XM_00672	.	.	NP_00130	XP_005260	protein_co	ENSG0000	63916	17233
ELMO3	18.9181	18.595	21.2491	9.32627	9.16369	8.71882	engulfment and cell motility 3 [Source:HGNC Symbol;Acc:HGNC:1	chr16:6723	NM_02471	.	.	.	NP_07898	.	protein_co	ENSG0000	79767	17289
ELMOD1	1.69926	1.29006	1.60815	1.89307	2.09734	1.71567	ELMO domain containing 1 [Source:HGNC Symbol;Acc:HGNC:253	chr11:1074	NM_00113	XM_01154	NR_02832	.	NP_00112	XP_011541	protein_co	ENSG0000	643923	25334
ELMOD2	12.0096	12.1387	13.6871	11.6486	13.0565	18.2577	ELMO domain containing 2 [Source:HGNC Symbol;Acc:HGNC:281	chr4:14144	NM_15370	XM_01153	.	.	NP_71491	XP_005262	protein_co	ENSG0000	255520	28111
ELMOD3	4.41115	4.18855	2.32159	3.7769	2.85863	2.05382	ELMO domain containing 3 [Source:HGNC Symbol;Acc:HGNC:261	chr2:85581	NM_00113	XM_01700	.	.	NP_00112	XP_005264	protein_co	ENSG0000	84173	26158
ELMSAN1	9.11529	10.5935	9.35006	6.95064	7.71026	7.70946	ELM2 and Myb/SANT domain containing 1 [Source:HGNC Symbol	chr14:7418	NM_00104	XM_01153	.	.	NP_00103	XP_005268	protein_co	ENSG0000	91748	19853
ELN	0.097334	0	0.060461	0.158337	0	0	elastin [Source:HGNC Symbol;Acc:HGNC:3327]	chr7:73442	NM_00050	XM_01151	.	.	NP_00049	XP_005250	protein_co	ENSG0000	2006	3327
ELOF1	65.2906	66.8322	57.8849	82.822	66.2471	38.8316	elongation factor 1 homolog [Source:HGNC Symbol;Acc:HGNC:28	chr19:1166	NM_03237	XM_01702	.	.	NP_11575	XP_016882	protein_co	ENSG0000	84337	28691
ELOVL1	80.6825	73.9584	61.1353	78.0233	65.9483	68.4359	ELOVL fatty acid elongase 1 [Source:HGNC Symbol;Acc:HGNC:144	chr1:43829	NM_00125	.	NR_10679	.	NP_00124	.	protein_co	ENSG0000	102466723	14418
ELOVL2	0	0	0	0	0	0	ELOVL fatty acid elongase 2 [Source:HGNC Symbol;Acc:HGNC:144	chr6:10980	NM_01777	XM_01151	.	.	NP_06024	XP_011513	protein_co	ENSG0000	54898	14416
ELOVL3	9.58158	7.90972	6.74216	12.3417	10.1444	10.2646	ELOVL fatty acid elongase 3 [Source:HGNC Symbol;Acc:HGNC:180	chr10:1039	NM_15231	XM_01154	.	.	NP_68952	XP_011538	protein_co	ENSG0000	83401	18047
ELOVL4	0	0	0	0	0	0	ELOVL fatty acid elongase 4 [Source:HGNC Symbol;Acc:HGNC:144	chr6:80624	NM_02272	.	.	.	NP_07356	.	protein_co	ENSG0000	6785	14415
ELOVL5	61.8212	58.2669	77.0069	69.4949	74.4548	67.2062	ELOVL fatty acid elongase 5 [Source:HGNC Symbol;Acc:HGNC:213	chr6:53132	NM_00124	.	.	.	NP_00122	.	protein_co	ENSG0000	60481	21308
ELOVL6	27.0396	27.5664	29.2989	25.77	28.596	28.7646	ELOVL fatty acid elongase 6 [Source:HGNC Symbol;Acc:HGNC:158	chr4:11096	NM_00113	XM_01153	.	.	NP_00112	XP_011530	protein_co	ENSG0000	79071	15829
ELOVL7	8.14625	7.64383	6.92281	5.94215	8.5632	9.27767	ELOVL fatty acid elongase 7 [Source:HGNC Symbol;Acc:HGNC:262	chr5:60047	NM_00110	XM_01700	.	.	NP_00109	XP_005248	protein_co	ENSG0000	79993	26292
ELP2	25.6814	27.915	33.0048	43.0973	42.4983	51.1643	elongator acetyltransferase complex subunit 2 [Source:HGNC Syn	chr18:3370	NM_00124	.	.	.	NP_00122	.	protein_co	ENSG0000	55250	18248
ELP3	20.15	19.8104	17.6259	20.2056	21.1829	22.6058	elongator acetyltransferase complex subunit 3 [Source:HGNC Syn	chr8:27947	NM_00128	XM_00671	.	.	NP_00127	XP_006716	protein_co	ENSG0000	55140	20696
ELP4	11.8144	11.5107	13.2959	12.3462	16.0816	14.8616	elongator acetyltransferase complex subunit 4 [Source:HGNC Syn	chr11:3153	NM_00128	XM_00525	.	.	NP_00127	.	protein_co	ENSG0000	26610	1171
ELP5	63.683	61.2643	46.1202	69.1116	47.6365	44.5561	elongator acetyltransferase complex subunit 5 [Source:HGNC Syn	chr17:7146	NM_01536	XM_01152	.	.	NP_05617	XP_011522	protein_co	ENSG0000	23587	30617
ELP6	21.2348	23.9825	19.8977	29.4476	21.089	21.3043	elongator acetyltransferase complex subunit 6 [Source:HGNC Syn	chr3:47537	NM_00103	XM_01153	.	.	NP_00102	XP_005265	protein_co	ENSG0000	54859	25976
ELSPBP1	0	0	0	0	0	0	epididymal sperm binding protein 1 [Source:HGNC Symbol;Acc:H	chr19:4849	NM_02214	XM_00672	.	.	NP_07142	.	protein_co	ENSG0000	64100	14417
EMB	5.99688	5.05301	5.63134	5.5512	7.51639	6.76833	embigin [Source:HGNC Symbol;Acc:HGNC:30465]	chr5:49692	NM_19844	XM_01154	.	.	NP_94085	XP_011541	protein_co	ENSG0000	133418	30465
EMC1	25.4882	27.6416	26.5121	26.6197	26.9371	23.2403	ER membrane protein complex subunit 1 [Source:HGNC Symbol;	chr1:19542	NM_00127	XM_00524	.	.	NP_00125	XP_005245	protein_co	ENSG0000	23065	28957
EMC10	87.2399	84.0604	68.1684	97.9312	88.503	66.531	ER membrane protein complex subunit 10 [Source:HGNC Symbol	chr19:5097	NM_17506	.	.	.	NP_77823	.	protein_co	ENSG0000	284361	27609
EMC2	28.9057	21.0196	22.2114	24.2156	31.2488	28.8505	ER membrane protein complex subunit 2 [Source:HGNC Symbol;	chr8:10945	NM_01467	.	.	.	NP_00131	.	protein_co	ENSG0000	9694	28963
EMC3	8.00323	6.5599	5.94257	7.4538	6.25122	4.86359	ER membrane protein complex subunit 3 [Source:HGNC Symbol;	chr3:10004	NM_01844	XM_01153	.	.	NP_06091	XP_011532	protein_co	ENSG0000	55831	23999
EMC4	87.4589	85.7246	72.7697	98.5937	93.3352	78.6123	ER membrane protein complex subunit 4 [Source:HGNC Symbol;	chr15:3451	NM_00128	.	.	.	NP_00127	.	protein_co	ENSG0000	51234	28032
EMC6	105.404	99.5311	87.0133	131.476	104.929	87.9849	ER membrane protein complex subunit 6 [Source:HGNC Symbol;	chr17:3572	NM_00101	.	.	.	NP_00101	.	protein_co	ENSG0000	83460	28430
EMC7	38.5443	33.1472	27.9793	33.8738	34.862	35.3871	ER membrane protein complex subunit 7 [Source:HGNC Symbol;	chr15:3437	NM_02015	.	.	.	NP_06453	.	protein_co	ENSG0000	56851	24301
EMC8	52.784	54.3704	48.2741	65.7523	56.5841	44.6884	ER membrane protein complex subunit 8 [Source:HGNC Symbol;	chr16:8581	NM_00114	.	.	.	NP_00113	.	protein_co	ENSG0000	10328	7864
EMC9	24.1654	24.1867	19.3725	29.4842	21.6609	18.7264	ER membrane protein complex subunit 9 [Source:HGNC Symbol;	chr14:2460	NM_01604	XM_01153	.	.	NP_05713	XP_005267	protein_co	ENSG0000	51016	20273
EMCN	0	0	0	0	0	0	endomucin [Source:HGNC Symbol;Acc:HGNC:16041]	chr4:10131	NM_00115	XM_01153	.	.	NP_00115	XP_011530	protein_co	ENSG0000	51705	16041
EMD	111.788	102.005	89.8596	121.764	108.6	85.1415	emerin [Source:HGNC Symbol;Acc:HGNC:3331]	chrX:15360	NM_00011	.	.	.	NP_00010	.	protein_co	ENSG0000	2010	3331
EME1	12.5899	13.1502	12.1	14.5246	15.1641	10.4999	essential meiotic structure-specific endonuclease 1 [Source:HGN	chr17:4845	NM_00116	XM_01152	.	.	NP_00115	XP_005257	protein_co	ENSG0000	146956	24965
EME2	3.74324	3.7096	5.14441	2.54422	2.22541	2.59427	essential meiotic structure-specific endonuclease subunit 2 [Sour	chr16:1823	NM_00125	.	.	.	NP_00124	.	protein_co	ENSG0000	197342	27289

EMG1	19.6582	19.5294	16.8206	23.0893	18.2455	15.7597	EMG1, N1-specific pseudouridine methyltransferase [Source:HGN	chr12:7080	NM_00633	XM_01152	.	.	NP_00632	.	protein_co	ENSG0000	10436	16912
EMID1	6.38505	5.94762	9.71873	6.16884	5.03432	7.18968	EMI domain containing 1 [Source:HGNC Symbol;Acc:HGNC:18036	chr22:2960	NM_00126	XM_01152	.	.	NP_00125	XP_005261	protein_co	ENSG0000	129080	18036
EMILIN1	0.089196	0.191879	0.153177	0.234296	0.158374	0.051021	elastin microfibril interfacer 1 [Source:HGNC Symbol;Acc:HGNC:1	chr2:27301	NM_00704	XM_00671	.	.	NP_00897	.	protein_co	ENSG0000	11117	19880
EMILIN2	2.85919	2.89894	2.36095	3.25269	2.30745	2.40736	elastin microfibril interfacer 2 [Source:HGNC Symbol;Acc:HGNC:1	chr18:2847	NM_03204	.	.	.	NP_11443	.	protein_co	ENSG0000	84034	19881
EMILIN3	0.020157	0.019879	0	0.021564	0	0	elastin microfibril interfacer 3 [Source:HGNC Symbol;Acc:HGNC:1	chr20:3996	NM_05284	.	.	.	NP_44307	.	protein_co	ENSG0000	90187	16123
EML1	2.17068	2.46147	2.08332	3.68287	4.03074	3.64312	echinoderm microtubule associated protein like 1 [Source:HGNC	chr14:1002	NM_00100	XM_01153	.	.	NP_00100	XP_005267	protein_co	ENSG0000	2009	3330
EML2	29.7694	30.869	27.664	26.881	24.2629	27.2305	echinoderm microtubule associated protein like 2 [Source:HGNC	chr19:4611	NM_00119	XM_01152	NR_02988	.	NP_00118	XP_006723	protein_co	ENSG0000	442902	18035
EML3	23.2124	27.4265	29.3472	18.6272	16.0094	15.7886	echinoderm microtubule associated protein like 3 [Source:HGNC	chr11:6236	NM_00130	XM_01154	.	.	NP_00128	XP_005273	protein_co	ENSG0000	256364	26666
EML4	16.7877	18.8377	21.5087	15.4245	20.5726	22.0415	echinoderm microtubule associated protein like 4 [Source:HGNC	chr2:42396	NM_00114	XM_00671	.	.	NP_00113	XP_005264	protein_co	ENSG0000	27436	1316
EML5	0.729281	0.698338	0.580502	0.440534	0.522757	0.662066	echinoderm microtubule associated protein like 5 [Source:HGNC	chr14:8902	NM_18338	XM_01153	.	.	NP_89924	XP_006720	protein_co	ENSG0000	161436	18197
EML6	1.04269	1.01799	1.57139	0.797576	0.815118	0.855885	echinoderm microtubule associated protein like 6 [Source:HGNC	chr2:54951	NM_00103	XM_01153	.	.	NP_00103	XP_016859	protein_co	ENSG0000	400954	35412
EMP1	10.8488	10.7335	12.9718	12.526	15.6069	16.6048	epithelial membrane protein 1 [Source:HGNC Symbol;Acc:HGNC:	chr12:1334	NM_00142	.	.	.	NP_00141	.	protein_co	ENSG0000	2012	3333
EMP2	11.4003	10.2606	9.65702	15.6144	16.3504	12.1947	epithelial membrane protein 2 [Source:HGNC Symbol;Acc:HGNC:	chr16:1062	NM_00142	XM_00672	.	.	NP_00141	XP_006720	protein_co	ENSG0000	2013	3334
EMP3	127.059	125.163	79.372	116.498	76.8534	66.0705	epithelial membrane protein 3 [Source:HGNC Symbol;Acc:HGNC:	chr19:4882	NM_00131	XM_01152	.	.	NP_00130	XP_011524	protein_co	ENSG0000	2014	3335
EMSY	5.29261	5.61376	5.13607	4.68968	5.59141	5.69169	EMSY, BRCA2 interacting transcriptional repressor [Source:HGNC	chr11:7615	NM_00130	XM_01154	.	.	NP_00128	XP_005274	protein_co	ENSG0000	56946	18071
EMX1	2.11829	1.43199	0.856499	1.14895	1.40614	1.30863	empty spiracles homeobox 1 [Source:HGNC Symbol;Acc:HGNC:33	chr2:73143	NM_00409	.	.	.	NP_00408	.	protein_co	ENSG0000	2016	3340
EMX2	0	0	0	0.034939	0.03689	0	empty spiracles homeobox 2 [Source:HGNC Symbol;Acc:HGNC:33	chr10:1193	NM_00116	.	.	.	NP_00115	.	protein_co	ENSG0000	2018	3341
EN1	0	0	0	0	0	0	engrailed homeobox 1 [Source:HGNC Symbol;Acc:HGNC:3342]	chr2:11959	NM_00142	.	.	.	NP_00141	.	protein_co	ENSG0000	2019	3342
EN2	0.521799	0.457021	0.357867	0.191153	0.397588	0.437447	engrailed homeobox 2 [Source:HGNC Symbol;Acc:HGNC:3343]	chr7:15525	NM_00142	.	.	.	NP_00141	.	protein_co	ENSG0000	2020	3343
ENAH	23.8681	27.0612	26.1208	25.4736	28.5637	30.0898	enabled homolog (Drosophila) [Source:HGNC Symbol;Acc:HGNC:	chr1:22567	NM_00100	XM_01700	.	.	NP_00100	XP_005273	protein_co	ENSG0000	55740	18271
ENAM	0.012145	0.012914	0	0.013724	0	0	enamelin [Source:HGNC Symbol;Acc:HGNC:3344]	chr4:71494	NM_03188	XM_00671	.	.	NP_11409	XP_006714	protein_co	ENSG0000	10117	3344
ENC1	21.9929	21.6936	29.927	25.273	30.3962	38.3994	ectodermal-neural cortex 1 [Source:HGNC Symbol;Acc:HGNC:334	chr5:73923	NM_00125	XM_01154	.	.	NP_00124	XP_011541	protein_co	ENSG0000	8507	3345
ENDOD1	23.3555	23.6129	28.7725	25.2714	26.7228	29.4702	endonuclease domain containing 1 [Source:HGNC Symbol;Acc:HG	chr11:9482	NM_01503	.	.	.	NP_05585	.	protein_co	ENSG0000	23052	29129
ENDOG	16.735	15.4643	15.7129	18.2897	13.3542	14.9738	endonuclease G [Source:HGNC Symbol;Acc:HGNC:3346]	chr9:13158	NM_00443	.	.	.	NP_00442	.	protein_co	ENSG0000	2021	3346
ENDOU	0.043546	0	0.024676	0.031312	0	0.045416	endonuclease, poly(U) specific [Source:HGNC Symbol;Acc:HGNC:	chr12:4810	NM_00117	.	.	.	NP_00116	.	protein_co	ENSG0000	8909	14369
ENDOV	10.7735	10.6175	10.7967	12.8229	10.5551	13.8631	endonuclease V [Source:HGNC Symbol;Acc:HGNC:26640]	chr17:7838	NM_00116	XM_01702	.	.	NP_00115	XP_005257	protein_co	ENSG0000	284131	26640
ENG	0	0	0.074461	0	0	0	endoglin [Source:HGNC Symbol;Acc:HGNC:3349]	chr9:13057	NM_00011	.	.	.	NP_00010	.	protein_co	ENSG0000	2022	3349
ENGASE	8.41056	9.16844	10.7115	9.12425	8.63306	6.96331	endo-beta-N-acetylglucosaminidase [Source:HGNC Symbol;Acc:H	chr17:7707	NM_00104	XM_00672	.	.	NP_00103	.	protein_co	ENSG0000	64772	24622
ENHO	8.43104	7.63849	7.88234	9.28291	7.08552	6.06962	energy homeostasis associated [Source:HGNC Symbol;Acc:HGNC:	chr9:34521	NM_19857	.	.	.	NP_94097	.	protein_co	ENSG0000	375704	24838
ENKD1	12.144	10.5768	10.6887	3.07378	2.91618	3.42681	enkurin domain containing 1 [Source:HGNC Symbol;Acc:HGNC:25	chr16:6769	NM_03214	.	.	.	NP_11551	.	protein_co	ENSG0000	84080	25246
ENKUR	0.02494	0.026773	0.06526	0.121677	0.181821	0.106091	enkurin, TRPC channel interacting protein [Source:HGNC Symbol;	chr10:2527	NM_00127	XM_01151	.	.	NP_00125	XP_006717	protein_co	ENSG0000	219670	28388
ENO1	1352.36	1383.68	1224.44	1749.6	1455.76	1318.62	enolase 1 [Source:HGNC Symbol;Acc:HGNC:3350]	chr1:89210	NM_00142	XM_00671	.	.	NP_00141	XP_006710	protein_co	ENSG0000	2023	3350
ENO2	30.5932	33.4841	33.8095	16.3285	18.3442	14.6232	enolase 2 [Source:HGNC Symbol;Acc:HGNC:3353]	chr12:7023	NM_00197	.	.	.	NP_00196	.	protein_co	ENSG0000	2026	3353
ENO3	4.60322	4.5218	4.17411	4.41224	2.52517	2.70841	enolase 3 [Source:HGNC Symbol;Acc:HGNC:3354]	chr17:4854	NM_00119	XM_01152	.	.	NP_00118	XP_005256	protein_co	ENSG0000	2027	3354
ENO4	0.128029	0.05361	0.114511	0.028587	0.028868	0.04238	enolase family member 4 [Source:HGNC Symbol;Acc:HGNC:3167	chr10:1184	NM_00124	XM_01153	.	.	NP_00122	XP_006717	protein_co	ENSG0000	387712	31670
ENOPH1	44.1054	43.6064	37.4531	53.2585	45.5492	41.6026	enolase-phosphatase 1 [Source:HGNC Symbol;Acc:HGNC:24599]	chr4:83351	NM_00129	.	.	.	NP_00127	.	protein_co	ENSG0000	58478	24599
ENOSF1	14.2739	13.8902	13.1866	12.2399	10.6687	10.9467	enolase superfamily member 1 [Source:HGNC Symbol;Acc:HGNC:	chr18:6576	NM_00112	XM_01152	.	.	NP_00111	XP_011523	protein_co	ENSG0000	55556	30365
ENOX1	2.35733	2.40529	2.09237	1.87854	2.33104	2.02678	ecto-NOX disulfide-thiol exchanger 1 [Source:HGNC Symbol;Acc:H	chr13:4378	NM_00112	XM_01153	.	.	NP_00112	XP_005266	protein_co	ENSG0000	55068	25474
ENOX2	5.037	4.45438	3.82034	4.30686	4.72456	4.91451	ecto-NOX disulfide-thiol exchanger 2 [Source:HGNC Symbol;Acc:H	chrX:12975	NM_00128	XM_01153	.	.	NP_00126	XP_005262	protein_co	ENSG0000	10495	2259
ENPEP	0.971034	1.03039	0.78613	0.658963	0.734874	0.842331	glutamyl aminopeptidase [Source:HGNC Symbol;Acc:HGNC:3355]	chr4:11139	NM_00197	.	.	.	NP_00196	.	protein_co	ENSG0000	2028	3355
ENPP1	2.55833	2.70177	2.85871	3.03502	2.90262	3.98293	ectonucleotide pyrophosphatase/phosphodiesterase 1 [Source:H	chr6:13212	NM_00620	.	.	.	NP_00619	.	protein_co	ENSG0000	5167	3356
ENPP2	0.078165	0.070858	0.204741	0.205086	0.194695	0.779764	ectonucleotide pyrophosphatase/phosphodiesterase 2 [Source:H	chr8:12056	NM_00104	XM_01701	.	.	NP_00103	XP_006716	protein_co	ENSG0000	5168	3357
ENPP3	0.141573	0.082452	0.10652	0.116624	0.085642	0.08829	ectonucleotide pyrophosphatase/phosphodiesterase 3 [Source:H	chr6:13194	NM_00502	XM_01153	.	.	NP_00501	XP_011534	protein_co	ENSG0000	5169	3358
ENPP4	6.82345	6.75928	7.66077	6.60064	7.531	9.44874	ectonucleotide pyrophosphatase/phosphodiesterase 4 (putative)	chr6:46097	NM_01493	.	.	.	NP_05575	.	protein_co	ENSG0000	22875	3359
ENPP5	3.22429	3.26008	3.74135	2.24284	2.8012	3.51618	ectonucleotide pyrophosphatase/phosphodiesterase 5 (putative)	chr6:46126	NM_00129	XM_01151	.	.	NP_00127	XP_005249	protein_co	ENSG0000	59084	13717
ENPP6	0	0	0.016198	0	0	0.019091	ectonucleotide pyrophosphatase/phosphodiesterase 6 [Source:H	chr4:18500	NM_15334	.	.	.	NP_69917	.	protein_co	ENSG0000	133121	23409
ENPP7	0	0	0	0	0	0	ectonucleotide pyrophosphatase/phosphodiesterase 7 [Source:H	chr17:7770	NM_17854	.	.	.	NP_84863	.	protein_co	ENSG0000	339221	23764
ENSA	93.8126	88.461	71.6768	96.1682	87.007	75.273	endosulfine alpha [Source:HGNC Symbol;Acc:HGNC:3360]	chr1:15057	NM_00443	.	.	.	NP_00442	.	protein_co	ENSG0000	2029	3360
ENTHD1	0	0	0	0.032432	0	0	ENTH domain containing 1 [Source:HGNC Symbol;Acc:HGNC:2635	chr22:4013	NM_15251	XM_01152	.	.	NP_68972	XP_006724	protein_co	ENSG0000	150350	26352
ENTPD1	0.015233	0.032468	0.01322	0.034522	0.0515	0	ectonucleoside triphosphate diphosphohydrolase 1 [Source:HGN	chr10:9747	NM_00109	XM_01154	.	.	NP_00109	XP_011538	protein_co	ENSG0000	953	3363
ENTPD2	8.40523	10.6532	12.6998	8.10224	7.17413	9.90276	ectonucleoside triphosphate diphosphohydrolase 2 [Source:HGN	chr9:13994	NM_00124	XM_01151	.	.	NP_00123	XP_011517	protein_co	ENSG0000	954	3364
ENTPD3	0	0	0	0	0	0	ectonucleoside triphosphate diphosphohydrolase 3 [Source:HGN	chr3:40428	NM_00124	XM_00526	.	.	NP_00123	.	protein_co	ENSG0000	956	3365
ENTPD4	15.4644	15.974	15.7844	11.7522	13.0251	15.3345	ectonucleoside triphosphate diphosphohydrolase 4 [Source:HGN	chr8:23154	NM_00112	.	.	.	NP_00112	.	protein_co	ENSG0000	9583	14573
ENTPD5	5.64195	5.75406	6.3142	4.56179	5.48339	5.8997	ectonucleoside triphosphate diphosphohydrolase 5 [Source:HGN	chr14:7431	NM_00124	XM_01153	.	.	NP_00124	XP_005268	protein_co	ENSG0000	957	3367
ENTPD6	32.902	31.4783	29.6828	34.2641	31.4317	32.4285	ectonucleoside triphosphate diphosphohydrolase 6 (putative) [So	chr20:2517	NM_00111	XM_01702	.	.	NP_00110	XP_005260	protein_co	ENSG0000	955	3368
ENTPD7	6.91129	6.74564	6.61374	7.50138	8.80157	8.07344	ectonucleoside triphosphate diphosphohydrolase 7 [Source:HGN	chr10:1014	NM_02035	.	.	.	NP_06508	.	protein_co	ENSG0000	57089	19745
ENTPD8	0.398568	0.463058	0.821839	0.167539	0.203913	0.294883	ectonucleoside triphosphate diphosphohydrolase 8 [Source:HGN	chr9:14031	NM_00103	XM_01151	.	.	NP_00102	XP_011516	protein_co	ENSG0000	377841	24860
ENY2	129.272	122.094	107.198	148.395	113.735	100.99	ENY2, transcription and export complex 2 subunit [Source:HGNC	chr8:11025	NM_00119	.	.	.	NP_00118	.	protein_co	ENSG0000	56943	24449
EOGT	5.03774	4.48202	4.50685	4.81283	4.29565	4.78309	EGF domain specific O-linked N-acetylglucosamine transferase [S	chr3:69024	NM_00127	XM_01153	.	.	NP_00126	XP_005264	protein_co	ENSG0000	285203	28526

EOMES	1.72505	2.79858	2.54425	2.73702	2.30204	1.60291	eomesodermin [Source:HGNC Symbol;Acc:HGNC:3372]	chr3:27757	NM_00127	XM_00526	.	.	NP_00126	XP_005265	protein_co	ENSG0000	8320	3372
EP300	8.95589	10.011	8.08135	7.34933	7.44508	8.36174	E1A binding protein p300 [Source:HGNC Symbol;Acc:HGNC:3373]	chr22:4148	NM_00142	XM_00672	NR_03169	.	NP_00142	XP_006724	protein_co	ENSG0000	100302237	3373
EP400	8.98215	10.0045	9.03463	8.03864	8.5112	9.12111	E1A binding protein p400 [Source:HGNC Symbol;Acc:HGNC:1195	chr12:1324	NM_01540	.	.	.	NP_05622	.	protein_co	ENSG0000	57634	11958
EP400NL	1.75131	1.67365	2.26487	1.52356	1.07838	1.20228	EP400 N-terminal like [Source:HGNC Symbol;Acc:HGNC:26602]	chr12:1325	.	.	.	.	.	.	protein_co	ENSG0000	.	26602
EPAS1	7.49967	7.89295	9.98432	7.78492	7.21221	8.33504	endothelial PAS domain protein 1 [Source:HGNC Symbol;Acc:HGN	chr2:46524	NM_00143	XM_01153	.	.	NP_00142	XP_011531	protein_co	ENSG0000	2034	3374
EPB41	16.0456	15.9221	18.7267	8.21874	9.30858	9.601	erythrocyte membrane protein band 4.1 [Source:HGNC Symbol;A	chr1:29213	NM_00116	XM_01154	.	.	NP_00115	XP_005245	protein_co	ENSG0000	2035	3377
EPB41L1	8.93865	8.533	11.5263	10.2094	9.31914	8.39545	erythrocyte membrane protein band 4.1 like 1 [Source:HGNC Syn	chr20:3467	NM_00125	XM_01702	.	.	NP_00124	XP_011526	protein_co	ENSG0000	2036	3378
EPB41L2	25.4264	29.8917	27.5007	26.4769	28.0816	27.2501	erythrocyte membrane protein band 4.1 like 2 [Source:HGNC Syn	chr6:13116	NM_00113	XM_01701	.	.	NP_00112	XP_006715	protein_co	ENSG0000	2037	3379
EPB41L3	0	0	0	0	0	0	erythrocyte membrane protein band 4.1 like 3 [Source:HGNC Syn	chr18:5392	NM_00128	XM_01702	.	.	NP_00126	XP_011523	protein_co	ENSG0000	23136	3380
EPB41L4A	2.36998	2.01527	2.36316	1.00194	1.01875	1.22803	erythrocyte membrane protein band 4.1 like 4A [Source:HGNC Sy	chr5:11149	NM_02214	XM_01154	.	.	NP_07142	XP_011541	protein_co	ENSG0000	64097	13278
EPB41L4A-AS2	0.123409	0.692736	0.419345	0.128847	0	0	EPB41L4A antisense RNA 2 (head to head) [Source:HGNC Symbol;	chr5:11175	.	.	NR_02770	.	.	.	lincRNA	ENSG0000	54508	25643
EPB41L4B	7.02071	6.141	6.21888	6.37431	7.81109	5.73181	erythrocyte membrane protein band 4.1 like 4B [Source:HGNC Sy	chr9:11193	NM_01842	XM_01701	.	.	NP_06089	XP_011517	protein_co	ENSG0000	54566	19818
EPB41L5	6.11993	6.14324	7.60281	6.131	7.56917	7.93099	erythrocyte membrane protein band 4.1 like 5 [Source:HGNC Syn	chr2:12077	NM_00118	XM_01700	.	.	NP_00117	XP_006712	protein_co	ENSG0000	57669	19819
EPB42	0	0	0	0	0	0	erythrocyte membrane protein band 4.2 [Source:HGNC Symbol;A	chr15:4347	NM_00011	XM_01152	.	.	NP_00011	XP_005254	protein_co	ENSG0000	2038	3381
EPC1	6.429	6.01054	4.74322	5.54946	6.31234	6.84702	enhancer of polycomb homolog 1 [Source:HGNC Symbol;Acc:HGN	chr10:3255	NM_00127	XM_00671	NR_10416	.	NP_00125	XP_006717	protein_co	ENSG0000	80314	19876
EPC2	7.75922	6.53245	6.63787	5.73867	6.61476	6.78959	enhancer of polycomb homolog 2 [Source:HGNC Symbol;Acc:HGN	chr2:14940	NM_01563	XM_01151	.	.	NP_05644	XP_011509	protein_co	ENSG0000	26122	24543
EPCAM	168.735	173.283	136.309	185.133	181.033	175.636	epithelial cell adhesion molecule [Source:HGNC Symbol;Acc:HGN	chr2:47572	NM_00235	.	.	.	NP_00234	.	protein_co	ENSG0000	4072	11529
EPDR1	10.3893	11.0497	8.46666	11.4579	10.0504	7.46313	ependymin related 1 [Source:HGNC Symbol;Acc:HGNC:17572]	chr7:37723	NM_00124	.	.	.	NP_00122	.	protein_co	ENSG0000	54749	17572
EPG5	2.43794	2.74341	2.83379	1.59479	1.77663	2.29859	ectopic P-granules autophagy protein 5 homolog [Source:HGNC S	chr18:4342	NM_02096	XM_01152	.	.	NP_06601	XP_011524	protein_co	ENSG0000	57724	29331
EPGN	0.411911	0.076362	0.06198	0.242245	0.079079	0.448897	epithelial mitogen [Source:HGNC Symbol;Acc:HGNC:17470]	chr4:75174	NM_00127	XM_01153	.	.	NP_00125	XP_005265	protein_co	ENSG0000	255324	17470
EPHA1	6.72767	6.20423	8.00507	8.02593	6.54662	8.52962	EPH receptor A1 [Source:HGNC Symbol;Acc:HGNC:3385]	chr7:14308	NM_00523	XM_00671	.	.	NP_00522	XP_006715	protein_co	ENSG0000	2041	3385
EPHA10	3.39153	2.84564	3.67249	3.09844	2.6723	2.38886	EPH receptor A10 [Source:HGNC Symbol;Acc:HGNC:19987]	chr1:38181	NM_00109	.	.	.	NP_00109	.	protein_co	ENSG0000	284656	19987
EPHA2	35.3267	36.2323	45.1126	29.0662	28.1263	45.3569	EPH receptor A2 [Source:HGNC Symbol;Acc:HGNC:3386]	chr1:16450	NM_00132	.	.	.	NP_00131	XP_016856	protein_co	ENSG0000	1969	3386
EPHA3	0	0	0	0	0	0	EPH receptor A3 [Source:HGNC Symbol;Acc:HGNC:3387]	chr3:89156	NM_00523	XM_00526	.	.	NP_00522	XP_005264	protein_co	ENSG0000	2042	3387
EPHA4	1.45251	1.761	1.30471	1.70399	2.03528	1.97188	EPH receptor A4 [Source:HGNC Symbol;Acc:HGNC:3388]	chr2:22228	NM_00130	XM_00524	.	.	NP_00129	XP_005246	protein_co	ENSG0000	2043	3388
EPHA5	0	0	0	0	0	0	EPH receptor A5 [Source:HGNC Symbol;Acc:HGNC:3389]	chr4:66185	NM_00128	XM_01153	.	.	NP_00126	XP_005265	protein_co	ENSG0000	2044	3389
EPHA6	0	0	0	0	0	0	EPH receptor A6 [Source:HGNC Symbol;Acc:HGNC:19296]	chr3:96533	NM_00108	XM_00671	.	.	NP_00107	XP_006713	protein_co	ENSG0000	285220	19296
EPHA7	0	0.013219	0	0.014058	0	0	EPH receptor A7 [Source:HGNC Symbol;Acc:HGNC:3390]	chr6:93949	NM_00128	XM_01701	.	.	NP_00127	XP_005248	protein_co	ENSG0000	2045	3390
EPHA8	0.05393	0.097405	0.142569	0.161649	0.061018	0.156738	EPH receptor A8 [Source:HGNC Symbol;Acc:HGNC:3391]	chr1:22890	NM_00100	XM_01154	.	.	NP_00100	XP_011539	protein_co	ENSG0000	2046	3391
EPHB1	0.014327	0.029278	0.042548	0.133	0.056493	0.030085	EPH receptor B1 [Source:HGNC Symbol;Acc:HGNC:3392]	chr3:13451	NM_00444	XM_01151	.	.	NP_00443	.	protein_co	ENSG0000	2047	3392
EPHB2	8.26991	8.83058	11.1738	8.59059	7.81243	7.57528	EPH receptor B2 [Source:HGNC Symbol;Acc:HGNC:3393]	chr1:23037	NM_00130	XM_00671	.	.	NP_00129	XP_006710	protein_co	ENSG0000	2048	3393
EPHB3	2.62297	2.26897	2.68018	3.24521	2.83788	2.54774	EPH receptor B3 [Source:HGNC Symbol;Acc:HGNC:3394]	chr3:18427	NM_00444	.	.	.	NP_00443	.	protein_co	ENSG0000	2049	3394
EPHB4	25.1051	27.3458	26.8403	31.7051	29.884	26.1862	EPH receptor B4 [Source:HGNC Symbol;Acc:HGNC:3395]	chr7:10040	NM_00444	.	.	.	NP_00443	XP_016867	protein_co	ENSG0000	2050	3395
EPHB6	4.37189	3.91527	4.91198	5.17018	5.21217	5.25267	EPH receptor B6 [Source:HGNC Symbol;Acc:HGNC:3396]	chr7:14255	NM_00128	XM_01151	.	.	NP_00126	XP_006715	protein_co	ENSG0000	2051	3396
EPHX1	26.3463	28.2001	22.3582	30.4918	26.6131	27.9245	epoxide hydrolase 1 [Source:HGNC Symbol;Acc:HGNC:3401]	chr1:22599	NM_00012	.	.	.	NP_00011	.	protein_co	ENSG0000	2052	3401
EPHX2	6.62143	6.1747	7.58189	5.47981	2.91791	3.0011	epoxide hydrolase 2 [Source:HGNC Symbol;Acc:HGNC:3402]	chr8:27348	NM_00125	.	.	.	NP_00124	.	protein_co	ENSG0000	2053	3402
EPHX3	0.050737	0	0	0	0	0.054856	epoxide hydrolase 3 [Source:HGNC Symbol;Acc:HGNC:23760]	chr19:1533	NM_00114	.	.	.	NP_00113	.	protein_co	ENSG0000	79852	23760
EPHX4	3.4219	2.72332	1.90892	2.42763	2.22708	2.25246	epoxide hydrolase 4 [Source:HGNC Symbol;Acc:HGNC:23758]	chr1:92495	NM_17356	.	.	.	NP_77583	.	protein_co	ENSG0000	253152	23758
EPM2A	1.58253	1.33963	1.78121	1.59886	1.55437	2.71837	epilepsy, progressive myoclonus type 2A, Lafora disease (laforin)	chr6:14594	NM_00101	XM_01153	.	.	NP_00101	XP_011534	protein_co	ENSG0000	7957	3413
EPM2AIP1	6.5034	6.22241	5.7972	4.73933	6.63113	5.41953	EPM2A interacting protein 1 [Source:HGNC Symbol;Acc:HGNC:19	chr3:37026	NM_01480	.	.	.	NP_05562	.	protein_co	ENSG0000	9852	19735
EPN1	41.8636	42.148	39.7849	27.6993	23.0259	22.3053	epsin 1 [Source:HGNC Symbol;Acc:HGNC:21604]	chr19:5618	NM_00113	XM_01702	.	.	NP_00112	XP_005258	protein_co	ENSG0000	29924	21604
EPN2	13.0113	15.5418	16.7091	15.8049	13.5855	16.2965	epsin 2 [Source:HGNC Symbol;Acc:HGNC:18639]	chr17:1914	NM_00110	.	.	.	NP_00109	.	protein_co	ENSG0000	22905	18639
EPN3	7.4897	8.02475	9.91153	8.08739	7.51024	6.58185	epsin 3 [Source:HGNC Symbol;Acc:HGNC:18235]	chr17:4860	NM_01795	.	.	.	NP_06042	.	protein_co	ENSG0000	55040	18235
EPO	0.307104	0.183416	0.159329	0.132672	0.141634	0.133926	erythropoietin [Source:HGNC Symbol;Acc:HGNC:3415]	chr7:10031	NM_00079	.	.	.	NP_00079	.	protein_co	ENSG0000	2056	3415
EPOR	8.91375	8.41116	9.08957	7.82066	8.75731	5.98806	erythropoietin receptor [Source:HGNC Symbol;Acc:HGNC:3416]	chr19:1148	NM_00012	.	.	.	NP_00011	.	protein_co	ENSG0000	2057	3416
EPPIN	0	0	0	0	0	0	epididymal peptidase inhibitor [Source:HGNC Symbol;Acc:HGNC:	chr20:4416	NM_02039	.	.	.	NP_06513	.	protein_co	ENSG0000	57119	15932
EPPIN-WFDC6	0	0	0	0	0	0	EPPIN-WFDC6 readthrough [Source:HGNC Symbol;Acc:HGNC:388	chr20:4416	NM_00119	.	.	.	NP_00118	.	protein_co	ENSG0000	100526773	38825
EPPK1	9.59356	11.4356	15.5921	9.22488	9.79392	9.78839	epiplakin 1 [Source:HGNC Symbol;Acc:HGNC:15577]	chr8:14493	NM_03130	XM_01701	.	.	NP_11259	XP_011515	protein_co	ENSG0000	83481	15577
EPRS	62.0719	62.0633	54.6542	54.5909	56.0432	82.3156	glutamyl-prolyl-tRNA synthetase [Source:HGNC Symbol;Acc:HGN	chr1:22014	NM_00444	.	.	.	NP_00443	.	protein_co	ENSG0000	2058	3418
EPS15	10.205	11.1795	10.7393	8.93624	11.3829	14.3795	epidermal growth factor receptor pathway substrate 15 [Source:	chr1:51819	NM_00198	XM_00527	.	.	NP_00197	XP_005270	protein_co	ENSG0000	2060	3419
EPS15L1	10.5307	11.0325	10.6612	12.18	10.9402	8.34901	epidermal growth factor receptor pathway substrate 15 like 1 [So	chr19:1646	NM_00125	.	.	.	NP_00124	XP_016882	protein_co	ENSG0000	58513	24634
EPS8	18.8386	18.752	16.7965	18.3603	17.2294	16.6315	epidermal growth factor receptor pathway substrate 8 [Source:H	chr12:1577	NM_00444	XM_01152	.	.	NP_00443	XP_005253	protein_co	ENSG0000	2059	3420
EPS8L1	8.72329	8.8799	10.9092	7.63833	5.91249	9.09547	EPS8 like 1 [Source:HGNC Symbol;Acc:HGNC:21295]	chr19:5558	NM_01772	XM_01152	.	.	NP_06019	XP_005259	protein_co	ENSG0000	54869	21295
EPS8L2	43.1473	44.3499	60.8405	40.1192	33.0047	30.2033	EPS8 like 2 [Source:HGNC Symbol;Acc:HGNC:21296]	chr11:7061	NM_02277	XM_01701	.	.	NP_07360	XP_016873	protein_co	ENSG0000	64787	21296
EPS8L3	0.032723	0.032549	0.102658	0.104548	0.07536	0.128494	EPS8 like 3 [Source:HGNC Symbol;Acc:HGNC:21297]	chr1:11029	NM_00131	XM_01700	.	.	NP_00130	XP_011540	protein_co	ENSG0000	79574	21297
EPSTI1	0.473337	0.350532	0.22731	0.262071	0.290563	0.564823	epithelial stromal interaction 1 (breast) [Source:HGNC Symbol;Ac	chr13:4346	NM_00100	XM_01702	.	.	NP_00100	XP_005266	protein_co	ENSG0000	94240	16465
EPT1	23.9244	25.0766	21.8862	23.8197	30.682	29.8849	ethanolaminephosphotransferase 1 [Source:HGNC Symbol;Acc:H	chr2:26531	NM_03350	.	.	.	NP_27704	.	protein_co	ENSG0000	85465	29361
EPX	0	0	0	0.062926	0	0	eosinophil peroxidase [Source:HGNC Symbol;Acc:HGNC:3423]	chr17:5627	NM_00050	.	.	.	NP_00049	.	protein_co	ENSG0000	8288	3423
EPYC	0	0	0	0	0	0	epiphycan [Source:HGNC Symbol;Acc:HGNC:3053]	chr12:9135	NM_00495	XM_01153	.	.	NP_00494	XP_011536	protein_co	ENSG0000	1833	3053

EQTN	0	0	0	0	0	0	equatorin [Source:HGNC Symbol;Acc:HGNC:1359]	chr9:27284	NM_00116	.	.	.	NP_00115	.	protein_co	ENSG0000	54586	1359
ERAL1	32.2193	32.565	30.4575	36.5979	31.5209	24.7092	Era like 12S mitochondrial rRNA chaperone 1 [Source:HGNC Symb	chr17:2708	NM_00131	.	.	.	NP_00130	.	protein_co	ENSG0000	26284	3424
ERAP1	8.17263	8.30075	4.9819	3.84314	5.27827	2.81332	endoplasmic reticulum aminopeptidase 1 [Source:HGNC Symbol;	chr5:95997	NM_00104	XM_01154	.	.	NP_00103	XP_005272	protein_co	ENSG0000	51752	18173
ERAP2	1.27408	1.39232	0.677842	1.01997	1.28884	0.616674	endoplasmic reticulum aminopeptidase 2 [Source:HGNC Symbol;	chr5:96211	NM_00113	XM_01154	.	.	NP_00112	XP_011541	protein_co	ENSG0000	64167	29499
ERAS	0.101781	0	0.104452	0.130154	0.118358	0	ES cell expressed Ras [Source:HGNC Symbol;Acc:HGNC:5174]	chrX:48684	NM_18153	.	.	.	NP_853510	.	protein_co	ENSG0000	3266	5174
ERBB2	21.3307	21.3362	20.8515	21.1834	19.6125	18.5807	erb-b2 receptor tyrosine kinase 2 [Source:HGNC Symbol;Acc:HGN	chr17:3782	NM_00100	.	.	.	NP_00100	.	protein_co	ENSG0000	2064	3430
ERBB3	11.3994	10.8954	11.1721	9.60564	8.00185	11.2884	erb-b2 receptor tyrosine kinase 3 [Source:HGNC Symbol;Acc:HGN	chr12:5647	NM_00100	.	.	.	NP_00100	.	protein_co	ENSG0000	2065	3431
ERBB4	0.006794	0.029311	0.047285	0	0	0.006563	erb-b2 receptor tyrosine kinase 4 [Source:HGNC Symbol;Acc:HGN	chr2:21224	NM_00104	XM_01700	.	.	NP_00103	XP_005246	protein_co	ENSG0000	2066	3432
ERBIN	13.959	13.8482	14.3781	13.2283	16.6152	20.0894	erbb2 interacting protein [Source:HGNC Symbol;Acc:HGNC:15842	chr5:65222	NM_00100	XM_01700	.	.	NP_00100	XP_005248	protein_co	ENSG0000	55914	15842
ERC1	7.00279	7.05832	7.68168	6.93703	7.09112	7.439	ELKS/RAB6-interacting/CAST family member 1 [Source:HGNC Sym	chr12:1100	NM_00130	XM_01701	.	.	NP_00128	XP_011519	protein_co	ENSG0000	23085	17072
ERC2	0	0	0	0	0	0	ELKS/RAB6-interacting/CAST family member 2 [Source:HGNC Sym	chr3:55542	NM_01557	XM_01153	.	.	NP_05639	XP_016861	protein_co	ENSG0000	26059	31922
ERCC1	37.3761	38.6728	51.6151	40.0683	34.0924	41.6139	ERCC excision repair 1, endonuclease non-catalytic subunit [Sour	chr19:4588	NM_00116	XM_01152	.	.	NP_00115	XP_005258	protein_co	ENSG0000	2067	3433
ERCC2	18.2815	19.1767	21.6655	19.4628	14.9829	17.8098	ERCC excision repair 2, TFIIH core complex helicase subunit [Sour	chr19:4584	NM_00040	XM_01702	.	.	NP_00039	XP_011524	protein_co	ENSG0000	2068	3434
ERCC3	28.2478	28.0807	25.6051	34.205	31.684	26.1684	ERCC excision repair 3, TFIIH core complex helicase subunit [Sour	chr2:12801	NM_00012	XM_01151	.	.	NP_00011	XP_011509	protein_co	ENSG0000	2071	3435
ERCC4	2.25427	2.8138	2.94265	1.83348	2.63589	3.14273	ERCC excision repair 4, endonuclease catalytic subunit [Source:H	chr16:1401	NM_00523	XM_01152	.	.	NP_00522	XP_011520	protein_co	ENSG0000	2072	3436
ERCC5	6.37676	6.2813	6.19222	5.23476	5.94912	5.19194	ERCC excision repair 5, endonuclease [Source:HGNC Symbol;Acc:	chr13:1034	NM_00012	.	.	.	NP_00011	.	protein_co	ENSG0000	2073	3437
ERCC6	3.3926	3.90686	3.96658	3.55664	4.14218	4.28597	ERCC excision repair 6, chromatin remodeling factor [Source:HGN	chr10:5066	NM_00012	.	.	.	NP_00011	.	protein_co	ENSG0000	2074	3438
ERCC6L	7.97114	8.39009	6.79622	9.88881	11.67	8.57551	ERCC excision repair 6 like, spindle assembly checkpoint helicase	chrX:71401	NM_01766	.	.	.	NP_06013	.	protein_co	ENSG0000	54821	20794
ERCC6L2	2.61569	2.54647	2.52046	2.10783	2.3105	2.63117	ERCC excision repair 6 like 2 [Source:HGNC Symbol;Acc:HGNC:26	chr9:98637	NM_00101	XM_01151	.	.	NP_00101	.	protein_co	ENSG0000	375748	26922
ERCC8	6.09446	5.91914	5.92586	7.19344	6.58618	7.50797	ERCC excision repair 8, CSA ubiquitin ligase complex subunit [Sou	chr5:60169	NM_00008	.	.	.	NP_00007	.	protein_co	ENSG0000	1161	3439
EREG	168.135	193.992	181.227	131.955	140.572	193.357	epiregulin [Source:HGNC Symbol;Acc:HGNC:3443]	chr4:75230	NM_00143	.	.	.	NP_00142	.	protein_co	ENSG0000	2069	3443
ERF	48.7021	49.4865	42.0295	50.3306	45.976	37.6548	ETS2 repressor factor [Source:HGNC Symbol;Acc:HGNC:3444]	chr19:4273	NM_00130	XM_01702	.	.	NP_00128	XP_005258	protein_co	ENSG0000	2077	3444
ERFE	2.22649	3.7351	2.56911	2.88447	1.17103	3.11515	erythroferrone [Source:HGNC Symbol;Acc:HGNC:26727]	chr2:23906	NM_00129	.	.	.	NP_00127	.	protein_co	ENSG0000	151176	26727
ERG	0	0	0	0	0	0	ERG, ETS transcription factor [Source:HGNC Symbol;Acc:HGNC:34	chr21:3975	NM_00113	XM_01152	NR_11194	.	NP_00112	XP_011527	protein_co	ENSG0000	2078	3446
ERGIC1	43.1813	43.9873	47.5013	44.4823	40.9963	43.3006	endoplasmic reticulum-golgi intermediate compartment 1 [Sourc	chr5:17226	NM_00103	XM_01153	.	.	NP_00102	XP_011532	protein_co	ENSG0000	57222	29205
ERGIC2	8.03721	8.48583	8.22818	8.49943	9.36107	10.9709	ERGIC and golgi 2 [Source:HGNC Symbol;Acc:HGNC:30208]	chr12:2949	NM_01657	.	.	.	NP_05765	.	protein_co	ENSG0000	51290	30208
ERGIC3	106.227	107.498	110.91	123.822	99.0139	107.539	ERGIC and golgi 3 [Source:HGNC Symbol;Acc:HGNC:15927]	chr20:3412	NM_01596	.	.	.	NP_05705	.	protein_co	ENSG0000	51614	15927
ERH	202.128	196.463	186.081	240.281	224.14	240.36	enhancer of rudimentary homolog (Drosophila) [Source:HGNC Sy	chr14:6984	NM_00445	.	.	.	NP_00444	.	protein_co	ENSG0000	2079	3447
ERI1	16.8753	16.2401	21.2586	18.506	22.9376	20.9649	exoribonuclease 1 [Source:HGNC Symbol;Acc:HGNC:23994]	chr8:88596	NM_15333	XM_01154	.	.	NP_69916	XP_005272	protein_co	ENSG0000	90459	23994
ERI2	12.0181	12.5434	12.9487	12.5792	15.899	13.6687	ERI1 exoribonuclease family member 2 [Source:HGNC Symbol;Ac	chr16:2077	NM_00114	.	.	.	NP_00113	.	protein_co	ENSG0000	112479	30541
ERI3	37.3549	37.4618	28.4851	39.4318	33.6433	27.9909	ERI1 exoribonuclease family member 3 [Source:HGNC Symbol;Ac	chr1:44686	NM_00130	XM_01700	.	.	NP_00128	XP_005271	protein_co	ENSG0000	79033	17276
ERICH1	9.02712	8.09083	8.82758	7.61738	7.53036	6.43958	glutamate rich 1 [Source:HGNC Symbol;Acc:HGNC:27234]	chr8:56474	NM_20733	XM_01153	.	.	NP_00129	XP_011533	protein_co	ENSG0000	157697	27234
ERICH2	0.578916	0.972534	1.3434	1.28462	0.395095	2.46677	glutamate rich 2 [Source:HGNC Symbol;Acc:HGNC:44395]	chr2:17164	NM_00128	.	.	.	NP_00127	.	protein_co	ENSG0000	285141	44395
ERICH3	0	0	0	0	0	0	glutamate rich 3 [Source:HGNC Symbol;Acc:HGNC:25346]	chr1:75033	NM_00100	XM_01154	.	.	NP_00100	XP_016855	protein_co	ENSG0000	127254	25346
ERICH4	0.066474	0	0	0.072295	0	0	glutamate rich 4 [Source:HGNC Symbol;Acc:HGNC:34497]	chr19:4194	NM_00113	.	.	.	NP_00112	.	protein_co	ENSG0000	100170765	34497
ERICH5	5.52875	6.48292	5.56795	9.10405	8.07376	6.80095	glutamate rich 5 [Source:HGNC Symbol;Acc:HGNC:26823]	chr8:99076	NM_00117	.	.	.	NP_00116	.	protein_co	ENSG0000	203111	26823
ERICH6	0	0.033304	0	0	0	0	glutamate rich 6 [Source:HGNC Symbol;Acc:HGNC:28602]	chr3:15037	NM_00130	XM_00524	.	.	NP_00129	XP_005247	protein_co	ENSG0000	131831	28602
ERICH6B	0	0	0	0	0	0	glutamate rich 6B [Source:HGNC Symbol;Acc:HGNC:26523]	chr13:4611	NM_18254	XM_01702	.	.	NP_87234	XP_011533	protein_co	ENSG0000	220081	26523
ERLEC1	17.7871	17.6661	16.7843	15.4618	17.6677	20.0271	endoplasmic reticulum lectin 1 [Source:HGNC Symbol;Acc:HGNC:	chr2:53897	NM_00112	XM_01153	.	.	NP_00112	XP_011531	protein_co	ENSG0000	27248	25222
ERLIN1	32.404	34.0832	28.2388	34.7372	34.2282	30.4921	ER lipid raft associated 1 [Source:HGNC Symbol;Acc:HGNC:16947	chr10:1019	NM_00110	XM_01153	.	.	NP_00109	XP_005269	protein_co	ENSG0000	10613	16947
ERLIN2	10.1802	10.9551	9.53566	9.46542	10.4042	10.4734	ER lipid raft associated 2 [Source:HGNC Symbol;Acc:HGNC:1356]	chr8:37594	NM_00100	XM_00671	.	.	NP_00100	XP_005273	protein_co	ENSG0000	11160	1356
ERMAP	2.2759	1.52829	1.55389	1.26647	1.13366	1.9629	erythroblast membrane associated protein (Scianna blood group)	chr1:43272	NM_00101	XM_01154	.	.	NP_00101	XP_006710	protein_co	ENSG0000	114625	15743
ERMARD	9.90649	9.01807	10.3021	8.01215	9.23837	8.19625	ER membrane associated RNA degradation [Source:HGNC Symbo	chr6:17015	NM_00127	XM_01153	.	.	NP_00126	XP_011534	protein_co	ENSG0000	55780	21056
ERMN	0	0	0	0	0	0	ermin [Source:HGNC Symbol;Acc:HGNC:29208]	chr2:15817	NM_00100	.	.	.	NP_00100	.	protein_co	ENSG0000	57471	29208
ERMP1	13.9164	14.5315	15.9834	13.322	16.1503	21.5296	endoplasmic reticulum metallopeptidase 1 [Source:HGNC Symbo	chr9:57845	NM_02489	XM_01701	.	.	NP_07917	XP_005251	protein_co	ENSG0000	79956	23703
ERN1	5.67199	6.53839	4.89016	5.54985	5.57999	7.89661	endoplasmic reticulum to nucleus signaling 1 [Source:HGNC Symb	chr17:6211	NM_00143	.	.	.	NP_00142	XP_016879	protein_co	ENSG0000	2081	3449
ERN2	0.13833	0.091082	0.019858	0	0.053581	0.127593	endoplasmic reticulum to nucleus signaling 2 [Source:HGNC Symb	chr16:2369	NM_00130	XM_01154	.	.	NP_00129	XP_005255	protein_co	ENSG0000	10595	16942
ERO1A	28.4364	28.7757	25.0281	23.7361	28.7354	32.0175	endoplasmic reticulum oxidoreductase 1 alpha [Source:HGNC Sy	chr14:5310	NM_01458	XM_01153	.	.	NP_05539	XP_011534	protein_co	ENSG0000	30001	13280
ERO1B	3.4491	3.07527	2.20623	2.22136	3.06082	3.90019	endoplasmic reticulum oxidoreductase 1 beta [Source:HGNC Sym	chr1:23637	NM_01989	.	.	.	NP_06394	.	protein_co	ENSG0000	56605	14355
ERP27	0.064936	0	0	0.081015	0	0.072952	endoplasmic reticulum protein 27 [Source:HGNC Symbol;Acc:HG	chr12:1506	NM_00130	.	.	.	NP_00128	.	protein_co	ENSG0000	121506	26495
ERP29	126.851	122.505	95.661	130.692	104.679	143.592	endoplasmic reticulum protein 29 [Source:HGNC Symbol;Acc:HG	chr12:1124	NM_00103	.	.	.	NP_00102	XP_016874	protein_co	ENSG0000	10961	13799
ERP44	9.01357	8.7785	6.8359	8.26389	9.27304	8.24219	endoplasmic reticulum protein 44 [Source:HGNC Symbol;Acc:HG	chr9:10274	NM_01505	.	.	.	NP_05586	.	protein_co	ENSG0000	23071	18311
ERRFI1	236.514	238.985	302.979	244.447	280.601	414.988	ERBB receptor feedback inhibitor 1 [Source:HGNC Symbol;Acc:H	chr1:80644	NM_01894	XM_00671	.	.	NP_06182	XP_005263	protein_co	ENSG0000	54206	18185
ERV3-1	2.72384	2.79962	3.0137	2.46691	2.5739	3.28251	endogenous retrovirus group 3 member 1 [Source:HGNC Symbol;	chr7:64432	NM_00100	.	.	.	NP_00100	.	protein_co	ENSG0000	2086	3454
ERVFRD-1	0	0	0	0	0	0	endogenous retrovirus group FRD member 1 [Source:HGNC Symb	chr6:11094	NM_20758	.	.	.	NP_99746	.	protein_co	ENSG0000	405754	33823
ERVK3-1	28.9041	28.3614	21.5784	31.801	27.5807	18.8813	endogenous retrovirus group K3 member 1 [Source:HGNC Symbo	chr19:5881	.	.	.	.	.	.	protein_co	ENSG0000	.	30466
ERVMER34-1	15.1464	14.261	14.8811	15.1763	19.0751	13.8034	endogenous retrovirus group MER34 member 1 [Source:HGNC Sy	chr4:53608	NM_00124	.	.	.	NP_00122	.	protein_co	ENSG0000	100288413	42970
ERVV-1	0	0	0	0	0	0	endogenous retrovirus group V member 1 [Source:HGNC Symbol;	chr19:5351	NM_15247	.	.	.	NP_68968	.	protein_co	ENSG0000	147664	26501
ERVV-2	0	0	0	0	0	0	endogenous retrovirus group V member 2 [Source:HGNC Symbol;	chr19:5354	NM_00119	.	.	.	NP_00117	.	protein_co	ENSG0000	100271846	39051

ERVW-1	0	0	0	0	0	0	endogenous retrovirus group W member 1 [Source:HGNC Symbo	chr7:92097	NM_00113	.	.	.	NP_00112	.	protein_co	ENSG0000	30816	13525
ESAM	4.36616	3.43235	3.30093	4.42097	2.82339	3.04824	endothelial cell adhesion molecule [Source:HGNC Symbol;Acc:HG	chr11:1246	NM_13896	XM_01154	.	.	NP_62041	XP_011541	protein_co	ENSG0000	90952	17474
ESCO1	7.94035	7.56431	8.52494	6.45379	8.30301	12.5765	establishment of sister chromatid cohesion N-acetyltransferase 1	chr18:1910	NM_05291	XM_01152	.	.	NP_44314	XP_011524	protein_co	ENSG0000	114799	24645
ESCO2	11.0648	10.0763	9.63727	16.622	15.993	12.7936	establishment of sister chromatid cohesion N-acetyltransferase 2	chr8:27631	NM_00101	XM_01154	.	.	NP_00101	XP_011542	protein_co	ENSG0000	157570	27230
ESD	109.38	104.029	102.535	91.56	92.1868	101.363	esterase D [Source:HGNC Symbol;Acc:HGNC:3465]	chr13:4734	NM_00198	XM_01153	.	.	NP_00197	XP_005266	protein_co	ENSG0000	2098	3465
ESF1	15.9242	14.1004	12.7189	14.8198	16.8816	11.4981	ESF1 nucleolar pre-rRNA processing protein homolog [Source:HG	chr20:1369	NM_00127	.	.	.	NP_00126	XP_016883	protein_co	ENSG0000	51575	15898
ESM1	0	0	0	0	0	0	endothelial cell specific molecule 1 [Source:HGNC Symbol;Acc:HG	chr5:54273	NM_00113	.	.	.	NP_00112	.	protein_co	ENSG0000	11082	3466
ESPL1	13.3027	13.3803	10.4327	16.9994	16.147	12.9942	extra spindle pole bodies like 1, separase [Source:HGNC Symbol;A	chr12:5366	NM_01229	XM_01702	.	.	NP_03642	XP_006719	protein_co	ENSG0000	9700	16856
ESPN	23.4991	25.1945	30.6775	21.4281	18.6863	19.5032	espin [Source:HGNC Symbol;Acc:HGNC:13281]	chr1:64848	NM_03147	XM_01154	.	.	NP_11366	XP_005263	protein_co	ENSG0000	83715	13281
ESPNL	0.069461	0.056712	0.164527	0.177255	0.139824	0.188098	espin-like [Source:HGNC Symbol;Acc:HGNC:27937]	chr2:23900	NM_00130	XM_00671	.	.	NP_00129	XP_005246	protein_co	ENSG0000	339768	27937
ESR1	0	0.013714	0.033987	0	0	0	estrogen receptor 1 [Source:HGNC Symbol;Acc:HGNC:3467]	chr6:15201	NM_00012	XM_01153	.	.	NP_00011	XP_006715	protein_co	ENSG0000	2099	3467
ESR2	0.014036	0.053449	0	0.078171	0.037174	0.01923	estrogen receptor 2 [Source:HGNC Symbol;Acc:HGNC:3468]	chr14:6431	NM_00104	XM_01153	.	.	NP_00103	XP_011534	protein_co	ENSG0000	2100	3468
ESRP1	45.2306	47.2137	41.0812	52.2213	54.5559	74.2289	epithelial splicing regulatory protein 1 [Source:HGNC Symbol;Acc	chr8:95653	NM_00103	XM_00525	.	.	NP_00103	XP_005251	protein_co	ENSG0000	54845	25966
ESRP2	19.1062	18.9106	18.0534	20.8346	17.5645	14.8199	epithelial splicing regulatory protein 2 [Source:HGNC Symbol;Acc	chr16:6811	NM_02493	XM_01152	NR_10683	.	NP_07921	XP_005256	protein_co	ENSG0000	102466194	26152
ESRRA	38.2801	36.3022	35.5747	33.6922	32.5178	27.8718	estrogen related receptor alpha [Source:HGNC Symbol;Acc:HGNC	chr11:6407	NM_00128	.	.	.	NP_00126	XP_016872	protein_co	ENSG0000	2101	3471
ESRRB	0.171785	0.522664	0.593717	0.266741	0.253715	0.442455	estrogen related receptor beta [Source:HGNC Symbol;Acc:HGNC:	chr14:7687	NM_00445	XM_01153	.	.	NP_00444	XP_011534	protein_co	ENSG0000	2103	3473
ESRRG	0	0	0	0	0	0	estrogen related receptor gamma [Source:HGNC Symbol;Acc:HGN	chr1:21667	NM_00113	XM_01700	.	.	NP_00112	XP_006711	protein_co	ENSG0000	2104	3474
ESX1	0	0	0	0	0	0	ESX homeobox 1 [Source:HGNC Symbol;Acc:HGNC:14865]	chrX:10349	NM_15344	.	.	.	NP_70314	.	protein_co	ENSG0000	80712	14865
ESYT1	33.8107	35.0612	30.0557	34.2103	32.0391	36.108	extended synaptotagmin 1 [Source:HGNC Symbol;Acc:HGNC:295	chr12:5652	NM_00118	.	.	.	NP_00117	.	protein_co	ENSG0000	23344	29534
ESYT2	22.7806	23.4937	23.3701	20.6437	20.9991	21.9	extended synaptotagmin 2 [Source:HGNC Symbol;Acc:HGNC:222	chr7:15852	NM_02072	.	.	.	NP_06577	.	protein_co	ENSG0000	57488	22211
ESYT3	0	0	0	0	0	0	extended synaptotagmin 3 [Source:HGNC Symbol;Acc:HGNC:242	chr3:13815	NM_03191	XM_01151	.	.	NP_00130	XP_011511	protein_co	ENSG0000	83850	24295
ETAA1	4.2266	3.83651	4.8231	3.8189	5.75152	6.48409	Ewing tumor associated antigen 1 [Source:HGNC Symbol;Acc:HGN	chr2:67624	NM_01900	XM_00526	.	.	NP_06187	.	protein_co	ENSG0000	54465	24648
ETF1	90.2163	86.6567	80.6552	103.292	110.675	102.45	eukaryotic translation termination factor 1 [Source:HGNC Symbo	chr5:13784	NM_00125	XM_00527	.	.	NP_00124	XP_005271	protein_co	ENSG0000	2107	3477
ETFA	56.5554	51.2832	57.6103	66.4705	60.3784	71.8174	electron transfer flavoprotein alpha subunit [Source:HGNC Symb	chr15:7650	NM_00012	.	.	.	NP_00011	.	protein_co	ENSG0000	2108	3481
ETFB	62.2459	63.5194	67.2851	76.6175	63.3869	54.7266	electron transfer flavoprotein beta subunit [Source:HGNC Symbo	chr19:5184	NM_00101	.	.	.	NP_00101	.	protein_co	ENSG0000	2109	3482
ETFBKMT	0.673988	0.641098	0.90964	0.818925	0.228765	1.06445	electron transfer flavoprotein beta subunit lysine methyltransfer	chr12:3180	NM_00113	XM_00671	.	.	NP_00112	XP_006719	protein_co	ENSG0000	254013	28739
ETFDH	6.32933	6.68546	7.1627	6.32005	6.80561	10.7844	electron transfer flavoprotein dehydrogenase [Source:HGNC Sym	chr4:15959	NM_00128	.	.	.	NP_00126	.	protein_co	ENSG0000	2110	3483
ETHE1	42.459	44.0038	48.1901	51.3211	38.5498	42.5168	ETHE1, persulfide dioxygenase [Source:HGNC Symbol;Acc:HGNC:	chr19:4401	NM_01429	XM_00525	.	.	NP_00130	XP_005258	protein_co	ENSG0000	23474	23287
ETNK1	16.3812	17.2335	21.6129	15.9125	17.5268	19.476	ethanolamine kinase 1 [Source:HGNC Symbol;Acc:HGNC:24649]	chr12:2277	NM_00103	.	.	.	NP_00103	.	protein_co	ENSG0000	55500	24649
ETNK2	8.9725	9.20394	10.2859	12.4826	10.3835	10.5041	ethanolamine kinase 2 [Source:HGNC Symbol;Acc:HGNC:25575]	chr1:20410	NM_00129	XM_01150	.	.	NP_00128	XP_011508	protein_co	ENSG0000	55224	25575
ETNPPL	0	0	0	0	0	0	ethanolamine-phosphate phospho-lyase [Source:HGNC Symbol;A	chr4:10966	NM_00114	XM_00671	.	.	NP_00114	XP_006714	protein_co	ENSG0000	64850	14404
ETS1	6.87618	7.57758	4.45746	5.19448	5.6849	6.91628	ETS proto-oncogene 1, transcription factor [Source:HGNC Symbo	chr11:1283	NM_00114	XM_01701	.	.	NP_00113	XP_011540	protein_co	ENSG0000	2113	3488
ETS2	19.5619	20.6931	21.7024	31.3037	31.3253	28.8208	ETS proto-oncogene 2, transcription factor [Source:HGNC Symbo	chr21:4017	NM_00125	XM_00526	.	.	NP_00124	XP_005260	protein_co	ENSG0000	2114	3489
ETV1	6.01463	6.21573	7.95896	6.4085	7.23007	9.32203	ETS variant 1 [Source:HGNC Symbol;Acc:HGNC:3490]	chr7:13930	NM_00116	XM_01151	.	.	NP_00115	XP_005249	protein_co	ENSG0000	2115	3490
ETV2	1.49109	2.32719	1.17705	1.20207	1.21481	0.770409	ETS variant 2 [Source:HGNC Symbol;Acc:HGNC:3491]	chr19:3613	NM_00130	XM_01152	.	.	NP_00128	XP_005258	protein_co	ENSG0000	2116	3491
ETV3	11.7598	13.109	11.2572	12.2296	12.6249	11.4648	ETS variant 3 [Source:HGNC Symbol;Acc:HGNC:3492]	chr1:15709	NM_00114	XM_00671	.	.	NP_00113	XP_006711	protein_co	ENSG0000	2117	3492
ETV3L	0	0	0	0	0	0	ETS variant 3 like [Source:HGNC Symbol;Acc:HGNC:33834]	chr1:15706	NM_00100	.	.	.	NP_00100	.	protein_co	ENSG0000	440695	33834
ETV4	26.7267	25.1247	30.9754	34.0235	29.3846	33.3647	ETS variant 4 [Source:HGNC Symbol;Acc:HGNC:3493]	chr17:4160	NM_00107	XM_01152	.	.	NP_00107	XP_011522	protein_co	ENSG0000	2118	3493
ETV5	26.5579	30.9523	26.8779	25.7285	24.8633	50.0183	ETS variant 5 [Source:HGNC Symbol;Acc:HGNC:3494]	chr3:18576	NM_00445	.	.	.	NP_00444	.	protein_co	ENSG0000	2119	3494
ETV6	4.72239	4.75901	5.7113	2.59281	2.65061	4.36845	ETS variant 6 [Source:HGNC Symbol;Acc:HGNC:3495]	chr12:1180	NM_00198	XM_01152	.	.	NP_00197	XP_011518	protein_co	ENSG0000	2120	3495
ETV7	1.73149	1.82784	1.89095	0.539204	0.589919	0.297571	ETS variant 7 [Source:HGNC Symbol;Acc:HGNC:18160]	chr6:36321	NM_00120	XM_01151	.	.	NP_00119	XP_011512	protein_co	ENSG0000	51513	18160
EVA1A	1.66475	1.54161	1.5141	1.72107	1.72378	1.25761	eva-1 homolog A, regulator of programmed cell death [Source:H	chr2:75719	NM_00113	.	.	.	NP_00112	.	protein_co	ENSG0000	84141	25816
EVA1B	12.9224	15.9112	14.7141	14.9556	9.61975	16.0042	eva-1 homolog B [Source:HGNC Symbol;Acc:HGNC:25558]	chr1:36787	NM_00130	.	.	.	NP_00129	XP_016857	protein_co	ENSG0000	55194	25558
EVA1C	9.46644	10.4309	10.5421	9.77859	6.16716	6.92501	eva-1 homolog C [Source:HGNC Symbol;Acc:HGNC:13239]	chr21:3378	NM_00128	XM_01702	.	.	NP_00127	XP_006724	protein_co	ENSG0000	59271	13239
EVC	0.010835	0.01084	0.009401	0.011736	0	0.040716	EvC ciliary complex subunit 1 [Source:HGNC Symbol;Acc:HGNC:34	chr4:57129	NM_00130	XM_00671	.	.	NP_00129	XP_005248	protein_co	ENSG0000	2121	3497
EVC2	0	0	0	0	0	0	EvC ciliary complex subunit 2 [Source:HGNC Symbol;Acc:HGNC:1	chr4:55641	NM_00116	XM_01151	.	.	NP_00115	XP_016863	protein_co	ENSG0000	132884	19747
EVI2A	0	0	0	0	0	0	ecotropic viral integration site 2A [Source:HGNC Symbol;Acc:HGN	chr17:2942	NM_00100	.	.	.	NP_00100	.	protein_co	ENSG0000	2123	3499
EVI2B	0	0	0	0	0	0	ecotropic viral integration site 2B [Source:HGNC Symbol;Acc:HGN	chr17:2942	NM_00649	XM_00525	.	.	NP_00648	XP_005258	protein_co	ENSG0000	2124	3500
EVI5	2.79108	2.9733	3.72188	2.71845	3.2318	3.6129	ecotropic viral integration site 5 [Source:HGNC Symbol;Acc:HGNC	chr1:92974	NM_00130	XM_01154	.	.	NP_00129	XP_011540	protein_co	ENSG0000	7813	3501
EVI5L	6.30832	6.62206	6.87103	6.36931	6.31303	9.09961	ecotropic viral integration site 5 like [Source:HGNC Symbol;Acc:H	chr19:7895	NM_00115	XM_00527	.	.	NP_00115	XP_005272	protein_co	ENSG0000	115704	30464
EVL	3.59649	3.41693	4.08318	3.9105	4.0257	3.70236	Enah/Vasp-like [Source:HGNC Symbol;Acc:HGNC:20234]	chr14:1004	NM_01633	XM_01702	.	.	NP_05742	XP_005267	protein_co	ENSG0000	51466	20234
EVPL	10.3838	9.76333	15.3635	10.4367	10.3633	16.1253	envoplakin [Source:HGNC Symbol;Acc:HGNC:3503]	chr17:7400	NM_00132	XM_01152	.	.	NP_00130	XP_011522	protein_co	ENSG0000	2125	3503
EVPLL	0	0	0	0	0	0	envoplakin like [Source:HGNC Symbol;Acc:HGNC:35236]	chr17:1828	NM_00114	XM_01152	.	.	NP_00113	XP_011522	protein_co	ENSG0000	645027	35236
EVX1	0.077161	0.037679	0.131411	0.102311	0.044911	0.254994	even-skipped homeobox 1 [Source:HGNC Symbol;Acc:HGNC:350	chr7:27282	NM_00130	.	.	.	NP_00129	.	protein_co	ENSG0000	2128	3506
EVX2	0	0	0	0.032134	0	0.029961	even-skipped homeobox 2 [Source:HGNC Symbol;Acc:HGNC:350	chr2:17694	NM_00108	XM_01151	.	.	NP_00107	XP_011509	protein_co	ENSG0000	344191	3507
EWSR1	133.331	140.775	107.162	154.856	136.904	107.598	EWS RNA binding protein 1 [Source:HGNC Symbol;Acc:HGNC:350	chr22:2966	NM_00116	XM_01153	.	.	NP_00115	XP_005261	protein_co	ENSG0000	2130	3508
EXD1	0.057384	0	0	0.064805	0.029035	0.025927	exonuclease 3'-5' domain containing 1 [Source:HGNC Symbol;Acc	chr15:4147	NM_00128	XM_01702	.	.	NP_00127	XP_006720	protein_co	ENSG0000	161829	28507
EXD2	12.3535	12.1465	12.6312	9.69866	9.57153	9.94389	exonuclease 3'-5' domain containing 2 [Source:HGNC Symbol;Acc	chr14:6965	NM_00119	XM_01153	.	.	NP_00118	XP_005267	protein_co	ENSG0000	55218	20217
EXD3	5.50852	5.48098	8.36863	4.22427	3.03145	6.61218	exonuclease 3'-5' domain containing 3 [Source:HGNC Symbol;Acc	chr9:14020	NM_00128	XM_01151	.	.	NP_00127	XP_011517	protein_co	ENSG0000	54932	26023

EXO1	15.7505	15.2757	12.0413	16.2969	16.6164	14.3697	exonuclease 1 [Source:HGNC Symbol;Acc:HGNC:3511]	chr1:24201	NM_00131	XM_01154	.	.	NP_00130	XP_006711	protein_co	ENSG0000	9156	3511
EXO5	0.776243	0.720463	0.542555	1.20083	1.2661	1.71587	exonuclease 5 [Source:HGNC Symbol;Acc:HGNC:26115]	chr1:40974	NM_02277	XM_01700	.	.	NP_07361	XP_005271	protein_co	ENSG0000	64789	26115
EXOC1	8.12523	7.87911	9.01784	7.69345	9.57837	9.40134	exocyst complex component 1 [Source:HGNC Symbol;Acc:HGNC:3	chr4:56719	NM_00102	XM_01153	.	.	NP_00102	XP_005265	protein_co	ENSG0000	55763	30380
EXOC2	9.96443	9.65218	11.2846	11.0858	11.3378	13.0791	exocyst complex component 2 [Source:HGNC Symbol;Acc:HGNC:2	chr6:48513	NM_01830	XM_01701	.	.	NP_06077	XP_016866	protein_co	ENSG0000	55770	24968
EXOC3	14.7389	14.6629	16.5237	14.3692	11.6413	11.4645	exocyst complex component 3 [Source:HGNC Symbol;Acc:HGNC:3	chr5:44327	NM_00727	XM_01151	.	.	NP_00920	XP_011512	protein_co	ENSG0000	11336	30378
EXOC3L1	0	0.034627	0	0.075355	0	0.039457	exocyst complex component 3 like 1 [Source:HGNC Symbol;Acc:H	chr16:6721	NM_17851	.	.	.	NP_84861	.	protein_co	ENSG0000	283849	27540
EXOC3L2	0	0	0	0	0	0	Exocyst complex component 3-like protein 2 [Source:UniProtKB/	chr19:4571	NM_13856	.	.	.	.	.	protein_co	ENSG0000	.	.
EXOC3L4	0	0.08999	0	0	0	0	exocyst complex component 3 like 4 [Source:HGNC Symbol;Acc:H	chr14:1035	NM_00107	XM_01153	.	.	NP_00107	XP_011535	protein_co	ENSG0000	91828	20120
EXOC4	7.58537	7.70936	8.81062	7.04811	7.06035	8.59169	exocyst complex component 4 [Source:HGNC Symbol;Acc:HGNC:3	chr7:13293	NM_00103	.	.	.	NP_00103	XP_005250	protein_co	ENSG0000	60412	30389
EXOC5	13.0682	12.4754	16.0123	11.3295	18.2144	19.7556	exocyst complex component 5 [Source:HGNC Symbol;Acc:HGNC:1	chr14:5766	NM_00654	XM_00526	.	.	NP_00653	XP_005267	protein_co	ENSG0000	10640	10696
EXOC6	4.86547	4.81714	6.47848	5.81666	6.78224	8.2273	exocyst complex component 6 [Source:HGNC Symbol;Acc:HGNC:2	chr10:9459	NM_00101	XM_01701	.	.	NP_00101	XP_016871	protein_co	ENSG0000	54536	23196
EXOC6B	8.11727	8.46765	10.3635	8.15789	9.39802	10.1733	exocyst complex component 6B [Source:HGNC Symbol;Acc:HGNC	chr2:72403	NM_00132	XM_01700	.	.	NP_00130	XP_005264	protein_co	ENSG0000	23233	17085
EXOC7	36.7605	36.6417	39.7825	31.1667	27.9343	25.2824	exocyst complex component 7 [Source:HGNC Symbol;Acc:HGNC:2	chr17:7407	NM_00101	XM_00672	.	.	NP_00101	XP_006721	protein_co	ENSG0000	23265	23214
EXOC8	6.4627	6.50117	6.36112	6.24887	6.71135	6.53225	exocyst complex component 8 [Source:HGNC Symbol;Acc:HGNC:2	chr1:23146	NM_17587	.	.	.	NP_78707	.	protein_co	ENSG0000	149371	24659
EXOG	4.0887	9.84349	5.55394	8.83518	10.0077	2.98847	endo/exonuclease (5'-3'), endonuclease G-like [Source:HGNC Sym	chr3:38537	NM_00114	XM_00526	.	.	NP_00113	XP_005265	protein_co	ENSG0000	9941	3347
EXOSC1	43.9292	39.3205	32.5125	41.6987	32.9347	34.3591	exosome component 1 [Source:HGNC Symbol;Acc:HGNC:17286]	chr10:9919	NM_01604	XM_01153	.	.	NP_00130	XP_011538	protein_co	ENSG0000	51013	17286
EXOSC10	39.4188	37.0735	32.8621	41.967	41.541	35.1051	exosome component 10 [Source:HGNC Symbol;Acc:HGNC:9138]	chr1:11126	NM_00100	XM_00526	.	.	NP_00100	XP_005263	protein_co	ENSG0000	5394	9138
EXOSC2	32.7337	32.9408	29.1236	38.9878	36.3911	26.5062	exosome component 2 [Source:HGNC Symbol;Acc:HGNC:17097]	chr9:13356	NM_00128	XM_00671	.	.	NP_00126	XP_005272	protein_co	ENSG0000	23404	17097
EXOSC3	38.4777	37.069	39.5322	49.2881	51.0865	31.8344	exosome component 3 [Source:HGNC Symbol;Acc:HGNC:17944]	chr9:37779	NM_00100	.	.	.	NP_00100	.	protein_co	ENSG0000	51010	17944
EXOSC4	76.9962	76.5154	73.434	101.383	75.5525	60.2192	exosome component 4 [Source:HGNC Symbol;Acc:HGNC:18189]	chr8:14513	NM_01903	XM_01151	NR_10690	.	NP_06191	.	protein_co	ENSG0000	102465510	18189
EXOSC5	47.4037	41.6377	37.2454	44.5258	35.9299	29.7831	exosome component 5 [Source:HGNC Symbol;Acc:HGNC:24662]	chr19:4183	NM_02015	.	.	.	NP_06454	.	protein_co	ENSG0000	56915	24662
EXOSC6	12.2444	13.5185	11.3648	16.136	13.9885	13.952	exosome component 6 [Source:HGNC Symbol;Acc:HGNC:19055]	chr16:7028	NM_05821	.	.	.	NP_47812	.	protein_co	ENSG0000	118460	19055
EXOSC7	40.7073	37.8513	34.109	49.1322	41.3792	37.3314	exosome component 7 [Source:HGNC Symbol;Acc:HGNC:28112]	chr3:45017	NM_01500	.	.	XR_00174	NP_05581	.	protein_co	ENSG0000	23016	28112
EXOSC8	48.485	45.9154	33.1665	40.7967	37.1416	51.929	exosome component 8 [Source:HGNC Symbol;Acc:HGNC:17035]	chr13:3757	NM_18150	XM_00671	.	.	NP_85248	XP_006719	protein_co	ENSG0000	11340	17035
EXOSC9	55.3707	51.5522	42.2976	57.4696	55.288	44.7071	exosome component 9 [Source:HGNC Symbol;Acc:HGNC:9137]	chr4:12272	NM_00103	.	.	.	NP_00102	.	protein_co	ENSG0000	5393	9137
EXPH5	3.09768	3.5279	3.25802	3.86422	5.03497	4.51067	exophilin 5 [Source:HGNC Symbol;Acc:HGNC:30578]	chr11:1083	NM_00114	XM_01701	.	.	NP_00113	XP_005271	protein_co	ENSG0000	23086	30578
EXT1	4.1314	4.69962	3.77271	3.21458	3.30456	3.90499	exostosin glycosyltransferase 1 [Source:HGNC Symbol;Acc:HGNC:	chr8:11880	NM_00012	.	.	.	NP_00011	.	protein_co	ENSG0000	2131	3512
EXT2	27.3165	25.7465	26.5168	28.7946	26.2775	25.9955	exostosin glycosyltransferase 2 [Source:HGNC Symbol;Acc:HGNC:	chr11:4411	NM_00040	XM_01151	.	.	NP_00039	XP_011518	protein_co	ENSG0000	2132	3513
EXTL1	0.02327	0.045595	0.079489	0.024684	0	0	exostosin like glycosyltransferase 1 [Source:HGNC Symbol;Acc:HG	chr1:26348	NM_00445	XM_00524	.	.	NP_00444	XP_005245	protein_co	ENSG0000	2134	3515
EXTL2	11.9504	10.3669	12.3243	9.74621	11.5878	15.623	exostosin like glycosyltransferase 2 [Source:HGNC Symbol;Acc:HG	chr1:10133	NM_00103	XM_01700	.	.	NP_00102	XP_005270	protein_co	ENSG0000	2135	3516
EXTL3	25.7273	25.4723	27.4095	31.4792	30.9436	30.3555	exostosin like glycosyltransferase 3 [Source:HGNC Symbol;Acc:HG	chr8:28480	NM_00144	XM_01154	.	.	NP_00143	XP_011542	protein_co	ENSG0000	2137	3518
EYA1	0	0	0.037504	0	0	0	EYA transcriptional coactivator and phosphatase 1 [Source:HGNC	chr8:72109	NM_00050	XM_01701	.	.	NP_00049	XP_011515	protein_co	ENSG0000	2138	3519
EYA2	0	0	0	0	0	0	EYA transcriptional coactivator and phosphatase 2 [Source:HGNC	chr20:4552	NM_00524	XM_00526	.	.	NP_00523	XP_005260	protein_co	ENSG0000	2139	3520
EYA3	12.0704	11.6762	13.0892	10.0683	11.3022	9.58724	EYA transcriptional coactivator and phosphatase 3 [Source:HGNC	chr1:28296	NM_00128	XM_01154	NR_10421	.	NP_00126	XP_006710	protein_co	ENSG0000	2140	3521
EYA4	0.025235	0	0.018932	0	0	0.036964	EYA transcriptional coactivator and phosphatase 4 [Source:HGNC	chr6:13356	NM_00130	XM_01153	.	.	NP_00128	XP_005266	protein_co	ENSG0000	2070	3522
EYS	0	0	0	0	0	0	eyes shut homolog (Drosophila) [Source:HGNC Symbol;Acc:HGNC	chr6:64429	NM_00114	.	.	.	NP_00113	.	protein_co	ENSG0000	346007	21555
EZH1	6.8372	7.94818	7.98968	3.99589	4.1748	5.08624	enhancer of zeste 1 polycomb repressive complex 2 subunit [Sou	chr17:4085	NM_00132	XM_01702	.	.	NP_00130	XP_005257	protein_co	ENSG0000	2145	3526
EZH2	26.7791	26.8169	25.9091	33.677	37.4758	36.0466	enhancer of zeste 2 polycomb repressive complex 2 subunit [Sou	chr7:14850	NM_00120	XM_01151	.	.	NP_00119	XP_005250	protein_co	ENSG0000	2146	3527
EZR	355.88	358.693	455.274	333.378	316.7	396.512	ezrin [Source:HGNC Symbol;Acc:HGNC:12691]	chr6:15918	NM_00111	.	.	.	NP_00110	.	protein_co	ENSG0000	7430	12691
F10	0.821472	0.626206	1.48023	0.980017	1.44541	2.30696	coagulation factor X [Source:HGNC Symbol;Acc:HGNC:3528]	chr13:1137	NM_00050	XM_00526	.	.	NP_00049	XP_005268	protein_co	ENSG0000	2159	3528
F11	0	0	0	0	0	0	coagulation factor XI [Source:HGNC Symbol;Acc:HGNC:3529]	chr4:18718	NM_00012	XM_00671	.	.	NP_00011	XP_005262	protein_co	ENSG0000	2160	3529
F11R	44.8052	44.1884	43.5836	41.679	38.8498	47.312	F11 receptor [Source:HGNC Symbol;Acc:HGNC:14685]	chr1:16096	NM_01694	.	.	.	NP_05864	.	protein_co	ENSG0000	50848	14685
F12	9.02959	8.29374	8.7194	12.7013	9.6659	6.69083	coagulation factor XII [Source:HGNC Symbol;Acc:HGNC:3530]	chr5:17682	NM_00050	XM_01153	.	.	NP_00049	XP_011532	protein_co	ENSG0000	2161	3530
F13A1	0	0	0	0	0	0	coagulation factor XIII A chain [Source:HGNC Symbol;Acc:HGNC:3	chr6:61443	NM_00012	XM_01151	.	.	NP_00012	XP_006715	protein_co	ENSG0000	2162	3531
F13B	0.029665	0.03199	0	0	0	0	coagulation factor XIII B chain [Source:HGNC Symbol;Acc:HGNC:3	chr1:19700	NM_00199	XM_01150	.	.	NP_00198	XP_011507	protein_co	ENSG0000	2165	3534
F2	0.109645	0.065483	0.094963	0.157259	0.084864	0	coagulation factor II, thrombin [Source:HGNC Symbol;Acc:HGNC:3	chr11:4674	NM_00050	.	.	.	NP_00049	.	protein_co	ENSG0000	2147	3535
F2R	13.0663	12.1311	17.4705	17.951	20.3257	20.1668	coagulation factor II thrombin receptor [Source:HGNC Symbol;Ac	chr5:76011	NM_00199	.	.	.	NP_00198	.	protein_co	ENSG0000	2149	3537
F2RL1	65.7066	68.133	65.0147	113.906	106.424	98.8579	F2R like trypsin receptor 1 [Source:HGNC Symbol;Acc:HGNC:3538	chr5:76114	NM_00524	XM_01700	.	.	NP_00523	XP_016864	protein_co	ENSG0000	2150	3538
F2RL2	0.085091	0.07778	0	0.128154	0.157069	0.480459	coagulation factor II thrombin receptor like 2 [Source:HGNC Symb	chr5:75699	NM_00125	.	.	.	NP_00124	.	protein_co	ENSG0000	2151	3539
F2RL3	0	0	0	0	0	0	F2R like thrombin/trypsin receptor 3 [Source:HGNC Symbol;Acc:H	chr19:1699	NM_00395	.	.	.	NP_00394	.	protein_co	ENSG0000	9002	3540
F3	11.1754	11.1538	12.4159	13.8383	12.5673	14.0707	coagulation factor III, tissue factor [Source:HGNC Symbol;Acc:HG	chr1:94994	NM_00117	.	.	.	NP_00117	.	protein_co	ENSG0000	2152	3541
F5	0.230136	0.134724	0.461057	0.085971	0.142582	0.163724	coagulation factor V [Source:HGNC Symbol;Acc:HGNC:3542]	chr1:16948	NM_00013	.	.	.	NP_00012	XP_016856	protein_co	ENSG0000	2153	3542
F7	0.021591	0	0	0	0	0.023405	coagulation factor VII [Source:HGNC Symbol;Acc:HGNC:3544]	chr13:1137	NM_00013	XM_01153	.	.	NP_00012	XP_006720	protein_co	ENSG0000	2155	3544
F8	2.1167	2.53157	2.64702	2.05662	1.5969	1.64489	coagulation factor VIII [Source:HGNC Symbol;Acc:HGNC:3546]	chrX:15406	NM_00013	XM_01153	.	.	NP_00012	.	protein_co	ENSG0000	2157	3546
F8A1	10.1195	10.3368	9.82832	11.114	10.4568	9.54465	coagulation factor VIII-associated 1 [Source:HGNC Symbol;Acc:HG	chrX:15406	NM_01215	.	.	.	NP_03628	.	protein_co	ENSG0000	8263	3547
F8A2	0	0	0	0	0	0	coagulation factor VIII-associated 2 [Source:HGNC Symbol;Acc:HG	chrX:15461	NM_00100	.	.	.	NP_00100	.	protein_co	ENSG0000	474383	31849
F8A3	0.257791	0	0	0	0	0	coagulation factor VIII-associated 3 [Source:HGNC Symbol;Acc:HG	chrX:15468	NM_00100	.	.	.	NP_00100	.	protein_co	ENSG0000	474384	31850
F9	0	0	0	0	0	0	coagulation factor IX [Source:HGNC Symbol;Acc:HGNC:3551]	chrX:13861	NM_00013	XM_00526	.	.	NP_00012	XP_005262	protein_co	ENSG0000	2158	3551
FA2H	2.48053	2.81246	3.76529	2.15411	2.60011	3.80088	fatty acid 2-hydroxylase [Source:HGNC Symbol;Acc:HGNC:21197]	chr16:7474	NM_02430	.	.	.	NP_07728	.	protein_co	ENSG0000	79152	21197

FAAH	7.29485	7.03802	7.70983	7.48151	7.15165	5.34514	fatty acid amide hydrolase [Source:HGNC Symbol;Acc:HGNC:3553	chr1:46859	NM_00144	.	.	.	NP_00143	.	protein_co	ENSG0000	2166	3553
FAAH2	3.09195	3.33134	2.90647	2.70388	2.11696	3.78853	fatty acid amide hydrolase 2 [Source:HGNC Symbol;Acc:HGNC:26	chrX:57313	NM_17491	XM_01153	.	.	NP_77757	XP_005262	protein_co	ENSG0000	158584	26440
FAAP100	20.5963	20.4161	23.1132	19.2593	15.6671	13.5148	Fanconi anemia core complex associated protein 100 [Source:HG	chr17:7950	NM_02516	XM_01702	.	.	NP_07943	XP_006722	protein_co	ENSG0000	80233	26171
FAAP20	54.7001	57.3213	63.5982	54.0788	39.7907	54.9346	Fanconi anemia core complex associated protein 20 [Source:HGN	chr1:20049	NM_00125	XM_01154	.	.	NP_00124	XP_006710	protein_co	ENSG0000	199990	26428
FAAP24	2.48412	3.32439	3.3727	3.88082	2.38353	2.41227	Fanconi anemia core complex associated protein 24 [Source:HGN	chr19:3346	NM_00130	.	.	.	NP_00128	.	protein_co	ENSG0000	91442	28467
FABP1	0	0	0	0	0	0	fatty acid binding protein 1 [Source:HGNC Symbol;Acc:HGNC:355	chr2:88422	NM_00144	.	.	.	NP_00143	.	protein_co	ENSG0000	2168	3555
FABP12	0	0	0	0	0	0.280045	fatty acid binding protein 12 [Source:HGNC Symbol;Acc:HGNC:34	chr8:82437	NM_00110	XM_01151	.	.	NP_00109	XP_005251	protein_co	ENSG0000	646486	34524
FABP2	0	0	0	0	0	0	fatty acid binding protein 2 [Source:HGNC Symbol;Acc:HGNC:355	chr4:12023	NM_00013	.	.	.	NP_00012	.	protein_co	ENSG0000	2169	3556
FABP3	0	0	0	0.098078	0	0.092651	fatty acid binding protein 3 [Source:HGNC Symbol;Acc:HGNC:355	chr1:31838	NM_00410	.	.	.	NP_00130	.	protein_co	ENSG0000	2170	3557
FABP4	0	0	0	0	0	0	fatty acid binding protein 4 [Source:HGNC Symbol;Acc:HGNC:355	chr8:82390	NM_00144	.	.	.	NP_00143	.	protein_co	ENSG0000	2167	3559
FABP5	191.872	168.64	169.357	245.056	217.835	175.341	fatty acid binding protein 5 [Source:HGNC Symbol;Acc:HGNC:356	chr8:82192	NM_00144	.	.	.	NP_00143	.	protein_co	ENSG0000	2171	3560
FABP6	6.1583	3.13602	6.62897	4.13308	2.58368	3.2354	fatty acid binding protein 6 [Source:HGNC Symbol;Acc:HGNC:356	chr5:15961	NM_00104	XM_01153	.	.	NP_00103	XP_006714	protein_co	ENSG0000	2172	3561
FABP7	0	0	0	0	0	0	fatty acid binding protein 7 [Source:HGNC Symbol;Acc:HGNC:356	chr6:12310	NM_00144	XM_00526	.	.	NP_00130	XP_005266	protein_co	ENSG0000	2173	3562
FABP9	0	0	0	0	0	0	fatty acid binding protein 9 [Source:HGNC Symbol;Acc:HGNC:356	chr8:82370	NM_00108	.	.	.	NP_00107	.	protein_co	ENSG0000	646480	3563
FADD	21.9368	22.4985	21.4374	23.4049	21.3436	24.4034	Fas associated via death domain [Source:HGNC Symbol;Acc:HGN(	chr11:7004	NM_00382	.	.	.	NP_00381	.	protein_co	ENSG0000	8772	3573
FADS1	23.3656	23.2448	29.8911	23.1039	23.1163	29.5579	fatty acid desaturase 1 [Source:HGNC Symbol;Acc:HGNC:3574]	chr11:6155	NM_01340	XM_01154	NR_03172	.	NP_03753	XP_011543	protein_co	ENSG0000	100302263	3574
FADS2	10.5183	12.4464	6.83888	12.1857	11.1106	9.92118	fatty acid desaturase 2 [Source:HGNC Symbol;Acc:HGNC:3575]	chr11:6155	NM_00128	.	.	.	NP_00126	.	protein_co	ENSG0000	9415	3575
FADS3	26.4988	25.3942	30.4064	20.4754	17.2652	18.0102	fatty acid desaturase 3 [Source:HGNC Symbol;Acc:HGNC:3576]	chr11:6164	NM_02172	XM_01701	.	.	NP_06837	XP_011543	protein_co	ENSG0000	3995	3576
FADS6	0	0	0	0	0	0.043409	fatty acid desaturase 6 [Source:HGNC Symbol;Acc:HGNC:30459]	chr17:7287	NM_17812	XM_00525	.	.	NP_83522	XP_005257	protein_co	ENSG0000	283985	30459
FAF1	19.8545	20.6575	18.2978	21.495	20.25	18.9442	Fas associated factor 1 [Source:HGNC Symbol;Acc:HGNC:3578]	chr1:50902	NM_00705	.	.	.	NP_00898	.	protein_co	ENSG0000	11124	3578
FAF2	31.5508	31.5474	32.6632	32.9451	34.6297	35.5989	Fas associated factor family member 2 [Source:HGNC Symbol;Acc	chr5:17587	NM_01461	XM_01153	.	.	NP_05542	XP_011532	protein_co	ENSG0000	23197	24666
FAH	12.2602	11.2688	12.5227	13.3872	10.927	8.02264	fumarylacetoacetate hydrolase [Source:HGNC Symbol;Acc:HGNC:	chr15:8044	NM_00013	.	.	.	NP_00012	.	protein_co	ENSG0000	2184	3579
FAHD1	18.861	19.5063	19.4515	21.4496	18.2176	15.5564	fumarylacetoacetate hydrolase domain containing 1 [Source:HGN	chr16:1857	NM_00101	.	.	.	NP_00101	.	protein_co	ENSG0000	81889	14169
FAHD2A	7.11067	7.95604	8.35827	8.49736	6.14407	6.38233	fumarylacetoacetate hydrolase domain containing 2A [Source:HG	chr2:96068	NM_01604	XM_01151	.	.	NP_05712	XP_011509	protein_co	ENSG0000	51011	24252
FAHD2B	7.02689	6.84372	8.67407	8.214	6.32714	7.1806	fumarylacetoacetate hydrolase domain containing 2B [Source:HG	chr2:97749	NM_00132	XM_00526	.	.	NP_00130	XP_005263	protein_co	ENSG0000	151313	25318
FAIM	17.0676	17.3253	13.2779	20.6907	19.2018	16.4573	Fas apoptotic inhibitory molecule [Source:HGNC Symbol;Acc:HGN	chr3:13832	NM_00103	XM_01151	.	.	NP_00102	XP_011511	protein_co	ENSG0000	55179	18703
FAIM2	0	0	0	0	0	0	Fas apoptotic inhibitory molecule 2 [Source:HGNC Symbol;Acc:H(	chr12:5026	NM_01230	XM_01701	.	.	NP_03643	XP_005268	protein_co	ENSG0000	23017	17067
FAM101A	0.238869	0.117341	0.282343	0.042457	0	0.044048	family with sequence similarity 101 member A [Source:HGNC Syn	chr12:1247	NM_00120	.	.	.	NP_00119	.	protein_co	ENSG0000	100533183	27051
FAM101B	22.0436	22.7175	29.9866	23.4159	23.4884	26.9218	family with sequence similarity 101 member B [Source:HGNC Syn	chr17:2897	NM_18270	.	.	.	NP_87436	.	protein_co	ENSG0000	359845	28705
FAM102A	12.9717	13.1158	10.8841	8.2798	7.76392	8.35384	family with sequence similarity 102 member A [Source:HGNC Syn	chr9:13070	NM_00103	XM_01151	.	.	NP_00103	XP_005252	protein_co	ENSG0000	399665	31419
FAM102B	7.12057	7.17608	7.5552	9.70137	12.7428	10.9572	family with sequence similarity 102 member B [Source:HGNC Syn	chr1:10910	NM_00101	.	.	.	NP_00101	.	protein_co	ENSG0000	284611	27637
FAM103A1	15.937	15.7515	16.2253	16.4501	13.0238	10.7096	family with sequence similarity 103 member A1 [Source:HGNC Sy	chr15:8365	NM_03145	.	.	.	NP_11364	.	protein_co	ENSG0000	83640	31022
FAM104A	25.6369	25.8751	27.9415	28.6501	27.5055	30.4347	family with sequence similarity 104 member A [Source:HGNC Syn	chr17:7118	NM_00109	.	.	.	NP_00109	.	protein_co	ENSG0000	84923	25918
FAM104B	15.3968	13.3552	11.2688	15.1333	12.5771	13.1062	family with sequence similarity 104 member B [Source:HGNC Syn	chrX:55169	NM_00116	XM_00672	.	.	NP_00116	XP_016885	protein_co	ENSG0000	90736	25085
FAM105A	3.77928	4.02511	3.99969	4.3092	3.77696	4.0153	family with sequence similarity 105 member A [Source:HGNC Syn	chr5:14581	NM_01901	XM_01151	.	.	NP_06189	XP_011512	protein_co	ENSG0000	54491	25629
FAM106A	0	0	0	0	0	0	family with sequence similarity 106 member A [Source:HGNC Syn	chr17:1842	.	.	.	.	.	.	protein_co	ENSG0000	.	25682
FAM107A	0.057572	0	0.019937	0.025233	0.052298	0	family with sequence similarity 107 member A [Source:HGNC Syn	chr3:58549	NM_00107	XM_00526	.	.	NP_00107	XP_005264	protein_co	ENSG0000	11170	30827
FAM107B	18.6871	18.9149	16.8563	20.4708	21.6187	62.8624	family with sequence similarity 107 member B [Source:HGNC Syn	chr10:1456	NM_00128	XM_01701	.	.	NP_00126	XP_005252	protein_co	ENSG0000	83641	23726
FAM109A	7.79397	7.64082	7.20361	7.83794	7.16248	6.23001	family with sequence similarity 109 member A [Source:HGNC Syn	chr12:1117	NM_00117	XM_01153	.	.	NP_00117	XP_006719	protein_co	ENSG0000	144717	26509
FAM109B	0.109938	0.219871	0.063131	0.039698	0.084312	0	family with sequence similarity 109 member B [Source:HGNC Syn	chr22:4247	NM_00100	XM_00526	.	.	NP_00100	XP_005261	protein_co	ENSG0000	150368	27161
FAM110A	17.9043	16.5298	16.2184	21.5047	18.9076	21.3109	family with sequence similarity 110 member A [Source:HGNC Syn	chr20:8143	NM_00104	XM_01152	.	.	NP_00103	XP_011527	protein_co	ENSG0000	83541	16188
FAM110B	0	0	0	0	0	0	family with sequence similarity 110 member B [Source:HGNC Syn	chr8:58907	NM_14718	XM_00525	.	.	NP_67172	XP_005251	protein_co	ENSG0000	90362	28587
FAM110C	7.21288	6.71678	9.15007	6.18398	7.4141	10.6232	family with sequence similarity 110 member C [Source:HGNC Syn	chr2:38813	NM_00107	XM_01151	.	.	NP_00107	XP_011508	protein_co	ENSG0000	642273	33340
FAM110D	0.309781	0.304771	0.231797	0.250674	0	0.294584	family with sequence similarity 110 member D [Source:HGNC Syn	chr1:26485	NM_02486	XM_01154	.	.	NP_07914	XP_011540	protein_co	ENSG0000	79927	25860
FAM111A	21.7191	18.4491	16.6141	15.636	19.9624	18.4896	family with sequence similarity 111 member A [Source:HGNC Syn	chr11:5891	NM_00114	.	.	.	NP_00113	.	protein_co	ENSG0000	63901	24725
FAM111B	9.65856	7.6288	6.46902	9.85177	15.5537	11.4109	family with sequence similarity 111 member B [Source:HGNC Syn	chr11:5887	NM_00114	.	.	.	NP_00113	.	protein_co	ENSG0000	374393	24200
FAM114A1	9.79433	9.97733	10.522	10.4591	10.1426	12.243	family with sequence similarity 114 member A1 [Source:HGNC Sy	chr4:38869	NM_13838	XM_00671	.	.	NP_61239	XP_005262	protein_co	ENSG0000	92689	25087
FAM114A2	6.30743	7.21669	7.48323	7.29451	6.86203	7.75434	family with sequence similarity 114 member A2 [Source:HGNC Sy	chr5:15336	NM_00131	XM_01153	.	.	NP_00130	XP_005268	protein_co	ENSG0000	10827	1333
FAM117A	8.36121	8.62338	12.7564	10.1676	8.69628	9.55336	family with sequence similarity 117 member A [Source:HGNC Syn	chr17:4778	NM_03080	.	.	.	NP_11042	.	protein_co	ENSG0000	81558	24179
FAM117B	2.78815	3.50523	5.29661	3.08974	3.39808	5.61244	family with sequence similarity 117 member B [Source:HGNC Syn	chr2:20349	NM_17351	.	.	.	NP_77578	.	protein_co	ENSG0000	150864	14440
FAM118A	11.2376	11.7003	13.1717	11.4968	10.9732	10.7496	family with sequence similarity 118 member A [Source:HGNC Syn	chr22:4570	NM_00110	XM_01153	.	.	NP_00109	.	protein_co	ENSG0000	55007	1313
FAM118B	11.8428	10.8655	9.90665	14.6207	11.8528	11.6499	family with sequence similarity 118 member B [Source:HGNC Syn	chr11:1260	NM_02455	XM_01154	.	.	NP_07883	XP_005271	protein_co	ENSG0000	79607	26110
FAM120A	38.635	44.2048	39.4446	39.941	43.4101	37.6297	family with sequence similarity 120A [Source:HGNC Symbol;Acc:H	chr9:96208	NM_00128	XM_01151	.	.	NP_00127	XP_005251	protein_co	ENSG0000	23196	13247
FAM120AOS	25.2078	20.8981	20.246	19.6915	20.7137	17.005	family with sequence similarity 120A opposite strand [Source:HG	chr9:96208	NM_00132	.	NR_13623	.	NP_00130	.	protein_co	ENSG0000	158293	23389
FAM120B	11.9722	12.3799	13.359	12.3988	12.4555	14.1951	family with sequence similarity 120B [Source:HGNC Symbol;Acc:H	chr6:17059	NM_00128	XM_00671	.	.	NP_00127	.	protein_co	ENSG0000	84498	21109
FAM120C	2.89624	3.22071	2.53104	2.89101	1.98578	2.70087	family with sequence similarity 120C [Source:HGNC Symbol;Acc:H	chrX:54094	NM_00130	.	.	.	NP_00128	.	protein_co	ENSG0000	54954	16949
FAM122A	4.95864	5.36636	4.4735	4.46307	5.24497	4.86908	family with sequence similarity 122A [Source:HGNC Symbol;Acc:H	chr9:71320	NM_13833	.	.	.	NP_61220	.	protein_co	ENSG0000	.	23490
FAM122B	40.1842	47.2036	43.3998	42.5098	41.7477	34.6061	family with sequence similarity 122B [Source:HGNC Symbol;Acc:H	chrX:13390	NM_00116	XM_01153	.	.	NP_00116	XP_006724	protein_co	ENSG0000	159090	30490
FAM122C	1.61263	1.35589	1.99129	1.46337	1.22275	2.01019	family with sequence similarity 122C [Source:HGNC Symbol;Acc:H	chrX:13390	NM_00117	XM_00526	.	.	NP_00116	XP_005262	protein_co	ENSG0000	159091	25202

FAM124A	2.04522	1.95829	2.23325	1.96392	1.9866	1.90657	family with sequence similarity 124 member A [Source:HGNC Syn	chr13:5179	NM_00124	.	.	.	NP_00122	XP_016875	protein_co	ENSG0000	220108	26413
FAM124B	0	0	0	0	0	0	family with sequence similarity 124 member B [Source:HGNC Syn	chr2:22524	NM_00112	.	.	.	NP_00111	.	protein_co	ENSG0000	79843	26224
FAM126A	8.30026	9.14947	7.96602	11.9555	14.126	11.0316	family with sequence similarity 126 member A [Source:HGNC Syn	chr7:22973	NM_03258	XM_01151	.	.	NP_11597	XP_00524	protein_co	ENSG0000	84668	24587
FAM126B	1.29284	1.86322	1.73769	1.53361	1.98003	1.67682	family with sequence similarity 126 member B [Source:HGNC Syn	chr2:20183	NM_00132	XM_01151	.	.	NP_00130	XP_006712	protein_co	ENSG0000	285172	28593
FAM127A	41.8753	38.3184	48.1197	43.3346	40.932	38.3416	family with sequence similarity 127 member A [Source:HGNC Syn	chrX:13416	NM_00107	.	.	.	NP_00107	.	protein_co	ENSG0000	8933	2569
FAM127B	52.391	51.7422	49.7792	65.9746	58.5953	53.0288	family with sequence similarity 127 member B [Source:HGNC Syn	chrX:13418	NM_00107	.	.	.	NP_00107	.	protein_co	ENSG0000	26071	24514
FAM127C	0	0	0	0.102819	0.103686	0	family with sequence similarity 127 member C [Source:HGNC Syn	chrX:13415	NM_00107	.	.	.	NP_00107	.	protein_co	ENSG0000	441518	33156
FAM129A	2.60584	2.97441	1.29356	4.02639	4.08168	8.30528	family with sequence similarity 129 member A [Source:HGNC Syn	chr1:18476	NM_05296	XM_01150	.	.	NP_44319	XP_011507	protein_co	ENSG0000	116496	16784
FAM129B	60.5907	61.1472	63.9409	70.0227	61.0143	59.0895	family with sequence similarity 129 member B [Source:HGNC Syn	chr9:13026	NM_00103	XM_01151	.	.	NP_00103	XP_005252	protein_co	ENSG0000	64855	25282
FAM129C	0.074702	0.155274	0	0.092209	0.063272	0.123593	family with sequence similarity 129 member C [Source:HGNC Syn	chr19:1763	NM_00109	XM_01152	.	.	NP_00109	XP_005259	protein_co	ENSG0000	199786	24130
FAM131A	8.8391	10.4977	9.19112	10.145	9.07987	8.41847	family with sequence similarity 131 member A [Source:HGNC Syn	chr3:18405	NM_00117	XM_01151	.	.	NP_00116	XP_005247	protein_co	ENSG0000	131408	28308
FAM131B	0.131621	0.189687	0.342295	0.1837	0.277582	0.282657	family with sequence similarity 131 member B [Source:HGNC Syn	chr7:14305	NM_00103	XM_00671	.	.	NP_00102	XP_005250	protein_co	ENSG0000	9715	22202
FAM131C	5.62117	6.42588	7.56172	7.08763	5.89241	5.51868	family with sequence similarity 131 member C [Source:HGNC Syn	chr1:16384	NM_18262	.	.	.	NP_87242	.	protein_co	ENSG0000	348487	26717
FAM132A	0.582757	0.446425	0.394884	0.493585	0	0.264853	family with sequence similarity 132 member A [Source:HGNC Syn	chr1:11778	NM_00101	XM_01154	.	.	NP_00101	XP_011539	protein_co	ENSG0000	388581	32308
FAM133A	0	0	0	0	0	0	family with sequence similarity 133 member A [Source:HGNC Syn	chrX:92929	NM_00117	XM_01153	.	.	NP_00116	XP_006724	protein_co	ENSG0000	286499	26748
FAM133B	9.41549	7.76005	9.48973	8.75075	10.0096	9.42658	family with sequence similarity 133 member B [Source:HGNC Syn	chr7:92190	NM_00104	XM_01151	.	.	NP_00103	XP_011514	protein_co	ENSG0000	257415	28629
FAM134A	22.8856	22.8469	20.8541	21.2395	19.3227	14.9758	family with sequence similarity 134 member A [Source:HGNC Syn	chr2:22004	NM_02429	XM_00524	.	.	NP_07726	XP_005246	protein_co	ENSG0000	79137	28450
FAM134B	2.32642	2.44283	2.86778	2.57413	2.35759	3.01465	family with sequence similarity 134 member B [Source:HGNC Syn	chr5:16473	NM_00103	XM_01151	.	.	NP_00103	XP_011512	protein_co	ENSG0000	54463	25964
FAM134C	17.6363	17.8085	17.121	12.8008	12.092	14.0845	family with sequence similarity 134 member C [Source:HGNC Syn	chr17:4073	NM_17812	XM_01152	.	.	NP_83522	XP_011522	protein_co	ENSG0000	162427	27258
FAM135A	4.18832	3.63247	3.79113	3.08275	5.59213	5.00942	family with sequence similarity 135 member A [Source:HGNC Syn	chr6:71123	NM_00110	XM_01701	.	.	NP_00109	XP_005248	protein_co	ENSG0000	57579	21084
FAM135B	0	0	0	0	0	0	family with sequence similarity 135 member B [Source:HGNC Syn	chr8:13914	NM_01591	XM_01151	.	.	NP_05699	XP_011515	protein_co	ENSG0000	51059	28029
FAM136A	68.2259	61.3443	62.3048	78.849	81.7841	64.5054	family with sequence similarity 136 member A [Source:HGNC Syn	chr2:70523	NM_03282	.	.	.	NP_11621	.	protein_co	ENSG0000	84908	25911
FAM13A	6.02763	5.46322	4.91341	5.34096	5.45354	5.97122	family with sequence similarity 13 member A [Source:HGNC Sym	chr4:89647	NM_00101	XM_01700	.	.	NP_00101	XP_005262	protein_co	ENSG0000	10144	19367
FAM13B	4.56302	4.55656	4.69425	4.46597	5.59728	4.34611	family with sequence similarity 13 member B [Source:HGNC Sym	chr5:13722	NM_00110	XM_01700	.	.	NP_00109	XP_005272	protein_co	ENSG0000	51306	1335
FAM13C	0.088016	0.071668	0.021996	0.028404	0	0	family with sequence similarity 13 member C [Source:HGNC Sym	chr10:6093	NM_00100	XM_01701	.	.	NP_00100	XP_005269	protein_co	ENSG0000	220965	19371
FAM149A	2.26777	3.02697	3.10304	1.71004	2.03657	2.38727	family with sequence similarity 149 member A [Source:HGNC Syn	chr4:18702	NM_00100	XM_01700	.	.	NP_00100	XP_005262	protein_co	ENSG0000	25854	24527
FAM149B1	2.24393	2.12603	3.32009	2.18434	2.22832	3.12316	family with sequence similarity 149 member B1 [Source:HGNC Sy	chr10:7492	NM_17334	XM_01153	.	.	NP_77548	XP_005269	protein_co	ENSG0000	317662	29162
FAM150A	0	0	0	0	0	0	family with sequence similarity 150 member A [Source:HGNC Syn	chr8:53446	NM_20741	.	.	.	NP_99729	.	protein_co	ENSG0000	389658	33775
FAM150B	0	0	0	0	0	0	family with sequence similarity 150 member B [Source:HGNC Syn	chr2:27955	NM_00100	XM_00671	.	.	NP_00100	XP_005264	protein_co	ENSG0000	285016	27683
FAM151A	0	0	0.031214	0.117198	0	0.039674	family with sequence similarity 151 member A [Source:HGNC Syn	chr1:55013	NM_17678	XM_00671	.	.	NP_78895	XP_006710	protein_co	ENSG0000	338094	25032
FAM151B	0.202241	0.270906	0.438374	0.23039	0.447869	0.294145	family with sequence similarity 151 member B [Source:HGNC Syn	chr5:79783	NM_20554	XM_01154	.	.	NP_99111	XP_006714	protein_co	ENSG0000	167555	33716
FAM153A	0	0	0	0	0	0	family with sequence similarity 153 member A [Source:HGNC Syn	chr5:17714	NM_17366	XM_01153	.	.	NP_77593	XP_005265	protein_co	ENSG0000	285596	29940
FAM153B	0	0	0	0	0	0	family with sequence similarity 153 member B [Source:HGNC Syn	chr5:17548	NM_00126	.	NR_03840	.	NP_00125	.	protein_co	ENSG0000	100507387	27323
FAM153C	0	0	0	0	0	0	family with sequence similarity 153 member C [Source:HGNC Syn	chr5:17743	.	.	.	.	.	.	protein_co	ENSG0000	.	33936
FAM155A	0	0	0	0	0	0.010051	family with sequence similarity 155 member A [Source:HGNC Syn	chr13:1078	NM_00108	.	.	.	NP_00107	.	protein_co	ENSG0000	728215	33877
FAM155B	6.0071	6.79514	4.60081	5.48837	5.81836	3.51021	family with sequence similarity 155 member B [Source:HGNC Syn	chrX:68725	NM_01568	.	.	.	NP_05650	.	protein_co	ENSG0000	27112	30701
FAM156A	0.062442	0.189039	0.033714	0.207127	0.277622	0.112806	family with sequence similarity 156 member A [Source:HGNC Syn	chrX:52976	NM_00124	XM_01153	.	.	NP_00122	XP_005262	protein_co	ENSG0000	727866	30114
FAM156B	0.850186	0.880339	0.473303	0.567634	0.700369	0.262377	family with sequence similarity 156 member B [Source:HGNC Syn	chrX:52926	NM_00109	XM_01153	.	.	NP_00109	XP_005262	protein_co	ENSG0000	727866	31962
FAM159A	0	0	0	0	0	0	family with sequence similarity 159 member A [Source:HGNC Syn	chr1:53099	NM_00104	.	NR_13515	.	NP_00103	.	protein_co	ENSG0000	348378	28757
FAM159B	0	0	0	0	0	0	family with sequence similarity 159 member B [Source:HGNC Syn	chr5:63986	NM_00116	.	.	.	NP_00115	.	protein_co	ENSG0000	100132916	34236
FAM160A1	1.94838	1.86268	2.1108	1.57408	1.58684	2.14371	family with sequence similarity 160 member A1 [Source:HGNC Sy	chr4:15233	NM_00110	XM_01153	.	.	NP_00110	XP_005263	protein_co	ENSG0000	729830	34237
FAM160A2	11.7395	11.1041	11.3619	11.5666	11.1423	11.0824	family with sequence similarity 160 member A2 [Source:HGNC Sy	chr11:6232	NM_00109	XM_01152	.	.	NP_00109	XP_006718	protein_co	ENSG0000	84067	25378
FAM160B1	6.75519	6.82331	6.97985	4.83284	5.7331	7.51006	family with sequence similarity 160 member B1 [Source:HGNC Sy	chr10:1165	NM_00113	.	.	.	NP_00112	.	protein_co	ENSG0000	57700	29320
FAM160B2	11.0959	10.9648	10.9404	9.67599	8.89598	8.61857	family with sequence similarity 160 member B2 [Source:HGNC Sy	chr8:21946	NM_02274	XM_01154	.	.	NP_07358	XP_006716	protein_co	ENSG0000	64760	16492
FAM161A	4.23972	4.59123	5.13426	3.86474	4.40209	4.08495	family with sequence similarity 161 member A [Source:HGNC Syn	chr2:62051	NM_00120	XM_01700	.	.	NP_00118	XP_016860	protein_co	ENSG0000	84140	25808
FAM161B	1.34455	1.34921	0.907736	0.831592	1.05189	0.999357	family with sequence similarity 161 member B [Source:HGNC Syn	chr14:7431	NM_15244	XM_01153	.	.	NP_68965	XP_011534	protein_co	ENSG0000	145483	19854
FAM162A	46.5397	48.27	53.2705	64.772	48.6326	58.1814	family with sequence similarity 162 member A [Source:HGNC Syn	chr3:12210	NM_01436	.	.	.	NP_05518	.	protein_co	ENSG0000	26355	17865
FAM162B	0	0	0	0	0	0	family with sequence similarity 162 member B [Source:HGNC Syn	chr6:11707	NM_00108	.	.	.	NP_00107	.	protein_co	ENSG0000	221303	21549
FAM163A	0	0	0.023986	0	0	0	family with sequence similarity 163 member A [Source:HGNC Syn	chr1:17971	NM_17350	XM_01700	.	.	NP_77578	XP_005244	protein_co	ENSG0000	148753	28274
FAM163B	0	0	0	0	0	0	family with sequence similarity 163 member B [Source:HGNC Syn	chr9:13644	NM_00108	XM_01151	.	.	NP_00107	XP_005272	protein_co	ENSG0000	642968	33277
FAM166A	0	0.070902	0.062313	0.076554	0	0	family with sequence similarity 166 member A [Source:HGNC Syn	chr9:14013	NM_00100	XM_01151	.	.	NP_00100	XP_011516	protein_co	ENSG0000	401565	33818
FAM166B	0.358566	0.51206	0.429288	0.957863	0.874592	1.11449	family with sequence similarity 166 member B [Source:HGNC Syn	chr9:35538	NM_00109	XM_01151	.	.	NP_00109	XP_011516	protein_co	ENSG0000	730112	34242
FAM167A	2.99025	2.41285	2.39468	1.54225	1.4902	3.34151	family with sequence similarity 167 member A [Source:HGNC Syn	chr8:11278	NM_05327	XM_01154	.	.	NP_44450	XP_005272	protein_co	ENSG0000	83648	15549
FAM167B	0.655107	0.552068	0.242757	0.398384	0.215579	0.62268	family with sequence similarity 167 member B [Source:HGNC Syn	chr1:32712	NM_03264	.	.	.	NP_11603	.	protein_co	ENSG0000	84734	28133
FAM168A	12.6988	14.9166	14.8431	12.2497	13.3637	12.97	family with sequence similarity 168 member A [Source:HGNC Syn	chr11:7311	NM_00128	XM_00671	.	.	NP_00127	XP_016872	protein_co	ENSG0000	23201	28999
FAM168B	52.0746	55.9121	58.6589	56.304	58.962	61.8198	family with sequence similarity 168 member B [Source:HGNC Syn	chr2:13180	NM_00100	XM_01700	.	.	NP_00100	XP_005263	protein_co	ENSG0000	130074	27016
FAM169A	7.06802	6.93529	6.49709	6.14339	8.06668	8.3102	family with sequence similarity 169 member A [Source:HGNC Syn	chr5:73923	NM_01556	XM_01154	.	.	NP_05638	XP_005248	protein_co	ENSG0000	26049	29138
FAM170A	0	0	0.041491	0	0	0	family with sequence similarity 170 member A [Source:HGNC Syn	chr5:11896	NM_00116	.	.	.	NP_00115	.	protein_co	ENSG0000	340069	27963
FAM170B	0	0	0.046947	0	0	0	family with sequence similarity 170 member B [Source:HGNC Syn	chr10:5033	NM_00116	.	.	.	NP_00115	.	protein_co	ENSG0000	170370	19736

FAM171A1	14.9639	15.2133	15.7836	12.9105	12.1837	10.057	family with sequence similarity 171 member A1 [Source:HGNC Sy	chr10:1525	NM_00101	XM_01701	.	.	NP_001010	XP_011517	protein_co	ENSG0000	221061	23522
FAM171A2	10.289	9.577	10.5945	14.3994	13.4601	11.4828	family with sequence similarity 171 member A2 [Source:HGNC Sy	chr17:4243	NM_19847	.	.	.	NP_940877	.	protein_co	ENSG0000	284069	30480
FAM171B	5.10812	5.19252	4.649	4.99782	6.12157	7.17366	family with sequence similarity 171 member B [Source:HGNC Syn	chr2:18755	NM_17745	XM_00524	.	.	NP_803237	.	protein_co	ENSG0000	165215	29412
FAM172A	5.76518	7.20419	6.68818	3.92023	5.04907	6.71409	family with sequence similarity 172 member A [Source:HGNC Syn	chr5:92953	NM_00116	XM_01154	NR_03175	.	NP_00115	XP_005272	protein_co	ENSG0000	100313887	25365
FAM173A	29.6467	27.5955	25.5402	23.5554	22.5063	17.6086	family with sequence similarity 173 member A [Source:HGNC Syn	chr16:7709	NM_00127	.	.	.	NP_00125	.	protein_co	ENSG0000	65990	14152
FAM173B	10.947	10.1919	8.37105	9.09763	10.1718	9.91056	family with sequence similarity 173 member B [Source:HGNC Syn	chr5:10226	NM_00125	XM_01151	.	.	NP_00124	XP_011512	protein_co	ENSG0000	134145	27029
FAM174A	6.41402	10.0025	6.77799	9.01556	7.88991	7.55456	family with sequence similarity 174 member A [Source:HGNC Syn	chr5:99871	NM_19850	.	.	.	NP_94090	XP_006714	protein_co	ENSG0000	345757	24943
FAM174B	0.034006	0	0.058426	0.07491	0.107749	0.070684	family with sequence similarity 174 member B [Source:HGNC Syn	chr15:9316	NM_20744	.	.	.	NP_99732	.	protein_co	ENSG0000	400451	34339
FAM175A	7.33943	5.35394	8.0467	4.96721	5.33	7.63559	family with sequence similarity 175 member A [Source:HGNC Syn	chr4:84377	NM_13907	XM_01700	.	.	NP_62077	XP_005263	protein_co	ENSG0000	84142	25829
FAM175B	16.4252	17.7093	15.1775	15.9256	17.8959	17.8407	family with sequence similarity 175 member B [Source:HGNC Syn	chr10:1264	NM_03218	XM_01153	.	.	NP_11555	XP_011537	protein_co	ENSG0000	23172	28975
FAM177A1	26.8604	25.1532	27.9342	23.8102	22.0335	25.8505	family with sequence similarity 177 member A1 [Source:HGNC Sy	chr14:3551	NM_00107	.	.	.	NP_00107	.	protein_co	ENSG0000	283635	19829
FAM177B	0	0	0	0	0	0	family with sequence similarity 177 member B [Source:HGNC Syn	chr1:22291	NM_20746	.	.	.	NP_00131	XP_016856	protein_co	ENSG0000	400823	34395
FAM178B	0	0	0	0	0.197402	0	family with sequence similarity 178 member B [Source:HGNC Syn	chr2:97541	NM_00112	XM_01151	.	.	NP_00111	XP_011509	protein_co	ENSG0000	51252	28036
FAM179A	0.048119	0.024847	0.088563	0.04666	0.040721	0.055719	family with sequence similarity 179 member A [Source:HGNC Syn	chr2:29204	NM_19928	XM_01153	.	.	NP_95497	XP_016858	protein_co	ENSG0000	165186	33715
FAM179B	0.492875	0.353005	0.366447	0.452596	0.541652	0.342229	family with sequence similarity 179 member B [Source:HGNC Syn	chr14:4543	NM_00130	XM_01153	.	.	NP_00129	XP_005267	protein_co	ENSG0000	23116	19959
FAM180A	0.323989	0.582997	0.125189	0.078091	0.161253	0.286344	family with sequence similarity 180 member A [Source:HGNC Syn	chr7:13541	NM_20585	XM_00671	.	.	NP_99532	XP_006716	protein_co	ENSG0000	389558	33773
FAM180B	0	0	0.050933	0	0.067531	0.06328	family with sequence similarity 180 member B [Source:HGNC Syn	chr11:4760	NM_00116	.	.	.	NP_00115	.	protein_co	ENSG0000	399888	34451
FAM181A	0	0	0	0	0	0	family with sequence similarity 181 member A [Source:HGNC Syn	chr14:9438	NM_00120	.	.	.	NP_00119	XP_011535	protein_co	ENSG0000	90050	20491
FAM181B	0	0	0	0	0	0	family with sequence similarity 181 member B [Source:HGNC Syn	chr11:8244	NM_17588	.	.	.	NP_78708	.	protein_co	ENSG0000	220382	28512
FAM182B	0.05581	0	0	0	0	0.061098	family with sequence similarity 182 member B [Source:HGNC Syn	chr20:2575	.	.	NR_02706	.	.	.	protein_co	ENSG0000	728882	34503
FAM183A	0	0	0.25391	0.156519	0	0	family with sequence similarity 183 member A [Source:HGNC Syn	chr1:43610	NM_00110	XM_00527	.	.	NP_00109	XP_005270	protein_co	ENSG0000	440585	34347
FAM184A	0.676286	0.62919	0.682372	0.791994	0.874629	0.988887	family with sequence similarity 184 member A [Source:HGNC Syn	chr6:11928	NM_00110	.	.	.	NP_00109	.	protein_co	ENSG0000	79632	20991
FAM184B	0.039411	0.030258	0.034398	0	0	0.010284	family with sequence similarity 184 member B [Source:HGNC Syn	chr4:17616	NM_01568	XM_01151	.	.	NP_05650	.	protein_co	ENSG0000	27146	29235
FAM185A	2.43106	2.65815	2.67944	1.96317	2.18132	2.41466	family with sequence similarity 185 member A [Source:HGNC Syn	chr7:10238	NM_00114	XM_01151	.	.	NP_00113	XP_006715	protein_co	ENSG0000	222234	22412
FAM186A	0	0	0	0	0	0	family with sequence similarity 186 member A [Source:HGNC Syn	chr12:5072	NM_00114	XM_01153	.	.	NP_00113	XP_006719	protein_co	ENSG0000	121006	26980
FAM186B	0.076434	0.115367	0.113392	0	0.044102	0.103429	family with sequence similarity 186 member B [Source:HGNC Syn	chr12:4997	NM_03213	XM_01153	.	.	NP_11550	XP_006719	protein_co	ENSG0000	84070	25296
FAM187A	1.14166	1.05378	1.23226	0.871821	0.705093	1.00857	family with sequence similarity 187 member A [Source:HGNC Syn	chr17:4297	NM_00125	.	.	.	NP_00124	.	protein_co	ENSG0000	388389	35153
FAM187B	0	0	0	0	0	0	family with sequence similarity 187 member B [Source:HGNC Syn	chr19:3571	NM_15248	.	.	.	NP_68969	.	protein_co	ENSG0000	148109	26366
FAM188A	9.40145	8.93	9.15367	10.2598	11.708	12.1421	family with sequence similarity 188 member A [Source:HGNC Syn	chr10:1582	NM_02494	XM_01151	.	.	NP_07922	XP_005252	protein_co	ENSG0000	80013	23578
FAM188B	2.16846	2.63022	2.85785	3.21089	3.25315	1.93736	family with sequence similarity 188 member B [Source:HGNC Syn	chr7:30811	NM_03222	.	.	.	NP_11559	.	protein_co	ENSG0000	84182	21916
FAM189A1	0.282515	0.229808	0.363621	0.325279	0.42633	0.802098	family with sequence similarity 189 member A1 [Source:HGNC Sy	chr15:2941	NM_01530	XM_01152	.	.	NP_05612	XP_011519	protein_co	ENSG0000	23359	29075
FAM189A2	0.904741	1.27984	1.26266	0.511941	0.748426	0.865243	family with sequence similarity 189 member A2 [Source:HGNC Sy	chr9:71939	NM_00112	XM_01701	.	.	NP_00112	XP_005252	protein_co	ENSG0000	9413	24820
FAM189B	30.8761	32.2292	32.4224	30.3379	25.8938	21.6113	family with sequence similarity 189 member B [Source:HGNC Syn	chr1:15521	NM_00126	XM_00671	.	.	NP_00125	XP_005244	protein_co	ENSG0000	10712	1233
FAM192A	34.4121	32.9696	30.2152	35.4104	27.7364	27.573	family with sequence similarity 192 member A [Source:HGNC Syn	chr16:5718	NM_02494	XM_01702	.	.	NP_07922	XP_005256	protein_co	ENSG0000	80011	29856
FAM193A	4.48923	4.03273	3.75409	3.60084	3.62846	3.34817	family with sequence similarity 193 member A [Source:HGNC Syn	chr4:25383	NM_00125	XM_01151	.	.	NP_00124	XP_006713	protein_co	ENSG0000	8603	16822
FAM193B	8.33452	7.68935	6.76526	8.17637	6.73167	6.25279	family with sequence similarity 193 member B [Source:HGNC Syn	chr5:17694	NM_00119	XM_01153	.	.	NP_00117	XP_006714	protein_co	ENSG0000	54540	25524
FAM196A	0	0	0	0	0	0	family with sequence similarity 196 member A [Source:HGNC Syn	chr10:1285	NM_00103	XM_01154	.	.	NP_00103	XP_005252	protein_co	ENSG0000	642938	33859
FAM196B	0	0	0	0	0	0	family with sequence similarity 196 member B [Source:HGNC Syn	chr5:16906	NM_00112	XM_01153	.	.	NP_00112	XP_011532	protein_co	ENSG0000	100131897	37271
FAM198A	0.711946	0.671784	0.614635	0.938526	0.982859	0.676087	family with sequence similarity 198 member A [Source:HGNC Syn	chr3:43021	NM_00112	.	.	.	NP_00112	.	protein_co	ENSG0000	729085	24485
FAM198B	1.02686	0.801569	0.799006	0.90664	1.10017	0.334271	family with sequence similarity 198 member B [Source:HGNC Syn	chr4:15904	NM_00103	.	.	.	NP_00102	.	protein_co	ENSG0000	51313	25312
FAM199X	10.605	11.9359	12.4238	10.5251	11.8062	12.0999	family with sequence similarity 199, X-linked [Source:HGNC Symb	chrX:10341	NM_20731	XM_01702	.	.	NP_99720	XP_005262	protein_co	ENSG0000	139231	25195
FAM19A1	0	0	0	0	0	0	family with sequence similarity 19 member A1, C-C motif chemok	chr3:68053	NM_00125	.	.	.	NP_00123	XP_016861	protein_co	ENSG0000	407738	21587
FAM19A2	0.251162	0.297803	0.440084	0.135215	0.186513	0.324356	family with sequence similarity 19 member A2, C-C motif chemok	chr12:6210	NM_17853	XM_01153	.	.	NP_84863	XP_006719	protein_co	ENSG0000	338811	21589
FAM19A3	1.60238	1.90621	1.88061	1.34642	1.35004	0.730429	family with sequence similarity 19 member A3, C-C motif chemok	chr1:11326	NM_00100	XM_00671	.	.	NP_00100	XP_005270	protein_co	ENSG0000	284467	21590
FAM19A4	0	0	0.111977	0.049097	0.096007	0.127871	family with sequence similarity 19 member A4, C-C motif chemok	chr3:68780	NM_00100	XM_01153	.	.	NP_00100	XP_011531	protein_co	ENSG0000	151647	21591
FAM19A5	0	0	0	0	0	0	family with sequence similarity 19 member A5, C-C motif chemok	chr22:4888	NM_00108	.	.	.	NP_00107	.	protein_co	ENSG0000	25817	21592
FAM200A	3.29671	2.79759	3.19137	3.0548	4.11404	4.1148	family with sequence similarity 200 member A [Source:HGNC Syn	chr7:99143	NM_14511	XM_01151	.	.	NP_65980	XP_005250	protein_co	ENSG0000	221786	25401
FAM200B	3.78048	4.0072	3.48238	4.57011	4.97968	4.90634	family with sequence similarity 200 member B [Source:HGNC Syn	chr4:15683	NM_00114	XM_01700	.	.	NP_00113	XP_016863	protein_co	ENSG0000	285550	27740
FAM203B	0.515963	0.460253	0.651671	0.453899	0.699839	0.479679	family with sequence similarity 203, member B [Source:HGNC Syr	chr8:14543	.	.	.	.	.	.	protein_co	ENSG0000	728071	32323
FAM204A	29.9196	25.274	24.0819	25.3409	26.2797	25.0412	family with sequence similarity 204 member A [Source:HGNC Syn	chr10:1200	NM_00113	XM_00671	.	.	NP_00112	XP_005270	protein_co	ENSG0000	63877	25794
FAM205A	0	0	0	0	0	0	family with sequence similarity 205 member A [Source:HGNC Syn	chr9:34723	NM_00114	.	.	.	NP_00113	.	protein_co	ENSG0000	259308	41911
FAM205C	0	0	0	0	0	0	family with sequence similarity 205 member C [Source:HGNC Syn	chr9:34889	NM_00130	XM_01701	.	.	NP_00129	XP_016869	protein_co	ENSG0000	100129969	42673
FAM206A	14.1528	12.3973	15.3817	10.047	14.9867	13.4116	family with sequence similarity 206 member A [Source:HGNC Syn	chr9:11169	NM_01783	XM_01151	.	.	NP_06030	XP_011517	protein_co	ENSG0000	54942	1364
FAM207A	30.4626	29.8601	26.8233	34.9296	27.5711	26.6479	family with sequence similarity 207 member A [Source:HGNC Syn	chr21:4635	NM_00131	XM_00526	.	.	NP_00130	XP_005261	protein_co	ENSG0000	85395	15811
FAM208A	10.4458	10.4781	10.6636	8.66083	13.184	12.8495	family with sequence similarity 208 member A [Source:HGNC Syn	chr3:56591	NM_00111	XM_01153	.	.	NP_00110	XP_005265	protein_co	ENSG0000	23272	30314
FAM208B	19.7321	20.6742	21.3138	16.1018	20.5335	18.4202	family with sequence similarity 208 member B [Source:HGNC Syn	chr10:5726	NM_00132	XM_01701	.	.	NP_00130	XP_005252	protein_co	ENSG0000	54906	23484
FAM209A	0.297302	0.101609	0.341483	0.218174	0.336488	0.311213	family with sequence similarity 209 member A [Source:HGNC Syn	chr20:5504	NM_00101	.	.	.	NP_00101	.	protein_co	ENSG0000	200232	16100
FAM209B	0.282865	0.293295	0.736753	0.314528	0.638262	0.145561	family with sequence similarity 209 member B [Source:HGNC Syn	chr20:5510	NM_00101	.	.	.	NP_00101	.	protein_co	ENSG0000	388799	16101
FAM20A	0	0	0	0	0.027591	0	family with sequence similarity 20 member A [Source:HGNC Symb	chr17:6650	NM_00124	XM_01152	NR_02775	.	NP_00123	XP_006722	protein_co	ENSG0000	54757	23015

FAM20B	12.2833	11.9655	12.6746	11.0341	13.3074	17.5728	family with sequence similarity 20 member B [Source:HGNC Symb	chr1:17899	NM_00132	XM_01151	.	.	NP_00131	XP_011508	protein_co	ENSG0000	9917	23017
FAM20C	9.3165	10.4942	10.3921	13.4675	12.4136	10.4346	family with sequence similarity 20 member C [Source:HGNC Symb	chr7:19296	NM_02022	.	.	.	NP_064608	.	protein_co	ENSG0000	56975	22140
FAM210A	7.91954	8.76552	7.59343	8.72006	9.52855	9.02837	family with sequence similarity 210 member A [Source:HGNC Syn	chr18:1366	NM_00109	XM_00672	.	.	NP_00109	XP_006722	protein_co	ENSG0000	125228	28346
FAM210B	13.3891	13.9783	17.9136	12.095	13.9594	14.757	family with sequence similarity 210 member B [Source:HGNC Syn	chr20:5493	NM_08082	.	.	.	NP_54301	.	protein_co	ENSG0000	116151	16102
FAM212A	1.62691	1.9839	1.76202	1.88857	1.62626	0.914265	family with sequence similarity 212 member A [Source:HGNC Syn	chr3:49840	NM_20337	.	.	.	NP_976248	.	protein_co	ENSG0000	389119	32480
FAM212B	1.8889	2.07593	2.59804	2.0835	2.3102	1.44827	family with sequence similarity 212 member B [Source:HGNC Syn	chr1:11226	NM_01909	XM_01154	NR_12596	.	NP_06197	XP_011540	protein_co	ENSG0000	101928718	28045
FAM213A	3.67968	3.00413	4.26058	1.58253	1.41341	1.80501	family with sequence similarity 213 member A [Source:HGNC Syn	chr10:8216	NM_00124	XM_01701	.	.	NP_00123	XP_011538	protein_co	ENSG0000	84293	28651
FAM213B	20.9904	22.6015	22.5938	21.9496	19.5076	14.2043	family with sequence similarity 213 member B [Source:HGNC Syn	chr1:25181	NM_00119	XM_01154	NR_03663	.	NP_00118	XP_006710	protein_co	ENSG0000	127281	28390
FAM214A	1.32293	1.22296	1.99284	0.998373	1.2312	1.5748	family with sequence similarity 214 member A [Source:HGNC Syn	chr15:5287	NM_00128	XM_01152	.	.	NP_00127	XP_005254	protein_co	ENSG0000	56204	25609
FAM214B	6.8223	6.79862	7.21922	6.42512	5.50753	5.95109	family with sequence similarity 214 member B [Source:HGNC Syn	chr9:35104	NM_00131	XM_01151	.	.	NP_00130	XP_005251	protein_co	ENSG0000	80256	25666
FAM216A	9.06645	7.92887	9.01036	10.8343	9.84641	7.98461	family with sequence similarity 216 member A [Source:HGNC Syn	chr12:1108	NM_01330	XM_00525	.	.	NP_03743	XP_005253	protein_co	ENSG0000	29902	30180
FAM216B	0	0	0	0	0	0	family with sequence similarity 216 member B [Source:HGNC Syn	chr13:4335	NM_00131	XM_00526	.	.	NP_00130	XP_005266	protein_co	ENSG0000	144809	26883
FAM217A	0	0	0	0	0	0	family with sequence similarity 217 member A [Source:HGNC Syn	chr6:40685	NM_17356	XM_01151	.	.	NP_77583	XP_005249	protein_co	ENSG0000	222826	21362
FAM217B	4.96084	5.29553	4.84912	4.67987	5.90066	4.53386	family with sequence similarity 217 member B [Source:HGNC Syn	chr20:5850	NM_00119	XM_01152	.	.	NP_00117	XP_011527	protein_co	ENSG0000	63939	16170
FAM218A	0	0	0	0	0	0	family with sequence similarity 218 member A [Source:HGNC Syn	chr4:16587	NM_15302	.	.	.	NP_69457	.	protein_co	ENSG0000	152756	26466
FAM219A	6.13005	6.04948	5.86416	7.55236	6.16217	4.75727	family with sequence similarity 219 member A [Source:HGNC Syn	chr9:34398	NM_00118	XM_01151	.	.	NP_00117	XP_011516	protein_co	ENSG0000	203259	19920
FAM219B	14.0104	15.9668	14.2245	10.3123	9.43739	11.441	family with sequence similarity 219 member B [Source:HGNC Syn	chr15:7518	NM_00132	XM_00525	.	.	NP_00130	XP_005254	protein_co	ENSG0000	57184	24695
FAM21A	0.876946	0.854475	1.03471	0.741005	0.720577	0.745277	family with sequence similarity 21 member A [Source:HGNC Symb	chr10:5182	NM_00100	XM_01701	.	.	NP_00100	XP_005269	protein_co	ENSG0000	387680	23416
FAM21B	0.927529	0.723566	0.8092	0.820809	0.781943	0.700859	family with sequence similarity 21, member B [Source:HGNC Sym	chr10:4789	NM_01823	XM_00527	.	XR_247516	NP_06070	XP_005272	protein_co	ENSG0000	55747	23417
FAM21C	2.9248	2.70321	2.54482	2.42286	2.65601	2.23826	family with sequence similarity 21 member C [Source:HGNC Symb	chr10:4622	NM_00116	XM_01701	.	.	NP_00116	XP_011537	protein_co	ENSG0000	253725	23414
FAM21D	0	0	0	0	0	0	family with sequence similarity 21, member D [Source:HGNC Sym	chr10:5118	.	.	.	.	.	.	protein_co	ENSG0000	.	23418
FAM220A	17.9517	12.6167	13.8475	9.75626	13.7184	14.5586	family with sequence similarity 220 member A [Source:HGNC Syn	chr7:63690	NM_00103	.	.	.	NP_00103	.	protein_co	ENSG0000	84792	22422
FAM221A	2.36646	2.60871	3.22546	0	0	0	family with sequence similarity 221 member A [Source:HGNC Syn	chr7:23719	NM_00112	XM_01151	.	.	NP_00112	XP_011513	protein_co	ENSG0000	340277	27977
FAM221B	0	0	0	0	0	0	family with sequence similarity 221 member B [Source:HGNC Syn	chr9:35812	NM_00101	.	.	.	NP_00101	XP_006716	protein_co	ENSG0000	392307	30762
FAM222A	2.64809	3.19835	2.77563	4.05681	4.12978	3.36765	family with sequence similarity 222 member A [Source:HGNC Syn	chr12:1101	NM_03282	XM_00671	.	.	NP_11621	XP_006719	protein_co	ENSG0000	84915	25915
FAM222B	8.73021	9.43498	10.0934	8.66221	8.44662	8.0825	family with sequence similarity 222 member B [Source:HGNC Syn	chr17:2708	NM_00107	XM_01702	.	.	NP_00107	XP_011523	protein_co	ENSG0000	55731	25563
FAM227A	1.42594	1.37687	1.46694	0.834389	0.948226	1.0366	family with sequence similarity 227 member A [Source:HGNC Syn	chr22:3897	NM_00101	XM_01153	.	XR_937892	NP_00101	XP_006724	protein_co	ENSG0000	646851	44197
FAM227B	1.75042	1.57224	1.94551	0.993623	1.38697	1.18976	family with sequence similarity 227 member B [Source:HGNC Syn	chr15:4944	NM_15264	XM_01152	.	.	NP_68986	XP_005254	protein_co	ENSG0000	196951	26543
FAM228A	0	0	0	0.095533	0	0	family with sequence similarity 228 member A [Source:HGNC Syn	chr2:24397	NM_00104	.	.	.	NP_00103	.	protein_co	ENSG0000	653140	34418
FAM228B	0.242694	0.569551	0.702193	0.6951	0.441344	1.00561	family with sequence similarity 228 member B [Source:HGNC Syn	chr2:24299	NM_00114	.	.	.	NP_00113	.	protein_co	ENSG0000	375190	24736
FAM229A	2.14086	1.41349	2.50689	2.25418	0.512001	2.97165	family with sequence similarity 229 member A [Source:HGNC Syn	chr1:32826	NM_00116	.	.	.	NP_00116	.	protein_co	ENSG0000	100128071	44652
FAM229B	9.72039	7.43889	11.3286	16.7213	9.84184	12.5148	family with sequence similarity 229 member B [Source:HGNC Syn	chr6:11240	NM_00103	.	.	.	NP_00102	XP_016866	protein_co	ENSG0000	619208	33858
FAM230A	0	0	0	0	0.012977	0	.	chr22:2069	.	.	.	.	.	.	antisense	ENSG0000	.	.
FAM231A	0	0	0	0	0	0	family with sequence similarity 231 member A [Source:HGNC Syn	chr1:16999	NM_00128	.	.	.	NP_00126	XP_006726	protein_co	ENSG0000	102723383	49427
FAM231C	0	0	0	0	0	0	family with sequence similarity 231 member C [Source:HGNC Syn	chr1:17060	.	XM_00671	.	.	NP_00129	XP_006711	protein_co	ENSG0000	102723383	49508
FAM231D	5.54E-08	5.82E-08	0.022616	0.058386	0.047973	0.025844	family with sequence similarity 231 member D [Source:HGNC Syn	chr1:14967	.	.	.	.	.	XP_005276	protein_co	ENSG0000	100996721	49509
FAM234A	10.9258	10.6937	13.9223	9.13896	8.54737	10.6405	family with sequence similarity 234 member A [Source:HGNC Syn	chr16:2845	NM_00128	XM_01702	.	.	NP_00127	XP_005255	protein_co	ENSG0000	83986	14163
FAM234B	5.8779	6.52689	7.98912	6.49182	7.44014	9.86033	family with sequence similarity 234 member B [Source:HGNC Syn	chr12:1319	NM_02085	.	.	.	NP_06590	XP_016875	protein_co	ENSG0000	57613	29288
FAM24A	0	0	0	0	0	0	family with sequence similarity 24 member A [Source:HGNC Symb	chr10:1246	NM_00102	.	.	.	NP_00102	XP_016871	protein_co	ENSG0000	118670	23470
FAM24B	4.03818	3.79434	3.77457	2.8248	3.00637	1.94795	family with sequence similarity 24 member B [Source:HGNC Symb	chr10:1245	NM_00120	.	NR_03791	.	NP_00119	.	protein_co	ENSG0000	196792	23475
FAM25A	3.80762	2.6178	2.82336	8.99163	2.0869	4.1697	family with sequence similarity 25 member A [Source:HGNC Symb	chr10:8878	NM_00114	.	.	.	NP_00113	.	protein_co	ENSG0000	643161	23436
FAM25B	0	0	0	0	0	0	family with sequence similarity 25 member G [Source:HGNC Symb	chr10:4717	NM_00113	.	.	.	NP_00113	.	protein_co	ENSG0000	100133093	23590
FAM25C	0	0	0	0	0	0	family with sequence similarity 25 member C [Source:HGNC Symb	chr10:4920	NM_00113	.	.	.	NP_00113	.	protein_co	ENSG0000	644054	23586
FAM25G	0	0	0	0	0	0	family with sequence similarity 25 member G [Source:HGNC Symb	chr10:4824	NM_00113	.	.	.	NP_00113	.	protein_co	ENSG0000	100133093	23590
FAM26D	0.084263	0	0	0	0	0	family with sequence similarity 26 member D [Source:HGNC Symb	chr6:11681	NM_00125	XM_01153	.	.	NP_00124	XP_005266	protein_co	ENSG0000	221301	21094
FAM26E	0.145821	0.11313	0.147847	0.194225	0.107034	0.094461	family with sequence similarity 26 member E [Source:HGNC Symb	chr6:11681	NM_15371	.	.	.	NP_71492	.	protein_co	ENSG0000	254228	21568
FAM26F	0.21653	0.909336	0.188413	0	0	0.426705	family with sequence similarity 26 member F [Source:HGNC Symb	chr6:11678	NM_00101	XM_01153	.	.	NP_00101	XP_005267	protein_co	ENSG0000	441168	33391
FAM27A	0.621154	0.122693	0.324853	0.662834	0.289539	0.137005	family with sequence similarity 27, member A [Source:HGNC Sym	chr9:45727	.	.	.	.	.	.	protein_co	ENSG0000	.	19729
FAM27B	0	0	0	0	0	0	family with sequence similarity 27 member B [Source:HGNC Symb	chr9:67792	.	.	.	.	.	.	processed_	ENSG0000	.	23667
FAM27D1	0	0	0	0	0	0	family with sequence similarity 27 member D1 [Source:HGNC Syn	chr9:46389	.	.	.	.	.	.	processed_	ENSG0000	.	32015
FAM27E1	0.049802	0.050093	0	0	0.114365	0	family with sequence similarity 27, member E1 [Source:HGNC Syr	chr9:46385	.	.	.	.	.	.	protein_co	ENSG0000	.	32014
FAM27E2	0.048774	0	0	0.106216	0.11199	0.052413	family with sequence similarity 27 member E2 [Source:HGNC Syn	chr9:45733	.	.	.	.	.	.	processed_	ENSG0000	.	32013
FAM27E3	0.089579	0.31586	0.155673	0.195669	0.308	0.287968	family with sequence similarity 27 member E3 [Source:HGNC Syn	chr9:67784	.	.	NR_10383	.	.	.	transcribed	ENSG0000	100131997	28655
FAM32A	66.3173	63.5408	62.6612	71.3201	66.8055	57.3037	family with sequence similarity 32 member A [Source:HGNC Symb	chr19:1629	NM_01407	.	.	.	NP_05479	.	protein_co	ENSG0000	26017	24563
FAM35A	13.2556	12.2915	12.6934	13.5586	17.2935	17.6889	family with sequence similarity 35 member A [Source:HGNC Symb	chr10:8885	NM_01905	XM_01701	.	.	NP_06192	XP_005269	protein_co	ENSG0000	54537	28773
FAM3A	6.67235	6.81354	7.97882	6.157	4.58547	5.26703	family with sequence similarity 3 member A [Source:HGNC Symbo	chrX:15373	NM_00117	XM_00672	.	.	NP_00116	XP_005274	protein_co	ENSG0000	60343	13749
FAM3B	0	0	0.072245	0	0.093113	0	family with sequence similarity 3 member B [Source:HGNC Symbo	chr21:4268	NM_05818	XM_01152	.	.	NP_47806	XP_011527	protein_co	ENSG0000	54097	1253
FAM3C	55.2303	62.8607	90.7656	54.7517	58.3408	79.4077	family with sequence similarity 3 member C [Source:HGNC Symbo	chr7:12098	NM_00104	XM_01151	.	.	NP_00103	XP_011514	protein_co	ENSG0000	10447	18664
FAM3D	0	0	0	0.058789	0	0	family with sequence similarity 3 member D [Source:HGNC Symbo	chr3:58619	NM_13880	XM_01153	.	.	NP_62016	XP_005264	protein_co	ENSG0000	131177	18665

FAM43A	6.79855	6.96137	6.05325	4.47457	6.35317	9.65797	family with sequence similarity 43 member A [Source:HGNC Symb	chr3:19440	NM_15369	.	.	.	NP_710157	.	protein_co	ENSG0000	131583	26888
FAM43B	0	0	0	0	0	0	family with sequence similarity 43 member B [Source:HGNC Symb	chr1:20878	NM_20733	.	.	.	NP_997217	.	protein_co	ENSG0000	163933	31791
FAM45A	8.65296	8.81593	11.2795	8.88974	10.7727	12.7898	family with sequence similarity 45 member A [Source:HGNC Symb	chr10:1208	NM_00130	XM_01153	.	.	NP_00129	XP_006717	protein_co	ENSG0000	404636	31793
FAM46A	9.02985	9.04972	7.3117	10.809	12.5587	10.4464	family with sequence similarity 46 member A [Source:HGNC Symb	chr6:82455	NM_01763	.	.	.	NP_06010	.	protein_co	ENSG0000	55603	18345
FAM46B	4.11604	4.53409	3.2035	3.63051	3.09154	2.31444	family with sequence similarity 46 member B [Source:HGNC Symb	chr1:27331	NM_05294	.	.	.	NP_443175	.	protein_co	ENSG0000	115572	28273
FAM46C	0.779945	0.677967	1.39773	0.834676	0.958103	1.60392	family with sequence similarity 46 member C [Source:HGNC Symb	chr1:11814	NM_01770	.	.	.	NP_06017	.	protein_co	ENSG0000	54855	24712
FAM46D	0	0	0	0	0	0	family with sequence similarity 46 member D [Source:HGNC Symb	chrX:79591	NM_00117	XM_01153	.	.	NP_00116	XP_011529	protein_co	ENSG0000	169966	28399
FAM47A	0	0	0	0	0	0	family with sequence similarity 47 member A [Source:HGNC Symb	chrX:34147	NM_20340	.	.	.	NP_98195	.	protein_co	ENSG0000	158724	29962
FAM47B	0	0	0	0	0	0	family with sequence similarity 47 member B [Source:HGNC Symb	chrX:34960	NM_15263	.	.	.	NP_68984	.	protein_co	ENSG0000	170062	26659
FAM47C	0	0	0	0	0	0	family with sequence similarity 47 member C [Source:HGNC Symb	chrX:37026	NM_00101	.	.	.	NP_00101	.	protein_co	ENSG0000	442444	25301
FAM47E	0.454288	0.22434	0.307479	0.568612	0.271043	0.24589	family with sequence similarity 47 member E [Source:HGNC Symb	chr4:77135	NM_00113	.	.	.	NP_00113	.	protein_co	ENSG0000	100129583	34343
FAM47E-STBD1	0.370076	0.971401	0.506294	0.346314	0.167966	0.296399	FAM47E-STBD1 readthrough [Source:HGNC Symbol;Acc:HGNC:44	chr4:77135	NM_00124	.	.	.	NP_00122	.	protein_co	ENSG0000	100631383	44667
FAM49A	0.73481	0.647328	1.04616	1.22972	1.81984	2.64111	family with sequence similarity 49 member A [Source:HGNC Symb	chr2:16730	NM_03079	XM_00671	.	.	NP_11042	XP_005262	protein_co	ENSG0000	81553	25373
FAM49B	54.4269	48.9538	61.2084	55.3274	67.9073	80.8225	family with sequence similarity 49 member B [Source:HGNC Symb	chr8:13085	NM_00125	XM_01701	.	.	NP_00124	XP_005251	protein_co	ENSG0000	51571	25216
FAM50A	29.3149	27.2397	30.603	31.7087	25.261	24.8613	family with sequence similarity 50 member A [Source:HGNC Symb	chrX:15367	NM_00469	.	.	.	NP_00469	.	protein_co	ENSG0000	9130	18786
FAM50B	0.318051	0.184637	0.319362	0.44061	0.685662	0.94077	family with sequence similarity 50 member B [Source:HGNC Symb	chr6:38496	NM_01213	XM_01701	.	.	NP_03626	XP_016866	protein_co	ENSG0000	26240	18789
FAM53A	0.411974	0.440754	0.592528	0.517857	0.576039	0.22399	family with sequence similarity 53 member A [Source:HGNC Symb	chr4:16416	NM_00101	XM_01151	.	.	NP_00101	XP_005248	protein_co	ENSG0000	152877	31860
FAM53B	10.955	11.2068	11.4419	11.4059	10.6814	9.79796	family with sequence similarity 53 member B [Source:HGNC Symb	chr10:1263	NM_01466	.	.	.	NP_05547	.	protein_co	ENSG0000	9679	28968
FAM53C	21.3582	21.5976	21.0652	22.8342	22.1409	23.737	family with sequence similarity 53 member C [Source:HGNC Symb	chr5:13762	NM_00113	.	.	.	NP_00112	.	protein_co	ENSG0000	51307	1336
FAM57A	24.7487	22.8044	19.5818	28.4239	22.7244	17.5419	family with sequence similarity 57 member A [Source:HGNC Symb	chr17:6356	NM_00131	.	.	.	NP_00130	.	protein_co	ENSG0000	79850	29646
FAM57B	0.256362	0.22613	0.232176	0.251417	0.298659	0.572148	family with sequence similarity 57 member B [Source:HGNC Symb	chr16:3003	NM_00131	XM_01702	.	.	NP_00130	XP_005255	protein_co	ENSG0000	83723	25295
FAM58A	21.7924	22.3978	19.3488	30.5392	25.6059	16.9363	family with sequence similarity 58 member A [Source:HGNC Symb	chrX:15285	NM_00113	XM_01153	.	.	NP_00112	XP_005277	protein_co	ENSG0000	92002	28434
FAM60A	61.1171	61.1115	68.1277	66.6465	77.5804	90.2733	family with sequence similarity 60 member A [Source:HGNC Symb	chr12:3143	NM_00113	XM_01152	.	.	NP_00112	XP_005253	protein_co	ENSG0000	58516	30702
FAM63A	0.806026	0.667581	0.633144	0.725687	0.676688	0.663645	family with sequence similarity 63 member A [Source:HGNC Symb	chr1:15096	NM_00104	XM_01150	.	.	NP_00103	XP_005245	protein_co	ENSG0000	55793	25648
FAM63B	2.2478	2.49096	4.77297	2.03513	2.09238	3.3066	family with sequence similarity 63 member B [Source:HGNC Symb	chr15:5906	NM_00104	XM_01152	.	.	NP_00103	XP_011519	protein_co	ENSG0000	54629	26954
FAM64A	31.9937	28.4589	30.0234	71.7654	55.1816	43.6866	family with sequence similarity 64 member A [Source:HGNC Symb	chr17:6347	NM_00119	XM_01152	.	.	NP_00118	XP_011522	protein_co	ENSG0000	54478	25483
FAM65A	15.7718	15.9805	15.6139	19.4436	18.4698	14.3932	family with sequence similarity 65 member A [Source:HGNC Symb	chr16:6756	NM_00119	XM_01702	.	.	NP_00118	XP_011521	protein_co	ENSG0000	79567	25836
FAM65B	0	0.057573	0.01113	0	0	0.012921	family with sequence similarity 65 member B [Source:HGNC Symb	chr6:24797	NM_00128	XM_01701	.	.	NP_00127	XP_006715	protein_co	ENSG0000	9750	13872
FAM65C	0.032676	0.048556	0.07061	0.08773	0.037736	0.035705	family with sequence similarity 65 member C [Source:HGNC Symb	chr20:4920	NM_00129	XM_01152	.	.	NP_00127	XP_005260	protein_co	ENSG0000	140876	16168
FAM69A	4.72366	4.12125	5.93412	3.47704	4.27266	6.27684	family with sequence similarity 69 member A [Source:HGNC Symb	chr1:93297	NM_00100	.	.	.	NP_00100	.	protein_co	ENSG0000	388650	32213
FAM69B	12.1119	12.6878	14.8388	13.1184	11.7259	10.1425	family with sequence similarity 69 member B [Source:HGNC Symb	chr9:13960	NM_15242	.	.	.	NP_68963	XP_006717	protein_co	ENSG0000	138311	28290
FAM69C	0	0	0	0	0	0	family with sequence similarity 69 member C [Source:HGNC Symb	chr18:7210	NM_00104	.	.	.	NP_00103	.	protein_co	ENSG0000	125704	31729
FAM71A	0	0	0	0	0	0	family with sequence similarity 71 member A [Source:HGNC Symb	chr1:21279	NM_15360	.	.	.	NP_70583	.	protein_co	ENSG0000	149647	26541
FAM71B	0	0	0	0	0	0	family with sequence similarity 71 member B [Source:HGNC Symb	chr5:15658	NM_13089	.	.	.	NP_57096	.	protein_co	ENSG0000	153745	28397
FAM71C	0	0	0	0	0	0	family with sequence similarity 71 member C [Source:HGNC Symb	chr12:9903	NM_15336	.	.	.	NP_69919	.	protein_co	ENSG0000	196472	28594
FAM71D	0	0	0	0.038343	0	0.034394	family with sequence similarity 71 member D [Source:HGNC Symb	chr14:6765	NM_17352	XM_01153	.	.	NP_77579	XP_011534	protein_co	ENSG0000	161142	20101
FAM71E1	9.4851	8.66207	10.9398	21.4842	12.9259	14.133	family with sequence similarity 71 member E1 [Source:HGNC Sym	chr19:5097	NM_00130	XM_00525	.	.	NP_00129	XP_005258	protein_co	ENSG0000	112703	25107
FAM71E2	0	0	0.020459	0	0	0	family with sequence similarity 71 member E2 [Source:HGNC Sym	chr19:5586	NM_00114	.	.	.	NP_00113	.	protein_co	ENSG0000	284418	25278
FAM71F1	0.076166	0	0	0	0	0	family with sequence similarity 71 member F1 [Source:HGNC Sym	chr7:12834	NM_00128	.	.	.	NP_00126	XP_016868	protein_co	ENSG0000	84691	30704
FAM72A	4.69792	6.3567	5.96159	6.96795	7.74032	8.69127	family with sequence similarity 72 member A [Source:HGNC Symb	chr1:20613	NM_00112	XM_01150	.	.	NP_00111	XP_006711	protein_co	ENSG0000	729533	24044
FAM72B	2.44127	2.37055	2.53745	3.05683	4.75693	4.29967	family with sequence similarity 72 member B [Source:HGNC Symb	chr1:12083	NM_00110	XM_01154	.	.	NP_00109	XP_011540	protein_co	ENSG0000	653820	24805
FAM72C	0.484052	0.74965	0.506622	0.570355	0.625694	0.985198	family with sequence similarity 72 member C [Source:HGNC Symb	chr1:14944	NM_00128	.	.	.	NP_00127	.	protein_co	ENSG0000	554282	30602
FAM72D	3.84393	2.66852	2.59877	3.47576	3.9319	4.41683	family with sequence similarity 72 member D [Source:HGNC Symb	chr1:14389	NM_20741	.	.	.	NP_99730	.	protein_co	ENSG0000	728833	33593
FAM73A	4.15023	4.24055	3.65274	2.7195	3.50014	3.07339	mitoguardin 1 [Source:HGNC Symbol;Acc:HGNC:24741]	chr1:78245	NM_00127	XM_01700	.	.	NP_00125	XP_005270	protein_co	ENSG0000	374986	24741
FAM73B	7.05775	7.16864	7.42978	6.41726	6.37043	4.60561	mitoguardin 2 [Source:HGNC Symbol;Acc:HGNC:23621]	chr9:13179	NM_03280	XM_01151	.	.	NP_11619	XP_005252	protein_co	ENSG0000	84895	23621
FAM76A	3.24986	4.09284	3.88038	3.36913	3.47371	3.72614	family with sequence similarity 76 member A [Source:HGNC Symb	chr1:28052	NM_00114	.	.	.	NP_00113	.	protein_co	ENSG0000	199870	28530
FAM76B	5.86281	6.06712	5.19325	4.26125	5.709	6.44958	family with sequence similarity 76 member B [Source:HGNC Symb	chr11:9550	NM_14466	XM_01154	.	.	NP_65326	XP_005273	protein_co	ENSG0000	143684	28492
FAM78A	1.68994	2.30596	3.05525	1.59909	2.06602	2.17885	family with sequence similarity 78 member A [Source:HGNC Symb	chr9:13413	NM_03338	.	.	.	NP_20374	.	protein_co	ENSG0000	286336	25465
FAM78B	0.524318	0.781468	1.04201	0.791349	0.914709	0.746436	family with sequence similarity 78 member B [Source:HGNC Symb	chr1:16603	NM_00101	XM_01700	.	.	NP_00101	XP_016855	protein_co	ENSG0000	149297	13495
FAM81A	1.51353	2.10822	1.73886	2.67696	2.0868	1.96132	family with sequence similarity 81 member A [Source:HGNC Symb	chr15:5973	NM_15245	XM_01702	.	.	NP_68966	XP_005254	protein_co	ENSG0000	145773	28379
FAM81B	0	0.04312	0.142611	0.092085	0.090072	0.120238	family with sequence similarity 81 member B [Source:HGNC Symb	chr5:94727	NM_15254	XM_00527	.	.	NP_68976	XP_005271	protein_co	ENSG0000	153643	26335
FAM83A	0.238505	0.054765	0.25445	0.088484	0.169454	0.415786	family with sequence similarity 83 member A [Source:HGNC Symb	chr8:12419	NM_00128	.	.	.	NP_00127	.	protein_co	ENSG0000	84985	28210
FAM83B	4.31393	4.03881	4.32977	3.31587	4.01347	4.39008	family with sequence similarity 83 member B [Source:HGNC Symb	chr6:54711	NM_00101	XM_01151	.	.	NP_00101	XP_006715	protein_co	ENSG0000	222584	21357
FAM83C	0	0.020961	0	0.022612	0	0.022908	family with sequence similarity 83 member C [Source:HGNC Symb	chr20:3387	NM_17846	.	.	.	NP_84856	.	protein_co	ENSG0000	128876	16121
FAM83D	34.7191	33.2853	31.8359	60.9297	53.7219	34.7635	family with sequence similarity 83 member D [Source:HGNC Symb	chr20:3755	NM_03091	.	.	.	NP_11218	.	protein_co	ENSG0000	81610	16122
FAM83E	0.161375	0.308204	0.274068	0.563309	0.380148	0.427735	family with sequence similarity 83 member E [Source:HGNC Symb	chr19:4910	NM_01770	XM_00525	.	.	NP_06017	XP_005259	protein_co	ENSG0000	54854	25972
FAM83F	9.25218	10.8337	11.1724	9.1225	9.18987	7.09056	family with sequence similarity 83 member F [Source:HGNC Symb	chr22:4039	NM_13843	.	.	.	NP_61244	.	protein_co	ENSG0000	113828	25148
FAM83G	17.8143	17.2433	15.866	18.1896	17.775	16.8199	family with sequence similarity 83 member G [Source:HGNC Symb	chr17:1885	NM_00103	XM_01702	.	.	NP_00103	XP_016880	protein_co	ENSG0000	644815	32554
FAM83H	50.275	53.5951	44.369	42.048	37.3445	30.6088	family with sequence similarity 83 member H [Source:HGNC Symb	chr8:14480	NM_19848	XM_00525	.	.	NP_94089	XP_005250	protein_co	ENSG0000	286077	24797

FAM83H-AS1	19.8642	18.849	19.8531	18.702	20.6717	17.4139	FAM83H antisense RNA 1 (head to head) [Source:HGNC Symbol;A	chr8:14481	.	.	.	.	.	.	protein_co	ENSG0000	.	48720
FAM84A	0	0	0	0	0	0	family with sequence similarity 84 member A [Source:HGNC Symb	chr2:14772	NM_14517	.	.	.	NP_660158	.	protein_co	ENSG0000	151354	20743
FAM84B	14.5264	16.793	13.5859	10.9231	10.694	23.1387	family with sequence similarity 84 member B [Source:HGNC Symb	chr8:12756	NM_17491	XM_01701	.	.	NP_777571	XP_016868	protein_co	ENSG0000	157638	24166
FAM86B1	2.14184	1.69548	2.01968	1.79304	2.50034	2.27559	family with sequence similarity 86 member B1 [Source:HGNC Syn	chr8:12039	NM_00108	XM_01154	.	.	NP_001077	XP_006716	protein_co	ENSG0000	100287013	28268
FAM86B2	0.309766	0.605454	0.381394	0.230503	0.150132	0.133868	family with sequence similarity 86 member B2 [Source:HGNC Syn	chr8:12283	NM_00113	XM_01154	.	.	NP_001131	XP_005273	protein_co	ENSG0000	653333	32222
FAM86C1	8.01188	8.33816	7.23728	7.40884	8.60817	7.34016	family with sequence similarity 86 member C1 [Source:HGNC Syn	chr11:7149	NM_00109	.	.	.	NP_001093	.	protein_co	ENSG0000	55199	25561
FAM89A	17.0134	17.1314	20.385	21.6635	22.888	21.9636	family with sequence similarity 89 member A [Source:HGNC Symb	chr1:23115	NM_19855	.	NR_031593	.	NP_940954	.	protein_co	ENSG0000	100302132	25057
FAM89B	41.8864	40.5254	48.7442	44.2915	32.4904	33.9818	family with sequence similarity 89 member B [Source:HGNC Symb	chr11:6533	NM_00109	.	.	.	NP_001092	.	protein_co	ENSG0000	23625	16708
FAM8A1	7.65651	7.21684	9.5973	7.20525	8.99272	11.8617	family with sequence similarity 8 member A1 [Source:HGNC Symb	chr6:17600	NM_01625	.	.	.	NP_057339	.	protein_co	ENSG0000	51439	16372
FAM90A1	0.158648	0.275271	0.336698	0.149369	0.158384	0.060084	family with sequence similarity 90 member A1 [Source:HGNC Syn	chr12:8373	NM_00131	XM_01701	.	.	NP_001306	XP_011519	protein_co	ENSG0000	55138	25526
FAM90A26	0	0	0	0	0	0	family with sequence similarity 90 member A26 [Source:HGNC Sy	chr4:91721	.	.	.	.	.	.	protein_co	ENSG0000	.	43746
FAM91A1	29.8146	31.3874	30.0421	32.1261	36.3292	36.69	family with sequence similarity 91 member A1 [Source:HGNC Syn	chr8:12478	NM_00131	XM_01151	.	.	NP_001304	.	protein_co	ENSG0000	157769	26306
FAM92A1	51.9761	60.5074	50.6777	61.6341	54.9345	64.0154	family with sequence similarity 92 member A1 [Source:HGNC Syn	chr8:94712	NM_00128	XM_00525	.	.	NP_001269	.	protein_co	ENSG0000	137392	30452
FAM92B	0.472726	0.409402	0.618598	0.344628	0.180247	0.334393	family with sequence similarity 92 member B [Source:HGNC Symb	chr16:8513	NM_19849	XM_01152	.	.	NP_940893	XP_005255	protein_co	ENSG0000	339145	24781
FAM96A	76.5552	78.0332	59.5274	79.8846	69.2266	57.006	family with sequence similarity 96 member A [Source:HGNC Symb	chr15:6436	NM_00101	.	.	.	NP_001014	.	protein_co	ENSG0000	84191	26235
FAM96B	125.545	126.13	108.436	157.757	109.085	91.3754	family with sequence similarity 96 member B [Source:HGNC Symb	chr16:6696	NM_01606	.	.	.	NP_057146	.	protein_co	ENSG0000	51647	24261
FAM98A	28.0895	28.1309	27.028	30.0377	35.2651	22.3004	family with sequence similarity 98 member A [Source:HGNC Symb	chr2:33808	NM_00130	.	.	.	NP_001291	.	protein_co	ENSG0000	25940	24520
FAM98B	21.6768	21.2527	21.2353	20.977	25.4631	22.7786	family with sequence similarity 98 member B [Source:HGNC Symb	chr15:3874	NM_17361	.	.	.	NP_775882	.	protein_co	ENSG0000	283742	26773
FAM98C	21.7292	22.263	20.9576	23.4875	21.5461	13.0182	family with sequence similarity 98 member C [Source:HGNC Symb	chr19:3889	NM_17490	.	.	.	NP_777565	.	protein_co	ENSG0000	147965	27119
FAM9A	0	0	0	0	0	0	family with sequence similarity 9 member A [Source:HGNC Symbo	chrX:87588	NM_00117	XM_01154	.	.	NP_001164	XP_011543	protein_co	ENSG0000	171482	18403
FAM9B	0	0	0	0	0	0	family with sequence similarity 9 member B [Source:HGNC Symbo	chrX:89922	NM_20584	XM_01154	.	.	NP_995321	XP_011543	protein_co	ENSG0000	171483	18404
FAM9C	0	0	0	0	0	0.015747	family with sequence similarity 9 member C [Source:HGNC Symbo	chrX:13053	NM_17490	XM_01702	.	.	NP_777561	XP_005274	protein_co	ENSG0000	171484	18405
FAN1	9.40451	8.91902	9.43332	8.41898	10.7546	9.20415	FANCD2/FANCI-associated nuclease 1 [Source:HGNC Symbol;Acc:	chr15:3119	NM_00114	XM_01152	.	.	NP_001139	XP_005254	protein_co	ENSG0000	22909	29170
FANCA	21.0102	21.7184	20.4571	21.6606	19.8039	19.1281	Fanconi anemia complementation group A [Source:HGNC Symbol	chr16:8977	NM_00013	XM_01152	.	.	NP_000126	XP_005256	protein_co	ENSG0000	2175	3582
FANCB	2.22218	1.54173	2.43092	1.58742	3.04451	2.32555	Fanconi anemia complementation group B [Source:HGNC Symbol	chrX:14861	NM_00101	XM_01702	.	.	NP_001018	XP_016884	protein_co	ENSG0000	2187	3583
FANCC	6.96521	7.21961	6.07489	6.54409	6.45601	6.01802	Fanconi anemia complementation group C [Source:HGNC Symbol	chr9:97861	NM_00013	XM_01151	.	.	NP_000127	XP_006717	protein_co	ENSG0000	2176	3584
FANCD2	16.962	16.4461	15.7786	16.769	19.1737	14.8281	Fanconi anemia complementation group D2 [Source:HGNC Symbo	chr3:10068	NM_00101	XM_00671	.	.	NP_001018	XP_005265	protein_co	ENSG0000	2177	3585
FANCD2OS	0	0	0	0	0	0	FANCD2 opposite strand [Source:HGNC Symbol;Acc:HGNC:28623]	chr3:10068	NM_00116	XM_01153	.	.	NP_001158	XP_011531	protein_co	ENSG0000	115795	28623
FANCE	10.8132	11.2054	11.1994	12.6304	11.5462	10.4262	Fanconi anemia complementation group E [Source:HGNC Symbol	chr6:35420	NM_02192	XM_01151	.	.	NP_068741	XP_005248	protein_co	ENSG0000	2178	3586
FANCF	3.36594	3.99429	2.83291	4.26132	3.1669	2.8328	Fanconi anemia complementation group F [Source:HGNC Symbol	chr11:2264	NM_02272	.	.	.	NP_073562	.	protein_co	ENSG0000	2188	3587
FANCG	23.985	25.2703	19.5622	29.2728	24.0639	18.4342	Fanconi anemia complementation group G [Source:HGNC Symbo	chr9:35073	NM_00462	.	.	.	NP_004620	.	protein_co	ENSG0000	2189	3588
FANCI	33.9019	34.4382	29.8202	34.1582	37.9689	32.1527	Fanconi anemia complementation group I [Source:HGNC Symbol;	chr15:8978	NM_00111	XM_01152	.	.	NP_001106	XP_011520	protein_co	ENSG0000	55215	25568
FANCL	11.9281	10.4525	13.5341	9.6182	12.2868	10.236	Fanconi anemia complementation group L [Source:HGNC Symbol,	chr2:58134	NM_00111	XM_01153	.	.	NP_001108	XP_005264	protein_co	ENSG0000	55120	20748
FANCM	2.07125	1.60282	2.74167	2.46633	3.4481	2.67513	Fanconi anemia complementation group M [Source:HGNC Symbo	chr14:4560	NM_00130	XM_01153	.	.	NP_001295	XP_005267	protein_co	ENSG0000	57697	23168
FANK1	1.74217	2.11038	2.26928	2.04417	2.22755	2.63274	fibronectin type III and ankyrin repeat domains 1 [Source:HGNC S	chr10:1275	NM_14523	XM_01154	.	.	NP_660278	XP_005270	protein_co	ENSG0000	92565	23527
FAP	0.029814	0.032611	0	0.034766	0	0	fibroblast activation protein alpha [Source:HGNC Symbol;Acc:HGI	chr2:16302	NM_00129	XM_01151	.	.	NP_001278	XP_011509	protein_co	ENSG0000	2191	3590
FAR1	25.385	26.9168	26.9081	24.6363	27.822	29.5372	fatty acyl-CoA reductase 1 [Source:HGNC Symbol;Acc:HGNC:2622	chr11:1369	NM_03222	XM_01152	.	.	NP_115604	XP_011518	protein_co	ENSG0000	84188	26222
FAR2	3.38707	3.61289	3.31491	3.44381	3.39634	3.14438	fatty acyl-CoA reductase 2 [Source:HGNC Symbol;Acc:HGNC:2553	chr12:2930	NM_00127	XM_01152	.	.	NP_001258	XP_011519	protein_co	ENSG0000	55711	25531
FARP1	7.13007	7.68756	11.0509	8.87353	8.16591	10.5367	FERM, ARH/RhoGEF and pleckstrin domain protein 1 [Source:HGI	chr13:9879	NM_00100	XM_01152	.	.	NP_001001	XP_011519	protein_co	ENSG0000	10160	3591
FARP2	4.53459	4.74402	5.43564	6.167	5.51283	4.24216	FERM, ARH/RhoGEF and pleckstrin domain protein 2 [Source:HGI	chr2:24229	NM_00128	XM_01151	.	.	NP_001269	XP_011510	protein_co	ENSG0000	9855	16460
FARS2	6.96459	5.77758	6.24791	6.45713	4.96348	5.02498	phenylalanyl-tRNA synthetase 2, mitochondrial [Source:HGNC Sy	chr6:52612	NM_00656	XM_00524	.	.	NP_001305	XP_005248	protein_co	ENSG0000	10667	21062
FARSA	73.829	73.7204	68.0886	103.824	84.1435	55.1035	phenylalanyl-tRNA synthetase alpha subunit [Source:HGNC Symb	chr19:1303	NM_00446	.	.	.	NP_004452	.	protein_co	ENSG0000	2193	3592
FARSB	32.2268	31.7281	30.9399	34.5225	37.0711	32.5566	phenylalanyl-tRNA synthetase beta subunit [Source:HGNC Symbo	chr2:22343	NM_00568	XM_01151	.	.	NP_005678	XP_006712	protein_co	ENSG0000	10056	17800
FAS	10.2812	9.70435	9.78486	8.07965	11.545	6.62121	Fas cell surface death receptor [Source:HGNC Symbol;Acc:HGNC:	chr10:9075	NM_00004	XM_01153	.	.	NP_000034	XP_011538	protein_co	ENSG0000	355	11920
FASLG	0	0	0	0	0	0	Fas ligand [Source:HGNC Symbol;Acc:HGNC:11936]	chr1:17262	NM_00063	.	.	.	NP_000630	.	protein_co	ENSG0000	356	11936
FASN	139.726	154.102	195.389	179.68	175.72	173.675	fatty acid synthase [Source:HGNC Symbol;Acc:HGNC:3594]	chr17:8003	NM_00410	XM_01152	.	.	NP_004095	XP_011521	protein_co	ENSG0000	2194	3594
FASTK	37.4159	35.7671	33.1886	41.2714	32.7147	30.3013	Fas activated serine/threonine kinase [Source:HGNC Symbol;Acc:	chr7:15077	NM_00125	XM_01151	.	.	NP_001245	XP_005249	protein_co	ENSG0000	10922	24676
FASTKD1	10.4905	9.27264	11.8914	8.89807	12.9356	11.162	FAST kinase domains 1 [Source:HGNC Symbol;Acc:HGNC:26150]	chr2:17038	NM_00128	XM_00671	.	.	NP_001268	XP_006712	protein_co	ENSG0000	79675	26150
FASTKD2	19.9595	17.1083	19.2018	19.4002	27.0121	18.3561	FAST kinase domains 2 [Source:HGNC Symbol;Acc:HGNC:29160]	chr2:20760	NM_00113	.	.	.	NP_001129	.	protein_co	ENSG0000	22868	29160
FASTKD3	7.99194	6.93013	6.58565	6.832	7.91987	6.62601	FAST kinase domains 3 [Source:HGNC Symbol;Acc:HGNC:28758]	chr5:78592	NM_02409	XM_00671	.	.	NP_076996	XP_006714	protein_co	ENSG0000	79072	28758
FASTKD5	17.1235	16.343	18.2665	16.0645	17.9061	14.2431	FAST kinase domains 5 [Source:HGNC Symbol;Acc:HGNC:25790]	chr20:3088	NM_02182	.	.	.	NP_068598	.	protein_co	ENSG0000	60493	25790
FAT1	128.209	136.604	165.841	28.6937	35.1816	37.2724	FAT atypical cadherin 1 [Source:HGNC Symbol;Acc:HGNC:3595]	chr4:18750	NM_00524	XM_00671	.	.	NP_005236	XP_005262	protein_co	ENSG0000	2195	3595
FAT2	0.029735	0.034848	0.058995	0.018669	0.009661	0.026779	FAT atypical cadherin 2 [Source:HGNC Symbol;Acc:HGNC:3596]	chr5:15088	NM_00144	XM_01700	.	.	NP_001438	XP_006714	protein_co	ENSG0000	2196	3596
FAT3	0	0	0.009566	0	0	0	FAT atypical cadherin 3 [Source:HGNC Symbol;Acc:HGNC:23112]	chr11:9208	NM_00100	XM_01701	.	.	NP_001008	XP_016872	protein_co	ENSG0000	120114	23112
FAT4	0.25832	0.280824	0.599401	0.180564	0.276862	0.556939	FAT atypical cadherin 4 [Source:HGNC Symbol;Acc:HGNC:23109]	chr4:12623	NM_00129	XM_01153	.	.	NP_001278	XP_011530	protein_co	ENSG0000	79633	23109
FATE1	0	0	0	0	0	0	fetal and adult testis expressed 1 [Source:HGNC Symbol;Acc:HGN	chrX:15088	NM_03308	.	.	.	NP_149076	.	protein_co	ENSG0000	89885	24683
FAU	786.463	691.657	682.788	1017.23	728.078	628.581	FAU, ubiquitin like and ribosomal protein S30 fusion [Source:HGN	chr11:6488	NM_00199	.	.	.	NP_001988	.	protein_co	ENSG0000	2197	3597
FAXC	2.30175	2.65406	2.22677	3.17961	3.06297	2.71889	failed axon connections homolog [Source:HGNC Symbol;Acc:HGN	chr6:99719	NM_03251	XM_01153	.	.	NP_115900	.	protein_co	ENSG0000	84553	20742
FAXDC2	0.043835	0	0.019032	0	0	0	fatty acid hydroxylase domain containing 2 [Source:HGNC Symbo	chr5:15419	NM_03238	XM_00671	.	.	NP_115761	XP_006714	protein_co	ENSG0000	10826	1334

FBF1	4.25554	4.14316	4.2751	3.06967	3.30379	3.45721	Fas binding factor 1 [Source:HGNC Symbol;Acc:HGNC:24674]	chr17:7390	NM_00108	.	.	.	NP_00107	.	protein_co	ENSG0000	85302	24674
FBL	202.641	198.122	189.918	243.537	194.876	188.355	fibrillarin [Source:HGNC Symbol;Acc:HGNC:3599]	chr19:4032	NM_00143	XM_01152	.	.	NP_00142	XP_005258	protein_co	ENSG0000	2091	3599
FBLIM1	1.33644	1.22099	1.96973	1.1283	0.819728	1.52082	filamin binding LIM protein 1 [Source:HGNC Symbol;Acc:HGNC:24	chr1:16083	NM_00102	XM_01700	.	.	NP_00101	XP_005245	protein_co	ENSG0000	54751	24686
FBLL1	0.168488	0.079391	0	0.176581	0	0.09726	fibrillarin-like 1 [Source:HGNC Symbol;Acc:HGNC:35458]	chr5:16795	.	.	.	.	.	.	protein_co	ENSG0000	.	35458
FBLN1	8.61961	8.85621	11.7058	9.97652	9.32653	10.1776	fibulin 1 [Source:HGNC Symbol;Acc:HGNC:3600]	chr22:4589	NM_00199	.	.	.	NP_00198	.	protein_co	ENSG0000	2192	3600
FBLN2	0.018344	0.071883	0.047208	0.038982	0	0.082037	fibulin 2 [Source:HGNC Symbol;Acc:HGNC:3601]	chr3:13590	NM_00100	XM_00671	.	.	NP_00100	XP_006713	protein_co	ENSG0000	2199	3601
FBLN5	1.39202	1.31503	2.05563	0.928284	1.11215	1.34944	fibulin 5 [Source:HGNC Symbol;Acc:HGNC:3602]	chr14:9233	NM_00632	XM_01153	.	.	NP_00632	XP_005267	protein_co	ENSG0000	10516	3602
FBLN7	0.089549	0.088906	0.042505	0.053576	0	0	fibulin 7 [Source:HGNC Symbol;Acc:HGNC:26740]	chr2:11289	NM_00112	XM_01151	.	.	NP_00112	XP_011508	protein_co	ENSG0000	129804	26740
FBN1	0.167325	0.120497	0.165142	0.090268	0.149762	0.198566	fibrillin 1 [Source:HGNC Symbol;Acc:HGNC:3603]	chr15:4870	NM_00013	.	.	.	NP_00012	.	protein_co	ENSG0000	2200	3603
FBN2	0.116223	0.136866	0.100652	0.043703	0.043344	0.00679	fibrillin 2 [Source:HGNC Symbol;Acc:HGNC:3604]	chr5:12759	NM_00199	.	.	.	NP_00199	.	protein_co	ENSG0000	2201	3604
FBN3	0.763167	0.814271	1.29794	0.862736	0.965979	1.0198	fibrillin 3 [Source:HGNC Symbol;Acc:HGNC:18794]	chr19:8130	NM_00132	XM_01702	.	.	NP_00130	XP_011526	protein_co	ENSG0000	84467	18794
FBP1	0	0.056521	0	0.182993	0.194649	0	fructose-bisphosphatase 1 [Source:HGNC Symbol;Acc:HGNC:3606	chr9:97365	NM_00050	XM_00671	.	.	NP_00049	XP_006717	protein_co	ENSG0000	2203	3606
FBP2	0	0.059209	0	0	0.067158	0	fructose-bisphosphatase 2 [Source:HGNC Symbol;Acc:HGNC:3607	chr9:97321	NM_00383	.	.	.	NP_00382	.	protein_co	ENSG0000	8789	3607
FBRS	22.4593	22.4016	20.8131	19.264	17.373	18.5841	fibrosin [Source:HGNC Symbol;Acc:HGNC:20442]	chr16:3066	NM_00110	XM_01154	.	.	NP_00109	XP_011544	protein_co	ENSG0000	64319	20442
FBRSL1	20.0483	19.6787	19.3313	22.9023	20.4152	18.5877	fibrosin like 1 [Source:HGNC Symbol;Acc:HGNC:29308]	chr12:1330	NM_00114	XM_01153	.	.	NP_00113	XP_005266	protein_co	ENSG0000	57666	29308
FBXL12	15.3472	14.7863	13.5088	16.3804	12.9604	14.0552	F-box and leucine rich repeat protein 12 [Source:HGNC Symbol;A	chr19:9920	NM_00131	XM_01152	.	.	NP_00130	XP_005260	protein_co	ENSG0000	54850	13611
FBXL13	0.19051	0.29148	0.289323	0.162845	0.200158	0.298382	F-box and leucine rich repeat protein 13 [Source:HGNC Symbol;A	chr7:10245	NM_00111	XM_01701	.	.	NP_00110	XP_005250	protein_co	ENSG0000	222235	21658
FBXL14	8.49415	10.1546	8.65931	11.8044	10.2954	7.48975	F-box and leucine rich repeat protein 14 [Source:HGNC Symbol;A	chr12:1675	NM_15244	XM_01152	.	.	NP_68965	XP_016874	protein_co	ENSG0000	144699	28624
FBXL15	9.93778	11.2989	9.0006	10.2951	8.60703	6.92883	F-box and leucine rich repeat protein 15 [Source:HGNC Symbol;A	chr10:1041	NM_02432	XM_00527	.	.	NP_07730	XP_005270	protein_co	ENSG0000	79176	28155
FBXL16	24.6197	27.3305	24.0875	20.9362	19.0557	20.8863	F-box and leucine rich repeat protein 16 [Source:HGNC Symbol;A	chr16:7424	NM_15335	.	.	.	NP_69918	.	protein_co	ENSG0000	146330	14150
FBXL17	3.20164	3.72702	4.32691	1.80734	2.19018	3.44354	F-box and leucine rich repeat protein 17 [Source:HGNC Symbol;A	chr5:10719	NM_00116	XM_00527	.	.	NP_00115	XP_005272	protein_co	ENSG0000	64839	13615
FBXL18	6.77697	6.36231	7.18169	8.10591	8.92906	7.49846	F-box and leucine rich repeat protein 18 [Source:HGNC Symbol;A	chr7:55200	NM_02496	.	.	.	NP_07923	XP_011513	protein_co	ENSG0000	80028	21874
FBXL19	22.3724	20.7767	20.5404	14.7184	13.2969	10.2217	F-box and leucine rich repeat protein 19 [Source:HGNC Symbol;A	chr16:3093	NM_00109	.	.	.	NP_00109	.	protein_co	ENSG0000	54620	25300
FBXL2	5.78169	4.83969	4.99333	4.09551	4.37171	4.8225	F-box and leucine rich repeat protein 2 [Source:HGNC Symbol;Ac	chr3:33318	NM_00117	XM_00526	.	.	NP_00116	XP_005265	protein_co	ENSG0000	25827	13598
FBXL20	3.23431	2.44194	3.41226	1.69896	1.86241	2.95642	F-box and leucine rich repeat protein 20 [Source:HGNC Symbol;A	chr17:3740	NM_00118	XM_01152	.	.	NP_00117	XP_005257	protein_co	ENSG0000	84961	24679
FBXL22	0.453181	0.400875	0.092877	0.266447	0.283487	0.191041	F-box and leucine rich repeat protein 22 [Source:HGNC Symbol;A	chr15:6388	NM_20337	.	.	.	NP_97630	.	protein_co	ENSG0000	283807	27537
FBXL3	8.96637	8.89582	8.99951	7.93425	9.71842	9.13441	F-box and leucine rich repeat protein 3 [Source:HGNC Symbol;Ac	chr13:7757	NM_01215	XM_00526	.	.	NP_03629	XP_005266	protein_co	ENSG0000	26224	13599
FBXL4	6.51355	6.2746	7.24037	4.51967	6.48277	7.21663	F-box and leucine rich repeat protein 4 [Source:HGNC Symbol;Ac	chr6:99316	NM_00127	XM_00526	.	.	NP_00126	XP_005266	protein_co	ENSG0000	26235	13601
FBXL5	8.66005	8.19821	10.036	8.41332	11.4795	13.3697	F-box and leucine rich repeat protein 5 [Source:HGNC Symbol;Ac	chr4:15606	NM_00119	XM_01151	.	.	NP_00118	XP_006714	protein_co	ENSG0000	26234	13602
FBXL6	45.2853	41.7834	37.4178	41.5659	35.2357	30.784	F-box and leucine rich repeat protein 6 [Source:HGNC Symbol;Ac	chr8:14557	NM_01216	.	.	.	NP_03629	.	protein_co	ENSG0000	26233	13603
FBXL7	0.021611	0	0	0	0	0	F-box and leucine rich repeat protein 7 [Source:HGNC Symbol;Ac	chr5:15500	NM_00127	XM_01151	.	.	NP_00126	XP_011512	protein_co	ENSG0000	23194	13604
FBXL8	6.10571	10.3543	10.7654	5.07982	5.82362	3.38008	F-box and leucine rich repeat protein 8 [Source:HGNC Symbol;Ac	chr16:6718	NM_01837	.	.	.	NP_06084	.	protein_co	ENSG0000	55336	17875
FBXO10	3.15504	3.2269	2.24084	1.91708	1.95538	1.88936	F-box protein 10 [Source:HGNC Symbol;Acc:HGNC:13589]	chr9:37510	NM_01216	XM_01701	.	.	NP_03629	XP_011516	protein_co	ENSG0000	26267	13589
FBXO11	13.6208	12.3454	13.6799	11.585	16.9708	20.7348	F-box protein 11 [Source:HGNC Symbol;Acc:HGNC:13590]	chr2:48010	NM_00119	XM_01700	.	.	NP_00117	XP_005264	protein_co	ENSG0000	80204	13590
FBXO15	0.24912	0.313424	0.386891	0.111509	0.616087	0.348822	F-box protein 15 [Source:HGNC Symbol;Acc:HGNC:13617]	chr18:7174	NM_00114	XM_01702	.	.	NP_00113	XP_011524	protein_co	ENSG0000	201456	13617
FBXO16	5.97301	6.43405	5.32331	7.92895	5.89552	5.60805	F-box protein 16 [Source:HGNC Symbol;Acc:HGNC:13618]	chr8:28285	NM_00125	.	.	.	NP_00124	.	protein_co	ENSG0000	157574	13618
FBXO17	6.49653	7.0405	6.3956	7.63664	6.25981	6.10415	F-box protein 17 [Source:HGNC Symbol;Acc:HGNC:18754]	chr19:3940	NM_02490	.	.	.	NP_07918	.	protein_co	ENSG0000	115290	18754
FBXO18	20.3255	19.5939	23.2827	21.8361	18.8463	19.8753	F-box protein, helicase, 18 [Source:HGNC Symbol;Acc:HGNC:1362	chr10:5932	NM_00125	XM_01701	.	.	NP_00124	XP_011518	protein_co	ENSG0000	84893	13620
FBXO2	58.1875	61.6842	75.4936	36.8228	28.7401	37.6088	F-box protein 2 [Source:HGNC Symbol;Acc:HGNC:13581]	chr1:11708	NM_01216	.	.	.	NP_03630	.	protein_co	ENSG0000	26232	13581
FBXO21	18.7704	20.3991	18.7544	18.5908	20.3616	21.6214	F-box protein 21 [Source:HGNC Symbol;Acc:HGNC:13592]	chr12:1175	NM_01500	.	.	.	NP_05581	.	protein_co	ENSG0000	23014	13592
FBXO22	21.2982	22.3318	22.1505	19.7894	18.5988	17.2523	F-box protein 22 [Source:HGNC Symbol;Acc:HGNC:13593]	chr15:7619	NM_01217	.	.	.	NP_03630	.	protein_co	ENSG0000	26263	13593
FBXO24	0.571186	0.556048	0.209426	0.338264	0.408488	0.766753	F-box protein 24 [Source:HGNC Symbol;Acc:HGNC:13595]	chr7:10018	NM_00116	XM_01151	.	.	NP_00115	XP_005250	protein_co	ENSG0000	26261	13595
FBXO25	5.95741	5.83356	6.36106	4.42096	4.79519	6.5212	F-box protein 25 [Source:HGNC Symbol;Acc:HGNC:13596]	chr8:35680	NM_01217	XM_01701	.	.	NP_03630	XP_011533	protein_co	ENSG0000	26260	13596
FBXO27	6.82232	6.56077	5.14062	5.61625	4.93751	5.05295	F-box protein 27 [Source:HGNC Symbol;Acc:HGNC:18753]	chr19:3951	NM_17882	.	.	.	NP_84914	.	protein_co	ENSG0000	126433	18753
FBXO28	17.7544	18.0513	17.7239	19.1715	19.7002	16.4753	F-box protein 28 [Source:HGNC Symbol;Acc:HGNC:29046]	chr1:22430	NM_00113	.	.	.	NP_00112	.	protein_co	ENSG0000	23219	29046
FBXO3	15.2438	13.43	17.0972	12.6489	14.8791	16.171	F-box protein 3 [Source:HGNC Symbol;Acc:HGNC:13582]	chr11:3376	NM_01217	XM_01151	.	.	NP_03630	XP_011518	protein_co	ENSG0000	26273	13582
FBXO30	5.64614	5.41449	4.80023	5.068	5.82788	5.25597	F-box protein 30 [Source:HGNC Symbol;Acc:HGNC:15600]	chr6:14611	NM_03214	XM_01153	.	.	NP_11552	XP_005267	protein_co	ENSG0000	84085	15600
FBXO31	11.941	11.5477	11.1041	11.0428	10.7479	9.46059	F-box protein 31 [Source:HGNC Symbol;Acc:HGNC:16510]	chr16:8733	NM_02473	.	.	.	NP_07901	.	protein_co	ENSG0000	79791	16510
FBXO32	1.73755	1.93294	2.09459	1.35569	1.20006	0.935051	F-box protein 32 [Source:HGNC Symbol;Acc:HGNC:16731]	chr8:12451	NM_00124	.	.	.	NP_00122	.	protein_co	ENSG0000	114907	16731
FBXO33	8.42936	8.50933	8.71125	8.25066	9.06879	10.1339	F-box protein 33 [Source:HGNC Symbol;Acc:HGNC:19833]	chr14:3986	NM_20330	.	.	.	NP_97604	.	protein_co	ENSG0000	254170	19833
FBXO34	14.5358	13.9258	14.2818	14.2997	15.5526	15.5261	F-box protein 34 [Source:HGNC Symbol;Acc:HGNC:20201]	chr14:5573	NM_01794	XM_01702	.	.	NP_06041	XP_005267	protein_co	ENSG0000	55030	20201
FBXO36	2.45463	2.86624	3.7345	3.06215	3.33833	3.42029	F-box protein 36 [Source:HGNC Symbol;Acc:HGNC:27020]	chr2:23078	NM_17489	XM_00524	.	.	NP_77755	XP_005246	protein_co	ENSG0000	130888	27020
FBXO38	10.2039	9.55263	10.5133	9.45449	9.76431	10.347	F-box protein 38 [Source:HGNC Symbol;Acc:HGNC:28844]	chr5:14776	NM_00127	XM_00671	.	.	NP_00125	XP_005268	protein_co	ENSG0000	81545	28844
FBXO39	0	0	0	0	0	0	F-box protein 39 [Source:HGNC Symbol;Acc:HGNC:28565]	chr17:6679	NM_15323	XM_01152	.	.	NP_69496	XP_011521	protein_co	ENSG0000	162517	28565
FBXO4	3.77776	4.25317	5.33614	2.84231	2.788	3.42998	F-box protein 4 [Source:HGNC Symbol;Acc:HGNC:13583]	chr5:41925	NM_00129	.	.	.	NP_00128	.	protein_co	ENSG0000	26272	13583
FBXO40	0	0	0	0	0	0	F-box protein 40 [Source:HGNC Symbol;Acc:HGNC:29816]	chr3:12131	NM_01629	.	.	.	NP_05738	.	protein_co	ENSG0000	51725	29816
FBXO41	12.8025	13.0515	14.7223	11.9857	11.2267	10.5415	F-box protein 41 [Source:HGNC Symbol;Acc:HGNC:29409]	chr2:73481	NM_00108	XM_01153	.	.	NP_00107	XP_006712	protein_co	ENSG0000	150726	29409
FBXO42	6.93306	7.46409	6.65309	6.38031	7.21129	6.27693	F-box protein 42 [Source:HGNC Symbol;Acc:HGNC:29249]	chr1:16573	NM_01899	XM_00671	.	.	NP_06186	XP_006710	protein_co	ENSG0000	54455	29249
FBXO43	0.134562	0.174554	0.258861	0.124379	0.088637	0.233284	F-box protein 43 [Source:HGNC Symbol;Acc:HGNC:28521]	chr8:10114	NM_00102	XM_01151	.	.	NP_00102	XP_011515	protein_co	ENSG0000	286151	28521

FBXO44	19.8888	18.8608	24.1602	17.6404	14.6027	10.0802	F-box protein 44 [Source:HGNC Symbol;Acc:HGNC:24847]	chr1:11708	NM_00101	XM_01154	.	.	NP_001014	XP_005263	protein_co	ENSG0000	93611	24847
FBXO45	13.7975	14.2217	14.6913	15.1536	17.0225	17.6954	F-box protein 45 [Source:HGNC Symbol;Acc:HGNC:29148]	chr3:19628	NM_00110	.	.	.	NP_001099	.	protein_co	ENSG0000	200933	29148
FBXO46	11.8738	12.0614	12.4583	12.0626	11.1438	13.0264	F-box protein 46 [Source:HGNC Symbol;Acc:HGNC:25069]	chr19:4621	NM_00108	.	.	.	NP_001073	.	protein_co	ENSG0000	23403	25069
FBXO47	0	0	0	0	0	0	F-box protein 47 [Source:HGNC Symbol;Acc:HGNC:31969]	chr17:3709	NM_00100	XM_01152	.	.	NP_001008	XP_011523	protein_co	ENSG0000	494188	31969
FBXO48	0.851826	0.918188	0.751821	0.42938	0.448051	0.936374	F-box protein 48 [Source:HGNC Symbol;Acc:HGNC:33857]	chr2:68686	NM_00102	XM_01700	.	.	NP_001019	XP_005264	protein_co	ENSG0000	554251	33857
FBXO5	28.7023	27.9134	27.1478	30.6416	38.1992	37.8765	F-box protein 5 [Source:HGNC Symbol;Acc:HGNC:13584]	chr6:15329	NM_00114	.	.	.	NP_001135	.	protein_co	ENSG0000	26271	13584
FBXO6	19.0389	18.0699	17.2276	16.2726	11.8779	10.7587	F-box protein 6 [Source:HGNC Symbol;Acc:HGNC:13585]	chr1:11724	NM_01843	XM_01154	.	.	NP_060908	XP_005263	protein_co	ENSG0000	26270	13585
FBXO7	53.8143	52.8734	46.7043	55.3352	52.1372	50.2434	F-box protein 7 [Source:HGNC Symbol;Acc:HGNC:13586]	chr22:3287	NM_00103	XM_01153	.	.	NP_001028	XP_011528	protein_co	ENSG0000	25793	13586
FBXO8	4.42751	3.36569	3.37352	3.18997	4.75353	6.35449	F-box protein 8 [Source:HGNC Symbol;Acc:HGNC:13587]	chr4:17515	NM_01218	.	.	.	NP_036312	.	protein_co	ENSG0000	26269	13587
FBXO9	20.5901	18.4648	20.961	18.4419	17.6035	15.6555	F-box protein 9 [Source:HGNC Symbol;Acc:HGNC:13588]	chr6:52929	NM_01234	XM_01151	.	.	NP_036479	XP_005249	protein_co	ENSG0000	26268	13588
FBXW10	0.071099	0.035742	0	0	0	0.052375	F-box and WD repeat domain containing 10 [Source:HGNC Symbo	chr17:1864	NM_00126	XM_01152	.	.	NP_001254	XP_006721	protein_co	ENSG0000	10517	1211
FBXW11	20.4859	22.1769	23.0742	20.4938	22.229	23.8409	F-box and WD repeat domain containing 11 [Source:HGNC Symbo	chr5:17128	NM_01230	XM_01700	.	.	NP_036432	XP_005265	protein_co	ENSG0000	23291	13607
FBXW12	0	0.068267	0	0.072821	0	0	F-box and WD repeat domain containing 12 [Source:HGNC Symbo	chr3:48413	NM_00115	XM_01153	.	.	NP_001153	XP_011531	protein_co	ENSG0000	285231	20729
FBXW2	12.8958	14.077	14.7356	14.4888	14.6741	13.7836	F-box and WD repeat domain containing 2 [Source:HGNC Symbol	chr9:12351	NM_01216	XM_01151	.	.	NP_036296	XP_005251	protein_co	ENSG0000	26190	13608
FBXW4	12.9528	12.7512	13.4331	12.9513	13.6457	15.5907	F-box and WD repeat domain containing 4 [Source:HGNC Symbol	chr10:1033	NM_02203	XM_00527	.	.	NP_071322	XP_005270	protein_co	ENSG0000	6468	10847
FBXW5	39.7156	38.8371	37.7219	41.7935	37.5412	37.1003	F-box and WD repeat domain containing 5 [Source:HGNC Symbol	chr9:13983	NM_01899	XM_01151	.	.	NP_061871	XP_005266	protein_co	ENSG0000	54461	13613
FBXW7	7.21085	8.11448	7.25926	6.91504	7.90092	7.78044	F-box and WD repeat domain containing 7 [Source:HGNC Symbol	chr4:15324	NM_00101	XM_01153	.	.	NP_001013	XP_011530	protein_co	ENSG0000	55294	16712
FBXW8	5.52435	6.08202	5.64952	4.34998	5.37785	4.8272	F-box and WD repeat domain containing 8 [Source:HGNC Symbol	chr12:1173	NM_01217	XM_01153	.	.	NP_036306	XP_005253	protein_co	ENSG0000	26259	13597
FBXW9	10.8987	10.1162	9.47672	8.33529	7.49215	7.37222	F-box and WD repeat domain containing 9 [Source:HGNC Symbol	chr19:1279	NM_03230	XM_00526	.	.	NP_115677	XP_005260	protein_co	ENSG0000	84261	28136
FCAMR	0	0	0.01956	0	0	0	Fc fragment of IgA and IgM receptor [Source:HGNC Symbol;Acc:H	chr1:20713	NM_00112	XM_01151	.	.	NP_001116	XP_011508	protein_co	ENSG0000	83953	24692
FCAR	0	0	0	0	0	0	Fc fragment of IgA receptor [Source:HGNC Symbol;Acc:HGNC:360	chr19:5538	NM_00200	.	.	.	NP_001991	.	protein_co	ENSG0000	2204	3608
FCER1A	0	0	0	0	0.106295	0	Fc fragment of IgE receptor Ia [Source:HGNC Symbol;Acc:HGNC:3	chr1:15925	NM_00200	.	.	.	NP_001992	.	protein_co	ENSG0000	2205	3609
FCER1G	0.681742	0.353517	0.44347	0.944761	0.761995	1.0517	Fc fragment of IgE receptor Ig [Source:HGNC Symbol;Acc:HGNC:3	chr1:16118	NM_00410	.	.	.	NP_004097	.	protein_co	ENSG0000	2207	3611
FCER2	0	0	0	0.050019	0	0.050091	Fc fragment of IgE receptor II [Source:HGNC Symbol;Acc:HGNC:36	chr19:7753	NM_00120	XM_00527	.	.	NP_001193	XP_005272	protein_co	ENSG0000	2208	3612
FCF1	40.7179	40.3913	35.2357	44.0256	47.1141	41.6921	FCF1 rRNA-processing protein [Source:HGNC Symbol;Acc:HGNC:2	chr14:7517	NM_01596	XM_01153	.	.	NP_001305	XP_005267	protein_co	ENSG0000	51077	20220
FCGBP	0.78164	0.895844	0.856991	0.869197	1.00023	0.853899	Fc fragment of IgG binding protein [Source:HGNC Symbol;Acc:HG	chr19:4035	NM_00389	.	.	.	NP_003881	.	protein_co	ENSG0000	8857	13572
FCGR1A	0	0	0	0	0	0	Fc fragment of IgG receptor Ia [Source:HGNC Symbol;Acc:HGNC:3	chr1:14975	NM_00056	XM_00524	.	.	NP_000557	XP_005245	protein_co	ENSG0000	2209	3613
FCGR1B	0	0	0	0	0	0	Fc fragment of IgG receptor Ib [Source:HGNC Symbol;Acc:HGNC:3	chr1:12092	NM_00100	.	.	.	NP_001004	XP_016856	protein_co	ENSG0000	2210	3614
FCGR2A	0.089427	0.093506	0.081836	0.052334	0.053257	0.030546	Fc fragment of IgG receptor IIa [Source:HGNC Symbol;Acc:HGNC:	chr1:16147	NM_00113	XM_01150	.	.	NP_001129	XP_011507	protein_co	ENSG0000	9103	3616
FCGR2B	0	0	0	0	0	0	Fc fragment of IgG receptor IIb [Source:HGNC Symbol;Acc:HGNC:	chr1:16163	NM_00100	XM_01700	.	.	NP_001002	XP_016856	protein_co	ENSG0000	2213	3618
FCGR3A	0	0	0	0	0	0	Fc fragment of IgG receptor IIIa [Source:HGNC Symbol;Acc:HGNC	chr1:16151	NM_00056	.	.	.	NP_000560	.	protein_co	ENSG0000	2214	3619
FCGR3B	0	0	0.027997	0	0	0	Fc fragment of IgG receptor IIIb [Source:HGNC Symbol;Acc:HGNC	chr1:16159	NM_00057	.	.	.	NP_000561	.	protein_co	ENSG0000	2215	3620
FCGRT	1.27792	0.307309	1.1214	1.46368	0.907997	0.704202	Fc fragment of IgG receptor and transporter [Source:HGNC Symb	chr19:5001	NM_00113	XM_01152	.	.	NP_001129	XP_011524	protein_co	ENSG0000	2217	3621
FCHO1	8.28573	9.45823	9.25497	11.5264	7.79113	7.41247	FCH domain only 1 [Source:HGNC Symbol;Acc:HGNC:29002]	chr19:1785	NM_00116	XM_01702	.	.	NP_001154	XP_005259	protein_co	ENSG0000	23149	29002
FCHO2	4.88975	3.98761	4.39817	4.06708	3.98753	4.92633	FCH domain only 2 [Source:HGNC Symbol;Acc:HGNC:25180]	chr5:72251	NM_00114	.	.	.	NP_001139	.	protein_co	ENSG0000	115548	25180
FCHSD1	5.67715	7.19746	9.45117	5.78677	4.98345	6.70555	FCH and double SH3 domains 1 [Source:HGNC Symbol;Acc:HGNC:	chr5:14101	NM_03344	XM_01153	.	.	NP_258260	XP_005268	protein_co	ENSG0000	89848	25463
FCHSD2	4.72781	5.04844	6.48942	3.44554	4.78841	4.32966	FCH and double SH3 domains 2 [Source:HGNC Symbol;Acc:HGNC:	chr11:7252	NM_01482	XM_01701	.	.	NP_055639	XP_011543	protein_co	ENSG0000	9873	29114
FCMR	0.082386	0.063839	0.106169	0.04525	0	0.068586	Fc fragment of IgM receptor [Source:HGNC Symbol;Acc:HGNC:14	chr1:20707	NM_00114	XM_00527	.	.	NP_001135	XP_005273	protein_co	ENSG0000	9214	14315
FCN1	0	0	0	0	0	0	ficolin 1 [Source:HGNC Symbol;Acc:HGNC:3623]	chr9:13779	NM_00200	.	.	.	NP_001994	.	protein_co	ENSG0000	2219	3623
FCN2	0	0	0	0	0	0	ficolin 2 [Source:HGNC Symbol;Acc:HGNC:3624]	chr9:13777	NM_00410	.	.	.	NP_004099	XP_006717	protein_co	ENSG0000	2220	3624
FCN3	0	0.08841	0.380736	0.095661	0.303756	0.284354	ficolin 3 [Source:HGNC Symbol;Acc:HGNC:3625]	chr1:27695	NM_00366	.	.	.	NP_003656	.	protein_co	ENSG0000	8547	3625
FCRL1	0	0	0	0	0	0	Fc receptor like 1 [Source:HGNC Symbol;Acc:HGNC:18509]	chr1:15776	NM_00115	XM_01150	.	.	NP_001152	XP_005244	protein_co	ENSG0000	115350	18509
FCRL2	0	0	0	0	0	0	Fc receptor like 2 [Source:HGNC Symbol;Acc:HGNC:14875]	chr1:15771	NM_03076	XM_01150	.	.	NP_110391	XP_011508	protein_co	ENSG0000	79368	14875
FCRL3	0	0	0	0.017009	0	0	Fc receptor like 3 [Source:HGNC Symbol;Acc:HGNC:18506]	chr1:15764	NM_00132	XM_01150	NR_135216	.	NP_001307	XP_006711	protein_co	ENSG0000	115352	18506
FCRL4	0	0	0	0	0	0	Fc receptor like 4 [Source:HGNC Symbol;Acc:HGNC:18507]	chr1:15754	NM_03128	XM_01151	.	.	NP_112572	XP_011508	protein_co	ENSG0000	83417	18507
FCRL5	0	0	0	0.014692	0	0	Fc receptor like 5 [Source:HGNC Symbol;Acc:HGNC:18508]	chr1:15748	NM_00119	XM_01151	.	.	NP_001182	XP_011508	protein_co	ENSG0000	83416	18508
FCRL6	0	0.055095	0	0	0	0	Fc receptor like 6 [Source:HGNC Symbol;Acc:HGNC:31910]	chr1:15977	NM_00100	XM_01150	.	.	NP_001004	XP_005245	protein_co	ENSG0000	343413	31910
FCRLA	0	0.056038	0.091748	0.059631	0.05915	0.052877	Fc receptor like A [Source:HGNC Symbol;Acc:HGNC:18504]	chr1:16167	NM_00118	XM_01151	.	.	NP_001171	XP_006711	protein_co	ENSG0000	84824	18504
FCRLB	0.610298	0.994217	0.757182	0.600351	0.574615	1.03145	Fc receptor like B [Source:HGNC Symbol;Acc:HGNC:26431]	chr1:16169	NM_00100	XM_01150	.	.	NP_001002	XP_011507	protein_co	ENSG0000	127943	26431
FDCSP	0	0	0	0	0	0	follicular dendritic cell secreted protein [Source:HGNC Symbol;Ac	chr4:71091	NM_15299	.	.	.	NP_694542	.	protein_co	ENSG0000	260436	19215
FDFT1	96.4416	92.964	130.065	160.111	144.489	168.737	farnesyl-diphosphate farnesyltransferase 1 [Source:HGNC Symbo	chr8:11653	NM_00128	XM_00671	.	.	NP_001274	XP_016868	protein_co	ENSG0000	2222	3629
FDPS	122.219	107.959	125.257	183.821	141.266	172.719	farnesyl diphosphate synthase [Source:HGNC Symbol;Acc:HGNC:3	chr1:15527	NM_00113	XM_00524	.	.	NP_001129	XP_005245	protein_co	ENSG0000	2224	3631
FDX1	11.4035	11.2381	11.9809	18.3225	15.9714	14.9206	ferredoxin 1 [Source:HGNC Symbol;Acc:HGNC:3638]	chr11:1103	NM_00410	.	.	.	NP_004100	.	protein_co	ENSG0000	2230	3638
FDX2	18.5243	15.6553	12.8726	20.4767	14.3543	11.7773	ferredoxin 2 [Source:HGNC Symbol;Acc:HGNC:30546]	chr19:1042	NM_00103	.	.	.	NP_001026	.	protein_co	ENSG0000	112812	30546
FDXACB1	3.45504	3.82817	2.86027	3.44023	3.32599	2.29517	ferredoxin-fold anticodon binding domain containing 1 [Source:H	chr11:1116	NM_13837	.	.	.	NP_612387	.	protein_co	ENSG0000	91893	25110
FDXR	66.8786	64.7059	83.4614	84.597	66.9321	48.9916	ferredoxin reductase [Source:HGNC Symbol;Acc:HGNC:3642]	chr17:7285	NM_00125	XM_01152	.	.	NP_001244	XP_006721	protein_co	ENSG0000	2232	3642
FECH	18.9044	19.313	19.3157	18.0011	18.9777	19.1701	ferrochelatase [Source:HGNC Symbol;Acc:HGNC:3647]	chr18:5521	NM_00014	XM_01152	.	.	NP_000131	XP_011524	protein_co	ENSG0000	2235	3647
FEM1A	9.49001	10.5257	9.16115	11.4132	9.69565	8.67578	fem-1 homolog A [Source:HGNC Symbol;Acc:HGNC:16934]	chr19:4791	NM_01870	.	.	.	NP_061178	.	protein_co	ENSG0000	55527	16934
FEM1B	11.7526	12.3436	11.302	11.283	13.5785	13.2725	fem-1 homolog B [Source:HGNC Symbol;Acc:HGNC:3649]	chr15:6857	NM_01532	XM_00672	.	.	NP_056137	.	protein_co	ENSG0000	10116	3649

FEM1C	8.90112	8.91823	7.92245	7.88109	8.8665	9.84098	fem-1 homolog C [Source:HGNC Symbol;Acc:HGNC:16933]	chr5:11485	NM_02017	XM_00527	.	.	NP_06456	XP_005272	protein_co	ENSG0000	56929	16933
FEN1	98.4246	95.2647	85.3721	119.775	104.364	74.0914	flap structure-specific endonuclease 1 [Source:HGNC Symbol;Acc	chr11:6155	NM_00411	.	.	.	NP_00410	.	protein_co	ENSG0000	2237	3650
FER	7.90579	9.17654	9.57161	4.25179	7.31801	7.87366	FER tyrosine kinase [Source:HGNC Symbol;Acc:HGNC:3655]	chr5:10808	NM_00130	XM_01154	.	.	NP_00129	XP_011541	protein_co	ENSG0000	2241	3655
FER1L5	0.009747	0.039208	0.050485	0.136901	0.066602	0.135199	fer-1 like family member 5 [Source:HGNC Symbol;Acc:HGNC:1904	chr2:97308	NM_00129	XM_01151	.	.	NP_00128	XP_011510	protein_co	ENSG0000	90342	19044
FER1L6	0.018074	0.009368	0.055443	0.05014	0	0.04667	fer-1 like family member 6 [Source:HGNC Symbol;Acc:HGNC:2806	chr8:12486	NM_00103	XM_01151	.	.	NP_00103	XP_006716	protein_co	ENSG0000	654463	28065
FERD3L	0	0	0	0	0	0	Fer3 like bHLH transcription factor [Source:HGNC Symbol;Acc:HG	chr7:19184	NM_15289	.	.	.	NP_69086	.	protein_co	ENSG0000	222894	16660
FERMT1	10.9929	13.4951	14.3155	15.3605	16.158	16.6794	fermitin family member 1 [Source:HGNC Symbol;Acc:HGNC:1588	chr20:6055	NM_01767	.	.	.	NP_06014	.	protein_co	ENSG0000	55612	15889
FERMT2	9.61188	9.81414	11.6244	11.9582	12.4871	14.7094	fermitin family member 2 [Source:HGNC Symbol;Acc:HGNC:1576	chr14:5332	NM_00113	XM_00672	.	.	NP_00112	XP_005267	protein_co	ENSG0000	10979	15767
FERMT3	0.66735	0.44502	0.732558	0.998156	1.20706	0.577774	fermitin family member 3 [Source:HGNC Symbol;Acc:HGNC:2315	chr11:6397	NM_03147	XM_01701	.	.	NP_11365	XP_011543	protein_co	ENSG0000	83706	23151
FES	4.26825	4.29922	4.33417	4.72651	4.15943	3.45729	FES proto-oncogene, tyrosine kinase [Source:HGNC Symbol;Acc:H	chr15:9142	NM_00114	XM_01702	.	.	NP_00113	XP_005254	protein_co	ENSG0000	2242	3657
FETUB	0.080492	0	0	0.18676	0.091027	0.101687	fetuin B [Source:HGNC Symbol;Acc:HGNC:3658]	chr3:18635	NM_00130	XM_01151	.	.	NP_00129	XP_005247	protein_co	ENSG0000	26998	3658
FEV	0	0	0	0	0	0	FEV, ETS transcription factor [Source:HGNC Symbol;Acc:HGNC:18	chr2:21984	NM_01752	.	.	.	NP_05999	.	protein_co	ENSG0000	54738	18562
FEZ1	0.951278	1.34081	1.09461	2.69515	1.11282	1.49636	fasciculation and elongation protein zeta 1 [Source:HGNC Symbol	chr11:1253	NM_00510	XM_00527	.	.	NP_00509	XP_005271	protein_co	ENSG0000	9638	3659
FEZ2	7.32736	6.59556	7.94917	5.81439	7.65922	8.7274	fasciculation and elongation protein zeta 2 [Source:HGNC Symbol	chr2:36779	NM_00104	XM_00671	.	.	NP_00103	XP_006712	protein_co	ENSG0000	9637	3660
FEZF1	0.256331	0.206418	0.175515	0.616088	0.157124	0.437442	FEZ family zinc finger 1 [Source:HGNC Symbol;Acc:HGNC:22788]	chr7:12194	NM_00102	XM_01151	.	.	NP_00101	XP_005250	protein_co	ENSG0000	389549	22788
FEZF2	0	0	0	0	0	0	FEZ family zinc finger 2 [Source:HGNC Symbol;Acc:HGNC:13506]	chr3:62355	NM_01800	XM_01153	.	.	NP_06047	.	protein_co	ENSG0000	55079	13506
FFAR1	0	0	0	0.043383	0	0.042411	free fatty acid receptor 1 [Source:HGNC Symbol;Acc:HGNC:4498]	chr19:3584	NM_00530	.	.	.	NP_00529	.	protein_co	ENSG0000	2864	4498
FFAR2	0	0	0	0	0	0	free fatty acid receptor 2 [Source:HGNC Symbol;Acc:HGNC:4501]	chr19:3593	NM_00530	XM_01702	.	.	NP_00529	XP_016882	protein_co	ENSG0000	2867	4501
FFAR3	0	0	0	0	0	0	free fatty acid receptor 3 [Source:HGNC Symbol;Acc:HGNC:4499]	chr19:3584	NM_00530	.	.	.	NP_00529	XP_011525	protein_co	ENSG0000	2865	4499
FFAR4	0	0	0	0.023508	0	0	free fatty acid receptor 4 [Source:HGNC Symbol;Acc:HGNC:19061	chr10:9532	NM_00119	.	.	.	NP_00118	.	protein_co	ENSG0000	338557	19061
FGA	0.027725	0	0	0	0	0	fibrinogen alpha chain [Source:HGNC Symbol;Acc:HGNC:3661]	chr4:15550	NM_00050	.	.	.	NP_00049	.	protein_co	ENSG0000	2243	3661
FGB	0	0	0	0	0	0.047207	fibrinogen beta chain [Source:HGNC Symbol;Acc:HGNC:3662]	chr4:15548	NM_00118	.	.	.	NP_00117	.	protein_co	ENSG0000	2244	3662
FGD1	2.46112	2.44292	2.89237	3.31116	2.81454	3.29499	FYVE, RhoGEF and PH domain containing 1 [Source:HGNC Symbol	chrX:54466	NM_00446	.	.	.	NP_00445	.	protein_co	ENSG0000	2245	3663
FGD2	0.074024	0.07337	0.042758	0.105921	0	0.190045	FYVE, RhoGEF and PH domain containing 2 [Source:HGNC Symbol	chr6:36973	NM_17355	XM_01701	.	.	NP_77582	XP_016865	protein_co	ENSG0000	221472	3664
FGD3	1.47769	1.21952	1.46852	2.15362	1.49853	1.5291	FYVE, RhoGEF and PH domain containing 3 [Source:HGNC Symbol	chr9:95709	NM_00108	XM_01701	.	.	NP_00107	XP_016870	protein_co	ENSG0000	89846	16027
FGD4	1.96291	1.94802	1.87061	1.8386	1.76333	2.01072	FYVE, RhoGEF and PH domain containing 4 [Source:HGNC Symbol	chr12:3255	NM_00130	XM_01701	.	.	NP_00129	XP_005253	protein_co	ENSG0000	121512	19125
FGD5	0	0.011201	0	0.048348	0.012819	0.048144	FYVE, RhoGEF and PH domain containing 5 [Source:HGNC Symbol	chr3:14860	NM_00132	XM_01153	.	.	NP_00130	XP_011531	protein_co	ENSG0000	152273	19117
FGD6	4.18403	4.49917	3.84793	2.84069	3.61334	3.3537	FYVE, RhoGEF and PH domain containing 6 [Source:HGNC Symbol	chr12:9547	NM_01835	.	.	.	NP_06082	.	protein_co	ENSG0000	55785	21740
FGF1	0.136053	0.087376	0	0.133774	0.09172	0.114858	fibroblast growth factor 1 [Source:HGNC Symbol;Acc:HGNC:3665	chr5:14197	NM_00080	XM_01153	.	.	NP_00079	XP_005268	protein_co	ENSG0000	2246	3665
FGF10	0	0	0	0	0	0	fibroblast growth factor 10 [Source:HGNC Symbol;Acc:HGNC:366	chr5:44303	NM_00446	XM_00524	.	.	NP_00445	XP_005248	protein_co	ENSG0000	2255	3666
FGF11	6.90953	5.99048	6.27201	5.20961	4.90577	4.14185	fibroblast growth factor 11 [Source:HGNC Symbol;Acc:HGNC:366	chr17:7341	NM_00130	.	.	.	NP_00129	.	protein_co	ENSG0000	2256	3667
FGF12	0	0	0	0	0	0	fibroblast growth factor 12 [Source:HGNC Symbol;Acc:HGNC:366	chr3:19185	NM_00411	XM_01700	.	.	NP_00410	XP_005247	protein_co	ENSG0000	2257	3668
FGF13	0.130034	0	0	0	0	0	fibroblast growth factor 13 [Source:HGNC Symbol;Acc:HGNC:367	chrX:13769	NM_00113	XM_01153	.	.	NP_00113	XP_005262	protein_co	ENSG0000	2258	3670
FGF14	0	0	0	0	0	0	fibroblast growth factor 14 [Source:HGNC Symbol;Acc:HGNC:367	chr13:1021	NM_00411	.	.	.	NP_00410	.	protein_co	ENSG0000	2259	3671
FGF16	0	0	0	0	0	0	fibroblast growth factor 16 [Source:HGNC Symbol;Acc:HGNC:367	chrX:76709	NM_00386	.	.	.	NP_00385	.	protein_co	ENSG0000	8823	3672
FGF17	0.562689	0.273454	0.905048	0.358616	0.326514	0.3783	fibroblast growth factor 17 [Source:HGNC Symbol;Acc:HGNC:367	chr8:21899	NM_00130	.	.	.	NP_00129	.	protein_co	ENSG0000	8822	3673
FGF18	0.893461	0.708694	0.962602	0.873124	0.827659	0.338594	fibroblast growth factor 18 [Source:HGNC Symbol;Acc:HGNC:367	chr5:17084	NM_00386	.	.	.	NP_00385	.	protein_co	ENSG0000	8817	3674
FGF19	19.7498	18.686	21.7992	16.6271	17.6112	38.9071	fibroblast growth factor 19 [Source:HGNC Symbol;Acc:HGNC:367	chr11:6951	NM_00511	.	.	.	NP_00510	.	protein_co	ENSG0000	9965	3675
FGF2	5.10287	5.95951	3.06909	7.57991	7.12265	5.89477	fibroblast growth factor 2 [Source:HGNC Symbol;Acc:HGNC:3676	chr4:12374	NM_00200	.	.	.	NP_00199	.	protein_co	ENSG0000	2247	3676
FGF20	0.05367	0	0	0	0	0	fibroblast growth factor 20 [Source:HGNC Symbol;Acc:HGNC:367	chr8:16849	NM_01985	.	.	.	NP_06282	.	protein_co	ENSG0000	26281	3677
FGF21	0	0.192046	0.084231	0	0	0.436999	fibroblast growth factor 21 [Source:HGNC Symbol;Acc:HGNC:367	chr19:4925	NM_01911	XM_00525	.	.	NP_06198	XP_005258	protein_co	ENSG0000	26291	3678
FGF22	0.14709	0.069303	0.693114	0.230655	0.350801	0.584751	fibroblast growth factor 22 [Source:HGNC Symbol;Acc:HGNC:367	chr19:6398	NM_00130	.	.	.	NP_00128	.	protein_co	ENSG0000	27006	3679
FGF23	0	0	0.04868	0.031251	0.031676	0.028828	fibroblast growth factor 23 [Source:HGNC Symbol;Acc:HGNC:368	chr12:4477	NM_02063	.	.	.	NP_06568	.	protein_co	ENSG0000	8074	3680
FGF3	0	0	0	0	0	0	fibroblast growth factor 3 [Source:HGNC Symbol;Acc:HGNC:3681	chr11:6962	NM_00524	.	.	.	NP_00523	.	protein_co	ENSG0000	2248	3681
FGF4	0	0	0	0	0	8.38E-08	fibroblast growth factor 4 [Source:HGNC Symbol;Acc:HGNC:3682	chr11:6958	NM_00200	XM_00671	.	.	NP_00199	XP_006718	protein_co	ENSG0000	2249	3682
FGF5	0	0	0	0	0	0	fibroblast growth factor 5 [Source:HGNC Symbol;Acc:HGNC:3683	chr4:81187	NM_00446	.	.	.	NP_00445	.	protein_co	ENSG0000	2250	3683
FGF6	0	0	0	0	0	0	fibroblast growth factor 6 [Source:HGNC Symbol;Acc:HGNC:3684	chr12:4543	NM_02099	.	.	.	NP_06627	.	protein_co	ENSG0000	2251	3684
FGF7	0.225835	0.049523	0.118272	0.078962	0.123145	0.148749	fibroblast growth factor 7 [Source:HGNC Symbol;Acc:HGNC:3685	chr15:4944	NM_00200	.	.	.	NP_00200	.	protein_co	ENSG0000	2252	3685
FGF8	0.577439	0.440685	1.2195	1.04373	0.786636	1.38851	fibroblast growth factor 8 [Source:HGNC Symbol;Acc:HGNC:3686	chr10:1035	NM_00120	.	.	.	NP_00119	.	protein_co	ENSG0000	2253	3686
FGF9	2.18412	2.59873	3.19749	1.42836	2.04681	3.35006	fibroblast growth factor 9 [Source:HGNC Symbol;Acc:HGNC:3687	chr13:2224	NM_00201	.	.	.	NP_00200	.	protein_co	ENSG0000	2254	3687
FGFBP1	4.09256	3.76617	2.71732	4.5323	5.32074	4.34653	fibroblast growth factor binding protein 1 [Source:HGNC Symbol;	chr4:15937	NM_00513	.	.	.	NP_00512	.	protein_co	ENSG0000	9982	19695
FGFBP2	0.190021	0.064206	0.055143	0.068907	0.216976	0.06781	fibroblast growth factor binding protein 2 [Source:HGNC Symbol;	chr4:15961	NM_03195	.	.	.	NP_11415	.	protein_co	ENSG0000	83888	29451
FGFBP3	1.60146	1.78225	1.67019	1.85463	2.06837	1.50113	fibroblast growth factor binding protein 3 [Source:HGNC Symbol;	chr10:9366	NM_15242	.	.	.	NP_68964	.	protein_co	ENSG0000	143282	23428
FGFR1	17.2418	17.5472	17.7594	17.6217	17.0418	15.1658	fibroblast growth factor receptor 1 [Source:HGNC Symbol;Acc:HG	chr8:38268	NM_00117	XM_01701	.	.	NP_00116	XP_006716	protein_co	ENSG0000	2260	3688
FGFR1OP	11.4423	11.8764	10.1229	11.0997	11.9017	8.32363	FGFR1 oncogene partner [Source:HGNC Symbol;Acc:HGNC:17012	chr6:16741	NM_00127	.	.	.	NP_00126	.	protein_co	ENSG0000	11116	17012
FGFR1OP2	13.0278	12.3335	11.5102	14.4398	11.3201	16.5711	FGFR1 oncogene partner 2 [Source:HGNC Symbol;Acc:HGNC:2309	chr12:2709	NM_00117	.	.	.	NP_00116	.	protein_co	ENSG0000	26127	23098
FGFR2	6.31915	5.5872	6.83457	7.73106	7.79645	7.71482	fibroblast growth factor receptor 2 [Source:HGNC Symbol;Acc:HG	chr10:1232	NM_00014	XM_01153	.	.	NP_00013	XP_006717	protein_co	ENSG0000	2263	3689
FGFR3	9.54584	8.96715	12.3139	11.8368	13.0446	12.3334	fibroblast growth factor receptor 3 [Source:HGNC Symbol;Acc:HG	chr4:17950	NM_00014	XM_01151	.	.	NP_00013	XP_006713	protein_co	ENSG0000	2261	3690
FGFR4	23.4897	24.6111	27.7697	33.2467	27.0528	27.257	fibroblast growth factor receptor 4 [Source:HGNC Symbol;Acc:HG	chr5:17651	NM_00129	XM_01153	.	.	NP_00127	XP_005265	protein_co	ENSG0000	2264	3691

FGFRL1	24.9565	27.5954	24.2642	27.3663	23.5743	26.9251	fibroblast growth factor receptor-like 1 [Source:HGNC Symbol;Ac	chr4:10037	NM_00100	XM_01151	.	.	NP_00100	XP_011511	protein_co	ENSG0000	53834	3693
FGG	0.08935	0.143044	0.229166	0	0.093132	0.131779	fibrinogen gamma chain [Source:HGNC Symbol;Acc:HGNC:3694]	chr4:15552	NM_00050	.	.	.	NP_00050	.	protein_co	ENSG0000	2266	3694
FGGY	2.93561	3.20084	3.66015	4.18916	3.09168	3.48795	FGGY carbohydrate kinase domain containing [Source:HGNC Sym	chr1:59762	NM_00111	XM_01700	.	.	NP_00110	XP_011540	protein_co	ENSG0000	55277	25610
FGL1	0.176335	0	0	0	0	0	fibrinogen like 1 [Source:HGNC Symbol;Acc:HGNC:3695]	chr8:17721	NM_00446	.	.	.	NP_00445	XP_016868	protein_co	ENSG0000	2267	3695
FGL2	0	0	0	0	0	0	fibrinogen like 2 [Source:HGNC Symbol;Acc:HGNC:3696]	chr7:76751	NM_00668	.	.	.	NP_00667	.	protein_co	ENSG0000	10875	3696
FGR	0	0	0	0	0	0	FGR proto-oncogene, Src family tyrosine kinase [Source:HGNC Sy	chr1:27938	NM_00104	XM_01154	.	.	NP_00103	XP_006710	protein_co	ENSG0000	2268	3697
FH	43.7674	44.4968	42.7131	45.979	45.064	42.2143	fumarate hydratase [Source:HGNC Symbol;Acc:HGNC:3700]	chr1:24166	NM_00014	XM_01154	.	.	NP_00013	XP_011542	protein_co	ENSG0000	2271	3700
FHAD1	1.30145	0.899375	1.11599	1.14937	1.03727	1.29523	forkhead associated phosphopeptide binding domain 1 [Source:H	chr1:15573	NM_05292	XM_01154	.	.	NP_44316	XP_011538	protein_co	ENSG0000	114827	29408
FHDC1	1.58884	1.55513	1.79789	1.3285	1.46193	1.76807	FH2 domain containing 1 [Source:HGNC Symbol;Acc:HGNC:29363	chr4:15385	NM_03339	XM_01153	.	.	NP_20375	XP_005263	protein_co	ENSG0000	85462	29363
FHIT	11.5675	7.0746	7.85103	4.77678	2.45973	10.0507	fragile histidine triad [Source:HGNC Symbol;Acc:HGNC:3701]	chr3:59735	NM_00116	XM_01153	.	.	NP_00115	XP_011531	protein_co	ENSG0000	2272	3701
FHL1	6.99913	7.47143	11.5674	6.94081	8.63678	10.7778	four and a half LIM domains 1 [Source:HGNC Symbol;Acc:HGNC:3	chrX:13522	NM_00115	XM_01702	.	.	NP_00115	XP_006724	protein_co	ENSG0000	2273	3702
FHL2	61.5223	62.7453	67.0986	67.3187	67.5871	67.1802	four and a half LIM domains 2 [Source:HGNC Symbol;Acc:HGNC:3	chr2:10597	NM_00103	XM_01151	.	.	NP_00103	XP_005263	protein_co	ENSG0000	2274	3703
FHL3	14.8791	14.4977	13.103	14.3423	13.2921	10.8723	four and a half LIM domains 3 [Source:HGNC Symbol;Acc:HGNC:3	chr1:38462	NM_00124	.	.	.	NP_00123	.	protein_co	ENSG0000	2275	3704
FHL5	0	0	0	0	0	0	four and a half LIM domains 5 [Source:HGNC Symbol;Acc:HGNC:1	chr6:97010	NM_00117	XM_00671	.	.	NP_00116	XP_006715	protein_co	ENSG0000	9457	17371
FHOD1	11.4147	10.6343	12.6276	12.7424	11.6347	9.40797	formin homology 2 domain containing 1 [Source:HGNC Symbol;A	chr16:6726	NM_01324	XM_01152	.	.	NP_03737	XP_011521	protein_co	ENSG0000	29109	17905
FHOD3	2.3938	2.06478	2.49777	1.96841	1.79137	2.18409	formin homology 2 domain containing 3 [Source:HGNC Symbol;A	chr18:3387	NM_00128	XM_01152	.	.	NP_00126	XP_005258	protein_co	ENSG0000	80206	26178
FIBCD1	6.21963	6.50646	10.0251	6.41678	6.41082	7.61432	fibrinogen C domain containing 1 [Source:HGNC Symbol;Acc:HGN	chr9:13377	NM_00114	.	.	.	NP_00113	.	protein_co	ENSG0000	84929	25922
FIBIN	0	0	0	0	0	0	fin bud initiation factor homolog (zebrafish) [Source:HGNC Symbo	chr11:2701	NM_20337	.	.	.	NP_97624	.	protein_co	ENSG0000	387758	33747
FIBP	56.67	59.6916	50.2334	65.719	54.2805	45.2738	FGF1 intracellular binding protein [Source:HGNC Symbol;Acc:HGN	chr11:6565	NM_00421	.	.	.	NP_00420	.	protein_co	ENSG0000	9158	3705
FICD	3.09844	4.33726	2.31785	3.90337	3.16024	2.4956	FIC domain containing [Source:HGNC Symbol;Acc:HGNC:18416]	chr12:1089	NM_00707	XM_01153	.	.	NP_00900	XP_011536	protein_co	ENSG0000	11153	18416
FIG4	6.53079	6.55602	9.77972	6.5199	7.67752	7.95382	FIG4 phosphoinositide 5-phosphatase [Source:HGNC Symbol;Acc	chr6:11001	NM_01484	XM_01153	.	.	NP_05566	XP_011534	protein_co	ENSG0000	9896	16873
FIGLA	0	0	0	0	0	0	folliculogenesis specific bHLH transcription factor [Source:HGNC S	chr2:71004	NM_00100	.	.	.	NP_00100	.	protein_co	ENSG0000	344018	24669
FIGN	0	0	0	0	0	0	fidgetin, microtubule severing factor [Source:HGNC Symbol;Acc:H	chr2:16444	NM_01808	XM_01151	.	.	NP_00130	XP_011509	protein_co	ENSG0000	55137	13285
FIGNL1	12.9103	12.6402	8.58959	10.1636	10.9961	11.4548	fidgetin like 1 [Source:HGNC Symbol;Acc:HGNC:13286]	chr7:50511	NM_00104	XM_01151	.	.	NP_00103	XP_011513	protein_co	ENSG0000	63979	13286
FILIP1	0	0.014039	0.03498	0.015025	0.012901	0.013269	filamin A interacting protein 1 [Source:HGNC Symbol;Acc:HGNC:2	chr6:76001	NM_00128	XM_00524	.	.	NP_00127	XP_005248	protein_co	ENSG0000	27145	21015
FILIP1L	0	0	0	0	0	0	filamin A interacting protein 1 like [Source:HGNC Symbol;Acc:HG	chr3:99536	NM_00104	XM_01151	.	.	NP_00103	XP_006713	protein_co	ENSG0000	11259	24589
FIP1L1	28.2649	30.7239	27.7856	31.6143	32.7953	27.0232	factor interacting with PAPOLA and CPSF1 [Source:HGNC Symbol;	chr4:54243	NM_00113	XM_00526	.	.	NP_00112	XP_005265	protein_co	ENSG0000	81608	19124
FIS1	87.4422	85.9815	79.8511	85.4585	61.8776	62.8766	fission, mitochondrial 1 [Source:HGNC Symbol;Acc:HGNC:21689]	chr7:10088	NM_01606	.	.	.	NP_05715	.	protein_co	ENSG0000	51024	21689
FITM1	0.304179	0.308264	0.172226	0.431524	0.353807	6.31E-52	fat storage inducing transmembrane protein 1 [Source:HGNC Sym	chr14:2454	NM_20340	.	.	.	NP_98194	.	protein_co	ENSG0000	161247	33714
FITM2	2.4496	3.04691	4.34275	3.40121	3.88549	3.99432	fat storage inducing transmembrane protein 2 [Source:HGNC Sym	chr20:4293	NM_00108	.	.	.	NP_00107	.	protein_co	ENSG0000	128486	16135
FIZ1	7.42419	7.46243	6.55824	7.59312	6.09337	5.17443	FLT3 interacting zinc finger 1 [Source:HGNC Symbol;Acc:HGNC:25	chr19:5610	NM_03283	XM_00525	.	.	NP_11622	XP_005259	protein_co	ENSG0000	84922	25917
FJX1	9.20353	9.49467	9.64003	11.2909	11.3162	8.06084	four jointed box 1 [Source:HGNC Symbol;Acc:HGNC:17166]	chr11:3563	NM_01434	.	.	.	NP_05515	.	protein_co	ENSG0000	24147	17166
FKBP10	27.2075	27.6618	34.8563	9.61429	8.17362	7.62076	FK506 binding protein 10 [Source:HGNC Symbol;Acc:HGNC:18169	chr17:3996	NM_02193	XM_01152	.	.	NP_06875	XP_011523	protein_co	ENSG0000	60681	18169
FKBP11	6.56107	5.32798	7.6375	7.54549	7.33335	7.18692	FK506 binding protein 11 [Source:HGNC Symbol;Acc:HGNC:18624	chr12:4929	NM_00114	.	.	.	NP_00113	.	protein_co	ENSG0000	51303	18624
FKBP14	4.54011	5.18196	4.9149	3.66945	4.80022	4.33484	FK506 binding protein 14 [Source:HGNC Symbol;Acc:HGNC:18625	chr7:30050	NM_01794	.	.	.	NP_06041	.	protein_co	ENSG0000	55033	18625
FKBP15	7.25393	7.56197	6.30935	6.44812	6.59553	5.59677	FK506 binding protein 15 [Source:HGNC Symbol;Acc:HGNC:23397	chr9:11592	NM_01525	XM_00671	.	.	NP_05607	XP_006717	protein_co	ENSG0000	23307	23397
FKBP1A	220.559	208.664	225.469	217.335	212.27	217.387	FK506 binding protein 1A [Source:HGNC Symbol;Acc:HGNC:3711]	chr20:1349	NM_00080	.	NR_10692	.	NP_00079	.	protein_co	ENSG0000	102465524	3711
FKBP1B	8.25516	8.4082	12.3617	8.72946	7.96483	10.6895	FK506 binding protein 1B [Source:HGNC Symbol;Acc:HGNC:3712]	chr2:24272	NM_00132	.	.	.	NP_00130	XP_016859	protein_co	ENSG0000	2281	3712
FKBP1C	23.6613	23.1916	24.0613	21.2288	17.8386	17.6435	FK506 binding protein 1C [Source:HGNC Symbol;Acc:HGNC:21376	chr6:63921	.	.	.	.	.	.	protein_co	ENSG0000	.	21376
FKBP2	109.874	103.056	79.0192	100.415	87.9219	74.3446	FK506 binding protein 2 [Source:HGNC Symbol;Acc:HGNC:3718]	chr11:6400	NM_00113	XM_00527	.	.	NP_00112	XP_005273	protein_co	ENSG0000	2286	3718
FKBP3	44.0664	40.7048	46.1384	47.3388	52.8111	61.2937	FK506 binding protein 3 [Source:HGNC Symbol;Acc:HGNC:3719]	chr14:4555	NM_00201	.	.	.	NP_00200	XP_016876	protein_co	ENSG0000	2287	3719
FKBP4	120.825	120.754	86.0345	167.886	143.502	88.9323	FK506 binding protein 4 [Source:HGNC Symbol;Acc:HGNC:3720]	chr12:2904	NM_00201	XM_01152	.	.	NP_00200	XP_011519	protein_co	ENSG0000	2288	3720
FKBP5	25.9155	25.8915	34.2214	27.7795	29.6315	29.9243	FK506 binding protein 5 [Source:HGNC Symbol;Acc:HGNC:3721]	chr6:35541	NM_00114	.	.	.	NP_00113	.	protein_co	ENSG0000	2289	3721
FKBP6	0	0	0.045721	0.177367	0	0	FK506 binding protein 6 [Source:HGNC Symbol;Acc:HGNC:3722]	chr7:72742	NM_00113	XM_01701	.	.	NP_00112	XP_006716	protein_co	ENSG0000	8468	3722
FKBP7	4.31993	4.2651	4.6917	6.22981	7.29331	4.47825	FK506 binding protein 7 [Source:HGNC Symbol;Acc:HGNC:3723]	chr2:17932	NM_00113	.	.	.	NP_00112	.	protein_co	ENSG0000	51661	3723
FKBP8	121.681	118.798	124.268	133.347	109.179	125.807	FK506 binding protein 8 [Source:HGNC Symbol;Acc:HGNC:3724]	chr19:1864	NM_00130	XM_01152	.	.	NP_00129	XP_011526	protein_co	ENSG0000	23770	3724
FKBP9	23.7159	25.7727	24.3071	27.4603	27.5377	37.0475	FK506 binding protein 9 [Source:HGNC Symbol;Acc:HGNC:3725]	chr7:32997	NM_00128	.	.	.	NP_00127	.	protein_co	ENSG0000	11328	3725
FKBPL	14.4151	14.1947	12.1786	18.5432	14.4136	13.5401	FK506 binding protein like [Source:HGNC Symbol;Acc:HGNC:1394	chr6:32096	NM_02211	XM_01151	.	.	NP_07139	XP_011513	protein_co	ENSG0000	63943	13949
FKRP	8.1874	7.70426	6.201	6.44972	6.644	5.61223	fukutin related protein [Source:HGNC Symbol;Acc:HGNC:17997]	chr19:4722	NM_00103	XM_01152	.	.	NP_00103	XP_005259	protein_co	ENSG0000	79147	17997
FKSG48	0	0	0	0	0	0	.	chr1:33439	.	.	.	.	.	.	protein_co	ENSG0000	.	.
FKSG52	0	0	0	0	0	0	.	chr5:58264	.	.	.	.	.	.	protein_co	ENSG0000	.	.
FKSG61	0	0	0	0	0	0	.	chr14:7786	.	.	.	.	.	.	protein_co	ENSG0000	.	.
FKSG63	0	0	0	0	0	0	.	chr16:7025	.	.	.	.	.	.	protein_co	ENSG0000	.	.
FKSG68	0.705496	0	0	0	0	0	.	chr21:3727	.	.	.	.	.	.	protein_co	ENSG0000	.	.
FKTN	4.53007	4.88872	4.837	3.54702	5.3571	5.48405	fukutin [Source:HGNC Symbol;Acc:HGNC:3622]	chr9:10832	NM_00107	XM_01701	.	.	NP_00107	XP_016869	protein_co	ENSG0000	2218	3622
FLAD1	42.5893	41.3436	35.1366	59.7496	49.7112	36.8408	flavin adenine dinucleotide synthetase 1 [Source:HGNC Symbol;A	chr1:15495	NM_00118	XM_00524	.	.	NP_00117	.	protein_co	ENSG0000	80308	24671
FLCN	5.51715	4.72786	4.58345	5.88268	5.49941	4.39532	folliculin [Source:HGNC Symbol;Acc:HGNC:27310]	chr17:1711	NM_14460	XM_01152	.	.	NP_65320	XP_011522	protein_co	ENSG0000	201163	27310
FLG	0	0	0	0	0	0	filaggrin [Source:HGNC Symbol;Acc:HGNC:3748]	chr1:15227	NM_00201	XM_01150	.	.	NP_00200	.	protein_co	ENSG0000	2312	3748
FLG2	0	0	0	0	0	0	filaggrin family member 2 [Source:HGNC Symbol;Acc:HGNC:3327	chr1:15232	NM_00101	XM_01150	.	.	NP_00101	XP_011507	protein_co	ENSG0000	388698	33276

FLI1	0	0	0	0	0	0	Fli-1 proto-oncogene, ETS transcription factor [Source:HGNC Sym	chr11:1285	NM_00116	XM_01154	.	.	NP_00116	XP_011541	protein_co	ENSG0000	2313	3749
FLII	42.4326	44.8203	39.2689	42.2204	37.7639	37.296	FLII, actin remodeling protein [Source:HGNC Symbol;Acc:HGNC:3	chr17:1812	NM_00125	XM_00525	.	.	NP_00124	XP_005256	protein_co	ENSG0000	2314	3750
FLJ00273	0.285733	0	0	0	0	0	.	chr9:35041	.	.	.	.	.	.	protein_co	ENSG0000	.	.
FLJ00325	0	0	0	0	0	0	.	chr7:10854	.	.	.	.	.	.	protein_co	ENSG0000	.	.
FLJ00388	0	0	0	0	0	0	.	chr1:48226	.	.	.	.	.	.	protein_co	ENSG0000	.	.
FLJ00418	0.064279	0.04165	0.073482	0	0.025098	0.048121	.	chr16:7069	.	.	.	.	.	.	protein_co	ENSG0000	.	.
FLJ14816	0	0	0	0	0	0	long intergenic non-protein coding RNA 1101 [Source:HGNC Sym	chr2:12122	.	.	.	.	.	.	protein_co	ENSG0000	.	25923
FLJ20306	0	0.048888	0.042448	0	0.113334	0.052879	CDNA FLJ20306 fis, clone HEP06881; Putative uncharacterized pr	chr7:64487	.	.	.	.	.	.	protein_co	ENSG0000	.	.
FLJ20373	7.99E-24	4.67E-38	4.66E-38	6.82E-59	2.84E-10	1.51E-13	.	chr2:10231	.	.	.	.	.	.	protein_co	ENSG0000	.	.
FLJ22763	0	0	0	0	0	0	uncharacterized LOC401081 [Source:EntrezGene;Acc:401081]	chr3:10885	.	.	NR_03397	.	.	.	protein_co	ENSG0000	401081	.
FLNA	84.0482	93.0982	73.348	89.8951	91.3814	88.3691	filamin A [Source:HGNC Symbol;Acc:HGNC:3754]	chrX:15357	NM_00111	XM_01153	.	.	NP_00110	XP_011529	protein_co	ENSG0000	2316	3754
FLNB	31.7789	31.5498	29.9686	32.0677	29.3839	34.5684	filamin B [Source:HGNC Symbol;Acc:HGNC:3755]	chr3:57994	NM_00116	XM_00526	.	.	NP_00115	XP_005265	protein_co	ENSG0000	2317	3755
FLNC	11.1651	12.5856	10.3868	11.1355	11.5183	8.97717	filamin C [Source:HGNC Symbol;Acc:HGNC:3756]	chr7:12847	NM_00112	.	.	.	NP_00112	.	protein_co	ENSG0000	2318	3756
FLOT1	32.4711	32.5275	23.679	49.1083	38.8223	29.4651	flotillin 1 [Source:HGNC Symbol;Acc:HGNC:3757]	chr6:30695	NM_00580	XM_00671	.	.	NP_00579	XP_005248	protein_co	ENSG0000	10211	3757
FLOT2	33.2707	34.8718	42.0409	38.8064	33.5107	36.0648	flotillin 2 [Source:HGNC Symbol;Acc:HGNC:3758]	chr17:2720	NM_00447	XM_01702	.	.	NP_00446	XP_005258	protein_co	ENSG0000	2319	3758
FLRT1	0	0.038736	0.016884	0	0	0.173021	fibronectin leucine rich transmembrane protein 1 [Source:HGNC	chr11:6374	NM_01328	XM_01154	.	.	NP_03741	XP_005273	protein_co	ENSG0000	23769	3760
FLRT2	0	0	0	0	0	0	fibronectin leucine rich transmembrane protein 2 [Source:HGNC	chr14:8599	NM_01323	XM_01153	.	.	NP_03736	XP_005267	protein_co	ENSG0000	23768	3761
FLRT3	0.146074	0.112406	0.217244	0	0.02303	0.060695	fibronectin leucine rich transmembrane protein 3 [Source:HGNC	chr20:1397	NM_01328	XM_01152	.	.	NP_03741	XP_005260	protein_co	ENSG0000	23767	3762
FLT1	0	0	0.008238	0	0	0	fms related tyrosine kinase 1 [Source:HGNC Symbol;Acc:HGNC:37	chr13:2887	NM_00115	XM_01153	.	.	NP_00115	XP_011533	protein_co	ENSG0000	2321	3763
FLT3	0.154182	0.266397	0.218128	0.087613	0.085869	0.19041	fms related tyrosine kinase 3 [Source:HGNC Symbol;Acc:HGNC:37	chr13:2857	NM_00411	XM_01153	.	.	NP_00411	XP_011533	protein_co	ENSG0000	2322	3765
FLT3LG	2.29204	2.1642	2.217	1.743	1.76076	1.4765	fms related tyrosine kinase 3 ligand [Source:HGNC Symbol;Acc:H	chr19:4997	NM_00120	XM_01702	.	.	NP_00119	XP_005258	protein_co	ENSG0000	2323	3766
FLT4	0.019991	0	0.051328	0.042618	0.023453	0.019566	fms related tyrosine kinase 4 [Source:HGNC Symbol;Acc:HGNC:37	chr5:18002	NM_00202	XM_01700	.	.	NP_00201	XP_011532	protein_co	ENSG0000	2324	3767
FLVCR1	5.98881	6.53159	6.45692	5.63702	6.87875	6.34468	feline leukemia virus subgroup C cellular receptor 1 [Source:HGN	chr1:21303	NM_01405	XM_01150	.	.	NP_05477	XP_011507	protein_co	ENSG0000	28982	24682
FLVCR2	0.660591	0.505488	0.586417	1.05366	1.08505	1.29216	feline leukemia virus subgroup C cellular receptor family member	chr14:7604	NM_00119	.	.	.	NP_00118	.	protein_co	ENSG0000	55640	20105
FLYWCH1	12.274	12.8212	13.1234	12.2148	10.155	13.9291	FLYWCH-type zinc finger 1 [Source:HGNC Symbol;Acc:HGNC:2540	chr16:2961	NM_00130	XM_01152	.	.	NP_00129	XP_005255	protein_co	ENSG0000	84256	25404
FLYWCH2	29.6367	31.8066	31.7496	32.4241	22.2311	24.2642	FLYWCH family member 2 [Source:HGNC Symbol;Acc:HGNC:2517	chr16:2933	NM_00114	XM_00525	.	.	NP_00113	XP_005255	protein_co	ENSG0000	114984	25178
FMN1	0.138895	0.074884	0.211632	0.10851	0.244741	0.247319	formin 1 [Source:HGNC Symbol;Acc:HGNC:3768]	chr15:3305	NM_00110	XM_01702	.	.	NP_00109	XP_011519	protein_co	ENSG0000	342184	3768
FMN2	0	0	0	0	0	0.041393	formin 2 [Source:HGNC Symbol;Acc:HGNC:14074]	chr1:24025	NM_00130	XM_00527	.	.	NP_00129	.	protein_co	ENSG0000	56776	14074
FMNL1	1.635	1.73288	2.3306	2.03161	2.30388	1.58366	formin like 1 [Source:HGNC Symbol;Acc:HGNC:1212]	chr17:4329	NM_00589	XM_01152	.	.	NP_00588	XP_006722	protein_co	ENSG0000	752	1212
FMNL2	10.0033	9.76448	13.0457	8.16244	11.8841	12.4081	formin like 2 [Source:HGNC Symbol;Acc:HGNC:18267]	chr2:15319	NM_05290	XM_01151	.	.	NP_44313	XP_005246	protein_co	ENSG0000	114793	18267
FMNL3	1.29145	1.42534	1.38934	1.49494	1.49261	1.53422	formin like 3 [Source:HGNC Symbol;Acc:HGNC:23698]	chr12:5001	NM_17573	XM_01153	.	.	NP_78386	XP_005269	protein_co	ENSG0000	91010	23698
FMO1	0	0	0	0	0	0	flavin containing monooxygenase 1 [Source:HGNC Symbol;Acc:H	chr1:17121	NM_00128	XM_00671	.	.	NP_00126	XP_005245	protein_co	ENSG0000	2326	3769
FMO2	0	0	0	0	0	0	flavin containing monooxygenase 2 [Source:HGNC Symbol;Acc:H	chr1:17115	NM_00146	.	.	.	NP_00145	.	protein_co	ENSG0000	2327	3770
FMO3	0	0	0	0	0	0	flavin containing monooxygenase 3 [Source:HGNC Symbol;Acc:H	chr1:17106	NM_00100	XM_01150	.	.	NP_00100	XP_011507	protein_co	ENSG0000	2328	3771
FMO4	1.13962	1.28557	1.10569	1.03497	1.00972	1.44601	flavin containing monooxygenase 4 [Source:HGNC Symbol;Acc:H	chr1:17128	NM_00202	XM_00524	.	.	NP_00201	XP_005245	protein_co	ENSG0000	2329	3772
FMO5	0	0.0824	0.061008	0.04349	0.088262	0.039756	flavin containing monooxygenase 5 [Source:HGNC Symbol;Acc:H	chr1:14665	NM_00114	XM_01150	.	.	NP_00113	XP_005273	protein_co	ENSG0000	2330	3773
FMOD	0.022143	0	0	0.072686	0	0.023347	fibromodulin [Source:HGNC Symbol;Acc:HGNC:3774]	chr1:20330	NM_00202	.	.	.	NP_00201	.	protein_co	ENSG0000	2331	3774
FMR1	9.12292	9.72043	8.4454	9.18856	12.2427	11.7829	fragile X mental retardation 1 [Source:HGNC Symbol;Acc:HGNC:3	chrX:14699	NM_00118	.	.	.	NP_00117	.	protein_co	ENSG0000	2332	3775
FMR1NB	0	0	0	0	0	0	fragile X mental retardation 1 neighbor [Source:HGNC Symbol;Ac	chrX:14706	NM_15257	.	.	.	NP_68979	.	protein_co	ENSG0000	158521	26372
FN1	1.14174	1.02509	2.56919	1.99477	2.07697	4.81399	fibronectin 1 [Source:HGNC Symbol;Acc:HGNC:3778]	chr2:21622	NM_00130	XM_01700	.	.	NP_00129	XP_005246	protein_co	ENSG0000	2335	3778
FN3K	3.55785	3.61738	4.732	5.19071	5.03307	4.50397	fructosamine 3 kinase [Source:HGNC Symbol;Acc:HGNC:24822]	chr17:8069	NM_02215	.	.	.	NP_07144	.	protein_co	ENSG0000	64122	24822
FN3KRP	30.9114	30.5997	29.7698	28.9658	26.8561	23.037	fructosamine 3 kinase related protein [Source:HGNC Symbol;Acc:	chr17:8067	NM_02461	.	.	.	NP_07889	.	protein_co	ENSG0000	79672	25700
FNBP1	5.54577	5.58119	6.78828	5.31134	5.04911	5.59514	formin binding protein 1 [Source:HGNC Symbol;Acc:HGNC:17069	chr9:13264	NM_01503	XM_01701	.	.	NP_05584	XP_005251	protein_co	ENSG0000	23048	17069
FNBP1L	14.7319	15.1534	13.8157	16.8442	20.5838	23.021	formin binding protein 1 like [Source:HGNC Symbol;Acc:HGNC:20	chr1:93913	NM_00102	XM_01154	.	.	NP_00102	XP_011539	protein_co	ENSG0000	54874	20851
FNBP4	13.1333	14.1603	15.3877	13.0315	14.2691	14.1556	formin binding protein 4 [Source:HGNC Symbol;Acc:HGNC:19752	chr11:4773	NM_00131	XM_00525	.	.	NP_00130	.	protein_co	ENSG0000	23360	19752
FNDC1	0.011135	0.033473	0.076962	0.024213	0.012795	0.023993	fibronectin type III domain containing 1 [Source:HGNC Symbol;Ac	chr6:15959	NM_03253	XM_01153	.	.	NP_11592	XP_011534	protein_co	ENSG0000	84624	21184
FNDC10	8.33332	8.44607	8.21693	11.0994	8.28252	6.29962	fibronectin type III domain containing 10 [Source:HGNC Symbol;A	chr1:15333	NM_00124	.	.	.	NP_00122	.	protein_co	ENSG0000	643988	42951
FNDC11	0.934117	0.814366	1.45507	1.31343	1.31624	1.94706	fibronectin type III domain containing 11 [Source:HGNC Symbol;A	chr20:6218	NM_00131	XM_01152	.	.	NP_00130	XP_005260	protein_co	ENSG0000	79025	28764
FNDC3A	13.6689	14.7588	12.5849	11.0253	14.0152	14.264	fibronectin type III domain containing 3A [Source:HGNC Symbol;A	chr13:4955	NM_00107	XM_01702	.	.	NP_00107	XP_011533	protein_co	ENSG0000	22862	20296
FNDC3B	8.58726	10.695	7.06084	8.71325	9.19189	8.69009	fibronectin type III domain containing 3B [Source:HGNC Symbol;A	chr3:17175	NM_00113	XM_01151	.	.	NP_00112	.	protein_co	ENSG0000	64778	24670
FNDC4	2.28864	2.69637	2.69888	2.10847	1.84312	1.98662	fibronectin type III domain containing 4 [Source:HGNC Symbol;Ac	chr2:27714	NM_02282	XM_00526	.	.	NP_07373	XP_005264	protein_co	ENSG0000	64838	20239
FNDC5	0	0	0	0	0	0	fibronectin type III domain containing 5 [Source:HGNC Symbol;Ac	chr1:33327	NM_00117	.	.	.	NP_00116	.	protein_co	ENSG0000	252995	20240
FNDC7	0	0	0.054866	0.023545	0	0.021532	fibronectin type III domain containing 7 [Source:HGNC Symbol;Ac	chr1:10925	NM_00114	XM_01154	.	.	NP_00113	.	protein_co	ENSG0000	163479	26668
FNDC8	0.052198	0	0	0	0	0	fibronectin type III domain containing 8 [Source:HGNC Symbol;Ac	chr17:3344	NM_01755	.	.	.	NP_06002	.	protein_co	ENSG0000	54752	25286
FNDC9	0	0	0	0	0	0	fibronectin type III domain containing 9 [Source:HGNC Symbol;Ac	chr5:15669	NM_00100	.	.	.	NP_00100	.	protein_co	ENSG0000	408263	33547
FNIP1	5.40881	5.37988	5.66161	5.21731	5.20076	6.50044	folliculin interacting protein 1 [Source:HGNC Symbol;Acc:HGNC:2	chr5:13075	NM_00100	.	.	.	NP_00100	.	protein_co	ENSG0000	96459	29418
FNIP2	1.41504	1.63831	1.57169	2.00386	2.28348	2.47514	folliculin interacting protein 2 [Source:HGNC Symbol;Acc:HGNC:2	chr4:15969	NM_00132	XM_01153	.	.	NP_00131	XP_005263	protein_co	ENSG0000	57600	29280
FNTA	26.7236	23.111	29.9919	23.7794	26.1555	33.6762	farnesyltransferase, CAAX box, alpha [Source:HGNC Symbol;Acc:H	chr8:42911	NM_00202	.	.	.	NP_00201	.	protein_co	ENSG0000	2339	3782
FNTB	15.9203	14.8602	13.1686	16.5427	17.5429	15.3569	farnesyltransferase, CAAX box, beta [Source:HGNC Symbol;Acc:H	chr14:6538	NM_00120	.	.	.	NP_00118	.	protein_co	ENSG0000	2342	3785

FOCAD	11.7449	12.5398	12.0942	12.5896	11.5103	14.5995	focadhesin [Source:HGNC Symbol;Acc:HGNC:23377]	chr9:20658	NM_01779	XM_01701	.	.	NP_06026	XP_005251	protein_co	ENSG0000	54914	23377
FOLH1	0	0	0	0	0	0	folate hydrolase 1 [Source:HGNC Symbol;Acc:HGNC:3788]	chr11:4916	NM_00101	XM_01701	.	.	NP_00101	XP_016872	protein_co	ENSG0000	219595	3788
FOLR1	3.06484	3.40552	5.76965	7.73729	6.68428	10.7886	folate receptor 1 [Source:HGNC Symbol;Acc:HGNC:3791]	chr11:7190	NM_00080	.	.	.	NP_00079	.	protein_co	ENSG0000	2348	3791
FOLR2	0	0	0	0	0	0	folate receptor beta [Source:HGNC Symbol;Acc:HGNC:3793]	chr11:7192	NM_00080	XM_01154	.	.	NP_00079	XP_005273	protein_co	ENSG0000	2350	3793
FOLR3	0.109545	0	0	0	0	0.119543	folate receptor 3 [Source:HGNC Symbol;Acc:HGNC:3795]	chr11:7184	NM_00080	XM_01154	.	.	NP_00079	XP_011543	protein_co	ENSG0000	2352	3795
FOPNL	35.5536	35.1224	38.5515	44.9179	42.9373	50.3417	FGFR1OP N-terminal like [Source:HGNC Symbol;Acc:HGNC:26435	chr16:1595	NM_00130	.	.	.	NP_00129	.	protein_co	ENSG0000	123811	26435
FOS	5.53618	6.13022	9.2525	6.04108	4.6885	5.73317	Fos proto-oncogene, AP-1 transcription factor subunit [Source:HC	chr14:7574	NM_00525	.	.	.	NP_00524	.	protein_co	ENSG0000	2353	3796
FOSB	2.48717	3.52007	1.39176	1.51276	1.58739	1.05341	FosB proto-oncogene, AP-1 transcription factor subunit [Source:H	chr19:4588	NM_00111	XM_00525	.	.	NP_00110	XP_005258	protein_co	ENSG0000	2354	3797
FOSL1	99.3747	104.164	59.5041	119.15	109.294	78.2944	FOS like 1, AP-1 transcription factor subunit [Source:HGNC Symbo	chr11:6565	NM_00130	.	.	.	NP_00128	.	protein_co	ENSG0000	8061	13718
FOSL2	11.9668	12.8799	10.7992	12.9415	15.5145	11.7534	FOS like 2, AP-1 transcription factor subunit [Source:HGNC Symbo	chr2:28615	NM_00525	XM_00671	.	.	NP_00524	XP_006712	protein_co	ENSG0000	2355	3798
FOXA1	1.13565	0.931797	1.37949	0.525026	1.12797	0.839324	forkhead box A1 [Source:HGNC Symbol;Acc:HGNC:5021]	chr14:3805	NM_00449	.	.	.	NP_00448	XP_016876	protein_co	ENSG0000	3169	5021
FOXA2	0.336124	0.410716	0.636166	0.406248	0.442802	0.509884	forkhead box A2 [Source:HGNC Symbol;Acc:HGNC:5022]	chr20:2256	NM_02178	XM_00672	.	.	NP_06855	XP_006723	protein_co	ENSG0000	3170	5022
FOXA3	5.17149	5.31486	5.19151	6.4346	5.11365	5.71017	forkhead box A3 [Source:HGNC Symbol;Acc:HGNC:5023]	chr19:4636	NM_00449	.	.	.	NP_00448	.	protein_co	ENSG0000	3171	5023
FOXB1	0	0	0	0	0	0	forkhead box B1 [Source:HGNC Symbol;Acc:HGNC:3799]	chr15:6029	NM_01218	.	.	.	NP_03631	.	protein_co	ENSG0000	27023	3799
FOXB2	0	0	0	0	0	0	forkhead box B2 [Source:HGNC Symbol;Acc:HGNC:23315]	chr9:79634	NM_00101	.	.	.	NP_00101	.	protein_co	ENSG0000	442425	23315
FOXC1	17.0614	17.4204	30.9151	19.2106	20.3355	37.6862	forkhead box C1 [Source:HGNC Symbol;Acc:HGNC:3800]	chr6:16102	NM_00145	.	.	.	NP_00144	.	protein_co	ENSG0000	2296	3800
FOXC2	0.046995	0.183821	0.040081	0.202593	0.055047	0.206768	forkhead box C2 [Source:HGNC Symbol;Acc:HGNC:3801]	chr16:8660	NM_00525	.	.	.	NP_00524	.	protein_co	ENSG0000	2303	3801
FOXD1	16.5592	16.6229	18.2021	22.8986	21.5164	22.6119	forkhead box D1 [Source:HGNC Symbol;Acc:HGNC:3802]	chr5:72742	NM_00447	.	.	.	NP_00446	.	protein_co	ENSG0000	2297	3802
FOXD2	1.10628	0.878535	0.837687	0.782764	0.70241	0.83237	forkhead box D2 [Source:HGNC Symbol;Acc:HGNC:3803]	chr1:47901	NM_00447	.	.	.	NP_00446	.	protein_co	ENSG0000	2306	3803
FOXD3	0.301078	0.141836	0.188976	0.078786	0.089825	0	forkhead box D3 [Source:HGNC Symbol;Acc:HGNC:3804]	chr1:63788	NM_01218	.	.	.	NP_03631	.	protein_co	ENSG0000	27022	3804
FOXD4	2.41874	2.93176	1.55173	2.20703	2.47946	2.21545	forkhead box D4 [Source:HGNC Symbol;Acc:HGNC:3805]	chr9:11623	NM_20730	.	.	.	NP_99718	.	protein_co	ENSG0000	2298	3805
FOXD4L1	0.404958	0.399566	0.345927	0.392617	0.37536	0.264843	forkhead box D4-like 1 [Source:HGNC Symbol;Acc:HGNC:18521]	chr2:11425	NM_01218	.	.	.	NP_03631	.	protein_co	ENSG0000	200350	18521
FOXD4L2	0	0	0	0	0	0	forkhead box D4-like 2 [Source:HGNC Symbol;Acc:HGNC:24813]	chr9:42717	NM_00109	.	.	.	NP_00109	.	protein_co	ENSG0000	349334	24813
FOXD4L3	0	0.162946	0	0.118861	0	0.061026	forkhead box D4-like 3 [Source:HGNC Symbol;Acc:HGNC:18523]	chr9:70917	NM_19913	.	.	.	NP_95458	.	protein_co	ENSG0000	286380	18523
FOXD4L4	0	0	0	0	0	0	forkhead box D4-like 4 [Source:HGNC Symbol;Acc:HGNC:23762]	chr9:70426	NM_19924	.	.	.	NP_95471	.	protein_co	ENSG0000	349334	23762
FOXD4L5	0	0	0	0.034534	0	0.033346	forkhead box D4-like 5 [Source:HGNC Symbol;Acc:HGNC:18522]	chr9:70175	NM_00112	.	.	.	NP_00111	.	protein_co	ENSG0000	653427	18522
FOXD4L6	0	0	0.032258	0	0.08677	0	forkhead box D4-like 6 [Source:HGNC Symbol;Acc:HGNC:31986]	chr9:69199	NM_00108	.	.	.	NP_00107	.	protein_co	ENSG0000	653404	31986
FOXE1	0	0	0	0	0	0	forkhead box E1 [Source:HGNC Symbol;Acc:HGNC:3806]	chr9:10061	NM_00447	.	.	.	NP_00446	.	protein_co	ENSG0000	2304	3806
FOXE3	0.33564	0.322257	0.280298	0.533777	0.490993	0.379666	forkhead box E3 [Source:HGNC Symbol;Acc:HGNC:3808]	chr1:47881	NM_01218	.	.	.	NP_03631	.	protein_co	ENSG0000	2301	3808
FOXF1	2.05945	2.34666	2.76018	2.24808	2.25854	2.33322	forkhead box F1 [Source:HGNC Symbol;Acc:HGNC:3809]	chr16:8654	NM_00145	.	.	.	NP_00144	.	protein_co	ENSG0000	2294	3809
FOXF2	0	0	0	0	0	0	forkhead box F2 [Source:HGNC Symbol;Acc:HGNC:3810]	chr6:13900	NM_00145	.	.	.	NP_00144	.	protein_co	ENSG0000	2295	3810
FOXG1	0	0	0	0	0	0	forkhead box G1 [Source:HGNC Symbol;Acc:HGNC:3811]	chr14:2923	NM_00524	.	.	.	NP_00524	.	protein_co	ENSG0000	2290	3811
FOXH1	0.149938	0.147605	0.28989	0	0.087108	0.124914	forkhead box H1 [Source:HGNC Symbol;Acc:HGNC:3814]	chr8:14569	NM_00392	.	.	.	NP_00391	.	protein_co	ENSG0000	8928	3814
FOXI1	0	0	0	0	0	0	forkhead box I1 [Source:HGNC Symbol;Acc:HGNC:3815]	chr5:16953	NM_01218	.	.	.	NP_03632	.	protein_co	ENSG0000	2299	3815
FOXI2	0	0	0	0	0	0	forkhead box I2 [Source:HGNC Symbol;Acc:HGNC:32448]	chr10:1295	NM_20742	.	.	.	NP_99730	.	protein_co	ENSG0000	399823	32448
FOXI3	0	0.037212	0	0	0	0	forkhead box I3 [Source:HGNC Symbol;Acc:HGNC:35123]	chr2:88746	NM_00113	.	.	.	NP_00112	.	protein_co	ENSG0000	344167	35123
FOXJ1	0.100016	0.064462	0.486252	0.070601	0.078427	0.037679	forkhead box J1 [Source:HGNC Symbol;Acc:HGNC:3816]	chr17:7413	NM_00145	.	.	.	NP_00144	.	protein_co	ENSG0000	2302	3816
FOXJ2	7.54414	8.21654	7.62907	7.99881	8.65631	9.34933	forkhead box J2 [Source:HGNC Symbol;Acc:HGNC:24818]	chr12:8185	NM_01841	XM_01152	.	.	NP_06088	XP_011519	protein_co	ENSG0000	55810	24818
FOXJ3	18.6168	20.4498	19.7351	17.9995	20.9433	20.9109	forkhead box J3 [Source:HGNC Symbol;Acc:HGNC:29178]	chr1:42642	NM_00119	XM_01700	.	.	NP_00118	XP_005270	protein_co	ENSG0000	22887	29178
FOXK1	13.9761	15.1987	15.999	12.2268	13.8864	12.7139	forkhead box K1 [Source:HGNC Symbol;Acc:HGNC:23480]	chr7:47219	NM_00103	.	.	.	NP_00103	.	protein_co	ENSG0000	221937	23480
FOXK2	36.6118	38.2505	40.2127	43.5141	45.9144	41.7636	forkhead box K2 [Source:HGNC Symbol;Acc:HGNC:6036]	chr17:8047	NM_00451	.	.	.	NP_00450	.	protein_co	ENSG0000	3607	6036
FOXL1	1.38194	1.45617	1.76176	2.45207	1.56887	2.77213	forkhead box L1 [Source:HGNC Symbol;Acc:HGNC:3817]	chr16:8661	NM_00525	.	.	.	NP_00524	.	protein_co	ENSG0000	2300	3817
FOXL2	0	0	0	0	0	0	forkhead box L2 [Source:HGNC Symbol;Acc:HGNC:1092]	chr3:13866	NM_02306	.	.	.	NP_07555	.	protein_co	ENSG0000	668	1092
FOXL2NB	0.671856	0.612468	0.428222	0.246325	0.253906	0.259662	FOXL2 neighbor [Source:HGNC Symbol;Acc:HGNC:34428]	chr3:13866	NM_00104	.	.	.	NP_00103	.	protein_co	ENSG0000	401089	34428
FOXM1	27.3508	32.2743	21.8773	39.9288	36.2705	22.3243	forkhead box M1 [Source:HGNC Symbol;Acc:HGNC:3818]	chr12:2966	NM_00124	XM_01152	.	.	NP_00123	XP_005253	protein_co	ENSG0000	2305	3818
FOXN1	0	0	0	0	0	0	forkhead box N1 [Source:HGNC Symbol;Acc:HGNC:12765]	chr17:2683	NM_00359	XM_01702	.	.	NP_00358	XP_005258	protein_co	ENSG0000	8456	12765
FOXN2	7.99621	8.89533	9.54672	7.86143	8.8809	10.7166	forkhead box N2 [Source:HGNC Symbol;Acc:HGNC:5281]	chr2:48541	NM_00215	XM_01700	.	.	NP_00214	XP_005264	protein_co	ENSG0000	3344	5281
FOXN3	5.49194	5.83414	7.68825	4.55346	5.08093	6.49137	forkhead box N3 [Source:HGNC Symbol;Acc:HGNC:1928]	chr14:8962	NM_00108	.	.	.	NP_00107	.	protein_co	ENSG0000	1112	1928
FOXN4	0.18495	0.16135	0.233943	0.249638	0.23787	0.336267	forkhead box N4 [Source:HGNC Symbol;Acc:HGNC:21399]	chr12:1097	NM_21359	XM_01153	.	.	NP_99876	XP_011536	protein_co	ENSG0000	121643	21399
FOXO1	3.29317	3.60196	3.85568	2.08213	2.36614	3.19023	forkhead box O1 [Source:HGNC Symbol;Acc:HGNC:3819]	chr13:4112	NM_00201	.	.	.	NP_00200	.	protein_co	ENSG0000	2308	3819
FOXO3	10.6303	11.5828	10.4705	8.34568	9.30538	13.9299	forkhead box O3 [Source:HGNC Symbol;Acc:HGNC:3821]	chr6:10888	NM_00145	XM_01153	.	.	NP_00144	XP_005266	protein_co	ENSG0000	2309	3821
FOXO4	5.02541	4.16769	4.99443	4.21074	4.45167	5.49215	forkhead box O4 [Source:HGNC Symbol;Acc:HGNC:7139]	chrX:70316	NM_00117	.	.	.	NP_00116	.	protein_co	ENSG0000	4303	7139
FOXO6	2.82237	2.49841	2.80231	2.61667	1.7383	2.6548	forkhead box O6 [Source:HGNC Symbol;Acc:HGNC:24814]	chr1:41827	NM_00129	.	.	.	NP_00127	.	protein_co	ENSG0000	100132074	24814
FOXP1	1.26645	1.80199	1.56335	1.28498	2.49979	3.07992	forkhead box P1 [Source:HGNC Symbol;Acc:HGNC:3823]	chr3:71003	NM_00101	XM_01700	.	.	NP_00101	XP_005264	protein_co	ENSG0000	27086	3823
FOXP2	0.443772	0.661309	0.783423	0.230684	0.342669	0.636017	forkhead box P2 [Source:HGNC Symbol;Acc:HGNC:13875]	chr7:11372	NM_00117	XM_01151	.	.	NP_00116	XP_016868	protein_co	ENSG0000	93986	13875
FOXP3	0.067276	0.063682	0.193167	0.141937	0.222445	0.293991	forkhead box P3 [Source:HGNC Symbol;Acc:HGNC:6106]	chrX:49091	NM_00111	XM_01154	.	.	NP_00110	XP_006724	protein_co	ENSG0000	50943	6106
FOXP4	12.8901	13.0831	13.4939	14.0677	12.9298	11.8663	forkhead box P4 [Source:HGNC Symbol;Acc:HGNC:20842]	chr6:41514	NM_00101	XM_01151	.	.	NP_00101	XP_006715	protein_co	ENSG0000	116113	20842
FOXQ1	0.131939	0.128296	0.559566	0.28552	0.153796	0.215632	forkhead box Q1 [Source:HGNC Symbol;Acc:HGNC:20951]	chr6:13126	NM_03326	.	.	.	NP_15028	.	protein_co	ENSG0000	94234	20951
FOXR1	0	0	0	0	0	0	forkhead box R1 [Source:HGNC Symbol;Acc:HGNC:29980]	chr11:1188	NM_18172	XM_01154	.	.	NP_85907	XP_016873	protein_co	ENSG0000	283150	29980

FOXR2	0	0	0	0	0	0	forkhead box R2 [Source:HGNC Symbol;Acc:HGNC:30469]	chrX:55649	NM_19845	.	.	.	NP_94085	.	protein_co	ENSG0000	139628	30469
FOXRED1	24.1117	22.8876	22.1342	22.9542	22.0998	17.3667	FAD dependent oxidoreductase domain containing 1 [Source:HGI	chr11:1261	NM_01754	XM_01701	.	.	NP_06001	XP_011541	protein_co	ENSG0000	55572	26927
FOXRED2	7.71611	7.80586	5.89267	8.96727	8.15753	5.56188	FAD dependent oxidoreductase domain containing 2 [Source:HGI	chr22:3688	NM_00110	XM_01153	.	.	NP_00109	.	protein_co	ENSG0000	80020	26264
FOXS1	0	0.075167	0	0	0	0.086981	forkhead box S1 [Source:HGNC Symbol;Acc:HGNC:3735]	chr20:3043	NM_00411	.	.	.	NP_00410	.	protein_co	ENSG0000	2307	3735
FP15737	3.83E-06	3.94E-06	4.64E-06	1.82E-06	6.01E-06	2.28E-05	.	chr8:2338	.	.	.	.	.	.	protein_co	ENSG0000	.	.
FPGS	32.455	34.8267	32.471	36.262	28.6893	23.6991	folylpolyglutamate synthase [Source:HGNC Symbol;Acc:HGNC:38	chr9:1305	NM_00101	XM_01151	NR_11017	.	NP_00101	XP_005251	protein_co	ENSG0000	2356	3824
FPGT	4.93981	6.25728	4.29918	4.24025	4.69511	5.12612	fucose-1-phosphate guanylyltransferase [Source:HGNC Symbol;A	chr1:7466	NM_00119	.	.	.	NP_00118	.	protein_co	ENSG0000	8790	3825
FPGT-TNNI3K	0.22365	0.331367	0.393552	0.168278	0.190848	0.265484	FPGT-TNNI3K readthrough [Source:HGNC Symbol;Acc:HGNC:429	chr1:7466	NM_00111	.	.	.	NP_00110	.	protein_co	ENSG0000	100526835	42952
FPR1	0	0.080728	0.067877	0.215797	0.176578	0.241838	formyl peptide receptor 1 [Source:HGNC Symbol;Acc:HGNC:3826	chr19:5224	NM_00119	.	.	.	NP_00118	.	protein_co	ENSG0000	2357	3826
FPR2	0	0.04245	0	0	0	0	formyl peptide receptor 2 [Source:HGNC Symbol;Acc:HGNC:3827	chr19:5226	NM_00100	XM_00672	.	.	NP_00100	XP_006723	protein_co	ENSG0000	2358	3827
FPR3	0	0	0	0	0	0	formyl peptide receptor 3 [Source:HGNC Symbol;Acc:HGNC:3828	chr19:5229	NM_00203	XM_01152	.	.	NP_00202	XP_011524	protein_co	ENSG0000	2359	3828
FRA10AC1	6.64123	6.95594	6.04248	7.87246	7.50384	8.21116	fragile site, folic acid type, rare, fra(10)(q23.3) or fra(10)(q24.2) c	chr10:9542	NM_14524	XM_01153	.	.	NP_66028	XP_005269	protein_co	ENSG0000	118924	1162
FRAS1	3.98766	4.3337	9.69332	2.02265	2.32385	4.83311	Fraser extracellular matrix complex subunit 1 [Source:HGNC Sym	chr4:7897	NM_00116	XM_00671	.	.	NP_00115	XP_006714	protein_co	ENSG0000	80144	19185
FRAT1	0.953684	0.32533	0.706929	0.403388	0.384917	0.994229	frequently rearranged in advanced T-cell lymphomas 1 [Source:H	chr10:9907	NM_00547	.	.	.	NP_00547	.	protein_co	ENSG0000	10023	3944
FRAT2	6.85849	8.29679	7.93189	5.30516	4.55247	8.25432	frequently rearranged in advanced T-cell lymphomas 2 [Source:H	chr10:9909	NM_01208	.	.	.	NP_03621	.	protein_co	ENSG0000	23401	16048
FREM1	0	0	0	0	0	0	FRAS1 related extracellular matrix 1 [Source:HGNC Symbol;Acc:H	chr9:1473	NM_00117	XM_01151	.	.	NP_00117	XP_005251	protein_co	ENSG0000	158326	23399
FREM2	0	0.00478	0	0	0	0	FRAS1 related extracellular matrix protein 2 [Source:HGNC Symb	chr13:392	NM_20736	.	.	.	NP_99724	.	protein_co	ENSG0000	341640	25396
FREM3	0	0	0	0	0	0	FRAS1 related extracellular matrix 3 [Source:HGNC Symbol;Acc:H	chr4:1444	NM_00116	.	.	.	NP_00116	.	protein_co	ENSG0000	166752	25172
FRG1	32.4461	29.559	25.4418	26.2287	25.6571	29.2968	FSHD region gene 1 [Source:HGNC Symbol;Acc:HGNC:3954]	chr4:1908	NM_00447	.	.	.	NP_00446	.	protein_co	ENSG0000	2483	3954
FRG2	0.038271	0	0.066965	0	0.085705	0	FSHD region gene 2 [Source:HGNC Symbol;Acc:HGNC:19136]	chr4:19094	NM_00100	XM_01153	.	.	NP_00100	XP_01153	protein_co	ENSG0000	448831	19136
FRG2B	0	0	0	0	0	0	FSHD region gene 2 family member B [Source:HGNC Symbol;Acc:	chr10:1354	NM_00108	XM_01153	.	.	NP_00107	XP_011538	protein_co	ENSG0000	441581	33518
FRG2C	0	0	0	0	0.042475	0	FSHD region gene 2 family member C [Source:HGNC Symbol;Acc:	chr3:7571	NM_00112	XM_01153	.	.	NP_00111	XP_011531	protein_co	ENSG0000	100288801	33626
FRK	1.23782	1.30888	1.70506	1.0101	1.32409	2.0483	fyn related Src family tyrosine kinase [Source:HGNC Symbol;Acc:H	chr6:1162	NM_00203	XM_01153	.	.	NP_00202	XP_00526	protein_co	ENSG0000	2444	3955
FRMD1	0	0	0	0	0	0	FERM domain containing 1 [Source:HGNC Symbol;Acc:HGNC:212	chr6:1684	NM_00112	XM_01153	.	.	NP_00111	XP_011534	protein_co	ENSG0000	79981	21240
FRMD3	0.352157	0.439579	0.336246	0.127347	0.3223	0.217939	FERM domain containing 3 [Source:HGNC Symbol;Acc:HGNC:241	chr9:8585	NM_00124	XM_01701	.	.	NP_00123	XP_01687	protein_co	ENSG0000	257019	24125
FRMD4A	0.24669	0.147636	0.214239	0.079607	0.140228	0.128265	FERM domain containing 4A [Source:HGNC Symbol;Acc:HGNC:25	chr10:1362	NM_00131	XM_01151	.	.	NP_00130	XP_005252	protein_co	ENSG0000	55691	25491
FRMD4B	0.728996	0.721595	0.834638	0.906596	1.17326	1.35848	FERM domain containing 4B [Source:HGNC Symbol;Acc:HGNC:24	chr3:6921	NM_01512	XM_01700	.	.	NP_05593	XP_005264	protein_co	ENSG0000	23150	24886
FRMD5	17.335	17.6994	22.387	21.0021	18.9178	21.9137	FERM domain containing 5 [Source:HGNC Symbol;Acc:HGNC:282	chr15:441	NM_00128	XM_01152	.	.	NP_00127	XP_005254	protein_co	ENSG0000	84978	28214
FRMD6	3.63832	3.83414	4.3715	3.94638	4.39514	6.51457	FERM domain containing 6 [Source:HGNC Symbol;Acc:HGNC:198	chr14:519	NM_00104	XM_01153	.	.	NP_00103	XP_00672	protein_co	ENSG0000	122786	19839
FRMD7	0	0	0	0	0	0	FERM domain containing 7 [Source:HGNC Symbol;Acc:HGNC:807	chrX:1312	NM_00130	XM_01702	.	.	NP_00129	XP_005262	protein_co	ENSG0000	90167	8079
FRMD8	16.1438	16.1578	19.4675	17.3623	16.6494	12.1094	FERM domain containing 8 [Source:HGNC Symbol;Acc:HGNC:254	chr11:651	NM_00130	.	.	.	NP_00128	.	protein_co	ENSG0000	83786	25462
FRMPD1	0.171466	0.201205	0.206093	0.273835	0.240452	0.196177	FERM and PDZ domain containing 1 [Source:HGNC Symbol;Acc:H	chr9:3765	NM_01490	XM_01701	.	.	NP_05572	XP_01151	protein_co	ENSG0000	22844	29159
FRMPD2	0.059855	0.073479	0.180524	0.112926	0.080616	0.124978	FERM and PDZ domain containing 2 [Source:HGNC Symbol;Acc:H	chr10:493	NM_00101	XM_01153	.	.	NP_00101	XP_011537	protein_co	ENSG0000	143162	28572
FRMPD3	0.242498	0.282372	0.31017	0.115194	0.22311	0.179247	FERM and PDZ domain containing 3 [Source:HGNC Symbol;Acc:H	chrX:1067	NM_03242	XM_01702	.	.	NP_11580	XP_006724	protein_co	ENSG0000	84443	29382
FRMPD4	0	0	0	0	0	0	FERM and PDZ domain containing 4 [Source:HGNC Symbol;Acc:H	chrX:1215	NM_01472	XM_01702	.	.	NP_05554	XP_005274	protein_co	ENSG0000	9758	29007
FRRS1	3.66117	3.32599	3.91376	3.33505	3.45526	3.88036	ferric chelate reductase 1 [Source:HGNC Symbol;Acc:HGNC:2762	chr1:1001	NM_00101	XM_01154	.	.	NP_00101	XP_00527	protein_co	ENSG0000	391059	27622
FRRS1L	0	0	0	0	0	0	ferric chelate reductase 1 like [Source:HGNC Symbol;Acc:HGNC:1	chr9:1118	NM_01433	.	.	.	NP_05514	.	protein_co	ENSG0000	23732	1362
FRS2	6.04959	6.94141	5.81011	4.7491	6.6339	6.31182	fibroblast growth factor receptor substrate 2 [Source:HGNC Symb	chr12:698	NM_00104	XM_01701	.	.	NP_00103	XP_016874	protein_co	ENSG0000	10818	16971
FRS3	2.65737	2.65015	2.38185	1.96013	2.20549	2.4808	fibroblast growth factor receptor substrate 3 [Source:HGNC Symb	chr6:4173	NM_00665	XM_01151	.	.	NP_00664	XP_011512	protein_co	ENSG0000	10817	16970
FRY	0.329509	0.282113	0.38936	0.191349	0.200537	0.370784	FRY microtubule binding protein [Source:HGNC Symbol;Acc:HGN	chr13:326	NM_02303	XM_01702	.	.	NP_07546	XP_006719	protein_co	ENSG0000	10129	20367
FRYL	8.36877	9.53791	8.0356	8.07204	11.1912	11.7074	FRY like transcription coactivator [Source:HGNC Symbol;Acc:HGN	chr4:4849	NM_01503	XM_01700	.	.	NP_05584	XP_005248	protein_co	ENSG0000	285527	29127
FRZB	0.286199	0.233099	0.355388	0.143054	0.142405	0.351141	frizzled-related protein [Source:HGNC Symbol;Acc:HGNC:3959]	chr2:1836	NM_00146	.	.	.	NP_00145	.	protein_co	ENSG0000	2487	3959
FSBP	2.3E-06	0.259668	0.256221	1.43E-06	0.170832	0.350616	fibrinogen silencer binding protein [Source:HGNC Symbol;Acc:HG	chr8:95384	NM_00125	.	.	.	NP_00124	.	protein_co	ENSG0000	100861412	43653
FSCB	0	0	0	0	0	0	fibrous sheath CABYR binding protein [Source:HGNC Symbol;Acc:	chr14:4497	NM_03213	.	.	.	NP_11551	.	protein_co	ENSG0000	84075	20494
FSCN1	154.906	167.921	170.742	227.896	196.824	197.338	fascin actin-bundling protein 1 [Source:HGNC Symbol;Acc:HGNC:	chr7:56324	NM_00308	.	.	.	NP_00307	.	protein_co	ENSG0000	6624	11148
FSCN2	1.04437	1.22493	1.79818	0.063276	0.63902	0.389417	fascin actin-bundling protein 2, retinal [Source:HGNC Symbol;Acc	chr17:794	NM_00107	XM_01152	.	.	NP_00107	XP_005257	protein_co	ENSG0000	25794	3960
FSCN3	0.064377	0.032805	0.167046	0.035158	0.036692	0.03392	fascin actin-bundling protein 3 [Source:HGNC Symbol;Acc:HGNC:	chr7:1272	NM_02036	.	.	.	NP_06510	.	protein_co	ENSG0000	29999	3961
FSD1	10.724	9.85417	12.3652	11.3343	9.18916	8.19222	fibronectin type III and SPRY domain containing 1 [Source:HGNC	chr19:4304	NM_02433	XM_00525	.	.	NP_07730	XP_005259	protein_co	ENSG0000	79187	13745
FSD1L	2.28851	2.61867	1.919	2.08496	3.05789	2.52731	fibronectin type III and SPRY domain containing 1 like [Source:HG	chr9:10821	NM_00114	XM_01701	.	.	NP_00113	XP_005252	protein_co	ENSG0000	83856	13753
FSD2	0	0	0.042207	0	0	0	fibronectin type III and SPRY domain containing 2 [Source:HGNC	chr15:8342	NM_00100	XM_01152	.	.	NP_00100	XP_005272	protein_co	ENSG0000	123722	18024
FSHB	0	0	0	0	0	0	follicle stimulating hormone beta subunit [Source:HGNC Symbol;	chr11:302	NM_00051	XM_01151	.	.	NP_00050	XP_011518	protein_co	ENSG0000	2488	3964
FSHR	0	0	0	0	0	0	follicle stimulating hormone receptor [Source:HGNC Symbol;Acc:	chr2:4918	NM_00014	XM_01153	.	.	NP_00013	XP_011531	protein_co	ENSG0000	2492	3969
FSIP1	0.268114	0.078804	0.3415	0.224223	0.480624	0.282591	fibrous sheath interacting protein 1 [Source:HGNC Symbol;Acc:H	chr15:398	NM_00132	XM_01152	.	.	NP_00131	XP_005254	protein_co	ENSG0000	161835	21674
FSIP2	0	0.007733	0	0	0	0	fibrous sheath interacting protein 2 [Source:HGNC Symbol;Acc:H	chr2:1866	NM_17365	XM_01151	.	.	NP_77592	.	protein_co	ENSG0000	401024	21675
FST	0.102626	0.178362	0.087034	0.076689	0.076518	0.068971	follistatin [Source:HGNC Symbol;Acc:HGNC:3971]	chr5:5277	NM_00635	XM_00524	.	.	NP_00634	XP_005248	protein_co	ENSG0000	10468	3971
FSTL1	6.26066	6.50217	6.47282	6.17504	6.87552	5.70449	follistatin like 1 [Source:HGNC Symbol;Acc:HGNC:3972]	chr3:12011	NM_00708	.	NR_02958	.	NP_00901	.	protein_co	ENSG0000	406975	3972
FSTL3	16.9096	17.6119	14.2579	13.6462	10.7671	9.50304	follistatin like 3 [Source:HGNC Symbol;Acc:HGNC:3973]	chr19:676	NM_00586	.	.	.	NP_00585	.	protein_co	ENSG0000	10272	3973
FSTL4	0.224324	0.257495	0.132645	0.27647	0.117805	0.136935	follistatin like 4 [Source:HGNC Symbol;Acc:HGNC:21389]	chr5:1325	NM_01508	XM_01154	.	.	NP_05589	XP_011541	protein_co	ENSG0000	23105	21389
FSTL5	0.090678	0	0.039531	0	0	0.015349	follistatin like 5 [Source:HGNC Symbol;Acc:HGNC:21386]	chr4:1623	NM_00112	XM_01153	.	.	NP_00112	XP_01153	protein_co	ENSG0000	56884	21386

FTCD	0.100949	0.086964	0.086353	0	0.111112	0.050672	formimidoyltransferase cyclodeaminase [Source:HGNC Symbol;A	chr21:4755	NM_00132	XM_01152	.	.	NP_00130	XP_006724	protein_co	ENSG0000	10841	3974
FTCDNL1	1.5713	0.745229	1.09296	0.737854	0.889938	0.812141	formiminotransferase cyclodeaminase N-terminal like [Source:H	chr2:20062	.	.	.	.	.	.	protein_co	ENSG0000	.	48661
FTH1	1896.95	1858.84	1202.02	2295.66	1882.76	1722.06	ferritin heavy chain 1 [Source:HGNC Symbol;Acc:HGNC:3976]	chr11:6171	NM_00203	.	.	.	NP_00202	.	protein_co	ENSG0000	2495	3976
FTHL17	0	0	0	0	0	0	ferritin heavy chain like 17 [Source:HGNC Symbol;Acc:HGNC:3987	chrX:31089	NM_03189	.	.	.	NP_11410	.	protein_co	ENSG0000	53940	3987
FTL	2181.51	2097.57	1536.41	1886.9	1403.56	1705.17	ferritin light chain [Source:HGNC Symbol;Acc:HGNC:3999]	chr19:4946	NM_00014	.	.	.	NP_00013	.	protein_co	ENSG0000	2512	3999
FTMT	0	0	0	0	0	0	ferritin mitochondrial [Source:HGNC Symbol;Acc:HGNC:17345]	chr5:12118	NM_17747	.	.	.	NP_80343	.	protein_co	ENSG0000	94033	17345
FTO	20.4575	22.646	26.6131	17.976	17.9144	20.0621	fat mass and obesity associated [Source:HGNC Symbol;Acc:HGNC	chr16:5363	NM_00108	XM_01152	.	.	NP_00107	XP_011521	protein_co	ENSG0000	79068	24678
FTSJ1	36.3142	34.4882	29.0129	38.8932	35.4874	27.9125	FtsJ RNA methyltransferase homolog 1 (E. coli) [Source:HGNC Syn	chrX:48334	NM_00128	XM_01154	.	.	NP_00126	XP_005272	protein_co	ENSG0000	24140	13254
FTSJ3	36.3068	36.3419	33.2938	40.0601	40.038	25.6433	FtsJ homolog 3 [Source:HGNC Symbol;Acc:HGNC:17136]	chr17:6189	NM_01764	.	.	.	NP_06011	.	protein_co	ENSG0000	117246	17136
FUBP1	65.3853	62.9415	51.6868	67.3118	68.9477	53.048	far upstream element binding protein 1 [Source:HGNC Symbol;Ac	chr1:78414	NM_00130	XM_01154	.	.	NP_00129	XP_011540	protein_co	ENSG0000	8880	4004
FUBP3	33.2486	38.0899	35.1612	38.5507	36.4211	33.6733	far upstream element binding protein 3 [Source:HGNC Symbol;Ac	chr9:13345	NM_00393	XM_01151	.	.	NP_00392	XP_005272	protein_co	ENSG0000	8939	4005
FUCA1	22.3216	21.2195	35.6101	19.1368	21.2297	26.4098	fucosidase, alpha-L- 1, tissue [Source:HGNC Symbol;Acc:HGNC:40	chr1:24171	NM_00014	XM_00524	.	.	NP_00013	XP_005245	protein_co	ENSG0000	2517	4006
FUCA2	42.6994	41.3642	34.2995	39.0656	37.2232	34.1045	fucosidase, alpha-L- 2, plasma [Source:HGNC Symbol;Acc:HGNC:4	chr6:14381	NM_03202	.	.	.	NP_11440	.	protein_co	ENSG0000	2519	4008
FUK	5.80091	5.78795	5.59357	4.2635	3.76833	6.66941	fucokinase [Source:HGNC Symbol;Acc:HGNC:29500]	chr16:7048	NM_14505	XM_01702	.	.	NP_65949	XP_006721	protein_co	ENSG0000	197258	29500
FUNDC1	17.7143	15.3337	14.7242	20.2033	23.7313	20.5961	FUN14 domain containing 1 [Source:HGNC Symbol;Acc:HGNC:28	chrX:44382	NM_17379	.	.	.	NP_77615	.	protein_co	ENSG0000	139341	28746
FUNDC2	5.93894	5.92732	4.48567	5.1466	4.05166	4.59644	FUN14 domain containing 2 [Source:HGNC Symbol;Acc:HGNC:24	chrX:15425	NM_02393	.	.	.	NP_07642	.	protein_co	ENSG0000	65991	24925
FUOM	10.5934	9.70404	12.6026	10.0466	6.64142	6.35671	fucose mutarotase [Source:HGNC Symbol;Acc:HGNC:24733]	chr10:1351	NM_00109	XM_01153	.	.	NP_00109	XP_011537	protein_co	ENSG0000	282969	24733
FURIN	20.6518	21.6756	21.9638	20.4486	17.9035	21.5568	furin, paired basic amino acid cleaving enzyme [Source:HGNC Syn	chr15:9141	NM_00128	.	.	.	NP_00127	.	protein_co	ENSG0000	5045	8568
FUS	194.856	186.233	170.68	196.723	195.305	183.792	FUS RNA binding protein [Source:HGNC Symbol;Acc:HGNC:4010]	chr16:3119	NM_00117	XM_01154	.	.	NP_00116	XP_005255	protein_co	ENSG0000	2521	4010
FUT1	6.5292	6.74765	5.47304	5.79708	5.61003	9.41377	fucosyltransferase 1 (H blood group) [Source:HGNC Symbol;Acc:H	chr19:4925	NM_00014	XM_01702	.	.	NP_00013	XP_006723	protein_co	ENSG0000	2523	4012
FUT10	6.98504	7.33268	6.8794	8.59714	8.58434	7.51517	fucosyltransferase 10 [Source:HGNC Symbol;Acc:HGNC:19234]	chr8:33228	NM_03266	.	.	.	NP_11605	.	protein_co	ENSG0000	84750	19234
FUT11	9.35994	9.78989	10.4272	9.95998	10.0962	10.2209	fucosyltransferase 11 [Source:HGNC Symbol;Acc:HGNC:19233]	chr10:7553	NM_00128	.	.	.	NP_00127	XP_006717	protein_co	ENSG0000	170384	19233
FUT2	2.65947	3.7402	2.80462	3.31255	2.69993	2.79965	fucosyltransferase 2 [Source:HGNC Symbol;Acc:HGNC:4013]	chr19:4919	NM_00051	.	.	.	NP_00050	.	protein_co	ENSG0000	2524	4013
FUT3	0.980499	0.733489	0.601686	0.197627	0.141585	1.17824	fucosyltransferase 3 (Lewis blood group) [Source:HGNC Symbol;A	chr19:5842	NM_00014	XM_01152	.	.	NP_00014	XP_011526	protein_co	ENSG0000	2525	4014
FUT4	4.52306	4.86369	4.43404	4.6248	4.53059	6.36352	fucosyltransferase 4 [Source:HGNC Symbol;Acc:HGNC:4015]	chr11:9427	NM_00203	.	.	.	NP_00202	.	protein_co	ENSG0000	2526	4015
FUT5	0.045922	0	0.039323	0	0.107354	0.10211	fucosyltransferase 5 [Source:HGNC Symbol;Acc:HGNC:4016]	chr19:5865	NM_00203	.	.	.	NP_00202	.	protein_co	ENSG0000	2527	4016
FUT6	0	0.032703	0	0	0	0	fucosyltransferase 6 [Source:HGNC Symbol;Acc:HGNC:4017]	chr19:5830	NM_00015	XM_01152	.	.	NP_00014	XP_005259	protein_co	ENSG0000	2528	4017
FUT7	0	0	0.029593	0	0	0	fucosyltransferase 7 [Source:HGNC Symbol;Acc:HGNC:4018]	chr9:13992	NM_00447	.	.	.	NP_00447	.	protein_co	ENSG0000	2529	4018
FUT8	8.58564	8.54979	9.25222	6.55721	8.59169	10.9011	fucosyltransferase 8 [Source:HGNC Symbol;Acc:HGNC:4019]	chr14:6587	NM_00448	XM_01153	.	.	NP_00447	XP_011534	protein_co	ENSG0000	2530	4019
FUT9	0	0	0	0	0	0	fucosyltransferase 9 [Source:HGNC Symbol;Acc:HGNC:4020]	chr6:96463	NM_00658	XM_01701	.	.	NP_00657	XP_011533	protein_co	ENSG0000	10690	4020
FUZ	0.454193	0.317828	0.699575	1.0031	0.687128	0.575587	fuzzy planar cell polarity protein [Source:HGNC Symbol;Acc:HGN	chr19:5027	NM_00117	XM_01702	.	.	NP_00116	XP_006723	protein_co	ENSG0000	80199	26219
FXN	23.9176	22.0501	21.8147	25.7966	19.895	16.4964	frataxin [Source:HGNC Symbol;Acc:HGNC:3951]	chr9:71650	NM_00014	.	.	.	NP_00013	.	protein_co	ENSG0000	2395	3951
FXR1	82.9436	80.2048	87.4912	84.8662	98.5514	97.0025	FMR1 autosomal homolog 1 [Source:HGNC Symbol;Acc:HGNC:40	chr3:18063	NM_00101	XM_01151	.	.	NP_00101	XP_005247	protein_co	ENSG0000	8087	4023
FXR2	29.4296	30.8054	26.0123	33.9024	30.3305	27.7973	FMR1 autosomal homolog 2 [Source:HGNC Symbol;Acc:HGNC:40	chr17:7486	NM_00486	.	.	.	NP_00485	.	protein_co	ENSG0000	9513	4024
FXYD1	1.84751	3.20674	2.04774	1.17249	1.03511	3.1334	FXYD domain containing ion transport regulator 1 [Source:HGNC	chr19:3562	NM_00127	.	.	.	NP_00126	XP_016882	protein_co	ENSG0000	5348	4025
FXYD2	5.2E-08	0.21965	0.18894	0	0	0	FXYD domain containing ion transport regulator 2 [Source:HGNC	chr11:1176	NM_00168	.	.	.	NP_00167	.	protein_co	ENSG0000	486	4026
FXYD3	10.4105	11.3214	14.5419	19.391	16.41	15.6309	FXYD domain containing ion transport regulator 3 [Source:HGNC	chr19:3560	NM_00113	XM_01702	NR_10694	.	NP_00112	XP_005259	protein_co	ENSG0000	10246620	4027
FXYD4	0.249245	0.125652	0.330405	2.56063	1.4269	2.14447	FXYD domain containing ion transport regulator 4 [Source:HGNC	chr10:4386	NM_17316	.	.	.	NP_00117	.	protein_co	ENSG0000	53828	4028
FXYD5	53.1112	54.5108	50.8606	58.0929	40.8187	42.0327	FXYD domain containing ion transport regulator 5 [Source:HGNC	chr19:3564	NM_00116	XM_00672	.	.	NP_00115	XP_006723	protein_co	ENSG0000	53827	4029
FXYD6	1.63092	1.62164	2.44656	2.39772	1.58632	3.60545	FXYD domain containing ion transport regulator 6 [Source:HGNC	chr11:1176	NM_00116	.	.	.	NP_00115	.	protein_co	ENSG0000	53826	4030
FXYD6-FXYD2	0.401988	3.11E-38	1.84E-45	1.33E-41	7.16E-53	1E-111	FXYD6-FXYD2 readthrough [Source:HGNC Symbol;Acc:HGNC:399	chr11:1176	NM_00120	.	.	.	NP_00119	.	protein_co	ENSG0000	100533181	39978
FXYD7	0.349914	0.156378	0.968735	0	0.221524	0.209473	FXYD domain containing ion transport regulator 7 [Source:HGNC	chr19:3563	NM_02200	.	.	.	NP_07128	.	protein_co	ENSG0000	53822	4034
FYB	0	0	0.024394	0	0	0	FYN binding protein [Source:HGNC Symbol;Acc:HGNC:4036]	chr5:39105	NM_00124	XM_01700	.	.	NP_00123	XP_006714	protein_co	ENSG0000	2533	4036
FYCO1	5.58697	6.26028	7.17078	5.90816	6.41721	6.04155	FYVE and coiled-coil domain containing 1 [Source:HGNC Symbol;	chr3:45959	NM_02451	XM_01153	.	.	NP_07878	XP_006713	protein_co	ENSG0000	79443	14673
FYN	6.01319	6.99458	5.70603	5.47488	5.21075	9.30299	FYN proto-oncogene, Src family tyrosine kinase [Source:HGNC Sy	chr6:11198	NM_00203	XM_01153	.	.	NP_00202	XP_005266	protein_co	ENSG0000	2534	4037
FYTTD1	25.8252	24.3474	27.7028	25.3422	29.4486	27.5887	forty-two-three domain containing 1 [Source:HGNC Symbol;Acc:H	chr3:19739	NM_00101	.	.	.	NP_00101	.	protein_co	ENSG0000	84248	25407
FZD1	4.16082	3.87294	3.65974	4.19152	3.40726	3.28616	frizzled class receptor 1 [Source:HGNC Symbol;Acc:HGNC:4038]	chr7:90893	NM_00350	.	.	.	NP_00349	.	protein_co	ENSG0000	8321	4038
FZD10	0	0	0	0	0	0	frizzled class receptor 10 [Source:HGNC Symbol;Acc:HGNC:4039]	chr12:1306	NM_00719	.	.	.	NP_00912	.	protein_co	ENSG0000	11211	4039
FZD2	10.3103	10.9412	12.3376	9.44932	8.84734	9.75695	frizzled class receptor 2 [Source:HGNC Symbol;Acc:HGNC:4040]	chr17:4263	NM_00146	.	.	.	NP_00145	.	protein_co	ENSG0000	2535	4040
FZD3	2.64914	2.73105	2.3684	2.94651	3.31565	3.35823	frizzled class receptor 3 [Source:HGNC Symbol;Acc:HGNC:4041]	chr8:28351	NM_01741	XM_01154	.	.	NP_05910	.	protein_co	ENSG0000	7976	4041
FZD4	3.44918	3.56374	3.17927	3.31701	4.09576	5.27891	frizzled class receptor 4 [Source:HGNC Symbol;Acc:HGNC:4042]	chr11:8651	NM_01219	.	.	.	NP_03632	.	protein_co	ENSG0000	8322	4042
FZD5	4.04137	4.46749	4.98653	3.81998	4.3277	4.54631	frizzled class receptor 5 [Source:HGNC Symbol;Acc:HGNC:4043]	chr2:20862	NM_00346	.	.	.	NP_00345	.	protein_co	ENSG0000	7855	4043
FZD6	25.8297	23.7233	22.8205	24.3846	28.329	32.2438	frizzled class receptor 6 [Source:HGNC Symbol;Acc:HGNC:4044]	chr8:10431	NM_00116	.	.	.	NP_00115	.	protein_co	ENSG0000	8323	4044
FZD7	4.89912	4.97449	5.81356	4.88869	5.43066	8.00086	frizzled class receptor 7 [Source:HGNC Symbol;Acc:HGNC:4045]	chr2:20289	NM_00350	.	.	.	NP_00349	.	protein_co	ENSG0000	8324	4045
FZD8	0	0.066483	0.145191	0.147292	0.081468	0.114713	frizzled class receptor 8 [Source:HGNC Symbol;Acc:HGNC:4046]	chr10:3592	NM_03186	.	NR_03983	.	NP_11407	.	protein_co	ENSG0000	10061650	4046
FZD9	0.485367	0.409337	0.770732	0.905017	0.514012	0.678902	frizzled class receptor 9 [Source:HGNC Symbol;Acc:HGNC:4047]	chr7:72848	NM_00350	.	.	.	NP_00349	.	protein_co	ENSG0000	8326	4047
FZR1	17.3875	17.812	17.1697	21.6522	18.8873	17.8467	fizzy/cell division cycle 20 related 1 [Source:HGNC Symbol;Acc:H	chr19:3506	NM_00113	XM_00525	.	.	NP_00112	XP_005259	protein_co	ENSG0000	51343	24824
G0S2	0	0	0	0	0	0	G0/G1 switch 2 [Source:HGNC Symbol;Acc:HGNC:30229]	chr1:20984	NM_01571	.	.	.	NP_05652	.	protein_co	ENSG0000	50486	30229
G2E3	9.83995	9.28254	11.882	11.7717	18.4031	15.9497	G2/M-phase specific E3 ubiquitin protein ligase [Source:HGNC Sy	chr14:3102	NM_00130	XM_01153	.	.	NP_00129	XP_011535	protein_co	ENSG0000	55632	20338

G3BP1	115.155	120.983	108.366	145.996	128.165	120.324	G3BP stress granule assembly factor 1 [Source:HGNC Symbol;Acc	chr5:15112	NM_00575	XM_00671	.	.	NP_00574	XP_006714	protein_co	ENSG0000	10146	30292
G3BP2	68.9259	70.6207	78.4528	78.5935	91.2528	96.1322	G3BP stress granule assembly factor 2 [Source:HGNC Symbol;Acc	chr4:76567	NM_01229	XM_01700	.	.	NP_03642	XP_005263	protein_co	ENSG0000	9908	30291
G6PC	0	0	0	0	0	0	glucose-6-phosphatase catalytic subunit [Source:HGNC Symbol;A	chr17:4105	NM_00015	.	.	.	NP_00014	.	protein_co	ENSG0000	2538	4056
G6PC2	0	0	0	0	0	0	glucose-6-phosphatase catalytic subunit 2 [Source:HGNC Symbol;	chr2:16975	NM_00108	XM_01151	.	.	NP_00107	XP_011509	protein_co	ENSG0000	57818	28906
G6PC3	59.2413	57.4383	50.2469	45.5313	39.9789	31.3599	glucose-6-phosphatase catalytic subunit 3 [Source:HGNC Symbol;	chr17:4214	NM_13838	XM_01152	.	.	NP_61239	XP_011523	protein_co	ENSG0000	92579	24861
G6PD	41.8796	47.144	48.8878	42.7402	41.5798	45.8785	glucose-6-phosphate dehydrogenase [Source:HGNC Symbol;Acc:I	chrX:15375	NM_00040	XM_00527	.	.	NP_00039	.	protein_co	ENSG0000	2539	4057
GAA	39.7145	43.057	51.8914	41.1629	38.781	35.5947	glucosidase alpha, acid [Source:HGNC Symbol;Acc:HGNC:4065]	chr17:7807	NM_00015	XM_00525	NR_13484	.	NP_00014	XP_005257	protein_co	ENSG0000	2548	4065
GAB1	0.952724	1.06363	1.33329	0.693593	0.743327	0.948026	GRB2 associated binding protein 1 [Source:HGNC Symbol;Acc:HG	chr4:14425	NM_00203	XM_00526	.	.	NP_00203	XP_016863	protein_co	ENSG0000	2549	4066
GAB2	0.926692	1.06593	1.08709	0.479828	0.533043	0.549599	GRB2 associated binding protein 2 [Source:HGNC Symbol;Acc:HG	chr11:7792	NM_01229	XM_00671	.	.	NP_03642	XP_006718	protein_co	ENSG0000	9846	14458
GAB3	0.214826	0.211905	0.148399	0.075226	0.117984	0.115849	GRB2 associated binding protein 3 [Source:HGNC Symbol;Acc:HG	chrX:15390	NM_00108	XM_01153	NR_10411	.	NP_00107	XP_005274	protein_co	ENSG0000	139716	17515
GAB4	0	0	0	0	0	0	GRB2 associated binding protein family member 4 [Source:HGNC	chr22:1744	NM_00103	.	.	.	NP_00103	XP_016884	protein_co	ENSG0000	128954	18325
GABARAP	350.291	323.613	308.302	354.165	283.467	332.654	GABA type A receptor-associated protein [Source:HGNC Symbol;A	chr17:7143	NM_00727	.	.	.	NP_00920	.	protein_co	ENSG0000	11337	4067
GABARAPL1	32.9706	29.1948	32.6316	22.3319	22.52	36.4711	GABA type A receptor associated protein like 1 [Source:HGNC Syr	chr12:1036	NM_03141	XM_00525	.	.	NP_11360	XP_005253	protein_co	ENSG0000	23710	4068
GABARAPL2	85.3865	84.57	89.6113	83.7578	82.1946	81.2707	GABA type A receptor associated protein like 2 [Source:HGNC Syr	chr16:7560	NM_00728	.	.	.	NP_00921	.	protein_co	ENSG0000	11345	13291
GABBR1	6.2596	5.78636	7.31108	3.49115	4.04961	5.39808	gamma-aminobutyric acid type B receptor subunit 1 [Source:HGN	chr6:29523	NM_00147	XM_01151	.	.	NP_00130	XP_005249	protein_co	ENSG0000	2550	4070
GABBR2	0.01245	0.012669	0.086205	0.027324	0.014233	0.026286	gamma-aminobutyric acid type B receptor subunit 2 [Source:HGN	chr9:10105	NM_00545	.	.	.	NP_00544	.	protein_co	ENSG0000	9568	4507
GABPA	8.31264	8.59284	8.18834	7.07247	7.53549	9.02441	GA binding protein transcription factor alpha subunit [Source:HG	chr21:2701	NM_00119	XM_01702	.	.	NP_00118	XP_005260	protein_co	ENSG0000	2551	4071
GABPB1	11.0164	10.2191	10.9832	11.2165	10.1684	12.9981	GA binding protein transcription factor beta subunit 1 [Source:HG	chr15:5056	NM_00204	XM_01152	NR_02689	.	NP_00130	XP_005254	protein_co	ENSG0000	55056	4074
GABPB2	2.85193	2.78491	2.40899	2.21181	2.50521	3.47919	GA binding protein transcription factor beta subunit 2 [Source:HG	chr1:15104	NM_14461	XM_01150	.	.	NP_00131	XP_005244	protein_co	ENSG0000	126626	28441
GABRA1	0	0	0	0	0	0	gamma-aminobutyric acid type A receptor alpha1 subunit [Source	chr5:16127	NM_00080	.	.	.	NP_00079	.	protein_co	ENSG0000	2554	4075
GABRA2	0	0	0	0	0	0	gamma-aminobutyric acid type A receptor alpha2 subunit [Source	chr4:46250	NM_00080	XM_01151	.	.	NP_00079	XP_011511	protein_co	ENSG0000	2555	4076
GABRA3	0	0.02031	0.016851	0	0	0.019861	gamma-aminobutyric acid type A receptor alpha3 subunit [Source	chrX:15133	NM_00080	XM_01153	.	.	NP_00079	XP_006724	protein_co	ENSG0000	2556	4077
GABRA4	0	0	0	0	0	0	gamma-aminobutyric acid type A receptor alpha4 subunit [Source	chr4:46920	NM_00080	XM_01151	.	.	NP_00080	.	protein_co	ENSG0000	2557	4078
GABRA5	0	0	0	0	0	0	gamma-aminobutyric acid type A receptor alpha5 subunit [Source	chr15:2678	NM_00081	XM_00672	.	.	NP_00080	XP_005268	protein_co	ENSG0000	2558	4079
GABRA6	0	0	0	0	0	0	gamma-aminobutyric acid type A receptor alpha6 subunit [Source	chr5:16111	NM_00081	.	.	.	NP_00080	.	protein_co	ENSG0000	2559	4080
GABRB1	0	0	0	0	0	0	gamma-aminobutyric acid type A receptor beta1 subunit [Source	chr4:47033	NM_00081	.	.	.	NP_00080	.	protein_co	ENSG0000	2560	4081
GABRB2	0.011449	0	0	0	0	0	gamma-aminobutyric acid type A receptor beta2 subunit [Source	chr5:16071	NM_00081	.	.	.	NP_00080	.	protein_co	ENSG0000	2561	4082
GABRB3	0	0	0	0	0	0	gamma-aminobutyric acid type A receptor beta3 subunit [Source	chr15:2678	NM_00081	XM_01152	NR_10380	.	NP_00080	XP_011519	protein_co	ENSG0000	2562	4083
GABRD	8.44209	8.69802	7.3962	8.32604	7.46363	6.45941	gamma-aminobutyric acid type A receptor delta subunit [Source:	chr1:19507	NM_00081	.	.	.	NP_00080	XP_011539	protein_co	ENSG0000	2563	4084
GABRE	0.602126	0.369449	0.23574	0.577568	0.676224	0.789734	gamma-aminobutyric acid type A receptor epsilon subunit [Sourc	chrX:15112	NM_00496	XM_01153	NR_02963	.	NP_00495	.	protein_co	ENSG0000	574412	4085
GABRG1	0	0	0	0	0	0	gamma-aminobutyric acid type A receptor gamma1 subunit [Sou	chr4:46037	NM_17353	XM_01700	.	.	NP_77580	XP_016863	protein_co	ENSG0000	2565	4086
GABRG2	1.64485	1.21048	1.67741	1.37229	1.4488	1.94645	gamma-aminobutyric acid type A receptor gamma2 subunit [Sou	chr5:16149	NM_00081	.	.	.	NP_00080	.	protein_co	ENSG0000	2566	4087
GABRG3	0	0	0	0	0	0	gamma-aminobutyric acid type A receptor gamma3 subunit [Sou	chr15:2721	NM_00127	XM_01152	.	.	NP_00125	XP_011519	protein_co	ENSG0000	2567	4088
GABRP	0	0	0	0	0	0.024237	gamma-aminobutyric acid type A receptor pi subunit [Source:HG	chr5:17020	NM_00129	XM_01153	.	.	NP_00127	XP_011532	protein_co	ENSG0000	2568	4089
GABRQ	3.44795	3.94557	3.28148	4.50891	5.19987	4.29012	gamma-aminobutyric acid type A receptor theta subunit [Source:	chrX:15180	NM_01855	.	.	.	NP_06102	.	protein_co	ENSG0000	55879	14454
GABRR1	0	0	0	0	0	0	gamma-aminobutyric acid type A receptor rho1 subunit [Source:H	chr6:89887	NM_00125	XM_01153	.	.	NP_00124	XP_011534	protein_co	ENSG0000	2569	4090
GABRR2	0.126185	0.042913	0.145246	0.046013	0.09588	0.088677	gamma-aminobutyric acid type A receptor rho2 subunit [Source:H	chr6:89967	NM_00204	.	.	.	NP_00203	.	protein_co	ENSG0000	2570	4091
GABRR3	0	0	0	0	0	0	gamma-aminobutyric acid type A receptor rho3 subunit (gene/ps	chr3:97705	NM_00110	.	.	.	NP_00109	.	protein_co	ENSG0000	200959	17969
GAD1	5.65101	4.92098	4.7924	5.54496	5.41076	4.19731	glutamate decarboxylase 1 [Source:HGNC Symbol;Acc:HGNC:409	chr2:17167	NM_00081	XM_01700	.	.	NP_00080	XP_005246	protein_co	ENSG0000	2571	4092
GAD2	0	0	0.026184	0	0	0	glutamate decarboxylase 2 [Source:HGNC Symbol;Acc:HGNC:409	chr10:2650	NM_00081	.	.	.	NP_00080	.	protein_co	ENSG0000	2572	4093
GADD45A	57.5581	59.1254	47.2805	57.106	56.6152	98.1292	growth arrest and DNA damage inducible alpha [Source:HGNC Sy	chr1:68150	NM_00119	.	.	.	NP_00118	.	protein_co	ENSG0000	1647	4095
GADD45B	16.0617	15.7716	11.3063	12.9588	12.1584	11.9867	growth arrest and DNA damage inducible beta [Source:HGNC Syn	chr19:2476	NM_01567	.	.	.	NP_05649	XP_016882	protein_co	ENSG0000	4616	4096
GADD45G	0.760475	0.412759	0.388222	0.688224	0.814699	0.741164	growth arrest and DNA damage inducible gamma [Source:HGNC S	chr9:92219	NM_00670	.	.	.	NP_00669	.	protein_co	ENSG0000	10912	4097
GADD45GIP1	42.2437	40.711	34.8295	57.5869	37.812	30.4419	GADD45G interacting protein 1 [Source:HGNC Symbol;Acc:HGNC:	chr19:1305	NM_05285	.	.	.	NP_44308	.	protein_co	ENSG0000	90480	29996
GADL1	0	0	0	0.023868	0	0	glutamate decarboxylase like 1 [Source:HGNC Symbol;Acc:HGNC:	chr3:30767	NM_20735	.	.	.	NP_99724	XP_016861	protein_co	ENSG0000	339896	27949
GAGE1	0	0	0	0	0	0	G antigen 1 [Source:HGNC Symbol;Acc:HGNC:4098]	chrX:49354	NM_00104	.	.	.	NP_00103	XP_011542	protein_co	ENSG0000	100008586	4098
GAGE10	0	0	0	0	0	0	G antigen 10 [Source:HGNC Symbol;Acc:HGNC:30968]	chrX:49160	NM_00109	.	.	.	NP_00109	.	protein_co	ENSG0000	102724473	30968
GAGE12B	0	0	0	0	0	0	G antigen 12B [Source:HGNC Symbol;Acc:HGNC:26779]	chrX:49294	.	.	.	.	.	.	protein_co	ENSG0000	.	26779
GAGE12C	0	0	0	0	0	0	G antigen 12C [Source:HGNC Symbol;Acc:HGNC:28402]	chrX:49296	NM_00109	.	.	.	NP_00109	.	protein_co	ENSG0000	729428	28402
GAGE12D	0	0	0	0	0	0	G antigen 12D [Source:HGNC Symbol;Acc:HGNC:31904]	chrX:49306	NM_00112	.	.	.	NP_00112	.	protein_co	ENSG0000	100132399	31904
GAGE12E	0	0	0	0	0	0	G antigen 12E [Source:HGNC Symbol;Acc:HGNC:31905]	chrX:49315	NM_00109	.	.	.	NP_00109	.	protein_co	ENSG0000	729431	31905
GAGE12F	0	0	0	0	0	0	G antigen 12F [Source:HGNC Symbol;Acc:HGNC:31906]	chrX:49325	NM_00109	.	.	.	NP_00109	.	protein_co	ENSG0000	729408	31906
GAGE12G	0	0	0	0	0	0	G antigen 12G [Source:HGNC Symbol;Acc:HGNC:31907]	chrX:49335	NM_00109	.	.	.	NP_00109	.	protein_co	ENSG0000	645073	31907
GAGE12H	0	0	0	0	0	0	G antigen 12H [Source:HGNC Symbol;Acc:HGNC:31908]	chrX:49344	NM_00109	.	.	.	NP_00109	.	protein_co	ENSG0000	729442	31908
GAGE12I	0	0	0	0	0	0	G antigen 12I [Source:HGNC Symbol;Acc:4105]	chrX:49216	NM_02112	.	.	.	NP_06694	.	protein_co	ENSG0000	2576	4105
GAGE12J	0	0	0	0	0	0	G antigen 12J [Source:HGNC Symbol;Acc:HGNC:17778]	chrX:49178	NM_00109	.	.	.	NP_00109	.	protein_co	ENSG0000	729396	17778
GAGE13	0	0	0	0	0	0	G antigen 13 [Source:HGNC Symbol;Acc:HGNC:29081]	chrX:49188	NM_00109	.	.	.	NP_00109	.	protein_co	ENSG0000	645037	29081
GAGE2A	0	0	0	0	0	0	G antigen 2A [Source:HGNC Symbol;Acc:HGNC:4099]	chrX:49354	NM_00112	.	.	.	NP_00112	.	protein_co	ENSG0000	100101629	4099
GAGE2B	0	0	0	0	0	0	G antigen 2B [Source:HGNC Symbol;Acc:31957]	chrX:49235	NM_00109	.	.	.	NP_00109	.	protein_co	ENSG0000	2574	31957

GAGE2C	0	0	0	0	0	0	G antigen 2C [Source:HGNC Symbol;Acc:31958]	chrX:49226	NM_00147	.	.	.	NP_00146	.	protein_co	ENSG0000	2574	31958
GAGE2D	0	0	0	0	0	0	G antigen 2D [Source:HGNC Symbol;Acc:31959]	chrX:49207	NM_01219	.	.	.	NP_03632	.	protein_co	ENSG0000	729408	31959
GAGE2E	0	0	0	0	0	0	G antigen 2E [Source:HGNC Symbol;Acc:HGNC:31960]	chrX:49197	.	.	.	.	.	.	protein_co	ENSG0000	.	31960
GAK	24.8632	24.8602	27.5368	27.0146	26.2464	22.9191	cyclin G associated kinase [Source:HGNC Symbol;Acc:HGNC:4113	chr4:84306	NM_00128	XM_01151	.	.	NP_00130	XP_005272	protein_co	ENSG0000	2580	4113
GAL	102.256	102.966	72.5303	124.058	106.33	56.1927	galanin and GMAP prepropeptide [Source:HGNC Symbol;Acc:HGN	chr11:6845	NM_01597	.	.	.	NP_05705	.	protein_co	ENSG0000	51083	4114
GAL3ST1	0.479571	0.788977	0.821908	0.942743	0.926281	1.02134	galactose-3-O-sulfotransferase 1 [Source:HGNC Symbol;Acc:HGN	chr22:3095	NM_00131	XM_01702	.	.	NP_00130	XP_005261	protein_co	ENSG0000	9514	24240
GAL3ST2	0.087962	0.084152	0.074407	0	0	0	galactose-3-O-sulfotransferase 2 [Source:HGNC Symbol;Acc:HGN	chr2:24271	NM_02213	.	.	.	NP_07141	.	protein_co	ENSG0000	64090	24869
GAL3ST3	0	0	0	0	0	0	galactose-3-O-sulfotransferase 3 [Source:HGNC Symbol;Acc:HGN	chr11:6580	NM_03303	.	.	.	NP_14902	.	protein_co	ENSG0000	89792	24144
GAL3ST4	1.05936	1.34647	2.14198	2.51251	1.74697	2.79327	galactose-3-O-sulfotransferase 4 [Source:HGNC Symbol;Acc:HGN	chr7:99756	NM_02463	.	.	.	NP_07891	.	protein_co	ENSG0000	79690	24145
GALC	0	0	0	0	0	0	galactosylceramidase [Source:HGNC Symbol;Acc:HGNC:4115]	chr14:8839	NM_00015	XM_01153	.	.	NP_00014	XP_011534	protein_co	ENSG0000	2581	4115
GALE	46.594	45.2988	61.0903	42.4569	35.6588	44.7942	UDP-galactose-4-epimerase [Source:HGNC Symbol;Acc:HGNC:41	chr1:24122	NM_00040	.	.	.	NP_00039	.	protein_co	ENSG0000	2582	4116
GALK1	28.4065	27.7798	30.3806	32.2448	25.7619	26.6115	galactokinase 1 [Source:HGNC Symbol;Acc:HGNC:4118]	chr17:7371	NM_00015	.	.	.	NP_00014	.	protein_co	ENSG0000	2584	4118
GALK2	7.38075	7.65042	6.97585	8.64878	6.96381	8.23214	galactokinase 2 [Source:HGNC Symbol;Acc:HGNC:4119]	chr15:4944	NM_00100	XM_00672	.	.	NP_00100	XP_005254	protein_co	ENSG0000	2585	4119
GALM	7.67213	5.81147	7.49422	6.87191	6.63995	5.64473	galactose mutarotase [Source:HGNC Symbol;Acc:HGNC:24063]	chr2:38893	NM_13880	.	.	.	NP_62015	.	protein_co	ENSG0000	130589	24063
GALNS	11.0393	10.9379	11.8201	10.258	9.42887	8.84159	galactosamine (N-acetyl)-6-sulfatase [Source:HGNC Symbol;Acc:H	chr16:8888	NM_00051	XM_00525	.	.	NP_00050	XP_005256	protein_co	ENSG0000	2588	4122
GALNT1	24.2898	22.2913	21.8421	21.8199	25.2623	27.8813	polypeptide N-acetylgalactosaminyltransferase 1 [Source:HGNC S	chr18:3323	NM_02047	XM_01702	.	.	NP_06520	XP_005258	protein_co	ENSG0000	2589	4123
GALNT10	10.6214	10.7466	12.4962	13.602	13.612	18.2934	polypeptide N-acetylgalactosaminyltransferase 10 [Source:HGNC	chr5:15357	NM_19832	.	.	.	NP_93808	.	protein_co	ENSG0000	55568	19873
GALNT11	13.5225	13.1369	16.4615	13.4503	12.7234	13.8157	polypeptide N-acetylgalactosaminyltransferase 11 [Source:HGNC	chr7:15172	NM_00130	XM_00671	.	.	NP_00129	XP_006716	protein_co	ENSG0000	63917	19875
GALNT12	13.7447	13.7157	18.8194	15.2287	13.0167	15.1147	polypeptide N-acetylgalactosaminyltransferase 12 [Source:HGNC	chr9:10156	NM_02464	XM_01151	.	.	NP_07891	XP_006717	protein_co	ENSG0000	79695	19877
GALNT13	0.086595	0.098088	0.170542	0.017401	0.033455	0.055536	polypeptide N-acetylgalactosaminyltransferase 13 [Source:HGNC	chr2:15472	NM_00130	XM_01700	.	.	NP_00128	XP_011508	protein_co	ENSG0000	114805	23242
GALNT14	5.7331	5.51032	5.37363	8.44786	8.73808	6.7785	polypeptide N-acetylgalactosaminyltransferase 14 [Source:HGNC	chr2:31133	NM_00125	XM_01153	.	.	NP_00124	XP_011531	protein_co	ENSG0000	79623	22946
GALNT15	0	0	0	0	0	0	polypeptide N-acetylgalactosaminyltransferase 15 [Source:HGNC	chr3:16216	NM_05411	XM_00526	.	.	NP_00130	XP_005264	protein_co	ENSG0000	117248	21531
GALNT16	0	0.043146	0.012068	0	0.073665	0	polypeptide N-acetylgalactosaminyltransferase 16 [Source:HGNC	chr14:6972	NM_00116	XM_01153	.	.	NP_00116	XP_011535	protein_co	ENSG0000	57452	23233
GALNT18	11.589	14.5814	15.2257	16.9021	15.4883	12.9815	polypeptide N-acetylgalactosaminyltransferase 18 [Source:HGNC	chr11:1129	NM_19851	XM_01152	.	.	NP_94091	XP_006718	protein_co	ENSG0000	374378	30488
GALNT2	20.2565	20.9007	24.8961	20.9287	20.946	21.8084	polypeptide N-acetylgalactosaminyltransferase 2 [Source:HGNC S	chr1:23020	NM_00129	XM_01154	.	.	NP_00127	.	protein_co	ENSG0000	2590	4124
GALNT3	7.26539	8.21307	5.09384	10.8036	9.0018	12.6023	polypeptide N-acetylgalactosaminyltransferase 3 [Source:HGNC S	chr2:16660	NM_00448	XM_01151	.	.	NP_00447	XP_005246	protein_co	ENSG0000	2591	4125
GALNT4	0.92404	1.17288	1.36143	0.504419	0.742535	1.25469	polypeptide N-acetylgalactosaminyltransferase 4 [Source:HGNC S	chr12:8981	NM_00377	.	.	.	NP_00376	.	protein_co	ENSG0000	8693	4126
GALNT5	6.1814	6.94519	5.35773	3.77329	3.93458	2.53829	polypeptide N-acetylgalactosaminyltransferase 5 [Source:HGNC S	chr2:15811	NM_01456	.	.	.	NP_05538	XP_016858	protein_co	ENSG0000	11227	4127
GALNT6	5.26946	4.93051	5.64499	5.29895	4.9098	4.12191	polypeptide N-acetylgalactosaminyltransferase 6 [Source:HGNC S	chr12:5174	NM_00721	XM_01153	.	.	NP_00914	XP_005268	protein_co	ENSG0000	11226	4128
GALNT7	12.2723	14.4604	12.0197	10.931	13.2254	10.8828	polypeptide N-acetylgalactosaminyltransferase 7 [Source:HGNC S	chr4:17408	NM_01742	XM_01153	.	.	NP_05911	XP_005263	protein_co	ENSG0000	51809	4129
GALNT8	0	0	0	0	0	0.014604	polypeptide N-acetylgalactosaminyltransferase 8 [Source:HGNC S	chr12:4918	NM_00223	XM_00525	.	.	NP_00222	XP_005253	protein_co	ENSG0000	26290	4130
GALNT9	0.105127	0.269617	0.313766	0.065694	0.123097	0.185817	polypeptide N-acetylgalactosaminyltransferase 9 [Source:HGNC S	chr12:1326	NM_00112	.	.	.	NP_00111	.	protein_co	ENSG0000	50614	4131
GALNTL5	0	0	0	0	0	0	polypeptide N-acetylgalactosaminyltransferase-like 5 [Source:HG	chr7:15165	NM_14529	XM_01151	.	.	NP_66033	XP_006715	protein_co	ENSG0000	168391	21725
GALNTL6	0	0	0	0	0	0	polypeptide N-acetylgalactosaminyltransferase-like 6 [Source:HG	chr4:17273	NM_00103	XM_01153	.	.	NP_00103	XP_011530	protein_co	ENSG0000	442117	33844
GALP	0	0	0	0	0	0	galanin like peptide [Source:HGNC Symbol;Acc:HGNC:24840]	chr19:5668	NM_00114	.	.	.	NP_00113	.	protein_co	ENSG0000	85569	24840
GALR1	0	0	0	0	0	0	galanin receptor 1 [Source:HGNC Symbol;Acc:HGNC:4132]	chr18:7496	NM_00148	.	.	.	NP_00147	.	protein_co	ENSG0000	2587	4132
GALR2	0	0.112123	0.196187	0.496907	0.426476	0.273355	galanin receptor 2 [Source:HGNC Symbol;Acc:HGNC:4133]	chr17:7407	NM_00385	.	.	.	NP_00384	.	protein_co	ENSG0000	8811	4133
GALR3	0.498432	0.156807	0	0.173585	0.199235	0.191271	galanin receptor 3 [Source:HGNC Symbol;Acc:HGNC:4134]	chr22:3821	NM_00361	XM_01153	.	.	NP_00360	XP_011528	protein_co	ENSG0000	8484	4134
GALT	9.02284	10.3211	11.6883	8.17625	6.16992	6.56043	galactose-1-phosphate uridylyltransferase [Source:HGNC Symbol;	chr9:34646	NM_00015	.	.	.	NP_00014	.	protein_co	ENSG0000	2592	4135
GAMT	77.4414	68.5484	78.7698	80.9598	63.1597	50.0446	guanidinoacetate N-methyltransferase [Source:HGNC Symbol;Acc	chr19:1397	NM_00015	.	.	.	NP_00014	.	protein_co	ENSG0000	2593	4136
GAN	4.38896	4.86144	5.71144	4.2437	5.12914	5.75151	gigaxonin [Source:HGNC Symbol;Acc:HGNC:4137]	chr16:8134	NM_02204	.	NR_03987	.	NP_07132	.	protein_co	ENSG0000	10061615	4137
GANAB	203.208	212.442	200.299	196.98	195.476	220.89	glucosidase II alpha subunit [Source:HGNC Symbol;Acc:HGNC:413	chr11:6239	NM_00127	XM_01701	.	.	NP_00126	XP_016872	protein_co	ENSG0000	23193	4138
GANC	3.61147	4.19794	3.67054	3.93979	3.76316	4.05552	glucosidase alpha, neutral C [Source:HGNC Symbol;Acc:HGNC:41	chr15:4250	NM_00130	.	.	.	NP_00128	.	protein_co	ENSG0000	2595	4139
GAP43	0	0	0	0	0	0	growth associated protein 43 [Source:HGNC Symbol;Acc:HGNC:4	chr3:11534	NM_00113	.	.	.	NP_00112	XP_016861	protein_co	ENSG0000	2596	4140
GAPDH	4033.42	3871.55	3552.84	4214.98	3721.36	3621.22	glyceraldehyde-3-phosphate dehydrogenase [Source:HGNC Symb	chr12:6643	NM_00125	.	.	.	NP_00124	.	protein_co	ENSG0000	2597	4141
GAPDHS	0	0	0	0	0	0	glyceraldehyde-3-phosphate dehydrogenase, spermatogenic [Sou	chr19:3602	NM_01436	.	.	.	NP_05517	.	protein_co	ENSG0000	26330	24864
GAPT	0	0	0	0	0	0	GRB2-binding adaptor protein, transmembrane [Source:HGNC Sy	chr5:57787	NM_00130	.	.	.	NP_00129	.	protein_co	ENSG0000	202309	26588
GAPVD1	12.8833	13.2906	13.4745	12.0501	13.9387	14.6919	GTPase activating protein and VPS9 domains 1 [Source:HGNC Syn	chr9:12802	NM_00128	XM_01151	.	.	NP_00126	XP_005251	protein_co	ENSG0000	26130	23375
GAR1	23.9962	23.9073	23.3903	29.5111	37.7937	32.6466	GAR1 ribonucleoprotein [Source:HGNC Symbol;Acc:HGNC:14264]	chr4:11073	NM_01898	.	.	.	NP_06185	.	protein_co	ENSG0000	54433	14264
GAREM1	2.38212	2.58291	2.76209	2.77588	2.79103	2.73571	GRB2 associated regulator of MAPK1 subtype 1 [Source:HGNC Sy	chr18:2984	NM_00124	.	.	.	NP_00122	.	protein_co	ENSG0000	64762	26136
GAREM2	8.35007	8.00198	8.25152	7.85316	6.84393	6.45717	GRB2 associated regulator of MAPK1 subtype 2 [Source:HGNC Sy	chr2:26395	NM_00116	XM_01153	.	.	NP_00116	XP_006712	protein_co	ENSG0000	150946	27172
GARNL3	1.1986	1.09084	1.56731	1.07522	1.16452	1.0633	GTPase activating Rap/RanGAP domain like 3 [Source:HGNC Symb	chr9:13002	NM_00128	XM_01151	.	.	NP_00127	XP_005252	protein_co	ENSG0000	84253	25425
GARS	135.074	152.08	93.5503	98.2428	94.9574	289.224	glycyl-tRNA synthetase [Source:HGNC Symbol;Acc:HGNC:4162]	chr7:30634	NM_00131	XM_00671	.	.	NP_00130	XP_006715	protein_co	ENSG0000	2617	4162
GART	92.7055	95.2588	87.2025	97.6086	92.5596	111.196	phosphoribosylglycinamide formyltransferase, phosphoribosylgly	chr21:3487	NM_00081	XM_01152	.	.	NP_00081	XP_005260	protein_co	ENSG0000	2618	4163
GAS1	0.093592	0.046585	0.079442	0.051152	0.107575	0.148637	growth arrest specific 1 [Source:HGNC Symbol;Acc:HGNC:4165]	chr9:89559	NM_00204	.	.	.	NP_00203	.	protein_co	ENSG0000	2619	4165
GAS2	0.036423	0	0	0	0	0	growth arrest specific 2 [Source:HGNC Symbol;Acc:HGNC:4167]	chr11:2268	NM_00114	XM_01151	.	.	NP_00113	XP_011518	protein_co	ENSG0000	2620	4167
GAS2L1	17.2901	17.49	14.9738	17.3922	15.6472	12.1772	growth arrest specific 2 like 1 [Source:HGNC Symbol;Acc:HGNC:1	chr22:2970	NM_00127	XM_01702	.	.	NP_00126	XP_011528	protein_co	ENSG0000	10634	16955
GAS2L2	0	0	0.02081	0	0	0	growth arrest specific 2 like 2 [Source:HGNC Symbol;Acc:HGNC:2	chr17:3407	NM_13928	XM_00672	.	.	NP_64481	.	protein_co	ENSG0000	246176	24846
GAS2L3	4.51521	6.07686	5.79559	7.95768	9.66936	10.3635	growth arrest specific 2 like 3 [Source:HGNC Symbol;Acc:HGNC:2	chr12:1009	NM_00130	XM_01153	.	.	NP_00129	XP_005268	protein_co	ENSG0000	283431	27475

GAS6	44.9394	50.6648	46.0537	44.9683	35.3673	28.7764	growth arrest specific 6 [Source:HGNC Symbol;Acc:HGNC:4168]	chr13:1145	NM_00082	.	.	.	NP_00081	.	protein_co	ENSG0000	2621	4168
GAS7	0.84337	1.01292	1.55181	1.12668	1.51061	2.0358	growth arrest specific 7 [Source:HGNC Symbol;Acc:HGNC:4169]	chr17:9813	NM_00113	XM_01702	.	.	NP_00112	XP_00525	protein_co	ENSG0000	8522	4169
GAS8	11.9159	11.4131	9.60074	12.3986	9.61653	8.22818	growth arrest specific 8 [Source:HGNC Symbol;Acc:HGNC:4166]	chr16:9008	NM_00128	XM_01702	.	.	NP_00127	XP_00672	protein_co	ENSG0000	2622	4166
GAST	0.205601	0	0	2.4008	1.89876	0.675578	gastrin [Source:HGNC Symbol;Acc:HGNC:4164]	chr17:3986	NM_00080	.	.	.	NP_00079	.	protein_co	ENSG0000	2520	4164
GATA1	0	0	0	0.117787	0.085262	0	GATA binding protein 1 [Source:HGNC Symbol;Acc:HGNC:4170]	chrX:4864	NM_00204	XM_01154	.	.	NP_00204	XP_01154	protein_co	ENSG0000	2623	4170
GATA2	4.70243	4.57672	6.5667	7.99856	6.53572	4.56914	GATA binding protein 2 [Source:HGNC Symbol;Acc:HGNC:4171]	chr3:1281	NM_00114	.	.	.	NP_00113	.	protein_co	ENSG0000	2624	4171
GATA3	1.07998	0.793538	0.979824	1.08992	0.688471	0.738609	GATA binding protein 3 [Source:HGNC Symbol;Acc:HGNC:4172]	chr10:809	NM_00100	XM_00525	.	.	NP_00100	XP_00525	protein_co	ENSG0000	2625	4172
GATA4	0	0	0	0	0	0	GATA binding protein 4 [Source:HGNC Symbol;Acc:HGNC:4173]	chr8:1153	NM_00130	XM_01154	.	.	NP_00129	XP_00527	protein_co	ENSG0000	2626	4173
GATA5	0	0	0	0	0	0	GATA binding protein 5 [Source:HGNC Symbol;Acc:HGNC:15802]	chr20:6103	NM_08047	XM_00672	.	.	NP_53672	XP_00672	protein_co	ENSG0000	140628	15802
GATA6	4.48811	4.16485	3.97759	3.44201	4.02394	3.87499	GATA binding protein 6 [Source:HGNC Symbol;Acc:HGNC:4174]	chr18:1974	NM_00525	.	.	.	NP_00524	.	protein_co	ENSG0000	2627	4174
GATAD1	12.0551	11.5721	12.1809	10.0978	10.7951	12.6745	GATA zinc finger domain containing 1 [Source:HGNC Symbol;Acc:	chr7:9207	NM_02116	.	.	.	NP_06699	.	protein_co	ENSG0000	57798	29941
GATAD2A	24.9721	25.3238	25.5662	24.618	25.241	25.2126	GATA zinc finger domain containing 2A [Source:HGNC Symbol;Ac	chr19:1949	NM_00130	XM_01152	.	.	NP_00128	XP_00526	protein_co	ENSG0000	54815	29989
GATAD2B	12.5965	12.5351	12.051	10.4548	11.8944	12.7888	GATA zinc finger domain containing 2B [Source:HGNC Symbol;Ac	chr1:1537	NM_02069	XM_01150	.	.	NP_06575	XP_00524	protein_co	ENSG0000	57459	30778
GATB	13.9724	12.9006	13.2103	18.9766	14.0235	11.4406	glutamyl-tRNA amidotransferase subunit B [Source:HGNC Symbo	chr4:1525	NM_00456	XM_01153	.	.	NP_00455	XP_00526	protein_co	ENSG0000	5188	8849
GATC	16.3874	15.2932	16.3762	16.7596	14.8022	14.4468	glutamyl-tRNA amidotransferase subunit C [Source:HGNC Symbo	chr12:1208	NM_17681	.	.	.	NP_78978	.	protein_co	ENSG0000	283459	25068
GATM	0.981096	1.28414	0.995951	0.750165	1.02104	1.63131	glycine amidinotransferase [Source:HGNC Symbol;Acc:HGNC:417	chr15:4565	NM_00148	.	.	.	NP_00147	.	protein_co	ENSG0000	2628	4175
GATS	1.8821	1.97637	2.7425	1.46653	1.45555	1.63696	GATS, stromal antigen 3 opposite strand [Source:HGNC Symbol;A	chr7:9977	NM_17883	.	.	.	NP_84915	XP_01686	protein_co	ENSG0000	352954	29954
GATSL1	0	0	0	0	0	0	GATS protein-like 1 [Source:HGNC Symbol;Acc:37072]	chr7:74379	.	.	.	.	.	.	protein_co	ENSG0000	.	37072
GATSL2	0	0.202825	0.05828	0	0.159364	0.150573	GATS protein like 2 [Source:HGNC Symbol;Acc:HGNC:37073]	chr7:7480	NM_00114	.	.	.	NP_00113	.	protein_co	ENSG0000	729438	37073
GATSL3	2.43948	2.28003	3.7884	1.11063	0.922419	1.28353	GATS protein like 3 [Source:HGNC Symbol;Acc:HGNC:34423]	chr22:3068	NM_00103	.	.	.	NP_00103	.	protein_co	ENSG0000	652968	34423
GBA	18.4752	20.9002	13.6029	14.9847	15.2029	12.4435	glucosylceramidase beta [Source:HGNC Symbol;Acc:HGNC:4177]	chr1:1552	NM_00015	XM_01150	.	.	NP_00014	XP_00671	protein_co	ENSG0000	2629	4177
GBA2	18.616	19.443	22.333	17.5819	16.8528	19.3975	glucosylceramidase beta 2 [Source:HGNC Symbol;Acc:HGNC:1898	chr9:3569	NM_02094	XM_01701	.	.	NP_06599	XP_00525	protein_co	ENSG0000	57704	18986
GBAS	32.4467	32.3633	40.3494	30.5794	33.8261	43.4126	glioblastoma amplified sequence [Source:HGNC Symbol;Acc:HGN	chr7:5603	NM_00120	.	.	.	NP_00118	.	protein_co	ENSG0000	2631	4179
GBE1	14.8484	14.4927	12.7325	16.532	19.6263	14.878	glucan (1,4-alpha-), branching enzyme 1 [Source:HGNC Symbol;A	chr3:8153	NM_00015	.	.	.	NP_00014	.	protein_co	ENSG0000	2632	4180
GBF1	24.3691	25.2672	23.363	24.0036	21.4417	19.7409	golgi brefeldin A resistant guanine nucleotide exchange factor 1 [	chr10:104	NM_00119	XM_01154	.	.	NP_00118	XP_00527	protein_co	ENSG0000	8729	4181
GBGT1	0	0	0	0	0	0	globoside alpha-1,3-N-acetylgalactosaminyltransferase 1 [Source	chr9:1360	NM_00128	.	.	.	NP_00126	.	protein_co	ENSG0000	26301	20460
GBP1	0.038653	0.081312	0	0	0.043298	0	guanylate binding protein 1 [Source:HGNC Symbol;Acc:HGNC:418	chr1:8951	NM_00205	.	.	.	NP_00204	.	protein_co	ENSG0000	2633	4182
GBP2	0.406254	0.342647	0.379402	0.056399	0.057494	0.026136	guanylate binding protein 2 [Source:HGNC Symbol;Acc:HGNC:418	chr1:8957	NM_00412	.	.	.	NP_00411	.	protein_co	ENSG0000	2634	4183
GBP3	0.639858	0.402478	0.367052	0.225462	0.290144	0.259494	guanylate binding protein 3 [Source:HGNC Symbol;Acc:HGNC:418	chr1:8947	NM_01828	XM_01154	.	.	NP_06075	XP_00527	protein_co	ENSG0000	2635	4184
GBP4	0	0	0	0	0	0	guanylate binding protein 4 [Source:HGNC Symbol;Acc:HGNC:204	chr1:8964	NM_05294	.	.	.	NP_44317	.	protein_co	ENSG0000	115361	20480
GBP5	0.06193	0.064811	0.054167	0.069141	0.06986	0.094667	guanylate binding protein 5 [Source:HGNC Symbol;Acc:HGNC:198	chr1:8972	NM_00113	.	.	.	NP_00112	.	protein_co	ENSG0000	115362	19895
GBP6	0	0	0	0	0	0	guanylate binding protein family member 6 [Source:HGNC Symbo	chr1:8982	NM_19846	XM_01154	.	.	NP_94086	XP_01153	protein_co	ENSG0000	163351	25395
GBP7	0	0	0	0	0	0	guanylate binding protein 7 [Source:HGNC Symbol;Acc:HGNC:296	chr1:8959	NM_20739	.	.	.	NP_99728	.	protein_co	ENSG0000	388646	29606
GBX1	0.553799	0.537944	0.874651	0.294864	0.216444	0.309568	gastrulation brain homeobox 1 [Source:HGNC Symbol;Acc:HGNC:	chr7:1508	NM_00109	.	.	.	NP_00109	.	protein_co	ENSG0000	2636	4185
GBX2	1.89536	2.8049	2.6941	2.34575	2.14193	2.74296	gastrulation brain homeobox 2 [Source:HGNC Symbol;Acc:HGNC:	chr2:2370	NM_00130	.	.	.	NP_00128	.	protein_co	ENSG0000	2637	4186
GC	0	0	0	0	0	0	GC, vitamin D binding protein [Source:HGNC Symbol;Acc:HGNC:4	chr4:7260	NM_00058	XM_00671	.	.	NP_00057	XP_00671	protein_co	ENSG0000	2638	4187
GCA	12.4556	12.9367	14.7227	14.1881	14.8887	17.7177	grancalcin [Source:HGNC Symbol;Acc:HGNC:15990]	chr2:1631	NM_01219	XM_01151	.	.	NP_03633	XP_00671	protein_co	ENSG0000	25801	15990
GCAT	39.3814	37.9226	45.7228	42.0978	33.9232	24.3283	glycine C-acetyltransferase [Source:HGNC Symbol;Acc:HGNC:418	chr22:382	NM_00117	XM_01153	.	.	NP_00116	XP_00526	protein_co	ENSG0000	23464	4188
GCC1	14.2901	15.5108	11.2519	9.15588	10.5647	9.93565	GRIP and coiled-coil domain containing 1 [Source:HGNC Symbol;A	chr7:1272	NM_02452	.	.	.	NP_07879	.	protein_co	ENSG0000	79571	19095
GCC2	3.57597	3.61884	4.01992	1.5275	2.12888	2.90893	GRIP and coiled-coil domain containing 2 [Source:HGNC Symbol;A	chr2:1090	NM_18145	XM_00671	.	.	NP_85211	XP_00671	protein_co	ENSG0000	9648	23218
GCDH	8.33649	8.34626	9.77466	9.35094	7.40098	6.24486	glutaryl-CoA dehydrogenase [Source:HGNC Symbol;Acc:HGNC:41	chr19:130	NM_00015	XM_01152	.	.	NP_00015	XP_00672	protein_co	ENSG0000	2639	4189
GCFC2	5.99334	7.23746	6.95775	6.47374	6.45829	7.36127	GC-rich sequence DNA-binding factor 2 [Source:HGNC Symbol;Ac	chr2:7587	NM_00120	XM_01153	.	.	NP_00118	XP_00526	protein_co	ENSG0000	6936	1317
GCG	0	0	0	0	0	0	glucagon [Source:HGNC Symbol;Acc:HGNC:4191]	chr2:1629	NM_00205	.	.	.	NP_00204	.	protein_co	ENSG0000	2641	4191
GCGR	0	0.043944	0	0	0	0	glucagon receptor [Source:HGNC Symbol;Acc:HGNC:4192]	chr17:797	NM_00016	XM_01702	.	.	NP_00015	XP_00672	protein_co	ENSG0000	2642	4192
GCH1	12.793	12.8708	14.6427	16.0753	17.5228	21.193	GTP cyclohydrolase 1 [Source:HGNC Symbol;Acc:HGNC:4193]	chr14:553	NM_00016	XM_00526	.	.	NP_00015	XP_00526	protein_co	ENSG0000	2643	4193
GCHFR	1.65623	1.19412	4.9724	2.0772	1.89225	4.10156	GTP cyclohydrolase I feedback regulator [Source:HGNC Symbol;A	chr15:410	NM_00525	.	.	.	NP_00524	.	protein_co	ENSG0000	2644	4194
GCK	0.098011	0.07721	0.02832	0.069857	0.076227	0.036079	glucokinase [Source:HGNC Symbol;Acc:HGNC:4195]	chr7:4418	NM_00016	.	.	.	NP_00015	XP_01686	protein_co	ENSG0000	2645	4195
GCKR	0	0.029923	0	0	0.033759	0	glucokinase regulator [Source:HGNC Symbol;Acc:HGNC:4196]	chr2:2771	NM_00148	XM_01153	.	.	NP_00147	.	protein_co	ENSG0000	2646	4196
GCLC	24.3311	25.9394	19.8373	20.8498	21.2696	25.2008	glutamate-cysteine ligase catalytic subunit [Source:HGNC Symbol	chr6:5336	NM_00119	.	.	.	NP_00118	.	protein_co	ENSG0000	2729	4311
GCLM	12.0384	12.7657	8.43571	11.5259	12.6852	10.0656	glutamate-cysteine ligase modifier subunit [Source:HGNC Symbol	chr1:9435	NM_00206	XM_01154	.	.	NP_00129	XP_00527	protein_co	ENSG0000	2730	4312
GCM1	0.056893	0	0	0.031857	0	0.028871	glial cells missing homolog 1 [Source:HGNC Symbol;Acc:HGNC:41	chr6:5299	NM_00364	.	.	.	NP_00363	.	protein_co	ENSG0000	8521	4197
GCM2	0	0	0.051567	0	0.066357	0.030062	glial cells missing homolog 2 [Source:HGNC Symbol;Acc:HGNC:41	chr6:1087	NM_00475	XM_01151	.	.	NP_00474	XP_01151	protein_co	ENSG0000	9247	4198
GCN1	39.2913	41.2756	41.1917	41.6764	41.2522	32.1526	GCN1, eIF2 alpha kinase activator homolog [Source:HGNC Symbo	chr12:120	NM_00683	.	.	.	NP_00682	.	protein_co	ENSG0000	10985	4199
GCNT1	2.73307	3.17476	3.14598	3.02561	3.37218	3.45224	glucosaminyl (N-acetyl) transferase 1, core 2 [Source:HGNC Symb	chr9:7905	NM_00109	XM_01701	.	.	NP_00109	XP_00671	protein_co	ENSG0000	2650	4203
GCNT2	0.259591	0.322165	0.233408	0.152252	0.373245	0.351351	glucosaminyl (N-acetyl) transferase 2, I-branching enzyme (I bloo	chr6:1049	NM_00149	XM_01151	.	.	NP_00148	XP_00524	protein_co	ENSG0000	2651	4204
GCNT3	0.977311	1.78274	1.18327	1.07783	1.26543	1.17784	glucosaminyl (N-acetyl) transferase 3, mucin type [Source:HGNC	chr15:599	NM_00475	.	.	.	NP_00474	.	protein_co	ENSG0000	9245	4205
GCNT4	0.113379	0.088544	0.070383	0.112711	0.07053	0.092038	glucosaminyl (N-acetyl) transferase 4, core 2 [Source:HGNC Symb	chr5:7432	NM_01659	.	.	.	NP_05767	.	protein_co	ENSG0000	51301	17973
GCNT7	0	0	0	0	0	0	glucosaminyl (N-acetyl) transferase family member 7 [Source:HG	chr20:550	NM_08061	.	.	.	NP_54218	.	protein_co	ENSG0000	140687	16099
GCOM1	0.21001	1.5E-09	0.213734	0.29237	0.149488	0.098722	GRINL1A complex locus 1 [Source:HGNC Symbol;Acc:HGNC:2642	chr15:578	NM_00101	.	.	.	NP_00101	.	protein_co	ENSG0000	145781	26424

GCSAM	0	0	0.02085	0	0	0	germinal center associated signaling and motility [Source:HGNC S	chr3:11180	NM_00119	XM_01151	.	.	NP_00117	XP_005247	protein_co	ENSG0000	257144	20253
GCSAML	0	0	0	0	0	0.021172	germinal center associated signaling and motility like [Source:HG	chr1:24767	NM_00128	XM_01700	.	.	NP_00126	XP_011542	protein_co	ENSG0000	148823	29583
GCSH	49.7696	46.4191	54.0914	69.9266	48.6882	57.498	glycine cleavage system protein H [Source:HGNC Symbol;Acc:HGN	chr16:8111	NM_00448	.	.	.	NP_00447	XP_016878	protein_co	ENSG0000	2653	4208
GDA	20.9266	21.3087	16.7778	16.8953	20.3817	20.0583	guanine deaminase [Source:HGNC Symbol;Acc:HGNC:4212]	chr9:74729	NM_00124	XM_01701	.	.	NP_00122	XP_005252	protein_co	ENSG0000	9615	4212
GDAP1	10.5541	10.3646	10.2497	13.5169	15.0785	15.1408	ganglioside induced differentiation associated protein 1 [Source:H	chr8:75262	NM_00104	.	.	.	NP_00103	.	protein_co	ENSG0000	54332	15968
GDAP1L1	0	0	0	0	0	0.138719	ganglioside induced differentiation associated protein 1 like 1 [So	chr20:4287	NM_00125	.	.	.	NP_00124	.	protein_co	ENSG0000	78997	4213
GDAP2	3.91097	2.91895	3.01658	3.28331	4.24176	4.27427	ganglioside induced differentiation associated protein 2 [Source:H	chr1:11840	NM_00113	XM_01154	.	.	NP_00112	XP_005271	protein_co	ENSG0000	54834	18010
GDE1	25.2704	24.9865	25.1852	32.9671	30.1701	25.3347	glycerophosphodiester phosphodiesterase 1 [Source:HGNC Symb	chr16:1951	NM_00132	XM_00525	.	.	NP_00131	.	protein_co	ENSG0000	51573	29644
GDF1	4.57E-66	0.531781	0.370456	0.152943	0	0.088093	growth differentiation factor 1 [Source:HGNC Symbol;Acc:HGNC:	chr19:1897	NM_00149	.	.	.	NP_00148	.	protein_co	ENSG0000	2657	4214
GDF10	0	0	0	0	0	0	growth differentiation factor 10 [Source:HGNC Symbol;Acc:HGNC	chr10:4842	NM_00496	XM_00671	.	.	NP_00495	.	protein_co	ENSG0000	2662	4215
GDF11	1.54936	1.41906	1.43553	1.09707	1.07919	0.800692	growth differentiation factor 11 [Source:HGNC Symbol;Acc:HGNC	chr12:5613	NM_00581	XM_00671	.	.	NP_00580	XP_006719	protein_co	ENSG0000	10220	4216
GDF15	273.605	280.808	364.012	153.449	133.835	454.119	growth differentiation factor 15 [Source:HGNC Symbol;Acc:HGNC	chr19:1849	NM_00486	.	.	.	NP_00485	.	protein_co	ENSG0000	9518	30142
GDF2	0	0	0	0	0	0	growth differentiation factor 2 [Source:HGNC Symbol;Acc:HGNC:	chr10:4841	NM_01620	.	.	.	NP_05728	.	protein_co	ENSG0000	2658	4217
GDF3	0	0	0	0	0	0	growth differentiation factor 3 [Source:HGNC Symbol;Acc:HGNC:	chr12:7842	NM_02063	.	.	.	NP_06568	.	protein_co	ENSG0000	9573	4218
GDF5	0.063155	0.09393	0.054681	0.033931	0.073253	0.03473	growth differentiation factor 5 [Source:HGNC Symbol;Acc:HGNC:	chr20:3402	NM_00055	XM_01152	.	.	NP_00054	XP_011527	protein_co	ENSG0000	8200	4220
GDF5OS	0	0	0	0	0	0	growth differentiation factor 5 opposite strand [Source:HGNC Syr	chr20:3402	.	.	.	.	.	.	protein_co	ENSG0000	.	33435
GDF6	0	0	0	0	0	0	growth differentiation factor 6 [Source:HGNC Symbol;Acc:HGNC:	chr8:97154	NM_00100	.	.	.	NP_00100	.	protein_co	ENSG0000	392255	4221
GDF7	0	0	0	0	0	0	growth differentiation factor 7 [Source:HGNC Symbol;Acc:HGNC:	chr2:20866	NM_18282	.	.	.	NP_87824	.	protein_co	ENSG0000	151449	4222
GDF9	0.218891	0.567878	0.271087	0.353232	0.208382	0.492857	growth differentiation factor 9 [Source:HGNC Symbol;Acc:HGNC:	chr5:13219	NM_00128	XM_01154	.	.	NP_00127	XP_005272	protein_co	ENSG0000	2661	4224
GDI1	54.3626	52.9867	55.9001	64.0744	57.6477	60.4806	GDP dissociation inhibitor 1 [Source:HGNC Symbol;Acc:HGNC:422	chrX:15366	NM_00149	.	.	.	NP_00148	.	protein_co	ENSG0000	2664	4226
GDI2	85.8953	81.2248	80.4476	101.295	98.9394	97.957	GDP dissociation inhibitor 2 [Source:HGNC Symbol;Acc:HGNC:422	chr10:5807	NM_00111	.	.	.	NP_00110	XP_016871	protein_co	ENSG0000	2665	4227
GDNF	0	0	0	0	0	0	glial cell derived neurotrophic factor [Source:HGNC Symbol;Acc:H	chr5:37812	NM_00051	XM_01151	.	.	NP_00050	XP_011512	protein_co	ENSG0000	2668	4232
GDPD1	4.1887	5.46303	6.65579	5.55765	7.07457	9.56073	glycerophosphodiester phosphodiesterase domain containing 1 [	chr17:5729	NM_00116	.	.	.	NP_00115	.	protein_co	ENSG0000	284161	20883
GDPD2	0	0	0	0	0.041472	0.038637	glycerophosphodiester phosphodiesterase domain containing 2 [	chrX:69642	NM_00117	XM_01153	.	.	NP_00116	XP_011529	protein_co	ENSG0000	54857	25974
GDPD3	1.4915	0.628924	1.09171	1.23555	0.92784	1.31117	glycerophosphodiester phosphodiesterase domain containing 3 [	chr16:3011	NM_02430	.	.	.	NP_07728	.	protein_co	ENSG0000	79153	28638
GDPD4	0	0	0	0	0	0	glycerophosphodiester phosphodiesterase domain containing 4 [	chr11:7692	NM_18283	XM_01154	.	.	NP_87825	XP_011543	protein_co	ENSG0000	220032	24849
GDPD5	8.9079	8.93236	8.56777	8.60009	7.99185	7.98672	glycerophosphodiester phosphodiesterase domain containing 5 [	chr11:7514	NM_03079	XM_01154	.	.	NP_11041	XP_006718	protein_co	ENSG0000	81544	28804
GDPGP1	2.58258	2.95889	3.47605	3.40085	2.35063	2.92526	GDP-D-glucose phosphorylase 1 [Source:HGNC Symbol;Acc:HGNC	chr15:9077	NM_00101	XM_01152	.	.	NP_00101	XP_011519	protein_co	ENSG0000	390637	34360
GEM	5.78649	5.78646	4.94632	7.18133	7.40426	8.80648	GTP binding protein overexpressed in skeletal muscle [Source:HG	chr8:95261	NM_00526	.	.	.	NP_00525	.	protein_co	ENSG0000	2669	4234
GEMIN2	13.6213	14.179	12.7146	17.5827	14.754	17.4718	gem nuclear organelle associated protein 2 [Source:HGNC Symbo	chr14:3958	NM_00100	.	.	.	NP_00100	.	protein_co	ENSG0000	8487	10884
GEMIN4	32.8391	32.8632	29.6178	39.253	35.2405	23.8588	gem nuclear organelle associated protein 4 [Source:HGNC Symbo	chr17:6476	NM_01572	XM_01152	.	.	NP_05653	XP_005256	protein_co	ENSG0000	50628	15717
GEMIN5	17.4412	18.9412	18.4499	24.524	24.1623	15.7875	gem nuclear organelle associated protein 5 [Source:HGNC Symbo	chr5:15426	NM_00125	.	.	.	NP_00123	.	protein_co	ENSG0000	25929	20043
GEMIN6	20.3891	24.6217	21.1273	28.3691	26.5071	16.7589	gem nuclear organelle associated protein 6 [Source:HGNC Symbo	chr2:38978	NM_02477	.	.	.	NP_07905	.	protein_co	ENSG0000	79833	20044
GEMIN7	35.4096	34.6087	35.0507	41.4648	34.1107	32.702	gem nuclear organelle associated protein 7 [Source:HGNC Symbo	chr19:4558	NM_00100	XM_01152	.	.	NP_00100	XP_005259	protein_co	ENSG0000	79760	20045
GEMIN8	10.1358	8.32417	8.00606	8.15242	8.58619	8.32193	gem nuclear organelle associated protein 8 [Source:HGNC Symbo	chrX:14026	NM_00104	.	.	.	NP_00103	XP_016885	protein_co	ENSG0000	54960	26044
GEN1	5.24262	4.55523	4.71153	4.31349	7.42826	6.64046	GEN1, Holliday junction 5' flap endonuclease [Source:HGNC Symb	chr2:17845	NM_00113	XM_01153	.	.	NP_00112	XP_005262	protein_co	ENSG0000	348654	26881
GET4	33.0662	29.4338	29.5721	34.3967	30.3404	26.7613	golgi to ER traffic protein 4 [Source:HGNC Symbol;Acc:HGNC:216	chr7:91618	NM_01594	.	.	.	NP_05703	.	protein_co	ENSG0000	51608	21690
GFAP	0.021206	0.040506	0	0.219875	0.122566	0.139363	glial fibrillary acidic protein [Source:HGNC Symbol;Acc:HGNC:423	chr17:4298	NM_00113	.	.	.	NP_00112	XP_016879	protein_co	ENSG0000	2670	4235
GFER	35.0076	27.0169	29.4176	36.819	27.3196	20.9762	growth factor, augmenter of liver regeneration [Source:HGNC Syr	chr16:2034	NM_00526	.	.	.	NP_00525	.	protein_co	ENSG0000	2671	4236
GFI1	2.74895	2.57371	4.44887	2.22501	2.42123	3.40505	growth factor independent 1 transcriptional repressor [Source:H	chr1:92940	NM_00112	XM_01154	.	.	NP_00112	XP_005270	protein_co	ENSG0000	2672	4237
GFI1B	0	0	0	0.057167	0.061582	0	growth factor independent 1B transcriptional repressor [Source:H	chr9:13582	NM_00113	XM_01151	.	.	NP_00112	XP_006717	protein_co	ENSG0000	8328	4238
GFM1	15.8735	15.9165	17.4306	21.9822	23.6285	18.3427	G elongation factor mitochondrial 1 [Source:HGNC Symbol;Acc:H	chr3:15836	NM_00130	XM_01151	.	.	NP_00129	XP_006713	protein_co	ENSG0000	85476	13780
GFM2	18.8051	17.0937	18.4278	17.1438	18.8516	16.3044	G elongation factor mitochondrial 2 [Source:HGNC Symbol;Acc:H	chr5:73923	NM_00128	XM_01154	.	.	NP_00126	XP_011541	protein_co	ENSG0000	84340	29682
GFOD1	2.01838	1.73501	2.35167	1.41632	2.17631	2.84599	glucose-fructose oxidoreductase domain containing 1 [Source:HG	chr6:13358	NM_00124	XM_01151	NR_03845	.	NP_00122	XP_011513	protein_co	ENSG0000	54438	21096
GFOD2	12.5938	12.1902	12.4717	13.4358	13.8916	10.2681	glucose-fructose oxidoreductase domain containing 2 [Source:HG	chr16:6770	NM_00124	.	.	.	NP_00123	XP_016879	protein_co	ENSG0000	81577	28159
GFPT1	14.259	15.0538	11.8284	15.2473	17.0848	28.4333	glutamine--fructose-6-phosphate transaminase 1 [Source:HGNC S	chr2:69546	NM_00124	.	.	.	NP_00123	XP_016859	protein_co	ENSG0000	2673	4241
GFPT2	4.76249	5.94452	7.86147	5.64557	4.97727	7.09016	glutamine-fructose-6-phosphate transaminase 2 [Source:HGNC S	chr5:17972	NM_00511	XM_00671	.	.	NP_00510	XP_006715	protein_co	ENSG0000	9945	4242
GFRA1	0	0	0	0	0	0.037332	GDNF family receptor alpha 1 [Source:HGNC Symbol;Acc:HGNC:4	chr10:1178	NM_00114	XM_01153	.	.	NP_00113	XP_005269	protein_co	ENSG0000	2674	4243
GFRA2	0	0	0	0.076633	0.044909	0.159068	GDNF family receptor alpha 2 [Source:HGNC Symbol;Acc:HGNC:4	chr8:21547	NM_00116	XM_01154	.	.	NP_00115	XP_006716	protein_co	ENSG0000	2675	4244
GFRA3	0.088521	0.149313	0.037845	0	0.115231	0.048384	GDNF family receptor alpha 3 [Source:HGNC Symbol;Acc:HGNC:4	chr5:13758	NM_00149	.	.	.	NP_00148	.	protein_co	ENSG0000	2676	4245
GFRA4	0	0	0	0	0	0	GDNF family receptor alpha 4 [Source:HGNC Symbol;Acc:HGNC:1	chr20:3639	NM_02213	.	.	.	NP_07142	XP_005260	protein_co	ENSG0000	64096	13821
GFRAL	0	0	0.039338	0	0	0.045102	GDNF family receptor alpha like [Source:HGNC Symbol;Acc:HGNC	chr6:55192	NM_20741	.	.	.	NP_99729	.	protein_co	ENSG0000	389400	32789
GFY	4.00094	4.05615	3.86565	0.505009	0.263005	0.250487	golgi associated olfactory signaling regulator [Source:HGNC Symb	chr19:4992	NM_00119	XM_01152	.	.	NP_00118	XP_011524	protein_co	ENSG0000	100507003	44663
GGA1	20.181	19.7358	25.2198	19.0864	19.6609	21.6418	golgi associated, gamma adaptin ear containing, ARF binding prot	chr22:3800	NM_00100	XM_01702	.	.	NP_00100	XP_005261	protein_co	ENSG0000	26088	17842
GGA2	17.7836	18.6666	18.1181	18.8444	18.005	15.8343	golgi associated, gamma adaptin ear containing, ARF binding prot	chr16:2347	NM_01504	XM_01154	.	.	NP_05585	XP_016878	protein_co	ENSG0000	23062	16064
GGA3	16.2461	15.8209	16.7414	15.4959	15.7563	13.6874	golgi associated, gamma adaptin ear containing, ARF binding prot	chr17:7323	NM_00117	XM_01152	.	.	NP_00116	XP_011522	protein_co	ENSG0000	23163	17079
GGACT	1.29844	1.58123	1.59191	1.18868	1.72656	2.10958	gamma-glutamylamine cyclotransferase [Source:HGNC Symbol;A	chr13:1007	NM_00119	XM_01152	.	.	NP_00118	XP_005254	protein_co	ENSG0000	87769	25100
GGCT	55.6323	53.8444	58.6788	54.4369	60.0482	57.4318	gamma-glutamylcyclotransferase [Source:HGNC Symbol;Acc:HGN	chr7:30536	NM_00119	.	.	.	NP_00118	.	protein_co	ENSG0000	79017	21705
GGCX	7.04755	9.02243	8.21068	8.24712	6.66855	8.88134	gamma-glutamyl carboxylase [Source:HGNC Symbol;Acc:HGNC:4	chr2:85766	NM_00082	XM_01153	.	.	NP_00081	XP_005264	protein_co	ENSG0000	2677	4247

GGH	36.0928	34.7096	32.549	37.6595	39.8129	40.3648	gamma-glutamyl hydrolase [Source:HGNC Symbol;Acc:HGNC:424	chr8:63927	NM_00387	.	.	.	NP_00386	.	protein_co	ENSG0000(	8836	4248
GGN	0.172325	0.10897	0.145344	0	0	0.265444	gametogenetin [Source:HGNC Symbol;Acc:HGNC:18869]	chr19:3887	NM_15265	XM_01152	.	.	NP_68987(	XP_005258	protein_co	ENSG0000(	199720	18869
GGNBP2	29.6626	28.7904	29.5128	28.8752	32.2985	30.6301	gametogenetin binding protein 2 [Source:HGNC Symbol;Acc:HGN	chr17:3490	NM_02483	XM_01152	.	.	NP_07911	XP_005257	protein_co	ENSG0000(	79893	19357
GGPS1	16.9071	15.019	18.0795	15.3789	17.5748	24.1084	geranylgeranyl diphosphate synthase 1 [Source:HGNC Symbol;Ac	chr1:23533	NM_00103	.	.	.	NP_00103	.	protein_co	ENSG0000(	9453	4249
GGT1	8.31266	5.97344	6.12077	1.39556	0.965446	1.15587	gamma-glutamyltransferase 1 [Source:HGNC Symbol;Acc:HGNC:4	chr22:2497	NM_00128	XM_00526	.	.	NP_00127	XP_005261	protein_co	ENSG0000(	102724197	4250
GGT2	0	0	0	0	0	0	gamma-glutamyltransferase 2 [Source:HGNC Symbol;Acc:HGNC:4	chr22:2156	.	XM_01153	.	.	.	XP_011544	protein_co	ENSG0000(	102724197	4251
GGT5	0	0.068298	0.297816	0	0.080821	0.18612	gamma-glutamyltransferase 5 [Source:HGNC Symbol;Acc:HGNC:4	chr22:2461	NM_00109	XM_01702	.	.	NP_00109	XP_005261	protein_co	ENSG0000(	2687	4260
GGT6	0	0.054124	0.02715	0.033761	0.077631	0.073871	gamma-glutamyltransferase 6 [Source:HGNC Symbol;Acc:HGNC:2	chr17:4460	NM_00112	.	.	.	NP_00111	.	protein_co	ENSG0000(	124975	26891
GGT7	3.41574	2.83607	4.75209	4.04393	4.5966	5.55435	gamma-glutamyltransferase 7 [Source:HGNC Symbol;Acc:HGNC:4	chr20:3343	NM_17802	XM_01702	.	.	NP_82115	XP_016883	protein_co	ENSG0000(	2686	4259
GGTLC1	0	0	0	0	0	0	gamma-glutamyltransferase light chain 1 [Source:HGNC Symbol;A	chr20:2396	NM_17831	XM_01152	.	.	NP_84256	XP_005260	protein_co	ENSG0000(	92086	16437
GGTLC2	0	0	0.126011	0	0	0	gamma-glutamyltransferase light chain 2 [Source:HGNC Symbol;A	chr22:2298	NM_00128	XM_01153	.	.	NP_00126	XP_005261	protein_co	ENSG0000(	91227	18596
GGTLC3	0	0	0	0	0	0	gamma-glutamyltransferase light chain 3 [Source:HGNC Symbol;A	chr22:2036	.	.	.	.	.	.	protein_co	ENSG0000(	.	33426
GH1	0	0	0	0.137305	0	0	growth hormone 1 [Source:HGNC Symbol;Acc:HGNC:4261]	chr17:6198	NM_00051	XM_01152	.	.	NP_00050	.	protein_co	ENSG0000(	2688	4261
GH2	0.268563	0	0	0	0.167089	0	growth hormone 2 [Source:HGNC Symbol;Acc:HGNC:4262]	chr17:6194	NM_00205	.	.	.	NP_00205	.	protein_co	ENSG0000(	2689	4262
GHDC	5.86212	8.14218	6.20839	8.23101	7.48772	5.15266	GH3 domain containing [Source:HGNC Symbol;Acc:HGNC:24438]	chr17:4034	NM_00114	.	.	.	NP_00113	.	protein_co	ENSG0000(	84514	24438
GHITM	138.929	143.283	124.535	143.876	143.164	167.633	growth hormone inducible transmembrane protein [Source:HGN(	chr10:8589	NM_01439	.	.	.	NP_05520	.	protein_co	ENSG0000(	27069	17281
GHR	0	0.019697	0	0	0	0	growth hormone receptor [Source:HGNC Symbol;Acc:HGNC:4263	chr5:42423	NM_00016	XM_01151	.	.	NP_00015	.	protein_co	ENSG0000(	2690	4263
GHRH	0	0	0	0	0	0	growth hormone releasing hormone [Source:HGNC Symbol;Acc:H	chr20:3587	NM_00118	XM_01152	.	.	NP_00117	XP_011527	protein_co	ENSG0000(	2691	4265
GHRHR	0	0	0	0.112488	0	0.110092	growth hormone releasing hormone receptor [Source:HGNC Sym	chr7:31003	NM_00082	XM_01151	.	.	NP_00081	XP_011513	protein_co	ENSG0000(	2692	4266
GHRL	0	0.055309	0	0	0	0	ghrelin/obestatin prepropeptide [Source:HGNC Symbol;Acc:HGN(	chr3:10327	NM_00113	.	.	.	NP_00112	XP_016862	protein_co	ENSG0000(	51738	18129
GHSR	0	0	0	0	0	0	growth hormone secretagogue receptor [Source:HGNC Symbol;A	chr3:17216	NM_00412	.	.	.	NP_00411	.	protein_co	ENSG0000(	2693	4267
GID4	5.03502	4.60003	5.62042	4.18786	4.79325	5.39247	GID complex subunit 4 homolog [Source:HGNC Symbol;Acc:HGN(	chr17:1794	NM_02405	XM_00525	.	.	NP_07695	XP_005256	protein_co	ENSG0000(	79018	28453
GID8	23.7396	25.3453	23.1608	24.275	23.0688	22.6953	GID complex subunit 8 homolog [Source:HGNC Symbol;Acc:HGN(	chr20:6156	NM_01789	.	.	.	NP_06036	.	protein_co	ENSG0000(	54994	15857
GIF	0	0	0	0	0	0	gastric intrinsic factor [Source:HGNC Symbol;Acc:HGNC:4268]	chr11:5959	NM_00514	XM_01154	.	.	NP_00513	XP_011543	protein_co	ENSG0000(	2694	4268
GIGYF1	14.1777	13.8111	13.9695	11.1177	10.169	11.0983	GRB10 interacting GYF protein 1 [Source:HGNC Symbol;Acc:HGN(	chr7:10027	NM_02257	XM_01151	.	.	NP_07209	XP_005250	protein_co	ENSG0000(	64599	9126
GIGYF2	14.9443	15.8961	15.9177	13.2312	14.5168	13.9729	GRB10 interacting GYF protein 2 [Source:HGNC Symbol;Acc:HGN(	chr2:23356	NM_00110	.	NR_10349	.	NP_00109	.	protein_co	ENSG0000(	26058	11960
GIMAP1	0	0	0	0	0	0	GTPase, IMAP family member 1 [Source:HGNC Symbol;Acc:HGNC	chr7:15041	NM_13075	.	.	.	NP_57011	.	protein_co	ENSG0000(	170575	23237
GIMAP1-GIMAP5	0	0	0	0	0	0	GIMAP1-GIMAP5 readthrough [Source:HGNC Symbol;Acc:HGNC:5	chr7:15041	NM_00119	.	.	.	NP_00118	.	protein_co	ENSG0000(	100527949	51257
GIMAP2	0	0	0	0	0	0	GTPase, IMAP family member 2 [Source:HGNC Symbol;Acc:HGNC	chr7:15038	NM_01566	.	.	.	NP_05647	.	protein_co	ENSG0000(	26157	21789
GIMAP4	0.032692	0	0.035395	0	0	0	GTPase, IMAP family member 4 [Source:HGNC Symbol;Acc:HGNC	chr7:15026	NM_01832	XM_00525	.	.	NP_06079	XP_005250	protein_co	ENSG0000(	55303	21872
GIMAP5	0	0	0	0	0	0	GTPase, IMAP family member 5 [Source:HGNC Symbol;Acc:HGNC	chr7:15041	NM_01838	.	.	.	NP_06085	.	protein_co	ENSG0000(	55340	18005
GIMAP6	0	0	0	0	0	0	GTPase, IMAP family member 6 [Source:HGNC Symbol;Acc:HGNC	chr7:15032	NM_00124	.	.	.	NP_00123	.	protein_co	ENSG0000(	474344	21918
GIMAP7	0	0	0	0	0	0	GTPase, IMAP family member 7 [Source:HGNC Symbol;Acc:HGNC	chr7:15021	NM_15323	.	.	.	NP_69496	.	protein_co	ENSG0000(	168537	22404
GIMAP8	0	0	0	0	0	0	GTPase, IMAP family member 8 [Source:HGNC Symbol;Acc:HGNC	chr7:15014	NM_17557	XM_00524	.	.	NP_78316	XP_005250	protein_co	ENSG0000(	155038	21792
GIMD1	0	0	0	0	0	0	GIMAP family P-loop NTPase domain containing 1 [Source:HGNC	chr4:10727	NM_00119	.	.	.	NP_00118	XP_011529	protein_co	ENSG0000(	100507096	44141
GIN1	4.13468	4.1267	3.95581	3.11638	6.287	5.42524	gypsy retrotransposon integrase 1 [Source:HGNC Symbol;Acc:HG	chr5:10242	NM_00131	XM_00527	.	.	NP_00130	XP_005272	protein_co	ENSG0000(	54826	25959
GINM1	17.2505	15.3089	18.5541	13.1406	17.4996	24.4527	glycoprotein integral membrane 1 [Source:HGNC Symbol;Acc:HG	chr6:14988	NM_13878	.	.	.	NP_62014	.	protein_co	ENSG0000(	116254	21074
GINS1	17.7102	19.4934	17.6113	22.8545	22.3539	19.8253	GINS complex subunit 1 [Source:HGNC Symbol;Acc:HGNC:28980]	chr20:2538	NM_02106	XM_01702	.	.	NP_06654	XP_016883	protein_co	ENSG0000(	9837	28980
GINS2	32.655	32.2589	25.394	33.6285	28.7859	21.8485	GINS complex subunit 2 [Source:HGNC Symbol;Acc:HGNC:24575]	chr16:8564	NM_01609	.	.	.	NP_05717	.	protein_co	ENSG0000(	51659	24575
GINS3	16.7164	18.4036	12.5905	21.9614	22.3864	15.8068	GINS complex subunit 3 [Source:HGNC Symbol;Acc:HGNC:25851]	chr16:5842	NM_00112	.	.	.	NP_00111	.	protein_co	ENSG0000(	64785	25851
GINS4	12.3453	12.7643	11.1039	13.7791	14.3467	12.7534	GINS complex subunit 4 [Source:HGNC Symbol;Acc:HGNC:28226]	chr8:41386	NM_03233	XM_00527	.	.	NP_11571	XP_005273	protein_co	ENSG0000(	84296	28226
GIP	0	0	0	0.336821	0.359303	0.111437	gastric inhibitory polypeptide [Source:HGNC Symbol;Acc:HGNC:4	chr17:4703	NM_00412	.	.	.	NP_00411	.	protein_co	ENSG0000(	2695	4270
GIPC1	69.4044	68.1243	66.1493	79.8769	64.3394	61.489	GIPC PDZ domain containing family member 1 [Source:HGNC Sym	chr19:1458	NM_00571	XM_01702	.	.	NP_00570	XP_011525	protein_co	ENSG0000(	10755	1226
GIPC2	0.025591	0	0	0	0.028026	0	GIPC PDZ domain containing family member 2 [Source:HGNC Sym	chr1:78511	NM_01765	.	.	.	NP_06012	.	protein_co	ENSG0000(	54810	18177
GIPC3	0	0.0153	0	0	0	0.016363	GIPC PDZ domain containing family member 3 [Source:HGNC Sym	chr19:3585	NM_13326	XM_00525	.	.	NP_57356	XP_005259	protein_co	ENSG0000(	126326	18183
GIPR	1.62953	1.75301	3.09346	0.709095	0.728062	1.35663	gastric inhibitory polypeptide receptor [Source:HGNC Symbol;Acc	chr19:4617	NM_00016	XM_01152	.	.	NP_00015	XP_011525	protein_co	ENSG0000(	2696	4271
GIT1	36.1806	37.3693	32.2492	41.1365	37.1346	25.6901	GIT ArfGAP 1 [Source:HGNC Symbol;Acc:HGNC:4272]	chr17:2790	NM_00108	XM_01152	.	.	NP_00107	XP_011522	protein_co	ENSG0000(	28964	4272
GIT2	7.47267	9.04762	8.51262	7.09545	8.24647	7.53902	GIT ArfGAP 2 [Source:HGNC Symbol;Acc:HGNC:4273]	chr12:1103	NM_00113	XM_01702	.	.	NP_00112	XP_005254	protein_co	ENSG0000(	9815	4273
GJA1	0	0	0.024219	0	0.093306	0.1096	gap junction protein alpha 1 [Source:HGNC Symbol;Acc:HGNC:42	chr6:12175	NM_00016	.	.	.	NP_00015	.	protein_co	ENSG0000(	2697	4274
GJA10	0	0	0	0	0	0	gap junction protein alpha 10 [Source:HGNC Symbol;Acc:HGNC:1	chr6:90604	NM_03260	.	.	.	NP_11599	.	protein_co	ENSG0000(	84694	16995
GJA3	4.20718	4.07634	5.3555	3.672	4.28162	4.316	gap junction protein alpha 3 [Source:HGNC Symbol;Acc:HGNC:42	chr13:2071	NM_02195	XM_01153	.	.	NP_06877	XP_005266	protein_co	ENSG0000(	2700	4277
GJA4	0	0	0	0.056701	0	0	gap junction protein alpha 4 [Source:HGNC Symbol;Acc:HGNC:42	chr1:35258	NM_00206	XM_00527	.	.	NP_00205	XP_005270	protein_co	ENSG0000(	2701	4278
GJA5	0	0.023255	0.019555	0.04967	0	0.023444	gap junction protein alpha 5 [Source:HGNC Symbol;Acc:HGNC:42	chr1:14722	NM_00526	XM_01150	.	.	NP_00525	XP_005273	protein_co	ENSG0000(	2702	4279
GJA8	0	0	0	0	0	0	gap junction protein alpha 8 [Source:HGNC Symbol;Acc:HGNC:42	chr1:14738	NM_00526	XM_01150	.	.	NP_00525	XP_011507	protein_co	ENSG0000(	2703	4281
GJA9	0	0	0	0	0	0	gap junction protein alpha 9 [Source:HGNC Symbol;Acc:HGNC:19	chr1:39328	NM_03077	.	.	.	NP_11039	.	protein_co	ENSG0000(	81025	19155
GJB1	0	0	0	0	0	0	gap junction protein beta 1 [Source:HGNC Symbol;Acc:HGNC:428	chrX:70435	NM_00016	XM_01153	.	.	NP_00015	XP_011529	protein_co	ENSG0000(	2705	4283
GJB2	18.8352	19.1042	22.8245	18.5873	19.8071	21.7347	gap junction protein beta 2 [Source:HGNC Symbol;Acc:HGNC:428	chr13:2076	NM_00400	XM_01153	.	.	NP_00399	XP_011533	protein_co	ENSG0000(	2706	4284
GJB3	19.6746	21.3768	16.7466	18.8634	17.9883	14.5846	gap junction protein beta 3 [Source:HGNC Symbol;Acc:HGNC:428	chr1:35246	NM_00100	.	.	.	NP_00100	.	protein_co	ENSG0000(	2707	4285
GJB4	0.446486	0.797161	0.643868	0.47991	0.171618	0.215498	gap junction protein beta 4 [Source:HGNC Symbol;Acc:HGNC:428	chr1:35225	NM_15321	XM_01154	.	.	NP_69494	XP_011538	protein_co	ENSG0000(	127534	4286

GJB5	5.06404	5.74772	4.88357	7.40862	5.06779	3.80208	gap junction protein beta 5 [Source:HGNC Symbol;Acc:HGNC:428	chr1:3522(	NM_0052(	XM_00527	.	.	NP_00525	XP_00527(	protein_co	ENSG0000(	2709	4287
GJB6	0	0	0	0	0.101658	0.045547	gap junction protein beta 6 [Source:HGNC Symbol;Acc:HGNC:428	chr13:2079	NM_00111	XM_01702	.	.	NP_00110	XP_01687	protein_co	ENSG0000(	10804	4288
GJB7	0	0	0	0	0	0	gap junction protein beta 7 [Source:HGNC Symbol;Acc:HGNC:166	chr6:87992	NM_19856	XM_00524	.	.	NP_94097(	XP_00524	protein_co	ENSG0000(	375519	16690
GJC1	13.6228	13.6095	13.6188	15.3857	18.4418	16.9808	gap junction protein gamma 1 [Source:HGNC Symbol;Acc:HGNC:4	chr17:4287	NM_00108	XM_00525	.	.	NP_00107	XP_00525(	protein_co	ENSG0000(	10052	4280
GJC2	0.40057	0.571213	0.426038	0.471549	0.533562	0.400581	gap junction protein gamma 2 [Source:HGNC Symbol;Acc:HGNC:1	chr1:22833	NM_02043	.	.	.	NP_06516	.	protein_co	ENSG0000(	57165	17494
GJC3	0	0.146525	0.062189	0.314936	0.24143	0.148516	gap junction protein gamma 3 [Source:HGNC Symbol;Acc:HGNC:1	chr7:9952(	NM_18153	.	.	.	NP_85351	.	protein_co	ENSG0000(	349149	17495
GJD2	0	0	0	0	0	0	gap junction protein delta 2 [Source:HGNC Symbol;Acc:HGNC:19	chr15:3504	NM_02066	.	.	.	NP_06571	.	protein_co	ENSG0000(	57369	19154
GJD3	0	0	0	0	0	0	gap junction protein delta 3 [Source:HGNC Symbol;Acc:HGNC:19	chr17:3851	NM_15221	.	.	.	NP_68934	.	protein_co	ENSG0000(	125111	19147
GJD4	0	0.126556	0	0	0.075936	0	gap junction protein delta 4 [Source:HGNC Symbol;Acc:HGNC:23	chr10:3589	NM_15336	.	.	.	NP_69919	.	protein_co	ENSG0000(	219770	23296
GJE1	0	0	0	0	0	0	gap junction protein epsilon 1 [Source:HGNC Symbol;Acc:HGNC:3	chr6:14245	.		.	.	.	.	protein_co	ENSG0000(	.	33251
GK	1.56375	2.1912	2.09013	2.0032	2.12521	2.0077	glycerol kinase [Source:HGNC Symbol;Acc:HGNC:4289]	chrX:30671	NM_00016	XM_01154	.	.	NP_00015	XP_00527	protein_co	ENSG0000(	2710	4289
GK2	0	0	0	0	0	0	glycerol kinase 2 [Source:HGNC Symbol;Acc:HGNC:4291]	chr4:80327	NM_03321	.	.	.	NP_14999	.	protein_co	ENSG0000(	2712	4291
GK5	3.13435	3.42482	3.02276	2.71155	3.17507	3.55485	glycerol kinase 5 (putative) [Source:HGNC Symbol;Acc:HGNC:286	chr3:14187	NM_00103	XM_01700	.	.	NP_00103	XP_01151(	protein_co	ENSG0000(	256356	28635
GKAP1	2.43743	1.56162	1.61343	1.58054	2.15715	2.80391	G kinase anchoring protein 1 [Source:HGNC Symbol;Acc:HGNC:17	chr9:86354	NM_00113	XM_01151	.	.	NP_00112	XP_00525	protein_co	ENSG0000(	80318	17496
GKN1	0	0	0	0.09171	0	0	gastrokine 1 [Source:HGNC Symbol;Acc:HGNC:23217]	chr2:69201	NM_01961	.	.	.	NP_06256	.	protein_co	ENSG0000(	56287	23217
GKN2	0	0	0	0	0	0	gastrokine 2 [Source:HGNC Symbol;Acc:HGNC:24588]	chr2:69172	NM_18253	.	.	.	NP_87234	.	protein_co	ENSG0000(	200504	24588
GLA	31.9311	29.4268	23.8846	31.1676	30.1936	21.5978	galactosidase alpha [Source:HGNC Symbol;Acc:HGNC:4296]	chrX:10064	NM_00016	.	.	.	NP_00016	.	protein_co	ENSG0000(	2717	4296
GLB1	16.7151	16.875	16.9588	16.2505	12.5045	15.954	galactosidase beta 1 [Source:HGNC Symbol;Acc:HGNC:4298]	chr3:33038	NM_0004(	.	.	.	NP_00039	.	protein_co	ENSG0000(	2720	4298
GLB1L	2.29032	1.95723	2.53545	1.88603	1.26791	1.90691	galactosidase beta 1 like [Source:HGNC Symbol;Acc:HGNC:28129	chr2:22009	NM_00128	XM_01151	.	.	NP_00127	XP_01151(	protein_co	ENSG0000(	79411	28129
GLB1L2	17.4176	18.1635	17.8726	19.729	16.1965	14.9415	galactosidase beta 1 like 2 [Source:HGNC Symbol;Acc:HGNC:2512	chr11:1342	NM_13834	XM_00671	.	.	NP_61235	XP_00671	protein_co	ENSG0000(	89944	25129
GLB1L3	0	0	0	0	0	0.029238	galactosidase beta 1 like 3 [Source:HGNC Symbol;Acc:HGNC:2514	chr11:1341	NM_00108	XM_01154	.	.	NP_00107	XP_01154(	protein_co	ENSG0000(	112937	25147
GLCCI1	0.772073	0.684696	1.29463	0.618949	0.882133	1.35062	glucocorticoid induced 1 [Source:HGNC Symbol;Acc:HGNC:18713	chr7:80084	NM_13842	.	.	.	NP_61243	.	protein_co	ENSG0000(	113263	18713
GLCE	8.62329	9.04395	11.6914	8.27994	11.1002	13.5964	glucuronic acid epimerase [Source:HGNC Symbol;Acc:HGNC:1785	chr15:6945	NM_00132	XM_01702	.	.	NP_00131	XP_00525	protein_co	ENSG0000(	26035	17855
GLDC	2.12718	1.48972	2.62793	2.26892	1.66176	2.12014	glycine decarboxylase [Source:HGNC Symbol;Acc:HGNC:4313]	chr9:65324	NM_00017	.	.	.	NP_00016	.	protein_co	ENSG0000(	2731	4313
GLDN	0.517845	0.65919	0.900358	0.182413	0.176159	0.614645	gliomedin [Source:HGNC Symbol;Acc:HGNC:29514]	chr15:5163	NM_18178	XM_01702	.	.	NP_86145	XP_01151	protein_co	ENSG0000(	342035	29514
GLE1	23.0284	20.7179	21.0527	20.3929	21.0164	18.5209	GLE1, RNA export mediator [Source:HGNC Symbol;Acc:HGNC:431	chr9:13126	NM_0010(	XM_01151	.	.	NP_00100	XP_00671	protein_co	ENSG0000(	2733	4315
GLG1	67.2171	72.0826	78.4148	66.961	66.6204	86.5567	golgi glycoprotein 1 [Source:HGNC Symbol;Acc:HGNC:4316]	chr16:7448	NM_00114	.	.	.	NP_00113	.	protein_co	ENSG0000(	2734	4316
GLI1	0.870791	0.760525	1.08184	0.839834	0.929415	1.04105	GLI family zinc finger 1 [Source:HGNC Symbol;Acc:HGNC:4317]	chr12:5785	NM_00116	XM_01153	.	.	NP_00115	XP_00526	protein_co	ENSG0000(	2735	4317
GLI2	1.55368	1.44379	1.55526	1.3647	1.07296	0.835108	GLI family zinc finger 2 [Source:HGNC Symbol;Acc:HGNC:4318]	chr2:12154	NM_00527	XM_01151	.	.	NP_00526	XP_00671	protein_co	ENSG0000(	2736	4318
GLI3	0	0	0	0	0	0	GLI family zinc finger 3 [Source:HGNC Symbol;Acc:HGNC:4319]	chr7:4200(	NM_00016	XM_01151	.	.	NP_00015	XP_00524	protein_co	ENSG0000(	2737	4319
GLI4	21.9127	23.3211	25.9756	21.3231	18.9281	21.2826	GLI family zinc finger 4 [Source:HGNC Symbol;Acc:HGNC:4320]	chr8:14432	NM_13846	.	.	.	NP_61247	.	protein_co	ENSG0000(	2738	4320
GLIPR1	0.173155	0.086858	0.081543	0.937841	0.759343	0.35601	GLI pathogenesis related 1 [Source:HGNC Symbol;Acc:HGNC:170(	chr12:7587	NM_00685	XM_01153	.	.	NP_00684	XP_01153(	protein_co	ENSG0000(	11010	17001
GLIPR1L1	0	0.114806	0	0.122653	0	0	GLI pathogenesis related 1 like 1 [Source:HGNC Symbol;Acc:HGN(	chr12:7572	NM_0013(	.	.	.	NP_00129	.	protein_co	ENSG0000(	256710	28392
GLIPR1L2	0.457522	0.187812	0.225665	0.090683	0.428705	0.10918	GLI pathogenesis-related 1 like 2 [Source:HGNC Symbol;Acc:HGN	chr12:7578	NM_00127	XM_01153	.	.	NP_00125	XP_01153(	protein_co	ENSG0000(	144321	28592
GLIPR2	15.9148	15.278	18.3981	21.2407	18.5576	17.1516	GLI pathogenesis related 2 [Source:HGNC Symbol;Acc:HGNC:180(	chr9:3613(	NM_00128	.	.	.	NP_00127	.	protein_co	ENSG0000(	152007	18007
GLIS1	0.213916	0.136923	0.393372	0.074673	0.168127	0.328683	GLIS family zinc finger 1 [Source:HGNC Symbol;Acc:HGNC:29525]	chr1:53971	NM_14719	XM_01700	.	.	NP_67172	XP_01685	protein_co	ENSG0000(	148979	29525
GLIS2	5.46183	6.2618	6.27116	5.41021	5.1242	4.05999	GLIS family zinc finger 2 [Source:HGNC Symbol;Acc:HGNC:29450]	chr16:4364	NM_00131	XM_00525	.	.	NP_00130	XP_00525	protein_co	ENSG0000(	84662	29450
GLIS3	0.789276	1.07894	1.07407	0.14553	0.363869	0.331483	GLIS family zinc finger 3 [Source:HGNC Symbol;Acc:HGNC:28510]	chr9:38241	NM_00104	XM_01151	.	.	NP_00103	XP_00525	protein_co	ENSG0000(	169792	28510
GLMN	4.5379	3.47846	5.07064	4.23378	6.95244	7.17611	glomulin, FKBP associated protein [Source:HGNC Symbol;Acc:HG	chr1:92711	NM_05327	XM_01154	.	.	NP_00130	XP_00527(	protein_co	ENSG0000(	11146	14373
GLMP	21.0401	23.5408	21.2017	18.7218	16.8186	15.3245	glycosylated lysosomal membrane protein [Source:HGNC Symbol	chr1:15626	NM_00125	.	.	.	NP_00124	.	protein_co	ENSG0000(	112770	29436
GLO1	140.815	141.176	164.956	176.941	207.297	237.188	glyoxalase I [Source:HGNC Symbol;Acc:HGNC:4323]	chr6:38643	NM_0067(	.	.	.	NP_00669	.	protein_co	ENSG0000(	2739	4323
GLOD4	57.6948	58.1746	54.5089	69.2141	64.1903	53.0797	glyoxalase domain containing 4 [Source:HGNC Symbol;Acc:HGNC	chr17:6623	NM_01608	XM_00672	.	.	NP_05716	XP_00672	protein_co	ENSG0000(	51031	14111
GLOD5	0	0	0	0.106209	0	0.203695	glyoxalase domain containing 5 [Source:HGNC Symbol;Acc:HGNC	chrX:4862(	NM_00108	XM_01154	.	.	NP_00107	XP_01154	protein_co	ENSG0000(	392465	33358
GLP1R	0	0	0	0	0	0	glucagon like peptide 1 receptor [Source:HGNC Symbol;Acc:HGN(	chr6:39016	NM_00206	.	.	.	NP_00205	XP_01686	protein_co	ENSG0000(	2740	4324
GLP2R	0.016879	0	0	0.01871	0	0	glucagon like peptide 2 receptor [Source:HGNC Symbol;Acc:HGN(	chr17:9725	NM_00424	XM_01702	.	.	NP_00423	XP_00525(	protein_co	ENSG0000(	9340	4325
GLRA1	0	0	0	0	0	0	glycine receptor alpha 1 [Source:HGNC Symbol;Acc:HGNC:4326]	chr5:1512(	NM_00017	.	.	.	NP_00016	XP_01686	protein_co	ENSG0000(	2741	4326
GLRA2	0	0	0	0	0	0	glycine receptor alpha 2 [Source:HGNC Symbol;Acc:HGNC:4327]	chrX:14547	NM_00111	XM_01154	.	.	NP_00111	XP_00672	protein_co	ENSG0000(	2742	4327
GLRA3	0	0	0	0	0	0	glycine receptor alpha 3 [Source:HGNC Symbol;Acc:HGNC:4328]	chr4:17555	NM_00104	XM_01153	.	.	NP_00103	XP_01686	protein_co	ENSG0000(	8001	4328
GLRA4	0	0	0	0	0	0	glycine receptor alpha 4 [Source:HGNC Symbol;Acc:HGNC:31715]	chrX:10296	NM_00102	.	.	.	NP_00101	.	protein_co	ENSG0000(	441509	31715
GLRB	0	0	0	0	0	0	glycine receptor beta [Source:HGNC Symbol;Acc:HGNC:4329]	chr4:15799	NM_00082	XM_01153	.	.	NP_00081	XP_01686	protein_co	ENSG0000(	2743	4329
GLRX	30.5317	28.023	30.9042	29.326	23.3682	35.8136	glutaredoxin [Source:HGNC Symbol;Acc:HGNC:4330]	chr5:95066	NM_00111	.	.	.	NP_00111	.	protein_co	ENSG0000(	2745	4330
GLRX2	21.3072	18.3835	19.2206	23.3965	23.0365	27.3892	glutaredoxin 2 [Source:HGNC Symbol;Acc:HGNC:16065]	chr1:1930(	NM_00124	.	.	.	NP_00123	.	protein_co	ENSG0000(	51022	16065
GLRX3	43.6021	40.7922	41.5404	55.8599	45.6564	42.4415	glutaredoxin 3 [Source:HGNC Symbol;Acc:HGNC:15987]	chr10:1319	NM_00119	XM_01701	.	.	NP_00118	XP_01687(	protein_co	ENSG0000(	10539	15987
GLRX5	52.3757	52.6929	51.7033	82.4356	74.8713	64.6138	glutaredoxin 5 [Source:HGNC Symbol;Acc:HGNC:20134]	chr14:960(	NM_01641	.	.	.	NP_05750	.	protein_co	ENSG0000(	51218	20134
GLS	19.4382	23.3159	26.0197	24.3749	26.5288	32.5553	glutaminase [Source:HGNC Symbol;Acc:HGNC:4331]	chr2:19174	NM_00125	XM_00671	.	.	NP_00124	XP_00524	protein_co	ENSG0000(	2744	4331
GLS2	0.031505	0	0	0	0.036136	0	glutaminase 2 [Source:HGNC Symbol;Acc:HGNC:29570]	chr12:5686	NM_00128	XM_01701	.	.	NP_00126	XP_00526	protein_co	ENSG0000(	27165	29570
GLT1D1	0	0	0	0	0	0	glycosyltransferase 1 domain containing 1 [Source:HGNC Symbol	chr12:1293	NM_14466	XM_01153	.	.	NP_65327(	XP_00525	protein_co	ENSG0000(	144423	26483
GLT6D1	0	0.11423	0	0	0	0	glycosyltransferase 6 domain containing 1 [Source:HGNC Symbol	chr9:13851	NM_18297	XM_01151	.	.	NP_89201	XP_01151	protein_co	ENSG0000(	360203	23671
GLT8D1	23.7072	23.8052	23.8806	18.3493	22.0371	23.4764	glycosyltransferase 8 domain containing 1 [Source:HGNC Symbol	chr3:52719	NM_00101	.	.	.	NP_00101	XP_01686	protein_co	ENSG0000(	55830	24870

GLT8D2	3.74994	2.71959	2.51217	2.73562	2.81843	3.00759	glycosyltransferase 8 domain containing 2 [Source:HGNC Symbol;	chr12:1043	NM_03130	XM_01153	.	.	NP_00130	XP_005269	protein_co	ENSG0000	83468	24890
GLTP	19.1648	20.3388	19.9612	20.5408	19.7092	20.2749	glycolipid transfer protein [Source:HGNC Symbol;Acc:HGNC:2486	chr12:1102	NM_01643	XM_01153	.	.	NP_05751	XP_011536	protein_co	ENSG0000	51228	24867
GLTPD2	0	0	0	0	0	0	glycolipid transfer protein domain containing 2 [Source:HGNC Sy	chr17:4692	NM_00101	XM_00525	.	.	NP_00101	XP_005256	protein_co	ENSG0000	388323	33756
GLTSCR1	2.59331	3.12656	2.36451	2.66343	2.54294	2.96837	glioma tumor suppressor candidate region gene 1 [Source:HGNC	chr19:4811	NM_01571	XM_01152	.	.	NP_05652	XP_005258	protein_co	ENSG0000	29998	4332
GLTSCR1L	5.62994	5.82847	5.63106	4.27569	5.01068	6.23936	GLTSCR1 like [Source:HGNC Symbol;Acc:HGNC:21111]	chr6:42714	NM_00131	XM_00524	.	.	NP_00130	XP_005249	protein_co	ENSG0000	23506	21111
GLTSCR2	152.195	148.506	174.913	112.682	93.7881	138.428	glioma tumor suppressor candidate region gene 2 [Source:HGNC	chr19:4824	NM_01571	.	.	.	NP_05652	.	protein_co	ENSG0000	29997	4333
GLUD1	57.8394	59.4817	64.4895	66.8222	64.4825	79.9577	glutamate dehydrogenase 1 [Source:HGNC Symbol;Acc:HGNC:43	chr10:8881	NM_00527	XM_01153	.	.	NP_00526	.	protein_co	ENSG0000	2746	4335
GLUD2	7.18643	7.0726	8.45124	6.85471	9.04804	9.93724	glutamate dehydrogenase 2 [Source:HGNC Symbol;Acc:HGNC:43	chrX:12018	NM_01208	.	.	.	NP_03621	.	protein_co	ENSG0000	2747	4336
GLUL	55.2668	53.4613	57.3628	70.8176	62.2372	66.0345	glutamate-ammonia ligase [Source:HGNC Symbol;Acc:HGNC:4341	chr1:18235	NM_00103	XM_00671	.	.	NP_00102	XP_006711	protein_co	ENSG0000	2752	4341
GLYAT	0	0	0	0	0	0	glycine-N-acyltransferase [Source:HGNC Symbol;Acc:HGNC:13734	chr11:5847	NM_00583	.	.	.	NP_00582	.	protein_co	ENSG0000	10249	13734
GLYATL1	0.075064	0.078391	0.065893	0.047443	0.094132	0.076075	glycine-N-acyltransferase like 1 [Source:HGNC Symbol;Acc:HGNC:	chr11:5869	NM_00122	XM_01154	.	XR_132895	NP_00120	XP_011543	protein_co	ENSG0000	100287413	30519
GLYATL1P3	0	0	0	0	0	0	glycine-N-acyltransferase like 1 pseudogene 3 [Source:HGNC Sym	chr11:5885	.	.	.	.	.	.	protein_co	ENSG0000	.	37865
GLYATL2	0	0	0	0	0	0	glycine-N-acyltransferase like 2 [Source:HGNC Symbol;Acc:HGNC:	chr11:5860	NM_14501	XM_01701	.	.	NP_65945	XP_011543	protein_co	ENSG0000	219970	24178
GLYATL3	0	0	0	0	0	0	glycine-N-acyltransferase like 3 [Source:HGNC Symbol;Acc:HGNC:	chr6:49467	NM_00101	XM_01151	.	.	NP_00101	XP_011512	protein_co	ENSG0000	389396	21349
GLYCTK	3.39344	4.50302	2.27301	2.37929	1.39754	1.12712	glycerate kinase [Source:HGNC Symbol;Acc:HGNC:24247]	chr3:52321	NM_00114	XM_00526	.	.	NP_00113	XP_005264	protein_co	ENSG0000	132158	24247
GLYR1	19.6084	19.0437	17.6259	19.9314	19.5751	15.6347	glyoxylate reductase 1 homolog [Source:HGNC Symbol;Acc:HGNC	chr16:4853	NM_00130	XM_01702	.	.	NP_00129	XP_005255	protein_co	ENSG0000	84656	24434
GM2A	16.7492	15.8141	17.1144	14.4566	15.2946	20.3601	GM2 ganglioside activator [Source:HGNC Symbol;Acc:HGNC:4367	chr5:15063	NM_00040	.	.	.	NP_00039	.	protein_co	ENSG0000	2760	4367
GMCL1	6.44321	7.02395	7.57327	7.35593	7.64449	9.60015	germ cell-less, spermatogenesis associated 1 [Source:HGNC Symb	chr2:70056	NM_17843	XM_01153	.	.	NP_84852	XP_011531	protein_co	ENSG0000	64395	23843
GMDS	10.7939	10.3241	10.7196	12.4007	8.03745	11.8413	GDP-mannose 4,6-dehydratase [Source:HGNC Symbol;Acc:HGNC:	chr6:16240	NM_00125	XM_01151	.	.	NP_00124	XP_011512	protein_co	ENSG0000	2762	4369
GMEB1	5.16158	4.81381	4.89947	4.60487	5.13126	6.22276	glucocorticoid modulatory element binding protein 1 [Source:HG	chr1:28995	NM_00658	XM_01154	.	.	NP_00130	XP_011538	protein_co	ENSG0000	10691	4370
GMEB2	6.852	6.93429	5.93896	7.44157	6.88759	7.27802	glucocorticoid modulatory element binding protein 2 [Source:HG	chr20:6221	NM_01238	XM_01152	.	.	NP_03651	XP_005260	protein_co	ENSG0000	26205	4371
GMFB	18.4907	19.4116	20.0886	19.7309	22.5818	30.1826	glia maturation factor beta [Source:HGNC Symbol;Acc:HGNC:437	chr14:5494	NM_00412	XM_00526	.	.	NP_00411	.	protein_co	ENSG0000	2764	4373
GMFG	0.277249	0	0.342903	0.174895	0.427594	0	glia maturation factor gamma [Source:HGNC Symbol;Acc:HGNC:4	chr19:3981	NM_00130	.	.	.	NP_00128	.	protein_co	ENSG0000	9535	4374
GMIP	4.54878	4.60883	6.44563	5.32577	4.61859	5.31459	GEM interacting protein [Source:HGNC Symbol;Acc:HGNC:24852]	chr19:1974	NM_00128	XM_01152	.	.	NP_00127	XP_005259	protein_co	ENSG0000	51291	24852
GML	0	0	0	0	0	0	glycosylphosphatidylinositol anchored molecule like [Source:HGN	chr8:14391	NM_00206	XM_01151	.	.	NP_00205	XP_011515	protein_co	ENSG0000	2765	4375
GMNC	0.027045	0.14586	0.070214	0.246463	0.119289	0.184257	geminin coiled-coil domain containing [Source:HGNC Symbol;Acc	chr3:19057	NM_00114	.	.	.	NP_00114	.	protein_co	ENSG0000	647309	40049
GMNN	48.5761	43.8859	41.3548	64.4251	62.3297	45.0964	geminin, DNA replication inhibitor [Source:HGNC Symbol;Acc:HG	chr6:24775	NM_00125	XM_00524	.	.	NP_00123	XP_005249	protein_co	ENSG0000	51053	17493
GMPPA	43.879	44.72	40.9915	49.0953	38.247	30.6618	GDP-mannose pyrophosphorylase A [Source:HGNC Symbol;Acc:H	chr2:22036	NM_01333	XM_01700	.	.	NP_03746	XP_005246	protein_co	ENSG0000	29926	22923
GMPPB	17.3969	18.3319	13.9283	18.4336	17.2902	11.7368	GDP-mannose pyrophosphorylase B [Source:HGNC Symbol;Acc:H	chr3:49726	NM_01333	.	.	.	NP_03746	.	protein_co	ENSG0000	29925	22932
GMPR	10.5434	10.9833	13.2686	13.9133	12.0136	10.9361	guanosine monophosphate reductase [Source:HGNC Symbol;Acc:	chr6:16238	NM_00687	XM_01151	.	.	NP_00686	XP_011512	protein_co	ENSG0000	2766	4376
GMPR2	44.9824	45.2971	46.3846	41.578	37.9027	35.1839	guanosine monophosphate reductase 2 [Source:HGNC Symbol;A	chr14:2468	NM_00100	XM_00672	.	.	NP_00100	XP_005267	protein_co	ENSG0000	51292	4377
GMPS	43.0952	40.4041	42.5129	51.9524	48.9694	45.8687	guanine monophosphate synthase [Source:HGNC Symbol;Acc:HG	chr3:15558	NM_00387	XM_01700	.	.	NP_00386	XP_011511	protein_co	ENSG0000	8833	4378
GNA11	32.7419	33.1061	27.9999	38.6378	34.7453	31.6879	G protein subunit alpha 11 [Source:HGNC Symbol;Acc:HGNC:437	chr19:3094	NM_00206	.	.	.	NP_00205	.	protein_co	ENSG0000	2767	4379
GNA12	23.7515	23.1473	23.8505	23.9872	24.0332	21.035	G protein subunit alpha 12 [Source:HGNC Symbol;Acc:HGNC:438	chr7:27677	NM_00128	.	.	.	NP_00126	.	protein_co	ENSG0000	2768	4380
GNA13	21.2393	20.2292	21.3475	17.8526	26.3862	27.7487	G protein subunit alpha 13 [Source:HGNC Symbol;Acc:HGNC:438	chr17:6300	NM_00128	.	.	.	NP_00126	.	protein_co	ENSG0000	10672	4381
GNA14	0.033785	0.069863	0.087601	0.075254	0.076634	0.069012	G protein subunit alpha 14 [Source:HGNC Symbol;Acc:HGNC:438	chr9:80037	NM_00429	.	.	.	NP_00428	.	protein_co	ENSG0000	9630	4382
GNA15	0.107009	0.069695	0.462522	0.229248	0.417961	0.277832	G protein subunit alpha 15 [Source:HGNC Symbol;Acc:HGNC:438	chr19:3136	NM_00206	.	.	.	NP_00205	.	protein_co	ENSG0000	2769	4383
GNAI1	63.7699	61.3388	56.3707	73.2185	90.1389	69.2779	G protein subunit alpha i1 [Source:HGNC Symbol;Acc:HGNC:4384	chr7:79764	NM_00125	.	.	.	NP_00124	.	protein_co	ENSG0000	2770	4384
GNAI2	92.1176	91.8731	97.7892	105.66	90.6941	100.576	G protein subunit alpha i2 [Source:HGNC Symbol;Acc:HGNC:4385	chr3:50263	NM_00116	.	.	.	NP_00115	.	protein_co	ENSG0000	2771	4385
GNAI3	5.54621	5.52918	5.88321	5.96698	6.19021	6.89784	G protein subunit alpha i3 [Source:HGNC Symbol;Acc:HGNC:4387	chr1:11009	NM_00649	.	NR_02958	.	NP_00648	.	protein_co	ENSG0000	406974	4387
GNAL	1.77861	1.93106	2.78212	1.44436	1.86003	2.28937	G protein subunit alpha L [Source:HGNC Symbol;Acc:HGNC:4388]	chr18:1168	NM_00114	XM_01152	.	.	NP_00113	XP_006722	protein_co	ENSG0000	2774	4388
GNAO1	0	0	0	0	0	0	G protein subunit alpha o1 [Source:HGNC Symbol;Acc:HGNC:438	chr16:5622	NM_02098	.	.	.	NP_06626	.	protein_co	ENSG0000	2775	4389
GNAQ	8.2822	9.35596	9.26404	7.17267	10.2427	13.4301	G protein subunit alpha q [Source:HGNC Symbol;Acc:HGNC:4390]	chr9:80331	NM_00207	.	.	.	NP_00206	.	protein_co	ENSG0000	2776	4390
GNAS	308.27	311.17	300.088	341.176	305.896	336.149	GNAS complex locus [Source:HGNC Symbol;Acc:HGNC:4392]	chr20:5741	NM_00051	XM_01702	NR_13227	.	NP_00050	XP_016883	protein_co	ENSG0000	2778	4392
GNAT1	0.086189	0.083757	0.200332	0	0.067719	0	G protein subunit alpha transducin 1 [Source:HGNC Symbol;Acc:H	chr3:50229	NM_00017	.	.	.	NP_00016	.	protein_co	ENSG0000	2779	4393
GNAT2	0.182145	0.141566	0.159378	0.150888	0.361437	0.282761	G protein subunit alpha transducin 2 [Source:HGNC Symbol;Acc:H	chr1:11009	NM_00527	XM_01154	.	.	NP_00526	XP_011539	protein_co	ENSG0000	2780	4394
GNAT3	0	0	0	0	0	0	G protein subunit alpha transducin 3 [Source:HGNC Symbol;Acc:H	chr7:79998	NM_00110	XM_01151	.	.	NP_00109	XP_011514	protein_co	ENSG0000	346562	22800
GNAZ	5.98132	5.27284	6.73718	4.75888	4.95016	6.28809	G protein subunit alpha z [Source:HGNC Symbol;Acc:HGNC:4395]	chr22:2340	NM_00207	.	.	.	NP_00206	.	protein_co	ENSG0000	2781	4395
GNB1	145.462	148.009	135.464	148.818	154.314	170.204	G protein subunit beta 1 [Source:HGNC Symbol;Acc:HGNC:4396]	chr1:17167	NM_00128	XM_01700	.	.	NP_00126	XP_016856	protein_co	ENSG0000	2782	4396
GNB1L	7.95967	6.24118	7.19398	8.21693	6.75593	6.9568	G protein subunit beta 1 like [Source:HGNC Symbol;Acc:HGNC:43	chr22:1974	NM_05300	.	.	.	NP_44373	.	protein_co	ENSG0000	54584	4397
GNB2	152.693	153.101	140.018	189.116	159.137	130.601	G protein subunit beta 2 [Source:HGNC Symbol;Acc:HGNC:4398]	chr7:10027	NM_00527	.	.	.	NP_00526	.	protein_co	ENSG0000	2783	4398
GNB3	1.63094	1.73165	1.22762	1.41355	1.44218	1.32336	G protein subunit beta 3 [Source:HGNC Symbol;Acc:HGNC:4400]	chr12:6949	NM_00129	XM_01152	.	.	NP_00128	XP_011519	protein_co	ENSG0000	2784	4400
GNB4	0	0	0	0	0	0	G protein subunit beta 4 [Source:HGNC Symbol;Acc:HGNC:20731]	chr3:17911	NM_02162	XM_00671	.	.	NP_06764	XP_005247	protein_co	ENSG0000	59345	20731
GNB5	13.7573	14.6989	15.1102	17.6779	14.6195	14.1083	G protein subunit beta 5 [Source:HGNC Symbol;Acc:HGNC:4401]	chr15:5240	NM_00657	XM_01152	.	.	NP_00656	XP_011519	protein_co	ENSG0000	10681	4401
GNE	3.48928	3.94119	5.44421	4.2022	4.32151	4.56317	glucosamine (UDP-N-acetyl)-2-epimerase/N-acetylmannosamine	chr9:36214	NM_00112	XM_01701	.	.	NP_00112	XP_005251	protein_co	ENSG0000	10020	23657
GNG10	32.3824	32.8991	36.2189	26.18	24.9077	31.0691	G protein subunit gamma 10 [Source:HGNC Symbol;Acc:HGNC:44	chr9:11439	NM_00101	.	.	.	NP_00101	.	protein_co	ENSG0000	2790	4402
GNG11	0	0.066558	0	0	0	0.030483	G protein subunit gamma 11 [Source:HGNC Symbol;Acc:HGNC:44	chr7:93551	NM_00412	.	.	.	NP_00411	.	protein_co	ENSG0000	2791	4403
GNG12	34.3559	36.1311	36.1762	36.2003	41.7504	50.8142	G protein subunit gamma 12 [Source:HGNC Symbol;Acc:HGNC:19	chr1:68167	NM_01884	XM_01700	.	.	NP_06132	XP_016857	protein_co	ENSG0000	55970	19663
GNG13	0.192532	0.094807	1.56951	0.203227	0.112485	0.325933	G protein subunit gamma 13 [Source:HGNC Symbol;Acc:HGNC:14	chr16:8349	NM_01654	.	.	.	NP_05762	.	protein_co	ENSG0000	51764	14131

GNG2	0.521952	0.351644	1.20873	0.717467	1.03852	1.18479	G protein subunit gamma 2 [Source:HGNC Symbol;Acc:HGNC:440	chr14:5229	NM_00124	XM_01702	.	.	NP_00123	XP_006720	protein_co	ENSG0000	54331	4404
GNG3	0	0	0	0	0.122223	0	G protein subunit gamma 3 [Source:HGNC Symbol;Acc:HGNC:440	chr11:6245	NM_01220	XM_00671	.	.	NP_03633	XP_006718	protein_co	ENSG0000	2785	4405
GNG4	5.13759	6.81678	7.8076	8.76728	6.4935	8.38838	G protein subunit gamma 4 [Source:HGNC Symbol;Acc:HGNC:440	chr1:23571	NM_00109	XM_01154	.	.	NP_00109	XP_006711	protein_co	ENSG0000	2786	4407
GNG5	139.142	110.862	121.656	195.427	210.691	208.883	G protein subunit gamma 5 [Source:HGNC Symbol;Acc:HGNC:440	chr1:84964	NM_00527	.	.	.	NP_00526	.	protein_co	ENSG0000	2787	4408
GNG7	5.07621	4.98832	6.29828	6.3426	6.31753	7.24062	G protein subunit gamma 7 [Source:HGNC Symbol;Acc:HGNC:441	chr19:2511	NM_05284	XM_01702	.	.	NP_44307	XP_016882	protein_co	ENSG0000	2788	4410
GNG8	0	0	0	0	0	0	G protein subunit gamma 8 [Source:HGNC Symbol;Acc:HGNC:196	chr19:4713	NM_03325	.	.	.	NP_15028	XP_016882	protein_co	ENSG0000	94235	19664
GNGT1	0.323002	0.157452	0.607528	0.210397	0.338707	1.08785	G protein subunit gamma transducin 1 [Source:HGNC Symbol;Acc	chr7:93220	NM_00132	.	.	.	NP_00131	.	protein_co	ENSG0000	2792	4411
GNGT2	0	0	0.189868	0.165707	0	0.079576	G protein subunit gamma transducin 2 [Source:HGNC Symbol;Acc	chr17:4728	NM_00119	XM_00672	.	.	NP_00118	XP_006721	protein_co	ENSG0000	2793	4412
GNL1	15.0324	16.7202	17.1431	18.0467	16.2109	14.6833	G protein nucleolar 1 (putative) [Source:HGNC Symbol;Acc:HGNC	chr6:30509	NM_00527	XM_00524	.	.	NP_00526	XP_005249	protein_co	ENSG0000	2794	4413
GNL2	52.7591	47.7567	46.3817	52.2352	50.6025	40.0704	G protein nucleolar 2 [Source:HGNC Symbol;Acc:HGNC:29925]	chr1:38022	NM_01328	XM_01154	.	.	NP_03741	.	protein_co	ENSG0000	29889	29925
GNL3	143.311	139.946	128.408	167.396	156.078	119.934	G protein nucleolar 3 [Source:HGNC Symbol;Acc:HGNC:29931]	chr3:52719	NM_01436	.	.	.	NP_05518	.	protein_co	ENSG0000	26354	29931
GNL3L	11.1146	12.2538	12.2183	13.6213	13.1455	13.3801	G protein nucleolar 3 like [Source:HGNC Symbol;Acc:HGNC:25553	chrX:54556	NM_00118	XM_01153	.	.	NP_00117	XP_011529	protein_co	ENSG0000	54552	25553
GNLY	0	0	0	0	0	0.109334	granulysin [Source:HGNC Symbol;Acc:HGNC:4414]	chr2:85921	NM_00130	.	.	.	NP_00128	.	protein_co	ENSG0000	10578	4414
GNMT	0.140233	0.069839	0.301825	0	0	0.152707	glycine N-methyltransferase [Source:HGNC Symbol;Acc:HGNC:44	chr6:42928	NM_01896	.	.	.	NP_00130	.	protein_co	ENSG0000	27232	4415
GNPAT	24.1861	22.5209	33.8069	21.72	23.6321	26.0752	glyceronephosphate O-acyltransferase [Source:HGNC Symbol;Acc	chr1:23137	NM_01423	XM_01154	.	.	NP_05505	XP_005273	protein_co	ENSG0000	8443	4416
GNPDA1	45.3092	46.7943	52.7571	55.9479	53.1953	40.9338	glucosamine-6-phosphate deaminase 1 [Source:HGNC Symbol;Ac	chr5:14137	NM_00547	XM_01153	.	.	NP_00546	XP_005268	protein_co	ENSG0000	10007	4417
GNPDA2	8.2113	9.28815	10.0769	9.66398	9.68682	11.1043	glucosamine-6-phosphate deaminase 2 [Source:HGNC Symbol;Ac	chr4:44703	NM_00127	XM_01151	.	.	NP_00125	.	protein_co	ENSG0000	132789	21526
GNPNAT1	18.7153	19.6574	17.0592	19.1052	21.7652	21.3588	glucosamine-phosphate N-acetyltransferase 1 [Source:HGNC Sym	chr14:5324	NM_19806	XM_00672	.	.	NP_93233	XP_005268	protein_co	ENSG0000	64841	19980
GNPTAB	15.8053	15.767	17.1068	15.5355	18.6684	18.3585	N-acetylglucosamine-1-phosphate transferase alpha and beta sul	chr12:1021	NM_02431	XM_01701	.	.	NP_07728	XP_006719	protein_co	ENSG0000	79158	29670
GNPTG	18.6856	19.4411	18.9591	19.5423	15.3553	12.0726	N-acetylglucosamine-1-phosphate transferase gamma subunit [S	chr16:1401	NM_03252	.	.	.	NP_11590	XP_016879	protein_co	ENSG0000	84572	23026
GNRH1	1.06495	1.03338	1.24079	1.15546	2.26606	2.26622	gonadotropin releasing hormone 1 [Source:HGNC Symbol;Acc:HG	chr8:25276	NM_00082	.	.	.	NP_00081	.	protein_co	ENSG0000	2796	4419
GNRH2	0.108141	0.754226	0.093774	0.389582	0.984529	0.630397	gonadotropin releasing hormone 2 [Source:HGNC Symbol;Acc:HG	chr20:3024	NM_00131	.	.	.	NP_00129	XP_016883	protein_co	ENSG0000	2797	4420
GNRHR	0	0	0	0	0	0	gonadotropin releasing hormone receptor [Source:HGNC Symbol	chr4:68605	NM_00040	.	.	.	NP_00039	.	protein_co	ENSG0000	2798	4421
GNS	23.0507	23.4625	26.9893	19.4561	21.4335	24.8896	glucosamine (N-acetyl)-6-sulfatase [Source:HGNC Symbol;Acc:HG	chr12:6510	NM_00207	.	.	.	NP_00206	.	protein_co	ENSG0000	2799	4422
GOLGA1	5.95647	5.82155	5.001	5.44866	5.53223	4.89458	golgin A1 [Source:HGNC Symbol;Acc:HGNC:4424]	chr9:12764	NM_00207	XM_00671	.	.	NP_00206	XP_005251	protein_co	ENSG0000	2800	4424
GOLGA2	23.5703	25.5534	17.4545	17.2521	16.0252	20.1322	golgin A2 [Source:HGNC Symbol;Acc:HGNC:4425]	chr9:13101	NM_00448	XM_00525	.	.	NP_00447	XP_005251	protein_co	ENSG0000	2801	4425
GOLGA3	12.0101	12.1446	9.92353	9.55453	10.2496	8.70962	golgin A3 [Source:HGNC Symbol;Acc:HGNC:4426]	chr12:1333	NM_00589	XM_01153	.	.	NP_00588	XP_005266	protein_co	ENSG0000	2802	4426
GOLGA4	14.8013	14.617	16.3769	12.4755	18.0357	14.548	golgin A4 [Source:HGNC Symbol;Acc:HGNC:4427]	chr3:37284	NM_00117	XM_01700	.	.	NP_00116	XP_005265	protein_co	ENSG0000	2803	4427
GOLGA5	15.8068	15.69	13.898	12.4715	13.3487	14.7383	golgin A5 [Source:HGNC Symbol;Acc:HGNC:4428]	chr14:9326	NM_00511	XM_01153	.	.	NP_00510	XP_011535	protein_co	ENSG0000	9950	4428
GOLGA6A	0	0	0.01585	0	0	0.019237	golgin A6 family member A [Source:HGNC Symbol;Acc:HGNC:135	chr15:7436	NM_00103	XM_01152	.	.	NP_00103	.	protein_co	ENSG0000	342096	13567
GOLGA6B	0	0	0	0	0	0	golgin A6 family member B [Source:HGNC Symbol;Acc:HGNC:322	chr15:7294	NM_01865	XM_01152	.	.	NP_06112	.	protein_co	ENSG0000	55889	32205
GOLGA6C	0	0	0	0	0	0	golgin A6 family member C [Source:HGNC Symbol;Acc:HGNC:322	chr15:7555	NM_00116	.	.	.	NP_00115	.	protein_co	ENSG0000	653641	32206
GOLGA6D	0	0	0.020605	0	0.020671	0	golgin A6 family member D [Source:HGNC Symbol;Acc:HGNC:322	chr15:7555	NM_00114	.	.	.	NP_00113	.	protein_co	ENSG0000	653643	32204
GOLGA6L10	0	0	0.027944	0	0	0	golgin A6 family-like 10 [Source:HGNC Symbol;Acc:HGNC:37228]	chr15:8263	NM_00116	XM_00672	.	.	NP_00115	XP_006725	protein_co	ENSG0000	10013322	37228
GOLGA6L2	0	0	0	0	0	0	golgin A6 family-like 2 [Source:HGNC Symbol;Acc:HGNC:26695]	chr15:2368	NM_00130	.	.	.	NP_00129	.	protein_co	ENSG0000	283685	26695
GOLGA6L22	0	0	0	0	0	0	golgin A6 family-like 22 [Source:HGNC Symbol;Acc:HGNC:50289]	chr15:2273	NM_00127	XM_01154	.	.	.	XP_011542	protein_co	ENSG0000	10272362	50289
GOLGA6L4	0.218894	0.372221	0.299099	0.267919	0.201921	0.235367	golgin A6 family-like 4 [Source:HGNC Symbol;Acc:HGNC:27256]	chr15:8490	NM_00126	.	.	.	NP_00125	XP_016877	protein_co	ENSG0000	643707	27256
GOLGA6L6	0	0	0	0	0	0	golgin A6 family-like 6 [Source:HGNC Symbol;Acc:HGNC:37225]	chr15:2073	NM_00114	.	.	.	NP_00113	.	protein_co	ENSG0000	727832	37225
GOLGA6L7P	0	0	0	0	0.044696	0	golgin A6 family-like 7, pseudogene [Source:HGNC Symbol;Acc:H	chr15:2908	.	.	NR_04756	.	.	.	protein_co	ENSG0000	728310	37442
GOLGA6L9	0.418734	0.76398	0.540166	0.450652	0.448024	0.510869	golgin A6 family-like 9 [Source:HGNC Symbol;Acc:HGNC:37229]	chr15:8272	NM_19818	XM_01152	.	.	NP_93782	XP_011519	protein_co	ENSG0000	440295	37229
GOLGA7	27.0045	24.1619	24.9991	20.882	20.5526	27.4156	golgin A7 [Source:HGNC Symbol;Acc:HGNC:24876]	chr8:41347	NM_00100	.	.	.	NP_00100	.	protein_co	ENSG0000	51125	24876
GOLGA7B	0.850377	0.827011	0.566104	0.963983	0.754104	0.801288	golgin A7 family member B [Source:HGNC Symbol;Acc:HGNC:316	chr10:9960	NM_00101	.	.	.	NP_00101	.	protein_co	ENSG0000	401647	31668
GOLGA8A	5.03844	3.59687	4.96475	2.89461	4.50706	3.31945	golgin A8 family member A [Source:HGNC Symbol;Acc:HGNC:319	chr15:3467	NM_18107	XM_01702	.	.	NP_85142	XP_006720	protein_co	ENSG0000	23015	31972
GOLGA8B	5.43018	4.55706	6.99726	3.51417	4.24723	4.29498	golgin A8 family member B [Source:HGNC Symbol;Acc:HGNC:319	chr15:3481	NM_00102	XM_01152	NR_02740	.	NP_00101	XP_011519	protein_co	ENSG0000	440270	31973
GOLGA8F	0	0	0	0	0	0	golgin A8 family member F [Source:HGNC Symbol;Acc:HGNC:323	chr15:2862	.	.	.	.	.	.	protein_co	ENSG0000	.	32378
GOLGA8G	0	0	0	0	0	0	golgin A8 family member G [Source:HGNC Symbol;Acc:HGNC:253	chr15:2876	.	.	NR_03335	.	.	.	protein_co	ENSG0000	283768	25328
GOLGA8H	0.163696	0.249374	0.287807	0.298031	0.468912	0.348228	golgin A8 family member H [Source:HGNC Symbol;Acc:HGNC:374	chr15:3089	NM_00128	XM_01152	.	.	NP_00126	.	protein_co	ENSG0000	728498	37443
GOLGA8I	0.02742	0	0.011961	0.030879	0	0.02744	golgin A8 family, member I [Source:HGNC Symbol;Acc:26660]	chr15:2325	.	XM_00311	NR_02407	.	.	XP_005276	protein_co	ENSG0000	283796	26660
GOLGA8J	0	0	0	0	0	0	golgin A8 family member J [Source:HGNC Symbol;Acc:HGNC:3865	chr15:3037	NM_00128	XM_00672	.	.	NP_00126	XP_006720	protein_co	ENSG0000	653073	38650
GOLGA8K	0.303058	0.308136	0.048453	0.120519	0.220721	0.146289	golgin A8 family member K [Source:HGNC Symbol;Acc:HGNC:386	chr15:3268	NM_00128	.	.	.	NP_00126	.	protein_co	ENSG0000	653125	38652
GOLGA8M	0	0	0	0	0.031136	0	golgin A8 family member M [Source:HGNC Symbol;Acc:HGNC:444	chr15:2894	NM_00128	XM_01152	.	.	NP_00126	XP_005268	protein_co	ENSG0000	653720	44404
GOLGA8N	0.149676	0.042955	0.166097	0.137917	0.076119	0.149875	golgin A8 family member N [Source:HGNC Symbol;Acc:HGNC:444	chr15:3288	NM_00128	XM_01699	.	.	NP_00126	XP_016855	protein_co	ENSG0000	107984057	44405
GOLGA8O	0.078634	0.053215	0.161416	0.172043	0.030049	0.055809	golgin A8 family member O [Source:HGNC Symbol;Acc:HGNC:444	chr15:3273	NM_00127	XM_01152	.	.	NP_00126	XP_011520	protein_co	ENSG0000	728047	44406
GOLGA8Q	0	0	0	0	0	0	golgin A8 family member Q [Source:HGNC Symbol;Acc:HGNC:444	chr15:3084	.	.	.	.	.	.	protein_co	ENSG0000	728047	44408
GOLGA8R	0.054704	0.055581	0	0	0.031324	0.087285	golgin A8 family member R [Source:HGNC Symbol;Acc:HGNC:444	chr15:3069	NM_00128	.	.	.	NP_00126	XP_016855	protein_co	ENSG0000	107984057	44407
GOLGA8S	0	0	0	0	0	0	golgin A8 family member S [Source:HGNC Symbol;Acc:HGNC:444	chr15:2359	.	.	.	.	.	.	protein_co	ENSG0000	.	44409
GOLGA8T	0	0	0	0	0	0	golgin A8 family member T [Source:HGNC Symbol;Acc:HGNC:444	chr15:3042	.	.	.	.	.	.	protein_co	ENSG0000	.	44410
GOLGB1	10.1607	9.31976	9.84552	6.6219	7.6605	9.11244	golgin B1 [Source:HGNC Symbol;Acc:HGNC:4429]	chr3:12138	NM_00125	XM_01700	.	.	NP_00124	XP_005247	protein_co	ENSG0000	2804	4429
GOLIM4	4.85054	5.34132	4.41989	5.43168	5.68919	6.24179	golgi integral membrane protein 4 [Source:HGNC Symbol;Acc:HG	chr3:16772	NM_00130	XM_00524	.	.	NP_00129	XP_005247	protein_co	ENSG0000	27333	15448

GOLM1	45.849	46.7665	40.9008	45.6957	50.2313	41.9401	golgi membrane protein 1 [Source:HGNC Symbol;Acc:HGNC:1545	chr9:88641	NM_01654	.	.	.	NP_05763	.	protein_co	ENSG0000	51280	15451
GOLPH3	37.3429	44.3969	39.3574	36.8741	38.6815	42.9893	golgi phosphoprotein 3 [Source:HGNC Symbol;Acc:HGNC:15452]	chr5:32124	NM_02213	.	.	.	NP_07141	.	protein_co	ENSG0000	64083	15452
GOLPH3L	11.9643	11.9876	11.881	10.4383	12.5691	13.3178	golgi phosphoprotein 3 like [Source:HGNC Symbol;Acc:HGNC:248	chr1:15061	NM_01817	XM_00671	.	.	NP_06064	XP_006711	protein_co	ENSG0000	55204	24882
GOLT1A	6.14604	6.41695	8.20904	5.48527	4.2809	4.35005	golgi transport 1A [Source:HGNC Symbol;Acc:HGNC:24766]	chr1:20416	NM_19844	.	.	.	NP_940849	XP_016855	protein_co	ENSG0000	127845	24766
GOLT1B	29.3249	53.3008	30.3417	27.2831	32.9011	29.7379	golgi transport 1B [Source:HGNC Symbol;Acc:HGNC:20175]	chr12:2165	NM_01607	.	.	.	NP_05715	XP_016874	protein_co	ENSG0000	51026	20175
GON4L	7.13154	6.88442	7.29136	6.37301	6.97875	6.47508	gon-4 like [Source:HGNC Symbol;Acc:HGNC:25973]	chr1:15571	NM_00128	XM_01150	.	.	NP_00126	XP_005245	protein_co	ENSG0000	54856	25973
GOPC	12.4515	13.5709	13.4224	12.1101	16.1595	17.5679	golgi associated PDZ and coiled-coil motif containing [Source:HG	chr6:11760	NM_00101	.	.	.	NP_00101	.	protein_co	ENSG0000	57120	17643
GORAB	5.49076	4.55133	3.87375	3.8214	4.14025	4.4379	golgin, RAB6 interacting [Source:HGNC Symbol;Acc:HGNC:25676]	chr1:17050	NM_00114	XM_01151	.	.	NP_00113	XP_011508	protein_co	ENSG0000	92344	25676
GORASP1	14.0146	13.9548	15.977	13.7651	12.6523	11.4899	golgi reassembly stacking protein 1 [Source:HGNC Symbol;Acc:HG	chr3:39138	NM_00127	XM_01153	.	.	NP_00126	XP_006713	protein_co	ENSG0000	64689	16769
GORASP2	43.9011	43.9005	45.0902	42.2016	41.8235	40.2762	golgi reassembly stacking protein 2 [Source:HGNC Symbol;Acc:HG	chr2:17178	NM_00120	.	.	.	NP_00118	.	protein_co	ENSG0000	26003	17500
GOSR1	13.9706	17.683	12.9229	15.5668	16.6253	14.4632	golgi SNAP receptor complex member 1 [Source:HGNC Symbol;A	chr17:2880	NM_00100	XM_00525	.	.	NP_00100	XP_005258	protein_co	ENSG0000	9527	4430
GOSR2	33.8528	35.4063	34.2405	35.5822	28.2434	20.8195	golgi SNAP receptor complex member 2 [Source:HGNC Symbol;A	chr17:4500	NM_00101	XM_00525	.	.	NP_00101	XP_005257	protein_co	ENSG0000	9570	4431
GOT1	68.2921	68.4743	53.6508	78.1225	67.2832	133.388	glutamic-oxaloacetic transaminase 1 [Source:HGNC Symbol;Acc:H	chr10:1011	NM_00207	.	.	.	NP_00207	.	protein_co	ENSG0000	2805	4432
GOT1L1	0	0	0	0	0	0	glutamic-oxaloacetic transaminase 1-like 1 [Source:HGNC Symbo	chr8:37791	NM_15241	XM_00671	.	.	NP_68962	XP_005273	protein_co	ENSG0000	137362	28487
GOT2	82.5946	83.1192	86.6399	103.288	89.6906	80.7222	glutamic-oxaloacetic transaminase 2 [Source:HGNC Symbol;Acc:H	chr16:5874	NM_00128	.	.	.	NP_00127	.	protein_co	ENSG0000	2806	4433
GP1BA	0	0.031892	0	0	0.036084	0.033824	glycoprotein Ib platelet alpha subunit [Source:HGNC Symbol;Acc:	chr17:4883	NM_00017	.	.	.	NP_00016	.	protein_co	ENSG0000	2811	4439
GP1BB	3.88413	4.37239	3.15871	5.21362	3.60805	5.10486	glycoprotein Ib platelet beta subunit [Source:HGNC Symbol;Acc:H	chr22:1970	NM_00040	.	.	.	NP_00039	.	protein_co	ENSG0000	2812	4440
GP2	0	0	0	0	0	0	glycoprotein 2 [Source:HGNC Symbol;Acc:HGNC:4441]	chr16:2032	NM_00100	XM_01702	.	.	NP_00100	XP_016878	protein_co	ENSG0000	2813	4441
GP5	0.03175	0.032132	0.053956	0.034807	0.036088	0.033369	glycoprotein V platelet [Source:HGNC Symbol;Acc:HGNC:4443]	chr3:19411	NM_00448	.	.	.	NP_00447	.	protein_co	ENSG0000	2814	4443
GP6	0	0.039468	0.033603	0.042622	0	0	glycoprotein VI platelet [Source:HGNC Symbol;Acc:HGNC:14388]	chr19:5552	NM_00108	.	.	.	NP_00107	.	protein_co	ENSG0000	51206	14388
GP9	0	0	0	0	0	0	glycoprotein IX platelet [Source:HGNC Symbol;Acc:HGNC:4444]	chr3:12877	NM_00017	XM_01151	.	.	NP_00016	XP_005247	protein_co	ENSG0000	2815	4444
GPA33	0.174895	0.02533	0.173014	0.108616	0.057009	0.053267	glycoprotein A33 [Source:HGNC Symbol;Acc:HGNC:4445]	chr1:16702	NM_00581	.	.	.	NP_00580	.	protein_co	ENSG0000	10223	4445
GPAA1	64.4884	70.4634	63.0182	59.5289	52.3497	48.7643	glycosylphosphatidylinositol anchor attachment 1 [Source:HGNC	chr8:14513	NM_00380	.	.	.	NP_00379	.	protein_co	ENSG0000	8733	4446
GPALPP1	12.2489	10.538	9.0552	12.2008	12.4819	9.97096	GPALPP motifs containing 1 [Source:HGNC Symbol;Acc:HGNC:202	chr13:4556	NM_01855	XM_01153	.	.	NP_00130	XP_005266	protein_co	ENSG0000	55425	20298
GPAM	12.839	13.0101	10.8868	12.5695	16.3232	16.1821	glycerol-3-phosphate acyltransferase, mitochondrial [Source:HGN	chr10:1139	NM_00124	XM_00526	.	.	NP_00123	XP_005270	protein_co	ENSG0000	57678	24865
GPANK1	13.8218	12.6263	11.1555	12.1203	10.3389	10.3195	G-patch domain and ankyrin repeats 1 [Source:HGNC Symbol;Acc	chr6:31629	NM_00119	XM_01151	.	.	NP_00118	XP_005249	protein_co	ENSG0000	7918	13920
GPAT2	14.6423	17.1854	14.8747	20.5672	20.5925	13.2226	glycerol-3-phosphate acyltransferase 2, mitochondrial [Source:HG	chr2:96687	NM_00132	XM_01700	.	.	NP_00130	XP_005263	protein_co	ENSG0000	150763	27168
GPAT3	13.1093	13.4717	12.2911	11.3448	13.7166	18.3209	glycerol-3-phosphate acyltransferase 3 [Source:HGNC Symbol;Ac	chr4:84457	NM_00125	XM_01700	.	.	NP_00124	XP_011530	protein_co	ENSG0000	84803	28157
GPAT4	19.5578	20.8844	19.4863	18.383	19.451	17.2485	glycerol-3-phosphate acyltransferase 4 [Source:HGNC Symbol;Ac	chr8:41435	NM_17881	XM_01154	.	.	NP_84893	XP_011542	protein_co	ENSG0000	137964	20880
GPATCH1	3.75308	3.81364	3.42411	3.79941	3.49898	2.87503	G-patch domain containing 1 [Source:HGNC Symbol;Acc:HGNC:24	chr19:3357	NM_01802	.	.	.	NP_06049	.	protein_co	ENSG0000	55094	24658
GPATCH11	4.23172	4.87164	3.65363	3.8981	4.49558	4.05955	G-patch domain containing 11 [Source:HGNC Symbol;Acc:HGNC:2	chr2:37311	NM_00127	XM_01700	.	.	NP_00126	XP_016859	protein_co	ENSG0000	253635	26768
GPATCH2	5.05222	5.60211	4.33648	5.18204	5.11441	4.9501	G-patch domain containing 2 [Source:HGNC Symbol;Acc:HGNC:25	chr1:21760	NM_00129	XM_01150	.	.	NP_00128	XP_011507	protein_co	ENSG0000	55105	25499
GPATCH2L	11.5391	11.8699	13.876	13.273	12.8169	15.6244	G-patch domain containing 2 like [Source:HGNC Symbol;Acc:HGN	chr14:7661	NM_01792	XM_01702	.	.	NP_06039	XP_006720	protein_co	ENSG0000	55668	20210
GPATCH3	7.7781	7.63015	6.48783	9.1402	6.94452	4.99182	G-patch domain containing 3 [Source:HGNC Symbol;Acc:HGNC:25	chr1:27216	NM_02207	.	.	.	NP_07136	.	protein_co	ENSG0000	63906	25720
GPATCH4	27.914	27.1509	22.1622	26.3768	25.3434	17.4077	G-patch domain containing 4 [Source:HGNC Symbol;Acc:HGNC:25	chr1:15656	NM_01559	XM_00524	.	.	NP_05640	XP_005245	protein_co	ENSG0000	54865	25982
GPATCH8	8.21691	9.27167	8.54959	7.85899	8.38217	7.66532	G-patch domain containing 8 [Source:HGNC Symbol;Acc:HGNC:29	chr17:4247	NM_00100	.	.	.	NP_00100	XP_016879	protein_co	ENSG0000	23131	29066
GPBAR1	0.160282	0.084914	0.084044	0.305022	0.072338	0.099822	G protein-coupled bile acid receptor 1 [Source:HGNC Symbol;Acc	chr2:21912	NM_00107	XM_01151	.	.	NP_00107	XP_011509	protein_co	ENSG0000	151306	19680
GPBP1	32.7603	30.3705	29.9818	31.7883	36.327	38.0089	GC-rich promoter binding protein 1 [Source:HGNC Symbol;Acc:HG	chr5:56469	NM_00112	XM_00671	.	.	NP_00112	XP_005248	protein_co	ENSG0000	65056	29520
GPBP1L1	32.0097	31.5225	27.4791	29.2458	28.8592	32.4878	GC-rich promoter binding protein 1 like 1 [Source:HGNC Symbol;	chr1:46092	NM_02163	XM_00527	.	.	NP_06765	XP_005271	protein_co	ENSG0000	60313	28843
GPC1	42.1014	44.839	39.4119	56.5796	48.7291	40.2663	glypican 1 [Source:HGNC Symbol;Acc:HGNC:4449]	chr2:24137	NM_00208	XM_01151	.	.	NP_00207	XP_011509	protein_co	ENSG0000	2817	4449
GPC2	2.72066	2.41822	3.14095	3.12354	2.76319	3.45645	glypican 2 [Source:HGNC Symbol;Acc:HGNC:4450]	chr7:99767	NM_15274	.	.	.	NP_68995	.	protein_co	ENSG0000	221914	4450
GPC3	0.802788	0.708142	1.19726	0.699117	0.646029	0.674964	glypican 3 [Source:HGNC Symbol;Acc:HGNC:4451]	chrX:13266	NM_00116	.	.	.	NP_00115	.	protein_co	ENSG0000	2719	4451
GPC4	5.65638	6.28647	6.353	5.87604	5.45328	7.75393	glypican 4 [Source:HGNC Symbol;Acc:HGNC:4452]	chrX:13243	NM_00144	.	.	.	NP_00143	.	protein_co	ENSG0000	2239	4452
GPC5	0	0	0.02243	0.200497	0.104929	0.026087	glypican 5 [Source:HGNC Symbol;Acc:HGNC:4453]	chr13:9205	NM_00446	.	.	.	NP_00445	XP_016875	protein_co	ENSG0000	2262	4453
GPC6	0	0	0	0	0	0	glypican 6 [Source:HGNC Symbol;Acc:HGNC:4454]	chr13:9387	NM_00570	XM_01152	.	.	NP_00569	XP_011519	protein_co	ENSG0000	10082	4454
GPCPD1	8.71571	8.24201	7.561	6.4556	7.10053	9.71394	glycerophosphocholine phosphodiesterase 1 [Source:HGNC Symb	chr20:5525	NM_01959	XM_00672	.	.	NP_06253	XP_005260	protein_co	ENSG0000	56261	26957
GPD1	0.742459	0.694074	1.29499	1.66486	1.31387	1.15094	glycerol-3-phosphate dehydrogenase 1 [Source:HGNC Symbol;Ac	chr12:5049	NM_00125	.	.	.	NP_00124	.	protein_co	ENSG0000	2819	4455
GPD1L	9.79797	11.0351	13.6271	8.81265	10.3893	10.5493	glycerol-3-phosphate dehydrogenase 1-like [Source:HGNC Symbo	chr3:32148	NM_01514	XM_00671	.	.	NP_05595	XP_006713	protein_co	ENSG0000	23171	28956
GPD2	27.8961	29.4218	27.4128	36.4342	44.6006	38.4008	glycerol-3-phosphate dehydrogenase 2 [Source:HGNC Symbol;Ac	chr2:15729	NM_00040	XM_01151	.	.	NP_00039	XP_005246	protein_co	ENSG0000	2820	4456
GPER1	0.729482	0.58182	0.596805	1.12472	1.36421	1.03619	G protein-coupled estrogen receptor 1 [Source:HGNC Symbol;Acc	chr7:10366	NM_00103	.	.	.	NP_00103	.	protein_co	ENSG0000	2852	4485
GPHA2	0.129619	0	0.147808	0	0	0	glycoprotein hormone alpha 2 [Source:HGNC Symbol;Acc:HGNC:1	chr11:6469	NM_13076	XM_01154	.	.	NP_57012	XP_011543	protein_co	ENSG0000	170589	18054
GPHB5	0	0	0	0	0	0	glycoprotein hormone beta 5 [Source:HGNC Symbol;Acc:HGNC:18	chr14:6377	NM_14517	.	.	.	NP_66015	.	protein_co	ENSG0000	122876	18055
GPHN	7.88867	7.55778	7.59147	8.05006	7.41771	7.90281	gephyrin [Source:HGNC Symbol;Acc:HGNC:15465]	chr14:6697	NM_00102	XM_01153	.	.	NP_00101	XP_005267	protein_co	ENSG0000	10243	15465
GPI	193.178	205.475	212.092	230.692	200.004	179.238	glucose-6-phosphate isomerase [Source:HGNC Symbol;Acc:HGNC	chr19:3485	NM_00017	XM_01152	.	.	NP_00016	XP_005258	protein_co	ENSG0000	2821	4458
GPIHBP1	0.032044	0.062905	0.055528	0	0.037428	0	glycosylphosphatidylinositol anchored high density lipoprotein bi	chr8:14429	NM_00130	.	.	.	NP_00128	.	protein_co	ENSG0000	338328	24945
GPKOW	16.5231	15.4236	13.1692	16.1969	14.1019	15.7674	G-patch domain and KOW motifs [Source:HGNC Symbol;Acc:HGN	chrX:48970	NM_01569	XM_01702	.	.	NP_05651	XP_016884	protein_co	ENSG0000	27238	30677
GPLD1	0.613159	0.824945	1.11558	0.568164	0.669482	0.815587	glycosylphosphatidylinositol specific phospholipase D1 [Source:H	chr6:24428	NM_00150	XM_00524	.	.	NP_00149	XP_016866	protein_co	ENSG0000	2822	4459
GPM6A	1.20162	1.27654	2.50217	0.977843	0.839887	1.8441	glycoprotein M6A [Source:HGNC Symbol;Acc:HGNC:4460]	chr4:17655	NM_00126	XM_01153	.	.	NP_00124	XP_006714	protein_co	ENSG0000	2823	4460
GPM6B	0	0	0	0	0.026975	0	glycoprotein M6B [Source:HGNC Symbol;Acc:HGNC:4461]	chrX:13789	NM_00100	XM_01154	.	.	NP_00100	XP_005274	protein_co	ENSG0000	2824	4461

GPN1	25.0698	25.3646	21.1442	28.7869	27.7022	21.4939	GPN-loop GTPase 1 [Source:HGNC Symbol;Acc:HGNC:17030]	chr2:27848	NM_00114	.	.	.	NP_00113	.	protein_co	ENSG0000	11321	17030
GPN2	11.5023	12.381	12.117	10.9955	10.6995	8.6877	GPN-loop GTPase 2 [Source:HGNC Symbol;Acc:HGNC:25513]	chr1:27202	NM_01806	.	.	.	NP_06053	.	protein_co	ENSG0000	54707	25513
GPN3	22.1889	19.5137	21.4904	24.9995	26.8099	23.34	GPN-loop GTPase 3 [Source:HGNC Symbol;Acc:HGNC:30186]	chr12:1108	NM_00116	XM_00525	.	.	NP_00115	XP_005253	protein_co	ENSG0000	51184	30186
GPNMB	1.08908	0.944697	0.999674	0.884468	1.06129	1.06726	glycoprotein nmb [Source:HGNC Symbol;Acc:HGNC:4462]	chr7:23286	NM_00100	XM_00524	.	.	NP_00100	XP_005249	protein_co	ENSG0000	10457	4462
GPR1	0.042243	0	0	0	0	0	G protein-coupled receptor 1 [Source:HGNC Symbol;Acc:HGNC:4	chr2:20704	NM_00109	XM_00524	.	.	NP_00109	XP_005246	protein_co	ENSG0000	2825	4463
GPR101	0	0	0	0	0	0	G protein-coupled receptor 101 [Source:HGNC Symbol;Acc:HGNC	chrX:13611	NM_05402	.	.	.	NP_47336	.	protein_co	ENSG0000	83550	14963
GPR107	17.8769	17.77	18.1894	15.4694	16.9871	20.6554	G protein-coupled receptor 107 [Source:HGNC Symbol;Acc:HGNC	chr9:13281	NM_00113	.	.	.	NP_00113	XP_01687	protein_co	ENSG0000	57720	17830
GPR108	34.9411	31.7534	29.6269	26.2275	23.6056	27.5758	G protein-coupled receptor 108 [Source:HGNC Symbol;Acc:HGNC	chr19:6729	NM_00108	XM_01152	NR_10684	.	NP_00107	XP_006722	protein_co	ENSG0000	102465474	17829
GPR119	0	0	0.091829	0	0.122572	0	G protein-coupled receptor 119 [Source:HGNC Symbol;Acc:HGNC	chrX:12951	NM_17847	.	.	.	NP_84856	.	protein_co	ENSG0000	139760	19060
GPR12	0	0	0	0	0	0	G protein-coupled receptor 12 [Source:HGNC Symbol;Acc:HGNC:	chr13:2732	NM_00528	XM_00526	.	.	NP_00527	XP_005266	protein_co	ENSG0000	2835	4466
GPR132	0.06154	0	0	0.032992	0	0.033884	G protein-coupled receptor 132 [Source:HGNC Symbol;Acc:HGNC	chr14:1055	NM_00127	.	.	.	NP_00126	.	protein_co	ENSG0000	29933	17482
GPR135	0.789516	0.925558	1.24791	0.2484	0.733982	0.736024	G protein-coupled receptor 135 [Source:HGNC Symbol;Acc:HGNC	chr14:5992	NM_02257	XM_01153	.	.	NP_07209	XP_011535	protein_co	ENSG0000	64582	19991
GPR137	8.81739	7.50058	6.90941	7.31969	5.49799	5.83533	G protein-coupled receptor 137 [Source:HGNC Symbol;Acc:HGNC	chr11:6403	NM_00117	XM_01701	.	.	NP_00116	XP_005274	protein_co	ENSG0000	56834	24300
GPR137B	6.04624	6.6716	10.9528	7.44876	7.59963	7.50076	G protein-coupled receptor 137B [Source:HGNC Symbol;Acc:HGN	chr1:23630	NM_00327	.	.	.	NP_00326	XP_016857	protein_co	ENSG0000	7107	11862
GPR137C	0.462516	0.168978	0.642913	0.089545	0.39104	0.844671	G protein-coupled receptor 137C [Source:HGNC Symbol;Acc:HGN	chr14:5301	NM_00109	.	.	.	NP_00109	.	protein_co	ENSG0000	283554	25445
GPR139	0	0	0	0	0	0	G protein-coupled receptor 139 [Source:HGNC Symbol;Acc:HGNC	chr16:2004	NM_00100	.	.	.	NP_00100	.	protein_co	ENSG0000	124274	19995
GPR141	0	0	0	0	0	0	G protein-coupled receptor 141 [Source:HGNC Symbol;Acc:HGNC	chr7:37723	NM_18179	XM_01151	.	.	NP_86145	XP_011513	protein_co	ENSG0000	353345	19997
GPR142	0	0.060348	0.052342	0	0	0.066934	G protein-coupled receptor 142 [Source:HGNC Symbol;Acc:HGNC	chr17:7236	NM_18179	.	.	.	NP_86145	.	protein_co	ENSG0000	350383	20088
GPR143	0.719798	1.1417	1.67799	2.7425	2.653	2.454	G protein-coupled receptor 143 [Source:HGNC Symbol;Acc:HGNC	chrX:96933	NM_00027	.	.	.	NP_00026	XP_005274	protein_co	ENSG0000	4935	20145
GPR146	0.410479	0.454812	0.130317	0.384888	0.120432	0.395201	G protein-coupled receptor 146 [Source:HGNC Symbol;Acc:HGNC	chr7:10366	NM_00130	XM_00671	.	.	NP_00129	XP_006715	protein_co	ENSG0000	115330	21718
GPR148	0	0	0	0	0	0	G protein-coupled receptor 148 [Source:HGNC Symbol;Acc:HGNC	chr2:13148	NM_20736	XM_01151	.	.	NP_99724	XP_011509	protein_co	ENSG0000	344561	23623
GPR149	0	0	0	0	0	0	G protein-coupled receptor 149 [Source:HGNC Symbol;Acc:HGNC	chr3:15405	NM_00103	.	.	.	NP_00103	.	protein_co	ENSG0000	344758	23627
GPR15	0.518774	0.233172	0.829693	1.65539	1.48346	1.12055	G protein-coupled receptor 15 [Source:HGNC Symbol;Acc:HGNC:	chr3:98250	NM_00529	.	.	.	NP_00528	.	protein_co	ENSG0000	2838	4469
GPR150	0	0	0	0	0	0	G protein-coupled receptor 150 [Source:HGNC Symbol;Acc:HGNC	chr5:94955	NM_19924	XM_01154	.	.	NP_95471	.	protein_co	ENSG0000	285601	23628
GPR151	0	0	0	0	0	0	G protein-coupled receptor 151 [Source:HGNC Symbol;Acc:HGNC	chr5:14589	NM_19425	.	.	.	NP_91922	.	protein_co	ENSG0000	134391	23624
GPR152	0	0	0	0	0	0	G protein-coupled receptor 152 [Source:HGNC Symbol;Acc:HGNC	chr11:6721	NM_20699	.	.	.	NP_99688	.	protein_co	ENSG0000	390212	23622
GPR153	3.18604	2.68293	5.44818	2.81422	2.40136	3.21256	G protein-coupled receptor 153 [Source:HGNC Symbol;Acc:HGNC	chr1:63074	NM_20737	XM_01700	.	.	NP_99725	XP_011539	protein_co	ENSG0000	387509	23618
GPR155	1.05375	1.04884	1.78503	0.933579	1.50646	1.52857	G protein-coupled receptor 155 [Source:HGNC Symbol;Acc:HGNC	chr2:17529	NM_00103	XM_01700	.	.	NP_00102	XP_016858	protein_co	ENSG0000	151556	22951
GPR156	0.064107	0.150201	0.055706	0.035414	0	0	G protein-coupled receptor 156 [Source:HGNC Symbol;Acc:HGNC	chr3:11988	NM_00116	XM_01700	.	.	NP_00116	XP_005247	protein_co	ENSG0000	165829	20844
GPR157	2.98365	3.36428	4.3529	3.36931	3.53835	3.72377	G protein-coupled receptor 157 [Source:HGNC Symbol;Acc:HGNC	chr1:91603	NM_02498	XM_00526	.	.	NP_07925	XP_005263	protein_co	ENSG0000	80045	23687
GPR158	1.03861	0.877645	0.663735	0.536614	0.68631	0.853935	G protein-coupled receptor 158 [Source:HGNC Symbol;Acc:HGNC	chr10:2546	NM_02075	.	.	.	NP_06580	.	protein_co	ENSG0000	57512	23689
GPR160	4.05643	4.70121	5.87085	3.66356	4.2749	6.13141	G protein-coupled receptor 160 [Source:HGNC Symbol;Acc:HGNC	chr3:16975	NM_01437	XM_01151	.	.	NP_05518	XP_005247	protein_co	ENSG0000	26996	23693
GPR161	7.17749	8.43818	8.17931	9.22006	7.89595	6.71817	G protein-coupled receptor 161 [Source:HGNC Symbol;Acc:HGNC	chr1:16804	NM_00126	XM_01700	.	.	NP_00125	XP_005245	protein_co	ENSG0000	23432	23694
GPR162	2.2117	1.62822	1.51052	1.72414	1.00082	1.1692	G protein-coupled receptor 162 [Source:HGNC Symbol;Acc:HGNC	chr12:6930	NM_01444	.	.	.	NP_05526	.	protein_co	ENSG0000	27239	16693
GPR17	0.091533	0.083732	0	0.044437	0.098118	0	G protein-coupled receptor 17 [Source:HGNC Symbol;Acc:HGNC:	chr2:12839	NM_00116	.	.	.	NP_00115	XP_016859	protein_co	ENSG0000	2840	4471
GPR171	0	0	0	0	0	0	G protein-coupled receptor 171 [Source:HGNC Symbol;Acc:HGNC	chr3:15080	NM_01330	XM_00524	.	.	NP_03744	XP_005247	protein_co	ENSG0000	29909	30057
GPR173	2.33695	2.10845	2.05985	2.02419	1.81069	2.01399	G protein-coupled receptor 173 [Source:HGNC Symbol;Acc:HGNC	chrX:53078	NM_01896	XM_01153	.	.	NP_06184	XP_011529	protein_co	ENSG0000	54328	18186
GPR174	0	0	0	0	0	0	G protein-coupled receptor 174 [Source:HGNC Symbol;Acc:HGNC	chrX:78426	NM_03255	.	.	.	NP_11594	.	protein_co	ENSG0000	84636	30245
GPR176	0.923443	1.1344	1.06643	0.380483	0.410087	0.61486	G protein-coupled receptor 176 [Source:HGNC Symbol;Acc:HGNC	chr15:4009	NM_00127	.	.	.	NP_00125	.	protein_co	ENSG0000	11245	32370
GPR179	0.121336	0.093504	0.112217	0.186198	0.196238	0.199423	G protein-coupled receptor 179 [Source:HGNC Symbol;Acc:HGNC	chr17:3648	NM_00100	.	.	.	NP_00100	.	protein_co	ENSG0000	440435	31371
GPR18	0	0	0	0	0	0	G protein-coupled receptor 18 [Source:HGNC Symbol;Acc:HGNC:	chr13:9985	NM_00109	XM_01152	.	.	NP_00109	XP_006720	protein_co	ENSG0000	2841	4472
GPR180	3.43415	3.66015	3.37409	3.07949	4.27605	5.23829	G protein-coupled receptor 180 [Source:HGNC Symbol;Acc:HGNC	chr13:9525	NM_18098	.	.	.	NP_85132	.	protein_co	ENSG0000	160897	28899
GPR182	0	0	0	0	0	0.024548	G protein-coupled receptor 182 [Source:HGNC Symbol;Acc:HGNC	chr12:5738	NM_00726	XM_01153	.	.	NP_00919	XP_011536	protein_co	ENSG0000	11318	13708
GPR183	0.055307	0.238286	0.047752	0.063459	0.121388	0.106963	G protein-coupled receptor 183 [Source:HGNC Symbol;Acc:HGNC	chr13:9985	NM_00495	.	.	.	NP_00494	XP_016875	protein_co	ENSG0000	1880	3128
GPR19	1.20558	0.768014	0.548171	0.476517	0.832071	0.633538	G protein-coupled receptor 19 [Source:HGNC Symbol;Acc:HGNC:	chr12:1281	NM_00614	XM_01152	.	.	NP_00613	XP_011518	protein_co	ENSG0000	2842	4473
GPR20	0.800594	0.853035	0.880131	1.35232	2.28431	0.726602	G protein-coupled receptor 20 [Source:HGNC Symbol;Acc:HGNC:	chr8:14236	NM_00529	XM_01151	.	.	NP_00528	XP_011515	protein_co	ENSG0000	2843	4475
GPR21	0	0	0	0	0	0	G protein-coupled receptor 21 [Source:HGNC Symbol;Acc:HGNC:	chr9:12570	.	.	.	.	.	.	protein_co	ENSG0000	.	4476
GPR22	0	0	0	0	0	0	G protein-coupled receptor 22 [Source:HGNC Symbol;Acc:HGNC:	chr7:10680	NM_00529	XM_01151	.	.	NP_00528	XP_011514	protein_co	ENSG0000	2845	4477
GPR25	0	0	0	0	0	0	G protein-coupled receptor 25 [Source:HGNC Symbol;Acc:HGNC:	chr1:20084	NM_00529	.	.	.	NP_00528	.	protein_co	ENSG0000	2848	4480
GPR26	0	0	0	0	0	0	G protein-coupled receptor 26 [Source:HGNC Symbol;Acc:HGNC:	chr10:1254	NM_15344	.	.	.	NP_70314	.	protein_co	ENSG0000	2849	4481
GPR27	0.054128	0	0.046066	0.240203	0.061331	0.055548	G protein-coupled receptor 27 [Source:HGNC Symbol;Acc:HGNC:	chr3:71724	NM_01897	.	.	.	NP_06184	.	protein_co	ENSG0000	2850	4482
GPR3	4.61285	3.41415	3.67223	5.43951	4.94655	2.04886	G protein-coupled receptor 3 [Source:HGNC Symbol;Acc:HGNC:4	chr1:27719	NM_00528	.	.	.	NP_00527	.	protein_co	ENSG0000	2827	4484
GPR31	0	0	0	0	0	0	G protein-coupled receptor 31 [Source:HGNC Symbol;Acc:HGNC:	chr6:16756	NM_00529	.	.	.	NP_00529	.	protein_co	ENSG0000	2853	4486
GPR32	0	0.089335	0	0	0	0	G protein-coupled receptor 32 [Source:HGNC Symbol;Acc:HGNC:	chr19:5127	NM_00150	.	.	.	NP_00149	.	protein_co	ENSG0000	2854	4487
GPR33	0	0	0	0	0	0	G protein-coupled receptor 33 (gene/pseudogene) [Source:HGNC	chr14:3195	NM_00119	.	.	.	NP_00118	.	protein_co	ENSG0000	2856	4489
GPR34	0	0	0	0	0	0	G protein-coupled receptor 34 [Source:HGNC Symbol;Acc:HGNC:	chrX:41374	NM_00109	XM_00527	.	.	NP_00109	XP_005272	protein_co	ENSG0000	2857	4490
GPR35	0.976766	0.592106	1.38487	0.398217	1.08096	1.09251	G protein-coupled receptor 35 [Source:HGNC Symbol;Acc:HGNC:	chr2:24152	NM_00119	.	.	.	NP_00118	.	protein_co	ENSG0000	2859	4492
GPR37	1.18488	1.47068	1.6323	2.18164	1.75696	2.08344	G protein-coupled receptor 37 [Source:HGNC Symbol;Acc:HGNC:	chr7:12438	NM_00530	.	.	.	NP_00529	.	protein_co	ENSG0000	2861	4494
GPR37L1	0.062871	0.063003	0.151692	0.108404	0.028833	0.040793	G protein-coupled receptor 37 like 1 [Source:HGNC Symbol;Acc:H	chr1:20209	NM_00476	.	.	.	NP_00475	.	protein_co	ENSG0000	9283	14923

GPR39	4.69275	5.3046	2.90666	3.86742	3.23401	3.29116	G protein-coupled receptor 39 [Source:HGNC Symbol;Acc:HGNC:4	chr2:13317	NM_00150	.	.	.	NP_00149	XP_011509	protein_co	ENSG0000	2863	4496
GPR4	0.121795	0.030722	0.052169	0.066184	0.139353	0.097407	G protein-coupled receptor 4 [Source:HGNC Symbol;Acc:HGNC:44	chr19:4603	NM_00528	XM_01702	.	.	NP_00527	XP_016882	protein_co	ENSG0000	2828	4497
GPR42	0	0	0	0	0	0	G protein-coupled receptor 42 (gene/pseudogene) [Source:HGNC	chr19:3586	.	.	.	.	.	.	protein_co	ENSG0000	.	4500
GPR45	0.091277	0.08955	0.386485	0.583989	0.42493	0.504566	G protein-coupled receptor 45 [Source:HGNC Symbol;Acc:HGNC:4	chr2:10585	NM_00722	.	.	.	NP_00915	.	protein_co	ENSG0000	11250	4503
GPR50	0.042768	0.043503	0.036441	0	0	0	G protein-coupled receptor 50 [Source:HGNC Symbol;Acc:HGNC:4	chrX:15034	NM_00422	.	.	.	NP_00421	.	protein_co	ENSG0000	9248	4506
GPR52	0	0	0	0.118327	0	0	G protein-coupled receptor 52 [Source:HGNC Symbol;Acc:HGNC:4	chr1:17412	NM_00568	.	.	.	NP_00567	.	protein_co	ENSG0000	9293	4508
GPR55	0.027505	0.027965	0	0	0	0.029136	G protein-coupled receptor 55 [Source:HGNC Symbol;Acc:HGNC:4	chr2:23177	NM_00568	XM_01151	.	.	NP_00567	XP_005247	protein_co	ENSG0000	9290	4511
GPR6	0	0	0	0	0	0	G protein-coupled receptor 6 [Source:HGNC Symbol;Acc:HGNC:45	chr6:11029	NM_00128	.	.	.	NP_00127	.	protein_co	ENSG0000	2830	4515
GPR61	0	0	0.013278	0	0.051541	0	G protein-coupled receptor 61 [Source:HGNC Symbol;Acc:HGNC:1	chr1:11008	NM_03193	XM_01154	.	.	NP_11414	XP_011540	protein_co	ENSG0000	83873	13300
GPR62	0.091753	0	0	0	0	0.050645	G protein-coupled receptor 62 [Source:HGNC Symbol;Acc:HGNC:1	chr3:51989	NM_08086	.	.	.	NP_54314	.	protein_co	ENSG0000	118442	13301
GPR63	3.10082	3.45743	2.74192	3.40595	3.6609	3.33106	G protein-coupled receptor 63 [Source:HGNC Symbol;Acc:HGNC:1	chr6:97242	NM_00114	XM_01153	.	.	NP_00113	XP_006715	protein_co	ENSG0000	81491	13302
GPR65	0	0	0	0	0	0	G protein-coupled receptor 65 [Source:HGNC Symbol;Acc:HGNC:4	chr14:8847	NM_00360	.	.	.	NP_00359	.	protein_co	ENSG0000	8477	4517
GPR68	0	0.054295	0.023832	0.029462	0	0.030124	G protein-coupled receptor 68 [Source:HGNC Symbol;Acc:HGNC:4	chr14:9169	NM_00117	XM_01153	.	.	NP_00117	XP_005268	protein_co	ENSG0000	8111	4519
GPR75	1.21057	0.687659	1.2779	0.474858	0.660635	0.686346	G protein-coupled receptor 75 [Source:HGNC Symbol;Acc:HGNC:4	chr2:53897	NM_00679	.	.	.	NP_00678	.	protein_co	ENSG0000	10936	4526
GPR75-ASB3	5.16E-06	0.305407	1.79E-11	6.69E-15	4.67E-05	2.01E-08	GPR75-ASB3 readthrough [Source:HGNC Symbol;Acc:HGNC:4004	chr2:53897	NM_00116	.	.	.	NP_00115	.	protein_co	ENSG0000	100302652	40043
GPR78	0	0	0	0	0	0	G protein-coupled receptor 78 [Source:HGNC Symbol;Acc:HGNC:4	chr4:85822	NM_08081	.	.	.	NP_54300	.	protein_co	ENSG0000	27201	4528
GPR82	0	0	0	0	0	0	G protein-coupled receptor 82 [Source:HGNC Symbol;Acc:HGNC:4	chrX:41374	NM_08081	XM_01702	.	.	NP_54300	XP_016884	protein_co	ENSG0000	27197	4533
GPR83	0.263027	0.183029	0.197395	0.173387	0.059183	0.14326	G protein-coupled receptor 83 [Source:HGNC Symbol;Acc:HGNC:4	chr11:9411	NM_01654	XM_01154	.	.	NP_05762	XP_011540	protein_co	ENSG0000	10888	4523
GPR84	0	0.058644	0.099617	0.079814	0.040771	0.061464	G protein-coupled receptor 84 [Source:HGNC Symbol;Acc:HGNC:4	chr12:5475	NM_02037	XM_01153	.	.	NP_06510	XP_011536	protein_co	ENSG0000	53831	4535
GPR85	0	0	0	0	0	0	G protein-coupled receptor 85 [Source:HGNC Symbol;Acc:HGNC:4	chr7:11272	NM_00114	XM_00525	.	.	NP_00113	XP_005250	protein_co	ENSG0000	54329	4536
GPR87	3.68501	2.81511	2.16192	2.76068	2.19731	2.2615	G protein-coupled receptor 87 [Source:HGNC Symbol;Acc:HGNC:4	chr3:15080	NM_02391	.	.	.	NP_07640	.	protein_co	ENSG0000	53836	4538
GPR88	0.034604	0	0	0	0	0	G protein-coupled receptor 88 [Source:HGNC Symbol;Acc:HGNC:4	chr1:10100	NM_02204	.	.	.	NP_07133	.	protein_co	ENSG0000	54112	4539
GPR89A	2.24231	1.88478	2.32805	2.31955	1.63248	1.94681	G protein-coupled receptor 89A [Source:HGNC Symbol;Acc:HGNC	chr1:14576	NM_00109	XM_01150	.	.	NP_00109	XP_006711	protein_co	ENSG0000	653519	31984
GPR89B	0.477045	0.229449	0.301118	0.391715	0.386008	0.128804	G protein-coupled receptor 89B [Source:HGNC Symbol;Acc:HGNC	chr1:14740	NM_01633	XM_01150	.	.	NP_05741	XP_005277	protein_co	ENSG0000	51463	13840
GPR89C	0	0	0	0	0	0	G protein-coupled receptor 89C [Source:HGNC Symbol;Acc:31985	chr1:14588	NM_00109	.	.	.	NP_00109	XP_005273	protein_co	ENSG0000	728932	31985
GPRASP1	0.197473	0.245813	0.225916	0.354846	0.266308	0.198831	G protein-coupled receptor associated sorting protein 1 [Source:	chrX:10190	NM_00109	.	.	.	NP_00109	XP_016885	protein_co	ENSG0000	9737	24834
GPRASP2	1.45885	1.53306	1.72276	1.43126	1.76992	1.85391	G protein-coupled receptor associated sorting protein 2 [Source:	chrX:10196	NM_00100	.	.	.	NP_00100	.	protein_co	ENSG0000	100528062	25169
GPRC5A	36.5368	37.2041	84.2348	27.0202	28.2761	39.1099	G protein-coupled receptor class C group 5 member A [Source:HG	chr12:1304	NM_00397	.	NR_03034	.	NP_00397	.	protein_co	ENSG0000	693199	9836
GPRC5B	4.39503	5.08614	5.84471	6.53312	5.82903	4.90001	G protein-coupled receptor class C group 5 member B [Source:HG	chr16:1971	NM_01623	XM_00672	.	.	NP_05731	XP_006721	protein_co	ENSG0000	51704	13308
GPRC5C	23.0608	23.5561	24.0245	28.5949	25.1596	25.2798	G protein-coupled receptor class C group 5 member C [Source:HG	chr17:7242	NM_01865	.	.	.	NP_06112	XP_005257	protein_co	ENSG0000	55890	13309
GPRC5D	0	0	0	0	0	0	G protein-coupled receptor class C group 5 member D [Source:HG	chr12:1309	NM_01865	XM_01152	.	.	NP_06112	XP_005253	protein_co	ENSG0000	55507	13310
GPRC6A	0	0	0	0	0	0	G protein-coupled receptor class C group 6 member A [Source:HG	chr6:11711	NM_00128	.	.	.	NP_00127	XP_016865	protein_co	ENSG0000	222545	18510
GPRIN1	11.2256	11.3971	11.5904	11.1294	9.7163	6.58576	G protein regulated inducer of neurite outgrowth 1 [Source:HGN	chr5:17596	NM_05289	XM_01153	.	.	NP_44313	XP_005265	protein_co	ENSG0000	114787	24835
GPRIN2	1.17245	1.3437	1.41036	1.18266	1.19255	1.08338	G protein regulated inducer of neurite outgrowth 2 [Source:HGN	chr10:4699	NM_01469	XM_01154	.	.	NP_05551	XP_005277	protein_co	ENSG0000	9721	23730
GPRIN3	0.156437	0.125833	0.376791	0.178201	0.245262	0.288228	GPRIN family member 3 [Source:HGNC Symbol;Acc:HGNC:27733]	chr4:90157	NM_19828	XM_00526	.	.	NP_93802	XP_005262	protein_co	ENSG0000	285513	27733
GPS1	84.5476	82.7033	79.4905	95.799	77.8373	64.9652	G protein pathway suppressor 1 [Source:HGNC Symbol;Acc:HGNC	chr17:8000	NM_00132	XM_01702	.	.	NP_00130	XP_005256	protein_co	ENSG0000	2873	4549
GPS2	30.5925	30.6346	26.4975	33.5317	28.7375	23.4588	G protein pathway suppressor 2 [Source:HGNC Symbol;Acc:HGNC	chr17:7215	NM_00448	.	.	.	NP_00448	.	protein_co	ENSG0000	2874	4550
GPSM1	11.5273	13.6587	15.0516	10.5457	10.3539	10.029	G-protein signaling modulator 1 [Source:HGNC Symbol;Acc:HGNC	chr9:13922	NM_00114	XM_01151	.	.	NP_00113	XP_011516	protein_co	ENSG0000	26086	17858
GPSM2	18.2154	15.736	16.184	25.1595	30.4499	26.441	G-protein signaling modulator 2 [Source:HGNC Symbol;Acc:HGNC	chr1:10935	NM_01329	XM_01154	.	.	NP_00130	XP_005270	protein_co	ENSG0000	29899	29501
GPSM3	1.03422	1.23843	0.789534	1.20145	1.29062	0.847009	G-protein signaling modulator 3 [Source:HGNC Symbol;Acc:HGNC	chr6:32158	NM_00127	.	.	.	NP_00126	.	protein_co	ENSG0000	63940	13945
GPT	0.054865	0.31562	0.370138	0.114357	0.193679	0.062628	glutamic--pyruvic transaminase [Source:HGNC Symbol;Acc:HGNC	chr8:14572	NM_00530	XM_01151	.	.	NP_00530	XP_011515	protein_co	ENSG0000	2875	4552
GPT2	18.1232	18.9203	15.3234	15.6851	16.1563	36.597	glutamic--pyruvic transaminase 2 [Source:HGNC Symbol;Acc:HGN	chr16:4691	NM_00114	.	.	.	NP_00113	.	protein_co	ENSG0000	84706	18062
GPX1	351.641	351.28	431.473	467.825	331.879	352.799	glutathione peroxidase 1 [Source:HGNC Symbol;Acc:HGNC:4553]	chr3:49394	NM_00058	.	.	.	NP_00057	.	protein_co	ENSG0000	2876	4553
GPX2	0.087213	0	0	0.095199	0	0	glutathione peroxidase 2 [Source:HGNC Symbol;Acc:HGNC:4554]	chr14:6538	NM_00208	.	.	.	NP_00207	.	protein_co	ENSG0000	2877	4554
GPX3	0	0	0	0	0	0	glutathione peroxidase 3 [Source:HGNC Symbol;Acc:HGNC:4555]	chr5:15039	NM_00208	.	.	.	NP_00207	.	protein_co	ENSG0000	2878	4555
GPX4	321.847	335.691	337.805	334.497	240.161	285.534	glutathione peroxidase 4 [Source:HGNC Symbol;Acc:HGNC:4556]	chr19:1103	NM_00103	.	.	.	NP_00103	.	protein_co	ENSG0000	2879	4556
GPX5	0	0	0	0	0	0	glutathione peroxidase 5 [Source:HGNC Symbol;Acc:HGNC:4557]	chr6:28493	NM_00150	.	.	.	NP_00150	.	protein_co	ENSG0000	2880	4557
GPX6	0	0	0	0	0	0	glutathione peroxidase 6 [Source:HGNC Symbol;Acc:HGNC:4558]	chr6:28471	NM_18270	.	.	.	NP_87436	.	protein_co	ENSG0000	257202	4558
GPX7	0.065558	0	0	0.144766	0	0.068282	glutathione peroxidase 7 [Source:HGNC Symbol;Acc:HGNC:4559]	chr1:53068	NM_01569	.	.	.	NP_05651	.	protein_co	ENSG0000	2882	4559
GPX8	39.7757	41.304	25.8777	43.9688	45.6403	53.1562	glutathione peroxidase 8 (putative) [Source:HGNC Symbol;Acc:HG	chr5:54408	NM_00100	XM_01154	NR_13133	.	NP_00100	XP_006714	protein_co	ENSG0000	493869	33100
GRAMD1A	27.7061	25.4479	30.8872	25.8639	27.0178	21.36	GRAM domain containing 1A [Source:HGNC Symbol;Acc:HGNC:29	chr19:3548	NM_00113	XM_01702	.	.	NP_00112	XP_011525	protein_co	ENSG0000	57655	29305
GRAMD1B	1.48267	1.17287	0.717192	1.67226	1.43233	2.96634	GRAM domain containing 1B [Source:HGNC Symbol;Acc:HGNC:29	chr11:1232	NM_00128	XM_01701	.	.	NP_00127	XP_005271	protein_co	ENSG0000	57476	29214
GRAMD1C	1.5117	1.80561	2.9949	2.10269	2.81305	3.73188	GRAM domain containing 1C [Source:HGNC Symbol;Acc:HGNC:25	chr3:11355	NM_00117	XM_01151	.	.	NP_00116	XP_005247	protein_co	ENSG0000	54762	25252
GRAMD2	3.81966	4.27159	4.23801	4.26848	4.63521	3.7658	GRAM domain containing 2 [Source:HGNC Symbol;Acc:HGNC:272	chr15:7245	NM_00101	XM_01702	.	.	NP_00101	XP_011519	protein_co	ENSG0000	196996	27287
GRAMD3	5.05246	4.42975	4.33442	5.76508	5.16306	7.16052	GRAM domain containing 3 [Source:HGNC Symbol;Acc:HGNC:249	chr5:12569	NM_00114	XM_01154	.	.	NP_00113	XP_005272	protein_co	ENSG0000	65983	24911
GRAMD4	8.84393	9.29785	9.32472	7.46515	6.81865	6.9863	GRAM domain containing 4 [Source:HGNC Symbol;Acc:HGNC:291	chr22:4701	NM_01512	XM_01702	.	.	NP_05593	XP_005261	protein_co	ENSG0000	23151	29113
GRAP	0.328888	0.075803	0.281367	0.110927	0.058576	0.027668	GRB2-related adaptor protein [Source:HGNC Symbol;Acc:HGNC:4	chr17:1885	NM_00661	XM_00525	.	.	NP_00660	XP_005256	protein_co	ENSG0000	400581	4562
GRAP2	0.055754	0	0.059348	0.056088	0.128187	0.121163	GRB2-related adaptor protein 2 [Source:HGNC Symbol;Acc:HGNC	chr22:4029	NM_00129	XM_01153	.	.	NP_00127	XP_006724	protein_co	ENSG0000	9402	4563
GRAPL	0	0	0	0	0	0	GRB2 related adaptor protein like [Source:HGNC Symbol;Acc:HGN	chr17:1903	NM_00112	XM_01702	.	.	NP_00112	XP_016880	protein_co	ENSG0000	400581	37240

GRASP	0.210843	0.204006	0.40671	0.448582	0.633427	0.371519	general receptor for phosphoinositides 1 associated scaffold prot	chr12:5240	NM_00127	XM_01153	.	.	NP_00125	XP_005268	protein_co	ENSG0000	160622	18707
GRB10	11.5047	11.8905	7.65646	10.1064	10.326	17.6906	growth factor receptor bound protein 10 [Source:HGNC Symbol;A	chr7:50657	NM_00100	XM_01701	.	.	NP_00100	XP_005271	protein_co	ENSG0000	2887	4564
GRB14	3.9539	3.4407	3.53203	4.47353	4.43342	4.66785	growth factor receptor bound protein 14 [Source:HGNC Symbol;A	chr2:16534	NM_00449	.	.	.	NP_00448	.	protein_co	ENSG0000	2888	4565
GRB2	91.1927	95.6523	89.6191	105.217	97.6281	92.268	growth factor receptor bound protein 2 [Source:HGNC Symbol;Ac	chr17:7331	NM_00208	.	.	.	NP_00207	.	protein_co	ENSG0000	2885	4566
GRB7	15.1204	15.4891	14.8937	12.8057	10.9089	12.589	growth factor receptor bound protein 7 [Source:HGNC Symbol;Ac	chr17:3789	NM_00103	XM_01152	.	.	NP_00102	XP_016880	protein_co	ENSG0000	2886	4567
GREB1	1.70057	2.06608	2.13481	2.05924	1.97105	2.32661	growth regulation by estrogen in breast cancer 1 [Source:HGNC S	chr2:11674	NM_01466	XM_01151	.	.	NP_05548	XP_005246	protein_co	ENSG0000	9687	24885
GREB1L	1.10465	1.1737	1.53825	1.68612	2.11894	2.25827	growth regulation by estrogen in breast cancer 1 like [Source:HG	chr18:1882	NM_00114	XM_01152	.	.	NP_00113	XP_006722	protein_co	ENSG0000	80000	31042
GREM1	0	0.03114	0	0	0	0	gremlin 1, DAN family BMP antagonist [Source:HGNC Symbol;Acc	chr15:3301	NM_00119	.	.	.	NP_00117	.	protein_co	ENSG0000	26585	2001
GREM2	0	0	0	0	0	0	gremlin 2, DAN family BMP antagonist [Source:HGNC Symbol;Acc	chr1:24065	NM_02246	XM_01154	.	.	NP_07191	XP_005273	protein_co	ENSG0000	64388	17655
GRHL1	2.8201	3.46459	3.54263	3.62025	3.43792	4.84682	grainyhead like transcription factor 1 [Source:HGNC Symbol;Acc:H	chr2:10091	NM_19818	XM_01151	.	.	NP_93782	XP_005246	protein_co	ENSG0000	29841	17923
GRHL2	5.98736	6.93878	6.6058	7.26717	7.54446	8.37606	grainyhead like transcription factor 2 [Source:HGNC Symbol;Acc:H	chr8:10250	NM_02491	XM_01151	.	.	NP_07919	XP_011515	protein_co	ENSG0000	79977	2799
GRHL3	0.33693	0.229603	0.327076	0.082147	0.110366	0.139052	grainyhead like transcription factor 3 [Source:HGNC Symbol;Acc:H	chr1:24645	NM_00119	XM_01154	.	.	NP_00118	XP_011540	protein_co	ENSG0000	57822	25839
GRHPR	60.1263	58.2851	61.5175	68.0682	53.3221	55.0964	glyoxylate and hydroxypyruvate reductase [Source:HGNC Symbol	chr9:37422	NM_01220	XM_01151	.	.	NP_03633	XP_011516	protein_co	ENSG0000	9380	4570
GRIA1	0	0.013574	0	0	0	0	glutamate ionotropic receptor AMPA type subunit 1 [Source:HGN	chr5:15287	NM_00082	XM_01700	.	.	NP_00081	XP_011535	protein_co	ENSG0000	2890	4571
GRIA2	0	0	0.018456	0	0	0	glutamate ionotropic receptor AMPA type subunit 2 [Source:HGN	chr4:15814	NM_00082	XM_01700	.	.	NP_00081	XP_016863	protein_co	ENSG0000	2891	4572
GRIA3	0	0	0	0	0	0	glutamate ionotropic receptor AMPA type subunit 3 [Source:HGN	chrX:12231	NM_00082	.	.	.	NP_00081	.	protein_co	ENSG0000	2892	4573
GRIA4	0	0	0	0	0	0	glutamate ionotropic receptor AMPA type subunit 4 [Source:HGN	chr11:1054	NM_00082	XM_01154	.	.	NP_00082	XP_005271	protein_co	ENSG0000	2893	4574
GRID1	1.04924	1.06408	0.940164	0.830953	1.03437	0.741221	glutamate ionotropic receptor delta type subunit 1 [Source:HGN	chr10:8735	NM_01755	XM_01153	.	.	NP_06002	XP_011538	protein_co	ENSG0000	2894	4575
GRID2	0	0	0	0	0	0	glutamate ionotropic receptor delta type subunit 2 [Source:HGN	chr4:93224	NM_00128	XM_01153	.	.	NP_00127	XP_011530	protein_co	ENSG0000	2895	4576
GRID2IP	0.783939	0.53771	1.2002	0.915699	0.988651	1.21604	Grid2 interacting protein [Source:HGNC Symbol;Acc:HGNC:18464	chr7:65370	NM_00114	XM_01151	.	.	NP_00113	XP_011513	protein_co	ENSG0000	392862	18464
GRIFIN	0	0	0	0	0	0	galectin-related inter-fiber protein [Source:HGNC Symbol;Acc:HG	chr7:25144	.	.	.	.	NP_00127	.	protein_co	ENSG0000	402635	4577
GRIK1	0	0	0	0	0	0	glutamate ionotropic receptor kainate type subunit 1 [Source:HG	chr21:3090	NM_00083	XM_00672	.	.	NP_00082	XP_005260	protein_co	ENSG0000	2897	4579
GRIK1-AS2	0.077917	0	0	0	0	0	GRIK1 antisense RNA 2 [Source:HGNC Symbol;Acc:1282]	chr21:3090	.	.	.	.	.	.	protein_co	ENSG0000	.	1282
GRIK2	0.038104	0.12194	0.241029	0.021625	0.063645	0.112431	glutamate ionotropic receptor kainate type subunit 2 [Source:HG	chr6:10184	NM_00116	XM_01153	.	.	NP_00115	XP_005267	protein_co	ENSG0000	2898	4580
GRIK3	0	0	0	0	0	0	glutamate ionotropic receptor kainate type subunit 3 [Source:HG	chr1:37261	NM_00083	.	.	.	NP_00082	.	protein_co	ENSG0000	2899	4581
GRIK4	0.095777	0.057587	0.263864	0.311943	0.174801	0.376597	glutamate ionotropic receptor kainate type subunit 4 [Source:HG	chr11:1203	NM_00128	XM_01154	.	.	NP_00126	XP_011541	protein_co	ENSG0000	2900	4582
GRIK5	0.457782	0.580062	0.785784	0.306213	0.424492	0.595401	glutamate ionotropic receptor kainate type subunit 5 [Source:HG	chr19:4250	NM_00130	XM_01152	.	.	NP_00128	XP_005258	protein_co	ENSG0000	2901	4583
GRIN1	3.27908	3.07792	3.98597	1.88752	1.68811	1.92338	glutamate ionotropic receptor NMDA type subunit 1 [Source:HGN	chr9:14003	NM_00083	XM_01151	.	.	NP_00082	XP_005266	protein_co	ENSG0000	2902	4584
GRIN2A	0.01497	0.015412	0.026023	0	0	0	glutamate ionotropic receptor NMDA type subunit 2A [Source:HG	chr16:9852	NM_00083	XM_01702	.	.	NP_00082	XP_011520	protein_co	ENSG0000	2903	4585
GRIN2B	0.549416	0.539325	1.25989	0.367115	0.504586	0.56152	glutamate ionotropic receptor NMDA type subunit 2B [Source:HG	chr12:1369	NM_00083	XM_01701	.	.	NP_00082	XP_005253	protein_co	ENSG0000	2904	4586
GRIN2C	0.757983	0.402021	0.740571	0.625255	0.530892	0.784495	glutamate ionotropic receptor NMDA type subunit 2C [Source:HG	chr17:7283	NM_00083	XM_01152	.	.	NP_00082	XP_006721	protein_co	ENSG0000	2905	4587
GRIN2D	10.6009	10.5537	12.4662	9.95723	9.7607	9.21616	glutamate ionotropic receptor NMDA type subunit 2D [Source:HG	chr19:4889	NM_00083	XM_01152	.	.	NP_00082	XP_011525	protein_co	ENSG0000	2906	4588
GRIN3A	0	0	0	0	0	0	glutamate ionotropic receptor NMDA type subunit 3A [Source:HG	chr9:10433	NM_13344	.	.	.	NP_59770	.	protein_co	ENSG0000	116443	16767
GRIN3B	0.152946	0.071788	0.224362	0	0.045923	0.223516	glutamate ionotropic receptor NMDA type subunit 3B [Source:HG	chr19:1000	NM_13869	.	.	.	NP_61963	.	protein_co	ENSG0000	116444	16768
GRINA	119.734	122.616	124.187	109.683	97.7595	88.0384	glutamate ionotropic receptor NMDA type subunit associated pro	chr8:14506	NM_00083	XM_00525	.	.	NP_00082	XP_005250	protein_co	ENSG0000	2907	4589
GRIP1	0	0.013749	0.064387	0.044186	0.029391	0.039649	glutamate receptor interacting protein 1 [Source:HGNC Symbol;A	chr12:6669	NM_00117	XM_01153	.	.	NP_00117	XP_005268	protein_co	ENSG0000	23426	18708
GRIP2	0.27424	0.160153	0.23095	0.280963	0.238397	0.21128	glutamate receptor interacting protein 2 [Source:HGNC Symbol;A	chr3:14444	NM_00108	XM_01153	.	.	NP_00107	XP_011532	protein_co	ENSG0000	80852	23841
GRIPAP1	9.76231	8.54986	9.61399	7.47068	8.0604	9.61464	GRIP1 associated protein 1 [Source:HGNC Symbol;Acc:HGNC:187	chrX:48830	NM_02013	XM_01702	.	.	NP_06452	XP_011542	protein_co	ENSG0000	56850	18706
GRK1	0	0	0	0.017313	0	0.017838	G protein-coupled receptor kinase 1 [Source:HGNC Symbol;Acc:H	chr13:1143	.	.	.	.	.	.	protein_co	ENSG0000	.	10013
GRK2	35.8432	36.4028	35.0958	45.4994	38.0765	42.3858	G protein-coupled receptor kinase 2 [Source:HGNC Symbol;Acc:H	chr11:6703	NM_00161	XM_01154	.	.	NP_00161	XP_011543	protein_co	ENSG0000	156	289
GRK3	3.99114	4.055	4.69176	4.85298	5.06832	6.15309	G protein-coupled receptor kinase 3 [Source:HGNC Symbol;Acc:H	chr22:2596	NM_00516	XM_01152	.	.	NP_00515	XP_011528	protein_co	ENSG0000	157	290
GRK4	2.57521	2.57617	2.6377	1.96252	2.33733	1.80797	G protein-coupled receptor kinase 4 [Source:HGNC Symbol;Acc:H	chr4:29653	NM_00100	XM_01151	.	.	NP_00100	XP_005248	protein_co	ENSG0000	2868	4543
GRK5	3.5421	3.76857	3.48755	3.35616	3.9819	2.46089	G protein-coupled receptor kinase 5 [Source:HGNC Symbol;Acc:H	chr10:1209	NM_00530	XM_00526	.	.	NP_00529	XP_005269	protein_co	ENSG0000	2869	4544
GRK6	41.5694	38.1395	42.9458	53.6695	46.3461	36.992	G protein-coupled receptor kinase 6 [Source:HGNC Symbol;Acc:H	chr5:17685	NM_00100	XM_01153	.	.	NP_00100	XP_006714	protein_co	ENSG0000	2870	4545
GRK7	0.047064	0.024443	0.020407	0	0.026542	0	G protein-coupled receptor kinase 7 [Source:HGNC Symbol;Acc:H	chr3:14149	NM_13920	XM_01151	.	.	NP_63194	XP_011510	protein_co	ENSG0000	131890	17031
GRM1	0	0	0.032921	0.012715	0	0	glutamate metabotropic receptor 1 [Source:HGNC Symbol;Acc:HG	chr6:14634	NM_00127	XM_01153	.	.	NP_00126	XP_011534	protein_co	ENSG0000	2911	4593
GRM2	0.095406	0.06106	0	0.051209	0	0	glutamate metabotropic receptor 2 [Source:HGNC Symbol;Acc:HG	chr3:51741	NM_00083	XM_01153	.	.	NP_00083	XP_011531	protein_co	ENSG0000	2912	4594
GRM3	0	0	0	0	0	0	glutamate metabotropic receptor 3 [Source:HGNC Symbol;Acc:HG	chr7:86273	NM_00084	XM_01151	.	.	NP_00083	XP_011514	protein_co	ENSG0000	2913	4595
GRM4	0.475271	0.524083	1.13119	0.617667	1.05915	0.642516	glutamate metabotropic receptor 4 [Source:HGNC Symbol;Acc:HG	chr6:33986	NM_00084	XM_01701	.	.	NP_00083	XP_016866	protein_co	ENSG0000	2914	4596
GRM5	0	0	0.009026	0	0.011666	0	glutamate metabotropic receptor 5 [Source:HGNC Symbol;Acc:HG	chr11:8823	NM_00084	XM_01701	.	.	NP_00083	XP_006718	protein_co	ENSG0000	2915	4597
GRM6	0	0	0	0	0	0	glutamate metabotropic receptor 6 [Source:HGNC Symbol;Acc:HG	chr5:17840	NM_00084	.	.	.	NP_00083	.	protein_co	ENSG0000	2916	4598
GRM7	0	0	0	0	0	0	glutamate metabotropic receptor 7 [Source:HGNC Symbol;Acc:HG	chr3:69028	NM_00084	.	.	.	NP_00083	.	protein_co	ENSG0000	2917	4599
GRM8	0.054586	0.075555	0.061702	0.02039	0.020519	0.073886	glutamate metabotropic receptor 8 [Source:HGNC Symbol;Acc:HG	chr7:12607	NM_00084	XM_01151	.	.	NP_00083	XP_006716	protein_co	ENSG0000	2918	4600
GRN	86.562	86.2997	98.7245	84.9645	69.9754	78.711	granulin [Source:HGNC Symbol;Acc:HGNC:4601]	chr17:4242	NM_00208	XM_00525	.	.	NP_00207	XP_005257	protein_co	ENSG0000	2896	4601
GRP	0	0.197713	0	0.111033	0	0	gastrin releasing peptide [Source:HGNC Symbol;Acc:HGNC:4605]	chr18:5688	NM_00101	XM_01702	.	.	NP_00101	XP_016881	protein_co	ENSG0000	2922	4605
GRPEL1	43.8709	44.6129	41.7598	55.8811	51.0659	42.7548	GrpE like 1, mitochondrial [Source:HGNC Symbol;Acc:HGNC:1969	chr4:70606	NM_02519	.	.	.	NP_07947	.	protein_co	ENSG0000	80273	19696
GRPEL2	17.0603	15.6193	15.8857	18.0116	20.0622	31.2076	GrpE like 2, mitochondrial [Source:HGNC Symbol;Acc:HGNC:2106	chr5:14872	NM_15240	.	.	.	NP_68962	.	protein_co	ENSG0000	134266	21060
GRPR	0.04808	0	0.165682	0.213203	0.215776	0.098321	gastrin releasing peptide receptor [Source:HGNC Symbol;Acc:HGN	chrX:16141	NM_00531	.	.	.	NP_00530	.	protein_co	ENSG0000	2925	4609
GRSF1	86.5771	85.2616	83.6751	95.7753	109.602	87.8431	G-rich RNA sequence binding factor 1 [Source:HGNC Symbol;Acc:	chr4:71681	NM_00109	XM_01153	.	.	NP_00109	XP_005265	protein_co	ENSG0000	2926	4610

GRTP1	12.7555	15.3073	16.4272	13.3102	11.1835	11.1684	growth hormone regulated TBC protein 1 [Source:HGNC Symbol;	chr13:1139	NM_00128	XM_00526	.	.	NP_001273	XP_005268	protein_co	ENSG00000	79774	20310
GRWD1	21.1082	21.8663	18.2314	26.5242	23.2201	14.8027	glutamate rich WD repeat containing 1 [Source:HGNC Symbol;Ac	chr19:4894	NM_03148	.	.	.	NP_113673	.	protein_co	ENSG00000	83743	21270
GRXCR1	0	0	0	0	0	0	glutaredoxin and cysteine rich domain containing 1 [Source:HGN	chr4:42895	NM_00108	.	.	.	NP_001073	.	protein_co	ENSG00000	389207	31673
GRXCR2	0	0	0	0	0	0	glutaredoxin and cysteine rich domain containing 2 [Source:HGN	chr5:14523	NM_00108	XM_01153	.	.	NP_001073	XP_011535	protein_co	ENSG00000	643226	33862
GSAP	0.048695	0.026112	0.127839	0.174317	0.026988	0.071197	gamma-secretase activating protein [Source:HGNC Symbol;Acc:H	chr7:76940	NM_01743	XM_01151	.	.	NP_059135	XP_011514	protein_co	ENSG00000	54103	28042
GSC	0	0	0	0	0	0	goosecoid homeobox [Source:HGNC Symbol;Acc:HGNC:4612]	chr14:9523	NM_17384	.	.	.	NP_776248	.	protein_co	ENSG00000	145258	4612
GSC2	0	0	0	0	0	0	goosecoid homeobox 2 [Source:HGNC Symbol;Acc:HGNC:4613]	chr22:1913	NM_00531	.	.	.	NP_005306	.	protein_co	ENSG00000	2928	4613
GSDMA	0	0	0	0.040798	0	0.032547	gasdermin A [Source:HGNC Symbol;Acc:HGNC:13311]	chr17:3810	NM_17817	XM_00672	.	.	NP_835465	XP_006721	protein_co	ENSG00000	284110	13311
GSDMB	5.75246	5.37409	10.7576	4.94104	5.15611	8.37869	gasdermin B [Source:HGNC Symbol;Acc:HGNC:23690]	chr17:3806	NM_00104	XM_01152	.	.	NP_001035	XP_011523	protein_co	ENSG00000	55876	23690
GSDMC	0	0.077314	0.161852	0	0.041633	0.113601	gasdermin C [Source:HGNC Symbol;Acc:HGNC:7151]	chr8:13076	NM_03141	XM_01151	.	.	NP_113603	XP_011515	protein_co	ENSG00000	56169	7151
GSDMD	48.4195	47.8638	45.1173	57.3476	45.3006	43.3054	gasdermin D [Source:HGNC Symbol;Acc:HGNC:25697]	chr8:14463	NM_00116	XM_01151	.	.	NP_001159	XP_011515	protein_co	ENSG00000	79792	25697
GSE1	22.4697	24.2373	22.0183	14.376	15.7868	19.8382	Gse1 coiled-coil protein [Source:HGNC Symbol;Acc:HGNC:28979]	chr16:8564	NM_00113	XM_01702	.	.	NP_001127	XP_005255	protein_co	ENSG00000	23199	28979
GSG1	0	0	0	0	0	0.076771	germ cell associated 1 [Source:HGNC Symbol;Acc:HGNC:19716]	chr12:1323	NM_00108	XM_01152	.	.	NP_001074	XP_005253	protein_co	ENSG00000	83445	19716
GSG1L	0.065969	0.134472	0	0	0	0	GSG1 like [Source:HGNC Symbol;Acc:HGNC:28283]	chr16:2779	NM_00110	XM_01154	.	.	NP_001103	.	protein_co	ENSG00000	146395	28283
GSG1L2	0	0	0	0	0	0	GSG1 like 2 [Source:HGNC Symbol;Acc:HGNC:51826]	chr17:9705	NM_00131	.	.	.	NP_001297	.	protein_co	ENSG00000	644070	51826
GSG2	8.64906	9.8613	8.9243	10.1287	10.8293	9.30997	germ cell associated 2, haspin [Source:HGNC Symbol;Acc:HGNC:1	chr17:3617	NM_03196	.	.	.	NP_114171	.	protein_co	ENSG00000	83903	19682
GSK3A	45.4275	43.9764	39.3456	52.7864	46.9741	37.3931	glycogen synthase kinase 3 alpha [Source:HGNC Symbol;Acc:HGN	chr19:4273	NM_01988	.	.	.	NP_063937	.	protein_co	ENSG00000	2931	4616
GSK3B	15.0449	15.4619	17.1873	14.8135	16.7516	19.497	glycogen synthase kinase 3 beta [Source:HGNC Symbol;Acc:HGN	chr3:11954	NM_00114	XM_00671	.	.	NP_001139	XP_006713	protein_co	ENSG00000	2932	4617
GSKIP	11.848	14.9587	12.6843	15.8301	13.2471	17.2551	GSK3B interacting protein [Source:HGNC Symbol;Acc:HGNC:2034	chr14:9674	NM_00127	.	.	.	NP_001258	XP_016876	protein_co	ENSG00000	51527	20343
GSN	32.2941	32.275	49.2435	30.2559	25.9859	35.4149	gelsolin [Source:HGNC Symbol;Acc:HGNC:4620]	chr9:12397	NM_00017	XM_01151	.	.	NP_000168	XP_005252	protein_co	ENSG00000	2934	4620
GSPT1	63.7575	63.7251	62.3231	71.3258	77.8165	66.3525	G1 to S phase transition 1 [Source:HGNC Symbol;Acc:HGNC:4621	chr16:1196	NM_00113	.	.	.	NP_001123	.	protein_co	ENSG00000	2935	4621
GSPT2	0.237912	0.111242	0.207009	0.179448	0.088959	0.105203	G1 to S phase transition 2 [Source:HGNC Symbol;Acc:HGNC:4622	chrX:51486	NM_01809	.	.	.	NP_060564	.	protein_co	ENSG00000	23708	4622
GSR	60.5164	64.0711	65.9126	69.8221	66.1346	63.0075	glutathione-disulfide reductase [Source:HGNC Symbol;Acc:HGNC:	chr8:30535	NM_00063	.	.	.	NP_000628	.	protein_co	ENSG00000	2936	4623
GSS	46.2565	49.1608	52.4634	61.8516	48.4877	49.3726	glutathione synthetase [Source:HGNC Symbol;Acc:HGNC:4624]	chr20:3351	NM_00017	XM_01152	.	.	NP_000169	XP_005260	protein_co	ENSG00000	2937	4624
GSTA1	0.276326	0.217669	0.241526	0.077406	0.309302	0.280133	glutathione S-transferase alpha 1 [Source:HGNC Symbol;Acc:HGN	chr6:52656	NM_14574	.	.	.	NP_665683	.	protein_co	ENSG00000	2938	4626
GSTA2	0.118564	0.189461	0.671878	0.134017	0.328943	0.294435	glutathione S-transferase alpha 2 [Source:HGNC Symbol;Acc:HGN	chr6:52614	NM_00084	XM_01151	.	.	NP_000837	XP_011512	protein_co	ENSG00000	2939	4627
GSTA3	0	0.107356	0	0	0.114112	0	glutathione S-transferase alpha 3 [Source:HGNC Symbol;Acc:HGN	chr6:52761	NM_00084	XM_00671	.	.	NP_000838	XP_006715	protein_co	ENSG00000	2940	4628
GSTA4	1.97951	1.48956	3.24234	1.9497	2.33991	2.44169	glutathione S-transferase alpha 4 [Source:HGNC Symbol;Acc:HGN	chr6:52843	NM_00151	XM_00524	.	.	NP_001503	XP_005249	protein_co	ENSG00000	2941	4629
GSTA5	0	0	0	0	0	0	glutathione S-transferase alpha 5 [Source:HGNC Symbol;Acc:HGN	chr6:52696	NM_15369	.	.	.	NP_714543	.	protein_co	ENSG00000	221357	19662
GSTCD	8.7481	9.33446	7.19655	10.7121	9.58674	8.96517	glutathione S-transferase C-terminal domain containing [Source:H	chr4:10660	NM_00103	XM_01153	.	.	NP_001026	XP_005263	protein_co	ENSG00000	79807	25806
GSTK1	66.0373	60.5761	75.3577	67.5726	50.6517	50.8224	glutathione S-transferase kappa 1 [Source:HGNC Symbol;Acc:HGN	chr7:14296	NM_00114	.	.	.	NP_001137	.	protein_co	ENSG00000	373156	16906
GSTM1	0.064259	0	0.144495	0	0	0.067212	glutathione S-transferase mu 1 [Source:HGNC Symbol;Acc:HGNC:	chr1:11019	NM_00056	XM_00527	.	.	NP_000552	XP_005270	protein_co	ENSG00000	2944	4632
GSTM2	0.271078	0.252546	0.703306	0.501862	0.381473	0.425805	glutathione S-transferase mu 2 [Source:HGNC Symbol;Acc:HGNC:	chr1:11019	NM_00084	.	.	.	NP_000839	.	protein_co	ENSG00000	2946	4634
GSTM3	0	0	0	0	0	0	glutathione S-transferase mu 3 [Source:HGNC Symbol;Acc:HGNC:	chr1:11027	NM_00084	.	.	.	NP_000840	.	protein_co	ENSG00000	2947	4635
GSTM4	9.94851	10.6815	10.8798	8.74669	8.73469	9.24266	glutathione S-transferase mu 4 [Source:HGNC Symbol;Acc:HGNC:	chr1:11019	NM_00085	.	.	.	NP_000841	.	protein_co	ENSG00000	2948	4636
GSTM5	0	0	0	0	0	0	glutathione S-transferase mu 5 [Source:HGNC Symbol;Acc:HGNC:	chr1:11025	NM_00085	XM_00527	.	.	NP_000842	XP_005270	protein_co	ENSG00000	2949	4637
GSTO1	168.104	140.918	155.866	212.12	165.554	186.381	glutathione S-transferase omega 1 [Source:HGNC Symbol;Acc:HG	chr10:1060	NM_00119	.	.	.	NP_001177	.	protein_co	ENSG00000	9446	13312
GSTO2	60.4736	61.2249	60.2142	68.3055	58.6081	58.7433	glutathione S-transferase omega 2 [Source:HGNC Symbol;Acc:HG	chr10:1060	NM_00119	XM_01153	.	.	NP_001177	XP_011537	protein_co	ENSG00000	119391	23064
GSTP1	664.744	644.696	569.503	738.53	555.23	540.632	glutathione S-transferase pi 1 [Source:HGNC Symbol;Acc:HGNC:4	chr11:6735	NM_00085	.	.	.	NP_000843	.	protein_co	ENSG00000	2950	4638
GSTT1	0	0	0	0	0	0	glutathione S-transferase theta 1 [Source:HGNC Symbol;Acc:HGN	chr22:2437	NM_00085	XM_00526	.	.	NP_000844	.	protein_co	ENSG00000	2952	4641
GSTT2B	7.35051	7.32285	11.5703	8.63066	4.49463	4.27583	glutathione S-transferase theta 2B (gene/pseudogene) [Source:H	chr22:2429	NM_00108	XM_00526	.	.	NP_001074	XP_005261	protein_co	ENSG00000	653689	33437
GSTZ1	17.826	18.4578	20.2231	22.2025	16.2106	20.6294	glutathione S-transferase zeta 1 [Source:HGNC Symbol;Acc:HGN	chr14:7774	NM_00151	XM_01153	.	.	NP_001299	XP_005267	protein_co	ENSG00000	2954	4643
GSX1	0	0	0	0	0	0	GS homeobox 1 [Source:HGNC Symbol;Acc:HGNC:20374]	chr13:2836	NM_14565	.	.	.	NP_663632	.	protein_co	ENSG00000	219409	20374
GSX2	0	0	0	0	0	0	GS homeobox 2 [Source:HGNC Symbol;Acc:HGNC:24959]	chr4:54243	NM_13326	.	.	.	NP_573574	.	protein_co	ENSG00000	170825	24959
GTDC1	4.49284	4.46903	4.84612	3.87256	3.95236	6.85806	glycosyltransferase like domain containing 1 [Source:HGNC Symb	chr2:14469	NM_00100	XM_01700	.	.	NP_001006	XP_005263	protein_co	ENSG00000	79712	20887
GTF2A1	10.3887	10.9457	9.77843	13.2492	16.7823	15.8862	general transcription factor IIA subunit 1 [Source:HGNC Symbol;A	chr14:8164	NM_00127	.	.	.	NP_001265	.	protein_co	ENSG00000	2957	4646
GTF2A1L	3.33E-20	0	0	0	0	0.078988	general transcription factor IIA subunit 1 like [Source:HGNC Symb	chr2:48757	NM_00119	.	.	.	NP_001180	.	protein_co	ENSG00000	11036	30727
GTF2A2	41.7321	36.1465	44.3666	59.9912	49.6606	54.0258	general transcription factor IIA subunit 2 [Source:HGNC Symbol;A	chr15:5993	NM_00132	XM_00525	.	.	NP_001307	XP_005254	protein_co	ENSG00000	2958	4647
GTF2B	38.4984	35.1692	33.4625	37.8114	33.883	35.3926	general transcription factor IIB [Source:HGNC Symbol;Acc:HGNC:	chr1:89318	NM_00151	.	.	.	NP_001505	XP_011539	protein_co	ENSG00000	2959	4648
GTF2E1	8.21459	9.27936	9.06471	8.73799	9.18017	9.55353	general transcription factor IIE subunit 1 [Source:HGNC Symbol;A	chr3:12046	NM_00551	XM_01151	.	.	NP_005504	XP_011511	protein_co	ENSG00000	2960	4650
GTF2E2	34.5885	30.2888	37.4799	38.1861	37.3492	43.3902	general transcription factor IIE subunit 2 [Source:HGNC Symbol;A	chr8:30435	NM_00209	.	.	.	NP_002086	XP_016868	protein_co	ENSG00000	2961	4651
GTF2F1	46.2853	47.8578	40.3147	47.6396	42.8325	34.8684	general transcription factor IIF subunit 1 [Source:HGNC Symbol;A	chr19:6379	NM_00209	.	.	.	NP_002087	.	protein_co	ENSG00000	2962	4652
GTF2F2	37.7134	34.5061	36.3358	38.9489	35.3336	37.5836	general transcription factor IIF subunit 2 [Source:HGNC Symbol;A	chr13:4569	NM_00412	XM_01153	.	.	NP_004119	XP_011533	protein_co	ENSG00000	2963	4653
GTF2H1	19.5592	19.5498	18.7182	20.5522	21.1733	19.7328	general transcription factor IIH subunit 1 [Source:HGNC Symbol;A	chr11:1834	NM_00114	XM_00671	.	.	NP_001135	XP_006718	protein_co	ENSG00000	2965	4655
GTF2H2	7.16904	7.40831	7.47447	7.7871	9.06541	6.95507	general transcription factor IIH subunit 2 [Source:HGNC Symbol;A	chr5:70328	NM_00151	XM_00524	.	.	NP_001506	XP_016864	protein_co	ENSG00000	2966	4656
GTF2H2C	15.1302	13.184	13.0154	15.2876	14.8469	13.2079	GTF2H2 family member C [Source:HGNC Symbol;Acc:HGNC:3139	chr5:68856	NM_00109	XM_01154	.	.	NP_001092	XP_005248	protein_co	ENSG00000	728340	31394
GTF2H3	35.2155	36.5321	39.0763	34.5243	41.8083	39.4506	general transcription factor IIH subunit 3 [Source:HGNC Symbol;A	chr12:1240	NM_00127	XM_01701	.	.	NP_001258	XP_016874	protein_co	ENSG00000	2967	4657
GTF2H4	33.1618	31.3349	29.7388	37.7847	31.0261	27.5421	general transcription factor IIH subunit 4 [Source:HGNC Symbol;A	chr6:30875	NM_00151	.	.	.	NP_001508	.	protein_co	ENSG00000	2968	4658
GTF2H5	4.78222	4.26022	5.82435	4.35457	4.22098	3.69734	general transcription factor IIH subunit 5 [Source:HGNC Symbol;A	chr6:15858	NM_20711	XM_01701	.	.	NP_997001	XP_016866	protein_co	ENSG00000	404672	21157

GTF2I	41.2213	38.5168	44.0441	34.9745	37.7979	36.3917	general transcription factor IIi [Source:HGNC Symbol;Acc:HGNC:4	chr7:74072	NM_00116	XM_01151	.	.	NP_00115	.	protein_co	ENSG0000	2969	4659
GTF2IRD1	8.88621	8.78265	7.82609	8.99273	8.70204	8.64078	GTF2I repeat domain containing 1 [Source:HGNC Symbol;Acc:HGI	chr7:73868	NM_00119	XM_01701	.	.	NP_00118	XP_006716	protein_co	ENSG0000	9569	4661
GTF2IRD2	0.427864	0.251954	0.372309	0.170079	0.103504	0.25209	GTF2I repeat domain containing 2 [Source:HGNC Symbol;Acc:HGI	chr7:74210	NM_00128	XM_01151	.	.	NP_00126	.	protein_co	ENSG0000	84163	30775
GTF2IRD2B	0.44663	0.285489	0.4213	0.226978	0.443811	0.429681	GTF2I repeat domain containing 2B [Source:HGNC Symbol;Acc:HC	chr7:74508	NM_00100	XM_01151	.	.	NP_00100	.	protein_co	ENSG0000	389524	33125
GTF3A	96.3119	94.5905	70.4359	119.982	99.6644	73.1338	general transcription factor IIIA [Source:HGNC Symbol;Acc:HGNC	chr13:2799	NM_00209	.	.	.	NP_00208	.	protein_co	ENSG0000	2971	4662
GTF3C1	20.8477	22.2713	20.2018	22.8105	21.964	18.8764	general transcription factor IIIC subunit 1 [Source:HGNC Symbol;A	chr16:2747	NM_00128	XM_01154	.	.	NP_00127	.	protein_co	ENSG0000	2975	4664
GTF3C2	26.1322	27.2717	25.5194	28.9169	27.6002	21.5864	general transcription factor IIIC subunit 2 [Source:HGNC Symbol;A	chr2:27548	NM_00103	XM_01153	.	.	NP_00103	XP_005264	protein_co	ENSG0000	2976	4665
GTF3C3	18.4934	18.0978	18.7078	20.0722	21.4145	20.084	general transcription factor IIIC subunit 3 [Source:HGNC Symbol;A	chr2:19762	NM_00120	.	.	.	NP_00119	.	protein_co	ENSG0000	9330	4666
GTF3C4	15.3432	16.145	16.2287	18.0357	20.9845	17.718	general transcription factor IIIC subunit 4 [Source:HGNC Symbol;A	chr9:13546	NM_01220	.	.	.	NP_03633	.	protein_co	ENSG0000	9329	4667
GTF3C5	37.1122	39.0394	32.348	50.1772	43.7011	32.6207	general transcription factor IIIC subunit 5 [Source:HGNC Symbol;A	chr9:13590	NM_00112	.	.	.	NP_00111	XP_016870	protein_co	ENSG0000	9328	4668
GTF3C6	29.1146	26.8036	21.1794	32.8192	32.0129	25.1605	general transcription factor IIIC subunit 6 [Source:HGNC Symbol;A	chr6:11127	NM_13840	.	.	.	NP_61241	.	protein_co	ENSG0000	112495	20872
GTPBP1	11.3915	11.8882	11.6081	12.2424	11.7913	11.5516	GTP binding protein 1 [Source:HGNC Symbol;Acc:HGNC:4669]	chr22:3910	NM_00428	XM_01153	.	.	NP_00427	XP_011528	protein_co	ENSG0000	9567	4669
GTPBP10	10.5251	8.88618	8.90074	8.47818	10.3594	11.2887	GTP binding protein 10 [Source:HGNC Symbol;Acc:HGNC:25106]	chr7:89975	NM_00104	.	NR_11008	.	NP_00103	.	protein_co	ENSG0000	10192744	25106
GTPBP2	22.8615	23.8032	17.3171	17.7267	16.3729	34.6707	GTP binding protein 2 [Source:HGNC Symbol;Acc:HGNC:4670]	chr6:43588	NM_00128	.	.	.	NP_00127	.	protein_co	ENSG0000	54676	4670
GTPBP3	23.5989	23.7304	23.0713	22.1347	20.0927	19.1544	GTP binding protein 3 (mitochondrial) [Source:HGNC Symbol;Acc:	chr19:1744	NM_00112	.	.	.	NP_00112	.	protein_co	ENSG0000	84705	14880
GTPBP4	30.6744	31.8402	29.2789	37.2878	36.9452	29.6969	GTP binding protein 4 [Source:HGNC Symbol;Acc:HGNC:21535]	chr10:1033	NM_01234	.	.	.	NP_03647	.	protein_co	ENSG0000	23560	21535
GTPBP6	9.27829	8.34684	8.23197	11.1215	7.90041	7.80859	GTP binding protein 6 (putative) [Source:HGNC Symbol;Acc:HGN(	chrX:22141	NM_01222	XM_01154	.	.	NP_03635	XP_006724	protein_co	ENSG0000	8225	30189
GTPBP8	6.86003	6.86659	7.85392	6.98648	10.0913	7.16414	GTP binding protein 8 (putative) [Source:HGNC Symbol;Acc:HGN(	chr3:11270	NM_01417	.	.	.	NP_05488	.	protein_co	ENSG0000	29083	25007
GTSE1	19.6306	20.6881	17.4727	29.9354	30.0706	19.8234	G2 and S-phase expressed 1 [Source:HGNC Symbol;Acc:HGNC:13	chr22:4669	NM_01642	XM_00526	.	.	NP_05751	XP_005261	protein_co	ENSG0000	51512	13698
GTSF1	156.707	143.598	121.236	206.615	185.249	197.786	gametocyte specific factor 1 [Source:HGNC Symbol;Acc:HGNC:26	chr12:5484	NM_14459	XM_01701	.	.	NP_65319	XP_016874	protein_co	ENSG0000	121355	26565
GTSF1L	0	0	0	0	0	0	gametocyte specific factor 1 like [Source:HGNC Symbol;Acc:HGN(	chr20:4235	NM_00100	XM_00526	.	.	NP_00100	XP_005260	protein_co	ENSG0000	149699	16198
GUCA1A	0	0	0	0.071815	0	0	guanylate cyclase activator 1A [Source:HGNC Symbol;Acc:HGNC:4	chr6:42123	NM_00040	XM_01151	.	.	NP_00040	XP_011512	protein_co	ENSG0000	2978	4678
GUCA1B	1.94697	2.37224	1.58935	2.23624	1.97306	2.09009	guanylate cyclase activator 1B [Source:HGNC Symbol;Acc:HGNC:4	chr6:42152	NM_00209	.	.	.	NP_00208	.	protein_co	ENSG0000	2979	4679
GUCA1C	0	0	0	0	0.095245	0	guanylate cyclase activator 1C [Source:HGNC Symbol;Acc:HGNC:4	chr3:10862	NM_00545	XM_00524	.	.	NP_00545	XP_005247	protein_co	ENSG0000	9626	4680
GUCA2A	0	0	0	0	0	0	guanylate cyclase activator 2A [Source:HGNC Symbol;Acc:HGNC:4	chr1:42628	NM_03355	.	.	.	NP_29103	.	protein_co	ENSG0000	2980	4682
GUCA2B	0	0	0	0	0	0	guanylate cyclase activator 2B [Source:HGNC Symbol;Acc:HGNC:4	chr1:42619	NM_00710	.	.	.	NP_00903	.	protein_co	ENSG0000	2981	4683
GUCD1	21.072	23.0297	21.5738	21.8997	20.5013	18.015	guanylyl cyclase domain containing 1 [Source:HGNC Symbol;Acc:I	chr22:2493	NM_00128	XM_00526	.	.	NP_00127	XP_005261	protein_co	ENSG0000	83606	14237
GUCY1A2	0.027102	0.029758	0.025678	0.013713	0.012864	0.028896	guanylate cyclase 1 soluble subunit alpha 2 [Source:HGNC Symbo	chr11:1065	NM_00085	.	.	.	NP_00084	.	protein_co	ENSG0000	2977	4684
GUCY1A3	0	0	0	0	0	0	guanylate cyclase 1 soluble subunit alpha [Source:HGNC Symbol;A	chr4:15658	NM_00085	XM_01153	.	.	NP_00084	XP_005263	protein_co	ENSG0000	2982	4685
GUCY1B3	0	0	0	0	0	0	guanylate cyclase 1 soluble subunit beta [Source:HGNC Symbol;A	chr4:15668	NM_00085	XM_01700	.	.	NP_00084	XP_011530	protein_co	ENSG0000	2983	4687
GUCY2C	0	0	0.014234	0.01845	0	0	guanylate cyclase 2C [Source:HGNC Symbol;Acc:HGNC:4688]	chr12:1476	NM_00496	XM_01152	.	.	NP_00495	XP_011518	protein_co	ENSG0000	2984	4688
GUCY2D	0.200948	0.024392	0.085549	0.053462	0.147201	0.084384	guanylate cyclase 2D, retinal [Source:HGNC Symbol;Acc:HGNC:46	chr17:7905	NM_00018	XM_01152	.	.	NP_00017	XP_011522	protein_co	ENSG0000	3000	4689
GUCY2F	0	0.0198	0.049102	0.021164	0	0.038214	guanylate cyclase 2F, retinal [Source:HGNC Symbol;Acc:HGNC:46	chrX:10861	NM_00152	.	.	.	NP_00151	.	protein_co	ENSG0000	2986	4691
GUF1	11.4306	11.316	12.7124	12.8051	16.3772	15.5011	GUF1 homolog, GTPase [Source:HGNC Symbol;Acc:HGNC:25799]	chr4:44680	NM_02192	XM_01151	.	.	NP_06874	XP_005248	protein_co	ENSG0000	60558	25799
GUK1	118.481	120.791	101.535	138.126	106.435	84.5664	guanylate kinase 1 [Source:HGNC Symbol;Acc:HGNC:4693]	chr1:22832	NM_00085	XM_01700	.	.	NP_00084	XP_005273	protein_co	ENSG0000	2987	4693
GULP1	16.3396	18.3329	13.7632	15.2731	18.5463	17.4787	GULP, engulfment adaptor PTB domain containing 1 [Source:HGN	chr2:18915	NM_00125	XM_01151	.	.	NP_00123	XP_006712	protein_co	ENSG0000	51454	18649
GUSB	15.3952	14.2265	12.1968	20.1652	16.9048	14.0183	glucuronidase beta [Source:HGNC Symbol;Acc:HGNC:4696]	chr7:65425	NM_00018	XM_01151	.	.	NP_00017	XP_005250	protein_co	ENSG0000	2990	4696
GVQW1	0.045112	0.093958	0.116953	0	0.203334	0.092224	GVQW motif containing 1 [Source:HGNC Symbol;Acc:HGNC:3142	chr9:32540	.	.	.	.	.	.	protein_co	ENSG0000	.	31424
GVQW2	0.685504	0.6242	0.68153	0.779673	1.21491	1.07595	GVQW motif containing 2 [Source:HGNC Symbol;Acc:HGNC:5171	chr6:13904	NM_00124	.	.	.	NP_00122	.	protein_co	ENSG0000	100507462	51715
GXYLT1	4.53283	5.34641	3.82095	3.33747	5.05991	5.03222	glucoside xylosyltransferase 1 [Source:HGNC Symbol;Acc:HGNC:2	chr12:4247	NM_00109	XM_01701	.	.	NP_00109	XP_016874	protein_co	ENSG0000	283464	27482
GXYLT2	1.27419	1.34566	2.15304	1.80095	2.26028	2.99803	glucoside xylosyltransferase 2 [Source:HGNC Symbol;Acc:HGNC:3	chr3:72937	NM_00108	.	.	.	NP_00107	.	protein_co	ENSG0000	727936	33383
GYG1	20.5236	20.8921	23.0239	23.5232	24.6723	23.8784	glycogenin 1 [Source:HGNC Symbol;Acc:HGNC:4699]	chr3:14870	NM_00118	.	.	.	NP_00117	.	protein_co	ENSG0000	2992	4699
GYG2	0	0	0	0	0	0	glycogenin 2 [Source:HGNC Symbol;Acc:HGNC:4700]	chrX:27468	NM_00107	XM_01702	.	.	NP_00107	XP_011543	protein_co	ENSG0000	8908	4700
GYPA	0.123489	0	0	0	0	0	glycophorin A (MNS blood group) [Source:HGNC Symbol;Acc:HGN	chr4:14503	NM_00130	XM_01700	.	.	NP_00129	XP_011530	protein_co	ENSG0000	2993	4702
GYPB	0	0	0	0	0	0	glycophorin B (MNS blood group) [Source:HGNC Symbol;Acc:HGN	chr4:14491	NM_00130	.	.	.	NP_00129	XP_011530	protein_co	ENSG0000	2994	4703
GYPC	0	0	0	0	0	0	glycophorin C (Gerbich blood group) [Source:HGNC Symbol;Acc:H	chr2:12741	NM_00125	.	.	.	NP_00124	.	protein_co	ENSG0000	2995	4704
GYPE	0	0	0	0	0	0	glycophorin E (MNS blood group) [Source:HGNC Symbol;Acc:HGN	chr4:14479	NM_00210	.	.	.	NP_00209	XP_016863	protein_co	ENSG0000	2996	4705
GYS1	22.6645	24.0021	21.0935	24.858	22.9283	16.9222	glycogen synthase 1 [Source:HGNC Symbol;Acc:HGNC:4706]	chr19:4947	NM_00116	.	.	.	NP_00115	.	protein_co	ENSG0000	2997	4706
GYS2	0.133826	0.355653	0.27256	0.126426	0.148986	0.154239	glycogen synthase 2 [Source:HGNC Symbol;Acc:HGNC:4707]	chr12:2168	NM_02195	XM_00671	.	.	NP_06877	XP_005253	protein_co	ENSG0000	2998	4707
GZF1	5.9595	6.49191	5.62194	5.39825	6.977	7.09039	GDNF inducible zinc finger protein 1 [Source:HGNC Symbol;Acc:H	chr20:2334	NM_00131	XM_01152	.	.	NP_00130	XP_005260	protein_co	ENSG0000	64412	15808
GZMA	0	0	0	0	0	0	granzyme A [Source:HGNC Symbol;Acc:HGNC:4708]	chr5:54398	NM_00614	.	.	.	NP_00613	.	protein_co	ENSG0000	3001	4708
GZMB	0	0	0	0.155824	0	0.152316	granzyme B [Source:HGNC Symbol;Acc:HGNC:4709]	chr14:2510	NM_00413	XM_01153	.	.	NP_00412	XP_011534	protein_co	ENSG0000	3002	4709
GZMH	0	0	0	0	0	0	granzyme H [Source:HGNC Symbol;Acc:HGNC:4710]	chr14:2507	NM_00127	XM_01153	.	.	NP_00125	XP_011534	protein_co	ENSG0000	2999	4710
GZMK	0	0	0	0	0	0	granzyme K [Source:HGNC Symbol;Acc:HGNC:4711]	chr5:54320	NM_00210	.	.	.	NP_00209	.	protein_co	ENSG0000	3003	4711
GZMM	0.34405	0	0.097598	0.607961	0.811428	0.519329	granzyme M [Source:HGNC Symbol;Acc:HGNC:4712]	chr19:5440	NM_00125	.	.	.	NP_00124	.	protein_co	ENSG0000	3004	4712
H1F0	283.244	289.244	393.078	360.076	359.899	498.388	H1 histone family member 0 [Source:HGNC Symbol;Acc:HGNC:47	chr22:3820	NM_00531	.	.	.	NP_00530	.	protein_co	ENSG0000	3005	4714
H1FNT	0	0	0	0	0	0	H1 histone family member N, testis specific [Source:HGNC Symbo	chr12:4872	NM_18178	.	.	.	NP_86145	.	protein_co	ENSG0000	341567	24893
H1FOO	0	0	0	0	0	0	H1 histone family member O, oocyte specific [Source:HGNC Symb	chr3:12926	NM_00130	XM_00524	.	.	NP_00129	XP_005247	protein_co	ENSG0000	132243	18463
H1FX	109.788	113.311	84.2715	137.945	119.304	165.163	H1 histone family member X [Source:HGNC Symbol;Acc:HGNC:47	chr3:12903	NM_00602	.	.	.	NP_00601	.	protein_co	ENSG0000	8971	4722

H2AFB1	0	0	0	0	0	0	H2A histone family member B1 [Source:HGNC Symbol;Acc:HGNC:	chrX:15406	NM_00101	.	.	.	NP_00101	.	protein_co	ENSG0000	474382	22516
H2AFB2	0	0	0	0	0	0	H2A histone family member B2 [Source:HGNC Symbol;Acc:HGNC:	chrX:15461	NM_00101	.	.	.	NP_00101	.	protein_co	ENSG0000	474381	18298
H2AFB3	0	0	0	0	0	0	H2A histone family member B3 [Source:HGNC Symbol;Acc:HGNC:	chrX:15468	NM_08072	.	.	.	NP_54245	.	protein_co	ENSG0000	83740	14455
H2AFJ	20.4346	19.0398	20.8387	23.5471	19.6936	22.7181	H2A histone family member J [Source:HGNC Symbol;Acc:HGNC:1	chr12:1492	NM_17792	.	.	.	NP_80876	.	protein_co	ENSG0000	55766	14456
H2AFV	134.01	135.08	112.358	152.168	138.446	170.784	H2A histone family member V [Source:HGNC Symbol;Acc:HGNC:2	chr7:44866	NM_01241	.	.	.	NP_03654	.	protein_co	ENSG0000	94239	20664
H2AFX	177.244	176.712	168.59	294.649	230.901	240.527	H2A histone family member X [Source:HGNC Symbol;Acc:HGNC:4	chr11:1189	NM_00210	.	.	.	NP_00209	.	protein_co	ENSG0000	3014	4739
H2AFY	119	129.458	108.556	144.451	126.961	104.58	H2A histone family member Y [Source:HGNC Symbol;Acc:HGNC:4	chr5:13466	NM_00104	XM_01154	.	.	NP_00103	XP_005272	protein_co	ENSG0000	9555	4740
H2AFY2	28.2814	27.7498	24.2744	37.9306	31.7379	28.1524	H2A histone family member Y2 [Source:HGNC Symbol;Acc:HGNC:	chr10:7181	NM_01864	.	.	.	NP_06111	.	protein_co	ENSG0000	55506	14453
H2AFZ	720.557	692.537	573.029	938.225	818.942	989.62	H2A histone family member Z [Source:HGNC Symbol;Acc:HGNC:4	chr4:10086	NM_00210	.	.	.	NP_00209	.	protein_co	ENSG0000	3015	4741
H2BFM	0	0	0	0	0	0	H2B histone family member M [Source:HGNC Symbol;Acc:HGNC:	chrX:10329	NM_00116	XM_01153	.	.	NP_00115	XP_006724	protein_co	ENSG0000	286436	27867
H2BFS	0	0	0	0	0	0	H2B histone family member S [Source:HGNC Symbol;Acc:HGNC:4	chr21:4494	.	XM_00672	.	.	.	XP_006723	protein_co	ENSG0000	102724334	4762
H2BFWT	0	0	0	0	0	0	H2B histone family member W, testis specific [Source:HGNC Sym	chrX:10326	NM_00100	.	.	.	NP_00100	.	protein_co	ENSG0000	158983	27252
H3F3A	96.8165	91.1094	101.53	138.811	108.726	122.414	H3 histone, family 3A [Source:HGNC Symbol;Acc:HGNC:4764]	chr1:22624	NM_00210	.	.	.	NP_00209	.	protein_co	ENSG0000	3020	4764
H3F3B	1258.52	1302.39	870.891	1761.31	1464.68	1227.68	H3 histone, family 3B [Source:HGNC Symbol;Acc:HGNC:4765]	chr17:7377	NM_00532	.	NR_03989	.	NP_00531	.	protein_co	ENSG0000	100616282	4765
H3F3C	1.16909	0.909182	0.585478	0.651614	0.662573	1.0862	H3 histone, family 3C [Source:HGNC Symbol;Acc:HGNC:33164]	chr12:3194	NM_00101	.	.	.	NP_00101	.	protein_co	ENSG0000	440093	33164
H6PD	5.37988	5.18626	8.69187	3.35415	3.52785	7.44354	hexose-6-phosphate dehydrogenase/glucose 1-dehydrogenase [S	chr1:92948	NM_00128	XM_01154	.	.	NP_00126	XP_005263	protein_co	ENSG0000	9563	4795
HAAO	0	0	0	0	0	0	3-hydroxyanthranilate 3,4-dioxygenase [Source:HGNC Symbol;Ac	chr2:42994	NM_01220	XM_01153	.	.	NP_03633	XP_005264	protein_co	ENSG0000	23498	4796
HABP2	0.043894	0.068077	0.057225	0	0	0.022849	hyaluronan binding protein 2 [Source:HGNC Symbol;Acc:HGNC:4	chr10:1153	NM_00117	.	.	.	NP_00117	.	protein_co	ENSG0000	3026	4798
HABP4	11.4723	12.756	16.3006	16.1997	13.0531	16.7824	hyaluronan binding protein 4 [Source:HGNC Symbol;Acc:HGNC:1	chr9:99212	NM_01428	.	.	.	NP_05509	.	protein_co	ENSG0000	22927	17062
HACD1	3.71792	3.53971	5.5946	1.82631	2.91674	3.52524	3-hydroxyacyl-CoA dehydratase 1 [Source:HGNC Symbol;Acc:HGN	chr10:1763	NM_01424	.	.	.	NP_05505	.	protein_co	ENSG0000	9200	9639
HACD2	27.5762	26.2197	22.813	24.0818	30.9851	30.2726	3-hydroxyacyl-CoA dehydratase 2 [Source:HGNC Symbol;Acc:HGN	chr3:12321	NM_19840	.	.	.	NP_94068	.	protein_co	ENSG0000	201562	9640
HACD3	46.9146	44.674	45.2581	51.7097	60.8323	47.9592	3-hydroxyacyl-CoA dehydratase 3 [Source:HGNC Symbol;Acc:HGN	chr15:6582	NM_01639	XM_01152	.	.	NP_05747	XP_011519	protein_co	ENSG0000	51495	24175
HACD4	0	0	0	0	0.012926	0	3-hydroxyacyl-CoA dehydratase 4 [Source:HGNC Symbol;Acc:HGN	chr9:20999	NM_00101	.	.	.	NP_00101	.	protein_co	ENSG0000	401494	20920
HACE1	4.17088	4.8091	4.08172	4.04486	4.87495	5.47199	HECT domain and ankyrin repeat containing E3 ubiquitin protein	chr6:10517	NM_00132	XM_01153	.	.	NP_00130	.	protein_co	ENSG0000	57531	21033
HACL1	9.45232	9.31929	9.62992	12.2291	10.2478	9.46332	2-hydroxyacyl-CoA lyase 1 [Source:HGNC Symbol;Acc:HGNC:1785	chr3:15602	NM_00128	.	.	.	NP_00127	.	protein_co	ENSG0000	26061	17856
HADH	19.0522	18.8787	19.8256	24.5366	22.4952	24.7285	hydroxyacyl-CoA dehydrogenase [Source:HGNC Symbol;Acc:HGN	chr4:10891	NM_00118	XM_00526	.	.	NP_00117	XP_005263	protein_co	ENSG0000	3033	4799
HADHA	57.3315	54.0971	60.6356	66.5922	56.3264	67.9125	hydroxyacyl-CoA dehydrogenase/3-ketoacyl-CoA thiolase/enoyl-	chr2:26413	NM_00018	.	.	.	NP_00017	.	protein_co	ENSG0000	3030	4801
HADHB	41.9194	40.7189	50.6124	40.8929	41.3581	50.454	hydroxyacyl-CoA dehydrogenase/3-ketoacyl-CoA thiolase/enoyl-	chr2:26467	NM_00018	XM_01153	.	.	NP_00017	XP_011531	protein_co	ENSG0000	3032	4803
HAGH	16.4376	18.5976	19.581	20.63	14.2989	17.3784	hydroxyacylglutathione hydrolase [Source:HGNC Symbol;Acc:HG	chr16:1857	NM_00104	XM_01152	.	.	NP_00103	XP_011520	protein_co	ENSG0000	3029	4805
HAGHL	27.0384	27.298	30.5471	25.9436	20.006	23.0227	hydroxyacylglutathione hydrolase-like [Source:HGNC Symbol;Acc	chr16:7769	NM_00129	XM_01152	.	.	NP_00127	XP_005255	protein_co	ENSG0000	84264	14177
HAL	0.076851	0.440413	0.281055	0.234749	0.303929	0.267712	histidine ammonia-lyase [Source:HGNC Symbol;Acc:HGNC:4806]	chr12:9636	NM_00125	XM_01153	.	.	NP_00124	XP_011536	protein_co	ENSG0000	3034	4806
HAMP	0	0	0	0	0	0	hepcidin antimicrobial peptide [Source:HGNC Symbol;Acc:HGNC:	chr19:3577	NM_02117	.	.	.	NP_06699	.	protein_co	ENSG0000	57817	15598
HAND1	0	0	0	0.223195	0.079591	0.150365	heart and neural crest derivatives expressed 1 [Source:HGNC Sym	chr5:15385	NM_00482	XM_00526	.	.	NP_00481	XP_005268	protein_co	ENSG0000	9421	4807
HAND2	0	0	0	0	0	0	heart and neural crest derivatives expressed 2 [Source:HGNC Sym	chr4:17444	NM_02197	.	.	.	NP_06880	.	protein_co	ENSG0000	9464	4808
HAO1	0	0	0	0	0	0	hydroxyacid oxidase 1 [Source:HGNC Symbol;Acc:HGNC:4809]	chr20:7863	NM_01754	.	.	.	NP_06001	.	protein_co	ENSG0000	54363	4809
HAO2	0	0	0	0	0	0	hydroxyacid oxidase 2 [Source:HGNC Symbol;Acc:HGNC:4810]	chr1:11991	NM_00100	XM_01154	.	.	NP_00100	XP_011539	protein_co	ENSG0000	51179	4810
HAP1	0.764604	0.843049	1.61037	0.839341	0.710076	1.02591	huntingtin associated protein 1 [Source:HGNC Symbol;Acc:HGNC:	chr17:3987	NM_00107	.	.	.	NP_00107	.	protein_co	ENSG0000	9001	4812
HAPLN1	0	0.017134	0	0.018235	0	0	hyaluronan and proteoglycan link protein 1 [Source:HGNC Symbo	chr5:82933	NM_00188	XM_01154	.	.	NP_00187	XP_011541	protein_co	ENSG0000	1404	2380
HAPLN2	0.062028	0	0.105761	0	0	0.069912	hyaluronan and proteoglycan link protein 2 [Source:HGNC Symbo	chr1:15658	NM_02181	XM_01150	.	.	NP_06858	XP_005245	protein_co	ENSG0000	60484	17410
HAPLN3	7.81701	7.08153	12.8936	9.21656	6.66667	9.14428	hyaluronan and proteoglycan link protein 3 [Source:HGNC Symbo	chr15:8942	NM_00130	XM_01152	.	.	NP_00129	XP_011519	protein_co	ENSG0000	145864	21446
HAPLN4	0.026314	0	0.022489	0	0.030477	0	hyaluronan and proteoglycan link protein 4 [Source:HGNC Symbo	chr19:1936	NM_02300	.	.	.	NP_07537	.	protein_co	ENSG0000	404037	31357
HARBI1	2.07963	2.43714	2.00022	2.60608	2.12184	1.60713	harbinger transposase derived 1 [Source:HGNC Symbol;Acc:HGN	chr11:4662	NM_17381	.	.	.	NP_77617	XP_016873	protein_co	ENSG0000	283254	26522
HARS	59.6884	58.3026	56.0845	67.7571	63.8598	47.8097	histidyl-tRNA synthetase [Source:HGNC Symbol;Acc:HGNC:4816]	chr5:14004	NM_00125	.	.	.	NP_00124	.	protein_co	ENSG0000	3035	4816
HARS2	38.3946	37.222	32.9593	45.7714	41.5072	27.7831	histidyl-tRNA synthetase 2, mitochondrial [Source:HGNC Symbol;	chr5:14004	NM_00127	XM_01153	.	.	NP_00126	XP_011535	protein_co	ENSG0000	23438	4817
HAS1	0	0	0	0	0	0	hyaluronan synthase 1 [Source:HGNC Symbol;Acc:HGNC:4818]	chr19:5221	NM_00129	.	.	.	NP_00128	.	protein_co	ENSG0000	3036	4818
HAS2	0.098808	0.128187	0.102856	0.022684	0.108945	0.018999	hyaluronan synthase 2 [Source:HGNC Symbol;Acc:HGNC:4819]	chr8:12262	NM_00532	XM_01151	.	.	NP_00531	XP_011515	protein_co	ENSG0000	3037	4819
HAS3	26.1932	27.4787	49.4112	47.987	48.1547	33.2745	hyaluronan synthase 3 [Source:HGNC Symbol;Acc:HGNC:4820]	chr16:6913	NM_00119	XM_01152	.	.	NP_00118	XP_005255	protein_co	ENSG0000	3038	4820
HAT1	57.9677	48.807	57.2757	56.0941	76.2598	81.2424	histone acetyltransferase 1 [Source:HGNC Symbol;Acc:HGNC:482	chr2:17277	NM_00364	XM_01151	.	.	NP_00363	XP_006712	protein_co	ENSG0000	8520	4821
HAUS1	34.2484	29.8885	26.62	40.6663	39.6796	37.0204	HAUS augmin like complex subunit 1 [Source:HGNC Symbol;Acc:H	chr18:4368	NM_13844	.	.	.	NP_61245	.	protein_co	ENSG0000	115106	25174
HAUS2	18.3211	18.8651	17.2289	17.3175	22.6894	16.812	HAUS augmin like complex subunit 2 [Source:HGNC Symbol;Acc:H	chr15:4284	NM_00113	XM_01702	.	.	NP_00112	XP_016877	protein_co	ENSG0000	55142	25530
HAUS3	9.80125	9.72108	9.80029	7.83394	12.3052	8.96815	HAUS augmin like complex subunit 3 [Source:HGNC Symbol;Acc:H	chr4:20736	NM_00130	.	.	.	NP_00129	.	protein_co	ENSG0000	79441	28719
HAUS4	22.9818	25.7589	22.945	25.0115	21.9732	22.498	HAUS augmin like complex subunit 4 [Source:HGNC Symbol;Acc:H	chr14:2341	NM_00116	.	NR_03985	.	NP_00115	.	protein_co	ENSG0000	100616424	20163
HAUS5	9.69279	10.9757	9.55065	10.1367	9.53355	8.76202	HAUS augmin like complex subunit 5 [Source:HGNC Symbol;Acc:H	chr19:3610	NM_01530	XM_00672	.	.	NP_05611	.	protein_co	ENSG0000	23354	29130
HAUS6	11.8341	11.3495	12.1056	13.5795	18.9271	16.354	HAUS augmin like complex subunit 6 [Source:HGNC Symbol;Acc:H	chr9:19053	NM_00127	.	.	.	NP_00125	.	protein_co	ENSG0000	54801	25948
HAUS7	7.77289	9.15144	7.2034	10.1502	10.6838	5.66484	HAUS augmin like complex subunit 7 [Source:HGNC Symbol;Acc:H	chrX:15271	NM_01751	.	.	.	NP_05998	.	protein_co	ENSG0000	55559	32979
HAUS8	11.5711	10.0908	8.93391	14.2328	11.145	9.35731	HAUS augmin like complex subunit 8 [Source:HGNC Symbol;Acc:H	chr19:1716	NM_00101	XM_01152	.	.	NP_00101	XP_005260	protein_co	ENSG0000	93323	30532
HAVCR1	0	0	0	0	0	0	hepatitis A virus cellular receptor 1 [Source:HGNC Symbol;Acc:HG	chr5:15645	NM_00109	XM_01153	.	.	NP_00109	XP_006714	protein_co	ENSG0000	26762	17866
HAVCR2	0.050063	0	0.021742	0.028067	0.084022	0.085534	hepatitis A virus cellular receptor 2 [Source:HGNC Symbol;Acc:HG	chr5:15651	NM_03278	.	.	.	NP_11617	.	protein_co	ENSG0000	84868	18437
HAX1	69.2921	64.0306	48.992	73.8429	53.4672	87.7531	HCLS1 associated protein X-1 [Source:HGNC Symbol;Acc:HGNC:1	chr1:15424	NM_00101	.	.	.	NP_00101	.	protein_co	ENSG0000	10456	16915

HBA1	0	0.727785	0.420737	0.265475	1.14917	0	hemoglobin subunit alpha 1 [Source:HGNC Symbol;Acc:HGNC:482	chr16:2266	NM_00055	.	.	.	NP_000549	.	protein_co	ENSG0000	3039	4823
HBA2	0.716018	0.704444	0.81502	1.53756	0.834412	2.10503	hemoglobin subunit alpha 2 [Source:HGNC Symbol;Acc:HGNC:482	chr16:2228	NM_00051	.	.	.	NP_000508	.	protein_co	ENSG0000	3040	4824
HBB	0	0	0	0.139368	0.147107	0	hemoglobin subunit beta [Source:HGNC Symbol;Acc:HGNC:4827]	chr11:5246	NM_00051	.	.	.	NP_000509	.	protein_co	ENSG0000	3043	4827
HBCBP	0	0	0	0	0	0	HBcAg-binding protein; Uncharacterized protein [Source:UniProt	chr12:5738	.	.	.	.	.	.	protein_co	ENSG0000	.	.
HBD	0.095966	0	0	0	0	0	hemoglobin subunit delta [Source:HGNC Symbol;Acc:HGNC:4829]	chr11:5253	NM_00051	.	.	.	NP_000510	.	protein_co	ENSG0000	3045	4829
HBE1	80.5405	75.5944	94.0482	38.43	30.1032	45.8544	hemoglobin subunit epsilon 1 [Source:HGNC Symbol;Acc:HGNC:4	chr11:5269	NM_00533	XM_01152	.	.	NP_005321	XP_011518	protein_co	ENSG0000	3046	4830
HBEGF	4.99445	5.07752	5.02295	6.09038	4.80931	10.1707	heparin binding EGF like growth factor [Source:HGNC Symbol;Acc	chr5:13971	NM_00194	.	.	.	NP_001936	.	protein_co	ENSG0000	1839	3059
HBG1	0	0	0.163583	0	0	0	hemoglobin subunit gamma 1 [Source:HGNC Symbol;Acc:HGNC:4	chr11:5269	NM_00055	.	.	.	NP_000550	.	protein_co	ENSG0000	3047	4831
HBG2	11.323	12.5143	13.2212	5.54001	4.98057	6.38271	hemoglobin subunit gamma 2 [Source:HGNC Symbol;Acc:HGNC:4	chr11:5269	NM_00018	.	.	.	NP_000175	.	protein_co	ENSG0000	3048	4832
HBM	0	0	0	0	0	0	hemoglobin subunit mu [Source:HGNC Symbol;Acc:HGNC:4826]	chr16:2159	NM_00100	.	.	.	NP_001003	.	protein_co	ENSG0000	3042	4826
HBP1	8.85076	9.00043	10.2012	6.09563	6.1782	10.2301	HMG-box transcription factor 1 [Source:HGNC Symbol;Acc:HGNC	chr7:10680	NM_00124	XM_00525	.	.	NP_001231	XP_005250	protein_co	ENSG0000	26959	23200
HBQ1	0	0	0	0	0	0	hemoglobin subunit theta 1 [Source:HGNC Symbol;Acc:HGNC:483	chr16:2304	NM_00533	.	.	.	NP_005322	.	protein_co	ENSG0000	3049	4833
HBS1L	37.9657	33.7391	38.1781	43.5358	49.9203	50.5286	HBS1 like translational GTPase [Source:HGNC Symbol;Acc:HGNC:4	chr6:13528	NM_00114	.	.	.	NP_001138	.	protein_co	ENSG0000	10767	4834
HBZ	0	0.401589	0	0.292755	0	0.151863	hemoglobin subunit zeta [Source:HGNC Symbol;Acc:HGNC:4835]	chr16:2026	NM_00533	XM_01152	.	.	NP_005323	XP_005255	protein_co	ENSG0000	3050	4835
HCAR1	0	0	0	0	0	0	hydroxycarboxylic acid receptor 1 [Source:HGNC Symbol;Acc:HGN	chr12:1232	NM_03255	.	.	.	NP_115943	.	protein_co	ENSG0000	27198	4532
HCAR2	0	0	0	0.04158	0	0	hydroxycarboxylic acid receptor 2 [Source:HGNC Symbol;Acc:HGN	chr12:1231	NM_17755	.	.	.	NP_808219	.	protein_co	ENSG0000	338442	24827
HCAR3	0	0	0	0	0	0	hydroxycarboxylic acid receptor 3 [Source:HGNC Symbol;Acc:HGN	chr12:1231	NM_00601	.	.	.	NP_006009	.	protein_co	ENSG0000	8843	16824
HCCS	26.62	25.1897	24.7215	33.1252	32.4908	24.6513	holocytochrome c synthase [Source:HGNC Symbol;Acc:HGNC:483	chrX:11129	NM_00112	.	.	.	NP_001116	.	protein_co	ENSG0000	3052	4837
HCFC1	16.2183	17.3501	13.7715	17.115	17.2627	12.9482	host cell factor C1 [Source:HGNC Symbol;Acc:HGNC:4839]	chrX:15321	NM_00533	XM_01153	.	.	NP_005325	XP_006724	protein_co	ENSG0000	3054	4839
HCFC1R1	43.7007	39.8844	29.9616	55.6968	43.6744	39.7216	host cell factor C1 regulator 1 [Source:HGNC Symbol;Acc:HGNC:2	chr16:3072	NM_00100	XM_01152	.	.	NP_001002	XP_011520	protein_co	ENSG0000	54985	21198
HCFC2	1.52529	1.95132	1.86064	1.35761	1.82368	1.69501	host cell factor C2 [Source:HGNC Symbol;Acc:HGNC:24972]	chr12:1044	NM_01332	XM_01153	.	.	NP_037452	.	protein_co	ENSG0000	29915	24972
HCG27	0.400697	0.367165	0.542429	0.343886	0.402587	0.509073	HLA complex group 27 (non-protein coding) [Source:HGNC Symbo	chr6:31165	.	.	.	.	.	.	protein_co	ENSG0000	253018	27366
HCK	0	0.059661	0.052071	0	0	0.076318	HCK proto-oncogene, Src family tyrosine kinase [Source:HGNC Sy	chr20:3063	NM_00117	.	.	.	NP_001165	.	protein_co	ENSG0000	3055	4840
HCLS1	0.233386	0.234495	0.159703	0.031709	0.083747	0.094098	hematopoietic cell-specific Lyn substrate 1 [Source:HGNC Symbol	chr3:12135	NM_00129	.	.	.	NP_001278	.	protein_co	ENSG0000	3059	4844
HCN1	0	0.019588	0.055381	0.031375	0.010069	0.053075	hyperpolarization activated cyclic nucleotide gated potassium cha	chr5:45255	NM_02107	.	.	.	NP_066550	.	protein_co	ENSG0000	348980	4845
HCN2	0.777558	0.86848	1.56205	0.79432	0.476871	0.45712	hyperpolarization activated cyclic nucleotide gated potassium cha	chr19:5898	NM_00119	.	.	.	NP_001185	.	protein_co	ENSG0000	610	4846
HCN3	2.80651	2.85	3.47489	1.84402	2.12136	1.34638	hyperpolarization activated cyclic nucleotide gated potassium cha	chr1:15524	NM_02089	XM_01150	.	.	NP_065948	XP_011508	protein_co	ENSG0000	57657	19183
HCN4	0.548255	0.903735	1.04562	0.707623	0.831838	1.01126	hyperpolarization activated cyclic nucleotide gated potassium cha	chr15:7361	NM_00547	.	.	.	NP_005468	.	protein_co	ENSG0000	10021	16882
HCRT	0	0	0	0	0	0	hypocretin neuropeptide precursor [Source:HGNC Symbol;Acc:HG	chr17:4033	NM_00152	.	.	.	NP_001515	.	protein_co	ENSG0000	3060	4847
HCRTR1	0	0	0	0	0	0	hypocretin receptor 1 [Source:HGNC Symbol;Acc:HGNC:4848]	chr1:32083	NM_00152	XM_01154	.	.	NP_001516	XP_011539	protein_co	ENSG0000	3061	4848
HCRTR2	0	0	0	0	0	0	hypocretin receptor 2 [Source:HGNC Symbol;Acc:HGNC:4849]	chr6:54971	NM_00152	.	.	.	NP_001517	XP_016866	protein_co	ENSG0000	3062	4849
HCST	0.732745	0.917737	2.15728	1.00496	0.625763	1.30515	hematopoietic cell signal transducer [Source:HGNC Symbol;Acc:H	chr19:3639	NM_00100	.	.	.	NP_001007	.	protein_co	ENSG0000	10870	16977
HDAC1	129.587	125.689	140.379	144.39	131.469	150.977	histone deacetylase 1 [Source:HGNC Symbol;Acc:HGNC:4852]	chr1:32757	NM_00496	.	.	.	NP_004955	.	protein_co	ENSG0000	3065	4852
HDAC10	9.57938	8.63623	11.2331	7.24475	7.10822	6.4614	histone deacetylase 10 [Source:HGNC Symbol;Acc:HGNC:18128]	chr22:5068	NM_00115	.	.	.	NP_001152	.	protein_co	ENSG0000	83933	18128
HDAC11	7.40558	6.81944	8.3358	8.83792	6.86683	6.72843	histone deacetylase 11 [Source:HGNC Symbol;Acc:HGNC:19086]	chr3:13521	NM_00113	XM_01153	.	.	NP_001129	XP_005265	protein_co	ENSG0000	79885	19086
HDAC2	80.7451	79.3937	75.0113	100.127	102.322	100.493	histone deacetylase 2 [Source:HGNC Symbol;Acc:HGNC:4853]	chr6:11425	NM_00152	XM_01153	.	.	NP_001518	XP_011534	protein_co	ENSG0000	3066	4853
HDAC3	40.7929	46.7323	47.5816	54.3857	44.4293	41.3556	histone deacetylase 3 [Source:HGNC Symbol;Acc:HGNC:4854]	chr5:14100	NM_00388	XM_01153	.	.	NP_003874	XP_011535	protein_co	ENSG0000	8841	4854
HDAC4	3.18959	3.166	4.30074	2.69669	3.00377	5.84999	histone deacetylase 4 [Source:HGNC Symbol;Acc:HGNC:14063]	chr2:23996	NM_00603	XM_01151	.	.	NP_006028	XP_006712	protein_co	ENSG0000	9759	14063
HDAC5	12.5816	13.304	11.8033	11.8807	10.5296	13.2325	histone deacetylase 5 [Source:HGNC Symbol;Acc:HGNC:14068]	chr17:4215	NM_00101	XM_01702	.	.	NP_001015	XP_005256	protein_co	ENSG0000	10014	14068
HDAC6	10.3547	10.3149	11.4371	12.6833	11.0086	10.2223	histone deacetylase 6 [Source:HGNC Symbol;Acc:HGNC:14064]	chrX:48660	NM_00132	XM_00672	.	.	NP_001308	XP_005272	protein_co	ENSG0000	10013	14064
HDAC7	10.8575	10.2264	9.1758	10.3211	10.7543	7.5532	histone deacetylase 7 [Source:HGNC Symbol;Acc:HGNC:14067]	chr12:4812	NM_00109	XM_01701	.	.	NP_001091	XP_005269	protein_co	ENSG0000	51564	14067
HDAC8	9.25077	8.22693	9.53491	11.2742	9.10886	9.31647	histone deacetylase 8 [Source:HGNC Symbol;Acc:HGNC:13315]	chrX:71549	NM_00116	XM_01153	.	.	NP_001159	XP_011529	protein_co	ENSG0000	55869	13315
HDAC9	3.43102	3.8194	1.80035	1.1611	1.22844	1.39514	histone deacetylase 9 [Source:HGNC Symbol;Acc:HGNC:14065]	chr7:18126	NM_00120	XM_01701	.	.	NP_001191	XP_011513	protein_co	ENSG0000	9734	14065
HDC	0	0	0	0	0	0	histidine decarboxylase [Source:HGNC Symbol;Acc:HGNC:4855]	chr15:5053	NM_00130	XM_01152	.	.	NP_001293	XP_011519	protein_co	ENSG0000	3067	4855
HDDC2	61.9604	60.2633	55.9765	90.3524	76.0325	76.4519	HD domain containing 2 [Source:HGNC Symbol;Acc:HGNC:21078]	chr6:12559	NM_01606	.	.	.	NP_057147	.	protein_co	ENSG0000	51020	21078
HDDC3	15.8672	13.2985	16.212	15.3224	9.65659	9.08724	HD domain containing 3 [Source:HGNC Symbol;Acc:HGNC:30522]	chr15:9147	NM_00128	.	.	.	NP_001273	.	protein_co	ENSG0000	374659	30522
HDGF	208.538	204.265	147.018	244.911	210.18	152.23	hepatoma-derived growth factor [Source:HGNC Symbol;Acc:HGN	chr1:15671	NM_00112	.	.	.	NP_001119	.	protein_co	ENSG0000	3068	4856
HDGFL1	0	0	0	0	0	0	hepatoma derived growth factor-like 1 [Source:HGNC Symbol;Acc	chr6:22569	NM_13857	.	.	.	NP_612641	.	protein_co	ENSG0000	154150	21095
HDHD2	11.5266	10.3327	12.0409	10.9727	12.7345	14.1029	haloacid dehalogenase like hydrolase domain containing 2 [Sourc	chr18:4463	NM_00131	XM_01152	.	.	NP_001305	XP_006722	protein_co	ENSG0000	84064	25364
HDHD3	15.4654	14.3242	16.6647	16.4243	15.4122	12.4229	haloacid dehalogenase like hydrolase domain containing 3 [Sourc	chr9:11613	NM_00130	.	.	.	NP_001291	.	protein_co	ENSG0000	81932	28171
HDLBP	101.625	107.91	98.4023	83.0141	78.6355	78.4324	high density lipoprotein binding protein [Source:HGNC Symbol;Ac	chr2:24216	NM_00124	XM_01700	.	.	NP_001230	XP_005247	protein_co	ENSG0000	3069	4857
HDX	0	0	0	0	0	0	highly divergent homeobox [Source:HGNC Symbol;Acc:HGNC:264	chrX:83572	NM_00117	XM_01153	.	.	NP_001170	XP_006724	protein_co	ENSG0000	139324	26411
HEATR1	19.9376	19.1946	20.8718	19.6896	23.2082	18.8976	HEAT repeat containing 1 [Source:HGNC Symbol;Acc:HGNC:25517	chr1:23668	NM_01807	XM_01154	.	.	NP_060542	XP_011542	protein_co	ENSG0000	55127	25517
HEATR3	6.81248	8.0034	8.60725	8.56829	10.4266	8.87231	HEAT repeat containing 3 [Source:HGNC Symbol;Acc:HGNC:26087	chr16:5009	NM_18292	XM_01152	.	.	NP_891552	XP_005256	protein_co	ENSG0000	55027	26087
HEATR4	0	0	0	0	0	0	HEAT repeat containing 4 [Source:HGNC Symbol;Acc:HGNC:16761	chr14:7394	NM_00122	XM_01153	.	.	NP_001207	XP_006720	protein_co	ENSG0000	399671	16761
HEATR5A	8.23789	7.8033	11.6744	9.67221	8.63973	9.42534	HEAT repeat containing 5A [Source:HGNC Symbol;Acc:HGNC:2027	chr14:3176	NM_01547	.	.	.	NP_056288	.	protein_co	ENSG0000	25938	20276
HEATR5B	5.26323	5.8408	5.8272	3.6157	4.45903	4.51158	HEAT repeat containing 5B [Source:HGNC Symbol;Acc:HGNC:2927	chr2:37208	NM_01902	XM_01700	.	.	NP_061897	XP_006712	protein_co	ENSG0000	54497	29273
HEATR6	11.4247	12.3324	11.2799	10.2498	11.3805	12.8682	HEAT repeat containing 6 [Source:HGNC Symbol;Acc:HGNC:24076	chr17:5812	NM_02207	XM_01152	.	.	NP_071353	XP_005257	protein_co	ENSG0000	63897	24076
HEATR9	0	0	0	0	0	0	HEAT repeat containing 9 [Source:HGNC Symbol;Acc:HGNC:26548	chr17:3418	NM_00132	XM_01152	.	.	NP_001308	XP_005278	protein_co	ENSG0000	256957	26548

HEBP1	19.5365	22.9508	25.207	24.3041	19.7949	24.3717	heme binding protein 1 [Source:HGNC Symbol;Acc:HGNC:17176]	chr12:1312	NM_01598	.	.	.	NP_05707	.	protein_co	ENSG0000	50865	17176
HEBP2	23.9247	23.9778	24.0389	27.3203	25.7863	27.6601	heme binding protein 2 [Source:HGNC Symbol;Acc:HGNC:15716]	chr6:13872	NM_01432	.	.	.	NP_00131	XP_016866	protein_co	ENSG0000	23593	15716
HECA	3.79133	3.86603	4.5074	2.84412	4.28818	5.83769	hdc homolog, cell cycle regulator [Source:HGNC Symbol;Acc:HGN	chr6:13945	NM_01621	.	.	.	NP_05730	.	protein_co	ENSG0000	51696	21041
HECTD1	22.3089	22.9684	21.5769	19.656	24.9739	24.6704	HECT domain E3 ubiquitin protein ligase 1 [Source:HGNC Symbol;	chr14:3156	NM_01538	XM_01153	.	.	NP_05619	XP_005267	protein_co	ENSG0000	25831	20157
HECTD2	2.25333	2.17251	1.59165	1.71938	2.3705	2.16914	HECT domain E3 ubiquitin protein ligase 2 [Source:HGNC Symbol;	chr10:9317	NM_00128	XM_01701	.	.	NP_00127	XP_016871	protein_co	ENSG0000	143279	26736
HECTD3	13.251	13.2225	14.2959	7.36139	6.73094	6.17846	HECT domain E3 ubiquitin protein ligase 3 [Source:HGNC Symbol;	chr1:45468	NM_02460	XM_01154	.	.	NP_07887	XP_011540	protein_co	ENSG0000	79654	26117
HECTD4	3.26962	3.60735	4.34831	2.52172	3.00214	2.81939	HECT domain E3 ubiquitin protein ligase 4 [Source:HGNC Symbol;	chr12:1125	NM_00110	.	NR_10692	.	NP_00110	.	protein_co	ENSG0000	102465519	26611
HECW1	1.23511	1.31644	1.20804	0.554444	0.947983	0.814965	HECT, C2 and WW domain containing E3 ubiquitin protein ligase	chr7:43152	NM_00128	XM_01701	.	.	NP_00127	XP_005249	protein_co	ENSG0000	23072	22195
HECW2	0.412621	0.329933	0.429597	0.381893	0.367526	0.356643	HECT, C2 and WW domain containing E3 ubiquitin protein ligase	chr2:19705	NM_00130	XM_01700	.	.	NP_00129	XP_006712	protein_co	ENSG0000	57520	29853
HEG1	1.14039	1.05289	1.24188	0.836439	0.989436	1.20161	heart development protein with EGF like domains 1 [Source:HGN	chr3:12468	NM_02073	XM_00524	.	.	NP_06578	XP_005247	protein_co	ENSG0000	57493	29227
HELB	1.53837	1.86373	1.83966	1.25433	1.51157	1.86096	DNA helicase B [Source:HGNC Symbol;Acc:HGNC:17196]	chr12:6669	NM_03364	XM_00526	.	.	NP_38746	XP_005269	protein_co	ENSG0000	92797	17196
HELLS	17.9905	15.3355	17.2709	14.1459	15.2444	14.2757	helicase, lymphoid-specific [Source:HGNC Symbol;Acc:HGNC:486	chr10:9616	NM_00128	.	.	.	NP_00127	.	protein_co	ENSG0000	3070	4861
HELQ	3.44405	2.71132	3.2893	3.13676	3.74468	3.54634	helicase, POLQ-like [Source:HGNC Symbol;Acc:HGNC:18536]	chr4:84328	NM_00129	XM_01153	.	.	NP_00128	XP_005262	protein_co	ENSG0000	113510	18536
HELT	0	0	0	0	0	0	helt bHLH transcription factor [Source:HGNC Symbol;Acc:HGNC:3	chr4:18593	NM_00130	.	.	.	NP_00128	.	protein_co	ENSG0000	391723	33783
HELZ	7.35265	6.69626	8.43678	5.83768	7.6617	7.17111	helicase with zinc finger [Source:HGNC Symbol;Acc:HGNC:16878]	chr17:6506	NM_01487	XM_01702	.	.	NP_05569	XP_005257	protein_co	ENSG0000	9931	16878
HELZ2	7.82129	8.96287	8.36224	7.04649	6.95341	8.94124	helicase with zinc finger 2 [Source:HGNC Symbol;Acc:HGNC:3002	chr20:6218	NM_00103	XM_01152	.	.	NP_00103	XP_011527	protein_co	ENSG0000	85441	30021
HEMGN	0	0	0	0	0	0	hemogen [Source:HGNC Symbol;Acc:HGNC:17509]	chr9:10068	NM_01843	XM_01151	.	.	NP_06090	XP_005252	protein_co	ENSG0000	55363	17509
HEMK1	9.10371	8.41956	9.32013	5.89117	5.11403	6.64121	HemK methyltransferase family member 1 [Source:HGNC Symbol	chr3:50595	NM_01617	XM_01153	.	.	NP_00130	XP_011532	protein_co	ENSG0000	51409	24923
HENMT1	0	0	0.040637	0	0	0	HEN1 methyltransferase homolog 1 [Source:HGNC Symbol;Acc:H	chr1:10919	NM_00110	XM_00527	.	.	NP_00109	XP_005270	protein_co	ENSG0000	113802	26400
HEPACAM	0	0	0	0	0	0	hepatic and glial cell adhesion molecule [Source:HGNC Symbol;A	chr11:1247	NM_15272	XM_01154	.	.	NP_68993	XP_005271	protein_co	ENSG0000	220296	26361
HEPACAM2	0	0.040699	0.033449	0	0.04313	0.038627	HEPACAM family member 2 [Source:HGNC Symbol;Acc:HGNC:27	chr7:92817	NM_00103	XM_01151	.	.	NP_00103	XP_011514	protein_co	ENSG0000	253012	27364
HEPH	0.286301	0.511467	0.31476	0.146897	0.094657	0.094033	hephaestin [Source:HGNC Symbol;Acc:HGNC:4866]	chrX:65382	NM_00113	XM_01153	.	.	NP_00112	XP_006724	protein_co	ENSG0000	9843	4866
HEPHL1	0	0.013271	0.021878	0.014172	0	0	hephaestin like 1 [Source:HGNC Symbol;Acc:HGNC:30477]	chr11:9375	NM_00109	.	.	.	NP_00109	.	protein_co	ENSG0000	341208	30477
HEPN1	0	0	0	0	0	0	hepatocellular carcinoma, down-regulated 1 [Source:HGNC Symb	chr11:1247	NM_00103	.	.	.	NP_00103	.	protein_co	ENSG0000	641654	34400
HERC1	2.9926	3.22235	3.58161	2.4758	2.87364	2.81234	HECT and RLD domain containing E3 ubiquitin protein ligase fami	chr15:6390	NM_00392	XM_01152	.	.	NP_00391	XP_016878	protein_co	ENSG0000	8925	4867
HERC2	10.2205	10.0997	10.2963	10.111	9.81637	8.73512	HECT and RLD domain containing E3 ubiquitin protein ligase 2 [So	chr15:2835	NM_00466	XM_01702	.	.	NP_00465	XP_005268	protein_co	ENSG0000	8924	4868
HERC3	1.91807	2.43425	3.01529	2.05548	3.33059	3.77026	HECT and RLD domain containing E3 ubiquitin protein ligase 3 [So	chr4:89513	NM_00127	XM_00526	.	.	NP_00125	XP_005263	protein_co	ENSG0000	8916	4876
HERC4	9.24355	9.75808	11.3878	9.50382	10.4215	10.9985	HECT and RLD domain containing E3 ubiquitin protein ligase 4 [So	chr10:6968	NM_00127	XM_01153	.	.	NP_00126	XP_011537	protein_co	ENSG0000	26091	24521
HERC5	18.4429	17.6658	5.96995	14.1959	16.7561	19.664	HECT and RLD domain containing E3 ubiquitin protein ligase 5 [So	chr4:89378	NM_01632	XM_01153	.	.	NP_05740	XP_011530	protein_co	ENSG0000	51191	24368
HERC6	4.88482	5.48106	3.96682	3.59798	4.52526	3.38527	HECT and RLD domain containing E3 ubiquitin protein ligase fami	chr4:89299	NM_00116	XM_01153	.	.	NP_00115	XP_005263	protein_co	ENSG0000	55008	26072
HERPUD1	84.9197	85.5703	27.0849	57.0443	50.735	74.854	homocysteine inducible ER protein with ubiquitin like domain 1 [	chr16:5696	NM_00101	XM_00672	.	.	NP_00101	.	protein_co	ENSG0000	9709	13744
HERPUD2	8.59639	8.21649	9.9102	7.63288	6.91665	10.4807	HERPUD family member 2 [Source:HGNC Symbol;Acc:HGNC:2191	chr7:35672	NM_02237	XM_01701	.	.	NP_07176	XP_016867	protein_co	ENSG0000	64224	21915
HES1	9.47672	9.13381	17.712	14.5369	13.7839	23.6903	hes family bHLH transcription factor 1 [Source:HGNC Symbol;Acc	chr3:19385	NM_00552	.	.	.	NP_00551	.	protein_co	ENSG0000	3280	5192
HES2	9.15189	9.82303	10.6041	9.2714	8.17937	10.5234	hes family bHLH transcription factor 2 [Source:HGNC Symbol;Acc	chr1:64724	NM_01908	XM_01154	.	.	NP_06196	XP_011539	protein_co	ENSG0000	54626	16005
HES3	0	0	0	0	0	0	hes family bHLH transcription factor 3 [Source:HGNC Symbol;Acc	chr1:63042	NM_00102	XM_01154	.	.	NP_00101	.	protein_co	ENSG0000	390992	26226
HES4	55.8024	67.2033	52.2253	64.5858	44.5724	59.3144	hes family bHLH transcription factor 4 [Source:HGNC Symbol;Acc	chr1:93434	NM_00114	XM_00524	.	.	NP_00113	XP_005244	protein_co	ENSG0000	57801	24149
HES5	0	0	0	0	0	0.10985	hes family bHLH transcription factor 5 [Source:HGNC Symbol;Acc	chr1:24601	NM_00101	XM_00524	.	.	NP_00101	XP_005244	protein_co	ENSG0000	388585	19764
HES6	46.2597	48.7871	60.5482	61.9958	51.1198	47.298	hes family bHLH transcription factor 6 [Source:HGNC Symbol;Acc	chr2:23914	NM_00114	.	.	.	NP_00113	.	protein_co	ENSG0000	55502	18254
HES7	0.0708	0.069305	0.060701	0	0.527758	0.256048	hes family bHLH transcription factor 7 [Source:HGNC Symbol;Acc	chr17:8023	NM_00116	XM_01152	.	.	NP_00115	.	protein_co	ENSG0000	84667	15977
HESX1	1.46414	1.61995	0.958967	1.15442	1.68271	1.42099	HESX homeobox 1 [Source:HGNC Symbol;Acc:HGNC:4877]	chr3:57231	NM_00386	XM_01153	.	.	NP_00385	XP_005265	protein_co	ENSG0000	8820	4877
HEXA	25.2835	28.9908	23.9372	19.3091	16.6878	17.6845	hexosaminidase subunit alpha [Source:HGNC Symbol;Acc:HGNC:4	chr15:7263	NM_00052	.	.	.	NP_00051	.	protein_co	ENSG0000	3073	4878
HEXB	54.4943	54.042	55.6355	54.3381	58.375	67.5124	hexosaminidase subunit beta [Source:HGNC Symbol;Acc:HGNC:4	chr5:73923	NM_00052	.	.	.	NP_00051	.	protein_co	ENSG0000	3074	4879
HEXDC	8.33916	7.27793	9.50625	6.39791	4.94706	7.21121	hexosaminidase D [Source:HGNC Symbol;Acc:HGNC:26307]	chr17:8034	NM_17362	XM_00672	.	.	NP_77589	XP_005256	protein_co	ENSG0000	284004	26307
HEXIM1	38.7517	40.1725	40.3695	44.5968	46.0849	38.6874	hexamethylene bisacetamide inducible 1 [Source:HGNC Symbol;A	chr17:4322	NM_00646	.	.	.	NP_00645	.	protein_co	ENSG0000	10614	24953
HEXIM2	7.65645	7.59849	8.80214	11.4803	10.5555	9.2986	hexamethylene bisacetamide inducible 2 [Source:HGNC Symbol;A	chr17:4323	NM_00130	XM_01152	.	.	NP_00129	XP_006721	protein_co	ENSG0000	124790	28591
HEY1	1.24927	0.750149	0.295391	0.575265	0.582323	1.55888	hes related family bHLH transcription factor with YRPW motif 1 [	chr8:80676	NM_00104	.	.	.	NP_00103	.	protein_co	ENSG0000	23462	4880
HEY2	0.136449	0.281616	0.32329	0.151898	0.043202	0.270889	hes related family bHLH transcription factor with YRPW motif 2 [	chr6:12606	NM_01225	XM_01701	.	.	NP_03639	XP_011533	protein_co	ENSG0000	23493	4881
HEYL	5.39857	5.11909	5.58876	5.13319	4.83814	4.47375	hes related family bHLH transcription factor with YRPW motif-like	chr1:40089	NM_01457	.	.	.	NP_05538	XP_005270	protein_co	ENSG0000	26508	4882
HFE	6.15685	7.58488	5.2921	6.53286	8.11345	5.12226	hemochromatosis [Source:HGNC Symbol;Acc:HGNC:4886]	chr6:26087	NM_00041	XM_01151	.	.	NP_00040	XP_011512	protein_co	ENSG0000	3077	4886
HFE2	0	0	0	0	0	0	hemochromatosis type 2 (juvenile) [Source:HGNC Symbol;Acc:HG	chr1:14539	NM_14527	XM_00527	.	.	NP_00130	XP_005272	protein_co	ENSG0000	148738	4887
HFM1	0.06169	0.050754	0.080747	0.035929	0.06713	0.087032	HFM1, ATP dependent DNA helicase homolog [Source:HGNC Sym	chr1:91726	NM_00101	XM_01154	.	.	NP_00101	XP_011539	protein_co	ENSG0000	164045	20193
HGD	0.180072	0.075319	0.125302	0.20105	0.161648	0.146449	homogentisate 1,2-dioxygenase [Source:HGNC Symbol;Acc:HGNC	chr3:12034	NM_00018	XM_00524	.	.	NP_00017	XP_005247	protein_co	ENSG0000	3081	4892
HGF	0	0	0	0	0	0	hepatocyte growth factor [Source:HGNC Symbol;Acc:HGNC:4893]	chr7:81328	NM_00060	XM_01151	.	.	NP_00059	XP_006716	protein_co	ENSG0000	3082	4893
HGFAC	0.047207	0.090605	0.076541	0.049778	0.05554	0	HGF activator [Source:HGNC Symbol;Acc:HGNC:4894]	chr4:34436	NM_00129	XM_01151	.	.	NP_00128	.	protein_co	ENSG0000	3083	4894
HGH1	1.74487	2.03035	1.41014	1.64158	1.65224	0.722665	HGH1 homolog [Source:HGNC Symbol;Acc:HGNC:24161]	chr8:14519	NM_01645	.	.	.	NP_05754	.	protein_co	ENSG0000	51236	24161
HGS	74.9181	77.7219	64.7756	90.4348	77.2931	62.5257	hepatocyte growth factor-regulated tyrosine kinase substrate [So	chr17:7965	NM_00471	XM_01152	.	.	NP_00470	XP_011523	protein_co	ENSG0000	9146	4897
HGSNAT	3.66027	4.36063	5.38864	2.82804	3.24817	4.94374	heparan-alpha-glucosaminide N-acetyltransferase [Source:HGNC	chr8:42995	NM_15241	XM_00527	.	.	NP_68963	XP_005273	protein_co	ENSG0000	138050	26527
HHAT	2.07439	1.4201	3.4404	1.41269	1.3288	2.28059	hedgehog acyltransferase [Source:HGNC Symbol;Acc:HGNC:1827	chr1:21050	NM_00112	XM_01700	.	.	NP_00111	XP_006711	protein_co	ENSG0000	55733	18270
HHATL	0.052601	0.106189	0.042074	0.114159	0	0.056132	hedgehog acyltransferase-like [Source:HGNC Symbol;Acc:HGNC:1	chr3:42734	NM_02070	XM_01700	.	.	NP_06575	XP_006713	protein_co	ENSG0000	57467	13242

HHEX	9.67904	8.99049	8.84578	12.1407	9.15173	7.55371	hematopoietically expressed homeobox [Source:HGNC Symbol;A	chr10:9444	NM_00272	.	.	.	NP_00272	.	protein_co	ENSG0000	3087	4901
HHIP	0	0	0	0	0.010117	0	hedgehog interacting protein [Source:HGNC Symbol;Acc:HGNC:1	chr4:14556	NM_02247	XM_00671	.	.	NP_07192	XP_005263	protein_co	ENSG0000	64399	14866
HHIPL1	0.043262	0.011228	0	0.09167	0	0.012073	HHIP like 1 [Source:HGNC Symbol;Acc:HGNC:19710]	chr14:1001	NM_00112	XM_01702	.	.	NP_00112	XP_00672	protein_co	ENSG0000	84439	19710
HHIPL2	0.085066	0.231012	0.049065	0.093036	0.161955	0	HHIP like 2 [Source:HGNC Symbol;Acc:HGNC:25842]	chr1:2226	NM_02474	XM_01150	.	.	NP_07902	XP_011508	protein_co	ENSG0000	79802	25842
HHLA1	0	0	0	0	0	0	HERV-H LTR-associating 1 [Source:HGNC Symbol;Acc:HGNC:4904]	chr8:1330	NM_00114	.	.	.	NP_00113	.	protein_co	ENSG0000	10086	4904
HHLA2	0	0	0	0	0.055359	0	HERV-H LTR-associating 2 [Source:HGNC Symbol;Acc:HGNC:4905]	chr3:10801	NM_00128	XM_01151	.	.	NP_00126	XP_005247	protein_co	ENSG0000	11148	4905
HHLA3	9.66499	7.54585	7.64905	11.0699	7.68683	11.4245	HERV-H LTR-associating 3 [Source:HGNC Symbol;Acc:HGNC:4906]	chr1:7082	NM_00103	.	.	.	NP_00102	.	protein_co	ENSG0000	11147	4906
HIBADH	22.8063	24.015	28.2113	24.914	23.6972	35.0585	3-hydroxyisobutyrate dehydrogenase [Source:HGNC Symbol;Acc:	chr7:2756	NM_15274	.	.	.	NP_68995	.	protein_co	ENSG0000	11112	4907
HIBCH	9.80549	9.42928	11.9794	9.8138	11.8281	10.0588	3-hydroxyisobutyryl-CoA hydrolase [Source:HGNC Symbol;Acc:H	chr2:1910	NM_01436	XM_01151	.	.	NP_05517	XP_01150	protein_co	ENSG0000	26275	4908
HIC1	0.013222	0.070409	0.038749	0.014161	0.167096	0.043775	hypermethylated in cancer 1 [Source:HGNC Symbol;Acc:HGNC:49	chr17:1958	NM_00109	.	.	.	NP_00109	.	protein_co	ENSG0000	3090	4909
HIC2	3.18746	3.33015	3.31104	3.26796	3.66889	2.96393	hypermethylated in cancer 2 [Source:HGNC Symbol;Acc:HGNC:18	chr22:217	NM_01509	XM_01702	.	.	NP_05590	XP_005261	protein_co	ENSG0000	23119	18595
HID1	13.7547	14.2768	17.1324	15.7863	12.9025	15.2706	HID1 domain containing [Source:HGNC Symbol;Acc:HGNC:15736]	chr17:7294	NM_03063	XM_00525	.	.	NP_08513	XP_005257	protein_co	ENSG0000	283987	15736
HIF1A	41.6186	42.6531	47.0546	43.1769	48.4242	51.0655	hypoxia inducible factor 1 alpha subunit [Source:HGNC Symbol;A	chr14:621	NM_00124	.	.	.	NP_00123	.	protein_co	ENSG0000	3091	4910
HIF1AN	13.6434	14.6489	14.6131	13.0486	13.4538	13.0254	hypoxia inducible factor 1 alpha subunit inhibitor [Source:HGNC	chr10:1022	NM_0179	XM_01153	.	.	NP_06037	XP_011538	protein_co	ENSG0000	55662	17113
HIF3A	0.015961	0.016201	0.013727	0.05472	0.054065	0	hypoxia inducible factor 3 alpha subunit [Source:HGNC Symbol;A	chr19:468	NM_02246	XM_00672	.	.	NP_07190	XP_005259	protein_co	ENSG0000	64344	15825
HIGD1A	105.025	98.0574	101.154	149.863	142.651	141.79	HIG1 hypoxia inducible domain family member 1A [Source:HGNC	chr3:4282	NM_00109	.	.	.	NP_00109	.	protein_co	ENSG0000	25994	29527
HIGD1B	0	0	0	0	0	0	HIG1 hypoxia inducible domain family member 1B [Source:HGNC	chr17:4292	NM_00127	XM_01152	.	.	NP_00125	XP_006722	protein_co	ENSG0000	51751	24318
HIGD1C	0	0	0	0	0	0	HIG1 hypoxia inducible domain family member 1C [Source:HGNC	chr12:5134	NM_0011	.	.	.	NP_00110	.	protein_co	ENSG0000	613227	28044
HIGD2A	168.039	152.609	129.16	184.902	145.826	138.217	HIG1 hypoxia inducible domain family member 2A [Source:HGNC	chr5:17581	NM_13882	.	.	.	NP_62017	.	protein_co	ENSG0000	192286	28311
HIGD2B	0	0	0	0	0	0	HIG1 hypoxia inducible domain family member 2B [Source:HGNC	chr15:729	.	.	.	.	.	.	protein_co	ENSG0000	.	26984
HIKESHI	42.4717	32.0998	38.4923	46.3406	41.1952	47.9939	Hikeshi, heat shock protein nuclear import factor [Source:HGNC	chr11:8601	NM_0164	XM_01154	.	.	NP_05748	XP_016873	protein_co	ENSG0000	51501	26938
HILPDA	25.7164	25.376	20.5332	34.2836	31.6122	23.44	hypoxia inducible lipid droplet associated [Source:HGNC Symbol;	chr7:1280	NM_00109	.	.	.	NP_00109	.	protein_co	ENSG0000	29923	28859
HINFP	11.1716	11.7077	9.96591	10.4364	10.9701	8.36148	histone H4 transcription factor [Source:HGNC Symbol;Acc:HGNC:	chr11:118	NM_00124	XM_01154	.	.	NP_00123	XP_011541	protein_co	ENSG0000	25988	17850
HINT1	318.823	313.246	261.194	389.242	311.106	318.567	histidine triad nucleotide binding protein 1 [Source:HGNC Symbo	chr5:1304	NM_00534	XM_01154	.	.	NP_00533	XP_011541	protein_co	ENSG0000	3094	4912
HINT2	35.0904	37.1265	25.9281	45.9411	30.9875	40.3887	histidine triad nucleotide binding protein 2 [Source:HGNC Symbo	chr9:35812	NM_03259	.	.	.	NP_11598	.	protein_co	ENSG0000	84681	18344
HINT3	8.5811	9.72018	8.64124	7.86901	9.57347	9.06529	histidine triad nucleotide binding protein 3 [Source:HGNC Symbo	chr6:12627	NM_13857	.	.	.	NP_61263	.	protein_co	ENSG0000	135114	18468
HIP1	8.26295	8.27858	8.56041	8.5772	8.89355	8.26772	huntingtin interacting protein 1 [Source:HGNC Symbol;Acc:HGNC	chr7:75162	NM_00124	XM_01701	.	.	NP_00123	XP_00525	protein_co	ENSG0000	3092	4913
HIP1R	19.3125	21.39	19.1399	21.4156	20.6805	20.6588	huntingtin interacting protein 1 related [Source:HGNC Symbol;Ac	chr12:123	NM_00395	XM_01153	.	.	NP_00395	XP_011537	protein_co	ENSG0000	9026	18415
HIPK1	11.2927	12.1774	11.5804	10.5812	11.0025	11.5871	homeodomain interacting protein kinase 1 [Source:HGNC Symbo	chr1:11447	NM_1526	XM_01700	.	.	NP_68990	XP_00527	protein_co	ENSG0000	204851	19006
HIPK2	11.463	12.2802	11.5547	9.52594	10.9305	11.8502	homeodomain interacting protein kinase 2 [Source:HGNC Symbo	chr7:13924	NM_00111	XM_01701	.	.	NP_00110	XP_006715	protein_co	ENSG0000	28996	14402
HIPK3	8.54527	9.18887	11.3242	7.94369	9.35764	10.8071	homeodomain interacting protein kinase 3 [Source:HGNC Symbo	chr11:3327	NM_00104	XM_01701	.	.	NP_00104	XP_005252	protein_co	ENSG0000	10114	4915
HIPK4	0.166061	0.550882	0.17067	0.387252	0.350936	0.299354	homeodomain interacting protein kinase 4 [Source:HGNC Symbo	chr19:408	NM_14468	XM_00672	.	.	NP_65328	XP_006723	protein_co	ENSG0000	147746	19007
HIRA	14.1531	14.5444	13.613	16.5588	15.1212	14.1334	histone cell cycle regulator [Source:HGNC Symbol;Acc:HGNC:491	chr22:1931	NM_00332	.	.	.	NP_00331	.	protein_co	ENSG0000	7290	4916
HIRIP3	15.428	16.0606	11.4472	15.6556	17.313	13.7997	HIRA interacting protein 3 [Source:HGNC Symbol;Acc:HGNC:4917	chr16:300	NM_00119	.	.	.	NP_00118	.	protein_co	ENSG0000	8479	4917
HIST1H1A	0	0	0	0	0	0	histone cluster 1, H1a [Source:HGNC Symbol;Acc:HGNC:4715]	chr6:26017	NM_00532	.	.	.	NP_00531	.	protein_co	ENSG0000	3024	4715
HIST1H1B	0	0	0	0	0	0	histone cluster 1, H1b [Source:HGNC Symbol;Acc:HGNC:4719]	chr6:2783	NM_00532	.	.	.	NP_00531	.	protein_co	ENSG0000	3009	4719
HIST1H1C	15.4919	12.9954	14.3637	21.6811	22.5191	23.4123	histone cluster 1, H1c [Source:HGNC Symbol;Acc:HGNC:4716]	chr6:2605	NM_00531	.	.	.	NP_00531	.	protein_co	ENSG0000	3006	4716
HIST1H1D	0	0	0	0	0	0	histone cluster 1, H1d [Source:HGNC Symbol;Acc:HGNC:4717]	chr6:26234	NM_00532	.	.	.	NP_00531	.	protein_co	ENSG0000	3007	4717
HIST1H1E	0.380476	0.989748	0.218495	0.271348	0.451396	1.14099	histone cluster 1, H1e [Source:HGNC Symbol;Acc:HGNC:4718]	chr6:2615	NM_00532	.	.	.	NP_00531	.	protein_co	ENSG0000	3008	4718
HIST1H1T	0	0	0.102899	0	0	0	histone cluster 1, H1t [Source:HGNC Symbol;Acc:HGNC:4720]	chr6:2610	NM_00532	.	.	.	NP_00531	.	protein_co	ENSG0000	3010	4720
HIST1H2AA	0	0	0	0	0	0	histone cluster 1, H2aa [Source:HGNC Symbol;Acc:HGNC:18729]	chr6:2572	NM_17074	.	.	.	NP_73446	.	protein_co	ENSG0000	221613	18729
HIST1H2AB	0	0	0.350477	0	0.482782	0.451732	histone cluster 1, H2ab [Source:HGNC Symbol;Acc:HGNC:4734]	chr6:2603	NM_00351	.	.	.	NP_00350	.	protein_co	ENSG0000	8335	4734
HIST1H2AC	3.57193	3.33989	3.92544	2.51071	5.41403	5.73313	histone cluster 1, H2ac [Source:HGNC Symbol;Acc:HGNC:4733]	chr6:26124	NM_00351	.	.	.	NP_00350	.	protein_co	ENSG0000	8334	4733
HIST1H2AD	1.9E-06	1.48E-19	1.4E-05	3.23E-05	7.23E-29	3.33E-12	histone cluster 1, H2ad [Source:HGNC Symbol;Acc:HGNC:4729]	chr6:2619	NM_0210	.	.	.	NP_06640	.	protein_co	ENSG0000	3013	4729
HIST1H2AE	0	0	0	0	0	0	histone cluster 1, H2ae [Source:HGNC Symbol;Acc:HGNC:4724]	chr6:26217	NM_02105	.	.	.	NP_06639	.	protein_co	ENSG0000	3012	4724
HIST1H2AG	0.815643	0.813177	0.705343	1.00904	1.48224	1.29186	histone cluster 1, H2ag [Source:HGNC Symbol;Acc:HGNC:4737]	chr6:2710	NM_0210	.	.	.	NP_06640	.	protein_co	ENSG0000	8969	4737
HIST1H2AH	0.376136	0.368706	0.321694	0	0.439257	0.824868	histone cluster 1, H2ah [Source:HGNC Symbol;Acc:HGNC:13671]	chr6:27114	NM_08059	.	.	.	NP_54216	.	protein_co	ENSG0000	85235	13671
HIST1H2AI	0.661462	0.646599	0.568732	0.944706	0.777274	0.246105	histone cluster 1, H2ai [Source:HGNC Symbol;Acc:HGNC:4725]	chr6:2777	NM_0035	.	.	.	NP_00350	.	protein_co	ENSG0000	8329	4725
HIST1H2AJ	0.73103	0	0	1.56621	0.859117	0.80948	histone cluster 1, H2aj [Source:HGNC Symbol;Acc:HGNC:4727]	chr6:27782	NM_0210	.	.	.	NP_06654	.	protein_co	ENSG0000	8331	4727
HIST1H2AK	1.40933	0.341407	0.903414	0	0.82429	0	histone cluster 1, H2ak [Source:HGNC Symbol;Acc:HGNC:4726]	chr6:2780	NM_00351	.	.	.	NP_00350	.	protein_co	ENSG0000	8330	4726
HIST1H2AL	0	0	0	0	0	0	histone cluster 1, H2al [Source:HGNC Symbol;Acc:HGNC:4730]	chr6:2780	NM_00351	.	.	.	NP_00350	.	protein_co	ENSG0000	8332	4730
HIST1H2AM	0.783476	0.758642	0	1.68029	0	1.72382	histone cluster 1, H2am [Source:HGNC Symbol;Acc:HGNC:4735]	chr6:2786	NM_00351	.	.	.	NP_00350	.	protein_co	ENSG0000	8336	4735
HIST1H2BA	0	0	0	0	0	0	histone cluster 1, H2ba [Source:HGNC Symbol;Acc:HGNC:18730]	chr6:25727	NM_17061	.	.	.	NP_73375	.	protein_co	ENSG0000	255626	18730
HIST1H2BB	0	0	0	0	0	0	histone cluster 1, H2bb [Source:HGNC Symbol;Acc:HGNC:4751]	chr6:2604	NM_0210	.	.	.	NP_06640	.	protein_co	ENSG0000	3018	4751
HIST1H2BC	1.40128	2.23067	1.33817	2.03579	3.11024	2.97808	histone cluster 1, H2bc [Source:HGNC Symbol;Acc:HGNC:4757]	chr6:2611	NM_00352	.	.	.	NP_00351	.	protein_co	ENSG0000	8347	4757
HIST1H2BD	8.02567	5.02186	8.38959	6.58571	6.83549	7.47898	histone cluster 1, H2bd [Source:HGNC Symbol;Acc:HGNC:4747]	chr6:2615	NM_0210	XM_00524	.	.	NP_06640	XP_00524	protein_co	ENSG0000	3017	4747
HIST1H2BE	0.222937	0.152825	0.064417	0.413009	0.335151	0.228471	histone cluster 1, H2be [Source:HGNC Symbol;Acc:HGNC:4753]	chr6:26172	NM_00352	.	.	.	NP_00351	.	protein_co	ENSG0000	8344	4753
HIST1H2BF	0.090318	0.094397	0.077637	0	0	0.091184	histone cluster 1, H2bf [Source:HGNC Symbol;Acc:HGNC:4752]	chr6:2619	NM_00352	.	.	.	NP_00351	.	protein_co	ENSG0000	8343	4752
HIST1H2BG	0	0	0	0.277483	0	0	histone cluster 1, H2bg [Source:HGNC Symbol;Acc:HGNC:4746]	chr6:2621	NM_00351	.	.	.	NP_00350	.	protein_co	ENSG0000	8339	4746

HIST1H2BH	0	0	0	0	0	0	histone cluster 1, H2bh [Source:HGNC Symbol;Acc:HGNC:4755]	chr6:26251	NM_00352	.	.	.	NP_00351	.	protein_co	ENSG0000	8345	4755
HIST1H2BI	0	0	0	0	0	0	histone cluster 1, H2bi [Source:HGNC Symbol;Acc:HGNC:4756]	chr6:26273	NM_00352	.	.	.	NP_00351	.	protein_co	ENSG0000	8346	4756
HIST1H2BJ	1.69692	0.310528	0.533804	1.48236	0.689451	0.814406	histone cluster 1, H2bj [Source:HGNC Symbol;Acc:HGNC:4761]	chr6:27093	NM_02105	.	.	.	NP_06640	.	protein_co	ENSG0000	8970	4761
HIST1H2BK	20.7074	17.0777	17.4302	25.0861	18.7574	14.3364	histone cluster 1, H2bk [Source:HGNC Symbol;Acc:HGNC:13954]	chr6:27114	NM_08059	.	.	.	NP_00129	.	protein_co	ENSG0000	85236	13954
HIST1H2BL	0.220493	0	0.190763	0.237649	0	0	histone cluster 1, H2bl [Source:HGNC Symbol;Acc:HGNC:4748]	chr6:27775	NM_00351	.	.	.	NP_00351	.	protein_co	ENSG0000	8340	4748
HIST1H2BM	0.290724	0	0.252678	0	0	0	histone cluster 1, H2bm [Source:HGNC Symbol;Acc:HGNC:4750]	chr6:27782	NM_00352	.	.	.	NP_00351	.	protein_co	ENSG0000	8342	4750
HIST1H2BN	0.540769	0.253354	1.30808	0.423327	0.719396	0.975301	histone cluster 1, H2bn [Source:HGNC Symbol;Acc:HGNC:4749]	chr6:27805	NM_00352	.	.	.	NP_00351	.	protein_co	ENSG0000	8341	4749
HIST1H2BO	1.27532	0	0.552785	0.346755	0	1.02367	histone cluster 1, H2bo [Source:HGNC Symbol;Acc:HGNC:4758]	chr6:27861	NM_00352	.	.	.	NP_00351	.	protein_co	ENSG0000	8348	4758
HIST1H3A	0.411335	0.818752	0.346683	0.449511	0.953689	0	histone cluster 1, H3a [Source:HGNC Symbol;Acc:HGNC:4766]	chr6:26020	NM_00352	.	.	.	NP_00352	.	protein_co	ENSG0000	8350	4766
HIST1H3B	0	0.749268	0.325862	0	0	0.839147	histone cluster 1, H3b [Source:HGNC Symbol;Acc:HGNC:4776]	chr6:26031	NM_00353	.	.	.	NP_00352	.	protein_co	ENSG0000	8358	4776
HIST1H3C	0	0	0	0	0	0	histone cluster 1, H3c [Source:HGNC Symbol;Acc:HGNC:4768]	chr6:26045	NM_00353	.	.	.	NP_00352	.	protein_co	ENSG0000	8352	4768
HIST1H3D	1.47751	0.000358	4.03E-08	0.215089	5.8E-05	1.71E-05	histone cluster 1, H3d [Source:HGNC Symbol;Acc:HGNC:4767]	chr6:26196	NM_00353	.	.	.	NP_00352	.	protein_co	ENSG0000	8351	4767
HIST1H3E	2.74458	1.85699	2.46182	1.52651	1.96394	1.38826	histone cluster 1, H3e [Source:HGNC Symbol;Acc:HGNC:4769]	chr6:26224	NM_00353	.	.	.	NP_00352	.	protein_co	ENSG0000	8353	4769
HIST1H3F	0	0	0	0	0	0	histone cluster 1, H3f [Source:HGNC Symbol;Acc:HGNC:4773]	chr6:26250	NM_02101	.	.	.	NP_06629	.	protein_co	ENSG0000	8968	4773
HIST1H3G	0	0	0	0	0	0	histone cluster 1, H3g [Source:HGNC Symbol;Acc:HGNC:4772]	chr6:26269	NM_00353	.	.	.	NP_00352	.	protein_co	ENSG0000	8355	4772
HIST1H3H	1.31441	1.15984	1.52284	1.9829	2.23902	1.92489	histone cluster 1, H3h [Source:HGNC Symbol;Acc:HGNC:4775]	chr6:27777	NM_00353	.	.	.	NP_00352	.	protein_co	ENSG0000	8357	4775
HIST1H3I	0	0	0	0	0	0	histone cluster 1, H3i [Source:HGNC Symbol;Acc:HGNC:4771]	chr6:27839	NM_00353	.	.	.	NP_00352	.	protein_co	ENSG0000	8354	4771
HIST1H3J	0	1.05849	0.302756	0.771167	0.421929	0.39359	histone cluster 1, H3j [Source:HGNC Symbol;Acc:HGNC:4774]	chr6:27858	NM_00353	.	.	.	NP_00352	.	protein_co	ENSG0000	8356	4774
HIST1H4A	0	0.446028	0	0	0	0	histone cluster 1, H4a [Source:HGNC Symbol;Acc:HGNC:4781]	chr6:26021	NM_00353	.	.	.	NP_00352	.	protein_co	ENSG0000	8359	4781
HIST1H4B	0	0.752947	0.32223	0.818757	0	0.794627	histone cluster 1, H4b [Source:HGNC Symbol;Acc:HGNC:4789]	chr6:26027	NM_00354	.	.	.	NP_00353	.	protein_co	ENSG0000	8366	4789
HIST1H4C	0.325624	0.325298	0.558737	0.711258	0.371905	0.675622	histone cluster 1, H4c [Source:HGNC Symbol;Acc:HGNC:4787]	chr6:26104	NM_00354	.	.	.	NP_00353	.	protein_co	ENSG0000	8364	4787
HIST1H4D	1.10437	0.552828	0	0.60029	0	0	histone cluster 1, H4d [Source:HGNC Symbol;Acc:HGNC:4782]	chr6:26188	NM_00353	.	.	.	NP_00353	.	protein_co	ENSG0000	8360	4782
HIST1H4E	0.74788	0.388038	0.511364	0.588204	0.336986	0.680769	histone cluster 1, H4e [Source:HGNC Symbol;Acc:HGNC:4790]	chr6:26204	NM_00354	.	.	.	NP_00353	.	protein_co	ENSG0000	8367	4790
HIST1H4F	0	0	0	0	0	0	histone cluster 1, H4f [Source:HGNC Symbol;Acc:HGNC:4783]	chr6:26240	NM_00354	.	.	.	NP_00353	.	protein_co	ENSG0000	8361	4783
HIST1H4G	0	0	0	0	0	0	histone cluster 1, H4g [Source:HGNC Symbol;Acc:HGNC:4792]	chr6:26246	NM_00354	.	.	.	NP_00353	.	protein_co	ENSG0000	8369	4792
HIST1H4H	0.29043	0.294801	0	0.226466	0.111991	0.199137	histone cluster 1, H4h [Source:HGNC Symbol;Acc:HGNC:4788]	chr6:26281	NM_00354	.	.	.	NP_00353	.	protein_co	ENSG0000	8365	4788
HIST1H4I	0	0	0.320381	0.082879	0.083167	0.371371	histone cluster 1, H4i [Source:HGNC Symbol;Acc:HGNC:4793]	chr6:27106	NM_00349	.	.	.	NP_00348	.	protein_co	ENSG0000	8294	4793
HIST1H4J	0	0	0	0	0	0.422902	histone cluster 1, H4j [Source:HGNC Symbol;Acc:HGNC:4785]	chr6:27791	NM_02196	.	.	.	NP_06880	.	protein_co	ENSG0000	8363	4785
HIST1H4K	0	0	0	0	0	0	histone cluster 1, H4k [Source:HGNC Symbol;Acc:HGNC:4784]	chr6:27798	NM_00354	.	.	.	NP_00353	.	protein_co	ENSG0000	8362	4784
HIST1H4L	0	0	0	0	0	0	histone cluster 1, H4l [Source:HGNC Symbol;Acc:HGNC:4791]	chr6:27840	NM_00354	.	.	.	NP_00353	.	protein_co	ENSG0000	8368	4791
HIST2H2AA3	0.103172	0	0	0	0	0	histone cluster 2, H2aa3 [Source:HGNC Symbol;Acc:HGNC:4736]	chr1:14981	NM_00351	.	.	.	NP_00350	.	protein_co	ENSG0000	8337	4736
HIST2H2AA4	0	0	0	0	0	0	histone cluster 2, H2aa3 [Source:HGNC Symbol;Acc:HGNC:4736]	chr1:14982	NM_00104	.	.	.	NP_00350	.	protein_co	ENSG0000	8337	4736
HIST2H2AB	0	0	0	0	0.454044	0	histone cluster 2, H2ab [Source:HGNC Symbol;Acc:HGNC:20508]	chr1:14985	NM_17506	.	.	.	NP_77823	.	protein_co	ENSG0000	317772	20508
HIST2H2AC	3.74315	3.56437	1.60964	6.33307	4.44922	5.45995	histone cluster 2, H2ac [Source:HGNC Symbol;Acc:HGNC:4738]	chr1:14985	NM_00351	.	.	.	NP_00350	.	protein_co	ENSG0000	8338	4738
HIST2H2BE	1.08069	0.798327	0.702743	1.55259	0.694274	1.51726	histone cluster 2, H2be [Source:HGNC Symbol;Acc:HGNC:4760]	chr1:14985	NM_00352	.	.	.	NP_00351	.	protein_co	ENSG0000	8349	4760
HIST2H2BF	0.314477	0.540608	0.077844	0.301053	0.269801	0.200087	histone cluster 2, H2bf [Source:HGNC Symbol;Acc:HGNC:24700]	chr1:14975	NM_00102	.	.	.	NP_00101	.	protein_co	ENSG0000	440689	24700
HIST2H3A	0	0	0	0	0	0	histone cluster 2, H3a [Source:HGNC Symbol;Acc:HGNC:20505]	chr1:14982	NM_00100	.	.	.	NP_00100	.	protein_co	ENSG0000	333932	20505
HIST2H3C	0	0	0	0	0	0	histone cluster 2, H3c [Source:HGNC Symbol;Acc:HGNC:20503]	chr1:14981	NM_02105	.	.	.	NP_06640	.	protein_co	ENSG0000	126961	20503
HIST2H3D	0	0	0	0	0	0	histone cluster 2, H3d [Source:HGNC Symbol;Acc:HGNC:25311]	chr1:14978	NM_00112	.	.	.	NP_00111	.	protein_co	ENSG0000	653604	25311
HIST2H3PS2	0	0	0	0	0	0	histone cluster 2, H3, pseudogene 2 [Source:HGNC Symbol;Acc:H	chr1:14940	.	.	.	.	.	.	protein_co	ENSG0000	.	32060
HIST2H4A	0	0	0	0	0	0	histone cluster 2, H4a [Source:HGNC Symbol;Acc:HGNC:4794]	chr1:14980	NM_00354	.	.	.	NP_00353	.	protein_co	ENSG0000	8370	4794
HIST2H4B	0	0	0	0	0	0.105626	histone cluster 2, H4b [Source:HGNC Symbol;Acc:HGNC:29607]	chr1:14983	NM_00103	.	.	.	NP_00102	.	protein_co	ENSG0000	554313	29607
HIST3H2A	8.49571	8.10029	11.4567	19.5618	13.8287	15.2733	histone cluster 3, H2a [Source:HGNC Symbol;Acc:HGNC:20507]	chr1:22864	NM_03344	.	.	.	NP_25428	.	protein_co	ENSG0000	92815	20507
HIST3H2BB	0.180291	0.317941	0.115972	0.198049	0.155698	0.095709	histone cluster 3, H2bb [Source:HGNC Symbol;Acc:HGNC:20514]	chr1:22864	NM_17505	.	.	.	NP_77822	.	protein_co	ENSG0000	128312	20514
HIST3H3	0	0	0	0	0	0	histone cluster 3, H3 [Source:HGNC Symbol;Acc:HGNC:4778]	chr1:22861	NM_00349	.	.	.	NP_00348	.	protein_co	ENSG0000	8290	4778
HIST4H4	0.197873	0	0	0.42627	0	0.430152	histone cluster 4, H4 [Source:HGNC Symbol;Acc:HGNC:20510]	chr12:1492	NM_17505	.	.	.	NP_77822	.	protein_co	ENSG0000	121504	20510
HIVEP1	2.43364	2.00459	2.42385	1.41865	1.45424	2.24573	human immunodeficiency virus type I enhancer binding protein 1	chr6:12012	NM_00211	XM_01151	.	.	NP_00210	XP_01151	protein_co	ENSG0000	3096	4920
HIVEP2	3.15857	3.86087	3.06546	1.64393	2.04714	2.30616	human immunodeficiency virus type I enhancer binding protein 2	chr6:14307	NM_00673	XM_01701	.	.	NP_00672	XP_01686	protein_co	ENSG0000	3097	4921
HIVEP3	0.595635	0.707574	0.911447	0.677594	0.668515	0.577798	human immunodeficiency virus type I enhancer binding protein 3	chr1:41972	NM_00112	XM_01700	NR_03826	.	NP_00112	XP_00671	protein_co	ENSG0000	59269	13561
HJURP	24.4476	24.8575	22.4649	37.0253	35.499	31.9055	Holliday junction recognition protein [Source:HGNC Symbol;Acc:H	chr2:23474	NM_00128	.	.	.	NP_00126	.	protein_co	ENSG0000	55355	25444
HK1	22.7162	23.5368	28.7718	26.8874	24.243	24.8704	hexokinase 1 [Source:HGNC Symbol;Acc:HGNC:4922]	chr10:7102	NM_00018	XM_01153	.	.	NP_00017	XP_00526	protein_co	ENSG0000	3098	4922
HK2	17.9968	20.067	15.5381	19.9184	23.7704	19.5761	hexokinase 2 [Source:HGNC Symbol;Acc:HGNC:4923]	chr2:75061	NM_00018	XM_01153	.	.	NP_00018	XP_00526	protein_co	ENSG0000	3099	4923
HK3	0.024875	0	0.021332	0.02668	0	0	hexokinase 3 [Source:HGNC Symbol;Acc:HGNC:4925]	chr5:17623	NM_00211	.	.	.	NP_00210	XP_01686	protein_co	ENSG0000	3101	4925
HKDC1	1.78761	1.83994	1.19892	1.30801	1.25659	4.2459	hexokinase domain containing 1 [Source:HGNC Symbol;Acc:HGN	chr10:7098	NM_02513	.	.	.	NP_07940	.	protein_co	ENSG0000	80201	23302
HKR1	4.90843	5.4936	5.76904	4.81772	4.38964	4.66375	HKR1, GLI-Kruppel zinc finger family member [Source:HGNC Symb	chr19:3780	NM_18178	XM_01702	.	.	NP_86145	XP_01688	protein_co	ENSG0000	284459	4928
HLA-A	313.861	313.408	250.484	293.657	252.932	260.892	major histocompatibility complex, class I, A [Source:HGNC Symbo	chr6:29909	NM_00211	.	.	.	NP_00210	.	protein_co	ENSG0000	3105	4931
HLA-B	309.354	304.054	184.556	230.386	202.352	191.314	major histocompatibility complex, class I, B [Source:HGNC Symbo	chr6:31321	NM_00551	XM_01151	.	.	NP_00550	XP_01151	protein_co	ENSG0000	3106	4932
HLA-C	361.262	369.006	259.146	332.912	286.742	269.853	major histocompatibility complex, class I, C [Source:HGNC Symbo	chr6:31236	NM_00211	.	.	.	NP_00210	.	protein_co	ENSG0000	3107	4933
HLA-DMA	11.0569	9.56787	8.99695	8.47594	9.21817	7.29143	major histocompatibility complex, class II, DM alpha [Source:HGN	chr6:32902	NM_00612	.	.	.	NP_00611	.	protein_co	ENSG0000	3108	4934

HLA-DMB	1.44888	1.87363	1.6451	2.7567	1.92101	1.99973	major histocompatibility complex, class II, DM beta [Source:HGN(	chr6:32902	NM_00211	.	.	.	NP_00210(	.	protein_co	ENSG0000(	3109	4935
HLA-DOA	0.290965	0.192419	0.57827	0.275148	0.236088	0.34635	major histocompatibility complex, class II, DO alpha [Source:HGN	chr6:32971	NM_00211	.	.	.	NP_00211(	.	protein_co	ENSG0000(	3111	4936
HLA-DOB	0.847699	0.565461	0.94777	0.613529	0.586106	0.340023	major histocompatibility complex, class II, DO beta [Source:HGNC	chr6:32780	NM_00212	.	.	.	NP_00211	.	protein_co	ENSG0000(	3112	4937
HLA-DPA1	1.04333	1.04402	0.831478	1.1642	0.725945	0.398293	major histocompatibility complex, class II, DP alpha 1 [Source:HG	chr6:33032	NM_00124	.	.	.	NP_00122(	.	protein_co	ENSG0000(	3113	4938
HLA-DPB1	0.79134	1.47198	0.945751	0.545049	0.33691	0.308459	major histocompatibility complex, class II, DP beta 1 [Source:HGN	chr6:33032	NM_00212	.	.	.	NP_00211	.	protein_co	ENSG0000(	3115	4940
HLA-DQA1	0	0	0	0	0	0	major histocompatibility complex, class II, DQ alpha 1 [Source:HG	chr6:32605	NM_00212	.	.	.	NP_00211	XP_006715	protein_co	ENSG0000(	3117	4942
HLA-DQA2	0	0	0	0	0	0	major histocompatibility complex, class II, DQ alpha 2 [Source:HG	chr6:32709	NM_02005	.	.	.	NP_06444(	.	protein_co	ENSG0000(	3118	4943
HLA-DQB1	2.11776	1.42851	2.66372	2.70777	1.89161	2.3671	major histocompatibility complex, class II, DQ beta 1 [Source:HGN	chr6:32627	NM_00124	.	.	.	NP_00123(	.	protein_co	ENSG0000(	3119	4944
HLA-DQB2	0.067862	0.405879	0.872741	0.732357	0.235445	0.662686	major histocompatibility complex, class II, DQ beta 2 [Source:HGN	chr6:32723	NM_00119	XM_01151	.	.	NP_00118	XP_005249	protein_co	ENSG0000(	3120	4945
HLA-DRA	4.16416	3.71069	5.7285	1.68612	1.12636	1.99108	major histocompatibility complex, class II, DR alpha [Source:HGN(	chr6:32407	NM_01911	.	.	.	NP_06198	.	protein_co	ENSG0000(	3122	4947
HLA-DRB1	5.15521	4.30853	6.04767	4.6575	3.85479	2.77536	major histocompatibility complex, class II, DR beta 1 [Source:HGN	chr6:32520	NM_00212	.	.	.	NP_00211	.	protein_co	ENSG0000(	3123	4948
HLA-DRB5	0.400049	0.347311	0.542623	0.561743	0.325395	0.303636	major histocompatibility complex, class II, DR beta 5 [Source:HGN	chr6:32485	NM_00212	.	.	.	NP_00211(	XP_011512	protein_co	ENSG0000(	3127	4953
HLA-E	73.3012	72.2651	77.5255	62.6724	57.9098	72.6165	major histocompatibility complex, class I, E [Source:HGNC Symbo	chr6:30457	NM_00551	XM_01701	.	.	NP_00550	XP_016866	protein_co	ENSG0000(	3133	4962
HLA-F	15.7823	14.03	13.3147	10.2216	9.11465	9.48927	major histocompatibility complex, class I, F [Source:HGNC Symbo	chr6:29690	NM_00109	XM_00524	.	.	NP_00109	XP_011512	protein_co	ENSG0000(	3134	4963
HLA-G	0.382521	0.456055	0.132798	0.070451	0.118812	0.071513	major histocompatibility complex, class I, G [Source:HGNC Symbo	chr6:29794	NM_00212	XM_01701	.	.	NP_00211	XP_016866	protein_co	ENSG0000(	3135	4964
HLCS	5.94305	5.74397	6.45005	4.28491	5.09341	7.45335	holocarboxylase synthetase [Source:HGNC Symbol;Acc:HGNC:497	chr21:3812	NM_00041	XM_01152	.	.	NP_00040	XP_005261	protein_co	ENSG0000(	3141	4976
HLF	0.887325	0.739155	0.550904	0.661283	0.76531	1.20347	HLF, PAR bZIP transcription factor [Source:HGNC Symbol;Acc:HGN	chr17:5334	NM_00212	XM_01152	.	.	NP_00211	XP_005257	protein_co	ENSG0000(	3131	4977
HLTF	20.0055	20.1424	23.529	20.0637	23.3173	30.7302	helicase like transcription factor [Source:HGNC Symbol;Acc:HGN(	chr3:14874	NM_00131	XM_01151	.	.	NP_00130	XP_005247	protein_co	ENSG0000(	6596	11099
HLX	0.685138	1.47943	1.1217	1.34521	1.22866	0.707781	H2.0 like homeobox [Source:HGNC Symbol;Acc:HGNC:4978]	chr1:22105	NM_02195	.	.	.	NP_06877	.	protein_co	ENSG0000(	3142	4978
HM13	88.9748	92.5801	79.665	89.4215	69.2015	68.5645	histocompatibility minor 13 [Source:HGNC Symbol;Acc:HGNC:164	chr20:3010	NM_03078	XM_01152	.	.	NP_11041(	.	protein_co	ENSG0000(	81502	16435
HMBOX1	2.75235	3.36974	4.2412	2.20016	3.23655	3.38305	homeobox containing 1 [Source:HGNC Symbol;Acc:HGNC:26137]	chr8:28747	NM_00113	XM_00671	.	.	NP_00112	XP_005273	protein_co	ENSG0000(	79618	26137
HMBS	36.1531	37.1996	32.5203	48.5711	34.7406	30.5443	hydroxymethylbilane synthase [Source:HGNC Symbol;Acc:HGNC:	chr11:1189	NM_00019	XM_01154	.	.	NP_00018	XP_005271	protein_co	ENSG0000(	3145	4982
HMCES	44.2899	44.7191	43.2823	54.0205	45.0022	44.3695	5-hydroxymethylcytosine binding, ES cell specific [Source:HGNC S	chr3:12899	NM_00100	XM_01151	.	.	NP_00100(	XP_005247	protein_co	ENSG0000(	56941	24446
HMCN1	0.098546	0.078129	0.103897	0.063304	0.039424	0.098692	hemicentin 1 [Source:HGNC Symbol;Acc:HGNC:19194]	chr1:18570	NM_03193	XM_01151	.	.	NP_11414	XP_011508	protein_co	ENSG0000(	83872	19194
HMCN2	0.431175	0.49483	0.438619	0.181175	0.609739	0.109202	hemicentin 2 [Source:HGNC Symbol;Acc:HGNC:21293]	chr9:13328	NM_00129	XM_01151	.	.	NP_00127	XP_011516	protein_co	ENSG0000(	256158	21293
HMG20A	14.351	15.5369	17.455	13.5474	14.1476	19.6545	high mobility group 20A [Source:HGNC Symbol;Acc:HGNC:5001]	chr15:7771	NM_00130	XM_01152	.	.	NP_00129	XP_011519	protein_co	ENSG0000(	10363	5001
HMG20B	78.9728	77.5685	92.5288	122.25	88.9969	108.997	high mobility group 20B [Source:HGNC Symbol;Acc:HGNC:5002]	chr19:3572	NM_00633	XM_01702	.	.	NP_00633(	XP_016881	protein_co	ENSG0000(	10362	5002
HMGA1	927.955	939.555	1185.55	847.606	724.853	676.804	high mobility group AT-hook 1 [Source:HGNC Symbol;Acc:HGNC:5	chr6:34204	NM_00131	XM_01151	.	.	NP_00130(	XP_005249	protein_co	ENSG0000(	3159	5010
HMGA2	79.3543	83.7789	75.8024	86.2158	95.5143	96.394	high mobility group AT-hook 2 [Source:HGNC Symbol;Acc:HGNC:5	chr12:6621	NM_00130	.	.	.	NP_00128	XP_016875	protein_co	ENSG0000(	8091	5009
HMGB1	584.534	570.712	391.271	771.219	679.261	634.258	high mobility group box 1 [Source:HGNC Symbol;Acc:HGNC:4983]	chr13:3103	NM_00212	XM_01153	.	.	NP_00130(	XP_005266	protein_co	ENSG0000(	3146	4983
HMGB2	321.58	302.84	212.521	419.175	355.389	344.053	high mobility group box 2 [Source:HGNC Symbol;Acc:HGNC:5000]	chr4:17425	NM_00113	.	.	.	NP_00112	.	protein_co	ENSG0000(	3148	5000
HMGB3	79.6613	76.6698	59.3798	114.631	97.7245	83.2928	high mobility group box 3 [Source:HGNC Symbol;Acc:HGNC:5004]	chrX:15015	NM_00130	.	.	.	NP_00128	.	protein_co	ENSG0000(	3149	5004
HMGB4	0	0	0	0	0	0	high mobility group box 4 [Source:HGNC Symbol;Acc:HGNC:2495	chr1:33979	NM_14520	.	NR_03326	.	NP_66020(	.	protein_co	ENSG0000(	127540	24954
HMGCL	19.7975	19.6844	20.9922	20.5247	16.6439	21.8376	3-hydroxymethyl-3-methylglutaryl-CoA lyase [Source:HGNC Symb	chr1:24128	NM_00019	.	.	.	NP_00018	.	protein_co	ENSG0000(	3155	5005
HMGCLL1	0	0	0	0	0	0	3-hydroxymethyl-3-methylglutaryl-CoA lyase like 1 [Source:HGNC	chr6:55299	NM_00104	.	.	.	NP_00103	.	protein_co	ENSG0000(	54511	21359
HMGCR	49.8382	50.2267	52.5252	63.2216	69.6635	82.8292	3-hydroxy-3-methylglutaryl-CoA reductase [Source:HGNC Symbol	chr5:74632	NM_00085	XM_01154	.	.	NP_00085(	XP_011541	protein_co	ENSG0000(	3156	5006
HMGCS1	57.3794	53.5621	53.6461	45.2395	51.8416	62.4856	3-hydroxy-3-methylglutaryl-CoA synthase 1 [Source:HGNC Symbo	chr5:43289	NM_00109	XM_01700	.	.	NP_00109	XP_011512	protein_co	ENSG0000(	3157	5007
HMGCS2	0	0	0	0	0	0	3-hydroxy-3-methylglutaryl-CoA synthase 2 [Source:HGNC Symbo	chr1:12029	NM_00116	XM_01154	.	.	NP_00115	XP_011539	protein_co	ENSG0000(	3158	5008
HMGN1	233.883	229.575	210.961	256.926	241.098	270.96	high mobility group nucleosome binding domain 1 [Source:HGNC	chr21:4071	NM_00496	XM_01152	.	.	NP_00495	XP_011527	protein_co	ENSG0000(	3150	4984
HMGN2	297.304	297.671	175.136	343.058	318.061	201.272	high mobility group nucleosomal binding domain 2 [Source:HGNC	chr1:26799	NM_00551	.	.	.	NP_00550	.	protein_co	ENSG0000(	3151	4986
HMGN3	32.9003	36.2856	28.7744	35.7943	37.0636	32.654	high mobility group nucleosomal binding domain 3 [Source:HGNC	chr6:79910	NM_00120	XM_00671	.	.	NP_00118	.	protein_co	ENSG0000(	9324	12312
HMGN4	80.1931	79.1158	73.0252	76.4516	74.1198	75.1786	high mobility group nucleosomal binding domain 4 [Source:HGNC	chr6:26538	NM_00635	.	.	.	NP_00634	XP_016865	protein_co	ENSG0000(	10473	4989
HMGN5	2.50598	2.30794	2.52616	3.00406	2.73838	2.87356	high mobility group nucleosome binding domain 5 [Source:HGNC	chrX:80369	NM_03076	XM_00672	.	.	NP_11039(	XP_006724	protein_co	ENSG0000(	79366	8013
HMGXB3	19.5139	19.651	19.3876	23.5592	21.2026	17.723	HMG-box containing 3 [Source:HGNC Symbol;Acc:HGNC:28982]	chr5:14937	NM_01498	XM_00671	.	.	NP_05579	XP_006714	protein_co	ENSG0000(	22993	28982
HMGXB4	16.8273	17.4939	16.0638	16.0409	16.6065	18.4471	HMG-box containing 4 [Source:HGNC Symbol;Acc:HGNC:5003]	chr22:3565	NM_00100	XM_01152	.	.	NP_00100	XP_006724	protein_co	ENSG0000(	10042	5003
HMHB1	0	0	0	0	0.304875	0	histocompatibility minor HB-1 [Source:HGNC Symbol;Acc:HGNC:2	chr5:14319	NM_02118	.	.	.	NP_06700	.	protein_co	ENSG0000(	57824	29677
HMMR	20.1333	18.6967	19.5737	26.3081	34.2718	33.667	hyaluronan mediated motility receptor [Source:HGNC Symbol;Ac	chr5:16288	NM_00114	.	.	.	NP_00113(	.	protein_co	ENSG0000(	3161	5012
HMOX1	14.4269	16.7172	13.7729	20.7485	17.0905	18.0066	heme oxygenase 1 [Source:HGNC Symbol;Acc:HGNC:5013]	chr22:3577	NM_00213	.	.	.	NP_00212	.	protein_co	ENSG0000(	3162	5013
HMOX2	41.6639	38.2372	38.5444	51.21	37.3509	34.4738	heme oxygenase 2 [Source:HGNC Symbol;Acc:HGNC:5014]	chr16:4511	NM_00112	XM_01702	.	.	NP_00112(	XP_011520	protein_co	ENSG0000(	3163	5014
HMP19	0.059774	0	0	0	0.034265	0.031608	HMP19 protein [Source:EntrezGene;Acc:51617]	chr5:17347	NM_01598	.	.	.	NP_05706	.	protein_co	ENSG0000(	51617	.
HMSD	5.37934	0.877207	3.88183	6.70151	2.09164	2.52771	histocompatibility minor serpin domain containing [Source:HGNC	chr18:6161	NM_00112	.	.	.	NP_00111(	.	protein_co	ENSG0000(	284293	23037
HMX1	0	0	0	0	0	0.242153	H6 family homeobox 1 [Source:HGNC Symbol;Acc:HGNC:5017]	chr4:88478	NM_00130	XM_00524	.	.	NP_00129	XP_005248	protein_co	ENSG0000(	3166	5017
HMX2	0	0	0	0	0	0	H6 family homeobox 2 [Source:HGNC Symbol;Acc:HGNC:5018]	chr10:1249	NM_00551	XM_00526	.	.	NP_00551(	XP_005269	protein_co	ENSG0000(	3167	5018
HMX3	0	0	0	0	0	0	H6 family homeobox 3 [Source:HGNC Symbol;Acc:HGNC:5019]	chr10:1248	NM_00110	.	.	.	NP_00109	.	protein_co	ENSG0000(	340784	5019
HN1	373.435	379.043	316.028	701.837	516.874	413.849	hematological and neurological expressed 1 [Source:HGNC Symb	chr17:7313	NM_00100	.	.	.	NP_00100	.	protein_co	ENSG0000(	51155	14569
HN1L	67.2724	67.8446	61.4438	74.8815	77.1766	62.7129	hematological and neurological expressed 1 like [Source:HGNC Sy	chr16:1728	NM_14457	.	.	.	NP_65317	.	protein_co	ENSG0000(	90861	14137
HNF1A	0.038869	0	0	0	0.045825	0.044457	HNF1 homeobox A [Source:HGNC Symbol;Acc:HGNC:11621]	chr12:1214	NM_00054	XM_01153	.	.	NP_00053(	XP_005253	protein_co	ENSG0000(	6927	11621
HNF1B	1.71542	1.98178	1.93119	2.54266	2.87488	2.40417	HNF1 homeobox B [Source:HGNC Symbol;Acc:HGNC:11630]	chr17:3604	NM_00045	XM_01152	.	.	NP_00044	XP_011523	protein_co	ENSG0000(	6928	11630
HNF4A	1.99109	1.98311	2.29179	2.77673	2.63493	3.29157	hepatocyte nuclear factor 4 alpha [Source:HGNC Symbol;Acc:HGN	chr20:4298	NM_00045	XM_00526	.	.	NP_00044	XP_005260	protein_co	ENSG0000(	3172	5024

HNF4G	2.222	1.67125	1.92366	1.30653	1.92974	1.37715	hepatocyte nuclear factor 4 gamma [Source:HGNC Symbol;Acc:H	chr8:7632	NM_00413	XM_01151	.	.	NP_00412	XP_01151	protein_co	ENSG0000	3174	5026
HNMT	0	0	0	0	0	0.058658	histamine N-methyltransferase [Source:HGNC Symbol;Acc:HGNC:	chr2:13872	NM_00102	.	.	.	NP_00101	.	protein_co	ENSG0000	3176	5028
HNRNPA0	36.9727	41.7495	34.4679	46.3197	42.6051	40.5316	heterogeneous nuclear ribonucleoprotein A0 [Source:HGNC Syml	chr5:13708	NM_00680	.	.	.	NP_00679	.	protein_co	ENSG0000	10949	5030
HNRNPA1	660.229	659.189	675.213	729.56	665.709	743.905	heterogeneous nuclear ribonucleoprotein A1 [Source:HGNC Syml	chr12:5467	NM_00213	XM_00526	.	.	NP_00212	XP_005268	protein_co	ENSG0000	3178	5031
HNRNPA1L2	2.5348	2.41745	1.71251	2.40358	2.28772	2.76897	heterogeneous nuclear ribonucleoprotein A1-like 2 [Source:HGN	chr13:5321	NM_00101	.	.	.	NP_00101	.	protein_co	ENSG0000	144983	27067
HNRNPA2B1	723.597	674.26	615.623	699.462	794.955	783.232	heterogeneous nuclear ribonucleoprotein A2/B1 [Source:HGNC S	chr7:26229	NM_00213	XM_00671	.	.	NP_00212	XP_005249	protein_co	ENSG0000	3181	5033
HNRNPA3	195.381	195.661	178.675	221.665	248.337	240.619	heterogeneous nuclear ribonucleoprotein A3 [Source:HGNC Syml	chr2:17807	NM_19424	XM_00671	NR_03964	.	NP_91922	XP_005246	protein_co	ENSG0000	100616394	24941
HNRNPAB	193.154	181.18	161.346	231.57	234.779	197.606	heterogeneous nuclear ribonucleoprotein A/B [Source:HGNC Sym	chr5:17763	NM_00449	.	.	.	NP_00449	.	protein_co	ENSG0000	3182	5034
HNRNPC	543.165	506.673	437.843	648.189	553.016	610.02	heterogeneous nuclear ribonucleoprotein C (C1/C2) [Source:HGN	chr14:2167	NM_00107	XM_01153	.	.	NP_00107	XP_00672	protein_co	ENSG0000	3183	5035
HNRNPCL1	0.252219	0.521523	0.166155	0.42024	0.212175	0.257122	heterogeneous nuclear ribonucleoprotein C-like 1 [Source:HGNC	chr1:12907	NM_00101	.	.	.	NP_00101	.	protein_co	ENSG0000	343069	29295
HNRNPCL2	0.124039	0.193027	0.108976	0	0.138962	0	heterogeneous nuclear ribonucleoprotein C-like 2 [Source:HGNC	chr1:13182	NM_00113	.	.	.	.	.	protein_co	ENSG0000	440563	48813
HNRNPCL4	0	4.13E-07	4.87E-07	0	0	0	heterogeneous nuclear ribonucleoprotein C-like 4 [Source:HGNC	chr1:13182	NM_00130	.	.	.	NP_00128	.	protein_co	ENSG0000	101060301	51333
HNRNPD	205.491	200.152	178.037	237.048	255.567	233.066	heterogeneous nuclear ribonucleoprotein D [Source:HGNC Symb	chr4:83273	NM_00100	.	.	.	NP_00100	.	protein_co	ENSG0000	3184	5036
HNRNPDL	184.755	171.898	142.507	211.676	210.383	134.367	heterogeneous nuclear ribonucleoprotein D like [Source:HGNC Sy	chr4:83343	NM_00120	.	.	.	NP_00119	.	protein_co	ENSG0000	9987	5037
HNRNPF	207.579	217.9	183.1	238.16	219.589	192.861	heterogeneous nuclear ribonucleoprotein F [Source:HGNC Symb	chr10:4388	NM_00109	.	.	.	NP_00109	.	protein_co	ENSG0000	3185	5039
HNRNPH1	146.213	152.071	175.999	163.358	155.971	171.923	heterogeneous nuclear ribonucleoprotein H1 (H) [Source:HGNC S	chr5:17904	NM_00125	XM_01700	.	.	NP_00124	XP_005265	protein_co	ENSG0000	3187	5041
HNRNPH2	54.119	55.4563	52.7426	56.6409	59.2987	54.1338	heterogeneous nuclear ribonucleoprotein H2 (H') [Source:HGNC	chrX:10064	NM_00103	.	.	.	NP_00102	.	protein_co	ENSG0000	3188	5042
HNRNPH3	65.0285	63.2826	59.5238	74.8109	80.4833	72.2606	heterogeneous nuclear ribonucleoprotein H3 [Source:HGNC Syml	chr10:7004	NM_00132	XM_00671	.	.	NP_00130	XP_005269	protein_co	ENSG0000	3189	5043
HNRNPK	468.407	458.862	393.46	502.185	480.838	442.851	heterogeneous nuclear ribonucleoprotein K [Source:HGNC Symb	chr9:86582	NM_00131	XM_01701	NR_02960	.	NP_00130	XP_005252	protein_co	ENSG0000	407043	5044
HNRNPL	213.412	217.658	181.912	263.759	235.802	210.733	heterogeneous nuclear ribonucleoprotein L [Source:HGNC Symb	chr19:3930	NM_00100	XM_01152	.	.	NP_00100	XP_011525	protein_co	ENSG0000	3191	5045
HNRNPLL	20.1496	17.9476	20.3785	21.452	25.7835	32.073	heterogeneous nuclear ribonucleoprotein L like [Source:HGNC Sy	chr2:38789	NM_00114	XM_01153	.	.	NP_00113	XP_005264	protein_co	ENSG0000	92906	25127
HNRNPM	204.004	212.72	127.913	237.767	221.787	148.907	heterogeneous nuclear ribonucleoprotein M [Source:HGNC Symb	chr19:8509	NM_00129	XM_00527	.	.	NP_00128	XP_005272	protein_co	ENSG0000	4670	5046
HNRNPR	106.384	110.303	77.046	103.58	107.834	101.091	heterogeneous nuclear ribonucleoprotein R [Source:HGNC Symb	chr1:23631	NM_00110	XM_01154	.	.	NP_00109	XP_005245	protein_co	ENSG0000	10236	5047
HNRNPU	242.591	242.235	199.09	260.079	273.019	250.939	heterogeneous nuclear ribonucleoprotein U [Source:HGNC Symb	chr1:24501	NM_00450	.	.	.	NP_00449	XP_016856	protein_co	ENSG0000	3192	5048
HNRNPUL1	63.8938	65.4134	57.7535	74.1722	67.3908	69.0184	heterogeneous nuclear ribonucleoprotein U like 1 [Source:HGNC	chr19:4176	NM_00704	XM_01152	.	.	NP_00128	XP_005258	protein_co	ENSG0000	11100	17011
HNRNPUL2	41.8373	44.8826	35.4307	44.9218	44.4651	39.1157	heterogeneous nuclear ribonucleoprotein U like 2 [Source:HGNC	chr11:6248	NM_00107	.	.	.	NP_00107	.	protein_co	ENSG0000	221092	25451
HOGA1	0.494838	0.757032	0.696782	0.986096	0.546285	0.476509	4-hydroxy-2-oxoglutarate aldolase 1 [Source:HGNC Symbol;Acc:H	chr10:9934	NM_00113	.	.	.	NP_00112	.	protein_co	ENSG0000	112817	25155
HOMER1	5.22428	6.1035	5.52101	6.33477	6.5018	6.4968	homer scaffolding protein 1 [Source:HGNC Symbol;Acc:HGNC:17	chr5:78668	NM_00127	.	.	.	NP_00126	.	protein_co	ENSG0000	9456	17512
HOMER2	0.385361	0.487969	0.279589	0.45262	0.257004	0.409182	homer scaffolding protein 2 [Source:HGNC Symbol;Acc:HGNC:17	chr15:8351	NM_00483	XM_01152	.	.	NP_00483	XP_005272	protein_co	ENSG0000	9455	17513
HOMER3	28.3541	25.4669	27.5255	17.2763	13.3349	17.2818	homer scaffolding protein 3 [Source:HGNC Symbol;Acc:HGNC:17	chr19:1904	NM_00114	XM_00672	.	.	NP_00113	XP_006723	protein_co	ENSG0000	9454	17514
HOMEZ	6.70918	6.60876	7.77574	7.16484	6.94476	7.61946	homeobox and leucine zipper encoding [Source:HGNC Symbol;Ac	chr14:2365	NM_02083	.	.	.	NP_06588	.	protein_co	ENSG0000	57594	20164
HOOK1	7.49743	7.97797	8.06577	7.61476	9.58608	10.787	hook microtubule tethering protein 1 [Source:HGNC Symbol;Acc:	chr1:60280	NM_01588	XM_01154	.	.	NP_05697	XP_011539	protein_co	ENSG0000	51361	19884
HOOK2	16.0731	16.3881	17.8155	13.7256	14.0869	15.2702	hook microtubule tethering protein 2 [Source:HGNC Symbol;Acc:	chr19:1287	NM_00110	XM_01152	.	.	NP_00109	XP_005259	protein_co	ENSG0000	29911	19885
HOOK3	2.43749	2.30715	2.89524	2.28563	2.86271	3.28343	hook microtubule tethering protein 3 [Source:HGNC Symbol;Acc:	chr8:42752	NM_03241	.	.	.	NP_11578	.	protein_co	ENSG0000	84376	23576
HOPX	0	0	0	0	0	0	HOP homeobox [Source:HGNC Symbol;Acc:HGNC:24961]	chr4:57514	NM_00114	XM_01700	.	.	NP_00113	XP_006714	protein_co	ENSG0000	84525	24961
HORMAD1	0	0	0	0	0	0	HORMA domain containing 1 [Source:HGNC Symbol;Acc:HGNC:2	chr1:15067	NM_00119	XM_01151	.	.	NP_00118	XP_006711	protein_co	ENSG0000	84072	25245
HORMAD2	0	0	0.033903	0	0	0	HORMA domain containing 2 [Source:HGNC Symbol;Acc:HGNC:2	chr22:3047	NM_15251	XM_01152	.	.	NP_00131	XP_011528	protein_co	ENSG0000	150280	28383
HOXA1	1.3474	1.63646	1.84334	1.42307	1.23405	1.54315	homeobox A1 [Source:HGNC Symbol;Acc:HGNC:5099]	chr7:27132	NM_00552	.	.	.	NP_00551	.	protein_co	ENSG0000	3198	5099
HOXA10	20.4355	19.2829	18.0958	23.4749	21.7255	21.2908	homeobox A10 [Source:HGNC Symbol;Acc:HGNC:5100]	chr7:27202	NM_01895	.	.	.	NP_06182	.	protein_co	ENSG0000	3206	5100
HOXA10-HOXA9	7E-19	1.07E-16	7.75E-26	1.95E-16	7.86E-29	5.67E-43	.	chr7:27202	.	.	.	.	.	.	protein_co	ENSG0000	.	.
HOXA11	6.14391	8.01815	5.6778	8.57479	9.1809	6.50965	homeobox A11 [Source:HGNC Symbol;Acc:HGNC:5101]	chr7:27221	NM_00552	.	.	.	NP_00551	.	protein_co	ENSG0000	3207	5101
HOXA13	1.882	2.23751	1.98459	1.51765	1.79357	1.96478	homeobox A13 [Source:HGNC Symbol;Acc:HGNC:5102]	chr7:27233	NM_00052	XM_01151	.	.	NP_00051	XP_011513	protein_co	ENSG0000	3209	5102
HOXA2	0.257125	0.633454	0.400851	0.228523	0	0.160138	homeobox A2 [Source:HGNC Symbol;Acc:HGNC:5103]	chr7:27139	NM_00673	.	.	.	NP_00672	.	protein_co	ENSG0000	3199	5103
HOXA3	2.52989	3.25145	2.89599	0.712819	0.820252	0.952166	homeobox A3 [Source:HGNC Symbol;Acc:HGNC:5104]	chr7:27145	NM_03066	XM_01151	.	.	NP_10937	XP_005249	protein_co	ENSG0000	3200	5104
HOXA4	0.80654	1.10603	1.48494	0.377793	0.790914	0.428662	homeobox A4 [Source:HGNC Symbol;Acc:HGNC:5105]	chr7:27145	NM_00214	.	.	.	NP_00213	.	protein_co	ENSG0000	3201	5105
HOXA5	7.05481	7.52109	6.50725	4.5436	5.20117	5.2906	homeobox A5 [Source:HGNC Symbol;Acc:HGNC:5106]	chr7:27180	NM_01910	.	.	.	NP_06197	.	protein_co	ENSG0000	3202	5106
HOXA6	5.18201	3.69705	4.43198	2.46864	3.09379	1.92069	homeobox A6 [Source:HGNC Symbol;Acc:HGNC:5107]	chr7:27185	NM_02401	.	.	.	NP_07691	.	protein_co	ENSG0000	3203	5107
HOXA7	14.7953	12.8343	12.6745	13.7216	14.6052	13.4551	homeobox A7 [Source:HGNC Symbol;Acc:HGNC:5108]	chr7:27193	NM_00689	.	.	.	NP_00882	.	protein_co	ENSG0000	3204	5108
HOXA9	68.59	71.3785	71.9644	50.039	51.2423	64.3017	homeobox A9 [Source:HGNC Symbol;Acc:HGNC:5109]	chr7:27202	NM_15273	.	NR_02991	.	NP_68995	.	protein_co	ENSG0000	442920	5109
HOXB1	0	0	0	0	0	0	homeobox B1 [Source:HGNC Symbol;Acc:HGNC:5111]	chr17:4660	NM_00214	.	.	.	NP_00213	.	protein_co	ENSG0000	3211	5111
HOXB13	4.53644	4.5046	5.70226	4.91956	3.9539	4.26544	homeobox B13 [Source:HGNC Symbol;Acc:HGNC:5112]	chr17:4680	NM_00636	.	.	.	NP_00635	.	protein_co	ENSG0000	10481	5112
HOXB2	0.772478	1.02061	1.31523	0.563969	0.684521	1.10469	homeobox B2 [Source:HGNC Symbol;Acc:HGNC:5113]	chr17:4662	NM_00214	.	.	.	NP_00213	.	protein_co	ENSG0000	3212	5113
HOXB3	6.63248	7.30217	9.47981	6.67749	4.20002	3.89812	homeobox B3 [Source:HGNC Symbol;Acc:HGNC:5114]	chr17:4662	NM_00214	XM_01152	.	.	NP_00213	XP_005257	protein_co	ENSG0000	3213	5114
HOXB4	5.26574	6.83962	8.10118	3.66238	3.55753	2.55725	homeobox B4 [Source:HGNC Symbol;Acc:HGNC:5115]	chr17:4662	NM_02401	.	.	.	NP_07692	.	protein_co	ENSG0000	3214	5115
HOXB5	11.6163	11.5283	13.6616	8.35368	7.68001	8.88637	homeobox B5 [Source:HGNC Symbol;Acc:HGNC:5116]	chr17:4666	NM_00214	.	.	.	NP_00213	.	protein_co	ENSG0000	3215	5116
HOXB6	16.9839	17.2216	24.7984	11.2806	10.812	9.73451	homeobox B6 [Source:HGNC Symbol;Acc:HGNC:5117]	chr17:4667	NM_01895	XM_00672	.	.	NP_06182	XP_005257	protein_co	ENSG0000	3216	5117
HOXB7	34.8816	36.8352	30.425	38.8404	31.9019	25.9053	homeobox B7 [Source:HGNC Symbol;Acc:HGNC:5118]	chr17:4668	NM_00450	.	.	.	NP_00449	.	protein_co	ENSG0000	3217	5118
HOXB8	37.6486	32.6731	38.2991	13.1518	11.6416	9.99346	homeobox B8 [Source:HGNC Symbol;Acc:HGNC:5119]	chr17:4668	NM_02401	XM_00525	.	.	NP_07692	XP_005257	protein_co	ENSG0000	3218	5119
HOXB9	16.5935	18.0612	19.8311	9.60315	9.20515	15.3667	homeobox B9 [Source:HGNC Symbol;Acc:HGNC:5120]	chr17:4669	NM_02401	.	.	.	NP_07692	.	protein_co	ENSG0000	3219	5120

HOXC10	1.36901	1.16824	1.68994	2.10694	1.47591	1.97211	homeobox C10 [Source:HGNC Symbol;Acc:HGNC:5122]	chr12:5437	NM_01740	.	.	.	NP_05910	.	protein_co	ENSG0000	3226	5122
HOXC11	0.213672	0.105565	0.365019	0.289043	0.36885	0	homeobox C11 [Source:HGNC Symbol;Acc:HGNC:5123]	chr12:5436	NM_01421	.	.	.	NP_05502	.	protein_co	ENSG0000	3227	5123
HOXC12	0.025433	0	0	0	0	0.055088	homeobox C12 [Source:HGNC Symbol;Acc:HGNC:5124]	chr12:5434	NM_17386	.	.	.	NP_77627	.	protein_co	ENSG0000	3228	5124
HOXC13	2.38679	2.45208	3.58978	2.94173	3.14601	2.36271	homeobox C13 [Source:HGNC Symbol;Acc:HGNC:5125]	chr12:5433	NM_01741	.	.	.	NP_05910	.	protein_co	ENSG0000	3229	5125
HOXC4	1.39501	0.779461	1.53861	1.27578	1.01819	0.505323	homeobox C4 [Source:HGNC Symbol;Acc:HGNC:5126]	chr12:5437	NM_01462	.	.	.	NP_05543	.	protein_co	ENSG0000	3221	5126
HOXC5	1.08231	1.18814	1.04896	1.23739	1.17562	0.655288	homeobox C5 [Source:HGNC Symbol;Acc:HGNC:5127]	chr12:5437	NM_01895	.	.	.	NP_06182	.	protein_co	ENSG0000	3222	5127
HOXC6	3.87272	3.89891	3.66303	3.63993	3.53308	2.60943	homeobox C6 [Source:HGNC Symbol;Acc:HGNC:5128]	chr12:5437	NM_00450	.	.	.	NP_00449	.	protein_co	ENSG0000	3223	5128
HOXC8	1.19408	0.857944	0.489308	0.929705	1.47805	1.0294	homeobox C8 [Source:HGNC Symbol;Acc:HGNC:5129]	chr12:5437	NM_02265	.	.	.	NP_07314	.	protein_co	ENSG0000	3224	5129
HOXC9	6.65482	6.93793	5.92897	8.73466	6.36611	6.95274	homeobox C9 [Source:HGNC Symbol;Acc:HGNC:5130]	chr12:5437	NM_00689	.	.	.	NP_00882	.	protein_co	ENSG0000	3225	5130
HOXD1	0	0	0.101809	0.130024	0	0	homeobox D1 [Source:HGNC Symbol;Acc:HGNC:5132]	chr2:17705	NM_02450	.	NR_10699	.	NP_07877	.	protein_co	ENSG0000	102465802	5132
HOXD10	0.127056	0.043611	0.110062	0.046975	0	0.174879	homeobox D10 [Source:HGNC Symbol;Acc:HGNC:5133]	chr2:17698	NM_00214	.	.	.	NP_00213	.	protein_co	ENSG0000	3236	5133
HOXD11	0.088568	0.171264	0.388096	0	0.311027	0.098314	homeobox D11 [Source:HGNC Symbol;Acc:HGNC:5134]	chr2:17697	NM_02119	.	.	.	NP_06701	.	protein_co	ENSG0000	3237	5134
HOXD12	0	0.172574	0	0	0.191087	0.182669	homeobox D12 [Source:HGNC Symbol;Acc:HGNC:5135]	chr2:17696	NM_02119	.	.	.	NP_06701	.	protein_co	ENSG0000	3238	5135
HOXD13	0	0	0	0.048417	0.04923	0	homeobox D13 [Source:HGNC Symbol;Acc:HGNC:5136]	chr2:17695	NM_00052	.	.	.	NP_00051	.	protein_co	ENSG0000	3239	5136
HOXD3	0.124227	0.123264	0.070292	0.22317	0.047809	0.42995	homeobox D3 [Source:HGNC Symbol;Acc:HGNC:5137]	chr2:17702	NM_00689	XM_01151	.	.	NP_00882	XP_005246	protein_co	ENSG0000	3232	5137
HOXD4	0.504022	1.3243	0.542241	0.480424	0.364256	0.60778	homeobox D4 [Source:HGNC Symbol;Acc:HGNC:5138]	chr2:17701	NM_01462	.	.	.	NP_05543	.	protein_co	ENSG0000	3233	5138
HOXD8	5.76112	6.73383	6.78517	5.08503	3.48746	8.97883	homeobox D8 [Source:HGNC Symbol;Acc:HGNC:5139]	chr2:17699	NM_00119	.	.	.	NP_00118	.	protein_co	ENSG0000	3234	5139
HOXD9	0.064238	0	0.054562	0	0	0.070362	homeobox D9 [Source:HGNC Symbol;Acc:HGNC:5140]	chr2:17698	NM_01421	.	.	.	NP_05502	.	protein_co	ENSG0000	3235	5140
HP	0.110288	0	0	0	0	0	haptoglobin [Source:HGNC Symbol;Acc:HGNC:5141]	chr16:7208	NM_00112	XM_00525	.	.	NP_00111	XP_005255	protein_co	ENSG0000	3240	5141
HP1BP3	58.1094	60.8617	55.4122	67.4001	70.873	59.9985	heterochromatin protein 1 binding protein 3 [Source:HGNC Symb	chr1:21069	NM_01628	XM_01700	.	.	NP_05737	XP_005245	protein_co	ENSG0000	50809	24973
HPCA	0.220117	0.054465	0.190072	0.117915	0.127708	0	hippocalcin [Source:HGNC Symbol;Acc:HGNC:5144]	chr1:33352	NM_00214	XM_00527	.	.	NP_00213	XP_005270	protein_co	ENSG0000	3208	5144
HPCAL1	40.3394	38.986	46.3241	42.9335	37.6779	42.9709	hippocalcin like 1 [Source:HGNC Symbol;Acc:HGNC:5145]	chr2:10443	NM_00125	XM_01151	.	.	NP_00124	XP_005246	protein_co	ENSG0000	3241	5145
HPCAL4	2.8643	2.92017	4.04235	3.54321	3.8458	4.64231	hippocalcin like 4 [Source:HGNC Symbol;Acc:HGNC:18212]	chr1:40144	NM_00128	XM_01700	.	.	NP_00126	XP_016856	protein_co	ENSG0000	51440	18212
HPD	0.435179	0.358939	0.447677	0.257598	0.22867	0.042425	4-hydroxyphenylpyruvate dioxygenase [Source:HGNC Symbol;Acc	chr12:1222	NM_00117	.	.	.	NP_00116	.	protein_co	ENSG0000	3242	5147
HPDL	0	0	0	0	0	0	4-hydroxyphenylpyruvate dioxygenase like [Source:HGNC Symbol	chr1:45792	NM_03275	.	.	.	NP_11614	.	protein_co	ENSG0000	84842	28242
HPF1	16.6674	17.7438	17.5055	16.7261	24.093	22.524	histone PARylation factor 1 [Source:HGNC Symbol;Acc:HGNC:260	chr4:17065	NM_01786	.	.	.	NP_06033	.	protein_co	ENSG0000	54969	26051
HPGD	0.163858	0.301952	0.304448	0.053287	0.123287	0.263364	hydroxyprostaglandin dehydrogenase 15-(NAD) [Source:HGNC Sy	chr4:17541	NM_00086	XM_01153	.	.	NP_00085	XP_011530	protein_co	ENSG0000	3248	5154
HPGDS	0	0	0	0	0	0	hematopoietic prostaglandin D synthase [Source:HGNC Symbol;A	chr4:95219	NM_01448	.	.	.	NP_05530	.	protein_co	ENSG0000	27306	17890
HPN	0.204793	0.036621	0.175074	0.174771	0.059624	0.170965	hepsin [Source:HGNC Symbol;Acc:HGNC:5155]	chr19:3553	NM_00215	XM_00672	.	.	NP_00214	XP_005258	protein_co	ENSG0000	3249	5155
HPR	0	0	0	0	0	0	haptoglobin-related protein [Source:HGNC Symbol;Acc:HGNC:51	chr16:7209	NM_02099	.	.	.	NP_06627	.	protein_co	ENSG0000	3250	5156
HPRT1	32.2602	27.5325	32.5886	38.7903	43.1907	37.1057	hypoxanthine phosphoribosyltransferase 1 [Source:HGNC Symbo	chrX:13359	NM_00019	.	.	.	NP_00018	.	protein_co	ENSG0000	3251	5157
HPS1	16.3107	15.8061	15.1105	17.5923	12.3446	11.1433	HPS1, biogenesis of lysosomal organelles complex 3 subunit 1 [So	chr10:1001	NM_00019	XM_00671	.	.	NP_00018	XP_005269	protein_co	ENSG0000	3257	5163
HPS3	6.51805	6.20218	7.29074	6.26352	7.88616	7.62191	HPS3, biogenesis of lysosomal organelles complex 2 subunit 1 [So	chr3:14884	NM_00130	XM_01151	.	.	NP_00129	XP_011511	protein_co	ENSG0000	84343	15597
HPS4	12.3923	12.7082	11.3322	11.7201	12.0707	11.1081	HPS4, biogenesis of lysosomal organelles complex 3 subunit 2 [So	chr22:2684	NM_02208	XM_01702	.	.	NP_07136	XP_006724	protein_co	ENSG0000	89781	15844
HPS5	7.98542	9.23405	8.58739	9.26274	9.91768	9.48224	HPS5, biogenesis of lysosomal organelles complex 2 subunit 2 [So	chr11:1830	NM_00721	XM_01701	.	.	NP_00914	XP_011518	protein_co	ENSG0000	11234	17022
HPS6	28.7293	29.1019	29.924	29.4896	28.3005	22.0532	HPS6, biogenesis of lysosomal organelles complex 2 subunit 3 [So	chr10:1038	NM_02474	.	.	.	NP_07902	.	protein_co	ENSG0000	79803	18817
HPSE	5.24804	5.42729	5.82289	7.10166	8.78981	9.82669	heparanase [Source:HGNC Symbol;Acc:HGNC:5164]	chr4:84213	NM_00109	.	.	.	NP_00109	.	protein_co	ENSG0000	10855	5164
HPSE2	0.018355	0.038038	0.115884	0.103791	0.108267	0.094005	heparanase 2 (inactive) [Source:HGNC Symbol;Acc:HGNC:18374]	chr10:1002	NM_00116	XM_01701	.	.	NP_00115	XP_006718	protein_co	ENSG0000	60495	18374
HPX	0.665803	0.928179	0.716234	0.474537	0.349096	0.370159	hemopexin [Source:HGNC Symbol;Acc:HGNC:5171]	chr11:6452	NM_00061	XM_00525	.	.	NP_00060	XP_005252	protein_co	ENSG0000	3263	5171
HR	4.76018	4.67288	4.42758	7.40336	6.77648	5.71979	hair growth associated [Source:HGNC Symbol;Acc:HGNC:5172]	chr8:21971	NM_00514	XM_00671	.	.	NP_00513	XP_005273	protein_co	ENSG0000	55806	5172
HRAS	52.5753	42.8847	42.2823	60.2665	56.3625	38.335	HRas proto-oncogene, GTPase [Source:HGNC Symbol;Acc:HGNC:	chr11:5322	NM_00113	.	.	.	NP_00112	.	protein_co	ENSG0000	3265	5173
HRASLS	0	0	0	0	0	0	HRAS like suppressor [Source:HGNC Symbol;Acc:HGNC:14922]	chr3:19295	NM_02038	.	.	.	NP_06511	.	protein_co	ENSG0000	57110	14922
HRASLS2	0	0.109628	0.374654	0	0	0.114755	HRAS like suppressor 2 [Source:HGNC Symbol;Acc:HGNC:17824]	chr11:6332	NM_01787	.	.	.	NP_06034	.	protein_co	ENSG0000	54979	17824
HRASLS5	0	0	0	0	0	0	HRAS like suppressor family member 5 [Source:HGNC Symbol;Acc	chr11:6322	NM_00114	XM_01154	.	.	NP_00114	XP_011543	protein_co	ENSG0000	117245	24978
HRC	0.030525	0.030556	0.027436	0	0	0	histidine rich calcium binding protein [Source:HGNC Symbol;Acc:	chr19:4965	NM_00215	.	.	.	NP_00214	XP_016882	protein_co	ENSG0000	3270	5178
HRCT1	0.087097	0.257764	0.60438	0.369471	0.204523	0.192502	histidine rich carboxyl terminus 1 [Source:HGNC Symbol;Acc:HGN	chr9:35906	NM_00103	.	.	.	NP_00103	.	protein_co	ENSG0000	646962	33872
HRG	0.032769	0.273099	0	0.072711	0.110761	0.302933	histidine rich glycoprotein [Source:HGNC Symbol;Acc:HGNC:5181	chr3:18638	NM_00041	XM_00524	.	.	NP_00040	XP_005247	protein_co	ENSG0000	3273	5181
HRH1	3.87966	4.22624	3.32393	3.33202	3.59636	3.63293	histamine receptor H1 [Source:HGNC Symbol;Acc:HGNC:5182]	chr3:11178	NM_00086	XM_01700	.	.	NP_00085	XP_011531	protein_co	ENSG0000	3269	5182
HRH2	0	0	0	0.019982	0	0	histamine receptor H2 [Source:HGNC Symbol;Acc:HGNC:5183]	chr5:17508	NM_00113	XM_01153	.	.	NP_00112	XP_005265	protein_co	ENSG0000	3274	5183
HRH3	0	0	0	0	0	0	histamine receptor H3 [Source:HGNC Symbol;Acc:HGNC:5184]	chr20:6079	NM_00723	XM_00526	.	.	NP_00916	XP_005260	protein_co	ENSG0000	11255	5184
HRH4	0.104954	0	0.024405	0.032065	0.09436	0.207717	histamine receptor H4 [Source:HGNC Symbol;Acc:HGNC:17383]	chr18:2204	NM_00114	.	.	.	NP_00113	.	protein_co	ENSG0000	59340	17383
HRK	1.38157	1.48632	1.06917	0.771573	0.653087	1.00355	harakiri, BCL2 interacting protein [Source:HGNC Symbol;Acc:HGN	chr12:1172	NM_00380	.	.	.	NP_00379	XP_016875	protein_co	ENSG0000	8739	5185
HRNR	0	0	0.0207	0	0	0	hornerin [Source:HGNC Symbol;Acc:HGNC:20846]	chr1:15218	NM_00100	.	.	.	NP_00100	.	protein_co	ENSG0000	388697	20846
HS1BP3	9.12089	9.03366	9.67349	10.3497	9.04924	8.65183	HCLS1 binding protein 3 [Source:HGNC Symbol;Acc:HGNC:24979]	chr2:20817	NM_02246	XM_01700	.	.	NP_07190	XP_016860	protein_co	ENSG0000	64342	24979
HS2ST1	10.4332	11.5271	13.8683	9.45678	10.9558	14.2303	heparan sulfate 2-O-sulfotransferase 1 [Source:HGNC Symbol;Acc	chr1:87380	NM_00113	.	.	.	NP_00112	.	protein_co	ENSG0000	9653	5193
HS3ST1	9.23408	9.36331	8.43105	14.3148	13.2143	12.2021	heparan sulfate-glucosamine 3-sulfotransferase 1 [Source:HGNC	chr4:11394	NM_00511	XM_01151	.	.	NP_00510	XP_011512	protein_co	ENSG0000	9957	5194
HS3ST2	0	0	0.035314	0	0	0	heparan sulfate-glucosamine 3-sulfotransferase 2 [Source:HGNC	chr16:2282	NM_00604	.	.	.	NP_00603	.	protein_co	ENSG0000	9956	5195
HS3ST3A1	0	0	0.107395	0.266716	0	0.138968	heparan sulfate-glucosamine 3-sulfotransferase 3A1 [Source:HGN	chr17:1339	NM_00604	.	.	.	NP_00603	XP_016880	protein_co	ENSG0000	9955	5196
HS3ST3B1	0.579739	0.695538	0.65415	0.972623	1.12072	1.08479	heparan sulfate-glucosamine 3-sulfotransferase 3B1 [Source:HGN	chr17:1420	NM_00604	.	.	.	NP_00603	.	protein_co	ENSG0000	9953	5198

HS3ST4	0	0	0	0	0	0	heparan sulfate-glucosamine 3-sulfotransferase 4 [Source:HGNC	chr16:2570	NM_00604	.	.	.	NP_00603	.	protein_co	ENSG0000	9951	5200
HS3ST5	0	0.024983	0	0	0	0	heparan sulfate-glucosamine 3-sulfotransferase 5 [Source:HGNC	chr6:11437	NM_15361	XM_01153	.	.	NP_705840	XP_006715	protein_co	ENSG0000	222537	19419
HS3ST6	0.116831	0	0.391783	0.122162	0	0	heparan sulfate-glucosamine 3-sulfotransferase 6 [Source:HGNC	chr16:1961	NM_00100	.	.	.	NP_00100	.	protein_co	ENSG0000	64711	14178
HS6ST1	14.8012	14.2831	14.9998	17.0164	15.5741	11.5465	heparan sulfate 6-O-sulfotransferase 1 [Source:HGNC Symbol;Acc	chr2:12902	NM_00480	.	.	.	NP_00479	.	protein_co	ENSG0000	9394	5201
HS6ST2	15.1701	15.946	15.4575	14.3389	13.725	9.93949	heparan sulfate 6-O-sulfotransferase 2 [Source:HGNC Symbol;Acc	chrX:13176	NM_00107	XM_01702	.	.	NP_00107	XP_005262	protein_co	ENSG0000	90161	19133
HS6ST3	0.042905	0.057116	0.149709	0.024324	0.059554	0.053061	heparan sulfate 6-O-sulfotransferase 3 [Source:HGNC Symbol;Acc	chr13:9674	NM_15345	.	.	.	NP_70315	.	protein_co	ENSG0000	266722	19134
hsa-mir-150	0	0.096087	0	0	0	0	hsa-mir-150 [Source:miRBase;Acc:MI0000479]	chr19:5000	.	.	.	.	.	.	protein_co	ENSG0000	.	.
HSBP1	108.695	108.551	97.6193	128.798	96.8376	101.813	heat shock factor binding protein 1 [Source:HGNC Symbol;Acc:HG	chr16:8384	NM_00153	.	.	.	NP_00152	.	protein_co	ENSG0000	3281	5203
HSBP1L1	31.8561	27.5852	26.5832	29.0108	24.1024	25.6136	heat shock factor binding protein 1 like 1 [Source:HGNC Symbol;A	chr18:7772	NM_00113	.	.	.	NP_00112	.	protein_co	ENSG0000	440498	37243
HSCB	7.67301	10.1482	6.77612	7.49992	7.42939	7.65449	HscB mitochondrial iron-sulfur cluster cochaperone [Source:HGN	chr22:2913	NM_00131	XM_01702	.	.	NP_00130	XP_005261	protein_co	ENSG0000	150274	28913
HSD11B1	0	0	0	0	0	0	hydroxysteroid 11-beta dehydrogenase 1 [Source:HGNC Symbol;A	chr1:20985	NM_00120	.	.	.	NP_00119	.	protein_co	ENSG0000	3290	5208
HSD11B1L	3.89482	2.19693	3.84839	3.02824	2.54951	2.77127	hydroxysteroid 11-beta dehydrogenase 1 like [Source:HGNC Sym	chr19:5674	NM_00126	.	.	.	NP_00125	.	protein_co	ENSG0000	374875	30419
HSD11B2	5.87663	5.09783	7.0546	5.54027	6.12106	4.48517	hydroxysteroid 11-beta dehydrogenase 2 [Source:HGNC Symbol;A	chr16:6746	NM_00019	.	.	.	NP_00018	.	protein_co	ENSG0000	3291	5209
HSD17B1	1.14665	1.18515	1.84427	1.00938	0.789057	1.07064	hydroxysteroid 17-beta dehydrogenase 1 [Source:HGNC Symbol;A	chr17:4070	NM_00041	XM_01152	.	.	NP_00040	XP_005257	protein_co	ENSG0000	3292	5210
HSD17B10	98.9934	102.821	83.1486	131.614	100.802	69.4382	hydroxysteroid 17-beta dehydrogenase 10 [Source:HGNC Symbol	chrX:53458	NM_00103	.	.	.	NP_00103	.	protein_co	ENSG0000	3028	4800
HSD17B11	32.314	29.379	24.1937	34.1037	30.0882	36.3418	hydroxysteroid 17-beta dehydrogenase 11 [Source:HGNC Symbol	chr4:88257	NM_01624	XM_00671	.	.	NP_05732	XP_006714	protein_co	ENSG0000	51170	22960
HSD17B12	29.9301	29.6982	28.8346	32.4347	31.6624	37.9429	hydroxysteroid 17-beta dehydrogenase 12 [Source:HGNC Symbol	chr11:4370	NM_01614	XM_01152	.	.	NP_05722	XP_011518	protein_co	ENSG0000	51144	18646
HSD17B13	0.243883	0.120292	0.211889	0.317913	0.269697	0.479508	hydroxysteroid 17-beta dehydrogenase 13 [Source:HGNC Symbol	chr4:88224	NM_00113	.	.	.	NP_00112	.	protein_co	ENSG0000	345275	18685
HSD17B14	1.36434	1.12742	0.678897	1.103	0.839249	1.04017	hydroxysteroid 17-beta dehydrogenase 14 [Source:HGNC Symbol	chr19:4931	NM_01624	XM_00525	.	.	NP_05733	XP_005259	protein_co	ENSG0000	51171	23238
HSD17B2	0.057192	0	0	0	0	0	hydroxysteroid 17-beta dehydrogenase 2 [Source:HGNC Symbol;A	chr16:8206	NM_00215	.	.	.	NP_00214	.	protein_co	ENSG0000	3294	5211
HSD17B3	0.168591	0	0.243455	0.156898	0.095169	0	hydroxysteroid 17-beta dehydrogenase 3 [Source:HGNC Symbol;A	chr9:98997	NM_00019	XM_01151	.	.	NP_00018	XP_011516	protein_co	ENSG0000	3293	5212
HSD17B4	34.3518	33.2052	35.7149	34.3271	34.138	38.7061	hydroxysteroid 17-beta dehydrogenase 4 [Source:HGNC Symbol;A	chr5:11878	NM_00041	.	.	.	NP_00040	.	protein_co	ENSG0000	3295	5213
HSD17B6	1.6237	1.70784	1.62041	1.8884	1.68173	1.46998	hydroxysteroid 17-beta dehydrogenase 6 [Source:HGNC Symbol;A	chr12:5712	NM_00372	XM_01153	.	.	NP_00371	XP_005269	protein_co	ENSG0000	8630	23316
HSD17B7	16.3785	14.7404	15.8056	16.4492	16.7616	18.9233	hydroxysteroid 17-beta dehydrogenase 7 [Source:HGNC Symbol;A	chr1:16276	NM_00130	.	.	.	NP_00129	.	protein_co	ENSG0000	51478	5215
HSD17B8	13.2293	13.243	9.54697	13.7989	11.8046	11.2597	hydroxysteroid 17-beta dehydrogenase 8 [Source:HGNC Symbol;A	chr6:33172	NM_01423	.	.	.	NP_05504	.	protein_co	ENSG0000	7923	3554
HSD3B1	0	0	0	0	0	0	hydroxy-delta-5-steroid dehydrogenase, 3 beta- and steroid delta	chr1:12004	NM_00086	XM_01700	.	.	NP_00085	XP_016856	protein_co	ENSG0000	3283	5217
HSD3B2	0	0	0	0	0	0	hydroxy-delta-5-steroid dehydrogenase, 3 beta- and steroid delta	chr1:11995	NM_00019	.	.	.	NP_00018	.	protein_co	ENSG0000	3284	5218
HSD3B7	5.96767	6.81924	5.94472	9.57314	8.71167	4.61058	hydroxy-delta-5-steroid dehydrogenase, 3 beta- and steroid delta	chr16:3099	NM_00114	XM_01702	.	.	NP_00113	XP_005255	protein_co	ENSG0000	80270	18324
HSDL1	20.3494	21.1807	21.0534	18.7592	18.3301	21.4815	hydroxysteroid dehydrogenase like 1 [Source:HGNC Symbol;Acc:H	chr16:8415	NM_00114	XM_00525	.	.	NP_00113	XP_005256	protein_co	ENSG0000	83693	16475
HSDL2	14.3976	15.2774	18.6376	17.4289	14.9729	16.6176	hydroxysteroid dehydrogenase like 2 [Source:HGNC Symbol;Acc:H	chr9:11514	NM_00119	.	.	.	NP_00118	.	protein_co	ENSG0000	84263	18572
HSF1	50.8862	50.0681	48.0055	54.0744	44.8415	41.7283	heat shock transcription factor 1 [Source:HGNC Symbol;Acc:HGN	chr8:14551	NM_00552	XM_01701	.	.	NP_00551	XP_005272	protein_co	ENSG0000	3297	5224
HSF2	12.4243	13.577	15.1645	15.3357	16.018	15.7991	heat shock transcription factor 2 [Source:HGNC Symbol;Acc:HGN	chr6:12272	NM_00113	.	.	.	NP_00112	.	protein_co	ENSG0000	3298	5225
HSF2BP	0.329459	0.127163	0.249227	0.458097	0.187528	0.127281	heat shock transcription factor 2 binding protein [Source:HGNC S	chr21:4494	NM_00703	XM_01152	.	.	NP_00896	XP_011527	protein_co	ENSG0000	11077	5226
HSF4	3.75243	2.14199	3.99557	2.99899	2.01542	2.87285	heat shock transcription factor 4 [Source:HGNC Symbol;Acc:HGN	chr16:6719	NM_00104	.	.	.	NP_00103	.	protein_co	ENSG0000	3299	5227
HSF5	0	0	0	0.025084	0	0	heat shock transcription factor 5 [Source:HGNC Symbol;Acc:HGN	chr17:5649	NM_00108	XM_01152	.	.	NP_00107	XP_011522	protein_co	ENSG0000	124535	26862
HSFX1	0	0	0	0	0	0	heat shock transcription factor family, X-linked 1 [Source:HGNC S	chrX:14885	NM_01615	.	.	.	NP_05723	.	protein_co	ENSG0000	100506164	29603
HSFX2	0	0	0	0	0	0	heat shock transcription factor family, X-linked 2 [Source:HGNC S	chrX:14867	NM_00116	.	.	.	NP_00115	.	protein_co	ENSG0000	100130086	32701
HSFY1	0	0	0	0	0	0	heat shock transcription factor, Y-linked 1 [Source:HGNC Symbol;	chrY:20708	NM_03310	XM_00526	.	.	NP_14909	XP_005262	protein_co	ENSG0000	159119	18568
HSFY2	0	0	0	0	0	0	heat shock transcription factor, Y-linked 2 [Source:HGNC Symbol;	chrY:20893	NM_00100	XM_00526	NR_00350	.	NP_00100	XP_005262	protein_co	ENSG0000	159119	23950
HSH2D	9.17069	9.5066	9.4043	6.6431	6.1065	6.62285	hematopoietic SH2 domain containing [Source:HGNC Symbol;Acc	chr19:1622	NM_03285	XM_01152	.	.	NP_11624	XP_011526	protein_co	ENSG0000	84941	24920
HSP90AA1	1434.58	1348.3	1058.72	2075.2	2148.03	1477.83	heat shock protein 90 alpha family class A member 1 [Source:HGI	chr14:1025	NM_00101	XM_01153	.	.	NP_00101	XP_011535	protein_co	ENSG0000	3320	5253
HSP90AB1	1883.13	1876.25	1416.12	2093.26	1946.56	1417.42	heat shock protein 90 alpha family class B member 1 [Source:HGI	chr6:44213	NM_00127	.	.	.	NP_00125	.	protein_co	ENSG0000	3326	5258
HSP90B1	1165.68	1080.35	462.961	907.495	1031.73	976.463	heat shock protein 90 beta family member 1 [Source:HGNC Symb	chr12:1043	NM_00329	.	NR_03742	.	NP_00329	.	protein_co	ENSG0000	100500842	12028
HSPA12A	8.90552	9.45343	12.0023	8.76591	10.7803	9.61888	heat shock protein family A (Hsp70) member 12A [Source:HGNC	chr10:1184	NM_02501	XM_01153	.	.	NP_07929	XP_005269	protein_co	ENSG0000	259217	19022
HSPA12B	0	0	0	0	0	0	heat shock protein family A (Hsp70) member 12B [Source:HGNC	chr20:3713	NM_00119	XM_01702	.	.	NP_00118	XP_005260	protein_co	ENSG0000	116835	16193
HSPA13	17.7781	17.4996	12.3188	14.7798	14.9285	24.8461	heat shock protein family A (Hsp70) member 13 [Source:HGNC Sy	chr21:1574	NM_00694	.	.	.	NP_00887	.	protein_co	ENSG0000	6782	11375
HSPA14	27.0218	24.1803	22.7439	25.6462	29.6858	23.8176	heat shock protein family A (Hsp70) member 14 [Source:HGNC Sy	chr10:1486	NM_00127	.	.	.	NP_00126	.	protein_co	ENSG0000	51182	29526
HSPA1A	2.27741	2.25186	1.40452	3.72035	3.19183	1.20776	heat shock protein family A (Hsp70) member 1A [Source:HGNC Sy	chr6:31783	NM_00534	.	.	.	NP_00533	.	protein_co	ENSG0000	3303	5232
HSPA1B	54.547	54.7471	32.5189	88.8335	77.7521	25.3258	heat shock protein family A (Hsp70) member 1B [Source:HGNC Sy	chr6:31795	NM_00534	.	.	.	NP_00533	.	protein_co	ENSG0000	3304	5233
HSPA1L	0.341495	0.351509	0.297644	0.080752	0.277668	0.152479	heat shock protein family A (Hsp70) member 1 like [Source:HGNC	chr6:31777	NM_00552	XM_01151	.	.	NP_00551	XP_005249	protein_co	ENSG0000	3305	5234
HSPA2	1.3695	1.62246	1.69304	1.64776	1.10958	1.6287	heat shock protein family A (Hsp70) member 2 [Source:HGNC Syn	chr14:6500	NM_02197	.	.	.	NP_06881	.	protein_co	ENSG0000	3306	5235
HSPA4	92.5876	96.0079	95.5385	107.419	119.242	94.01	heat shock protein family A (Hsp70) member 4 [Source:HGNC Syn	chr5:13238	NM_00215	.	.	.	NP_00214	.	protein_co	ENSG0000	3308	5237
HSPA4L	11.0714	11.7812	12.5107	11.9935	16.5061	11.1607	heat shock protein family A (Hsp70) member 4 like [Source:HGNC	chr4:12870	NM_01427	XM_01153	.	.	NP_00130	XP_011530	protein_co	ENSG0000	22824	17041
HSPA5	657.017	658.851	253.667	606.587	588.727	490.814	heat shock protein family A (Hsp70) member 5 [Source:HGNC Syn	chr9:12799	NM_00534	.	.	.	NP_00533	.	protein_co	ENSG0000	3309	5238
HSPA6	0	0.142697	0	0.217381	0.033735	0.031821	heat shock protein family A (Hsp70) member 6 [Source:HGNC Syn	chr1:16149	NM_00215	.	.	.	NP_00214	.	protein_co	ENSG0000	3310	5239
HSPA8	1353.41	1306.65	1068.21	1803.62	1641.67	721.63	heat shock protein family A (Hsp70) member 8 [Source:HGNC Syn	chr11:1229	NM_00659	XM_01154	.	.	NP_00658	XP_011541	protein_co	ENSG0000	3312	5241
HSPA9	224.97	220.112	177.174	236.558	228.572	269.124	heat shock protein family A (Hsp70) member 9 [Source:HGNC Syn	chr5:13789	NM_00413	.	.	.	NP_00412	.	protein_co	ENSG0000	3313	5244
HSPB1	576.788	593.274	405.317	819.716	612.709	469.656	heat shock protein family B (small) member 1 [Source:HGNC Sym	chr7:75931	NM_00154	.	.	.	NP_00153	.	protein_co	ENSG0000	3315	5246
HSPB11	33.1424	25.0987	33.8202	38.8733	45.955	36.5303	heat shock protein family B (small) member 11 [Source:HGNC Syn	chr1:54382	NM_01612	XM_00527	.	.	NP_00130	XP_005270	protein_co	ENSG0000	51668	25019

HSPB2	0	0.133721	0.115734	0	0	0.073787	heat shock protein family B (small) member 2 [Source:HGNC Sym	chr11:1117	NM_00154	.	.	.	NP_00153	.	protein_co	ENSG0000	3316	5247
HSPB3	0	0	0	0	0	0	heat shock protein family B (small) member 3 [Source:HGNC Sym	chr5:53751	NM_00630	.	.	.	NP_00629	.	protein_co	ENSG0000	8988	5248
HSPB6	0.070146	0.13892	0.059764	0.074799	0.272502	0.22936	heat shock protein family B (small) member 6 [Source:HGNC Sym	chr19:3624	NM_14461	.	.	.	NP_65321	.	protein_co	ENSG0000	126393	26511
HSPB7	0	0	0.032103	0	0	0	heat shock protein family B (small) member 7 [Source:HGNC Sym	chr1:1634	NM_01442	XM_01154	.	.	NP_05523	XP_01153	protein_co	ENSG0000	27129	5249
HSPB8	6.67566	4.8198	4.13656	7.14549	6.35131	5.33331	heat shock protein family B (small) member 8 [Source:HGNC Sym	chr12:1196	NM_01436	.	.	.	NP_05518	.	protein_co	ENSG0000	26353	30171
HSPB9	0.04589	0	0	0	0	0	heat shock protein family B (small) member 9 [Source:HGNC Sym	chr17:4026	NM_03319	.	.	.	NP_14997	.	protein_co	ENSG0000	94086	30589
HSPBAP1	3.63843	4.12578	3.67592	3.87278	4.28463	4.07643	HSPB1 associated protein 1 [Source:HGNC Symbol;Acc:HGNC:163	chr3:12245	NM_02461	XM_01151	.	.	NP_07888	XP_011511	protein_co	ENSG0000	79663	16389
HSPBP1	40.5658	40.0034	32.1306	47.8451	39.4131	34.75	HSPA (Hsp70) binding protein 1 [Source:HGNC Symbol;Acc:HGNC	chr19:5577	NM_00113	XM_01702	.	.	NP_00112	XP_005258	protein_co	ENSG0000	23640	24989
HSPD1	726.194	687.241	638.102	842.765	923.555	722.724	heat shock protein family D (Hsp60) member 1 [Source:HGNC Syr	chr2:19835	NM_00215	XM_00524	.	.	NP_00214	XP_005246	protein_co	ENSG0000	3329	5261
HSPE1	755.079	716.935	496.129	874.371	738.611	488.645	heat shock protein family E (Hsp10) member 1 [Source:HGNC Syn	chr2:19835	NM_00215	.	.	.	NP_00214	.	protein_co	ENSG0000	3336	5269
HSPE1-MOB4	8.85008	8.5849	8.43819	5.0989	12.3584	8.38926	HSPE1-MOB4 readthrough [Source:HGNC Symbol;Acc:HGNC:491	chr2:19835	NM_00120	.	.	.	NP_00118	.	protein_co	ENSG0000	100529241	49184
HSPG2	5.70377	6.1615	9.66974	3.54925	3.77559	4.83573	heparan sulfate proteoglycan 2 [Source:HGNC Symbol;Acc:HGNC:	chr1:22138	NM_00129	XM_01700	.	.	NP_00127	XP_01153	protein_co	ENSG0000	3339	5273
HSPH1	88.9576	81.7367	55.3276	127.484	160.798	72.4278	heat shock protein family H (Hsp110) member 1 [Source:HGNC Sy	chr13:3170	NM_00128	XM_01153	.	.	NP_00127	XP_005266	protein_co	ENSG0000	10808	16969
HTATIP2	48.7954	48.0796	39.1963	52.9294	45.5736	52.8695	HIV-1 Tat interactive protein 2 [Source:HGNC Symbol;Acc:HGNC:1	chr11:2038	NM_00109	.	.	.	NP_00109	.	protein_co	ENSG0000	10553	16637
HTATSF1	37.9131	38.7025	32.3606	41.349	42.3916	33.5654	HIV-1 Tat specific factor 1 [Source:HGNC Symbol;Acc:HGNC:5276	chrX:13557	NM_00116	XM_00526	.	.	NP_00115	XP_005262	protein_co	ENSG0000	27336	5276
HTN1	0	0	0	0	0	0	histatin 1 [Source:HGNC Symbol;Acc:HGNC:5283]	chr4:70916	NM_00215	.	.	.	NP_00215	.	protein_co	ENSG0000	3346	5283
HTN3	0	0	0	0	0	0	histatin 3 [Source:HGNC Symbol;Acc:HGNC:5284]	chr4:70894	NM_00020	.	.	.	NP_00019	.	protein_co	ENSG0000	3347	5284
HTR1A	0	0	0	0	0	0	5-hydroxytryptamine receptor 1A [Source:HGNC Symbol;Acc:HGN	chr5:63256	NM_00052	.	.	.	NP_00051	.	protein_co	ENSG0000	3350	5286
HTR1B	0.192276	0.67273	0.411507	0.261097	0.110811	0.207047	5-hydroxytryptamine receptor 1B [Source:HGNC Symbol;Acc:HGN	chr6:78171	NM_00086	.	.	.	NP_00085	.	protein_co	ENSG0000	3351	5287
HTR1D	3.83641	4.33116	2.97875	5.06984	5.54974	3.43412	5-hydroxytryptamine receptor 1D [Source:HGNC Symbol;Acc:HGN	chr1:23518	NM_00086	.	.	.	NP_00085	.	protein_co	ENSG0000	3352	5289
HTR1E	0	0	0	0	0	0	5-hydroxytryptamine receptor 1E [Source:HGNC Symbol;Acc:HGN	chr6:87647	NM_00086	XM_01153	.	.	NP_00085	XP_011534	protein_co	ENSG0000	3354	5291
HTR1F	0	0	0	0	0	0	5-hydroxytryptamine receptor 1F [Source:HGNC Symbol;Acc:HGN	chr3:88039	NM_00086	XM_01153	.	.	NP_00085	XP_005264	protein_co	ENSG0000	3355	5292
HTR2A	0	0	0	0	0	0	5-hydroxytryptamine receptor 2A [Source:HGNC Symbol;Acc:HGN	chr13:4740	NM_00062	.	.	.	NP_00061	.	protein_co	ENSG0000	3356	5293
HTR2B	0	0	0	0	0	0	5-hydroxytryptamine receptor 2B [Source:HGNC Symbol;Acc:HGN	chr2:23192	NM_00086	XM_01151	.	.	NP_00085	XP_006712	protein_co	ENSG0000	3357	5294
HTR2C	0	0	0	0	0	0	5-hydroxytryptamine receptor 2C [Source:HGNC Symbol;Acc:HGN	chrX:11381	NM_00086	.	.	.	NP_00085	.	protein_co	ENSG0000	3358	5295
HTR3A	0.036537	0.037098	0.03151	0.11989	0.038007	0.077364	5-hydroxytryptamine receptor 3A [Source:HGNC Symbol;Acc:HGN	chr11:1138	NM_00086	.	.	.	NP_00086	.	protein_co	ENSG0000	3359	5297
HTR3B	0	0	0	0	0	0	5-hydroxytryptamine receptor 3B [Source:HGNC Symbol;Acc:HGN	chr11:1137	NM_00602	XM_01154	.	.	NP_00601	XP_011541	protein_co	ENSG0000	9177	5298
HTR3C	0	0	0	0	0	0	5-hydroxytryptamine receptor 3C [Source:HGNC Symbol;Acc:HGN	chr3:18377	NM_13077	.	.	.	NP_57012	.	protein_co	ENSG0000	170572	24003
HTR3D	0	0	0	0	0	0.093286	5-hydroxytryptamine receptor 3D [Source:HGNC Symbol;Acc:HGN	chr3:18374	NM_00114	XM_01700	.	.	NP_00113	XP_016861	protein_co	ENSG0000	200909	24004
HTR3E	0	0	0	0	0	0	5-hydroxytryptamine receptor 3E [Source:HGNC Symbol;Acc:HGN	chr3:18381	NM_00125	.	.	.	NP_00124	.	protein_co	ENSG0000	285242	24005
HTR4	0	0	0	0	0	0	5-hydroxytryptamine receptor 4 [Source:HGNC Symbol;Acc:HGN	chr5:14783	NM_00087	.	.	.	NP_00086	.	protein_co	ENSG0000	3360	5299
HTR5A	0	0	0	0	0	0	5-hydroxytryptamine receptor 5A [Source:HGNC Symbol;Acc:HGN	chr7:15486	NM_02401	.	.	.	NP_07691	.	protein_co	ENSG0000	3361	5300
HTR6	0.571422	0.547799	0.597947	0.074957	0.084439	0.491164	5-hydroxytryptamine receptor 6 [Source:HGNC Symbol;Acc:HGN	chr1:19991	NM_00087	.	.	.	NP_00086	.	protein_co	ENSG0000	3362	5301
HTR7	9.97055	8.55582	10.4953	7.95962	8.98172	9.3804	5-hydroxytryptamine receptor 7 [Source:HGNC Symbol;Acc:HGN	chr10:9250	NM_00087	.	.	.	NP_00086	.	protein_co	ENSG0000	3363	5302
HTRA1	0	0.123535	0.070457	0.044724	0.047046	0.086927	HtrA serine peptidase 1 [Source:HGNC Symbol;Acc:HGNC:9476]	chr10:1242	NM_00277	.	.	.	NP_00276	.	protein_co	ENSG0000	5654	9476
HTRA2	21.2902	19.7858	20.48	22.3502	16.4472	15.6572	HtrA serine peptidase 2 [Source:HGNC Symbol;Acc:HGNC:14348]	chr2:74753	NM_01324	XM_00526	.	.	NP_00130	.	protein_co	ENSG0000	27429	14348
HTRA3	0.069099	0.067455	0	0.07373	0.040286	0.038605	HtrA serine peptidase 3 [Source:HGNC Symbol;Acc:HGNC:30406]	chr4:82714	NM_00129	XM_01151	.	.	NP_00128	XP_011511	protein_co	ENSG0000	94031	30406
HTRA4	0	0	0	0	0	0	HtrA serine peptidase 4 [Source:HGNC Symbol;Acc:HGNC:26909]	chr8:38831	NM_15369	XM_01154	.	.	NP_71015	.	protein_co	ENSG0000	203100	26909
HTT	6.19479	6.96115	7.9999	6.21161	6.55097	6.13869	huntingtin [Source:HGNC Symbol;Acc:HGNC:4851]	chr4:30764	NM_00211	.	.	.	NP_00210	.	protein_co	ENSG0000	3064	4851
HUG1	0	0	0	0	0	0	.	chr10:1028	.	.	.	.	.	.	protein_co	ENSG0000	.	
HUNK	0	0	0.008624	0	0	0	hormonally up-regulated Neu-associated kinase [Source:HGNC Sy	chr21:3324	NM_01458	XM_01152	.	.	NP_05540	XP_011527	protein_co	ENSG0000	30811	13326
HUS1	8.0482	7.07175	8.65017	6.6426	6.96937	5.62341	HUS1 checkpoint clamp component [Source:HGNC Symbol;Acc:H	chr7:48002	NM_00450	XM_01701	.	.	NP_00449	XP_016867	protein_co	ENSG0000	3364	5309
HUS1B	0.200883	0.198787	0	0	0.46533	0.325849	HUS1 checkpoint clamp component B [Source:HGNC Symbol;Acc:	chr6:48513	NM_14895	.	.	.	NP_68376	.	protein_co	ENSG0000	135458	16485
HUWE1	31.4796	35.4041	33.3546	31.9748	33.0578	32.3181	HECT, UBA and WWE domain containing 1, E3 ubiquitin protein li	chrX:53559	NM_03140	XM_01153	.	.	NP_11358	XP_005262	protein_co	ENSG0000	10075	30892
HVCN1	0.142012	0.057725	0	0.052191	0.129733	0.226444	hydrogen voltage gated channel 1 [Source:HGNC Symbol;Acc:HG	chr12:1110	NM_00104	XM_01702	.	.	NP_00103	XP_005254	protein_co	ENSG0000	84329	28240
HYAL1	1.60768	2.06126	3.99734	2.45026	2.84235	3.154	hyaluronoglucosaminidase 1 [Source:HGNC Symbol;Acc:HGNC:53	chr3:50337	NM_03315	XM_01153	.	.	NP_14934	XP_011531	protein_co	ENSG0000	3373	5320
HYAL2	41.1366	38.538	42.8948	44.7502	39.8181	31.4965	hyaluronoglucosaminidase 2 [Source:HGNC Symbol;Acc:HGNC:53	chr3:50355	NM_00377	XM_00526	.	.	NP_00376	XP_005265	protein_co	ENSG0000	8692	5321
HYAL3	4.13533	4.18216	4.76417	4.94776	3.82888	4.6085	hyaluronoglucosaminidase 3 [Source:HGNC Symbol;Acc:HGNC:53	chr3:50316	NM_00120	.	.	.	NP_00118	.	protein_co	ENSG0000	8372	5322
HYAL4	0	0	0	0	0	0.050801	hyaluronoglucosaminidase 4 [Source:HGNC Symbol;Acc:HGNC:53	chr7:12348	NM_01226	XM_01151	.	.	NP_03640	XP_011514	protein_co	ENSG0000	23553	5323
HYDIN	0	0.044178	0	0	0	0	HYDIN, axonemal central pair apparatus protein [Source:HGNC Sy	chr16:7084	NM_00119	XM_01152	.	.	NP_00118	XP_006721	protein_co	ENSG0000	54768	19368
HYI	6.14499	5.83705	5.78052	3.34994	3.91848	2.57786	hydroxypyruvate isomerase (putative) [Source:HGNC Symbol;Acc	chr1:43855	NM_00119	XM_01154	.	.	NP_00117	XP_005271	protein_co	ENSG0000	81888	26948
HYKK	3.82998	3.67651	4.86805	5.29875	5.14432	3.26418	hydroxylysine kinase [Source:HGNC Symbol;Acc:HGNC:34403]	chr15:7879	NM_00101	.	.	.	NP_00101	XP_011519	protein_co	ENSG0000	123688	34403
HYLS1	14.6337	12.613	11.6436	17.1669	17.5898	18.1602	HYLS1, centriolar and ciliogenesis associated [Source:HGNC Symb	chr11:1257	NM_00113	XM_01154	.	.	NP_00112	XP_005271	protein_co	ENSG0000	219844	26558
HYOU1	141.982	144.352	69.8434	129.54	118.992	110.608	hypoxia up-regulated 1 [Source:HGNC Symbol;Acc:HGNC:16931]	chr11:1189	NM_00113	XM_01701	.	.	NP_00112	XP_005271	protein_co	ENSG0000	10525	16931
HYPK	77.0494	70.0564	51.8534	97.2689	68.4973	49.4607	huntingtin interacting protein K [Source:HGNC Symbol;Acc:HGNC	chr15:4403	NM_01640	.	.	.	NP_00118	.	protein_co	ENSG0000	25764	18418
HYPM	0	0	0	0	0	0	huntingtin interacting protein M [Source:HGNC Symbol;Acc:HGN	chrX:37208	NM_01227	.	.	.	NP_03640	.	protein_co	ENSG0000	25763	18417
IAH1	15.7688	14.4456	17.4341	17.2647	12.5723	18.0712	isoamyl acetate-hydrolyzing esterase 1 homolog [Source:HGNC S	chr2:96139	NM_00103	XM_00524	.	.	NP_00103	XP_016859	protein_co	ENSG0000	285148	27696
IAPP	0	0.055846	0	0	0	0	islet amyloid polypeptide [Source:HGNC Symbol;Acc:HGNC:5329]	chr12:2141	NM_00041	.	.	.	NP_00040	.	protein_co	ENSG0000	3375	5329
IARS	88.9262	89.1376	76.0339	78.8022	89.1283	127.177	isoleucyl-tRNA synthetase [Source:HGNC Symbol;Acc:HGNC:5330	chr9:94972	NM_00216	.	.	.	NP_00215	.	protein_co	ENSG0000	3376	5330

IARS2	52.9421	53.0145	65.6068	53.4799	59.445	62.5372	isoleucyl-tRNA synthetase 2, mitochondrial [Source:HGNC Symbo	chr1:22026	NM_01806	.	.	.	NP_060530	.	protein_co	ENSG0000(	55699	29685
IBA57	2.98749	2.66983	3.23388	3.37818	3.2228	2.72188	IBA57 homolog, iron-sulfur cluster assembly [Source:HGNC Symb	chr1:22835	NM_00101	.	.	.	NP_001010	.	protein_co	ENSG0000(	200205	27302
IBSP	0	0	0	0	0	0	integrin binding sialoprotein [Source:HGNC Symbol;Acc:HGNC:53	chr4:88720	NM_00496	.	.	.	NP_004958	.	protein_co	ENSG0000(	3381	5341
IBTK	14.1225	15.0533	13.0149	13.6043	19.6533	20.1778	inhibitor of Bruton tyrosine kinase [Source:HGNC Symbol;Acc:HG	chr6:82879	NM_00130	XM_00671	.	.	NP_001287	XP_006715	protein_co	ENSG0000(	25998	17853
ICA1	6.63744	5.23328	7.52616	5.61342	5.7545	7.34586	islet cell autoantigen 1 [Source:HGNC Symbol;Acc:HGNC:5343]	chr7:81528	NM_00113	XM_01701	.	.	NP_001129	XP_005249	protein_co	ENSG0000(	3382	5343
ICA1L	0.582707	0.523465	0.674364	0.694954	0.71182	0.814529	islet cell autoantigen 1 like [Source:HGNC Symbol;Acc:HGNC:1444	chr2:20363	NM_00128	.	.	.	NP_001275	.	protein_co	ENSG0000(	130026	14442
ICAM1	4.33957	5.44482	7.42193	8.66258	7.64918	7.58703	intercellular adhesion molecule 1 [Source:HGNC Symbol;Acc:HGN	chr19:1038	NM_00020	.	.	.	NP_000192	.	protein_co	ENSG0000(	3383	5344
ICAM2	0	0	0	0.081629	0	0.324865	intercellular adhesion molecule 2 [Source:HGNC Symbol;Acc:HGN	chr17:6207	NM_00087	.	.	.	NP_000864	.	protein_co	ENSG0000(	3384	5345
ICAM3	4.58292	3.40686	3.38312	3.01124	3.2121	2.15717	intercellular adhesion molecule 3 [Source:HGNC Symbol;Acc:HGN	chr19:1044	NM_00216	XM_01152	.	.	NP_001307	XP_011526	protein_co	ENSG0000(	3385	5346
ICAM4	0.171379	0.08453	0.119683	0.185518	0.19948	0.093486	intercellular adhesion molecule 4 (Landsteiner-Wiener blood grou	chr19:1039	NM_00103	.	.	.	NP_001034	.	protein_co	ENSG0000(	3386	5347
ICAM5	10.5958	11.3641	15.4696	7.44009	7.41317	11.9973	intercellular adhesion molecule 5 [Source:HGNC Symbol;Acc:HGN	chr19:1040	NM_00325	XM_01152	.	.	NP_003250	XP_011526	protein_co	ENSG0000(	7087	5348
ICE1	14.4515	14.5447	14.0195	12.5816	15.761	12.6678	interactor of little elongation complex ELL subunit 1 [Source:HGN	chr5:54228	NM_01532	.	.	.	NP_056140	XP_011512	protein_co	ENSG0000(	23379	29154
ICE2	8.50928	8.49897	8.33692	7.81066	9.85803	11.2626	interactor of little elongation complex ELL subunit 2 [Source:HGN	chr15:6071	NM_00101	XM_01152	.	.	NP_001018	XP_005254	protein_co	ENSG0000(	79664	29885
ICK	5.19223	5.53294	5.32874	5.03175	5.91377	7.03856	intestinal cell kinase [Source:HGNC Symbol;Acc:HGNC:21219]	chr6:52866	NM_01492	XM_01701	.	.	NP_055735	XP_011512	protein_co	ENSG0000(	22858	21219
ICMT	35.5199	35.0348	28.9865	36.2743	34.8244	30.6162	isoprenylcysteine carboxyl methyltransferase [Source:HGNC Sym	chr1:62662	NM_01240	XM_01154	.	.	NP_036537	XP_011539	protein_co	ENSG0000(	23463	5350
ICOS	0.036956	0	0	0	0	0	inducible T-cell costimulator [Source:HGNC Symbol;Acc:HGNC:53	chr2:20480	NM_01209	.	.	.	NP_036224	.	protein_co	ENSG0000(	29851	5351
ICOSLG	4.79815	4.59952	4.48052	3.56423	3.49991	2.43741	inducible T-cell costimulator ligand [Source:HGNC Symbol;Acc:HG	chr21:4564	NM_00128	XM_01152	.	.	NP_001269	XP_011527	protein_co	ENSG0000(	102723996	17087
ID1	197.205	190.583	256.073	295.86	251.098	399.435	inhibitor of DNA binding 1, HLH protein [Source:HGNC Symbol;Ac	chr20:3019	NM_00216	.	.	.	NP_002156	.	protein_co	ENSG0000(	3397	5360
ID2	56.947	52.8657	96.4477	124.67	105.149	156.415	inhibitor of DNA binding 2, HLH protein [Source:HGNC Symbol;Ac	chr2:88189	NM_00216	.	.	.	NP_002157	.	protein_co	ENSG0000(	3398	5361
ID3	67.2637	69.0686	48.4689	133.403	97.4164	105.018	inhibitor of DNA binding 3, HLH protein [Source:HGNC Symbol;Ac	chr1:23884	NM_00216	.	.	.	NP_002158	.	protein_co	ENSG0000(	3399	5362
ID4	0	0.16647	0.183559	0.24166	0.421335	0.743973	inhibitor of DNA binding 4, HLH protein [Source:HGNC Symbol;Ac	chr6:19837	NM_00154	.	.	.	NP_001537	.	protein_co	ENSG0000(	3400	5363
IDE	15.3069	15.4302	14.2834	8.47261	9.93213	9.35772	insulin degrading enzyme [Source:HGNC Symbol;Acc:HGNC:5381]	chr10:9421	NM_00116	XM_00526	.	.	NP_001159	XP_016871	protein_co	ENSG0000(	3416	5381
IDH1	48.2013	45.5262	51.6717	58.0183	58.0017	74.8581	isocitrate dehydrogenase (NADP(+)) 1, cytosolic [Source:HGNC Sy	chr2:20910	NM_00128	.	.	.	NP_001269	.	protein_co	ENSG0000(	3417	5382
IDH2	23.4479	22.2787	33.8583	38.2615	33.0424	37.653	isocitrate dehydrogenase (NADP(+)) 2, mitochondrial [Source:HG	chr15:9062	NM_00128	.	.	.	NP_001276	.	protein_co	ENSG0000(	3418	5383
IDH3A	29.6237	33.8696	33.2409	27.3504	38.6929	28.2534	isocitrate dehydrogenase 3 (NAD(+)) alpha [Source:HGNC Symbol	chr15:7844	NM_00553	XM_00525	.	.	NP_005521	XP_005254	protein_co	ENSG0000(	3419	5384
IDH3B	64.3279	58.5377	62.2226	70.8108	55.9343	53.6835	isocitrate dehydrogenase 3 (NAD(+)) beta [Source:HGNC Symbol;	chr20:2639	NM_00125	XM_00526	.	.	NP_001245	XP_005260	protein_co	ENSG0000(	3420	5385
IDH3G	32.7172	34.978	31.5789	32.6153	25.5445	19.6408	isocitrate dehydrogenase 3 (NAD(+)) gamma [Source:HGNC Symb	chrX:15305	NM_00413	.	.	.	NP_004126	.	protein_co	ENSG0000(	3421	5386
IDI1	37.5213	35.8342	44.6211	57.0679	60.5376	71.7244	isopentenyl-diphosphate delta isomerase 1 [Source:HGNC Symbo	chr10:1085	NM_00450	.	.	.	NP_004499	.	protein_co	ENSG0000(	3422	5387
IDI2	0	0	0	0	0	0	isopentenyl-diphosphate delta isomerase 2 [Source:HGNC Symbo	chr10:1033	NM_03326	.	.	.	NP_150286	.	protein_co	ENSG0000(	91734	23487
IDNK	2.09017	1.30033	2.18852	1.06026	1.23238	3.31907	IDNK, gluconokinase [Source:HGNC Symbol;Acc:HGNC:31367]	chr9:86237	NM_00100	XM_01151	.	.	NP_001001	XP_006717	protein_co	ENSG0000(	414328	31367
IDO1	0	0	0	0	0	0	indoleamine 2,3-dioxygenase 1 [Source:HGNC Symbol;Acc:HGNC:	chr8:39759	NM_00216	.	.	.	NP_002155	.	protein_co	ENSG0000(	3620	6059
IDO2	0	0	0	0	0	0	indoleamine 2,3-dioxygenase 2 [Source:HGNC Symbol;Acc:HGNC:	chr8:39792	NM_19429	.	.	.	NP_919270	.	protein_co	ENSG0000(	169355	27269
IDS	21.296	22.5443	20.1775	15.1258	13.3668	14.6831	iduronate 2-sulfatase [Source:HGNC Symbol;Acc:HGNC:5389]	chrX:14855	NM_00020	.	.	.	NP_000193	.	protein_co	ENSG0000(	3423	5389
IDUA	4.27927	4.40385	6.84312	1.92582	1.29927	2.22864	iduronidase, alpha-L- [Source:HGNC Symbol;Acc:HGNC:5391]	chr4:97286	NM_00020	XM_01151	.	.	NP_000194	XP_006713	protein_co	ENSG0000(	3425	5391
IER2	92.4666	95.7849	103.799	90.398	81.7549	128.643	immediate early response 2 [Source:HGNC Symbol;Acc:HGNC:288	chr19:1326	NM_00490	.	.	.	NP_004898	.	protein_co	ENSG0000(	9592	28871
IER3	575.315	562.394	640.521	463.129	415.773	820.103	immediate early response 3 [Source:HGNC Symbol;Acc:HGNC:539	chr6:30710	NM_00389	.	.	.	NP_003888	.	protein_co	ENSG0000(	8870	5392
IER3IP1	13.9946	13.4892	10.9371	16.2593	15.3863	15.1512	immediate early response 3 interacting protein 1 [Source:HGNC S	chr18:4467	NM_01609	.	.	.	NP_057181	.	protein_co	ENSG0000(	51124	18550
IER5	24.4792	25.9319	23.9171	30.9633	25.8821	25.3353	immediate early response 5 [Source:HGNC Symbol;Acc:HGNC:539	chr1:18105	NM_01654	.	.	.	NP_057629	.	protein_co	ENSG0000(	51278	5393
IER5L	56.0228	58.4227	70.4876	56.3403	45.2234	70.3015	immediate early response 5 like [Source:HGNC Symbol;Acc:HGNC	chr9:13193	NM_20343	.	.	.	NP_982258	.	protein_co	ENSG0000(	389792	23679
IFFO1	0.150911	0.029625	0.052257	0.032234	0.184997	0.03383	intermediate filament family orphan 1 [Source:HGNC Symbol;Acc	chr12:6647	NM_00103	XM_01701	.	.	NP_001034	XP_006719	protein_co	ENSG0000(	25900	24970
IFFO2	5.94139	5.72643	7.637	7.46937	7.49997	5.76261	intermediate filament family orphan 2 [Source:HGNC Symbol;Acc	chr1:19230	NM_00113	XM_01154	.	.	NP_001129	XP_011538	protein_co	ENSG0000(	126917	27006
IFI16	0.17581	0.294593	0.133056	0.341956	0.306352	0.099407	interferon gamma inducible protein 16 [Source:HGNC Symbol;Acc	chr1:15897	NM_00120	XM_01150	.	.	NP_001193	XP_005245	protein_co	ENSG0000(	3428	5395
IFI27	73.1953	73.816	4.85075	9.5283	7.67327	3.98389	interferon alpha inducible protein 27 [Source:HGNC Symbol;Acc:H	chr14:9457	NM_00128	.	.	.	NP_001275	.	protein_co	ENSG0000(	3429	5397
IFI27L1	21.0786	17.2682	14.4759	16.5831	12.6599	11.1274	interferon alpha inducible protein 27 like 1 [Source:HGNC Symbo	chr14:9454	NM_14524	XM_01153	.	.	NP_660292	XP_011534	protein_co	ENSG0000(	122509	19754
IFI27L2	63.8487	54.1381	70.1101	72.6489	51.6976	57.6304	interferon alpha inducible protein 27 like 2 [Source:HGNC Symbo	chr14:9459	NM_03203	.	.	.	NP_114425	.	protein_co	ENSG0000(	83982	19753
IFI30	97.2267	94.2188	83.0285	114.2	93.959	73.2618	IFI30, lysosomal thiol reductase [Source:HGNC Symbol;Acc:HGNC	chr19:1828	NM_00633	.	.	.	NP_006323	.	protein_co	ENSG0000(	10437	5398
IFI35	12.0906	13.8146	7.86256	14.2656	9.23432	10.0437	interferon induced protein 35 [Source:HGNC Symbol;Acc:HGNC:5	chr17:4115	NM_00553	XM_00525	.	.	NP_005524	XP_005257	protein_co	ENSG0000(	3430	5399
IFI44	8.07959	8.56028	0.82304	7.22466	6.94242	4.23241	interferon induced protein 44 [Source:HGNC Symbol;Acc:HGNC:1	chr1:79115	NM_00641	XM_01154	NR_135640	.	NP_006408	XP_011538	protein_co	ENSG0000(	10561	16938
IFI44L	0.105123	0.185608	0.022927	0	0.014423	0	interferon induced protein 44 like [Source:HGNC Symbol;Acc:HGN	chr1:79086	NM_00682	XM_01154	.	.	NP_006811	XP_011538	protein_co	ENSG0000(	10964	17817
IFI6	239.671	217.473	42.8225	79.2745	62.5746	75.1703	interferon alpha inducible protein 6 [Source:HGNC Symbol;Acc:H	chr1:27992	NM_00203	.	.	.	NP_002029	.	protein_co	ENSG0000(	2537	4054
IFIH1	8.88197	9.00858	4.19455	4.94781	8.11654	6.83623	interferon induced with helicase C domain 1 [Source:HGNC Symb	chr2:16312	NM_02216	XM_01151	.	.	NP_071451	.	protein_co	ENSG0000(	64135	18873
IFIT1	68.5199	66.7163	3.96411	61.3321	51.0752	40.2671	interferon induced protein with tetratricopeptide repeats 1 [Sour	chr10:9097	NM_00127	.	.	.	NP_001257	.	protein_co	ENSG0000(	3434	5407
IFIT1B	0	0	0	0	0	0	interferon induced protein with tetratricopeptide repeats 1B [Sou	chr10:9097	NM_00101	.	.	.	NP_001010	.	protein_co	ENSG0000(	439996	23442
IFIT2	33.4627	35.2345	1.92911	18.5857	19.1344	19.0679	interferon induced protein with tetratricopeptide repeats 2 [Sour	chr10:9097	NM_00154	.	.	.	NP_001538	.	protein_co	ENSG0000(	3433	5409
IFIT3	18.5666	17.2428	1.64379	16.8375	17.7111	12.4099	interferon induced protein with tetratricopeptide repeats 3 [Sour	chr10:9097	NM_00103	.	.	.	NP_001026	.	protein_co	ENSG0000(	3437	5411
IFIT5	15.2006	16.3311	10.7991	13.4	13.9177	11.5327	interferon induced protein with tetratricopeptide repeats 5 [Sour	chr10:9117	NM_01242	.	.	.	NP_036552	.	protein_co	ENSG0000(	24138	13328
IFITM1	9.0023	9.78997	4.9135	0.669423	0.228539	0.652879	interferon induced transmembrane protein 1 [Source:HGNC Sym	chr11:3135	NM_00364	.	.	.	NP_003632	.	protein_co	ENSG0000(	8519	5412
IFITM10	0.025659	7.4E-118	0.127453	0.092844	0.030751	0.143466	interferon induced transmembrane protein 10 [Source:HGNC Syn	chr11:1753	NM_00117	.	.	.	NP_001164	.	protein_co	ENSG0000(	402778	40022
IFITM2	111.578	104.693	124.912	110.491	84.249	78.5656	interferon induced transmembrane protein 2 [Source:HGNC Sym	chr11:3076	NM_00643	.	.	.	NP_006426	.	protein_co	ENSG0000(	10581	5413

IFITM3	488.682	478.798	303.567	440.881	318.873	252.594	interferon induced transmembrane protein 3 [Source:HGNC Symb	chr11:3196	NM_02103	.	.	.	NP_06636	.	protein_co	ENSG0000	10410	5414
IFITM5	0	0	0	0	0	0	interferon induced transmembrane protein 5 [Source:HGNC Symb	chr11:2981	NM_00102	.	.	.	NP_00102	.	protein_co	ENSG0000	387733	16644
IFNA1	0	0	0	0	0	0	interferon alpha 1 [Source:HGNC Symbol;Acc:HGNC:5417]	chr9:21409	NM_02401	.	.	.	NP_07691	.	protein_co	ENSG0000	3439	5417
IFNA10	0	0	0	0	0	0	interferon alpha 10 [Source:HGNC Symbol;Acc:HGNC:5418]	chr9:21206	NM_00217	.	.	.	NP_00216	.	protein_co	ENSG0000	3446	5418
IFNA13	0	0	0	0	0	0	interferon alpha 13 [Source:HGNC Symbol;Acc:HGNC:5419]	chr9:21367	NM_00690	.	.	.	NP_00883	.	protein_co	ENSG0000	3447	5419
IFNA14	0	0	0	0	0	0	interferon alpha 14 [Source:HGNC Symbol;Acc:HGNC:5420]	chr9:21239	NM_00217	.	.	.	NP_00216	.	protein_co	ENSG0000	3448	5420
IFNA16	0	0	0	0	0	0	interferon alpha 16 [Source:HGNC Symbol;Acc:HGNC:5421]	chr9:21216	NM_00217	.	.	.	NP_00216	.	protein_co	ENSG0000	3449	5421
IFNA17	0	0	0	0	0	0	interferon alpha 17 [Source:HGNC Symbol;Acc:HGNC:5422]	chr9:21227	NM_02126	.	.	.	NP_06709	.	protein_co	ENSG0000	3451	5422
IFNA2	0	0	0	0	0	0	interferon alpha 2 [Source:HGNC Symbol;Acc:HGNC:5423]	chr9:21384	NM_00060	.	.	.	NP_00059	.	protein_co	ENSG0000	3440	5423
IFNA21	0	0	0	0	0	0	interferon alpha 21 [Source:HGNC Symbol;Acc:HGNC:5424]	chr9:21165	NM_00217	.	.	.	NP_00216	.	protein_co	ENSG0000	3452	5424
IFNA4	0	0	0	0	0	0	interferon alpha 4 [Source:HGNC Symbol;Acc:HGNC:5425]	chr9:21186	NM_02106	.	.	.	NP_06654	.	protein_co	ENSG0000	3441	5425
IFNA5	0	0	0	0.160733	0	0	interferon alpha 5 [Source:HGNC Symbol;Acc:HGNC:5426]	chr9:21304	NM_00216	.	.	.	NP_00216	.	protein_co	ENSG0000	3442	5426
IFNA6	0	0	0	0	0	0	interferon alpha 6 [Source:HGNC Symbol;Acc:HGNC:5427]	chr9:21349	NM_02100	.	.	.	NP_06628	.	protein_co	ENSG0000	3443	5427
IFNA7	0	0	0	0	0	0	interferon alpha 7 [Source:HGNC Symbol;Acc:HGNC:5428]	chr9:21201	NM_02105	.	.	.	NP_06640	.	protein_co	ENSG0000	3444	5428
IFNA8	0	0	0	0	0	0	interferon alpha 8 [Source:HGNC Symbol;Acc:HGNC:5429]	chr9:21409	NM_00217	.	.	.	NP_00216	.	protein_co	ENSG0000	3445	5429
IFNAR1	7.73565	8.16829	8.19089	5.79193	7.63946	9.00202	interferon alpha and beta receptor subunit 1 [Source:HGNC Symb	chr21:3469	NM_00062	XM_00526	.	.	NP_00062	XP_005261	protein_co	ENSG0000	3454	5432
IFNAR2	6.93265	7.27976	9.28463	5.39336	7.91415	8.73247	interferon alpha and beta receptor subunit 2 [Source:HGNC Symb	chr21:3460	NM_00087	.	.	.	NP_00086	.	protein_co	ENSG0000	3455	5433
IFNB1	0.762376	0.26904	0	0.569996	0.094012	0.339131	interferon beta 1 [Source:HGNC Symbol;Acc:HGNC:5434]	chr9:21077	NM_00217	.	.	.	NP_00216	.	protein_co	ENSG0000	3456	5434
IFNE	0.429204	0.230938	0.516025	0.184491	0.470568	0.260026	interferon epsilon [Source:HGNC Symbol;Acc:HGNC:18163]	chr9:21480	NM_17689	.	.	.	NP_79537	.	protein_co	ENSG0000	338376	18163
IFNG	0	0	0	0	0	0	interferon gamma [Source:HGNC Symbol;Acc:HGNC:5438]	chr12:6854	NM_00061	.	.	.	NP_00061	.	protein_co	ENSG0000	3458	5438
IFNGR1	27.8712	25.9534	28.5584	15.1553	17.7146	18.9976	interferon gamma receptor 1 [Source:HGNC Symbol;Acc:HGNC:5	chr6:13751	NM_00041	XM_01153	.	.	NP_00040	XP_006715	protein_co	ENSG0000	3459	5439
IFNGR2	46.3362	44.9293	43.5433	39.2388	31.1129	32.6827	interferon gamma receptor 2 (interferon gamma transducer 1) [S	chr21:3477	NM_00553	XM_00526	.	.	NP_00131	XP_005261	protein_co	ENSG0000	3460	5440
IFNK	0	0	0	0	0	0	interferon kappa [Source:HGNC Symbol;Acc:HGNC:21714]	chr9:27325	NM_02012	.	.	.	NP_06450	.	protein_co	ENSG0000	56832	21714
IFNL1	3.62891	4.66838	0	4.52375	4.69289	6.63951	interferon lambda 1 [Source:HGNC Symbol;Acc:HGNC:18363]	chr19:3978	NM_17214	.	.	.	NP_74215	.	protein_co	ENSG0000	282618	18363
IFNL2	1.94633	1.51778	0	2.53422	0.47952	1.83576	interferon lambda 2 [Source:HGNC Symbol;Acc:HGNC:18364]	chr19:3975	NM_17213	XM_01152	.	.	NP_74215	XP_011525	protein_co	ENSG0000	282616	18364
IFNL3	0.497286	1.4248	0.123957	1.59838	1.90957	2.37141	interferon lambda 3 [Source:HGNC Symbol;Acc:HGNC:18365]	chr19:3973	NM_17213	XM_01152	.	.	NP_74215	XP_005258	protein_co	ENSG0000	282617	18365
IFNLR1	4.51043	4.61072	3.60007	3.90749	3.78003	3.64203	interferon lambda receptor 1 [Source:HGNC Symbol;Acc:HGNC:1	chr1:24480	NM_17074	XM_01700	.	.	NP_73446	XP_016855	protein_co	ENSG0000	163702	18584
IFNW1	0	0	0	0	0	0	interferon omega 1 [Source:HGNC Symbol;Acc:HGNC:5448]	chr9:21140	NM_00217	.	.	.	NP_00216	.	protein_co	ENSG0000	3467	5448
IFRD1	24.5684	22.944	13.9378	19.3505	23.5959	32.3739	interferon related developmental regulator 1 [Source:HGNC Sym	chr7:11206	NM_00100	XM_01151	.	.	NP_00100	XP_011514	protein_co	ENSG0000	3475	5456
IFRD2	60.8893	61.4811	51.456	67.0142	57.4076	40.5131	interferon related developmental regulator 2 [Source:HGNC Sym	chr3:50316	NM_00676	.	.	.	NP_00675	.	protein_co	ENSG0000	7866	5457
IFRG15	21.4787	28.2489	24.062	30.2946	26.6246	23.539	Homo sapiens torsin A interacting protein 2 (TOR1AIP2), transcrip	chr1:17980	NM_02234	.	.	.	NP_07174	.	protein_co	ENSG0000	163590	.
IFT122	4.7294	4.24468	4.33946	5.66558	5.27011	4.37666	intraflagellar transport 122 [Source:HGNC Symbol;Acc:HGNC:135	chr3:12914	NM_00128	XM_01700	.	.	NP_00126	XP_005247	protein_co	ENSG0000	55764	13556
IFT140	4.36052	4.90322	4.9849	4.1018	3.65135	4.13084	intraflagellar transport 140 [Source:HGNC Symbol;Acc:HGNC:290	chr16:1543	NM_01471	XM_01702	.	.	NP_05552	XP_006721	protein_co	ENSG0000	9742	29077
IFT172	2.53207	2.49201	2.43631	1.90893	2.12449	1.65315	intraflagellar transport 172 [Source:HGNC Symbol;Acc:HGNC:303	chr2:27665	NM_01566	XM_01700	.	.	NP_05647	XP_005264	protein_co	ENSG0000	26160	30391
IFT20	13.2455	14.7982	16.4714	12.1081	10.5246	13.5984	intraflagellar transport 20 [Source:HGNC Symbol;Acc:HGNC:3098	chr17:2664	NM_00126	.	.	.	NP_00125	.	protein_co	ENSG0000	90410	30989
IFT22	20.3247	18.1145	18.5302	22.8364	19.4671	17.4476	intraflagellar transport 22 [Source:HGNC Symbol;Acc:HGNC:2189	chr7:10095	NM_00113	.	.	.	NP_00112	.	protein_co	ENSG0000	64792	21895
IFT27	10.206	10.0869	9.83996	12.4869	9.9445	11.6685	intraflagellar transport 27 [Source:HGNC Symbol;Acc:HGNC:1862	chr22:3715	NM_00117	XM_00672	.	.	NP_00117	XP_006724	protein_co	ENSG0000	11020	18626
IFT43	21.2932	18.2023	20.18	22.8442	18.8515	19.6487	intraflagellar transport 43 [Source:HGNC Symbol;Acc:HGNC:2966	chr14:7645	NM_00110	.	.	.	NP_00109	.	protein_co	ENSG0000	112752	29669
IFT46	9.15971	7.8726	7.28319	10.6782	9.07501	8.76095	intraflagellar transport 46 [Source:HGNC Symbol;Acc:HGNC:2614	chr11:1183	NM_00116	XM_01154	.	.	NP_00116	XP_011541	protein_co	ENSG0000	56912	26146
IFT52	15.3768	15.7845	17.2376	17.5157	16.9274	18.529	intraflagellar transport 52 [Source:HGNC Symbol;Acc:HGNC:1590	chr20:4221	NM_00130	.	.	.	NP_00129	.	protein_co	ENSG0000	51098	15901
IFT57	10.6509	10.9244	12.3713	13.5023	10.7313	9.77501	intraflagellar transport 57 [Source:HGNC Symbol;Acc:HGNC:1736	chr3:10787	NM_01801	.	.	.	NP_06048	.	protein_co	ENSG0000	55081	17367
IFT74	5.96313	5.53191	6.17966	5.17464	7.78724	6.88354	intraflagellar transport 74 [Source:HGNC Symbol;Acc:HGNC:2142	chr9:26904	NM_00109	XM_01151	.	.	NP_00109	XP_011516	protein_co	ENSG0000	80173	21424
IFT80	7.58499	6.61369	9.95296	6.42285	10.6981	12.0678	intraflagellar transport 80 [Source:HGNC Symbol;Acc:HGNC:2926	chr3:15997	NM_00119	.	.	.	NP_00117	.	protein_co	ENSG0000	57560	29262
IFT81	4.6655	5.6396	6.01794	4.20808	6.1083	6.36925	intraflagellar transport 81 [Source:HGNC Symbol;Acc:HGNC:1431	chr12:1105	NM_00114	XM_01153	.	.	NP_00113	XP_011536	protein_co	ENSG0000	28981	14313
IFT88	3.24823	3.37222	3.98022	3.3784	4.17206	5.65014	intraflagellar transport 88 [Source:HGNC Symbol;Acc:HGNC:2060	chr13:2114	NM_00653	XM_01153	.	.	NP_00130	XP_005266	protein_co	ENSG0000	8100	20606
IGBP1	29.7301	30.9102	40.4362	33.5876	30.5018	39.3366	immunoglobulin (CD79A) binding protein 1 [Source:HGNC Symbo	chrX:69353	NM_00155	XM_01702	.	.	NP_00154	XP_016884	protein_co	ENSG0000	3476	5461
IGDCC3	0.232758	0.142989	0.354866	0.154794	0.185891	0.174428	immunoglobulin superfamily DCC subclass member 3 [Source:HG	chr15:6561	NM_00488	XM_01152	.	.	NP_00487	XP_011520	protein_co	ENSG0000	9543	9700
IGDCC4	0.435436	0.291027	0.452466	0.242945	0.228102	0.255573	immunoglobulin superfamily DCC subclass member 4 [Source:HG	chr15:6567	NM_02096	XM_01702	.	.	NP_06601	XP_011520	protein_co	ENSG0000	57722	13770
IGF1	0	0	0	0	0	0	insulin like growth factor 1 [Source:HGNC Symbol;Acc:HGNC:5464	chr12:1027	NM_00061	XM_01701	.	.	NP_00060	XP_016874	protein_co	ENSG0000	3479	5464
IGF1R	2.92849	2.87169	3.58269	2.36789	2.59366	3.20155	insulin like growth factor 1 receptor [Source:HGNC Symbol;Acc:H	chr15:9919	NM_00087	XM_01702	.	.	NP_00086	XP_011519	protein_co	ENSG0000	3480	5465
IGF2	3.45255	3.47817	4.45302	3.54561	2.82515	3.24256	insulin like growth factor 2 [Source:HGNC Symbol;Acc:HGNC:5466	chr11:2150	NM_00061	.	.	.	NP_00060	.	protein_co	ENSG0000	3481	5466
IGF2BP1	0.076219	0.317856	0.149057	0.175182	0.150622	0.157155	insulin like growth factor 2 mRNA binding protein 1 [Source:HGN	chr17:4707	NM_00116	XM_01702	.	.	NP_00115	XP_005257	protein_co	ENSG0000	10642	28866
IGF2BP2	34.2275	32.4561	36.9599	36.0943	36.7892	43.9437	insulin like growth factor 2 mRNA binding protein 2 [Source:HGN	chr3:18536	NM_00100	XM_01700	.	.	NP_00100	XP_016861	protein_co	ENSG0000	10644	28867
IGF2BP3	31.7965	32.6536	43.7456	40.9165	43.3999	62.1287	insulin like growth factor 2 mRNA binding protein 3 [Source:HGN	chr7:23338	NM_00654	XM_01151	.	.	NP_00653	XP_011513	protein_co	ENSG0000	10643	28868
IGF2R	10.7333	11.7599	11.0059	9.4965	10.2781	10.6561	insulin like growth factor 2 receptor [Source:HGNC Symbol;Acc:H	chr6:16039	NM_00087	.	.	.	NP_00086	.	protein_co	ENSG0000	3482	5467
IGFALS	0	0	0.141381	0.353789	0	0.064372	insulin like growth factor binding protein acid labile subunit [Sour	chr16:1840	NM_00114	.	.	.	NP_00113	.	protein_co	ENSG0000	3483	5468
IGFBP1	0	0	0	0	0	0	insulin like growth factor binding protein 1 [Source:HGNC Symbol	chr7:45927	NM_00059	.	.	.	NP_00058	.	protein_co	ENSG0000	3484	5469
IGFBP2	98.8941	97.534	104.078	152.664	115.574	128.059	insulin like growth factor binding protein 2 [Source:HGNC Symbol	chr2:21749	NM_00059	.	.	.	NP_00058	.	protein_co	ENSG0000	3485	5471
IGFBP3	0.110598	0.383006	0.731551	0	0.302952	0.131062	insulin like growth factor binding protein 3 [Source:HGNC Symbol	chr7:45951	NM_00059	.	.	.	NP_00058	XP_016867	protein_co	ENSG0000	3486	5472

IGFBP4	0	0	0	0	0	0.043807	insulin like growth factor binding protein 4 [Source:HGNC Symbol	chr17:3859	NM_00155	.	.	.	NP_00154	.	protein_co	ENSG0000	3487	5473
IGFBP5	0.08701	0.075713	0.064805	0.095309	0.070821	0.144387	insulin like growth factor binding protein 5 [Source:HGNC Symbol	chr2:21753	NM_00059	.	.	.	NP_00059	.	protein_co	ENSG0000	3488	5474
IGFBP6	19.7293	24.2357	31.5751	15.4975	12.5351	8.55302	insulin like growth factor binding protein 6 [Source:HGNC Symbol	chr12:5349	NM_00217	.	.	.	NP_00216	XP_016874	protein_co	ENSG0000	3489	5475
IGFBP7	0	0	0	0	0	0	insulin like growth factor binding protein 7 [Source:HGNC Symbol	chr4:57829	NM_00125	.	.	.	NP_00124	.	protein_co	ENSG0000	3490	5476
IGFBPL1	0	0	0	0	0	0	insulin like growth factor binding protein like 1 [Source:HGNC Syn	chr9:38408	NM_00100	.	.	.	NP_00100	.	protein_co	ENSG0000	347252	20081
IGFL1	0	0	0	0	0	0	IGF like family member 1 [Source:HGNC Symbol;Acc:HGNC:24093	chr19:4673	NM_19854	.	.	.	NP_94094	.	protein_co	ENSG0000	374918	24093
IGFL2	0	0.17434	0	0.371056	0	0	IGF like family member 2 [Source:HGNC Symbol;Acc:HGNC:32929	chr19:4665	NM_00100	XM_01152	.	.	NP_00100	XP_011524	protein_co	ENSG0000	147920	32929
IGFL3	0	0	0	0	0	0	IGF like family member 3 [Source:HGNC Symbol;Acc:HGNC:32930	chr19:4662	NM_20739	.	.	.	NP_99727	.	protein_co	ENSG0000	388555	32930
IGFL4	0	0	0	0	0	0	IGF like family member 4 [Source:HGNC Symbol;Acc:HGNC:32931	chr19:4654	NM_00100	.	.	.	NP_00100	.	protein_co	ENSG0000	444882	32931
IGFLR1	3.70048	3.3994	3.15213	5.09462	3.14373	2.73665	IGF like family receptor 1 [Source:HGNC Symbol;Acc:HGNC:23620	chr19:3620	NM_02466	.	.	.	NP_07893	.	protein_co	ENSG0000	79713	23620
IGFN1	0.014336	0.004692	0.025006	0.005072	0.005362	0.004963	immunoglobulin-like and fibronectin type III domain containing 1	chr1:20115	NM_00116	XM_01151	.	.	NP_00115	XP_005245	protein_co	ENSG0000	91156	24607
IGHMBP2	5.43258	6.46365	5.81853	6.88704	5.35011	4.95498	immunoglobulin mu binding protein 2 [Source:HGNC Symbol;Acc	chr11:6867	NM_00218	XM_00527	.	.	NP_00217	XP_005274	protein_co	ENSG0000	3508	5542
IGIP	3.62869	2.60823	2.54009	2.4529	2.20297	3.35536	IgA inducing protein [Source:HGNC Symbol;Acc:HGNC:33847]	chr5:13950	NM_00100	.	.	.	NP_00100	.	protein_co	ENSG0000	492311	33847
IGLL1	0	0	0	0	0	0	immunoglobulin lambda like polypeptide 1 [Source:HGNC Symbo	chr22:2391	NM_02007	XM_01153	.	.	NP_06445	XP_011528	protein_co	ENSG0000	3543	5870
IGLL5	0	0	0	0	0	0	immunoglobulin lambda like polypeptide 5 [Source:HGNC Symbo	chr22:2322	NM_00117	.	.	.	NP_00117	.	protein_co	ENSG0000	100423062	38476
IGLON5	0	0	0	0	0	0	IgLON family member 5 [Source:HGNC Symbol;Acc:HGNC:34550]	chr19:5181	NM_00110	.	.	.	NP_00109	.	protein_co	ENSG0000	402665	34550
IGSF1	0.424007	0.560704	0.399761	0.080276	0.192957	0.201063	immunoglobulin superfamily member 1 [Source:HGNC Symbol;Ac	chrX:13040	NM_00117	XM_01153	.	.	NP_00116	XP_011529	protein_co	ENSG0000	3547	5948
IGSF10	0	0	0.00548	0	0.013969	0	immunoglobulin superfamily member 10 [Source:HGNC Symbol;A	chr3:15080	NM_17882	XM_01151	.	.	NP_84914	XP_011511	protein_co	ENSG0000	285313	26384
IGSF11	0.360868	0.16688	0.274963	0.261352	0.105789	0.369931	immunoglobulin superfamily member 11 [Source:HGNC Symbol;A	chr3:11861	NM_00101	XM_01700	.	.	NP_00101	XP_006713	protein_co	ENSG0000	152404	16669
IGSF21	0	0	0	0	0	0	immunoglobin superfamily member 21 [Source:HGNC Symbol;Ac	chr1:18434	NM_03288	.	.	.	NP_11626	XP_016858	protein_co	ENSG0000	84966	28246
IGSF22	0.012847	0	0.022379	0.013908	0.029713	0.013876	immunoglobulin superfamily member 22 [Source:HGNC Symbol;A	chr11:1872	NM_17358	XM_01152	.	.	NP_77585	XP_011518	protein_co	ENSG0000	283284	26750
IGSF23	0	0	0	0	0	0	immunoglobulin superfamily member 23 [Source:HGNC Symbol;A	chr19:4511	NM_00120	.	.	.	NP_00119	.	protein_co	ENSG0000	147710	40040
IGSF3	9.33123	10.3374	10.7774	8.67707	8.90058	12.3118	immunoglobulin superfamily member 3 [Source:HGNC Symbol;Ac	chr1:11711	NM_00100	XM_01154	.	.	NP_00100	XP_005270	protein_co	ENSG0000	3321	5950
IGSF5	0.072483	0.037857	0.031391	0.324932	0.2437	0.293604	immunoglobulin superfamily member 5 [Source:HGNC Symbol;Ac	chr21:4111	NM_00108	.	.	.	NP_00107	.	protein_co	ENSG0000	150084	5952
IGSF6	0	0	0	0	0	0	immunoglobulin superfamily member 6 [Source:HGNC Symbol;Ac	chr16:2161	NM_00584	.	.	.	NP_00584	.	protein_co	ENSG0000	10261	5953
IGSF8	18.7867	17.6565	19.0779	18.5896	17.0137	17.6926	immunoglobulin superfamily member 8 [Source:HGNC Symbol;Ac	chr1:16006	NM_00120	XM_01700	.	.	NP_00119	XP_005245	protein_co	ENSG0000	93185	17813
IGSF9	4.81517	5.46362	4.42413	6.54866	7.30452	5.34348	immunoglobulin superfamily member 9 [Source:HGNC Symbol;Ac	chr1:15989	NM_00113	XM_01150	.	.	NP_00112	XP_011508	protein_co	ENSG0000	57549	18132
IGSF9B	1.41404	1.47288	1.76918	1.00829	1.43361	1.28084	immunoglobulin superfamily member 9B [Source:HGNC Symbol;A	chr11:1337	NM_00127	XM_01154	.	.	NP_00126	XP_011540	protein_co	ENSG0000	22997	32326
IHH	0.091303	0.04457	0.038838	0.04856	0	0.152608	indian hedgehog [Source:HGNC Symbol;Acc:HGNC:5956]	chr2:21991	NM_00218	.	.	.	NP_00217	.	protein_co	ENSG0000	3549	5956
IK	53.973	53.6961	48.1989	64.7436	51.2821	47.9563	IK cytokine, down-regulator of HLA II [Source:HGNC Symbol;Acc:H	chr5:14001	NM_00608	.	NR_03742	.	NP_00607	.	protein_co	ENSG0000	100500820	5958
IKBIP	10.2653	10.7512	14.948	11.2934	14.2939	12.5817	IKBKB interacting protein [Source:HGNC Symbol;Acc:HGNC:26430	chr12:9900	NM_15368	.	.	.	NP_71015	.	protein_co	ENSG0000	121457	26430
IKBKAP	15.5024	16.6294	15.5099	15.652	15.7971	13.522	inhibitor of kappa light polypeptide gene enhancer in B-cells, kina	chr9:11162	NM_00131	XM_01151	.	.	NP_00130	XP_011517	protein_co	ENSG0000	8518	5959
IKBKB	8.54053	7.95504	9.36752	7.77349	6.37612	4.56086	inhibitor of kappa light polypeptide gene enhancer in B-cells, kina	chr8:42128	NM_00119	XM_01154	.	.	NP_00117	XP_005273	protein_co	ENSG0000	3551	5960
IKBKE	3.95939	3.59448	3.7052	1.67347	1.39992	1.18346	inhibitor of kappa light polypeptide gene enhancer in B-cells, kina	chr1:20664	NM_00119	XM_00527	.	.	NP_00118	XP_005273	protein_co	ENSG0000	9641	14552
IKBKG	7.29612	7.42202	5.38539	6.59063	5.57757	4.59008	inhibitor of kappa light polypeptide gene enhancer in B-cells, kina	chrX:15375	NM_00109	XM_01153	.	.	NP_00109	XP_005274	protein_co	ENSG0000	8517	5961
IKZF1	0	0	0	0	0	0	IKAROS family zinc finger 1 [Source:HGNC Symbol;Acc:HGNC:131	chr7:50344	NM_00122	XM_01701	.	.	NP_00120	XP_011513	protein_co	ENSG0000	10320	13176
IKZF2	0.200068	0.170613	0.397521	0.121875	0.167278	0.285106	IKAROS family zinc finger 2 [Source:HGNC Symbol;Acc:HGNC:131	chr2:21386	NM_00107	XM_01700	.	.	NP_00107	XP_005246	protein_co	ENSG0000	22807	13177
IKZF3	0.045794	0.088365	0.153909	0.060276	0.139185	0.113053	IKAROS family zinc finger 3 [Source:HGNC Symbol;Acc:HGNC:131	chr17:3791	NM_00125	.	.	.	NP_00124	.	protein_co	ENSG0000	22806	13178
IKZF4	2.30473	2.53158	2.31484	2.37639	1.91686	2.2086	IKAROS family zinc finger 4 [Source:HGNC Symbol;Acc:HGNC:131	chr12:5640	NM_02246	XM_01701	.	.	NP_07191	XP_005269	protein_co	ENSG0000	64375	13179
IKZF5	7.08635	7.89099	7.19879	5.69273	6.33032	5.83324	IKAROS family zinc finger 5 [Source:HGNC Symbol;Acc:HGNC:142	chr10:1247	NM_00127	XM_01701	.	.	NP_00125	XP_006718	protein_co	ENSG0000	64376	14283
IL10	0	0	0	0	0	0	interleukin 10 [Source:HGNC Symbol;Acc:HGNC:5962]	chr1:20694	NM_00057	XM_01150	.	.	NP_00056	XP_011507	protein_co	ENSG0000	3586	5962
IL10RA	0.877794	0.553783	0.675317	0.530608	0.645401	0.83335	interleukin 10 receptor subunit alpha [Source:HGNC Symbol;Acc:	chr11:1178	NM_00155	.	.	.	NP_00154	.	protein_co	ENSG0000	3587	5964
IL10RB	9.67729	8.93128	11.3353	9.64681	8.76753	9.87397	interleukin 10 receptor subunit beta [Source:HGNC Symbol;Acc:H	chr21:3460	NM_00062	.	.	.	NP_00061	.	protein_co	ENSG0000	3588	5965
IL11	0.94449	0.850783	1.83913	0.878873	1.05313	2.06558	interleukin 11 [Source:HGNC Symbol;Acc:HGNC:5966]	chr19:5587	NM_00064	.	.	.	NP_00063	.	protein_co	ENSG0000	3589	5966
IL11RA	3.73849	3.84048	4.80644	5.92652	4.47886	5.12959	interleukin 11 receptor subunit alpha [Source:HGNC Symbol;Acc:	chr9:34646	NM_00114	.	.	.	NP_00113	.	protein_co	ENSG0000	3590	5967
IL12A	2.39125	2.37546	2.80265	2.43179	2.20137	1.81219	interleukin 12A [Source:HGNC Symbol;Acc:HGNC:5969]	chr3:15970	NM_00088	.	.	.	NP_00087	.	protein_co	ENSG0000	3592	5969
IL12B	0.07492	0	0.043661	0.027994	0.056066	0.025267	interleukin 12B [Source:HGNC Symbol;Acc:HGNC:5970]	chr5:15874	NM_00218	.	.	.	NP_00217	.	protein_co	ENSG0000	3593	5970
IL12RB1	0.026629	0	0	0	0	0.045242	interleukin 12 receptor subunit beta 1 [Source:HGNC Symbol;Acc	chr19:1816	NM_00129	XM_01152	.	.	NP_00127	XP_006722	protein_co	ENSG0000	3594	5971
IL12RB2	0	0.018946	0	0	0	0.026285	interleukin 12 receptor subunit beta 2 [Source:HGNC Symbol;Acc	chr1:67773	NM_00125	XM_01154	.	.	NP_00124	XP_005270	protein_co	ENSG0000	3595	5972
IL13	0	0.064686	0	0	0.071939	0	interleukin 13 [Source:HGNC Symbol;Acc:HGNC:5973]	chr5:13199	NM_00218	.	.	.	NP_00217	.	protein_co	ENSG0000	3596	5973
IL13RA1	11.3949	11.6443	16.2269	11.3463	13.602	18.1555	interleukin 13 receptor subunit alpha 1 [Source:HGNC Symbol;Ac	chrX:11786	NM_00156	XM_01153	.	.	NP_00155	XP_011529	protein_co	ENSG0000	3597	5974
IL13RA2	0	0	0.050491	0	0	0	interleukin 13 receptor subunit alpha 2 [Source:HGNC Symbol;Ac	chrX:11423	NM_00064	.	.	.	NP_00063	.	protein_co	ENSG0000	3598	5975
IL15	2.0557	2.1682	0.882008	0.671825	1.53465	1.49978	interleukin 15 [Source:HGNC Symbol;Acc:HGNC:5977]	chr4:14255	NM_00058	.	.	.	NP_00057	.	protein_co	ENSG0000	3600	5977
IL15RA	5.93083	7.17664	5.38753	5.39011	3.97119	3.38758	interleukin 15 receptor subunit alpha [Source:HGNC Symbol;Acc:	chr10:5985	NM_00124	XM_01151	.	.	NP_00123	XP_011517	protein_co	ENSG0000	3601	5978
IL16	0.033229	0.026849	0.047056	0	0.030809	0	interleukin 16 [Source:HGNC Symbol;Acc:HGNC:5980]	chr15:8148	NM_00117	XM_01152	.	.	NP_00116	XP_005254	protein_co	ENSG0000	3603	5980
IL17A	0	0	0	0	0	0	interleukin 17A [Source:HGNC Symbol;Acc:HGNC:5981]	chr6:52051	NM_00219	.	.	.	NP_00218	.	protein_co	ENSG0000	3605	5981
IL17B	0.61246	0.36209	0.315775	0.522556	0.286464	0.950534	interleukin 17B [Source:HGNC Symbol;Acc:HGNC:5982]	chr5:14875	NM_01444	.	.	.	NP_05525	.	protein_co	ENSG0000	27190	5982
IL17C	0	0	0.087983	0	0	0	interleukin 17C [Source:HGNC Symbol;Acc:HGNC:5983]	chr16:8870	NM_01327	.	.	.	NP_03741	.	protein_co	ENSG0000	27189	5983
IL17D	0.24178	0.181375	0.257851	0.264562	0.069204	0.19218	interleukin 17D [Source:HGNC Symbol;Acc:HGNC:5984]	chr13:2127	NM_13828	.	.	.	NP_61214	XP_005266	protein_co	ENSG0000	53342	5984

IL17F	0	0	0	0	0	0	interleukin 17F [Source:HGNC Symbol;Acc:HGNC:16404]	chr6:52101	NM_05287	XM_01151	.	.	NP_44310	XP_011512	protein_co	ENSG0000	112744	16404
IL17RA	5.3533	5.06837	4.87112	4.60812	4.63834	4.51218	interleukin 17 receptor A [Source:HGNC Symbol;Acc:HGNC:5985]	chr22:1756	NM_00128	.	.	.	NP_00127	.	protein_co	ENSG0000	23765	5985
IL17RB	7.11627	6.47966	6.73561	8.08234	8.54376	5.23024	interleukin 17 receptor B [Source:HGNC Symbol;Acc:HGNC:18015	chr3:53880	NM_01872	XM_01153	.	.	NP_06119	XP_005265	protein_co	ENSG0000	55540	18015
IL17RC	6.84049	7.22607	6.65372	4.74421	4.66853	4.35224	interleukin 17 receptor C [Source:HGNC Symbol;Acc:HGNC:18358	chr3:99587	NM_00120	XM_01700	NR_03780	.	NP_00119	XP_016862	protein_co	ENSG0000	84818	18358
IL17RD	3.25385	3.43289	5.4655	4.04957	4.09653	6.36014	interleukin 17 receptor D [Source:HGNC Symbol;Acc:HGNC:17616	chr3:57124	NM_00131	XM_01153	.	.	NP_00130	XP_005265	protein_co	ENSG0000	54756	17616
IL17RE	4.19484	3.87818	5.70125	4.41399	4.24648	4.41897	interleukin 17 receptor E [Source:HGNC Symbol;Acc:HGNC:18439	chr3:99443	NM_00119	XM_01153	.	.	NP_00118	XP_006713	protein_co	ENSG0000	132014	18439
IL17REL	0	0	0.042074	0	0	0	interleukin 17 receptor E like [Source:HGNC Symbol;Acc:HGNC:33	chr22:5043	NM_00100	.	.	.	NP_00100	.	protein_co	ENSG0000	400935	33808
IL18	20.89	19.6648	27.2792	16.4542	17.6428	25.5755	interleukin 18 [Source:HGNC Symbol;Acc:HGNC:5986]	chr11:1120	NM_00124	XM_01154	.	.	NP_00123	XP_011541	protein_co	ENSG0000	3606	5986
IL18BP	2.62471	1.90362	3.13982	2.54723	3.09661	3.77421	interleukin 18 binding protein [Source:HGNC Symbol;Acc:HGNC:5	chr11:7170	NM_00103	XM_01701	.	.	NP_00103	XP_006718	protein_co	ENSG0000	10068	5987
IL18R1	1.16649	1.11298	0.899912	1.51046	0.97697	0.859789	interleukin 18 receptor 1 [Source:HGNC Symbol;Acc:HGNC:5988]	chr2:10297	NM_00128	XM_01151	.	.	NP_00126	XP_005264	protein_co	ENSG0000	8809	5988
IL18RAP	0.04908	0	0	0.027807	0	0	interleukin 18 receptor accessory protein [Source:HGNC Symbol;A	chr2:10303	NM_00385	XM_01151	.	.	NP_00384	XP_011510	protein_co	ENSG0000	8807	5989
IL19	0	0	0	0	0	0	interleukin 19 [Source:HGNC Symbol;Acc:HGNC:5990]	chr1:20697	NM_01337	.	.	.	NP_03750	.	protein_co	ENSG0000	29949	5990
IL1A	0.430053	0.423961	0.282043	0.285402	0.039508	0.315689	interleukin 1 alpha [Source:HGNC Symbol;Acc:HGNC:5991]	chr2:11353	NM_00057	.	.	.	NP_00056	.	protein_co	ENSG0000	3552	5991
IL1B	0	0	0	0	0	0.048108	interleukin 1 beta [Source:HGNC Symbol;Acc:HGNC:5992]	chr2:11358	NM_00057	.	.	.	NP_00056	.	protein_co	ENSG0000	3553	5992
IL1F10	0	0	0	0	0	0	interleukin 1 family member 10 (theta) [Source:HGNC Symbol;Acc	chr2:11382	NM_03255	.	.	.	NP_11594	.	protein_co	ENSG0000	84639	15552
IL1R1	3.66519	3.0321	3.31921	4.20318	3.81643	4.95117	interleukin 1 receptor type 1 [Source:HGNC Symbol;Acc:HGNC:59	chr2:10268	NM_00087	XM_01700	.	.	NP_00086	XP_005263	protein_co	ENSG0000	3554	5993
IL1R2	0.048817	0	0.439433	0.160558	0.064169	0.303194	interleukin 1 receptor type 2 [Source:HGNC Symbol;Acc:HGNC:59	chr2:10260	NM_00126	XM_01151	.	.	NP_00124	XP_006712	protein_co	ENSG0000	7850	5994
IL1RAP	0.853108	0.831471	1.3591	0.967962	1.2534	2.20304	interleukin 1 receptor accessory protein [Source:HGNC Symbol;A	chr3:19023	NM_00116	XM_01151	.	.	NP_00116	XP_005247	protein_co	ENSG0000	3556	5995
IL1RAPL1	0.15581	0.124156	0.441158	0.066473	0.108461	0.076916	interleukin 1 receptor accessory protein like 1 [Source:HGNC Sym	chrX:28605	NM_01427	XM_01154	.	.	NP_05508	XP_005274	protein_co	ENSG0000	11141	5996
IL1RAPL2	0	0	0	0	0	0	interleukin 1 receptor accessory protein like 2 [Source:HGNC Sym	chrX:10381	NM_01741	XM_01153	.	.	NP_05911	XP_016884	protein_co	ENSG0000	26280	5997
IL1RL1	0	0	0	0	0	0.035051	interleukin 1 receptor like 1 [Source:HGNC Symbol;Acc:HGNC:599	chr2:10292	NM_00128	XM_01151	.	.	NP_00126	XP_006712	protein_co	ENSG0000	9173	5998
IL1RL2	0	0	0	0	0	0	interleukin 1 receptor like 2 [Source:HGNC Symbol;Acc:HGNC:599	chr2:10280	NM_00385	XM_01151	.	.	NP_00384	XP_005264	protein_co	ENSG0000	8808	5999
IL1RN	0.186508	0.14814	0.319551	0.421681	0.410648	0.495142	interleukin 1 receptor antagonist [Source:HGNC Symbol;Acc:HGN	chr2:11386	NM_00057	XM_01151	.	.	NP_00056	XP_005263	protein_co	ENSG0000	3557	6000
IL2	0	0	0	0	0	0	interleukin 2 [Source:HGNC Symbol;Acc:HGNC:6001]	chr4:12337	NM_00058	.	.	.	NP_00057	.	protein_co	ENSG0000	3558	6001
IL20	0	0	0	0	0	0	interleukin 20 [Source:HGNC Symbol;Acc:HGNC:6002]	chr1:20703	NM_01872	XM_00527	.	.	NP_06119	XP_005273	protein_co	ENSG0000	50604	6002
IL20RA	0.436178	0.925372	0.736853	1.12621	1.26135	0.867115	interleukin 20 receptor subunit alpha [Source:HGNC Symbol;Acc:	chr6:13732	NM_00127	XM_01701	.	.	NP_00126	XP_006715	protein_co	ENSG0000	53832	6003
IL20RB	1.3283	1.7201	0.56429	1.15621	0.665379	4.4844	interleukin 20 receptor subunit beta [Source:HGNC Symbol;Acc:H	chr3:13667	NM_14471	XM_01151	.	.	NP_65331	XP_011511	protein_co	ENSG0000	53833	6004
IL21	0	0	0	0	0	0	interleukin 21 [Source:HGNC Symbol;Acc:HGNC:6005]	chr4:12353	NM_00120	.	.	.	NP_00119	.	protein_co	ENSG0000	59067	6005
IL21R	0.152312	0.277008	0.361788	0.596523	0.454769	2.77519	interleukin 21 receptor [Source:HGNC Symbol;Acc:HGNC:6006]	chr16:2741	NM_02179	XM_01154	.	.	NP_06857	XP_011544	protein_co	ENSG0000	50615	6006
IL22	0	0	0	0	0	0	interleukin 22 [Source:HGNC Symbol;Acc:HGNC:14900]	chr12:6864	NM_02052	.	.	.	NP_06538	.	protein_co	ENSG0000	50616	14900
IL22RA1	2.12055	1.97154	1.17234	2.14651	2.20489	0.946808	interleukin 22 receptor subunit alpha 1 [Source:HGNC Symbol;Ac	chr1:24446	NM_02125	XM_01154	.	.	NP_06708	XP_011540	protein_co	ENSG0000	58985	13700
IL22RA2	0	0	0	0	0	0	interleukin 22 receptor subunit alpha 2 [Source:HGNC Symbol;Ac	chr6:13746	NM_05296	.	.	.	NP_44319	.	protein_co	ENSG0000	116379	14901
IL23A	2.31641	2.04053	0.537429	0.585462	0.884783	0.905869	interleukin 23 subunit alpha [Source:HGNC Symbol;Acc:HGNC:15	chr12:5673	NM_01658	XM_01153	.	.	NP_05766	XP_011536	protein_co	ENSG0000	51561	15488
IL23R	0	0	0	0	0	0	interleukin 23 receptor [Source:HGNC Symbol;Acc:HGNC:19100]	chr1:67557	NM_14470	XM_01154	.	.	NP_65330	XP_011539	protein_co	ENSG0000	149233	19100
IL24	0	0	0	0.1205	0	0	interleukin 24 [Source:HGNC Symbol;Acc:HGNC:11346]	chr1:20707	NM_00118	.	.	.	NP_00117	.	protein_co	ENSG0000	11009	11346
IL25	0	0	0	0	0	0	interleukin 25 [Source:HGNC Symbol;Acc:HGNC:13765]	chr14:2384	NM_02278	.	.	.	NP_07362	.	protein_co	ENSG0000	64806	13765
IL26	0	0	0	0	0	0	interleukin 26 [Source:HGNC Symbol;Acc:HGNC:17119]	chr12:6859	NM_01840	XM_01153	.	.	NP_06087	XP_011536	protein_co	ENSG0000	55801	17119
IL27	0	0	0	0	0	0	interleukin 27 [Source:HGNC Symbol;Acc:HGNC:19157]	chr16:2851	NM_14565	.	.	.	NP_66363	XP_011544	protein_co	ENSG0000	246778	19157
IL27RA	12.7461	12.9988	15.6462	12.6809	12.0924	12.3345	interleukin 27 receptor subunit alpha [Source:HGNC Symbol;Acc:	chr19:1414	NM_00484	.	.	.	NP_00483	.	protein_co	ENSG0000	9466	17290
IL2RA	0	0	0	0	0	0	interleukin 2 receptor subunit alpha [Source:HGNC Symbol;Acc:H	chr10:6052	NM_00041	XM_00525	.	.	NP_00040	XP_005252	protein_co	ENSG0000	3559	6008
IL2RB	0.019879	0	0.017091	0.064209	0.022817	0.043325	interleukin 2 receptor subunit beta [Source:HGNC Symbol;Acc:HG	chr22:3752	NM_00087	.	.	.	NP_00086	.	protein_co	ENSG0000	3560	6009
IL2RG	0.148785	0	0	0	0	0	interleukin 2 receptor subunit gamma [Source:HGNC Symbol;Acc	chrX:70327	NM_00020	.	.	.	NP_00019	.	protein_co	ENSG0000	3561	6010
IL3	0	0	0	0	0	0	interleukin 3 [Source:HGNC Symbol;Acc:HGNC:6011]	chr5:13139	NM_00058	.	.	.	NP_00057	.	protein_co	ENSG0000	3562	6011
IL31	0	0	0	0	0	0	interleukin 31 [Source:HGNC Symbol;Acc:HGNC:19372]	chr12:1226	NM_00101	XM_01153	.	.	NP_00101	XP_011536	protein_co	ENSG0000	386653	19372
IL31RA	0.421225	0.552123	0.134861	0.278274	0.205013	0.287115	interleukin 31 receptor A [Source:HGNC Symbol;Acc:HGNC:18969	chr5:55147	NM_00124	XM_01154	.	.	NP_00122	XP_011541	protein_co	ENSG0000	133396	18969
IL32	10.584	10.9998	9.01309	0.595326	0.518024	0.258448	interleukin 32 [Source:HGNC Symbol;Acc:HGNC:16830]	chr16:3115	NM_00101	XM_00525	.	.	NP_00101	XP_005255	protein_co	ENSG0000	9235	16830
IL33	0	0	0	0	0	0	interleukin 33 [Source:HGNC Symbol;Acc:HGNC:16028]	chr9:62157	NM_00119	XM_01151	.	.	NP_00118	XP_005251	protein_co	ENSG0000	90865	16028
IL34	0	0	0.046329	0	0.125589	0.090488	interleukin 34 [Source:HGNC Symbol;Acc:HGNC:28529]	chr16:7061	NM_00117	XM_01152	.	.	NP_00116	XP_011521	protein_co	ENSG0000	146433	28529
IL36A	0	0	0	0	0	0	interleukin 36, alpha [Source:HGNC Symbol;Acc:HGNC:15562]	chr2:11376	NM_01444	XM_01151	.	.	NP_05525	XP_005263	protein_co	ENSG0000	27179	15562
IL36B	0	0	0	0	0	0	interleukin 36, beta [Source:HGNC Symbol;Acc:HGNC:15564]	chr2:11377	NM_01443	XM_01151	.	.	NP_05525	XP_011509	protein_co	ENSG0000	27177	15564
IL36G	0	0	0.071714	0.09149	0	0.083158	interleukin 36, gamma [Source:HGNC Symbol;Acc:HGNC:15741]	chr2:11373	NM_00127	XM_01151	.	.	NP_00126	XP_011509	protein_co	ENSG0000	56300	15741
IL36RN	0	0	0	0.030812	0	0	interleukin 36 receptor antagonist [Source:HGNC Symbol;Acc:HG	chr2:11381	NM_01227	.	.	.	NP_03640	.	protein_co	ENSG0000	26525	15561
IL37	0	0	0.141439	0.176845	0.119412	0.279841	interleukin 37 [Source:HGNC Symbol;Acc:HGNC:15563]	chr2:11367	NM_01443	XM_01151	.	.	NP_05525	XP_011509	protein_co	ENSG0000	27178	15563
IL3RA	0	0	0	0	0	0	interleukin 3 receptor subunit alpha [Source:HGNC Symbol;Acc:H	chrX:14555	NM_00126	XM_01703	.	.	NP_00125	XP_005274	protein_co	ENSG0000	3563	6012
IL4	0	0	0	0	0	0	interleukin 4 [Source:HGNC Symbol;Acc:HGNC:6014]	chr5:13200	NM_00058	.	.	.	NP_00058	.	protein_co	ENSG0000	3565	6014
IL4I1	0.145603	0.093618	0.103853	0.102955	0.172009	0.054784	interleukin 4 induced 1 [Source:HGNC Symbol;Acc:HGNC:19094]	chr19:5039	NM_00125	.	.	.	NP_00124	.	protein_co	ENSG0000	259307	19094
IL4R	11.0004	10.2341	10.3557	11.7539	10.9157	11.6902	interleukin 4 receptor [Source:HGNC Symbol;Acc:HGNC:6015]	chr16:2732	NM_00041	XM_01154	.	.	NP_00040	XP_011544	protein_co	ENSG0000	3566	6015
IL5	0	0	0.167333	0	0	0.092968	interleukin 5 [Source:HGNC Symbol;Acc:HGNC:6016]	chr5:13187	NM_00087	XM_01154	.	.	NP_00087	XP_011541	protein_co	ENSG0000	3567	6016
IL5RA	0	0	0	0	0	0	interleukin 5 receptor subunit alpha [Source:HGNC Symbol;Acc:H	chr3:31080	NM_00056	XM_01153	.	.	NP_00055	XP_011531	protein_co	ENSG0000	3568	6017

IL6	0	0.119531	0	0	0	0.118173	interleukin 6 [Source:HGNC Symbol;Acc:HGNC:6018]	chr7:22765	NM_00060	XM_01151	.	.	NP_00059	XP_005249	protein_co	ENSG0000	3569	6018
[truncated: 2,726,986 more chars]
